# Folivory elicits a strong defense reaction in *Catharanthus roseus*: metabolomic and transcriptomic analyses reveal distinct local and systemic responses

**DOI:** 10.1038/srep40453

**Published:** 2017-01-17

**Authors:** Thomas Dugé de Bernonville, Inês Carqueijeiro, Arnaud Lanoue, Florent Lafontaine, Paloma Sánchez Bel, Franziska Liesecke, Karine Musset, Audrey Oudin, Gaëlle Glévarec, Olivier Pichon, Sébastien Besseau, Marc Clastre, Benoit St-Pierre, Victor Flors, Stéphane Maury, Elisabeth Huguet, Sarah E. O’Connor, Vincent Courdavault

**Affiliations:** 1 Université François-Rabelais de Tours, EA2106 “Biomolécules et Biotechnologies Végétales”, Tours, France; 2 Metabolic Integration and Cell Signaling Group, Plant Physiology Section, Department of CAMN, Universitat Jaume I, Spain; 3 Institut de Recherche sur la Biologie de l’Insecte, UMR 7261, CNRS/Université François-Rabelais de Tours, Tours, France; 4 Université d’Orléans, CoST, Laboratoire de Biologie des Ligneux et des Grandes Cultures (LBLGC), EA 1207, USC1328 INRA, Orléans, France; 5 The John Innes Centre, Department of Biological Chemistry, Norwich NR4 7UH, United Kingdom

## Abstract

Plants deploy distinct secondary metabolisms to cope with environment pressure and to face bio-aggressors notably through the production of biologically active alkaloids. This metabolism-type is particularly elaborated in *Catharanthus roseus* that synthesizes more than a hundred different monoterpene indole alkaloids (MIAs). While the characterization of their biosynthetic pathway now reaches completion, still little is known about the role of MIAs during biotic attacks. As a consequence, we developed a new plant/herbivore interaction system by challenging *C. roseus* leaves with *Manduca sexta* larvae. Transcriptomic and metabolic analyses demonstrated that *C. roseus* respond to folivory by both local and systemic processes relying on the activation of specific gene sets and biosynthesis of distinct MIAs following jasmonate production. While a huge local accumulation of strictosidine was monitored in attacked leaves that could repel caterpillars through its protein reticulation properties, newly developed leaves displayed an increased biosynthesis of the toxic strictosidine-derived MIAs, vindoline and catharanthine, produced by up-regulation of MIA biosynthetic genes. In this context, leaf consumption resulted in a rapid death of caterpillars that could be linked to the MIA dimerization observed in intestinal tracts. Furthermore, this study also highlights the overall transcriptomic control of the plant defense processes occurring during herbivory.

The Madagascar periwinkle (*Catharanthus roseus* (L.) G. Don; Apocynaceae) is one of the most studied plants displaying an active secondary metabolism (also called specialized metabolism), reaching up the status of “model non-model system” during the last decade^1^. *C. roseus* synthesizes a myriad of monoterpene indole alkaloids (MIAs) that have been proposed to mediate plant adaptation to the environment, especially during biotic interactions^2^. A good example of such a role relies on the proposed phytoanticipin function of strictosidine which upon leaf attack and membrane leakage can be enzymatically deglucosylated to form a highly reactive aglycone. This conversion induces a massive protein reticulation that was suggested to limit aggressor attacks, the so-called “nuclear time-bomb” process^3^. Other studies have also established the toxicity of several MIAs against pests or herbivores but essentially by feeding experiments using high concentrations of selected MIAs or total leaf extracts^4^,^5^,^6^.

For several decades, these cytotoxic MIAs have been valorized as pharmaceutical compounds used to treat human diseases such as the antineoplastic vinblastine and vincristine inhibiting tubulin polymerization, or the antihypertensive ajmalicine^7^. However, their very low amounts *in planta* restrict their supply and have prompted the search for alternative production sources. In addition, MIA complex stereochemistry renders complete chemical synthesis uneconomical. To date, the dimeric MIAs vinblastine and vincristine used in anticancer treatments are produced by the chemical condensation of their monomeric precursors vindoline and catharanthine extracted from leaves of *C. roseus*^8^ (Fig. 1). The recent development of bioengineering approaches based on the elaboration of MIA producing yeast strains following multiple gene transfer as well as on metabolic pathway expression in heterologous plants offer new alternatives but requires the elucidation of the whole MIA biosynthetic pathway^9^,^10^,^11^.

The synthesis of MIAs *in planta* relies on a complex route involving at least thirty enzymatic steps, characterized over 25 years and especially during the last 5 years^12^ (Fig. 1). Basically, MIAs originate from the condensation of a monoterpene precursor, secologanin with an indole precursor, tryptamine, catalyzed by strictosidine synthase (STR). The resulting strictosidine is subsequently deglucosylated by strictosidine β-D-glucosidase (SGD) leading to the formation of the plethora of MIAs synthetized in *C. roseus* such as vindoline and catharanthine as well as additional scaffolds in other MIA producing plants^2^. *In planta*, the condensation of vindoline and catharanthine initiates the synthesis of dimeric MIAs through the formation of anhydrovinblastine that also displays cytotoxic properties^13^.

While MIAs accumulate in nearly all *C. roseus* organs, leaves constitute one of the main sites of accumulation and display a high diversity of MIAs with unique biosynthetic traits like the synthesis of vindoline and its demethoxylated derivative vindorosine^6^,^14^,^15^. The complexity in the number of steps is overlaid by the spatial organization of the pathway which is distributed in at least three different cell-types and five distinct subcellular compartments^16^ (Fig. 1). This multi-site organization implies potential inter- and intra-cellular transport of metabolites but also involves the evolution of distinct enzyme isoforms harboring specific functions in MIAs synthesis, as exemplified with two recently identified secologanin synthase (SLS) isoforms displaying specific and complementary gene expression profiles within plant organs^17^. In the last years, the combination of RNA-seq based transcriptome analyses and gene invalidation procedures based on virus-induced gene silencing allowed the identification of these isoforms as well as other missing genes from this pathway^18^,^19^,^20^,^21^,^22^,^23^,^24^. However, as the pathway deciphering progresses, new complementary transcriptome resources are still required to facilitate and complete the identification of still uncovered enzymes or regulators of the pathway.

The use of contrasting physiological states is known to facilitate primary or specialized metabolism understanding as well as pathway discovery in phytochemical genomic, in particular through gene and metabolite clustering analyses^19^. From this point of view, the biosynthesis of MIAs in *C. roseus* appears to be a tightly regulated process influenced notably by environmental factors and phytohormones. For instance, light and UV stimulate MIA production by promoting MIA biosynthetic gene expression and the corresponding enzymatic activities^25^,^26^,^27^,^28^,^29^. Furthermore, mechanical wounding engenders a similar positive effect while drought stress causes the decrease of the MIA content^30^,^31^. Plant hormones also exhibit pronounced and well documented effects such as the stimulatory properties of cytokinins and ethylene as well as the antagonist role of auxins on MIA biosynthesis^32^,^33^,^34^. On the other hand, jasmonic acid (JA) derivatives have been classically described to mediate plant responses to wounding, necrotrophic fungi and chewing insects, often in a synergistic way with ethylene^35^,^36^,^37^. In *C. roseus,* JA and its derivative methyl jasmonate (MeJA) also trigger the biosynthesis of MIAs mainly through the activation of octadecanoid–responsive Catharanthus AP2/ERF-domain transcription factors (ORCA)^38^. Those transcription factors are able to bind JA-responsive elements (JERE) in the promoters of MIA genes such as STR^39^,^40^. Interestingly, the JA signal also seems to mediate or potentiate the effects of some abiotic and biotic stimuli in *C. roseus* including light exposure or fungal extract treatments^41^,^42^. Such treatments with fungal extracts drastically increase MIA biosynthesis through the rapid stimulation of MIA biosynthetic gene expression including tryptophan decarboxylase (TDC) and STR in cell cultures and hairy roots of *C. roseus*^43^,^44^,^45^. In the light of those data, the use of biotic agents combining several of these signals could lead to a strong and robust modulation of the MIA pathway, thus constituting a valuable tool to decipher MIA metabolism^46^. This postulate is supported by the prominent results retrieved from the herbivory of the tobacco hornworm *Manduca sexta* on *Nicotiana* sp, enabling notably the elucidation of nicotine alkaloid metabolism^47^. While *C. roseus* can be challenged by different types of aggressors, only mollicute infections and especially phytoplasma have been shown to trigger MIA metabolism on whole plants through induction of gene expression^48^,^49^,^50^,^51^. To our knowledge, no other interaction between *C. roseus* plants and other bioaggressors has been characterized at the molecular level, may be due to the lack of easily propagated pests of this plant. Using the model *M. sexta*/*Nicotiana* sp. as a guideline, we developed a new plant-herbivore system based on the non-host interaction of *C. roseus* and *M. sexta* larvae, to investigate how *C. roseus* deploys MIA metabolism in response to herbivory. By combining targeted metabolic analyses and RNA sequencing, we demonstrated that folivory of *C. roseus* caused both local and systemic induction of MIA biosynthesis resulting from the induction of specific MIA biosynthetic gene expression.

## Results and Discussion

### *M. sexta* larvae consumed *C. roseus* leaves and died

To determine whether an efficient interaction can be established between *C. roseus* and *M. sexta*, caterpillars were placed on leaves of *C. roseus* and its host plant *N. tabacum*. In both cases, *M. sexta* larvae were able to consume substantial amounts of leaves during the first 2 h (Fig. 2a,b,e,f). However, independent choice experiments showed an expected and clear preference for *N. tabacum* (Supplemental Figure S1a). Feeding on tobacco leaves remained constant for the next 20 h but progressively decreased on periwinkle up to a total arrest usually observed from 72 h onwards (Fig. 2c,d,g,h). In this context, no weight gain was measured for larvae feeding on *C. roseus* in contrast to larvae feeding on the host plant *N. tabacum* (Supplemental Figure S1b). This was also accompanied by pronounced morphologic alterations (such as body softening; Fig. 2i,j) and intense browning followed by the death of caterpillars (Fig. 2k). Dissection of larvae revealed internal alterations/damages compared to control caterpillars fed on tobacco (Supplemental Figure S1c). Metabolic analyses of the larval gut also revealed the presence of multiple MIAs including the monomers vindoline and catharanthine and especially of their condensation product anhydrovinblastine. This qualitative MIA composition was quite similar to that found in leaves, thus confirming leaf ingestion. However, the dimer/monomer (anhydrovinblastine/catharanthine and vindoline) ratio was dramatically increased compared to those observed in the whole leaf (Supplemental Figure S1d) suggesting that the observed dimerization process contributes to caterpillar intoxication and may rely on the gathering of vindoline and catharanthine that were suggested to accumulate in different leaf compartments^6^. This differs from the MIA content of the intestinal tract of *Bombyx mori* fed with *C. roseus* leaf extracts, where guts of the insect only contained monomers^6^. Taken altogether, our results indicate that *M. sexta* is able to feed long enough on *C. roseus* despite of its toxic compounds to establish a model for analyzing metabolic and transcriptomic changes in *C. roseus*.

### MIA metabolism is induced by herbivory

Leaf consumption by *M. sexta* has been reported to induce the biosynthesis of several defense compounds in *Nicotiana* sp around three days post-attack^52^. We therefore analyzed the MIA composition of damaged *C. roseus* leaves in a similar time range. Leaves were challenged for 2 h with larvae and MIA accumulation was monitored at 48 h and 72 h post-feeding in both locally damaged and distal non damaged leaves, as well as in leaves newly emerged 1 week (168 h) after attack and finally compared to the MIA content of control leaves that had not encountered any herbivory and that were sampled accordingly (Supplemental Figure S2a). Relative quantification was performed on the main MIAs accumulating in leaves (catharanthine and vindoline) but also on minor compounds including ajmalicine, serpentine, anhydrovinblastine, vindorosine and strictosidine. In damaged and distal leaves, slight but significant changes (*p* < 0.05) were observed 48 h and 72 h post attack except for ajmalicine, serpentine, vindorosine and vindoline (Fig. 3). The largest increase was observed for strictosidine at 48 h and 72 h in local leaves (*p* = 0.003 and *p* = 0.001 respectively). This latter was present at trace levels in leaves of control plants (4,549 ± 2,863 AU) but strongly accumulated 48 h and 72 h post-attack (up to x 15 and x 19 respectively in locally damaged leaves). By contrast, only minor increases of strictosidine were monitored at such times when leaves were mechanically wounded suggesting that strictosidine accumulation was specifically induced by the biotic interaction (Supplemental Figure S2b). In addition, in newly emerged leaves 1-week post attack, all MIAs except anhydrovinblastine were significantly enhanced suggesting that a systemic signal induced by herbivory may have triggered the accumulation of MIAs as a probable defense response. The absence of anhydrovinblastine increase in young leaves was expected since it has already been shown to exclusively accumulate in older leaves^6^. Interestingly, only a moderate increase of strictosidine (1.8 times more accumulated; *p* = 0.02, Wilcoxon rank sum test) was observed in newly emerged leaves that might reflect the consumption of this compound to allow the dramatic increase in the biosynthesis of the downstream MIAs including catharanthine, vindoline or anhydrovinblastine. Based on these results, one could speculate that *C. roseus* set up two distinct responses to herbivory, a local and quickly induced one relying on strictosidine accumulation which may cross link proteins following its deglucosylation catalyzed by SGD after membrane leakage^3^, and a systemic and long-term mechanism involving a higher accumulation of toxic MIAs in newly developing organs.

### Herbivory of *C. roseus* leaves led to a marked transcriptional reprogramming

As described for several specialized metabolisms, increase in MIA biosynthesis in *C. roseus* is usually preceded by the activation of the corresponding biosynthetic genes^33^. Moreover, we already reported that MIA biosynthetic genes also respond to fungal elicitors and hormones by an enhanced expression from 8 h to 24 h post-treatment^53^,^54^. We therefore analyzed the transcriptional reprogramming of *C. roseus* leaves subjected to herbivory in four independent experiments following this timeline (Supplemental Figure S2c, Table 1). In all experiments, caterpillars were allowed to feed for 2 h and subsequently removed, indicating the beginning of the kinetics. Total RNA were extracted from Manduca-damaged (Ms) and control (Ctrl, intact plants of the same age and physiological status) leaves at 6 h (Ms6h and Ctrl6h), 8 h (Ms8h and Ctrl8h) and 24 h after the feeding period. In this latter time, leaves were split along the midrib to analyze the damaged (MsDamaged24h) and the intact (MsIntact24h) halves separately. In addition, we analyzed the systemic responses in intact and newly formed leaves obtained 1 week after initial consumption of plant leaves (Ms1wk) and in non-attacked control plants (Ctrl1wk). These 9 samples were then sequenced with the Illumina technology in a paired-end design at an average sequencing depth of 27 million of reads per sample. The resulting reads were pseudo-aligned at a very good rate (>98%) to the CDF97 reference transcriptome^17^ for *C. roseus* to estimate the abundance of each transcript with Salmon^55^.

At each time, log2 of Transcript Per Million (TPM) fold changes between the attacked leaf and the control leaves were calculated (Ms6h/Ctrl6h, Ms8h/Ctrl8h, MsDamaged24h/Ctrl24h, MsIntact24h/Ctrl24h and Ms1wk/Ctrl1wk). Differentially expressed genes were identified in the 5 comparisons at a *p*-value < 0.001 without setting a cut-off threshold for log2 fold changes because of slight differences in attacked areas between samples. The number of transcripts accumulating at higher or lower amounts in attacked leaves compared to control leaves (considered as up-regulated and down-regulated genes respectively) considerably changed over time (Table 2). Overall, changes were more important at 8 h (a total of 2,360 transcripts) and 24 h (1,620 and 1,503 for MsDamaged24h and MsIntact24h respectively). This demonstrated that the intensity of transcriptional responses to herbivory reached a maximum at 8 h and 24 h as previously observed with plant hormone treatments. By contrast, only 333 and 402 up-regulated transcripts and 291 and 366 down-regulated ones were retained for shorter (Ms6h) and prolonged responses (Ms1wk), respectively. These results showed that challenging leaves of *C. roseus* with a chewing insect led to a transcriptional response at least 6 h after initial feeding and to a systemic transcriptome reprogramming in newly developed leaves.

To gain more insights into leaf responses to *M. sexta* herbivory, the lists of differentially expressed transcripts (up-regulated and down-regulated) obtained from the 5 comparisons (attacked vs control) were cross-compared with a Venn diagram analysis to evaluate similarities and specificities of each (Fig. 4a, Supplemental Figure S3). Concerning up-regulated transcripts, a total of 2,588 were more highly expressed in attacked leaves in at least one comparison. It is noteworthy that this number includes a certain degree of redundancy between transcripts, owing to the reference transcriptome which was specifically built to capture isoform complexity in *C. roseus*^17^. Out of these 2,588 transcripts, we defined 5 specific transcript sets induced at each sampling time corresponding to 21% of the up-regulated transcripts for Ms6h (Fig. 4a, dark blue, 71 transcripts over 333), 47% for Ms8h (light green, 696 transcripts over 1,470), 19% for MsDamaged24h (dark green, 230 transcripts over 1,171), 17% for MsIntact24h (red, 186 transcripts over 1,072) and 73% for Ms1wk (orange, 295 transcripts over 402). In addition, we also defined a core set of 813 transcripts (light blue, 31% of the 2,588 transcripts) found in at least 2 different sampling times, including 20 transcripts (0.7%) commonly found in the 5 comparisons (Fig. 4a). In the light of this distribution, largest specificities were observed at 8 h and 1 week after attack in newly emerged leaves reflecting once again the previously measured modification of the MIA content.

Besides up-regulated genes, a lower number of transcripts (1,745) were found to be down-regulated in response to attack in at least one comparison. In this case, overlaps between differentially expressed transcripts were less pronounced than observed for up-regulated ones (Fig. 4b). Indeed, only 337 transcripts (20%, light blue) were common to at least 2 sampling times and were defined as the core set of down-regulated transcripts during herbivory. The Ms8h and Ms1w specific transcript sets (light green and orange, respectively) were once again the most divergent with 577 and 281 down-regulated transcripts, reinforcing thus the prominence of these two conditions in response to folivory.

Finally, out of the 4,333 (2,588 + 1,745) transcripts whose expression was altered upon leaf herbivory, we were able to find homologies (Blastx, *e*-value < 1e–10) for 3,189 transcripts (73%) with Uniprot proteins. This mapping was used to retrieve PFAM and Uniprot keyword information in order to classify the functions characterizing the *C. roseus* responses to *M. sexta*. The next paragraphs focus on the main functions identified in the core sets of up- and down-regulated transcripts but also in specific transcript sets according to the Venn diagram analysis (Fig. 4). All information on transcripts and functions are available in Supplemental Tables S1, S2, S3 and S4.

### *C. roseus* leaf consumption induces a potential photosynthesis breakdown

Analysis of the core set of down-regulated transcripts revealed a strong representation of photosynthesis-related elements. This was the most striking feature of this set of genes. Indeed, many Uniprot keywords related to this process (e.g. Photosynthesis, Chlorophyll biosynthesis) were significantly enriched (*p*-value < 1e–10) within transcripts from this core set (Fig. 4d, Supplementary Table S3 and Supplemental Figure S3b). Accordingly, the PFAM domain PF00504.18 Chlorophyll A/B binding protein was also well represented (24 transcripts) although not significantly enriched (*p*-value = 0.09; Supplementary Table S4). Interestingly, a similar trend was also detected in transcripts specifically associated with Ms8h, MsDamaged24h and MsIntact24h (Fig. 4d). This apparent alteration in photosynthesis-related processes might reflect a reallocation of cell activities towards other processes including defense. Such an effect has been already described in several plant herbivore interactions and particularly for *M. sexta*-mediated herbivory^56^,^57^. In addition, this down-regulation of photosynthesis has been shown to be associated with the production of JA in fed parts^58^. Moreover, many pentatricopeptide-repeat (PPR) containing proteins were also down-regulated at the different sampling times (Supplemental Figure S3b). These proteins were previously shown to be involved in organelle biosynthesis^59^. In our case, they might be linked to potential modifications in chloroplast biogenesis and associated to the proposed decrease in photosynthesis.

### Herbivory caused induction of PR-proteins, terpene synthases and JA metabolism

Within our defined core set of 813 up-regulated genes, many transcripts related to known defense processes were identified (Supplemental Tables S3 and S3). For instance, it contained 17 transcripts related to PFAM domain PF00407.16 (Pathogenesis-related protein Bet v I family, from the PR-10 family). In particular, transcripts homologous to *Solanum tuberosum* pathogenesis-related protein STH-1 and 2 (PRS1_SOLTU and PRS2_SOLTU) and a previously identified probable intracellular pathogenesis-related protein T1 from *C. roseus* (IPRT1_CATRO) were strongly induced in fed leaves (Figs 4c and 5a). A transcript encoding Pathogenesis-Related protein 5 (PR-5) was also identified. Interestingly, these genes types were also over-represented in specific gene sets and notably in the 24 h samples. The Uniprot keyword ‘Pathogenesis-related protein’ was significantly represented in the MsDamaged24h specific gene set (*p* = 0.0003) and the PFAM domain PF00407.16 in the MsDamaged24h and MsIntact24h intersection set (*p* < 1e–05) (Supplemental Tables S3 and S4). Interestingly, cysteine proteinase inhibitors homologous to CYT5_ARATH and CYSP_PEA known to be induced by herbivory and to inhibit insect proteolytic enzymes^60^ were mostly found in these lists, in particular in the MsDamaged24h specific gene set but also in the core set of common transcripts such as the cystatin CYTI_VIGUN (Supplemental Tables S3 and S4). This strong expression of defense proteins during herbivory indicated that *C. roseus* deployed defense responses complementary to MIA biosynthesis.

Aside from these genes related to direct defenses, several up-regulated transcripts suggested a recruitment of indirect defenses through modulations of terpene metabolism. This was illustrated by terpene synthase homologs including *Vitis vinifera* (-)-germacrene D synthase (TPSGD_VITVI), *Quercus ilex* Myrcene synthase (MYRS_QUEIL), *Malus domestica* (E,E)-alpha-farnesene synthase (AFS1_MALDO), *Ricinus communis* Alpha-farnese synthase (TPS7_RICCO) and *Fragaria ananassa* nerolidol Synthase (NES1_FRAAN) catalyzing the synthesis of monoterpenes and sesquiterpenes. Such an activation could result in a *de novo* production of volatile terpene compounds upon herbivory that have been described to limit insect attacks by mediating attraction of parasitoids^61^. This apparent stimulation of sesquiterpene metabolism may also suggest modulations of leaf triterpene content, specifically ursolic acid, which has anti-insect feeding activity and accumulates at high level in *C. roseus* leaves^62^,^63^. However, only one transcript related to triterpene metabolism and encoding a putative squalene synthase from *N. benthamiana* (FDFT_NICBE), was significantly up-regulated in the Ms1wk specific set.

The second feature of the core set of transcripts up-regulated upon herbivory corresponded to the marked association with JA biosynthesis and signaling as revealed by the significant enrichments of ‘Lipid metabolism’ (25 transcripts, p = 1e–04) and ‘Oxylipin biosynthesis’ terms (7 transcripts, p = 1e–07) (Fig. 4c). For example, we observed a high expression of transcripts homologous to *S. tuberosum* Linoleate 13S-lipoxygenase 3–1 (LOX31_SOLTU), *Arabidopsis thaliana* Linoleate 9S-lipoxygenase 5 (LOX5_ARATH), *A. thaliana* Allene oxide cyclase 4 (AOC4_ARATH; although this latter was not annotated in Uniprot as related to JA biosynthesis), *Solanum lycopersicon* 12-oxophytodienoate reductase 3 (OPR3_SOLLC), *A. thaliana* Jasmonate O-methyltransferase (JMT_ARATH) and *A. thaliana* MYB108 transcription factor (MY108_ARATH) (Fig. 5b). In addition, 7 transcripts with a predicted Tify domain were also up-regulated (PF06200.11; Fig. 5b, Supplemental Table S4). Tify-domain containing proteins are known JA-related transcription factors like TI10A_ARATH, reinforcing the probable activation of the JA signaling pathway upon feeding^64^. This was further illustrated by the presence of transcripts displaying putative AP2 domains and homology to the ORCA3 transcription factor, a key component of the JA-induced MIA biosynthesis^39^. Interestingly, orthologs of the jasmonate biosynthesis genes AOC4_ARATH and LOX5_ARATH were also found in the 20 genes common to the five gene lists, thus highlighting the prominence of JA biosynthesis in the periwinkle response to herbivory. This phenomenon was confirmed by quantification of oxophytodienoic acid (OPDA), JA and its active form jasmonoyl-isoleucine (JA-Ile) in *C. roseus* leaves, 24 h and 48 h after their intial consumption by *M. sexta* (performed during 2 hours as for other analyses; Fig. 6). In both cases, higher amounts of these compounds were detected in damaged leaves showing that synthesis of JA was strongly triggered during herbivory. Such JA accumulation in attacked leaves has been already observed during *Nicotiana attenuata*/*M. sexta* interaction^65^ and suggests that the *C. roseus*/*M. sexta* interaction induces a representative response of plants to chewing insects.

In addition to JA, our analysis of *M. sexta*-induced gene expression in *C. roseus* also indicated the activation of several elements related to ethylene biosynthesis and signaling. The core set of transcripts contained a transcript homologous to 1-aminocyclopropane-1-carboxylate oxidase (ACCO_ACTDE), also known as the ethylene-forming enzyme^66^. Furthermore, we observed the up-regulation of a MAPKK (M2K9_ARATH) related to the MPK3/MPK6 signaling pathway that leads to the phosphorylation of ETHYLENE INSENSITIVE3 (EIN3) to trigger its activation resulting in the transactivation of its target genes^67^. Consistently, transcription factors related to ethylene signaling were also found in the same lists such as ERF08_ARATH and RAP24_ARATH. In this respect, we found that the regulatory motif GCCGC(C/G) was significantly enriched in the promoters of the intersections between lists of up-regulated transcripts at 8 and 24 h (Supplemental Figure S4a). This motif was reported to be an important binding site of ERF transcription factors^68^. Such activation could illustrate the ethylene burst occurring after folivory by *M. sexta* that could be involved in the cross talk with jasmonate signaling known to mediate plant responses to herbivore interactions^35^,^36^.

### Herbivory sequentially activated two sets of MIA biosynthetic genes

Consistent with the huge modification of the MIA content of *C. roseus* plants challenged with *M. sexta* larvae (Fig. 3), the expression of transcripts associated with MIA metabolism was strongly altered. The Uniprot keyword “Alkaloid metabolism” was significantly (7 transcripts, *p* = 1e–08) enriched in the core set of up-regulated transcripts (Fig. 4c, Supplemental Tables S1 and S3). This set also contained transcripts representing 3 steps (TRPE_ARATH, TRPD_ARATH and TRPA2_ARATH) out of 5 for tryptophan biosynthesis (pathway significantly enriched, *p* = 1e–07) which leads to tryptamine, the indole MIA precursor. Many of the previously known MIA biosynthetic genes were similarly identified in the global gene lists (Fig. 5c).

**Figure 1:**
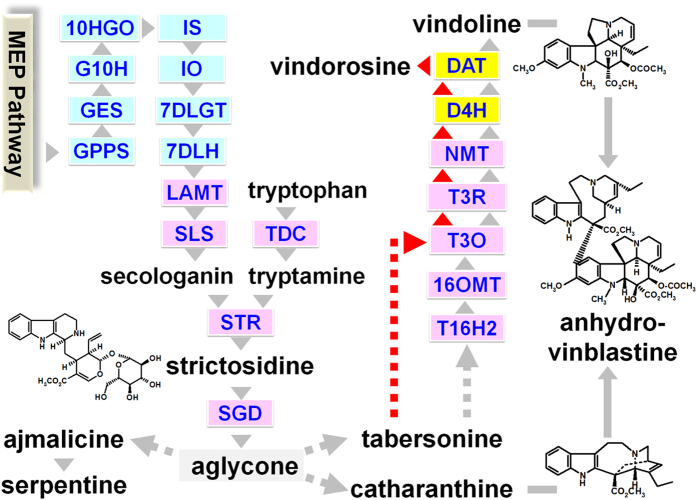
Biosynthetic pathway of MIAs in *C. roseus*. Simplified representation of the MIA biosynthesis in *C. roseus* leaves highlighting the cellular organization of the pathway in internal phloem associated parenchyma (blue rectangles), epidermis (pink rectangles), laticifers/idioblastes (yellow rectangles). Known single enzymatic steps are indicated by grey/red arrowheads and abbreviation of enzyme names. Broken grey/red arrows indicate unknown enzymatic steps. Conversion of tabersonine may occur in two ways to generate vindorosine (red arrows) or vindoline (grey). MEP, methyl-D-erythritol phosphate; GPPS, geranyl diphosphate synthase; GES, geraniol synthase; G10H, geraniol 10-hydroxylase; 10HGO, 10-hydroxygeraniol oxidoreductase; IO, iridoid oxidase; IS, iridoid synthase; 7DLGT, 7-deoxyloganetic acid glucosyltransferase; 7DLH, 7-deoxyloganic acid 7-hydroxylase; LAMT, loganic acid O-methyltransferase; SLS, secologanin synthase; TDC, tryptophan decarboxylase; STR, strictosidine synthase; SGD, strictosidine β-glucosidase; T16H2, tabersonine 16-hydroxylase 2; 16OMT, 16-hydroxytabersonine O-methyltransferase; T3O, tabersonine 3-oxidase; T3R, tabersonine 3-reductase; NMT, 16-methoxy-2,3-dihydrotabersonine N-methyltransferase; D4H, desacetoxyvindoline 4-hydroxylase; DAT, deacetylvindoline 4-O-acetyltransferase.

**Figure 2:**
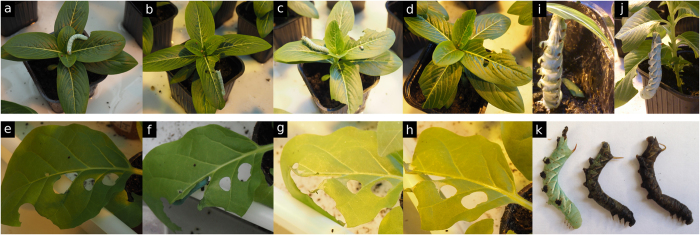
*Manduca sexta* larvae feeding of *C. roseus* (**a–d**) and *N. tabacum* (**e–h**) leaves. Pictures were taken 2 h (**a,e**), 24 h (**b,f**), 48 h (**c,g**) and 72 h (**d,h**) after placing caterpillars on leaves. (**i,j**) Typical morphologic alteration of caterpillars feeding on *C. roseus*, hanging by their softened end. k, browning phenotypes usually obtained after 72 h of feeding.

**Figure 3:**
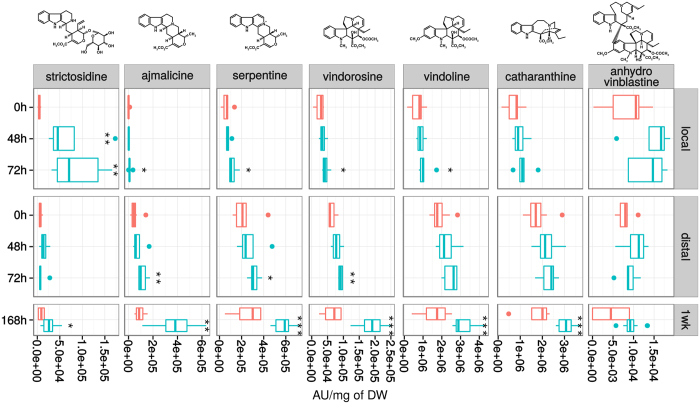
Monoterpene Indole Alkaloid (MIA) accumulation in *C. roseus* leaves fed by *M. sexta*. MIA accumulation was monitored 48 h and 72 h after an initial 2 h feeding period, as well as in newly emerged leaves one week after (168 h). Asterisks denote significance levels at *p < 0.05, **p < 0.01 and ***p < 0.001 (non parametric Wilcoxon rank sum test). Red boxes, control (independent, intact plants; see Supplemental Figure S2a); blue boxes, after feeding with *M. sexta*. AU, arbitrary unit normalized by sample mass.

Interestingly, these transcripts displayed a 2-step induction profile directly correlated with the variation of the leaf MIA content. The pronounced accumulation of strictosidine observed 48 h and 72 h after leaf attack was indeed preceded by a strong induction of the epidermis gene set ensuring LAMT, SLS1, STR and SGD gene expression. Indeed the 4 corresponding transcripts were commonly found upregulated in Ms6h, Ms8h and MsDamaged24 h (Fig. 5c). Such induction of gene expression was confirmed by qPCR analyses as illustrated for SLS1, STR and SGD (Fig. 7). Interestingly, expression of genes related to preceding reactions of biosynthesis localized in IPAP cells (from the MEP pathway genes to 7DLH) did not display such high induction suggesting a restriction of the response to epidermis expressed genes (Figs 1 and 5c). Similarly, no marked induction of genes catalyzing later steps of the MIA pathway were observed in agreement with the absence of vindoline, catharanthine or ajmalicine induction 72 h after leaf attack (Figs 2 and 5C). On the other hand, the induction of STR expression can be explained by the increase in JA biosynthesis probably leading to the induction of ORCA3 expression that has been previously described to transactivate the STR promoter by binding to conserved motif in its target gene promoters^40^,^69^. We found that this motif, CACGTG was significantly (*e*-value = 2e–008) represented in the promoters of the core set of induced genes thus confirming the prominent role of ORCA3 in plant response to herbivory (Supplemental Figure S4b). Interestingly, we also observed the local induction of the MATE transporter (CRO_T006097 – encoded by SRR342023_TR31426_c4_g1_i1_len = 2080) located close to STR in the *C. roseus* genome, which may reinforce its proposed involvement in the transport of MIA precursors^70^. Altogether, the concomitant dramatic accumulation of strictosidine and strong induction of SGD (Figs 3 and 5c) are in good agreement with the “nuclear time-bomb” defense system hypothesis. The potential massive formation of the reactive strictosidine aglycon upon deglucosylation ensuing might cause protein reticulation limiting leaf digestibility and/or damages in the early phase of the infestation as observed on *M. sexta* dissected guts (Fig. 2, Supplemental Figure S1c).

**Figure 4:**
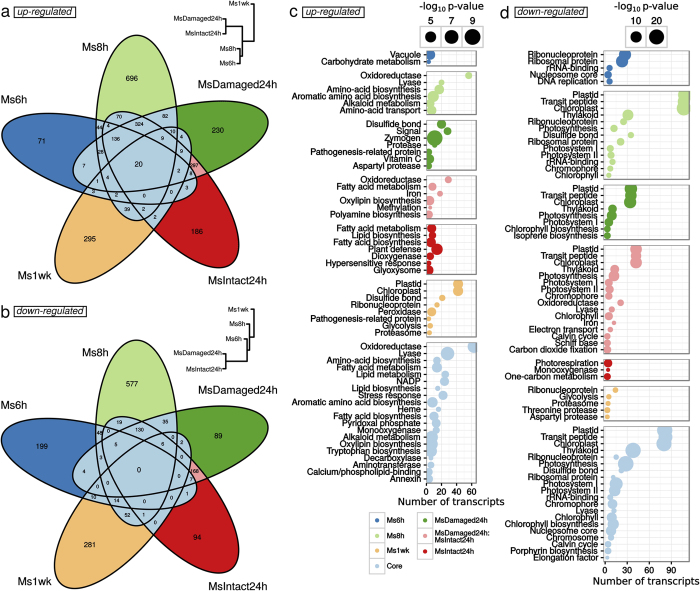
Functional classification of differentially expressed transcripts in leaves of *C. roseus* fed by *M. sexta* compared to undamaged control leaves. For each of the 5 comparisons ((Ms6h/Ctrl6h, Ms8h/Ctrl8h, MsDamaged24h/Ctrl24h, MsIntact24h/Ctrl24h and Ms1wk/Ctrl1wk), lists of significantly (*p* < 0.001) up-regulated (**a,c**) or down-regulated transcripts (**b,d**) were analyzed with Venn diagrams (**a,b**). In **a** and **b**, hierarchical clustering trees depict dissimilarities between samples calculated on the 2,588 and 1,745 transcripts that were respectively significantly up-regulated or down-regulated in at least one comparison. Intersections between these lists were used to identify core sets of herbivory-modulated transcripts (modulation at least at two different sampling times) clear blue sectors) and sample-specific transcripts (other sectors). The pink sectors correspond to transcripts commonly modulated both in MsDamaged24h and MsIntact24h and were therefore not included in the core sets. (**c**,**d**) Uniprot keyword classification of lists of differentially accumulated transcripts. The number of transcripts is indicated for each term by a dot in which the diameter is proportional to the *p*-value of enrichment testing (Hypergeometric distribution). Only keywords attributed to more than 2 transcripts and that were significantly enriched (Hypergeometric distribution, *p*-value < 0.001) are represented. Lists of up- and down-regulated transcripts are available in Supplementary Tables S1 and S2 respectively. Details for Uniprot keyword analysis are presented in Supplementary Table 3.

**Figure 5:**
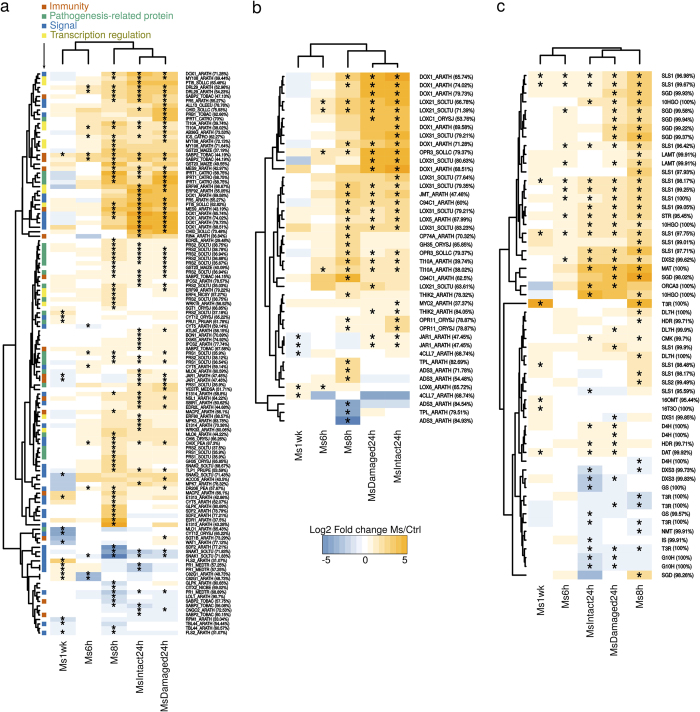
Thematic representation of *C. roseus* transcripts related to defense, jasmonate and MIA metabolisms. Transcripts with significant log2 fold changes (fed vs control) in at least one comparison were clustered according to their expression profiles. (**a**,**b**) Transcripts associated with “Plant defense” and “Oxylipin”/“Jasmonic acid” Uniprot keywords respectively. Transcripts from CDF97 assembly were annotated by searching homologies (Blastx, *e*-value < 1e–10) against the Uniprot database (the % of identity is indicated between brackets). Transcripts associated with the keyword “Plant defense” were further grouped according to the other keywords they were annotated with, i.e. Pathogenesis-Related proteins, Signal, Immunity and Transcription regulation. (**c**) Transcripts homologous to known MIA genes (blast score > 1100, %id >95%). Asterisks indicate that the expression of the corresponding transcript significantly differed between the attacked and the control samples (Linear model, *p*-value < 0.001).

**Figure 6:**
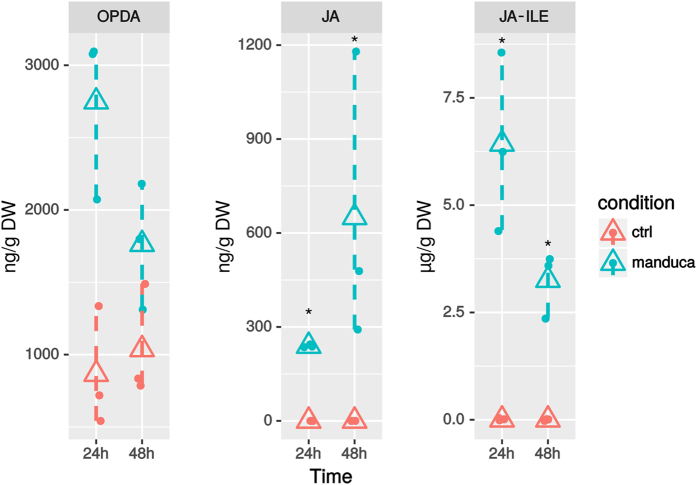
Oxylipin accumulation in *C. roseus* leaves fed by *M. sexta* after 24 and 48 h. JA, jasmonic acid; OPDA, oxo-phytodienoic acid; JA-Ile, Jasmonoyl-isoleucine. Triangles indicate arithmetic mean and bars correspond to confidence limits (non-parametric bootstrap). Asteriks denote significant differences between fed and control leaves (Wilcoxon rank sum test, *p*-value < 0.05, n = 3).

**Figure 7:**
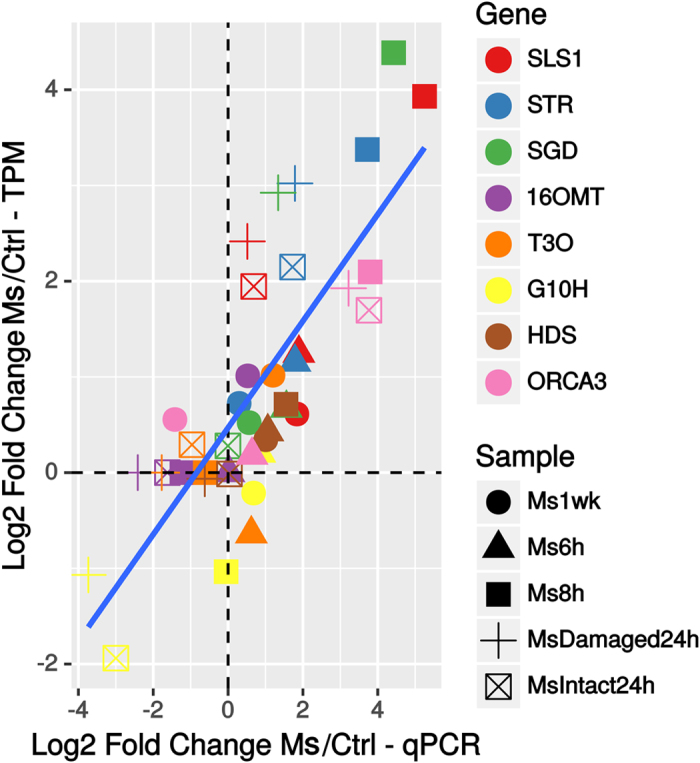
Comparison of expression levels (log2) obtained by qPCR and RNA-seq (Transcripts per Million) for candidate genes. A regression line (blue) and its confidence intervals (95%, shaded) are depicted.

The second step of the MIA metabolism variation was observed in newly developed leaves, one week after attack (Ms1wk) and was characterized by the induction of a distinct set of genes (Fig. 4a, Fig. 5c, Supplemental Table S1). While the induction of expression of SLS, STR and SGD was not sustained, up-regulation of downstream genes of the pathway was observed and notably for those involved in vindoline and vindorosine biosynthesis including 16OMT (for vindoline only), T3O and T3R. This induction, validated by qPCR analysis, might have caused the increase of the vindoline amount in newly developed leaves reflecting the systemic response deployed by *C. roseus* against *M. sexta* (Fig. 7). While no molecular explanation of the increase of the other MIAs can be provided now, it is tempting to hypothesize that some of the missing biosynthetic genes could be retrieved from this analysis.

## Conclusion

**Figure 8:**
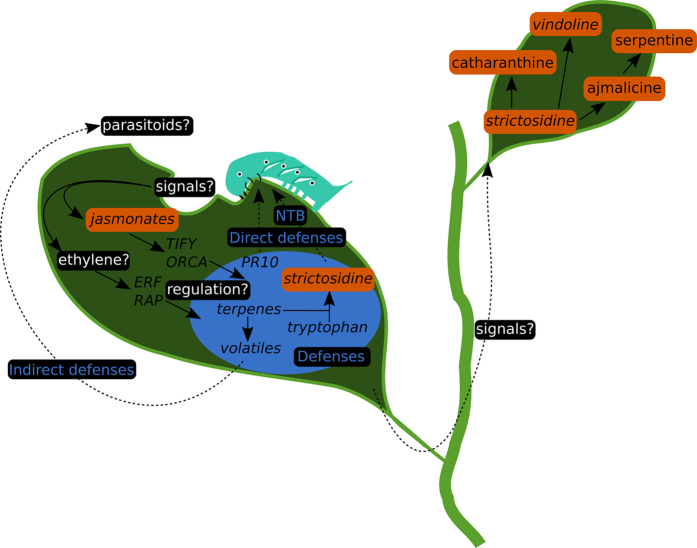
Molecular events associated with folivory in *C. roseus*. Orange boxes, supported by compound measurements, italics, supported by gene expression measurements. Potential signals in the oral secretions of *M. sexta* together with damage-associated molecular patterns are likely to activate jasmonate and signaling pathways in *C. roseus*. Those two pathways might respectively use TIFY/ORCA and ERF/RAP transcription factors to control the activation of sets of potential defenses (within blue circle). Strictosidine may interfere with caterpillars by acting as a Nuclear Time Bomb (NTB). NBT involves a massive production of strictosidine aglycone, an efficient protein-cross-linker,by the nucleus-localized SGD, following vacuole disruption and the resultant release of strictosidine. Still unknown signals may control the increased biosynthesis of MIA in distal, newly formed leaves.

Despite the fact that MIA biosynthesis and toxicity have been studied for more than 50 years, still little is known about the role of these compounds in Madagascar periwinkle against biotic attacks. By studying the interaction between *M. sexta* and *C. roseus* using targeted metabolic and transcriptome analyses, we provided compelling evidence of the activation of the JA and ethylene signaling pathways and of distinct plant defense processes (Fig. 8). One of the main features of the folivory response was the local and systematic activation of MIA metabolism that relied on the induction of specific MIA biosynthetic gene subsets and differential MIA production (Figs 3 and 5c). The huge local accumulation of strictosidine combined with SGD expression may illustrate the importance of the ‘nuclear time bomb’ mechanism and the reactive strictosidine aglycone as a first barrier against aggressors whilst the synthesis of downstream MIAs in newly developing leaves may ensure an enhanced protection to overcome later attacks. In this context, leaf consumption resulted in quick and marked effects on caterpillar health status leading to death (Fig. 2i–k). Interestingly, analyses of the intestinal tracts of dying larvae compared to leaves revealed the main presence of anhydrovinblastine suggesting that catharanthine and vindoline dimerization only occurred after leaf consumption (Supplemental Figure S1d). Finally, the strong induction of MIA biosynthetic gene expression upon herbivory suggests that our RNA-seq data will constitute a new valuable resource to pursue the identification of missing genes involved in these complex metabolic pathways.

## Methods

### Feeding of *M. sexta* larvae on *C. roseus* leaves

Seeds of *C. roseus* (Apricot Sunstorm cultivar, B and T world seeds, Aigues Vives, France) were germinated in a greenhouse and the resulting plants were grown in individual pot at 28 °C under a 16 h light/8 h dark cycle for eight weeks. *M. sexta* larvae were reared on artificial diet at 27 °C under 16 h light/8 h dark photoperiod and 70 ± 5% relative humidity until reaching the 3^rd^ instar. For transcriptomics and metabolic analysis young caterpillars were laid on leaves in the morning and allowed to feed for 2 hours before being removed as depicted in Supplemental Figure S2a–c.

### Weight gain measurements, choice experiment and larvae analysis

For the weight gain experiment, larvae were allowed to feed continuously on *N. tabacum* and *C. roseus* plants at 23 °C. Individual caterpillars were identified, initially weighted and laid on individual plants. Data were recorded 24, 48, and 72 h after the beginning of the experiment. For choice experiment, larvae were fed with 2.2 cm-diameter leaf disks of *C. roseus* and *N. tabacum* placed on wet filter paper in 15 cm-diameter Petri dishes. Leaf disk consumption was monitored during 120 min and the experiment was repeated twice.

### RNA extraction and sequencing

RNAs were extracted from leaves with Trizol (Life Technologies) following the manufacturer’s recommendations with slight modifications. After precipitating with isopropanol and washing with 70% ethanol, RNA pellets were re-suspended in 100 μL of RNase free water and remaining sugars were precipitated by adding 10% ethanol (final concentration) and incubating 5 minutes at 4 °C. The supernatant obtained after centrifugation (5 min at 15,000 g) was further precipitated by addition of 0.1 volume of 3 M sodium acetate pH 5.2 and 2.5 volume of 100% ethanol for 2 h at −20 °C. The tubes were centrifuged 15 minutes at 12,000 g and 4 °C and the resulting pellet was washed with 70% ethanol and re-suspended in RNAse-free water. RNA concentration was estimated with a Nanodrop spectrophotometer (Thermo). A total of 9 transcriptomes (Ms6h/Ctrl6h, Ms8h/Ctrl8h, MsDamaged24h/Ctrl24h, MsIntact24h/Ctrl24h and Ms1wk/Ctrl1wk) were sequenced as single replicates by Eurofins Genomics using the Illumina HiSeq2000/2500 technology. Samples were sequenced in the paired-end mode (2 × 100 pb). The resulting fastq files were cleaned with Trimmomatic with default parameters (using TruSeq3 primer sequence). For quantification of transcript accumulation, reads were pseudo-aligned on CDF97 reference transcript sequences^17^ and counted with Salmon^55^ in the variational bayesian optimized (–vbo) quasi-mapping mode with biase correction (–biasCorrect). The resulting quant.sf files were combined and processed with R. Differentially accumulated transcripts were identified by considering each experiment as unique (without replicate) and by fitting a linear model to each gene with the ‘exactTest’ function of the edgeR Bioconductor package^71^. Biological variability was estimated by setting the square-root dispersion at 0.4. To balance the absence of biological replicates, differentially expressed genes were set if the *p*-value in the test was below 0.001. In addition, functions altered in response to folivory were focused on genes that were significantly modulated in at least two comparisons.

### Annotation and term enrichment analysis

The CDF97 reference transcriptome was annotated with the Trinotate v3.0 pipeline against Uniprot (Blastx and Blastp on Transdecoder predicted ORFs) and PFAM (hmmscan) databases. Uniprot keywords were retrieved by using Uniprot predicted homologs in CDF97. Enrichment tests of functional terms were performed by comparing effectives to a hypergeometric distribution (phyper function in R). All graphics were made with ggplot2 package. For promoter analysis, scaffolds and predicted CDS of *C. roseus* genome sequencing project^70^ were retrieved from Dryad (http://datadryad.org/resource/doi:10.5061/dryad.hs593). CDS were mapped on scaffolds using megablast (BLAST + suite 2.2.29^72^). When possible the 500 pb upstream the start codon were obtained for each CDS. The MEME suite (v4.11.2) was used to detect new ungapped motifs with DREME program using default parameter^73^. GOMO was next used to analyze the representation of candidate motifs in Arabidopsis genome.

### qPCR analysis

Targeted gene expression measurement was performed by qPCR using primers described in Supplemental Table S5, after cDNA synthesis as described previously^17^.

### MIA quantification

MIAs were extracted from lyophilized samples (C. roseus leaves and M. sexta intestinal tracts) by grinding tissues with a mixer mill (Restch, MM 400) during 3 min at the maximal frequency. The resulting powders were incubated in 1 ml methanol (containing 0.1% formic acid) and under vigorous shaking during 1 hour at 24 °C. After centrifugation (15,000 g; 15 minutes), supernatants were collected and used for quantification.

The MIA content of *C. roseus* leaves and of *M. sexta* intestinal tracts were determined using an UPLC-MS chromatography system coupled to a SQD mass spectrometer equipped with an electrospray ionization (ESI) source controlled by Masslynx 4.1 software (Waters, Milford, MA). Analyte separation was performed on a Waters Acquity HSS T3 C18 column (150 mm × 2.1 mm, i.d. 1.8 μm) with a flow rate of 0.4 mL/min at 55 °C and the volume of injection was 5 μL. The following linear elution gradient was used: acetonitrile-water-formic acid from 10:90:0.1 to 50:50:0.1 over 5 min. The capillary and sample cone voltages were 3,000 V and 30 V, respectively. The cone and desolvation gas flow rates were 60 and 800 Lh − 1. MS experiments were carried out in positive mode in the selected ion-monitoring mode using m/z 337 for catharanthine ([M + H]+, RT = 12.33 min), m/z 457 for vindoline ([M + H]+, RT = 14.69 min), m/z 793 for anhydrovinblastine ([M + H]+, RT = 16.5 min), m/z 427 for vindorosine ([M + H]+, RT = 15.03 min), m/z 353 for ajmalicine ([M + H]+, RT = 11.7 min), m/z 531 for strictosidine ([M + H]+, RT = 10.39 min), m/z 349 for serpentine ([M + H]+, RT = 13.01 min). The acquired data was processed by the QuanLynx™ software (Waters, UK). Relative quantification was performed by correcting peak areas by sample masses.

### Quantification of oxophytodienoic acid, jasmonic acid, and jasmonoyl-isoleucine

Samples stored at −80 °C were freeze dried and powdered for subsequent analysis. Thirty milligrams of freeze dried powder were extracted at 4 °C with 1 ml of H2O:MeOH (90:10) containing 100 ng/ml of internal standards. After 20 min of incubation, samples were centrifuged at full speed for 15 min at 4 °C. The supernatant was recovered and adjusted to pH 2.8 with 6% acetic acid, and subsequently partitioned twice against diethylether. The fractions were pooled and dried in a speed vacuum and resuspended in H2O:MeOH (90:10). A 20 μl aliquot was injected into an Acquity ultra-performance liquid chromatography system (UPLC) (Waters, Mildford, MA, USA) interfaced to a triple quadrupole mass spectrometer (TQD, Waters, Manchester, UK). The LC separation was performed by HPLC Kinetex C18 analytical column 5 μm particle size, 2.1 × 100 mm (Phenomenex). The chromatographic conditions and mass spectrometry were performed as described previously^74^.

### Statistical procedures

Statistical differences between means were non-parametrically tested in R^75^. The Wilcoxon rank sum test was used to avoid any conflict with the distribution of data. This was particularly the case for peak area in the metabolic analysis which displayed heteroscedasticity. P-values of tests were corrected with False Discovery Rate. Specific statistical procedures for RNA-seq data are presented above. Graphics were made with the ‘ggplot2’ package^76^.

## Additional Information

**How to cite this article:** Dugé de Bernonville, T. *et al*. Folivory elicits a strong defense reaction in *Catharanthus roseus*: metabolomic and transcriptomic analyses reveal distinct local and systemic responses. *Sci. Rep.* **7**, 40453; doi: 10.1038/srep40453 (2017).

**Publisher's note:** Springer Nature remains neutral with regard to jurisdictional claims in published maps and institutional affiliations.

## Supplementary Material

Supplementary Information

## Figures and Tables

**Table 1:** Description of samples deposited in EBI ENA under accession number PRJEB14626.

Sample name	Sample description	Sample accession	Read counts	Percent of reads mapped on CDF97
Ms6h	Manduca damaged leaves 6 h	ERR1512369	23,772,392	98.81%
Ctrl6h	Leaves from control plants 6 h	ERR1512370	27,591,053	98.82%
Ms8h	Manduca damaged leaves 8 h	ERR1512371	27,774,796	98.93%
Ctrl8h	Leaves from control plants 8 h	ERR1512372	34,082,314	98.87%
MsDamaged24h	Manduca damaged leaves 24 h (damaged part of the leaf)	ERR1512372	31,252,515	98.88%
MsIntact24h	Manduca damaged leaves 24 h (intact part of the leaf)	ERR1512372	18,495,538	98.84%
Ctrl24h	Leaves from control plants 24 h	ERR1512372	30,061,186	98.97%
Ms1wk	New leaf from Manduca damaged plant after 1 week	ERR1512376	27,394,833	98.63%
Ctrl1wk	New leaf from control plant after 1 week	ERR1512376	27,226,966	98.68%

**Table 2:** Number of differentially accumulated transcripts.

Compared conditions	Time	Up-regulated (Log2 fold change >0)	Down-regulated (Log2 fold change <0)
Ms6h/Ctrl6h	6 h	333	291
Ms8h/Ctrl8h	8 h	1,470	890
MsDamaged24h/Ctrl24h	24 h	1,171	449
MsIntact24h/Ctrl24h	24 h	1,072	431
Ms1wk/Ctrl1wk	1 week	402	366
