# Supplementary material for: Folivory elicits a strong defense reaction in Catharanthus roseus: metabolomic and transcriptomic analyses reveal distinct local and systemic responses
Source: Sci Rep. 2017 Jan 17;7:40453. doi: 10.1038/srep40453 (PMC5240345; doi:10.1038/srep40453)
Supplement: Supplementary Information [file srep40453-s1.pdf]

Supplementary Information for *Folivory elicits a strong defense reaction in Catharanthus roseus: metabolomic and transcriptomic analyses reveal distinct local and systemic responses*

Thomas Dugé de Bernonville1*, Inês Carqueijeiro1*, Arnaud Lanoue1, Florent Lafontaine1, Paloma Sánchez Bel2, Franziska Liesecke1, Karine Musset3, Audrey Oudin1, Gaëlle Glévarec1, Olivier Pichon1, Sébastien Besseau1, Marc Clastre1, Benoit St-Pierre1, Victor Flors2, Stéphane Maury4, Elisabeth Huguet3, Sarah E. O'Connor5, Vincent Courdavault1#

*These authors have contributed equally to this work.

1Université François-Rabelais de Tours, EA2106 "Biomolécules et Biotechnologies Végétales", Tours, France.

2Metabolic Integration and Cell Signaling Group, Plant Physiology Section, Department of CAMN, Universitat Jaume I, Spain.

3Institut de Recherche sur la Biologie de l'Insecte, UMR 7261, CNRS/Université François-Rabelais de Tours, Tours, France.

4Université d'Orléans, CoST, Laboratoire de Biologie des Ligneux et des Grandes Cultures (LBLGC), EA 1207, USC1328 INRA, Orléans, France.

5 The John Innes Centre, Department of Biological Chemistry, Norwich NR4 7UH, United Kingdom.

#Corresponding author :

Vincent Courdavault

Université François-Rabelais de Tours - EA2106 "Biomolécules et Biotechnologies Végétales", UFR Sciences et Techniques, 37200, Tours, France

Tel :+33 (0) 247367023

e-mail: vincent.courdavault@univ-tours.fr

This pdf file contains internal links which will help you browsing the present Supplementary Information.

Content of the Supplementary Information:

Supplemental Figure S1: M. sexta is able to feed on C. roseus. A, preferential choice assay. Caterpillars were laid besides N. tabacum (Nta) and C. roseus (Cro) leaf discs. Pictures were taken 5' (left) and 120' (right) after the beginning of the experiment. B, Larval weights obtained 24h, 48h et and 72h after continuous feeding on N. tabacum and C. roseus. Pictures on the right show typical consumed areas on each plant after 2h (top) and 24h (down). C, Left panel, M. sexta larvae dissected after 24h of feeding on N. tabacum (left) or C. roseus (right) showing the presence of plant tissues in the intestinal tract. Right panel, dissection of caterpillar after 72h feeding on C. roseus showing intense browning of the intestinal tract. D, Targeted LC-MS analysis of catharanthine, vindoline and anhydrovinblastine in leaf tissues and caterpillar intestinal tract (sampled on the larva shown in right panel of C).
Supplemental Figure S2: Experimental design to test MIA accumulation in leaves of C. roseus during herbivory. a, design for metabolic analysis. b, comparison of caterpillar and mechanical wounding effects – During 2 hours, leaves were subjected to caterpillar attack or to mechanical wounding (performed with a clamp every 10 min to mimic the chewing effect – similar area of leaves were removed). 48h and 72h post-treatment, leaves were collected and strictosidine quantified. c, design for transcriptomic analyses.
Supplemental Figure S3: PFAM classification of transcripts significantly modulated in leaves of C. roseus fed by M. sexta. Lists of differentially expressed transcripts were obtained for individual experiments (Figure 4). The number of transcripts is indicated for each domain by a dot which diameter is proportional to the p-value of enrichment testing (Hypergeometric distribution). Only PFAM attributed to more than 4 transcripts are represented. a, classification of up-regulated transcripts, b, classification of down-regulated transcripts.
Supplemental Figure S4: Motifs significantly enriched in the 300bp promoters in up-regulated transcripts. Motifs were discovered with DREME from MEME suite. a, enrichment of GCCGCC box in transcripts commonly up-regulated in at least 3 samples. b, enrichment of CACGTG box in the core set of up-regulated transcripts. For each motif, gene ontology (GO) term represented among Arabidopsis genes containing the corresponding motif are reported (processed with GOMO).
Supplemental Table S1. Description of transcripts up-regulated upon challenge with M. sexta found in the intersections between lists of each experiment.
Supplemental Table S2. Description of transcripts down-regulated upon challenge with M. sexta found in the intersections between lists of each experiment.
Supplemental Table S3. Most represented Uniprot keywords in transcripts modulated upon challenge with M. sexta. Enrichment tests were performed by comparison with a hypergeometric distribution.
Supplemental Table S4. Most represented PFAM domains Uniprot keywords in transcripts modulated upon challenge with M. sexta. Enrichment tests were performed by comparison with a hypergeometric distribution.
Supplemental Table S5. Primers used in this study.

Supplemental Figure S1

M. sexta is able to feed on C. roseus. A, preferential choice assay. Caterpillars were laid besides N. tabacum (Nta) and C. roseus (Cro) leaf discs. Pictures were taken 5' (left) and 120' (right) after the beginning of the experiment. B, Larval weights obtained 24h, 48h et and 72h after continuous feeding on N. tabacum and C. roseus. Pictures on the right show typical consumed areas on each plant after 2h (top) and 24h (down). C, Left panel, M. sexta larvae dissected after 24h of feeding on N. tabacum (left) or C. roseus (right) showing the presence of plant tissues in the intestinal tract. Right panel, dissection of caterpillar after 72h feeding on C. roseus showing intense browning of the intestinal tract. D, Targeted LC-MS analysis of catharanthine, vindoline and anhydrovinblastine in leaf tissues and caterpillar intestinal tract (sampled on the larva shown in right panel of C).

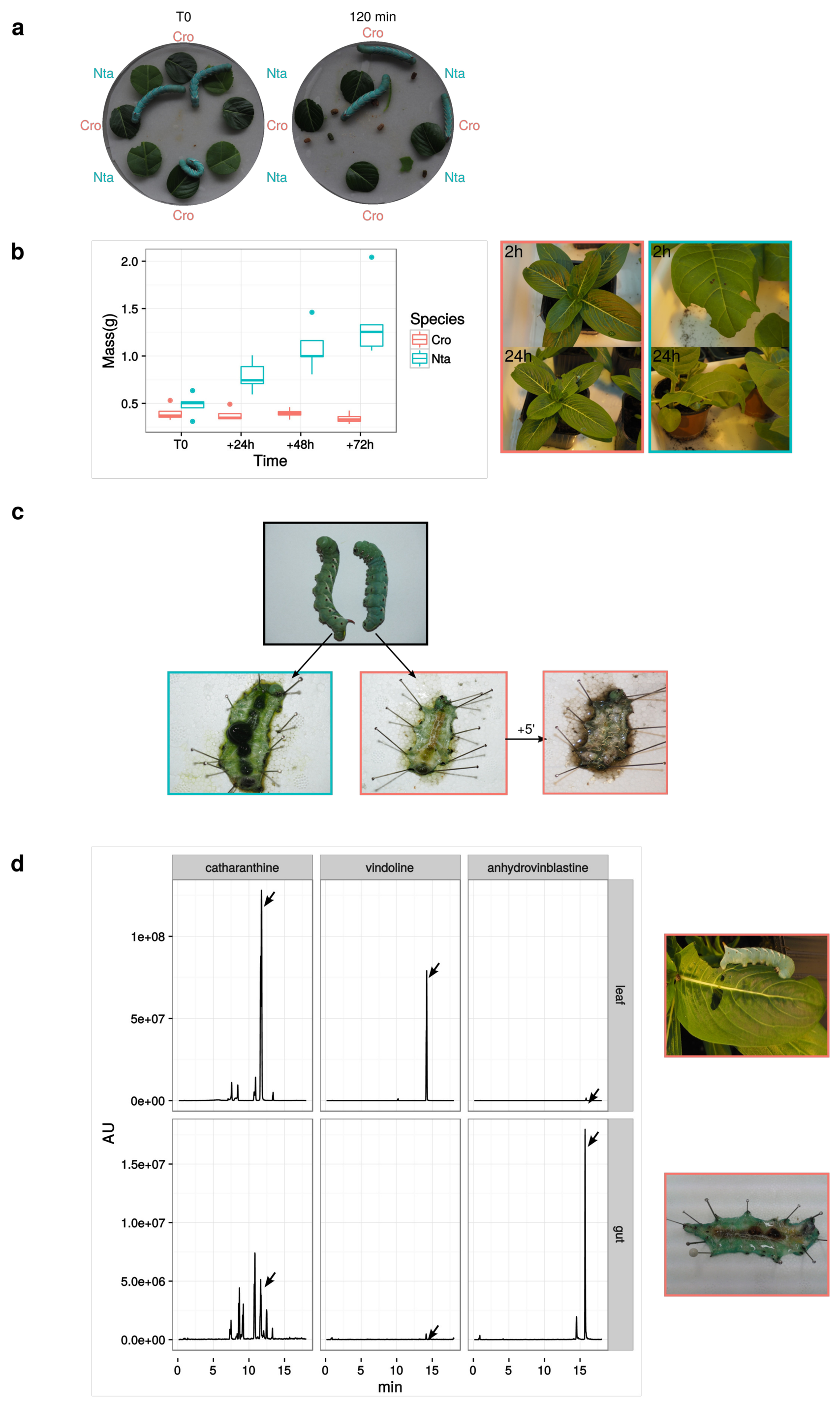

Supplemental Figure S2

Experimental design to test MIA accumulation in leaves of C. roseus during herbivory. a, design for metabolic analysis. b, comparison of caterpillar and mechanical wounding effects – During 2 hours, leaves were subjected to caterpillar attack or to mechanical wounding (performed with a clamp every 10 min to mimic the chewing effect – similar area of leaves were removed). 48h and 72h post-treatment, leaves were collected and strictosidine quantified. c, design for transcriptomic analyses.

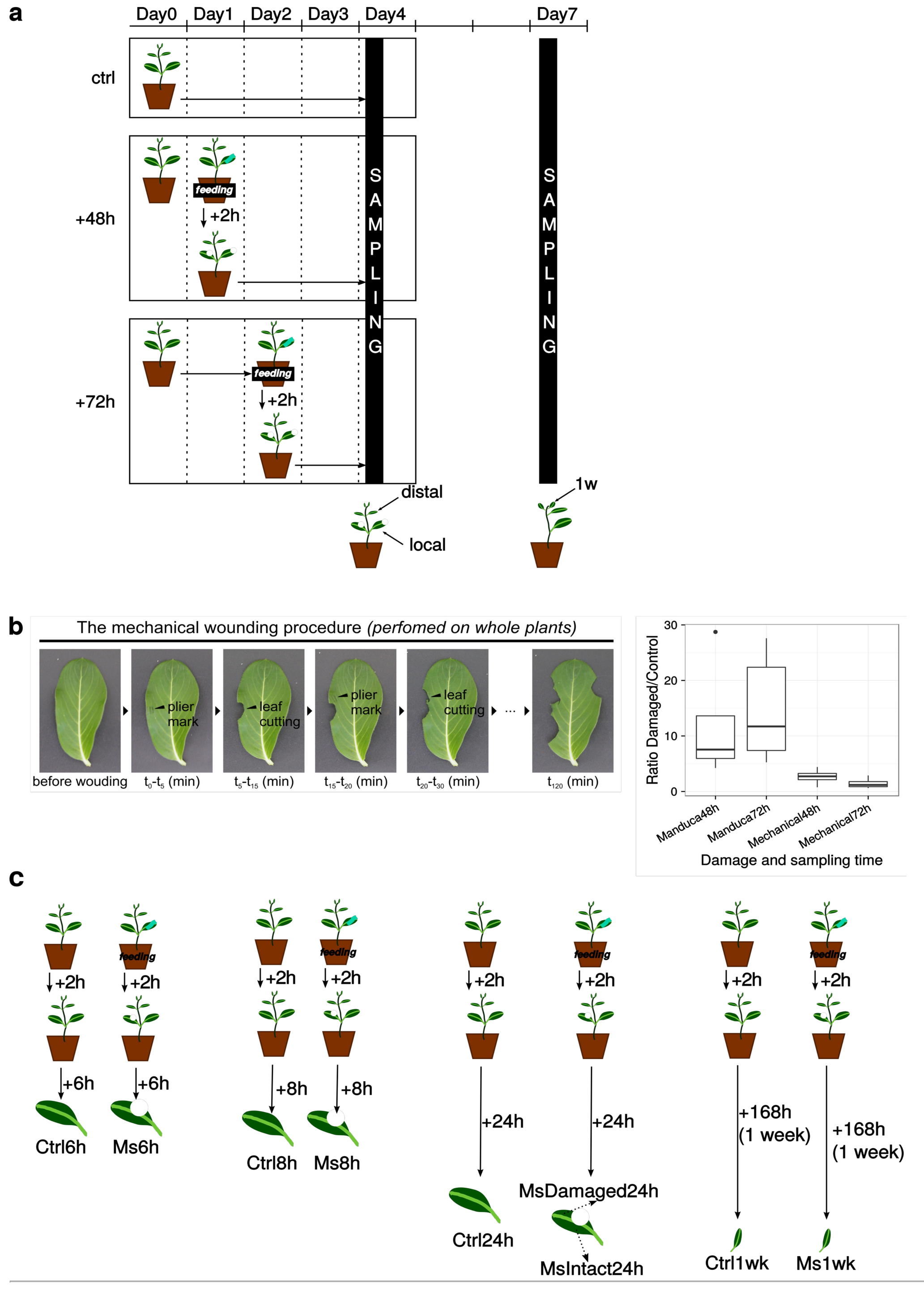

Supplemental Figure S3

PFAM classification of transcripts significantly modulated in leaves of C. roseus fed by M. sexta. Lists of differentially expressed transcripts were obtained for individual experiments (Figure 4). The number of transcripts is indicated for each domain by a dot which diameter is proportional to the p-value of enrichment testing (Hypergeometric distribution). Only PFAM attributed to more than 4 transcripts are represented. a, classification of up-regulated transcripts, b, classification of down-regulated transcripts

a

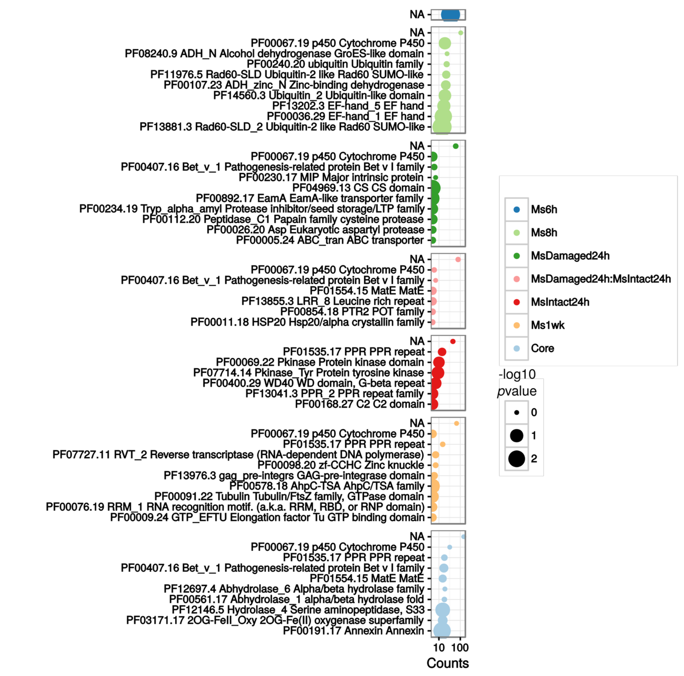

b

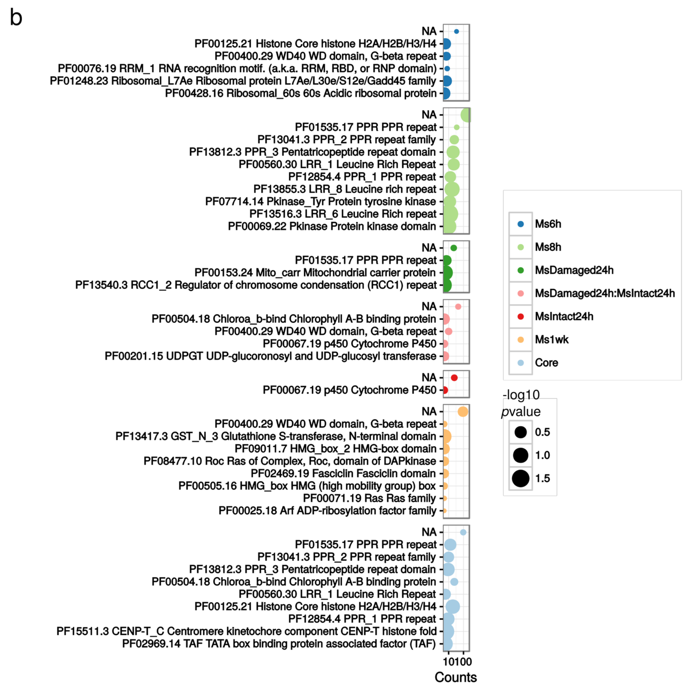

Supplemental Figure S4

Motifs significantly enriched in the 300bp promoters in up-regulated transcripts. Motifs were discovered with DREME from MEME suite. a, enrichment of GCCGCC box in transcripts commonly up-regulated in at least 3 samples. b, enrichment of CACGTG box in the core set of up-regulated transcripts. For each motif, gene ontology (GO) term represented among Arabidopsis genes containing the corresponding motif are reported (processed with GOMO).

a

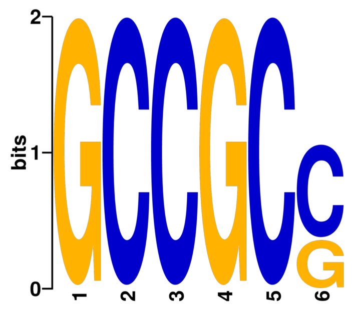

b

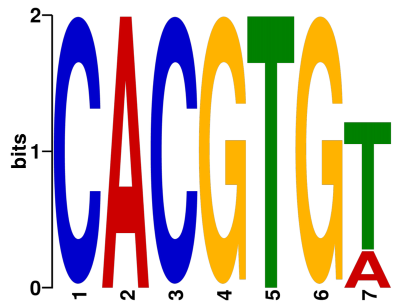

Supplemental Table S1

Description of transcripts up-regulated upon challenge with M. sexta found in the intersections between lists of each experiment.

Core
Ms1wk
Ms6h
Ms8h
MsDamaged24h
MsDamaged24h_MsIntact24h
MsIntact24h

Core

Transcript (CDF97)	Uniprot homolog	BLASTX Position	BLASTX %Identity	BLASTX evalue	Uniprot descr	Pfam	PValue_Ms6h	logFC_Ms6h	PValue_Ms8h	logFC_Ms8h	PValue_MsDamaged24h	logFC_MsDamaged24h	PValue_MsIntact24h	logFC_MsIntact24h	PValue_Ms1wk	logFC_Ms1wk	Entry.name	Protein names	Gene names	Organism	Pathway	Keywords
SRR342017_TR6167_c0_g1_i1_len=1094	PNAE_RAUSE	Q:129-881,H:12-262	45.74%ID	E:1e-63	Polyneuridine-aldehyde esterase	PF12146.5^Hydrolase_4^Serine aminopeptidase, S33^10-119^E:6e-07`PF00561.17^Abhydrolase_1^alpha/beta hydrolase fold^14-123^E:3.2e-09`PF12697.4^Abhydrolase_6^Alpha/beta hydrolase family^15-255^E:1.4e-14	0	1,723	0,003	-1,574	0,007	-0,964	0,08	0,528	0	7,349	PNAE_RAUSE	Polyneuridine-aldehyde esterase (EC 3.1.1.78) (Polyneuridine aldehyde esterase)	PNAE	Rauvolfia serpentina (Serpentine wood) (Ophioxylon serpentinum)	PATHWAY: Alkaloid biosynthesis; ajmaline biosynthesis.	3D-structure; Alkaloid metabolism; Direct protein sequencing; Hydrolase; Serine esterase
SRR646604_TR17021_c2_g3_i5_len=1249	NA	NA	NA	NA	NA	.	0	2,047	0,681	-0,086	0,939	0,017	0,487	0,147	0	5,706	.	.	.	.	.	.
SRR648707_TR16521_c0_g2_i1_len=3328	LOX6_ARATH	Q:407-3022,H:37-917	65.72%ID	E:0	Lipoxygenase 6, chloroplastic	PF01477.20^PLAT^PLAT/LH2 domain^159-221^E:2.4e-05`PF00305.16^Lipoxygenase^Lipoxygenase^234-903^E:0	0,001	1,536	0,007	1,569	0,935	0,026	0,485	0,168	0	1,733	LOX6_ARATH	Lipoxygenase 6, chloroplastic (AtLOX6) (EC 1.13.11.12)	LOX6 At1g67560 F12B7.11	Arabidopsis thaliana (Mouse-ear cress)	PATHWAY: Lipid metabolism; oxylipin biosynthesis. {ECO:0000255|PROSITE-ProRule:PRU00726}.	Chloroplast; Complete proteome; Dioxygenase; Fatty acid biosynthesis; Fatty acid metabolism; Iron; Lipid biosynthesis; Lipid metabolism; Metal-binding; Oxidoreductase; Oxylipin biosynthesis; Plastid; Reference proteome; Transit peptide
SRR1144633_TR15074_c1_g1_i1_len=591	COPA3_ORYSJ	Q:4-591,H:106-301	95.41%ID	E:3e-132	Coatomer subunit alpha-3	PF00400.29^WD40^WD domain, G-beta repeat^2-17^E:0.0028`PF00400.29^WD40^WD domain, G-beta repeat^22-59^E:1.4e-08`PF00400.29^WD40^WD domain, G-beta repeat^94-127^E:3.7e-05`PF12894.4^ANAPC4_WD40^Anaphase-promoting complex subunit 4 WD40 domain^107-187^E:2.8e-05`PF00400.29^WD40^WD domain, G-beta repeat^136-172^E:1.6e-06	0,326	-0,356	0,001	1,996	0,09	-0,703	0,721	-0,151	0	1,831	COPA3_ORYSJ	Coatomer subunit alpha-3 (Alpha-coat protein 3) (Alpha-COP 3)	Os09g0127800 LOC_Os09g04110 OsJ_027299	Oryza sativa subsp. japonica (Rice)		Complete proteome; Cytoplasm; Cytoplasmic vesicle; ER-Golgi transport; Golgi apparatus; Membrane; Protein transport; Reference proteome; Repeat; Transport; WD repeat
SRR1144634_TR25396_c0_g1_i1_len=504	NRP2_ARATH	Q:144-485,H:1-107	70.18%ID	E:1e-44	NAP1-related protein 2	PF00956.15^NAP^Nucleosome assembly protein (NAP)^33-77^E:2.3e-07	0	-8,412	0	6,758	1	0	1	2,591	0	6,924	NRP2_ARATH	NAP1-related protein 2 (Nucleosome/chromatin assembly factor group A5) (Protein SET homolog 2)	NRP2 NFA5 At1g18800 F6A14.10	Arabidopsis thaliana (Mouse-ear cress)		Alternative splicing; Chaperone; Coiled coil; Complete proteome; Cytoplasm; Nucleus; Reference proteome
SRR122238_TR21935_c0_g1_i4_len=2255	PTR27_ARATH	Q:2085-391,H:1-555	77.35%ID	E:0	Protein NRT1/ PTR FAMILY 6.2	PF00854.18^PTR2^POT family^96-530^E:1.7e-104	0,283	0,625	0	3,033	0,653	0,115	0,019	0,572	0	2,319	PTR27_ARATH	Protein NRT1/ PTR FAMILY 6.2 (AtNPF6.2) (Nitrate transporter 1.4)	NPF6.2 NRT1.4 NTP2 At2g26690 F18A8.6	Arabidopsis thaliana (Mouse-ear cress)		Complete proteome; Membrane; Nitrate assimilation; Reference proteome; Symport; Transmembrane; Transmembrane helix; Transport
SRR122238_TR29842_c1_g3_i2_len=760	ARF2_ORYSJ	Q:152-694,H:1-181	98.9%ID	E:3e-130	ADP-ribosylation factor 2	PF00025.18^Arf^ADP-ribosylation factor family^5-177^E:3.1e-80`PF09439.7^SRPRB^Signal recognition particle receptor beta subunit^16-143^E:8.8e-14`PF08477.10^Roc^Ras of Complex, Roc, domain of DAPkinase^19-129^E:1.1e-13`PF04670.9^Gtr1_RagA^Gtr1/RagA G protein conserved region^19-142^E:2.5e-10`PF01926.20^MMR_HSR1^50S ribosome-binding GTPase^19-122^E:4.9e-05`PF00071.19^Ras^Ras family^20-172^E:1.1e-12	0,464	-0,237	0	1,178	1	0,001	0,751	0,114	0	0,903	ARF2_ORYSJ	ADP-ribosylation factor 2	ARF Os05g0489600 LOC_Os05g41060 OJ1119_H02.17	Oryza sativa subsp. japonica (Rice)		Complete proteome; ER-Golgi transport; GTP-binding; Golgi apparatus; Lipoprotein; Myristate; Nucleotide-binding; Protein transport; Reference proteome; Transport
SRR122238_TR47348_c0_g2_i2_len=1421	AROF_SOLLC	Q:1205-18,H:27-407	80.56%ID	E:0	Phospho-2-dehydro-3-deoxyheptonate aldolase 1, chloroplastic	PF01474.13^DAHP_synth_2^Class-II DAHP synthetase family^72-418^E:1.3e-145	0,028	0,542	0	3,799	0,034	0,58	0,101	0,464	0	2,336	AROF_SOLLC	Phospho-2-dehydro-3-deoxyheptonate aldolase 1, chloroplastic (EC 2.5.1.54) (3-deoxy-D-arabino-heptulosonate 7-phosphate synthase 1) (DAHP synthase 1) (Phospho-2-keto-3-deoxyheptonate aldolase 1)		Solanum lycopersicum (Tomato) (Lycopersicon esculentum)	PATHWAY: Metabolic intermediate biosynthesis; chorismate biosynthesis; chorismate from D-erythrose 4-phosphate and phosphoenolpyruvate: step 1/7.	Amino-acid biosynthesis; Aromatic amino acid biosynthesis; Chloroplast; Complete proteome; Plastid; Reference proteome; Transferase; Transit peptide
SRR122238_TR61114_c0_g2_i1_len=522	AROC2_SOLLC	Q:95-520,H:1-139	76.06%ID	E:3e-63	Chorismate synthase 2, chloroplastic	PF01264.18^Chorismate_synt^Chorismate synthase^91-172^E:1e-32	0,429	-0,515	0	7,813	0,623	0,42	0,52	0,463	0	7,752	AROC2_SOLLC	Chorismate synthase 2, chloroplastic (EC 4.2.3.5) (5-enolpyruvylshikimate-3-phosphate phospholyase 2)	CS2	Solanum lycopersicum (Tomato) (Lycopersicon esculentum)	PATHWAY: Metabolic intermediate biosynthesis; chorismate biosynthesis; chorismate from D-erythrose 4-phosphate and phosphoenolpyruvate: step 7/7.	Amino-acid biosynthesis; Aromatic amino acid biosynthesis; Chloroplast; Complete proteome; Lyase; Plastid; Reference proteome; Transit peptide

SRR122238_TR73954_c0_g1_i3_len=839	NA	NA	NA	NA	NA	.	0,129	0,829	0	1,586	0,012	0,858	0,175	0,488	0	7,872	.	.	.	.	.	.
SRR122238_TR77156_c0_g1_i2_len=836	UEV1C_ARATH	Q:697-260,H:1-145	89.73%ID	E:2e-88	Ubiquitin-conjugating enzyme E2 variant 1C	PF00179.23^UQ_con^Ubiquitin-conjugating enzyme^89-182^E:2e-14	1	-0,022	0	1,866	0,579	0,204	0,005	-1,123	0	8,434	UEV1C_ARATH	Ubiquitin-conjugating enzyme E2 variant 1C (Ubc enzyme variant 1C) (Protein MMS ZWEI HOMOLOG 3)	UEV1C MMZ3 At2g36060 F11F19.3	Arabidopsis thaliana (Mouse-ear cress)		Alternative splicing; Complete proteome; Reference proteome
SRR1271857_TR2593_c0_g2_i3_len=1500	GOLS2_ARATH	Q:176-1168,H:14-335	72.81%ID	E:1e-175	Galactinol synthase 2	PF01501.17^Glyco_transf_8^Glycosyl transferase family 8^28-272^E:1.8e-41	0,01	1,772	0	2,345	0,004	0,753	0,098	0,447	0,001	0,996	GOLS2_ARATH	Galactinol synthase 2 (AtGolS2) (GolS-2) (EC 2.4.1.123)	GOLS2 At1g56600 F25P12.95	Arabidopsis thaliana (Mouse-ear cress)		Carbohydrate metabolism; Complete proteome; Cytoplasm; Galactose metabolism; Glycosyltransferase; Manganese; Metal-binding; Reference proteome; Transferase
SRR1271857_TR26829_c0_g1_i1_len=2197	RFS2_ARATH	Q:248-2032,H:1-586	77.98%ID	E:0	Probable galactinol--sucrose galactosyltransferase 2	PF05691.9^Raffinose_syn^Raffinose synthase or seed imbibition protein Sip1^7-576^E:4.1e-269	0,333	0,616	0	3,977	0,224	0,367	0,206	0,386	0	2,405	RFS2_ARATH	Probable galactinol--sucrose galactosyltransferase 2 (EC 2.4.1.82) (Protein SEED IMBIBITION 2) (Raffinose synthase 2)	RFS2 RS2 SIP2 At3g57520 T8H10.120	Arabidopsis thaliana (Mouse-ear cress)		Alternative splicing; Carbohydrate metabolism; Complete proteome; Glycosyltransferase; Reference proteome; Transferase
SRR1271857_TR40399_c0_g1_i1_len=2792	TMN9_ARATH	Q:2432-558,H:16-644	82.83%ID	E:0	Transmembrane 9 superfamily member 9 {ECO:0000305}	PF02990.13^EMP70^Endomembrane protein 70^61-598^E:3.4e-220	0,568	0,182	0	1,93	0,504	0,219	0,144	0,456	0,001	1,311	TMN9_ARATH	Transmembrane 9 superfamily member 9 (Endomembrane protein 2) (Transmembrane nine protein 9) (AtTMN9)	TMN9 EMP2 At5g25100 T11H3_110	Arabidopsis thaliana (Mouse-ear cress)		Alternative splicing; Complete proteome; Endosome; Golgi apparatus; Membrane; Reference proteome; Signal; Transmembrane; Transmembrane helix
SRR1271858_TR3401_c0_g2_i1_len=2902	CDC48_SOYBN	Q:2755-341,H:1-807	94.18%ID	E:0	Cell division cycle protein 48 homolog	PF02359.15^CDC48_N^Cell division protein 48 (CDC48), N-terminal domain^30-111^E:3.9e-23`PF02933.14^CDC48_2^Cell division protein 48 (CDC48), domain 2^132-195^E:6.2e-12`PF00004.26^AAA^ATPase family associated with various cellular activities (AAA)^245-374^E:2.8e-48`PF05496.9^RuvB_N^Holliday junction DNA helicase ruvB N-terminus^245-320^E:5.8e-06`PF07724.11^AAA_2^AAA domain (Cdc48 subfamily)^245-338^E:4e-06`PF07728.11^AAA_5^AAA domain (dynein-related subfamily)^245-363^E:5.7e-05`PF05496.9^RuvB_N^Holliday junction DNA helicase ruvB N-terminus^517-552^E:1.1e-05`PF00004.26^AAA^ATPase family associated with various cellular activities (AAA)^518-651^E:6.6e-47	0,44	-0,254	0	2,945	1	-0,032	0,085	0,609	0	2,23	CDC48_SOYBN	Cell division cycle protein 48 homolog (Valosin-containing protein homolog) (VCP)	CDC48	Glycine max (Soybean) (Glycine hispida)		ATP-binding; Cell cycle; Cell membrane; Complete proteome; Membrane; Nucleotide-binding; Reference proteome; Repeat
SRR1271858_TR19224_c0_g2_i1_len=607	NA	NA	NA	NA	NA	PF11744.5^ALMT^Aluminium activated malate transporter^57-111^E:3.2e-21	0,034	0,824	0	6,802	0,005	1,32	0,005	1,319	0,001	1,788	.	.	.	.	.	.
SRR1271858_TR58701_c1_g1_i1_len=1833	E1313_ARATH	Q:1567-212,H:24-456	42.86%ID	E:7e-125	Glucan endo-1,3-beta-glucosidase 13	PF00332.15^Glyco_hydro_17^Glycosyl hydrolases family 17^39-355^E:3.4e-99`PF07983.10^X8^X8 domain^399-470^E:7.5e-22	0,632	0,249	0	2,781	0,01	2,133	0,517	0,767	0	4,389	E1313_ARATH	Glucan endo-1,3-beta-glucosidase 13 (EC 3.2.1.39) ((1->3)-beta-glucan endohydrolase 13) ((1->3)-beta-glucanase 13) (Beta-1,3-endoglucanase 13) (Beta-1,3-glucanase 13)	At5g56590 MIK19.3	Arabidopsis thaliana (Mouse-ear cress)		Cell membrane; Cell wall; Cell wall biogenesis/degradation; Complete proteome; Disulfide bond; GPI-anchor; Glycoprotein; Glycosidase; Hydrolase; Lipoprotein; Membrane; Plant defense; Reference proteome; Secreted; Signal
SRR1271859_TR815_c0_g1_i1_len=855	NA	NA	NA	NA	NA	.	0,399	-0,187	0	1,483	0,866	0,041	0,052	-0,448	0	11,959	.	.	.	.	.	.
SRR1271859_TR10523_c2_g1_i2_len=689	C71A8_MENPI	Q:108-674,H:284-472	48.68%ID	E:6e-58	Cytochrome P450 71A8	PF00067.19^p450^Cytochrome P450^18-203^E:5.5e-51	0,778	-0,083	0	2,354	0,016	0,762	0	-1,79	0	2,112	C71A8_MENPI	Cytochrome P450 71A8 (EC 1.14.-.-)	CYP71A8	Mentha piperita (Peppermint) (Mentha aquatica ? Mentha spicata)		Heme; Iron; Membrane; Metal-binding; Monooxygenase; Oxidoreductase; Transmembrane; Transmembrane helix
SRR1271859_TR28455_c0_g1_i2_len=1369	EF1A_SOLLC	Q:189-1367,H:1-393	97.96%ID	E:0	Elongation factor 1-alpha	PF00009.24^GTP_EFTU^Elongation factor Tu GTP binding domain^6-221^E:7.2e-53`PF03144.22^GTP_EFTU_D2^Elongation factor Tu domain 2^248-313^E:5.9e-15`PF03143.14^GTP_EFTU_D3^Elongation factor Tu C-terminal domain^322-392^E:1.2e-17	0,014	-0,7	0	1,142	0,636	0,186	1	-0,027	0	1,376	EF1A_SOLLC	Elongation factor 1-alpha (EF-1-alpha)		Solanum lycopersicum (Tomato) (Lycopersicon esculentum)		Complete proteome; Cytoplasm; Elongation factor; GTP-binding; Nucleotide-binding; Phosphoprotein; Protein biosynthesis; Reference proteome
SRR1271859_TR38030_c0_g1_i1_len=3201	EF2_BETVU	Q:3016-488,H:1-843	92.17%ID	E:0	Elongation factor 2	PF00009.24^GTP_EFTU^Elongation factor Tu GTP binding domain^17-342^E:1.5e-64`PF03144.22^GTP_EFTU_D2^Elongation factor Tu domain 2^393-468^E:3.4e-12`PF14492.3^EFG_II^Elongation Factor G, domain II^487-549^E:4.6e-12`PF03764.15^EFG_IV^Elongation factor G, domain IV^610-721^E:3.7e-33`PF00679.21^EFG_C^Elongation factor G C-terminus^724-811^E:1.3e-20	0,004	-0,702	0	2,067	0,247	-0,317	0,862	-0,058	0	1,42	EF2_BETVU	Elongation factor 2 (EF-2)		Beta vulgaris (Sugar beet)		Cytoplasm; Elongation factor; GTP-binding; Nucleotide-binding; Phosphoprotein; Protein biosynthesis
SRR342017_TR18107_c0_g2_i1_len=825	UBAX1_ARATH	Q:343-819,H:138-293	54.88%ID	E:4e-43	.	PF08059.10^SEP^SEP domain^125-181^E:2.2e-22	0,635	-0,335	0,001	3,624	0,5	-0,59	1	0,137	0	3,634	PUX5_ARATH	Plant UBX domain-containing protein 5 (PUX5)	PUX5 At4g15410 dl3750w FCAALL.290	Arabidopsis thaliana (Mouse-ear cress)		Complete proteome; Reference proteome; Ubl conjugation pathway
SRR342019_TR519_c0_g1_i1_len=692	NA	NA	NA	NA	NA	.	0,084	0,546	0	1,064	0,767	0,076	0,337	0,23	0	7,129	.	.	.	.	.	.
SRR342019_TR5968_c2_g9_i3_len=682	NA	NA	NA	NA	NA	.	0,101	0,416	0	8,493	0,378	-0,23	0,281	-0,281	0	1,264	.	.	.	.	.	.
SRR342022_TR961_c4_g1_i2_len=616	AROG_SOLTU	Q:55-615,H:305-491	94.65%ID	E:6e-129	Phospho-2-dehydro-3-deoxyheptonate aldolase 2, chloroplastic	PF01474.13^DAHP_synth_2^Class-II DAHP synthetase family^1-173^E:7.8e-85	0,011	0,61	0	2,857	0,022	0,595	0,147	0,38	0	1,342	AROG_SOLTU	Phospho-2-dehydro-3-deoxyheptonate aldolase 2, chloroplastic (EC 2.5.1.54) (3-deoxy-D-arabino-heptulosonate 7-phosphate synthase 2) (DAHP synthase 2) (Phospho-2-keto-3-deoxyheptonate aldolase 2)	SHKB ARO2	Solanum tuberosum (Potato)	PATHWAY: Metabolic intermediate biosynthesis; chorismate biosynthesis; chorismate from D-erythrose 4-phosphate and phosphoenolpyruvate: step 1/7.	Amino-acid biosynthesis; Aromatic amino acid biosynthesis; Chloroplast; Complete proteome; Plastid; Reference proteome; Transferase; Transit peptide
SRR342022_TR4690_c0_g1_i4_len=2277	GLYM_SOLTU	Q:356-1909,H:1-518	90.56%ID	E:0	Serine hydroxymethyltransferase, mitochondrial	PF00464.16^SHMT^Serine hydroxymethyltransferase^58-454^E:1.2e-210	0,217	-0,322	0	9,911	0	-1,75	0	-0,886	0	1,298	GLYM_SOLTU	Serine hydroxymethyltransferase, mitochondrial (SHMT) (EC 2.1.2.1) (Glycine hydroxymethyltransferase) (Serine methylase)		Solanum tuberosum (Potato)	PATHWAY: One-carbon metabolism; tetrahydrofolate interconversion.	Complete proteome; Mitochondrion; One-carbon metabolism; Pyridoxal phosphate; Reference proteome; Transferase; Transit peptide
SRR342022_TR21447_c1_g1_i6_len=742	NA	NA	NA	NA	NA	PF00248.18^Aldo_ket_red^Aldo/keto reductase family^120-247^E:4.5e-23	0,721	-0,18	0	3,563	0,297	-0,42	0,816	0,084	0	8,409	.	.	.	.	.	.
SRR342023_TR831_c2_g6_i5_len=859	MTHR1_MAIZE	Q:113-859,H:1-249	88.76%ID	E:9e-161	Methylenetetrahydrofolate reductase 1	.	0,03	0,963	0	7,229	0,017	-1,365	0,132	-0,825	0	8,371	MTHR1_MAIZE	Methylenetetrahydrofolate reductase 1 (EC 1.5.1.20) (ZmMTHFR1)		Zea mays (Maize)	PATHWAY: One-carbon metabolism; tetrahydrofolate interconversion.	Complete proteome; FAD; Flavoprotein; NAD; Oxidoreductase; Reference proteome
SRR342023_TR23697_c2_g1_i9_len=1234	RFS2_ARATH	Q:113-1234,H:226-597	85.29%ID	E:0	Probable galactinol--sucrose galactosyltransferase 2	PF05691.9^Raffinose_syn^Raffinose synthase or seed imbibition protein Sip1^1-251^E:4.3e-141	0,56	0,895	0	3,813	0,162	0,407	0,825	-0,088	0	6,349	RFS2_ARATH	Probable galactinol--sucrose galactosyltransferase 2 (EC 2.4.1.82) (Protein SEED IMBIBITION 2) (Raffinose synthase 2)	RFS2 RS2 SIP2 At3g57520 T8H10.120	Arabidopsis thaliana (Mouse-ear cress)		Alternative splicing; Carbohydrate metabolism; Complete proteome; Glycosyltransferase; Reference proteome; Transferase
SRR342023_TR30529_c0_g9_i1_len=1364	IF4A2_NICPL	Q:212-1153,H:1-314	95.54%ID	E:0	Eukaryotic initiation factor 4A-2	PF00270.26^DEAD^DEAD/DEAH box helicase^65-226^E:2.8e-40`PF04851.12^ResIII^Type III restriction enzyme, res subunit^83-223^E:8.6e-07	0,058	-0,532	0	4,418	0,329	0,303	0,05	0,593	0	1,349	IF4A2_NICPL	Eukaryotic initiation factor 4A-2 (eIF-4A-2) (EC 3.6.4.13) (ATP-dependent RNA helicase eIF4A-2)		Nicotiana plumbaginifolia (Leadwort-leaved tobacco) (Tex-Mex tobacco)		ATP-binding; Helicase; Hydrolase; Initiation factor; Nucleotide-binding; Protein biosynthesis; RNA-binding
SRR646572_TR2358_c0_g1_i1_len=1543	NA	NA	NA	NA	NA	PF06200.11^tify^tify domain^120-151^E:1.5e-16`PF09425.7^CCT_2^Divergent CCT motif^258-271^E:1.4e-05	1	0	0	3,427	0,129	-5,085	0,129	-5,085	0	6,745	.	.	.	.	.	.
SRR646572_TR21988_c0_g1_i1_len=2298	BIP5_TOBAC	Q:237-2174,H:1-646	95.2%ID	E:0	Luminal-binding protein 5	PF00012.17^HSP70^Hsp70 protein^38-645^E:4.1e-268`PF06723.10^MreB_Mbl^MreB/Mbl protein^151-405^E:6.5e-17	0,119	-0,534	0	2,291	0,537	-0,376	0,142	-0,751	0	5,588	BIP5_TOBAC	Luminal-binding protein 5 (BiP 5) (78 kDa glucose-regulated protein homolog 5) (GRP-78-5)	BIP5	Nicotiana tabacum (Common tobacco)		ATP-binding; Endoplasmic reticulum; Glycoprotein; Nucleotide-binding; Signal
SRR646572_TR26211_c1_g3_i2_len=2581	HSP7C_PETHY	Q:2324-378,H:1-651	93.24%ID	E:0	Heat shock cognate 70 kDa protein	PF00012.17^HSP70^Hsp70 protein^9-618^E:4.7e-268`PF06723.10^MreB_Mbl^MreB/Mbl protein^142-380^E:5.5e-14	0,844	-0,049	0	1,984	0,192	0,303	0,007	0,613	0,001	0,978	HSP7C_PETHY	Heat shock cognate 70 kDa protein	HSP70	Petunia hybrida (Petunia)		ATP-binding; Nucleotide-binding; Stress response
SRR646596_TR31160_c0_g1_i5_len=5738	PP226_ARATH	Q:4997-4020,H:54-379	44.48%ID	E:1e-82	Pentatricopeptide repeat-containing protein At3g13160, mitochondrial	PF01535.17^PPR^PPR repeat^160-185^E:0.00039`PF17177.1^PPR_long^Pentacotripeptide-repeat region of PROPR^167-276^E:1.1e-07`PF13812.3^PPR_3^Pentatricopeptide repeat domain^175-233^E:2.8e-09`PF01535.17^PPR^PPR repeat^191-217^E:0.09`PF12854.4^PPR_1^PPR repeat^221-252^E:1.8e-12`PF13041.3^PPR_2^PPR repeat family^223-270^E:4.9e-16`PF01535.17^PPR^PPR repeat^226-256^E:6e-07`PF13812.3^PPR_3^Pentatricopeptide repeat domain^247-302^E:1.8e-07`PF12854.4^PPR_1^PPR repeat^255-287^E:8.9e-06`PF13041.3^PPR_2^PPR repeat family^258-305^E:8.8e-08`PF01535.17^PPR^PPR repeat^261-290^E:0.0007`PF13041.3^PPR_2^PPR repeat family^294-340^E:9.6e-11`PF01535.17^PPR^PPR repeat^297-326^E:0.0036`PF13812.3^PPR_3^Pentatricopeptide repeat domain^317-371^E:4e-11`PF13041.3^PPR_2^PPR repeat family^329-373^E:3.9e-11`PF01535.17^PPR^PPR repeat^331-361^E:1.8e-06	0,001	2,499	0	2,354	0,017	4,264	0,002	4,701	0	2,293	PP226_ARATH	Pentatricopeptide repeat-containing protein At3g13160, mitochondrial	At3g13160 MJG19.11	Arabidopsis thaliana (Mouse-ear cress)		Complete proteome; Mitochondrion; Reference proteome; Repeat; Transit peptide
SRR646604_TR37414_c0_g2_i3_len=1472	GOLS2_ARATH	Q:1330-338,H:14-335	72.81%ID	E:3e-176	Galactinol synthase 2	PF01501.17^Glyco_transf_8^Glycosyl transferase family 8^28-272^E:1.8e-41	0,007	2,121	0	2,757	0,018	0,628	0,186	0,361	0	4,219	GOLS2_ARATH	Galactinol synthase 2 (AtGolS2) (GolS-2) (EC 2.4.1.123)	GOLS2 At1g56600 F25P12.95	Arabidopsis thaliana (Mouse-ear cress)		Carbohydrate metabolism; Complete proteome; Cytoplasm; Galactose metabolism; Glycosyltransferase; Manganese; Metal-binding; Reference proteome; Transferase
SRR648705_TR23795_c0_g1_i1_len=2325	YTHD2_MOUSE	Q:1266-1826,H:382-565	48.17%ID	E:7e-53	YTH domain-containing family protein 2 {ECO:0000305}	PF04146.12^YTH^YT521-B-like domain^346-487^E:2.4e-35	1	-0,02	0,001	1,232	0,064	0,672	0,01	0,886	0	3,648	YTHD2_MOUSE	YTH domain-containing family protein 2	Ythdf2	Mus musculus (Mouse)		Acetylation; Complete proteome; Cytoplasm; Phosphoprotein; RNA-binding; Reference proteome
SRR648705_TR25099_c1_g3_i4_len=1258	GEDH1_OCIBA	Q:226-936,H:11-246	57.38%ID	E:2e-71	Geraniol dehydrogenase 1	PF08240.9^ADH_N^Alcohol dehydrogenase GroES-like domain^39-152^E:4.4e-27	0,196	-0,419	0	4,309	1	-0,062	0,812	0,143	0	2,88	GEDH1_OCIBA	Geraniol dehydrogenase 1 (ObaGEDH1) (EC 1.1.1.183)	GEDH1	Ocimum basilicum (Sweet basil)		Metal-binding; NADP; Oxidoreductase; Zinc
SRR648705_TR28357_c0_g1_i2_len=2820	THIC_ARATH	Q:242-2173,H:3-643	85.74%ID	E:0	Phosphomethylpyrimidine synthase, chloroplastic	PF01964.15^ThiC_Rad_SAM^Radical SAM ThiC family^166-586^E:7.1e-198	0,397	-0,214	0	9,752	0,129	-4,919	0,129	-4,919	0	9,711	THIC_ARATH	Phosphomethylpyrimidine synthase, chloroplastic (EC 4.1.99.17) (Hydroxymethylpyrimidine phosphate synthase) (HMP-P synthase) (HMP-phosphate synthase) (HMPP synthase) (Protein PYRIMIDINE REQUIRING) (Thiamine biosynthesis protein ThiC) (Protein THIAMINE C)	THIC PY At2g29630 T27A16.27	Arabidopsis thaliana (Mouse-ear cress)	PATHWAY: Cofactor biosynthesis; thiamine diphosphate biosynthesis.	3D-structure; 4Fe-4S; Chloroplast; Complete proteome; Iron; Iron-sulfur; Lyase; Metal-binding; Plastid; Reference proteome; S-adenosyl-L-methionine; Thiamine biosynthesis; Transit peptide; Zinc
SRR924147_TR14862_c0_g1_i2_len=815	UBQ11_ARATH	Q:181-813,H:1-211	100%ID	E:1e-148	Polyubiquitin 11	PF14560.3^Ubiquitin_2^Ubiquitin-like domain^59-129^E:0.00021`PF11976.5^Rad60-SLD^Ubiquitin-2 like Rad60 SUMO-like^61-131^E:7.9e-16`PF00240.20^ubiquitin^Ubiquitin family^63-134^E:1.2e-33`PF13881.3^Rad60-SLD_2^Ubiquitin-2 like Rad60 SUMO-like^70-131^E:0.1`PF11976.5^Rad60-SLD^Ubiquitin-2 like Rad60 SUMO-like^137-207^E:7.9e-16`PF00240.20^ubiquitin^Ubiquitin family^139-210^E:1.2e-33`PF14560.3^Ubiquitin_2^Ubiquitin-like domain^148-205^E:0.0005`PF13881.3^Rad60-SLD_2^Ubiquitin-2 like Rad60 SUMO-like^149-207^E:0.12`PF11976.5^Rad60-SLD^Ubiquitin-2 like Rad60 SUMO-like^213-271^E:1.3e-11`PF00240.20^ubiquitin^Ubiquitin family^215-271^E:1.2e-25`PF14560.3^Ubiquitin_2^Ubiquitin-like domain^224-258^E:0.016	0,708	0,129	0	1,903	0,909	0,049	0,778	0,095	0	1,632	UBQ11_ARATH	Polyubiquitin 11 [Cleaved into: Ubiquitin]	UBQ11 At4g05050 C17L7.6 T32N4.13	Arabidopsis thaliana (Mouse-ear cress)		Alternative splicing; Complete proteome; Cytoplasm; Isopeptide bond; Nucleus; Reference proteome; Repeat; Ubl conjugation; Ubl conjugation pathway
SRR924147_TR19409_c0_g2_i1_len=1533	ODPB3_ARATH	Q:170-1384,H:1-406	84.07%ID	E:0	Pyruvate dehydrogenase E1 component subunit beta-3, chloroplastic	PF02779.21^Transket_pyr^Transketolase, pyrimidine binding domain^89-259^E:1.2e-46`PF02780.17^Transketolase_C^Transketolase, C-terminal domain^275-396^E:1.5e-35	0,626	-0,223	0	1,765	0,129	-0,525	0,71	0,138	0,001	1,415	ODPB3_ARATH	Pyruvate dehydrogenase E1 component subunit beta-3, chloroplastic (EC 1.2.4.1)	E1-BETA-2 At2g34590 T31E10.7	Arabidopsis thaliana (Mouse-ear cress)		Chloroplast; Complete proteome; Glycolysis; Oxidoreductase; Plastid; Pyruvate; Reference proteome; Thiamine pyrophosphate; Transit peptide
SRR924148_TR26347_c0_g1_i3_len=1594	MTDH_APIGR	Q:1269-208,H:4-359	57.7%ID	E:8e-133	Mannitol dehydrogenase	PF08240.9^ADH_N^Alcohol dehydrogenase GroES-like domain^37-146^E:3.3e-23`PF02826.16^2-Hacid_dh_C^D-isomer specific 2-hydroxyacid dehydrogenase, NAD binding domain^177-227^E:3e-06`PF00107.23^ADH_zinc_N^Zinc-binding dehydrogenase^190-313^E:5.5e-14	0,013	-0,58	0	7,423	0,124	-0,455	0,468	-0,214	0	8,383	MTDH_APIGR	Mannitol dehydrogenase (EC 1.1.1.255) (NAD-dependent mannitol dehydrogenase)	MTD	Apium graveolens (Celery)		Cytoplasm; Metal-binding; NAD; Oxidoreductase; Zinc
SRR924148_TR28419_c0_g3_i7_len=1288	PIP1_SOLLC	Q:347-1114,H:1-256	86.72%ID	E:3e-160	Probable aquaporin PIP-type pTOM75	PF00230.17^MIP^Major intrinsic protein^45-256^E:5.9e-73	0,241	-0,674	0	2,936	0,22	0,487	0,562	-0,241	0	1,855	PIP1_SOLLC	Probable aquaporin PIP-type pTOM75 (Ripening-associated membrane protein) (RAMP)		Solanum lycopersicum (Tomato) (Lycopersicon esculentum)		Cell membrane; Complete proteome; Membrane; Reference proteome; Repeat; Stress response; Transmembrane; Transmembrane helix; Transport
SRR342023_TR18646_c0_g1_i1_len=508	GOLS2_ARATH	Q:198-506,H:14-116	82.52%ID	E:2e-54	Galactinol synthase 2	PF01501.17^Glyco_transf_8^Glycosyl transferase family 8^28-118^E:4.1e-09	0	6,669	0	7,825	0,496	-0,263	0,011	0,801	0	9,306	GOLS2_ARATH	Galactinol synthase 2 (AtGolS2) (GolS-2) (EC 2.4.1.123)	GOLS2 At1g56600 F25P12.95	Arabidopsis thaliana (Mouse-ear cress)		Carbohydrate metabolism; Complete proteome; Cytoplasm; Galactose metabolism; Glycosyltransferase; Manganese; Metal-binding; Reference proteome; Transferase
SRR342023_TR31063_c0_g4_i4_len=854	TRPB_CAMAC	Q:24-854,H:27-306	83.21%ID	E:2e-168	Tryptophan synthase beta chain 2, chloroplastic	PF00291.22^PALP^Pyridoxal-phosphate dependent enzyme^100-284^E:2.6e-29	0	1,355	0	9,327	0,699	-0,154	0,95	-0,04	0	1,594	TRPB_CAMAC	Tryptophan synthase beta chain 2, chloroplastic (EC 4.2.1.20)	TSB	Camptotheca acuminata (Happy tree)	PATHWAY: Amino-acid biosynthesis; L-tryptophan biosynthesis; L-tryptophan from chorismate: step 5/5.	Amino-acid biosynthesis; Aromatic amino acid biosynthesis; Chloroplast; Lyase; Plastid; Pyridoxal phosphate; Transit peptide; Tryptophan biosynthesis
SRR1144634_TR33976_c0_g2_i1_len=1286	NA	NA	NA	NA	NA	PF05627.8^AvrRpt-cleavage^Cleavage site for pathogenic type III effector avirulence factor Avr^199-235^E:2.6e-22	0	4,674	0,129	4,616	0,003	2,699	0	3,546	1	-0,586	.	.	.	.	.	.
SRR122238_TR46358_c0_g2_i1_len=1302	NA	NA	NA	NA	NA	.	0,001	1,602	0,007	1,095	0,002	1,176	0	1,61	0,76	0,608	.	.	.	.	.	.
SRR342019_TR22055_c0_g6_i8_len=2444	U89A2_ARATH	Q:1622-240,H:19-465	51.72%ID	E:9e-158	UDP-glycosyltransferase 89A2	PF00201.15^UDPGT^UDP-glucoronosyl and UDP-glucosyl transferase^263-435^E:9.1e-18	0	2,185	0,007	2,16	0,009	1,615	0	2,887	0,206	0,664	U89A2_ARATH	UDP-glycosyltransferase 89A2 (EC 2.4.1.-)	UGT89A2 At5g03490 F12E4.260	Arabidopsis thaliana (Mouse-ear cress)		Complete proteome; Glycosyltransferase; Reference proteome; Transferase
SRR1144633_TR4333_c0_g2_i1_len=829	TCTP_ELAGV	Q:706-203,H:1-168	87.5%ID	E:3e-84	Translationally-controlled tumor protein homolog	PF00838.14^TCTP^Translationally controlled tumour protein^42-205^E:2.1e-58	0,882	0,122	0	3,042	0,16	1,382	0	2,58	1	0	TCTP_ELAGV	Translationally-controlled tumor protein homolog (TCTP)	TCTP	Elaeis guineensis var. tenera (Oil palm)		Calcium; Cytoplasm
SRR1144633_TR36337_c0_g1_i1_len=1588	NA	NA	NA	NA	NA	.	0,959	0,016	0	1,275	0,003	0,692	0	0,827	0,132	0,676	.	.	.	.	.	.
SRR1144634_TR8906_c0_g1_i2_len=3144	NA	NA	NA	NA	NA	PF13855.3^LRR_8^Leucine rich repeat^448-504^E:8.4e-11`PF12799.4^LRR_4^Leucine Rich repeats (2 copies)^471-507^E:3.3e-08`PF14580.3^LRR_9^Leucine-rich repeat^471-553^E:2.6e-08`PF12799.4^LRR_4^Leucine Rich repeats (2 copies)^492-533^E:1.3e-07`PF13516.3^LRR_6^Leucine Rich repeat^492-505^E:1.7`PF13855.3^LRR_8^Leucine rich repeat^493-549^E:4.5e-08`PF13516.3^LRR_6^Leucine Rich repeat^538-551^E:0.35	0,079	0,538	0	1,481	0,003	0,721	0	1,285	1	-0,038	.	.	.	.	.	.
SRR1144634_TR15551_c0_g2_i2_len=1282	GSTF_HYOMU	Q:748-251,H:48-212	68.67%ID	E:3e-79	Glutathione S-transferase	PF00043.22^GST_C^Glutathione S-transferase, C-terminal domain^9-103^E:2.9e-16`PF13410.3^GST_C_2^Glutathione S-transferase, C-terminal domain^31-97^E:4.5e-09`PF14497.3^GST_C_3^Glutathione S-transferase, C-terminal domain^37-104^E:9.6e-06	0,073	-1,985	0,001	1,75	0,003	3,262	0	3,687	1	0,198	GSTF_HYOMU	Glutathione S-transferase (EC 2.5.1.18) (25 kDa auxin-binding protein) (GST class-phi)		Hyoscyamus muticus (Egyptian henbane)		Auxin signaling pathway; Direct protein sequencing; Transferase
SRR1144634_TR23126_c3_g3_i2_len=696	PRS2_SOLTU	Q:597-130,H:1-155	38.75%ID	E:2e-25	Pathogenesis-related protein STH-2	PF00407.16^Bet_v_1^Pathogenesis-related protein Bet v I family^34-183^E:8.4e-12	1	0,059	0	2,295	0,017	0,857	0,001	1,16	0,472	0,777	PRS2_SOLTU	Pathogenesis-related protein STH-2	STH-2	Solanum tuberosum (Potato)		Complete proteome; Pathogenesis-related protein; Plant defense; Reference proteome
SRR122237_TR14951_c0_g1_i1_len=1315	GOLS2_SOLLC	Q:1251-241,H:1-337	81.23%ID	E:0	Galactinol synthase 2	PF01501.17^Glyco_transf_8^Glycosyl transferase family 8^49-292^E:4.9e-41	0,164	0,97	0	2,335	0,002	1,562	0	1,753	1	0,284	GOLS2_SOLLC	Galactinol synthase 2 (GolS-2) (SlGolS2) (EC 2.4.1.123)	GOLS2	Solanum lycopersicum (Tomato) (Lycopersicon esculentum)		Carbohydrate metabolism; Complete proteome; Cytoplasm; Galactose metabolism; Glycosyltransferase; Manganese; Metal-binding; Reference proteome; Transferase
SRR122238_TR6887_c0_g2_i1_len=1739	HST_TOBAC	Q:176-1477,H:1-435	86.01%ID	E:0	Shikimate O-hydroxycinnamoyltransferase	PF02458.12^Transferase^Transferase family^1-429^E:7.8e-109	0,281	0,564	0	3,392	0,022	0,717	0	1,271	0,196	0,807	HST_TOBAC	Shikimate O-hydroxycinnamoyltransferase (EC 2.3.1.133) (Hydroxycinnamoyl transferase) (Hydroxycinnamoyl-Coenzyme A shikimate/quinate hydroxycinnamoyltransferase)	HST HCT	Nicotiana tabacum (Common tobacco)		Acyltransferase; Direct protein sequencing; Transferase
SRR122238_TR37332_c0_g1_i1_len=1339	CSE_ARATH	Q:173-1108,H:21-330	82.37%ID	E:0	Caffeoylshikimate esterase	PF12146.5^Hydrolase_4^Serine aminopeptidase, S33^59-302^E:3.2e-52`PF12697.4^Abhydrolase_6^Alpha/beta hydrolase family^66-304^E:4e-07	0,38	0,588	0	4,278	0,066	0,709	0,001	1,17	0,077	0,916	CSE_ARATH	Caffeoylshikimate esterase (EC 3.1.1.-) (Lysophospholipase 2) (LysoPL2)	CSE At1g52760 F14G24.3	Arabidopsis thaliana (Mouse-ear cress)		Cell membrane; Complete proteome; Hydrolase; Lignin biosynthesis; Membrane; Reference proteome
SRR122238_TR61991_c0_g1_i1_len=1161	ARGI1_ARATH	Q:1098-334,H:88-342	92.16%ID	E:8e-172	Arginase 1, mitochondrial	PF00491.18^Arginase^Arginase family^4-201^E:1.7e-58	0,402	-0,902	0,001	1,949	0,005	4,237	0,001	4,711	0,848	-0,144	ARGI1_ARATH	Arginase 1, mitochondrial (EC 3.5.3.1) (Arginine amidohydrolase 1)	ARGAH1 At4g08900 T3H13.7	Arabidopsis thaliana (Mouse-ear cress)	PATHWAY: Nitrogen metabolism; urea cycle; L-ornithine and urea from L-arginine: step 1/1.	Arginine metabolism; Complete proteome; Hydrolase; Manganese; Metal-binding; Mitochondrion; Reference proteome; Transit peptide
SRR122238_TR74721_c0_g1_i1_len=1411	TKPR2_ARATH	Q:82-1044,H:1-321	70.4%ID	E:3e-174	Tetraketide alpha-pyrone reductase 2	PF08659.7^KR^KR domain^30-157^E:2.2e-05`PF01370.18^Epimerase^NAD dependent epimerase/dehydratase family^32-269^E:1.1e-26`PF02719.12^Polysacc_synt_2^Polysaccharide biosynthesis protein^32-153^E:3.7e-07`PF01073.16^3Beta_HSD^3-beta hydroxysteroid dehydrogenase/isomerase family^33-266^E:3.2e-20`PF16363.2^GDP_Man_Dehyd^GDP-mannose 4,6 dehydratase^33-289^E:1e-16`PF07993.9^NAD_binding_4^Male sterility protein^33-256^E:8.1e-16`PF05368.10^NmrA^NmrA-like family^33-109^E:8.2e-08`PF13460.3^NAD_binding_10^NAD(P)H-binding^35-217^E:7.3e-15	1	3,013	0	6,893	0,002	6,369	0	6,761	1	3,562	TKPR2_ARATH	Tetraketide alpha-pyrone reductase 2 (EC 1.1.1.-) (Protein CINNAMOYL-COA REDUCTASE-LIKE 6)	TKPR2 CCRL6 At1g68540 T26J14.11	Arabidopsis thaliana (Mouse-ear cress)		Acetylation; Alternative splicing; Complete proteome; Cytoplasm; NADP; Oxidoreductase; Reference proteome
SRR122238_TR78776_c0_g1_i4_len=868	NA	NA	NA	NA	NA	PF13259.3^DUF4050^Protein of unknown function (DUF4050)^73-113^E:3.2e-11	0,095	0,504	0	2,012	0,01	0,83	0,001	1,062	1	-0,012	.	.	.	.	.	.
SRR122238_TR87584_c0_g1_i5_len=927	FER3_ARATH	Q:149-604,H:1-155	56.77%ID	E:5e-57	Ferredoxin-3, chloroplastic	PF00111.24^Fer2^2Fe-2S iron-sulfur cluster binding domain^65-138^E:2.6e-21	0,108	0,728	0	2,29	0,004	1,159	0	1,829	0	-8,72	FER3_ARATH	Ferredoxin-3, chloroplastic (AtFd3)	FD3 At2g27510 F10A12.19	Arabidopsis thaliana (Mouse-ear cress)		2Fe-2S; Acetylation; Chloroplast; Complete proteome; Electron transport; Iron; Iron-sulfur; Metal-binding; Plastid; Reference proteome; Transit peptide; Transport
SRR1271857_TR5433_c0_g2_i3_len=2219	CYSZ_CUCMA	Q:1987-596,H:12-475	88.17%ID	E:0	Citrate synthase, glyoxysomal	PF00285.18^Citrate_synt^Citrate synthase, C-terminal domain^101-469^E:2.8e-134	0,811	-0,318	0	3,073	0,044	2,337	0	3,332	1	0,871	CYSZ_CUCMA	Citrate synthase, glyoxysomal (EC 2.3.3.16) (GCS)		Cucurbita maxima (Pumpkin) (Winter squash)	PATHWAY: Carbohydrate metabolism; glyoxylate cycle; isocitrate from oxaloacetate: step 1/2.	Direct protein sequencing; Glyoxylate bypass; Glyoxysome; Peroxisome; Transferase; Transit peptide; Tricarboxylic acid cycle
SRR1271857_TR9218_c0_g4_i3_len=1163	NA	NA	NA	NA	NA	.	0,286	0,305	0	0,856	0,004	0,656	0	1,123	0,054	0,723	.	.	.	.	.	.
SRR1271857_TR9959_c0_g1_i24_len=2120	NA	NA	NA	NA	NA	PF08491.7^SE^Squalene epoxidase^78-167^E:9.5e-05	0,581	0,342	0	2,908	0,001	2,042	0	3,03	0,822	0,357	.	.	.	.	.	.
SRR1271858_TR57555_c0_g1_i4_len=965	NA	NA	NA	NA	NA	.	0,804	0,1	0	2,58	0,014	0,782	0	1,174	0,03	0,793	.	.	.	.	.	.
SRR1271858_TR57555_c0_g1_i5_len=1914	NA	NA	NA	NA	NA	.	0,32	0,273	0	0,921	0,007	0,732	0	1,046	0,065	0,534	.	.	.	.	.	.
SRR1271858_TR64995_c1_g2_i5_len=1699	E1314_ARATH	Q:546-1571,H:28-370	68.8%ID	E:9e-174	Glucan endo-1,3-beta-glucosidase 14	PF00332.15^Glyco_hydro_17^Glycosyl hydrolases family 17^28-344^E:9.2e-94	0,258	0,931	0	2,302	0,003	2,148	0	3,17	1	0,182	E1314_ARATH	Glucan endo-1,3-beta-glucosidase 14 (EC 3.2.1.39) ((1->3)-beta-glucan endohydrolase 14) ((1->3)-beta-glucanase 14) (Beta-1,3-endoglucanase 14) (Beta-1,3-glucanase 14)	At2g27500 F10A12.18	Arabidopsis thaliana (Mouse-ear cress)		Alternative splicing; Cell membrane; Cell wall; Cell wall biogenesis/degradation; Complete proteome; Cytoplasm; GPI-anchor; Glycoprotein; Glycosidase; Hydrolase; Lipoprotein; Membrane; Plant defense; Reference proteome; Secreted; Signal
SRR1271859_TR1161_c1_g1_i2_len=4252	PDR3_TOBAC	Q:4251-49,H:47-1447	74.15%ID	E:0	Pleiotropic drug resistance protein 3	PF14510.3^ABC_trans_N^ABC-transporter extracellular N-terminal^64-114^E:1.8e-07`PF00005.24^ABC_tran^ABC transporter^140-322^E:7.8e-17`PF01061.21^ABC2_membrane^ABC-2 type transporter^475-686^E:3.8e-36`PF08370.8^PDR_assoc^Plant PDR ABC transporter associated^691-750^E:1.6e-21`PF00005.24^ABC_tran^ABC transporter^831-980^E:3.4e-19`PF01061.21^ABC2_membrane^ABC-2 type transporter^1127-1340^E:2e-53	0,472	0,811	0	4,331	0,025	2,554	0	3,535	1	0,018	PDR3_TOBAC	Pleiotropic drug resistance protein 3 (NtPDR3)	PDR3	Nicotiana tabacum (Common tobacco)		ATP-binding; Membrane; Nucleotide-binding; Repeat; Transmembrane; Transmembrane helix; Transport
SRR1271859_TR10897_c0_g1_i5_len=1920	AROC2_SOLLC	Q:1612-326,H:1-426	83.45%ID	E:0	Chorismate synthase 2, chloroplastic	PF01264.18^Chorismate_synt^Chorismate synthase^60-407^E:8.6e-140	0,464	0,223	0	1,869	0,008	0,744	0,001	0,945	1	-0,063	AROC2_SOLLC	Chorismate synthase 2, chloroplastic (EC 4.2.3.5) (5-enolpyruvylshikimate-3-phosphate phospholyase 2)	CS2	Solanum lycopersicum (Tomato) (Lycopersicon esculentum)	PATHWAY: Metabolic intermediate biosynthesis; chorismate biosynthesis; chorismate from D-erythrose 4-phosphate and phosphoenolpyruvate: step 7/7.	Amino-acid biosynthesis; Aromatic amino acid biosynthesis; Chloroplast; Complete proteome; Lyase; Plastid; Reference proteome; Transit peptide
SRR1271859_TR19123_c0_g2_i1_len=1875	SPZX_ARATH	Q:222-1379,H:1-389	64.27%ID	E:4e-164	Serpin-ZX	PF00079.17^Serpin^Serpin (serine protease inhibitor)^10-386^E:3.3e-99	0,578	0,228	0	1,408	0,006	0,964	0	1,25	0,767	0,122	SPZX_ARATH	Serpin-ZX (ArathZx) (AtSerpin1) (Serpin-1)	At1g47710 F16N3.3 T2E6.22	Arabidopsis thaliana (Mouse-ear cress)		3D-structure; Apoplast; Complete proteome; Glycoprotein; Protease inhibitor; Reference proteome; Secreted; Serine protease inhibitor
SRR1271859_TR35644_c0_g1_i6_len=2224	AB13I_ARATH	Q:454-1389,H:38-345	80.63%ID	E:5e-173	.	PF00005.24^ABC_tran^ABC transporter^106-261^E:3.7e-30	0,151	0,655	0	2,729	0,001	1,542	0	1,831	0,86	0,161	TGD3_ARATH	Protein TRIGALACTOSYLDIACYLGLYCEROL 3, chloroplastic (ABC transporter I family member 13) (ABC transporter ABCI.13) (AtABCI13) (Non-intrinsic ABC protein 11) (AtNAP11)	TGD3 ABCI13 NAP11 At1g65410 T8F5.19	Arabidopsis thaliana (Mouse-ear cress)		ATP-binding; Chloroplast; Complete proteome; Lipid transport; Nucleotide-binding; Plastid; Reference proteome; Transit peptide; Transport
SRR1271859_TR38961_c0_g1_i1_len=1017	PRS2_SOLTU	Q:740-273,H:1-155	38.12%ID	E:3e-24	Pathogenesis-related protein STH-2	PF00407.16^Bet_v_1^Pathogenesis-related protein Bet v I family^1-150^E:5.3e-12	0,798	0,336	0	1,921	0,021	0,664	0,001	0,942	0,732	-0,641	PRS2_SOLTU	Pathogenesis-related protein STH-2	STH-2	Solanum tuberosum (Potato)		Complete proteome; Pathogenesis-related protein; Plant defense; Reference proteome
SRR1271859_TR45289_c0_g1_i3_len=1673	NA	NA	NA	NA	NA	.	1	0,465	0	7,062	0,003	1,416	0	2,052	0,254	3,39	.	.	.	.	.	.
SRR342017_TR18428_c3_g5_i6_len=3652	OPR11_ORYSJ	Q:2135-1071,H:9-363	78.87%ID	E:0	Putative 12-oxophytodienoate reductase 11	PF00724.17^Oxidored_FMN^NADH:flavin oxidoreductase / NADH oxidase family^20-354^E:6.5e-89	0,886	0,138	0	1,431	0,073	1,892	0	3,861	1	0,151	OPR11_ORYSJ	Putative 12-oxophytodienoate reductase 11 (EC 1.3.1.-) (OPDA-reductase 11) (OsOPR11)	OPR11 OPR3 OsJ_20711	Oryza sativa subsp. japonica (Rice)		Complete proteome; FMN; Fatty acid biosynthesis; Fatty acid metabolism; Flavoprotein; Lipid biosynthesis; Lipid metabolism; NADP; Oxidoreductase; Oxylipin biosynthesis; Reference proteome
SRR342017_TR18700_c0_g1_i1_len=1744	C74B2_ARATH	Q:1572-211,H:38-492	55.92%ID	E:0	Linolenate hydroperoxide lyase, chloroplastic {ECO:0000305|PubMed:9701595}	PF00067.19^p450^Cytochrome P450^161-502^E:3.4e-25	0,002	1,016	0	2,199	0,006	0,653	0,001	0,797	0,052	0,479	C74B2_ARATH	Linolenate hydroperoxide lyase, chloroplastic (EC 4.2.99.-) (Cytochrome P450 74B2) (Hydroperoxide lyase 1)	CYP74B2 HPL1	Arabidopsis thaliana (Mouse-ear cress)		Chloroplast; Heme; Iron; Lyase; Metal-binding; Plastid; Transit peptide
SRR342017_TR22867_c3_g6_i4_len=994	NA	NA	NA	NA	NA	PF13405.3^EF-hand_6^EF-hand domain^19-44^E:5.9e-06`PF13499.3^EF-hand_7^EF-hand domain pair^19-89^E:1.8e-09`PF13202.3^EF-hand_5^EF hand^20-43^E:4.8e-07`PF13833.3^EF-hand_8^EF-hand domain pair^20-40^E:0.02`PF13202.3^EF-hand_5^EF hand^73-89^E:0.018`PF13202.3^EF-hand_5^EF hand^113-135^E:8.9e-06`PF13405.3^EF-hand_6^EF-hand domain^113-138^E:2.1e-05`PF13833.3^EF-hand_8^EF-hand domain pair^114-138^E:0.011	0,233	0,79	0	2,456	0,008	0,745	0	1,82	0,62	-0,237	.	.	.	.	.	.
SRR342017_TR25990_c0_g1_i2_len=2253	CIGR1_ORYSJ	Q:301-2046,H:1-571	57.14%ID	E:0	Chitin-inducible gibberellin-responsive protein 1	PF03514.11^GRAS^GRAS domain family^212-581^E:8.5e-128	0,312	0,755	0	3,77	0,01	1,127	0	1,792	0,793	-0,158	CIGR1_ORYSJ	Chitin-inducible gibberellin-responsive protein 1	CIGR1 Os07g0545800 LOC_Os07g36170 P0006G05.102	Oryza sativa subsp. japonica (Rice)		Complete proteome; Nucleus; Reference proteome; Transcription; Transcription regulation
SRR342017_TR27692_c0_g1_i2_len=1548	10HGO_CATRO	Q:1448-540,H:1-303	97.36%ID	E:2e-175	8-hydroxygeraniol dehydrogenase	PF08240.9^ADH_N^Alcohol dehydrogenase GroES-like domain^38-151^E:6.2e-29`PF02826.16^2-Hacid_dh_C^D-isomer specific 2-hydroxyacid dehydrogenase, NAD binding domain^177-226^E:5.2e-06`PF01262.18^AlaDh_PNT_C^Alanine dehydrogenase/PNT, C-terminal domain^185-256^E:6.9e-06`PF00107.23^ADH_zinc_N^Zinc-binding dehydrogenase^194-295^E:8.5e-14	1	0,089	0	4,474	0,005	6,26	0	7,174	1	-1,832	10HGO_CATRO	8-hydroxygeraniol dehydrogenase (Cr10HGO) (EC 1.1.1.324)	10HGO	Catharanthus roseus (Madagascar periwinkle) (Vinca rosea)		Metal-binding; NADP; Oxidoreductase; Zinc
SRR342019_TR16612_c0_g1_i7_len=2717	ALKBH_ARATH	Q:166-1242,H:1-345	60.99%ID	E:3e-135	Alpha-ketoglutarate-dependent dioxygenase alkB	PF13532.3^2OG-FeII_Oxy_2^2OG-Fe(II) oxygenase superfamily^171-356^E:2.8e-38	0,87	0,195	0	1,932	0,004	0,903	0	1,295	1	-0,23	ALKBH_ARATH	Alpha-ketoglutarate-dependent dioxygenase alkB (EC 1.14.11.-) (Alkylated DNA repair protein alkB homolog)	At1g11780 F25C20.6	Arabidopsis thaliana (Mouse-ear cress)		Complete proteome; DNA damage; DNA repair; Dioxygenase; Iron; Metal-binding; Oxidoreductase; Reference proteome
SRR342019_TR24844_c0_g1_i1_len=1199	AX22B_VIGRR	Q:129-704,H:1-196	65.67%ID	E:2e-83	Auxin-induced protein 22B	PF02309.13^AUX_IAA^AUX/IAA family^41-189^E:3.8e-55	1	0,051	0	1,89	0,089	0,447	0,001	0,882	0,064	-0,934	AX22B_VIGRR	Auxin-induced protein 22B (Indole-3-acetic acid-induced protein ARG4)	AUX22B ARG4	Vigna radiata var. radiata (Mung bean) (Phaseolus aureus)		Auxin signaling pathway; Nucleus; Repressor; Transcription; Transcription regulation
SRR342019_TR29297_c2_g5_i1_len=1342	EXPA1_ARATH	Q:172-897,H:7-250	85.66%ID	E:4e-115	Expansin-A1	PF03330.15^DPBB_1^Lytic transglycolase^60-145^E:3.7e-22`PF01357.18^Pollen_allerg_1^Pollen allergen^156-233^E:1.2e-30	0,087	0,475	0	1,499	0,855	-0,066	0	1,466	0,175	0,41	EXPA1_ARATH	Expansin-A1 (AtEXPA1) (Alpha-expansin-1) (At-EXP1) (AtEx1) (Ath-ExpAlpha-1.2)	EXPA1 EXP1 At1g69530 F10D13_18	Arabidopsis thaliana (Mouse-ear cress)		Alternative splicing; Cell wall; Cell wall biogenesis/degradation; Complete proteome; Disulfide bond; Membrane; Reference proteome; Secreted; Signal
SRR342019_TR38071_c4_g2_i9_len=2062	ARGE_ARATH	Q:11-805,H:12-276	74.06%ID	E:5e-136	Acetylornithine deacetylase	PF01546.25^Peptidase_M20^Peptidase family M20/M25/M40^90-194^E:8.9e-20	0,007	2,004	0	3,095	0,004	4,251	0	5,04	0,095	0,744	ARGE_ARATH	Acetylornithine deacetylase (EC 3.5.1.16) (N-acetylornithinase) (AO) (Acetylornithinase) (NAO)	At4g17830 dl4945w T6K21	Arabidopsis thaliana (Mouse-ear cress)	PATHWAY: Amino-acid biosynthesis; L-arginine biosynthesis; L-ornithine from N(2)-acetyl-L-ornithine (linear): step 1/1.	Alternative splicing; Amino-acid biosynthesis; Arginine biosynthesis; Cobalt; Complete proteome; Hydrolase; Metal-binding; Reference proteome; Zinc
SRR342022_TR1339_c0_g1_i1_len=988	NA	NA	NA	NA	NA	.	0,019	1,06	0	2,115	0,407	0,453	0	1,635	0,781	0,542	.	.	.	.	.	.
SRR342022_TR5402_c1_g18_i2_len=3100	THD1_ARATH	Q:990-2561,H:72-592	76.24%ID	E:0	Threonine dehydratase biosynthetic, chloroplastic	PF04576.12^Zein-binding^Zein-binding^21-111^E:6.5e-33`PF00291.22^PALP^Pyridoxal-phosphate dependent enzyme^370-656^E:2.7e-81`PF00585.15^Thr_dehydrat_C^C-terminal regulatory domain of Threonine dehydratase^669-759^E:2.9e-22`PF00585.15^Thr_dehydrat_C^C-terminal regulatory domain of Threonine dehydratase^765-850^E:6.4e-23	1	0,151	0	3,729	0,059	1,753	0,001	2,62	1	0,065	THD1_ARATH	Threonine dehydratase biosynthetic, chloroplastic (EC 4.3.1.19) (Threonine deaminase) (TD)	OMR1 At3g10050 T22K18.12	Arabidopsis thaliana (Mouse-ear cress)	PATHWAY: Amino-acid biosynthesis; L-isoleucine biosynthesis; 2-oxobutanoate from L-threonine: step 1/1.	Allosteric enzyme; Amino-acid biosynthesis; Branched-chain amino acid biosynthesis; Chloroplast; Complete proteome; Isoleucine biosynthesis; Lyase; Plastid; Pyridoxal phosphate; Reference proteome; Repeat; Transit peptide
SRR342022_TR9319_c1_g5_i2_len=1841	ABAH2_ARATH	Q:286-1590,H:48-479	72.54%ID	E:0	Abscisic acid 8'-hydroxylase 2	PF00067.19^p450^Cytochrome P450^63-474^E:3.1e-74	0,428	0,372	0	1,349	0,021	0,914	0	1,877	0,683	-0,221	ABAH2_ARATH	Abscisic acid 8'-hydroxylase 2 (ABA 8'-hydroxylase 2) (EC 1.14.13.93) (Cytochrome P450 707A2)	CYP707A2 At2g29090 T9I4.17	Arabidopsis thaliana (Mouse-ear cress)	PATHWAY: Plant hormone degradation; abscisic acid degradation.	Complete proteome; Heme; Iron; Membrane; Metal-binding; Monooxygenase; NADP; Oxidoreductase; Reference proteome; Stress response; Transmembrane; Transmembrane helix
SRR342022_TR13973_c1_g18_i1_len=2290	PTR30_ARATH	Q:118-1878,H:1-581	72.96%ID	E:0	Protein NRT1/ PTR FAMILY 5.1	PF00854.18^PTR2^POT family^97-525^E:4.7e-108	0,807	0,121	0	2,961	0,014	0,9	0	1,454	1	0,003	PTR30_ARATH	Protein NRT1/ PTR FAMILY 5.1 (AtNPF5.1)	NPF5.1 At2g40460 T2P4.19	Arabidopsis thaliana (Mouse-ear cress)		Complete proteome; Membrane; Phosphoprotein; Reference proteome; Transmembrane; Transmembrane helix; Transport
SRR342022_TR21932_c0_g1_i2_len=2000	ISPE_SOLLC	Q:1518-460,H:31-382	83.29%ID	E:0	4-diphosphocytidyl-2-C-methyl-D-erythritol kinase, chloroplastic/chromoplastic	PF00288.23^GHMP_kinases_N^GHMP kinases N terminal domain^182-224^E:3.5e-06`PF08544.10^GHMP_kinases_C^GHMP kinases C terminal^310-364^E:0.00022	0,208	0,343	0	1,592	0,007	0,84	0	1,186	1	-0,138	ISPE_SOLLC	4-diphosphocytidyl-2-C-methyl-D-erythritol kinase, chloroplastic/chromoplastic (EC 2.7.1.148) (4-(cytidine-5'-diphospho)-2-C-methyl-D-erythritol kinase) (CMK) (Ripening-associated protein pTOM41) (Fragment)	ISPE	Solanum lycopersicum (Tomato) (Lycopersicon esculentum)	PATHWAY: Isoprenoid biosynthesis; isopentenyl diphosphate biosynthesis via DXP pathway; isopentenyl diphosphate from 1-deoxy-D-xylulose 5-phosphate: step 3/6.	ATP-binding; Chloroplast; Chromoplast; Complete proteome; Isoprene biosynthesis; Kinase; Nucleotide-binding; Plastid; Reference proteome; Transferase; Transit peptide
SRR342023_TR14691_c0_g2_i1_len=2457	HT1_ARATH	Q:1295-2008,H:110-341	38.52%ID	E:6e-42	Serine/threonine-protein kinase HT1	PF12796.4^Ank_2^Ankyrin repeats (3 copies)^47-135^E:5e-14`PF13637.3^Ank_4^Ankyrin repeats (many copies)^47-96^E:1.5e-07`PF00023.27^Ank^Ankyrin repeat^76-106^E:6.4e-05`PF13606.3^Ank_3^Ankyrin repeat^76-103^E:0.00035`PF07714.14^Pkinase_Tyr^Protein tyrosine kinase^175-418^E:1.6e-56`PF00069.22^Pkinase^Protein kinase domain^177-415^E:3.9e-51	0,093	0,979	0	2,666	0,004	2,082	0	3,412	0,472	-0,699	HT1_ARATH	Serine/threonine-protein kinase HT1 (EC 2.7.11.1) (High leaf temperature protein 1)	HT1 At1g62400 F24O1.13	Arabidopsis thaliana (Mouse-ear cress)		ATP-binding; Complete proteome; Kinase; Nucleotide-binding; Reference proteome; Serine/threonine-protein kinase; Transferase
SRR342023_TR24474_c0_g1_i1_len=724	SUI1_ORYSJ	Q:215-553,H:1-115	92.17%ID	E:1e-70	Protein translation factor SUI1 homolog	PF01253.19^SUI1^Translation initiation factor SUI1^28-102^E:5.4e-26	0,709	0,109	0	1,197	0,008	0,734	0	1,07	0,829	0,051	SUI1_ORYSJ	Protein translation factor SUI1 homolog (Protein GOS2) (Protein eIF1) (Translation initiation factor 1)	GOS2 Os07g0529800 LOC_Os07g34589	Oryza sativa subsp. japonica (Rice)		Complete proteome; Initiation factor; Protein biosynthesis; Reference proteome; Translation regulation
SRR342023_TR24864_c0_g3_i1_len=988	KPRS2_SPIOL	Q:494-988,H:39-206	77.98%ID	E:8e-89	Ribose-phosphate pyrophosphokinase 2, chloroplastic	PF13793.3^Pribosyltran_N^N-terminal domain of ribose phosphate pyrophosphokinase^104-220^E:9.7e-50	1	0,006	0	3,39	0,066	0,982	0,001	1,62	0,564	0,508	KPRS2_SPIOL	Ribose-phosphate pyrophosphokinase 2, chloroplastic (EC 2.7.6.1) (Phosphoribosyl pyrophosphate synthase 2)	PRS2	Spinacia oleracea (Spinach)		ATP-binding; Chloroplast; Kinase; Magnesium; Metal-binding; Nucleotide biosynthesis; Nucleotide-binding; Plastid; Transferase; Transit peptide
SRR646572_TR16312_c0_g4_i1_len=1107	NA	NA	NA	NA	NA	.	1	0,046	0	2,521	0,014	1,449	0	2,126	1	0,904	.	.	.	.	.	.
SRR646604_TR9738_c0_g2_i1_len=1683	NA	NA	NA	NA	NA	.	0,319	1,106	0	3,241	0,017	2,023	0	3,36	1	1,856	.	.	.	.	.	.
SRR646604_TR31316_c0_g2_i1_len=3589	THD1_ARATH	Q:2587-1724,H:72-357	77.24%ID	E:3e-136	Threonine dehydratase biosynthetic, chloroplastic	PF00504.18^Chloroa_b-bind^Chlorophyll A-B binding protein^84-199^E:8.2e-27`PF00291.22^PALP^Pyridoxal-phosphate dependent enzyme^375-620^E:1.5e-73	0,839	0,14	0	2,628	0,05	1,033	0	2,157	1	-0,575	THD1_ARATH	Threonine dehydratase biosynthetic, chloroplastic (EC 4.3.1.19) (Threonine deaminase) (TD)	OMR1 At3g10050 T22K18.12	Arabidopsis thaliana (Mouse-ear cress)	PATHWAY: Amino-acid biosynthesis; L-isoleucine biosynthesis; 2-oxobutanoate from L-threonine: step 1/1.	Allosteric enzyme; Amino-acid biosynthesis; Branched-chain amino acid biosynthesis; Chloroplast; Complete proteome; Isoleucine biosynthesis; Lyase; Plastid; Pyridoxal phosphate; Reference proteome; Repeat; Transit peptide
SRR646604_TR37510_c1_g1_i2_len=2524	KPRS2_SPIOL	Q:1657-596,H:39-395	84.87%ID	E:0	Ribose-phosphate pyrophosphokinase 2, chloroplastic	PF13793.3^Pribosyltran_N^N-terminal domain of ribose phosphate pyrophosphokinase^2-98^E:9.5e-43`PF00156.24^Pribosyltran^Phosphoribosyl transferase domain^133-229^E:1.7e-15`PF14572.3^Pribosyl_synth^Phosphoribosyl synthetase-associated domain^184-291^E:2.1e-23	0,83	0,126	0	2,268	0,002	1,493	0	2,059	0,718	-0,177	KPRS2_SPIOL	Ribose-phosphate pyrophosphokinase 2, chloroplastic (EC 2.7.6.1) (Phosphoribosyl pyrophosphate synthase 2)	PRS2	Spinacia oleracea (Spinach)		ATP-binding; Chloroplast; Kinase; Magnesium; Metal-binding; Nucleotide biosynthesis; Nucleotide-binding; Plastid; Transferase; Transit peptide
SRR648705_TR3147_c0_g2_i1_len=2470	NA	NA	NA	NA	NA	PF07676.9^PD40^WD40-like Beta Propeller Repeat^518-547^E:0.00018`PF07676.9^PD40^WD40-like Beta Propeller Repeat^561-587^E:2.4e-07	0,865	-0,229	0	2,178	0,001	2,133	0	4,027	0,524	0,635	.	.	.	.	.	.
SRR648705_TR3706_c0_g2_i1_len=1432	NA	NA	NA	NA	NA	PF00168.27^C2^C2 domain^6-99^E:2.1e-09	0,436	0,237	0	1,575	0,014	0,79	0	1,12	0,403	0,261	.	.	.	.	.	.
SRR648705_TR12973_c0_g2_i1_len=3944	CLPD_ARATH	Q:3008-318,H:42-943	70.88%ID	E:0	Chaperone protein ClpD, chloroplastic {ECO:0000303|PubMed:11299370}	PF02861.17^Clp_N^Clp amino terminal domain, pathogenicity island component^116-164^E:2e-06`PF02861.17^Clp_N^Clp amino terminal domain, pathogenicity island component^200-248^E:8.5e-10`PF00004.26^AAA^ATPase family associated with various cellular activities (AAA)^337-439^E:6.2e-09`PF07724.11^AAA_2^AAA domain (Cdc48 subfamily)^679-854^E:2.9e-51`PF00004.26^AAA^ATPase family associated with various cellular activities (AAA)^684-792^E:2.8e-06`PF07728.11^AAA_5^AAA domain (dynein-related subfamily)^684-804^E:2e-11`PF10431.6^ClpB_D2-small^C-terminal, D2-small domain, of ClpB protein^860-939^E:1.8e-22	0,111	0,884	0	3,945	0,001	1,705	0	1,897	1	0,826	CLPD_ARATH	Chaperone protein ClpD, chloroplastic (EC 3.6.1.3) (ATP-dependent Clp protease ATP-binding subunit ClpD homolog) (Casein lytic proteinase D) (ERD1 protein) (Protein EARLY RESPONSIVE TO DEHYDRATION 1) (Protein SENESCENCE ASSOCIATED GENE 15)	CLPD ERD1 SAG15 At5g51070 K3K7.27	Arabidopsis thaliana (Mouse-ear cress)		ATP-binding; Chaperone; Chloroplast; Complete proteome; Hydrolase; Nucleotide-binding; Plastid; Reference proteome; Repeat; Stress response; Transit peptide
SRR648705_TR19380_c1_g2_i4_len=1366	NA	NA	NA	NA	NA	.	0,487	0,317	0	1,582	0,005	1,201	0	1,548	0,111	-0,886	.	.	.	.	.	.
SRR648705_TR19880_c0_g1_i2_len=1719	BEBT_TOBAC	Q:235-1251,H:3-340	76.11%ID	E:0	Benzyl alcohol O-benzoyltransferase	PF02458.12^Transferase^Transferase family^14-341^E:5.9e-66	0,195	-1,177	0	3,102	0,164	0,875	0	1,985	1	0,562	BEBT_TOBAC	Benzyl alcohol O-benzoyltransferase (EC 2.3.1.196) (Benzoyl coenzyme A:benzyl alcohol benzoyl transferase)	HSR201	Nicotiana tabacum (Common tobacco)		Acyltransferase; Transferase
SRR648705_TR21981_c0_g1_i1_len=994	ZAT8_ARATH	Q:764-324,H:12-150	46.05%ID	E:9e-31	Zinc finger protein ZAT8	PF13912.3^zf-C2H2_6^C2H2-type zinc finger^113-137^E:2.3e-10`PF13912.3^zf-C2H2_6^C2H2-type zinc finger^157-182^E:1.7e-09	1	2,397	0	5,812	0,043	3,273	0	5,337	1	0,028	ZAT8_ARATH	Zinc finger protein ZAT8	ZAT8 At3g46080 F12M12.50	Arabidopsis thaliana (Mouse-ear cress)		Complete proteome; Metal-binding; Nucleus; Reference proteome; Repeat; Transcription; Transcription regulation; Zinc; Zinc-finger
SRR648705_TR23749_c0_g2_i2_len=3398	OPR11_ORYSJ	Q:338-1402,H:9-363	78.87%ID	E:0	Putative 12-oxophytodienoate reductase 11	.	0,534	-0,278	0	1,736	0,461	1,261	0	3,611	0,678	-0,149	OPR11_ORYSJ	Putative 12-oxophytodienoate reductase 11 (EC 1.3.1.-) (OPDA-reductase 11) (OsOPR11)	OPR11 OPR3 OsJ_20711	Oryza sativa subsp. japonica (Rice)		Complete proteome; FMN; Fatty acid biosynthesis; Fatty acid metabolism; Flavoprotein; Lipid biosynthesis; Lipid metabolism; NADP; Oxidoreductase; Oxylipin biosynthesis; Reference proteome
SRR648705_TR31575_c0_g1_i2_len=2132	4CL_VANPL	Q:2011-353,H:1-553	99.82%ID	E:0	4-coumarate--CoA ligase	PF00501.25^AMP-binding^AMP-binding enzyme^37-451^E:1.5e-107`PF13193.3^AMP-binding_C^AMP-binding enzyme C-terminal domain^460-535^E:3e-15	0,298	0,633	0	5,21	0,004	0,784	0	1,439	0,003	1,871	4CL_VANPL	4-coumarate--CoA ligase (4CL) (EC 6.2.1.12) (4-coumaroyl-CoA synthase)	4CL	Vanilla planifolia (Vanilla)	PATHWAY: Phytoalexin biosynthesis; 3,4',5-trihydroxystilbene biosynthesis; 3,4',5-trihydroxystilbene from trans-4-coumarate: step 1/2.	ATP-binding; Ligase; Nucleotide-binding; Phenylpropanoid metabolism
																					PATHWAY: Amino-acid biosynthesis; L-lysine biosynthesis via DAP pathway; (S)-	

SRR648705_TR42446_c0_g1_i1_len=1554	DHAS_SYNY3	Q:1240-233,H:7-334	54.17%ID	E:8e-118	Aspartate-semialdehyde dehydrogenase	PF01118.21^Semialdhyde_dh^Semialdehyde dehydrogenase, NAD binding domain^52-164^E:1.8e-32`PF02774.15^Semialdhyde_dhC^Semialdehyde dehydrogenase, dimerisation domain^192-372^E:1.1e-44	0,058	-0,889	0	1,244	0,027	1,351	0	2,216	0,356	-1,249	DHAS_SYNY3	Aspartate-semialdehyde dehydrogenase (ASA dehydrogenase) (ASADH) (EC 1.2.1.11) (Aspartate-beta-semialdehyde dehydrogenase)	asd slr0549	Synechocystis sp. (strain PCC 6803 / Kazusa)	tetrahydrodipicolinate from L-aspartate: step 2/4.; PATHWAY: Amino-acid biosynthesis; L-methionine biosynthesis via de novo pathway; L-homoserine from L-aspartate: step 2/3.; PATHWAY: Amino-acid biosynthesis; L-threonine biosynthesis; L-threonine from L-aspartate: step 2/5.	Amino-acid biosynthesis; Complete proteome; Diaminopimelate biosynthesis; Lysine biosynthesis; Methionine biosynthesis; NADP; Oxidoreductase; Reference proteome; Threonine biosynthesis
SRR648705_TR43829_c0_g1_i2_len=2224	CYSZ_CUCMA	Q:197-1708,H:12-515	86.93%ID	E:0	Citrate synthase, glyoxysomal	PF00285.18^Citrate_synt^Citrate synthase, C-terminal domain^101-469^E:3.4e-134	0,333	0,402	0	2,438	0,002	1,455	0	1,671	0,735	0,203	CYSZ_CUCMA	Citrate synthase, glyoxysomal (EC 2.3.3.16) (GCS)		Cucurbita maxima (Pumpkin) (Winter squash)	PATHWAY: Carbohydrate metabolism; glyoxylate cycle; isocitrate from oxaloacetate: step 1/2.	Direct protein sequencing; Glyoxylate bypass; Glyoxysome; Peroxisome; Transferase; Transit peptide; Tricarboxylic acid cycle
SRR648705_TR44108_c0_g1_i1_len=1039	NA	NA	NA	NA	NA	.	1	0,492	0	2,588	0,001	4,414	0	5,064	0,517	-0,824	.	.	.	.	.	.
SRR648707_TR3831_c0_g4_i1_len=3888	MMT1_WEDBI	Q:274-3525,H:1-1087	73.33%ID	E:0	Methionine S-methyltransferase	PF05175.11^MTS^Methyltransferase small domain^119-172^E:1.3e-06`PF06325.10^PrmA^Ribosomal protein L11 methyltransferase (PrmA)^119-172^E:1.1e-05`PF00155.18^Aminotran_1_2^Aminotransferase class I and II^734-1074^E:2.3e-16	0,819	0,117	0	2,637	0,003	1,463	0	2,46	0,705	0,253	MMT1_WEDBI	Methionine S-methyltransferase (EC 2.1.1.12) (AdoMet:Met S-methyltransferase)	MMT1 MMT	Wedelia biflora (Beach sunflower) (Wollastonia biflora)		Cytoplasm; Direct protein sequencing; Methyltransferase; S-adenosyl-L-methionine; Transferase
SRR648707_TR6083_c0_g1_i1_len=1103	NA	NA	NA	NA	NA	.	0,006	1,276	0	1,79	0,003	1,299	0	1,747	0,02	1,071	.	.	.	.	.	.
SRR648707_TR6922_c1_g1_i3_len=1527	DBR_TOBAC	Q:1333-302,H:1-343	82.56%ID	E:0	2-alkenal reductase (NADP(+)-dependent)	PF16884.2^ADH_N_2^N-terminal domain of oxidoreductase^8-116^E:1.6e-26`PF00107.23^ADH_zinc_N^Zinc-binding dehydrogenase^166-293^E:1.6e-24`PF13602.3^ADH_zinc_N_2^Zinc-binding dehydrogenase^200-337^E:1.7e-07	0,452	0,504	0	2,354	0,001	1,383	0	1,577	1	0,055	DBR_TOBAC	2-alkenal reductase (NADP(+)-dependent) (EC 1.3.1.102) (Alkenal double bound reductase) (Allyl-alcohol dehydrogenase) (EC 1.1.1.54) (Allylic alcohol dehydrogenase 1) (allyl-ADH1) (Flavin-free double bond reductase) (NtDBR) (Pulegone reductase) (NtRed-1)	DBR	Nicotiana tabacum (Common tobacco)		3D-structure; Direct protein sequencing; NADP; Oxidoreductase
SRR648707_TR8635_c0_g2_i1_len=1931	UGT9_GARJA	Q:271-1596,H:1-435	56.66%ID	E:1e-157	Beta-D-glucosyl crocetin beta-1,6-glucosyltransferase	PF00201.15^UDPGT^UDP-glucoronosyl and UDP-glucosyl transferase^251-421^E:3.7e-22`PF04101.13^Glyco_tran_28_C^Glycosyltransferase family 28 C-terminal domain^356-436^E:0.00025	0,692	1,257	0	5,046	0,002	3,151	0	3,956	1	0,328	UGT9_GARJA	Beta-D-glucosyl crocetin beta-1,6-glucosyltransferase (EC 2.4.1.330) (UDP-glucose glucosyltransferase 9) (GjUGT9) (UDP-glycosyltransferase 94E5)	UGT94E5 UGT9	Gardenia jasminoides (Cape jasmine) (Gardenia augusta)		Glycosyltransferase; Transferase
SRR648707_TR9966_c0_g1_i1_len=3049	RSH3C_ARATH	Q:2510-324,H:6-712	68.48%ID	E:0	Probable GTP diphosphokinase RSH3, chloroplastic	PF13328.3^HD_4^HD domain^238-390^E:9.3e-43`PF01966.19^HD^HD domain^255-359^E:2.5e-06`PF04607.14^RelA_SpoT^Region found in RelA / SpoT proteins^449-558^E:1.3e-36	0,103	1,561	0	5,064	0,003	2,231	0	2,621	1	0,507	RSH3C_ARATH	Probable GTP diphosphokinase RSH3, chloroplastic (EC 2.7.6.5) (RelA/SpoT homolog 3) (AtRSH3) (ppGpp synthetase RSH3)	RSH3 At1g54130 F15I1.23	Arabidopsis thaliana (Mouse-ear cress)		ATP-binding; Chloroplast; Complete proteome; GTP-binding; Kinase; Nucleotide-binding; Plastid; Reference proteome; Stress response; Transferase; Transit peptide
SRR648707_TR10026_c0_g2_i1_len=2229	PMGI_MESCR	Q:210-1880,H:1-557	89.05%ID	E:0	2,3-bisphosphoglycerate-independent phosphoglycerate mutase	PF01676.15^Metalloenzyme^Metalloenzyme superfamily^22-545^E:4.7e-101`PF06415.10^iPGM_N^BPG-independent PGAM N-terminus (iPGM_N)^102-322^E:6.4e-59	0,001	-2,264	0	2,743	0,006	1,921	0,001	2,244	0	-2,869	PMGI_MESCR	2,3-bisphosphoglycerate-independent phosphoglycerate mutase (BPG-independent PGAM) (Phosphoglyceromutase) (EC 5.4.2.12) (PGAM-I)	PGM1	Mesembryanthemum crystallinum (Common ice plant) (Cryophytum crystallinum)	PATHWAY: Carbohydrate degradation; glycolysis; pyruvate from D-glyceraldehyde 3-phosphate: step 3/5.	Cytoplasm; Glycolysis; Isomerase; Manganese; Metal-binding; Stress response
SRR648707_TR13267_c0_g1_i1_len=2224	HEM11_CUCSA	Q:206-1759,H:34-552	69.17%ID	E:0	Glutamyl-tRNA reductase 1, chloroplastic	PF05201.12^GlutR_N^Glutamyl-tRNAGlu reductase, N-terminal domain^98-248^E:5.3e-40`PF01488.17^Shikimate_DH^Shikimate / quinate 5-dehydrogenase^263-405^E:4.5e-45`PF02423.12^OCD_Mu_crystall^Ornithine cyclodeaminase/mu-crystallin family^277-366^E:1.6e-07`PF03807.14^F420_oxidored^NADP oxidoreductase coenzyme F420-dependent^278-347^E:3.4e-08`PF03446.12^NAD_binding_2^NAD binding domain of 6-phosphogluconate dehydrogenase^278-379^E:2e-06`PF00745.17^GlutR_dimer^Glutamyl-tRNAGlu reductase, dimerisation domain^420-523^E:9.7e-26	0,228	0,965	0	2,526	0,002	2,132	0	2,489	0,461	-0,908	HEM11_CUCSA	Glutamyl-tRNA reductase 1, chloroplastic (GluTR) (EC 1.2.1.70)	HEMA1	Cucumis sativus (Cucumber)	PATHWAY: Porphyrin-containing compound metabolism; protoporphyrin-IX biosynthesis; 5-aminolevulinate from L-glutamyl-tRNA(Glu): step 1/2.	Chlorophyll biosynthesis; Chloroplast; NADP; Oxidoreductase; Plastid; Porphyrin biosynthesis; Transit peptide
SRR648707_TR34996_c1_g1_i2_len=2295	TT12_ARATH	Q:781-2106,H:51-493	41.22%ID	E:2e-93	.	PF01554.15^MatE^MatE^40-198^E:4.2e-23`PF01554.15^MatE^MatE^260-420^E:2.6e-25	0,452	0,634	0	2,603	0,003	1,293	0	2,091	0,298	-0,714	DTX41_ARATH	Protein DETOXIFICATION 41 (AtDTX41) (Multidrug and toxic compound extrusion protein 41) (MATE protein 41) (Protein TANNIN-DEFICIENT SEED 3) (Protein TRANSPARENT TESTA 12)	DTX41 TDS3 TT12 At3g59030 F17J16_80	Arabidopsis thaliana (Mouse-ear cress)	PATHWAY: Secondary metabolite biosynthesis; flavonoid biosynthesis.	Antiport; Complete proteome; Flavonoid biosynthesis; Membrane; Reference proteome; Transmembrane; Transmembrane helix; Transport; Vacuole
SRR924147_TR8076_c0_g2_i1_len=1466	ROMT_VITVI	Q:375-1394,H:14-356	57.39%ID	E:2e-123	Trans-resveratrol di-O-methyltransferase	PF08100.8^Dimerisation^Dimerisation domain^38-86^E:6.4e-18`PF00891.15^Methyltransf_2^O-methyltransferase^127-346^E:8.6e-65	0,502	2,357	0	6,108	0,034	3,97	0	5,063	1	-0,975	ROMT_VITVI	Trans-resveratrol di-O-methyltransferase (EC 2.1.1.240) (Resveratrol O-methyltransferase) (ROMT) (VvROMT)	ROMT VIT_12s0028g01880	Vitis vinifera (Grape)		Complete proteome; Methyltransferase; Reference proteome; S-adenosyl-L-methionine; Transferase
SRR924147_TR16793_c0_g1_i1_len=1821	LGT_CITUN	Q:1702-284,H:1-471	63.66%ID	E:0	Limonoid UDP-glucosyltransferase	PF00201.15^UDPGT^UDP-glucoronosyl and UDP-glucosyl transferase^262-395^E:5.6e-22	0,56	0,932	0	4,667	0,019	0,827	0,001	1,149	0,129	1,163	LGT_CITUN	Limonoid UDP-glucosyltransferase (LGTase) (Limonoid GTase) (Limonoid glucosyltransferase) (EC 2.4.1.210)		Citrus unshiu (Satsuma mandarin) (Citrus nobilis var. unshiu)		Glycosyltransferase; Transferase
SRR924147_TR20190_c0_g1_i3_len=1689	AKRC9_ARATH	Q:281-1222,H:1-315	73.33%ID	E:8e-161	Aldo-keto reductase family 4 member C9	PF00248.18^Aldo_ket_red^Aldo/keto reductase family^19-285^E:1.1e-46	0,936	0,057	0	2,284	0,003	1,734	0	2,713	1	0,05	AKRC9_ARATH	Aldo-keto reductase family 4 member C9 (EC 1.1.1.-)	AKR4C9 At2g37770 F13M22 T8P21.32	Arabidopsis thaliana (Mouse-ear cress)		3D-structure; Acetylation; Alternative splicing; Complete proteome; Detoxification; NADP; Oxidoreductase; Reference proteome; Stress response
SRR924148_TR11602_c0_g1_i9_len=2687	THIC1_ARATH	Q:2506-1376,H:2-378	83.82%ID	E:0	Acetyl-CoA acetyltransferase, cytosolic 1	PF00108.20^Thiolase_N^Thiolase, N-terminal domain^15-273^E:2e-91`PF00109.23^ketoacyl-synt^Beta-ketoacyl synthase, N-terminal domain^96-136^E:0.00022`PF02803.15^Thiolase_C^Thiolase, C-terminal domain^283-403^E:1.5e-44	0,48	0,318	0	1,74	0,005	1,571	0,001	1,82	1	0,125	THIC1_ARATH	Acetyl-CoA acetyltransferase, cytosolic 1 (EC 2.3.1.9) (Cytosolic acetoacetyl-CoA thiolase 1) (Thiolase 1) (Protein EMBRYO DEFECTIVE 1276)	AAT1 EMB1276 At5g48230 MIF21.12	Arabidopsis thaliana (Mouse-ear cress)	PATHWAY: Metabolic intermediate biosynthesis; (R)-mevalonate biosynthesis; (R)-mevalonate from acetyl-CoA: step 1/3.	Acyltransferase; Alternative splicing; Complete proteome; Cytoplasm; Isoprene biosynthesis; Metal-binding; Potassium; Reference proteome; Transferase
SRR924148_TR13893_c4_g6_i5_len=2758	Y8948_DICDI	Q:312-1067,H:19-261	30.27%ID	E:2e-25	Putative methyltransferase DDB_G0268948	.	1	-0,116	0	7,375	0,001	2,572	0	2,875	1	0	Y8948_DICDI	Putative methyltransferase DDB_G0268948 (EC 2.1.1.-)	DDB_G0268948	Dictyostelium discoideum (Slime mold)		Complete proteome; Methyltransferase; Reference proteome; S-adenosyl-L-methionine; Transferase
SRR924148_TR18024_c0_g1_i1_len=636	EXPA1_ARATH	Q:489-1,H:7-171	86.06%ID	E:1e-66	Expansin-A1	PF03330.15^DPBB_1^Lytic transglycolase^103-188^E:2.6e-22	0,292	0,278	0	2,215	0,364	-0,282	0	1,45	0,018	1,148	EXPA1_ARATH	Expansin-A1 (AtEXPA1) (Alpha-expansin-1) (At-EXP1) (AtEx1) (Ath-ExpAlpha-1.2)	EXPA1 EXP1 At1g69530 F10D13_18	Arabidopsis thaliana (Mouse-ear cress)		Alternative splicing; Cell wall; Cell wall biogenesis/degradation; Complete proteome; Disulfide bond; Membrane; Reference proteome; Secreted; Signal
SRR122238_TR16898_c0_g1_i1_len=553	NA	NA	NA	NA	NA	.	0	8,597	0,688	0,124	0	1,353	0,108	0,508	0	-3,847	.	.	.	.	.	.
SRR122238_TR60344_c0_g1_i2_len=702	NA	NA	NA	NA	NA	.	0	1,183	0,821	0,095	0	2,112	0,001	1,063	0,369	0,29	.	.	.	.	.	.
SRR1271859_TR22866_c2_g2_i17_len=2668	TPSGD_VITVI	Q:1240-2517,H:134-557	54.93%ID	E:2e-161	(-)-germacrene D synthase	PF01397.18^Terpene_synth^Terpene synthase, N-terminal domain^1-66^E:4.7e-12`PF03936.13^Terpene_synth_C^Terpene synthase family, metal binding domain^97-363^E:1.7e-100	0,001	1,93	0,002	2,463	0	2,097	0,001	1,411	0,446	0,318	TPSGD_VITVI	(-)-germacrene D synthase (EC 4.2.3.22) (EC 4.2.3.75)	VIT_19s0014g04930 VIT_00014569001 VITISV_013313 VITISV_030783 Vv19s0014g04930	Vitis vinifera (Grape)	PATHWAY: Secondary metabolite biosynthesis; terpenoid biosynthesis.	Complete proteome; Cytoplasm; Lyase; Magnesium; Metal-binding; Reference proteome
SRR342019_TR36227_c0_g1_i1_len=768	VTI12_ARATH	Q:714-274,H:53-199	70.75%ID	E:2e-66	Vesicle transport v-SNARE 12	PF05008.12^V-SNARE^Vesicle transport v-SNARE protein N-terminus^19-57^E:6.3e-11`PF12352.5^V-SNARE_C^Snare region anchored in the vesicle membrane C-terminus^95-159^E:2.9e-24	0,001	6,637	0,783	0,201	0	6,948	1	0	0	-7,499	VTI12_ARATH	Vesicle transport v-SNARE 12 (AtVTI12) (Vesicle soluble NSF attachment protein receptor VTI1b) (AtVTI1b) (Vesicle transport v-SNARE protein VTI1b)	VTI12 VTI1B At1g26670 T24P13.5	Arabidopsis thaliana (Mouse-ear cress)		Acetylation; Coiled coil; Complete proteome; Golgi apparatus; Membrane; Protein transport; Reference proteome; Transmembrane; Transmembrane helix; Transport
SRR342023_TR9968_c1_g2_i1_len=1694	NA	NA	NA	NA	NA	PF05050.9^Methyltransf_21^Methyltransferase FkbM domain^293-444^E:0.00017	0	3,731	0,008	3,856	0	5,042	0,042	1,933	0,254	2,581	.	.	.	.	.	.
SRR648705_TR18574_c0_g1_i1_len=782	12KD_FRAAN	Q:64-438,H:1-109	57.6%ID	E:1e-37	Auxin-repressed 12.5 kDa protein	PF05564.9^Auxin_repressed^Dormancy/auxin associated protein^24-143^E:1.9e-56	0	7,677	0,003	1,309	0	6,786	0,129	4,955	0,629	-1,198	12KD_FRAAN	Auxin-repressed 12.5 kDa protein		Fragaria ananassa (Strawberry)		Auxin signaling pathway
SRR648707_TR6095_c0_g1_i1_len=568	SUT_SPIOL	Q:568-308,H:431-517	72.41%ID	E:4e-33	Sucrose transport protein	.	0	7,028	0	-2,224	0	7,892	1	0	1	0	SUT_SPIOL	Sucrose transport protein (Sucrose permease) (Sucrose-proton symporter)		Spinacia oleracea (Spinach)	PATHWAY: Glycan biosynthesis; sucrose metabolism.	Membrane; Sugar transport; Symport; Transmembrane; Transmembrane helix; Transport
SRR1144633_TR16170_c0_g3_i14_len=1726	ACOX4_ARATH	Q:1538-288,H:1-430	78.84%ID	E:0	Acyl-coenzyme A oxidase 4, peroxisomal	PF02771.13^Acyl-CoA_dh_N^Acyl-CoA dehydrogenase, N-terminal domain^47-152^E:1.8e-22`PF02770.16^Acyl-CoA_dh_M^Acyl-CoA dehydrogenase, middle domain^158-250^E:1.1e-22`PF00441.21^Acyl-CoA_dh_1^Acyl-CoA dehydrogenase, C-terminal domain^267-408^E:3.7e-25	0,003	1,633	0	1,867	0	1,896	0,005	1,57	1	0,022	ACOX4_ARATH	Acyl-coenzyme A oxidase 4, peroxisomal (AOX 4) (EC 1.3.3.6) (G6p) (Short-chain acyl-CoA oxidase) (AtCX4) (AtG6) (SAOX)	ACX4 G6 At3g51840 ATEM1.9	Arabidopsis thaliana (Mouse-ear cress)		3D-structure; Acetylation; Complete proteome; FAD; Fatty acid metabolism; Flavoprotein; Glyoxysome; Lipid metabolism; Oxidoreductase; Peroxisome; Reference proteome
SRR1144634_TR24946_c0_g1_i1_len=769	NA	NA	NA	NA	NA	.	0,254	3,507	0	5,211	0	2,01	0,064	0,957	1	0	.	.	.	.	.	.
SRR122236_TR3878_c0_g2_i1_len=680	NA	NA	NA	NA	NA	.	0,373	0,198	0	2,935	0	1,094	0,014	0,595	0,326	-0,3	.	.	.	.	.	.
SRR122238_TR3803_c0_g1_i1_len=948	WUN1_SOLTU	Q:281-607,H:1-105	67.89%ID	E:2e-43	Wound-induced protein 1	PF07107.8^WI12^Wound-induced protein WI12^94-202^E:2.8e-44	0,062	0,603	0	2,072	0	1,436	0,004	1,018	0	-1,487	WUN1_SOLTU	Wound-induced protein 1	WUN1	Solanum tuberosum (Potato)		Complete proteome; Reference proteome
SRR122238_TR10709_c0_g2_i2_len=1148	GST23_MAIZE	Q:229-873,H:3-218	40.09%ID	E:2e-41	Glutathione transferase GST 23	PF02798.17^GST_N^Glutathione S-transferase, N-terminal domain^4-75^E:6.5e-14`PF13417.3^GST_N_3^Glutathione S-transferase, N-terminal domain^7-78^E:6e-16`PF13409.3^GST_N_2^Glutathione S-transferase, N-terminal domain^12-76^E:3e-09`PF00043.22^GST_C^Glutathione S-transferase, C-terminal domain^107-193^E:1.7e-05`PF14497.3^GST_C_3^Glutathione S-transferase, C-terminal domain^116-198^E:2.5e-05	0,316	0,637	0,001	1,674	0,001	1,93	0,08	1,208	1	-0,107	GST23_MAIZE	Glutathione transferase GST 23 (EC 2.5.1.18) (Glutathione transferase GST 36)		Zea mays (Maize)		Complete proteome; Plant defense; Reference proteome; Transferase
SRR122238_TR32671_c0_g1_i4_len=2031	NPC2_ARATH	Q:261-1721,H:24-512	75.26%ID	E:0	Non-specific phospholipase C2	PF04185.11^Phosphoesterase^Phosphoesterase family^36-395^E:2.4e-108	0,34	0,463	0	1,782	0	1,952	0,005	1,173	0,454	0,369	NPC2_ARATH	Non-specific phospholipase C2 (EC 3.1.-.-)	NPC2 At2g26870 F12C20.9	Arabidopsis thaliana (Mouse-ear cress)		Complete proteome; Hydrolase; Reference proteome; Secreted; Signal
SRR122238_TR34090_c0_g1_i1_len=728	21KD_DAUCA	Q:539-54,H:28-189	56.17%ID	E:1e-64	21 kDa protein	PF04043.12^PMEI^Plant invertase/pectin methylesterase inhibitor^63-219^E:5.7e-35	0,8	0,072	0	2,186	0	1,354	0,356	0,368	1	0,048	21KD_DAUCA	21 kDa protein (1.2 protein)		Daucus carota (Wild carrot)		Signal
SRR122238_TR34437_c0_g1_i3_len=859	NA	NA	NA	NA	NA	.	0,101	0,529	0	1,443	0,001	1,327	0,008	1,102	1	-0,023	.	.	.	.	.	.
SRR122238_TR37876_c0_g1_i2_len=802	DCTP1_RAT	Q:675-337,H:20-132	49.56%ID	E:1e-21	dCTP pyrophosphatase 1	PF03819.14^MazG^MazG nucleotide pyrophosphohydrolase domain^34-102^E:1.7e-07`PF12643.4^MazG-like^MazG-like family^45-117^E:9e-08	0,133	1,154	0	5,143	0	4,555	0,004	3,553	0,732	0,765	DCTP1_RAT	dCTP pyrophosphatase 1 (EC 3.6.1.12) (Deoxycytidine-triphosphatase 1) (dCTPase 1) (RS21-C6) (XTP3-transactivated gene A protein homolog)	Dctpp1 Rs21c6 Xtp3tpa	Rattus norvegicus (Rat)		Acetylation; Complete proteome; Cytoplasm; Hydrolase; Magnesium; Metal-binding; Phosphoprotein; Reference proteome
SRR122238_TR57082_c0_g1_i1_len=607	NA	NA	NA	NA	NA	.	0,353	0,243	0	1,984	0	1,153	0,005	0,809	0,832	0,243	.	.	.	.	.	.
SRR122238_TR60996_c0_g1_i1_len=1427	NA	NA	NA	NA	NA	PF07816.8^DUF1645^Protein of unknown function (DUF1645)^74-282^E:3.8e-43	0,01	2,068	0	3,594	0	3,549	0,016	1,997	0,62	-0,499	.	.	.	.	.	.
SRR122238_TR63763_c0_g1_i5_len=1089	SRP_VITRI	Q:935-294,H:9-241	58.55%ID	E:5e-83	Stress-related protein	PF05755.9^REF^Rubber elongation factor protein (REF)^18-212^E:1.4e-78	0,297	0,408	0	1,455	0	1,837	0,009	1,203	1	-0,672	SRP_VITRI	Stress-related protein	SRP	Vitis riparia (Frost grape) (Vitis vulpina)		Stress response
SRR122238_TR73613_c0_g1_i4_len=825	NA	NA	NA	NA	NA	.	1	0,02	0	1,818	0	1,998	1	0,126	0,732	-0,441	.	.	.	.	.	.
SRR122238_TR80906_c0_g1_i3_len=1210	RTNLA_ARATH	Q:342-959,H:70-275	71.36%ID	E:1e-100	Reticulon-like protein B1	PF02453.14^Reticulon^Reticulon^70-225^E:4e-55	0,111	0,863	0	2,371	0	1,022	0,019	0,605	0,051	0,615	RTNLA_ARATH	Reticulon-like protein B1 (AtRTNLB1) (VirB2-interacting protein 1)	RTNLB1 BTI1 At4g23630 F9D16.100	Arabidopsis thaliana (Mouse-ear cress)		Acetylation; Cell membrane; Complete proteome; Endoplasmic reticulum; Membrane; Reference proteome; Transmembrane; Transmembrane helix
SRR122238_TR82636_c0_g6_i5_len=1138	PSB4_ARATH	Q:935-198,H:1-246	73.98%ID	E:2e-138	Proteasome subunit beta type-4	PF00227.23^Proteasome^Proteasome subunit^97-280^E:2.1e-33	0,345	0,325	0	1,545	0	1,554	0,001	1,155	1	0,109	PSB4_ARATH	Proteasome subunit beta type-4 (EC 3.4.25.1) (20S proteasome beta subunit G-1) (Proteasome component H) (Proteasome subunit beta type-7)	PBG1 PRCH At1g56450 F13N6.3	Arabidopsis thaliana (Mouse-ear cress)		Complete proteome; Cytoplasm; Hydrolase; Nucleus; Protease; Proteasome; Reference proteome; Threonine protease; Zymogen
SRR122238_TR85957_c0_g1_i2_len=618	NA	NA	NA	NA	NA	.	0,01	0,813	0	2,288	0	1,403	0,502	0,323	0	-0,914	.	.	.	.	.	.
SRR122238_TR88422_c0_g2_i1_len=1022	ERD2B_ARATH	Q:253-924,H:2-214	35.37%ID	E:7e-29	ER lumen protein-retaining receptor B	PF00810.15^ER_lumen_recept^ER lumen protein retaining receptor^73-215^E:1e-39	0,042	-0,896	0	1,863	0	2,195	0,011	1,472	0,559	0,381	ERD2B_ARATH	ER lumen protein-retaining receptor B	ERD2B ERD2.2 At3g25040	Arabidopsis thaliana (Mouse-ear cress)		Complete proteome; ER-Golgi transport; Endoplasmic reticulum; Golgi apparatus; Membrane; Protein transport; Receptor; Reference proteome; Transmembrane; Transmembrane helix; Transport
SRR1271857_TR7694_c0_g2_i1_len=1410	NA	NA	NA	NA	NA	.	0,14	0,479	0	1,482	0	2,31	0,004	1,78	0,341	0,391	.	.	.	.	.	.
SRR1271857_TR15103_c0_g1_i2_len=3614	IKU2_ARATH	Q:1212-3107,H:362-968	52.96%ID	E:0	Receptor-like protein kinase HAIKU2	PF00560.30^LRR_1^Leucine Rich Repeat^189-210^E:0.39`PF00069.22^Pkinase^Protein kinase domain^329-515^E:1.5e-32`PF07714.14^Pkinase_Tyr^Protein tyrosine kinase^330-504^E:3.7e-24`PF07714.14^Pkinase_Tyr^Protein tyrosine kinase^542-643^E:2.7e-08	0,356	1,137	0	4,446	0	4,922	0,005	3,957	0,635	0,344	IKU2_ARATH	Receptor-like protein kinase HAIKU2 (EC 2.7.11.1)	IKU2 At3g19700 MMB12.19	Arabidopsis thaliana (Mouse-ear cress)		ATP-binding; Complete proteome; Glycoprotein; Kinase; Leucine-rich repeat; Membrane; Nucleotide-binding; Phosphoprotein; Receptor; Reference proteome; Repeat; Serine/threonine-protein kinase; Signal; Transferase; Transmembrane; Transmembrane helix
SRR1271858_TR6827_c0_g3_i2_len=543	NA	NA	NA	NA	NA	PF14299.3^PP2^Phloem protein 2^1-108^E:4.2e-15	0,502	1,948	0	5,776	0	7,987	0,034	5,528	0,381	-2,256	.	.	.	.	.	.
SRR1271858_TR8518_c1_g1_i2_len=2379	HSBH1_SOYBN	Q:202-963,H:124-375	82.81%ID	E:1e-137	Homeobox protein SBH1	PF13202.3^EF-hand_5^EF hand^37-54^E:0.00085	0,56	0,679	0	3,756	0,001	1,406	0,232	0,599	1	1,227	HSBH1_SOYBN	Homeobox protein SBH1	H1	Glycine max (Soybean) (Glycine hispida)		Activator; Complete proteome; DNA-binding; Homeobox; Nucleus; Reference proteome; Transcription; Transcription regulation
SRR1271858_TR11242_c0_g1_i1_len=368	NA	NA	NA	NA	NA	.	0,124	1,013	0	2,808	0	4,224	0,132	2,625	0,132	1,474	.	.	.	.	.	.
SRR1271858_TR21137_c0_g1_i7_len=1352	GDL71_ARATH	Q:1107-604,H:186-352	56.55%ID	E:9e-63	GDSL esterase/lipase At5g03610	.	1	1,068	0,001	3,617	0,001	3,959	0,001	3,91	1	1,581	GDL71_ARATH	GDSL esterase/lipase At5g03610 (EC 3.1.1.-) (Extracellular lipase At5g03610)	At5g03610 F17C15.30	Arabidopsis thaliana (Mouse-ear cress)		Complete proteome; Glycoprotein; Hydrolase; Lipid degradation; Lipid metabolism; Reference proteome; Secreted; Signal
SRR1271858_TR46473_c0_g1_i1_len=1193	WRK71_ARATH	Q:621-878,H:123-208	87.21%ID	E:2e-46	Probable WRKY transcription factor 71	PF03106.12^WRKY^WRKY DNA -binding domain^220-277^E:1.1e-26	0,129	5,163	0	6,904	0,001	6,639	0,254	4,446	1	-1,393	WRK71_ARATH	Probable WRKY transcription factor 71 (WRKY DNA-binding protein 71)	WRKY71 At1g29860 F1N18.10	Arabidopsis thaliana (Mouse-ear cress)		Complete proteome; DNA-binding; Nucleus; Reference proteome; Transcription; Transcription regulation
SRR1271858_TR66707_c0_g1_i6_len=1464	BCB1_ARATH	Q:315-641,H:23-130	47.71%ID	E:1e-23	Blue copper protein	PF02298.14^Cu_bind_like^Plastocyanin-like domain^45-122^E:6.9e-22`PF02298.14^Cu_bind_like^Plastocyanin-like domain^184-271^E:1.5e-20	0,009	4,397	0	7,617	0	7,739	0,009	6,101	1	-1,355	BCB1_ARATH	Blue copper protein (Blue copper-binding protein) (AtBCB) (Phytocyanin 1) (Stellacyanin)	BCB AWI 32 At5g20230 F5O24.120	Arabidopsis thaliana (Mouse-ear cress)		Cell membrane; Complete proteome; Copper; Disulfide bond; Electron transport; GPI-anchor; Glycoprotein; Lipoprotein; Membrane; Metal-binding; Reference proteome; Signal; Transport
SRR1271859_TR5282_c0_g1_i1_len=1012	NA	NA	NA	NA	NA	.	0,41	0,316	0	2,37	0	1,744	0,044	1,03	0,111	-0,495	.	.	.	.	.	.
SRR1271859_TR10638_c0_g2_i1_len=1456	NA	NA	NA	NA	NA	PF13202.3^EF-hand_5^EF hand^37-54^E:0.00085	0,255	0,61	0	2,509	0	1,274	0,079	0,532	0,117	0,959	.	.	.	.	.	.
SRR1271859_TR11478_c0_g1_i5_len=1404	XTH30_ARATH	Q:409-1161,H:63-311	72.51%ID	E:2e-137	Probable xyloglucan endotransglucosylase/hydrolase protein 30	PF00722.18^Glyco_hydro_16^Glycosyl hydrolases family 16^5-158^E:1.8e-50`PF06955.9^XET_C^Xyloglucan endo-transglycosylase (XET) C-terminus^200-237^E:5e-15	0,502	3,829	0,001	6,654	0	7,256	0,009	6,104	1	-1,511	XTH30_ARATH	Probable xyloglucan endotransglucosylase/hydrolase protein 30 (At-XTH30) (XTH-30) (EC 2.4.1.207)	XTH30 XTR4 At1g32170 F3C3.5	Arabidopsis thaliana (Mouse-ear cress)		Apoplast; Cell wall; Cell wall biogenesis/degradation; Complete proteome; Disulfide bond; Glycoprotein; Glycosidase; Hydrolase; Reference proteome; Secreted; Signal; Transferase
SRR1271859_TR13491_c0_g1_i1_len=2116	PCP_MOUSE	Q:427-1746,H:46-490	38.63%ID	E:2e-98	Lysosomal Pro-X carboxypeptidase	PF05577.9^Peptidase_S28^Serine carboxypeptidase S28^91-506^E:3.8e-76	0,356	0,355	0	1,861	0	1,298	0,043	0,701	0,09	0,634	PCP_MOUSE	Lysosomal Pro-X carboxypeptidase (EC 3.4.16.2) (Proline carboxypeptidase) (Prolylcarboxypeptidase) (PRCP)	Prcp	Mus musculus (Mouse)		Alternative splicing; Carboxypeptidase; Complete proteome; Disulfide bond; Glycoprotein; Hydrolase; Lysosome; Protease; Reference proteome; Signal; Zymogen
SRR1271859_TR34739_c0_g2_i1_len=2778	BLH1_ARATH	Q:2018-447,H:190-680	54.4%ID	E:7e-129	BEL1-like homeodomain protein 1	PF07526.8^POX^Associated with HOX^225-371^E:1.4e-48`PF05920.8^Homeobox_KN^Homeobox KN domain^445-484^E:6.9e-18`PF00046.26^Homeobox^Homeobox domain^447-484^E:1.1e-06	0,011	1,399	0	1,999	0	1,486	0,002	1,198	0,48	0,289	BLH1_ARATH	BEL1-like homeodomain protein 1 (BEL1-like protein 1) (Protein EMBRYO SAC DEVELOPMENT ARREST 29)	BLH1 EDA29 At2g35940 F11F19.15	Arabidopsis thaliana (Mouse-ear cress)		Complete proteome; DNA-binding; Homeobox; Nucleus; Reference proteome; Transcription; Transcription regulation
SRR342017_TR432_c1_g1_i1_len=1655	MY1R1_SOLTU	Q:1241-642,H:24-218	44.17%ID	E:1e-34	Transcription factor MYB1R1	PF00249.28^Myb_DNA-binding^Myb-like DNA-binding domain^104-148^E:1.1e-10	0,365	0,541	0	2,047	0,001	1,296	0,005	1,088	0,298	0,685	MY1R1_SOLTU	Transcription factor MYB1R1 (Myb-related protein R1) (StMYB1R-1)		Solanum tuberosum (Potato)		Complete proteome; Cytoplasm; DNA-binding; Nucleus; Reference proteome; Stress response; Transcription; Transcription regulation
SRR342017_TR3499_c0_g1_i1_len=1589	T5AT_TAXWC	Q:1482-190,H:8-424	30.09%ID	E:3e-51	Taxadien-5-alpha-ol O-acetyltransferase	PF02458.12^Transferase^Transferase family^4-431^E:1.6e-68	0,62	0,525	0	3,211	0	3,638	0,001	3,478	0,103	-1,895	T5AT_TAXWC	Taxadien-5-alpha-ol O-acetyltransferase (EC 2.3.1.162) (Taxa-4(20),11(12)-dien-5alpha-ol-O-acetyltransferase) (Taxadienol acetyltransferase)		Taxus wallichiana var. chinensis (Chinese yew) (Taxus chinensis)	PATHWAY: Alkaloid biosynthesis; taxol biosynthesis; 10-deacetyl-2-debenzoylbaccatin III from taxa-4(20),11-dien-5alpha-ol: step 1/3.	Acyltransferase; Taxol biosynthesis; Transferase
SRR342017_TR4055_c1_g2_i1_len=716	NA	NA	NA	NA	NA	.	0,006	1,263	0	2,585	0	2,397	0,002	1,981	0,819	0,126	.	.	.	.	.	.
SRR342017_TR21089_c0_g3_i1_len=1256	PTR21_ARATH	Q:848-3,H:49-326	56.03%ID	E:6e-97	Protein NRT1/ PTR FAMILY 2.13	PF00854.18^PTR2^POT family^137-302^E:1.3e-30	0,122	0,64	0	4,822	0	2,231	0,013	1,349	0,044	1,199	PTR21_ARATH	Protein NRT1/ PTR FAMILY 2.13 (AtNPF2.13) (Nitrate transporter 1.7)	NPF2.13 NRT1.7 At1g69870 T17F3.10	Arabidopsis thaliana (Mouse-ear cress)		Cell membrane; Complete proteome; Membrane; Nitrate assimilation; Reference proteome; Symport; Transmembrane; Transmembrane helix; Transport
SRR342017_TR24788_c2_g1_i1_len=3471	BGAL3_ARATH	Q:5-1918,H:216-853	79%ID	E:0	Beta-galactosidase 3	PF01301.16^Glyco_hydro_35^Glycosyl hydrolases family 35^2-130^E:2.8e-40`PF02140.15^Gal_Lectin^Galactose binding lectin domain^554-631^E:2.8e-22	0,01	0,955	0	2,812	0	1,349	0,002	1,084	0,165	0,517	BGAL3_ARATH	Beta-galactosidase 3 (Lactase 3) (EC 3.2.1.23)	BGAL3 At4g36360 C7A10.1000 F23E13.200	Arabidopsis thaliana (Mouse-ear cress)		Alternative splicing; Apoplast; Complete proteome; Glycoprotein; Glycosidase; Hydrolase; Reference proteome; Secreted; Signal
SRR342017_TR27304_c2_g4_i5_len=1114	NPC1_PIG	Q:91-702,H:1019-1228	45.5%ID	E:2e-51	Niemann-Pick C1 protein	PF02460.15^Patched^Patched family^17-190^E:2.1e-28	0,935	0,05	0	1,549	0	2,178	0,002	1,361	0,934	-0,065	NPC1_PIG	Niemann-Pick C1 protein	NPC1	Sus scrofa (Pig)		Cholesterol metabolism; Complete proteome; Disulfide bond; Endosome; Glycoprotein; Lipid metabolism; Lysosome; Membrane; Reference proteome; Signal; Steroid metabolism; Sterol metabolism; Transmembrane; Transmembrane helix
SRR342019_TR7514_c2_g4_i2_len=746	IPRT1_CATRO	Q:681-202,H:1-157	68.75%ID	E:1e-66	Probable intracellular pathogenesis-related protein T1	PF00407.16^Bet_v_1^Pathogenesis-related protein Bet v I family^5-157^E:1e-19	0,502	2,433	0	6,636	0	7,286	0,014	3,328	1	-0,818	IPRT1_CATRO	Probable intracellular pathogenesis-related protein T1	PCKR3	Catharanthus roseus (Madagascar periwinkle) (Vinca rosea)		Direct protein sequencing; Glycoprotein; Pathogenesis-related protein; Plant defense
SRR342019_TR18688_c0_g1_i1_len=1206	PIP23_ARATH	Q:1025-183,H:1-281	82.21%ID	E:3e-168	Aquaporin PIP2-3	PF00230.17^MIP^Major intrinsic protein^89-324^E:3.1e-84	0,158	1,515	0	4,722	0	1,297	0,01	0,875	1	0,192	PIP23_ARATH	Aquaporin PIP2-3 (Plasma membrane intrinsic protein 2-3) (AtPIP2;3) (Plasma membrane intrinsic protein 2c) (PIP2c) (RD28-PIP) (TMP2C) (Water stress-induced tonoplast intrinsic protein) (WSI-TIP) [Cleaved into: Aquaporin PIP2-3, N-terminally processed]	PIP2-3 PIP2C RD28 At2g37180 T2N18.6	Arabidopsis thaliana (Mouse-ear cress)		Acetylation; Cell membrane; Complete proteome; Membrane; Phosphoprotein; Reference proteome; Repeat; Stress response; Transmembrane; Transmembrane helix; Transport
SRR342019_TR22641_c0_g1_i1_len=1019	CPC_CUCSA	Q:308-613,H:5-105	46.08%ID	E:7e-23	Cucumber peeling cupredoxin	PF02298.14^Cu_bind_like^Plastocyanin-like domain^111-198^E:9.3e-21	0,017	5,876	0	4,627	0	3,501	1	0,721	1	-0,603	CPC_CUCSA	Cucumber peeling cupredoxin (CPC) (Stellacyanin)		Cucumis sativus (Cucumber)		3D-structure; Copper; Direct protein sequencing; Disulfide bond; Electron transport; Glycoprotein; Hydroxylation; Metal-binding; Pyrrolidone carboxylic acid; Transport
SRR342019_TR29610_c0_g2_i1_len=1674	GDL87_ARATH	Q:139-1125,H:37-374	50.29%ID	E:1e-105	GDSL esterase/lipase At5g55050	PF00657.19^Lipase_GDSL^GDSL-like Lipase/Acylhydrolase^51-354^E:5.3e-24	0,044	2,223	0	3,466	0	2,776	0,007	1,978	1	-0,291	GDL87_ARATH	GDSL esterase/lipase At5g55050 (EC 3.1.1.-) (Extracellular lipase At5g55050)	At5g55050 K13P22.5	Arabidopsis thaliana (Mouse-ear cress)		Complete proteome; Glycoprotein; Hydrolase; Lipid degradation; Lipid metabolism; Reference proteome; Secreted; Signal
SRR342019_TR34700_c4_g2_i10_len=1095	GSTUA_ARATH	Q:925-104,H:2-230	36.23%ID	E:2e-42	Glutathione S-transferase U10	PF02798.17^GST_N^Glutathione S-transferase, N-terminal domain^26-96^E:6.7e-17`PF13417.3^GST_N_3^Glutathione S-transferase, N-terminal domain^28-103^E:3.6e-16`PF13409.3^GST_N_2^Glutathione S-transferase, N-terminal domain^34-97^E:4.9e-08	0,321	0,218	0	1,423	0	0,995	0,179	0,321	0,28	0,242	GSTUA_ARATH	Glutathione S-transferase U10 (AtGSTU10) (EC 2.5.1.18) (GST class-tau member 10)	GSTU10 At1g74590 F1M20.27	Arabidopsis thaliana (Mouse-ear cress)		Complete proteome; Cytoplasm; Detoxification; Reference proteome; Transferase
SRR342019_TR38454_c0_g4_i1_len=2252	INT2_ARATH	Q:200-1939,H:1-578	70.5%ID	E:0	Probable inositol transporter 2	PF00083.21^Sugar_tr^Sugar (and other) transporter^30-360^E:8.7e-95`PF07690.13^MFS_1^Major Facilitator Superfamily^35-359^E:4.8e-33`PF06609.10^TRI12^Fungal trichothecene efflux pump (TRI12)^75-218^E:1.4e-07`PF00083.21^Sugar_tr^Sugar (and other) transporter^447-552^E:3.7e-29	0,151	0,71	0	2,576	0	1,533	0,044	0,739	0,004	1,716	INT2_ARATH	Probable inositol transporter 2	INT2 At1g30220 F12P21.2	Arabidopsis thaliana (Mouse-ear cress)		Cell membrane; Complete proteome; Membrane; Reference proteome; Symport; Transmembrane; Transmembrane helix; Transport
SRR342019_TR39563_c1_g1_i1_len=1751	NA	NA	NA	NA	NA	PF01277.14^Oleosin^Oleosin^22-133^E:3.5e-30	0,254	3,24	0	4,414	0	7,283	0,009	4,74	1	-0,141	.	.	.	.	.	.
SRR342022_TR539_c0_g1_i1_len=460	NA	NA	NA	NA	NA	.	0,005	6,189	0,001	6,617	0	7,744	0,034	5,593	0,502	-2,86	.	.	.	.	.	.
SRR342022_TR7941_c0_g2_i1_len=788	NA	NA	NA	NA	NA	.	0,457	0,249	0	1,492	0,001	1,033	0,002	0,983	0,787	0,126	.	.	.	.	.	.
SRR342022_TR13122_c0_g2_i3_len=1449	GPX4_SOLLC	Q:617-1123,H:1-169	82.84%ID	E:3e-100	Probable phospholipid hydroperoxide glutathione peroxidase	PF08534.7^Redoxin^Redoxin^84-236^E:2.3e-07`PF00255.16^GSHPx^Glutathione peroxidase^85-193^E:5.4e-43`PF00578.18^AhpC-TSA^AhpC/TSA family^85-148^E:1.5e-09	0,2	0,469	0	2,899	0	1,798	0,016	1,128	1	0,567	GPX4_SOLLC	Probable phospholipid hydroperoxide glutathione peroxidase (PHGPx) (EC 1.11.1.12)	GPXle-1	Solanum lycopersicum (Tomato) (Lycopersicon esculentum)		Complete proteome; Cytoplasm; Oxidoreductase; Peroxidase; Reference proteome
SRR342022_TR15877_c0_g3_i2_len=923	NA	NA	NA	NA	NA	PF04570.11^zf-FLZ^zinc-finger of the FCS-type, C2-C2^14-60^E:1.1e-23	0,023	0,633	0	1,398	0	1,051	0,113	0,443	1	1,454	.	.	.	.	.	.
SRR342022_TR29430_c1_g2_i2_len=2382	ARGE_ARATH	Q:689-1591,H:46-346	71.52%ID	E:1e-152	Acetylornithine deacetylase	PF01546.25^Peptidase_M20^Peptidase family M20/M25/M40^2-177^E:9e-19`PF07687.11^M20_dimer^Peptidase dimerisation domain^107-221^E:8.9e-13	0,003	2,085	0	3,256	0	3,809	0,008	2,956	0,76	0,573	ARGE_ARATH	Acetylornithine deacetylase (EC 3.5.1.16) (N-acetylornithinase) (AO) (Acetylornithinase) (NAO)	At4g17830 dl4945w T6K21	Arabidopsis thaliana (Mouse-ear cress)	PATHWAY: Amino-acid biosynthesis; L-arginine biosynthesis; L-ornithine from N(2)-acetyl-L-ornithine (linear): step 1/1.	Alternative splicing; Amino-acid biosynthesis; Arginine biosynthesis; Cobalt; Complete proteome; Hydrolase; Metal-binding; Reference proteome; Zinc
SRR342023_TR304_c2_g4_i1_len=1349	NA	NA	NA	NA	NA	PF00257.16^Dehydrin^Dehydrin^3-81^E:1.8e-15`PF00257.16^Dehydrin^Dehydrin^103-127^E:3e-06	0,123	0,505	0	4,452	0	0,914	0,136	0,359	1	-0,104	.	.	.	.	.	.
SRR342023_TR1704_c0_g1_i1_len=1068	ERF99_ARATH	Q:183-413,H:91-167	79.22%ID	E:5e-36	Ethylene-responsive transcription factor 13	PF00847.17^AP2^AP2 domain^61-111^E:6.5e-13	0,006	1,19	0	2,346	0	1,677	0,394	0,455	0,187	1,197	ERF99_ARATH	Ethylene-responsive transcription factor 13 (AtERF13) (Ethylene-responsive element-binding factor 13) (EREBP-13)	ERF13 ERF-13 ERF099 At2g44840 T13E15.15	Arabidopsis thaliana (Mouse-ear cress)		Activator; Complete proteome; DNA-binding; Ethylene signaling pathway; Nucleus; Plant defense; Reference proteome; Transcription; Transcription regulation
SRR342023_TR9043_c0_g1_i2_len=737	Y2309_ARATH	Q:172-402,H:1-78	80.77%ID	E:8e-37	Uncharacterized protein At2g23090	.	0,271	0,286	0	0,937	0	0,805	0,006	0,633	1	0	Y2309_ARATH	Uncharacterized protein At2g23090	At2g23090 F21P24.15	Arabidopsis thaliana (Mouse-ear cress)		3D-structure; Complete proteome; Reference proteome
SRR342023_TR15478_c1_g1_i1_len=543	NA	NA	NA	NA	NA	.	0,02	0,543	0,001	0,813	0	1,229	0,241	0,341	0,485	-0,191	.	.	.	.	.	.
SRR342023_TR24096_c0_g2_i9_len=3664	IKU2_ARATH	Q:1286-420,H:675-968	62.37%ID	E:1e-102	Receptor-like protein kinase HAIKU2	PF11961.5^DUF3475^Domain of unknown function (DUF3475)^47-103^E:8.4e-26`PF05003.9^DUF668^Protein of unknown function (DUF668)^445-534^E:6.8e-31	0,255	0,647	0	2,565	0	2,413	0,012	1,68	1	-0,313	IKU2_ARATH	Receptor-like protein kinase HAIKU2 (EC 2.7.11.1)	IKU2 At3g19700 MMB12.19	Arabidopsis thaliana (Mouse-ear cress)		ATP-binding; Complete proteome; Glycoprotein; Kinase; Leucine-rich repeat; Membrane; Nucleotide-binding; Phosphoprotein; Receptor; Reference proteome; Repeat; Serine/threonine-protein kinase; Signal; Transferase; Transmembrane; Transmembrane helix
SRR342023_TR26267_c0_g1_i1_len=1198	CML36_ARATH	Q:695-946,H:124-209	59.3%ID	E:3e-36	Probable calcium-binding protein CML36	PF00036.29^EF-hand_1^EF hand^90-116^E:5e-08`PF13405.3^EF-hand_6^EF-hand domain^90-116^E:2.3e-07`PF13833.3^EF-hand_8^EF-hand domain pair^90-110^E:0.023`PF13499.3^EF-hand_7^EF-hand domain pair^91-148^E:5.8e-15`PF13202.3^EF-hand_5^EF hand^92-114^E:1.8e-08`PF13833.3^EF-hand_8^EF-hand domain pair^103-148^E:1.1e-09`PF13202.3^EF-hand_5^EF hand^133-150^E:0.0049	0,188	0,45	0	2,116	0	1,526	0,006	1,045	0,414	0,276	CML36_ARATH	Probable calcium-binding protein CML36 (Calmodulin-like protein 36)	CML36 At3g10190 F14P13.21	Arabidopsis thaliana (Mouse-ear cress)		Calcium; Complete proteome; Metal-binding; Reference proteome; Repeat
SRR342023_TR30617_c3_g3_i2_len=570	C72A1_CATRO	Q:2-394,H:394-524	95.42%ID	E:3e-85	Secologanin synthase	PF00067.19^p450^Cytochrome P450^2-104^E:6.9e-33	0,242	0,311	0	1,443	0,001	0,833	0,216	0,323	1	-0,751	C72A1_CATRO	Secologanin synthase (SLS) (EC 1.3.3.9) (CYPLXXII) (Cytochrome P450 72A1)	CYP72A1 CYP72 P450CR3	Catharanthus roseus (Madagascar periwinkle) (Vinca rosea)	PATHWAY: Alkaloid biosynthesis; secologanin biosynthesis.	Alkaloid metabolism; Heme; Iron; Membrane; Metal-binding; Monooxygenase; NADP; Oxidoreductase; Transmembrane; Transmembrane helix; Vacuole
SRR646572_TR24018_c0_g1_i1_len=866	ADF_VITVI	Q:119-541,H:3-143	85.82%ID	E:1e-76	Actin-depolymerizing factor	PF00241.17^Cofilin_ADF^Cofilin/tropomyosin-type actin-binding protein^8-129^E:1.6e-37	0,454	0,433	0	1,734	0,001	1,123	0,691	0,165	1	0,041	ADF_VITVI	Actin-depolymerizing factor (ADF)		Vitis vinifera (Grape)		Actin-binding
SRR646572_TR25614_c0_g1_i5_len=811	ARGE_ARATH	Q:811-347,H:286-438	64.52%ID	E:4e-66	Acetylornithine deacetylase	PF01546.25^Peptidase_M20^Peptidase family M20/M25/M40^61-152^E:6.2e-07	0,001	3,835	0	4,68	0	5,018	0,025	3,653	1	0,695	ARGE_ARATH	Acetylornithine deacetylase (EC 3.5.1.16) (N-acetylornithinase) (AO) (Acetylornithinase) (NAO)	At4g17830 dl4945w T6K21	Arabidopsis thaliana (Mouse-ear cress)	PATHWAY: Amino-acid biosynthesis; L-arginine biosynthesis; L-ornithine from N(2)-acetyl-L-ornithine (linear): step 1/1.	Alternative splicing; Amino-acid biosynthesis; Arginine biosynthesis; Cobalt; Complete proteome; Hydrolase; Metal-binding; Reference proteome; Zinc
SRR646572_TR28058_c0_g1_i2_len=2029	SG1_RAUSE	Q:1955-321,H:1-531	71.61%ID	E:0	Strictosidine-O-beta-D-glucosidase	PF00232.15^Glyco_hydro_1^Glycosyl hydrolase family 1^49-520^E:4.2e-155	0,283	0,676	0	9,672	0	7,037	0,254	4,566	1	0,508	SG1_RAUSE	Strictosidine-O-beta-D-glucosidase (EC 3.2.1.105)	SGR1	Rauvolfia serpentina (Serpentine wood) (Ophioxylon serpentinum)		3D-structure; Alkaloid metabolism; Glycosidase; Hydrolase
SRR646596_TR2459_c0_g1_i1_len=1745	HEX6_RICCO	Q:4-1476,H:13-503	76.58%ID	E:0	Hexose carrier protein HEX6	PF00083.21^Sugar_tr^Sugar (and other) transporter^15-475^E:1.5e-114`PF07690.13^MFS_1^Major Facilitator Superfamily^65-425^E:2.5e-22	0,111	1,306	0	2,858	0	4,46	0,076	2,406	1	0,134	HEX6_RICCO	Hexose carrier protein HEX6	HEX6	Ricinus communis (Castor bean)		Membrane; Sugar transport; Symport; Transmembrane; Transmembrane helix; Transport
SRR646596_TR8627_c0_g1_i2_len=1408	WTR41_ARATH	Q:1311-448,H:71-356	46.42%ID	E:6e-70	WAT1-related protein At5g40230	PF00892.17^EamA^EamA-like transporter family^94-229^E:1.2e-09	0,015	1,862	0	1,691	0	6,876	0,002	6,383	1	0	WTR41_ARATH	WAT1-related protein At5g40230	At5g40230 MSN9.130	Arabidopsis thaliana (Mouse-ear cress)		Complete proteome; Membrane; Reference proteome; Repeat; Transmembrane; Transmembrane helix
SRR646604_TR174_c3_g2_i1_len=1403	HSP7C_PETHY	Q:1204-2,H:1-401	97.01%ID	E:0	Heat shock cognate 70 kDa protein	PF00012.17^HSP70^Hsp70 protein^9-401^E:9.3e-190`PF06723.10^MreB_Mbl^MreB/Mbl protein^130-380^E:7.6e-15	0,629	1,42	0	6,325	0,001	1,111	0,002	1,031	1	0,126	HSP7C_PETHY	Heat shock cognate 70 kDa protein	HSP70	Petunia hybrida (Petunia)		ATP-binding; Nucleotide-binding; Stress response
SRR646604_TR12897_c1_g2_i4_len=781	SG1_RAUSE	Q:152-781,H:303-499	67.3%ID	E:3e-90	Strictosidine-O-beta-D-glucosidase	PF00232.15^Glyco_hydro_1^Glycosyl hydrolase family 1^7-199^E:6.5e-41	0,006	1,161	0	6,023	0	3,814	0,56	0,613	0,852	0,076	SG1_RAUSE	Strictosidine-O-beta-D-glucosidase (EC 3.2.1.105)	SGR1	Rauvolfia serpentina (Serpentine wood) (Ophioxylon serpentinum)		3D-structure; Alkaloid metabolism; Glycosidase; Hydrolase
SRR646604_TR25889_c0_g3_i1_len=1942	PTR21_ARATH	Q:1942-314,H:56-594	57.09%ID	E:0	Protein NRT1/ PTR FAMILY 2.13	PF07690.13^MFS_1^Major Facilitator Superfamily^19-480^E:2.3e-10`PF00854.18^PTR2^POT family^73-499^E:2.3e-82	0,014	0,979	0	4,344	0	7,137	0,129	5,09	0,356	-0,369	PTR21_ARATH	Protein NRT1/ PTR FAMILY 2.13 (AtNPF2.13) (Nitrate transporter 1.7)	NPF2.13 NRT1.7 At1g69870 T17F3.10	Arabidopsis thaliana (Mouse-ear cress)		Cell membrane; Complete proteome; Membrane; Nitrate assimilation; Reference proteome; Symport; Transmembrane; Transmembrane helix; Transport
SRR646604_TR34957_c0_g1_i1_len=1875	EF1A_MANES	Q:188-1492,H:1-435	98.16%ID	E:0	Elongation factor 1-alpha	PF00009.24^GTP_EFTU^Elongation factor Tu GTP binding domain^7-221^E:2.1e-52`PF03144.22^GTP_EFTU_D2^Elongation factor Tu domain 2^248-313^E:2.4e-14`PF03143.14^GTP_EFTU_D3^Elongation factor Tu C-terminal domain^322-429^E:1.3e-37	0,205	-0,351	0,001	0,896	0	1,139	0,004	0,896	0,833	0,058	EF1A_MANES	Elongation factor 1-alpha (EF-1-alpha)	EF1	Manihot esculenta (Cassava) (Jatropha manihot)		Cytoplasm; Elongation factor; GTP-binding; Nucleotide-binding; Phosphoprotein; Protein biosynthesis
SRR648705_TR21994_c0_g4_i4_len=2338	SG1_RAUSE	Q:219-1880,H:1-531	70.81%ID	E:0	Strictosidine-O-beta-D-glucosidase	PF00232.15^Glyco_hydro_1^Glycosyl hydrolase family 1^49-529^E:8.1e-153	0,048	0,673	0	4,387	0	2,924	0,748	0,279	0,053	0,522	SG1_RAUSE	Strictosidine-O-beta-D-glucosidase (EC 3.2.1.105)	SGR1	Rauvolfia serpentina (Serpentine wood) (Ophioxylon serpentinum)		3D-structure; Alkaloid metabolism; Glycosidase; Hydrolase
SRR648705_TR22083_c0_g2_i1_len=847	MY108_ARATH	Q:2-184,H:92-152	72.13%ID	E:5e-24	Transcription factor MYB108	PF00249.28^Myb_DNA-binding^Myb-like DNA-binding domain^1-26^E:9.6e-09	0,517	1,034	0	4,5	0	4,541	0,004	3,294	1	0,265	MY108_ARATH	Transcription factor MYB108 (Myb-related protein 108) (AtMYB108) (Protein BOTRYTIS-SUSCEPTIBLE 1)	MYB108 BOS1 At3g06490 F5E6.18	Arabidopsis thaliana (Mouse-ear cress)		Activator; Complete proteome; DNA-binding; Nucleus; Plant defense; Reference proteome; Repeat; Transcription; Transcription regulation; Ubl conjugation
SRR648705_TR24960_c0_g1_i1_len=1109	ADF2_PETHY	Q:378-794,H:1-139	87.77%ID	E:3e-72	Actin-depolymerizing factor 2	PF00241.17^Cofilin_ADF^Cofilin/tropomyosin-type actin-binding protein^14-135^E:2.2e-36	0,436	0,227	0	1,792	0	0,973	0,002	0,874	0,397	0,223	ADF2_PETHY	Actin-depolymerizing factor 2 (ADF-2)	ADF2	Petunia hybrida (Petunia)		Actin-binding
SRR648705_TR25311_c1_g1_i3_len=2668	LGUL_BRAOG	Q:2491-1661,H:6-281	82.31%ID	E:3e-161	Putative lactoylglutathione lyase	PF04674.9^Phi_1^Phosphate-induced protein 1 conserved region^75-337^E:2.5e-122	0,004	0,861	0	1,285	0	1,291	0,064	0,582	1	0,206	LGUL_BRAOG	Putative lactoylglutathione lyase (EC 4.4.1.5) (Aldoketomutase) (Glyoxalase I) (Glx I) (Ketone-aldehyde mutase) (Methylglyoxalase) (S-D-lactoylglutathione methylglyoxal lyase)		Brassica oleracea var. gemmifera (Brussel sprouts)	PATHWAY: Secondary metabolite metabolism; methylglyoxal degradation; (R)-lactate from methylglyoxal: step 1/2.	Lyase; Metal-binding; Repeat; Zinc
SRR648705_TR35694_c2_g7_i2_len=626	10HGO_CATRO	Q:272-3,H:2-91	73.33%ID	E:3e-40	8-hydroxygeraniol dehydrogenase	.	0,212	1,071	0	5,356	0	2,4	0,16	1,231	0,798	0,341	10HGO_CATRO	8-hydroxygeraniol dehydrogenase (Cr10HGO) (EC 1.1.1.324)	10HGO	Catharanthus roseus (Madagascar periwinkle) (Vinca rosea)		Metal-binding; NADP; Oxidoreductase; Zinc
SRR648705_TR42999_c0_g4_i1_len=913	NA	NA	NA	NA	NA	.	0,003	1,303	0	2,483	0	1,686	0,008	0,91	1	-0,029	.	.	.	.	.	.
SRR648707_TR4199_c0_g1_i3_len=5019	PDR2_ARATH	Q:1246-3840,H:135-991	80.51%ID	E:0	Probable manganese-transporting ATPase PDR2	PF00122.17^E1-E2_ATPase^E1-E2 ATPase^44-281^E:6.2e-21`PF00702.23^Hydrolase^haloacid dehalogenase-like hydrolase^291-520^E:4.1e-08`PF13246.3^Cation_ATPase^Cation transport ATPase (P-type)^347-422^E:2.5e-07	0,559	-0,326	0	3,002	0	2,358	0,214	0,794	0,173	-0,711	PDR2_ARATH	Probable manganese-transporting ATPase PDR2 (EC 3.6.3.-) (Protein MALE GAMETOGENESIS IMPAIRED ANTHERS) (Protein PHOSPHATE DEFICIENCY RESPONSE 2)	PDR2 MIA At5g23630 MQM1.11	Arabidopsis thaliana (Mouse-ear cress)		ATP-binding; Complete proteome; Endoplasmic reticulum; Hydrolase; Magnesium; Manganese; Membrane; Metal-binding; Nucleotide-binding; Reference proteome; Transmembrane; Transmembrane helix; Transport
SRR648707_TR25285_c3_g4_i4_len=4173	SG1_RAUSE	Q:4000-2339,H:1-531	69.73%ID	E:0	Strictosidine-O-beta-D-glucosidase	PF00232.15^Glyco_hydro_1^Glycosyl hydrolase family 1^49-529^E:5e-153	0,335	0,747	0	4,594	0	3,742	0,692	1,271	1	-0,011	SG1_RAUSE	Strictosidine-O-beta-D-glucosidase (EC 3.2.1.105)	SGR1	Rauvolfia serpentina (Serpentine wood) (Ophioxylon serpentinum)		3D-structure; Alkaloid metabolism; Glycosidase; Hydrolase
SRR924147_TR1813_c0_g1_i1_len=1225	WLIM1_ARATH	Q:993-475,H:3-176	72.99%ID	E:1e-87	LIM domain-containing protein WLIM1 {ECO:0000305}	PF00412.19^LIM^LIM domain^11-65^E:1.7e-10`PF00412.19^LIM^LIM domain^110-164^E:4.4e-10	1	0,069	0	2,854	0	2,03	0,094	1,124	1	0,043	WLIM1_ARATH	LIM domain-containing protein WLIM1 (Widely-expressed LIM protein 1) (AtWLIM1)	WLIM1 At1g10200 F14N23.8	Arabidopsis thaliana (Mouse-ear cress)		Acetylation; Actin-binding; Complete proteome; Cytoplasm; Cytoskeleton; LIM domain; Metal-binding; Reference proteome; Repeat; Zinc
SRR924147_TR3781_c0_g1_i1_len=2569	AL2B7_ARATH	Q:2306-795,H:30-534	81.39%ID	E:0	Aldehyde dehydrogenase family 2 member B7, mitochondrial	PF00171.19^Aldedh^Aldehyde dehydrogenase family^64-525^E:6.4e-178	0,407	0,656	0	2,806	0,001	3,014	0,517	1,227	1	-0,549	AL2B7_ARATH	Aldehyde dehydrogenase family 2 member B7, mitochondrial (ALDH2b) (EC 1.2.1.3)	ALDH2B7 ALDH3 At1g23800 F5O8.33 F5O8.35	Arabidopsis thaliana (Mouse-ear cress)		Complete proteome; Mitochondrion; NAD; Oxidoreductase; Reference proteome; Transit peptide
SRR924147_TR27800_c0_g1_i2_len=1466	NLTL5_ARATH	Q:922-665,H:41-126	76.74%ID	E:6e-37	Non-specific lipid-transfer protein-like protein At5g64080	PF00234.19^Tryp_alpha_amyl^Protease inhibitor/seed storage/LTP family^2-72^E:1.5e-07`PF14368.3^LTP_2^Probable lipid transfer^4-72^E:1.2e-12	0,11	0,612	0	1,715	0	4,448	0,01	2,231	0,316	0,666	NLTL5_ARATH	Non-specific lipid-transfer protein-like protein At5g64080	At5g64080 MHJ24.6	Arabidopsis thaliana (Mouse-ear cress)		Alternative splicing; Cell membrane; Complete proteome; Disulfide bond; GPI-anchor; Glycoprotein; Lipoprotein; Membrane; Reference proteome; Signal
SRR924148_TR1761_c0_g1_i1_len=928	NLTP2_PRUAR	Q:467-670,H:1-68	72.06%ID	E:9e-26	Non-specific lipid-transfer protein 2	.	0,008	1,621	0	2,406	0	2,425	0,019	1,674	0,92	-0,08	NLTP2_PRUAR	Non-specific lipid-transfer protein 2 (LTP 2)		Prunus armeniaca (Apricot) (Armeniaca vulgaris)		Direct protein sequencing; Lipid-binding; Transport
SRR924148_TR7540_c0_g1_i1_len=1942	ANXD1_ARATH	Q:1252-368,H:22-317	68.92%ID	E:9e-147	Annexin D1	PF00191.17^Annexin^Annexin^10-75^E:2.8e-17`PF00191.17^Annexin^Annexin^94-154^E:1.9e-14`PF00191.17^Annexin^Annexin^168-233^E:1.2e-15	0,166	0,858	0	2,355	0	2,037	0,016	1,462	0,042	0,853	ANXD1_ARATH	Annexin D1 (AnnAt1) (Annexin A1)	ANN1 ANNAT1 ANX23-ATH ATOXY5 OXY5 At1g35720 F14D7.2	Arabidopsis thaliana (Mouse-ear cress)		3D-structure; Acetylation; Annexin; Calcium; Calcium/phospholipid-binding; Complete proteome; Cytoplasm; Membrane; Reference proteome; Repeat; Stress response
SRR924148_TR16752_c0_g1_i1_len=547	NA	NA	NA	NA	NA	.	1	1,71	0	6,977	0	2,705	0,001	2,277	0,811	-0,29	.	.	.	.	.	.
SRR924148_TR17782_c0_g1_i2_len=1566	HIDM_GLYEC	Q:1336-407,H:16-327	45.77%ID	E:9e-89	2-hydroxyisoflavanone dehydratase	PF00135.25^COesterase^Carboxylesterase family^59-137^E:9.9e-06`PF07859.10^Abhydrolase_3^alpha/beta hydrolase fold^74-285^E:1.3e-35	0,213	0,278	0	2,117	0	1,56	0,013	0,609	0,791	-0,063	HIDM_GLYEC	2-hydroxyisoflavanone dehydratase (EC 3.1.1.1) (EC 4.2.1.105) (Carboxylesterase HIDM)	HIDM	Glycyrrhiza echinata (Licorice)	PATHWAY: Secondary metabolite biosynthesis; flavonoid biosynthesis.	Flavonoid biosynthesis; Hydrolase; Lyase
SRR924148_TR25300_c1_g2_i3_len=1538	UXS6_ARATH	Q:169-1203,H:1-343	86.09%ID	E:0	UDP-glucuronic acid decarboxylase 6	PF04321.14^RmlD_sub_bind^RmlD substrate binding domain^33-300^E:1.1e-07`PF01370.18^Epimerase^NAD dependent epimerase/dehydratase family^35-269^E:3.9e-45`PF02719.12^Polysacc_synt_2^Polysaccharide biosynthesis protein^35-146^E:2e-08`PF16363.2^GDP_Man_Dehyd^GDP-mannose 4,6 dehydratase^36-330^E:8.5e-54`PF01073.16^3Beta_HSD^3-beta hydroxysteroid dehydrogenase/isomerase family^36-187^E:1.9e-12`PF07993.9^NAD_binding_4^Male sterility protein^87-208^E:5.2e-07	0,948	0,041	0	1,341	0,001	1,44	0,011	1,113	0,452	0,289	UXS6_ARATH	UDP-glucuronic acid decarboxylase 6 (EC 4.1.1.35) (UDP-XYL synthase 6) (UDP-glucuronate decarboxylase 6) (UGD) (UXS-6)	UXS6 At2g28760 F8N16.5	Arabidopsis thaliana (Mouse-ear cress)	PATHWAY: Nucleotide-sugar biosynthesis; UDP-alpha-D-xylose biosynthesis; UDP-alpha-D-xylose from UDP-alpha-D-glucuronate: step 1/1.	Acetylation; Complete proteome; Cytoplasm; Decarboxylase; Lyase; NAD; Reference proteome
SRR924148_TR34256_c6_g1_i5_len=2115	SG1_RAUSE	Q:67-1491,H:1-454	69.33%ID	E:0	Strictosidine-O-beta-D-glucosidase	PF00232.15^Glyco_hydro_1^Glycosyl hydrolase family 1^49-475^E:1.1e-125	0,278	0,844	0	5,232	0	3,33	0,189	1,539	0,299	1,303	SG1_RAUSE	Strictosidine-O-beta-D-glucosidase (EC 3.2.1.105)	SGR1	Rauvolfia serpentina (Serpentine wood) (Ophioxylon serpentinum)		3D-structure; Alkaloid metabolism; Glycosidase; Hydrolase
SRR924148_TR34256_c6_g1_i8_len=632	SG1_RAUSE	Q:356-631,H:440-530	69.89%ID	E:2e-33	Strictosidine-O-beta-D-glucosidase	.	0,381	0,78	0	3,948	0	3,279	0,299	1,375	0,429	-0,467	SG1_RAUSE	Strictosidine-O-beta-D-glucosidase (EC 3.2.1.105)	SGR1	Rauvolfia serpentina (Serpentine wood) (Ophioxylon serpentinum)		3D-structure; Alkaloid metabolism; Glycosidase; Hydrolase
[illegible]	14335_SOLTU	Q:1206-442,H:1-[illegible]	93.75%ID	E:1e-171	14-3-3-like protein 16R	[illegible]	0,332	0,226	0,037	0,528	0,002	0,751	0	0,905	0	0,954	14335_SOLTU	14-3-3-like protein 16R		Solanum tuberosum (Potato)		Complete proteome; Reference proteome

SRR122238_TR38701_c2_g1_i1_len=1273	14335_SOLTU	[illegible]256	93.75%ID	E:1e-171	14-3-3-like protein 16R	PF00244.17^14-3-3^14-3-3 protein^33-258^E:7.5e-105	0,332	-0,226	0,037	0,328	0,002	0,751	0	0,905	0	0,954	14335_SOLTU	14-3-3-like protein 16R		Solanum tuberosum (Potato)		Complete proteome; Reference proteome
SRR342023_TR28516_c2_g2_i3_len=759	KIRO_ACTDE	Q:266-709,H:1-148	46.62%ID	E:2e-34	Kirola {ECO:0000303|PubMed:21309790}	PF00407.16^Bet_v_1^Pathogenesis-related protein Bet v I family^4-147^E:1.2e-32	0,686	-0,141	0,216	0,418	0,011	0,751	0	1,055	0	0,917	KIRO_ACTDE	Kirola (allergen Act d 11)		Actinidia deliciosa (Kiwi)		3D-structure; Acetylation; Allergen; Direct protein sequencing; Polymorphism
SRR122238_TR61844_c0_g3_i2_len=1160	RNLX_SOLLC	Q:953-291,H:1-220	66.67%ID	E:5e-106	Intracellular ribonuclease LX	PF00445.15^Ribonuclease_T2^Ribonuclease T2 family^52-232^E:3.2e-60	0,017	0,947	0,471	0,5	0,001	1,64	0,005	1,42	0	2,014	RNLX_SOLLC	Intracellular ribonuclease LX (RNase LX) (EC 3.1.27.1)	RNALX	Solanum lycopersicum (Tomato) (Lycopersicon esculentum)		Complete proteome; Cytoplasm; Direct protein sequencing; Disulfide bond; Endonuclease; Hydrolase; Nuclease; Reference proteome; Stress response; Zymogen
SRR342017_TR14537_c0_g1_i2_len=2077	AMYB_VIGUN	Q:177-1619,H:11-495	70.72%ID	E:0	Beta-amylase	PF01373.14^Glyco_hydro_14^Glycosyl hydrolase family 14^13-428^E:6e-119	0,087	0,534	0,275	0,448	0	1,449	0,502	0,33	0	0,917	AMYB_VIGUN	Beta-amylase (EC 3.2.1.2) (1,4-alpha-D-glucan maltohydrolase)	BMY1	Vigna unguiculata (Cowpea)		Carbohydrate metabolism; Glycosidase; Hydrolase; Polysaccharide degradation
SRR342019_TR35572_c1_g6_i1_len=1612	THCAS_CANSA	Q:140-1348,H:127-539	42.28%ID	E:7e-101	Tetrahydrocannabinolic acid synthase	PF01565.20^FAD_binding_4^FAD binding domain^15-104^E:4.9e-16`PF08031.9^BBE^Berberine and berberine like^357-415^E:5.2e-23	0,195	1,29	0,299	1,967	0,001	4,407	0,001	4,367	0	6,776	THCAS_CANSA	Tetrahydrocannabinolic acid synthase (THCAS) (EC 1.21.3.7) (Delta(1)-tetrahydrocannabinolic acid synthase) (THCA synthase)		Cannabis sativa (Hemp) (Marijuana)		3D-structure; Apoplast; Direct protein sequencing; Disulfide bond; FAD; Flavoprotein; Glycoprotein; Oxidoreductase; Secreted; Signal
SRR646572_TR33328_c0_g1_i1_len=1995	AMYB_VIGUN	Q:144-1586,H:11-495	70.52%ID	E:0	Beta-amylase	PF01373.14^Glyco_hydro_14^Glycosyl hydrolase family 14^13-428^E:3.7e-119	0,03	0,604	0,095	0,556	0	1,386	0,474	0,284	0	7,969	AMYB_VIGUN	Beta-amylase (EC 3.2.1.2) (1,4-alpha-D-glucan maltohydrolase)	BMY1	Vigna unguiculata (Cowpea)		Carbohydrate metabolism; Glycosidase; Hydrolase; Polysaccharide degradation
SRR122238_TR7054_c0_g1_i1_len=2273	P2C02_ARATH	Q:1980-1084,H:81-380	61.59%ID	E:5e-121	Probable protein phosphatase 2C 2	PF00481.18^PP2C^Protein phosphatase 2C^165-385^E:2e-56	0	3,45	0	7,19	0,001	5,561	0	6,226	1	-0,295	P2C02_ARATH	Probable protein phosphatase 2C 2 (AtPP2C02) (EC 3.1.3.16) (Protein phosphatase AP2C2)	At1g07160 F10K1.13	Arabidopsis thaliana (Mouse-ear cress)		Complete proteome; Hydrolase; Magnesium; Manganese; Metal-binding; Protein phosphatase; Reference proteome
SRR122238_TR61939_c0_g1_i15_len=3514	NES1_FRAAN	Q:3212-2130,H:14-338	46.54%ID	E:9e-91	(3S,6E)-nerolidol synthase 1	PF01535.17^PPR^PPR repeat^148-173^E:0.00038`PF17177.1^PPR_long^Pentacotripeptide-repeat region of PROPR^155-264^E:1e-07`PF13812.3^PPR_3^Pentatricopeptide repeat domain^163-221^E:2.7e-09`PF01535.17^PPR^PPR repeat^179-205^E:0.087`PF12854.4^PPR_1^PPR repeat^209-240^E:1.8e-12`PF13041.3^PPR_2^PPR repeat family^211-258^E:4.6e-16`PF01535.17^PPR^PPR repeat^214-244^E:5.8e-07`PF13812.3^PPR_3^Pentatricopeptide repeat domain^235-290^E:1.7e-07`PF12854.4^PPR_1^PPR repeat^243-275^E:8.6e-06`PF13041.3^PPR_2^PPR repeat family^246-293^E:8.4e-08`PF01535.17^PPR^PPR repeat^249-278^E:0.00068`PF13041.3^PPR_2^PPR repeat family^282-328^E:9.3e-11`PF01535.17^PPR^PPR repeat^285-314^E:0.0034`PF13812.3^PPR_3^Pentatricopeptide repeat domain^305-359^E:3.9e-11`PF13041.3^PPR_2^PPR repeat family^317-361^E:3.7e-11`PF01535.17^PPR^PPR repeat^319-349^E:1.7e-06	0	2,186	0	1,953	0,004	3,608	0	4,468	0,025	2,511	NES1_FRAAN	(3S,6E)-nerolidol synthase 1 (FaNES1) (EC 4.2.3.48)		Fragaria ananassa (Strawberry)	PATHWAY: Secondary metabolite biosynthesis; terpenoid biosynthesis.	Cytoplasm; Lyase; Magnesium; Manganese; Metal-binding
SRR342022_TR23185_c0_g3_i11_len=1836	MYRS_QUEIL	Q:1564-425,H:38-414	57.63%ID	E:4e-141	Myrcene synthase, chloroplastic	PF01397.18^Terpene_synth^Terpene synthase, N-terminal domain^67-243^E:3e-44`PF03936.13^Terpene_synth_C^Terpene synthase family, metal binding domain^275-418^E:1.3e-56	0	4,216	0	6,568	1	-3,468	0	4,595	1	0	MYRS_QUEIL	Myrcene synthase, chloroplastic (EC 4.2.3.15)		Quercus ilex (Holly oak)		Chloroplast; Lyase; Magnesium; Manganese; Metal-binding; Plastid; Transit peptide
SRR648707_TR4925_c0_g1_i2_len=2058	P2C30_ARATH	Q:949-1638,H:156-389	67.09%ID	E:1e-99	Probable protein phosphatase 2C 30	PF00481.18^PP2C^Protein phosphatase 2C^6-220^E:9.5e-56	0	6,029	0	8,845	0,002	5,327	0	5,822	0	-2,226	P2C30_ARATH	Probable protein phosphatase 2C 30 (AtPP2C30) (EC 3.1.3.16) (AthPP2C5)	PP2C5 At2g40180 T7M7.17	Arabidopsis thaliana (Mouse-ear cress)		Complete proteome; Hydrolase; Magnesium; Manganese; Metal-binding; Protein phosphatase; Reference proteome
SRR1144634_TR11657_c0_g2_i1_len=1291	PIP22_ORYSJ	Q:81-827,H:1-252	80.56%ID	E:1e-141	Probable aquaporin PIP2-2	PF00230.17^MIP^Major intrinsic protein^31-250^E:2.8e-73	0	1,932	0	4,172	0	1,188	0,023	0,48	0,007	1,374	PIP22_ORYSJ	Probable aquaporin PIP2-2 (OsPIP2;2) (Plasma membrane intrinsic protein 2-2)	PIP2-2 Os02g0629200 LOC_Os02g41860 B1469H02.22-1	Oryza sativa subsp. japonica (Rice)		Cell membrane; Complete proteome; Membrane; Reference proteome; Repeat; Transmembrane; Transmembrane helix; Transport
SRR1144634_TR14318_c0_g1_i1_len=972	SERA2_ARATH	Q:61-732,H:401-624	81.25%ID	E:1e-120	D-3-phosphoglycerate dehydrogenase 2, chloroplastic	PF01842.22^ACT^ACT domain^101-161^E:5.2e-11	0	1,585	0	2,934	0	1,887	0,009	0,783	0,23	0,291	SERA2_ARATH	D-3-phosphoglycerate dehydrogenase 2, chloroplastic (PGDH) (EC 1.1.1.95)	PGDH2 3-PGDH PGDH At1g17745 F11A6.8	Arabidopsis thaliana (Mouse-ear cress)	PATHWAY: Amino-acid biosynthesis; L-serine biosynthesis; L-serine from 3-phospho-D-glycerate: step 1/3.	Alternative splicing; Amino-acid biosynthesis; Chloroplast; Complete proteome; NAD; Oxidoreductase; Phosphoprotein; Plastid; Reference proteome; Serine biosynthesis; Transit peptide
SRR1144634_TR22915_c0_g1_i1_len=571	CVIF1_ARATH	Q:163-501,H:26-138	41.59%ID	E:2e-22	Cell wall / vacuolar inhibitor of fructosidase 1	PF04043.12^PMEI^Plant invertase/pectin methylesterase inhibitor^54-187^E:1e-13	0	1,217	0	1,991	0	1,233	0,932	-0,046	0	-7,8	CVIF1_ARATH	Cell wall / vacuolar inhibitor of fructosidase 1 (AtC/VIF1)	C/VIF1 CIF1 VIF1 At1g47960 T2J15.13	Arabidopsis thaliana (Mouse-ear cress)		Alternative splicing; Complete proteome; Disulfide bond; Glycoprotein; Reference proteome; Signal; Vacuole
SRR1144634_TR34616_c0_g1_i1_len=653	ML423_ARATH	Q:125-583,H:7-153	36.6%ID	E:6e-29	MLP-like protein 423	PF00407.16^Bet_v_1^Pathogenesis-related protein Bet v I family^3-153^E:4.4e-28	0	1,425	0	2,785	0,001	1,46	0,281	0,548	1	0	ML423_ARATH	MLP-like protein 423	MLP423 At1g24020 T23E23.17 T23E23_22	Arabidopsis thaliana (Mouse-ear cress)		Complete proteome; Reference proteome
SRR122238_TR7709_c0_g1_i3_len=827	NA	NA	NA	NA	NA	PF14299.3^PP2^Phloem protein 2^27-186^E:7.8e-28	0	4,79	0	6,692	0	5,058	0,381	2,186	0,381	0,823	.	.	.	.	.	.
SRR122238_TR20261_c1_g1_i1_len=928	NA	NA	NA	NA	NA	PF00847.17^AP2^AP2 domain^75-124^E:2.5e-14	0	5,162	0	5,968	0	6,112	0,129	3,83	1	-0,64	.	.	.	.	.	.
SRR122238_TR42251_c0_g1_i1_len=827	DR206_PEA	Q:741-175,H:1-184	57.67%ID	E:2e-73	Disease resistance response protein 206	PF03018.11^Dirigent^Dirigent-like protein^36-181^E:1.4e-38	0,001	1,329	0	2,071	0	1,72	0,009	1,344	0,254	-4,433	DR206_PEA	Disease resistance response protein 206 (Dirigent protein PI206)	PI206	Pisum sativum (Garden pea)		3D-structure; Apoplast; Disulfide bond; Glycoprotein; Pathogenesis-related protein; Plant defense; Secreted; Signal
SRR122238_TR44565_c0_g2_i1_len=1611	C81F1_ARATH	Q:65-1396,H:39-499	44.73%ID	E:5e-125	Cytochrome P450 81F1 {ECO:0000305}	PF00067.19^p450^Cytochrome P450^12-449^E:6.7e-90	0	1,507	0	3,237	0	1,654	0,02	0,866	1	0,47	C81F1_ARATH	Cytochrome P450 81F1 (EC 1.14.-.-)	CYP81F1 CYP91A2 At4g37430 F6G17.80	Arabidopsis thaliana (Mouse-ear cress)		Complete proteome; Heme; Iron; Membrane; Metal-binding; Monooxygenase; Oxidoreductase; Reference proteome; Transmembrane; Transmembrane helix
SRR122238_TR54493_c1_g2_i1_len=1095	NA	NA	NA	NA	NA	.	0	1,124	0	2,135	0	1,124	0,001	0,878	0,886	0,186	.	.	.	.	.	.
SRR1271858_TR25211_c0_g2_i1_len=675	NA	NA	NA	NA	NA	.	0	1,292	0	6,744	0	8,539	0,129	4,207	0	-2,021	.	.	.	.	.	.
SRR1271858_TR59441_c0_g1_i2_len=1968	NA	NA	NA	NA	NA	PF11891.5^DUF3411^Domain of unknown function (DUF3411)^203-381^E:1.3e-64	0	1,519	0	2,425	0,001	2,103	0,006	1,848	0,739	0,28	.	.	.	.	.	.
SRR1271859_TR27501_c0_g1_i1_len=893	CVIF1_ARATH	Q:257-691,H:26-162	37.24%ID	E:2e-22	Cell wall / vacuolar inhibitor of fructosidase 1	PF04043.12^PMEI^Plant invertase/pectin methylesterase inhibitor^28-172^E:2e-14	0	1,257	0	1,298	0	2,143	0,58	0,243	0,073	-2,39	CVIF1_ARATH	Cell wall / vacuolar inhibitor of fructosidase 1 (AtC/VIF1)	C/VIF1 CIF1 VIF1 At1g47960 T2J15.13	Arabidopsis thaliana (Mouse-ear cress)		Alternative splicing; Complete proteome; Disulfide bond; Glycoprotein; Reference proteome; Signal; Vacuole
SRR1271859_TR33815_c0_g2_i4_len=2647	LDOX_MALDO	Q:1392-2384,H:10-341	38.84%ID	E:3e-75	Leucoanthocyanidin dioxygenase	PF14226.3^DIOX_N^non-haem dioxygenase in morphine synthesis N-terminal^56-167^E:1e-32`PF03171.17^2OG-FeII_Oxy^2OG-Fe(II) oxygenase superfamily^216-314^E:7e-27	0	4,739	0	7,48	0	3,467	0,014	2,776	1	0,759	LDOX_MALDO	Leucoanthocyanidin dioxygenase (LDOX) (Leucocyanidin oxygenase) (EC 1.14.11.19) (Anthocyanidin synthase) (Leucoanthocyanidin hydroxylase)	ANS	Malus domestica (Apple) (Pyrus malus)	PATHWAY: Pigment biosynthesis; anthocyanin biosynthesis.	Dioxygenase; Flavonoid biosynthesis; Iron; Metal-binding; Oxidoreductase; Vitamin C
SRR1271859_TR38596_c0_g1_i7_len=1181	DCE_SOLLC	Q:1062-46,H:165-502	76.99%ID	E:0	Glutamate decarboxylase	PF00282.16^Pyridoxal_deC^Pyridoxal-dependent decarboxylase conserved domain^10-228^E:1.2e-66	0	1,444	0	1,848	0	2,084	0,002	1,274	0,43	0,637	DCE_SOLLC	Glutamate decarboxylase (GAD) (EC 4.1.1.15) (ERT D1)		Solanum lycopersicum (Tomato) (Lycopersicon esculentum)		Calmodulin-binding; Complete proteome; Decarboxylase; Lyase; Pyridoxal phosphate; Reference proteome
SRR342019_TR2813_c0_g5_i1_len=1177	NA	NA	NA	NA	NA	.	0	2,226	0	3,523	0	2,776	0,003	2,063	1	-0,108	.	.	.	.	.	.
SRR342019_TR34225_c0_g1_i1_len=617	PRS1_SOLTU	Q:85-528,H:1-155	35.9%ID	E:5e-21	Pathogenesis-related protein STH-21	PF00407.16^Bet_v_1^Pathogenesis-related protein Bet v I family^4-143^E:1.3e-12	0	1,02	0	1,571	0,001	0,715	0,002	0,653	1	-0,255	PRS1_SOLTU	Pathogenesis-related protein STH-21	STH-21	Solanum tuberosum (Potato)		Complete proteome; Pathogenesis-related protein; Plant defense; Reference proteome
SRR342019_TR36227_c6_g7_i2_len=893	C7BL3_CICIN	Q:838-245,H:290-486	42.93%ID	E:1e-57	Costunolide synthase	PF00067.19^p450^Cytochrome P450^3-185^E:2.1e-49	0	1,586	0	1,968	0,001	0,852	0,001	-0,994	0,16	1,341	C7BL3_CICIN	Costunolide synthase (CiCOS) (EC 1.14.13.120) (Cytochrome P450 71BL3)	CYP71BL3 COS	Cichorium intybus (Chicory)		Heme; Iron; Membrane; Metal-binding; Monooxygenase; NADP; Oxidoreductase; Transmembrane; Transmembrane helix
SRR342023_TR3935_c3_g7_i1_len=2430	SERA2_ARATH	Q:1973-336,H:79-624	84.62%ID	E:0	D-3-phosphoglycerate dehydrogenase 2, chloroplastic	PF00389.27^2-Hacid_dh^D-isomer specific 2-hydroxyacid dehydrogenase, catalytic domain^98-407^E:3.7e-35`PF02826.16^2-Hacid_dh_C^D-isomer specific 2-hydroxyacid dehydrogenase, NAD binding domain^200-375^E:5.3e-63`PF00670.18^AdoHcyase_NAD^S-adenosyl-L-homocysteine hydrolase, NAD binding domain^230-325^E:8.4e-05`PF07991.9^IlvN^Acetohydroxy acid isomeroreductase, NADPH-binding domain^232-391^E:8e-06`PF01842.22^ACT^ACT domain^567-627^E:4.6e-10	0	1,635	0	4,331	0	1,436	0,004	0,78	0,003	1,02	SERA2_ARATH	D-3-phosphoglycerate dehydrogenase 2, chloroplastic (PGDH) (EC 1.1.1.95)	PGDH2 3-PGDH PGDH At1g17745 F11A6.8	Arabidopsis thaliana (Mouse-ear cress)	PATHWAY: Amino-acid biosynthesis; L-serine biosynthesis; L-serine from 3-phospho-D-glycerate: step 1/3.	Alternative splicing; Amino-acid biosynthesis; Chloroplast; Complete proteome; NAD; Oxidoreductase; Phosphoprotein; Plastid; Reference proteome; Serine biosynthesis; Transit peptide
SRR342023_TR15323_c0_g2_i2_len=2096	SG1_RAUSE	Q:2001-340,H:1-531	70.45%ID	E:0	Strictosidine-O-beta-D-glucosidase	PF00232.15^Glyco_hydro_1^Glycosyl hydrolase family 1^49-529^E:2.6e-152	0	0,922	0	4,449	0	3,101	0,875	0,068	0,468	0,307	SG1_RAUSE	Strictosidine-O-beta-D-glucosidase (EC 3.2.1.105)	SGR1	Rauvolfia serpentina (Serpentine wood) (Ophioxylon serpentinum)		3D-structure; Alkaloid metabolism; Glycosidase; Hydrolase
SRR342023_TR17313_c0_g2_i1_len=529	NA	NA	NA	NA	NA	.	0	2,974	0,001	1,015	0	1,788	0,314	0,428	0,811	-0,314	.	.	.	.	.	.
SRR646596_TR9280_c1_g1_i2_len=521	NA	NA	NA	NA	NA	.	0	1,171	0	7,188	0	3,838	1	0,605	0	-1,602	.	.	.	.	.	.
SRR646604_TR1357_c0_g1_i1_len=1551	PIP25_ARATH	Q:1277-429,H:1-281	80.57%ID	E:1e-161	Probable aquaporin PIP2-5	PF00230.17^MIP^Major intrinsic protein^31-267^E:7.9e-84	0	1,842	0	3,817	0	1,338	0,006	0,604	0,028	0,611	PIP25_ARATH	Probable aquaporin PIP2-5 (Plasma membrane intrinsic protein 2-5) (AtPIP2;5) (Plasma membrane intrinsic protein 2d) (PIP2d) [Cleaved into: Probable aquaporin PIP2-5, N-terminally processed]	PIP2-5 PIP2D At3g54820 F28P10.200	Arabidopsis thaliana (Mouse-ear cress)		Acetylation; Cell membrane; Complete proteome; Membrane; Methylation; Phosphoprotein; Reference proteome; Repeat; Transmembrane; Transmembrane helix; Transport
SRR648705_TR12352_c0_g1_i6_len=1079	GSTUA_ARATH	Q:264-953,H:1-230	43.72%ID	E:5e-51	Glutathione S-transferase U10	PF02798.17^GST_N^Glutathione S-transferase, N-terminal domain^25-96^E:1.8e-16`PF13417.3^GST_N_3^Glutathione S-transferase, N-terminal domain^28-103^E:1.4e-15`PF13409.3^GST_N_2^Glutathione S-transferase, N-terminal domain^34-97^E:1.8e-07	0	1,492	0	3,21	0	3,28	1	0,129	0,967	-0,028	GSTUA_ARATH	Glutathione S-transferase U10 (AtGSTU10) (EC 2.5.1.18) (GST class-tau member 10)	GSTU10 At1g74590 F1M20.27	Arabidopsis thaliana (Mouse-ear cress)		Complete proteome; Cytoplasm; Detoxification; Reference proteome; Transferase
SRR648705_TR37787_c0_g1_i3_len=844	NA	NA	NA	NA	NA	.	0,001	5,133	0	8,865	0	4,866	0,629	2,101	1	-0,425	.	.	.	.	.	.
SRR648707_TR1151_c0_g3_i1_len=1633	MT878_ARATH	Q:253-1323,H:1-361	44.26%ID	E:1e-83	Probable S-adenosylmethionine-dependent methyltransferase At5g38780	PF03492.12^Methyltransf_7^SAM dependent carboxyl methyltransferase^69-368^E:4e-102	0	1,65	0	4,011	0	1,589	0,071	0,403	0,005	0,647	MT878_ARATH	Probable S-adenosylmethionine-dependent methyltransferase At5g38780 (EC 2.1.1.-)	At5g38780 MKD10.80	Arabidopsis thaliana (Mouse-ear cress)		Complete proteome; Methyltransferase; Reference proteome; S-adenosyl-L-methionine; Transferase
SRR648707_TR26086_c0_g1_i11_len=1848	TRPD_ARATH	Q:534-1208,H:110-334	84%ID	E:1e-141	Anthranilate phosphoribosyltransferase, chloroplastic	PF02885.14^Glycos_trans_3N^Glycosyl transferase family, helical bundle domain^66-124^E:2.7e-12`PF00591.18^Glycos_transf_3^Glycosyl transferase family, a/b domain^133-290^E:2.1e-71	0	1,476	0	2,824	0,001	1,135	0,004	0,976	0,081	0,622	TRPD_ARATH	Anthranilate phosphoribosyltransferase, chloroplastic (EC 2.4.2.18)	PAT1 At5g17990 MCM23.6	Arabidopsis thaliana (Mouse-ear cress)	PATHWAY: Amino-acid biosynthesis; L-tryptophan biosynthesis; L-tryptophan from chorismate: step 2/5.	Amino-acid biosynthesis; Aromatic amino acid biosynthesis; Chloroplast; Complete proteome; Glycosyltransferase; Plastid; Reference proteome; Transferase; Transit peptide; Tryptophan biosynthesis
SRR648707_TR35839_c0_g1_i2_len=1717	ILL6_ARATH	Q:1344-142,H:67-462	68.58%ID	E:0	IAA-amino acid hydrolase ILR1-like 6	PF01546.25^Peptidase_M20^Peptidase family M20/M25/M40^167-482^E:9.5e-36`PF07687.11^M20_dimer^Peptidase dimerisation domain^280-373^E:1.6e-06	0,001	3,714	0	6,88	0,001	6,555	0,034	5,502	1	1,043	ILL6_ARATH	IAA-amino acid hydrolase ILR1-like 6 (EC 3.5.1.-) (Protein gr1)	ILL6 GR1 At1g44350 T18F15.9	Arabidopsis thaliana (Mouse-ear cress)		Complete proteome; Hydrolase; Reference proteome; Signal
SRR924147_TR27644_c0_g1_i2_len=1043	NA	NA	NA	NA	NA	.	0	1,003	0	1,807	0	0,888	0,002	0,762	0,591	0,141	.	.	.	.	.	.
SRR1144634_TR14237_c0_g1_i4_len=622	NA	NA	NA	NA	NA	.	0,111	0,387	0	1,044	0,012	0,542	0	1,09	0	0,812	.	.	.	.	.	.
SRR342019_TR38071_c4_g2_i4_len=1544	ARGE_ARATH	Q:260-1432,H:1-394	67.09%ID	E:0	Acetylornithine deacetylase	PF01546.25^Peptidase_M20^Peptidase family M20/M25/M40^95-379^E:3.1e-23`PF07687.11^M20_dimer^Peptidase dimerisation domain^203-317^E:5.5e-13	0,052	1,666	0	3,036	0,002	4,402	0	4,843	0	1,741	ARGE_ARATH	Acetylornithine deacetylase (EC 3.5.1.16) (N-acetylornithinase) (AO) (Acetylornithinase) (NAO)	At4g17830 dl4945w T6K21	Arabidopsis thaliana (Mouse-ear cress)	PATHWAY: Amino-acid biosynthesis; L-arginine biosynthesis; L-ornithine from N(2)-acetyl-L-ornithine (linear): step 1/1.	Alternative splicing; Amino-acid biosynthesis; Arginine biosynthesis; Cobalt; Complete proteome; Hydrolase; Metal-binding; Reference proteome; Zinc
SRR646596_TR9632_c0_g1_i1_len=1847	TAR4_ARATH	Q:1665-346,H:6-449	52.8%ID	E:4e-159	Tryptophan aminotransferase-related protein 4	PF04863.10^EGF_alliinase^Alliinase EGF-like domain^40-94^E:2.4e-27`PF04864.10^Alliinase_C^Allinase^96-451^E:9.5e-143`PF00155.18^Aminotran_1_2^Aminotransferase class I and II^137-432^E:1.3e-06	0	2,345	0,004	2,429	0	3,048	0,006	2,377	0	3,116	TAR4_ARATH	Tryptophan aminotransferase-related protein 4 (EC 2.6.1.-)	TAR4 At1g34060 F12G12.12 F12G12.150	Arabidopsis thaliana (Mouse-ear cress)		Aminotransferase; Complete proteome; Membrane; Pyridoxal phosphate; Reference proteome; Transferase; Transmembrane; Transmembrane helix
SRR122238_TR29423_c1_g1_i7_len=1817	RFS2_ARATH	Q:1450-206,H:359-773	78.37%ID	E:0	Probable galactinol--sucrose galactosyltransferase 2	PF05691.9^Raffinose_syn^Raffinose synthase or seed imbibition protein Sip1^2-123^E:1.5e-51`PF05691.9^Raffinose_syn^Raffinose synthase or seed imbibition protein Sip1^123-521^E:7.7e-183	0,025	1,249	0	1,711	0	1,237	0,391	0,23	0	1,783	RFS2_ARATH	Probable galactinol--sucrose galactosyltransferase 2 (EC 2.4.1.82) (Protein SEED IMBIBITION 2) (Raffinose synthase 2)	RFS2 RS2 SIP2 At3g57520 T8H10.120	Arabidopsis thaliana (Mouse-ear cress)		Alternative splicing; Carbohydrate metabolism; Complete proteome; Glycosyltransferase; Reference proteome; Transferase
SRR122238_TR51320_c0_g2_i2_len=1710	BCAT5_ARATH	Q:130-1314,H:13-414	70.44%ID	E:0	Branched-chain-amino-acid aminotransferase 5, chloroplastic	PF01063.16^Aminotran_4^Amino-transferase class IV^156-394^E:1.4e-41	0,009	-5,939	0	7,09	0	6,711	0,129	4,989	0	7,39	BCAT5_ARATH	Branched-chain-amino-acid aminotransferase 5, chloroplastic (Atbcat-5) (EC 2.6.1.42)	BCAT5 At5g65780 F6H11.110 MPA24.13	Arabidopsis thaliana (Mouse-ear cress)	PATHWAY: Amino-acid biosynthesis; L-isoleucine biosynthesis; L-isoleucine from 2-oxobutanoate: step 4/4.; PATHWAY: Amino-acid biosynthesis; L-leucine biosynthesis; L-leucine from 3-methyl-2-oxobutanoate: step 4/4.; PATHWAY: Amino-acid biosynthesis; L-valine biosynthesis; L-valine from pyruvate: step 4/4.	Alternative splicing; Amino-acid biosynthesis; Aminotransferase; Branched-chain amino acid biosynthesis; Chloroplast; Complete proteome; Plastid; Pyridoxal phosphate; Reference proteome; Transferase; Transit peptide
SRR1271857_TR3142_c0_g1_i3_len=2155	PIP1_SOLLC	Q:1865-1236,H:1-210	87.62%ID	E:2e-127	Probable aquaporin PIP-type pTOM75	PF00230.17^MIP^Major intrinsic protein^45-210^E:5.8e-57	0,484	0,426	0	3,43	0	1,631	0,005	1,145	0	4,571	PIP1_SOLLC	Probable aquaporin PIP-type pTOM75 (Ripening-associated membrane protein) (RAMP)		Solanum lycopersicum (Tomato) (Lycopersicon esculentum)		Cell membrane; Complete proteome; Membrane; Reference proteome; Repeat; Stress response; Transmembrane; Transmembrane helix; Transport
SRR1271858_TR7347_c0_g2_i10_len=1896	DCE_SOLLC	Q:346-1851,H:2-502	79.28%ID	E:0	Glutamate decarboxylase	PF00282.16^Pyridoxal_deC^Pyridoxal-dependent decarboxylase conserved domain^33-381^E:3.8e-106	0,006	0,983	0	2,867	0	1,612	0,038	0,981	0	1,629	DCE_SOLLC	Glutamate decarboxylase (GAD) (EC 4.1.1.15) (ERT D1)		Solanum lycopersicum (Tomato) (Lycopersicon esculentum)		Calmodulin-binding; Complete proteome; Decarboxylase; Lyase; Pyridoxal phosphate; Reference proteome
SRR1271858_TR43612_c0_g1_i4_len=1111	NA	NA	NA	NA	NA	PF14364.3^DUF4408^Domain of unknown function (DUF4408)^80-104^E:4.4e-08	0,01	1,838	0	3,116	0	2,419	0,001	2,194	0	1,628	.	.	.	.	.	.
SRR342019_TR17274_c0_g1_i1_len=514	14337_ARATH	Q:1-459,H:7-159	88.89%ID	E:4e-95	14-3-3-like protein GF14 nu	PF00244.17^14-3-3^14-3-3 protein^5-154^E:9.2e-60	0,516	0,244	0	1,754	0,001	1,378	0,077	0,75	0	1,334	14337_ARATH	14-3-3-like protein GF14 nu (General regulatory factor 7)	GRF7 At3g02520 F16B3.15	Arabidopsis thaliana (Mouse-ear cress)		Complete proteome; Reference proteome
SRR342022_TR33543_c0_g1_i3_len=1830	GCAL1_ARATH	Q:800-231,H:60-249	88.95%ID	E:2e-116	Gamma carbonic anhydrase-like 1, mitochondrial	PF00257.16^Dehydrin^Dehydrin^47-174^E:2e-23`PF00257.16^Dehydrin^Dehydrin^196-220^E:9.4e-06	0,181	0,409	0	2,438	0	1,145	0,102	0,424	0	2,332	GCAL1_ARATH	Gamma carbonic anhydrase-like 1, mitochondrial (AtCAL1) (GAMMA CAL1)	GAMMACAL1 At5g63510 MLE2.14	Arabidopsis thaliana (Mouse-ear cress)		Alternative splicing; Complete proteome; Direct protein sequencing; Membrane; Metal-binding; Mitochondrion; Reference proteome; Transit peptide; Zinc
SRR648705_TR12352_c0_g1_i3_len=1005	GSTUA_ARATH	Q:196-879,H:2-230	43.04%ID	E:3e-51	Glutathione S-transferase U10	PF02798.17^GST_N^Glutathione S-transferase, N-terminal domain^26-96^E:2.6e-16`PF13417.3^GST_N_3^Glutathione S-transferase, N-terminal domain^28-103^E:1.4e-15`PF13409.3^GST_N_2^Glutathione S-transferase, N-terminal domain^34-97^E:1.8e-07	0,185	0,337	0	1,921	0	1,873	0,002	1,197	0	1,089	GSTUA_ARATH	Glutathione S-transferase U10 (AtGSTU10) (EC 2.5.1.18) (GST class-tau member 10)	GSTU10 At1g74590 F1M20.27	Arabidopsis thaliana (Mouse-ear cress)		Complete proteome; Cytoplasm; Detoxification; Reference proteome; Transferase
SRR648707_TR17303_c0_g2_i4_len=1601	NA	NA	NA	NA	NA	PF13456.3^RVT_3^Reverse transcriptase-like^145-266^E:3.8e-26`PF00075.21^RNase_H^RNase H^145-239^E:1.1e-08	0,183	-0,653	0	6,667	0	6,919	1	0	0	7,023	.	.	.	.	.	.
SRR648707_TR33835_c0_g6_i3_len=788	GSTUA_ARATH	Q:216-650,H:1-146	52.05%ID	E:2e-45	Glutathione S-transferase U10	PF02798.17^GST_N^Glutathione S-transferase, N-terminal domain^25-96^E:1.3e-16`PF13417.3^GST_N_3^Glutathione S-transferase, N-terminal domain^28-103^E:1e-15`PF13409.3^GST_N_2^Glutathione S-transferase, N-terminal domain^34-97^E:1.3e-07	0,172	0,318	0	3,664	0	1,807	0,512	0,229	0	0,893	GSTUA_ARATH	Glutathione S-transferase U10 (AtGSTU10) (EC 2.5.1.18) (GST class-tau member 10)	GSTU10 At1g74590 F1M20.27	Arabidopsis thaliana (Mouse-ear cress)		Complete proteome; Cytoplasm; Detoxification; Reference proteome; Transferase
SRR122238_TR61811_c0_g2_i1_len=517	PIP27_MAIZE	Q:325-2,H:1-109	75.23%ID	E:1e-51	Aquaporin PIP2-7	PF00230.17^MIP^Major intrinsic protein^95-172^E:3.8e-19	0	1,785	0	4,515	0	0,897	0,014	0,552	0	1,403	PIP27_MAIZE	Aquaporin PIP2-7 (Plasma membrane intrinsic protein 2-7) (ZmPIP2-7) (ZmPIP2;7)	PIP2-7	Zea mays (Maize)		Cell membrane; Complete proteome; Membrane; Reference proteome; Repeat; Transmembrane; Transmembrane helix; Transport
SRR342017_TR18257_c0_g1_i1_len=761	PIP21_ORYSJ	Q:12-635,H:80-287	86.54%ID	E:5e-130	Probable aquaporin PIP2-1	PF00230.17^MIP^Major intrinsic protein^7-195^E:1.2e-72	0	2,126	0	4,388	0	1,473	0,337	0,253	0	0,968	PIP21_ORYSJ	Probable aquaporin PIP2-1 (OsPIP2;1) (Plasma membrane intrinsic protein 2-1) (Plasma membrane intrinsic protein 2a) (PIP2a)	PIP2-1 PIP2a Os07g0448800 LOC_Os07g26690 OJ1047_A06.117 OsJ_023145	Oryza sativa subsp. japonica (Rice)		Cell membrane; Complete proteome; Membrane; Reference proteome; Repeat; Transmembrane; Transmembrane helix; Transport
SRR648707_TR34961_c0_g1_i1_len=1876	C7BL3_CICIN	Q:291-1673,H:33-486	38.53%ID	E:2e-116	Costunolide synthase	PF00067.19^p450^Cytochrome P450^34-480^E:5.1e-86	0	1,218	0	3,356	0,001	0,79	0,057	-0,476	0	1,255	C7BL3_CICIN	Costunolide synthase (CiCOS) (EC 1.14.13.120) (Cytochrome P450 71BL3)	CYP71BL3 COS	Cichorium intybus (Chicory)		Heme; Iron; Membrane; Metal-binding; Monooxygenase; NADP; Oxidoreductase; Transmembrane; Transmembrane helix
SRR924148_TR37769_c0_g1_i3_len=2445	AGT23_ARATH	Q:228-1616,H:20-481	75.59%ID	E:0	Alanine--glyoxylate aminotransferase 2 homolog 3, mitochondrial	PF00202.18^Aminotran_3^Aminotransferase class-III^89-483^E:5.3e-98	0	2,683	0	4,769	0	3,523	0,002	2,8	0	3,307	AGT23_ARATH	Alanine--glyoxylate aminotransferase 2 homolog 3, mitochondrial (EC 2.6.1.44) (Beta-alanine-pyruvate aminotransferase 3)	At3g08860 T16O11.21	Arabidopsis thaliana (Mouse-ear cress)		Aminotransferase; Complete proteome; Mitochondrion; Photorespiration; Pyridoxal phosphate; Reference proteome; Transferase; Transit peptide
SRR122238_TR57645_c0_g1_i2_len=874	C7BL3_CICIN	Q:75-668,H:290-486	42.93%ID	E:1e-57	Costunolide synthase	PF00067.19^p450^Cytochrome P450^2-166^E:3e-46	0	8,473	0,075	0,469	0	10,114	0	7,254	0,009	-0,589	C7BL3_CICIN	Costunolide synthase (CiCOS) (EC 1.14.13.120) (Cytochrome P450 71BL3)	CYP71BL3 COS	Cichorium intybus (Chicory)		Heme; Iron; Membrane; Metal-binding; Monooxygenase; NADP; Oxidoreductase; Transmembrane; Transmembrane helix
SRR342017_TR30366_c0_g3_i1_len=657	NA	NA	NA	NA	NA	.	0	1,195	0,01	0,62	0	2,599	0	1,351	0,142	0,397	.	.	.	.	.	.
SRR342022_TR17097_c2_g5_i1_len=2501	TPSGD_VITVI	Q:2424-784,H:19-557	53.75%ID	E:0	(-)-germacrene D synthase	PF01397.18^Terpene_synth^Terpene synthase, N-terminal domain^38-218^E:2.9e-51`PF03936.13^Terpene_synth_C^Terpene synthase family, metal binding domain^249-515^E:3.8e-100	0	5,384	1	2,398	0	9,941	0	9,442	1	0,924	TPSGD_VITVI	(-)-germacrene D synthase (EC 4.2.3.22) (EC 4.2.3.75)	VIT_19s0014g04930 VIT_00014569001 VITISV_013313 VITISV_030783 Vv19s0014g04930	Vitis vinifera (Grape)	PATHWAY: Secondary metabolite biosynthesis; terpenoid biosynthesis.	Complete proteome; Cytoplasm; Lyase; Magnesium; Metal-binding; Reference proteome
SRR342023_TR14526_c0_g1_i2_len=1978	ATP4_IPOBA	Q:131-736,H:1-200	76.73%ID	E:2e-95	ATP synthase subunit delta', mitochondrial	PF02823.13^ATP-synt_DE_N^ATP synthase, Delta/Epsilon chain, beta-sandwich domain^115-186^E:2.8e-17	0,001	1,898	0,014	1,313	0	2,869	0	2,928	0,204	-0,54	ATP4_IPOBA	ATP synthase subunit delta', mitochondrial (F-ATPase delta' subunit)		Ipomoea batatas (Sweet potato) (Convolvulus batatas)		ATP synthesis; CF(1); Direct protein sequencing; Hydrogen ion transport; Ion transport; Membrane; Mitochondrion; Mitochondrion inner membrane; Transit peptide; Transport
SRR646572_TR15307_c0_g4_i1_len=832	NA	NA	NA	NA	NA	PF07172.8^GRP^Glycine rich protein family^1-100^E:4.6e-10	0	1,133	0,535	0,19	0	4,972	0	3,743	0	-1,645	.	.	.	.	.	.
SRR646596_TR19953_c0_g1_i1_len=664	NA	NA	NA	NA	NA	.	0	7,426	1	3,463	0	8,327	0	8,986	0,502	3,751	.	.	.	.	.	.
SRR648705_TR2780_c0_g3_i1_len=850	SUMO2_ARATH	Q:649-383,H:6-93	89.89%ID	E:9e-49	Small ubiquitin-related modifier 2	PF11976.5^Rad60-SLD^Ubiquitin-2 like Rad60 SUMO-like^26-96^E:1.4e-25`PF00240.20^ubiquitin^Ubiquitin family^28-98^E:2.5e-14	0	1,302	0,756	0,109	0	1,062	0	0,994	0,836	-0,15	SUMO2_ARATH	Small ubiquitin-related modifier 2 (AtSUMO2)	SUMO2 SUM2 At5g55160 MCO15.11	Arabidopsis thaliana (Mouse-ear cress)		Alternative splicing; Complete proteome; Cytoplasm; Isopeptide bond; Nucleus; Reference proteome; Ubl conjugation pathway
SRR924147_TR732_c0_g1_i1_len=811	NA	NA	NA	NA	NA	.	0	7,034	0,001	6,489	0	9,833	0	7,074	1	0,939	.	.	.	.	.	.
SRR1144633_TR1165_c0_g1_i1_len=957	NA	NA	NA	NA	NA	PF02519.11^Auxin_inducible^Auxin responsive protein^51-149^E:2.2e-15	0,278	-0,831	0	4,367	0	4,284	0	5,291	0,282	-1,174	.	.	.	.	.	.
SRR1144633_TR1309_c1_g1_i1_len=811	NA	NA	NA	NA	NA	.	0,09	1,022	0	2,102	0	1,968	0	2,405	0,694	0,439	.	.	.	.	.	.
SRR1144633_TR2352_c0_g2_i1_len=2310	PLT5_ARATH	Q:2071-608,H:35-525	68.7%ID	E:0	Polyol transporter 5	PF00083.21^Sugar_tr^Sugar (and other) transporter^67-525^E:1.4e-109`PF07690.13^MFS_1^Major Facilitator Superfamily^73-465^E:4.1e-28	0,004	2,063	0	3,769	0	2,267	0	1,968	0,094	-1,199	PLT5_ARATH	Polyol transporter 5 (Protein POLYOL TRANSPORTER 5) (AtPLT5) (Sugar-proton symporter PLT5)	PLT5 At3g18830 MVE11.10 MVE11.22	Arabidopsis thaliana (Mouse-ear cress)		Cell membrane; Complete proteome; Membrane; Reference proteome; Sugar transport; Symport; Transmembrane; Transmembrane helix; Transport
SRR1144633_TR6816_c0_g1_i1_len=1331	GUAD_BACSU	Q:588-875,H:6-101	56.25%ID	E:2e-28	Guanine deaminase	PF00383.20^dCMP_cyt_deam_1^Cytidine and deoxycytidylate deaminase zinc-binding region^31-130^E:2.7e-24`PF14437.3^MafB19-deam^MafB19-like deaminase^32-146^E:4.8e-22	0,123	0,718	0	2,159	0	1,147	0	1,155	0,597	0,226	GUAD_BACSU	Guanine deaminase (GDEase) (Guanase) (Guanine aminase) (EC 3.5.4.3) (Guanine aminohydrolase) (GAH)	guaD gde BSU13170	Bacillus subtilis (strain 168)	PATHWAY: Purine metabolism; guanine degradation; xanthine from guanine: step 1/1.	3D-structure; Complete proteome; Hydrolase; Metal-binding; Purine metabolism; Reference proteome; Zinc
SRR1144633_TR7186_c0_g1_i1_len=1308	ATHB7_ARATH	Q:1059-739,H:34-140	67.29%ID	E:1e-39	Homeobox-leucine zipper protein ATHB-7	PF00046.26^Homeobox^Homeobox domain^31-85^E:1.1e-16`PF02183.15^HALZ^Homeobox associated leucine zipper^87-127^E:2.7e-13	0,044	2,169	0	4,445	0	2,8	0	1,977	1	1,415	ATHB7_ARATH	Homeobox-leucine zipper protein ATHB-7 (HD-ZIP protein ATHB-7) (Homeodomain transcription factor ATHB-7)	ATHB-7 At2g46680 T3A4.6	Arabidopsis thaliana (Mouse-ear cress)		Alternative splicing; Complete proteome; DNA-binding; Homeobox; Nucleus; Reference proteome; Stress response; Transcription; Transcription regulation
SRR1144633_TR10881_c0_g1_i1_len=1161	ERF08_ARATH	Q:731-360,H:28-166	59.71%ID	E:2e-43	Ethylene-responsive transcription factor ERF008	PF00847.17^AP2^AP2 domain^28-77^E:1.7e-10	0,679	0,375	0	2,789	0	1,678	0,001	1,495	1	-0,7	ERF08_ARATH	Ethylene-responsive transcription factor ERF008	ERF008 At2g23340 T20D16.3	Arabidopsis thaliana (Mouse-ear cress)		Activator; Complete proteome; DNA-binding; Ethylene signaling pathway; Nucleus; Reference proteome; Transcription; Transcription regulation
SRR1144633_TR22909_c1_g1_i1_len=934	CSPLB_RICCO	Q:735-136,H:3-202	77%ID	E:3e-97	CASP-like protein 2B1	PF04535.9^DUF588^Domain of unknown function (DUF588)^28-176^E:1.4e-43	0,03	0,638	0	1,736	0	1,802	0,001	1,049	0,781	-0,435	CSPLB_RICCO	CASP-like protein 2B1 (RcCASPL2B1)	RCOM_0864260	Ricinus communis (Castor bean)		Cell membrane; Complete proteome; Membrane; Reference proteome; Transmembrane; Transmembrane helix
SRR1144633_TR24313_c0_g2_i2_len=1720	STR12_ARATH	Q:913-221,H:70-299	55.84%ID	E:1e-84	Rhodanese-like/PpiC domain-containing protein 12, chloroplastic	PF13616.3^Rotamase_3^PPIC-type PPIASE domain^298-383^E:2.5e-15`PF00639.18^Rotamase^PPIC-type PPIASE domain^307-382^E:4.3e-14`PF00581.17^Rhodanese^Rhodanese-like domain^407-490^E:5.2e-06	0,226	1,812	0	7,652	0	4,231	0	4,294	1	1,679	STR12_ARATH	Rhodanese-like/PpiC domain-containing protein 12, chloroplastic (Sulfurtransferase 12) (AtStr12)	At5g19370 F7K24.120	Arabidopsis thaliana (Mouse-ear cress)		Acetylation; Chloroplast; Complete proteome; Plastid; Reference proteome; Transit peptide
SRR1144633_TR36724_c1_g1_i2_len=1424	PIP1_SOLLC	Q:234-1082,H:1-283	86.57%ID	E:2e-179	Probable aquaporin PIP-type pTOM75	PF00230.17^MIP^Major intrinsic protein^45-274^E:3.2e-84	0,245	0,266	0	1,287	0	1,511	0	0,915	1	0,298	PIP1_SOLLC	Probable aquaporin PIP-type pTOM75 (Ripening-associated membrane protein) (RAMP)		Solanum lycopersicum (Tomato) (Lycopersicon esculentum)		Cell membrane; Complete proteome; Membrane; Reference proteome; Repeat; Stress response; Transmembrane; Transmembrane helix; Transport
SRR1144633_TR37810_c0_g2_i2_len=1025	NA	NA	NA	NA	NA	.	0,083	0,75	0	1,674	0	1,715	0	1,641	1	0,626	.	.	.	.	.	.
SRR1144633_TR37810_c0_g2_i3_len=942	NA	NA	NA	NA	NA	.	0,003	1,259	0	1,183	0	1,653	0,001	1,557	1	0	.	.	.	.	.	.
SRR1144634_TR8901_c0_g1_i1_len=668	NA	NA	NA	NA	NA	PF14009.3^DUF4228^Domain of unknown function (DUF4228)^48-217^E:2.2e-26	1	0	0	7,899	0	1,932	0	2,329	1	-2,355	.	.	.	.	.	.
SRR1144634_TR9562_c0_g1_i4_len=3140	B2_DAUCA	Q:794-1249,H:55-206	91.45%ID	E:2e-93	B2 protein	PF10539.6^Dev_Cell_Death^Development and cell death domain^199-326^E:2.2e-41	0,108	0,763	0	2,127	0	1,647	0	1,883	0,629	2,203	B2_DAUCA	B2 protein		Daucus carota (Wild carrot)		
SRR1144634_TR10749_c0_g1_i2_len=1593	10HGO_CATRO	Q:1458-379,H:1-360	100%ID	E:0	8-hydroxygeraniol dehydrogenase	PF08240.9^ADH_N^Alcohol dehydrogenase GroES-like domain^38-151^E:8.9e-29`PF02826.16^2-Hacid_dh_C^D-isomer specific 2-hydroxyacid dehydrogenase, NAD binding domain^177-226^E:6.8e-06`PF01262.18^AlaDh_PNT_C^Alanine dehydrogenase/PNT, C-terminal domain^185-256^E:9.3e-06`PF00107.23^ADH_zinc_N^Zinc-binding dehydrogenase^194-317^E:1.2e-18	0,041	0,835	0	3,082	0	2,41	0	2,799	0,646	-0,328	10HGO_CATRO	8-hydroxygeraniol dehydrogenase (Cr10HGO) (EC 1.1.1.324)	10HGO	Catharanthus roseus (Madagascar periwinkle) (Vinca rosea)		Metal-binding; NADP; Oxidoreductase; Zinc
SRR1144634_TR11003_c0_g2_i7_len=772	GPX4_MESCR	Q:251-727,H:1-160	83.75%ID	E:5e-98	Probable phospholipid hydroperoxide glutathione peroxidase	PF08534.7^Redoxin^Redoxin^10-154^E:1.7e-07`PF00255.16^GSHPx^Glutathione peroxidase^11-119^E:2.2e-43`PF00578.18^AhpC-TSA^AhpC/TSA family^11-75^E:5.5e-10	0,271	0,34	0	3,976	0	1,874	0	1,615	1	1,985	GPX4_MESCR	Probable phospholipid hydroperoxide glutathione peroxidase (PHGPx) (EC 1.11.1.12)	GPXMC1	Mesembryanthemum crystallinum (Common ice plant) (Cryophytum crystallinum)		Cytoplasm; Oxidoreductase; Peroxidase
SRR1144634_TR11418_c1_g2_i1_len=1627	Y3158_ARATH	Q:1369-902,H:3-143	65.38%ID	E:7e-50	B3 domain-containing protein At3g11580	PF02362.18^B3^B3 DNA binding domain^116-241^E:3.1e-28	0,002	1,501	0	3,303	0	4,739	0	4,342	0,299	-1,38	Y3158_ARATH	B3 domain-containing protein At3g11580 (Protein AUXIN RESPONSE FACTOR 32)	ARF32 At3g11580 F24K9.25	Arabidopsis thaliana (Mouse-ear cress)		Alternative splicing; Complete proteome; DNA-binding; Nucleus; Reference proteome; Transcription; Transcription regulation
SRR1144634_TR13333_c0_g1_i1_len=842	NA	NA	NA	NA	NA	.	0,043	0,984	0	2,059	0	3,905	0	1,983	1	-0,813	.	.	.	.	.	.
SRR1144634_TR14795_c0_g4_i1_len=1925	SCGT_TOBAC	Q:961-1668,H:242-473	67.37%ID	E:1e-171	Scopoletin glucosyltransferase	.	0,312	0,608	0	4,441	0	3,327	0	4,818	0,258	-1,014	SCGT_TOBAC	Scopoletin glucosyltransferase (EC 2.4.1.128) (Phenylpropanoid:glucosyltransferase 1)	TOGT1	Nicotiana tabacum (Common tobacco)		Glycosyltransferase; Transferase
SRR1144634_TR23126_c3_g4_i2_len=688	PRS2_SOLTU	Q:589-134,H:1-155	35.03%ID	E:2e-23	Pathogenesis-related protein STH-2	PF00407.16^Bet_v_1^Pathogenesis-related protein Bet v I family^34-180^E:4.1e-12	0,019	0,54	0	2,568	0	1,44	0	1,492	0,381	1,41	PRS2_SOLTU	Pathogenesis-related protein STH-2	STH-2	Solanum tuberosum (Potato)		Complete proteome; Pathogenesis-related protein; Plant defense; Reference proteome
SRR1144634_TR27142_c0_g1_i4_len=2112	CSE_ARATH	Q:412-1278,H:36-323	35.86%ID	E:4e-52	Caffeoylshikimate esterase	PF12146.5^Hydrolase_4^Serine aminopeptidase, S33^137-379^E:5.8e-65`PF00561.17^Abhydrolase_1^alpha/beta hydrolase fold^139-375^E:1.9e-15`PF12697.4^Abhydrolase_6^Alpha/beta hydrolase family^143-381^E:9.9e-13	0,004	1,469	0	4,393	0	2,347	0	2,578	0,352	0,858	CSE_ARATH	Caffeoylshikimate esterase (EC 3.1.1.-) (Lysophospholipase 2) (LysoPL2)	CSE At1g52760 F14G24.3	Arabidopsis thaliana (Mouse-ear cress)		Cell membrane; Complete proteome; Hydrolase; Lignin biosynthesis; Membrane; Reference proteome
SRR1144634_TR28149_c0_g1_i1_len=645	PRS1_SOLTU	Q:567-124,H:1-155	36.54%ID	E:4e-22	Pathogenesis-related protein STH-21	PF00407.16^Bet_v_1^Pathogenesis-related protein Bet v I family^4-143^E:1e-12	0,082	0,933	0	0,939	0	0,955	0	0,955	1	-1,144	PRS1_SOLTU	Pathogenesis-related protein STH-21	STH-21	Solanum tuberosum (Potato)		Complete proteome; Pathogenesis-related protein; Plant defense; Reference proteome
SRR1144634_TR29160_c0_g1_i5_len=1010	UBC2_WHEAT	Q:744-289,H:1-152	96.05%ID	E:8e-106	Ubiquitin-conjugating enzyme E2 2	PF00179.23^UQ_con^Ubiquitin-conjugating enzyme^8-144^E:1.9e-52	0,484	-0,389	0	2,498	0	7,996	0	7,267	0,461	-1,351	UBC2_WHEAT	Ubiquitin-conjugating enzyme E2 2 (EC 2.3.2.23) (E2 ubiquitin-conjugating enzyme 2) (Ubiquitin carrier protein 2) (Ubiquitin-conjugating enzyme E2-16 kDa) (Ubiquitin-protein ligase 2)	UBC2 UBC1	Triticum aestivum (Wheat)	PATHWAY: Protein modification; protein ubiquitination. {ECO:0000255|PROSITE-ProRule:PRU00388}.	ATP-binding; Complete proteome; Nucleotide-binding; Reference proteome; Transferase; Ubl conjugation pathway
SRR1144634_TR33949_c0_g1_i2_len=1018	APX1_ARATH	Q:86-835,H:1-250	85.6%ID	E:4e-147	L-ascorbate peroxidase 1, cytosolic	PF00141.20^peroxidase^Peroxidase^22-227^E:4.7e-49	0,42	-0,182	0	1,665	0,001	0,789	0,001	0,777	0,7	-0,092	APX1_ARATH	L-ascorbate peroxidase 1, cytosolic (AP) (AtAPx01) (EC 1.11.1.11)	APX1 At1g07890 F24B9.2	Arabidopsis thaliana (Mouse-ear cress)		Alternative splicing; Calcium; Complete proteome; Cytoplasm; Direct protein sequencing; Heme; Hydrogen peroxide; Iron; Metal-binding; Oxidoreductase; Peroxidase; Phosphoprotein; Potassium; Reference proteome
SRR1144634_TR37303_c0_g2_i6_len=4832	LOX31_SOLTU	Q:2366-4831,H:1-826	79.21%ID	E:0	Linoleate 13S-lipoxygenase 3-1, chloroplastic	PF12146.5^Hydrolase_4^Serine aminopeptidase, S33^68-184^E:1e-08`PF01477.20^PLAT^PLAT/LH2 domain^592-676^E:1.4e-12`PF00305.16^Lipoxygenase^Lipoxygenase^689-1285^E:9.5e-273	0,05	1,004	0	3,803	0	2,61	0	3,358	1	0,694	LOX31_SOLTU	Linoleate 13S-lipoxygenase 3-1, chloroplastic (EC 1.13.11.12)	LOX3.1 LOX-H3	Solanum tuberosum (Potato)	PATHWAY: Lipid metabolism; oxylipin biosynthesis. {ECO:0000255|PROSITE-ProRule:PRU00726}.	Chloroplast; Complete proteome; Dioxygenase; Fatty acid biosynthesis; Fatty acid metabolism; Iron; Lipid biosynthesis; Lipid metabolism; Metal-binding; Oxidoreductase; Oxylipin biosynthesis; Plastid; Reference proteome; Thylakoid; Transit peptide
SRR122236_TR298_c0_g1_i1_len=791	GASAE_ARATH	Q:400-594,H:211-275	76.92%ID	E:2e-26	Gibberellin-regulated protein 14	PF02704.11^GASA^Gibberellin regulated protein^138-198^E:7.6e-19	0,023	1,827	0	4,891	0	8,66	0	9,195	0,266	-0,533	GASAE_ARATH	Gibberellin-regulated protein 14 (GAST1 protein homolog 14)	GASA14 At5g14920 F2G14.40	Arabidopsis thaliana (Mouse-ear cress)		Alternative splicing; Complete proteome; Disulfide bond; Gibberellin signaling pathway; Reference proteome; Secreted; Signal
SRR122237_TR5686_c0_g2_i1_len=1769	FAD3E_TOBAC	Q:1666-572,H:12-374	76.16%ID	E:0	Omega-3 fatty acid desaturase, endoplasmic reticulum	PF11960.5^DUF3474^Domain of unknown function (DUF3474)^6-62^E:7.5e-26`PF00487.21^FA_desaturase^Fatty acid desaturase^77-328^E:4.4e-33	0,29	0,358	0	2,32	0	2,532	0	3,026	0,004	-0,676	FAD3E_TOBAC	Omega-3 fatty acid desaturase, endoplasmic reticulum (EC 1.14.19.-)	FAD3	Nicotiana tabacum (Common tobacco)	PATHWAY: Lipid metabolism; polyunsaturated fatty acid biosynthesis.	Endoplasmic reticulum; Fatty acid biosynthesis; Fatty acid metabolism; Lipid biosynthesis; Lipid metabolism; Membrane; Oxidoreductase; Transmembrane; Transmembrane helix
SRR122237_TR18628_c0_g2_i1_len=534	NA	NA	NA	NA	NA	.	0,286	0,85	0	3,296	0	6,432	0	6,944	0,132	-1,567	.	.	.	.	.	.
SRR122237_TR28134_c0_g1_i1_len=746	PRS2_SOLTU	Q:110-565,H:1-155	36.94%ID	E:1e-24	Pathogenesis-related protein STH-2	PF00407.16^Bet_v_1^Pathogenesis-related protein Bet v I family^37-183^E:3.4e-13	0,029	1,132	0	1,737	0	1,691	0	1,658	0,019	0,492	PRS2_SOLTU	Pathogenesis-related protein STH-2	STH-2	Solanum tuberosum (Potato)		Complete proteome; Pathogenesis-related protein; Plant defense; Reference proteome
SRR122238_TR814_c0_g1_i2_len=757	IPRT1_CATRO	Q:100-579,H:1-157	68.75%ID	E:1e-66	Probable intracellular pathogenesis-related protein T1	PF00407.16^Bet_v_1^Pathogenesis-related protein Bet v I family^38-190^E:4.4e-19	0,76	0,71	0	6,45	0	7,666	0	3,568	0,381	-2,222	IPRT1_CATRO	Probable intracellular pathogenesis-related protein T1	PCKR3	Catharanthus roseus (Madagascar periwinkle) (Vinca rosea)		Direct protein sequencing; Glycoprotein; Pathogenesis-related protein; Plant defense
SRR122238_TR6367_c0_g1_i2_len=3126	LAL5_ARATH	Q:2989-1592,H:8-464	59.87%ID	E:6e-164	.	PF01554.15^MatE^MatE^57-216^E:1.4e-29`PF01554.15^MatE^MatE^278-439^E:1.1e-25	0,002	1,289	0	2,917	0	2,075	0	2,067	0,854	-0,18	DTX18_ARATH	Protein DETOXIFICATION 18 (AtDTX18) (Multidrug and toxic compound extrusion protein 18) (MATE protein 18) (Protein LIKE ALF5)	DTX18 LAL5 At3g23550 MDB19.3	Arabidopsis thaliana (Mouse-ear cress)		Complete proteome; Membrane; Reference proteome; Transmembrane; Transmembrane helix; Transport
SRR122238_TR7833_c0_g1_i1_len=1316	BI1L_ARATH	Q:894-196,H:24-256	52.97%ID	E:6e-69	BI1-like protein	PF01027.17^Bax1-I^Inhibitor of apoptosis-promoting Bax1^31-237^E:1.5e-47	0,729	0,262	0	2,297	0,001	1,866	0,001	1,896	1	-0,194	BI1L_ARATH	BI1-like protein	At4g15470 dl3775w FCAALL.58	Arabidopsis thaliana (Mouse-ear cress)		Complete proteome; Membrane; Reference proteome; Transmembrane; Transmembrane helix
SRR122238_TR8264_c1_g1_i3_len=1114	UBC4_WHEAT	Q:278-721,H:1-148	82.43%ID	E:6e-86	Ubiquitin-conjugating enzyme E2-23 kDa	PF00179.23^UQ_con^Ubiquitin-conjugating enzyme^12-142^E:1.5e-40	0,611	0,401	0	1,876	0	3	0	3,836	0,611	-0,318	UBC4_WHEAT	Ubiquitin-conjugating enzyme E2-23 kDa (EC 2.3.2.23) (E2 ubiquitin-conjugating enzyme) (Ubiquitin carrier protein) (Ubiquitin-protein ligase)	UBC4	Triticum aestivum (Wheat)	PATHWAY: Protein modification; protein ubiquitination. {ECO:0000255|PROSITE-ProRule:PRU00388}.	ATP-binding; Complete proteome; Nucleotide-binding; Reference proteome; Transferase; Ubl conjugation pathway
SRR122238_TR17655_c0_g1_i2_len=618	PRS2_SOLTU	Q:10-438,H:12-153	38.78%ID	E:6e-22	Pathogenesis-related protein STH-2	PF00407.16^Bet_v_1^Pathogenesis-related protein Bet v I family^4-142^E:1.5e-10	0,226	0,716	0	2,061	0	1,103	0	1,107	0,1	0,887	PRS2_SOLTU	Pathogenesis-related protein STH-2	STH-2	Solanum tuberosum (Potato)		Complete proteome; Pathogenesis-related protein; Plant defense; Reference proteome
SRR122238_TR20762_c0_g1_i2_len=771	NA	NA	NA	NA	NA	.	1	0,098	0	6,97	0	4,028	0	5,133	1	-0,174	.	.	.	.	.	.
SRR122238_TR21292_c1_g1_i1_len=1399	COMT1_CATRO	Q:79-1158,H:1-360	100%ID	E:0	Caffeic acid 3-O-methyltransferase	PF08100.8^Dimerisation^Dimerisation domain^34-85^E:1e-22`PF00891.15^Methyltransf_2^O-methyltransferase^129-345^E:7.8e-90	0,096	0,539	0	2,176	0	1,621	0	1,391	0,927	-0,042	COMT1_CATRO	Caffeic acid 3-O-methyltransferase (CAOMT) (COMT) (EC 2.1.1.68) (S-adenosysl-L-methionine:caffeic acid 3-O-methyltransferase)	COMT1	Catharanthus roseus (Madagascar periwinkle) (Vinca rosea)	PATHWAY: Aromatic compound metabolism; phenylpropanoid biosynthesis.	Lignin biosynthesis; Methyltransferase; S-adenosyl-L-methionine; Transferase
SRR122238_TR21630_c0_g1_i2_len=2241	NA	NA	NA	NA	NA	PF06200.11^tify^tify domain^98-128^E:7.1e-18	0,017	4,016	0	5,4	0	5,453	0	5,21	1	0,235	.	.	.	.	.	.
SRR122238_TR32710_c0_g1_i1_len=1131	NA	NA	NA	NA	NA	.	0,002	1,07	0	2,671	0	2,01	0	2,224	0,729	0,219	.	.	.	.	.	.
SRR122238_TR34712_c0_g1_i1_len=841	12KD_FRAAN	Q:65-439,H:1-109	57.6%ID	E:1e-37	Auxin-repressed 12.5 kDa protein	PF05564.9^Auxin_repressed^Dormancy/auxin associated protein^28-147^E:2.1e-56	0,005	0,643	0	1,069	0	2,604	0	2,24	0,18	0,378	12KD_FRAAN	Auxin-repressed 12.5 kDa protein		Fragaria ananassa (Strawberry)		Auxin signaling pathway
SRR122238_TR38504_c0_g1_i2_len=1384	BUP1_DICDI	Q:181-1251,H:5-390	59.33%ID	E:5e-154	Beta-ureidopropionase	PF00795.19^CN_hydrolase^Carbon-nitrogen hydrolase^134-381^E:6.5e-51	0,129	0,918	0	1,718	0	1,827	0	2,012	1	0,075	BUP1_DICDI	Beta-ureidopropionase (EC 3.5.1.6) (Beta-alanine synthase) (N-carbamoyl-beta-alanine amidohydrolase)	pyd3 bup1 upb1 DDB_G0274123	Dictyostelium discoideum (Slime mold)	PATHWAY: Amino-acid biosynthesis; beta-alanine biosynthesis.	Acetylation; Complete proteome; Direct protein sequencing; Hydrolase; Reference proteome
SRR122238_TR39267_c0_g2_i3_len=2340	PPH_ARATH	Q:471-1886,H:3-472	59.37%ID	E:0	Pheophytinase, chloroplastic	PF00561.17^Abhydrolase_1^alpha/beta hydrolase fold^157-439^E:1.7e-13`PF12697.4^Abhydrolase_6^Alpha/beta hydrolase family^159-445^E:4.6e-18	0,002	2,095	0	5,778	0	3,13	0	3,552	0,079	1,017	PPH_ARATH	Pheophytinase, chloroplastic (EC 3.1.1.-) (Pheophytin pheophorbide hydrolase) (Protein CO-REGULATED WITH NYE1)	PPH CRN1 At5g13800 MAC12.25	Arabidopsis thaliana (Mouse-ear cress)		Chlorophyll catabolism; Chloroplast; Complete proteome; Hydrolase; Membrane; Plastid; Reference proteome; Thylakoid; Transit peptide
SRR122238_TR40662_c1_g2_i7_len=2549	INVE_ARATH	Q:304-2142,H:1-617	72.34%ID	E:0	Alkaline/neutral invertase E, chloroplastic {ECO:0000305}	PF12899.4^Glyco_hydro_100^Alkaline and neutral invertase^131-572^E:4.9e-215	0,419	0,474	0,001	1,471	0	1,687	0	2,076	0,748	0,26	INVE_ARATH	Alkaline/neutral invertase E, chloroplastic (A/N-INVE) (EC 3.2.1.26)	INVE At5g22510 MQJ16.5	Arabidopsis thaliana (Mouse-ear cress)		Carbohydrate metabolism; Chloroplast; Complete proteome; Glycosidase; Hydrolase; Phosphoprotein; Plastid; Reference proteome; Transit peptide
SRR122238_TR42430_c0_g1_i1_len=615	NA	NA	NA	NA	NA	.	0,029	0,777	0	2,509	0	1,251	0	1,142	0,304	-0,42	.	.	.	.	.	.
SRR122238_TR42512_c0_g1_i16_len=1198	LGUL_CICAR	Q:953-396,H:1-186	80.65%ID	E:6e-110	Lactoylglutathione lyase	PF00903.22^Glyoxalase^Glyoxalase/Bleomycin resistance protein/Dioxygenase superfamily^29-171^E:5.6e-23`PF13669.3^Glyoxalase_4^Glyoxalase/Bleomycin resistance protein/Dioxygenase superfamily^30-153^E:3.9e-06	1	0	0	6,875	0	7,377	0	7,239	0,066	5,397	LGUL_CICAR	Lactoylglutathione lyase (EC 4.4.1.5) (Aldoketomutase) (Glyoxalase I) (Glx I) (Ketone-aldehyde mutase) (Methylglyoxalase) (S-D-lactoylglutathione methylglyoxal lyase)		Cicer arietinum (Chickpea) (Garbanzo)	PATHWAY: Secondary metabolite metabolism; methylglyoxal degradation; (R)-lactate from methylglyoxal: step 1/2.	Lyase; Metal-binding; Zinc
SRR122238_TR43316_c0_g1_i3_len=1378	NA	NA	NA	NA	NA	.	0,059	1,55	0	3,449	0	2,898	0	3,719	0,407	0,781	.	.	.	.	.	.
SRR122238_TR44242_c1_g1_i2_len=2780	ANXD2_ARATH	Q:290-1234,H:1-316	73.42%ID	E:5e-166	Annexin D2	PF00191.17^Annexin^Annexin^16-79^E:1.1e-21`PF00191.17^Annexin^Annexin^88-152^E:5.2e-17`PF00191.17^Annexin^Annexin^171-233^E:2.1e-12`PF00191.17^Annexin^Annexin^248-310^E:1e-17	0,493	-0,229	0	1,387	0	4,188	0	4,562	0,029	-0,779	ANXD2_ARATH	Annexin D2 (AnnAt2)	ANN2 ANNAT2 At5g65020 MXK3.27	Arabidopsis thaliana (Mouse-ear cress)		Acetylation; Alternative splicing; Annexin; Calcium; Calcium/phospholipid-binding; Complete proteome; Cytoplasm; Membrane; Reference proteome; Repeat
SRR122238_TR45975_c1_g1_i3_len=3347	LOX31_SOLTU	Q:619-3030,H:114-914	83.23%ID	E:0	Linoleate 13S-lipoxygenase 3-1, chloroplastic	PF00305.16^Lipoxygenase^Lipoxygenase^1-585^E:2.8e-266	0,023	1,736	0	3,909	0	3,68	0	4,294	1	3,41	LOX31_SOLTU	Linoleate 13S-lipoxygenase 3-1, chloroplastic (EC 1.13.11.12)	LOX3.1 LOX-H3	Solanum tuberosum (Potato)	PATHWAY: Lipid metabolism; oxylipin biosynthesis. {ECO:0000255|PROSITE-ProRule:PRU00726}.	Chloroplast; Complete proteome; Dioxygenase; Fatty acid biosynthesis; Fatty acid metabolism; Iron; Lipid biosynthesis; Lipid metabolism; Metal-binding; Oxidoreductase; Oxylipin biosynthesis; Plastid; Reference proteome; Thylakoid; Transit peptide

SRR122238_TR47659_c1_g2_i4_len=1025	NA	NA	NA	NA	NA	.	0,065	1,405	0	2,151	0	1,907	0,001	1,437	1	0,238	.	.	.	.	.	.
SRR122238_TR49802_c0_g1_i3_len=1065	SRG1_ARATH	Q:1033-218,H:83-356	51.46%ID	E:6e-103	Protein SRG1	PF14226.3^DIOX_N^non-haem dioxygenase in morphine synthesis N-terminal^12-78^E:6.2e-13`PF03171.17^2OG-FeII_Oxy^2OG-Fe(II) oxygenase superfamily^139-235^E:2.2e-25	0,129	2,924	0	7,444	0	6,787	0	6,095	1	0	SRG1_ARATH	Protein SRG1 (AtSRG1) (Protein SENESCENCE-RELATED GENE 1)	SRG1 At1g17020 F20D23.28 F6I1.30	Arabidopsis thaliana (Mouse-ear cress)		Complete proteome; Iron; Metal-binding; Oxidoreductase; Reference proteome
SRR122238_TR53155_c1_g1_i1_len=887	NA	NA	NA	NA	NA	.	0,629	1,092	0	4,796	0	4,536	0	4,896	1	2,191	.	.	.	.	.	.
SRR122238_TR58674_c0_g1_i2_len=1920	WTR41_ARATH	Q:1627-587,H:13-356	46.31%ID	E:8e-88	WAT1-related protein At5g40230	PF00892.17^EamA^EamA-like transporter family^17-156^E:2.9e-13`PF00892.17^EamA^EamA-like transporter family^187-322^E:2.4e-09	0,017	0,817	0	2,578	0	1,977	0	1,736	0,004	2,914	WTR41_ARATH	WAT1-related protein At5g40230	At5g40230 MSN9.130	Arabidopsis thaliana (Mouse-ear cress)		Complete proteome; Membrane; Reference proteome; Repeat; Transmembrane; Transmembrane helix
SRR122238_TR60351_c0_g1_i3_len=1058	OP24B_ARATH	Q:194-829,H:3-213	67.92%ID	E:4e-101	Outer envelope pore protein 24B, chloroplastic	.	0,088	0,512	0	1,529	0	1,995	0	2,32	0,352	-0,294	OP24B_ARATH	Outer envelope pore protein 24B, chloroplastic (Chloroplastic outer envelope pore protein of 24 kDa B)	OEP24B At5g42960 MBD2.16	Arabidopsis thaliana (Mouse-ear cress)		Chloroplast; Complete proteome; Ion transport; Membrane; Plastid; Plastid outer membrane; Porin; Reference proteome; Transmembrane; Transmembrane beta strand; Transport
SRR122238_TR62791_c2_g1_i6_len=2165	PTR21_ARATH	Q:1982-279,H:31-594	55.28%ID	E:0	Protein NRT1/ PTR FAMILY 2.13	PF00854.18^PTR2^POT family^155-585^E:1.9e-85	0,08	1,259	0	7,27	0	2,658	0	1,714	0,017	5,744	PTR21_ARATH	Protein NRT1/ PTR FAMILY 2.13 (AtNPF2.13) (Nitrate transporter 1.7)	NPF2.13 NRT1.7 At1g69870 T17F3.10	Arabidopsis thaliana (Mouse-ear cress)		Cell membrane; Complete proteome; Membrane; Nitrate assimilation; Reference proteome; Symport; Transmembrane; Transmembrane helix; Transport
SRR122238_TR62903_c2_g1_i4_len=1839	C81E8_MEDTR	Q:1611-265,H:43-494	45.26%ID	E:2e-131	Cytochrome P450 81E8 {ECO:0000303|PubMed:14617078}	PF00067.19^p450^Cytochrome P450^39-483^E:9.3e-88	1	-0,252	0,001	3,25	0	4,198	0	5,47	1	-0,984	C81E8_MEDTR	Cytochrome P450 81E8 (EC 1.14.13.-)	CYP81E8	Medicago truncatula (Barrel medic) (Medicago tribuloides)		Heme; Iron; Membrane; Metal-binding; Monooxygenase; NADP; Oxidoreductase; Transmembrane; Transmembrane helix
SRR122238_TR66612_c0_g1_i2_len=1471	MY108_ARATH	Q:1330-899,H:10-151	69.44%ID	E:2e-60	Transcription factor MYB108	PF00249.28^Myb_DNA-binding^Myb-like DNA-binding domain^61-108^E:1.1e-16`PF13921.3^Myb_DNA-bind_6^Myb-like DNA-binding domain^64-122^E:1.1e-13`PF00249.28^Myb_DNA-binding^Myb-like DNA-binding domain^114-157^E:1.3e-15`PF13921.3^Myb_DNA-bind_6^Myb-like DNA-binding domain^118-161^E:4.9e-06	0,356	1,29	0	3,119	0	5,501	0	5,805	0,56	-0,723	MY108_ARATH	Transcription factor MYB108 (Myb-related protein 108) (AtMYB108) (Protein BOTRYTIS-SUSCEPTIBLE 1)	MYB108 BOS1 At3g06490 F5E6.18	Arabidopsis thaliana (Mouse-ear cress)		Activator; Complete proteome; DNA-binding; Nucleus; Plant defense; Reference proteome; Repeat; Transcription; Transcription regulation; Ubl conjugation
SRR122238_TR67350_c0_g1_i1_len=1628	U74E2_ARATH	Q:53-1420,H:6-450	44.86%ID	E:3e-136	UDP-glycosyltransferase 74E2	PF00201.15^UDPGT^UDP-glucoronosyl and UDP-glucosyl transferase^292-449^E:4.4e-30	0,87	0,225	0	2,485	0	1,825	0	1,467	0,013	1,211	U74E2_ARATH	UDP-glycosyltransferase 74E2 (EC 2.4.1.-)	UGT74E2 At1g05680 F3F20.13	Arabidopsis thaliana (Mouse-ear cress)		Complete proteome; Glycosyltransferase; Reference proteome; Stress response; Transferase
SRR122238_TR68934_c0_g2_i3_len=1541	NA	NA	NA	NA	NA	.	0,438	0,314	0	1,946	0	1,23	0,001	1,209	0,781	0,396	.	.	.	.	.	.
SRR122238_TR76492_c0_g1_i2_len=1199	NA	NA	NA	NA	NA	.	0,006	1,124	0	1,993	0	2,211	0	1,733	0,355	-0,419	.	.	.	.	.	.
SRR122238_TR84651_c0_g1_i2_len=944	NDADA_ARATH	Q:737-309,H:1-143	87.41%ID	E:8e-87	NADH dehydrogenase [ubiquinone] 1 alpha subcomplex subunit 13-A	PF06212.9^GRIM-19^GRIM-19 protein^20-138^E:2.2e-42	0,657	-0,194	0	8,565	0	8,785	0	8,29	0,196	-0,541	NDADA_ARATH	NADH dehydrogenase [ubiquinone] 1 alpha subcomplex subunit 13-A (Protein MATERNAL EFFECT EMBRYO ARREST 4)	MEE4 At1g04630 T1G11.12	Arabidopsis thaliana (Mouse-ear cress)		Complete proteome; Electron transport; Membrane; Mitochondrion; Mitochondrion inner membrane; Reference proteome; Respiratory chain; Transmembrane; Transmembrane helix; Transport
SRR122238_TR86615_c1_g1_i2_len=1397	XTH30_ARATH	Q:1265-342,H:9-311	64.61%ID	E:2e-148	Probable xyloglucan endotransglucosylase/hydrolase protein 30	PF00722.18^Glyco_hydro_16^Glycosyl hydrolases family 16^45-226^E:2.7e-54`PF06955.9^XET_C^Xyloglucan endo-transglycosylase (XET) C-terminus^268-305^E:6.8e-15	1	2,324	0	5,927	0	4,567	0	4,035	1	0,355	XTH30_ARATH	Probable xyloglucan endotransglucosylase/hydrolase protein 30 (At-XTH30) (XTH-30) (EC 2.4.1.207)	XTH30 XTR4 At1g32170 F3C3.5	Arabidopsis thaliana (Mouse-ear cress)		Apoplast; Cell wall; Cell wall biogenesis/degradation; Complete proteome; Disulfide bond; Glycoprotein; Glycosidase; Hydrolase; Reference proteome; Secreted; Signal; Transferase
SRR122238_TR87584_c0_g1_i2_len=1109	FER3_ARATH	Q:331-786,H:1-155	56.77%ID	E:3e-56	Ferredoxin-3, chloroplastic	PF00111.24^Fer2^2Fe-2S iron-sulfur cluster binding domain^65-138^E:2.6e-21	0,184	0,347	0	3,73	0	1,45	0	1,825	0,443	-0,213	FER3_ARATH	Ferredoxin-3, chloroplastic (AtFd3)	FD3 At2g27510 F10A12.19	Arabidopsis thaliana (Mouse-ear cress)		2Fe-2S; Acetylation; Chloroplast; Complete proteome; Electron transport; Iron; Iron-sulfur; Metal-binding; Plastid; Reference proteome; Transit peptide; Transport
SRR1271857_TR5151_c0_g1_i1_len=696	EP1G_DAUCA	Q:547-74,H:26-183	67.09%ID	E:1e-71	Epidermis-specific secreted glycoprotein EP1	PF01453.21^B_lectin^D-mannose binding lectin^87-172^E:1.1e-26	0,007	-0,998	0	9,106	0	4,411	0	2,312	0	-1,524	EP1G_DAUCA	Epidermis-specific secreted glycoprotein EP1 (52/54 kDa medium protein)	EP1	Daucus carota (Wild carrot)		Direct protein sequencing; Glycoprotein; Lectin; Secreted; Signal
SRR1271857_TR6476_c0_g1_i2_len=887	PIP27_MAIZE	Q:864-1,H:1-287	81.66%ID	E:2e-152	Aquaporin PIP2-7	PF00230.17^MIP^Major intrinsic protein^41-276^E:3.5e-83	0,502	2,562	0	5,866	0	3,847	0	2,899	1	0,232	PIP27_MAIZE	Aquaporin PIP2-7 (Plasma membrane intrinsic protein 2-7) (ZmPIP2-7) (ZmPIP2;7)	PIP2-7	Zea mays (Maize)		Cell membrane; Complete proteome; Membrane; Reference proteome; Repeat; Transmembrane; Transmembrane helix; Transport
SRR1271857_TR10912_c0_g1_i3_len=1661	AOC4_ARATH	Q:1020-1412,H:117-248	68.18%ID	E:1e-49	Allene oxide cyclase 4, chloroplastic	.	0,004	1,818	0	4,532	0	2,994	0	3,249	0,398	0,342	AOC4_ARATH	Allene oxide cyclase 4, chloroplastic (EC 5.3.99.6)	AOC4 At1g13280 T6J4.4 T6J4_23	Arabidopsis thaliana (Mouse-ear cress)		Chloroplast; Complete proteome; Isomerase; Plastid; Reference proteome; Transit peptide
SRR1271857_TR11128_c0_g1_i2_len=1627	AOC4_ARATH	Q:743-1444,H:22-254	63.14%ID	E:3e-88	Allene oxide cyclase 4, chloroplastic	PF06351.8^Allene_ox_cyc^Allene oxide cyclase^80-252^E:1.2e-93	0,004	1,957	0	5,905	0	3,774	0	4,753	0,593	0,662	AOC4_ARATH	Allene oxide cyclase 4, chloroplastic (EC 5.3.99.6)	AOC4 At1g13280 T6J4.4 T6J4_23	Arabidopsis thaliana (Mouse-ear cress)		Chloroplast; Complete proteome; Isomerase; Plastid; Reference proteome; Transit peptide
SRR1271857_TR17545_c0_g1_i3_len=880	NA	NA	NA	NA	NA	.	1	0,707	0	3,76	0	4,687	0	4,882	0,076	3,277	.	.	.	.	.	.
SRR1271857_TR21990_c0_g1_i1_len=649	DCTP1_MOUSE	Q:217-519,H:30-130	48.51%ID	E:2e-28	dCTP pyrophosphatase 1	PF03819.14^MazG^MazG nucleotide pyrophosphohydrolase domain^76-149^E:2e-07`PF12643.4^MazG-like^MazG-like family^87-156^E:1.8e-07	0,01	2,514	0	1,939	0	2,083	0	2,881	0,299	-1,53	DCTP1_MOUSE	dCTP pyrophosphatase 1 (EC 3.6.1.12) (Deoxycytidine-triphosphatase 1) (dCTPase 1) (RS21-C6) (XTP3-transactivated gene A protein homolog)	Dctpp1 Tdrg-TL1	Mus musculus (Mouse)		3D-structure; Acetylation; Complete proteome; Cytoplasm; Hydrolase; Magnesium; Metal-binding; Nucleotide-binding; Phosphoprotein; Reference proteome
SRR1271857_TR27295_c0_g2_i1_len=1107	PX24B_DICDI	Q:153-737,H:1-182	31.28%ID	E:3e-25	PXMP2/4 family protein 2	PF04117.9^Mpv17_PMP22^Mpv17 / PMP22 family^129-189^E:1.2e-15	1	0,004	0	3,551	0	3,517	0,001	3,243	0,076	-2,445	PX24B_DICDI	PXMP2/4 family protein 2	DDB_G0278529	Dictyostelium discoideum (Slime mold)		Complete proteome; Membrane; Reference proteome; Transmembrane; Transmembrane helix
SRR1271857_TR32991_c0_g2_i1_len=825	NA	NA	NA	NA	NA	PF00582.23^Usp^Universal stress protein family^45-190^E:2.8e-26	1	3,408	0	4,301	0	3,459	0	2,459	1	0	.	.	.	.	.	.
SRR1271857_TR34670_c0_g1_i1_len=957	NA	NA	NA	NA	NA	PF06813.10^Nodulin-like^Nodulin-like^69-314^E:9.6e-95	0,461	1,168	0	3,986	0	5,317	0	6,32	1	-1,155	.	.	.	.	.	.
SRR1271857_TR37691_c0_g1_i1_len=1014	NA	NA	NA	NA	NA	PF06232.8^ATS3^Embryo-specific protein 3, (ATS3)^30-132^E:2.4e-08`PF01477.20^PLAT^PLAT/LH2 domain^36-153^E:2.8e-13	0,005	1,595	0	5,677	0	4,542	0	4,919	0,079	1,056	.	.	.	.	.	.
SRR1271858_TR1997_c0_g3_i1_len=1635	NA	NA	NA	NA	NA	.	0,195	1,381	0	3,585	0	2,445	0	2,263	1	0,559	.	.	.	.	.	.
SRR1271858_TR2946_c0_g1_i4_len=4181	PAO2_ARATH	Q:2382-2840,H:244-396	79.74%ID	E:1e-80	Probable polyamine oxidase 2	PF01593.21^Amino_oxidase^Flavin containing amine oxidoreductase^5-147^E:1.6e-30	0,003	1,799	0	4,457	0	3,995	0	4,631	0,854	0,263	PAO2_ARATH	Probable polyamine oxidase 2 (AtPAO2) (EC 1.5.3.-) (Amine oxidase 1)	PAO2 At2g43020 MFL8.12	Arabidopsis thaliana (Mouse-ear cress)		Complete proteome; FAD; Flavoprotein; Oxidoreductase; Peroxisome; Reference proteome
SRR1271858_TR5114_c0_g1_i8_len=3929	THD1_ARATH	Q:3108-2584,H:188-355	77.71%ID	E:2e-115	Threonine dehydratase biosynthetic, chloroplastic	PF00291.22^PALP^Pyridoxal-phosphate dependent enzyme^143-231^E:6.3e-27	0,357	0,662	0	3,029	0	2,897	0	3,746	0,034	-4,996	THD1_ARATH	Threonine dehydratase biosynthetic, chloroplastic (EC 4.3.1.19) (Threonine deaminase) (TD)	OMR1 At3g10050 T22K18.12	Arabidopsis thaliana (Mouse-ear cress)	PATHWAY: Amino-acid biosynthesis; L-isoleucine biosynthesis; 2-oxobutanoate from L-threonine: step 1/1.	Allosteric enzyme; Amino-acid biosynthesis; Branched-chain amino acid biosynthesis; Chloroplast; Complete proteome; Isoleucine biosynthesis; Lyase; Plastid; Pyridoxal phosphate; Reference proteome; Repeat; Transit peptide
SRR1271858_TR6554_c0_g1_i1_len=1057	NA	NA	NA	NA	NA	PF00561.17^Abhydrolase_1^alpha/beta hydrolase fold^53-157^E:2e-15`PF12697.4^Abhydrolase_6^Alpha/beta hydrolase family^54-271^E:1.1e-14	0,025	2,834	0	9,553	0	5,825	0	6,134	1	-0,307	.	.	.	.	.	.
SRR1271858_TR10263_c0_g2_i3_len=1017	NA	NA	NA	NA	NA	.	0,132	2,282	0	7,691	0	9,602	0	10,128	1	-0,092	.	.	.	.	.	.
SRR1271858_TR14849_c0_g1_i3_len=1333	NA	NA	NA	NA	NA	.	1	0,191	0	3,353	0	4,839	0	5,385	0,694	-0,318	.	.	.	.	.	.
SRR1271858_TR15848_c0_g1_i3_len=1769	CCR1_ARATH	Q:337-1287,H:11-322	50.16%ID	E:2e-86	Cinnamoyl-CoA reductase 1	PF01370.18^Epimerase^NAD dependent epimerase/dehydratase family^4-241^E:9.1e-24`PF05368.10^NmrA^NmrA-like family^4-100^E:1e-07`PF01073.16^3Beta_HSD^3-beta hydroxysteroid dehydrogenase/isomerase family^6-238^E:1.1e-20`PF07993.9^NAD_binding_4^Male sterility protein^6-236^E:9.1e-11`PF16363.2^GDP_Man_Dehyd^GDP-mannose 4,6 dehydratase^6-245^E:3.1e-10`PF13460.3^NAD_binding_10^NAD(P)H-binding^8-190^E:3e-13	0,659	0,146	0	1,484	0	2,344	0	2,104	0,76	0,113	CCR1_ARATH	Cinnamoyl-CoA reductase 1 (AtCCR1) (EC 1.2.1.44) (Protein IRREGULAR XYLEM 4)	CCR1 IRX4 At1g15950 T24D18.5	Arabidopsis thaliana (Mouse-ear cress)	PATHWAY: Aromatic compound metabolism; phenylpropanoid biosynthesis.	Alternative splicing; Complete proteome; Lignin biosynthesis; NADP; Oxidoreductase; Phosphoprotein; Reference proteome
SRR1271858_TR20404_c0_g2_i5_len=3343	CRSP_ARATH	Q:1692-2909,H:355-762	55.31%ID	E:6e-143	CO(2)-response secreted protease {ECO:0000303|PubMed:25043023}	PF05922.13^Inhibitor_I9^Peptidase inhibitor I9^7-69^E:5.2e-14`PF00082.19^Peptidase_S8^Subtilase family^97-284^E:3.5e-23	0,239	1,641	0	3,541	0	3,636	0	3,231	1	-0,202	CRSP_ARATH	CO(2)-response secreted protease (Subtilisin-like serine protease) (AtSBT5.2) (Tripeptidyl-peptidase II) (EC 3.4.14.10)	CRSP SBT5.2 At1g20160 T20H2.6	Arabidopsis thaliana (Mouse-ear cress)		Alternative splicing; Cell wall; Complete proteome; Hydrolase; Protease; Reference proteome; Secreted; Serine protease; Signal
SRR1271858_TR20619_c0_g1_i1_len=910	NA	NA	NA	NA	NA	.	0,461	0,905	0	4,669	0	2,472	0,001	1,732	0,025	1,997	.	.	.	.	.	.
SRR1271858_TR20656_c0_g1_i1_len=2019	NA	NA	NA	NA	NA	.	0,132	1,55	0	4,574	0	4,648	0	5,459	1	0,223	.	.	.	.	.	.
SRR1271858_TR21130_c1_g1_i5_len=1339	PIP27_MAIZE	Q:1241-378,H:1-287	82.7%ID	E:7e-151	Aquaporin PIP2-7	PF00230.17^MIP^Major intrinsic protein^34-269^E:4e-83	0,502	3,601	0	8,446	0	5,285	0	3,511	1	0	PIP27_MAIZE	Aquaporin PIP2-7 (Plasma membrane intrinsic protein 2-7) (ZmPIP2-7) (ZmPIP2;7)	PIP2-7	Zea mays (Maize)		Cell membrane; Complete proteome; Membrane; Reference proteome; Repeat; Transmembrane; Transmembrane helix; Transport
SRR1271858_TR28290_c0_g1_i1_len=1275	NA	NA	NA	NA	NA	.	0,003	1,459	0	2,105	0	2,007	0	1,622	0,255	-0,616	.	.	.	.	.	.
SRR1271858_TR28350_c0_g1_i3_len=1607	TLP1_PRUPE	Q:1265-558,H:16-246	53.59%ID	E:3e-73	Thaumatin-like protein 1	PF00314.14^Thaumatin^Thaumatin family^33-253^E:2.4e-84	0,257	0,856	0	3,831	0	1,873	0	2,082	0,118	-1,751	TLP1_PRUPE	Thaumatin-like protein 1 (PpAZ44)		Prunus persica (Peach) (Amygdalus persica)		Disulfide bond; Plant defense; Secreted; Signal
SRR1271858_TR32754_c0_g2_i1_len=840	NA	NA	NA	NA	NA	.	0,62	0,732	0	3,277	0	3,166	0,001	2,819	0	-2,698	.	.	.	.	.	.
SRR1271858_TR36359_c0_g2_i3_len=1956	NA	NA	NA	NA	NA	PF03619.13^Solute_trans_a^Organic solute transporter Ostalpha^9-84^E:2.5e-16	0,051	1,087	0	3,414	0	2,789	0	3,167	0,524	0,585	.	.	.	.	.	.
SRR1271858_TR37116_c0_g1_i1_len=1461	FABG_THEMA	Q:965-165,H:2-246	32.96%ID	E:6e-28	3-oxoacyl-[acyl-carrier-protein] reductase FabG	PF00106.22^adh_short^short chain dehydrogenase^17-226^E:9.2e-48`PF08659.7^KR^KR domain^18-196^E:9e-07`PF13561.3^adh_short_C2^Enoyl-(Acyl carrier protein) reductase^23-278^E:3.9e-45	0,56	0,819	0	6,408	0	6,36	0	7,049	1	0,454	FABG_THEMA	3-oxoacyl-[acyl-carrier-protein] reductase FabG (EC 1.1.1.100) (3-ketoacyl-acyl carrier protein reductase) (Beta-Ketoacyl-acyl carrier protein reductase) (Beta-ketoacyl-ACP reductase)	fabG TM_1724	Thermotoga maritima (strain ATCC 43589 / MSB8 / DSM 3109 / JCM 10099)	PATHWAY: Lipid metabolism; fatty acid biosynthesis.	Complete proteome; Fatty acid biosynthesis; Fatty acid metabolism; Lipid biosynthesis; Lipid metabolism; NADP; Oxidoreductase; Reference proteome
SRR1271858_TR37267_c0_g1_i1_len=930	NA	NA	NA	NA	NA	PF15697.2^DUF4666^Domain of unknown function (DUF4666)^60-99^E:5.1e-12`PF15697.2^DUF4666^Domain of unknown function (DUF4666)^107-141^E:7.5e-08	0,129	1,066	0,001	2,083	0	1,982	0	1,889	0,564	-0,423	.	.	.	.	.	.
SRR1271858_TR38460_c0_g1_i2_len=1583	NA	NA	NA	NA	NA	.	0,254	2,447	0	5,581	0	5,623	0	6,949	0,629	-1,167	.	.	.	.	.	.
SRR1271858_TR45931_c0_g1_i2_len=1180	NA	NA	NA	NA	NA	.	0,068	0,861	0	2,287	0	1,648	0	1,803	0,735	0,211	.	.	.	.	.	.
SRR1271858_TR46225_c0_g1_i1_len=2111	GATP1_SOLLC	Q:1905-391,H:4-515	82.81%ID	E:0	Gamma aminobutyrate transaminase 1, mitochondrial	PF00202.18^Aminotran_3^Aminotransferase class-III^77-495^E:3.5e-98	0,287	0,357	0	1,58	0	1,741	0	1,725	1	-0,364	GATP1_SOLLC	Gamma aminobutyrate transaminase 1, mitochondrial (Gamma-aminobutyrate transaminase isozyme 1) (LeGABA-TP1) (SlGABA-T1) (EC 2.6.1.96)	GABA-TP1 GABA-T1	Solanum lycopersicum (Tomato) (Lycopersicon esculentum)		Aminotransferase; Complete proteome; Mitochondrion; Pyridoxal phosphate; Reference proteome; Transferase; Transit peptide
SRR1271858_TR46904_c0_g1_i3_len=1857	C72A1_CATRO	Q:1148-309,H:245-524	97.14%ID	E:0	Secologanin synthase	PF00067.19^p450^Cytochrome P450^1-236^E:1.7e-63	0,002	1,094	0	3,233	0	3,116	0	2,619	0,38	0,535	C72A1_CATRO	Secologanin synthase (SLS) (EC 1.3.3.9) (CYPLXXII) (Cytochrome P450 72A1)	CYP72A1 CYP72 P450CR3	Catharanthus roseus (Madagascar periwinkle) (Vinca rosea)	PATHWAY: Alkaloid biosynthesis; secologanin biosynthesis.	Alkaloid metabolism; Heme; Iron; Membrane; Metal-binding; Monooxygenase; NADP; Oxidoreductase; Transmembrane; Transmembrane helix; Vacuole
SRR1271858_TR56764_c0_g1_i11_len=2126	IAA26_ARATH	Q:1278-1898,H:60-267	56.62%ID	E:1e-61	Auxin-responsive protein IAA26	PF02309.13^AUX_IAA^AUX/IAA family^43-342^E:2e-68	0,252	0,555	0	3,461	0	3,964	0	4,739	0,763	0,245	IAA26_ARATH	Auxin-responsive protein IAA26 (Indoleacetic acid-induced protein 26) (Phytochrome-associated protein 1)	IAA26 PAP1 At3g16500 MDC8.13	Arabidopsis thaliana (Mouse-ear cress)		Auxin signaling pathway; Complete proteome; Nucleus; Reference proteome; Repressor; Transcription; Transcription regulation
SRR1271858_TR57115_c0_g1_i3_len=1069	NA	NA	NA	NA	NA	PF06232.8^ATS3^Embryo-specific protein 3, (ATS3)^30-132^E:2.4e-08`PF01477.20^PLAT^PLAT/LH2 domain^36-153^E:3.4e-13	0,001	0,805	0	3,109	0	4,579	0	4,177	0,24	0,434	.	.	.	.	.	.
SRR1271858_TR60403_c2_g1_i3_len=3073	NA	NA	NA	NA	NA	.	0,001	4,178	0	4,244	0	3,412	0	2,97	1	-0,417	.	.	.	.	.	.
SRR1271858_TR60616_c0_g1_i3_len=617	NA	NA	NA	NA	NA	.	0,129	3,059	0	6,779	0	7,82	0,001	6,542	1	2,528	.	.	.	.	.	.
SRR1271858_TR61038_c0_g3_i2_len=925	10HGO_CATRO	Q:141-866,H:1-242	100%ID	E:6e-140	8-hydroxygeraniol dehydrogenase	PF08240.9^ADH_N^Alcohol dehydrogenase GroES-like domain^38-151^E:4.4e-29`PF02826.16^2-Hacid_dh_C^D-isomer specific 2-hydroxyacid dehydrogenase, NAD binding domain^177-225^E:4.3e-06`PF00107.23^ADH_zinc_N^Zinc-binding dehydrogenase^194-239^E:2.1e-07	0,35	0,575	0	5,058	0	2,07	0	2,876	0,142	0,587	10HGO_CATRO	8-hydroxygeraniol dehydrogenase (Cr10HGO) (EC 1.1.1.324)	10HGO	Catharanthus roseus (Madagascar periwinkle) (Vinca rosea)		Metal-binding; NADP; Oxidoreductase; Zinc
SRR1271858_TR61149_c0_g1_i1_len=1715	GATL9_ARATH	Q:1368-442,H:51-363	81.79%ID	E:0	Probable galacturonosyltransferase-like 9	PF01501.17^Glyco_transf_8^Glycosyl transferase family 8^77-337^E:1.4e-52	0,886	-0,167	0	2,014	0	2,652	0	3,179	0,004	-1,36	GATL9_ARATH	Probable galacturonosyltransferase-like 9 (EC 2.4.1.-) (Like glycosyl transferase 8)	GATL9 LGT8 At1g70090 F20P5.18	Arabidopsis thaliana (Mouse-ear cress)	PATHWAY: Glycan metabolism; pectin biosynthesis.	Cell wall biogenesis/degradation; Complete proteome; Glycoprotein; Glycosyltransferase; Golgi apparatus; Membrane; Reference proteome; Signal-anchor; Transferase; Transmembrane; Transmembrane helix
SRR1271858_TR63842_c0_g1_i1_len=1678	PLA11_ARATH	Q:303-1394,H:84-447	62.43%ID	E:3e-162	Phospholipase A(1) DAD1, chloroplastic	PF01764.22^Lipase_3^Lipase (class 3)^190-340^E:3e-37	0,629	1,588	0	5,787	0	5,909	0	6,989	1	0	PLA11_ARATH	Phospholipase A(1) DAD1, chloroplastic (EC 3.1.1.32) (Phospholipase A1-Ibeta1) (Protein DEFECTIVE IN ANTHER DEHISCENCE 1) (AtDAD1)	DAD1 At2g44810 T13E15.18	Arabidopsis thaliana (Mouse-ear cress)		Chloroplast; Complete proteome; Hydrolase; Lipid degradation; Lipid metabolism; Plastid; Reference proteome; Transit peptide
SRR1271858_TR64491_c0_g2_i1_len=1521	YODA_ARATH	Q:155-946,H:400-656	40.29%ID	E:2e-45	Mitogen-activated protein kinase kinase kinase YODA	PF00069.22^Pkinase^Protein kinase domain^5-266^E:2.2e-53`PF07714.14^Pkinase_Tyr^Protein tyrosine kinase^6-261^E:2.3e-33	0,005	6,218	0	3,904	0	6,256	0	6,425	1	-0,476	YODA_ARATH	Mitogen-activated protein kinase kinase kinase YODA (EC 2.7.11.25) (YODA MAPKK kinase)	YDA At1g63700 F24D7.11	Arabidopsis thaliana (Mouse-ear cress)		ATP-binding; Complete proteome; Developmental protein; Growth regulation; Kinase; Nucleotide-binding; Reference proteome; Transferase
SRR1271858_TR65581_c0_g1_i4_len=2815	LIP_STAHY	Q:662-1381,H:309-542	29.46%ID	E:1e-23	Lipase	.	0,003	2,057	0	3,278	0	2,55	0	2,495	1	-0,214	LIP_STAHY	Lipase (EC 3.1.1.3) (EC 3.1.1.32) (Phospholipase A1) (Triacylglycerol lipase) [Cleaved into: Lipase 86 kDa form; Lipase 46 kDa form]	lip	Staphylococcus hyicus		3D-structure; Calcium; Direct protein sequencing; Hydrolase; Lipid degradation; Lipid metabolism; Metal-binding; Secreted; Signal
SRR1271858_TR65593_c1_g3_i1_len=865	NA	NA	NA	NA	NA	.	0,692	1,043	0	3,246	0	2,435	0	2,423	1	-0,725	.	.	.	.	.	.
SRR1271858_TR68287_c3_g2_i11_len=4091	RBOHC_SOLTU	Q:3788-1779,H:16-687	81.43%ID	E:0	Respiratory burst oxidase homolog protein C	PF08414.7^NADPH_Ox^Respiratory burst NADPH oxidase^149-250^E:3.9e-40`PF13202.3^EF-hand_5^EF hand^252-274^E:0.004`PF01794.16^Ferric_reduct^Ferric reductase like transmembrane component^410-566^E:4e-23`PF08022.9^FAD_binding_8^FAD-binding domain^609-688^E:5e-28	0,16	1,14	0	3,215	0	6,675	0	8,093	0,502	1,559	RBOHC_SOLTU	Respiratory burst oxidase homolog protein C (EC 1.11.1.-) (EC 1.6.3.-) (NADPH oxidase RBOHC) (StRBOHC)	RBOHC	Solanum tuberosum (Potato)		Calcium; Coiled coil; Complete proteome; FAD; Flavoprotein; Membrane; Metal-binding; NADP; Oxidoreductase; Peroxidase; Phosphoprotein; Reference proteome; Repeat; Transmembrane; Transmembrane helix
SRR1271859_TR897_c0_g1_i1_len=3560	IKU2_ARATH	Q:3307-509,H:24-968	54.1%ID	E:0	Receptor-like protein kinase HAIKU2	PF08263.9^LRRNT_2^Leucine rich repeat N-terminal domain^40-78^E:1e-09`PF08263.9^LRRNT_2^Leucine rich repeat N-terminal domain^342-348^E:7000`PF00560.30^LRR_1^Leucine Rich Repeat^542-563^E:0.62`PF08263.9^LRRNT_2^Leucine rich repeat N-terminal domain^649-658^E:9600`PF00069.22^Pkinase^Protein kinase domain^682-965^E:3.1e-44`PF07714.14^Pkinase_Tyr^Protein tyrosine kinase^683-967^E:1.4e-39	0,111	1,33	0	5,575	0	4,328	0	3,706	1	0,309	IKU2_ARATH	Receptor-like protein kinase HAIKU2 (EC 2.7.11.1)	IKU2 At3g19700 MMB12.19	Arabidopsis thaliana (Mouse-ear cress)		ATP-binding; Complete proteome; Glycoprotein; Kinase; Leucine-rich repeat; Membrane; Nucleotide-binding; Phosphoprotein; Receptor; Reference proteome; Repeat; Serine/threonine-protein kinase; Signal; Transferase; Transmembrane; Transmembrane helix
SRR1271859_TR6380_c0_g1_i1_len=6125	NA	NA	NA	NA	NA	PF07765.9^KIP1^KIP1-like protein^14-87^E:3.5e-38	0,026	0,943	0	3,609	0	1,437	0	1,751	0,109	1,052	.	.	.	.	.	.
SRR1271859_TR7412_c1_g2_i1_len=2743	SPL6_ARATH	Q:989-1627,H:83-262	48.13%ID	E:4e-49	Squamosa promoter-binding-like protein 6	PF03110.11^SBP^SBP domain^174-247^E:8e-32	0,026	1,089	0	3,762	0	2,692	0	2,977	0,663	0,535	SPL6_ARATH	Squamosa promoter-binding-like protein 6	SPL6 At1g69170 F23O10.24 F4N2.13	Arabidopsis thaliana (Mouse-ear cress)		Complete proteome; DNA-binding; Metal-binding; Nucleus; Reference proteome; Transcription; Transcription regulation; Zinc; Zinc-finger
SRR1271859_TR8465_c1_g1_i1_len=2849	RAP24_ARATH	Q:907-1719,H:54-329	51.53%ID	E:1e-58	Ethylene-responsive transcription factor RAP2-4	PF00847.17^AP2^AP2 domain^203-251^E:3.9e-14	0,031	0,863	0	3,156	0	1,928	0	1,805	0,104	-0,492	RAP24_ARATH	Ethylene-responsive transcription factor RAP2-4 (Ethylene-responsive transcription factor ERF059) (Protein RELATED TO APETALA2 4)	RAP2-4 ERF059 At1g78080 F28K19.29 T11I11.1	Arabidopsis thaliana (Mouse-ear cress)		Activator; Complete proteome; DNA-binding; Ethylene signaling pathway; Nucleus; Reference proteome; Transcription; Transcription regulation
SRR1271859_TR10808_c0_g1_i10_len=2365	PPH_ARATH	Q:2002-791,H:3-402	62.47%ID	E:5e-172	Pheophytinase, chloroplastic	PF00561.17^Abhydrolase_1^alpha/beta hydrolase fold^157-439^E:1.1e-13`PF12697.4^Abhydrolase_6^Alpha/beta hydrolase family^159-443^E:7.4e-18	0,502	2,452	0	8,648	0	5,716	0	6,627	1	-0,813	PPH_ARATH	Pheophytinase, chloroplastic (EC 3.1.1.-) (Pheophytin pheophorbide hydrolase) (Protein CO-REGULATED WITH NYE1)	PPH CRN1 At5g13800 MAC12.25	Arabidopsis thaliana (Mouse-ear cress)		Chlorophyll catabolism; Chloroplast; Complete proteome; Hydrolase; Membrane; Plastid; Reference proteome; Thylakoid; Transit peptide
SRR1271859_TR11304_c0_g1_i1_len=2270	CIPK5_ORYSJ	Q:1791-430,H:1-442	70.02%ID	E:0	CBL-interacting protein kinase 5	PF00069.22^Pkinase^Protein kinase domain^18-272^E:1.9e-73`PF07714.14^Pkinase_Tyr^Protein tyrosine kinase^19-268^E:5.7e-40`PF14531.3^Kinase-like^Kinase-like^111-260^E:3.5e-09`PF03822.11^NAF^NAF domain^329-388^E:5.5e-22	0,409	0,43	0	2,447	0	1,423	0	1,792	0,423	0,381	CIPK5_ORYSJ	CBL-interacting protein kinase 5 (EC 2.7.11.1) (OsCIPK05)	CIPK5 Os01g0206700 LOC_Os01g10890 OsJ_000797 OSJNBa0016I09.5 P0451C06.36	Oryza sativa subsp. japonica (Rice)		ATP-binding; Complete proteome; Kinase; Manganese; Nucleotide-binding; Reference proteome; Serine/threonine-protein kinase; Transferase
SRR1271859_TR12174_c0_g1_i2_len=1710	WRK18_ARATH	Q:440-568,H:174-216	88.37%ID	E:2e-33	WRKY transcription factor 18	.	0,044	2,607	0	4,819	0	3,19	0	3,819	0,08	-1,292	WRK18_ARATH	WRKY transcription factor 18 (WRKY DNA-binding protein 18) (AtWRKY18)	WRKY18 At4g31800 F28M20.10	Arabidopsis thaliana (Mouse-ear cress)		Alternative splicing; Complete proteome; DNA-binding; Nucleus; Reference proteome; Transcription; Transcription regulation
SRR1271859_TR12356_c0_g2_i1_len=584	MIRA_SYNDU	Q:44-379,H:103-216	59.13%ID	E:6e-37	Miraculin	PF00197.15^Kunitz_legume^Trypsin and protease inhibitor^2-108^E:1.3e-26	0,003	0,755	0	1,59	0	2,085	0	1,819	0	-1,733	MIRA_SYNDU	Miraculin (MIR)		Synsepalum dulcificum (Miracle fruit) (Richadella dulcifica)		Direct protein sequencing; Disulfide bond; Glycoprotein; Signal; Taste-modifying protein
SRR1271859_TR16070_c0_g1_i2_len=2864	BH013_ARATH	Q:363-2156,H:1-584	52.15%ID	E:0	Transcription factor bHLH13	PF14215.3^bHLH-MYC_N^bHLH-MYC and R2R3-MYB transcription factors N-terminal^52-240^E:1e-54`PF00010.23^HLH^Helix-loop-helix DNA-binding domain^437-483^E:3.4e-10	0,798	0,304	0	2,687	0	4,737	0	5,218	1	0,001	BH013_ARATH	Transcription factor bHLH13 (Basic helix-loop-helix protein 13) (AtbHLH13) (bHLH 13) (Transcription factor EN 39) (bHLH transcription factor bHLH013)	BHLH13 EN39 At1g01260 F6F3.7	Arabidopsis thaliana (Mouse-ear cress)		Complete proteome; DNA-binding; Nucleus; Reference proteome; Transcription; Transcription regulation
SRR1271859_TR17852_c0_g2_i1_len=1088	BH035_ARATH	Q:133-846,H:5-240	53.47%ID	E:5e-69	Transcription factor bHLH35	PF00010.23^HLH^Helix-loop-helix DNA-binding domain^95-137^E:1.1e-11	0,044	1,03	0	3,66	0	3,557	0	3,518	0,748	0,297	BH035_ARATH	Transcription factor bHLH35 (Basic helix-loop-helix protein 35) (AtbHLH35) (bHLH 35) (Transcription factor EN 41) (bHLH transcription factor bHLH035)	BHLH35 EN41 At5g57150 MUL3.10	Arabidopsis thaliana (Mouse-ear cress)		Alternative splicing; Complete proteome; DNA-binding; Nucleus; Reference proteome; Transcription; Transcription regulation
SRR1271859_TR19051_c0_g2_i1_len=938	NA	NA	NA	NA	NA	PF06200.11^tify^tify domain^77-107^E:6.3e-15`PF09425.7^CCT_2^Divergent CCT motif^143-162^E:2.3e-08	0,158	1,613	0	5,176	0	6,89	0	7,416	1	-1,531	.	.	.	.	.	.
SRR1271859_TR21346_c0_g1_i1_len=1347	ASPGB_ARATH	Q:1190-222,H:3-324	83.28%ID	E:0	Probable isoaspartyl peptidase/L-asparaginase 2	PF01112.15^Asparaginase_2^Asparaginase^5-326^E:7.2e-108	0,489	-0,503	0	5,637	0	5,856	0	7,562	0,025	-1,924	ASPGB_ARATH	Probable isoaspartyl peptidase/L-asparaginase 2 (EC 3.4.19.5) (L-asparagine amidohydrolase 2) [Cleaved into: Isoaspartyl peptidase/L-asparaginase 2 subunit alpha; Isoaspartyl peptidase/L-asparaginase 2 subunit beta]	At3g16150 MSL1.19	Arabidopsis thaliana (Mouse-ear cress)		Alternative splicing; Autocatalytic cleavage; Complete proteome; Hydrolase; Protease; Reference proteome
SRR1271859_TR27908_c1_g1_i1_len=945	HSP7C_PETHY	Q:944-168,H:360-618	95.37%ID	E:3e-152	Heat shock cognate 70 kDa protein	PF00012.17^HSP70^Hsp70 protein^2-259^E:1.2e-91	1	1,578	0	4,071	0	2,44	0	1,629	1	1,067	HSP7C_PETHY	Heat shock cognate 70 kDa protein	HSP70	Petunia hybrida (Petunia)		ATP-binding; Nucleotide-binding; Stress response
SRR1271859_TR28170_c0_g1_i1_len=965	NA	NA	NA	NA	NA	.	0,195	1,325	0	3,868	0	4,121	0	4,383	0,239	1,548	.	.	.	.	.	.
SRR1271859_TR28775_c1_g4_i1_len=1553	Y1500_ARATH	Q:394-840,H:53-201	64.67%ID	E:1e-62	Probable tyrosine-protein phosphatase At1g05000	PF03162.10^Y_phosphatase2^Tyrosine phosphatase family^15-169^E:2.2e-56`PF13350.3^Y_phosphatase3^Tyrosine phosphatase family^90-142^E:1.5e-06	0,719	-0,284	0	2,687	0	3,212	0	4,362	1	0,124	Y1500_ARATH	Probable tyrosine-protein phosphatase At1g05000 (EC 3.1.3.48)	At1g05000 T7A14.14	Arabidopsis thaliana (Mouse-ear cress)		3D-structure; Alternative splicing; Complete proteome; Hydrolase; Protein phosphatase; Reference proteome
SRR1271859_TR29245_c0_g2_i1_len=1178	GSTF_HYOMU	Q:187-900,H:2-212	61.34%ID	E:2e-98	Glutathione S-transferase	PF00043.22^GST_C^Glutathione S-transferase, C-terminal domain^48-141^E:7e-16`PF13410.3^GST_C_2^Glutathione S-transferase, C-terminal domain^69-135^E:9.3e-09`PF14497.3^GST_C_3^Glutathione S-transferase, C-terminal domain^75-142^E:2.1e-05	0,588	0,213	0	2,336	0	2,425	0	2,214	0,006	0,648	GSTF_HYOMU	Glutathione S-transferase (EC 2.5.1.18) (25 kDa auxin-binding protein) (GST class-phi)		Hyoscyamus muticus (Egyptian henbane)		Auxin signaling pathway; Direct protein sequencing; Transferase
SRR1271859_TR31061_c2_g2_i2_len=1457	NAC72_ARATH	Q:1455-853,H:84-242	53.92%ID	E:8e-54	NAC domain-containing protein 72	.	0,226	2,179	0	7,041	0	7,453	0	7,341	1	0,874	NAC72_ARATH	NAC domain-containing protein 72 (ANAC072) (Protein RESPONSIVE TO DESICCATION 26)	NAC072 RD26 At4g27410 F27G19.10	Arabidopsis thaliana (Mouse-ear cress)		Alternative splicing; Complete proteome; DNA-binding; Nucleus; Reference proteome; Transcription; Transcription regulation
SRR1271859_TR32557_c0_g1_i1_len=1232	IF4E1_MAIZE	Q:233-802,H:30-217	78.42%ID	E:9e-107	Eukaryotic translation initiation factor 4E-1	PF01652.15^IF4E^Eukaryotic initiation factor 4E^50-205^E:3.9e-53	0,959	0,033	0	1,408	0	1,585	0	1,611	0,323	0,296	IF4E1_MAIZE	Eukaryotic translation initiation factor 4E-1 (eIF-4E-1) (eIF4E-1) (eIF-4F 25 kDa subunit) (eIF-4F p26 subunit) (mRNA cap-binding protein)		Zea mays (Maize)		Complete proteome; Disulfide bond; Initiation factor; Protein biosynthesis; RNA-binding; Reference proteome; Translation regulation
SRR1271859_TR34460_c0_g1_i2_len=1465	SABP2_TOBAC	Q:399-893,H:5-169	47.13%ID	E:4e-38	Salicylic acid-binding protein 2	PF00561.17^Abhydrolase_1^alpha/beta hydrolase fold^105-213^E:2.1e-14`PF12146.5^Hydrolase_4^Serine aminopeptidase, S33^106-211^E:3.1e-12`PF12697.4^Abhydrolase_6^Alpha/beta hydrolase family^106-210^E:2.1e-10	1	-0,181	0	3,512	0	4,153	0	4,652	1	1,178	SABP2_TOBAC	Salicylic acid-binding protein 2 (NtSABP2) (EC 3.1.1.-) (Methyl salicylate esterase)	SABP2	Nicotiana tabacum (Common tobacco)		3D-structure; Hydrolase; Immunity; Innate immunity; Plant defense
SRR1271859_TR34460_c0_g1_i4_len=1440	MES9_ARATH	Q:399-1169,H:2-255	43.19%ID	E:2e-63	Methylesterase 9	PF00561.17^Abhydrolase_1^alpha/beta hydrolase fold^105-344^E:6.5e-15`PF12697.4^Abhydrolase_6^Alpha/beta hydrolase family^106-349^E:1.4e-16`PF12146.5^Hydrolase_4^Serine aminopeptidase, S33^106-215^E:2.4e-12	0,062	0,928	0	6,473	0	5,181	0	6,001	0,196	0,748	MES9_ARATH	Methylesterase 9 (AtMES9) (EC 3.1.1.-)	MES9 At4g37150 AP22.78 C7A10.210	Arabidopsis thaliana (Mouse-ear cress)		Complete proteome; Hydrolase; Immunity; Innate immunity; Plant defense; Reference proteome
SRR1271859_TR37173_c0_g1_i6_len=1610	JMT_ARATH	Q:286-1422,H:2-387	47.46%ID	E:3e-104	Jasmonate O-methyltransferase	PF03492.12^Methyltransf_7^SAM dependent carboxyl methyltransferase^43-256^E:9.8e-86	0,01	2,04	0	5,42	0	4,594	0	5,229	1	0,14	JMT_ARATH	Jasmonate O-methyltransferase (EC 2.1.1.141) (S-adenosyl-L-methionine:jasmonic acid carboxyl methyltransferase)	JMT At1g19640 F14P1.3	Arabidopsis thaliana (Mouse-ear cress)	PATHWAY: Lipid metabolism; oxylipin biosynthesis.	Complete proteome; Cytoplasm; Fatty acid biosynthesis; Fatty acid metabolism; Lipid biosynthesis; Lipid metabolism; Methyltransferase; Nucleus; Oxylipin biosynthesis; Reference proteome; S-adenosyl-L-methionine; Transferase
SRR1271859_TR38142_c0_g1_i3_len=2406	CIPKC_ARATH	Q:2081-735,H:5-459	81.54%ID	E:0	CBL-interacting serine/threonine-protein kinase 12	PF00069.22^Pkinase^Protein kinase domain^53-307^E:8.3e-77`PF07714.14^Pkinase_Tyr^Protein tyrosine kinase^54-303^E:2.3e-40`PF14531.3^Kinase-like^Kinase-like^147-295^E:1.6e-09`PF03822.11^NAF^NAF domain^359-415^E:6e-24	0,429	0,524	0	2,666	0	1,834	0	2,241	0,623	0,432	CIPKC_ARATH	CBL-interacting serine/threonine-protein kinase 12 (EC 2.7.11.1) (SNF1-related kinase 3.9) (SOS2-like protein kinase PKS8)	CIPK12 PKS8 SnRK3.9 At4g18700 F28A21.110	Arabidopsis thaliana (Mouse-ear cress)		ATP-binding; Complete proteome; Kinase; Manganese; Nucleotide-binding; Phosphoprotein; Reference proteome; Serine/threonine-protein kinase; Transferase
SRR1271859_TR39144_c0_g1_i5_len=1581	NAGK_DICDI	Q:1124-159,H:3-316	33.13%ID	E:3e-50	N-acetyl-D-glucosamine kinase	PF01869.17^BcrAD_BadFG^BadF/BadG/BcrA/BcrD ATPase family^30-345^E:3.5e-51	0,593	0,553	0	2,395	0	2,765	0	3,08	1	-0,458	NAGK_DICDI	N-acetyl-D-glucosamine kinase (N-acetylglucosamine kinase) (EC 2.7.1.59) (GlcNAc kinase)	nagk DDB_G0284433	Dictyostelium discoideum (Slime mold)		ATP-binding; Complete proteome; Kinase; Nucleotide-binding; Reference proteome; Transferase
SRR1271859_TR39719_c1_g1_i1_len=1323	HYES_RAT	Q:179-1096,H:245-542	33.44%ID	E:4e-49	Bifunctional epoxide hydrolase 2	PF07224.8^Chlorophyllase^Chlorophyllase^19-116^E:9.8e-05`PF12740.4^Chlorophyllase2^Chlorophyllase enzyme^20-129^E:2.2e-07`PF00561.17^Abhydrolase_1^alpha/beta hydrolase fold^25-158^E:1.6e-17`PF12146.5^Hydrolase_4^Serine aminopeptidase, S33^26-143^E:1.1e-11`PF12697.4^Abhydrolase_6^Alpha/beta hydrolase family^27-311^E:9.9e-21	0,16	0,612	0	2,866	0	1,584	0	1,232	0,679	0,223	HYES_RAT	Bifunctional epoxide hydrolase 2 [Includes: Cytosolic epoxide hydrolase 2 (CEH) (EC 3.3.2.10) (Epoxide hydratase) (Soluble epoxide hydrolase) (SEH); Lipid-phosphate phosphatase (EC 3.1.3.76)]	Ephx2	Rattus norvegicus (Rat)		Acetylation; Aromatic hydrocarbons catabolism; Complete proteome; Cytoplasm; Detoxification; Direct protein sequencing; Hydrolase; Lipid metabolism; Lipoprotein; Magnesium; Metal-binding; Multifunctional enzyme; Peroxisome; Phosphoprotein; Reference proteome
SRR1271859_TR41321_c0_g1_i1_len=1998	C94C1_ARATH	Q:1754-378,H:31-494	60%ID	E:0	Cytochrome P450 94C1	PF00067.19^p450^Cytochrome P450^121-522^E:6.9e-58	0,001	1,713	0	3,425	0	4,029	0	4,1	0,352	-0,978	C94C1_ARATH	Cytochrome P450 94C1 (EC 1.14.-.-)	CYP94C1 At2g27690 F15K20.21	Arabidopsis thaliana (Mouse-ear cress)		Complete proteome; Heme; Iron; Jasmonic acid signaling pathway; Membrane; Metal-binding; Monooxygenase; Oxidoreductase; Reference proteome; Transmembrane; Transmembrane helix
SRR1271859_TR41420_c0_g1_i1_len=2164	LIP_STAHY	Q:1435-602,H:309-570	30.41%ID	E:3e-26	Lipase	.	0,002	1,836	0	3,943	0	2,254	0	2,352	0,62	-0,547	LIP_STAHY	Lipase (EC 3.1.1.3) (EC 3.1.1.32) (Phospholipase A1) (Triacylglycerol lipase) [Cleaved into: Lipase 86 kDa form; Lipase 46 kDa form]	lip	Staphylococcus hyicus		3D-structure; Calcium; Direct protein sequencing; Hydrolase; Lipid degradation; Lipid metabolism; Metal-binding; Secreted; Signal
SRR1271859_TR41710_c0_g1_i3_len=1393	SGRW_PEA	Q:230-952,H:1-236	70.66%ID	E:6e-116	Protein STAY-GREEN, chloroplastic	PF12638.4^Staygreen^Staygreen protein^54-205^E:3.3e-58	0,189	2,074	0	4,901	0	5,451	0	5,636	1	1,198	SGRW_PEA	Protein STAY-GREEN, chloroplastic (Protein STAYGREEN)	SGR	Pisum sativum (Garden pea)		Chloroplast; Plastid; Transit peptide
SRR1271859_TR43882_c0_g2_i1_len=2471	PTR8_ARATH	Q:1249-2139,H:288-581	53.36%ID	E:7e-107	Protein NRT1/ PTR FAMILY 2.9	PF00854.18^PTR2^POT family^128-326^E:2.1e-34	0,003	1,186	0	3,09	0	1,838	0	1,399	0,675	0,31	PTR8_ARATH	Protein NRT1/ PTR FAMILY 2.9 (AtNPF2.9) (Nitrate transporter 1.9)	NPF2.9 NRT1.9 At1g18880 F6A14.2	Arabidopsis thaliana (Mouse-ear cress)		Cell membrane; Complete proteome; Membrane; Reference proteome; Transmembrane; Transmembrane helix; Transport
SRR1271859_TR44464_c0_g1_i1_len=3383	SUS2_PEA	Q:2961-541,H:1-807	82.4%ID	E:0	Sucrose synthase 2	PF00862.16^Sucrose_synth^Sucrose synthase^10-556^E:1.1e-292`PF00534.17^Glycos_transf_1^Glycosyl transferases group 1^561-744^E:1.7e-32`PF13692.3^Glyco_trans_1_4^Glycosyl transferases group 1^574-727^E:2.1e-10	0,537	0,291	0	2,164	0	2,021	0	1,661	0,152	0,863	SUS2_PEA	Sucrose synthase 2 (EC 2.4.1.13) (Sucrose-UDP glucosyltransferase 2)	SUS2	Pisum sativum (Garden pea)		Glycosyltransferase; Transferase
SRR1271859_TR46848_c0_g2_i4_len=2239	NA	NA	NA	NA	NA	PF04199.10^Cyclase^Putative cyclase^60-213^E:1.4e-19	0,306	0,565	0	1,937	0	3,894	0	2,763	0,798	0,376	.	.	.	.	.	.
SRR1271859_TR46937_c0_g1_i3_len=1732	WRKY6_ARATH	Q:400-1233,H:204-498	60.93%ID	E:1e-81	WRKY transcription factor 6	PF03106.12^WRKY^WRKY DNA -binding domain^158-215^E:6.1e-27	0,118	1,762	0	6,914	0	4,02	0	4,521	0,732	-0,455	WRKY6_ARATH	WRKY transcription factor 6 (WRKY DNA-binding protein 6) (AtWRKY6)	WRKY6 At1g62300 F19K23.22	Arabidopsis thaliana (Mouse-ear cress)		Activator; Complete proteome; DNA-binding; Ethylene signaling pathway; Nucleus; Reference proteome; Repressor; Transcription; Transcription regulation
SRR342017_TR2816_c0_g1_i1_len=1302	PPCK1_ARATH	Q:1163-360,H:13-279	57.41%ID	E:1e-106	Phosphoenolpyruvate carboxylase kinase 1	PF00069.22^Pkinase^Protein kinase domain^10-268^E:5.6e-70`PF07714.14^Pkinase_Tyr^Protein tyrosine kinase^10-263^E:9e-29`PF14531.3^Kinase-like^Kinase-like^44-217^E:9.2e-10	0,077	0,662	0	2,033	0	2,702	0	2,988	0,204	0,552	PPCK1_ARATH	Phosphoenolpyruvate carboxylase kinase 1 (AtPPCK1) (EC 2.7.11.1)	PPCK1 At1g08650 F22O13.13	Arabidopsis thaliana (Mouse-ear cress)		ATP-binding; Alternative splicing; Complete proteome; Kinase; Nucleotide-binding; Pyruvate; Reference proteome; Serine/threonine-protein kinase; Transferase
SRR342017_TR3714_c0_g1_i2_len=2246	IAA26_ARATH	Q:1052-1657,H:60-261	51.63%ID	E:7e-52	Auxin-responsive protein IAA26	PF02309.13^AUX_IAA^AUX/IAA family^43-322^E:5.1e-56	0,832	0,311	0	3,89	0	3,87	0	4,569	0,299	1,209	IAA26_ARATH	Auxin-responsive protein IAA26 (Indoleacetic acid-induced protein 26) (Phytochrome-associated protein 1)	IAA26 PAP1 At3g16500 MDC8.13	Arabidopsis thaliana (Mouse-ear cress)		Auxin signaling pathway; Complete proteome; Nucleus; Reference proteome; Repressor; Transcription; Transcription regulation
SRR342017_TR5017_c0_g2_i5_len=1434	MIRA_SYNDU	Q:143-682,H:31-216	50.53%ID	E:3e-46	Miraculin	PF00197.15^Kunitz_legume^Trypsin and protease inhibitor^53-227^E:6.9e-42	0,001	0,84	0	2,057	0	2,022	0	1,92	0,005	-1,158	MIRA_SYNDU	Miraculin (MIR)		Synsepalum dulcificum (Miracle fruit) (Richadella dulcifica)		Direct protein sequencing; Disulfide bond; Glycoprotein; Signal; Taste-modifying protein
SRR342017_TR8814_c0_g1_i1_len=4768	ALDO4_ARATH	Q:496-4563,H:2-1333	58.24%ID	E:0	Benzaldehyde dehydrogenase (NAD(+))	PF00111.24^Fer2^2Fe-2S iron-sulfur cluster binding domain^23-90^E:2.6e-07`PF01799.17^Fer2_2^[2Fe-2S] binding domain^101-187^E:2.7e-22`PF00941.18^FAD_binding_5^FAD binding domain in molybdopterin dehydrogenase^244-423^E:3.8e-39`PF03450.14^CO_deh_flav_C^CO dehydrogenase flavoprotein C-terminal domain^444-546^E:1.3e-21`PF01315.19^Ald_Xan_dh_C^Aldehyde oxidase and xanthine dehydrogenase, a/b hammerhead domain^620-726^E:1.4e-24`PF02738.15^Ald_Xan_dh_C2^Molybdopterin-binding domain of aldehyde dehydrogenase^756-1277^E:5.8e-158	0,115	0,536	0	1,647	0	1,515	0,001	1,488	0,37	0,417	ALDO4_ARATH	Benzaldehyde dehydrogenase (NAD(+)) (EC 1.2.1.28) (Aldehyde oxidase 4) (AO-4) (AtAO-4) (AtAO2) (Indole-3-acetaldehyde oxidase) (IAA oxidase) (EC 1.2.3.7)	AAO4 AO2 At1g04580 T1G11.17	Arabidopsis thaliana (Mouse-ear cress)		2Fe-2S; Abscisic acid biosynthesis; Auxin biosynthesis; Complete proteome; Cytoplasm; FAD; Flavoprotein; Iron; Iron-sulfur; Metal-binding; Molybdenum; NAD; Oxidoreductase; Reference proteome
SRR342017_TR19436_c1_g5_i2_len=967	GILT_HUMAN	Q:584-36,H:62-243	37.23%ID	E:1e-29	Gamma-interferon-inducible lysosomal thiol reductase	PF03227.13^GILT^Gamma interferon inducible lysosomal thiol reductase (GILT)^41-143^E:3e-29	0,105	-1	0	7,226	0	8,307	0	7,927	1	0	GILT_HUMAN	Gamma-interferon-inducible lysosomal thiol reductase (EC 1.8.-.-) (Gamma-interferon-inducible protein IP-30) (Legumaturain)	IFI30 GILT IP30	Homo sapiens (Human)		Complete proteome; Direct protein sequencing; Disulfide bond; Glycoprotein; Immunity; Lysosome; Oxidoreductase; Redox-active center; Reference proteome; Secreted; Signal
SRR342017_TR25891_c0_g1_i6_len=1583	NA	NA	NA	NA	NA	PF07712.9^SURNod19^Stress up-regulated Nod 19^22-365^E:1.7e-169	0,016	1,265	0	3,982	0	2,799	0	2,864	0,666	0,269	.	.	.	.	.	.
SRR342017_TR27234_c0_g1_i1_len=2294	PME40_ARATH	Q:523-1962,H:37-518	55.56%ID	E:0	Probable pectinesterase/pectinesterase inhibitor 40	PF04043.12^PMEI^Plant invertase/pectin methylesterase inhibitor^63-210^E:1e-26`PF01095.16^Pectinesterase^Pectinesterase^258-555^E:1.2e-144	0,275	0,455	0	4,223	0	2,457	0	3,527	0,832	-0,328	PME40_ARATH	Probable pectinesterase/pectinesterase inhibitor 40 [Includes: Pectinesterase inhibitor 40 (Pectin methylesterase inhibitor 40); Pectinesterase 40 (PE 40) (EC 3.1.1.11) (Pectin methylesterase 40) (AtPME40)]	PME40 ARATH40 At4g02320 T14P8.1	Arabidopsis thaliana (Mouse-ear cress)	PATHWAY: Glycan metabolism; pectin degradation; 2-dehydro-3-deoxy-D-gluconate from pectin: step 1/5.	Aspartyl esterase; Cell wall; Cell wall biogenesis/degradation; Complete proteome; Disulfide bond; Glycoprotein; Hydrolase; Reference proteome; Secreted; Signal
SRR342019_TR2072_c0_g1_i7_len=1858	TI10A_ARATH	Q:1316-687,H:4-219	39.74%ID	E:1e-30	Protein TIFY 10A	PF06200.11^tify^tify domain^105-137^E:3.1e-19`PF09425.7^CCT_2^Divergent CCT motif^192-209^E:7.1e-08	0,003	3,129	0	4,956	0	2,747	0	2,362	1	0,274	TI10A_ARATH	Protein TIFY 10A (Jasmonate ZIM domain-containing protein 1)	TIFY10A JAZ1 At1g19180 T29M8.5	Arabidopsis thaliana (Mouse-ear cress)		3D-structure; Alternative splicing; Complete proteome; Jasmonic acid signaling pathway; Nucleus; Plant defense; Reference proteome; Transcription; Transcription regulation; Ubl conjugation
SRR342019_TR2192_c0_g1_i1_len=1163	NA	NA	NA	NA	NA	.	0,118	2,274	0	4,084	0	5,027	0	5,546	1	0,107	.	.	.	.	.	.
SRR342019_TR2192_c0_g2_i2_len=1054	IAA26_ARATH	Q:1020-307,H:60-267	48.8%ID	E:4e-60	Auxin-responsive protein IAA26	PF02309.13^AUX_IAA^AUX/IAA family^20-142^E:4.7e-34	0,732	0,555	0	3,109	0	4,361	0	4,952	1	0,328	IAA26_ARATH	Auxin-responsive protein IAA26 (Indoleacetic acid-induced protein 26) (Phytochrome-associated protein 1)	IAA26 PAP1 At3g16500 MDC8.13	Arabidopsis thaliana (Mouse-ear cress)		Auxin signaling pathway; Complete proteome; Nucleus; Reference proteome; Repressor; Transcription; Transcription regulation
SRR342019_TR2738_c0_g1_i1_len=1423	ACMAT_VITLA	Q:1301-873,H:10-150	62.07%ID	E:1e-47	Methanol O-anthraniloyltransferase	PF02458.12^Transferase^Transferase family^6-147^E:1.4e-23	1	-0,052	0	3,048	0	5,159	0	5,419	0,732	0,682	ACMAT_VITLA	Methanol O-anthraniloyltransferase (EC 2.3.1.232) (Anthraniloyl-CoA:methanol acyltransferase) (Benzyl alcohol O-benzoyltransferase) (EC 2.3.1.196)	AMAT	Vitis labrusca (Concord grape)		Acyltransferase; Direct protein sequencing; Transferase
SRR342019_TR2870_c0_g7_i15_len=3527	AFS1_MALDO	Q:2531-3382,H:274-559	54.7%ID	E:2e-95	(E,E)-alpha-farnesene synthase	PF03936.13^Terpene_synth_C^Terpene synthase family, metal binding domain^1-158^E:6.8e-43	1	0,494	0	5,248	0	5,755	0	6,206	1	1,017	AFS1_MALDO	(E,E)-alpha-farnesene synthase (MdAFS1) (EC 4.2.3.46) (Prenyltransferase) (EC 2.5.1.-)	AFS1 AFAR	Malus domestica (Apple) (Pyrus malus)		Cytoplasm; Lyase; Magnesium; Manganese; Metal-binding; Potassium; Transferase
SRR342019_TR3398_c0_g1_i2_len=810	GSTUI_ARATH	Q:699-133,H:38-227	68.95%ID	E:9e-87	Glutathione S-transferase U18	PF13417.3^GST_N_3^Glutathione S-transferase, N-terminal domain^4-47^E:1.8e-07`PF02798.17^GST_N^Glutathione S-transferase, N-terminal domain^11-47^E:8.8e-10`PF00043.22^GST_C^Glutathione S-transferase, C-terminal domain^68-169^E:5.3e-08	0,75	-0,108	0	3,082	0	1,298	0	1,331	0,319	-1,115	GSTUI_ARATH	Glutathione S-transferase U18 (AtGSTU18) (EC 2.5.1.18) (GST class-tau member 18) (Glutathione S-transferase 29)	GSTU18 GST29 At1g10360 F14N23.24	Arabidopsis thaliana (Mouse-ear cress)		Complete proteome; Cytoplasm; Detoxification; Reference proteome; Transferase
SRR342019_TR5096_c2_g6_i2_len=878	NA	NA	NA	NA	NA	PF14368.3^LTP_2^Probable lipid transfer^13-104^E:2.2e-19	0,481	0,186	0	2,871	0	1,939	0	1,94	1	-1,56	.	.	.	.	.	.
SRR342019_TR7445_c2_g3_i2_len=1912	SPEA_BACSU	Q:282-1715,H:7-474	35.14%ID	E:5e-82	Arginine decarboxylase	PF01276.17^OKR_DC_1^Orn/Lys/Arg decarboxylase, major domain^63-430^E:1.5e-65`PF00155.18^Aminotran_1_2^Aminotransferase class I and II^125-261^E:2e-10`PF00266.16^Aminotran_5^Aminotransferase class-V^125-289^E:2.2e-08`PF01212.18^Beta_elim_lyase^Beta-eliminating lyase^126-258^E:6.8e-05`PF01053.17^Cys_Met_Meta_PP^Cys/Met metabolism PLP-dependent enzyme^128-290^E:2.3e-05`PF01041.14^DegT_DnrJ_EryC1^DegT/DnrJ/EryC1/StrS aminotransferase family^138-264^E:9.4e-09`PF05889.10^SepSecS^O-phosphoseryl-tRNA(Sec) selenium transferase, SepSecS^160-288^E:1.1e-06`PF03711.12^OKR_DC_1_C^Orn/Lys/Arg decarboxylase, C-terminal domain^471-533^E:1.4e-08	0,21	-0,352	0	4,838	0	6,487	0	7,839	0,842	0,101	SPEA_BACSU	Arginine decarboxylase (EC 4.1.1.19)	speA cad BSU14630	Bacillus subtilis (strain 168)	PATHWAY: Amine and polyamine biosynthesis; agmatine biosynthesis; agmatine from L-arginine: step 1/1.	Complete proteome; Cytoplasm; Decarboxylase; Lyase; Polyamine biosynthesis; Putrescine biosynthesis; Pyridoxal phosphate; Reference proteome; Spermidine biosynthesis
SRR342019_TR9202_c0_g3_i1_len=330	NA	NA	NA	NA	NA	.	0,732	0,608	0	4,921	0	1,992	0	1,559	0,129	-2,81	.	.	.	.	.	.
SRR342019_TR11375_c0_g1_i2_len=1989	NA	NA	NA	NA	NA	PF05542.8^DUF760^Protein of unknown function (DUF760)^87-165^E:1.2e-[illegible]	0,594	0,254	0	2,475	0	1,383	0	2,158	0,123	0,722	[illegible]	[illegible]	[illegible]	[illegible]	[illegible]	[illegible]

[illegible]	NA	NA	NA	NA	NA	17`PF05542.8^DUF760^Protein of unknown function (DUF760)^260-345^E:2.7e-14	[illegible]	[illegible]	[illegible]	[illegible]	[illegible]	[illegible]	[illegible]	[illegible]	[illegible]	[illegible]	.	.	.	.	.	.
SRR342019_TR12005_c1_g2_i2_len=1751	TRPB_CAMAC	Q:1292-72,H:85-456	60.24%ID	E:4e-174	Tryptophan synthase beta chain 2, chloroplastic	PF00291.22^PALP^Pyridoxal-phosphate dependent enzyme^154-438^E:1.7e-40	0,254	3,585	0	9,378	0	6,809	0	7,882	1	-0,345	TRPB_CAMAC	Tryptophan synthase beta chain 2, chloroplastic (EC 4.2.1.20)	TSB	Camptotheca acuminata (Happy tree)	PATHWAY: Amino-acid biosynthesis; L-tryptophan biosynthesis; L-tryptophan from chorismate: step 5/5.	Amino-acid biosynthesis; Aromatic amino acid biosynthesis; Chloroplast; Lyase; Plastid; Pyridoxal phosphate; Transit peptide; Tryptophan biosynthesis
SRR342019_TR13660_c2_g2_i11_len=1714	B2_DAUCA	Q:918-466,H:55-205	92.05%ID	E:1e-97	B2 protein	PF10539.6^Dev_Cell_Death^Development and cell death domain^199-326^E:2.2e-41	0,317	0,542	0	2,036	0	1,744	0	1,897	0,334	0,31	B2_DAUCA	B2 protein		Daucus carota (Wild carrot)		
SRR342019_TR14141_c0_g4_i2_len=1188	PTR53_ARATH	Q:1004-6,H:27-359	56.59%ID	E:9e-134	Protein NRT1/ PTR FAMILY 2.11	PF00854.18^PTR2^POT family^95-338^E:3e-38	0,517	1,151	0	4,775	0	5,14	0	5,588	0,381	1,424	PTR53_ARATH	Protein NRT1/ PTR FAMILY 2.11 (AtNPF2.11) (Nitrate transporter 1.10) (Protein GLUCOSINOLATE TRANSPORTER-2)	NPF2.11 GTR2 NRT1.10 At5g62680 MRG21.10	Arabidopsis thaliana (Mouse-ear cress)		Cell membrane; Complete proteome; Membrane; Reference proteome; Transmembrane; Transmembrane helix; Transport
SRR342019_TR15113_c3_g1_i4_len=2062	PRS2_SOLTU	Q:1925-1470,H:1-155	35.67%ID	E:5e-22	Pathogenesis-related protein STH-2	PF00407.16^Bet_v_1^Pathogenesis-related protein Bet v I family^1-147^E:4e-13	0,316	0,678	0	1,905	0	1,76	0	1,456	1	0	PRS2_SOLTU	Pathogenesis-related protein STH-2	STH-2	Solanum tuberosum (Potato)		Complete proteome; Pathogenesis-related protein; Plant defense; Reference proteome
SRR342019_TR16511_c1_g3_i1_len=1606	TIP13_ARATH	Q:102-857,H:1-252	79.76%ID	E:2e-139	Aquaporin TIP1-3	PF00230.17^MIP^Major intrinsic protein^14-234^E:6.5e-74	0,005	6,253	0	5,759	0	4,293	0	2,543	1	0,197	TIP13_ARATH	Aquaporin TIP1-3 (Gamma-tonoplast intrinsic protein 3) (Gamma-TIP3) (Tonoplast intrinsic protein 1-3) (AtTIP1;3) [Cleaved into: Aquaporin TIP1-3, N-terminally processed]	TIP1-3 At4g01470 F11O4.1	Arabidopsis thaliana (Mouse-ear cress)		Acetylation; Complete proteome; Membrane; Reference proteome; Repeat; Stress response; Transmembrane; Transmembrane helix; Transport; Vacuole
SRR342019_TR22279_c1_g4_i2_len=1337	GSTF_HYOMU	Q:910-272,H:2-212	68.54%ID	E:3e-103	Glutathione S-transferase	PF02798.17^GST_N^Glutathione S-transferase, N-terminal domain^8-79^E:1.7e-15`PF13417.3^GST_N_3^Glutathione S-transferase, N-terminal domain^10-85^E:6.5e-13`PF13409.3^GST_N_2^Glutathione S-transferase, N-terminal domain^14-78^E:1.6e-05`PF00043.22^GST_C^Glutathione S-transferase, C-terminal domain^114-207^E:1.8e-15`PF13410.3^GST_C_2^Glutathione S-transferase, C-terminal domain^135-201^E:2e-08`PF14497.3^GST_C_3^Glutathione S-transferase, C-terminal domain^141-208^E:5.4e-05	0,009	0,614	0	3,141	0	2,53	0	2,086	0,071	0,822	GSTF_HYOMU	Glutathione S-transferase (EC 2.5.1.18) (25 kDa auxin-binding protein) (GST class-phi)		Hyoscyamus muticus (Egyptian henbane)		Auxin signaling pathway; Direct protein sequencing; Transferase
SRR342019_TR23181_c0_g1_i1_len=4456	GLR34_ARATH	Q:1032-3785,H:3-932	63.25%ID	E:0	Glutamate receptor 3.4	PF13458.3^Peripla_BP_6^Periplasmic binding protein^53-412^E:3e-22`PF01094.25^ANF_receptor^Receptor family ligand binding region^60-417^E:2e-82`PF10613.6^Lig_chan-Glu_bd^Ligated ion channel L-glutamate- and glycine-binding site^483-581^E:1.3e-10`PF00497.17^SBP_bac_3^Bacterial extracellular solute-binding proteins, family 3^491-820^E:1.8e-21`PF00060.23^Lig_chan^Ligand-gated ion channel^598-852^E:3.2e-36	0,012	1,294	0	2,615	0	2,567	0	2,834	1	-0,122	GLR34_ARATH	Glutamate receptor 3.4 (AtGLR4) (Ligand-gated ion channel 3.4)	GLR3.4 GLR4 GLUR3 At1g05200 YUP8H12.19	Arabidopsis thaliana (Mouse-ear cress)		Alternative splicing; Cell membrane; Chloroplast; Complete proteome; Glycoprotein; Ion channel; Ion transport; Ligand-gated ion channel; Membrane; Plastid; Receptor; Reference proteome; Signal; Stress response; Transmembrane; Transmembrane helix; Transport
SRR342019_TR23718_c0_g2_i1_len=2559	AIM1_ARATH	Q:2329-212,H:7-712	74.65%ID	E:0	Peroxisomal fatty acid beta-oxidation multifunctional protein AIM1	PF00378.17^ECH_1^Enoyl-CoA hydratase/isomerase^84-285^E:5.8e-37`PF16113.2^ECH_2^Enoyl-CoA hydratase/isomerase^89-270^E:6.9e-20`PF02737.15^3HCDH_N^3-hydroxyacyl-CoA dehydrogenase, NAD binding domain^381-559^E:8.4e-58`PF00725.19^3HCDH^3-hydroxyacyl-CoA dehydrogenase, C-terminal domain^562-655^E:3.8e-19`PF00725.19^3HCDH^3-hydroxyacyl-CoA dehydrogenase, C-terminal domain^694-774^E:0.00023	0,135	0,614	0	3,914	0,001	1,591	0	1,718	1	-0,229	AIM1_ARATH	Peroxisomal fatty acid beta-oxidation multifunctional protein AIM1 (Protein ABNORMAL INFLORESCENCE MERISTEM 1) (AtAIM1) [Includes: Enoyl-CoA hydratase/3-2-trans-enoyl-CoA isomerase/3-hydroxybutyryl-CoA epimerase (EC 4.2.1.17) (EC 5.1.2.3) (EC 5.3.3.8); 3-hydroxyacyl-CoA dehydrogenase (EC 1.1.1.35)]	AIM1 At4g29010 F19B15.40	Arabidopsis thaliana (Mouse-ear cress)	PATHWAY: Lipid metabolism; fatty acid beta-oxidation.	Complete proteome; Fatty acid metabolism; Isomerase; Lipid metabolism; Lyase; Multifunctional enzyme; NAD; Oxidoreductase; Peroxisome; Reference proteome
SRR342019_TR27096_c0_g1_i1_len=1477	DBR_TOBAC	Q:259-1290,H:1-343	82.56%ID	E:0	2-alkenal reductase (NADP(+)-dependent)	PF16884.2^ADH_N_2^N-terminal domain of oxidoreductase^8-116^E:1.6e-26`PF00107.23^ADH_zinc_N^Zinc-binding dehydrogenase^166-293^E:1.6e-24`PF13602.3^ADH_zinc_N_2^Zinc-binding dehydrogenase^200-337^E:1.7e-07	0,745	0,112	0	1,188	0	0,917	0,001	0,88	0,458	0,234	DBR_TOBAC	2-alkenal reductase (NADP(+)-dependent) (EC 1.3.1.102) (Alkenal double bound reductase) (Allyl-alcohol dehydrogenase) (EC 1.1.1.54) (Allylic alcohol dehydrogenase 1) (allyl-ADH1) (Flavin-free double bond reductase) (NtDBR) (Pulegone reductase) (NtRed-1)	DBR	Nicotiana tabacum (Common tobacco)		3D-structure; Direct protein sequencing; NADP; Oxidoreductase
SRR342019_TR32476_c0_g1_i1_len=1833	UFOG6_FRAAN	Q:1610-198,H:3-478	52.99%ID	E:8e-176	UDP-glucose flavonoid 3-O-glucosyltransferase 6 {ECO:0000303|PubMed:18487633}	PF00201.15^UDPGT^UDP-glucoronosyl and UDP-glucosyl transferase^260-397^E:1.3e-21	0,38	0,556	0	2,736	0	0,933	0	1,339	0,239	1,104	UFOG6_FRAAN	UDP-glucose flavonoid 3-O-glucosyltransferase 6 (EC 2.4.1.91) (Flavonol 3-O-glucosyltransferase 6) (FaGT6)	GT6	Fragaria ananassa (Strawberry)		Glycosyltransferase; Transferase
SRR342019_TR36177_c0_g4_i1_len=741	NA	NA	NA	NA	NA	PF13833.3^EF-hand_8^EF-hand domain pair^21-45^E:0.031`PF00036.29^EF-hand_1^EF hand^23-50^E:4.7e-07`PF13405.3^EF-hand_6^EF-hand domain^23-50^E:8.9e-07`PF13202.3^EF-hand_5^EF hand^24-46^E:2.1e-06`PF13202.3^EF-hand_5^EF hand^64-83^E:0.012	0,286	0,899	0	2,664	0	2,514	0	2,654	0,212	-1,069	.	.	.	.	.	.
SRR342019_TR39103_c0_g2_i1_len=1363	SRG1_ARATH	Q:1291-347,H:17-333	38.51%ID	E:2e-68	Protein SRG1	PF14226.3^DIOX_N^non-haem dioxygenase in morphine synthesis N-terminal^52-158^E:4.3e-24`PF03171.17^2OG-FeII_Oxy^2OG-Fe(II) oxygenase superfamily^206-303^E:5.5e-28	0,254	3,607	0	6,024	0	7,465	0	5,217	1	-1,033	SRG1_ARATH	Protein SRG1 (AtSRG1) (Protein SENESCENCE-RELATED GENE 1)	SRG1 At1g17020 F20D23.28 F6I1.30	Arabidopsis thaliana (Mouse-ear cress)		Complete proteome; Iron; Metal-binding; Oxidoreductase; Reference proteome
SRR342022_TR1215_c4_g6_i6_len=760	PRS2_SOLTU	Q:671-204,H:1-155	36.88%ID	E:2e-25	Pathogenesis-related protein STH-2	PF00407.16^Bet_v_1^Pathogenesis-related protein Bet v I family^30-179^E:1.1e-11	0,3	0,239	0	3,053	0	1,465	0	1,496	1	0,073	PRS2_SOLTU	Pathogenesis-related protein STH-2	STH-2	Solanum tuberosum (Potato)		Complete proteome; Pathogenesis-related protein; Plant defense; Reference proteome
SRR342022_TR2129_c0_g2_i2_len=1572	PLA17_ARATH	Q:311-1387,H:148-514	69.65%ID	E:0	Phospholipase A1-Igamma3, chloroplastic	PF01764.22^Lipase_3^Lipase (class 3)^103-259^E:1.2e-39	0,017	1,238	0	3,572	0	2,511	0	2,891	0,107	0,798	PLA17_ARATH	Phospholipase A1-Igamma3, chloroplastic (EC 3.1.1.-)	At1g51440 F5D21.19	Arabidopsis thaliana (Mouse-ear cress)		Chloroplast; Complete proteome; Hydrolase; Lipid degradation; Lipid metabolism; Plastid; Reference proteome; Transit peptide
SRR342022_TR2238_c7_g1_i2_len=2722	SABP2_TOBAC	Q:1032-262,H:5-259	44.15%ID	E:6e-61	Salicylic acid-binding protein 2	PF12146.5^Hydrolase_4^Serine aminopeptidase, S33^147-270^E:1.5e-07`PF00561.17^Abhydrolase_1^alpha/beta hydrolase fold^150-260^E:1.4e-10`PF12697.4^Abhydrolase_6^Alpha/beta hydrolase family^151-225^E:6.2e-06	0,732	0,897	0	1,548	0	1,274	0	1,982	1	1,354	SABP2_TOBAC	Salicylic acid-binding protein 2 (NtSABP2) (EC 3.1.1.-) (Methyl salicylate esterase)	SABP2	Nicotiana tabacum (Common tobacco)		3D-structure; Hydrolase; Immunity; Innate immunity; Plant defense
SRR342022_TR2984_c0_g5_i6_len=3797	DOX1_ARATH	Q:58-1767,H:1-565	71.28%ID	E:0	Alpha-dioxygenase 1	PF02403.19^Seryl_tRNA_N^Seryl-tRNA synthetase N-terminal domain^1-112^E:1.1e-26`PF00587.22^tRNA-synt_2b^tRNA synthetase class II core domain (G, H, P, S and T)^221-407^E:1.6e-35	0,76	0,566	0	3,896	0	5,33	0	6,15	0,517	-0,806	DOX1_ARATH	Alpha-dioxygenase 1 (Alpha DOX1) (EC 1.14.99.-) (Fatty acid dioxygenase AlphaDOX1) (Pathogen-induced oxygenase) (Plant alpha dioxygenase 1)	DOX1 DIOX1 PADOX-1 PIOX At3g01420 T13O15.6	Arabidopsis thaliana (Mouse-ear cress)		3D-structure; Complete proteome; Dioxygenase; Fatty acid biosynthesis; Fatty acid metabolism; Glycoprotein; Hypersensitive response; Iron; Lipid biosynthesis; Lipid metabolism; Metal-binding; Oxidoreductase; Oxylipin biosynthesis; Peroxidase; Plant defense; Reference proteome; Signal
SRR342022_TR6106_c0_g1_i1_len=509	NA	NA	NA	NA	NA	.	0,299	1,615	0	7,181	0	4,567	0	5,256	1	3,206	.	.	.	.	.	.
SRR342022_TR12801_c0_g3_i13_len=1371	MTDH_FRAAN	Q:1270-248,H:15-355	64.52%ID	E:2e-146	Probable mannitol dehydrogenase	PF08240.9^ADH_N^Alcohol dehydrogenase GroES-like domain^5-90^E:1.3e-15`PF00107.23^ADH_zinc_N^Zinc-binding dehydrogenase^134-257^E:2.2e-16	0,002	1,722	0	4,416	0	3,664	0	2,475	1	-0,015	MTDH_FRAAN	Probable mannitol dehydrogenase (EC 1.1.1.255) (NAD-dependent mannitol dehydrogenase)	CAD	Fragaria ananassa (Strawberry)		Metal-binding; NAD; Oxidoreductase; Zinc
SRR342022_TR13754_c0_g1_i2_len=952	RAE1C_ARATH	Q:263-868,H:15-216	93.07%ID	E:1e-118	Ras-related protein RABE1c	PF00025.18^Arf^ADP-ribosylation factor family^16-163^E:2.1e-15`PF00071.19^Ras^Ras family^17-178^E:1.6e-66`PF08477.10^Roc^Ras of Complex, Roc, domain of DAPkinase^17-132^E:4.6e-35`PF04670.9^Gtr1_RagA^Gtr1/RagA G protein conserved region^17-132^E:4.5e-05`PF01926.20^MMR_HSR1^50S ribosome-binding GTPase^18-128^E:4.7e-05	0,086	-0,97	0	7,651	0	7,822	0	7,548	0,049	1,391	RAE1C_ARATH	Ras-related protein RABE1c (AtRABE1c) (Ras-related protein Ara-3) (Ras-related protein Rab8A) (AtRab8A)	RABE1C ARA-3 RAB8A At3g46060 F12M12.30	Arabidopsis thaliana (Mouse-ear cress)		Cell membrane; Complete proteome; GTP-binding; Golgi apparatus; Lipoprotein; Membrane; Nucleotide-binding; Prenylation; Protein transport; Reference proteome; Transport
SRR342022_TR13846_c1_g2_i2_len=1683	FRI1_ARATH	Q:1545-790,H:6-255	65.08%ID	E:1e-96	Ferritin-1, chloroplastic	PF00210.21^Ferritin^Ferritin-like domain^95-238^E:6.1e-32	0,443	0,241	0	2,078	0	1,737	0	1,646	1	-0,407	FRI1_ARATH	Ferritin-1, chloroplastic (AtFer1) (EC 1.16.3.1)	FER1 At5g01600 F7A7.120	Arabidopsis thaliana (Mouse-ear cress)		Chloroplast; Complete proteome; Iron; Iron storage; Metal-binding; Oxidoreductase; Plastid; Reference proteome; Transit peptide
SRR342022_TR18252_c2_g4_i1_len=1124	NA	NA	NA	NA	NA	PF06747.10^CHCH^CHCH domain^158-192^E:1.8e-08	0,44	-0,198	0	0,892	0	1,217	0	1,063	0,184	-0,329	.	.	.	.	.	.
SRR342022_TR23364_c0_g1_i1_len=3450	CLPD_ARATH	Q:1412-3112,H:375-943	73.86%ID	E:0	Chaperone protein ClpD, chloroplastic {ECO:0000303|PubMed:11299370}	PF07724.11^AAA_2^AAA domain (Cdc48 subfamily)^226-401^E:9.5e-52`PF00158.23^Sigma54_activat^Sigma-54 interaction domain^228-346^E:0.00017`PF07728.11^AAA_5^AAA domain (dynein-related subfamily)^231-351^E:7.3e-12`PF00004.26^AAA^ATPase family associated with various cellular activities (AAA)^231-339^E:1e-06`PF10431.6^ClpB_D2-small^C-terminal, D2-small domain, of ClpB protein^407-486^E:7.4e-23	0,04	1,339	0	2,475	0	2,571	0	2,502	1	-0,064	CLPD_ARATH	Chaperone protein ClpD, chloroplastic (EC 3.6.1.3) (ATP-dependent Clp protease ATP-binding subunit ClpD homolog) (Casein lytic proteinase D) (ERD1 protein) (Protein EARLY RESPONSIVE TO DEHYDRATION 1) (Protein SENESCENCE ASSOCIATED GENE 15)	CLPD ERD1 SAG15 At5g51070 K3K7.27	Arabidopsis thaliana (Mouse-ear cress)		ATP-binding; Chaperone; Chloroplast; Complete proteome; Hydrolase; Nucleotide-binding; Plastid; Reference proteome; Repeat; Stress response; Transit peptide
SRR342022_TR27951_c0_g1_i1_len=798	NA	NA	NA	NA	NA	PF00582.23^Usp^Universal stress protein family^48-193^E:2.9e-26	0,051	1,135	0	2,636	0	2,542	0	2,234	1	-1,281	.	.	.	.	.	.
SRR342022_TR30528_c1_g2_i1_len=2981	MSL3_ARATH	Q:845-2551,H:42-659	57.84%ID	E:0	Mechanosensitive ion channel protein 3, chloroplastic	PF00924.15^MS_channel^Mechanosensitive ion channel^241-448^E:5.7e-30	0,001	1,348	0	3,09	0	2,642	0	2,498	1	0,451	MSL3_ARATH	Mechanosensitive ion channel protein 3, chloroplastic (Mechanosensitive channel of small conductance-like 3) (MscS-Like protein 3)	MSL3 At1g58200 F16M22.2 T18I24.11	Arabidopsis thaliana (Mouse-ear cress)		Chloroplast; Complete proteome; Ion channel; Ion transport; Membrane; Phosphoprotein; Plastid; Reference proteome; Transit peptide; Transmembrane; Transmembrane helix; Transport
SRR342022_TR30552_c2_g2_i5_len=4084	NA	NA	NA	NA	NA	PF07712.9^SURNod19^Stress up-regulated Nod 19^34-404^E:4.3e-186	0,298	0,679	0	2,891	0	2,512	0	2,536	0,832	-0,21	.	.	.	.	.	.
SRR342022_TR32777_c0_g2_i1_len=1741	RFS6_ARATH	Q:60-1610,H:261-749	73.17%ID	E:0	Probable galactinol–sucrose galactosyltransferase 6	PF05691.9^Raffinose_syn^Raffinose synthase or seed imbibition protein Sip1^9-518^E:8.1e-222	0,034	0,684	0	2,24	0	1,733	0	1,662	0,085	0,472	RFS6_ARATH	Probable galactinol–sucrose galactosyltransferase 6 (EC 2.4.1.82) (Protein DARK INDUCIBLE 10) (Raffinose synthase 6)	RFS6 DIN10 RS6 At5g20250 F5O24.140	Arabidopsis thaliana (Mouse-ear cress)		Alternative splicing; Carbohydrate metabolism; Complete proteome; Glycosyltransferase; Reference proteome; Transferase
SRR342022_TR33006_c0_g1_i1_len=616	ASPGB_ARATH	Q:153-614,H:3-156	85.71%ID	E:5e-93	Probable isoaspartyl peptidase/L-asparaginase 2	PF01112.15^Asparaginase_2^Asparaginase^5-155^E:8.3e-53	0,165	-0,762	0	5,674	0	4,922	0	6,843	0,254	-2,687	ASPGB_ARATH	Probable isoaspartyl peptidase/L-asparaginase 2 (EC 3.4.19.5) (L-asparagine amidohydrolase 2) [Cleaved into: Isoaspartyl peptidase/L-asparaginase 2 subunit alpha; Isoaspartyl peptidase/L-asparaginase 2 subunit beta]	At3g16150 MSL1.19	Arabidopsis thaliana (Mouse-ear cress)		Alternative splicing; Autocatalytic cleavage; Complete proteome; Hydrolase; Protease; Reference proteome
SRR342022_TR34054_c2_g1_i1_len=1654	NA	NA	NA	NA	NA	PF04640.11^PLATZ^PLATZ transcription factor^28-99^E:1.9e-29	0,052	1,621	0	2,472	0	2,55	0	2,562	1	0,253	.	.	.	.	.	.
SRR342022_TR34536_c1_g4_i1_len=2142	C98A2_SOYBN	Q:1644-181,H:22-509	85.66%ID	E:0	Cytochrome P450 98A2	PF00067.19^p450^Cytochrome P450^29-481^E:3.2e-102	0,115	0,742	0	3,582	0,001	1,106	0	1,772	0,278	0,759	C98A2_SOYBN	Cytochrome P450 98A2 (EC 1.14.-.-)	CYP98A2	Glycine max (Soybean) (Glycine hispida)		Complete proteome; Heme; Iron; Membrane; Metal-binding; Monooxygenase; Oxidoreductase; Reference proteome; Transmembrane; Transmembrane helix
SRR342023_TR798_c1_g19_i3_len=1000	MES9_ARATH	Q:175-942,H:2-254	42.97%ID	E:2e-64	Methylesterase 9	PF00561.17^Abhydrolase_1^alpha/beta hydrolase fold^45-284^E:4.1e-15`PF12697.4^Abhydrolase_6^Alpha/beta hydrolase family^46-289^E:8.1e-17`PF12146.5^Hydrolase_4^Serine aminopeptidase, S33^46-156^E:1.5e-12	0,816	-0,154	0	4,227	0	2,563	0	3,506	0,001	1,522	MES9_ARATH	Methylesterase 9 (AtMES9) (EC 3.1.1.-)	MES9 At4g37150 AP22.78 C7A10.210	Arabidopsis thaliana (Mouse-ear cress)		Complete proteome; Hydrolase; Immunity; Innate immunity; Plant defense; Reference proteome
SRR342023_TR1587_c2_g1_i2_len=4884	PHT14_ARATH	Q:2814-4337,H:1-508	84.45%ID	E:0	Inorganic phosphate transporter 1-4	PF00083.21^Sugar_tr^Sugar (and other) transporter^25-515^E:5e-49`PF07690.13^MFS_1^Major Facilitator Superfamily^69-457^E:2.1e-21	0,066	1,022	0	4,307	0	7,933	0	6,842	1	-0,773	PHT14_ARATH	Inorganic phosphate transporter 1-4 (AtPht1;4) (H(+)/Pi cotransporter)	PHT1-4 PHT4 PT2 At2g38940 T7F6.11	Arabidopsis thaliana (Mouse-ear cress)		Cell membrane; Complete proteome; Membrane; Phosphate transport; Phosphoprotein; Reference proteome; Symport; Transmembrane; Transmembrane helix; Transport
SRR342023_TR1698_c2_g1_i1_len=1005	KIC_ARATH	Q:727-431,H:21-119	72.73%ID	E:3e-45	Calcium-binding protein KIC	PF13833.3^EF-hand_8^EF-hand domain pair^43-94^E:5.3e-16	0,237	0,44	0	4,728	0	2,816	0	2,473	1	0	KIC_ARATH	Calcium-binding protein KIC (KCBP-interacting calcium-binding protein)	KIC At2g46600 F13A10.13	Arabidopsis thaliana (Mouse-ear cress)		3D-structure; Calcium; Complete proteome; Metal-binding; Reference proteome
SRR342023_TR2241_c0_g3_i1_len=522	NA	NA	NA	NA	NA	.	0,76	-0,576	0	4,26	0	3,812	0	4,063	0,732	0,769	.	.	.	.	.	.
SRR342023_TR16666_c0_g1_i5_len=4417	LOX5_ARATH	Q:228-2852,H:34-886	67.99%ID	E:0	Linoleate 9S-lipoxygenase 5, chloroplastic	PF01477.20^PLAT^PLAT/LH2 domain^81-173^E:2.1e-15`PF00305.16^Lipoxygenase^Lipoxygenase^187-624^E:2.2e-197	0,866	0,085	0	2,759	0	2,029	0	3,36	1	1,128	LOX5_ARATH	Linoleate 9S-lipoxygenase 5, chloroplastic (EC 1.13.11.58) (Lipoxygenase 5) (AtLOX5)	LOX5 At3g22400 MCB17.13	Arabidopsis thaliana (Mouse-ear cress)	PATHWAY: Lipid metabolism; oxylipin biosynthesis. {ECO:0000255|PROSITE-ProRule:PRU00726}.	Chloroplast; Complete proteome; Dioxygenase; Fatty acid biosynthesis; Fatty acid metabolism; Iron; Lipid biosynthesis; Lipid metabolism; Metal-binding; Oxidoreductase; Oxylipin biosynthesis; Plastid; Reference proteome
SRR342023_TR18599_c2_g5_i10_len=3676	DGAT2_ARATH	Q:745-1554,H:46-314	71.85%ID	E:5e-135	Diacylglycerol O-acyltransferase 2	PF03547.15^Mem_trans^Membrane transport protein^11-405^E:7.7e-65	0,017	2,06	0	3,653	0	5,904	0	5,67	0,239	1,585	DGAT2_ARATH	Diacylglycerol O-acyltransferase 2 (EC 2.3.1.20)	DGAT2 At3g51520 F26O13.160	Arabidopsis thaliana (Mouse-ear cress)	PATHWAY: Glycerolipid metabolism; triacylglycerol biosynthesis.	Acyltransferase; Complete proteome; Endoplasmic reticulum; Glycerol metabolism; Glycoprotein; Lipid biosynthesis; Lipid metabolism; Membrane; Reference proteome; Transferase; Transmembrane; Transmembrane helix
SRR342023_TR19871_c0_g1_i2_len=1614	BBD1_ARATH	Q:551-1231,H:94-320	77.97%ID	E:1e-127	Bifunctional nuclease 1	PF02577.11^DNase-RNase^Bifunctional nuclease^44-158^E:2.2e-22	0,539	0,221	0	2,922	0	2,861	0	3,99	0,899	0,071	BBD1_ARATH	Bifunctional nuclease 1 (AtBBD1) (EC 3.1.-.-)	BBD1 At1g75380 F1B16.9 F1B16_15	Arabidopsis thaliana (Mouse-ear cress)		Complete proteome; Hydrolase; Nuclease; Nucleus; Reference proteome
SRR342023_TR19929_c1_g1_i2_len=1377	PT504_ARATH	Q:334-1245,H:6-309	81.25%ID	E:0	Probable sugar phosphate/phosphate translocator At5g04160	PF08449.8^UAA^UAA transporter family^17-310^E:4.9e-14`PF03151.13^TPT^Triose-phosphate Transporter family^20-309^E:7.3e-46	0,763	-0,257	0	2,096	0	3,312	0	4,943	1	-0,046	PT504_ARATH	Probable sugar phosphate/phosphate translocator At5g04160	At5g04160 F21E1.80	Arabidopsis thaliana (Mouse-ear cress)		Complete proteome; Membrane; Pyruvate; Reference proteome; Sugar transport; Transmembrane; Transmembrane helix; Transport
SRR342023_TR22818_c0_g2_i1_len=446	NA	NA	NA	NA	NA	.	0,112	1,129	0	4,739	0	4,862	0	5,38	0,581	-0,356	.	.	.	.	.	.
SRR342023_TR27035_c0_g1_i1_len=2028	ERDL4_ARATH	Q:304-1767,H:1-488	77.71%ID	E:0	Sugar transporter ERD6-like 4	PF00083.21^Sugar_tr^Sugar (and other) transporter^52-484^E:2.4e-99`PF07690.13^MFS_1^Major Facilitator Superfamily^62-435^E:7.3e-25	0,398	0,403	0	2,003	0	1,578	0	2,22	0,847	0,14	ERDL4_ARATH	Sugar transporter ERD6-like 4	At1g19450 F18O14.22	Arabidopsis thaliana (Mouse-ear cress)		Complete proteome; Membrane; Reference proteome; Sugar transport; Transmembrane; Transmembrane helix; Transport
SRR342023_TR28027_c0_g2_i1_len=1586	NA	NA	NA	NA	NA	.	0,328	0,414	0	3,153	0	4,218	0	4,89	0,904	0,1	.	.	.	.	.	.
SRR342023_TR28680_c0_g1_i3_len=1608	C71DC_CATRO	Q:744-142,H:296-492	72.14%ID	E:1e-89	Tabersonine 16-hydroxylase	PF00067.19^p450^Cytochrome P450^1-173^E:8.4e-59	0,002	1,226	0,001	1,168	0	2,253	0	1,827	0,316	0,659	C71DC_CATRO	Tabersonine 16-hydroxylase (EC 1.14.13.73) (Cytochrome P450 71D12) (Fragment)	CYP71D12	Catharanthus roseus (Madagascar periwinkle) (Vinca rosea)	PATHWAY: Alkaloid biosynthesis; vindoline biosynthesis; vindoline from tabersonine: step 1/6.	Endoplasmic reticulum; Heme; Iron; Metal-binding; Monooxygenase; NADP; Oxidoreductase
SRR342023_TR29509_c0_g3_i3_len=2067	C7D55_HYOMU	Q:228-1592,H:49-502	52.3%ID	E:5e-163	Premnaspirodiene oxygenase	PF00067.19^p450^Cytochrome P450^33-486^E:1.3e-101	0,004	0,803	0	1,271	0	1,832	0	1,779	0,007	0,876	C7D55_HYOMU	Premnaspirodiene oxygenase (HPO) (EC 1.14.13.121) (Cytochrome P450 71D55)	CYP71D55	Hyoscyamus muticus (Egyptian henbane)		Heme; Iron; Membrane; Metal-binding; Monooxygenase; NADP; Oxidoreductase; Transmembrane; Transmembrane helix
SRR342023_TR29531_c0_g9_i2_len=1198	TSJT1_TOBAC	Q:186-626,H:1-148	44.3%ID	E:3e-35	Stem-specific protein TSJT1	PF12481.5^DUF3700^Aluminium induced protein^63-287^E:2.6e-89`PF13522.3^GATase_6^Glutamine amidotransferase domain^113-229^E:2.5e-08`PF13537.3^GATase_7^Glutamine amidotransferase domain^121-233^E:5.9e-11	0,412	0,213	0	1,361	0	1,12	0	1,458	0,002	0,986	TSJT1_TOBAC	Stem-specific protein TSJT1	TSJT1	Nicotiana tabacum (Common tobacco)		
SRR342023_TR30981_c0_g1_i1_len=940	BH147_ARATH	Q:395-832,H:49-201	56.69%ID	E:3e-46	Transcription factor bHLH147	.	0,007	1,004	0	2,369	0	1,856	0	2,028	1	-0,07	BH147_ARATH	Transcription factor bHLH147 (ATBS1 interacting factor 3) (Basic helix-loop-helix protein 147) (AtbHLH147) (bHLH 147) (Transcription factor EN 142) (bHLH transcription factor bHLH147)	BHLH147 AIF3 EN142 At3g17100 K14A17.17	Arabidopsis thaliana (Mouse-ear cress)		Complete proteome; DNA-binding; Nucleus; Reference proteome; Transcription; Transcription regulation
SRR342023_TR31426_c6_g4_i1_len=2314	TT12_ARATH	Q:1448-2062,H:289-493	44.88%ID	E:2e-41	.	PF01554.15^MatE^MatE^40-198^E:9.9e-24	0,76	0,698	0	2,214	0	3,258	0	3,689	1	-0,149	DTX41_ARATH	Protein DETOXIFICATION 41 (AtDTX41) (Multidrug and toxic compound extrusion protein 41) (MATE protein 41) (Protein TANNIN-DEFICIENT SEED 3) (Protein TRANSPARENT TESTA 12)	DTX41 TDS3 TT12 At3g59030 F17J16_80	Arabidopsis thaliana (Mouse-ear cress)	PATHWAY: Secondary metabolite biosynthesis; flavonoid biosynthesis.	Antiport; Complete proteome; Flavonoid biosynthesis; Membrane; Reference proteome; Transmembrane; Transmembrane helix; Transport; Vacuole
SRR342023_TR31454_c5_g6_i1_len=732	PRS2_SOLTU	Q:660-205,H:1-155	36.94%ID	E:4e-24	Pathogenesis-related protein STH-2	PF00407.16^Bet_v_1^Pathogenesis-related protein Bet v I family^25-171^E:4e-13	0,002	0,704	0	2,569	0	1,524	0	1,546	1	0,342	PRS2_SOLTU	Pathogenesis-related protein STH-2	STH-2	Solanum tuberosum (Potato)		Complete proteome; Pathogenesis-related protein; Plant defense; Reference proteome
SRR646572_TR25887_c0_g2_i5_len=1422	HS217_ARATH	Q:645-1154,H:9-184	54.55%ID	E:6e-59	21.7 kDa class VI heat shock protein	PF00011.18^HSP20^Hsp20/alpha crystallin family^87-179^E:1.4e-10	0,094	1,158	0	1,905	0	1,854	0	1,828	1	0,126	HS217_ARATH	21.7 kDa class VI heat shock protein (21.7 kDa heat shock protein) (AtHsp21.7)	HSP21.7 At5g54660 MRB17.16	Arabidopsis thaliana (Mouse-ear cress)		Complete proteome; Cytoplasm; Reference proteome; Stress response
SRR646572_TR35126_c0_g1_i1_len=1004	NA	NA	NA	NA	NA	PF12507.5^HCMV_UL139^Human Cytomegalovirus UL139 protein^22-139^E:1.7e-20	0,025	1,981	0	4,343	0	5,187	0	6,247	1	-3,501	.	.	.	.	.	.
SRR646572_TR35841_c0_g1_i1_len=752	NLTP_HELAN	Q:622-272,H:1-116	52.54%ID	E:6e-27	Non-specific lipid-transfer protein	PF14368.3^LTP_2^Probable lipid transfer^15-105^E:1.4e-05`PF00234.19^Tryp_alpha_amyl^Protease inhibitor/seed storage/LTP family^30-113^E:1e-07	0,341	0,207	0	0,899	0	2,823	0	1,673	0,146	0,506	NLTP_HELAN	Non-specific lipid-transfer protein (LTP) (NsLTP) (SDI-9)		Helianthus annuus (Common sunflower)		Disulfide bond; Lipid-binding; Signal; Transport
SRR646596_TR595_c0_g2_i1_len=1576	BH014_ARATH	Q:1381-143,H:39-421	35.22%ID	E:6e-59	Transcription factor bHLH14	PF14215.3^bHLH-MYC_N^bHLH-MYC and R2R3-MYB transcription factors N-terminal^26-185^E:1.4e-33`PF00010.23^HLH^Helix-loop-helix DNA-binding domain^260-305^E:2.1e-10	1	0,358	0	5,634	0	10,779	0	11,579	1	0	BH014_ARATH	Transcription factor bHLH14 (Basic helix-loop-helix protein 14) (AtbHLH14) (bHLH 14) (Transcription factor EN 33) (bHLH transcription factor bHLH014)	BHLH14 EN33 At4g00870 A_TM018A10.7 T18A10.17	Arabidopsis thaliana (Mouse-ear cress)		Complete proteome; DNA-binding; Nucleus; Reference proteome; Transcription; Transcription regulation
SRR646596_TR1244_c0_g1_i11_len=3593	SYK_ARATH	Q:292-1899,H:80-615	81.34%ID	E:0	.	PF01336.22^tRNA_anti-codon^OB-fold nucleic acid binding domain^88-166^E:2.9e-13`PF00152.17^tRNA-synt_2^tRNA synthetases class II (D, K and N)^206-555^E:2.8e-82	0,955	0,038	0	1,743	0	3,068	0	2,836	0,575	-0,198	SYKC_ARATH	Lysine--tRNA ligase, cytoplasmic (EC 6.1.1.6) (Lysyl-tRNA synthetase) (LysRS) (Lysyl-tRNA synthetase 1) (AtKRS-1)	At3g11710 T19F11.11	Arabidopsis thaliana (Mouse-ear cress)		ATP-binding; Acetylation; Aminoacyl-tRNA synthetase; Calcium; Coiled coil; Complete proteome; Cytoplasm; DNA-binding; Ligase; Metal-binding; Nucleotide-binding; Protein biosynthesis; Reference proteome
SRR646596_TR1756_c1_g1_i1_len=932	NA	NA	NA	NA	NA	PF01221.15^Dynein_light^Dynein light chain type 1^65-158^E:2.3e-26	1	2,138	0	7,114	0	5,34	0	6,238	1	0,555	.	.	.	.	.	.
SRR646596_TR2215_c0_g1_i1_len=805	IPRT1_CATRO	Q:152-631,H:1-157	68.75%ID	E:2e-66	Probable intracellular pathogenesis-related protein T1	PF00407.16^Bet_v_1^Pathogenesis-related protein Bet v I family^5-157^E:1.1e-19	0,76	0,576	0	7,742	0	7,24	0	3,097	0,027	-2,429	IPRT1_CATRO	Probable intracellular pathogenesis-related protein T1	PCKR3	Catharanthus roseus (Madagascar periwinkle) (Vinca rosea)		Direct protein sequencing; Glycoprotein; Pathogenesis-related protein; Plant defense
SRR646596_TR4674_c0_g1_i1_len=1491	ACMAT_VITLA	Q:1365-34,H:10-449	48.89%ID	E:3e-151	Methanol O-anthraniloyltransferase	PF02458.12^Transferase^Transferase family^6-437^E:6.3e-68	0,094	1,235	0	5,457	0	6,815	0	7,492	0,16	1,326	ACMAT_VITLA	Methanol O-anthraniloyltransferase (EC 2.3.1.232) (Anthraniloyl-CoA:methanol acyltransferase) (Benzyl alcohol O-benzoyltransferase) (EC 2.3.1.196)	AMAT	Vitis labrusca (Concord grape)		Acyltransferase; Direct protein sequencing; Transferase
SRR646596_TR4958_c0_g1_i3_len=2289	TRXH2_TOBAC	Q:763-503,H:30-116	82.76%ID	E:2e-42	Thioredoxin H-type 2	.	0,381	2,69	0	6,128	0	3,311	0	3,594	1	0,101	TRXH2_TOBAC	Thioredoxin H-type 2 (Trx-H2)		Nicotiana tabacum (Common tobacco)		Cytoplasm; Disulfide bond; Electron transport; Redox-active center; Transport
SRR646596_TR5611_c0_g1_i1_len=2176	NA	NA	NA	NA	NA	.	0,045	1,547	0	4,445	0	4,941	0	5,779	0,732	-0,713	.	.	.	.	.	.
SRR646596_TR8408_c0_g1_i1_len=1358	COMT1_CAPAN	Q:165-1220,H:9-359	54.55%ID	E:9e-133	Caffeic acid 3-O-methyltransferase	PF08100.8^Dimerisation^Dimerisation domain^32-83^E:1.4e-19`PF00891.15^Methyltransf_2^O-methyltransferase^128-346^E:3.4e-73	0,158	1,681	0	7,647	0	9,866	0	10,755	1	2,501	COMT1_CAPAN	Caffeic acid 3-O-methyltransferase (CAOMT) (COMT) (EC 2.1.1.68) (S-adenosysl-L-methionine:caffeic acid 3-O-methyltransferase)	COMT	Capsicum annuum (Bell pepper)	PATHWAY: Aromatic compound metabolism; phenylpropanoid biosynthesis.	Lignin biosynthesis; Methyltransferase; S-adenosyl-L-methionine; Transferase
SRR646596_TR12950_c0_g1_i2_len=1785	OPR3_SOLLC	Q:499-1647,H:9-390	79.37%ID	E:0	12-oxophytodienoate reductase 3	PF00724.17^Oxidored_FMN^NADH:flavin oxidoreductase / NADH oxidase family^10-359^E:4.7e-87	0,029	0,756	0	4,044	0	1,886	0	2,659	0,173	-0,674	OPR3_SOLLC	12-oxophytodienoate reductase 3 (EC 1.3.1.42) (12-oxophytodienoate-10,11-reductase 3) (OPDA-reductase 3) (LeOPR3)	OPR3	Solanum lycopersicum (Tomato) (Lycopersicon esculentum)	PATHWAY: Lipid metabolism; oxylipin biosynthesis.	3D-structure; Complete proteome; FMN; Fatty acid biosynthesis; Fatty acid metabolism; Flavoprotein; Lipid biosynthesis; Lipid metabolism; NADP; Oxidoreductase; Oxylipin biosynthesis; Peroxisome; Reference proteome
SRR646596_TR15830_c3_g1_i27_len=1817	FAD3E_TOBAC	Q:477-1571,H:12-374	76.16%ID	E:0	Omega-3 fatty acid desaturase, endoplasmic reticulum	PF11960.5^DUF3474^Domain of unknown function (DUF3474)^6-62^E:7.5e-26`PF00487.21^FA_desaturase^Fatty acid desaturase^77-328^E:4.4e-33	0,44	0,948	0	4,506	0	3,79	0	4,436	0,593	-0,669	FAD3E_TOBAC	Omega-3 fatty acid desaturase, endoplasmic reticulum (EC 1.14.19.-)	FAD3	Nicotiana tabacum (Common tobacco)	PATHWAY: Lipid metabolism; polyunsaturated fatty acid biosynthesis.	Endoplasmic reticulum; Fatty acid biosynthesis; Fatty acid metabolism; Lipid biosynthesis; Lipid metabolism; Membrane; Oxidoreductase; Transmembrane; Transmembrane helix
SRR646596_TR18446_c0_g1_i2_len=3570	NA	NA	NA	NA	NA	PF12507.5^HCMV_UL139^Human Cytomegalovirus UL139 protein^25-142^E:3.2e-21	0,076	2,389	0	5,604	0	6,615	0	7,389	1	0,826	.	.	.	.	.	.
SRR646596_TR19493_c0_g2_i1_len=747	NA	NA	NA	NA	NA	.	0,226	2,488	0	6,235	0	6,499	0	7,173	1	-0,541	.	.	.	.	.	.
SRR646596_TR19548_c2_g1_i1_len=1319	CLH2_ARATH	Q:71-916,H:5-299	42.14%ID	E:1e-55	Chlorophyllase-2, chloroplastic	PF07224.8^Chlorophyllase^Chlorophyllase^23-317^E:6.3e-90`PF12740.4^Chlorophyllase2^Chlorophyllase enzyme^53-301^E:3.7e-87`PF03403.10^PAF-AH_p_II^Platelet-activating factor acetylhydrolase, isoform II^60-99^E:2.4e-06	0,001	2,468	0	4,395	0	4,504	0	4,544	1	0,067	CLH2_ARATH	Chlorophyllase-2, chloroplastic (AtCLH2) (EC 3.1.1.14) (Chlorophyll-chlorophyllido hydrolase 2) (Chlase 2)	CLH2 At5g43860 MQD19.22	Arabidopsis thaliana (Mouse-ear cress)	PATHWAY: Porphyrin-containing compound metabolism; chlorophyll degradation.	Chlorophyll catabolism; Chloroplast; Complete proteome; Hydrolase; Membrane; Plastid; Reference proteome; Transit peptide
SRR646596_TR20372_c1_g1_i8_len=3816	AFS1_MALDO	Q:2823-3686,H:274-563	54.3%ID	E:2e-95	(E,E)-alpha-farnesene synthase	PF03936.13^Terpene_synth_C^Terpene synthase family, metal binding domain^1-158^E:7.1e-43	1	2,176	0	9,401	0	9,488	0	10,228	1	0	AFS1_MALDO	(E,E)-alpha-farnesene synthase (MdASF1) (EC 4.2.3.46) (Prenyltransferase) (EC 2.5.1.-)	AFS1 AFAR	Malus domestica (Apple) (Pyrus malus)		Cytoplasm; Lyase; Magnesium; Manganese; Metal-binding; Potassium; Transferase
SRR646596_TR20372_c1_g1_i16_len=1360	TPS7_RICCO	Q:474-1358,H:50-340	48.47%ID	E:5e-82	Alpha-farnesene synthase	PF01397.18^Terpene_synth^Terpene synthase, N-terminal domain^5-102^E:7.3e-24`PF03936.13^Terpene_synth_C^Terpene synthase family, metal binding domain^135-235^E:3.2e-37	1	1,217	0	6,243	0	9,605	0	10,798	1	0	TPS7_RICCO	Alpha-farnesene synthase (EC 4.2.3.46) ((E,E)-alpha-farnesene synthase) (Terpene synthase 7) (RcSeTPS7)	TPS7 RCOM_0906600	Ricinus communis (Castor bean)		Complete proteome; Lyase; Magnesium; Metal-binding; Reference proteome
SRR646596_TR25164_c0_g1_i3_len=1751	NA	NA	NA	NA	NA	PF00892.17^EamA^EamA-like transporter family^172-297^E:3.3e-09`PF00892.17^EamA^EamA-like transporter family^307-456^E:2.6e-13	0,001	1,701	0	2,539	0	3,116	0	3,48	0,407	0,622	.	.	.	.	.	.
SRR646596_TR25397_c0_g2_i2_len=1677	TRPB_CAMAC	Q:1193-87,H:85-456	66.4%ID	E:0	Tryptophan synthase beta chain 2, chloroplastic	PF00291.22^PALP^Pyridoxal-phosphate dependent enzyme^154-481^E:1e-50	0,003	2,239	0	8,623	0	8,115	0	9,2	1	1,074	TRPB_CAMAC	Tryptophan synthase beta chain 2, chloroplastic (EC 4.2.1.20)	TSB	Camptotheca acuminata (Happy tree)	PATHWAY: Amino-acid biosynthesis; L-tryptophan biosynthesis; L-tryptophan from chorismate: step 5/5.	Amino-acid biosynthesis; Aromatic amino acid biosynthesis; Chloroplast; Lyase; Plastid; Pyridoxal phosphate; Transit peptide; Tryptophan biosynthesis
SRR646596_TR26170_c0_g1_i1_len=2541	TRPE_ARATH	Q:341-1489,H:50-434	68.05%ID	E:0	Anthranilate synthase alpha subunit 1, chloroplastic	PF04715.10^Anth_synt_I_N^Anthranilate synthase component I, N terminal region^153-307^E:7.3e-28`PF00425.15^Chorismate_bind^chorismate binding enzyme^368-502^E:3.4e-36	1	-0,302	0	4,243	0	4,791	0	5,537	1	0,315	TRPE_ARATH	Anthranilate synthase alpha subunit 1, chloroplastic (EC 4.1.3.27) (Anthranilate synthase component 1-1) (Anthranilate synthase component I-1) (Protein A-METHYL TRYPTOPHAN RESISTANT 1) (Protein JASMONATE-INDUCED DEFECTIVE LATERAL ROOT 1) (Protein TRYPTOPHAN BIOSYNTHESIS 5) (Protein WEAK ETHYLENE INSENSITIVE 2)	ASA1 AMT1 JDL1 TRP5 WEI2 At5g05730 MJJ3.14	Arabidopsis thaliana (Mouse-ear cress)	PATHWAY: Amino-acid biosynthesis; L-tryptophan biosynthesis; L-tryptophan from chorismate: step 1/5.	Acetylation; Alternative splicing; Amino-acid biosynthesis; Aromatic amino acid biosynthesis; Chloroplast; Complete proteome; Lyase; Plastid; Reference proteome; Transit peptide; Tryptophan biosynthesis
SRR646596_TR28644_c0_g1_i2_len=2039	GPX1_PEA	Q:1895-1161,H:1-236	70.45%ID	E:5e-105	Phospholipid hydroperoxide glutathione peroxidase, chloroplastic	PF08534.7^Redoxin^Redoxin^85-160^E:4e-08`PF00255.16^GSHPx^Glutathione peroxidase^88-196^E:4.9e-44`PF00578.18^AhpC-TSA^AhpC/TSA family^88-150^E:4.3e-09	0,132	2,814	0	5,162	0	5,611	0	6,106	1	0,012	GPX1_PEA	Phospholipid hydroperoxide glutathione peroxidase, chloroplastic (PHGPx) (EC 1.11.1.12)		Pisum sativum (Garden pea)		Chloroplast; Oxidoreductase; Peroxidase; Plastid; Transit peptide
SRR646596_TR28759_c0_g2_i1_len=2422	DOX1_ARATH	Q:89-2119,H:1-639	65.74%ID	E:0	Alpha-dioxygenase 1	PF03098.12^An_peroxidase^Animal haem peroxidase^123-381^E:1.2e-55	0,517	0,975	0	4,672	0	7,802	0	8,592	0,299	-1,153	DOX1_ARATH	Alpha-dioxygenase 1 (Alpha DOX1) (EC 1.14.99.-) (Fatty acid dioxygenase AlphaDOX1) (Pathogen-induced oxygenase) (Plant alpha dioxygenase 1)	DOX1 DIOX1 PADOX-1 PIOX At3g01420 T13O15.6	Arabidopsis thaliana (Mouse-ear cress)		3D-structure; Complete proteome; Dioxygenase; Fatty acid biosynthesis; Fatty acid metabolism; Glycoprotein; Hypersensitive response; Iron; Lipid biosynthesis; Lipid metabolism; Metal-binding; Oxidoreductase; Oxylipin biosynthesis; Peroxidase; Plant defense; Reference proteome; Signal
SRR646596_TR28759_c0_g2_i2_len=2322	DOX1_ARATH	Q:89-1159,H:1-353	74.02%ID	E:0	Alpha-dioxygenase 1	PF03098.12^An_peroxidase^Animal haem peroxidase^123-381^E:1.2e-55	1	0,538	0	4,239	0	7,312	0	8,244	0,629	-1,622	DOX1_ARATH	Alpha-dioxygenase 1 (Alpha DOX1) (EC 1.14.99.-) (Fatty acid dioxygenase AlphaDOX1) (Pathogen-induced oxygenase) (Plant alpha dioxygenase 1)	DOX1 DIOX1 PADOX-1 PIOX At3g01420 T13O15.6	Arabidopsis thaliana (Mouse-ear cress)		3D-structure; Complete proteome; Dioxygenase; Fatty acid biosynthesis; Fatty acid metabolism; Glycoprotein; Hypersensitive response; Iron; Lipid biosynthesis; Lipid metabolism; Metal-binding; Oxidoreductase; Oxylipin biosynthesis; Peroxidase; Plant defense; Reference proteome; Signal
SRR646596_TR28962_c0_g1_i6_len=1981	C72A1_CATRO	Q:1104-265,H:245-524	97.86%ID	E:0	Secologanin synthase	PF00067.19^p450^Cytochrome P450^93-235^E:2.5e-16	0,158	1,626	0	5,843	0	4,053	0	3,669	1	1,667	C72A1_CATRO	Secologanin synthase (SLS) (EC 1.3.3.9) (CYPLXXII) (Cytochrome P450 72A1)	CYP72A1 CYP72 P450CR3	Catharanthus roseus (Madagascar periwinkle) (Vinca rosea)	PATHWAY: Alkaloid biosynthesis; secologanin biosynthesis.	Alkaloid metabolism; Heme; Iron; Membrane; Metal-binding; Monooxygenase; NADP; Oxidoreductase; Transmembrane; Transmembrane helix; Vacuole
SRR646596_TR30017_c1_g3_i2_len=3962	TPS7_RICCO	Q:353-1129,H:74-334	48.88%ID	E:1e-72	Alpha-farnesene synthase	PF01397.18^Terpene_synth^Terpene synthase, N-terminal domain^2-74^E:4.5e-10`PF03936.13^Terpene_synth_C^Terpene synthase family, metal binding domain^103-195^E:1.3e-27	1	0,347	0	6,678	0	7,852	0	9,252	1	0	TPS7_RICCO	Alpha-farnesene synthase (EC 4.2.3.46) ((E,E)-alpha-farnesene synthase) (Terpene synthase 7) (RcSeTPS7)	TPS7 RCOM_0906600	Ricinus communis (Castor bean)		Complete proteome; Lyase; Magnesium; Metal-binding; Reference proteome
SRR646596_TR32139_c1_g1_i1_len=1808	NA	NA	NA	NA	NA	PF03060.12^NMO^Nitronate monooxygenase^6-74^E:9.6e-13`PF00478.22^IMPDH^IMP dehydrogenase / GMP reductase domain^26-207^E:5.2e-08`PF03060.12^NMO^Nitronate monooxygenase^76-319^E:1.3e-42`PF01070.15^FMN_dh^FMN-dependent dehydrogenase^117-199^E:1e-07	0,051	1,074	0	2,897	0	3,307	0	2,985	0,679	-0,433	.	.	.	.	.	.
SRR646596_TR32486_c0_g1_i1_len=2139	NA	NA	NA	NA	NA	.	0,005	0,88	0	2,034	0	1,846	0	1,63	0,077	0,601	.	.	.	.	.	.
SRR646604_TR1107_c0_g1_i3_len=4655	PR5_ARATH	Q:1058-366,H:7-239	55.27%ID	E:2e-63	Pathogenesis-related protein 5	PF01764.22^Lipase_3^Lipase (class 3)^412-548^E:7.3e-26	0,707	0,415	0	2,137	0	3,781	0	4,689	1	-0,241	PR5_ARATH	Pathogenesis-related protein 5 (PR-5)	At1g75040 F9E10.11	Arabidopsis thaliana (Mouse-ear cress)		Apoplast; Complete proteome; Direct protein sequencing; Disulfide bond; Pathogenesis-related protein; Plant defense; Reference proteome; Secreted; Signal
SRR646604_TR2744_c0_g1_i3_len=1139	VSR1_ARATH	Q:111-872,H:357-612	79.69%ID	E:1e-136	Vacuolar-sorting receptor 1	.	0,046	0,863	0	3,194	0	3,012	0	3,377	0,601	0,264	VSR1_ARATH	Vacuolar-sorting receptor 1 (AtVSR1) (BP80-like protein b) (AtBP80b) (Epidermal growth factor receptor-like protein 1) (AtELP) (AtELP1) (Spot 3 protein)	VSR1 BP80B ELP ELP1 At3g52850 F8J2.20	Arabidopsis thaliana (Mouse-ear cress)		3D-structure; Calcium; Complete proteome; Cytoplasmic vesicle; Disulfide bond; EGF-like domain; Glycoprotein; Golgi apparatus; Membrane; Protein transport; Reference proteome; Repeat; Signal; Transmembrane; Transmembrane helix; Transport
SRR646604_TR2963_c0_g1_i1_len=4284	AB15B_ARATH	Q:4146-409,H:1-1238	72.32%ID	E:0	ABC transporter B family member 15	PF00664.20^ABC_membrane^ABC transporter transmembrane region^36-308^E:6.3e-46`PF00005.24^ABC_tran^ABC transporter^382-530^E:2.5e-33`PF00664.20^ABC_membrane^ABC transporter transmembrane region^683-952^E:1.2e-51`PF00005.24^ABC_tran^ABC transporter^1024-1172^E:9.8e-33	0,147	0,974	0	2,691	0	3,56	0	4,512	0,76	0,649	AB15B_ARATH	ABC transporter B family member 15 (ABC transporter ABCB.15) (AtABCB15) (Multidrug resistance protein 13) (P-glycoprotein 15)	ABCB15 MDR13 PGP15 At3g28345 MZF16.16	Arabidopsis thaliana (Mouse-ear cress)		ATP-binding; Complete proteome; Glycoprotein; Membrane; Nucleotide-binding; Reference proteome; Repeat; Transmembrane; Transmembrane helix; Transport
SRR646604_TR4901_c0_g1_i3_len=2933	DGAT2_ARATH	Q:550-1125,H:2-194	65.8%ID	E:2e-83	Diacylglycerol O-acyltransferase 2	PF03982.10^DAGAT^Diacylglycerol acyltransferase^52-204^E:4.8e-37	0,286	0,901	0	4,209	0	4,823	0	5,552	1	0,368	DGAT2_ARATH	Diacylglycerol O-acyltransferase 2 (EC 2.3.1.20)	DGAT2 At3g51520 F26O13.160	Arabidopsis thaliana (Mouse-ear cress)	PATHWAY: Glycerolipid metabolism; triacylglycerol biosynthesis.	Acyltransferase; Complete proteome; Endoplasmic reticulum; Glycerol metabolism; Glycoprotein; Lipid biosynthesis; Lipid metabolism; Membrane; Reference proteome; Transferase; Transmembrane; Transmembrane helix
SRR646604_TR5072_c0_g2_i4_len=1750	SDC1_ARATH	Q:217-1422,H:83-482	61.69%ID	E:0	Serine decarboxylase	PF00282.16^Pyridoxal_deC^Pyridoxal-dependent decarboxylase conserved domain^74-370^E:9.2e-25	0,629	0,817	0	7,699	0	10,156	0	11,636	1	0	SDC1_ARATH	Serine decarboxylase (AtSDC) (EC 4.1.1.-) (Protein EMBRYO DEFECTIVE 1075) (Serine decarboxylase 1) (AtSDC1)	SDC EMB1075 SDC1 At1g43710 F2J6.7	Arabidopsis thaliana (Mouse-ear cress)		Complete proteome; Cytoplasm; Decarboxylase; Lyase; Pyridoxal phosphate; Reference proteome
SRR646604_TR5846_c0_g1_i1_len=2084	NA	NA	NA	NA	NA	PF06813.10^Nodulin-like^Nodulin-like^74-319^E:6.2e-94`PF07690.13^MFS_1^Major Facilitator Superfamily^397-603^E:5.4e-12	0,299	1,191	0	5,006	0	7,644	0	8,552	1	0,694	.	.	.	.	.	.
SRR646604_TR6389_c0_g2_i1_len=773	BH035_ARATH	Q:690-52,H:5-215	53.18%ID	E:3e-70	Transcription factor bHLH35	PF00010.23^HLH^Helix-loop-helix DNA-binding domain^78-120^E:9.7e-12	0,01	2,425	0	4,705	0	2,776	0	3,64	1	0,923	BH035_ARATH	Transcription factor bHLH35 (Basic helix-loop-helix protein 35) (AtbHLH35) (bHLH 35) (Transcription factor EN 41) (bHLH transcription factor bHLH035)	BHLH35 EN41 At5g57150 MUL3.10	Arabidopsis thaliana (Mouse-ear cress)		Alternative splicing; Complete proteome; DNA-binding; Nucleus; Reference proteome; Transcription; Transcription regulation
SRR646604_TR7319_c0_g1_i1_len=1992	NA	NA	NA	NA	NA	PF00106.22^adh_short^short chain dehydrogenase^6-194^E:2.2e-25`PF13561.3^adh_short_C2^Enoyl-(Acyl carrier protein) reductase^17-253^E:1.7e-41	0,066	5,326	0	7,623	0	10,479	0	11,195	1	0	.	.	.	.	.	.
SRR646604_TR8743_c0_g2_i1_len=1590	PNC1_ARATH	Q:1197-241,H:1-320	73.44%ID	E:7e-144	Peroxisomal adenine nucleotide carrier 1	PF00153.24^Mito_carr^Mitochondrial carrier protein^7-97^E:1.6e-13`PF00153.24^Mito_carr^Mitochondrial carrier protein^105-188^E:8.5e-09`PF00153.24^Mito_carr^Mitochondrial carrier protein^202-295^E:2.2e-16	0,517	0,921	0	3,898	0	3,52	0	4,23	1	0,542	PNC1_ARATH	Peroxisomal adenine nucleotide carrier 1 (AtPNC1)	PNC1 At3g05290 T12H1.26	Arabidopsis thaliana (Mouse-ear cress)		Antiport; Complete proteome; Membrane; Peroxisome; Reference proteome; Repeat; Transmembrane; Transmembrane helix; Transport
SRR646604_TR9738_c0_g2_i5_len=2691	NA	NA	NA	NA	NA	.	1	1,785	0	4,88	0	7,364	0	7,747	1	0,614	.	.	.	.	.	.
SRR646604_TR10507_c0_g1_i1_len=1120	NA	NA	NA	NA	NA	PF06884.8^DUF1264^Protein of unknown function (DUF1264)^84-250^E:2.8e-69	0,005	4,269	0	6,112	0	7,181	0	5,858	0,118	1,659	.	.	.	.	.	.
SRR646604_TR11282_c0_g1_i1_len=1551	NA	NA	NA	NA	NA	PF04117.9^Mpv17_PMP22^Mpv17 / PMP22 family^250-311^E:1.9e-17	0,187	1,224	0	3,486	0	4,182	0	4,937	1	-0,486	.	.	.	.	.	.
SRR646604_TR22369_c0_g2_i29_len=2732	FAD3E_SOYBN	Q:801-505,H:246-321	68.69%ID	E:9e-68	Omega-3 fatty acid desaturase, endoplasmic reticulum	PF11960.5^DUF3474^Domain of unknown function (DUF3474)^6-62^E:1.6e-26`PF00487.21^FA_desaturase^Fatty acid desaturase^77-152^E:4.8e-13	0,118	2,067	0	4,846	0	5,188	0	5,431	1	-0,103	FAD3E_SOYBN	Omega-3 fatty acid desaturase, endoplasmic reticulum (EC 1.14.19.-)	FAD3	Glycine max (Soybean) (Glycine hispida)	PATHWAY: Lipid metabolism; polyunsaturated fatty acid biosynthesis.	Complete proteome; Endoplasmic reticulum; Fatty acid biosynthesis; Fatty acid metabolism; Lipid biosynthesis; Lipid metabolism; Membrane; Oxidoreductase; Reference proteome; Transmembrane; Transmembrane helix
SRR646604_TR24012_c0_g1_i1_len=426	NA	NA	NA	NA	NA	.	0,489	0,527	0	3,438	0	1,886	0	2,647	0,142	0,76	.	.	.	.	.	.
SRR646604_TR24656_c0_g2_i3_len=2411	TRPE_ARATH	Q:2056-422,H:50-595	71.48%ID	E:0	Anthranilate synthase alpha subunit 1, chloroplastic	PF04715.10^Anth_synt_I_N^Anthranilate synthase component I, N terminal region^158-312^E:1.3e-27`PF00425.15^Chorismate_bind^chorismate binding enzyme^373-645^E:3.2e-81	0,854	-0,257	0	4,651	0	3,239	0	4,255	1	0,284	TRPE_ARATH	Anthranilate synthase alpha subunit 1, chloroplastic (EC 4.1.3.27) (Anthranilate synthase component 1-1) (Anthranilate synthase component I-1) (Protein A-METHYL TRYPTOPHAN RESISTANT 1) (Protein JASMONATE-INDUCED DEFECTIVE LATERAL ROOT 1) (Protein TRYPTOPHAN BIOSYNTHESIS 5) (Protein WEAK ETHYLENE INSENSITIVE 2)	ASA1 AMT1 JDL1 TRP5 WEI2 At5g05730 MJJ3.14	Arabidopsis thaliana (Mouse-ear cress)	PATHWAY: Amino-acid biosynthesis; L-tryptophan biosynthesis; L-tryptophan from chorismate: step 1/5.	Acetylation; Alternative splicing; Amino-acid biosynthesis; Aromatic amino acid biosynthesis; Chloroplast; Complete proteome; Lyase; Plastid; Reference proteome; Transit peptide; Tryptophan biosynthesis
SRR646604_TR25379_c0_g1_i2_len=1322	NA	NA	NA	NA	NA	.	0,001	2,079	0	3,164	0	3,281	0	4,014	0,593	0,706	.	.	.	.	.	.
SRR646604_TR26737_c2_g1_i2_len=3166	CSCL4_ARATH	Q:776-2590,H:1-606	79.04%ID	E:0	CSC1-like protein At3g21620	PF13967.3^RSN1_TM^Late exocytosis, associated with Golgi transport^7-176^E:3.5e-52`PF14703.3^PHM7_cyt^Cytosolic domain of 10TM putative phosphate transporter^197-359^E:5.8e-49`PF02714.12^RSN1_7TM^Calcium-dependent channel, 7TM region, putative phosphate^370-612^E:1.3e-70	0,381	1,649	0	5,125	0,001	5,229	0	5,919	1	0,105	CSCL4_ARATH	CSC1-like protein At3g21620	At3g21620 MIL23.19	Arabidopsis thaliana (Mouse-ear cress)		Calcium; Complete proteome; Ion channel; Ion transport; Membrane; Reference proteome; Transmembrane; Transmembrane helix; Transport
SRR646604_TR26737_c2_g1_i7_len=3093	CSCL4_ARATH	Q:1814-3067,H:300-717	79.67%ID	E:0	CSC1-like protein At3g21620	PF14703.3^PHM7_cyt^Cytosolic domain of 10TM putative phosphate transporter^4-64^E:4.2e-14`PF02714.12^RSN1_7TM^Calcium-dependent channel, 7TM region, putative phosphate^75-347^E:9.3e-84	0,239	1,311	0	4,526	0	4,494	0	5,55	0,76	-0,566	CSCL4_ARATH	CSC1-like protein At3g21620	At3g21620 MIL23.19	Arabidopsis thaliana (Mouse-ear cress)		Calcium; Complete proteome; Ion channel; Ion transport; Membrane; Reference proteome; Transmembrane; Transmembrane helix; Transport
SRR646604_TR27677_c0_g1_i4_len=2620	PPH_ARATH	Q:474-1334,H:3-285	57.29%ID	E:7e-101	Pheophytinase, chloroplastic	PF00561.17^Abhydrolase_1^alpha/beta hydrolase fold^157-295^E:1.1e-11`PF12697.4^Abhydrolase_6^Alpha/beta hydrolase family^159-314^E:7.6e-13	1	0,812	0	4,643	0	8,285	0	8,771	1	-0,402	PPH_ARATH	Pheophytinase, chloroplastic (EC 3.1.1.-) (Pheophytin pheophorbide hydrolase) (Protein CO-REGULATED WITH NYE1)	PPH CRN1 At5g13800 MAC12.25	Arabidopsis thaliana (Mouse-ear cress)		Chlorophyll catabolism; Chloroplast; Complete proteome; Hydrolase; Membrane; Plastid; Reference proteome; Thylakoid; Transit peptide
SRR646604_TR27677_c0_g1_i6_len=2779	PPH_ARATH	Q:999-2414,H:3-472	59.37%ID	E:0	Pheophytinase, chloroplastic	PF00561.17^Abhydrolase_1^alpha/beta hydrolase fold^168-450^E:1.8e-13`PF12697.4^Abhydrolase_6^Alpha/beta hydrolase family^170-456^E:4.9e-18	0,502	1,99	0	5,338	0	4,753	0	5,393	1	0,169	PPH_ARATH	Pheophytinase, chloroplastic (EC 3.1.1.-) (Pheophytin pheophorbide hydrolase) (Protein CO-REGULATED WITH NYE1)	PPH CRN1 At5g13800 MAC12.25	Arabidopsis thaliana (Mouse-ear cress)		Chlorophyll catabolism; Chloroplast; Complete proteome; Hydrolase; Membrane; Plastid; Reference proteome; Thylakoid; Transit peptide
SRR646604_TR28091_c0_g2_i1_len=1576	DAT_CATRO	Q:1547-237,H:8-439	79.22%ID	E:0	Deacetylvindoline O-acetyltransferase	PF02458.12^Transferase^Transferase family^13-437^E:8.5e-65	1	2,57	0	7,308	0	7,055	0	6,949	1	0	DAT_CATRO	Deacetylvindoline O-acetyltransferase (EC 2.3.1.107) (Acetyl-coenzyme A:deacetylvindoline 4-O-acetyltransferase)	DAT	Catharanthus roseus (Madagascar periwinkle) (Vinca rosea)	PATHWAY: Alkaloid biosynthesis; vindoline biosynthesis; vindoline from tabersonine: step 6/6.	Acyltransferase; Coiled coil; Direct protein sequencing; Transferase
SRR646604_TR28167_c0_g2_i2_len=906	NA	NA	NA	NA	NA	PF05899.9^Cupin_3^Protein of unknown function (DUF861)^46-124^E:9.7e-32	0,187	1,153	0	3,85	0	2,562	0	2,926	1	-0,204	.	.	.	.	.	.
SRR646604_TR28322_c0_g1_i1_len=2020	C79D1_MANES	Q:406-1842,H:69-540	57.83%ID	E:0	Valine N-monooxygenase 1	PF00067.19^p450^Cytochrome P450^47-509^E:7.2e-76	1	0,076	0	3,357	0	7,366	0	8,243	0,629	1,397	C79D1_MANES	Valine N-monooxygenase 1 (EC 1.14.13.118) (Cytochrome P450 79D1)	CYP79D1	Manihot esculenta (Cassava) (Jatropha manihot)		Direct protein sequencing; Endoplasmic reticulum; Heme; Iron; Membrane; Metal-binding; Microsome; Monooxygenase; NADP; Oxidoreductase; Transmembrane; Transmembrane helix
SRR646604_TR28953_c0_g1_i1_len=1888	HQGT_RAUSE	Q:126-1565,H:5-470	79.38%ID	E:0	Hydroquinone glucosyltransferase	PF00201.15^UDPGT^UDP-glucoronosyl and UDP-glucosyl transferase^271-448^E:2.8e-21	0,052	1,494	0	5,318	0	2,294	0	2,587	0,02	1,118	HQGT_RAUSE	Hydroquinone glucosyltransferase (EC 2.4.1.218) (Arbutin synthase)	AS	Rauvolfia serpentina (Serpentine wood) (Ophioxylon serpentinum)		Direct protein sequencing; Glycosyltransferase; Transferase
						PF05368.10^NmrA^NmrA-like family^8-169^E:6e-42`PF01370.18^Epimerase^NAD dependent																

SRR646604_TR29137_c0_g1_i10_len=1773	EGS1_OCIBA	Q:108-632,H:7-181	69.71%ID	E:5e-81	Eugenol synthase 1	epimerase/dehydratase family^8-86^E:5.1e-06`PF01073.16^3Beta_HSD^3-beta hydroxysteroid dehydrogenase/isomerase family^9-98^E:1.2e-05`PF13460.3^NAD_binding_10^NAD(P)H-binding^12-149^E:1.6e-12	0,015	1,744	0	2,839	0	5,954	0	6,135	1	0,392	EGS1_OCIBA	Eugenol synthase 1 (EC 1.1.1.318)	EGS1	Ocimum basilicum (Sweet basil)	PATHWAY: Aromatic compound metabolism; phenylpropanoid biosynthesis.	3D-structure; NADP; Oxidoreductase; Phenylpropanoid metabolism
SRR646604_TR29855_c0_g1_i1_len=1229	DIOX3_PAPSO	Q:208-768,H:171-358	54.79%ID	E:1e-66	Codeine O-demethylase	PF03171.17^2OG-FeII_Oxy^2OG-Fe(II) oxygenase superfamily^29-125^E:7e-26	0,254	4,492	0	7,018	0	8,684	0	8,047	1	0	DIOX3_PAPSO	Codeine O-demethylase (EC 1.14.11.32)	CODM DIOX3	Papaver somniferum (Opium poppy)	PATHWAY: Alkaloid biosynthesis; morphine biosynthesis.	Alkaloid metabolism; Dioxygenase; Iron; Metal-binding; Methyltransferase; Oxidoreductase; Transferase
SRR646604_TR29872_c1_g1_i8_len=3925	C79D3_LOTJA	Q:176-1624,H:45-530	53.89%ID	E:3e-175	Isoleucine N-monooxygenase 1	PF00067.19^p450^Cytochrome P450^43-510^E:3.4e-84	1	-0,162	0	2,278	0	5,325	0	6,664	0,798	-0,455	C79D3_LOTJA	Isoleucine N-monooxygenase 1 (EC 1.14.13.117) (Cytochrome P450 79D3)	CYP79D3	Lotus japonicus (Lotus corniculatus var. japonicus)		Endoplasmic reticulum; Heme; Iron; Membrane; Metal-binding; Microsome; Monooxygenase; NADP; Oxidoreductase; Transmembrane; Transmembrane helix
SRR646604_TR30246_c1_g1_i3_len=2865	LOX31_SOLTU	Q:2778-49,H:1-914	79.35%ID	E:0	Linoleate 13S-lipoxygenase 3-1, chloroplastic	PF01477.20^PLAT^PLAT/LH2 domain^157-242^E:8e-13`PF00305.16^Lipoxygenase^Lipoxygenase^255-922^E:5.1e-303	0,088	1,564	0	4,912	0	3,981	0	4,539	1	0	LOX31_SOLTU	Linoleate 13S-lipoxygenase 3-1, chloroplastic (EC 1.13.11.12)	LOX3.1 LOX-H3	Solanum tuberosum (Potato)	PATHWAY: Lipid metabolism; oxylipin biosynthesis. {ECO:0000255|PROSITE-ProRule:PRU00726}.	Chloroplast; Complete proteome; Dioxygenase; Fatty acid biosynthesis; Fatty acid metabolism; Iron; Lipid biosynthesis; Lipid metabolism; Metal-binding; Oxidoreductase; Oxylipin biosynthesis; Plastid; Reference proteome; Thylakoid; Transit peptide
SRR646604_TR31106_c0_g1_i1_len=1331	NA	NA	NA	NA	NA	.	0,502	2,317	0	6,067	0	7,562	0	8,749	1	-0,305	.	.	.	.	.	.
SRR646604_TR33445_c0_g2_i1_len=1210	NA	NA	NA	NA	NA	PF02519.11^Auxin_inducible^Auxin responsive protein^84-154^E:4.4e-21	0,461	1,256	0	3,351	0	3,775	0	4,488	1	-0,086	.	.	.	.	.	.
SRR646604_TR37105_c3_g1_i5_len=4576	SDP1_ARATH	Q:1037-2467,H:1-474	83.05%ID	E:0	Triacylglycerol lipase SDP1	PF11815.5^DUF3336^Domain of unknown function (DUF3336)^99-229^E:2.7e-29`PF01734.19^Patatin^Patatin-like phospholipase^236-430^E:1.2e-18	0,312	0,92	0	2,379	0	5,785	0	6,023	0,798	0,348	SDP1_ARATH	Triacylglycerol lipase SDP1 (EC 3.1.1.3) (Protein SUGAR-DEPENDENT 1)	SDP1 At5g04040 F8F6.10 F8F6.250	Arabidopsis thaliana (Mouse-ear cress)		Complete proteome; Glycerol metabolism; Glycoprotein; Hydrolase; Lipid degradation; Lipid droplet; Lipid metabolism; Membrane; Reference proteome; Transmembrane; Transmembrane helix
SRR646604_TR37251_c0_g1_i1_len=852	MIRA_SYNDU	Q:119-676,H:30-217	38.14%ID	E:2e-31	Miraculin	PF00197.15^Kunitz_legume^Trypsin and protease inhibitor^46-224^E:3.4e-47	0,502	2,962	0	6,93	0	11,361	0	7,397	1	0	MIRA_SYNDU	Miraculin (MIR)		Synsepalum dulcificum (Miracle fruit) (Richadella dulcifica)		Direct protein sequencing; Disulfide bond; Glycoprotein; Signal; Taste-modifying protein
SRR646604_TR38185_c0_g1_i1_len=1662	NAC72_ARATH	Q:135-881,H:1-241	73.02%ID	E:2e-122	NAC domain-containing protein 72	PF02365.12^NAM^No apical meristem (NAM) protein^59-183^E:8e-26	0,694	0,395	0	1,927	0	2,078	0	2,337	0,505	0,392	NAC72_ARATH	NAC domain-containing protein 72 (ANAC072) (Protein RESPONSIVE TO DESICCATION 26)	NAC072 RD26 At4g27410 F27G19.10	Arabidopsis thaliana (Mouse-ear cress)		Alternative splicing; Complete proteome; DNA-binding; Nucleus; Reference proteome; Transcription; Transcription regulation
SRR646604_TR38644_c1_g1_i8_len=3869	AFS1_MALDO	Q:1177-314,H:274-563	54.3%ID	E:3e-95	(E,E)-alpha-farnesene synthase	PF03936.13^Terpene_synth_C^Terpene synthase family, metal binding domain^1-158^E:7.1e-43	1	2,333	0	6,834	0	7,73	0	8,737	1	-0,245	AFS1_MALDO	(E,E)-alpha-farnesene synthase (MdASF1) (EC 4.2.3.46) (Prenyltransferase) (EC 2.5.1.-)	AFS1 AFAR	Malus domestica (Apple) (Pyrus malus)		Cytoplasm; Lyase; Magnesium; Manganese; Metal-binding; Potassium; Transferase
SRR648705_TR7373_c0_g1_i1_len=1827	NAC2_ARATH	Q:1522-626,H:2-289	62.21%ID	E:4e-111	NAC domain-containing protein 2	PF02365.12^NAM^No apical meristem (NAM) protein^22-145^E:4.8e-27	0,01	2,599	0	8,341	0	3,604	0	4,99	1	0,883	NAC2_ARATH	NAC domain-containing protein 2 (ANAC002)	NAC002 ATAF1 At1g01720 T1N6.12	Arabidopsis thaliana (Mouse-ear cress)		Complete proteome; DNA-binding; Nucleus; Reference proteome; Transcription; Transcription regulation
SRR648705_TR9374_c0_g2_i2_len=2661	HSP83_IPONI	Q:2440-329,H:1-703	95.88%ID	E:0	Heat shock protein 83	PF13589.3^HATPase_c_3^Histidine kinase-, DNA gyrase B-, and HSP90-like ATPase^30-151^E:2.8e-09`PF02518.23^HATPase_c^Histidine kinase-, DNA gyrase B-, and HSP90-like ATPase^32-186^E:1.1e-16`PF00183.15^HSP90^Hsp90 protein^189-699^E:1.2e-241	0,781	-0,422	0,001	2,473	0	1,17	0,001	1,065	1	0,2	HSP83_IPONI	Heat shock protein 83	HSP83A	Ipomoea nil (Japanese morning glory) (Pharbitis nil)		ATP-binding; Chaperone; Cytoplasm; Nucleotide-binding; Stress response
SRR648705_TR11052_c0_g1_i3_len=1379	CBSX1_ARATH	Q:974-465,H:65-234	74.12%ID	E:8e-85	CBS domain-containing protein CBSX1, chloroplastic	PF00571.25^CBS^CBS domain^76-130^E:8.2e-13`PF00571.25^CBS^CBS domain^170-223^E:7.4e-18	0,004	1,66	0	3,718	0	3,046	0	2,168	0,226	2,682	CBSX1_ARATH	CBS domain-containing protein CBSX1, chloroplastic (CBS domain-containing protein 2) (AtCDCP2) (Protein LOSS OF THE TIMING OF ET AND JA BIOSYNTHESIS 2) (AtLEJ2)	CBSX1 CDCP2 At4g36910 AP22.61 C7A10.450	Arabidopsis thaliana (Mouse-ear cress)		3D-structure; Acetylation; CBS domain; Chloroplast; Complete proteome; Plastid; Reference proteome; Repeat; Transit peptide
SRR648705_TR12139_c0_g1_i1_len=4526	VSR1_ARATH	Q:4162-2318,H:3-621	76.9%ID	E:0	Vacuolar-sorting receptor 1	PF02225.19^PA^PA domain^92-161^E:2.1e-09`PF12662.4^cEGF^Complement Clr-like EGF-like^498-517^E:3.7e-07`PF07645.12^EGF_CA^Calcium-binding EGF domain^514-545^E:0.00012	0,409	0,4	0	2,525	0	1,848	0	2,277	0,691	0,255	VSR1_ARATH	Vacuolar-sorting receptor 1 (AtVSR1) (BP80-like protein b) (AtBP80b) (Epidermal growth factor receptor-like protein 1) (AtELP) (AtELP1) (Spot 3 protein)	VSR1 BP80B ELP ELP1 At3g52850 F8J2.20	Arabidopsis thaliana (Mouse-ear cress)		3D-structure; Calcium; Complete proteome; Cytoplasmic vesicle; Disulfide bond; EGF-like domain; Glycoprotein; Golgi apparatus; Membrane; Protein transport; Reference proteome; Repeat; Signal; Transmembrane; Transmembrane helix; Transport
SRR648705_TR12587_c0_g1_i1_len=2064	PTR21_ARATH	Q:1838-75,H:31-617	53.3%ID	E:0	Protein NRT1/ PTR FAMILY 2.13	PF07690.13^MFS_1^Major Facilitator Superfamily^115-580^E:7.8e-09`PF00854.18^PTR2^POT family^170-599^E:9.8e-82	1	1,428	0	7,148	0	3,545	0	2,623	1	0,377	PTR21_ARATH	Protein NRT1/ PTR FAMILY 2.13 (AtNPF2.13) (Nitrate transporter 1.7)	NPF2.13 NRT1.7 At1g69870 T17F3.10	Arabidopsis thaliana (Mouse-ear cress)		Cell membrane; Complete proteome; Membrane; Nitrate assimilation; Reference proteome; Symport; Transmembrane; Transmembrane helix; Transport
SRR648705_TR13222_c0_g2_i1_len=584	NA	NA	NA	NA	NA	.	0,059	1,553	0	3,607	0	6,151	0	6,57	1	-0,167	.	.	.	.	.	.
SRR648705_TR15518_c0_g1_i1_len=1550	NA	NA	NA	NA	NA	PF06200.11^tify^tify domain^37-67^E:6.5e-18`PF09425.7^CCT_2^Divergent CCT motif^127-151^E:2.6e-13	0,014	2,841	0	5,374	0	5,673	0	5,747	0,76	-0,46	.	.	.	.	.	.
SRR648705_TR17063_c0_g2_i2_len=1722	UGT9_GARJA	Q:265-1581,H:1-437	56.59%ID	E:3e-174	Beta-D-glucosyl crocetin beta-1,6-glucosyltransferase	PF00201.15^UDPGT^UDP-glucoronosyl and UDP-glucosyl transferase^263-395^E:9.9e-21	1	0,376	0	3,566	0	1,452	0	2,38	0,643	-0,455	UGT9_GARJA	Beta-D-glucosyl crocetin beta-1,6-glucosyltransferase (EC 2.4.1.330) (UDP-glucose glucosyltransferase 9) (GjUGT9) (UDP-glycosyltransferase 94E5)	UGT94E5 UGT9	Gardenia jasminoides (Cape jasmine) (Gardenia augusta)		Glycosyltransferase; Transferase
SRR648705_TR18211_c0_g1_i2_len=900	SGRM_PEA	Q:292-771,H:1-159	75.78%ID	E:4e-76	Non-functional protein STAY-GREEN, chloroplastic	PF12638.4^Staygreen^Staygreen protein^54-171^E:1.4e-35	1	0,911	0	5,386	0	3,844	0	3,744	1	0,382	SGRM_PEA	Non-functional protein STAY-GREEN, chloroplastic (Protein STAYGREEN)	SGR	Pisum sativum (Garden pea)		Chloroplast; Plastid; Transit peptide
SRR648705_TR18346_c2_g1_i1_len=2673	NA	NA	NA	NA	NA	.	1	0,705	0	4,935	0	1,566	0	2,076	0,629	1,609	.	.	.	.	.	.
SRR648705_TR25572_c0_g2_i1_len=721	NA	NA	NA	NA	NA	.	0,402	1,088	0	2,32	0	3,52	0	2,321	0,074	0,98	.	.	.	.	.	.
SRR648705_TR26993_c0_g1_i2_len=2232	CBP22_HORVU	Q:468-1766,H:1-436	70.18%ID	E:0	Serine carboxypeptidase II-2	PF00450.19^Peptidase_S10^Serine carboxypeptidase^45-461^E:5.7e-147	0,031	0,652	0	1,425	0	2,533	0	2,813	0,439	0,329	CBP22_HORVU	Serine carboxypeptidase II-2 (EC 3.4.16.6) (CP-MII.2) [Cleaved into: Serine carboxypeptidase II-2 chain A; Serine carboxypeptidase II-2 chain B] (Fragment)	CXP;2-2	Hordeum vulgare (Barley)		Carboxypeptidase; Direct protein sequencing; Disulfide bond; Glycoprotein; Hydrolase; Protease; Zymogen
SRR648705_TR33071_c0_g1_i1_len=1721	NA	NA	NA	NA	NA	PF00564.21^PB1^PB1 domain^29-114^E:6.1e-17	1	0,543	0	4,217	0	3,915	0	4,737	1	0,057	.	.	.	.	.	.
SRR648705_TR34167_c1_g1_i1_len=1738	NA	NA	NA	NA	NA	PF04720.9^PDDEXK_6^PDDEXK-like family of unknown function^61-248^E:9.1e-65	0,025	3,566	0	5,491	0	4,071	0	3,786	1	1,504	.	.	.	.	.	.
SRR648705_TR38568_c0_g1_i2_len=675	NA	NA	NA	NA	NA	.	0,003	3,077	0	3,847	0	5,017	0,001	3,774	1	-0,149	.	.	.	.	.	.
SRR648705_TR38919_c0_g2_i4_len=2033	NOTUM_MOUSE	Q:1758-721,H:107-456	27.32%ID	E:2e-21	Palmitoleoyl-protein carboxylesterase NOTUM {ECO:0000250|UniProtKB:Q6P988}	PF03283.10^PAE^Pectinacetylesterase^21-372^E:2.1e-168	0,679	0,452	0	2,335	0	1,223	0,001	1,097	1	-0,285	NOTUM_MOUSE	Palmitoleoyl-protein carboxylesterase NOTUM (EC 3.1.1.-)	Notum	Mus musculus (Mouse)		Alternative splicing; Complete proteome; Glycoprotein; Hydrolase; Phosphoprotein; Reference proteome; Secreted; Serine esterase; Signal; Wnt signaling pathway
SRR648705_TR42618_c0_g1_i1_len=1167	OFP8_ARATH	Q:312-112,H:154-220	64.18%ID	E:4e-21	Transcription repressor OFP8	PF04844.10^Ovate^Transcriptional repressor, ovate^243-299^E:1.8e-20	1	0,086	0	6,258	0	5,678	0	6,951	1	0,194	OFP8_ARATH	Transcription repressor OFP8 (Ovate family protein 8) (AtOFP8)	OFP8 At5g19650 T29J13	Arabidopsis thaliana (Mouse-ear cress)		Complete proteome; Nucleus; Reference proteome; Repressor; Transcription; Transcription regulation
SRR648705_TR43591_c0_g1_i19_len=3515	NA	NA	NA	NA	NA	PF12146.5^Hydrolase_4^Serine aminopeptidase, S33^37-157^E:2.2e-34`PF00561.17^Abhydrolase_1^alpha/beta hydrolase fold^39-148^E:1.1e-11`PF12697.4^Abhydrolase_6^Alpha/beta hydrolase family^43-149^E:1.5e-09	0,044	2,036	0	4,385	0	3,363	0	3,609	1	-0,015	.	.	.	.	.	.
SRR648705_TR44780_c0_g1_i1_len=1654	QOR1_ECOLI	Q:234-1205,H:1-327	47.4%ID	E:1e-89	Quinone oxidoreductase 1	PF08240.9^ADH_N^Alcohol dehydrogenase GroES-like domain^106-164^E:2.5e-08`PF00107.23^ADH_zinc_N^Zinc-binding dehydrogenase^229-342^E:3e-27`PF13602.3^ADH_zinc_N_2^Zinc-binding dehydrogenase^262-399^E:1.1e-20	0,352	0,323	0	1,525	0	2,372	0	2,468	0,931	0,075	QOR1_ECOLI	Quinone oxidoreductase 1 (EC 1.6.5.5) (NADPH:quinone reductase 1) (Zeta-crystallin homolog protein)	qorA hcz qor qor1 b4051 JW4011	Escherichia coli (strain K12)		3D-structure; Complete proteome; NADP; Oxidoreductase; Reference proteome
SRR648707_TR337_c1_g1_i1_len=1430	Y8948_DICDI	Q:817-53,H:15-261	32.32%ID	E:1e-30	Putative methyltransferase DDB_G0268948	PF13649.3^Methyltransf_25^Methyltransferase domain^37-128^E:9.1e-10`PF13847.3^Methyltransf_31^Methyltransferase domain^37-138^E:4.5e-08`PF08241.9^Methyltransf_11^Methyltransferase domain^39-132^E:1.3e-10`PF08242.9^Methyltransf_12^Methyltransferase domain^39-132^E:2.2e-08	0,237	0,44	0	2,409	0	1,971	0	2,627	0,781	0,621	Y8948_DICDI	Putative methyltransferase DDB_G0268948 (EC 2.1.1.-)	DDB_G0268948	Dictyostelium discoideum (Slime mold)		Complete proteome; Methyltransferase; Reference proteome; S-adenosyl-L-methionine; Transferase
SRR648707_TR3152_c0_g1_i1_len=1530	NAC72_ARATH	Q:139-885,H:1-241	73.02%ID	E:9e-124	NAC domain-containing protein 72	PF02365.12^NAM^No apical meristem (NAM) protein^61-185^E:4.9e-26	1	1,725	0	6,803	0	7,009	0	6,911	1	2,162	NAC72_ARATH	NAC domain-containing protein 72 (ANAC072) (Protein RESPONSIVE TO DESICCATION 26)	NAC072 RD26 At4g27410 F27G19.10	Arabidopsis thaliana (Mouse-ear cress)		Alternative splicing; Complete proteome; DNA-binding; Nucleus; Reference proteome; Transcription; Transcription regulation
SRR648707_TR3906_c1_g1_i2_len=1814	SRG1_ARATH	Q:1606-599,H:16-358	39.19%ID	E:7e-69	Protein SRG1	PF14226.3^DIOX_N^non-haem dioxygenase in morphine synthesis N-terminal^53-158^E:1e-25`PF03171.17^2OG-FeII_Oxy^2OG-Fe(II) oxygenase superfamily^207-303^E:4.6e-23	0,016	2,313	0	5,542	0	4,248	0	3,122	1	0	SRG1_ARATH	Protein SRG1 (AtSRG1) (Protein SENESCENCE-RELATED GENE 1)	SRG1 At1g17020 F20D23.28 F6I1.30	Arabidopsis thaliana (Mouse-ear cress)		Complete proteome; Iron; Metal-binding; Oxidoreductase; Reference proteome
SRR648707_TR4594_c0_g2_i1_len=986	IKU2_ARATH	Q:259-981,H:24-272	48.8%ID	E:7e-65	Receptor-like protein kinase HAIKU2	PF08263.9^LRRNT_2^Leucine rich repeat N-terminal domain^40-78^E:2e-10`PF13855.3^LRR_8^Leucine rich repeat^206-265^E:1.5e-06`PF00560.30^LRR_1^Leucine Rich Repeat^207-228^E:0.62	0,286	1,02	0	6,819	0	7,329	0	7,207	1	-0,743	IKU2_ARATH	Receptor-like protein kinase HAIKU2 (EC 2.7.11.1)	IKU2 At3g19700 MMB12.19	Arabidopsis thaliana (Mouse-ear cress)		ATP-binding; Complete proteome; Glycoprotein; Kinase; Leucine-rich repeat; Membrane; Nucleotide-binding; Phosphoprotein; Receptor; Reference proteome; Repeat; Serine/threonine-protein kinase; Signal; Transferase; Transmembrane; Transmembrane helix
SRR648707_TR9115_c0_g1_i1_len=463	NA	NA	NA	NA	NA	.	0,129	4,993	0	5,555	0	5,167	0	5,419	1	-3,182	.	.	.	.	.	.
SRR648707_TR9982_c0_g1_i1_len=1004	NA	NA	NA	NA	NA	.	1	-2,698	0	7,591	0	5,731	0	4,502	1	0,56	.	.	.	.	.	.
SRR648707_TR12944_c0_g1_i1_len=910	PTI5_SOLLC	Q:793-347,H:1-143	62.82%ID	E:8e-42	Pathogenesis-related genes transcriptional activator PTI5	PF00847.17^AP2^AP2 domain^66-116^E:6e-15	0,111	1,453	0	5,11	0	6,736	0	7,744	1	1,243	PTI5_SOLLC	Pathogenesis-related genes transcriptional activator PTI5 (PTO-interacting protein 5)	PTI5	Solanum lycopersicum (Tomato) (Lycopersicon esculentum)		Activator; Complete proteome; DNA-binding; Nucleus; Plant defense; Reference proteome; Transcription; Transcription regulation
SRR648707_TR15942_c0_g1_i1_len=1178	RFS6_ARATH	Q:804-40,H:1-263	73%ID	E:5e-133	Probable galactinol--sucrose galactosyltransferase 6	PF05691.9^Raffinose_syn^Raffinose synthase or seed imbibition protein Sip1^108-365^E:7.9e-107	0,856	0,08	0	3,744	0	1,548	0	1,768	0,402	1,204	RFS6_ARATH	Probable galactinol--sucrose galactosyltransferase 6 (EC 2.4.1.82) (Protein DARK INDUCIBLE 10) (Raffinose synthase 6)	RFS6 DIN10 RS6 At5g20250 F5O24.140	Arabidopsis thaliana (Mouse-ear cress)		Alternative splicing; Carbohydrate metabolism; Complete proteome; Glycosyltransferase; Reference proteome; Transferase
SRR648707_TR16177_c0_g2_i1_len=1206	NA	NA	NA	NA	NA	PF13639.3^zf-RING_2^Ring finger domain^115-160^E:3.9e-11`PF17123.2^zf-RING_11^RING-like zinc finger^115-145^E:6.3e-07`PF14634.3^zf-RING_5^zinc-RING finger domain^115-161^E:1.1e-06`PF13923.3^zf-C3HC4_2^Zinc finger, C3HC4 type (RING finger)^116-159^E:8.2e-07`PF00097.22^zf-C3HC4^Zinc finger, C3HC4 type (RING finger)^116-159^E:1.4e-05`PF12861.4^zf-ANAPC11^Anaphase-promoting complex subunit 11 RING-H2 finger^116-164^E:3.6e-05`PF13445.3^zf-RING_UBOX^RING-type zinc-finger^116-157^E:0.00092	0,133	1,13	0	3,788	0	4,196	0	4,696	1	-0,486	.	.	.	.	.	.
SRR648707_TR20187_c0_g3_i6_len=4057	MATE3_ARATH	Q:772-2133,H:60-541	60.58%ID	E:5e-165	.	PF01554.15^MatE^MatE^122-289^E:5.8e-11`PF14667.3^Polysacc_synt_C^Polysaccharide biosynthesis C-terminal domain^247-338^E:5.6e-07`PF01554.15^MatE^MatE^361-491^E:7.5e-12	0,014	1,804	0	4,283	0	3,866	0	4,086	0,444	0,477	DTX45_ARATH	Protein DETOXIFICATION 45, chloroplastic (AtDTX45) (Multidrug and toxic compound extrusion protein 45) (MATE protein 45)	DTX45 At4g38380 F22I13.150	Arabidopsis thaliana (Mouse-ear cress)		Chloroplast; Complete proteome; Membrane; Plastid; Reference proteome; Transit peptide; Transmembrane; Transmembrane helix; Transport
SRR648707_TR22697_c0_g1_i1_len=1576	DIOX3_PAPSO	Q:1078-425,H:140-358	54.34%ID	E:9e-86	Codeine O-demethylase	PF03171.17^2OG-FeII_Oxy^2OG-Fe(II) oxygenase superfamily^85-181^E:1.4e-25	0,129	4,163	0	6,722	0	8,369	0	7,459	1	0	DIOX3_PAPSO	Codeine O-demethylase (EC 1.14.11.32)	CODM DIOX3	Papaver somniferum (Opium poppy)	PATHWAY: Alkaloid biosynthesis; morphine biosynthesis.	Alkaloid metabolism; Dioxygenase; Iron; Metal-binding; Methyltransferase; Oxidoreductase; Transferase
SRR648707_TR22784_c0_g2_i2_len=1914	NAC2_ARATH	Q:657-1520,H:1-289	69.49%ID	E:4e-133	NAC domain-containing protein 2	PF02365.12^NAM^No apical meristem (NAM) protein^8-131^E:1.4e-27	0,032	1,292	0	3,568	0	3,273	0	3,67	0,444	0,467	NAC2_ARATH	NAC domain-containing protein 2 (ANAC002)	NAC002 ATAF1 At1g01720 T1N6.12	Arabidopsis thaliana (Mouse-ear cress)		Complete proteome; DNA-binding; Nucleus; Reference proteome; Transcription; Transcription regulation
SRR648707_TR23814_c0_g1_i1_len=2005	CNR8_MAIZE	Q:342-917,H:38-233	68.5%ID	E:2e-87	Cell number regulator 8	PF04749.14^PLAC8^PLAC8 family^71-209^E:5.9e-21	0,506	0,212	0	1,569	0	1,275	0	1,197	0,816	0,112	CNR8_MAIZE	Cell number regulator 8 (ZmCNR08)	CNR8 SAT5	Zea mays (Maize)		Complete proteome; Membrane; Reference proteome; Transmembrane; Transmembrane helix
SRR648707_TR23832_c1_g1_i1_len=1601	NA	NA	NA	NA	NA	PF04640.11^PLATZ^PLATZ transcription factor^69-140^E:2.8e-29	0,003	1,438	0	2,249	0	2,516	0	2,565	0,174	0,567	.	.	.	.	.	.
SRR648707_TR25545_c1_g1_i1_len=1181	NA	NA	NA	NA	NA	.	0,066	3,071	0	4,827	0	3,516	0	3,29	0,461	1,121	.	.	.	.	.	.
SRR648707_TR27118_c0_g1_i2_len=1921	MDAR_SOLLC	Q:122-1423,H:1-433	86.41%ID	E:0	Monodehydroascorbate reductase	PF07992.11^Pyr_redox_2^Pyridine nucleotide-disulphide oxidoreductase^47-362^E:6e-56`PF00070.24^Pyr_redox^Pyridine nucleotide-disulphide oxidoreductase^205-287^E:9.7e-14	0,22	0,313	0	2,302	0	1,66	0	1,665	0,034	-0,567	MDAR_SOLLC	Monodehydroascorbate reductase (MDAR) (EC 1.6.5.4) (Ascorbate free radical reductase) (AFR reductase)	AFRR	Solanum lycopersicum (Tomato) (Lycopersicon esculentum)		Complete proteome; Cytoplasm; FAD; Flavoprotein; NAD; Oxidoreductase; Redox-active center; Reference proteome
SRR648707_TR29077_c0_g2_i1_len=629	NA	NA	NA	NA	NA	.	0,189	2,076	0	6,342	0	6,759	0	6,93	0,356	-1,103	.	.	.	.	.	.
SRR648707_TR30848_c0_g1_i1_len=1760	AB7I_ARATH	Q:1701-226,H:1-475	54.36%ID	E:2e-173	Protein ABCI7, chloroplastic	PF01458.14^UPF0051^Uncharacterized protein family (UPF0051)^242-467^E:2.2e-59	0,292	0,271	0,001	0,94	0	0,891	0,001	0,876	0,143	0,499	AB7I_ARATH	Protein ABCI7, chloroplastic (ABC transporter I family member 7) (ABC transporter ABCI.7) (AtABCI7) (Non-intrinsic ABC protein 6) (Plastid SufD-like protein)	ABCI7 NAP6 At1g32500 F5D14.28	Arabidopsis thaliana (Mouse-ear cress)		Chloroplast; Complete proteome; Plastid; Reference proteome; Transit peptide
SRR648707_TR32619_c0_g1_i1_len=465	NA	NA	NA	NA	NA	.	0,132	2,615	0	4,528	0	5,285	0	5,482	1	0	.	.	.	.	.	.
SRR648707_TR33467_c0_g1_i1_len=2068	C81E1_GLYEC	Q:1822-479,H:54-493	47.02%ID	E:2e-147	Isoflavone 2'-hydroxylase	PF00067.19^p450^Cytochrome P450^30-485^E:1.8e-91	0,021	1,652	0	4,825	0	3,744	0	3,242	1	0,211	C81E1_GLYEC	Isoflavone 2'-hydroxylase (EC 1.14.13.89) (CYP GE-3) (Cytochrome P450 81E1) (Cytochrome P450 91A4)	CYP81E1 CYP91A4	Glycyrrhiza echinata (Licorice)		Heme; Iron; Membrane; Metal-binding; Monooxygenase; NADP; Oxidoreductase
SRR648707_TR34170_c0_g1_i1_len=611	NA	NA	NA	NA	NA	.	0,564	0,448	0	3,51	0	5,478	0	5,928	0,402	-1,169	.	.	.	.	.	.
SRR648707_TR34684_c0_g2_i2_len=2163	C81E8_MEDTR	Q:510-1436,H:176-490	45.37%ID	E:2e-105	Cytochrome P450 81E8 {ECO:0000303|PubMed:14617078}	PF00067.19^p450^Cytochrome P450^25-290^E:5.5e-67	1	-0,039	0	3,352	0	4,58	0	5,652	0,56	0,902	C81E8_MEDTR	Cytochrome P450 81E8 (EC 1.14.13.-)	CYP81E8	Medicago truncatula (Barrel medic) (Medicago tribuloides)		Heme; Iron; Membrane; Metal-binding; Monooxygenase; NADP; Oxidoreductase; Transmembrane; Transmembrane helix
SRR648707_TR36394_c2_g1_i5_len=1389	PIP27_MAIZE	Q:155-1018,H:1-287	82.01%ID	E:5e-150	Aquaporin PIP2-7	PF00230.17^MIP^Major intrinsic protein^34-269^E:3.2e-83	0,043	3,29	0	8,934	0	5,884	0	4,596	1	1,168	PIP27_MAIZE	Aquaporin PIP2-7 (Plasma membrane intrinsic protein 2-7) (ZmPIP2-7) (ZmPIP2;7)	PIP2-7	Zea mays (Maize)		Cell membrane; Complete proteome; Membrane; Reference proteome; Repeat; Transmembrane; Transmembrane helix; Transport
SRR648707_TR37199_c0_g1_i3_len=1848	NA	NA	NA	NA	NA	PF03547.15^Mem_trans^Membrane transport protein^10-408^E:6.7e-83	0,381	0,786	0	2,995	0	5,115	0	4,742	1	-0,025	.	.	.	.	.	.
SRR648707_TR37528_c0_g1_i1_len=1469	ATL51_ARATH	Q:230-1066,H:1-296	45.87%ID	E:3e-61	RING-H2 finger protein ATL51	PF13639.3^zf-RING_2^Ring finger domain^134-177^E:8.4e-14`PF17123.2^zf-RING_11^RING-like zinc finger^134-163^E:3.6e-10`PF13923.3^zf-C3HC4_2^Zinc finger, C3HC4 type (RING finger)^134-176^E:1.1e-06`PF00097.22^zf-C3HC4^Zinc finger, C3HC4 type (RING finger)^135-176^E:0.00011	0,006	1,726	0	2,336	0	1,803	0	1,742	0,299	1,549	ATL51_ARATH	RING-H2 finger protein ATL51	ATL51 At3g03550 T12J13.17 T21P5.3	Arabidopsis thaliana (Mouse-ear cress)	PATHWAY: Protein modification; protein ubiquitination.	Complete proteome; Membrane; Metal-binding; Reference proteome; Transmembrane; Transmembrane helix; Ubl conjugation pathway; Zinc; Zinc-finger
SRR648707_TR39385_c1_g1_i11_len=2270	DCAM_CATRO	Q:710-1780,H:1-357	100%ID	E:0	S-adenosylmethionine decarboxylase proenzyme	PF01536.13^SAM_decarbox^Adenosylmethionine decarboxylase^9-332^E:9.8e-110	0,107	0,407	0	1,727	0	0,867	0	1,025	0,674	0,105	DCAM_CATRO	S-adenosylmethionine decarboxylase proenzyme (AdoMetDC) (SAMDC) (EC 4.1.1.50) [Cleaved into: S-adenosylmethionine decarboxylase alpha chain; S-adenosylmethionine decarboxylase beta chain]	SAMDC	Catharanthus roseus (Madagascar periwinkle) (Vinca rosea)	PATHWAY: Amine and polyamine biosynthesis; S-adenosylmethioninamine biosynthesis; S-adenosylmethioninamine from S-adenosyl-L-methionine: step 1/1.	Autocatalytic cleavage; Decarboxylase; Lyase; Polyamine biosynthesis; Pyruvate; S-adenosyl-L-methionine; Schiff base; Spermidine biosynthesis; Zymogen
SRR648707_TR40821_c0_g1_i1_len=1711	T184C_BOVIN	Q:364-1215,H:39-325	29.93%ID	E:1e-35	Transmembrane protein 184C	PF03619.13^Solute_trans_a^Organic solute transporter Ostalpha^9-275^E:1e-68	0,002	0,973	0	3,555	0	2,256	0	2,103	0,189	0,553	T184C_BOVIN	Transmembrane protein 184C (Transmembrane protein 34)	TMEM184C TMEM34	Bos taurus (Bovine)		Complete proteome; Membrane; Reference proteome; Transmembrane; Transmembrane helix
SRR924147_TR2336_c0_g1_i1_len=1470	HIDM_GLYEC	Q:200-1144,H:12-327	47.37%ID	E:9e-98	2-hydroxyisoflavanone dehydratase	PF00135.25^COesterase^Carboxylesterase family^58-179^E:3.3e-07`PF07859.10^Abhydrolase_3^alpha/beta hydrolase fold^73-299^E:8.4e-38	0,06	0,807	0	2,47	0	2,274	0	2,352	0,458	0,451	HIDM_GLYEC	2-hydroxyisoflavanone dehydratase (EC 3.1.1.1) (EC 4.2.1.105) (Carboxylesterase HIDM)	HIDM	Glycyrrhiza echinata (Licorice)	PATHWAY: Secondary metabolite biosynthesis; flavonoid biosynthesis.	Flavonoid biosynthesis; Hydrolase; Lyase
SRR924147_TR11424_c0_g2_i1_len=1050	CRPM2_ARATH	Q:890-285,H:1-203	65.52%ID	E:2e-87	Cold-regulated 413 plasma membrane protein 2	PF05562.8^WCOR413^Cold acclimation protein WCOR413^9-189^E:8.3e-85	1	1,367	0	7,769	0	5,298	0	4,34	1	0	CRPM2_ARATH	Cold-regulated 413 plasma membrane protein 2 (AtCOR413-PM2)	COR413PM2 At3g50830 F18B3.110	Arabidopsis thaliana (Mouse-ear cress)		Cell membrane; Complete proteome; Membrane; Reference proteome; Transmembrane; Transmembrane helix
SRR924147_TR15016_c0_g1_i1_len=1466	ANXD1_ARATH	Q:1318-371,H:1-317	67.51%ID	E:9e-157	Annexin D1	PF00191.17^Annexin^Annexin^16-79^E:1.1e-21`PF00191.17^Annexin^Annexin^87-152^E:3.7e-17`PF00191.17^Annexin^Annexin^171-231^E:2.5e-14	0,226	0,414	0	2,356	0	2,482	0	2,04	0,007	0,933	ANXD1_ARATH	Annexin D1 (AnnAt1) (Annexin A1)	ANN1 ANNAT1 ANX23-ATH ATOXY5 OXY5 At1g35720 F14D7.2	Arabidopsis thaliana (Mouse-ear cress)		3D-structure; Acetylation; Annexin; Calcium; Calcium/phospholipid-binding; Complete proteome; Cytoplasm; Membrane; Reference proteome; Repeat; Stress response
SRR924147_TR31078_c1_g2_i2_len=2112	WRKY6_ARATH	Q:1994-546,H:1-498	50.84%ID	E:4e-98	WRKY transcription factor 6	PF03106.12^WRKY^WRKY DNA -binding domain^318-375^E:9.9e-27	0,118	2,328	0	3,797	0	4,395	0	5,02	1	0,106	WRKY6_ARATH	WRKY transcription factor 6 (WRKY DNA-binding protein 6) (AtWRKY6)	WRKY6 At1g62300 F19K23.22	Arabidopsis thaliana (Mouse-ear cress)		Activator; Complete proteome; DNA-binding; Ethylene signaling pathway; Nucleus; Reference proteome; Repressor; Transcription; Transcription regulation
SRR924147_TR32903_c0_g1_i1_len=1616	SGRW_PEA	Q:1237-515,H:1-236	71.07%ID	E:3e-115	Protein STAY-GREEN, chloroplastic	PF12638.4^Staygreen^Staygreen protein^54-205^E:3.3e-58	0,103	1,637	0	4,905	0	4,382	0	4,361	1	3,301	SGRW_PEA	Protein STAY-GREEN, chloroplastic (Protein STAYGREEN)	SGR	Pisum sativum (Garden pea)		Chloroplast; Plastid; Transit peptide
SRR924148_TR4179_c0_g2_i4_len=1472	TRPA2_ARATH	Q:266-1153,H:11-312	76.16%ID	E:6e-146	Tryptophan synthase alpha chain, chloroplastic	PF00290.17^Trp_syntA^Tryptophan synthase alpha chain^61-316^E:1.1e-95`PF00977.18^His_biosynth^Histidine biosynthesis protein^242-291^E:3.3e-06	0,001	0,781	0	2,39	0	1,073	0,001	0,945	1	0,009	TRPA2_ARATH	Tryptophan synthase alpha chain, chloroplastic (EC 4.2.1.20) (Indole-3-glycerol-phosphate lyase, chloroplastic) (EC 4.1.2.8) (Protein TRYPTOPHAN-REQUIRING 3)	TSA1 TRP3 TSA2 At3g54640 T14E10.210	Arabidopsis thaliana (Mouse-ear cress)	PATHWAY: Amino-acid biosynthesis; L-tryptophan biosynthesis; L-tryptophan from chorismate: step 5/5.	Amino-acid biosynthesis; Aromatic amino acid biosynthesis; Auxin biosynthesis; Chloroplast; Complete proteome; Lyase; Plastid; Reference proteome; Transit peptide; Tryptophan biosynthesis
SRR924148_TR9505_c0_g2_i1_len=1093	NA	NA	NA	NA	NA	.	0,155	1,13	0	2,199	0	2,255	0,001	2,157	0,643	0,442	.	.	.	.	.	.
SRR924148_TR10819_c2_g2_i1_len=1467	NA	NA	NA	NA	NA	PF00076.19^RRM_1^RNA recognition motif. (a.k.a. RRM, RBD, or RNP domain)^19-84^E:3e-08`PF13893.3^RRM_5^RNA recognition motif. (a.k.a. RRM, RBD, or RNP domain)^37-83^E:0.076	1	0,473	0	3,741	0	1,896	0	2,296	1	-0,357	.	.	.	.	.	.
SRR924148_TR12464_c0_g1_i1_len=521	NA	NA	NA	NA	NA	.	0,006	1,784	0	6,885	0	7,671	0	8,087	0,56	-0,734	.	.	.	.	.	.
SRR924148_TR16409_c0_g2_i1_len=1271	NA	NA	NA	NA	NA	.	1	1,234	0	2,342	0	1,85	0	1,871	0,017	5,796	.	.	.	.	.	.
SRR924148_TR20364_c1_g4_i12_len=3184	GATLA_ARATH	Q:866-1369,H:37-195	46.47%ID	E:3e-36	Probable galacturonosyltransferase-like 10	PF05542.8^DUF760^Protein of unknown function (DUF760)^87-165^E:1.3e-17`PF05542.8^DUF760^Protein of unknown function (DUF760)^260-363^E:1.5e-26	0,663	0,519	0	2,058	0	2,417	0	3,526	0,656	-0,32	GATLA_ARATH	Probable galacturonosyltransferase-like 10 (EC 2.4.1.-) (Galactinol synthase 8) (AtGolS8) (GolS-8)	GATL10 GOLS8 At3g28340 MZF16.17	Arabidopsis thaliana (Mouse-ear cress)	PATHWAY: Glycan metabolism; pectin biosynthesis.	Cell wall biogenesis/degradation; Complete proteome; Glycoprotein; Glycosyltransferase; Golgi apparatus; Membrane; Reference proteome; Signal-anchor; Transferase; Transmembrane; Transmembrane helix
SRR924148_TR26351_c1_g4_i1_len=2386	HSP7C_PETHY	Q:140-2086,H:1-651	92.35%ID	E:0	Heat shock cognate 70 kDa protein	PF00012.17^HSP70^Hsp70 protein^9-618^E:5e-270`PF06723.10^MreB_Mbl^MreB/Mbl protein^131-380^E:2.8e-14	0,16	1,296	0	4,402	0	1,366	0	1,205	0,798	0,291	HSP7C_PETHY	Heat shock cognate 70 kDa protein	HSP70	Petunia hybrida (Petunia)		ATP-binding; Nucleotide-binding; Stress response
SRR924148_TR26351_c2_g2_i1_len=1560	HSP72_SOLLC	Q:1558-272,H:218-644	94.41%ID	E:0	Heat shock cognate 70 kDa protein 2	PF00012.17^HSP70^Hsp70 protein^1-401^E:3.5e-157	0,212	0,509	0	1,184	0	1,07	0	0,968	0,017	-4,469	HSP72_SOLLC	Heat shock cognate 70 kDa protein 2	HSC-2	Solanum lycopersicum (Tomato) (Lycopersicon esculentum)		ATP-binding; Complete proteome; Nucleotide-binding; Reference proteome; Stress response
SRR924148_TR26518_c0_g1_i4_len=1979	SCP31_ARATH	Q:1517-186,H:16-471	65.79%ID	E:0	Serine carboxypeptidase-like 31	PF00450.19^Peptidase_S10^Serine carboxypeptidase^46-467^E:3.3e-136	0,008	2,858	0	4,626	0	2,519	0	2,269	1	-0,755	SCP31_ARATH	Serine carboxypeptidase-like 31 (EC 3.4.16.-)	SCPL31 At1g11080 T19D16.4	Arabidopsis thaliana (Mouse-ear cress)		Alternative splicing; Carboxypeptidase; Complete proteome; Disulfide bond; Glycoprotein; Hydrolase; Protease; Reference proteome; Secreted; Signal
SRR924148_TR26932_c1_g3_i1_len=1664	GSTUI_ARATH	Q:510-875,H:5-127	74.8%ID	E:3e-79	Glutathione S-transferase U18	PF02798.17^GST_N^Glutathione S-transferase, N-terminal domain^6-77^E:1.1e-16`PF13417.3^GST_N_3^Glutathione S-transferase, N-terminal domain^8-77^E:4.6e-16`PF13409.3^GST_N_2^Glutathione S-transferase, N-terminal domain^14-77^E:1.6e-07	0,889	0,073	0	3,528	0	1,276	0	1,4	0,865	-0,065	GSTUI_ARATH	Glutathione S-transferase U18 (AtGSTU18) (EC 2.5.1.18) (GST class-tau member 18) (Glutathione S-transferase 29)	GSTU18 GST29 At1g10360 F14N23.24	Arabidopsis thaliana (Mouse-ear cress)		Complete proteome; Cytoplasm; Detoxification; Reference proteome; Transferase
SRR924148_TR27396_c0_g2_i1_len=612	NA	NA	NA	NA	NA	.	0,08	1,378	0	3,489	0	3,365	0	2,968	1	-0,16	.	.	.	.	.	.
SRR924148_TR28419_c0_g3_i5_len=1480	PIP1_SOLLC	Q:347-1192,H:1-282	86.52%ID	E:5e-178	Probable aquaporin PIP-type pTOM75	PF00230.17^MIP^Major intrinsic protein^45-274^E:1e-83	0,021	0,547	0	2,265	0	1,553	0	0,861	0,74	0,084	PIP1_SOLLC	Probable aquaporin PIP-type pTOM75 (Ripening-associated membrane protein) (RAMP)		Solanum lycopersicum (Tomato) (Lycopersicon esculentum)		Cell membrane; Complete proteome; Membrane; Reference proteome; Repeat; Stress response; Transmembrane; Transmembrane helix; Transport
SRR924148_TR30626_c0_g2_i1_len=2780	MFPA_CUCSA	Q:292-2457,H:4-725	80.19%ID	E:0	Glyoxysomal fatty acid beta-oxidation multifunctional protein MFP-a	PF00378.17^ECH_1^Enoyl-CoA hydratase/isomerase^14-209^E:6.7e-40`PF16113.2^ECH_2^Enoyl-CoA hydratase/isomerase^26-192^E:3.2e-24`PF02737.15^3HCDH_N^3-hydroxyacyl-CoA dehydrogenase, NAD binding domain^312-490^E:1.5e-60`PF00725.19^3HCDH^3-hydroxyacyl-CoA dehydrogenase, C-terminal domain^493-586^E:9.3e-21	0,194	0,418	0	1,598	0	2,241	0	2,633	0,394	0,325	MFPA_CUCSA	Glyoxysomal fatty acid beta-oxidation multifunctional protein MFP-a [Includes: Enoyl-CoA hydratase/3-2-trans-enoyl-CoA isomerase/3-hydroxybutyryl-CoA epimerase (EC 4.2.1.17) (EC 5.1.2.3) (EC 5.3.3.8); 3-hydroxyacyl-CoA dehydrogenase (EC 1.1.1.35)]		Cucumis sativus (Cucumber)	PATHWAY: Lipid metabolism; fatty acid beta-oxidation.	Fatty acid metabolism; Glyoxysome; Isomerase; Lipid metabolism; Lyase; Multifunctional enzyme; NAD; Oxidoreductase; Peroxisome
SRR924148_TR37688_c0_g1_i1_len=1718	YQJG_ECOLI	Q:331-1284,H:15-328	46.77%ID	E:2e-90	Glutathionyl-hydroquinone reductase YqjG	PF13409.3^GST_N_2^Glutathione S-transferase, N-terminal domain^89-196^E:1.2e-19`PF14497.3^GST_C_3^Glutathione S-transferase, C-terminal domain^233-315^E:0.00011`PF00043.22^GST_C^Glutathione S-transferase, C-terminal domain^238-317^E:1.2e-07`PF13410.3^GST_C_2^Glutathione S-transferase, C-terminal domain^244-311^E:1.2e-09	0,239	0,446	0,001	1,248	0	1,556	0,001	1,459	0,927	0,073	YQJG_ECOLI	Glutathionyl-hydroquinone reductase YqjG (GS-HQR) (EC 1.8.-.-)	yqjG b3102 JW3073	Escherichia coli (strain K12)		3D-structure; Complete proteome; Oxidoreductase; Reference proteome
SRR924148_TR40931_c0_g1_i1_len=2638	IDN2_ARATH	Q:2174-336,H:21-637	51.61%ID	E:0	Protein INVOLVED IN DE NOVO 2 {ECO:0000303|PubMed:19915591}	PF03470.11^zf-XS^XS zinc finger domain^48-91^E:8.6e-19`PF03468.11^XS^XS domain^120-231^E:3.7e-38`PF03469.11^XH^XH domain^508-636^E:4.9e-53	0,045	0,667	0	2,707	0	2,863	0	3,358	0,84	0,227	IDN2_ARATH	Protein INVOLVED IN DE NOVO 2 (Protein RNA-DIRECTED DNA METHYLATION 12)	IDN2 RDM12 At3g48670 T8P19.180	Arabidopsis thaliana (Mouse-ear cress)		3D-structure; Coiled coil; Complete proteome; RNA-binding; RNA-mediated gene silencing; Reference proteome
SRR1271857_TR20542_c0_g1_i4_len=1910	ALMT9_ARATH	Q:501-1742,H:134-566	52.64%ID	E:3e-134	Aluminum-activated malate transporter 9	PF11744.5^ALMT^Aluminium activated malate transporter^2-392^E:2.6e-123	0,283	0,442	0,004	1,466	0	4,296	0	3,965	0	7,319	ALMT9_ARATH	Aluminum-activated malate transporter 9 (AtALMT9)	ALMT9 At3g18440 MYF24.16	Arabidopsis thaliana (Mouse-ear cress)		Chloride; Complete proteome; Ion channel; Ion transport; Membrane; Reference proteome; Transmembrane; Transmembrane helix; Transport; Vacuole
SRR1271857_TR40574_c0_g1_i3_len=1027	KIRO_ACTDE	Q:713-270,H:1-148	46.62%ID	E:1e-33	Kirola {ECO:0000303|PubMed:21309790}	PF00407.16^Bet_v_1^Pathogenesis-related protein Bet v I family^31-174^E:2.3e-32	0,907	0,026	0,874	0,036	0	0,881	0	0,759	0	1,125	KIRO_ACTDE	Kirola (allergen Act d 11)		Actinidia deliciosa (Kiwi)		3D-structure; Acetylation; Allergen; Direct protein sequencing; Polymorphism
SRR1271857_TR40877_c0_g1_i1_len=1170	ANX4_FRAAN	Q:1070-135,H:1-314	63.38%ID	E:1e-141	Annexin-like protein RJ4	PF00191.17^Annexin^Annexin^15-76^E:9.7e-20`PF00191.17^Annexin^Annexin^87-149^E:7.1e-09`PF00191.17^Annexin^Annexin^168-231^E:3.5e-11`PF00191.17^Annexin^Annexin^245-307^E:5.1e-18	1	-0,104	0,189	2	0	1,937	0	2,213	0	2,154	ANX4_FRAAN	Annexin-like protein RJ4		Fragaria ananassa (Strawberry)		Annexin; Calcium; Calcium/phospholipid-binding; Repeat
SRR1271858_TR14181_c0_g1_i2_len=692	KIRO_ACTDE	Q:650-228,H:8-148	46.81%ID	E:2e-31	Kirola {ECO:0000303|PubMed:21309790}	PF00407.16^Bet_v_1^Pathogenesis-related protein Bet v I family^18-154^E:6.5e-32	0,118	0,335	0,764	-0,071	0	1,691	0	1,068	0	0,781	KIRO_ACTDE	Kirola (allergen Act d 11)		Actinidia deliciosa (Kiwi)		3D-structure; Acetylation; Allergen; Direct protein sequencing; Polymorphism
SRR342017_TR7655_c0_g7_i10_len=2440	C76B6_CATRO	Q:999-2396,H:32-493	50.64%ID	E:3e-154	Geraniol 8-hydroxylase	PF00067.19^p450^Cytochrome P450^2-423^E:1.7e-96	0,027	2,464	0,593	0,667	0	1,245	0	1,335	0	1,472	C76B6_CATRO	Geraniol 8-hydroxylase (EC 1.14.13.152) (Cytochrome P450 76B6) (Geraniol 10-hydroxylase) (CrG10H)	CYP76B6 G10H	Catharanthus roseus (Madagascar periwinkle) (Vinca rosea)		Direct protein sequencing; Endoplasmic reticulum; Heme; Iron; Membrane; Metal-binding; Monooxygenase; NADP; Oxidoreductase; Transmembrane; Transmembrane helix
SRR342019_TR1547_c0_g2_i1_len=1144	NA	NA	NA	NA	NA	PF13833.3^EF-hand_8^EF-hand domain pair^36-96^E:3.5e-05`PF13202.3^EF-hand_5^EF hand^76-94^E:0.14	0,58	0,246	0,502	0,333	0,001	1,401	0	1,507	0,001	4,381	.	.	.	.	.	.
SRR342019_TR14878_c1_g2_i3_len=1789	DAT_CATRO	Q:73-1389,H:1-439	99.77%ID	E:0	Deacetylvindoline O-acetyltransferase	PF02458.12^Transferase^Transferase family^35-456^E:3.6e-66	0,437	0,193	0,43	0,22	0	1,74	0	1,44	0	1,026	DAT_CATRO	Deacetylvindoline O-acetyltransferase (EC 2.3.1.107) (Acetyl-coenzyme A:deacetylvindoline 4-O-acetyltransferase)	DAT	Catharanthus roseus (Madagascar periwinkle) (Vinca rosea)	PATHWAY: Alkaloid biosynthesis; vindoline biosynthesis; vindoline from tabersonine: step 6/6.	Acyltransferase; Coiled coil; Direct protein sequencing; Transferase
SRR646604_TR35379_c0_g2_i1_len=2307	PTR18_ARATH	Q:105-1883,H:1-593	55.85%ID	E:0	Protein NRT1/ PTR FAMILY 3.1	PF00854.18^PTR2^POT family^98-533^E:1.1e-94	1	0,606	1	0,804	0	1,809	0	1,723	0	3,04	PTR18_ARATH	Protein NRT1/ PTR FAMILY 3.1 (AtNPF3.1)	NPF3.1 NITR At1g68570 F24J5.19	Arabidopsis thaliana (Mouse-ear cress)		Complete proteome; Membrane; Nitrate assimilation; Reference proteome; Transmembrane; Transmembrane helix; Transport
SRR924148_TR8082_c0_g2_i1_len=1811	AAT_AQUAE	Q:407-1555,H:4-394	28.43%ID	E:2e-37	Aspartate aminotransferase	PF00155.18^Aminotran_1_2^Aminotransferase class I and II^31-380^E:1.6e-49`PF01053.17^Cys_Met_Meta_PP^Cys/Met metabolism PLP-dependent enzyme^91-207^E:9.4e-05	0,87	-0,166	0,16	-0,648	0	1,989	0	1,84	0	2,311	AAT_AQUAE	Aspartate aminotransferase (AspAT) (EC 2.6.1.1) (Transaminase A)	aspC aq_1969	Aquifex aeolicus (strain VF5)		Aminotransferase; Complete proteome; Cytoplasm; Pyridoxal phosphate; Reference proteome; Transferase
SRR1144633_TR1228_c0_g2_i1_len=1493	PUP3_ARATH	Q:357-1304,H:5-321	45.74%ID	E:6e-81	Purine permease 3	PF16913.2^PUNUT^Purine nucleobase transmembrane transport^23-345^E:4.7e-99	0	2,695	0	7,485	0	7,08	0	8,434	1	0,468	PUP3_ARATH	Purine permease 3 (AtPUP3)	PUP3 At1g28220 F3H9.12	Arabidopsis thaliana (Mouse-ear cress)		Complete proteome; Membrane; Reference proteome; Transmembrane; Transmembrane helix; Transport
SRR1144633_TR5772_c0_g3_i2_len=991	CYB5_TOBAC	Q:728-324,H:1-135	86.67%ID	E:3e-69	Cytochrome b5	PF00173.25^Cyt-b5^Cytochrome b5-like Heme/Steroid binding domain^9-80^E:1.1e-26	0	9,072	0	8,748	0	1,567	0	1,328	1	-1,052	CYB5_TOBAC	Cytochrome b5		Nicotiana tabacum (Common tobacco)		Electron transport; Endoplasmic reticulum; Heme; Iron; Membrane; Metal-binding; Microsome; Transmembrane; Transmembrane helix; Transport
SRR1144633_TR32951_c0_g1_i1_len=1954	CIPKB_ARATH	Q:1689-442,H:13-422	60.38%ID	E:5e-175	CBL-interacting serine/threonine-protein kinase 11	PF00069.22^Pkinase^Protein kinase domain^25-279^E:4.8e-76`PF07714.14^Pkinase_Tyr^Protein tyrosine kinase^27-275^E:3.6e-41`PF14531.3^Kinase-like^Kinase-like^121-264^E:2.7e-08`PF03822.11^NAF^NAF domain^315-367^E:5.5e-14	0	2,577	0	4,336	0	1,661	0	1,684	1	1,052	CIPKB_ARATH	CBL-interacting serine/threonine-protein kinase 11 (EC 2.7.11.1) (SNF1-related kinase 3.22) (SOS2-like protein kinase PKS5) (SOS3-interacting protein 4)	CIPK11 PKS5 SIP4 SnRK3.22 At2g30360 T9D9.17	Arabidopsis thaliana (Mouse-ear cress)		ATP-binding; Complete proteome; Kinase; Magnesium; Manganese; Nucleotide-binding; Phosphoprotein; Reference proteome; Serine/threonine-protein kinase; Transferase
SRR1144633_TR36204_c0_g1_i1_len=1331	NA	NA	NA	NA	NA	.	0,001	4,428	0	6,683	0	5,146	0	6,29	0,502	2,653	.	.	.	.	.	.
SRR1144634_TR9978_c0_g1_i1_len=585	NA	NA	NA	NA	NA	.	0,001	1,742	0	2,301	0	2,322	0	1,849	1	0	.	.	.	.	.	.
SRR1144634_TR10670_c0_g2_i11_len=898	ARGE_ARATH	Q:679-191,H:278-438	65.64%ID	E:2e-71	Acetylornithine deacetylase	.	0	3,061	0	4,602	0	5,701	0	4,122	0,005	0,978	ARGE_ARATH	Acetylornithine deacetylase (EC 3.5.1.16) (N-acetylornithinase) (AO) (Acetylornithinase) (NAO)	At4g17830 dl4945w T6K21	Arabidopsis thaliana (Mouse-ear cress)	PATHWAY: Amino-acid biosynthesis; L-arginine biosynthesis; L-ornithine from N(2)-acetyl-L-ornithine (linear): step 1/1.	Alternative splicing; Amino-acid biosynthesis; Arginine biosynthesis; Cobalt; Complete proteome; Hydrolase; Metal-binding; Reference proteome; Zinc
SRR1144634_TR17599_c0_g1_i1_len=761	P2B11_ARATH	Q:23-520,H:88-255	36.05%ID	E:5e-22	F-box protein PP2-B11	PF14299.3^PP2^Phloem protein 2^15-173^E:1.8e-31	0	4,363	0	9,065	0	9,084	0	6,679	1	-0,025	P2B11_ARATH	F-box protein PP2-B11 (Protein PHLOEM PROTEIN 2-LIKE B11) (AtPP2-B11) (SKP1-interacting partner 12)	PP2B11 SKIP12 At1g80110 F18B13.19	Arabidopsis thaliana (Mouse-ear cress)	PATHWAY: Protein modification; protein ubiquitination.	Complete proteome; Nucleus; Reference proteome; Ubl conjugation pathway
SRR1144634_TR28611_c0_g1_i1_len=775	NA	NA	NA	NA	NA	PF13419.3^HAD_2^Haloacid dehalogenase-like hydrolase^5-101^E:1.8e-18`PF00702.23^Hydrolase^haloacid dehalogenase-like hydrolase^6-94^E:1.6e-08`PF13242.3^Hydrolase_like^HAD-hyrolase-like^56-103^E:9.5e-07	0	2,098	0	2,763	0	2,349	0	2,541	0,025	3,634	.	.	.	.	.	.
SRR122236_TR27004_c0_g2_i2_len=735	CYTI_VIGUN	Q:93-377,H:1-95	70.53%ID	E:6e-41	Cysteine proteinase inhibitor	.	0	1,265	0	2,314	0	1,866	0	1,2	0,683	0,116	CYTI_VIGUN	Cysteine proteinase inhibitor (Cystatin)		Vigna unguiculata (Cowpea)		3D-structure; Protease inhibitor; Thiol protease inhibitor
SRR122238_TR813_c0_g1_i1_len=1167	TIP13_ARATH	Q:1022-267,H:1-252	79.76%ID	E:1e-141	Aquaporin TIP1-3	PF00230.17^MIP^Major intrinsic protein^14-234^E:6.5e-74	0	3,722	0	5,184	0	4,884	0	3,164	1	0,398	TIP13_ARATH	Aquaporin TIP1-3 (Gamma-tonoplast intrinsic protein 3) (Gamma-TIP3) (Tonoplast intrinsic protein 1-3) (AtTIP1;3) [Cleaved into: Aquaporin TIP1-3, N-terminally processed]	TIP1-3 At4g01470 F11O4.1	Arabidopsis thaliana (Mouse-ear cress)		Acetylation; Complete proteome; Membrane; Reference proteome; Repeat; Stress response; Transmembrane; Transmembrane helix; Transport; Vacuole
SRR122238_TR2609_c0_g1_i1_len=1753	ARGE_ARATH	Q:1679-375,H:1-438	68.34%ID	E:0	Acetylornithine deacetylase	PF01546.25^Peptidase_M20^Peptidase family M20/M25/M40^119-456^E:8.8e-31`PF07687.11^M20_dimer^Peptidase dimerisation domain^227-341^E:3.6e-12	0	2,44	0	3,703	0	4,817	0	4,473	0,007	1,003	ARGE_ARATH	Acetylornithine deacetylase (EC 3.5.1.16) (N-acetylornithinase) (AO) (Acetylornithinase) (NAO)	At4g17830 dl4945w T6K21	Arabidopsis thaliana (Mouse-ear cress)	PATHWAY: Amino-acid biosynthesis; L-arginine biosynthesis; L-ornithine from N(2)-acetyl-L-ornithine (linear): step 1/1.	Alternative splicing; Amino-acid biosynthesis; Arginine biosynthesis; Cobalt; Complete proteome; Hydrolase; Metal-binding; Reference proteome; Zinc
SRR122238_TR9216_c1_g1_i2_len=1145	SWT12_ARATH	Q:87-746,H:10-236	60.79%ID	E:2e-89	Bidirectional sugar transporter SWEET12 {ECO:0000303|PubMed:21107422}	PF03083.13^MtN3_slv^Sugar efflux transporter for intercellular exchange^12-96^E:4.7e-25`PF04193.11^PQ-loop^PQ loop repeat^14-67^E:9.2e-05`PF03083.13^MtN3_slv^Sugar efflux transporter for intercellular exchange^132-214^E:4e-26	0	2,902	0	3,397	0	1,639	0	1,767	0,84	0,351	SWT12_ARATH	Bidirectional sugar transporter SWEET12 (AtSWEET12) (MtN3-like protein) (Protein SUGARS WILL EVENTUALLY BE EXPORTED TRANSPORTERS 12)	SWEET12 MTN3 At5g23660 MQM1.8	Arabidopsis thaliana (Mouse-ear cress)		Cell membrane; Complete proteome; Developmental protein; Membrane; Reference proteome; Repeat; Sugar transport; Transmembrane; Transmembrane helix; Transport
SRR122238_TR17062_c0_g1_i5_len=1342	TI10A_ARATH	Q:1128-397,H:4-253	38.02%ID	E:7e-39	Protein TIFY 10A	PF06200.11^tify^tify domain^105-137^E:4.2e-19`PF09425.7^CCT_2^Divergent CCT motif^192-217^E:3.4e-13	0	2,506	0	4,269	0	2,706	0	2,714	0,906	0,076	TI10A_ARATH	Protein TIFY 10A (Jasmonate ZIM domain-containing protein 1)	TIFY10A JAZ1 At1g19180 T29M8.5	Arabidopsis thaliana (Mouse-ear cress)		3D-structure; Alternative splicing; Complete proteome; Jasmonic acid signaling pathway; Nucleus; Plant defense; Reference proteome; Transcription; Transcription regulation; Ubl conjugation
SRR122238_TR19465_c0_g3_i2_len=1290	NA	NA	NA	NA	NA	PF00106.22^adh_short^short chain dehydrogenase^6-194^E:2.2e-25`PF13561.3^adh_short_C2^Enoyl-(Acyl carrier protein) reductase^17-253^E:1.7e-41	0	1,851	0	4,853	0	6,794	0	7,56	0,679	-0,322	.	.	.	.	.	.
SRR122238_TR21630_c0_g1_i3_len=1535	NA	NA	NA	NA	NA	PF06200.11^tify^tify domain^98-128^E:1e-17`PF09425.7^CCT_2^Divergent CCT motif^188-212^E:4e-13	0	4,513	0	5,824	0	4,914	0	4,98	1	-0,165	.	.	.	.	.	.
SRR122238_TR21949_c0_g1_i1_len=1290	FNSI_PETCR	Q:1175-195,H:1-333	34.82%ID	E:1e-66	Flavone synthase	PF14226.3^DIOX_N^non-haem dioxygenase in morphine synthesis N-terminal^76-180^E:2.3e-27`PF03171.17^2OG-FeII_Oxy^2OG-Fe(II) oxygenase superfamily^228-326^E:6.9e-29	0	3,469	0	4,861	0	3,012	0	2,676	0,629	-1,435	FNSI_PETCR	Flavone synthase (EC 1.14.11.22) (Flavone synthase I)	FNSI	Petroselinum crispum (Parsley) (Petroselinum hortense)	PATHWAY: Secondary metabolite biosynthesis; flavonoid biosynthesis.	Cytoplasm; Flavonoid biosynthesis; Iron; Metal-binding; Oxidoreductase; Vitamin C
SRR122238_TR24079_c0_g2_i5_len=1499	NA	NA	NA	NA	NA	PF06200.11^tify^tify domain^120-151^E:1.8e-16`PF09425.7^CCT_2^Divergent CCT motif^258-278^E:5.7e-09	0	1,555	0	3,15	0	2,407	0	2,951	0,017	-5,782	.	.	.	.	.	.
SRR122238_TR24079_c0_g2_i9_len=1403	NA	NA	NA	NA	NA	PF06200.11^tify^tify domain^88-119^E:1.6e-16`PF09425.7^CCT_2^Divergent CCT motif^226-246^E:5e-09	0	2,066	0	2,985	0	2,141	0	2,419	0,115	0,784	.	.	.	.	.	.
SRR122238_TR26146_c1_g2_i2_len=1477	B2_DAUCA	Q:541-996,H:54-205	80.92%ID	E:4e-90	B2 protein	PF10539.6^Dev_Cell_Death^Development and cell death domain^202-328^E:2.1e-41	0	1,136	0	1,356	0	1,663	0	1,554	0,022	0,544	B2_DAUCA	B2 protein		Daucus carota (Wild carrot)		
SRR122238_TR26400_c0_g1_i2_len=974	NA	NA	NA	NA	NA	PF04570.11^zf-FLZ^zinc-finger of the FCS-type, C2-C2^69-113^E:2.8e-19	0	2,085	0	3,041	0	3,955	0	3,717	1	0,155	.	.	.	.	.	.
SRR122238_TR27056_c0_g1_i5_len=2087	NA	NA	NA	NA	NA	PF03959.10^FSH1^Serine hydrolase (FSH1)^10-203^E:1.5e-47	0	1,75	0	2,327	0	1,998	0	2,931	1	-0,325	.	.	.	.	.	.
SRR122238_TR33818_c0_g1_i1_len=1720	U91A1_ARATH	Q:1595-213,H:9-466	57.42%ID	E:0	UDP-glycosyltransferase 91A1	PF00201.15^UDPGT^UDP-glucoronosyl and UDP-glucosyl transferase^275-444^E:2.7e-17	0	2,053	0	3,476	0	4,717	0	5,372	0,258	0,911	U91A1_ARATH	UDP-glycosyltransferase 91A1 (EC 2.4.1.-)	UGT91A1 At2g22590 T9I22.3	Arabidopsis thaliana (Mouse-ear cress)		Complete proteome; Glycosyltransferase; Reference proteome; Transferase
SRR122238_TR37682_c0_g1_i1_len=548	NA	NA	NA	NA	NA	.	0	4,101	0	6,102	0	10,678	0	7,884	1	0	.	.	.	.	.	.
SRR122238_TR45263_c0_g1_i3_len=1590	DRL29_ARATH	Q:775-179,H:617-816	54.23%ID	E:3e-59	Probable disease resistance protein At4g33300	PF05659.8^RPW8^Arabidopsis broad-spectrum mildew resistance protein RPW8^38-165^E:2.6e-14`PF00931.19^NB-ARC^NB-ARC domain^221-288^E:5.7e-11	0	2,288	0	3,391	0	4,653	0	4,971	1	-0,035	DRL29_ARATH	Probable disease resistance protein At4g33300	At4g33300 F17M5.60	Arabidopsis thaliana (Mouse-ear cress)		ATP-binding; Coiled coil; Complete proteome; Leucine-rich repeat; Nucleotide-binding; Plant defense; Reference proteome; Repeat
SRR122238_TR47400_c1_g1_i3_len=1577	C72A1_CATRO	Q:106-1575,H:1-490	97.35%ID	E:0	Secologanin synthase	PF00067.19^p450^Cytochrome P450^90-490^E:2.6e-84	0	1,026	0	3,308	0	1,365	0	1,052	0,067	0,613	C72A1_CATRO	Secologanin synthase (SLS) (EC 1.3.3.9) (CYPLXXII) (Cytochrome P450 72A1)	CYP72A1 CYP72 P450CR3	Catharanthus roseus (Madagascar periwinkle) (Vinca rosea)	PATHWAY: Alkaloid biosynthesis; secologanin biosynthesis.	Alkaloid metabolism; Heme; Iron; Membrane; Metal-binding; Monooxygenase; NADP; Oxidoreductase; Transmembrane; Transmembrane helix; Vacuole
[illegible]	[illegible]	Q:200-403,H:1-[illegible]	[illegible]	[illegible]	[illegible]	[illegible]	[illegible]	[illegible]	[illegible]	[illegible]	[illegible]	[illegible]	[illegible]	[illegible]	[illegible]	[illegible]	[illegible]	[illegible]		Prunus armeniaca (Apricot)		[illegible]

[illegible]	NLTP2_PRUAR	[illegible] 68	[illegible]	[illegible]	Non-specific lipid-transfer protein 2	.	[illegible]	[illegible]	[illegible]	[illegible]	[illegible]	[illegible]	[illegible]	[illegible]	[illegible]	[illegible]	NLTP2_PRUAR	Non-specific lipid-transfer protein 2 (LTP 2)		[illegible] (Armeniaca vulgaris)		Direct protein sequencing; Lipid-binding; Transport
SRR122238_TR50696_c0_g1_i1_len=606	GRXC9_ARATH	Q:480-94,H:11-137	54.2%ID	E:3e-40	Glutaredoxin-C9	PF00462.21^Glutaredoxin^Glutaredoxin^47-115^E:2.5e-09	0	7,782	0	2,539	0	1,94	0	3,574	0,262	-0,536	GRXC9_ARATH	Glutaredoxin-C9 (AtGrxC9) (Protein ROXY 19)	GRXC9 GRX480 ROXY19 At1g28480 F3M18.8	Arabidopsis thaliana (Mouse-ear cress)		Complete proteome; Cytoplasm; Disulfide bond; Electron transport; Nucleus; Redox-active center; Reference proteome; Transport
SRR122238_TR65873_c1_g1_i1_len=574	CNR8_MAIZE	Q:272-571,H:38-140	65.42%ID	E:4e-39	Cell number regulator 8	PF04749.14^PLAC8^PLAC8 family^71-140^E:7.1e-09	0,001	1,533	0	4,65	0	1,673	0	2,001	0,02	0,854	CNR8_MAIZE	Cell number regulator 8 (ZmCNR08)	CNR8 SAT5	Zea mays (Maize)		Complete proteome; Membrane; Reference proteome; Transmembrane; Transmembrane helix
SRR122238_TR75737_c0_g1_i4_len=1213	AOC4_ARATH	Q:868-308,H:64-248	66.67%ID	E:2e-73	Allene oxide cyclase 4, chloroplastic	PF06351.8^Allene_ox_cyc^Allene oxide cyclase^77-249^E:1.7e-91	0	1,961	0	5,566	0	2,937	0	2,978	0,093	-1,044	AOC4_ARATH	Allene oxide cyclase 4, chloroplastic (EC 5.3.99.6)	AOC4 At1g13280 T6J4.4 T6J4_23	Arabidopsis thaliana (Mouse-ear cress)		Chloroplast; Complete proteome; Isomerase; Plastid; Reference proteome; Transit peptide
SRR1271857_TR30546_c0_g1_i1_len=1649	NA	NA	NA	NA	NA	PF00388.16^PI-PLC-X^Phosphatidylinositol-specific phospholipase C, X domain^81-174^E:2.6e-09	0	1,396	0	1,532	0	1,967	0	1,791	0,84	0,066	.	.	.	.	.	.
SRR1271857_TR32512_c2_g1_i3_len=1413	NA	NA	NA	NA	NA	PF06200.11^tify^tify domain^108-139^E:1.7e-16`PF09425.7^CCT_2^Divergent CCT motif^246-266^E:5.4e-09	0	1,565	0	3,732	0	1,67	0	1,967	1	-0,133	.	.	.	.	.	.
SRR1271857_TR37691_c0_g1_i3_len=1157	NA	NA	NA	NA	NA	PF06232.8^ATS3^Embryo-specific protein 3, (ATS3)^30-132^E:4.1e-07`PF01477.20^PLAT^PLAT/LH2 domain^36-153^E:2.2e-14	0	0,813	0	3,151	0	4,17	0	3,746	0,895	0,05	.	.	.	.	.	.
SRR1271858_TR9171_c0_g1_i1_len=639	TRXH2_ARATH	Q:144-506,H:11-130	52.07%ID	E:1e-44	Thioredoxin H2	PF14595.3^Thioredoxin_9^Thioredoxin^10-124^E:6.7e-06`PF04756.10^OST3_OST6^OST3 / OST6 family, transporter family^19-127^E:1.5e-06`PF00085.17^Thioredoxin^Thioredoxin^35-127^E:2e-29`PF13098.3^Thioredoxin_2^Thioredoxin-like domain^40-119^E:9.4e-07`PF00578.18^AhpC-TSA^AhpC/TSA family^42-119^E:8.7e-05	0	2,854	0	6,429	0	4,886	0	5,499	0,461	-1,107	TRXH2_ARATH	Thioredoxin H2 (AtTrxh2) (Thioredoxin 2) (AtTRX2)	TRX2 At5g39950 MYH19.14	Arabidopsis thaliana (Mouse-ear cress)		Complete proteome; Cytoplasm; Disulfide bond; Electron transport; Mitochondrion; Redox-active center; Reference proteome; Transport
SRR1271858_TR16116_c0_g1_i3_len=1778	LDOX_MALDO	Q:1382-567,H:10-281	37.82%ID	E:9e-58	Leucoanthocyanidin dioxygenase	PF14226.3^DIOX_N^non-haem dioxygenase in morphine synthesis N-terminal^56-167^E:6e-33`PF03171.17^2OG-FeII_Oxy^2OG-Fe(II) oxygenase superfamily^216-292^E:5.6e-15	0	5,829	0	12,489	0	3,773	0	2,766	1	0	LDOX_MALDO	Leucoanthocyanidin dioxygenase (LDOX) (Leucocyanidin oxygenase) (EC 1.14.11.19) (Anthocyanidin synthase) (Leucoanthocyanidin hydroxylase)	ANS	Malus domestica (Apple) (Pyrus malus)	PATHWAY: Pigment biosynthesis; anthocyanin biosynthesis.	Dioxygenase; Flavonoid biosynthesis; Iron; Metal-binding; Oxidoreductase; Vitamin C
SRR1271858_TR21137_c0_g1_i1_len=1857	GDL71_ARATH	Q:1545-604,H:39-352	59.37%ID	E:9e-134	GDSL esterase/lipase At5g03610	PF00657.19^Lipase_GDSL^GDSL-like Lipase/Acylhydrolase^55-356^E:3e-12	0	1,691	0	5,304	0	2,997	0	3,135	0,534	0,374	GDL71_ARATH	GDSL esterase/lipase At5g03610 (EC 3.1.1.-) (Extracellular lipase At5g03610)	At5g03610 F17C15.30	Arabidopsis thaliana (Mouse-ear cress)		Complete proteome; Glycoprotein; Hydrolase; Lipid degradation; Lipid metabolism; Reference proteome; Secreted; Signal
SRR1271858_TR43396_c1_g1_i1_len=2203	PTR28_ARATH	Q:1893-175,H:4-569	63.08%ID	E:0	Protein NRT1/ PTR FAMILY 5.6	PF07690.13^MFS_1^Major Facilitator Superfamily^91-516^E:6.2e-09`PF00854.18^PTR2^POT family^112-534^E:1e-94	0	4,942	0	9,905	0	4,653	0	4,55	1	0,15	PTR28_ARATH	Protein NRT1/ PTR FAMILY 5.6 (AtNPF5.6)	NPF5.6 At2g37900 T8P21.19	Arabidopsis thaliana (Mouse-ear cress)		Complete proteome; Membrane; Phosphoprotein; Reference proteome; Transmembrane; Transmembrane helix; Transport
SRR1271858_TR49971_c0_g1_i7_len=2709	NA	NA	NA	NA	NA	PF06200.11^tify^tify domain^65-96^E:9.6e-17	0,001	2,18	0	3,069	0	2,691	0	2,938	0,11	0,589	.	.	.	.	.	.
SRR1271858_TR49971_c0_g1_i26_len=3146	NA	NA	NA	NA	NA	PF09425.7^CCT_2^Divergent CCT motif^100-120^E:1.8e-09	0	2,496	0	2,779	0	3,051	0	3,143	0,875	0,083	.	.	.	.	.	.
SRR1271858_TR57498_c0_g2_i1_len=846	GSTX4_TOBAC	Q:58-579,H:47-221	72.57%ID	E:4e-89	Probable glutathione S-transferase	PF02798.17^GST_N^Glutathione S-transferase, N-terminal domain^16-50^E:1.2e-09`PF13417.3^GST_N_3^Glutathione S-transferase, N-terminal domain^18-54^E:6.7e-07`PF00043.22^GST_C^Glutathione S-transferase, C-terminal domain^89-167^E:3.2e-11`PF13410.3^GST_C_2^Glutathione S-transferase, C-terminal domain^96-161^E:2.4e-12	0	1,444	0	2,694	0	1,059	0,001	0,737	0,021	0,621	GSTX4_TOBAC	Probable glutathione S-transferase (EC 2.5.1.18) (Auxin-induced protein PCNT107)		Nicotiana tabacum (Common tobacco)		Auxin signaling pathway; Transferase
SRR1271859_TR797_c0_g1_i1_len=914	SRG1_ARATH	Q:817-56,H:16-279	46.97%ID	E:2e-81	Protein SRG1	PF14226.3^DIOX_N^non-haem dioxygenase in morphine synthesis N-terminal^56-136^E:1.5e-19`PF03171.17^2OG-FeII_Oxy^2OG-Fe(II) oxygenase superfamily^197-262^E:1.3e-14	0	6,992	0	6,778	0	10,204	0	10,199	1	1,039	SRG1_ARATH	Protein SRG1 (AtSRG1) (Protein SENESCENCE-RELATED GENE 1)	SRG1 At1g17020 F20D23.28 F6I1.30	Arabidopsis thaliana (Mouse-ear cress)		Complete proteome; Iron; Metal-binding; Oxidoreductase; Reference proteome
SRR1271859_TR7358_c0_g1_i1_len=2680	FAO2_LOTJA	Q:2002-137,H:119-748	62.11%ID	E:0	Long-chain-alcohol oxidase FAO2	PF00732.16^GMC_oxred_N^GMC oxidoreductase^91-353^E:2.4e-69`PF05199.10^GMC_oxred_C^GMC oxidoreductase^442-575^E:1.2e-19	0	5,799	0	4,59	0	2,293	0	2,447	0,402	1,137	FAO2_LOTJA	Long-chain-alcohol oxidase FAO2 (EC 1.1.3.20) (Long-chain fatty alcohol oxidase 2)	FAO2	Lotus japonicus (Lotus corniculatus var. japonicus)		FAD; Flavoprotein; Membrane; Oxidoreductase; Transmembrane; Transmembrane helix
SRR1271859_TR11727_c0_g1_i1_len=974	NA	NA	NA	NA	NA	PF06232.8^ATS3^Embryo-specific protein 3, (ATS3)^30-132^E:2.4e-08`PF01477.20^PLAT^PLAT/LH2 domain^36-153^E:3.4e-13	0	1,271	0	3,529	0	4,594	0	4,592	0,027	0,856	.	.	.	.	.	.
SRR1271859_TR18405_c0_g2_i1_len=2878	MYC4_ARATH	Q:565-2382,H:60-585	52.81%ID	E:1e-162	Transcription factor MYC4	PF14215.3^bHLH-MYC_N^bHLH-MYC and R2R3-MYB transcription factors N-terminal^1-81^E:3.1e-22`PF00010.23^HLH^Helix-loop-helix DNA-binding domain^327-372^E:3e-10	0	1,963	0	2,492	0	1,849	0	1,91	0,204	0,546	MYC4_ARATH	Transcription factor MYC4 (AtMYC4) (Basic helix-loop-helix protein 4) (AtbHLH4) (bHLH 4) (Transcription factor EN 37) (bHLH transcription factor bHLH004)	MYC4 BHLH4 EN37 At4g17880 T6K21.60	Arabidopsis thaliana (Mouse-ear cress)		Complete proteome; DNA-binding; Nucleus; Reference proteome; Transcription; Transcription regulation
SRR1271859_TR23354_c0_g2_i1_len=1562	NA	NA	NA	NA	NA	PF00257.16^Dehydrin^Dehydrin^10-178^E:2e-43	0	2,197	0	4,675	0	5,109	0	2,946	0	-2,756	.	.	.	.	.	.
SRR1271859_TR31628_c0_g1_i1_len=1321	SAMT_CLABR	Q:1258-167,H:1-357	56.56%ID	E:4e-146	Salicylate carboxymethyltransferase	PF03492.12^Methyltransf_7^SAM dependent carboxyl methyltransferase^39-363^E:1.9e-125	0	4,081	0	4,677	0	3,384	0	3,664	1	2,67	SAMT_CLABR	Salicylate carboxymethyltransferase (EC 2.1.1.274) (S-adenosyl-L-methionine:salicylate acid carboxylmethyltransferase) (CbSAMT) (Salicylate O-methyltransferase)	SAMT	Clarkia breweri (Fairy fans) (Eucharidium breweri)		3D-structure; Magnesium; Metal-binding; Methyltransferase; S-adenosyl-L-methionine; Transferase
SRR1271859_TR33815_c0_g1_i1_len=1249	SRG1_ARATH	Q:474-1040,H:168-356	39.15%ID	E:2e-40	Protein SRG1	PF03171.17^2OG-FeII_Oxy^2OG-Fe(II) oxygenase superfamily^26-124^E:1.6e-27	0	8,413	0	11,646	0	4,389	0	3,139	1	0,872	SRG1_ARATH	Protein SRG1 (AtSRG1) (Protein SENESCENCE-RELATED GENE 1)	SRG1 At1g17020 F20D23.28 F6I1.30	Arabidopsis thaliana (Mouse-ear cress)		Complete proteome; Iron; Metal-binding; Oxidoreductase; Reference proteome
SRR1271859_TR38939_c0_g1_i1_len=3435	NA	NA	NA	NA	NA	PF13419.3^HAD_2^Haloacid dehalogenase-like hydrolase^130-310^E:1.2e-20`PF00702.23^Hydrolase^haloacid dehalogenase-like hydrolase^213-303^E:4.5e-08`PF13242.3^Hydrolase_like^HAD-hyrolase-like^265-312^E:3.9e-06	0	3,072	0	4,499	0	1,952	0	2,396	1	1,04	.	.	.	.	.	.
SRR1271859_TR43882_c2_g5_i7_len=2157	PTR53_ARATH	Q:2016-256,H:27-615	54.9%ID	E:0	Protein NRT1/ PTR FAMILY 2.11	PF00854.18^PTR2^POT family^95-527^E:1e-80	0,001	1,247	0	3,435	0	5,118	0	4,948	1	0,036	PTR53_ARATH	Protein NRT1/ PTR FAMILY 2.11 (AtNPF2.11) (Nitrate transporter 1.10) (Protein GLUCOSINOLATE TRANSPORTER-2)	NPF2.11 GTR2 NRT1.10 At5g62680 MRG21.10	Arabidopsis thaliana (Mouse-ear cress)		Cell membrane; Complete proteome; Membrane; Reference proteome; Transmembrane; Transmembrane helix; Transport
SRR1271859_TR44431_c0_g1_i3_len=1795	ARGE_ARATH	Q:185-1495,H:1-438	67.57%ID	E:0	Acetylornithine deacetylase	PF01546.25^Peptidase_M20^Peptidase family M20/M25/M40^95-434^E:5.3e-29`PF07687.11^M20_dimer^Peptidase dimerisation domain^203-317^E:6.6e-13	0	2,937	0	5,461	0	5,453	0	4,171	1	1,453	ARGE_ARATH	Acetylornithine deacetylase (EC 3.5.1.16) (N-acetylornithinase) (AO) (Acetylornithinase) (NAO)	At4g17830 dl4945w T6K21	Arabidopsis thaliana (Mouse-ear cress)	PATHWAY: Amino-acid biosynthesis; L-arginine biosynthesis; L-ornithine from N(2)-acetyl-L-ornithine (linear): step 1/1.	Alternative splicing; Amino-acid biosynthesis; Arginine biosynthesis; Cobalt; Complete proteome; Hydrolase; Metal-binding; Reference proteome; Zinc
SRR342017_TR1488_c0_g1_i1_len=1558	NA	NA	NA	NA	NA	PF17181.1^EPF^Epidermal patterning factor proteins^96-146^E:7.6e-20	0	1,667	0	2,297	0	1,755	0	1,431	0,618	0,264	.	.	.	.	.	.
SRR342017_TR6175_c1_g1_i6_len=2137	SERC_ARATH	Q:205-1083,H:1-294	70.41%ID	E:2e-141	Phosphoserine phosphatase, chloroplastic	PF00702.23^Hydrolase^haloacid dehalogenase-like hydrolase^83-257^E:1.1e-16`PF12710.4^HAD^haloacid dehalogenase-like hydrolase^86-254^E:2.4e-12	0,001	1,165	0	2,133	0	1,739	0	1,848	0,73	0,14	SERC_ARATH	Phosphoserine phosphatase, chloroplastic (PSP) (PSPase) (EC 3.1.3.3) (O-phosphoserine phosphohydrolase)	PSP PSP1 At1g18640 F26I16.2	Arabidopsis thaliana (Mouse-ear cress)	PATHWAY: Amino-acid biosynthesis; L-serine biosynthesis; L-serine from 3-phospho-D-glycerate: step 3/3.	Amino-acid biosynthesis; Chloroplast; Complete proteome; Hydrolase; Magnesium; Metal-binding; Phosphoprotein; Plastid; Reference proteome; Serine biosynthesis; Transit peptide
SRR342019_TR769_c0_g1_i1_len=1320	NA	NA	NA	NA	NA	PF04193.11^PQ-loop^PQ loop repeat^37-93^E:1.4e-18`PF04193.11^PQ-loop^PQ loop repeat^256-313^E:5.5e-16	0	4,108	0	5,354	0	3,59	0	2,81	0,003	1,368	.	.	.	.	.	.
SRR342019_TR1484_c0_g1_i1_len=1241	NA	NA	NA	NA	NA	PF03350.13^UPF0114^Uncharacterized protein family, UPF0114^106-244^E:1.2e-37	0	1,113	0	1,904	0	1,881	0	1,958	0,596	0,17	.	.	.	.	.	.
SRR342019_TR3321_c0_g1_i2_len=676	SUMO2_ARATH	Q:148-414,H:6-93	89.89%ID	E:3e-49	Small ubiquitin-related modifier 2	PF11976.5^Rad60-SLD^Ubiquitin-2 like Rad60 SUMO-like^26-96^E:4e-25`PF00240.20^ubiquitin^Ubiquitin family^28-98^E:4.9e-14	0,001	1,133	0	1,386	0	1,584	0	1,511	0,862	-0,102	SUMO2_ARATH	Small ubiquitin-related modifier 2 (AtSUMO2)	SUMO2 SUM2 At5g55160 MCO15.11	Arabidopsis thaliana (Mouse-ear cress)		Alternative splicing; Complete proteome; Cytoplasm; Isopeptide bond; Nucleus; Reference proteome; Ubl conjugation pathway
SRR342019_TR3921_c0_g1_i1_len=1395	CSE_ARATH	Q:1127-309,H:50-323	35.4%ID	E:2e-49	Caffeoylshikimate esterase	PF12146.5^Hydrolase_4^Serine aminopeptidase, S33^34-272^E:3.2e-67`PF00561.17^Abhydrolase_1^alpha/beta hydrolase fold^39-148^E:1.1e-08`PF12697.4^Abhydrolase_6^Alpha/beta hydrolase family^39-270^E:9.2e-10	0	2,195	0	3,874	0	6,303	0	6,75	0,381	-0,785	CSE_ARATH	Caffeoylshikimate esterase (EC 3.1.1.-) (Lysophospholipase 2) (LysoPL2)	CSE At1g52760 F14G24.3	Arabidopsis thaliana (Mouse-ear cress)		Cell membrane; Complete proteome; Hydrolase; Lignin biosynthesis; Membrane; Reference proteome
SRR342019_TR4458_c0_g1_i1_len=1942	MYRS_QUEIL	Q:1692-466,H:38-414	53.55%ID	E:1e-134	Myrcene synthase, chloroplastic	PF01397.18^Terpene_synth^Terpene synthase, N-terminal domain^67-170^E:1.7e-24	0	4,335	0	9,243	0	9,985	0	9,901	1	0,755	MYRS_QUEIL	Myrcene synthase, chloroplastic (EC 4.2.3.15)		Quercus ilex (Holly oak)		Chloroplast; Lyase; Magnesium; Manganese; Metal-binding; Plastid; Transit peptide
SRR342019_TR5666_c0_g6_i1_len=1927	FACR5_ARATH	Q:228-1703,H:3-494	50.91%ID	E:6e-171	Probable fatty acyl-CoA reductase 5	PF07993.9^NAD_binding_4^Male sterility protein^18-325^E:2.4e-76`PF03015.16^Sterile^Male sterility protein^400-496^E:3.2e-12	0	2,205	0	6,764	0	5,096	0	6,562	0,356	1,327	FACR5_ARATH	Probable fatty acyl-CoA reductase 5 (EC 1.2.1.n2)	FAR5 At3g44550 F14L2.100	Arabidopsis thaliana (Mouse-ear cress)		Complete proteome; Lipid biosynthesis; Lipid metabolism; NADP; Oxidoreductase; Reference proteome
SRR342019_TR5968_c2_g4_i3_len=1281	B2_DAUCA	Q:675-220,H:54-205	80.92%ID	E:9e-91	B2 protein	PF10539.6^Dev_Cell_Death^Development and cell death domain^168-294^E:1.7e-41	0	0,871	0	2,49	0	1,277	0	1,511	0,312	0,763	B2_DAUCA	B2 protein		Daucus carota (Wild carrot)		
SRR342019_TR16670_c0_g1_i1_len=578	NA	NA	NA	NA	NA	.	0	3,334	0	4,758	0	4,035	0	3,638	0,014	2,973	.	.	.	.	.	.
SRR342019_TR21154_c0_g1_i1_len=5234	AB4C_ARATH	Q:4828-365,H:23-1516	67.95%ID	E:0	ABC transporter C family member 4	PF00664.20^ABC_membrane^ABC transporter transmembrane region^331-597^E:9.8e-29`PF00005.24^ABC_tran^ABC transporter^663-795^E:1.7e-19`PF00664.20^ABC_membrane^ABC transporter transmembrane region^982-1202^E:5.3e-29`PF00005.24^ABC_tran^ABC transporter^1283-1431^E:4.3e-28	0	2,332	0	3,748	0	2,24	0	1,666	1	-0,186	AB4C_ARATH	ABC transporter C family member 4 (ABC transporter ABCC.4) (AtABCC4) (EC 3.6.3.44) (ATP-energized glutathione S-conjugate pump 4) (Glutathione S-conjugate-transporting ATPase 4) (Multidrug resistance-associated protein 4)	ABCC4 EST3 MRP4 At2g47800 F17A22.19	Arabidopsis thaliana (Mouse-ear cress)		ATP-binding; Cell membrane; Complete proteome; Hydrolase; Membrane; Nucleotide-binding; Reference proteome; Repeat; Transmembrane; Transmembrane helix; Transport; Vacuole
SRR342019_TR22681_c0_g4_i3_len=1765	C74B2_ARATH	Q:1591-197,H:38-492	56.32%ID	E:1e-178	Linolenate hydroperoxide lyase, chloroplastic {ECO:0000305|PubMed:9701595}	PF00067.19^p450^Cytochrome P450^146-495^E:1.1e-24	0	1,326	0	1,076	0	0,998	0	0,916	1	0,375	C74B2_ARATH	Linolenate hydroperoxide lyase, chloroplastic (EC 4.2.99.-) (Cytochrome P450 74B2) (Hydroperoxide lyase 1)	CYP74B2 HPL1	Arabidopsis thaliana (Mouse-ear cress)		Chloroplast; Heme; Iron; Lyase; Metal-binding; Plastid; Transit peptide
SRR342019_TR27753_c0_g2_i1_len=774	GASAE_ARATH	Q:277-83,H:211-275	76.92%ID	E:2e-26	Gibberellin-regulated protein 14	PF02704.11^GASA^Gibberellin regulated protein^170-230^E:1e-18	0	1,452	0	4,741	0	4,934	0	5,266	0,643	-0,587	GASAE_ARATH	Gibberellin-regulated protein 14 (GAST1 protein homolog 14)	GASA14 At5g14920 F2G14.40	Arabidopsis thaliana (Mouse-ear cress)		Alternative splicing; Complete proteome; Disulfide bond; Gibberellin signaling pathway; Reference proteome; Secreted; Signal
SRR342019_TR28505_c2_g4_i4_len=1446	GCL1_ARATH	Q:3-1160,H:82-433	63.99%ID	E:5e-173	LanC-like protein GCL1	PF05147.10^LANC_like^Lanthionine synthetase C-like protein^2-242^E:1.1e-56	0	3,334	0	2,88	0	7,358	0,001	6,639	0,034	5,561	GCL1_ARATH	LanC-like protein GCL1 (G protein-coupled receptor 2-like protein 1) (Protein GCR2-like 1)	GCL1 At5g65280 MQN23.23	Arabidopsis thaliana (Mouse-ear cress)		Complete proteome; Reference proteome
SRR342019_TR28505_c2_g4_i6_len=2638	GCL1_ARATH	Q:1414-2352,H:126-433	69.97%ID	E:3e-150	LanC-like protein GCL1	PF05147.10^LANC_like^Lanthionine synthetase C-like protein^2-256^E:1.2e-65	0	3,522	0	4,913	0	4,656	0	4,893	0,189	1,712	GCL1_ARATH	LanC-like protein GCL1 (G protein-coupled receptor 2-like protein 1) (Protein GCR2-like 1)	GCL1 At5g65280 MQN23.23	Arabidopsis thaliana (Mouse-ear cress)		Complete proteome; Reference proteome
SRR342019_TR35584_c0_g2_i1_len=2436	ACOX1_ARATH	Q:255-2243,H:1-663	78.88%ID	E:0	Peroxisomal acyl-coenzyme A oxidase 1	PF14749.3^Acyl-CoA_ox_N^Acyl-coenzyme A oxidase N-terminal^17-132^E:1.4e-31`PF02770.16^Acyl-CoA_dh_M^Acyl-CoA dehydrogenase, middle domain^134-246^E:4.5e-12`PF01756.16^ACOX^Acyl-CoA oxidase^479-656^E:4.1e-46	0,001	1,066	0	2,473	0	2,64	0	2,703	0,255	0,591	ACOX1_ARATH	Peroxisomal acyl-coenzyme A oxidase 1 (AOX 1) (EC 1.3.3.6) (Long-chain acyl-CoA oxidase) (AtCX1)	ACX1 At4g16760 dl4405c FCAALL.119	Arabidopsis thaliana (Mouse-ear cress)		3D-structure; Alternative splicing; Complete proteome; Disulfide bond; FAD; Fatty acid metabolism; Flavoprotein; Lipid metabolism; Oxidoreductase; Peroxisome; Reference proteome
SRR342019_TR37278_c0_g1_i3_len=2454	LAL5_ARATH	Q:290-1687,H:8-464	59.87%ID	E:1e-166	.	PF01554.15^MatE^MatE^57-216^E:1.4e-29`PF01554.15^MatE^MatE^278-439^E:1.1e-25	0	1,538	0	3,871	0	2,082	0	2,148	0,528	-0,245	DTX18_ARATH	Protein DETOXIFICATION 18 (AtDTX18) (Multidrug and toxic compound extrusion protein 18) (MATE protein 18) (Protein LIKE ALF5)	DTX18 LAL5 At3g23550 MDB19.3	Arabidopsis thaliana (Mouse-ear cress)		Complete proteome; Membrane; Reference proteome; Transmembrane; Transmembrane helix; Transport
SRR342019_TR38071_c4_g2_i10_len=1933	ARGE_ARATH	Q:260-1570,H:1-438	67.8%ID	E:0	Acetylornithine deacetylase	PF01546.25^Peptidase_M20^Peptidase family M20/M25/M40^95-434^E:5.3e-29`PF07687.11^M20_dimer^Peptidase dimerisation domain^203-317^E:2.3e-12	0	3,436	0	4,858	0	5,05	0	4,711	0,058	0,816	ARGE_ARATH	Acetylornithine deacetylase (EC 3.5.1.16) (N-acetylornithinase) (AO) (Acetylornithinase) (NAO)	At4g17830 dl4945w T6K21	Arabidopsis thaliana (Mouse-ear cress)	PATHWAY: Amino-acid biosynthesis; L-arginine biosynthesis; L-ornithine from N(2)-acetyl-L-ornithine (linear): step 1/1.	Alternative splicing; Amino-acid biosynthesis; Arginine biosynthesis; Cobalt; Complete proteome; Hydrolase; Metal-binding; Reference proteome; Zinc
SRR342019_TR38071_c4_g2_i17_len=1977	ARGE_ARATH	Q:260-1570,H:1-438	67.8%ID	E:0	Acetylornithine deacetylase	PF01546.25^Peptidase_M20^Peptidase family M20/M25/M40^95-434^E:5.3e-29`PF07687.11^M20_dimer^Peptidase dimerisation domain^203-317^E:2.3e-12	0	3,085	0	5,935	0	6,318	0	4,301	1	0	ARGE_ARATH	Acetylornithine deacetylase (EC 3.5.1.16) (N-acetylornithinase) (AO) (Acetylornithinase) (NAO)	At4g17830 dl4945w T6K21	Arabidopsis thaliana (Mouse-ear cress)	PATHWAY: Amino-acid biosynthesis; L-arginine biosynthesis; L-ornithine from N(2)-acetyl-L-ornithine (linear): step 1/1.	Alternative splicing; Amino-acid biosynthesis; Arginine biosynthesis; Cobalt; Complete proteome; Hydrolase; Metal-binding; Reference proteome; Zinc
SRR342022_TR137_c3_g1_i6_len=1264	EGS1_OCIBA	Q:119-1045,H:7-314	66.99%ID	E:3e-143	Eugenol synthase 1	PF05368.10^NmrA^NmrA-like family^8-231^E:3.3e-55`PF01370.18^Epimerase^NAD dependent epimerase/dehydratase family^8-86^E:1e-05`PF01073.16^3Beta_HSD^3-beta hydroxysteroid dehydrogenase/isomerase family^9-98^E:2.5e-05`PF13460.3^NAD_binding_10^NAD(P)H-binding^12-149^E:4e-12	0	1,77	0	3,318	0	5,065	0	5,922	1	0,082	EGS1_OCIBA	Eugenol synthase 1 (EC 1.1.1.318)	EGS1	Ocimum basilicum (Sweet basil)	PATHWAY: Aromatic compound metabolism; phenylpropanoid biosynthesis.	3D-structure; NADP; Oxidoreductase; Phenylpropanoid metabolism
SRR342022_TR4918_c0_g2_i1_len=1468	STSY_CATRO	Q:106-1170,H:1-352	96.34%ID	E:0	Strictosidine synthase	PF08450.9^SGL^SMP-30/Gluconolaconase/LRE-like region^169-344^E:4.4e-07`PF03088.13^Str_synth^Strictosidine synthase^194-282^E:6.6e-24	0	1,154	0	3,375	0	3,022	0	2,148	1	0,724	STSY_CATRO	Strictosidine synthase (EC 4.3.3.2)	STR1 SSS	Catharanthus roseus (Madagascar periwinkle) (Vinca rosea)	PATHWAY: Alkaloid biosynthesis; 3alpha(S)-strictosidine biosynthesis; 3alpha(S)-strictosidine from secologanin and tryptamine: step 1/1.	Alkaloid metabolism; Direct protein sequencing; Glycoprotein; Lyase; Signal; Vacuole
SRR342022_TR5922_c0_g2_i1_len=2130	ICS_CATRO	Q:1950-376,H:43-579	62.27%ID	E:0	Isochorismate synthase, chloroplastic	PF00425.15^Chorismate_bind^chorismate binding enzyme^316-570^E:5.9e-55	0	1,974	0	2,848	0	3,154	0	2,978	1	0,393	ICS_CATRO	Isochorismate synthase, chloroplastic (EC 5.4.4.2)		Catharanthus roseus (Madagascar periwinkle) (Vinca rosea)		Chloroplast; Direct protein sequencing; Isomerase; Magnesium; Plant defense; Plastid; Transit peptide
SRR342022_TR7495_c0_g2_i1_len=1876	C83B1_ARATH	Q:198-1571,H:41-499	45.12%ID	E:2e-139	Cytochrome P450 83B1	PF00067.19^p450^Cytochrome P450^55-512^E:1.8e-103	0	3,911	0	6,176	0	9,752	0	10,653	1	2,541	C83B1_ARATH	Cytochrome P450 83B1 (EC 1.14.-.-) (Protein ALTERED TRYPTOPHAN REGULATION 4) (Protein RED ELONGATED 1) (Protein SUPERROOT 2)	CYP83B1 ATR4 RED1 RNT1 RUNT1 SUR2 At4g31500 F3L17.70	Arabidopsis thaliana (Mouse-ear cress)		Complete proteome; Growth regulation; Heme; Iron; Membrane; Metal-binding; Monooxygenase; Oxidoreductase; Reference proteome; Transmembrane; Transmembrane helix
SRR342022_TR9756_c0_g1_i4_len=2081	VINSY_RAUSE	Q:423-1793,H:5-407	34.91%ID	E:5e-77	Vinorine synthase	PF02458.12^Transferase^Transferase family^4-309^E:8.5e-68`PF02458.12^Transferase^Transferase family^336-459^E:2.4e-13	0,001	2,232	0	6,36	0	2,946	0	3,026	1	1,326	VINSY_RAUSE	Vinorine synthase (EC 2.3.1.160)	ACT	Rauvolfia serpentina (Serpentine wood) (Ophioxylon serpentinum)		3D-structure; Acyltransferase; Alkaloid metabolism; Direct protein sequencing; Transferase
SRR342022_TR13452_c0_g2_i1_len=703	HQGT_RAUSE	Q:703-311,H:342-470	80.92%ID	E:1e-64	Hydroquinone glucosyltransferase	PF00201.15^UDPGT^UDP-glucoronosyl and UDP-glucosyl transferase^1-91^E:7.5e-19	0	1,94	0	4,522	0	2,952	0	2,846	1	1,879	HQGT_RAUSE	Hydroquinone glucosyltransferase (EC 2.4.1.218) (Arbutin synthase)	AS	Rauvolfia serpentina (Serpentine wood) (Ophioxylon serpentinum)		Direct protein sequencing; Glycosyltransferase; Transferase
SRR342022_TR15270_c3_g3_i1_len=737	CYB5_BRAOB	Q:130-528,H:2-134	60.9%ID	E:1e-40	Cytochrome b5	PF00173.25^Cyt-b5^Cytochrome b5-like Heme/Steroid binding domain^10-81^E:7.1e-27	0	0,824	0	2,758	0	1,437	0	1,315	1	-1,134	CYB5_BRAOB	Cytochrome b5	CYB5	Brassica oleracea var. botrytis (Cauliflower)		Direct protein sequencing; Electron transport; Endoplasmic reticulum; Heme; Iron; Membrane; Metal-binding; Microsome; Transmembrane; Transmembrane helix; Transport
SRR342022_TR17158_c1_g3_i7_len=3511	GCL1_ARATH	Q:2315-3226,H:164-433	63.82%ID	E:2e-123	LanC-like protein GCL1	PF05147.10^LANC_like^Lanthionine synthetase C-like protein^2-142^E:2.4e-36	0	3,544	0	4,593	0	4,405	0	4,74	0,005	6,199	GCL1_ARATH	LanC-like protein GCL1 (G protein-coupled receptor 2-like protein 1) (Protein GCR2-like 1)	GCL1 At5g65280 MQN23.23	Arabidopsis thaliana (Mouse-ear cress)		Complete proteome; Reference proteome
SRR342022_TR21854_c3_g4_i2_len=4472	LOX21_SOLTU	Q:3797-1440,H:106-894	71.39%ID	E:0	Linoleate 13S-lipoxygenase 2-1, chloroplastic	PF00305.16^Lipoxygenase^Lipoxygenase^50-717^E:0	0	2,369	0	5,021	0	6,397	0	6,552	0,564	-0,489	LOX21_SOLTU	Linoleate 13S-lipoxygenase 2-1, chloroplastic (EC 1.13.11.12) (Lipoxygenase 2-1)	LOX2.1 LOX-H1	Solanum tuberosum (Potato)	PATHWAY: Lipid metabolism; oxylipin biosynthesis. {ECO:0000255|PROSITE-ProRule:PRU00726}.	Chloroplast; Complete proteome; Dioxygenase; Fatty acid biosynthesis; Fatty acid metabolism; Iron; Lipid biosynthesis; Lipid metabolism; Metal-binding; Oxidoreductase; Oxylipin biosynthesis; Plastid; Reference proteome; Thylakoid; Transit peptide
SRR342022_TR23185_c0_g3_i9_len=2204	MYRS_QUEIL	Q:1938-244,H:36-594	58.23%ID	E:0	Myrcene synthase, chloroplastic	PF01397.18^Terpene_synth^Terpene synthase, N-terminal domain^67-243^E:6.3e-44`PF03936.13^Terpene_synth_C^Terpene synthase family, metal binding domain^275-542^E:6.6e-100	0	5,107	0	9,293	0	7,739	0	7,656	1	0	MYRS_QUEIL	Myrcene synthase, chloroplastic (EC 4.2.3.15)		Quercus ilex (Holly oak)		Chloroplast; Lyase; Magnesium; Manganese; Metal-binding; Plastid; Transit peptide
SRR342022_TR27021_c0_g3_i2_len=1339	TAR4_ARATH	Q:97-1332,H:33-449	53.94%ID	E:2e-157	Tryptophan aminotransferase-related protein 4	PF04864.10^Alliinase_C^Allinase^1-341^E:1.4e-134`PF00155.18^Aminotran_1_2^Aminotransferase class I and II^27-322^E:3.2e-06	0	2,793	0	3,051	0	3,571	0	3,114	0,042	2,175	TAR4_ARATH	Tryptophan aminotransferase-related protein 4 (EC 2.6.1.-)	TAR4 At1g34060 F12G12.12 F12G12.150	Arabidopsis thaliana (Mouse-ear cress)		Aminotransferase; Complete proteome; Membrane; Pyridoxal phosphate; Reference proteome; Transferase; Transmembrane; Transmembrane helix
SRR342022_TR30684_c1_g2_i4_len=1695	FAD3E_TOBAC	Q:326-1420,H:12-374	76.16%ID	E:0	Omega-3 fatty acid desaturase, endoplasmic reticulum	PF11960.5^DUF3474^Domain of unknown function (DUF3474)^6-62^E:7.5e-26`PF00487.21^FA_desaturase^Fatty acid desaturase^77-328^E:4.4e-33	0	4,303	0	6,848	0	8,141	0	9,016	0,034	3,49	FAD3E_TOBAC	Omega-3 fatty acid desaturase, endoplasmic reticulum (EC 1.14.19.-)	FAD3	Nicotiana tabacum (Common tobacco)	PATHWAY: Lipid metabolism; polyunsaturated fatty acid biosynthesis.	Endoplasmic reticulum; Fatty acid biosynthesis; Fatty acid metabolism; Lipid biosynthesis; Lipid metabolism; Membrane; Oxidoreductase; Transmembrane; Transmembrane helix
SRR342022_TR30684_c1_g2_i9_len=1634	FAD3E_TOBAC	Q:265-1359,H:12-374	76.16%ID	E:0	Omega-3 fatty acid desaturase, endoplasmic reticulum	PF11960.5^DUF3474^Domain of unknown function (DUF3474)^6-62^E:7.5e-26`PF00487.21^FA_desaturase^Fatty acid desaturase^77-328^E:4.4e-33	0,001	2,741	0	6,66	0	4,86	0	5,723	1	-0,186	FAD3E_TOBAC	Omega-3 fatty acid desaturase, endoplasmic reticulum (EC 1.14.19.-)	FAD3	Nicotiana tabacum (Common tobacco)	PATHWAY: Lipid metabolism; polyunsaturated fatty acid biosynthesis.	Endoplasmic reticulum; Fatty acid biosynthesis; Fatty acid metabolism; Lipid biosynthesis; Lipid metabolism; Membrane; Oxidoreductase; Transmembrane; Transmembrane helix
SRR342022_TR31491_c0_g1_i2_len=1495	LDOX_MALDO	Q:367-1347,H:10-341	39.34%ID	E:3e-75	Leucoanthocyanidin dioxygenase	PF14226.3^DIOX_N^non-haem dioxygenase in morphine synthesis N-terminal^52-163^E:2e-27`PF03171.17^2OG-FeII_Oxy^2OG-Fe(II) oxygenase superfamily^212-310^E:1.5e-27	0	3,522	0	6,325	0	2,878	0	2,887	0,629	0,944	LDOX_MALDO	Leucoanthocyanidin dioxygenase (LDOX) (Leucocyanidin oxygenase) (EC 1.14.11.19) (Anthocyanidin synthase) (Leucoanthocyanidin hydroxylase)	ANS	Malus domestica (Apple) (Pyrus malus)	PATHWAY: Pigment biosynthesis; anthocyanin biosynthesis.	Dioxygenase; Flavonoid biosynthesis; Iron; Metal-binding; Oxidoreductase; Vitamin C
SRR342023_TR2068_c2_g6_i2_len=3638	PAO2_ARATH	Q:1961-501,H:1-490	80.41%ID	E:0	Probable polyamine oxidase 2	PF00890.21^FAD_binding_2^FAD binding domain^27-70^E:1.9e-06`PF13450.3^NAD_binding_8^NAD(P)-binding Rossmann-like domain^29-83^E:2.2e-13`PF01593.21^Amino_oxidase^Flavin containing amine oxidoreductase^34-453^E:9.6e-94	0	1,521	0	3,976	0	2,916	0	3,721	0,811	0,44	PAO2_ARATH	Probable polyamine oxidase 2 (AtPAO2) (EC 1.5.3.-) (Amine oxidase 1)	PAO2 At2g43020 MFL8.12	Arabidopsis thaliana (Mouse-ear cress)		Complete proteome; FAD; Flavoprotein; Oxidoreductase; Peroxisome; Reference proteome
SRR342023_TR2263_c2_g2_i14_len=1134	ARGE_ARATH	Q:2-1018,H:58-394	71.47%ID	E:9e-178	Acetylornithine deacetylase	PF01546.25^Peptidase_M20^Peptidase family M20/M25/M40^41-330^E:2.4e-22`PF07687.11^M20_dimer^Peptidase dimerisation domain^149-263^E:7.2e-12	0	2,497	0	4,748	0	4,424	0	3,258	0,282	1,123	ARGE_ARATH	Acetylornithine deacetylase (EC 3.5.1.16) (N-acetylornithinase) (AO) (Acetylornithinase) (NAO)	At4g17830 dl4945w T6K21	Arabidopsis thaliana (Mouse-ear cress)	PATHWAY: Amino-acid biosynthesis; L-arginine biosynthesis; L-ornithine from N(2)-acetyl-L-ornithine (linear): step 1/1.	Alternative splicing; Amino-acid biosynthesis; Arginine biosynthesis; Cobalt; Complete proteome; Hydrolase; Metal-binding; Reference proteome; Zinc
SRR342023_TR2555_c4_g2_i1_len=2165	NA	NA	NA	NA	NA	PF06200.11^tify^tify domain^65-96^E:1.4e-16`PF09425.7^CCT_2^Divergent CCT motif^203-223^E:4.2e-09	0,001	2,769	0	3,094	0	2,853	0	2,889	1	0,511	.	.	.	.	.	.
SRR342023_TR4584_c1_g4_i3_len=2527	HQGT_RAUSE	Q:1478-2527,H:27-364	78.29%ID	E:0	Hydroquinone glucosyltransferase	PF03360.13^Glyco_transf_43^Glycosyltransferase family 43^114-321^E:4.4e-72	0	1,578	0	4,475	0	2,445	0	2,588	1	0,791	HQGT_RAUSE	Hydroquinone glucosyltransferase (EC 2.4.1.218) (Arbutin synthase)	AS	Rauvolfia serpentina (Serpentine wood) (Ophioxylon serpentinum)		Direct protein sequencing; Glycosyltransferase; Transferase
SRR342023_TR8541_c0_g2_i1_len=637	RL112_ARATH	Q:133-636,H:1-168	95.83%ID	E:2e-106	60S ribosomal protein L11-2	PF00281.16^Ribosomal_L5^Ribosomal protein L5^9-62^E:8.3e-21`PF00673.18^Ribosomal_L5_C^ribosomal L5P family C-terminus^66-164^E:6.9e-21	0	8,634	0	1,321	0	1,433	0	1,389	0,691	-0,103	RL112_ARATH	60S ribosomal protein L11-2 (L16)	RPL11B At3g58700 T20N10_50; RPL11C RPL16B At4g18730 F28A21.140; RPL11D At5g45775 MRA19.26 MRA19_21	Arabidopsis thaliana (Mouse-ear cress)		Alternative splicing; Complete proteome; Cytoplasm; RNA-binding; Reference proteome; Ribonucleoprotein; Ribosomal protein; rRNA-binding
SRR342023_TR21501_c0_g2_i4_len=3046	PTR45_ARATH	Q:1876-2943,H:217-576	59.72%ID	E:1e-146	Protein NRT1/ PTR FAMILY 5.7	PF03283.10^PAE^Pectinacetylesterase^54-402^E:1e-163	0	3,423	0	5,07	0	2,714	0	2,053	1	-0,36	PTR45_ARATH	Protein NRT1/ PTR FAMILY 5.7 (AtNPF5.7)	NPF5.7 At3g53960 F5K20.260	Arabidopsis thaliana (Mouse-ear cress)		Complete proteome; Membrane; Phosphoprotein; Reference proteome; Transmembrane; Transmembrane helix; Transport
SRR342023_TR23966_c0_g4_i2_len=997	TAR4_ARATH	Q:888-421,H:296-449	48.72%ID	E:9e-50	Tryptophan aminotransferase-related protein 4	PF04864.10^Alliinase_C^Allinase^1-144^E:2.1e-46	0	2,91	0	2,155	0	4,419	0	3,364	1	2,747	TAR4_ARATH	Tryptophan aminotransferase-related protein 4 (EC 2.6.1.-)	TAR4 At1g34060 F12G12.12 F12G12.150	Arabidopsis thaliana (Mouse-ear cress)		Aminotransferase; Complete proteome; Membrane; Pyridoxal phosphate; Reference proteome; Transferase; Transmembrane; Transmembrane helix
SRR342023_TR25610_c0_g4_i1_len=1629	CLH2_ARATH	Q:1489-644,H:5-299	42.47%ID	E:6e-55	Chlorophyllase-2, chloroplastic	PF07224.8^Chlorophyllase^Chlorophyllase^2-295^E:4e-90`PF12740.4^Chlorophyllase2^Chlorophyllase enzyme^31-279^E:2.9e-87`PF03403.10^PAF-AH_p_II^Platelet-activating factor acetylhydrolase, isoform II^39-77^E:2.5e-06	0	2,221	0	4,248	0	4,126	0	4,111	0,788	0,104	CLH2_ARATH	Chlorophyllase-2, chloroplastic (AtCLH2) (EC 3.1.1.14) (Chlorophyll-chlorophyllido hydrolase 2) (Chlase 2)	CLH2 At5g43860 MQD19.22	Arabidopsis thaliana (Mouse-ear cress)	PATHWAY: Porphyrin-containing compound metabolism; chlorophyll degradation.	Chlorophyll catabolism; Chloroplast; Complete proteome; Hydrolase; Membrane; Plastid; Reference proteome; Transit peptide
SRR342023_TR26627_c0_g2_i1_len=1613	TPSGD_VITVI	Q:1496-147,H:19-460	53.33%ID	E:9e-164	(-)-germacrene D synthase	PF01397.18^Terpene_synth^Terpene synthase, N-terminal domain^19-199^E:1.8e-51`PF03936.13^Terpene_synth_C^Terpene synthase family, metal binding domain^230-456^E:1e-83	0	1,415	0	1,833	0	1,743	0	1,526	0,505	0,526	TPSGD_VITVI	(-)-germacrene D synthase (EC 4.2.3.22) (EC 4.2.3.75)	VIT_19s0014g04930 VIT_00014569001 VITISV_013313 VITISV_030783 Vv19s0014g04930	Vitis vinifera (Grape)	PATHWAY: Secondary metabolite biosynthesis; terpenoid biosynthesis.	Complete proteome; Cytoplasm; Lyase; Magnesium; Metal-binding; Reference proteome
SRR342023_TR28680_c0_g1_i1_len=1756	C71DC_CATRO	Q:1614-139,H:1-493	67.61%ID	E:0	Tabersonine 16-hydroxylase	PF00067.19^p450^Cytochrome P450^35-482^E:1.7e-100	0	1,005	0	2,356	0	2,329	0	2,247	0,008	0,742	C71DC_CATRO	Tabersonine 16-hydroxylase (EC 1.14.13.73) (Cytochrome P450 71D12) (Fragment)	CYP71D12	Catharanthus roseus (Madagascar periwinkle) (Vinca rosea)	PATHWAY: Alkaloid biosynthesis; vindoline biosynthesis; vindoline from tabersonine: step 1/6.	Endoplasmic reticulum; Heme; Iron; Metal-binding; Monooxygenase; NADP; Oxidoreductase
SRR342023_TR30099_c0_g1_i2_len=1398	CXE1_ACTER	Q:184-1128,H:21-333	45.6%ID	E:3e-97	Carboxylesterase 1	PF10340.6^DUF2424^Protein of unknown function (DUF2424)^67-184^E:2.3e-05`PF07859.10^Abhydrolase_3^alpha/beta hydrolase fold^82-301^E:1.6e-45	0	2,583	0	3,967	0	3,497	0	3,466	0,643	0,494	CXE1_ACTER	Carboxylesterase 1 (AeCXE1) (EC 3.1.1.1)	CXE1	Actinidia eriantha (Velvet vine) (Actinidia fulvicoma var. lanata)		3D-structure; Hydrolase; Serine esterase
SRR342023_TR30617_c3_g3_i4_len=672	C72A1_CATRO	Q:31-399,H:402-524	95.93%ID	E:2e-78	Secologanin synthase	PF00067.19^p450^Cytochrome P450^2-77^E:1.4e-22	0	1,353	0	2,779	0	2,7	0	2,21	0,542	0,17	C72A1_CATRO	Secologanin synthase (SLS) (EC 1.3.3.9) (CYPLXXII) (Cytochrome P450 72A1)	CYP72A1 CYP72 P450CR3	Catharanthus roseus (Madagascar periwinkle) (Vinca rosea)	PATHWAY: Alkaloid biosynthesis; secologanin biosynthesis.	Alkaloid metabolism; Heme; Iron; Membrane; Metal-binding; Monooxygenase; NADP; Oxidoreductase; Transmembrane; Transmembrane helix; Vacuole
SRR342023_TR31426_c4_g1_i1_len=2080	TT12_ARATH	Q:2006-438,H:1-504	46.29%ID	E:1e-139	.	PF01554.15^MatE^MatE^104-264^E:1.5e-34`PF01554.15^MatE^MatE^326-486^E:5.2e-30	0	0,964	0	2,758	0	1,506	0	1,131	1	0	DTX41_ARATH	Protein DETOXIFICATION 41 (AtDTX41) (Multidrug and toxic compound extrusion protein 41) (MATE protein 41) (Protein TANNIN-DEFICIENT SEED 3) (Protein TRANSPARENT TESTA 12)	DTX41 TDS3 TT12 At3g59030 F17J16_80	Arabidopsis thaliana (Mouse-ear cress)	PATHWAY: Secondary metabolite biosynthesis; flavonoid biosynthesis.	Antiport; Complete proteome; Flavonoid biosynthesis; Membrane; Reference proteome; Transmembrane; Transmembrane helix; Transport; Vacuole
SRR646596_TR1791_c0_g1_i1_len=791	NA	NA	NA	NA	NA	.	0	1,706	0	3,065	0	3,158	0	2,284	0,517	0,885	.	.	.	.	.	.
SRR646596_TR1932_c0_g1_i5_len=3832	MATE3_ARATH	Q:3363-1954,H:60-557	60.04%ID	E:5e-174	.	PF01554.15^MatE^MatE^122-289^E:6.2e-11`PF14667.3^Polysacc_synt_C^Polysaccharide biosynthesis C-terminal domain^247-338^E:8.8e-07`PF01554.15^MatE^MatE^361-491^E:7.6e-12	0	5,496	0	7,627	0	3,574	0	3,831	1	0,865	DTX45_ARATH	Protein DETOXIFICATION 45, chloroplastic (AtDTX45) (Multidrug and toxic compound extrusion protein 45) (MATE protein 45)	DTX45 At4g38380 F22I13.150	Arabidopsis thaliana (Mouse-ear cress)		Chloroplast; Complete proteome; Membrane; Plastid; Reference proteome; Transit peptide; Transmembrane; Transmembrane helix; Transport
SRR646596_TR8324_c0_g1_i1_len=1006	NA	NA	NA	NA	NA	PF14364.3^DUF4408^Domain of unknown function (DUF4408)^91-115^E:4.6e-08	0	1,919	0	3,1	0	1,691	0	1,693	0,381	1,787	.	.	.	.	.	.
SRR646596_TR12573_c1_g2_i3_len=1235	STSY_CATRO	Q:459-905,H:182-332	41.83%ID	E:1e-30	Strictosidine synthase	.	0	3,744	0	5,097	0	4,268	0	3,737	0,025	3,483	STSY_CATRO	Strictosidine synthase (EC 4.3.3.2)	STR1 SSS	Catharanthus roseus (Madagascar periwinkle) (Vinca rosea)	PATHWAY: Alkaloid biosynthesis; 3alpha(S)-strictosidine biosynthesis; 3alpha(S)-strictosidine from secologanin and tryptamine: step 1/1.	Alkaloid metabolism; Direct protein sequencing; Glycoprotein; Lyase; Signal; Vacuole
SRR646596_TR12743_c2_g4_i10_len=986	ARGE_ARATH	Q:631-353,H:346-438	55.91%ID	E:5e-29	Acetylornithine deacetylase	PF01546.25^Peptidase_M20^Peptidase family M20/M25/M40^5-96^E:5.4e-08	0	1,953	0	2,145	0	5,815	0	5,892	0,319	1,074	ARGE_ARATH	Acetylornithine deacetylase (EC 3.5.1.16) (N-acetylornithinase) (AO) (Acetylornithinase) (NAO)	At4g17830 dl4945w T6K21	Arabidopsis thaliana (Mouse-ear cress)	PATHWAY: Amino-acid biosynthesis; L-arginine biosynthesis; L-ornithine from N(2)-acetyl-L-ornithine (linear): step 1/1.	Alternative splicing; Amino-acid biosynthesis; Arginine biosynthesis; Cobalt; Complete proteome; Hydrolase; Metal-binding; Reference proteome; Zinc
SRR646596_TR12743_c2_g4_i13_len=877	ARGE_ARATH	Q:743-330,H:301-438	57.25%ID	E:5e-50	Acetylornithine deacetylase	.	0	1,814	0	2,795	0	5,061	0	5,647	0,392	-0,331	ARGE_ARATH	Acetylornithine deacetylase (EC 3.5.1.16) (N-acetylornithinase) (AO) (Acetylornithinase) (NAO)	At4g17830 dl4945w T6K21	Arabidopsis thaliana (Mouse-ear cress)	PATHWAY: Amino-acid biosynthesis; L-arginine biosynthesis; L-ornithine from N(2)-acetyl-L-ornithine (linear): step 1/1.	Alternative splicing; Amino-acid biosynthesis; Arginine biosynthesis; Cobalt; Complete proteome; Hydrolase; Metal-binding; Reference proteome; Zinc
SRR646596_TR12950_c0_g1_i3_len=1711	OPR3_SOLLC	Q:425-1573,H:9-390	79.37%ID	E:0	12-oxophytodienoate reductase 3	PF00724.17^Oxidored_FMN^NADH:flavin oxidoreductase / NADH oxidase family^10-359^E:4.7e-87	0	3,302	0	2,955	0	7,537	0	8,356	0,629	1,127	OPR3_SOLLC	12-oxophytodienoate reductase 3 (EC 1.3.1.42) (12-oxophytodienoate-10,11-reductase 3) (OPDA-reductase 3) (LeOPR3)	OPR3	Solanum lycopersicum (Tomato) (Lycopersicon esculentum)	PATHWAY: Lipid metabolism; oxylipin biosynthesis.	3D-structure; Complete proteome; FMN; Fatty acid biosynthesis; Fatty acid metabolism; Flavoprotein; Lipid biosynthesis; Lipid metabolism; NADP; Oxidoreductase; Oxylipin biosynthesis; Peroxisome; Reference proteome
SRR646596_TR18664_c0_g1_i1_len=2473	CBP1_ORYSJ	Q:1943-486,H:14-510	66.4%ID	E:0	Serine carboxypeptidase 1	PF00450.19^Peptidase_S10^Serine carboxypeptidase^36-493^E:5e-143	0	1,904	0	2,736	0	2,223	0	2,302	1	0,429	CBP1_ORYSJ	Serine carboxypeptidase 1 (EC 3.4.16.5) (Carboxypeptidase C) (Serine carboxypeptidase I) [Cleaved into: Serine carboxypeptidase 1 chain A (Serine carboxypeptidase I chain A); Serine carboxypeptidase 1 chain B (Serine carboxypeptidase I chain B)]	CBP1 Os12g0257000 LOC_Os12g15470	Oryza sativa subsp. japonica (Rice)		Carboxypeptidase; Complete proteome; Disulfide bond; Glycoprotein; Hydrolase; Protease; Reference proteome; Signal; Zymogen
SRR646596_TR18779_c1_g1_i1_len=852	PNAE_RAUSE	Q:85-564,H:9-171	48.47%ID	E:2e-40	Polyneuridine-aldehyde esterase	PF12146.5^Hydrolase_4^Serine aminopeptidase, S33^6-111^E:7.5e-08`PF00561.17^Abhydrolase_1^alpha/beta hydrolase fold^9-158^E:3.4e-13`PF12697.4^Abhydrolase_6^Alpha/beta hydrolase family^10-120^E:5.5e-10	0	4,549	0	6,574	0	4,482	0	4,866	0,132	3,003	PNAE_RAUSE	Polyneuridine-aldehyde esterase (EC 3.1.1.78) (Polyneuridine aldehyde esterase)	PNAE	Rauvolfia serpentina (Serpentine wood) (Ophioxylon serpentinum)	PATHWAY: Alkaloid biosynthesis; ajmaline biosynthesis.	3D-structure; Alkaloid metabolism; Direct protein sequencing; Hydrolase; Serine esterase
SRR646596_TR18779_c1_g1_i3_len=1079	SABP2_TOBAC	Q:91-852,H:5-260	44.19%ID	E:7e-57	Salicylic acid-binding protein 2	PF12146.5^Hydrolase_4^Serine aminopeptidase, S33^6-111^E:1.9e-07`PF00561.17^Abhydrolase_1^alpha/beta hydrolase fold^9-245^E:1.3e-13`PF12697.4^Abhydrolase_6^Alpha/beta hydrolase family^10-246^E:1.1e-12	0	4,454	0	4,446	0	4,607	0	4,939	0,003	1,973	SABP2_TOBAC	Salicylic acid-binding protein 2 (NtSABP2) (EC 3.1.1.-) (Methyl salicylate esterase)	SABP2	Nicotiana tabacum (Common tobacco)		3D-structure; Hydrolase; Immunity; Innate immunity; Plant defense
SRR646596_TR21402_c1_g1_i2_len=3911	Y3475_ARATH	Q:826-3738,H:21-1007	42.71%ID	E:0	Probable LRR receptor-like serine/threonine-protein kinase At3g47570	PF08263.9^LRRNT_2^Leucine rich repeat N-terminal domain^38-76^E:5.1e-09`PF13855.3^LRR_8^Leucine rich repeat^400-459^E:3.6e-08`PF00560.30^LRR_1^Leucine Rich Repeat^400-422^E:0.68`PF13855.3^LRR_8^Leucine rich repeat^423-483^E:3e-07`PF13855.3^LRR_8^Leucine rich repeat^521-579^E:4.8e-09`PF13516.3^LRR_6^Leucine Rich repeat^568-582^E:2`PF00069.22^Pkinase^Protein kinase domain^694-909^E:5e-36`PF07714.14^Pkinase_Tyr^Protein tyrosine kinase^694-911^E:1.8e-34`PF08263.9^LRRNT_2^Leucine rich repeat N-terminal domain^772-779^E:2900	0	2,266	0	5,234	0	4,43	0	4,736	1	1,138	Y3475_ARATH	Probable LRR receptor-like serine/threonine-protein kinase At3g47570 (EC 2.7.11.1)	At3g47570 F1P2.120	Arabidopsis thaliana (Mouse-ear cress)		ATP-binding; Cell membrane; Complete proteome; Glycoprotein; Kinase; Leucine-rich repeat; Membrane; Nucleotide-binding; Phosphoprotein; Receptor; Reference proteome; Repeat; Serine/threonine-protein kinase; Signal; Transferase; Transmembrane; Transmembrane helix
SRR646596_TR31742_c0_g1_i7_len=2326	DCE_SOLLC	Q:2207-1212,H:1-336	84.52%ID	E:0	Glutamate decarboxylase	PF00282.16^Pyridoxal_deC^Pyridoxal-dependent decarboxylase conserved domain^37-331^E:4.9e-88	0	3,434	0	2,215	0	2,377	0	2,055	0,407	0,797	DCE_SOLLC	Glutamate decarboxylase (GAD) (EC 4.1.1.15) (ERT D1)		Solanum lycopersicum (Tomato) (Lycopersicon esculentum)		Calmodulin-binding; Complete proteome; Decarboxylase; Lyase; Pyridoxal phosphate; Reference proteome
SRR646596_TR31742_c0_g1_i8_len=2832	DCE_SOLLC	Q:2044-650,H:28-502	79.37%ID	E:0	Glutamate decarboxylase	PF00282.16^Pyridoxal_deC^Pyridoxal-dependent decarboxylase conserved domain^14-358^E:2.2e-104	0	3,284	0	4,408	0	2,446	0	2,379	0,204	0,566	DCE_SOLLC	Glutamate decarboxylase (GAD) (EC 4.1.1.15) (ERT D1)		Solanum lycopersicum (Tomato) (Lycopersicon esculentum)		Calmodulin-binding; Complete proteome; Decarboxylase; Lyase; Pyridoxal phosphate; Reference proteome
SRR646596_TR31742_c0_g1_i10_len=2345	DCE_SOLLC	Q:1633-650,H:165-502	76.92%ID	E:0	Glutamate decarboxylase	PF00282.16^Pyridoxal_deC^Pyridoxal-dependent decarboxylase conserved domain^10-228^E:3.7e-66	0	3,335	0	2,43	0	2,589	0	2,396	0,067	0,625	DCE_SOLLC	Glutamate decarboxylase (GAD) (EC 4.1.1.15) (ERT D1)		Solanum lycopersicum (Tomato) (Lycopersicon esculentum)		Calmodulin-binding; Complete proteome; Decarboxylase; Lyase; Pyridoxal phosphate; Reference proteome
SRR646596_TR33896_c0_g4_i1_len=2624	DXS2_ORYSJ	Q:2171-213,H:59-712	78.44%ID	E:0	Probable 1-deoxy-D-xylulose-5-phosphate synthase 2, chloroplastic	PF13292.3^DXP_synthase_N^1-deoxy-D-xylulose-5-phosphate synthase^164-448^E:8.6e-109`PF00676.17^E1_dh^Dehydrogenase E1 component^276-335^E:9.1e-06`PF02779.21^Transket_pyr^Transketolase, pyrimidine binding domain^486-646^E:2.3e-41`PF02780.17^Transketolase_C^Transketolase, C-terminal domain^664-787^E:1.7e-34	0	1,605	0	4,372	0	3,228	0	4,001	1	0,027	DXS2_ORYSJ	Probable 1-deoxy-D-xylulose-5-phosphate synthase 2, chloroplastic (1-deoxyxylulose-5-phosphate synthase) (DXP synthase) (DXPS) (EC 2.2.1.7)	Os07g0190000 LOC_Os07g09190 OsJ_23394 OSJNBb0002L09.31 OSJNBb0003E08.7	Oryza sativa subsp. japonica (Rice)	PATHWAY: Metabolic intermediate biosynthesis; 1-deoxy-D-xylulose 5-phosphate biosynthesis; 1-deoxy-D-xylulose 5-phosphate from D-glyceraldehyde 3-phosphate and pyruvate: step 1/1.	Chloroplast; Complete proteome; Isoprene biosynthesis; Magnesium; Metal-binding; Plastid; Reference proteome; Thiamine biosynthesis; Thiamine pyrophosphate; Transferase; Transit peptide
SRR646604_TR1194_c0_g4_i4_len=1853	ARGE_ARATH	Q:235-1545,H:1-438	68.48%ID	E:0	Acetylornithine deacetylase	PF01546.25^Peptidase_M20^Peptidase family M20/M25/M40^95-434^E:5.5e-29`PF07687.11^M20_dimer^Peptidase dimerisation domain^203-317^E:3.3e-12	0	2,904	0	4,421	0	5,227	0	4,046	0,082	0,879	ARGE_ARATH	Acetylornithine deacetylase (EC 3.5.1.16) (N-acetylornithinase) (AO) (Acetylornithinase) (NAO)	At4g17830 dl4945w T6K21	Arabidopsis thaliana (Mouse-ear cress)	PATHWAY: Amino-acid biosynthesis; L-arginine biosynthesis; L-ornithine from N(2)-acetyl-L-ornithine (linear): step 1/1.	Alternative splicing; Amino-acid biosynthesis; Arginine biosynthesis; Cobalt; Complete proteome; Hydrolase; Metal-binding; Reference proteome; Zinc
SRR646604_TR1194_c0_g4_i14_len=1268	ARGE_ARATH	Q:268-1266,H:1-336	73%ID	E:4e-179	Acetylornithine deacetylase	PF01546.25^Peptidase_M20^Peptidase family M20/M25/M40^95-258^E:6.5e-20`PF07687.11^M20_dimer^Peptidase dimerisation domain^203-317^E:1.9e-12	0	1,82	0	3,406	0	4,266	0	5,023	0,002	2,52	ARGE_ARATH	Acetylornithine deacetylase (EC 3.5.1.16) (N-acetylornithinase) (AO) (Acetylornithinase) (NAO)	At4g17830 dl4945w T6K21	Arabidopsis thaliana (Mouse-ear cress)	PATHWAY: Amino-acid biosynthesis; L-arginine biosynthesis; L-ornithine from N(2)-acetyl-L-ornithine (linear): step 1/1.	Alternative splicing; Amino-acid biosynthesis; Arginine biosynthesis; Cobalt; Complete proteome; Hydrolase; Metal-binding; Reference proteome; Zinc
SRR646604_TR2878_c0_g1_i2_len=1076	NA	NA	NA	NA	NA	.	0	3,25	0	4,839	0	4,169	0	4,538	1	1,859	.	.	.	.	.	.
SRR646604_TR8057_c0_g1_i6_len=5066	LOX21_SOLTU	Q:115-2826,H:1-894	66.78%ID	E:0	Linoleate 13S-lipoxygenase 2-1, chloroplastic	PF01477.20^PLAT^PLAT/LH2 domain^144-212^E:6.2e-07`PF00305.16^Lipoxygenase^Lipoxygenase^225-892^E:0	0	2,447	0	5,449	0	6,736	0	7,329	1	0,136	LOX21_SOLTU	Linoleate 13S-lipoxygenase 2-1, chloroplastic (EC 1.13.11.12) (Lipoxygenase 2-1)	LOX2.1 LOX-H1	Solanum tuberosum (Potato)	PATHWAY: Lipid metabolism; oxylipin biosynthesis. {ECO:0000255|PROSITE-ProRule:PRU00726}.	Chloroplast; Complete proteome; Dioxygenase; Fatty acid biosynthesis; Fatty acid metabolism; Iron; Lipid biosynthesis; Lipid metabolism; Metal-binding; Oxidoreductase; Oxylipin biosynthesis; Plastid; Reference proteome; Thylakoid; Transit peptide
SRR646604_TR9752_c0_g1_i1_len=864	NA	NA	NA	NA	NA	.	0,001	6,563	0	6,677	0	3,025	0	3,12	1	2,348	.	.	.	.	.	.
SRR646604_TR22073_c0_g2_i6_len=3429	DRL29_ARATH	Q:264-2741,H:1-816	52.96%ID	E:0	Probable disease resistance protein At4g33300	PF05659.8^RPW8^Arabidopsis broad-spectrum mildew resistance protein RPW8^9-135^E:2.4e-13`PF00931.19^NB-ARC^NB-ARC domain^192-454^E:1.7e-38	0,001	2,395	0	3,727	0	4,916	0	5,249	0,517	0,827	DRL29_ARATH	Probable disease resistance protein At4g33300	At4g33300 F17M5.60	Arabidopsis thaliana (Mouse-ear cress)		ATP-binding; Coiled coil; Complete proteome; Leucine-rich repeat; Nucleotide-binding; Plant defense; Reference proteome; Repeat
SRR646604_TR29352_c0_g1_i4_len=1502	ACCO_ACTDE	Q:1329-382,H:3-319	84.54%ID	E:0	1-aminocyclopropane-1-carboxylate oxidase	PF14226.3^DIOX_N^non-haem dioxygenase in morphine synthesis N-terminal^60-150^E:5.3e-12`PF03171.17^2OG-FeII_Oxy^2OG-Fe(II) oxygenase superfamily^214-308^E:2.5e-28	0,001	0,749	0	3,171	0	2,486	0	2,109	0,039	0,492	ACCO_ACTDE	1-aminocyclopropane-1-carboxylate oxidase (ACC oxidase) (EC 1.14.17.4) (Ethylene-forming enzyme) (EFE)	ACO	Actinidia deliciosa (Kiwi)	PATHWAY: Alkene biosynthesis; ethylene biosynthesis via S-adenosyl-L-methionine; ethylene from S-adenosyl-L-methionine: step 2/2.	Ethylene biosynthesis; Fruit ripening; Iron; Metal-binding; Oxidoreductase; Vitamin C
SRR646604_TR32431_c0_g1_i1_len=1417	M2K9_ARATH	Q:1328-360,H:1-308	62.65%ID	E:1e-121	Mitogen-activated protein kinase kinase 9	PF00069.22^Pkinase^Protein kinase domain^57-321^E:8.3e-66`PF07714.14^Pkinase_Tyr^Protein tyrosine kinase^57-316^E:8.5e-39	0,001	1,271	0,001	1,441	0	3,055	0	3,093	0,046	-0,892	M2K9_ARATH	Mitogen-activated protein kinase kinase 9 (AtMKK9) (MAP kinase kinase 9) (EC 2.7.12.2)	MKK9 MAP2K9 At1g73500 T9L24.32	Arabidopsis thaliana (Mouse-ear cress)		ATP-binding; Complete proteome; Cytoplasm; Ethylene biosynthesis; Kinase; Nucleotide-binding; Nucleus; Phosphoprotein; Reference proteome; Serine/threonine-protein kinase; Stress response; Transferase
SRR646604_TR34700_c1_g2_i5_len=2039	C72A1_CATRO	Q:937-1776,H:245-524	95.71%ID	E:0	Secologanin synthase	PF00067.19^p450^Cytochrome P450^93-235^E:2.5e-16	0	1,192	0	3,316	0	1,996	0	1,648	0,002	1,077	C72A1_CATRO	Secologanin synthase (SLS) (EC 1.3.3.9) (CYPLXXII) (Cytochrome P450 72A1)	CYP72A1 CYP72 P450CR3	Catharanthus roseus (Madagascar periwinkle) (Vinca rosea)	PATHWAY: Alkaloid biosynthesis; secologanin biosynthesis.	Alkaloid metabolism; Heme; Iron; Membrane; Metal-binding; Monooxygenase; NADP; Oxidoreductase; Transmembrane; Transmembrane helix; Vacuole
SRR648705_TR27980_c0_g1_i1_len=4205	CBP1_ORYSJ	Q:497-1954,H:14-510	66.6%ID	E:0	Serine carboxypeptidase 1	PF00450.19^Peptidase_S10^Serine carboxypeptidase^36-493^E:4.5e-143	0	1,848	0	2,145	0	2,426	0	2,344	0,296	0,319	CBP1_ORYSJ	Serine carboxypeptidase 1 (EC 3.4.16.5) (Carboxypeptidase C) (Serine carboxypeptidase I) [Cleaved into: Serine carboxypeptidase 1 chain A (Serine carboxypeptidase I chain A); Serine carboxypeptidase 1 chain B (Serine carboxypeptidase I chain B)]	CBP1 Os12g0257000 LOC_Os12g15470	Oryza sativa subsp. japonica (Rice)		Carboxypeptidase; Complete proteome; Disulfide bond; Glycoprotein; Hydrolase; Protease; Reference proteome; Signal; Zymogen
SRR648705_TR41935_c0_g1_i2_len=2197	LPAAT_ARATH	Q:1700-600,H:48-415	79.62%ID	E:0	1-acylglycerol-3-phosphate O-acyltransferase	PF12740.4^Chlorophyllase2^Chlorophyllase enzyme^112-222^E:4.4e-07`PF00561.17^Abhydrolase_1^alpha/beta hydrolase fold^116-230^E:2.3e-19`PF12146.5^Hydrolase_4^Serine aminopeptidase, S33^116-224^E:2.8e-18`PF12697.4^Abhydrolase_6^Alpha/beta hydrolase family^118-384^E:1.1e-16`PF03096.11^Ndr^Ndr family^184-223^E:2e-05	0	1,339	0	3,362	0	1,917	0	2,304	1	-0,075	LPAAT_ARATH	1-acylglycerol-3-phosphate O-acyltransferase (EC 2.3.1.51) (Lipid droplet-binding protein CGI-58 homolog)	At4g24160 T19F6.4	Arabidopsis thaliana (Mouse-ear cress)		Acyltransferase; Alternative splicing; Complete proteome; Cytoplasm; Lipid biosynthesis; Lipid metabolism; Phospholipid biosynthesis; Phospholipid metabolism; Reference proteome; Transferase
SRR648707_TR2690_c0_g1_i3_len=2788	MYRS_QUEIL	Q:1901-2590,H:368-594	61.74%ID	E:7e-113	Myrcene synthase, chloroplastic	PF01397.18^Terpene_synth^Terpene synthase, N-terminal domain^67-243^E:1.2e-44	0	4,859	0	6,502	0	8,007	0	7,986	1	0,655	MYRS_QUEIL	Myrcene synthase, chloroplastic (EC 4.2.3.15)		Quercus ilex (Holly oak)		Chloroplast; Lyase; Magnesium; Manganese; Metal-binding; Plastid; Transit peptide
SRR648707_TR9308_c0_g1_i1_len=1181	MYB4_ORYSJ	Q:1016-669,H:1-116	87.93%ID	E:5e-69	Myb-related protein Myb4	PF00249.28^Myb_DNA-binding^Myb-like DNA-binding domain^14-61^E:5.9e-16`PF13921.3^Myb_DNA-bind_6^Myb-like DNA-binding domain^17-76^E:9.2e-13`PF00249.28^Myb_DNA-binding^Myb-like DNA-binding domain^67-112^E:2.4e-15`PF13921.3^Myb_DNA-bind_6^Myb-like DNA-binding domain^70-118^E:2.7e-06	0	1,776	0	2,594	0	3,04	0	2,509	0,024	1,327	MYB4_ORYSJ	Myb-related protein Myb4 (OsMyb4) (Transcription factor RLTR1)	MYB4 LTR1 Os04g0517100 LOC_Os04g43680 OSJNBa0073E02.6	Oryza sativa subsp. japonica (Rice)		Complete proteome; DNA-binding; Nucleus; Reference proteome; Repeat; Stress response; Transcription; Transcription regulation
SRR648707_TR13926_c0_g1_i3_len=4096	FAO2_LOTJA	Q:336-2456,H:31-748	60.5%ID	E:0	Long-chain-alcohol oxidase FAO2	PF00732.16^GMC_oxred_N^GMC oxidoreductase^246-508^E:4.1e-69`PF05199.10^GMC_oxred_C^GMC oxidoreductase^597-730^E:1.8e-19	0	4,249	0	2,008	0	2,802	0	2,911	0,195	-0,567	FAO2_LOTJA	Long-chain-alcohol oxidase FAO2 (EC 1.1.3.20) (Long-chain fatty alcohol oxidase 2)	FAO2	Lotus japonicus (Lotus corniculatus var. japonicus)		FAD; Flavoprotein; Membrane; Oxidoreductase; Transmembrane; Transmembrane helix
SRR648707_TR19558_c0_g2_i4_len=2022	C72A1_CATRO	Q:1888-317,H:1-524	99.43%ID	E:0	Secologanin synthase	PF00067.19^p450^Cytochrome P450^93-497^E:2e-87	0	1,248	0	3,931	0	2,416	0	1,945	0,038	0,612	C72A1_CATRO	Secologanin synthase (SLS) (EC 1.3.3.9) (CYPLXXII) (Cytochrome P450 72A1)	CYP72A1 CYP72 P450CR3	Catharanthus roseus (Madagascar periwinkle) (Vinca rosea)	PATHWAY: Alkaloid biosynthesis; secologanin biosynthesis.	Alkaloid metabolism; Heme; Iron; Membrane; Metal-binding; Monooxygenase; NADP; Oxidoreductase; Transmembrane; Transmembrane helix; Vacuole
SRR648707_TR19558_c0_g2_i12_len=1119	C72A1_CATRO	Q:797-315,H:373-524	91.3%ID	E:5e-95	Secologanin synthase	PF00067.19^p450^Cytochrome P450^1-114^E:1.3e-37	0	1,693	0	2,535	0	2,828	0	2,328	1	0,06	C72A1_CATRO	Secologanin synthase (SLS) (EC 1.3.3.9) (CYPLXXII) (Cytochrome P450 72A1)	CYP72A1 CYP72 P450CR3	Catharanthus roseus (Madagascar periwinkle) (Vinca rosea)	PATHWAY: Alkaloid biosynthesis; secologanin biosynthesis.	Alkaloid metabolism; Heme; Iron; Membrane; Metal-binding; Monooxygenase; NADP; Oxidoreductase; Transmembrane; Transmembrane helix; Vacuole
SRR648707_TR23313_c0_g1_i2_len=1386	CHIX_PEA	Q:1206-253,H:3-317	67.3%ID	E:5e-146	Endochitinase	PF00187.16^Chitin_bind_1^Chitin recognition protein^21-58^E:6.1e-15`PF00182.16^Glyco_hydro_19^Chitinase class I^81-312^E:4.5e-121	0,001	1,406	0	2,685	0	2	0	1,591	1	0,138	CHIX_PEA	Endochitinase (EC 3.2.1.14)		Pisum sativum (Garden pea)		Carbohydrate metabolism; Chitin degradation; Chitin-binding; Disulfide bond; Glycosidase; Hydrolase; Plant defense; Polysaccharide degradation; Signal
SRR648707_TR24142_c0_g2_i2_len=1738	ASB1_ARATH	Q:1229-[illegible]	81.65%ID	E:3e-127	Anthranilate synthase beta subunit 1, [illegible]	PF00117.25^GATase^Glutamine amidotransferase class-I^76-264^E:1.2e-[illegible]	0	1,293	0	3,533	0	3,355	0	2,974	0,574	0,269	ASB1_ARATH	Anthranilate synthase beta subunit 1, chloroplastic (EC 4.1.3.27) (Anthranilate synthase component 2-1) (Anthranilate synthase, glutamine amidotransferase component 2-1) (Protein TRYPTOPHAN [illegible]	ASB1 TRP4 WEI7 At1g25220 F4F7.39	Arabidopsis thaliana (Mouse-ear [illegible]	PATHWAY: Amino-acid biosynthesis; L-tryptophan biosynthesis; L-tryptophan from chorismate: [illegible]	Alternative splicing; Amino-acid biosynthesis; Aromatic amino acid biosynthesis; Chloroplast; Complete proteome; Glutamine amidotransferase; Lyase; Plastid; Reference [illegible]

[illegible]	ASB1_ARATH	[illegible]576,H:59-273	[illegible]	[illegible]	[illegible] chloroplastic	[illegible]47`PF07722.10^Peptidase_C26^Peptidase C26^144-249^E:3.4e-10	0	[illegible]	0	[illegible]	0	[illegible]	0	2,974	0,374	[illegible]	ASB1_ARATH	[illegible] BIOSYNTHESIS 4) (Protein WEAK ETHYLENE INSENSITIVE 7)	[illegible]	[illegible] cress)	[illegible] step 1/5.	[illegible] proteome; Transit peptide; Tryptophan biosynthesis
SRR648707_TR28936_c0_g1_i1_len=1053	FACR3_ARATH	Q:856-230,H:285-493	43.6%ID	E:2e-54	Fatty acyl-CoA reductase 3	PF03015.16^Sterile^Male sterility protein^121-217^E:6.3e-13	0	3,079	0	6,235	0	4,721	0	6,157	1	1,785	FACR3_ARATH	Fatty acyl-CoA reductase 3 (EC 1.2.1.n2) (Protein ECERIFERUM 4)	FAR3 CER4 At4g33790 T16L1.280	Arabidopsis thaliana (Mouse-ear cress)		Complete proteome; Endoplasmic reticulum; Lipid biosynthesis; Lipid metabolism; NADP; Oxidoreductase; Reference proteome
SRR648707_TR29295_c0_g1_i1_len=1842	U91A1_ARATH	Q:1691-309,H:9-466	56.77%ID	E:0	UDP-glycosyltransferase 91A1	PF00201.15^UDPGT^UDP-glucoronosyl and UDP-glucosyl transferase^275-444^E:2.7e-17	0	2,025	0	4,773	0	4,692	0	5,367	0,129	3,74	U91A1_ARATH	UDP-glycosyltransferase 91A1 (EC 2.4.1.-)	UGT91A1 At2g22590 T9I22.3	Arabidopsis thaliana (Mouse-ear cress)		Complete proteome; Glycosyltransferase; Reference proteome; Transferase
SRR648707_TR29907_c0_g1_i1_len=2033	PTR18_ARATH	Q:1955-237,H:12-590	74.18%ID	E:0	Protein NRT1/ PTR FAMILY 3.1	PF00854.18^PTR2^POT family^88-512^E:1.1e-101	0	1,926	0	4,91	0	1,34	0	0,906	1	-0,041	PTR18_ARATH	Protein NRT1/ PTR FAMILY 3.1 (AtNPF3.1)	NPF3.1 NITR At1g68570 F24J5.19	Arabidopsis thaliana (Mouse-ear cress)		Complete proteome; Membrane; Nitrate assimilation; Reference proteome; Transmembrane; Transmembrane helix; Transport
SRR924147_TR5199_c0_g1_i1_len=1978	WRK40_ARATH	Q:1610-600,H:6-302	47.93%ID	E:3e-84	Probable WRKY transcription factor 40	PF03106.12^WRKY^WRKY DNA -binding domain^181-239^E:3.3e-24	0	3,451	0	5,478	0	3,135	0	3,888	1	-0,336	WRK40_ARATH	Probable WRKY transcription factor 40 (WRKY DNA-binding protein 40)	WRKY40 At1g80840 F23A5.19	Arabidopsis thaliana (Mouse-ear cress)		Complete proteome; DNA-binding; Nucleus; Reference proteome; Transcription; Transcription regulation
SRR924147_TR15039_c0_g1_i1_len=3661	MATE3_ARATH	Q:3139-1730,H:60-557	60.04%ID	E:1e-174	.	PF01554.15^MatE^MatE^122-289^E:6.2e-11`PF14667.3^Polysacc_synt_C^Polysaccharide biosynthesis C-terminal domain^247-338^E:8.8e-07`PF01554.15^MatE^MatE^361-491^E:7.6e-12	0	1,809	0	3,862	0	1,821	0	2,35	1	0	DTX45_ARATH	Protein DETOXIFICATION 45, chloroplastic (AtDTX45) (Multidrug and toxic compound extrusion protein 45) (MATE protein 45)	DTX45 At4g38380 F22I13.150	Arabidopsis thaliana (Mouse-ear cress)		Chloroplast; Complete proteome; Membrane; Plastid; Reference proteome; Transit peptide; Transmembrane; Transmembrane helix; Transport
SRR924147_TR30716_c0_g1_i1_len=798	NA	NA	NA	NA	NA	.	0	2,33	0	4,244	0	6,032	0	3,779	0,904	-0,113	.	.	.	.	.	.
SRR924147_TR31524_c0_g1_i1_len=1812	AAT3_ARATH	Q:160-1470,H:13-449	82.61%ID	E:0	Aspartate aminotransferase 3, chloroplastic	PF00155.18^Aminotran_1_2^Aminotransferase class I and II^77-443^E:2.9e-97	0	2,721	0	6,191	0	5,396	0	5,012	1	-0,36	AAT3_ARATH	Aspartate aminotransferase 3, chloroplastic (EC 2.6.1.1) (Protein YELLOW-LEAF-SPECIFIC GENE 4) (Transaminase A)	ASP3 YLS4 At5g11520 F15N18.110	Arabidopsis thaliana (Mouse-ear cress)		Aminotransferase; Chloroplast; Complete proteome; Plastid; Pyridoxal phosphate; Reference proteome; Transferase; Transit peptide
SRR924148_TR30094_c0_g2_i2_len=1350	YL126_YEAST	Q:972-391,H:1-205	31.43%ID	E:3e-21	Putative glutamine amidotransferase YLR126C	PF00117.25^GATase^Glutamine amidotransferase class-I^55-192^E:3.7e-15`PF04204.13^HTS^Homoserine O-succinyltransferase^57-215^E:6.2e-05`PF07722.10^Peptidase_C26^Peptidase C26^84-189^E:1.1e-05	0	1,391	0	2,229	0	2,523	0	2,678	0,184	-0,362	YL126_YEAST	Putative glutamine amidotransferase YLR126C (EC 2.4.2.-)	YLR126C L3105	Saccharomyces cerevisiae (strain ATCC 204508 / S288c) (Baker's yeast)		Complete proteome; Cytoplasm; Glutamine amidotransferase; Reference proteome; Transferase
SRR342017_TR25892_c2_g4_i6_len=4889	POLX_TOBAC	Q:4888-3482,H:858-1323	55.65%ID	E:1e-150	Retrovirus-related Pol polyprotein from transposon TNT 1-94	PF14223.3^Retrotran_gag_2^gag-polypeptide of LTR copia-type^59-187^E:7e-34`PF00098.20^zf-CCHC^Zinc knuckle^227-242^E:0.00034`PF13976.3^gag_pre-integrs^GAG-pre-integrase domain^397-464^E:1.3e-14`PF00665.23^rve^Integrase core domain^480-594^E:2.5e-26	0	4,536	0	-1,503	0	1,941	0	1,869	0	3,153	POLX_TOBAC	Retrovirus-related Pol polyprotein from transposon TNT 1-94 [Includes: Protease (EC 3.4.23.-); Reverse transcriptase (EC 2.7.7.49); Endonuclease]		Nicotiana tabacum (Common tobacco)		Aspartyl protease; Endonuclease; Hydrolase; Metal-binding; Nuclease; Nucleotidyltransferase; Protease; RNA-directed DNA polymerase; Transferase; Transposable element; Zinc; Zinc-finger
SRR342022_TR3089_c3_g3_i9_len=1204	RNLX_SOLLC	Q:850-248,H:30-230	64.85%ID	E:9e-91	Intracellular ribonuclease LX	PF00445.15^Ribonuclease_T2^Ribonuclease T2 family^1-114^E:6.3e-34	0	0,87	0,276	0,306	0	1,692	0	1,175	0	1,479	RNLX_SOLLC	Intracellular ribonuclease LX (RNase LX) (EC 3.1.27.1)	RNALX	Solanum lycopersicum (Tomato) (Lycopersicon esculentum)		Complete proteome; Cytoplasm; Direct protein sequencing; Disulfide bond; Endonuclease; Hydrolase; Nuclease; Reference proteome; Stress response; Zymogen
SRR122238_TR12534_c1_g6_i3_len=2122	HSP7C_PETHY	Q:119-1972,H:1-618	96.12%ID	E:0	Heat shock cognate 70 kDa protein	PF00012.17^HSP70^Hsp70 protein^9-618^E:3.4e-267`PF06723.10^MreB_Mbl^MreB/Mbl protein^127-380^E:5.6e-14	0,129	0,886	0	4,682	0	1,021	0,001	0,838	0	1,606	HSP7C_PETHY	Heat shock cognate 70 kDa protein	HSP70	Petunia hybrida (Petunia)		ATP-binding; Nucleotide-binding; Stress response
SRR122238_TR12756_c0_g1_i1_len=1660	ACCH1_ARATH	Q:307-1347,H:20-365	48.29%ID	E:1e-114	1-aminocyclopropane-1-carboxylate oxidase homolog 1	PF14226.3^DIOX_N^non-haem dioxygenase in morphine synthesis N-terminal^110-209^E:8.6e-18`PF03171.17^2OG-FeII_Oxy^2OG-Fe(II) oxygenase superfamily^270-362^E:1.4e-21	0,377	0,187	0	0,849	0	2,105	0	2,748	0	1,025	ACCH1_ARATH	1-aminocyclopropane-1-carboxylate oxidase homolog 1	At1g06620 F12K11.24 F12K11.6	Arabidopsis thaliana (Mouse-ear cress)		Complete proteome; Iron; Metal-binding; Oxidoreductase; Reference proteome
SRR342017_TR9688_c1_g2_i3_len=830	NA	NA	NA	NA	NA	PF00168.27^C2^C2 domain^56-162^E:3.9e-08	0,287	0,302	0	1,777	0	1,886	0	2,04	0	9,28	.	.	.	.	.	.
SRR342017_TR30366_c0_g1_i1_len=668	NA	NA	NA	NA	NA	.	0,158	1,412	0	2,791	0	1,863	0	1,74	0	2,509	.	.	.	.	.	.
SRR342019_TR16142_c0_g1_i2_len=862	NA	NA	NA	NA	NA	PF04570.11^zf-FLZ^zinc-finger of the FCS-type, C2-C2^113-161^E:3.1e-24	0,326	0,324	0	1,506	0	1,481	0	1,705	0	2,147	.	.	.	.	.	.
SRR342022_TR17840_c0_g1_i1_len=2707	NA	NA	NA	NA	NA	.	0,124	0,416	0	1,258	0	1,561	0	1,644	0	3,714	.	.	.	.	.	.
SRR646596_TR12743_c2_g5_i3_len=1246	ARGE_ARATH	Q:268-1242,H:1-303	68.09%ID	E:4e-156	Acetylornithine deacetylase	PF07687.11^M20_dimer^Peptidase dimerisation domain^62-158^E:1.8e-08	0,001	1,635	0	3,75	0	4,697	0	5,127	0	1,751	ARGE_ARATH	Acetylornithine deacetylase (EC 3.5.1.16) (N-acetylornithinase) (AO) (Acetylornithinase) (NAO)	At4g17830 dl4945w T6K21	Arabidopsis thaliana (Mouse-ear cress)	PATHWAY: Amino-acid biosynthesis; L-arginine biosynthesis; L-ornithine from N(2)-acetyl-L-ornithine (linear): step 1/1.	Alternative splicing; Amino-acid biosynthesis; Arginine biosynthesis; Cobalt; Complete proteome; Hydrolase; Metal-binding; Reference proteome; Zinc
SRR646596_TR30289_c1_g1_i1_len=758	NLTP_HELAN	Q:135-485,H:1-116	52.54%ID	E:7e-27	Non-specific lipid-transfer protein	PF14368.3^LTP_2^Probable lipid transfer^15-105^E:1.4e-05`PF00234.19^Tryp_alpha_amyl^Protease inhibitor/seed storage/LTP family^30-113^E:1e-07	0,032	0,463	0	1,451	0	2,064	0	1,023	0	0,967	NLTP_HELAN	Non-specific lipid-transfer protein (LTP) (NsLTP) (SDI-9)		Helianthus annuus (Common sunflower)		Disulfide bond; Lipid-binding; Signal; Transport
SRR648705_TR41316_c0_g1_i3_len=1005	NA	NA	NA	NA	NA	PF06232.8^ATS3^Embryo-specific protein 3, (ATS3)^30-132^E:4.1e-07`PF01477.20^PLAT^PLAT/LH2 domain^36-153^E:2.2e-14	0,008	0,67	0	3,561	0	4,273	0	3,751	0	1,721	.	.	.	.	.	.
SRR1144634_TR26877_c0_g2_i1_len=505	NA	NA	NA	NA	NA	PF14226.3^DIOX_N^non-haem dioxygenase in morphine synthesis N-terminal^61-124^E:5.8e-17	0	1,565	0	4,074	0	2,211	0	2,107	0	2,281	.	.	.	.	.	.
SRR122238_TR14259_c0_g1_i1_len=1837	TAR4_ARATH	Q:1756-437,H:6-449	52.8%ID	E:4e-159	Tryptophan aminotransferase-related protein 4	PF04863.10^EGF_alliinase^Alliinase EGF-like domain^40-94^E:2.4e-27`PF04864.10^Alliinase_C^Allinase^96-451^E:9.5e-143`PF00155.18^Aminotran_1_2^Aminotransferase class I and II^137-432^E:1.3e-06	0	5,486	0	7,047	0	7,695	0	7,198	0	1,352	TAR4_ARATH	Tryptophan aminotransferase-related protein 4 (EC 2.6.1.-)	TAR4 At1g34060 F12G12.12 F12G12.150	Arabidopsis thaliana (Mouse-ear cress)		Aminotransferase; Complete proteome; Membrane; Pyridoxal phosphate; Reference proteome; Transferase; Transmembrane; Transmembrane helix
SRR122238_TR47400_c1_g1_i7_len=865	C72A1_CATRO	Q:103-846,H:1-248	97.98%ID	E:2e-178	Secologanin synthase	PF00067.19^p450^Cytochrome P450^93-236^E:2e-16	0	1,102	0	4,02	0	2,387	0	2,422	0	2,967	C72A1_CATRO	Secologanin synthase (SLS) (EC 1.3.3.9) (CYPLXXII) (Cytochrome P450 72A1)	CYP72A1 CYP72 P450CR3	Catharanthus roseus (Madagascar periwinkle) (Vinca rosea)	PATHWAY: Alkaloid biosynthesis; secologanin biosynthesis.	Alkaloid metabolism; Heme; Iron; Membrane; Metal-binding; Monooxygenase; NADP; Oxidoreductase; Transmembrane; Transmembrane helix; Vacuole
SRR122238_TR59774_c0_g1_i2_len=1287	STSY_RAUSE	Q:229-1095,H:37-324	43.34%ID	E:3e-75	Strictosidine synthase	PF08450.9^SGL^SMP-30/Gluconolaconase/LRE-like region^122-313^E:1.6e-06`PF03088.13^Str_synth^Strictosidine synthase^144-231^E:4.6e-25	0	2,435	0	3,284	0	3,742	0	2,723	0	2,506	STSY_RAUSE	Strictosidine synthase (EC 4.3.3.2)	STR1	Rauvolfia serpentina (Serpentine wood) (Ophioxylon serpentinum)	PATHWAY: Alkaloid biosynthesis; 3alpha(S)-strictosidine biosynthesis; 3alpha(S)-strictosidine from secologanin and tryptamine: step 1/1.	3D-structure; Alkaloid metabolism; Direct protein sequencing; Glycoprotein; Lyase; Signal; Vacuole
SRR122238_TR61939_c0_g1_i8_len=3198	NES1_FRAAN	Q:2896-1379,H:14-515	53.95%ID	E:3e-168	(3S,6E)-nerolidol synthase 1	PF01397.18^Terpene_synth^Terpene synthase, N-terminal domain^67-224^E:3.5e-42`PF03936.13^Terpene_synth_C^Terpene synthase family, metal binding domain^258-522^E:8.5e-76	0	3,04	0	4,257	0	2,385	0	2,974	0	2,351	NES1_FRAAN	(3S,6E)-nerolidol synthase 1 (FaNES1) (EC 4.2.3.48)		Fragaria ananassa (Strawberry)	PATHWAY: Secondary metabolite biosynthesis; terpenoid biosynthesis.	Cytoplasm; Lyase; Magnesium; Manganese; Metal-binding
SRR1271857_TR24914_c0_g2_i15_len=2512	DCE_PETHY	Q:142-1137,H:1-335	89.25%ID	E:0	Glutamate decarboxylase	PF00282.16^Pyridoxal_deC^Pyridoxal-dependent decarboxylase conserved domain^37-331^E:1.8e-92	0	2,48	0	2,012	0	2,402	0	2,212	0	0,797	DCE_PETHY	Glutamate decarboxylase (GAD) (EC 4.1.1.15)	GAD	Petunia hybrida (Petunia)		3D-structure; Calmodulin-binding; Decarboxylase; Lyase; Pyridoxal phosphate
SRR1271859_TR4959_c0_g1_i6_len=2049	CBP1_ORYSJ	Q:860-1852,H:136-469	65.87%ID	E:4e-179	Serine carboxypeptidase 1	PF00450.19^Peptidase_S10^Serine carboxypeptidase^2-320^E:9.6e-76	0	2,169	0	3,34	0	2,614	0	2,554	0	2,131	CBP1_ORYSJ	Serine carboxypeptidase 1 (EC 3.4.16.5) (Carboxypeptidase C) (Serine carboxypeptidase I) [Cleaved into: Serine carboxypeptidase 1 chain A (Serine carboxypeptidase I chain A); Serine carboxypeptidase 1 chain B (Serine carboxypeptidase I chain B)]	CBP1 Os12g0257000 LOC_Os12g15470	Oryza sativa subsp. japonica (Rice)		Carboxypeptidase; Complete proteome; Disulfide bond; Glycoprotein; Hydrolase; Protease; Reference proteome; Signal; Zymogen
SRR342019_TR29234_c2_g1_i5_len=927	C72A1_CATRO	Q:156-926,H:1-257	98.44%ID	E:0	Secologanin synthase	PF00067.19^p450^Cytochrome P450^93-238^E:8.5e-17	0	1,041	0	5,643	0	1,943	0	1,745	0	1,607	C72A1_CATRO	Secologanin synthase (SLS) (EC 1.3.3.9) (CYPLXXII) (Cytochrome P450 72A1)	CYP72A1 CYP72 P450CR3	Catharanthus roseus (Madagascar periwinkle) (Vinca rosea)	PATHWAY: Alkaloid biosynthesis; secologanin biosynthesis.	Alkaloid metabolism; Heme; Iron; Membrane; Metal-binding; Monooxygenase; NADP; Oxidoreductase; Transmembrane; Transmembrane helix; Vacuole
SRR342019_TR38836_c2_g1_i2_len=540	DCE_SOLLC	Q:116-538,H:1-142	83.1%ID	E:3e-69	Glutamate decarboxylase	PF00282.16^Pyridoxal_deC^Pyridoxal-dependent decarboxylase conserved domain^37-141^E:5.5e-26	0	2,65	0	6,769	0	3,135	0	3,703	0	1,973	DCE_SOLLC	Glutamate decarboxylase (GAD) (EC 4.1.1.15) (ERT D1)		Solanum lycopersicum (Tomato) (Lycopersicon esculentum)		Calmodulin-binding; Complete proteome; Decarboxylase; Lyase; Pyridoxal phosphate; Reference proteome
SRR342022_TR17158_c1_g3_i4_len=1780	GCL1_ARATH	Q:212-1495,H:1-433	63.64%ID	E:0	LanC-like protein GCL1	PF05147.10^LANC_like^Lanthionine synthetase C-like protein^71-427^E:2.4e-86	0	2,748	0	4,475	0	2,795	0	3,51	0	7,916	GCL1_ARATH	LanC-like protein GCL1 (G protein-coupled receptor 2-like protein 1) (Protein GCR2-like 1)	GCL1 At5g65280 MQN23.23	Arabidopsis thaliana (Mouse-ear cress)		Complete proteome; Reference proteome
SRR342022_TR26103_c2_g5_i3_len=1741	FLS_PETCR	Q:1472-630,H:44-328	38.06%ID	E:2e-53	Flavonol synthase/flavanone 3-hydroxylase	PF14226.3^DIOX_N^non-haem dioxygenase in morphine synthesis N-terminal^3-70^E:1.2e-10`PF03171.17^2OG-FeII_Oxy^2OG-Fe(II) oxygenase superfamily^119-211^E:2e-23	0	1,533	0	2,226	0	2,711	0	2,365	0	1,324	FLS_PETCR	Flavonol synthase/flavanone 3-hydroxylase (EC 1.14.11.23) (EC 1.14.11.9)	FLS	Petroselinum crispum (Parsley) (Petroselinum hortense)	PATHWAY: Secondary metabolite biosynthesis; flavonoid biosynthesis.	Dioxygenase; Flavonoid biosynthesis; Iron; Metal-binding; Oxidoreductase; Vitamin C
SRR342022_TR27013_c0_g6_i2_len=1057	SABP2_TOBAC	Q:799-38,H:5-260	44.19%ID	E:5e-57	Salicylic acid-binding protein 2	PF12146.5^Hydrolase_4^Serine aminopeptidase, S33^6-111^E:1.9e-07`PF00561.17^Abhydrolase_1^alpha/beta hydrolase fold^9-245^E:1.3e-13`PF12697.4^Abhydrolase_6^Alpha/beta hydrolase family^10-246^E:1.1e-12	0	3,473	0	5,298	0	5,147	0	5,337	0	2,625	SABP2_TOBAC	Salicylic acid-binding protein 2 (NtSABP2) (EC 3.1.1.-) (Methyl salicylate esterase)	SABP2	Nicotiana tabacum (Common tobacco)		3D-structure; Hydrolase; Immunity; Innate immunity; Plant defense
SRR342022_TR28792_c1_g1_i8_len=2262	PTR21_ARATH	Q:171-1892,H:45-617	53.9%ID	E:0	Protein NRT1/ PTR FAMILY 2.13	PF07690.13^MFS_1^Major Facilitator Superfamily^82-547^E:3e-10`PF00854.18^PTR2^POT family^137-568^E:2.2e-82	0	1,341	0	2,924	0	1,864	0	1,751	0,001	1,37	PTR21_ARATH	Protein NRT1/ PTR FAMILY 2.13 (AtNPF2.13) (Nitrate transporter 1.7)	NPF2.13 NRT1.7 At1g69870 T17F3.10	Arabidopsis thaliana (Mouse-ear cress)		Cell membrane; Complete proteome; Membrane; Nitrate assimilation; Reference proteome; Symport; Transmembrane; Transmembrane helix; Transport
SRR342023_TR7070_c5_g1_i3_len=551	ACCO_PEA	Q:128-487,H:144-261	43.8%ID	E:3e-27	1-aminocyclopropane-1-carboxylate oxidase	.	0	1,747	0	2,378	0	2,662	0	2,471	0	1,362	ACCO_PEA	1-aminocyclopropane-1-carboxylate oxidase (ACC oxidase) (EC 1.14.17.4) (Ethylene-forming enzyme) (EFE)	ACO	Pisum sativum (Garden pea)	PATHWAY: Alkene biosynthesis; ethylene biosynthesis via S-adenosyl-L-methionine; ethylene from S-adenosyl-L-methionine: step 2/2.	Ethylene biosynthesis; Fruit ripening; Iron; Metal-binding; Oxidoreductase; Vitamin C
SRR646596_TR12573_c1_g2_i1_len=1709	STSY_RAUSE	Q:666-1373,H:89-324	42.5%ID	E:5e-56	Strictosidine synthase	PF08450.9^SGL^SMP-30/Gluconolaconase/LRE-like region^31-246^E:6.3e-07`PF03088.13^Str_synth^Strictosidine synthase^77-164^E:2.9e-25	0	3,755	0	5,773	0	4,127	0	3,509	0	3,344	STSY_RAUSE	Strictosidine synthase (EC 4.3.3.2)	STR1	Rauvolfia serpentina (Serpentine wood) (Ophioxylon serpentinum)	PATHWAY: Alkaloid biosynthesis; 3alpha(S)-strictosidine biosynthesis; 3alpha(S)-strictosidine from secologanin and tryptamine: step 1/1.	3D-structure; Alkaloid metabolism; Direct protein sequencing; Glycoprotein; Lyase; Signal; Vacuole
SRR646596_TR26064_c0_g2_i1_len=1017	NA	NA	NA	NA	NA	PF00257.16^Dehydrin^Dehydrin^10-176^E:4.1e-43	0	2,413	0	4,94	0	5,355	0	3,389	0	4,133	.	.	.	.	.	.
SRR646596_TR31742_c0_g1_i6_len=2241	DCE_SOLLC	Q:2122-650,H:1-502	79.08%ID	E:0	Glutamate decarboxylase	PF00282.16^Pyridoxal_deC^Pyridoxal-dependent decarboxylase conserved domain^37-381^E:2.1e-104	0	2,48	0	2,982	0	2,709	0	2,648	0	2,967	DCE_SOLLC	Glutamate decarboxylase (GAD) (EC 4.1.1.15) (ERT D1)		Solanum lycopersicum (Tomato) (Lycopersicon esculentum)		Calmodulin-binding; Complete proteome; Decarboxylase; Lyase; Pyridoxal phosphate; Reference proteome
SRR646604_TR34700_c1_g2_i4_len=1523	C72A1_CATRO	Q:108-1259,H:1-384	98.96%ID	E:0	Secologanin synthase	PF00067.19^p450^Cytochrome P450^90-382^E:9e-43	0	1,288	0	4,215	0	2,46	0	2,042	0	1,323	C72A1_CATRO	Secologanin synthase (SLS) (EC 1.3.3.9) (CYPLXXII) (Cytochrome P450 72A1)	CYP72A1 CYP72 P450CR3	Catharanthus roseus (Madagascar periwinkle) (Vinca rosea)	PATHWAY: Alkaloid biosynthesis; secologanin biosynthesis.	Alkaloid metabolism; Heme; Iron; Membrane; Metal-binding; Monooxygenase; NADP; Oxidoreductase; Transmembrane; Transmembrane helix; Vacuole
SRR924147_TR18297_c0_g1_i13_len=1440	C72A1_CATRO	Q:120-1265,H:1-382	99.48%ID	E:0	Secologanin synthase	PF00067.19^p450^Cytochrome P450^93-382^E:1.7e-42	0	1,083	0	4,882	0	2,152	0	1,771	0	1,126	C72A1_CATRO	Secologanin synthase (SLS) (EC 1.3.3.9) (CYPLXXII) (Cytochrome P450 72A1)	CYP72A1 CYP72 P450CR3	Catharanthus roseus (Madagascar periwinkle) (Vinca rosea)	PATHWAY: Alkaloid biosynthesis; secologanin biosynthesis.	Alkaloid metabolism; Heme; Iron; Membrane; Metal-binding; Monooxygenase; NADP; Oxidoreductase; Transmembrane; Transmembrane helix; Vacuole
SRR924148_TR15471_c0_g2_i2_len=1833	DIOX3_PAPSO	Q:1505-477,H:18-360	33.43%ID	E:2e-55	Codeine O-demethylase	PF14226.3^DIOX_N^non-haem dioxygenase in morphine synthesis N-terminal^61-165^E:1.6e-22`PF03171.17^2OG-FeII_Oxy^2OG-Fe(II) oxygenase superfamily^214-306^E:3.7e-23	0	2,023	0	2,558	0	2,711	0	2,411	0	1,708	DIOX3_PAPSO	Codeine O-demethylase (EC 1.14.11.32)	CODM DIOX3	Papaver somniferum (Opium poppy)	PATHWAY: Alkaloid biosynthesis; morphine biosynthesis.	Alkaloid metabolism; Dioxygenase; Iron; Metal-binding; Methyltransferase; Oxidoreductase; Transferase
SRR1144633_TR19640_c0_g2_i1_len=920	RPBAL_ARATH	Q:84-290,H:1-69	91.3%ID	E:4e-39	DNA-directed RNA polymerase subunit 10-like protein {ECO:0000303|PubMed:19110459}	.	0	7,498	0	6,832	0	-7,425	0	-5,851	1	0,028	RPBAL_ARATH	DNA-directed RNA polymerase subunit 10-like protein (ABC10) (DNA-directed RNA polymerase III subunit L) (DNA-directed RNA polymerases I, II, and III subunit RPABC5) (RNA polymerases I, II, and III subunit ABC5) (RPB10 homolog)	NRPB10L At1g61700 T13M11.6	Arabidopsis thaliana (Mouse-ear cress)		Complete proteome; DNA-directed RNA polymerase; Metal-binding; Nucleus; Reference proteome; Transcription; Zinc
SRR1144633_TR29660_c0_g1_i3_len=1731	NA	NA	NA	NA	NA	.	0	1,512	0	1,371	0	-0,782	0,011	-0,53	0,484	-0,42	.	.	.	.	.	.
SRR1144633_TR33857_c0_g1_i1_len=1899	GGPPS_CATRO	Q:1436-372,H:1-357	96.92%ID	E:0	Geranylgeranyl pyrophosphate synthase, chloroplastic	PF00348.14^polyprenyl_synt^Polyprenyl synthetase^117-351^E:1.8e-57	0,001	1,025	0	1,71	0,002	0,934	0,39	0,3	0,821	0,109	GGPPS_CATRO	Geranylgeranyl pyrophosphate synthase, chloroplastic (GGPP synthase) (GGPS) (EC 2.5.1.-) ((2E,6E)-farnesyl diphosphate synthase) (Dimethylallyltranstransferase) (EC 2.5.1.1) (Farnesyl diphosphate synthase) (Farnesyltranstransferase) (EC 2.5.1.29) (Geranyltranstransferase) (EC 2.5.1.10)	GGPS1 GGC1	Catharanthus roseus (Madagascar periwinkle) (Vinca rosea)	PATHWAY: Isoprenoid biosynthesis; farnesyl diphosphate biosynthesis; farnesyl diphosphate from geranyl diphosphate and isopentenyl diphosphate: step 1/1.; PATHWAY: Isoprenoid biosynthesis; geranyl diphosphate biosynthesis; geranyl diphosphate from dimethylallyl diphosphate and isopentenyl diphosphate: step 1/1.; PATHWAY: Isoprenoid biosynthesis; geranylgeranyl diphosphate biosynthesis; geranylgeranyl diphosphate from farnesyl diphosphate and isopentenyl diphosphate: step 1/1.	Carotenoid biosynthesis; Chloroplast; Isoprene biosynthesis; Magnesium; Metal-binding; Plastid; Transferase; Transit peptide
SRR1144633_TR37464_c0_g3_i1_len=269	NA	NA	NA	NA	NA	.	0	1,318	0	2,312	0,255	0,444	0,223	0,476	1	-0,006	.	.	.	.	.	.
SRR1144634_TR19041_c0_g2_i1_len=1676	GOLS1_AJURE	Q:1466-669,H:1-261	84.96%ID	E:3e-163	Galactinol synthase 1	PF01501.17^Glyco_transf_8^Glycosyl transferase family 8^32-265^E:9.3e-40	0	1,176	0	1,661	0,083	0,367	0,063	0,392	1	1,721	GOLS1_AJURE	Galactinol synthase 1 (ArGolS1) (GolS-1) (EC 2.4.1.123)	GOLS1	Ajuga reptans (Bugle)		Carbohydrate metabolism; Cytoplasm; Galactose metabolism; Glycosyltransferase; Manganese; Metal-binding; Transferase
SRR1144634_TR23505_c0_g1_i1_len=660	NA	NA	NA	NA	NA	PF00810.15^ER_lumen_recept^ER lumen protein retaining receptor^1-49^E:2.2e-18	0	7,836	0	7,051	0,129	-3,245	0,129	-4,862	1	0	.	.	.	.	.	.
SRR1144634_TR39096_c0_g1_i1_len=869	NA	NA	NA	NA	NA	PF02466.16^Tim17^Tim17/Tim22/Tim23/Pmp24 family^35-127^E:1e-09	0	8,686	0	7,332	0	-6,345	0	-6,968	1	0	.	.	.	.	.	.
SRR122238_TR4557_c0_g1_i1_len=503	NA	NA	NA	NA	NA	.	0	1,135	0	1,335	0,503	0,167	0,017	0,568	0,053	-0,586	.	.	.	.	.	.
SRR122238_TR20218_c1_g1_i2_len=629	NA	NA	NA	NA	NA	PF13499.3^EF-hand_7^EF-hand domain pair^24-98^E:7.4e-10`PF13202.3^EF-hand_5^EF hand^26-47^E:0.00024`PF13202.3^EF-hand_5^EF hand^75-96^E:0.0013	0	1,048	0	1,07	0,005	0,693	0,223	0,313	0,894	0,051	.	.	.	.	.	.
SRR122238_TR58145_c0_g1_i1_len=868	NA	NA	NA	NA	NA	.	0	3,359	0	5,823	0,228	1,252	1	-0,282	1	0,304	.	.	.	.	.	.
SRR1271857_TR38410_c0_g1_i1_len=573	NA	NA	NA	NA	NA	.	0	1,544	0	0,791	0,145	-0,322	0,473	0,16	0,223	0,268	.	.	.	.	.	.
SRR1271859_TR2696_c0_g1_i1_len=1190	21KD_DAUCA	Q:353-841,H:27-191	46.67%ID	E:4e-46	21 kDa protein	PF04043.12^PMEI^Plant invertase/pectin methylesterase inhibitor^48-201^E:1.1e-34	0	1,414	0	2,46	0,088	-1,549	0,16	-1,217	0,402	-1,159	21KD_DAUCA	21 kDa protein (1.2 protein)		Daucus carota (Wild carrot)		Signal
SRR1271859_TR7367_c0_g1_i2_len=912	RBS_MALSP	Q:673-173,H:17-183	85.03%ID	E:7e-104	Ribulose bisphosphate carboxylase small chain, chloroplastic	PF12338.5^RbcS^Ribulose-1,5-bisphosphate carboxylase small subunit^4-44^E:4.4e-23`PF00101.17^RuBisCO_small^Ribulose bisphosphate carboxylase, small chain^71-179^E:1.3e-39	0	1,039	0,001	0,716	0	-1,471	0	-1,312	0,006	-0,585	RBS_MALSP	Ribulose bisphosphate carboxylase small chain, chloroplastic (RuBisCO small subunit) (EC 4.1.1.39)	RBCS	Malus sp. (Crab apple)		Calvin cycle; Carbon dioxide fixation; Chloroplast; Lyase; Monooxygenase; Oxidoreductase; Photorespiration; Photosynthesis; Plastid; Transit peptide
SRR1271859_TR17834_c0_g1_i1_len=512	NA	NA	NA	NA	NA	.	0	1,376	0	8,288	0,066	5,219	1	2,21	0	-2,11	.	.	.	.	.	.
SRR1271859_TR29215_c0_g2_i1_len=1507	BT1_ARATH	Q:1292-354,H:14-335	66.77%ID	E:3e-139	BTB/POZ and TAZ domain-containing protein 1	PF00651.28^BTB^BTB/POZ domain^84-178^E:6.7e-12`PF02135.13^zf-TAZ^TAZ zinc finger^267-353^E:1.7e-12	0	1,235	0	2,734	0,822	0,292	0,094	1,128	0,51	-0,319	BT1_ARATH	BTB/POZ and TAZ domain-containing protein 1 (BTB and TAZ domain protein 1)	BT1 At5g63160 MDC12.13	Arabidopsis thaliana (Mouse-ear cress)	PATHWAY: Protein modification; protein ubiquitination.	Complete proteome; Cytoplasm; Metal-binding; Nucleus; Reference proteome; Ubl conjugation pathway; Zinc; Zinc-finger
SRR342017_TR8859_c1_g4_i2_len=1411	GEDH1_OCIBA	Q:1273-218,H:3-358	62.29%ID	E:5e-128	Geraniol dehydrogenase 1	PF08240.9^ADH_N^Alcohol dehydrogenase GroES-like domain^82-190^E:9.2e-22`PF00107.23^ADH_zinc_N^Zinc-binding dehydrogenase^234-357^E:1.8e-13	0	2,234	0	2,524	0,286	0,953	0,56	-0,686	0,094	1,196	GEDH1_OCIBA	Geraniol dehydrogenase 1 (ObaGEDH1) (EC 1.1.1.183)	GEDH1	Ocimum basilicum (Sweet basil)		Metal-binding; NADP; Oxidoreductase; Zinc
SRR342017_TR12325_c0_g3_i2_len=1007	NA	NA	NA	NA	NA	.	0,001	2,117	0	6,212	0,254	4,604	1	3,646	0,56	0,738	.	.	.	.	.	.
SRR342017_TR22430_c1_g1_i8_len=3110	PP226_ARATH	Q:2639-1662,H:54-379	44.48%ID	E:2e-84	Pentatricopeptide repeat-containing protein At3g13160, mitochondrial	PF01535.17^PPR^PPR repeat^160-185^E:0.00039`PF17177.1^PPR_long^Pentacotripeptide-repeat region of PROPR^167-276^E:1.1e-07`PF13812.3^PPR_3^Pentatricopeptide repeat domain^175-233^E:2.8e-09`PF01535.17^PPR^PPR repeat^191-217^E:0.09`PF12854.4^PPR_1^PPR repeat^221-252^E:1.8e-12`PF13041.3^PPR_2^PPR repeat family^223-270^E:4.9e-16`PF01535.17^PPR^PPR repeat^226-256^E:6e-07`PF13812.3^PPR_3^Pentatricopeptide repeat domain^247-302^E:1.8e-07`PF12854.4^PPR_1^PPR repeat^255-287^E:8.9e-06`PF13041.3^PPR_2^PPR repeat family^258-305^E:8.8e-08`PF01535.17^PPR^PPR repeat^261-290^E:0.0007`PF13041.3^PPR_2^PPR repeat family^294-340^E:9.6e-11`PF01535.17^PPR^PPR repeat^297-326^E:0.0036`PF13812.3^PPR_3^Pentatricopeptide repeat domain^317-371^E:4e-11`PF13041.3^PPR_2^PPR repeat family^329-373^E:3.9e-11`PF01535.17^PPR^PPR repeat^331-361^E:1.8e-06	0	7,679	0	3,02	1	-0,048	0,732	0,732	0,025	1,259	PP226_ARATH	Pentatricopeptide repeat-containing protein At3g13160, mitochondrial	At3g13160 MJG19.11	Arabidopsis thaliana (Mouse-ear cress)		Complete proteome; Mitochondrion; Reference proteome; Repeat; Transit peptide
SRR342017_TR22466_c1_g1_i1_len=760	NA	NA	NA	NA	NA	PF00564.21^PB1^PB1 domain^5-85^E:9.4e-11	0	6,981	0	6,713	0,57	0,351	0,281	0,576	0,005	6,235	.	.	.	.	.	.
SRR342019_TR6910_c0_g1_i1_len=1531	WTR41_ARATH	Q:1415-822,H:14-216	51.18%ID	E:8e-87	WAT1-related protein At5g40230	PF00892.17^EamA^EamA-like transporter family^30-156^E:5.3e-12	0	2,099	0	2,449	0,017	2,123	0,517	0,847	1	-0,358	WTR41_ARATH	WAT1-related protein At5g40230	At5g40230 MSN9.130	Arabidopsis thaliana (Mouse-ear cress)		Complete proteome; Membrane; Reference proteome; Repeat; Transmembrane; Transmembrane helix
SRR342019_TR7099_c4_g2_i9_len=1574	MTDH_FRAAN	Q:441-1292,H:70-353	63.38%ID	E:2e-96	Probable mannitol dehydrogenase	PF08240.9^ADH_N^Alcohol dehydrogenase GroES-like domain^4-82^E:1.2e-14`PF02826.16^2-Hacid_dh_C^D-isomer specific 2-hydroxyacid dehydrogenase, NAD binding domain^105-156^E:8e-07`PF00107.23^ADH_zinc_N^Zinc-binding dehydrogenase^126-249^E:1.8e-19	0	1,204	0	1,396	0,41	0,351	0,328	-0,442	0,183	0,653	MTDH_FRAAN	Probable mannitol dehydrogenase (EC 1.1.1.255) (NAD-dependent mannitol dehydrogenase)	CAD	Fragaria ananassa (Strawberry)		Metal-binding; NAD; Oxidoreductase; Zinc
SRR342022_TR3169_c4_g14_i1_len=1681	HST_TOBAC	Q:385-1515,H:57-430	26.02%ID	E:2e-30	Shikimate O-hydroxycinnamoyltransferase	PF02458.12^Transferase^Transferase family^7-445^E:2.7e-93	0	1,949	0	2,853	1	1,362	1	1,966	0,211	0,791	HST_TOBAC	Shikimate O-hydroxycinnamoyltransferase (EC 2.3.1.133) (Hydroxycinnamoyl transferase) (Hydroxycinnamoyl-Coenzyme A shikimate/quinate hydroxycinnamoyltransferase)	HST HCT	Nicotiana tabacum (Common tobacco)		Acyltransferase; Direct protein sequencing; Transferase
SRR342022_TR13627_c2_g2_i6_len=1731	FAR_SIMCH	Q:1029-97,H:181-492	48.09%ID	E:2e-94	Alcohol-forming fatty acyl-CoA reductase	PF07993.9^NAD_binding_4^Male sterility protein^35-148^E:1e-26`PF03015.16^Sterile^Male sterility protein^223-319^E:1.4e-12	0	1,968	0	5,551	0,014	3,01	0,025	2,902	1	2,136	FAR_SIMCH	Alcohol-forming fatty acyl-CoA reductase (EC 1.2.1.84)		Simmondsia chinensis (Jojoba) (Buxus chinensis)		Lipid biosynthesis; Lipid metabolism; NADP; Oxidoreductase
SRR342023_TR1546_c4_g3_i1_len=2664	DCAM_IPONI	Q:1332-244,H:1-361	72.45%ID	E:0	S-adenosylmethionine decarboxylase proenzyme	PF01536.13^SAM_decarbox^Adenosylmethionine decarboxylase^9-332^E:9.8e-108	0,001	1,4	0	2,45	0,237	0,326	0,155	0,384	1	-0,1	DCAM_IPONI	S-adenosylmethionine decarboxylase proenzyme (AdoMetDC) (SAMDC) (EC 4.1.1.50) [Cleaved into: S-adenosylmethionine decarboxylase alpha chain; S-adenosylmethionine decarboxylase beta chain]	SAMDC SDCG1	Ipomoea nil (Japanese morning glory) (Pharbitis nil)	PATHWAY: Amine and polyamine biosynthesis; S-adenosylmethioninamine biosynthesis; S-adenosylmethioninamine from S-adenosyl-L-methionine: step 1/1.	Autocatalytic cleavage; Decarboxylase; Lyase; Polyamine biosynthesis; Pyruvate; S-adenosyl-L-methionine; Schiff base; Spermidine biosynthesis; Zymogen
SRR342023_TR8498_c0_g1_i2_len=923	NA	NA	NA	NA	NA	.	0	1,636	0	1,335	0,009	0,554	0,004	0,603	1	0,109	.	.	.	.	.	.
SRR342023_TR18599_c2_g5_i8_len=3489	DGAT2_ARATH	Q:518-1456,H:2-314	67.2%ID	E:2e-149	Diacylglycerol O-acyltransferase 2	PF03547.15^Mem_trans^Membrane transport protein^11-405^E:7.7e-65	0	2,336	0,001	1,482	0,732	0,569	0,629	-0,988	0,357	0,689	DGAT2_ARATH	Diacylglycerol O-acyltransferase 2 (EC 2.3.1.20)	DGAT2 At3g51520 F26O13.160	Arabidopsis thaliana (Mouse-ear cress)	PATHWAY: Glycerolipid metabolism; triacylglycerol biosynthesis.	Acyltransferase; Complete proteome; Endoplasmic reticulum; Glycerol metabolism; Glycoprotein; Lipid biosynthesis; Lipid metabolism; Membrane; Reference proteome; Transferase; Transmembrane; Transmembrane helix
SRR342023_TR19146_c0_g4_i3_len=1968	WTR7_ARATH	Q:595-1623,H:2-340	53.64%ID	E:4e-103	WAT1-related protein At1g43650	PF00892.17^EamA^EamA-like transporter family^15-150^E:1.1e-16`PF00892.17^EamA^EamA-like transporter family^188-325^E:2.5e-14	0	2,258	0	1,894	0,294	0,525	1	-0,1	0,239	1,297	WTR7_ARATH	WAT1-related protein At1g43650	At1g43650 F2J6.1	Arabidopsis thaliana (Mouse-ear cress)		Alternative splicing; Complete proteome; Membrane; Reference proteome; Repeat; Transmembrane; Transmembrane helix
SRR646572_TR18061_c3_g2_i4_len=1750	WTR41_ARATH	Q:1632-730,H:14-319	51.91%ID	E:1e-99	WAT1-related protein At5g40230	PF00892.17^EamA^EamA-like transporter family^31-156^E:1.2e-11`PF00892.17^EamA^EamA-like transporter family^190-308^E:1.3e-06	0	1,642	0	2,771	0,043	2,576	0,629	1,315	1	0,994	WTR41_ARATH	WAT1-related protein At5g40230	At5g40230 MSN9.130	Arabidopsis thaliana (Mouse-ear cress)		Complete proteome; Membrane; Reference proteome; Repeat; Transmembrane; Transmembrane helix
SRR646572_TR22346_c0_g1_i3_len=7946	PSBB_TOBAC	Q:313-1833,H:1-507	99.61%ID	E:0	Photosystem II CP47 reaction center protein {ECO:0000255|HAMAP-Rule:MF_01495}	PF00421.16^PSII^Photosystem II protein^1-500^E:5.6e-264	0	1,912	0	1,778	0,028	-0,893	0,117	-0,604	1	-0,094	PSBB_TOBAC	Photosystem II CP47 reaction center protein (PSII 47 kDa protein) (Protein CP-47)	psbB	Nicotiana tabacum (Common tobacco)		Chlorophyll; Chloroplast; Chromophore; Membrane; Photosynthesis; Photosystem II; Plastid; Thylakoid; Transmembrane; Transmembrane helix
SRR646596_TR1473_c0_g1_i14_len=1038	ASA2_ORYSJ	Q:310-801,H:442-605	85.98%ID	E:1e-81	Anthranilate synthase alpha subunit 2, chloroplastic	PF00425.15^Chorismate_bind^chorismate binding enzyme^1-133^E:5.5e-37	0	2,657	0	4,437	0,002	2,617	0,118	1,663	0,593	-0,807	ASA2_ORYSJ	Anthranilate synthase alpha subunit 2, chloroplastic (OsASA2) (EC 4.1.3.27)	OsASA2 OASA2 Os03g0264400 LOC_Os03g15780	Oryza sativa subsp. japonica (Rice)	PATHWAY: Amino-acid biosynthesis; L-tryptophan biosynthesis; L-tryptophan from chorismate: step 1/5.	Amino-acid biosynthesis; Aromatic amino acid biosynthesis; Chloroplast; Complete proteome; Lyase; Plastid; Reference proteome; Transit peptide; Tryptophan biosynthesis
SRR646596_TR10462_c0_g1_i1_len=1056	NA	NA	NA	NA	NA	.	0	1,691	0	1,703	0,855	0,043	0,318	-0,231	0,226	0,416	.	.	.	.	.	.
SRR648705_TR5576_c0_g1_i3_len=4219	TRPX_ARATH	Q:2949-1891,H:255-607	80.45%ID	E:9e-175	Anthranilate synthase alpha subunit 2, chloroplastic	PF00425.15^Chorismate_bind^chorismate binding enzyme^42-313^E:6.8e-86	0	2,037	0	3,272	0,009	1,582	0,028	1,439	1	-1,048	TRPX_ARATH	Anthranilate synthase alpha subunit 2, chloroplastic (EC 4.1.3.27) (Anthranilate synthase component 1-2) (Anthranilate synthase component I-2)	ASA2 At2g29690 T27A16.21	Arabidopsis thaliana (Mouse-ear cress)	PATHWAY: Amino-acid biosynthesis; L-tryptophan biosynthesis; L-tryptophan from chorismate: step 1/5.	Amino-acid biosynthesis; Aromatic amino acid biosynthesis; Chloroplast; Complete proteome; Lyase; Plastid; Reference proteome; Transit peptide; Tryptophan biosynthesis
SRR648705_TR18740_c1_g1_i1_len=785	LSM5_ARATH	Q:658-401,H:1-85	93.02%ID	E:1e-47	Sm-like protein LSM5 {ECO:0000305}	.	0	6,618	0	7,238	0,823	0,106	1	-0,056	0,008	0,764	LSM5_ARATH	Sm-like protein LSM5 (AtLSM5) (Protein SUPERSENSITIVE TO ABA AND DROUGHT 1) (AtSAD1) (U6 snRNA-associated Sm-like protein LSM5)	LSM5 SAD1 At5g48870 K24G6.21	Arabidopsis thaliana (Mouse-ear cress)		Acetylation; Complete proteome; Cytoplasm; Nucleus; RNA-binding; Reference proteome; Ribonucleoprotein; Spliceosome; Stress response; mRNA processing; mRNA splicing
SRR648705_TR18759_c0_g2_i3_len=2884	TRPB_CAMAC	Q:1301-588,H:229-466	94.54%ID	E:2e-153	Tryptophan synthase beta chain 2, chloroplastic	PF00291.22^PALP^Pyridoxal-phosphate dependent enzyme^126-235^E:2.6e-21	0	1,216	0	2,275	0,636	0,151	0,34	-0,288	0,767	0,123	TRPB_CAMAC	Tryptophan synthase beta chain 2, chloroplastic (EC 4.2.1.20)	TSB	Camptotheca acuminata (Happy tree)	PATHWAY: Amino-acid biosynthesis; L-tryptophan biosynthesis; L-tryptophan from chorismate: step 5/5.	Amino-acid biosynthesis; Aromatic amino acid biosynthesis; Chloroplast; Lyase; Plastid; Pyridoxal phosphate; Transit peptide; Tryptophan biosynthesis
SRR648707_TR1596_c0_g2_i1_len=2070	NA	NA	NA	NA	NA	PF01925.16^TauE^Sulfite exporter TauE/SafE^74-191^E:7.8e-12`PF01925.16^TauE^Sulfite exporter TauE/SafE^249-434^E:8.9e-15	0	1,811	0	3,9	0,008	0,768	0,027	0,659	0,518	0,361	.	.	.	.	.	.
SRR648707_TR15318_c0_g4_i3_len=2226	TRPB_CAMAC	Q:2050-641,H:1-466	83.86%ID	E:0	Tryptophan synthase beta chain 2, chloroplastic	PF00291.22^PALP^Pyridoxal-phosphate dependent enzyme^126-455^E:4e-49	0	1,24	0	2,338	0,479	-0,222	0,067	-0,588	1	0	TRPB_CAMAC	Tryptophan synthase beta chain 2, chloroplastic (EC 4.2.1.20)	TSB	Camptotheca acuminata (Happy tree)	PATHWAY: Amino-acid biosynthesis; L-tryptophan biosynthesis; L-tryptophan from chorismate: step 5/5.	Amino-acid biosynthesis; Aromatic amino acid biosynthesis; Chloroplast; Lyase; Plastid; Pyridoxal phosphate; Transit peptide; Tryptophan biosynthesis
SRR648707_TR26086_c0_g1_i14_len=2158	TRPD_ARATH	Q:1089-1946,H:157-442	79.72%ID	E:3e-149	Anthranilate phosphoribosyltransferase, chloroplastic	PF00591.18^Glycos_transf_3^Glycosyl transferase family, a/b domain^13-261^E:7.8e-99	0	1,39	0	2,585	0,006	1,086	0,138	0,658	0,203	0,486	TRPD_ARATH	Anthranilate phosphoribosyltransferase, chloroplastic (EC 2.4.2.18)	PAT1 At5g17990 MCM23.6	Arabidopsis thaliana (Mouse-ear cress)	PATHWAY: Amino-acid biosynthesis; L-tryptophan biosynthesis; L-tryptophan from chorismate: step 2/5.	Amino-acid biosynthesis; Aromatic amino acid biosynthesis; Chloroplast; Complete proteome; Glycosyltransferase; Plastid; Reference proteome; Transferase; Transit peptide; Tryptophan biosynthesis
SRR648707_TR29014_c0_g1_i2_len=2273	BT1_ARATH	Q:229-1077,H:14-304	69.07%ID	E:2e-125	BTB/POZ and TAZ domain-containing protein 1	PF00651.28^BTB^BTB/POZ domain^89-183^E:5.1e-12`PF02135.13^zf-TAZ^TAZ zinc finger^272-358^E:1.4e-12	0	1,8	0,001	1,218	1	0,035	0,692	0,7	0,364	0,287	BT1_ARATH	BTB/POZ and TAZ domain-containing protein 1 (BTB and TAZ domain protein 1)	BT1 At5g63160 MDC12.13	Arabidopsis thaliana (Mouse-ear cress)	PATHWAY: Protein modification; protein ubiquitination.	Complete proteome; Cytoplasm; Metal-binding; Nucleus; Reference proteome; Ubl conjugation pathway; Zinc; Zinc-finger
SRR648707_TR33272_c1_g1_i13_len=1712	TRPX_ARATH	Q:250-1488,H:26-446	71.02%ID	E:0	Anthranilate synthase alpha subunit 2, chloroplastic	PF04715.10^Anth_synt_I_N^Anthranilate synthase component I, N terminal region^104-258^E:1.1e-30`PF00425.15^Chorismate_bind^chorismate binding enzyme^319-451^E:3.2e-37	0	1,791	0	6,814	0,068	1,207	0,524	0,561	0,692	0,828	TRPX_ARATH	Anthranilate synthase alpha subunit 2, chloroplastic (EC 4.1.3.27) (Anthranilate synthase component 1-2) (Anthranilate synthase component I-2)	ASA2 At2g29690 T27A16.21	Arabidopsis thaliana (Mouse-ear cress)	PATHWAY: Amino-acid biosynthesis; L-tryptophan biosynthesis; L-tryptophan from chorismate: step 1/5.	Amino-acid biosynthesis; Aromatic amino acid biosynthesis; Chloroplast; Complete proteome; Lyase; Plastid; Reference proteome; Transit peptide; Tryptophan biosynthesis
SRR924147_TR20336_c0_g1_i3_len=2526	MTHR2_ARATH	Q:2140-359,H:1-594	82.15%ID	E:0	Methylenetetrahydrofolate reductase 2	PF02219.14^MTHFR^Methylenetetrahydrofolate reductase^6-301^E:1.7e-126	0	0,932	0	4,094	0,403	0,249	0,377	0,257	0,732	0,778	MTHR2_ARATH	Methylenetetrahydrofolate reductase 2 (AtMTHFR2) (EC 1.5.1.20)	MTHFR2 At2g44160 F6E13.29	Arabidopsis thaliana (Mouse-ear cress)	PATHWAY: One-carbon metabolism; tetrahydrofolate interconversion.	Complete proteome; FAD; Flavoprotein; NAD; Oxidoreductase; Reference proteome
SRR924148_TR20412_c0_g1_i1_len=2557	AFC2_ARATH	Q:2417-1575,H:1-206	61.57%ID	E:7e-102	Serine/threonine-protein kinase AFC2	PF00069.22^Pkinase^Protein kinase domain^106-161^E:2.1e-07	0	2,767	0	2,754	0,016	-1,313	0	-2,853	1	0	AFC2_ARATH	Serine/threonine-protein kinase AFC2 (EC 2.7.12.1)	AFC2 At4g24740 F22K18.60	Arabidopsis thaliana (Mouse-ear cress)		ATP-binding; Alternative splicing; Complete proteome; Kinase; Nucleotide-binding; Reference proteome; Serine/threonine-protein kinase; Transferase
SRR924148_TR35240_c0_g1_i2_len=1597	BT1_ARATH	Q:1166-321,H:45-335	69.07%ID	E:3e-127	BTB/POZ and TAZ domain-containing protein 1	PF02135.13^zf-TAZ^TAZ zinc finger^115-201^E:7.4e-13	0	2,388	0	1,227	0,299	1,647	0,189	1,837	1	0,41	BT1_ARATH	BTB/POZ and TAZ domain-containing protein 1 (BTB and TAZ domain protein 1)	BT1 At5g63160 MDC12.13	Arabidopsis thaliana (Mouse-ear cress)	PATHWAY: Protein modification; protein ubiquitination.	Complete proteome; Cytoplasm; Metal-binding; Nucleus; Reference proteome; Ubl conjugation pathway; Zinc; Zinc-finger
SRR924148_TR37769_c0_g1_i4_len=2268	AGT23_ARATH	Q:228-1616,H:20-481	75.59%ID	E:0	Alanine--glyoxylate aminotransferase 2 homolog 3, mitochondrial	PF00202.18^Aminotran_3^Aminotransferase class-III^89-483^E:5.3e-98	0	3,335	0	6,415	0,009	6,043	0,034	5,611	0,042	1,836	AGT23_ARATH	Alanine--glyoxylate aminotransferase 2 homolog 3, mitochondrial (EC 2.6.1.44) (Beta-alanine-pyruvate aminotransferase 3)	At3g08860 T16O11.21	Arabidopsis thaliana (Mouse-ear cress)		Aminotransferase; Complete proteome; Mitochondrion; Photorespiration; Pyridoxal phosphate; Reference proteome; Transferase; Transit peptide
SRR924148_TR38387_c0_g1_i1_len=1972	METK2_CATRO	Q:268-1446,H:1-393	100%ID	E:0	S-adenosylmethionine synthase 2	PF00438.17^S-AdoMet_synt_N^S-adenosylmethionine synthetase, N-terminal domain^4-101^E:6.5e-43`PF02772.13^S-AdoMet_synt_M^S-adenosylmethionine synthetase, central domain^117-238^E:4.5e-46`PF02773.13^S-AdoMet_synt_C^S-adenosylmethionine synthetase, C-terminal domain^240-381^E:1.3e-62	0	0,814	0	3,263	0,759	-0,076	0,127	-0,373	0,27	0,243	METK2_CATRO	S-adenosylmethionine synthase 2 (AdoMet synthase 2) (EC 2.5.1.6) (Methionine adenosyltransferase 2) (MAT 2)	SAMS2	Catharanthus roseus (Madagascar periwinkle) (Vinca rosea)	PATHWAY: Amino-acid biosynthesis; S-adenosyl-L-methionine biosynthesis; S-adenosyl-L-methionine from L-methionine: step 1/1.	ATP-binding; Cobalt; Cytoplasm; Magnesium; Manganese; Metal-binding; Nucleotide-binding; One-carbon metabolism; Potassium; Transferase

Ms1wk

Transcript (CDF97)	Uniprot homolog	BLASTX Position	BLASTX %Identity	BLASTX evalue	Uniprot descr	Pfam	PValue_Ms6h	logFC_Ms6h	PValue_Ms8h	logFC_Ms8h	PValue_MsDamaged24h	logFC_MsDamaged24h	PValue_MsIntact24h	logFC_MsIntact24h	PValue_Ms1wk	logFC_Ms1wk	Entry.name	Protein names	Gene names	Organism	Pathway	Keywords
SRR1144633_TR659_c0_g1_i1_len=807	PLY22_ARATH	Q:616-2,H:50-253	83.9%ID	E:3e-125	Probable pectate lyase 22	.	1	0,024	1	0,143	0	-1,773	0,002	-0,911	0	7,306	PLY22_ARATH	Probable pectate lyase 22 (EC 4.2.2.2)	At5g63180 MDC12_15	Arabidopsis thaliana (Mouse-ear cress)	PATHWAY: Glycan metabolism; pectin degradation; 2-dehydro-3-deoxy-D-gluconate from pectin: step 2/5.	Calcium; Complete proteome; Glycoprotein; Lyase; Metal-binding; Reference proteome; Signal
SRR1144633_TR6803_c0_g2_i1_len=593	NLTP_VIGUN	Q:146-385,H:20-99	52.5%ID	E:1e-22	Probable non-specific lipid-transfer protein AKCS9	PF14368.3^LTP_2^Probable lipid transfer^48-126^E:3.3e-07	0,396	-0,257	0,581	-0,412	0,004	1,691	0,037	1,296	0	2,095	NLTP_VIGUN	Probable non-specific lipid-transfer protein AKCS9 (LTP)		Vigna unguiculata (Cowpea)		Disulfide bond; Lipid-binding; Signal; Transport
SRR1144633_TR8670_c0_g1_i1_len=1161	HIDM_GLYEC	Q:150-1070,H:16-327	45.57%ID	E:4e-91	2-hydroxyisoflavanone dehydratase	PF00135.25^COesterase^Carboxylesterase family^63-119^E:3.6e-06`PF07859.10^Abhydrolase_3^alpha/beta hydrolase fold^77-285^E:2.9e-34	0,732	0,886	1	0,866	0,254	4,424	1	0	0	2,279	HIDM_GLYEC	2-hydroxyisoflavanone dehydratase (EC 3.1.1.1) (EC 4.2.1.105) (Carboxylesterase HIDM)	HIDM	Glycyrrhiza echinata (Licorice)	PATHWAY: Secondary metabolite biosynthesis; flavonoid biosynthesis.	Flavonoid biosynthesis; Hydrolase; Lyase
SRR1144633_TR8670_c0_g1_i2_len=805	HIDM_GLYEC	Q:150-731,H:16-207	50.76%ID	E:1e-58	2-hydroxyisoflavanone dehydratase	PF00135.25^COesterase^Carboxylesterase family^63-119^E:2e-06`PF07859.10^Abhydrolase_3^alpha/beta hydrolase fold^77-217^E:1.7e-31	0,593	-0,591	0,226	3,041	1	0,145	1	-0,29	0	1,63	HIDM_GLYEC	2-hydroxyisoflavanone dehydratase (EC 3.1.1.1) (EC 4.2.1.105) (Carboxylesterase HIDM)	HIDM	Glycyrrhiza echinata (Licorice)	PATHWAY: Secondary metabolite biosynthesis; flavonoid biosynthesis.	Flavonoid biosynthesis; Hydrolase; Lyase
SRR1144633_TR8670_c0_g1_i7_len=1285	HIDM_GLYEC	Q:262-1194,H:16-327	46.08%ID	E:3e-94	2-hydroxyisoflavanone dehydratase	PF00135.25^COesterase^Carboxylesterase family^62-122^E:3e-05`PF07859.10^Abhydrolase_3^alpha/beta hydrolase fold^80-288^E:3.4e-37	0,517	1,273	0,692	-0,843	0,452	-0,535	0,094	-1,259	0	1,954	HIDM_GLYEC	2-hydroxyisoflavanone dehydratase (EC 3.1.1.1) (EC 4.2.1.105) (Carboxylesterase HIDM)	HIDM	Glycyrrhiza echinata (Licorice)	PATHWAY: Secondary metabolite biosynthesis; flavonoid biosynthesis.	Flavonoid biosynthesis; Hydrolase; Lyase
SRR1144633_TR8696_c0_g1_i1_len=917	NDUB8_ARATH	Q:735-361,H:1-125	82.4%ID	E:1e-50	NADH dehydrogenase [ubiquinone] 1 beta subcomplex subunit 8, mitochondrial	.	0,927	0,048	0,568	-0,187	0,121	0,473	0,352	0,286	0	5,776	NDUB8_ARATH	NADH dehydrogenase [ubiquinone] 1 beta subcomplex subunit 8, mitochondrial	At5g47570 MNJ7.16	Arabidopsis thaliana (Mouse-ear cress)		Complete proteome; Electron transport; Membrane; Mitochondrion; Mitochondrion inner membrane; Reference proteome; Respiratory chain; Transit peptide; Transmembrane; Transmembrane helix; Transport
SRR1144633_TR11410_c0_g1_i3_len=1613	GALE2_CYATE	Q:1361-339,H:6-346	83.87%ID	E:0	UDP-glucose 4-epimerase GEPI48	PF04321.14^RmlD_sub_bind^RmlD substrate binding domain^5-165^E:6.2e-11`PF01370.18^Epimerase^NAD dependent epimerase/dehydratase family^7-269^E:9.1e-53`PF02719.12^Polysacc_synt_2^Polysaccharide biosynthesis protein^7-189^E:1.3e-13`PF16363.2^GDP_Man_Dehyd^GDP-mannose 4,6 dehydratase^8-331^E:1.3e-61`PF01073.16^3Beta_HSD^3-beta hydroxysteroid dehydrogenase/isomerase family^8-163^E:6.3e-13	0,606	0,141	0,003	0,761	0,304	-0,251	0,001	-0,808	0	4,244	GALE2_CYATE	UDP-glucose 4-epimerase GEPI48 (EC 5.1.3.2) (Galactowaldenase) (UDP-galactose 4-epimerase)		Cyamopsis tetragonoloba (Guar) (Cluster bean)	PATHWAY: Carbohydrate metabolism; galactose metabolism.	Carbohydrate metabolism; Galactose metabolism; Isomerase; NAD
SRR1144633_TR22705_c0_g1_i1_len=1281	CB22_TOBAC	Q:181-975,H:1-265	93.96%ID	E:0	Chlorophyll a-b binding protein 21, chloroplastic	PF00504.18^Chloroa_b-bind^Chlorophyll A-B binding protein^65-231^E:1.6e-50	0	-0,975	0,048	0,654	0,004	-0,781	0,172	-0,364	0	1,654	CB22_TOBAC	Chlorophyll a-b binding protein 21, chloroplastic (LHCII type I CAB-21) (LHCP)	CAB21	Nicotiana tabacum (Common tobacco)		Acetylation; Chlorophyll; Chloroplast; Chromophore; Magnesium; Membrane; Metal-binding; Phosphoprotein; Photosynthesis; Photosystem I; Photosystem II; Plastid; Thylakoid; Transit peptide; Transmembrane; Transmembrane helix
SRR1144633_TR27825_c0_g4_i1_len=5279	POLX_TOBAC	Q:1725-5252,H:1-1161	40.27%ID	E:0	Retrovirus-related Pol polyprotein from transposon TNT 1-94	PF14223.3^Retrotran_gag_2^gag-polypeptide of LTR copia-type^59-187^E:1.8e-33`PF00098.20^zf-CCHC^Zinc knuckle^227-242^E:0.00069`PF13976.3^gag_pre-integrs^GAG-pre-integrase domain^397-464^E:2.9e-14`PF00665.23^rve^Integrase core domain^480-594^E:6.3e-26`PF07727.11^RVT_2^Reverse transcriptase (RNA-dependent DNA polymerase)^854-1101^E:1.2e-91	0,014	0,761	0,777	0,226	0,329	-0,403	0,241	-0,467	0	2,392	POLX_TOBAC	Retrovirus-related Pol polyprotein from transposon TNT 1-94 [Includes: Protease (EC 3.4.23.-); Reverse transcriptase (EC 2.7.7.49); Endonuclease]		Nicotiana tabacum (Common tobacco)		Aspartyl protease; Endonuclease; Hydrolase; Metal-binding; Nuclease; Nucleotidyltransferase; Protease; RNA-directed DNA polymerase; Transferase; Transposable element; Zinc; Zinc-finger
SRR1144634_TR1138_c1_g1_i1_len=1173	CBSX1_ARATH	Q:419-1012,H:39-236	75.76%ID	E:1e-98	CBS domain-containing protein CBSX1, chloroplastic	PF00571.25^CBS^CBS domain^79-133^E:9.7e-14`PF00571.25^CBS^CBS domain^173-226^E:1.9e-16	0,517	-0,187	0,321	-0,286	0,715	0,142	0,513	0,222	0,001	1,059	CBSX1_ARATH	CBS domain-containing protein CBSX1, chloroplastic (CBS domain-containing protein 2) (AtCDCP2) (Protein LOSS OF THE TIMING OF ET AND JA BIOSYNTHESIS 2) (AtLEJ2)	CBSX1 CDCP2 At4g36910 AP22.61 C7A10.450	Arabidopsis thaliana (Mouse-ear cress)		3D-structure; Acetylation; CBS domain; Chloroplast; Complete proteome; Plastid; Reference proteome; Repeat; Transit peptide
SRR1144634_TR21610_c1_g4_i1_len=593	NA	NA	NA	NA	NA	PF01439.15^Metallothio_2^Metallothionein^40-116^E:4.1e-29	1	-0,001	0	-1,209	0,748	0,082	0,017	-0,592	0	0,989	.	.	.	.	.	.
SRR1144634_TR29100_c0_g1_i1_len=2934	TC159_ARATH	Q:1057-50,H:639-983	63.58%ID	E:4e-103	Translocase of chloroplast 159, chloroplastic	PF04548.13^AIG1^AIG1 family^749-881^E:3.9e-21`PF01926.20^MMR_HSR1^50S ribosome-binding GTPase^750-838^E:1e-07	0,419	-0,375	0,356	1,505	0,56	-0,731	0,643	0,361	0,001	2,165	TC159_ARATH	Translocase of chloroplast 159, chloroplastic (AtToc159) (EC 3.6.5.-) (159 kDa chloroplast outer envelope protein) (Plastid protein import 2) (Translocase of chloroplast 160, chloroplastic) (AtToc160) (Translocase of chloroplast 86, chloroplastic) (AtToc86)	TOC159 PPI2 TOC160 TOC86 At4g02510 T10P11.19 T14P8.24	Arabidopsis thaliana (Mouse-ear cress)		Chloroplast; Coiled coil; Complete proteome; Cytoplasm; GTP-binding; Hydrolase; Magnesium; Membrane; Metal-binding; Nucleotide-binding; Phosphoprotein; Plastid; Plastid outer membrane; Protein transport; Receptor; Reference proteome; Transmembrane; Transmembrane helix; Transport
SRR1144634_TR32664_c0_g1_i1_len=1622	CDSP_ARATH	Q:186-1079,H:1-298	67.76%ID	E:8e-139	Thioredoxin-like protein CDSP32, chloroplastic	PF00085.17^Thioredoxin^Thioredoxin^79-165^E:1.4e-07`PF00085.17^Thioredoxin^Thioredoxin^204-289^E:1.4e-11	0,272	-0,278	0,375	-0,212	0,002	-0,798	0,015	-0,61	0	5,958	CDSP_ARATH	Thioredoxin-like protein CDSP32, chloroplastic (Chloroplastic drought-induced stress protein of 32 KDa) (AtCDSP32)	CDSP32 At1g76080 T23E18.2	Arabidopsis thaliana (Mouse-ear cress)		Chloroplast; Complete proteome; Disulfide bond; Electron transport; Plastid; Redox-active center; Reference proteome; Transit peptide; Transport
SRR122236_TR8892_c0_g1_i1_len=796	GAT22_ARATH	Q:397-750,H:117-230	39.55%ID	E:7e-21	Putative GATA transcription factor 22	PF00320.24^GATA^GATA zinc finger^122-155^E:1.4e-17	0,629	-1,042	1	-0,336	1	0,193	1	-0,399	0,001	2,633	GAT22_ARATH	Putative GATA transcription factor 22	GATA22 At4g26150 F20B18.260	Arabidopsis thaliana (Mouse-ear cress)		Complete proteome; DNA-binding; Metal-binding; Nucleus; Reference proteome; Transcription; Transcription regulation; Zinc; Zinc-finger
SRR122236_TR10709_c1_g1_i1_len=904	ARF2B_ARATH	Q:903-373,H:5-181	99.44%ID	E:2e-126	ADP-ribosylation factor 2-B	PF00025.18^Arf^ADP-ribosylation factor family^2-173^E:3.8e-80`PF09439.7^SRPRB^Signal recognition particle receptor beta subunit^12-139^E:7.9e-14`PF08477.10^Roc^Ras of Complex, Roc, domain of DAPkinase^15-125^E:9.9e-14`PF04670.9^Gtr1_RagA^Gtr1/RagA G protein conserved region^15-138^E:2.3e-10`PF01926.20^MMR_HSR1^50S ribosome-binding GTPase^15-118^E:4.4e-05`PF00071.19^Ras^Ras family^16-168^E:9.5e-13	1	0	1	0	1	-0,139	1	-0,301	0	5,784	ARF2B_ARATH	ADP-ribosylation factor 2-B (AtARF2) (ARF1-like protein U5)	ARF2-B ARFA1-D ARFA2-B U5 At1g70490 F20B24.21 F24J13.6	Arabidopsis thaliana (Mouse-ear cress)		Complete proteome; ER-Golgi transport; GTP-binding; Golgi apparatus; Lipoprotein; Myristate; Nucleotide-binding; Protein transport; Reference proteome; Transport
SRR122236_TR15235_c0_g1_i2_len=915	RL9_PEA	Q:105-671,H:1-189	87.83%ID	E:2e-116	60S ribosomal protein L9	PF00347.20^Ribosomal_L6^Ribosomal protein L6^12-89^E:8.8e-11`PF00347.20^Ribosomal_L6^Ribosomal protein L6^102-180^E:6.5e-12	0,629	-1,157	0,732	0,545	1	2,251	1	3,332	0	9,92	RL9_PEA	60S ribosomal protein L9 (Gibberellin-regulated protein GA)	RPL9	Pisum sativum (Garden pea)		Ribonucleoprotein; Ribosomal protein
SRR122237_TR3203_c0_g1_i1_len=936	NA	NA	NA	NA	NA	.	1	0	1	0	1	0	1	0	0	5,601	.	.	.	.	.	.

SRR122237_TR6355_c0_g1_i1_len=1105	EXPA8_ARATH	Q:159-848,H:25-253	84.42%ID	E:6e-130	Expansin-A8	PF03330.15^DPBB_1^Lytic transglycolase^93-180^E:1.7e-23`PF01357.18^Pollen_allerg_1^Pollen allergen^191-268^E:5.3e-30	0,464	0,329	0,689	-0,205	0,003	-0,777	0,924	-0,029	0	1,848	EXPA8_ARATH	Expansin-A8 (AtEXPA8) (Alpha-expansin-8) (At-EXP8) (AtEx8) (Ath-ExpAlpha-1.11)	EXPA8 EXP8 At2g40610 T2P4.4	Arabidopsis thaliana (Mouse-ear cress)		Cell wall; Cell wall biogenesis/degradation; Complete proteome; Disulfide bond; Membrane; Reference proteome; Secreted; Signal
SRR122237_TR27637_c0_g1_i1_len=1982	INO1_TOBAC	Q:148-1677,H:1-510	94.12%ID	E:0	Inositol-3-phosphate synthase	PF07994.9^NAD_binding_5^Myo-inositol-1-phosphate synthase^62-494^E:1.7e-142`PF01658.14^Inos-1-P_synth^Myo-inositol-1-phosphate synthase^310-423^E:2.6e-48	0,555	-0,301	0,56	-0,626	1	0	0,001	-0,721	0	1,311	INO1_TOBAC	Inositol-3-phosphate synthase (MIP synthase) (EC 5.5.1.4) (Myo-inositol 1-phosphate synthase) (IPS) (MI-1-P synthase)		Nicotiana tabacum (Common tobacco)	PATHWAY: Polyol metabolism; myo-inositol biosynthesis; myo-inositol from D-glucose 6-phosphate: step 1/2.	Cytoplasm; Inositol biosynthesis; Isomerase; Lipid biosynthesis; Lipid metabolism; NAD; Phospholipid biosynthesis; Phospholipid metabolism
SRR122238_TR9817_c1_g4_i1_len=850	EF4L4_ARATH	Q:534-190,H:1-114	72.17%ID	E:1e-47	Protein ELF4-LIKE 4	PF07011.8^DUF1313^Protein of unknown function (DUF1313)^15-96^E:2e-40	1	0	1	2,638	0,017	-5,92	0,56	-0,762	0	7,173	EF4L4_ARATH	Protein ELF4-LIKE 4	EFL4 ELF4-L4 At1g17455 F1L3.15 F28G4.6	Arabidopsis thaliana (Mouse-ear cress)		Biological rhythms; Complete proteome; Nucleus; Reference proteome
SRR122238_TR12605_c0_g1_i3_len=3130	GCR2_ARATH	Q:293-1516,H:1-410	67.07%ID	E:0	LanC-like protein GCR2	PF08659.7^KR^KR domain^119-211^E:8.8e-09`PF00106.22^adh_short^short chain dehydrogenase^121-263^E:7.3e-21`PF13561.3^adh_short_C2^Enoyl-(Acyl carrier protein) reductase^125-262^E:1.7e-12	0,001	-0,748	0,001	-1,022	0,18	-0,796	0,08	-1,113	0	1,802	GCR2_ARATH	LanC-like protein GCR2 (G-protein coupled receptor 2)	GCR2 GPCR At1g52920 F14G24.19	Arabidopsis thaliana (Mouse-ear cress)		3D-structure; Abscisic acid signaling pathway; Complete proteome; Metal-binding; Reference proteome; Zinc
SRR122238_TR16068_c0_g1_i1_len=1495	DPM1_HUMAN	Q:600-1142,H:81-260	69.06%ID	E:2e-79	Dolichol-phosphate mannosyltransferase subunit 1	PF00535.23^Glycos_transf_2^Glycosyl transferase family 2^17-124^E:1.7e-19`PF13641.3^Glyco_tranf_2_3^Glycosyltransferase like family 2^22-155^E:1.4e-06`PF13506.3^Glyco_transf_21^Glycosyl transferase family 21^29-124^E:3.7e-07	1	0,104	1	0,852	1	1,158	1	1,778	0,001	6,57	DPM1_HUMAN	Dolichol-phosphate mannosyltransferase subunit 1 (EC 2.4.1.83) (Dolichol-phosphate mannose synthase subunit 1) (DPM synthase subunit 1) (Dolichyl-phosphate beta-D-mannosyltransferase subunit 1) (Mannose-P-dolichol synthase subunit 1) (MPD synthase subunit 1)	DPM1	Homo sapiens (Human)	PATHWAY: Protein modification; protein glycosylation.	Acetylation; Complete proteome; Congenital disorder of glycosylation; Congenital muscular dystrophy; Disease mutation; Dystroglycanopathy; Endoplasmic reticulum; Glycosyltransferase; Phosphoprotein; Reference proteome; Transferase
SRR122238_TR24014_c0_g1_i2_len=2256	CATA2_GOSHI	Q:411-1886,H:1-492	89.84%ID	E:0	Catalase isozyme 2	PF00199.16^Catalase^Catalase^18-398^E:1.4e-174`PF06628.9^Catalase-rel^Catalase-related immune-responsive^423-486^E:3.2e-16	0,799	-0,113	0,684	0,183	0,062	-0,396	0,466	-0,154	0	1,895	CATA2_GOSHI	Catalase isozyme 2 (EC 1.11.1.6)	CAT2 SU2	Gossypium hirsutum (Upland cotton) (Gossypium mexicanum)		Heme; Hydrogen peroxide; Iron; Metal-binding; Oxidoreductase; Peroxidase; Peroxisome
SRR122238_TR24095_c0_g1_i2_len=514	PIMT_WHEAT	Q:193-474,H:1-94	65.96%ID	E:2e-36	Protein-L-isoaspartate O-methyltransferase	PF01135.16^PCMT^Protein-L-isoaspartate(D-aspartate) O-methyltransferase (PCMT)^76-157^E:2.7e-27	0,832	-0,3	0,034	5,585	0,472	0,694	0,034	-5,622	0,001	6,568	PIMT_WHEAT	Protein-L-isoaspartate O-methyltransferase (PIMT) (EC 2.1.1.77) (L-isoaspartyl protein carboxyl methyltransferase) (Protein L-isoaspartyl methyltransferase) (Protein-beta-aspartate methyltransferase)	PCM	Triticum aestivum (Wheat)		Complete proteome; Cytoplasm; Direct protein sequencing; Methyltransferase; Reference proteome; S-adenosyl-L-methionine; Transferase
SRR122238_TR28591_c0_g1_i2_len=771	ELOF1_ORYSJ	Q:650-387,H:1-89	92.13%ID	E:1e-41	Transcription elongation factor 1 homolog	.	0,643	0,138	0,395	0,308	0,017	0,636	0,038	-0,605	0	1,59	ELOF1_ORYSJ	Transcription elongation factor 1 homolog	Os07g0631100 LOC_Os07g43730 OsJ_25233 P0519E12.3	Oryza sativa subsp. japonica (Rice)		Complete proteome; Metal-binding; Nucleus; Reference proteome; Transcription; Transcription regulation; Zinc; Zinc-finger
SRR122238_TR32490_c0_g2_i2_len=1707	ANM11_ARATH	Q:195-1325,H:18-390	74.34%ID	E:0	Protein arginine N-methyltransferase 1.1	PF05185.13^PRMT5^PRMT5 arginine-N-methyltransferase^84-355^E:4.3e-07`PF06325.10^PrmA^Ribosomal protein L11 methyltransferase (PrmA)^107-181^E:1.6e-09`PF05175.11^MTS^Methyltransferase small domain^108-180^E:9.2e-05`PF13649.3^Methyltransf_25^Methyltransferase domain^111-185^E:1.5e-08`PF08241.9^Methyltransf_11^Methyltransferase domain^112-197^E:2.8e-07	1	-1,379	1	0	1	0	1	0	0	6,829	ANM11_ARATH	Protein arginine N-methyltransferase 1.1 (AtPRMT11) (EC 2.1.1.-) (EC 2.1.1.125) (Arginine methyltransferase pam1) (Histone-arginine N-methyltransferase PRMT11)	PRMT11 PAM1 PRMT1.1 PRMT1B At4g29510 T16L4.20	Arabidopsis thaliana (Mouse-ear cress)		Chromatin regulator; Complete proteome; Cytoplasm; Methyltransferase; Nucleus; Reference proteome; S-adenosyl-L-methionine; Transcription; Transcription regulation; Transferase
SRR122238_TR39694_c2_g3_i1_len=1361	GGPPS_HEVBR	Q:1181-240,H:52-369	65.41%ID	E:4e-142	Geranylgeranyl pyrophosphate synthase, chloroplastic	PF00348.14^polyprenyl_synt^Polyprenyl synthetase^42-278^E:4.5e-49	1	0,01	1	-0,311	0,245	-0,507	0,108	-0,689	0	2,576	GGPPS_HEVBR	Geranylgeranyl pyrophosphate synthase, chloroplastic (GGPP synthase) (GGPS) (EC 2.5.1.-) ((2E,6E)-farnesyl diphosphate synthase) (Dimethylallyltranstransferase) (EC 2.5.1.1) (Farnesyl diphosphate synthase) (Farnesyltranstransferase) (EC 2.5.1.29) (Geranyltranstransferase) (EC 2.5.1.10)	GGPS GG GGPPS1	Hevea brasiliensis (Para rubber tree) (Siphonia brasiliensis)	PATHWAY: Isoprenoid biosynthesis; farnesyl diphosphate biosynthesis; farnesyl diphosphate from geranyl diphosphate and isopentenyl diphosphate: step 1/1.; PATHWAY: Isoprenoid biosynthesis; geranyl diphosphate biosynthesis; geranyl diphosphate from dimethylallyl diphosphate and isopentenyl diphosphate: step 1/1.; PATHWAY: Isoprenoid biosynthesis; geranylgeranyl diphosphate biosynthesis; geranylgeranyl diphosphate from farnesyl diphosphate and isopentenyl diphosphate: step 1/1.	Carotenoid biosynthesis; Chloroplast; Isoprene biosynthesis; Magnesium; Metal-binding; Plastid; Transferase; Transit peptide
SRR122238_TR45834_c0_g1_i3_len=605	HEM62_ARATH	Q:424-2,H:19-149	58.87%ID	E:5e-39	Coproporphyrinogen-III oxidase 2, chloroplastic	PF01218.15^Coprogen_oxidas^Coproporphyrinogen III oxidase^101-165^E:7.7e-18	1	0,823	1	0,141	1	-0,222	1	-0,24	0	5,503	HEM62_ARATH	Coproporphyrinogen-III oxidase 2, chloroplastic (AtCPO-II) (Coprogen oxidase) (Coproporphyrinogenase) (EC 1.3.3.3)	CPX2 HEMF2 At4g03205 F4C21	Arabidopsis thaliana (Mouse-ear cress)	PATHWAY: Porphyrin-containing compound metabolism; protoporphyrin-IX biosynthesis; protoporphyrinogen-IX from coproporphyrinogen-III (O2 route): step 1/1.; PATHWAY: Porphyrin-containing compound metabolism; chlorophyll biosynthesis.	Alternative splicing; Chlorophyll biosynthesis; Chloroplast; Complete proteome; Heme biosynthesis; Oxidoreductase; Plastid; Porphyrin biosynthesis; Reference proteome; Transit peptide
SRR122238_TR48850_c1_g1_i1_len=560	NA	NA	NA	NA	NA	.	0,781	-0,573	0,832	0,242	0,44	0,762	0,228	1,079	0	2,512	.	.	.	.	.	.
SRR122238_TR50670_c0_g1_i4_len=505	PPA2_ARATH	Q:502-20,H:124-286	62.58%ID	E:4e-65	Probable inactive purple acid phosphatase 2	PF16656.2^Pur_ac_phosph_N^Purple acid Phosphatase, N-terminal domain^21-123^E:5e-22	0,91	0,09	0,019	1,478	0,154	0,767	0,154	0,759	0,001	2,015	PPA2_ARATH	Probable inactive purple acid phosphatase 2	PAP2 At1g13900 F16A14.11	Arabidopsis thaliana (Mouse-ear cress)		Complete proteome; Glycoprotein; Iron; Metal-binding; Reference proteome; Secreted; Signal; Zinc
SRR122238_TR50728_c1_g1_i1_len=866	NA	NA	NA	NA	NA	PF00111.24^Fer2^2Fe-2S iron-sulfur cluster binding domain^143-191^E:1.3e-09	0,22	-0,401	0	-1,735	0,002	-1,223	0,013	-0,93	0	2,399	.	.	.	.	.	.
SRR122238_TR50993_c0_g1_i2_len=809	CVIF1_ARATH	Q:167-601,H:26-162	37.24%ID	E:5e-23	Cell wall / vacuolar inhibitor of fructosidase 1	PF04043.12^PMEI^Plant invertase/pectin methylesterase inhibitor^55-199^E:2.4e-14	0,502	3,794	1	-1,588	0,502	3,73	1	1,242	0	2,812	CVIF1_ARATH	Cell wall / vacuolar inhibitor of fructosidase 1 (AtC/VIF1)	C/VIF1 CIF1 VIF1 At1g47960 T2J15.13	Arabidopsis thaliana (Mouse-ear cress)		Alternative splicing; Complete proteome; Disulfide bond; Glycoprotein; Reference proteome; Signal; Vacuole
SRR122238_TR57600_c0_g1_i2_len=849	BAM5_ARATH	Q:720-127,H:12-210	76.88%ID	E:4e-101	Beta-amylase 5	PF01373.14^Glyco_hydro_14^Glycosyl hydrolase family 14^13-208^E:3e-66	0,146	0,49	0,826	0,156	0,001	1,35	0,591	0,282	0	4,665	BAM5_ARATH	Beta-amylase 5 (AtBeta-Amy) (EC 3.2.1.2) (1,4-alpha-D-glucan maltohydrolase) (Protein REDUCED BETA AMYLASE 1)	BAM5 BMY1 RAM1 At4g15210 dl3650c FCAALL.97	Arabidopsis thaliana (Mouse-ear cress)		Alternative splicing; Carbohydrate metabolism; Complete proteome; Cytoplasm; Glycosidase; Hydrolase; Polysaccharide degradation; Reference proteome
SRR122238_TR58493_c0_g1_i3_len=1388	NA	NA	NA	NA	NA	.	1	0	1	0	1	-0,572	1	-0,572	0,001	6,599	.	.	.	.	.	.
SRR122238_TR58493_c0_g1_i6_len=1387	NA	NA	NA	NA	NA	.	1	-0,291	1	-0,425	1	0,397	1	1,597	0,001	4,918	.	.	.	.	.	.
SRR122238_TR59597_c1_g1_i2_len=1706	PMGI_MESCR	Q:275-1405,H:181-557	89.66%ID	E:0	2,3-bisphosphoglycerate-independent phosphoglycerate mutase	PF06415.10^iPGM_N^BPG-independent PGAM N-terminus (iPGM_N)^8-142^E:3.5e-32`PF01676.15^Metalloenzyme^Metalloenzyme superfamily^147-365^E:1.5e-71	0,643	-0,135	0,269	0,294	0,014	0,683	0,032	0,6	0	1,029	PMGI_MESCR	2,3-bisphosphoglycerate-independent phosphoglycerate mutase (BPG-independent PGAM) (Phosphoglyceromutase) (EC 5.4.2.12) (PGAM-I)	PGM1	Mesembryanthemum crystallinum (Common ice plant) (Cryophytum crystallinum)	PATHWAY: Carbohydrate degradation; glycolysis; pyruvate from D-glyceraldehyde 3-phosphate: step 3/5.	Cytoplasm; Glycolysis; Isomerase; Manganese; Metal-binding; Stress response
SRR122238_TR63925_c0_g1_i2_len=1406	CYT12_ORYSJ	Q:1097-483,H:39-244	65.22%ID	E:1e-83	Cysteine proteinase inhibitor 12	PF00031.18^Cystatin^Cystatin domain^48-127^E:5.9e-15`PF16845.2^SQAPI^Aspartic acid proteinase inhibitor^51-127^E:5.3e-21	0,691	0,237	0,018	1,12	0,065	0,716	0,106	0,638	0	1,687	CYT12_ORYSJ	Cysteine proteinase inhibitor 12 (Oryzacystatin XII) (OC-XII) (Oryzacystatin-12)	Os01g0270100 LOC_Os01g16430 P0667A10.20	Oryza sativa subsp. japonica (Rice)		Complete proteome; Plant defense; Protease inhibitor; Reference proteome; Repeat; Secreted; Signal; Thiol protease inhibitor
SRR122238_TR65744_c0_g1_i1_len=916	FK151_ARATH	Q:145-540,H:23-153	82.58%ID	E:6e-73	Peptidyl-prolyl cis-trans isomerase FKBP15-1	PF00254.25^FKBP_C^FKBP-type peptidyl-prolyl cis-trans isomerase^72-163^E:1.1e-35	0,001	-4,462	1	-0,187	0,017	5,795	1	2,619	0	6,884	FK151_ARATH	Peptidyl-prolyl cis-trans isomerase FKBP15-1 (PPIase FKBP15-1) (EC 5.2.1.8) (15 kDa FK506-binding protein) (15 kDa FKBP) (FK506-binding protein 15-1) (AtFKBP15-1) (FK506-binding protein 2-1) (Immunophilin FKBP15-1) (Rotamase)	FKBP15-1 At3g25220 MJL12.17	Arabidopsis thaliana (Mouse-ear cress)		Complete proteome; Endoplasmic reticulum; Isomerase; Reference proteome; Rotamase; Signal
SRR122238_TR65776_c0_g1_i1_len=664	NA	NA	NA	NA	NA	PF08041.8^PetM^PetM family of cytochrome b6f complex subunit 7^118-145^E:4.1e-11	0,165	-0,315	0,506	0,161	0	-0,923	0,06	-0,408	0	1,194	.	.	.	.	.	.
SRR122238_TR66842_c2_g3_i3_len=1227	BBX19_ARATH	Q:893-351,H:1-154	60.77%ID	E:2e-65	B-box zinc finger protein 19 {ECO:0000303|PubMed:19920209}	PF00643.21^zf-B_box^B-box zinc finger^3-42^E:1e-06`PF00643.21^zf-B_box^B-box zinc finger^54-90^E:3.3e-07	1	0	1	0	1	0	1	0	0	8,055	BBX19_ARATH	B-box zinc finger protein 19 (Protein DOUBLE B-BOX 1B) (Protein SALT TOLERANCE HOMOLOG 5)	BBX19 DBB1B STH5 At4g38960 F19H22.60	Arabidopsis thaliana (Mouse-ear cress)		Alternative splicing; Complete proteome; Metal-binding; Nucleus; Reference proteome; Repeat; Repressor; Transcription; Transcription regulation; Zinc; Zinc-finger
SRR122238_TR68876_c0_g1_i5_len=1248	NA	NA	NA	NA	NA	.	0,299	-1,509	0,226	3,115	1	0	1	0	0	6,548	.	.	.	.	.	.
SRR122238_TR69655_c0_g1_i3_len=765	NA	NA	NA	NA	NA	.	1	0	1	0	1	0	1	0	0	6,861	.	.	.	.	.	.
SRR122238_TR71732_c0_g1_i4_len=1194	GPX4_TOBAC	Q:721-215,H:1-169	84.62%ID	E:7e-102	Probable phospholipid hydroperoxide glutathione peroxidase	PF08534.7^Redoxin^Redoxin^84-237^E:2.1e-07`PF00255.16^GSHPx^Glutathione peroxidase^85-193^E:5.4e-43`PF00578.18^AhpC-TSA^AhpC/TSA family^85-148^E:1.7e-09	1	0	1	2,223	1	0	1	0	0	4,186	GPX4_TOBAC	Probable phospholipid hydroperoxide glutathione peroxidase (PHGPx) (EC 1.11.1.12) (Nt-SubC08)		Nicotiana tabacum (Common tobacco)		Cytoplasm; Oxidoreductase; Peroxidase
SRR122238_TR72015_c0_g1_i6_len=590	CAHC_TOBAC	Q:192-590,H:59-192	67.91%ID	E:2e-59	Carbonic anhydrase, chloroplastic	PF00484.16^Pro_CA^Carbonic anhydrase^152-190^E:5.4e-12	1	-1,349	1	-2,334	1	2,976	1	0	0	8,486	CAHC_TOBAC	Carbonic anhydrase, chloroplastic (EC 4.2.1.1) (Carbonate dehydratase)		Nicotiana tabacum (Common tobacco)		Chloroplast; Lyase; Plastid; Transit peptide; Zinc
SRR122238_TR75687_c0_g1_i4_len=1439	DIOX2_ARATH	Q:1286-297,H:1-332	68.86%ID	E:8e-171	Probable 2-oxoglutarate-dependent dioxygenase At3g49630	PF14226.3^DIOX_N^non-haem dioxygenase in morphine synthesis N-terminal^8-136^E:1.8e-34`PF03171.17^2OG-FeII_Oxy^2OG-Fe(II) oxygenase superfamily^202-289^E:2.2e-22	1	0,039	0,811	-0,336	1	0,137	0,798	0,475	0	4,118	DIOX2_ARATH	Probable 2-oxoglutarate-dependent dioxygenase At3g49630 (EC 1.14.11.-)	At3g50210 F11C1.50	Arabidopsis thaliana (Mouse-ear cress)		Alternative splicing; Complete proteome; Dioxygenase; Iron; Metal-binding; Oxidoreductase; Reference proteome
SRR122238_TR82375_c0_g2_i1_len=1297	XTH32_ARATH	Q:162-944,H:37-297	73.95%ID	E:6e-138	Probable xyloglucan endotransglucosylase/hydrolase protein 32	PF00722.18^Glyco_hydro_16^Glycosyl hydrolases family 16^61-243^E:4.6e-54`PF06955.9^XET_C^Xyloglucan endo-transglycosylase (XET) C-terminus^278-314^E:1.3e-13	0,214	0,866	1	-0,086	1	0,683	1	-1,538	0	1,706	XTH32_ARATH	Probable xyloglucan endotransglucosylase/hydrolase protein 32 (At-XTH32) (XTH-32) (EC 2.4.1.207)	XTH32 At2g36870 T1J8.5	Arabidopsis thaliana (Mouse-ear cress)		Alternative splicing; Apoplast; Cell wall; Cell wall biogenesis/degradation; Complete proteome; Disulfide bond; Glycosidase; Hydrolase; Reference proteome; Secreted; Signal; Transferase
SRR122238_TR87175_c2_g1_i1_len=4037	POLX_TOBAC	Q:4-3333,H:235-1323	44.1%ID	E:0	Retrovirus-related Pol polyprotein from transposon TNT 1-94	PF13976.3^gag_pre-integrs^GAG-pre-integrase domain^168-235^E:1.8e-14`PF00665.23^rve^Integrase core domain^251-365^E:5.9e-26`PF07727.11^RVT_2^Reverse transcriptase (RNA-dependent DNA polymerase)^625-872^E:1.6e-92	0,692	0,544	0,692	-1,208	1	-0,03	1	-0,903	0	3,561	POLX_TOBAC	Retrovirus-related Pol polyprotein from transposon TNT 1-94 [Includes: Protease (EC 3.4.23.-); Reverse transcriptase (EC 2.7.7.49); Endonuclease]		Nicotiana tabacum (Common tobacco)		Aspartyl protease; Endonuclease; Hydrolase; Metal-binding; Nuclease; Nucleotidyltransferase; Protease; RNA-directed DNA polymerase; Transferase; Transposable element; Zinc; Zinc-finger
SRR122238_TR88011_c0_g2_i2_len=598	PGKH_TOBAC	Q:2-316,H:368-472	97.14%ID	E:4e-52	Phosphoglycerate kinase, chloroplastic	PF00162.16^PGK^Phosphoglycerate kinase^1-97^E:2.2e-39	1	1,051	1	1,239	1	1,241	1	1,119	0	10,581	PGKH_TOBAC	Phosphoglycerate kinase, chloroplastic (EC 2.7.2.3)		Nicotiana tabacum (Common tobacco)	PATHWAY: Carbohydrate biosynthesis; Calvin cycle.	ATP-binding; Calvin cycle; Chloroplast; Kinase; Nucleotide-binding; Plastid; Transferase; Transit peptide
SRR122238_TR89375_c0_g1_i7_len=999	ACL5_ARATH	Q:717-115,H:29-229	79.6%ID	E:2e-113	Thermospermine synthase ACAULIS5	PF01564.14^Spermine_synth^Spermine/spermidine synthase^41-228^E:1e-47	0,484	0,426	0,832	-0,26	0,623	0,368	0,52	0,477	0	2,474	ACL5_ARATH	Thermospermine synthase ACAULIS5 (EC 2.5.1.79)	ACL5 TKV At5g19530 T20D1.50	Arabidopsis thaliana (Mouse-ear cress)		Alternative splicing; Complete proteome; Polyamine biosynthesis; Reference proteome; Transferase
SRR1271857_TR2657_c0_g1_i8_len=3347	SYY_MIMIV	Q:1972-3000,H:10-342	45.09%ID	E:2e-86	Tyrosine--tRNA ligase	PF00579.22^tRNA-synt_1b^tRNA synthetases class I (W and Y)^68-357^E:9.8e-67	1	0,741	1	-0,494	1	1,324	0,629	1,714	0	4,581	SYY_MIMIV	Tyrosine--tRNA ligase (EC 6.1.1.1) (Tyrosyl-tRNA synthetase) (TyrRS)	YARS MIMI_L124	Acanthamoeba polyphaga mimivirus (APMV)		3D-structure; ATP-binding; Aminoacyl-tRNA synthetase; Complete proteome; Ligase; Nucleotide-binding; Protein biosynthesis; Reference proteome
SRR1271857_TR4548_c0_g1_i1_len=879	DNJ11_ARATH	Q:332-622,H:65-161	67.35%ID	E:4e-38	Chaperone protein dnaJ 11, chloroplastic	PF00226.28^DnaJ^DnaJ domain^112-175^E:2.2e-21	0,692	1,244	0,006	2,534	1	-0,062	0,76	-0,376	0	2,37	DNJ11_ARATH	Chaperone protein dnaJ 11, chloroplastic (AtDjC11) (AtJ11)	ATJ11 C11 J11 At4g36040 T19K4.170	Arabidopsis thaliana (Mouse-ear cress)		Chaperone; Chloroplast; Complete proteome; Direct protein sequencing; Plastid; Reference proteome; Transit peptide
SRR1271857_TR4645_c1_g1_i1_len=1192	NA	NA	NA	NA	NA	PF13266.3^DUF4057^Protein of unknown function (DUF4057)^3-214^E:3.6e-97	1	0,205	0,692	-1,113	1	1,537	1	2,331	0	3,485	.	.	.	.	.	.
SRR1271857_TR7063_c1_g1_i2_len=3022	SR54C_ARATH	Q:1131-355,H:306-564	86.1%ID	E:5e-150	Signal recognition particle 54 kDa protein, chloroplastic	PF02881.16^SRP54_N^SRP54-type protein, helical bundle domain^91-168^E:5.1e-20`PF06414.9^Zeta_toxin^Zeta toxin^183-292^E:1.7e-06`PF00448.19^SRP54^SRP54-type protein, GTPase domain^186-317^E:6e-50`PF01583.17^APS_kinase^Adenylylsulphate kinase^186-227^E:2.5e-05`PF02374.12^ArsA_ATPase^Anion-transporting ATPase^188-223^E:2.7e-05`PF01656.20^CbiA^CobQ/CobB/MinD/ParA nucleotide binding domain^196-305^E:7.1e-06	1	-0,323	0,066	-3,133	1	-0,572	1	-1,46	0	5,91	SR54C_ARATH	Signal recognition particle 54 kDa protein, chloroplastic (54 chloroplast protein) (54CP) (SRP54) (cpSRP54) (FFC)	FFC CPSRP54 At5g03940 F8F6_150	Arabidopsis thaliana (Mouse-ear cress)		3D-structure; Chloroplast; Complete proteome; GTP-binding; Nucleotide-binding; Plastid; RNA-binding; Reference proteome; Ribonucleoprotein; Signal recognition particle; Transit peptide
SRR1271857_TR7700_c0_g1_i4_len=667	RUXG_ARATH	Q:124-363,H:1-80	90%ID	E:2e-44	Probable small nuclear ribonucleoprotein G	.	1	3,502	1	0	1	1,512	1	0	0	7,911	RUXG_ARATH	Probable small nuclear ribonucleoprotein G (snRNP-G) (Sm protein G) (Sm-G) (SmG)	At2g23930 T29E15.13	Arabidopsis thaliana (Mouse-ear cress)		Alternative splicing; Complete proteome; Nucleus; RNA-binding; Reference proteome; Ribonucleoprotein; Spliceosome; mRNA processing; mRNA splicing
SRR1271857_TR7766_c1_g1_i1_len=1023	HSP80_SOLLC	Q:975-61,H:1-305	94.43%ID	E:2e-172	Heat shock cognate protein 80	PF13589.3^HATPase_c_3^Histidine kinase-, DNA gyrase B-, and HSP90-like ATPase^25-146^E:5.3e-11`PF02518.23^HATPase_c^Histidine kinase-, DNA gyrase B-, and HSP90-like ATPase^27-181^E:1.2e-16`PF00183.15^HSP90^Hsp90 protein^184-305^E:1.8e-43	0,132	-1,872	0,129	3,041	1	-0,641	1	0,373	0	1,45	HSP80_SOLLC	Heat shock cognate protein 80	HSC80	Solanum lycopersicum (Tomato) (Lycopersicon esculentum)		ATP-binding; Chaperone; Complete proteome; Cytoplasm; Nucleotide-binding; Reference proteome; Stress response
SRR1271857_TR9930_c0_g1_i2_len=1657	ENDUB_DANRE	Q:1234-440,H:7-275	41.18%ID	E:3e-57	Poly(U)-specific endoribonuclease-B	PF09412.7^XendoU^Endoribonuclease XendoU^88-352^E:5.1e-98	0,043	0,655	0,021	0,73	0,626	0,274	0,474	0,363	0	3,717	ENDUB_DANRE	Poly(U)-specific endoribonuclease-B (EC 3.1.-.-) (Protein endoU-B) (Uridylate-specific endoribonuclease-B)	endoub zgc:110053	Danio rerio (Zebrafish) (Brachydanio rerio)		Complete proteome; Endonuclease; Hydrolase; Manganese; Metal-binding; Nuclease; RNA-binding; Reference proteome
SRR1271857_TR11664_c0_g1_i4_len=887	BI1L_ARATH	Q:117-791,H:24-251	70.18%ID	E:6e-100	BI1-like protein	PF01027.17^Bax1-I^Inhibitor of apoptosis-promoting Bax1^60-263^E:4.2e-46	0,502	4,108	0,226	2,296	0,502	4,042	1	0	0	8,17	BI1L_ARATH	BI1-like protein	At4g15470 dl3775w FCAALL.58	Arabidopsis thaliana (Mouse-ear cress)		Complete proteome; Membrane; Reference proteome; Transmembrane; Transmembrane helix
SRR1271857_TR11665_c0_g1_i1_len=593	NA	NA	NA	NA	NA	.	0,005	6,198	1	0,327	0,489	-0,526	0,656	0,303	0	3,502	.	.	.	.	.	.
SRR1271857_TR12243_c0_g1_i3_len=1710	FENR1_TOBAC	Q:158-1105,H:29-343	89.24%ID	E:0	Ferredoxin--NADP reductase, leaf-type isozyme, chloroplastic	PF00970.21^FAD_binding_6^Oxidoreductase FAD-binding domain^140-226^E:0.00017`PF00175.18^NAD_binding_1^Oxidoreductase NAD-binding domain^240-355^E:8.2e-30	1	0,324	0,065	-1,499	0,333	-0,669	0,882	-0,2	0	4,846	FENR1_TOBAC	Ferredoxin--NADP reductase, leaf-type isozyme, chloroplastic (FNR) (EC 1.18.1.2)	PETH	Nicotiana tabacum (Common tobacco)	PATHWAY: Energy metabolism; photosynthesis.	Chloroplast; Electron transport; FAD; Flavoprotein; Membrane; NADP; Oxidoreductase; Photosynthesis; Plastid; Thylakoid; Transit peptide; Transport
SRR1271857_TR15487_c0_g1_i2_len=1173	PPSP1_ARATH	Q:991-338,H:16-236	56.11%ID	E:4e-94	Inorganic pyrophosphatase 1	PF06888.9^Put_Phosphatase^Putative Phosphatase^7-235^E:1.2e-89`PF12710.4^HAD^haloacid dehalogenase-like hydrolase^7-183^E:5.5e-09	1	0,505	1	0	1	0	1	0	0,001	6,543	PPSP1_ARATH	Inorganic pyrophosphatase 1 (AtPPsPase1) (PPi phosphatase 1) (Pyrophosphate-specific phosphatase 1) (EC 3.6.1.1) (Protein PHOSPHATE STARVATION-INDUCED GENE 2) (AtPS2)	PS2 At1g73010 F3N23.21	Arabidopsis thaliana (Mouse-ear cress)		Complete proteome; Hydrolase; Magnesium; Metal-binding; Reference proteome
SRR1271857_TR17224_c4_g3_i1_len=2093	TBB8_ARATH	Q:177-863,H:1-229	95.2%ID	E:7e-141	Tubulin beta-8 chain	PF00091.22^Tubulin^Tubulin/FtsZ family, GTPase domain^3-211^E:4.8e-69	1	0,071	1	-0,2	1	-0,595	1	-0,926	0	2,727	TBB8_ARATH	Tubulin beta-8 chain (Beta-8-tubulin)	TUBB8 TUB8 At5g23860 MRO11.10	Arabidopsis thaliana (Mouse-ear cress)		Complete proteome; Cytoplasm; Cytoskeleton; GTP-binding; Microtubule; Nucleotide-binding; Reference proteome
SRR1271857_TR18874_c2_g3_i2_len=1184	PTH2_BOVIN	Q:761-369,H:41-174	43.28%ID	E:8e-29	Peptidyl-tRNA hydrolase 2, mitochondrial	PF01981.13^PTH2^Peptidyl-tRNA hydrolase PTH2^81-194^E:5e-45	0,692	-0,671	1	-0,385	1	0,358	1	0,272	0	3,207	PTH2_BOVIN	Peptidyl-tRNA hydrolase 2, mitochondrial (PTH 2) (EC 3.1.1.29) (Bcl-2 inhibitor of transcription)	PTRH2 BIT1	Bos taurus (Bovine)		Complete proteome; Hydrolase; Isopeptide bond; Mitochondrion; Reference proteome; Transit peptide; Ubl conjugation
SRR1271857_TR19383_c0_g1_i2_len=1452	ATCA1_ARATH	Q:926-216,H:29-265	46.84%ID	E:1e-81	Alpha carbonic anhydrase 1, chloroplastic	PF00194.18^Carb_anhydrase^Eukaryotic-type carbonic anhydrase^35-267^E:5.1e-45	1	-0,529	1	-0,085	1	-0,322	1	-0,411	0	5,436	ATCA1_ARATH	Alpha carbonic anhydrase 1, chloroplastic (AtaCA1) (AtalphaCA1) (EC 4.2.1.1) (Alpha carbonate dehydratase 1)	ACA1 CAH1 At3g52720 F3C22.120	Arabidopsis thaliana (Mouse-ear cress)		Alternative splicing; Chloroplast; Complete proteome; Disulfide bond; Endoplasmic reticulum; Glycoprotein; Lyase; Metal-binding; Plastid; Reference proteome; Signal; Zinc
SRR1271857_TR19998_c0_g2_i1_len=1840	C82G1_ARATH	Q:224-1732,H:15-512	48.73%ID	E:4e-154	Cytochrome P450 82G1	PF00067.19^p450^Cytochrome P450^42-501^E:2.8e-87	0	-4,653	0,35	0,458	0,231	0,579	0,214	-0,913	0	1,514	C82G1_ARATH	Cytochrome P450 82G1 (EC 1.14.-.-)	CYP82G1 At3g25180 MJL12.5	Arabidopsis thaliana (Mouse-ear cress)	PATHWAY: Secondary metabolite biosynthesis; terpenoid biosynthesis.	Alternative splicing; Complete proteome; Heme; Iron; Membrane; Metal-binding; Monooxygenase; Oxidoreductase; Plant defense; Reference proteome; Transmembrane; Transmembrane helix
SRR1271857_TR20126_c0_g2_i1_len=938	NA	NA	NA	NA	NA	.	1	0,904	1	0	1	0	1	2,235	0,001	4,878	.	.	.	.	.	.
SRR1271857_TR23859_c0_g1_i5_len=1868	TRL11_ARATH	Q:1542-766,H:1-248	57.85%ID	E:3e-77	Thioredoxin-like 1-1, chloroplastic	PF00085.17^Thioredoxin^Thioredoxin^119-186^E:9.3e-13	0,866	-0,108	0,761	0,127	0,183	0,407	0,648	0,151	0	1,72	TRL11_ARATH	Thioredoxin-like 1-1, chloroplastic (Atypical cysteine/histidine-rich thioredoxin 4) (AtACHT4) (Lilium-type thioredoxin 1-1)	At1g08570 F22O13.5	Arabidopsis thaliana (Mouse-ear cress)		Alternative splicing; Chloroplast; Complete proteome; Disulfide bond; Electron transport; Plastid; Redox-active center; Reference proteome; Transit peptide; Transport
SRR1271857_TR26079_c0_g1_i2_len=1866	FDFT_NICBE	Q:345-1574,H:1-410	81.71%ID	E:0	Squalene synthase	PF00494.16^SQS_PSY^Squalene/phytoene synthase^45-315^E:3.9e-45	0,269	0,36	0,423	-0,272	0,781	0,092	0,528	0,185	0	3,456	FDFT_NICBE	Squalene synthase (SQS) (SS) (EC 2.5.1.21) (FPP:FPP farnesyltransferase) (Farnesyl-diphosphate farnesyltransferase)		Nicotiana benthamiana	PATHWAY: Terpene metabolism; lanosterol biosynthesis; lanosterol from farnesyl diphosphate: step 1/3.	Endoplasmic reticulum; Isoprene biosynthesis; Magnesium; Membrane; Multifunctional enzyme; NADP; Oxidoreductase; Transferase; Transmembrane; Transmembrane helix
SRR1271857_TR26079_c0_g1_i4_len=1887	FDFT_NICBE	Q:366-1595,H:1-410	81.71%ID	E:0	Squalene synthase	PF00494.16^SQS_PSY^Squalene/phytoene synthase^45-315^E:3.9e-45	0,608	-0,24	0,004	2,384	0,138	0,531	0,064	0,642	0	7,215	FDFT_NICBE	Squalene synthase (SQS) (SS) (EC 2.5.1.21) (FPP:FPP farnesyltransferase) (Farnesyl-diphosphate farnesyltransferase)		Nicotiana benthamiana	PATHWAY: Terpene metabolism; lanosterol biosynthesis; lanosterol from farnesyl diphosphate: step 1/3.	Endoplasmic reticulum; Isoprene biosynthesis; Magnesium; Membrane; Multifunctional enzyme; NADP; Oxidoreductase; Transferase; Transmembrane; Transmembrane helix
SRR1271857_TR33180_c0_g1_i2_len=1085	RR10_MESCR	Q:478-837,H:78-197	87.5%ID	E:1e-71	30S ribosomal protein S10, chloroplastic	.	0,046	-0,478	0	-1,197	0,124	-0,391	0,467	-0,188	0	7,485	RR10_MESCR	30S ribosomal protein S10, chloroplastic	RPS10	Mesembryanthemum crystallinum (Common ice plant) (Cryophytum crystallinum)		Chloroplast; Plastid; Ribonucleoprotein; Ribosomal protein; Transit peptide
SRR1271857_TR33537_c0_g1_i1_len=1236	R10A3_ARATH	Q:894-244,H:1-217	91.24%ID	E:2e-127	60S ribosomal protein L10a-3	PF00687.18^Ribosomal_L1^Ribosomal protein L1p/L10e family^23-213^E:1.1e-44	1	-1,047	1	-1,791	1	0,797	1	0,284	0	9,276	R10A3_ARATH	60S ribosomal protein L10a-3	RPL10AC At5g22440 MWD9.24	Arabidopsis thaliana (Mouse-ear cress)		Complete proteome; Reference proteome; Ribonucleoprotein; Ribosomal protein
SRR1271857_TR38014_c0_g1_i13_len=2357	TCPB_ARATH	Q:1545-400,H:146-527	90.05%ID	E:0	T-complex protein 1 subunit beta {ECO:0000303|PubMed:11599560}	PF00118.21^Cpn60_TCP1^TCP-1/cpn60 chaperonin family^2-363^E:3.5e-102	1	-0,007	0,629	0,901	1	0,27	1	0,333	0	5,911	TCPB_ARATH	T-complex protein 1 subunit beta (TCP-1-beta) (CCT-beta) (Chaperonin CCT2)	CCT2 At5g20890 F22D1.60	Arabidopsis thaliana (Mouse-ear cress)		ATP-binding; Chaperone; Complete proteome; Cytoplasm; Nucleotide-binding; Reference proteome
SRR1271857_TR40721_c0_g1_i8_len=976	NA	NA	NA	NA	NA	PF02341.12^RcbX^RbcX protein^112-219^E:7.5e-35	0,256	0,438	0,011	-0,86	1	0	1	0	0	6,89	.	.	.	.	.	.
SRR1271857_TR43439_c0_g4_i3_len=2904	GLYM_SOLTU	Q:2634-1591,H:1-347	93.97%ID	E:0	Serine hydroxymethyltransferase, mitochondrial	PF00464.16^SHMT^Serine hydroxymethyltransferase^58-348^E:1.6e-158	0,875	0,052	0,007	0,771	0	-1,114	0,003	-0,718	0	2,713	GLYM_SOLTU	Serine hydroxymethyltransferase, mitochondrial (SHMT) (EC 2.1.2.1) (Glycine hydroxymethyltransferase) (Serine methylase)		Solanum tuberosum (Potato)	PATHWAY: One-carbon metabolism; tetrahydrofolate interconversion.	Complete proteome; Mitochondrion; One-carbon metabolism; Pyridoxal phosphate; Reference proteome; Transferase; Transit peptide
SRR1271857_TR44327_c0_g1_i1_len=1953	EF1A_TOBAC	Q:1829-522,H:1-436	97.94%ID	E:0	Elongation factor 1-alpha	PF00009.24^GTP_EFTU^Elongation factor Tu GTP binding domain^47-262^E:1.3e-52`PF03144.22^GTP_EFTU_D2^Elongation factor Tu domain 2^289-354^E:8.3e-15`PF03143.14^GTP_EFTU_D3^Elongation factor Tu C-terminal domain^363-470^E:4.5e-38	1	-0,218	0,195	1,51	0,381	1,53	0,381	1,517	0	5,289	EF1A_TOBAC	Elongation factor 1-alpha (EF-1-alpha) (Vitronectin-like adhesion protein 1) (PVN1)		Nicotiana tabacum (Common tobacco)		Cytoplasm; Elongation factor; GTP-binding; Nucleotide-binding; Phosphoprotein; Protein biosynthesis
SRR1271858_TR566_c1_g2_i4_len=1709	NA	NA	NA	NA	NA	PF09598.7^Stm1_N^Stm1^1-76^E:8.9e-23`PF04774.12^HABP4_PAI-RBP1^Hyaluronan / mRNA binding family^155-263^E:3.3e-30	1	0	1	0	1	0	1	0	0	9,639	.	.	.	.	.	.
SRR1271858_TR629_c0_g1_i1_len=2226	NA	NA	NA	NA	NA	PF03031.15^NIF^NLI interacting factor-like phosphatase^318-500^E:1.3e-18	1	-1,922	1	-1,39	1	2,145	1	1,395	0	5,324	.	.	.	.	.	.
SRR1271858_TR2709_c3_g4_i4_len=1972	EF1A_TOBAC	Q:140-1447,H:1-436	97.94%ID	E:0	Elongation factor 1-alpha	PF00009.24^GTP_EFTU^Elongation factor Tu GTP binding domain^52-267^E:1.3e-52`PF03144.22^GTP_EFTU_D2^Elongation factor Tu domain 2^294-359^E:8.4e-15`PF03143.14^GTP_EFTU_D3^Elongation factor Tu C-terminal domain^368-475^E:4.6e-38	0,356	-1,289	1	-0,06	1	-0,641	0,692	-0,816	0	2,236	EF1A_TOBAC	Elongation factor 1-alpha (EF-1-alpha) (Vitronectin-like adhesion protein 1) (PVN1)		Nicotiana tabacum (Common tobacco)		Cytoplasm; Elongation factor; GTP-binding; Nucleotide-binding; Phosphoprotein; Protein biosynthesis
SRR1271858_TR5529_c1_g3_i3_len=1444	NA	NA	NA	NA	NA	.	1	1,206	1	-0,045	1	0	1	1,092	0	7,514	.	.	.	.	.	.
SRR1271858_TR7418_c0_g1_i1_len=2273	CBF5_ARATH	Q:2014-644,H:16-460	82.71%ID	E:0	H/ACA ribonucleoprotein complex subunit 4	PF08068.9^DKCLD^DKCLD (NUC011) domain^50-107^E:5.6e-31`PF01509.15^TruB_N^TruB family pseudouridylate synthase (N terminal domain)^111-227^E:2.1e-19`PF16198.2^TruB_C_2^tRNA pseudouridylate synthase B C-terminal domain^228-294^E:2.8e-22`PF01472.17^PUA^PUA domain^298-370^E:1.8e-21	0,001	-1,109	0,235	0,406	0,235	0,837	1	-0,263	0	3,461	CBF5_ARATH	H/ACA ribonucleoprotein complex subunit 4 (EC 5.4.99.-) (CBF5 homolog) (Dyskerin) (Nopp-140-associated protein of 57 kDa homolog) (AtNAP57) (Nucleolar protein NAP57 homolog)	CBF5 NAP57 At3g57150 F24I3.230	Arabidopsis thaliana (Mouse-ear cress)		Complete proteome; Isomerase; Nucleus; Phosphoprotein; RNA-binding; Reference proteome; Ribonucleoprotein; Ribosome biogenesis; rRNA processing
SRR1271858_TR15681_c0_g1_i1_len=1365	NA	NA	NA	NA	NA	.	1	0,963	1	-1,183	1	-1,011	1	-2,599	0	5,41	.	.	.	.	.	.
SRR1271858_TR26973_c1_g5_i2_len=2752	CGS1_ARATH	Q:1230-2435,H:163-563	85.82%ID	E:0	Cystathionine gamma-synthase 1, chloroplastic {ECO:0000305}	PF01053.17^Cys_Met_Meta_PP^Cys/Met metabolism PLP-dependent enzyme^159-542^E:2.8e-140	1	1,751	1	0,849	0,502	1,959	1	1,406	0	2,21	CGS1_ARATH	Cystathionine gamma-synthase 1, chloroplastic (AtCGS1) (EC 2.5.1.48) (METHIONINE OVERACCUMULATION 1) (O-succinylhomoserine (thiol)-lyase)	CGS1 CYS1 MTO1 At3g01120 T4P13.19	Arabidopsis thaliana (Mouse-ear cress)	PATHWAY: Amino-acid biosynthesis; L-methionine biosynthesis via de novo pathway; L-cystathionine from O-succinyl-L-homoserine: step 1/1. {ECO:0000305}.	Amino-acid biosynthesis; Chloroplast; Complete proteome; Direct protein sequencing; Methionine biosynthesis; Plastid; Polymorphism; Pyridoxal phosphate; Reference proteome; Transferase; Transit peptide
SRR1271858_TR34334_c0_g3_i13_len=4289	VIP1_HUMAN	Q:3777-1453,H:68-808	47.18%ID	E:0	Inositol hexakisphosphate and diphosphoinositol-pentakisphosphate kinase 1	PF00328.19^His_Phos_2^Histidine phosphatase superfamily (branch 2)^334-795^E:7.6e-110	1	-0,125	1	-0,906	1	-0,082	1	0,109	0	5,66	VIP1_HUMAN	Inositol hexakisphosphate and diphosphoinositol-pentakisphosphate kinase 1 (EC 2.7.4.21) (EC 2.7.4.24) (Diphosphoinositol pentakisphosphate kinase 1) (Histidine acid phosphatase domain-containing protein 2A) (IP6 kinase) (Inositol pyrophosphate synthase 1) (InsP6 and PP-IP5 kinase 1) (VIP1 homolog) (hsVIP1)	PPIP5K1 HISPPD2A IP6K IPS1 KIAA0377 VIP1	Homo sapiens (Human)		ATP-binding; Alternative splicing; Cell membrane; Complete proteome; Cytoplasm; Kinase; Membrane; Nucleotide-binding; Phosphoprotein; Reference proteome; Transferase
SRR1271858_TR44225_c0_g2_i1_len=659	NLTP_VIGUN	Q:507-265,H:20-99	57.32%ID	E:4e-23	Probable non-specific lipid-transfer protein AKCS9	.	0,15	-0,528	0	-1,224	0,023	0,822	0	-8,349	0	2,598	NLTP_VIGUN	Probable non-specific lipid-transfer protein AKCS9 (LTP)		Vigna unguiculata (Cowpea)		Disulfide bond; Lipid-binding; Signal; Transport
SRR1271858_TR46206_c0_g1_i2_len=3711	ANXD2_ARATH	Q:1301-2176,H:24-316	73.04%ID	E:1e-148	Annexin D2	PF13266.3^DUF4057^Protein of unknown function (DUF4057)^38-220^E:1.9e-92	1	0,765	1	2,474	1	-0,209	1	3,181	0	7,145	ANXD2_ARATH	Annexin D2 (AnnAt2)	ANN2 ANNAT2 At5g65020 MXK3.27	Arabidopsis thaliana (Mouse-ear cress)		Acetylation; Alternative splicing; Annexin; Calcium; Calcium/phospholipid-binding; Complete proteome; Cytoplasm; Membrane; Reference proteome; Repeat
SRR1271858_TR47859_c0_g2_i2_len=1379	EXPA4_ARATH	Q:159-875,H:19-257	90.38%ID	E:4e-159	Expansin-A4	PF03330.15^DPBB_1^Lytic transglycolase^68-155^E:1.3e-21`PF01357.18^Pollen_allerg_1^Pollen allergen^166-243^E:1.6e-24	1	0,044	0,299	-1,58	1	-0,268	1	-1,142	0	1,666	EXPA4_ARATH	Expansin-A4 (AtEXPA4) (Alpha-expansin-4) (At-EXP4) (AtEx4) (Ath-ExpAlpha-1.6)	EXPA4 EXP4 At2g39700 F17A14.7	Arabidopsis thaliana (Mouse-ear cress)		Cell wall; Cell wall biogenesis/degradation; Complete proteome; Direct protein sequencing; Disulfide bond; Membrane; Reference proteome; Secreted; Signal
SRR1271858_TR48117_c0_g1_i2_len=2641	NA	NA	NA	NA	NA	PF01490.15^Aa_trans^Transmembrane amino acid transporter protein^45-262^E:1.2e-31`PF03222.10^Trp_Tyr_perm^Tryptophan/tyrosine permease family^46-269^E:2e-09	1	0,234	1	0	1	0	1	0	0	7,593	.	.	.	.	.	.
SRR1271858_TR48724_c0_g3_i3_len=853	RL4_PRUAR	Q:84-851,H:9-261	83.59%ID	E:1e-147	60S ribosomal protein L4	PF00573.19^Ribosomal_L4^Ribosomal protein L4/L1 family^30-260^E:1.7e-36	1	0	1	0,89	1	0	1	0	0,001	6,574	RL4_PRUAR	60S ribosomal protein L4 (L1)	RPL4	Prunus armeniaca (Apricot) (Armeniaca vulgaris)		Ribonucleoprotein; Ribosomal protein
SRR1271858_TR51167_c0_g2_i3_len=2239	NA	NA	NA	NA	NA	.	1	-1,24	0	-6,624	0,502	3,271	0,502	3,176	0	7,262	.	.	.	.	.	.
SRR1271858_TR53419_c1_g1_i11_len=966	HMGL_IPONI	Q:39-431,H:1-126	71.76%ID	E:1e-44	HMG1/2-like protein	PF09011.7^HMG_box_2^HMG-box domain^49-121^E:2.5e-15`PF00505.16^HMG_box^HMG (high mobility group) box^52-121^E:1.5e-22	0,799	-0,072	0,016	-0,595	0,005	1,118	0,221	0,534	0	1,578	HMGL_IPONI	HMG1/2-like protein		Ipomoea nil (Japanese morning glory) (Pharbitis nil)		DNA-binding; Nucleus
SRR1271858_TR54721_c0_g2_i6_len=1472	GPX1_HELAN	Q:669-1127,H:14-166	78.43%ID	E:3e-71	Glutathione peroxidase 1	PF00255.16^GSHPx^Glutathione peroxidase^2-104^E:1.4e-41`PF00578.18^AhpC-TSA^AhpC/TSA family^2-57^E:6.9e-07`PF08534.7^Redoxin^Redoxin^4-55^E:1.4e-06	0,689	0,202	0,069	1,059	0,016	1,496	0,003	1,763	0	3,75	GPX1_HELAN	Glutathione peroxidase 1 (EC 1.11.1.9)	GPXHA-1	Helianthus annuus (Common sunflower)		Oxidoreductase; Peroxidase
SRR1271858_TR58027_c0_g1_i7_len=2710	SYT1_ARATH	Q:1358-2386,H:197-541	73.04%ID	E:2e-175	Synaptotagmin-1	PF00168.27^C2^C2 domain^170-274^E:8.8e-23`PF00168.27^C2^C2 domain^329-431^E:1.7e-13	1	-0,552	1	0,478	1	0,58	1	-0,212	0	2,821	SYT1_ARATH	Synaptotagmin-1 (NTMC2T1.1) (Synaptotagmin A)	SYT1 SYTA At2g20990 F26H11.25 F5H14.5	Arabidopsis thaliana (Mouse-ear cress)		Alternative splicing; Calcium; Cell membrane; Complete proteome; Endocytosis; Endosome; Host-virus interaction; Lipid-binding; Membrane; Metal-binding; Reference proteome; Repeat; Transmembrane; Transmembrane helix
SRR1271858_TR58106_c0_g1_i1_len=1181	VDAC1_SOLTU	Q:974-450,H:1-175	82.86%ID	E:4e-102	Mitochondrial outer membrane protein porin of 34 kDa	PF01459.19^Porin_3^Eukaryotic porin^5-175^E:3.1e-41	1	0	1	0	1	0	1	0	0	9,402	VDAC1_SOLTU	Mitochondrial outer membrane protein porin of 34 kDa (POM 34) (Voltage-dependent anion-selective channel protein) (VDAC)		Solanum tuberosum (Potato)		Complete proteome; Direct protein sequencing; Ion transport; Membrane; Mitochondrion; Mitochondrion outer membrane; Porin; Reference proteome; Transmembrane; Transmembrane beta strand; Transport
SRR1271858_TR61665_c1_g1_i2_len=4402	PP329_ARATH	Q:383-2662,H:4-761	48.62%ID	E:0	Pentatricopeptide repeat-containing protein At4g20770	PF01535.17^PPR^PPR repeat^74-103^E:2.9e-05`PF01535.17^PPR^PPR repeat^105-131^E:0.00043`PF13041.3^PPR_2^PPR repeat family^203-251^E:3.4e-07`PF01535.17^PPR^PPR repeat^206-235^E:0.00058`PF01535.17^PPR^PPR repeat^293-313^E:0.095`PF13041.3^PPR_2^PPR repeat family^319-364^E:2.1e-07`PF01535.17^PPR^PPR repeat^321-351^E:0.0011`PF01535.17^PPR^PPR repeat^356-382^E:1.3`PF12854.4^PPR_1^PPR repeat^381-413^E:4.6e-07`PF13041.3^PPR_2^PPR repeat family^384-431^E:8.2e-11`PF01535.17^PPR^PPR repeat^388-413^E:7.6e-09`PF01535.17^PPR^PPR repeat^488-517^E:0.056`PF01535.17^PPR^PPR repeat^561-584^E:0.0068`PF13041.3^PPR_2^PPR repeat family^586-634^E:1.2e-11`PF01535.17^PPR^PPR repeat^589-618^E:7.1e-06`PF13812.3^PPR_3^Pentatricopeptide repeat domain^618-670^E:0.0017`PF01535.17^PPR^PPR repeat^625-652^E:0.23`PF01535.17^PPR^PPR repeat^661-683^E:1.3	1	0,052	1	-1,219	1	0,757	1	-0,406	0	4,977	PP329_ARATH	Pentatricopeptide repeat-containing protein At4g20770	PCMP-E35 At4g20770 F21C20.120	Arabidopsis thaliana (Mouse-ear cress)		Complete proteome; Reference proteome; Repeat
SRR1271858_TR64616_c0_g1_i1_len=821	PRS2_SOLTU	Q:327-773,H:1-152	37.18%ID	E:2e-22	Pathogenesis-related protein STH-2	PF00407.16^Bet_v_1^Pathogenesis-related protein Bet v I family^1-147^E:8.3e-14	1	0	1	0	1	1,279	1	1,249	0	2,399	PRS2_SOLTU	Pathogenesis-related protein STH-2	STH-2	Solanum tuberosum (Potato)		Complete proteome; Pathogenesis-related protein; Plant defense; Reference proteome
SRR1271858_TR64995_c0_g1_i1_len=658	NA	NA	NA	NA	NA	.	0,735	-0,22	0,895	-0,146	0,174	-0,479	0,095	-0,572	0	4,789	.	.	.	.	.	.
SRR1271859_TR1496_c0_g1_i4_len=1473	RZP23_ORYSJ	Q:253-579,H:1-103	79.82%ID	E:8e-45	Serine/arginine-rich splicing factor RSZ23	PF00076.19^RRM_1^RNA recognition motif. (a.k.a. RRM, RBD, or RNP domain)^4-61^E:2.5e-17`PF00098.20^zf-CCHC^Zinc knuckle^93-108^E:8.2e-08	0,623	-0,331	1	0	1	0	0,017	5,919	0	6,81	RZP23_ORYSJ	Serine/arginine-rich splicing factor RSZ23 (RS-containing zinc finger protein 23) (Os-RSZ23) (Os-RSZp23)	RSZ23 RSZP23 Os02g0610600 LOC_Os02g39720 OJ1476_F05.20	Oryza sativa subsp. japonica (Rice)		Complete proteome; Metal-binding; Nucleus; Reference proteome; Zinc; Zinc-finger; mRNA processing; mRNA splicing
SRR1271859_TR2244_c0_g4_i3_len=2636	SFH8_ARATH	Q:2320-464,H:1-620	76%ID	E:0	Phosphatidylinositol/phosphatidylcholine transfer protein SFH8	PF03765.12^CRAL_TRIO_N^CRAL/TRIO, N-terminal domain^108-131^E:1.3e-05`PF00650.17^CRAL_TRIO^CRAL/TRIO domain^155-321^E:8e-34	1	0,033	0,502	1,792	1	0,529	1	0,214	0	5,677	SFH8_ARATH	Phosphatidylinositol/phosphatidylcholine transfer protein SFH8 (Protein SEC FOURTEEN HOMOLOGS 8) (AtSFH8)	SFH8 At2g21520 F3K23.28	Arabidopsis thaliana (Mouse-ear cress)		Alternative splicing; Cell membrane; Coiled coil; Complete proteome; Golgi apparatus; Membrane; Protein transport; Reference proteome; Transport
SRR1271859_TR7000_c4_g1_i1_len=1423	UBC2_WHEAT	Q:646-1101,H:1-152	96.05%ID	E:6e-103	Ubiquitin-conjugating enzyme E2 2	PF00179.23^UQ_con^Ubiquitin-conjugating enzyme^8-144^E:1.9e-52	1	0,438	1	0	1	0,557	1	-1,397	0	8,183	UBC2_WHEAT	Ubiquitin-conjugating enzyme E2 2 (EC 2.3.2.23) (E2 ubiquitin-conjugating enzyme 2) (Ubiquitin carrier protein 2) (Ubiquitin-conjugating enzyme E2-16 kDa) (Ubiquitin-protein ligase 2)	UBC2 UBC1	Triticum aestivum (Wheat)	PATHWAY: Protein modification; protein ubiquitination. {ECO:0000255|PROSITE-ProRule:PRU00388}.	ATP-binding; Complete proteome; Nucleotide-binding; Reference proteome; Transferase; Ubl conjugation pathway
SRR1271859_TR7000_c4_g1_i11_len=1085	UBC2_WHEAT	Q:299-754,H:1-152	96.05%ID	E:2e-104	Ubiquitin-conjugating enzyme E2 2	PF00179.23^UQ_con^Ubiquitin-conjugating enzyme^66-202^E:5.4e-52	0,889	-0,166	0,267	0,717	0,129	0,842	0,1	0,919	0	2,154	UBC2_WHEAT	Ubiquitin-conjugating enzyme E2 2 (EC 2.3.2.23) (E2 ubiquitin-conjugating enzyme 2) (Ubiquitin carrier protein 2) (Ubiquitin-conjugating enzyme E2-16 kDa) (Ubiquitin-protein ligase 2)	UBC2 UBC1	Triticum aestivum (Wheat)	PATHWAY: Protein modification; protein ubiquitination. {ECO:0000255|PROSITE-ProRule:PRU00388}.	ATP-binding; Complete proteome; Nucleotide-binding; Reference proteome; Transferase; Ubl conjugation pathway
SRR1271859_TR8788_c0_g1_i2_len=2978	UGDH1_SOYBN	Q:1200-2639,H:1-480	92.29%ID	E:0	UDP-glucose 6-dehydrogenase 1	PF03721.11^UDPG_MGDP_dh_N^UDP-glucose/GDP-mannose dehydrogenase family, NAD binding domain^21-202^E:5.8e-66`PF00984.16^UDPG_MGDP_dh^UDP-glucose/GDP-mannose dehydrogenase family, central domain^228-322^E:7.3e-32`PF03720.12^UDPG_MGDP_dh_C^UDP-glucose/GDP-mannose dehydrogenase family, UDP binding domain^346-469^E:1.5e-37	1	0,613	1	1,912	0,629	-1,563	0,629	-1,422	0	3,196	UGDH1_SOYBN	UDP-glucose 6-dehydrogenase 1 (UDP-Glc dehydrogenase 1) (UDP-GlcDH 1) (UDPGDH 1) (EC 1.1.1.22) (Gm-UGD1)	UGD1	Glycine max (Soybean) (Glycine hispida)	PATHWAY: Nucleotide-sugar biosynthesis; UDP-alpha-D-glucuronate biosynthesis; UDP-alpha-D-glucuronate from UDP-alpha-D-glucose: step 1/1.	Complete proteome; NAD; Oxidoreductase; Phosphoprotein; Reference proteome
SRR1271859_TR10403_c0_g3_i6_len=1649	NA	NA	NA	NA	NA	.	1	-2,32	1	0,627	1	-0,559	1	-0,716	0	4,891	.	.	.	.	.	.
SRR1271859_TR10543_c2_g1_i18_len=968	CYSK_SPIOL	Q:246-968,H:1-241	85.48%ID	E:8e-140	Cysteine synthase	PF00291.22^PALP^Pyridoxal-phosphate dependent enzyme^13-239^E:6.7e-52	1	0,846	1	0,442	1	-0,158	1	-0,488	0	1,109	CYSK_SPIOL	Cysteine synthase (EC 2.5.1.47) (CSase A) (O-acetylserine (thiol)-lyase) (OAS-TL A) (O-acetylserine sulfhydrylase)		Spinacia oleracea (Spinach)	PATHWAY: Amino-acid biosynthesis; L-cysteine biosynthesis; L-cysteine from L-serine: step 2/2.	Amino-acid biosynthesis; Cysteine biosynthesis; Cytoplasm; Direct protein sequencing; Pyridoxal phosphate; Transferase
SRR1271859_TR13163_c0_g2_i3_len=2727	SPX4_ARATH	Q:1347-454,H:1-303	62.06%ID	E:1e-107	SPX domain-containing protein 4	PF03105.16^SPX^SPX domain^9-67^E:3.8e-11`PF03105.16^SPX^SPX domain^78-122^E:3.5e-07`PF03105.16^SPX^SPX domain^139-176^E:7.8e-09	1	0,885	1	-0,486	1	0	1	0	0	6,657	SPX4_ARATH	SPX domain-containing protein 4 (Protein SPX DOMAIN GENE 4) (AtSPX4)	SPX4 At5g15330 F8M21_220	Arabidopsis thaliana (Mouse-ear cress)		Complete proteome; Reference proteome
SRR1271859_TR13703_c0_g1_i4_len=2267	THO4A_ARATH	Q:258-992,H:4-244	55.65%ID	E:2e-57	THO complex subunit 4A	PF00076.19^RRM_1^RNA recognition motif. (a.k.a. RRM, RBD, or RNP domain)^156-224^E:1.3e-15`PF13865.3^FoP_duplication^C-terminal duplication domain of Friend of PRMT1^254-306^E:1.8e-08	1	0,575	1	2,635	1	1,06	1	1,122	0	5,586	THO4A_ARATH	THO complex subunit 4A (ALYREF homolog 1) (AtALY1)	ALY1 THO4A At5g59950 MMN10.26	Arabidopsis thaliana (Mouse-ear cress)		Acetylation; Alternative splicing; Complete proteome; Nucleus; RNA-binding; Reference proteome; Transport; mRNA transport
SRR1271859_TR13950_c0_g1_i3_len=2369	NA	NA	NA	NA	NA	PF09405.7^Btz^CASC3/Barentsz eIF4AIII binding^142-249^E:2.9e-20	0,517	-0,926	1	-0,184	1	0,232	1	0	0	4,942	.	.	.	.	.	.
SRR1271859_TR13962_c2_g2_i1_len=1717	TBB6_MAIZE	Q:144-1433,H:1-430	94.65%ID	E:0	Tubulin beta-6 chain	PF00091.22^Tubulin^Tubulin/FtsZ family, GTPase domain^3-211^E:3.6e-68`PF03953.14^Tubulin_C^Tubulin C-terminal domain^261-381^E:5.5e-41	1	-0,095	1	0,124	1	1,868	1	2,615	0	5,653	TBB6_MAIZE	Tubulin beta-6 chain (Beta-6-tubulin)	TUBB6 TUB6	Zea mays (Maize)		Complete proteome; Cytoplasm; Cytoskeleton; GTP-binding; Microtubule; Nucleotide-binding; Reference proteome
SRR1271859_TR17355_c0_g1_i1_len=1457	FLS_PETHY	Q:1329-307,H:11-348	76.02%ID	E:0	Flavonol synthase/flavanone 3-hydroxylase	PF14226.3^DIOX_N^non-haem dioxygenase in morphine synthesis N-terminal^43-154^E:1.4e-25`PF03171.17^2OG-FeII_Oxy^2OG-Fe(II) oxygenase superfamily^204-300^E:3.3e-27	1	0	1	0	1	0	1	0	0	8,494	FLS_PETHY	Flavonol synthase/flavanone 3-hydroxylase (FLS) (EC 1.14.11.23) (EC 1.14.11.9)	FL	Petunia hybrida (Petunia)	PATHWAY: Secondary metabolite biosynthesis; flavonoid biosynthesis.	Cytoplasm; Dioxygenase; Flavonoid biosynthesis; Iron; Metal-binding; Oxidoreductase; Vitamin C
SRR1271859_TR18461_c0_g1_i6_len=2642	AB7G_ARATH	Q:2248-443,H:65-666	82.23%ID	E:0	ABC transporter G family member 7	PF00005.24^ABC_tran^ABC transporter^87-239^E:2.8e-23`PF01061.21^ABC2_membrane^ABC-2 type transporter^388-599^E:1.2e-41	0,524	-0,223	0,21	-0,529	0,145	-0,658	0,145	-0,628	0,001	1,317	AB7G_ARATH	ABC transporter G family member 7 (ABC transporter ABCG.7) (AtABCG7) (White-brown complex homolog protein 7) (AtWBC7)	ABCG7 WBC7 At2g01320 F10A8.20	Arabidopsis thaliana (Mouse-ear cress)		ATP-binding; Alternative splicing; Complete proteome; Membrane; Nucleotide-binding; Reference proteome; Transmembrane; Transmembrane helix; Transport
SRR1271859_TR19103_c0_g1_i6_len=3375	GLGB_SOLTU	Q:372-2306,H:1-653	77.17%ID	E:0	1,4-alpha-glucan-branching enzyme	PF02922.15^CBM_48^Carbohydrate-binding module 48 (Isoamylase N-terminal domain)^134-217^E:1.6e-20`PF00128.21^Alpha-amylase^Alpha amylase, catalytic domain^298-396^E:6.9e-11	0,211	0,738	0,402	1,128	0,111	1,285	0,593	0,62	0	1,687	GLGB_SOLTU	1,4-alpha-glucan-branching enzyme (EC 2.4.1.18) (Q-enzyme) (Starch-branching enzyme)	SBE1 SBE	Solanum tuberosum (Potato)	PATHWAY: Glycan biosynthesis; starch biosynthesis.	Amyloplast; Chloroplast; Complete proteome; Glycosyltransferase; Plastid; Reference proteome; Starch biosynthesis; Transferase
SRR1271859_TR23830_c0_g1_i2_len=1945	NA	NA	NA	NA	NA	PF04788.9^DUF620^Protein of unknown function (DUF620)^122-378^E:4.2e-121	0,461	1,15	0,158	1,649	0,132	2,172	0,629	1,191	0	6,318	.	.	.	.	.	.
SRR1271859_TR25327_c0_g1_i1_len=622	H4_SOYBN	Q:200-445,H:22-103	100%ID	E:2e-49	Histone H4	.	0,319	0,965	0	-6,689	0,643	-0,429	0,472	-0,672	0	6,755	H4_SOYBN	Histone H4		Glycine max (Soybean) (Glycine hispida)		Acetylation; Chromosome; Complete proteome; DNA-binding; Methylation; Nucleosome core; Nucleus; Reference proteome
SRR1271859_TR26429_c0_g3_i1_len=1023	CRD1_ARATH	Q:427-897,H:1-158	72.96%ID	E:1e-62	Magnesium-protoporphyrin IX monomethyl ester [oxidative] cyclase, chloroplastic	.	0	-1,843	0,009	6,097	0	-5,267	0	-1,996	0	3,481	CRD1_ARATH	Magnesium-protoporphyrin IX monomethyl ester [oxidative] cyclase, chloroplastic (Mg-protoporphyrin IX monomethyl ester oxidative cyclase) (EC 1.14.13.81) (Copper response defect 1 protein) (Dicarboxylate diiron protein) (AtZIP) (MPE-cyclase)	CRD1 ACSF AT103 CHL27 ZIP At3g56940 F24I3.20	Arabidopsis thaliana (Mouse-ear cress)	PATHWAY: Porphyrin-containing compound metabolism; chlorophyll biosynthesis.	Alternative splicing; Chlorophyll biosynthesis; Chloroplast; Complete proteome; Iron; Membrane; Metal-binding; NADP; Oxidoreductase; Photosynthesis; Plastid; Plastid inner membrane; Reference proteome; Thylakoid; Transit peptide
SRR1271859_TR26434_c0_g1_i1_len=1695	PSMD4_ARATH	Q:143-1306,H:1-368	62.92%ID	E:2e-143	26S proteasome non-ATPase regulatory subunit 4 homolog	PF13519.3^VWA_2^von Willebrand factor type A domain^6-115^E:1.4e-27`PF02809.17^UIM^Ubiquitin interaction motif^224-237^E:0.54`PF02809.17^UIM^Ubiquitin interaction motif^290-305^E:0.069`PF02809.17^UIM^Ubiquitin interaction motif^324-338^E:0.55	1	0	1	1,518	1	0	1	0	0	5,768	PSMD4_ARATH	26S proteasome non-ATPase regulatory subunit 4 homolog (26S proteasome regulatory subunit RPN10) (AtRPN10) (26S proteasome regulatory subunit S5A homolog) (Multiubiquitin chain-binding protein 1) (AtMCB1)	RPN10 MBP1 MCB1 At4g38630 F20M13.190 T9A14.7	Arabidopsis thaliana (Mouse-ear cress)		Complete proteome; Phosphoprotein; Proteasome; Reference proteome; Repeat
SRR1271859_TR28211_c0_g1_i3_len=934	FER3_ARATH	Q:819-364,H:1-155	56.77%ID	E:6e-57	Ferredoxin-3, chloroplastic	PF00111.24^Fer2^2Fe-2S iron-sulfur cluster binding domain^65-138^E:2.6e-21	1	-1,869	1	1,875	1	1,543	1	0	0	8,315	FER3_ARATH	Ferredoxin-3, chloroplastic (AtFd3)	FD3 At2g27510 F10A12.19	Arabidopsis thaliana (Mouse-ear cress)		2Fe-2S; Acetylation; Chloroplast; Complete proteome; Electron transport; Iron; Iron-sulfur; Metal-binding; Plastid; Reference proteome; Transit peptide; Transport
SRR1271859_TR31741_c0_g1_i3_len=2813	CSC1L_BRAJU	Q:405-2549,H:1-713	65.69%ID	E:0	CSC1-like protein ERD4	PF13967.3^RSN1_TM^Late exocytosis, associated with Golgi transport^5-165^E:5.5e-31`PF14703.3^PHM7_cyt^Cytosolic domain of 10TM putative phosphate transporter^187-354^E:2.3e-33`PF02714.12^RSN1_7TM^Calcium-dependent channel, 7TM region, putative phosphate^365-636^E:8.8e-78	0,373	0,333	0,008	1,377	0,9	-0,093	0,564	0,315	0	2,004	CSC1L_BRAJU	CSC1-like protein ERD4 (Protein EARLY-RESPONSIVE TO DEHYDRATION STRESS 4)	ERD4	Brassica juncea (Indian mustard) (Sinapis juncea)		Calcium; Chloroplast; Ion channel; Ion transport; Membrane; Plastid; Transmembrane; Transmembrane helix; Transport
SRR1271859_TR32580_c0_g3_i19_len=774	SC61A_DICDI	Q:172-753,H:2-247	52.44%ID	E:1e-78	Protein transport protein Sec61 subunit alpha	PF10559.6^Plug_translocon^Plug domain of Sec61p^42-76^E:2.3e-18`PF00344.17^SecY^SecY translocase^77-173^E:5.2e-16	0,44	-1,039	0,129	2,635	1	0,9	1	1,413	0	1,307	SC61A_DICDI	Protein transport protein Sec61 subunit alpha (Secretory 61 complex subunit alpha)	sec61a DDB_G0278885	Dictyostelium discoideum (Slime mold)		Complete proteome; Endoplasmic reticulum; Membrane; Protein transport; Reference proteome; Translocation; Transmembrane; Transmembrane helix; Transport
SRR1271859_TR33166_c3_g2_i3_len=1375	TI201_ARATH	Q:970-158,H:1-274	71.17%ID	E:1e-114	Protein TIC 20-I, chloroplastic {ECO:0000303|PubMed:23372012}	PF16166.2^TIC20^Chloroplast import apparatus Tic20-like^91-265^E:9.1e-78	1	-0,685	1	-1,343	1	0,676	1	0,767	0	6,14	TI201_ARATH	Protein TIC 20-I, chloroplastic (Translocon at the inner envelope membrane of chloroplasts 20-I) (AtTIC20-I)	TIC20-I At1g04940 F13M7.5 F13M7.7	Arabidopsis thaliana (Mouse-ear cress)		Chloroplast; Complete proteome; Membrane; Plastid; Plastid inner membrane; Protein transport; Reference proteome; Transit peptide; Transmembrane; Transmembrane helix; Transport

SRR1271859_TR38007_c0_g1_i1_len=1290	RS92_ARATH	Q:1083-547,H:1-179	89.94%ID	E:2e-113	40S ribosomal protein S9-2	PF00163.16^Ribosomal_S4^Ribosomal protein S4/S9 N-terminal domain^8-64^E:2.6e-09`PF01479.22^S4^S4 domain^109-152^E:2.7e-11	1	-0,691	1	0,657	1	-0,629	1	-0,629	0	9,59	RS92_ARATH	40S ribosomal protein S9-2	RPS9C At5g39850 MYH19.1 MYH19.10	Arabidopsis thaliana (Mouse-ear cress)		Complete proteome; RNA-binding; Reference proteome; Ribonucleoprotein; Ribosomal protein; rRNA-binding
SRR1271859_TR38906_c1_g1_i6_len=793	RAN3_ARATH	Q:632-48,H:5-199	99.49%ID	E:1e-143	GTP-binding nuclear protein Ran-3	PF00025.18^Arf^ADP-ribosylation factor family^13-139^E:2.2e-11`PF00071.19^Ras^Ras family^15-171^E:3.2e-50`PF08477.10^Roc^Ras of Complex, Roc, domain of DAPkinase^15-128^E:1.3e-29`PF00910.19^RNA_helicase^RNA helicase^17-139^E:2.6e-05	0,705	-0,24	0,472	0,326	0,025	1,184	0,412	0,506	0	1,314	RAN3_ARATH	GTP-binding nuclear protein Ran-3 (Ras-related nuclear protein 3)	RAN3 At5g55190 MCO15.14	Arabidopsis thaliana (Mouse-ear cress)		Complete proteome; GTP-binding; Nucleotide-binding; Nucleus; Protein transport; Reference proteome; Transport
SRR1271859_TR41686_c4_g1_i1_len=775	NA	NA	NA	NA	NA	PF02496.13^ABA_WDS^ABA/WDS induced protein^109-185^E:1.1e-35	1	-0,621	1	-0,299	0,502	2,139	1	-1,697	0	1,549	.	.	.	.	.	.
SRR1271859_TR43983_c0_g1_i3_len=1847	G3PP1_ARATH	Q:207-1466,H:1-422	81.8%ID	E:0	Glyceraldehyde-3-phosphate dehydrogenase GAPCP1, chloroplastic	PF00044.21^Gp_dh_N^Glyceraldehyde 3-phosphate dehydrogenase, NAD binding domain^86-234^E:8.5e-57`PF02800.17^Gp_dh_C^Glyceraldehyde 3-phosphate dehydrogenase, C-terminal domain^239-396^E:3.2e-69	1	0	1	0	1	0	1	0	0	7,509	G3PP1_ARATH	Glyceraldehyde-3-phosphate dehydrogenase GAPCP1, chloroplastic (EC 1.2.1.12) (Glyceraldehyde-3-phosphate dehydrogenase of plastid 1) (NAD-dependent glyceraldehydephosphate dehydrogenase chloroplastic 1)	GAPCP1 At1g79530 T8K14.5	Arabidopsis thaliana (Mouse-ear cress)		Acetylation; Chloroplast; Complete proteome; Glycolysis; NAD; Oxidoreductase; Plastid; Reference proteome; Transit peptide
SRR1271859_TR44144_c1_g2_i2_len=895	SP1L1_ARATH	Q:699-379,H:1-110	64.86%ID	E:8e-29	Protein SPIRAL1-like 1	.	1	-0,444	1	0,07	1	0,821	1	1,484	0	6,738	SP1L1_ARATH	Protein SPIRAL1-like 1	SP1L1 At1g26355 T1K7.26	Arabidopsis thaliana (Mouse-ear cress)		Complete proteome; Microtubule; Phosphoprotein; Reference proteome
SRR1271859_TR45325_c0_g1_i8_len=2399	NA	NA	NA	NA	NA	PF13417.3^GST_N_3^Glutathione S-transferase, N-terminal domain^141-216^E:1.1e-13	1	-0,037	1	-1,752	1	-1,436	1	-1,056	0	6,882	.	.	.	.	.	.
SRR1271859_TR45849_c0_g1_i1_len=2695	IQD1_ARATH	Q:1430-2383,H:137-444	47.75%ID	E:6e-70	Protein IQ-DOMAIN 1	PF13178.3^DUF4005^Protein of unknown function (DUF4005)^228-295^E:0.00015	1	-0,432	1	0,567	1	0,064	1	0,349	0,001	2,63	IQD1_ARATH	Protein IQ-DOMAIN 1	IQD1 At3g09710 F11F8.30	Arabidopsis thaliana (Mouse-ear cress)		Alternative splicing; Calmodulin-binding; Complete proteome; Nucleus; Reference proteome
SRR1271859_TR47370_c1_g1_i3_len=619	NLTP_PRUAV	Q:203-532,H:9-117	42.73%ID	E:2e-23	Non-specific lipid-transfer protein	PF14368.3^LTP_2^Probable lipid transfer^22-109^E:3.6e-06`PF00234.19^Tryp_alpha_amyl^Protease inhibitor/seed storage/LTP family^34-118^E:1.2e-06	0,355	-0,26	0,326	0,317	0,208	0,409	0,008	-1,052	0	2,088	NLTP_PRUAV	Non-specific lipid-transfer protein (LTP) (allergen Pru av 3)		Prunus avium (Cherry) (Cerasus avium)		Allergen; Disulfide bond; Lipid-binding; Signal; Transport
SRR342017_TR1488_c1_g2_i1_len=1762	PP153_ARATH	Q:332-1471,H:206-579	52.11%ID	E:2e-138	Pentatricopeptide repeat-containing protein At2g15690	PF01535.17^PPR^PPR repeat^166-187^E:0.048`PF01535.17^PPR^PPR repeat^192-220^E:0.00032`PF01535.17^PPR^PPR repeat^227-253^E:1.3`PF14432.3^DYW_deaminase^DYW family of nucleic acid deaminases^348-462^E:5.3e-34	1	-1,312	1	-0,715	1	0	1	0	0	6,698	PP153_ARATH	Pentatricopeptide repeat-containing protein At2g15690	PCMP-H66 At2g15690 F9O13.24	Arabidopsis thaliana (Mouse-ear cress)		Complete proteome; Reference proteome; Repeat
SRR342017_TR4084_c1_g1_i3_len=702	GPX4_MESCR	Q:264-701,H:1-147	83.67%ID	E:3e-89	Probable phospholipid hydroperoxide glutathione peroxidase	PF08534.7^Redoxin^Redoxin^97-157^E:2.2e-06`PF00255.16^GSHPx^Glutathione peroxidase^98-206^E:4.8e-43`PF00578.18^AhpC-TSA^AhpC/TSA family^98-161^E:1.6e-09	1	1,398	1	0	1	0	1	0	0	7,994	GPX4_MESCR	Probable phospholipid hydroperoxide glutathione peroxidase (PHGPx) (EC 1.11.1.12)	GPXMC1	Mesembryanthemum crystallinum (Common ice plant) (Cryophytum crystallinum)		Cytoplasm; Oxidoreductase; Peroxidase
SRR342017_TR6085_c1_g1_i1_len=954	TI201_ARATH	Q:682-2,H:1-230	66.09%ID	E:5e-82	Protein TIC 20-I, chloroplastic {ECO:0000303|PubMed:23372012}	PF16166.2^TIC20^Chloroplast import apparatus Tic20-like^91-227^E:7.5e-57	1	0	1	0,82	1	-2,586	1	-2,586	0	4,054	TI201_ARATH	Protein TIC 20-I, chloroplastic (Translocon at the inner envelope membrane of chloroplasts 20-I) (AtTIC20-I)	TIC20-I At1g04940 F13M7.5 F13M7.7	Arabidopsis thaliana (Mouse-ear cress)		Chloroplast; Complete proteome; Membrane; Plastid; Plastid inner membrane; Protein transport; Reference proteome; Transit peptide; Transmembrane; Transmembrane helix; Transport
SRR342017_TR7190_c0_g2_i3_len=760	PS10B_ARATH	Q:199-759,H:3-190	92.02%ID	E:5e-118	26S protease regulatory subunit S10B homolog B	.	1	0	1	0	1	0	1	0	0	6,992	PS10B_ARATH	26S protease regulatory subunit S10B homolog B (26S proteasome AAA-ATPase subunit RPT4b) (26S proteasome subunit S10B homolog B) (Regulatory particle triple-A ATPase subunit 4b)	RPT4B At1g45000 F27F5.8	Arabidopsis thaliana (Mouse-ear cress)		ATP-binding; Alternative splicing; Complete proteome; Cytoplasm; Nucleotide-binding; Nucleus; Proteasome; Reference proteome
SRR342017_TR9404_c0_g5_i1_len=774	SL30A_ARATH	Q:242-742,H:34-194	67.66%ID	E:2e-51	Serine/arginine-rich SC35-like splicing factor SCL30A	PF00076.19^RRM_1^RNA recognition motif. (a.k.a. RRM, RBD, or RNP domain)^86-155^E:3.2e-20	0,5	-0,777	1	0	1	0	1	0	0	6,853	SL30A_ARATH	Serine/arginine-rich SC35-like splicing factor SCL30A (At-SCL30A) (AtSCL30A) (SC35-like splicing factor 30A) (Serine/arginine-rich splicing factor 30A)	SCL30A At3g13570 K20M4.1	Arabidopsis thaliana (Mouse-ear cress)		Alternative splicing; Complete proteome; Nucleus; Phosphoprotein; RNA-binding; Reference proteome; Spliceosome; mRNA processing; mRNA splicing
SRR342017_TR11303_c0_g1_i1_len=1129	NA	NA	NA	NA	NA	.	0,001	-0,896	0,681	-0,165	0,001	-0,983	0,026	-0,631	0,001	1,294	.	.	.	.	.	.
SRR342017_TR11530_c2_g1_i2_len=818	R10A1_ARATH	Q:1-534,H:39-216	95.51%ID	E:8e-107	60S ribosomal protein L10a-1	PF00687.18^Ribosomal_L1^Ribosomal protein L1p/L10e family^4-172^E:6.8e-40	1	0	1	0,879	1	2,126	1	0	0	8,118	R10A1_ARATH	60S ribosomal protein L10a-1	RPL10AA At1g08360 T27G7.6	Arabidopsis thaliana (Mouse-ear cress)		Complete proteome; Reference proteome; Ribonucleoprotein; Ribosomal protein
SRR342017_TR12657_c1_g1_i1_len=630	USF_AQUPY	Q:534-1,H:26-207	34.76%ID	E:3e-22	Protein usf	PF01738.15^DLH^Dienelactone hydrolase family^9-125^E:4e-35	0,77	-0,157	0,075	0,709	0,273	0,363	0,228	0,409	0	1,13	USF_AQUPY	Protein usf	usf	Aquifex pyrophilus		
SRR342017_TR13616_c0_g1_i6_len=2277	FTZ22_ARATH	Q:314-1744,H:1-472	74.28%ID	E:0	Cell division protein FtsZ homolog 2-2, chloroplastic	PF00091.22^Tubulin^Tubulin/FtsZ family, GTPase domain^119-280^E:1.5e-37`PF12327.5^FtsZ_C^FtsZ family, C-terminal domain^330-424^E:7.4e-31	0,213	-0,395	0,16	-0,505	0,718	0,22	0,484	0,365	0	1,057	FTZ22_ARATH	Cell division protein FtsZ homolog 2-2, chloroplastic (AtFtsZ2-2) (Plastid division protein FTSZ2-2)	FTSZ2-2 At3g52750 F3C22.150	Arabidopsis thaliana (Mouse-ear cress)		Chloroplast; Complete proteome; GTP-binding; Membrane; Nucleotide-binding; Plastid; Reference proteome; Thylakoid; Transit peptide
SRR342017_TR14852_c1_g2_i1_len=1517	NA	NA	NA	NA	NA	.	1	0,01	1	0,013	1	-0,06	0,798	-0,291	0	6,453	.	.	.	.	.	.
SRR342017_TR14963_c0_g2_i2_len=942	HMGL_IPONI	Q:70-462,H:1-126	71.76%ID	E:1e-44	HMG1/2-like protein	PF09011.7^HMG_box_2^HMG-box domain^37-109^E:2.1e-15`PF00505.16^HMG_box^HMG (high mobility group) box^40-109^E:1.2e-22	0,113	0,444	0,006	-0,756	0,655	0,258	0,443	-0,42	0	4,387	HMGL_IPONI	HMG1/2-like protein		Ipomoea nil (Japanese morning glory) (Pharbitis nil)		DNA-binding; Nucleus
SRR342017_TR15164_c0_g2_i2_len=2891	NA	NA	NA	NA	NA	PF12796.4^Ank_2^Ankyrin repeats (3 copies)^196-279^E:1.2e-11`PF13637.3^Ank_4^Ankyrin repeats (many copies)^225-277^E:2.4e-06`PF13857.3^Ank_5^Ankyrin repeats (many copies)^250-298^E:2e-06`PF13857.3^Ank_5^Ankyrin repeats (many copies)^333-380^E:2.5e-06`PF00023.27^Ank^Ankyrin repeat^338-371^E:0.028	1	-0,146	0,502	1,783	1	-0,767	1	-0,462	0	2,051	.	.	.	.	.	.
SRR342017_TR15347_c0_g2_i2_len=1246	NA	NA	NA	NA	NA	.	0,643	0,367	0,017	-5,879	1	-0,27	0,239	-1,231	0	2,884	.	.	.	.	.	.
SRR342017_TR16388_c0_g1_i2_len=847	PSAG_ARATH	Q:191-613,H:27-160	72.34%ID	E:7e-67	Photosystem I reaction center subunit V, chloroplastic	PF01241.15^PSI_PSAK^Photosystem I psaG / psaK^53-144^E:2.5e-26	0,288	0,251	0	-1,58	0,04	-0,453	0,232	-0,261	0	1,027	PSAG_ARATH	Photosystem I reaction center subunit V, chloroplastic (PSI-G)	PSAG At1g55670 F20N2.21 F20N2.33 F20N2_3	Arabidopsis thaliana (Mouse-ear cress)		Chloroplast; Complete proteome; Membrane; Photosynthesis; Photosystem I; Plastid; Reference proteome; Thylakoid; Transit peptide; Transmembrane; Transmembrane helix
SRR342017_TR18707_c0_g1_i1_len=1596	CB23_TOBAC	Q:1136-342,H:1-265	92.45%ID	E:2e-178	Chlorophyll a-b binding protein 36, chloroplastic	PF00504.18^Chloroa_b-bind^Chlorophyll A-B binding protein^66-231^E:5.2e-50	0,546	-0,126	0	-0,817	0	-1,207	0	-0,981	0	0,8	CB23_TOBAC	Chlorophyll a-b binding protein 36, chloroplastic (LHCII type I CAB-36) (LHCP)	CAB36	Nicotiana tabacum (Common tobacco)		Acetylation; Chlorophyll; Chloroplast; Chromophore; Magnesium; Membrane; Metal-binding; Phosphoprotein; Photosynthesis; Photosystem I; Photosystem II; Plastid; Thylakoid; Transit peptide; Transmembrane; Transmembrane helix
SRR342017_TR19152_c1_g5_i2_len=1177	NA	NA	NA	NA	NA	.	1	-0,296	1	-0,864	1	0	1	1,362	0	4,569	.	.	.	.	.	.
SRR342017_TR20180_c0_g2_i6_len=1187	PGL1A_ARATH	Q:122-1093,H:1-324	72%ID	E:1e-140	PGR5-like protein 1A, chloroplastic	.	1	1,663	1	0	1	0	1	2,428	0	1,157	PGL1A_ARATH	PGR5-like protein 1A, chloroplastic (Ferredoxin-plastoquinone reductase 1)	PGRL1A At4g22890 F7H19.70	Arabidopsis thaliana (Mouse-ear cress)		Acetylation; Alternative splicing; Chloroplast; Complete proteome; Disulfide bond; Electron transport; Membrane; Plastid; Reference proteome; Thylakoid; Transit peptide; Transmembrane; Transmembrane helix; Transport
SRR342017_TR20345_c0_g2_i3_len=837	NA	NA	NA	NA	NA	.	1	0	1	0	1	0	1	0	0	7,78	.	.	.	.	.	.
SRR342017_TR22495_c2_g5_i8_len=2745	FLS2_ARATH	Q:1395-295,H:25-467	31.07%ID	E:2e-33	LRR receptor-like serine/threonine-protein kinase FLS2	PF08263.9^LRRNT_2^Leucine rich repeat N-terminal domain^86-119^E:4.2e-05`PF00560.30^LRR_1^Leucine Rich Repeat^167-187^E:0.054`PF13855.3^LRR_8^Leucine rich repeat^214-272^E:1.1e-08`PF00560.30^LRR_1^Leucine Rich Repeat^215-236^E:0.081`PF13516.3^LRR_6^Leucine Rich repeat^237-252^E:1.9`PF13855.3^LRR_8^Leucine rich repeat^283-342^E:6.8e-09`PF13516.3^LRR_6^Leucine Rich repeat^307-320^E:1.9`PF00560.30^LRR_1^Leucine Rich Repeat^308-326^E:0.31	1	-0,009	1	-1,668	1	-0,508	1	-0,294	0	4,642	FLS2_ARATH	LRR receptor-like serine/threonine-protein kinase FLS2 (EC 2.7.11.1) (Protein FLAGELLIN-SENSING 2) (Protein FLAGELLIN-SENSITIVE 2)	FLS2 At5g46330 MPL12.13 MPL12.8	Arabidopsis thaliana (Mouse-ear cress)		3D-structure; ATP-binding; Cell membrane; Complete proteome; Disulfide bond; Endosome; Glycoprotein; Kinase; Leucine-rich repeat; Membrane; Nucleotide-binding; Phosphoprotein; Plant defense; Receptor; Reference proteome; Repeat; Serine/threonine-protein kinase; Signal; Transferase; Transmembrane; Transmembrane helix; Ubl conjugation; Ubl conjugation pathway
SRR342017_TR23398_c0_g5_i1_len=697	ALF_SPIOL	Q:152-697,H:1-182	88.46%ID	E:5e-111	Fructose-bisphosphate aldolase, cytoplasmic isozyme	PF00274.16^Glycolytic^Fructose-bisphosphate aldolase class-I^11-182^E:3.2e-86	1	0,372	1	1,507	1	0,128	1	0,416	0	9,386	ALF_SPIOL	Fructose-bisphosphate aldolase, cytoplasmic isozyme (EC 4.1.2.13)		Spinacia oleracea (Spinach)	PATHWAY: Carbohydrate degradation; glycolysis; D-glyceraldehyde 3-phosphate and glycerone phosphate from D-glucose: step 4/4.	Cytoplasm; Glycolysis; Lyase; Schiff base
SRR342017_TR23398_c0_g6_i1_len=861	NA	NA	NA	NA	NA	.	1	-0,207	1	0,999	1	-0,562	1	-0,652	0	6,576	.	.	.	.	.	.
SRR342017_TR23665_c0_g6_i6_len=753	NA	NA	NA	NA	NA	.	1	0	1	0	1	0,976	1	0	0	6,246	.	.	.	.	.	.
SRR342017_TR25036_c0_g1_i1_len=566	NA	NA	NA	NA	NA	.	1	0	1	0	1	0	1	0	0	8,665	.	.	.	.	.	.
SRR342017_TR25222_c0_g2_i1_len=1299	NA	NA	NA	NA	NA	PF04949.10^Transcrip_act^Transcriptional activator^23-170^E:1.6e-74	0,548	0,227	0	-1,474	0,635	0,354	0,87	0,258	0	1,031	.	.	.	.	.	.
SRR342017_TR25536_c0_g1_i1_len=1037	NDK1_TOBAC	Q:141-584,H:1-148	95.95%ID	E:2e-87	Nucleoside diphosphate kinase 1	PF00334.16^NDK^Nucleoside diphosphate kinase^2-133^E:9.8e-55	0,006	-0,696	0,006	0,656	0,003	0,831	0,409	0,245	0	10,643	NDK1_TOBAC	Nucleoside diphosphate kinase 1 (EC 2.7.4.6) (Nucleoside diphosphate kinase I) (NDK I) (NDP kinase I) (NDPK I)		Nicotiana tabacum (Common tobacco)		ATP-binding; Kinase; Magnesium; Metal-binding; Nucleotide metabolism; Nucleotide-binding; Phosphoprotein; Transferase
SRR342017_TR25892_c2_g4_i3_len=1329	POLX_TOBAC	Q:1215-4,H:232-632	40.44%ID	E:3e-94	Retrovirus-related Pol polyprotein from transposon TNT 1-94	PF00098.20^zf-CCHC^Zinc knuckle^38-53^E:0.00023`PF13976.3^gag_pre-integrs^GAG-pre-integrase domain^208-275^E:2e-15`PF00665.23^rve^Integrase core domain^291-405^E:9.7e-26	1	1,242	1	-1,171	1	-1,18	1	-1,61	0	8,399	POLX_TOBAC	Retrovirus-related Pol polyprotein from transposon TNT 1-94 [Includes: Protease (EC 3.4.23.-); Reverse transcriptase (EC 2.7.7.49); Endonuclease]		Nicotiana tabacum (Common tobacco)		Aspartyl protease; Endonuclease; Hydrolase; Metal-binding; Nuclease; Nucleotidyltransferase; Protease; RNA-directed DNA polymerase; Transferase; Transposable element; Zinc; Zinc-finger
SRR342017_TR30126_c0_g1_i1_len=1309	PSBO_SOLTU	Q:1127-882,H:1-81	81.71%ID	E:2e-31	Oxygen-evolving enhancer protein 1, chloroplastic	.	0,249	-0,262	0,422	-0,194	0	-0,922	0,012	-0,546	0	0,838	PSBO_SOLTU	Oxygen-evolving enhancer protein 1, chloroplastic (OEE1) (33 kDa subunit of oxygen evolving system of photosystem II) (33 kDa thylakoid membrane protein) (OEC 33 kDa subunit)	PSBO	Solanum tuberosum (Potato)		Chloroplast; Complete proteome; Manganese; Membrane; Photosynthesis; Photosystem II; Plastid; Reference proteome; Thylakoid; Transit peptide
SRR342017_TR33136_c0_g3_i2_len=3709	FTSHC_ARATH	Q:1181-3163,H:348-1008	86.84%ID	E:0	ATP-dependent zinc metalloprotease FTSH 12, chloroplastic	PF00004.26^AAA^ATPase family associated with various cellular activities (AAA)^140-276^E:6.2e-32`PF07724.11^AAA_2^AAA domain (Cdc48 subfamily)^140-255^E:0.00026`PF01434.15^Peptidase_M41^Peptidase family M41^365-570^E:2.4e-23	0,854	0,25	0,811	0,263	1	0,331	0,811	0,492	0	1,898	FTSHC_ARATH	ATP-dependent zinc metalloprotease FTSH 12, chloroplastic (AtFTSH12) (EC 3.4.24.-)	FTSH12 At1g79560 T8K14.2	Arabidopsis thaliana (Mouse-ear cress)		ATP-binding; Chloroplast; Complete proteome; Hydrolase; Membrane; Metal-binding; Metalloprotease; Nucleotide-binding; Plastid; Protease; Reference proteome; Thylakoid; Transit peptide; Transmembrane; Transmembrane helix; Zinc
SRR342017_TR33575_c0_g1_i2_len=653	CSN7_ARATH	Q:40-348,H:156-258	72.82%ID	E:2e-37	COP9 signalosome complex subunit 7	.	1	0	1	0	1	0	1	0	0	7,618	CSN7_ARATH	COP9 signalosome complex subunit 7 (CSN complex subunit 7) (Protein FUSCA 5)	CSN7 FUS5 At1g02090 T7I23.24 T7I23.25	Arabidopsis thaliana (Mouse-ear cress)		3D-structure; Alternative splicing; Complete proteome; Cytoplasm; Developmental protein; Nucleus; Phosphoprotein; Phytochrome signaling pathway; Reference proteome; Signalosome
SRR342019_TR4213_c8_g3_i4_len=1198	GGAP1_ARATH	Q:610-62,H:1-179	73.22%ID	E:3e-77	GDP-L-galactose phosphorylase 1	.	0,108	0,702	1	0	0	-1,706	1	0,012	0	4,183	GGAP1_ARATH	GDP-L-galactose phosphorylase 1 (EC 2.7.7.69) (Protein VITAMIN C DEFECTIVE 2)	VTC2 At4g26850 F10M23.190	Arabidopsis thaliana (Mouse-ear cress)	PATHWAY: Cofactor biosynthesis; L-ascorbate biosynthesis via GDP-alpha-D-mannose pathway; L-ascorbate from GDP-alpha-D-mannose: step 2/5.	Ascorbate biosynthesis; Complete proteome; Cytoplasm; Guanine-nucleotide releasing factor; Hydrolase; Nucleotide-binding; Nucleotidyltransferase; Nucleus; Reference proteome; Transferase
SRR342019_TR8850_c1_g4_i1_len=1085	RL9_PEA	Q:905-327,H:1-193	87.05%ID	E:1e-116	60S ribosomal protein L9	PF00347.20^Ribosomal_L6^Ribosomal protein L6^12-89^E:5.8e-12`PF00347.20^Ribosomal_L6^Ribosomal protein L6^101-180^E:5e-12	0,004	-0,765	0,556	-0,15	0,017	0,81	0,229	0,436	0	4,189	RL9_PEA	60S ribosomal protein L9 (Gibberellin-regulated protein GA)	RPL9	Pisum sativum (Garden pea)		Ribonucleoprotein; Ribosomal protein
SRR342019_TR11481_c2_g8_i1_len=1501	PRX2B_ARATH	Q:175-660,H:1-162	81.48%ID	E:2e-92	Peroxiredoxin-2B	PF08534.7^Redoxin^Redoxin^64-217^E:1.8e-34`PF00578.18^AhpC-TSA^AhpC/TSA family^64-179^E:3e-12	1	-0,434	0,461	-1,441	1	0,386	1	-0,091	0	1,261	PRX2B_ARATH	Peroxiredoxin-2B (EC 1.11.1.15) (Peroxiredoxin IIB) (Peroxiredoxin TPx1) (Thioredoxin reductase 2B) (Thioredoxin-dependent peroxidase 1)	PRXIIB TPX1 At1g65980 F12P19.14	Arabidopsis thaliana (Mouse-ear cress)		Alternative splicing; Antioxidant; Complete proteome; Cytoplasm; Disulfide bond; Oxidoreductase; Peroxidase; Redox-active center; Reference proteome
SRR342019_TR18824_c6_g1_i2_len=1026	NLTP_HELAN	Q:107-451,H:1-114	52.59%ID	E:2e-25	Non-specific lipid-transfer protein	PF14368.3^LTP_2^Probable lipid transfer^15-105^E:1.3e-05`PF00234.19^Tryp_alpha_amyl^Protease inhibitor/seed storage/LTP family^30-113^E:9.6e-08	0,266	0,548	0,003	1,349	0,004	1,217	0,077	0,801	0,001	1,137	NLTP_HELAN	Non-specific lipid-transfer protein (LTP) (NsLTP) (SDI-9)		Helianthus annuus (Common sunflower)		Disulfide bond; Lipid-binding; Signal; Transport
SRR342019_TR20493_c2_g5_i2_len=1742	HHT1_ARATH	Q:1621-308,H:28-454	33.41%ID	E:9e-59	Omega-hydroxypalmitate O-feruloyl transferase	PF02458.12^Transferase^Transferase family^17-453^E:2e-113	0,472	-0,709	1	0,014	0,545	-0,537	1	-0,088	0,001	0,999	HHT1_ARATH	Omega-hydroxypalmitate O-feruloyl transferase (EC 2.3.1.188) (Omega-hydroxyacid hydroxycinnamoyltransferase) (Protein ALIPHATIC SUBERIN FERULOYL TRANSFERASE)	HHT1 ASFT At5g41040 MEE6.11	Arabidopsis thaliana (Mouse-ear cress)		Acyltransferase; Alternative splicing; Cell wall biogenesis/degradation; Complete proteome; Reference proteome; Transferase
SRR342019_TR20560_c4_g2_i6_len=4978	POLX_TOBAC	Q:3498-1,H:1-1151	40.54%ID	E:0	Retrovirus-related Pol polyprotein from transposon TNT 1-94	PF14223.3^Retrotran_gag_2^gag-polypeptide of LTR copia-type^59-187^E:1.8e-33`PF00098.20^zf-CCHC^Zinc knuckle^227-242^E:0.00068`PF13976.3^gag_pre-integrs^GAG-pre-integrase domain^397-464^E:2.9e-14`PF00665.23^rve^Integrase core domain^480-594^E:6.2e-26`PF07727.11^RVT_2^Reverse transcriptase (RNA-dependent DNA polymerase)^854-1101^E:2.7e-92	1	0,056	1	0,983	1	0,622	1	0,13	0	8,275	POLX_TOBAC	Retrovirus-related Pol polyprotein from transposon TNT 1-94 [Includes: Protease (EC 3.4.23.-); Reverse transcriptase (EC 2.7.7.49); Endonuclease]		Nicotiana tabacum (Common tobacco)		Aspartyl protease; Endonuclease; Hydrolase; Metal-binding; Nuclease; Nucleotidyltransferase; Protease; RNA-directed DNA polymerase; Transferase; Transposable element; Zinc; Zinc-finger
SRR342019_TR22616_c0_g3_i2_len=1056	PRU1_PRUAR	Q:795-325,H:3-159	61.78%ID	E:5e-59	Major allergen Pru ar 1	PF00407.16^Bet_v_1^Pathogenesis-related protein Bet v I family^4-156^E:1e-19	0,177	-0,297	0,355	0,234	1	0,003	0,565	0,139	0	1,06	PRU1_PRUAR	Major allergen Pru ar 1 (allergen Pru ar 1)		Prunus armeniaca (Apricot) (Armeniaca vulgaris)		Allergen; Pathogenesis-related protein; Plant defense
SRR342019_TR29234_c2_g1_i6_len=720	C72A1_CATRO	Q:118-720,H:1-201	98.01%ID	E:2e-142	Secologanin synthase	PF00067.19^p450^Cytochrome P450^93-195^E:1.7e-10	0,133	-0,615	0,381	2,011	0,266	0,554	0,183	0,623	0,001	1,405	C72A1_CATRO	Secologanin synthase (SLS) (EC 1.3.3.9) (CYPLXXII) (Cytochrome P450 72A1)	CYP72A1 CYP72 P450CR3	Catharanthus roseus (Madagascar periwinkle) (Vinca rosea)	PATHWAY: Alkaloid biosynthesis; secologanin biosynthesis.	Alkaloid metabolism; Heme; Iron; Membrane; Metal-binding; Monooxygenase; NADP; Oxidoreductase; Transmembrane; Transmembrane helix; Vacuole
SRR342019_TR30344_c1_g9_i8_len=3925	IF2C_ARATH	Q:623-3496,H:37-1010	69.09%ID	E:0	Translation initiation factor IF-2, chloroplastic	PF00009.24^GTP_EFTU^Elongation factor Tu GTP binding domain^474-634^E:4e-33`PF01926.20^MMR_HSR1^50S ribosome-binding GTPase^475-583^E:5.9e-08`PF00071.19^Ras^Ras family^475-633^E:2.4e-06`PF11987.5^IF-2^Translation-initiation factor 2^779-870^E:3.5e-29	0,039	1,081	1	-0,186	0,056	-0,983	0,288	-0,504	0	1,356	IF2C_ARATH	Translation initiation factor IF-2, chloroplastic	At1g17220 F20D23.8	Arabidopsis thaliana (Mouse-ear cress)		Chloroplast; Complete proteome; GTP-binding; Initiation factor; Nucleotide-binding; Plastid; Protein biosynthesis; Reference proteome; Transit peptide
SRR342019_TR38591_c0_g2_i1_len=725	C3H49_ARATH	Q:105-725,H:3-176	55.56%ID	E:2e-72	Zinc finger CCCH domain-containing protein 49	PF00642.21^zf-CCCH^Zinc finger C-x8-C-x5-C-x3-H type (and similar)^157-177^E:1.1e-06`PF14608.3^zf-CCCH_2^RNA-binding, Nab2-type zinc finger^165-177^E:0.69`PF14608.3^zf-CCCH_2^RNA-binding, Nab2-type zinc finger^192-208^E:0.0068	1	-0,574	0,254	3,61	1	-0,636	1	-0,476	0	3,349	C3H49_ARATH	Zinc finger CCCH domain-containing protein 49 (AtC3H49)	At4g29190 F17A13.10	Arabidopsis thaliana (Mouse-ear cress)		Complete proteome; DNA-binding; Metal-binding; Reference proteome; Repeat; Zinc; Zinc-finger
SRR342019_TR38994_c3_g3_i2_len=1657	NA	NA	NA	NA	NA	PF05050.9^Methyltransf_21^Methyltransferase FkbM domain^65-217^E:2.4e-05	1	1,959	1	2,367	0,254	3,979	1	2,253	0	2,247	.	.	.	.	.	.
SRR342022_TR7506_c0_g1_i1_len=1191	RR6_ARATH	Q:199-756,H:17-206	73.16%ID	E:1e-73	30S ribosomal protein S6 alpha, chloroplastic	PF01250.14^Ribosomal_S6^Ribosomal protein S6^96-194^E:7.2e-19	0,031	-0,516	0	-1,195	0,346	-0,255	0,773	-0,076	0	6,374	RR6_ARATH	30S ribosomal protein S6 alpha, chloroplastic	RPS6 At1g64510 F1N19.8	Arabidopsis thaliana (Mouse-ear cress)		Chloroplast; Complete proteome; Plastid; RNA-binding; Reference proteome; Ribonucleoprotein; Ribosomal protein; Transit peptide; rRNA-binding
SRR342022_TR10375_c0_g1_i12_len=2737	OPT7_ARATH	Q:1437-175,H:346-764	72.21%ID	E:0	Oligopeptide transporter 7	PF03169.12^OPT^OPT oligopeptide transporter protein^12-387^E:6.1e-95	0,007	0,933	0,002	1,31	0,001	1,524	0,011	1,294	0	1,439	OPT7_ARATH	Oligopeptide transporter 7 (AtOPT7)	OPT7 At4g10770 T12H20.7	Arabidopsis thaliana (Mouse-ear cress)		Complete proteome; Membrane; Peptide transport; Protein transport; Reference proteome; Transmembrane; Transmembrane helix; Transport
SRR342022_TR12295_c0_g1_i1_len=2579	RR1_SPIOL	Q:1633-365,H:1-408	82.08%ID	E:0	30S ribosomal protein S1, chloroplastic	PF00575.20^S1^S1 RNA binding domain^109-181^E:8.1e-05`PF00575.20^S1^S1 RNA binding domain^197-261^E:1.8e-08`PF00575.20^S1^S1 RNA binding domain^274-344^E:3.9e-22	0,394	-0,501	0,014	-1,498	1	-0,026	0,748	0,241	0	6,097	RR1_SPIOL	30S ribosomal protein S1, chloroplastic (CS1)	RPS1	Spinacia oleracea (Spinach)		Chloroplast; Direct protein sequencing; Plastid; RNA-binding; Repeat; Ribonucleoprotein; Ribosomal protein; Transit peptide
SRR342022_TR14927_c0_g1_i4_len=2019	C7A52_PANGI	Q:1714-284,H:1-479	80.79%ID	E:0	Beta-amyrin 28-oxidase	PF00067.19^p450^Cytochrome P450^36-451^E:2.2e-59	0,566	0,153	0,043	0,617	0,01	-0,673	0,119	-0,397	0,001	1,153	C7A52_PANGI	Beta-amyrin 28-oxidase (EC 1.14.13.-) (Cytochrome P450 CYP716A52v2)		Panax ginseng (Korean ginseng)		Heme; Iron; Membrane; Metal-binding; Monooxygenase; NADP; Oxidoreductase; Transmembrane; Transmembrane helix
SRR342022_TR15284_c5_g5_i2_len=2308	21KD_DAUCA	Q:361-846,H:28-189	56.17%ID	E:3e-60	21 kDa protein	.	0,241	-0,389	0,111	0,615	0,121	-0,849	0,518	-0,324	0	1,315	21KD_DAUCA	21 kDa protein (1.2 protein)		Daucus carota (Wild carrot)		Signal
SRR342022_TR17938_c0_g1_i1_len=1248	PSD8A_ARATH	Q:1142-342,H:1-267	80.9%ID	E:2e-149	26S proteasome non-ATPase regulatory subunit 8 homolog A	PF03399.13^SAC3_GANP^SAC3/GANP family^81-221^E:2.7e-06`PF10075.6^CSN8_PSD8_EIF3K^CSN8/PSMD8/EIF3K family^106-241^E:8.1e-28	0,506	-0,21	0,24	0,374	0,343	0,356	0,597	0,213	0	6,303	PSD8A_ARATH	26S proteasome non-ATPase regulatory subunit 8 homolog A (26S proteasome regulatory subunit RPN12a) (AtRPN12a) (26S proteasome regulatory subunit S14 homolog A)	RPN12A At1g64520 F1N19.9	Arabidopsis thaliana (Mouse-ear cress)		Acetylation; Complete proteome; Cytokinin signaling pathway; Proteasome; Reference proteome; Ubl conjugation
SRR342022_TR18074_c0_g3_i3_len=3143	AB26B_ARATH	Q:428-1597,H:99-485	69.23%ID	E:3e-163	ABC transporter B family member 26, chloroplastic	PF00664.20^ABC_membrane^ABC transporter transmembrane region^137-403^E:1e-38	0,547	-0,354	0,822	0,339	0,357	-0,725	0,471	-0,534	0	3,267	AB26B_ARATH	ABC transporter B family member 26, chloroplastic (ABC transporter ABCB.26) (AtABCB26) (Antigen peptide transporter-like 1) (Transporter associated with antigen processing-like protein 1) (AtTAP1)	ABCB26 TAP1 At1g70610 F24J13.18 F5A18.21	Arabidopsis thaliana (Mouse-ear cress)		ATP-binding; Chloroplast; Complete proteome; Membrane; Nucleotide-binding; Plastid; Reference proteome; Transit peptide; Transmembrane; Transmembrane helix; Transport
SRR342022_TR19824_c0_g1_i1_len=567	NA	NA	NA	NA	NA	.	0,763	-0,188	0,233	-0,808	1	0	1	0	0	7,262	.	.	.	.	.	.
SRR342022_TR21598_c2_g5_i1_len=4976	POLX_TOBAC	Q:22-2109,H:646-1323	46.72%ID	E:3e-176	Retrovirus-related Pol polyprotein from transposon TNT 1-94	PF07727.11^RVT_2^Reverse transcriptase (RNA-dependent DNA polymerase)^217-464^E:1.9e-93	0,007	0,686	0,014	-0,698	0,088	-0,483	0,021	-0,664	0	1,43	POLX_TOBAC	Retrovirus-related Pol polyprotein from transposon TNT 1-94 [Includes: Protease (EC 3.4.23.-); Reverse transcriptase (EC 2.7.7.49); Endonuclease]		Nicotiana tabacum (Common tobacco)		Aspartyl protease; Endonuclease; Hydrolase; Metal-binding; Nuclease; Nucleotidyltransferase; Protease; RNA-directed DNA polymerase; Transferase; Transposable element; Zinc; Zinc-finger
SRR342022_TR21598_c2_g5_i5_len=692	NA	NA	NA	NA	NA	.	1	0	1	0	1	0	1	0	0	7,105	.	.	.	.	.	.
SRR342022_TR23138_c3_g3_i12_len=1911	PLPD2_ARATH	Q:404-99,H:456-557	85.29%ID	E:2e-47	Dihydrolipoyl dehydrogenase 2, chloroplastic	PF13174.3^TPR_6^Tetratricopeptide repeat^219-245^E:0.013	0,577	0,181	0,048	0,576	0,176	0,501	0,118	0,574	0	1,369	PLPD2_ARATH	Dihydrolipoyl dehydrogenase 2, chloroplastic (ptLPD2) (EC 1.8.1.4) (Dihydrolipoamide dehydrogenase 2) (Protein LIPOAMIDE DEHYDROGENASE 2) (Pyruvate dehydrogenase complex E3 subunit 2) (E3-2) (PDC-E3 2)	LPD2 At4g16155 FCAALL.61	Arabidopsis thaliana (Mouse-ear cress)		Chloroplast; Complete proteome; Disulfide bond; FAD; Flavoprotein; NAD; Oxidoreductase; Plastid; Redox-active center; Reference proteome; Transit peptide
SRR342022_TR24136_c2_g2_i2_len=1019	BT3L4_DANRE	Q:137-502,H:1-123	58.54%ID	E:6e-41	Transcription factor BTF3 homolog 4	PF01849.15^NAC^NAC domain^36-91^E:5.1e-19	0,061	-0,719	0,644	-0,175	0,705	0,213	0,798	0,17	0	1,094	BT3L4_DANRE	Transcription factor BTF3 homolog 4 (Basic transcription factor 3-like 4)	btf3l4	Danio rerio (Zebrafish) (Brachydanio rerio)		Complete proteome; Reference proteome
SRR342022_TR26255_c0_g3_i2_len=702	NA	NA	NA	NA	NA	.	0,065	-1,53	0,381	-1,655	0	-5,868	0	-8,664	0,001	2,178	.	.	.	.	.	.
SRR342022_TR27941_c5_g1_i3_len=887	NA	NA	NA	NA	NA	.	0,022	1,449	0,381	1,547	0,043	2,564	0,043	2,569	0	1,166	.	.	.	.	.	.
SRR342022_TR29282_c0_g2_i1_len=693	PSAG_ARATH	Q:552-130,H:27-160	72.34%ID	E:2e-67	Photosystem I reaction center subunit V, chloroplastic	PF01241.15^PSI_PSAK^Photosystem I psaG / psaK^53-144^E:2.5e-26	0,659	-0,093	0	-1,45	0,004	-0,599	0,065	-0,382	0	1,478	PSAG_ARATH	Photosystem I reaction center subunit V, chloroplastic (PSI-G)	PSAG At1g55670 F20N2.21 F20N2.33 F20N2_3	Arabidopsis thaliana (Mouse-ear cress)		Chloroplast; Complete proteome; Membrane; Photosynthesis; Photosystem I; Plastid; Reference proteome; Thylakoid; Transit peptide; Transmembrane; Transmembrane helix
SRR342022_TR30154_c2_g10_i4_len=1487	PME3_CITSI	Q:1324-5,H:40-469	71.85%ID	E:0	Pectinesterase 3	PF04043.12^PMEI^Plant invertase/pectin methylesterase inhibitor^91-247^E:1.3e-28`PF01095.16^Pectinesterase^Pectinesterase^298-492^E:5.2e-97	0,17	-0,425	0,025	2,473	0,299	-1,872	0,034	-5,632	0	5,031	PME3_CITSI	Pectinesterase 3 (PE 3) (EC 3.1.1.11) (Pectin methylesterase 3)		Citrus sinensis (Sweet orange) (Citrus aurantium var. sinensis)	PATHWAY: Glycan metabolism; pectin degradation; 2-dehydro-3-deoxy-D-gluconate from pectin: step 1/5.	Aspartyl esterase; Cell wall; Cell wall biogenesis/degradation; Direct protein sequencing; Disulfide bond; Glycoprotein; Hydrolase; Secreted; Signal
SRR342022_TR31765_c1_g5_i1_len=1829	TBB8_ARATH	Q:901-1827,H:1-309	98.06%ID	E:0	Tubulin beta-8 chain	PF00091.22^Tubulin^Tubulin/FtsZ family, GTPase domain^43-251^E:1.5e-68`PF03953.14^Tubulin_C^Tubulin C-terminal domain^301-348^E:4.9e-13	0,065	-0,485	0,335	0,298	0,541	0,26	0,311	0,407	0	4,595	TBB8_ARATH	Tubulin beta-8 chain (Beta-8-tubulin)	TUBB8 TUB8 At5g23860 MRO11.10	Arabidopsis thaliana (Mouse-ear cress)		Complete proteome; Cytoplasm; Cytoskeleton; GTP-binding; Microtubule; Nucleotide-binding; Reference proteome
SRR342022_TR34286_c0_g2_i1_len=1131	NA	NA	NA	NA	NA	PF11623.5^DUF3252^Protein of unknown function (DUF3252)^174-225^E:2e-28	0,723	0,134	0	-1,784	0,602	-0,118	0,94	-0,019	0	4,824	.	.	.	.	.	.
SRR342023_TR1054_c0_g5_i6_len=4915	AB3C_ARATH	Q:913-4659,H:245-1510	51.96%ID	E:0	ABC transporter C family member 3	PF00664.20^ABC_membrane^ABC transporter transmembrane region^285-548^E:1.4e-22`PF00005.24^ABC_tran^ABC transporter^619-749^E:3.1e-13`PF00664.20^ABC_membrane^ABC transporter transmembrane region^901-1135^E:2.5e-28`PF00005.24^ABC_tran^ABC transporter^1229-1377^E:7.8e-31	1	0,657	1	0,042	1	0,534	1	0,832	0,001	1,543	AB3C_ARATH	ABC transporter C family member 3 (ABC transporter ABCC.3) (AtABCC3) (EC 3.6.3.44) (ATP-energized glutathione S-conjugate pump 3) (Glutathione S-conjugate-transporting ATPase 3) (Multidrug resistance-associated protein 3)	ABCC3 EST2 MRP3 At3g13080 MJG19.3	Arabidopsis thaliana (Mouse-ear cress)		ATP-binding; Alternative splicing; Complete proteome; Hydrolase; Membrane; Nucleotide-binding; Phosphoprotein; Reference proteome; Repeat; Transmembrane; Transmembrane helix; Transport
SRR342023_TR2280_c0_g1_i4_len=757	NA	NA	NA	NA	NA	PF13920.3^zf-C3HC4_3^Zinc finger, C3HC4 type (RING finger)^72-118^E:8.9e-07`PF13639.3^zf-RING_2^Ring finger domain^73-113^E:1.5e-07	1	0	1	0	1	0	1	1,041	0	7,065	.	.	.	.	.	.
SRR342023_TR3519_c2_g1_i1_len=1064	PLAS_SOLLC	Q:219-695,H:11-170	76.54%ID	E:3e-67	Plastocyanin, chloroplastic	PF00127.17^Copper-bind^Copper binding proteins, plastocyanin/azurin family^73-170^E:4.2e-36	0,249	-0,244	0	-1,228	0	-0,788	0,007	-0,563	0	0,951	PLAS_SOLLC	Plastocyanin, chloroplastic	PETE	Solanum lycopersicum (Tomato) (Lycopersicon esculentum)		Chloroplast; Complete proteome; Copper; Electron transport; Membrane; Metal-binding; Plastid; Reference proteome; Thylakoid; Transit peptide; Transport
SRR342023_TR8311_c1_g4_i6_len=1105	Y5566_ARATH	Q:695-339,H:1-117	63.03%ID	E:8e-36	Uncharacterized protein At5g65660	.	0,187	-0,586	0,016	-1,143	1	0,355	0,517	-0,741	0	5,934	Y5566_ARATH	Uncharacterized protein At5g65660	At5g65660 K21L13.18	Arabidopsis thaliana (Mouse-ear cress)		Complete proteome; Membrane; Reference proteome; Transmembrane; Transmembrane helix
SRR342023_TR9121_c5_g1_i3_len=1625	TBB7_GOSHI	Q:82-1359,H:1-426	94.84%ID	E:0	Tubulin beta-7 chain	PF00091.22^Tubulin^Tubulin/FtsZ family, GTPase domain^3-211^E:4e-69`PF03953.14^Tubulin_C^Tubulin C-terminal domain^261-381^E:5.8e-41	0,089	-0,42	0,444	-0,203	0,257	-0,903	0,874	0,129	0	8,852	TBB7_GOSHI	Tubulin beta-7 chain (Beta-7-tubulin)		Gossypium hirsutum (Upland cotton) (Gossypium mexicanum)		Cytoplasm; Cytoskeleton; GTP-binding; Microtubule; Nucleotide-binding
SRR342023_TR10616_c0_g1_i5_len=4184	ACA11_ARATH	Q:3684-493,H:1-1021	67.86%ID	E:0	Putative calcium-transporting ATPase 11, plasma membrane-type	PF12515.5^CaATP_NAI^Ca2+-ATPase N terminal autoinhibitory domain^7-50^E:2.4e-15`PF00690.23^Cation_ATPase_N^Cation transporter/ATPase, N-terminus^154-218^E:1.7e-15`PF00122.17^E1-E2_ATPase^E1-E2 ATPase^238-477^E:3.4e-45`PF00702.23^Hydrolase^haloacid dehalogenase-like hydrolase^484-801^E:2.5e-22`PF13246.3^Cation_ATPase^Cation transport ATPase (P-type)^540-624^E:3.7e-18`PF00689.18^Cation_ATPase_C^Cation transporting ATPase, C-terminus^872-1046^E:6.9e-45	0,69	-0,148	0,007	1,246	0,799	-0,109	0,325	0,368	0,001	1,71	ACA11_ARATH	Putative calcium-transporting ATPase 11, plasma membrane-type (EC 3.6.3.8) (Ca(2+)-ATPase isoform 11)	ACA11 At3g57330 F28O9.180	Arabidopsis thaliana (Mouse-ear cress)		ATP-binding; Calcium; Calcium transport; Calmodulin-binding; Complete proteome; Hydrolase; Ion transport; Magnesium; Membrane; Metal-binding; Nucleotide-binding; Reference proteome; Transmembrane; Transmembrane helix; Transport
SRR342023_TR18177_c2_g1_i2_len=1564	CLPAA_SOLLC	Q:1012-2,H:249-586	95.27%ID	E:0	ATP-dependent Clp protease ATP-binding subunit ClpA homolog CD4A, chloroplastic	PF00004.26^AAA^ATPase family associated with various cellular activities (AAA)^46-162^E:2.8e-15`PF07728.11^AAA_5^AAA domain (dynein-related subfamily)^46-124^E:2.3e-05`PF02151.16^UVR^UvrB/uvrC motif^258-293^E:3.1e-09	0,079	-0,849	1	1,712	0,052	-1,541	0,452	-0,612	0	1,541	CLPAA_SOLLC	ATP-dependent Clp protease ATP-binding subunit ClpA homolog CD4A, chloroplastic	CD4A	Solanum lycopersicum (Tomato) (Lycopersicon esculentum)		ATP-binding; Chaperone; Chloroplast; Complete proteome; Nucleotide-binding; Plastid; Reference proteome; Repeat; Transit peptide
SRR342023_TR20116_c0_g1_i1_len=534	NA	NA	NA	NA	NA	.	0,534	0,186	0	-1,758	0	-2,049	0	-2,412	0,001	1,176	.	.	.	.	.	.
SRR342023_TR20464_c3_g13_i1_len=952	NA	NA	NA	NA	NA	PF13920.3^zf-C3HC4_3^Zinc finger, C3HC4 type (RING finger)^48-94^E:3.2e-09`PF13639.3^zf-RING_2^Ring finger domain^49-89^E:8.9e-09`PF14447.3^Prok-RING_4^Prokaryotic RING finger family 4^51-93^E:1.2e-07`PF00097.22^zf-C3HC4^Zinc finger, C3HC4 type (RING finger)^51-88^E:1.6e-06`PF13445.3^zf-RING_UBOX^RING-type zinc-finger^51-86^E:0.00046	0,5	-0,532	0,195	-1,479	1	-0,33	1	-1,013	0	7,229	.	.	.	.	.	.
SRR342023_TR22207_c3_g7_i4_len=1603	NAGK_DICDI	Q:1257-298,H:4-316	37.58%ID	E:2e-59	N-acetyl-D-glucosamine kinase	PF01869.17^BcrAD_BadFG^BadF/BadG/BcrA/BcrD ATPase family^12-326^E:3.7e-57	0,802	0,115	0,812	0,087	0,152	0,838	0,25	0,644	0	7,457	NAGK_DICDI	N-acetyl-D-glucosamine kinase (N-acetylglucosamine kinase) (EC 2.7.1.59) (GlcNAc kinase)	nagk DDB_G0284433	Dictyostelium discoideum (Slime mold)		ATP-binding; Complete proteome; Kinase; Nucleotide-binding; Reference proteome; Transferase
SRR342023_TR23494_c5_g1_i1_len=651	PREP2_ARATH	Q:438-1,H:40-180	66.44%ID	E:8e-51	Presequence protease 2, chloroplastic/mitochondrial	.	1	0	1	0	1	0	1	0	0	8,749	PREP2_ARATH	Presequence protease 2, chloroplastic/mitochondrial (AtPreP2) (PreP 2) (EC 3.4.24.-) (Zinc metalloprotease 2) (AtZnMP2)	PREP2 ZNMP2 At1g49630 F14J22.13	Arabidopsis thaliana (Mouse-ear cress)		Chloroplast; Complete proteome; Hydrolase; Magnesium; Metal-binding; Metalloprotease; Mitochondrion; Plastid; Protease; Reference proteome; Transit peptide; Zinc
SRR342023_TR23701_c1_g3_i1_len=1180	CLPR2_ARATH	Q:1073-228,H:1-279	73.05%ID	E:5e-149	ATP-dependent Clp protease proteolytic subunit-related protein 2, chloroplastic {ECO:0000303|PubMed:11299370}	PF00574.20^CLP_protease^Clp protease^89-264^E:1.8e-63	0,287	-0,29	0,009	-0,755	0,528	-0,188	0,374	-0,266	0	7,405	CLPR2_ARATH	ATP-dependent Clp protease proteolytic subunit-related protein 2, chloroplastic (ClpR2) (nClpP2)	CLPR2 NCLPP2 At1g12410 F5O11.13	Arabidopsis thaliana (Mouse-ear cress)		3D-structure; Chloroplast; Complete proteome; Direct protein sequencing; Plastid; Reference proteome; Transit peptide
SRR342023_TR25396_c0_g3_i4_len=549	MD36B_ARATH	Q:357-506,H:67-116	84%ID	E:2e-22	Probable mediator of RNA polymerase II transcription subunit 36b	PF01269.14^Fibrillarin^Fibrillarin^77-117^E:1.9e-09	1	2,562	1	0	1	1,224	1	-1,914	0	7,361	MD36B_ARATH	Probable mediator of RNA polymerase II transcription subunit 36b (Histone-glutamine methyltransferase) (SKP1-interacting partner 7) (rRNA 2'-O-methyltransferase fibrillarin 1) (EC 2.1.1.-)	MED36B FBR1 FIB1 MED36_2 SKIP7 At5g52470 K24M7.22	Arabidopsis thaliana (Mouse-ear cress)		Alternative splicing; Complete proteome; Methyltransferase; Nucleus; RNA-binding; Reference proteome; Ribonucleoprotein; S-adenosyl-L-methionine; Transcription; Transcription regulation; Transferase; rRNA processing
SRR342023_TR25614_c1_g6_i1_len=732	METK1_CATRO	Q:589-2,H:1-196	99.49%ID	E:2e-141	S-adenosylmethionine synthase 1	PF00438.17^S-AdoMet_synt_N^S-adenosylmethionine synthetase, N-terminal domain^4-101^E:1.9e-43`PF02772.13^S-AdoMet_synt_M^S-adenosylmethionine synthetase, central domain^117-196^E:1e-32	0,103	1,59	0,502	3,834	0,001	-3,746	0	-6,815	0	1,31	METK1_CATRO	S-adenosylmethionine synthase 1 (AdoMet synthase 1) (EC 2.5.1.6) (Methionine adenosyltransferase 1) (MAT 1)	SAMS1	Catharanthus roseus (Madagascar periwinkle) (Vinca rosea)	PATHWAY: Amino-acid biosynthesis; S-adenosyl-L-methionine biosynthesis; S-adenosyl-L-methionine from L-methionine: step 1/1.	ATP-binding; Cobalt; Cytoplasm; Magnesium; Manganese; Metal-binding; Nucleotide-binding; One-carbon metabolism; Potassium; Transferase
SRR342023_TR27473_c2_g11_i6_len=698	ILV5_ARATH	Q:1-459,H:197-349	94.12%ID	E:1e-88	Ketol-acid reductoisomerase, chloroplastic	PF07991.9^IlvN^Acetohydroxy acid isomeroreductase, NADPH-binding domain^4-98^E:6.1e-13`PF01450.16^IlvC^Acetohydroxy acid isomeroreductase, catalytic domain^107-164^E:2.9e-17	0,014	-0,999	0,793	-0,211	0,484	-0,457	0,9	0,11	0	8,408	ILV5_ARATH	Ketol-acid reductoisomerase, chloroplastic (EC 1.1.1.86) (Acetohydroxy-acid reductoisomerase) (Alpha-keto-beta-hydroxylacyl reductoisomerase)	At3g58610 F14P22.200	Arabidopsis thaliana (Mouse-ear cress)	PATHWAY: Amino-acid biosynthesis; L-isoleucine biosynthesis; L-isoleucine from 2-oxobutanoate: step 2/4.; PATHWAY: Amino-acid biosynthesis; L-valine biosynthesis; L-valine from pyruvate: step 2/4.	Amino-acid biosynthesis; Branched-chain amino acid biosynthesis; Chloroplast; Complete proteome; Magnesium; Metal-binding; NADP; Oxidoreductase; Plastid; Reference proteome; Transit peptide
SRR342023_TR27560_c2_g1_i2_len=588	NA	NA	NA	NA	NA	.	1	-0,379	1	-0,813	1	0,794	0,299	1,705	0	1,042	.	.	.	.	.	.
SRR342023_TR29871_c4_g1_i1_len=945	GPX8_ARATH	Q:120-614,H:2-166	79.39%ID	E:7e-95	Probable glutathione peroxidase 8	PF00255.16^GSHPx^Glutathione peroxidase^37-145^E:8.7e-42`PF00578.18^AhpC-TSA^AhpC/TSA family^37-98^E:5.4e-08	0,912	-0,082	0,458	-0,424	0,35	0,587	0,35	0,6	0	6,882	GPX8_ARATH	Probable glutathione peroxidase 8 (EC 1.11.1.9)	GPX8 At1g63460 F2K11.16	Arabidopsis thaliana (Mouse-ear cress)		Complete proteome; Oxidoreductase; Peroxidase; Reference proteome
SRR342023_TR31010_c0_g1_i3_len=639	P2B11_ARATH	Q:93-584,H:88-255	36.69%ID	E:2e-21	F-box protein PP2-B11	PF14299.3^PP2^Phloem protein 2^37-194^E:4.2e-32	1	0	1	0	1	0	1	0	0	1,469	P2B11_ARATH	F-box protein PP2-B11 (Protein PHLOEM PROTEIN 2-LIKE B11) (AtPP2-B11) (SKP1-interacting partner 12)	PP2B11 SKIP12 At1g80110 F18B13.19	Arabidopsis thaliana (Mouse-ear cress)	PATHWAY: Protein modification; protein ubiquitination.	Complete proteome; Nucleus; Reference proteome; Ubl conjugation pathway
SRR342023_TR31672_c2_g2_i4_len=927	KSG10_ARATH	Q:610-2,H:1-199	76.85%ID	E:3e-108	Shaggy-related protein kinase kappa	PF00069.22^Pkinase^Protein kinase domain^89-203^E:5.6e-20`PF07714.14^Pkinase_Tyr^Protein tyrosine kinase^90-203^E:4.2e-10	1	0	1	0	1	0	1	0	0	6,753	KSG10_ARATH	Shaggy-related protein kinase kappa (EC 2.7.11.1) (ASK-kappa) (AtK-1)	ASK10 At1g09840 F21M12.23	Arabidopsis thaliana (Mouse-ear cress)		ATP-binding; Complete proteome; Kinase; Nucleotide-binding; Phosphoprotein; Reference proteome; Serine/threonine-protein kinase; Transferase
SRR646572_TR429_c0_g1_i4_len=1286	NA	NA	NA	NA	NA	.	1	-0,634	1	0	1	-1,052	1	-1,052	0	7,368	.	.	.	.	.	.
SRR646572_TR1408_c1_g1_i3_len=1502	POLX_TOBAC	Q:64-1500,H:5-484	33.06%ID	E:1e-74	Retrovirus-related Pol polyprotein from transposon TNT 1-94	PF14223.3^Retrotran_gag_2^gag-polypeptide of LTR copia-type^39-169^E:1.8e-33`PF00098.20^zf-CCHC^Zinc knuckle^209-224^E:0.00024`PF13976.3^gag_pre-integrs^GAG-pre-integrase domain^379-446^E:2.1e-11	0,732	0,68	1	0,159	1	0,062	1	-0,978	0	3,889	POLX_TOBAC	Retrovirus-related Pol polyprotein from transposon TNT 1-94 [Includes: Protease (EC 3.4.23.-); Reverse transcriptase (EC 2.7.7.49); Endonuclease]		Nicotiana tabacum (Common tobacco)		Aspartyl protease; Endonuclease; Hydrolase; Metal-binding; Nuclease; Nucleotidyltransferase; Protease; RNA-directed DNA polymerase; Transferase; Transposable element; Zinc; Zinc-finger
SRR646572_TR7228_c0_g1_i1_len=1069	PLAS_SOLLC	Q:847-371,H:11-170	77.16%ID	E:3e-68	Plastocyanin, chloroplastic	PF00127.17^Copper-bind^Copper binding proteins, plastocyanin/azurin family^73-170^E:2.1e-36	0,535	-0,272	0,004	-1,053	0,14	-0,441	0,648	-0,141	0	3,336	PLAS_SOLLC	Plastocyanin, chloroplastic	PETE	Solanum lycopersicum (Tomato) (Lycopersicon esculentum)		Chloroplast; Complete proteome; Copper; Electron transport; Membrane; Metal-binding; Plastid; Reference proteome; Thylakoid; Transit peptide; Transport
SRR646572_TR7923_c0_g1_i1_len=949	NA	NA	NA	NA	NA	.	0,581	0,468	0,429	-0,522	0,214	0,825	0,694	0,355	0	3,978	.	.	.	.	.	.
SRR646572_TR9880_c0_g1_i3_len=3102	NA	NA	NA	NA	NA	PF02536.11^mTERF^mTERF^209-282^E:7.4e-09`PF02536.11^mTERF^mTERF^271-462^E:5.7e-38	0,266	-0,477	0,046	-0,913	0,848	-0,218	1	-0,087	0	6,924	.	.	.	.	.	.
SRR646572_TR14099_c0_g1_i2_len=825	GASA4_ARATH	Q:535-278,H:20-106	70.45%ID	E:5e-34	Gibberellin-regulated protein 4	PF02704.11^GASA^Gibberellin regulated protein^47-106^E:3.7e-22	1	0	1	0	1	0	1	0	0,001	6,638	GASA4_ARATH	Gibberellin-regulated protein 4 (GAST1 protein homolog 4)	GASA4 At5g15230 F8M21.120	Arabidopsis thaliana (Mouse-ear cress)		Alternative splicing; Complete proteome; Disulfide bond; Gibberellin signaling pathway; Reference proteome; Secreted; Signal
SRR646572_TR21106_c0_g1_i1_len=866	R27A3_ARATH	Q:134-571,H:1-146	89.04%ID	E:8e-89	60S ribosomal protein L27a-3	PF00828.16^Ribosomal_L27A^Ribosomal proteins 50S-L15, 50S-L18e, 60S-L27A^72-187^E:6.7e-23	1	0	1	0	1	-0,145	1	-0,769	0	7,766	R27A3_ARATH	60S ribosomal protein L27a-3	RPL27AC At1g70600 F24J13.17 F5A18.22	Arabidopsis thaliana (Mouse-ear cress)		Complete proteome; Reference proteome; Ribonucleoprotein; Ribosomal protein
SRR646572_TR21462_c0_g1_i1_len=1231	NA	NA	NA	NA	NA	PF03652.12^RuvX^Holliday junction resolvase^23-158^E:1.9e-23	1	-0,011	1	-0,02	1	0,094	1	-0,068	0	6,855	.	.	.	.	.	.
SRR646572_TR23472_c0_g2_i1_len=2432	ORP3C_ARATH	Q:697-2067,H:1-457	82.53%ID	E:0	Oxysterol-binding protein-related protein 3C	PF01237.15^Oxysterol_BP^Oxysterol-binding protein^70-423^E:5.5e-101	1	0	1	-0,141	1	-0,091	1	-0,403	0	4,138	ORP3C_ARATH	Oxysterol-binding protein-related protein 3C (OSBP-related protein 3C)	ORP3C At5g59420 F2O15.10	Arabidopsis thaliana (Mouse-ear cress)		Complete proteome; Lipid transport; Lipid-binding; Reference proteome; Transport
SRR646572_TR29822_c0_g1_i1_len=1582	GEDH1_OCIBA	Q:161-1228,H:3-358	65.73%ID	E:8e-142	Geraniol dehydrogenase 1	PF08240.9^ADH_N^Alcohol dehydrogenase GroES-like domain^39-151^E:6.3e-28`PF00107.23^ADH_zinc_N^Zinc-binding dehydrogenase^195-318^E:7.5e-19`PF13602.3^ADH_zinc_N_2^Zinc-binding dehydrogenase^228-347^E:2.8e-07	1	0	1	1,979	1	0	1	0	0	7,306	GEDH1_OCIBA	Geraniol dehydrogenase 1 (ObaGEDH1) (EC 1.1.1.183)	GEDH1	Ocimum basilicum (Sweet basil)		Metal-binding; NADP; Oxidoreductase; Zinc
SRR646572_TR29822_c0_g1_i3_len=1634	MTDH_FRAAN	Q:278-1336,H:4-356	70.54%ID	E:1e-157	Probable mannitol dehydrogenase	PF08240.9^ADH_N^Alcohol dehydrogenase GroES-like domain^92-204^E:2.6e-26`PF00107.23^ADH_zinc_N^Zinc-binding dehydrogenase^248-371^E:1.3e-19	0,012	0,813	0,002	0,909	0,622	0,137	0,759	0,094	0	1,159	MTDH_FRAAN	Probable mannitol dehydrogenase (EC 1.1.1.255) (NAD-dependent mannitol dehydrogenase)	CAD	Fragaria ananassa (Strawberry)		Metal-binding; NAD; Oxidoreductase; Zinc
SRR646572_TR30079_c0_g1_i1_len=1778	PRP18_HUMAN	Q:477-1319,H:77-342	38.18%ID	E:7e-54	Pre-mRNA-splicing factor 18	PF08799.8^PRP4^pre-mRNA processing factor 4 (PRP4) like^15-41^E:1.9e-11`PF02840.12^Prp18^Prp18 domain^136-277^E:2.4e-62	1	0	1	0	0,76	-0,595	0,132	-2,104	0	6,801	PRP18_HUMAN	Pre-mRNA-splicing factor 18 (PRP18 homolog) (hPRP18)	PRPF18 HPRP18	Homo sapiens (Human)		3D-structure; Acetylation; Alternative splicing; Complete proteome; Nucleus; Reference proteome; Spliceosome; mRNA processing; mRNA splicing
SRR646572_TR34696_c0_g3_i1_len=637	NA	NA	NA	NA	NA	.	1	0,35	1	0,63	1	0,061	1	0,025	0	3,405	.	.	.	.	.	.
SRR646572_TR35457_c0_g1_i2_len=872	NA	NA	NA	NA	NA	.	1	-0,059	1	-1,82	1	0,554	1	-0,553	0	2,015	.	.	.	.	.	.
SRR646596_TR2901_c0_g1_i1_len=1885	CBDAS_CANSA	Q:162-1661,H:30-538	43.24%ID	E:3e-137	Cannabidiolic acid synthase	PF01565.20^FAD_binding_4^FAD binding domain^79-215^E:2.1e-28`PF08031.9^BBE^Berberine and berberine like^468-526^E:6e-23	0,381	1,474	0,502	3,639	0,381	2,622	0,381	2,496	0	4,919	CBDAS_CANSA	Cannabidiolic acid synthase (EC 1.21.3.8) (CBDA synthase.)	CBDAS	Cannabis sativa (Hemp) (Marijuana)		Apoplast; Direct protein sequencing; Disulfide bond; FAD; Flavoprotein; Glycoprotein; Oxidoreductase; Secreted; Signal
SRR646596_TR5516_c0_g1_i1_len=1977	OSB2_ARATH	Q:1641-694,H:84-366	40.69%ID	E:7e-66	Protein OSB2, chloroplastic	.	1	-1,257	1	0,472	1	-0,996	1	-0,054	0,001	5,556	OSB2_ARATH	Protein OSB2, chloroplastic (Organellar single-stranded DNA-binding protein 2) (Protein FLORAL ABSCISSION ASSOCIATED) (Protein PLASTID TRANSCRIPTIONALLY ACTIVE 9)	OSB2 FAA PTAC9 At4g20010 F18F4.110	Arabidopsis thaliana (Mouse-ear cress)		Alternative splicing; Chloroplast; Complete proteome; DNA-binding; Plastid; Reference proteome; Repeat; Transit peptide
SRR646596_TR6655_c0_g1_i2_len=1322	NA	NA	NA	NA	NA	.	1	1,529	1	0,691	1	1,155	1	-0,153	0	5,613	.	.	.	.	.	.
SRR646596_TR7727_c0_g1_i1_len=994	Y5957_ARATH	Q:299-862,H:19-183	45.45%ID	E:5e-29	Uncharacterized protein At5g39570	.	1	-0,724	0,692	0,799	1	-0,93	1	-0,146	0	5,635	Y5957_ARATH	Uncharacterized protein At5g39570	At5g39570 MIJ24_40	Arabidopsis thaliana (Mouse-ear cress)		Complete proteome; Direct protein sequencing; Phosphoprotein; Reference proteome
SRR646596_TR11634_c0_g1_i1_len=1661	NA	NA	NA	NA	NA	PF12678.4^zf-rbx1^RING-H2 zinc finger domain^83-130^E:8.3e-11`PF13639.3^zf-RING_2^Ring finger domain^87-130^E:3.1e-15`PF17123.2^zf-RING_11^RING-like zinc finger^87-116^E:4.2e-11`PF13923.3^zf-C3HC4_2^Zinc finger, C3HC4 type (RING finger)^87-129^E:7.6e-10`PF13445.3^zf-RING_UBOX^RING-type zinc-finger^88-127^E:2.9e-08`PF00097.22^zf-C3HC4^Zinc finger, C3HC4 type (RING finger)^88-129^E:2.7e-08	0,874	0,13	1	-0,128	0,272	0,484	0,231	0,502	0	5,638	.	.	.	.	.	.
SRR646596_TR12031_c0_g2_i1_len=780	NA	NA	NA	NA	NA	PF10950.5^DUF2775^Protein of unknown function (DUF2775)^42-152^E:2.5e-22`PF10950.5^DUF2775^Protein of unknown function (DUF2775)^159-230^E:2.5e-15	0,061	0,493	0,001	-3,029	1	1,311	1	1,875	0	0,937	.	.	.	.	.	.
SRR646596_TR16647_c0_g1_i1_len=763	Y5258_ARATH	Q:123-401,H:6-98	64.52%ID	E:5e-35	Uncharacterized protein At5g22580	PF07876.9^Dabb^Stress responsive A/B Barrel Domain^5-97^E:6.4e-23	1	2,594	1	3,683	0,502	3,756	1	0	0	8,97	Y5258_ARATH	Uncharacterized protein At5g22580	At5g22580 MQJ16.12	Arabidopsis thaliana (Mouse-ear cress)		3D-structure; Complete proteome; Magnesium; Metal-binding; Reference proteome

SRR646596_TR17100_c0_g3_i2_len=810	P2B11_ARATH	Q:73-564,H:88-255	36.31%ID	E:1e-21	F-box protein PP2-B11	PF14299.3^PP2^Phloem protein 2^3-160^E:1.9e-32	1	0	1	0	0,502	-4,266	0,502	-4,266	0	1,018	P2B11_ARATH	F-box protein PP2-B11 (Protein PHLOEM PROTEIN 2-LIKE B11) (AtPP2-B11) (SKP1-interacting partner 12)	PP2B11 SKIP12 At1g80110 F18B13.19	Arabidopsis thaliana (Mouse-ear cress)	PATHWAY: Protein modification; protein ubiquitination.	Complete proteome; Nucleus; Reference proteome; Ubl conjugation pathway
SRR646596_TR20825_c0_g1_i3_len=722	EF1A_MANES	Q:130-594,H:1-155	99.35%ID	E:2e-106	Elongation factor 1-alpha	PF00009.24^GTP_EFTU^Elongation factor Tu GTP binding domain^49-198^E:4.7e-44	0,056	-0,808	0,017	1,472	0,104	-0,877	0,142	-0,735	0,001	0,924	EF1A_MANES	Elongation factor 1-alpha (EF-1-alpha)	EF1	Manihot esculenta (Cassava) (Jatropha manihot)		Cytoplasm; Elongation factor; GTP-binding; Nucleotide-binding; Phosphoprotein; Protein biosynthesis
SRR646596_TR28974_c0_g1_i2_len=1953	MDAR_SOLLC	Q:245-1501,H:16-433	86.4%ID	E:0	Monodehydroascorbate reductase	PF07992.11^Pyr_redox_2^Pyridine nucleotide-disulphide oxidoreductase^2-132^E:3.6e-23	1	2,675	0,502	2,108	1	2,57	1	2,44	0	7,847	MDAR_SOLLC	Monodehydroascorbate reductase (MDAR) (EC 1.6.5.4) (Ascorbate free radical reductase) (AFR reductase)	AFRR	Solanum lycopersicum (Tomato) (Lycopersicon esculentum)		Complete proteome; Cytoplasm; FAD; Flavoprotein; NAD; Oxidoreductase; Redox-active center; Reference proteome
SRR646596_TR30857_c1_g1_i3_len=2797	PAL1_TOBAC	Q:2065-218,H:100-715	90.75%ID	E:0	Phenylalanine ammonia-lyase	PF00221.16^Lyase_aromatic^Aromatic amino acid lyase^2-440^E:1e-144	1	0,736	0,254	-4,601	1	0,006	1	-0,611	0	7,74	PAL1_TOBAC	Phenylalanine ammonia-lyase (EC 4.3.1.24)	TPA1 PALB	Nicotiana tabacum (Common tobacco)	PATHWAY: Phenylpropanoid metabolism; trans-cinnamate biosynthesis; trans-cinnamate from L-phenylalanine: step 1/1.	Cytoplasm; Lyase; Phenylpropanoid metabolism
SRR646596_TR30884_c0_g1_i1_len=1048	BGLT_TRIRP	Q:851-165,H:25-254	67.39%ID	E:8e-101	Cyanogenic beta-glucosidase	PF00232.15^Glyco_hydro_1^Glycosyl hydrolase family 1^69-293^E:1.1e-84	0,692	-0,88	1	0,727	1	-3,139	1	-3,139	0	1,503	BGLT_TRIRP	Cyanogenic beta-glucosidase (EC 3.2.1.21) (Linamarase) (Fragment)	LI	Trifolium repens (Creeping white clover)		3D-structure; Direct protein sequencing; Disulfide bond; Glycoprotein; Glycosidase; Hydrolase; Signal
SRR646596_TR33990_c0_g1_i1_len=770	NDUA2_ARATH	Q:636-352,H:1-95	66.32%ID	E:1e-36	NADH dehydrogenase [ubiquinone] 1 alpha subcomplex subunit 2	.	1	0	1	0	1	0	1	0	0	7,707	NDUA2_ARATH	NADH dehydrogenase [ubiquinone] 1 alpha subcomplex subunit 2	At5g47890 MCA23_23	Arabidopsis thaliana (Mouse-ear cress)		Complete proteome; Disulfide bond; Electron transport; Membrane; Mitochondrion; Mitochondrion inner membrane; Reference proteome; Respiratory chain; Transport
SRR646596_TR34697_c0_g1_i2_len=3167	PMT8_ARATH	Q:2813-951,H:1-623	79.01%ID	E:0	Probable methyltransferase PMT8	PF03141.13^Methyltransf_29^Putative S-adenosyl-L-methionine-dependent methyltransferase^97-608^E:5.6e-225`PF08241.9^Methyltransf_11^Methyltransferase domain^225-318^E:2.2e-06	0,816	-0,112	1	0,012	0,548	-0,287	0,415	-0,367	0	3,373	PMT8_ARATH	Probable methyltransferase PMT8 (EC 2.1.1.-)	At1g04430 F19P19.11	Arabidopsis thaliana (Mouse-ear cress)		Complete proteome; Glycoprotein; Golgi apparatus; Membrane; Methyltransferase; Reference proteome; Signal-anchor; Transferase; Transmembrane; Transmembrane helix
SRR646596_TR34714_c0_g3_i1_len=1888	GUN6_ARATH	Q:1888-548,H:174-619	75.06%ID	E:0	Endoglucanase 6	PF00759.16^Glyco_hydro_9^Glycosyl hydrolase family 9^2-312^E:5e-80`PF09478.7^CBM49^Carbohydrate binding domain CBM49^356-433^E:3.3e-27	0,692	1,139	0,517	-0,878	1	-0,557	0,629	-0,998	0	4,164	GUN6_ARATH	Endoglucanase 6 (EC 3.2.1.4) (Endo-1,4-beta glucanase 6)	At1g64390 F15H21.9	Arabidopsis thaliana (Mouse-ear cress)		Carbohydrate metabolism; Cell wall biogenesis/degradation; Cellulose degradation; Complete proteome; Glycoprotein; Glycosidase; Hydrolase; Polysaccharide degradation; Reference proteome; Secreted; Signal
SRR646604_TR174_c3_g1_i1_len=2299	HSP72_SOLLC	Q:2100-163,H:1-644	94.27%ID	E:0	Heat shock cognate 70 kDa protein 2	PF00012.17^HSP70^Hsp70 protein^9-618^E:4.5e-270`PF06723.10^MreB_Mbl^MreB/Mbl protein^131-380^E:4.1e-14	1	0,335	1	1,31	1	0,148	1	-0,5	0	9,079	HSP72_SOLLC	Heat shock cognate 70 kDa protein 2	HSC-2	Solanum lycopersicum (Tomato) (Lycopersicon esculentum)		ATP-binding; Complete proteome; Nucleotide-binding; Reference proteome; Stress response
SRR646604_TR607_c1_g2_i4_len=2319	RFS2_ARATH	Q:2-1423,H:177-653	80.79%ID	E:0	Probable galactinol--sucrose galactosyltransferase 2	PF05691.9^Raffinose_syn^Raffinose synthase or seed imbibition protein Sip1^1-456^E:5.6e-230	1	0,114	0,004	1,785	0,006	0,935	0,424	0,314	0	1,292	RFS2_ARATH	Probable galactinol--sucrose galactosyltransferase 2 (EC 2.4.1.82) (Protein SEED IMBIBITION 2) (Raffinose synthase 2)	RFS2 RS2 SIP2 At3g57520 T8H10.120	Arabidopsis thaliana (Mouse-ear cress)		Alternative splicing; Carbohydrate metabolism; Complete proteome; Glycosyltransferase; Reference proteome; Transferase
SRR646604_TR2448_c0_g12_i1_len=623	TSJT1_TOBAC	Q:442-2,H:1-148	44.97%ID	E:7e-38	Stem-specific protein TSJT1	PF12481.5^DUF3700^Aluminium induced protein^2-147^E:1.7e-54	1	0,312	0,023	1,79	0,136	0,597	0,004	1,046	0	1,159	TSJT1_TOBAC	Stem-specific protein TSJT1	TSJT1	Nicotiana tabacum (Common tobacco)		
SRR646604_TR5426_c0_g1_i1_len=6108	CLAH1_ARATH	Q:2778-5543,H:736-1657	93.49%ID	E:0	Clathrin heavy chain 1	PF00637.17^Clathrin^Region in Clathrin and VPS^73-201^E:5.3e-29`PF00637.17^Clathrin^Region in Clathrin and VPS^216-354^E:2e-31`PF00637.17^Clathrin^Region in Clathrin and VPS^368-504^E:1.1e-26`PF00637.17^Clathrin^Region in Clathrin and VPS^512-654^E:5.5e-29`PF00637.17^Clathrin^Region in Clathrin and VPS^662-802^E:5.4e-31	1	-0,272	1	0,427	1	-0,094	1	1,577	0	5,654	CLAH1_ARATH	Clathrin heavy chain 1	CHC1 At3g11130 F11B9.30 F9F8.6	Arabidopsis thaliana (Mouse-ear cress)		Acetylation; Coated pit; Complete proteome; Cytoplasmic vesicle; Endocytosis; Membrane; Reference proteome; Repeat
SRR646604_TR7370_c0_g2_i3_len=928	NA	NA	NA	NA	NA	.	1	0	1	1,617	1	1,593	1	-0,238	0	5,965	.	.	.	.	.	.
SRR646604_TR9504_c0_g1_i1_len=2929	SSY2_SOLTU	Q:326-2650,H:1-767	65.95%ID	E:0	Granule-bound starch synthase 2, chloroplastic/amyloplastic	PF08323.8^Glyco_transf_5^Starch synthase catalytic domain^299-541^E:1.2e-64`PF00534.17^Glycos_transf_1^Glycosyl transferases group 1^601-742^E:2.6e-16`PF13692.3^Glyco_trans_1_4^Glycosyl transferases group 1^604-743^E:5.9e-15	1	0	1	0,174	1	-0,535	1	-0,094	0	5,441	SSY2_SOLTU	Granule-bound starch synthase 2, chloroplastic/amyloplastic (EC 2.4.1.21) (Granule-bound starch synthase II) (GBSS-II) (SS II)	SS2 SSII	Solanum tuberosum (Potato)	PATHWAY: Glycan biosynthesis; starch biosynthesis.	Amyloplast; Chloroplast; Complete proteome; Direct protein sequencing; Glycosyltransferase; Plastid; Polymorphism; Reference proteome; Starch biosynthesis; Transferase; Transit peptide
SRR646604_TR14248_c0_g1_i4_len=1011	NA	NA	NA	NA	NA	.	1	-0,324	1	-1,261	0,502	-2,442	0,502	-2,401	0	5,225	.	.	.	.	.	.
SRR646604_TR14248_c1_g2_i1_len=2835	POLX_TOBAC	Q:8-2218,H:605-1323	47.11%ID	E:0	Retrovirus-related Pol polyprotein from transposon TNT 1-94	PF07727.11^RVT_2^Reverse transcriptase (RNA-dependent DNA polymerase)^253-500^E:3.9e-93	1	-0,912	1	0,224	1	0,047	1	-0,4	0	6,034	POLX_TOBAC	Retrovirus-related Pol polyprotein from transposon TNT 1-94 [Includes: Protease (EC 3.4.23.-); Reverse transcriptase (EC 2.7.7.49); Endonuclease]		Nicotiana tabacum (Common tobacco)		Aspartyl protease; Endonuclease; Hydrolase; Metal-binding; Nuclease; Nucleotidyltransferase; Protease; RNA-directed DNA polymerase; Transferase; Transposable element; Zinc; Zinc-finger
SRR646604_TR16577_c2_g1_i3_len=962	GOLS1_AJURE	Q:124-849,H:1-235	78.93%ID	E:3e-134	Galactinol synthase 1	PF01501.17^Glyco_transf_8^Glycosyl transferase family 8^73-254^E:1.8e-28	1	1,34	1	1,653	1	-0,034	1	0,886	0	1,493	GOLS1_AJURE	Galactinol synthase 1 (ArGolS1) (GolS-1) (EC 2.4.1.123)	GOLS1	Ajuga reptans (Bugle)		Carbohydrate metabolism; Cytoplasm; Galactose metabolism; Glycosyltransferase; Manganese; Metal-binding; Transferase
SRR646604_TR19244_c0_g1_i1_len=3008	EFGC2_SOYBN	Q:457-2766,H:13-780	87.05%ID	E:0	Elongation factor G-2, chloroplastic	PF00009.24^GTP_EFTU^Elongation factor Tu GTP binding domain^98-370^E:4.7e-71`PF03144.22^GTP_EFTU_D2^Elongation factor Tu domain 2^413-480^E:1.4e-15`PF14492.3^EFG_II^Elongation Factor G, domain II^493-566^E:4.7e-33`PF03764.15^EFG_IV^Elongation factor G, domain IV^568-686^E:2.3e-45`PF00679.21^EFG_C^Elongation factor G C-terminus^690-776^E:4.5e-26	0,073	-0,697	0,058	-0,96	0,008	-0,73	0,717	-0,105	0	1,185	EFGC2_SOYBN	Elongation factor G-2, chloroplastic (cEF-G 2)	fusA2	Glycine max (Soybean) (Glycine hispida)	PATHWAY: Protein biosynthesis; polypeptide chain elongation. {ECO:0000255|HAMAP-Rule:MF_03063}.	Chloroplast; Complete proteome; Elongation factor; GTP-binding; Nucleotide-binding; Plastid; Protein biosynthesis; Reference proteome; Transit peptide
SRR646604_TR19799_c2_g1_i3_len=1245	POLX_TOBAC	Q:1210-275,H:125-437	31.78%ID	E:2e-48	Retrovirus-related Pol polyprotein from transposon TNT 1-94	PF14223.3^Retrotran_gag_2^gag-polypeptide of LTR copia-type^3-74^E:2.1e-14`PF00098.20^zf-CCHC^Zinc knuckle^114-129^E:0.00012	1	0,808	1	0,111	1	-1,475	1	-1,591	0	6,129	POLX_TOBAC	Retrovirus-related Pol polyprotein from transposon TNT 1-94 [Includes: Protease (EC 3.4.23.-); Reverse transcriptase (EC 2.7.7.49); Endonuclease]		Nicotiana tabacum (Common tobacco)		Aspartyl protease; Endonuclease; Hydrolase; Metal-binding; Nuclease; Nucleotidyltransferase; Protease; RNA-directed DNA polymerase; Transferase; Transposable element; Zinc; Zinc-finger
SRR646604_TR19799_c2_g2_i1_len=518	NA	NA	NA	NA	NA	PF14223.3^Retrotran_gag_2^gag-polypeptide of LTR copia-type^112-170^E:4.3e-13	1	-0,173	1	-1,547	1	-1,649	1	0,198	0	6,683	.	.	.	.	.	.
SRR646604_TR20238_c0_g1_i2_len=574	NA	NA	NA	NA	NA	.	1	0	1	0	1	0	1	0	0,001	6,566	.	.	.	.	.	.
SRR646604_TR23014_c0_g1_i1_len=2243	ASNS3_ARATH	Q:180-1841,H:1-554	87.55%ID	E:0	Asparagine synthetase [glutamine-hydrolyzing] 3	PF13522.3^GATase_6^Glutamine amidotransferase domain^92-219^E:1.3e-35`PF13537.3^GATase_7^Glutamine amidotransferase domain^107-224^E:1e-43`PF12481.5^DUF3700^Aluminium induced protein^171-258^E:5e-10`PF00733.18^Asn_synthase^Asparagine synthase^269-570^E:5.6e-98	1	-0,329	1	0,784	1	-0,51	1	-0,482	0	4,739	ASNS3_ARATH	Asparagine synthetase [glutamine-hydrolyzing] 3 (EC 6.3.5.4) (Glutamine-dependent asparagine synthetase 3)	ASN3 At5g10240 F18D22.10 T31P16.230	Arabidopsis thaliana (Mouse-ear cress)	PATHWAY: Amino-acid biosynthesis; L-asparagine biosynthesis.	ATP-binding; Alternative splicing; Amino-acid biosynthesis; Asparagine biosynthesis; Complete proteome; Glutamine amidotransferase; Ligase; Nucleotide-binding; Reference proteome
SRR646604_TR23580_c0_g2_i1_len=1832	SCP51_ARATH	Q:204-1493,H:21-459	66.74%ID	E:0	Serine carboxypeptidase-like 51	PF00450.19^Peptidase_S10^Serine carboxypeptidase^36-449^E:9e-71	0,581	0,335	1	-0,045	1	0,306	1	0,074	0	3,458	SCP51_ARATH	Serine carboxypeptidase-like 51 (EC 3.4.16.-)	SCPL51 At2g27920 T1E2.16	Arabidopsis thaliana (Mouse-ear cress)		Alternative splicing; Carboxypeptidase; Complete proteome; Glycoprotein; Hydrolase; Protease; Reference proteome; Secreted; Signal
SRR646604_TR23924_c0_g1_i2_len=2113	GSH1_TOBAC	Q:288-1862,H:1-522	83.84%ID	E:0	Glutamate--cysteine ligase, chloroplastic	PF04107.10^GCS2^Glutamate-cysteine ligase family 2(GCS2)^147-435^E:1.3e-94	0,641	-0,257	0,13	0,671	0,691	0,26	0,183	0,646	0	1,955	GSH1_TOBAC	Glutamate--cysteine ligase, chloroplastic (EC 6.3.2.2) (Gamma-ECS) (GCS) (Gamma-glutamylcysteine synthetase)	GSH1	Nicotiana tabacum (Common tobacco)	PATHWAY: Sulfur metabolism; glutathione biosynthesis; glutathione from L-cysteine and L-glutamate: step 1/2.	ATP-binding; Chloroplast; Disulfide bond; Glutathione biosynthesis; Ligase; Nucleotide-binding; Plastid; Transit peptide
SRR646604_TR25316_c0_g2_i2_len=2192	APX1_ARATH	Q:1168-419,H:1-250	85.2%ID	E:5e-141	L-ascorbate peroxidase 1, cytosolic	PF00141.20^peroxidase^Peroxidase^22-227^E:4.9e-49	0,87	-0,225	0,114	0,872	0,052	1,507	0,111	1,338	0	3,134	APX1_ARATH	L-ascorbate peroxidase 1, cytosolic (AP) (AtAPx01) (EC 1.11.1.11)	APX1 At1g07890 F24B9.2	Arabidopsis thaliana (Mouse-ear cress)		Alternative splicing; Calcium; Complete proteome; Cytoplasm; Direct protein sequencing; Heme; Hydrogen peroxide; Iron; Metal-binding; Oxidoreductase; Peroxidase; Phosphoprotein; Potassium; Reference proteome
SRR646604_TR28714_c0_g1_i2_len=945	CB23_TOBAC	Q:45-734,H:38-265	92.17%ID	E:1e-151	Chlorophyll a-b binding protein 36, chloroplastic	PF00504.18^Chloroa_b-bind^Chlorophyll A-B binding protein^1-126^E:4e-30	0,099	-0,347	0	-1,9	0	-1,076	0	-0,855	0	1,744	CB23_TOBAC	Chlorophyll a-b binding protein 36, chloroplastic (LHCII type I CAB-36) (LHCP)	CAB36	Nicotiana tabacum (Common tobacco)		Acetylation; Chlorophyll; Chloroplast; Chromophore; Magnesium; Membrane; Metal-binding; Phosphoprotein; Photosynthesis; Photosystem I; Photosystem II; Plastid; Thylakoid; Transit peptide; Transmembrane; Transmembrane helix
SRR646604_TR30519_c0_g2_i1_len=2811	NA	NA	NA	NA	NA	PF00575.20^S1^S1 RNA binding domain^551-621^E:3.7e-11	1	0,056	1	-0,893	1	0,012	1	0,192	0	8,976	.	.	.	.	.	.
SRR646604_TR33779_c0_g1_i1_len=1957	C7D55_HYOMU	Q:1827-403,H:26-497	55.23%ID	E:1e-178	Premnaspirodiene oxygenase	PF00067.19^p450^Cytochrome P450^51-504^E:9.7e-104	1	-0,64	1	0	1	0	1	0,291	0	1,017	C7D55_HYOMU	Premnaspirodiene oxygenase (HPO) (EC 1.14.13.121) (Cytochrome P450 71D55)	CYP71D55	Hyoscyamus muticus (Egyptian henbane)		Heme; Iron; Membrane; Metal-binding; Monooxygenase; NADP; Oxidoreductase; Transmembrane; Transmembrane helix
SRR646604_TR34861_c0_g1_i1_len=1000	NA	NA	NA	NA	NA	.	1	0	1	1,579	1	-3,182	1	-1,67	0	6,969	.	.	.	.	.	.
SRR646604_TR37133_c2_g1_i4_len=1013	POLX_TOBAC	Q:520-1011,H:424-587	46.34%ID	E:1e-58	Retrovirus-related Pol polyprotein from transposon TNT 1-94	.	1	-0,139	1	-1,424	1	-1,439	0,502	-2,58	0	3,717	POLX_TOBAC	Retrovirus-related Pol polyprotein from transposon TNT 1-94 [Includes: Protease (EC 3.4.23.-); Reverse transcriptase (EC 2.7.7.49); Endonuclease]		Nicotiana tabacum (Common tobacco)		Aspartyl protease; Endonuclease; Hydrolase; Metal-binding; Nuclease; Nucleotidyltransferase; Protease; RNA-directed DNA polymerase; Transferase; Transposable element; Zinc; Zinc-finger
SRR648705_TR4879_c1_g1_i1_len=840	POLX_TOBAC	Q:2-292,H:1227-1323	52.58%ID	E:2e-24	Retrovirus-related Pol polyprotein from transposon TNT 1-94	.	1	2,046	1	-0,169	1	0,33	1	0,273	0	6,71	POLX_TOBAC	Retrovirus-related Pol polyprotein from transposon TNT 1-94 [Includes: Protease (EC 3.4.23.-); Reverse transcriptase (EC 2.7.7.49); Endonuclease]		Nicotiana tabacum (Common tobacco)		Aspartyl protease; Endonuclease; Hydrolase; Metal-binding; Nuclease; Nucleotidyltransferase; Protease; RNA-directed DNA polymerase; Transferase; Transposable element; Zinc; Zinc-finger
SRR648705_TR4879_c1_g4_i1_len=506	NA	NA	NA	NA	NA	.	1	0	1	0	1	0	1	0	0	8,93	.	.	.	.	.	.
SRR648705_TR4879_c1_g5_i1_len=956	POLX_TOBAC	Q:882-49,H:943-1218	52.52%ID	E:7e-93	Retrovirus-related Pol polyprotein from transposon TNT 1-94	PF07727.11^RVT_2^Reverse transcriptase (RNA-dependent DNA polymerase)^2-135^E:4.8e-41	1	-1,937	1	-2,373	1	2,049	1	1,191	0	6,242	POLX_TOBAC	Retrovirus-related Pol polyprotein from transposon TNT 1-94 [Includes: Protease (EC 3.4.23.-); Reverse transcriptase (EC 2.7.7.49); Endonuclease]		Nicotiana tabacum (Common tobacco)		Aspartyl protease; Endonuclease; Hydrolase; Metal-binding; Nuclease; Nucleotidyltransferase; Protease; RNA-directed DNA polymerase; Transferase; Transposable element; Zinc; Zinc-finger
SRR648705_TR5530_c0_g1_i1_len=1946	NA	NA	NA	NA	NA	PF05368.10^NmrA^NmrA-like family^20-91^E:2.4e-06`PF01370.18^Epimerase^NAD dependent epimerase/dehydratase family^20-90^E:9.5e-05`PF13460.3^NAD_binding_10^NAD(P)H-binding^24-140^E:1.2e-11`PF08547.9^CIA30^Complex I intermediate-associated protein 30 (CIA30)^154-323^E:1.2e-41`PF13460.3^NAD_binding_10^NAD(P)H-binding^335-435^E:2e-13	0,002	6,396	0,059	1,674	0,6	-0,319	1	0,014	0	2,139	.	.	.	.	.	.
SRR648705_TR6339_c0_g1_i1_len=1235	21KD_DAUCA	Q:869-369,H:28-192	49.7%ID	E:3e-52	21 kDa protein	PF04043.12^PMEI^Plant invertase/pectin methylesterase inhibitor^31-189^E:2.4e-36	0,286	-0,861	0,004	-2,893	1	-0,631	1	-1,616	0	4,871	21KD_DAUCA	21 kDa protein (1.2 protein)		Daucus carota (Wild carrot)		Signal
SRR648705_TR7307_c0_g1_i2_len=657	HMGL_IPONI	Q:615-166,H:1-126	63.33%ID	E:1e-43	HMG1/2-like protein	PF09011.7^HMG_box_2^HMG-box domain^37-109^E:3.4e-15`PF00505.16^HMG_box^HMG (high mobility group) box^40-109^E:1.9e-22	1	-0,08	0,454	-0,393	1	0,239	0,158	1,677	0	5,619	HMGL_IPONI	HMG1/2-like protein		Ipomoea nil (Japanese morning glory) (Pharbitis nil)		DNA-binding; Nucleus
SRR648705_TR13253_c0_g2_i1_len=3955	ALFC2_ARATH	Q:3425-2235,H:1-398	88.72%ID	E:0	Probable fructose-bisphosphate aldolase 2, chloroplastic	PF00274.16^Glycolytic^Fructose-bisphosphate aldolase class-I^53-397^E:2.1e-158	1	0	1	0	1	0	1	0	0	8,077	ALFC2_ARATH	Probable fructose-bisphosphate aldolase 2, chloroplastic (EC 4.1.2.13)	FBA2 At4g38970 F19H22.70	Arabidopsis thaliana (Mouse-ear cress)	PATHWAY: Carbohydrate degradation; glycolysis; D-glyceraldehyde 3-phosphate and glycerone phosphate from D-glucose: step 4/4.	Alternative splicing; Chloroplast; Complete proteome; Glycolysis; Lyase; Methylation; Phosphoprotein; Plastid; Reference proteome; Schiff base; Transit peptide
SRR648705_TR13253_c0_g2_i2_len=3854	ALFC2_ARATH	Q:3324-2134,H:1-398	88.72%ID	E:0	Probable fructose-bisphosphate aldolase 2, chloroplastic	PF00274.16^Glycolytic^Fructose-bisphosphate aldolase class-I^53-397^E:2.1e-158	0,552	-0,233	1	0,031	0	-0,925	0,019	-0,515	0	2,167	ALFC2_ARATH	Probable fructose-bisphosphate aldolase 2, chloroplastic (EC 4.1.2.13)	FBA2 At4g38970 F19H22.70	Arabidopsis thaliana (Mouse-ear cress)	PATHWAY: Carbohydrate degradation; glycolysis; D-glyceraldehyde 3-phosphate and glycerone phosphate from D-glucose: step 4/4.	Alternative splicing; Chloroplast; Complete proteome; Glycolysis; Lyase; Methylation; Phosphoprotein; Plastid; Reference proteome; Schiff base; Transit peptide
SRR648705_TR13379_c0_g1_i3_len=692	RS212_ARATH	Q:180-422,H:1-81	81.48%ID	E:3e-44	40S ribosomal protein S21-2	.	0	-7,167	0,282	-0,271	1	0,174	1	0,232	0	4,454	RS212_ARATH	40S ribosomal protein S21-2	RPS21C At5g27700 T1G16.30	Arabidopsis thaliana (Mouse-ear cress)		Acetylation; Complete proteome; Reference proteome; Ribonucleoprotein; Ribosomal protein
SRR648705_TR14618_c0_g1_i3_len=3093	4CLL7_ARATH	Q:441-2081,H:1-544	68.74%ID	E:0	4-coumarate--CoA ligase-like 7	PF00501.25^AMP-binding^AMP-binding enzyme^36-449^E:1.1e-101`PF13193.3^AMP-binding_C^AMP-binding enzyme C-terminal domain^458-533^E:1.2e-14	0,254	-4,759	1	0	0,009	-6,002	0,009	-6,002	0	1,666	4CLL7_ARATH	4-coumarate--CoA ligase-like 7 (EC 6.2.1.-) (4-coumarate--CoA ligase isoform 6) (At4CL6)	4CLL7 At4g05160 C17L7.80	Arabidopsis thaliana (Mouse-ear cress)		ATP-binding; Complete proteome; Fatty acid biosynthesis; Fatty acid metabolism; Ligase; Lipid biosynthesis; Lipid metabolism; Nucleotide-binding; Oxylipin biosynthesis; Peroxisome; Reference proteome
SRR648705_TR23292_c0_g2_i1_len=833	NA	NA	NA	NA	NA	.	0,254	4,779	0,454	-0,446	0,004	-2,36	0,278	-0,732	0	8,047	.	.	.	.	.	.
SRR648705_TR23308_c0_g3_i3_len=2394	SPDE_COFAR	Q:1383-1979,H:116-316	90.05%ID	E:5e-121	Spermidine synthase	PF01564.14^Spermine_synth^Spermine/spermidine synthase^1-137^E:2.8e-44	0,648	-0,281	0,405	0,327	0,736	-0,156	0,329	-0,4	0	5,42	SPDE_COFAR	Spermidine synthase (SPDSY) (EC 2.5.1.16) (Putrescine aminopropyltransferase)		Coffea arabica (Arabian coffee)	PATHWAY: Amine and polyamine biosynthesis; spermidine biosynthesis; spermidine from putrescine: step 1/1.	Polyamine biosynthesis; Spermidine biosynthesis; Transferase
SRR648705_TR27096_c1_g1_i1_len=652	RS191_ARATH	Q:535-107,H:1-143	86.71%ID	E:2e-88	40S ribosomal protein S19-1	PF01090.16^Ribosomal_S19e^Ribosomal protein S19e^6-140^E:7.4e-59	0	-1,286	0,003	0,996	0,657	0,17	0,53	-0,22	0	1,55	RS191_ARATH	40S ribosomal protein S19-1	RPS19A At3g02080 F1C9.13	Arabidopsis thaliana (Mouse-ear cress)		Complete proteome; Reference proteome; Ribonucleoprotein; Ribosomal protein
SRR648705_TR28356_c0_g1_i4_len=1138	C84A1_ARATH	Q:134-757,H:313-520	82.21%ID	E:2e-110	Cytochrome P450 84A1	PF00067.19^p450^Cytochrome P450^1-188^E:7.6e-62	1	0,142	1	0,506	1	0,111	1	-0,28	0,001	5,666	C84A1_ARATH	Cytochrome P450 84A1 (EC 1.14.-.-) (Ferulate-5-hydroxylase) (F5H)	CYP84A1 FAH1 At4g36220 F23E13.110	Arabidopsis thaliana (Mouse-ear cress)	PATHWAY: Aromatic compound metabolism; phenylpropanoid biosynthesis.	Acetylation; Complete proteome; Heme; Iron; Membrane; Metal-binding; Monooxygenase; Oxidoreductase; Reference proteome; Transmembrane; Transmembrane helix
SRR648705_TR35052_c0_g1_i1_len=1056	PR1_MEDTR	Q:665-255,H:40-173	57.25%ID	E:5e-48	Pathogenesis-related protein PR-1	PF00188.23^CAP^Cysteine-rich secretory protein family^65-185^E:8.4e-24	0,239	1,254	1	-0,8	0,01	-1,157	0	-4,108	0,001	1,713	PR1_MEDTR	Pathogenesis-related protein PR-1	PR-1	Medicago truncatula (Barrel medic) (Medicago tribuloides)		Disulfide bond; Pathogenesis-related protein; Plant defense; Signal
SRR648705_TR37308_c0_g1_i1_len=506	NA	NA	NA	NA	NA	.	0,083	-0,389	0,003	-0,69	0	-0,917	0	-0,948	0	0,925	.	.	.	.	.	.
SRR648705_TR41240_c0_g2_i2_len=1159	VPS29_ARATH	Q:840-277,H:1-188	86.7%ID	E:1e-116	Vacuolar protein sorting-associated protein 29	PF12850.4^Metallophos_2^Calcineurin-like phosphoesterase superfamily domain^4-157^E:2.1e-19	0,798	0,525	1	-0,334	0,56	0,899	0,132	1,524	0	3,735	VPS29_ARATH	Vacuolar protein sorting-associated protein 29 (Protein MAIGO 1) (Vesicle protein sorting 29)	VPS29 MAG1 At3g47810 T23J7.140	Arabidopsis thaliana (Mouse-ear cress)		Complete proteome; Cytoplasm; Endosome; Golgi apparatus; Membrane; Protein transport; Reference proteome; Transport
SRR648705_TR43844_c0_g2_i1_len=1151	TIP21_ARATH	Q:138-875,H:1-247	82.19%ID	E:4e-92	Aquaporin TIP2-1	PF00230.17^MIP^Major intrinsic protein^16-232^E:1.7e-73	1	0,459	1	0	1	0	1	0	0	10,07	TIP21_ARATH	Aquaporin TIP2-1 (Delta-tonoplast intrinsic protein) (Delta-TIP) (Tonoplast intrinsic protein 2-1) (AtTIP2;1) [Cleaved into: Aquaporin TIP2-1, N-terminally processed]	TIP2-1 At3g16240 MYA6.10	Arabidopsis thaliana (Mouse-ear cress)		Acetylation; Complete proteome; Membrane; Reference proteome; Repeat; Transmembrane; Transmembrane helix; Transport; Vacuole
SRR648707_TR693_c0_g1_i2_len=3595	CAPP2_ARATH	Q:580-3483,H:1-963	87.1%ID	E:0	Phosphoenolpyruvate carboxylase 2	PF00311.14^PEPcase^Phosphoenolpyruvate carboxylase^163-968^E:0	0,355	-0,326	0,684	0,221	0	-2,213	0	-1,384	0	1,399	CAPP2_ARATH	Phosphoenolpyruvate carboxylase 2 (AtPPC2) (PEPC 2) (PEPCase 2) (EC 4.1.1.31)	PPC2 At2g42600 F14N22.13	Arabidopsis thaliana (Mouse-ear cress)		Allosteric enzyme; Carbon dioxide fixation; Complete proteome; Cytoplasm; Lyase; Magnesium; Phosphoprotein; Photosynthesis; Reference proteome; Ubl conjugation
SRR648707_TR1124_c1_g1_i2_len=1052	HTOMT_CATRO	Q:32-1051,H:1-338	93.53%ID	E:0	Tabersonine 16-O-methyltransferase	PF08100.8^Dimerisation^Dimerisation domain^37-86^E:4.1e-17`PF00891.15^Methyltransf_2^O-methyltransferase^126-348^E:9.7e-64	1	0	1	0	1	0	1	0	0,001	1,011	HTOMT_CATRO	Tabersonine 16-O-methyltransferase (EC 2.1.1.94)	16OMT	Catharanthus roseus (Madagascar periwinkle) (Vinca rosea)		Cytoplasm; Methyltransferase; S-adenosyl-L-methionine; Transferase
SRR648707_TR6507_c0_g1_i1_len=1675	NA	NA	NA	NA	NA	PF07063.10^DUF1338^Domain of unknown function (DUF1338)^86-377^E:1.5e-72	1	-0,97	1	0,853	1	0,477	1	-0,061	0	1,702	.	.	.	.	.	.
SRR648707_TR11715_c0_g1_i4_len=2028	NA	NA	NA	NA	NA	.	0,593	-0,835	0,002	6,372	0,458	-0,492	0,559	-0,386	0	6,875	.	.	.	.	.	.
SRR648707_TR15037_c0_g1_i4_len=1027	NA	NA	NA	NA	NA	.	1	1,898	1	0	1	0	1	0	0	6,83	.	.	.	.	.	.
SRR648707_TR26479_c0_g1_i1_len=730	ROC1_NICSY	Q:232-678,H:14-150	59.06%ID	E:2e-37	29 kDa ribonucleoprotein A, chloroplastic	PF00076.19^RRM_1^RNA recognition motif. (a.k.a. RRM, RBD, or RNP domain)^101-154^E:7.8e-13`PF00076.19^RRM_1^RNA recognition motif. (a.k.a. RRM, RBD, or RNP domain)^149-178^E:0.00019	0,004	-0,636	0,418	0,206	0,214	-0,467	0,883	0,069	0	0,959	ROC1_NICSY	29 kDa ribonucleoprotein A, chloroplastic (CP29A)		Nicotiana sylvestris (Wood tobacco) (South American tobacco)		Chloroplast; Plastid; RNA-binding; Repeat; Ribonucleoprotein; Transit peptide; mRNA processing
SRR648707_TR26687_c0_g1_i1_len=952	NA	NA	NA	NA	NA	PF00276.17^Ribosomal_L23^Ribosomal protein L23^30-90^E:2.1e-15	1	-0,413	1	0,124	1	-0,767	1	0,538	0	7,112	.	.	.	.	.	.
SRR648707_TR39346_c0_g2_i1_len=1251	DREB3_ARATH	Q:795-277,H:47-221	60.8%ID	E:3e-46	Dehydration-responsive element-binding protein 3	PF00847.17^AP2^AP2 domain^60-110^E:9.6e-13	1	-0,217	1	-2,258	1	0,144	1	-0,224	0	4,084	DREB3_ARATH	Dehydration-responsive element-binding protein 3 (Protein TINY 2)	DREB3 ERF041 TINY2 At5g11590 F15N18.180	Arabidopsis thaliana (Mouse-ear cress)		Activator; Complete proteome; DNA-binding; Ethylene signaling pathway; Nucleus; Reference proteome; Transcription; Transcription regulation
SRR648707_TR41046_c0_g1_i4_len=1798	SPDE_COFAR	Q:374-1315,H:1-316	88.92%ID	E:0	Spermidine synthase	PF01564.14^Spermine_synth^Spermine/spermidine synthase^60-305^E:4.2e-98	0,862	-0,105	0,003	1,005	0,955	-0,025	0,278	-0,362	0	8,889	SPDE_COFAR	Spermidine synthase (SPDSY) (EC 2.5.1.16) (Putrescine aminopropyltransferase)		Coffea arabica (Arabian coffee)	PATHWAY: Amine and polyamine biosynthesis; spermidine biosynthesis; spermidine from putrescine: step 1/1.	Polyamine biosynthesis; Spermidine biosynthesis; Transferase
SRR924147_TR6858_c0_g1_i3_len=1697	KRR1_SCHPO	Q:275-1237,H:3-322	60.87%ID	E:3e-105	KRR1 small subunit processome component homolog	.	1	-0,077	1	3,125	1	-0,767	1	-0,361	0	5,837	KRR1_SCHPO	KRR1 small subunit processome component homolog (KRR-R motif-containing protein 1) (Ribosomal RNA assembly protein mis3)	mis3 krr1 SPBC25B2.05	Schizosaccharomyces pombe (strain 972 / ATCC 24843) (Fission yeast)		Complete proteome; Nucleus; RNA-binding; Reference proteome; Ribonucleoprotein; Ribosome biogenesis; rRNA processing
SRR924147_TR8210_c0_g2_i4_len=508	NA	NA	NA	NA	NA	.	1	1,571	1	-2,903	1	0	1	3,304	0	8,944	.	.	.	.	.	.
SRR924147_TR17555_c0_g1_i4_len=816	R10A3_ARATH	Q:156-758,H:1-201	92.54%ID	E:5e-118	60S ribosomal protein L10a-3	PF00687.18^Ribosomal_L1^Ribosomal protein L1p/L10e family^21-204^E:4.8e-40	1	0,154	0,62	0,502	1	0	0,002	6,283	0	2,266	R10A3_ARATH	60S ribosomal protein L10a-3	RPL10AC At5g22440 MWD9.24	Arabidopsis thaliana (Mouse-ear cress)		Complete proteome; Reference proteome; Ribonucleoprotein; Ribosomal protein
SRR924147_TR23789_c0_g1_i1_len=401	NA	NA	NA	NA	NA	.	0,337	-0,336	0,056	0,937	0,502	-0,325	0,689	0,19	0,001	2,149	.	.	.	.	.	.
SRR924147_TR34790_c0_g1_i1_len=1751	G3PC_SINAL	Q:197-1168,H:1-324	91.05%ID	E:0	Glyceraldehyde-3-phosphate dehydrogenase, cytosolic	PF00044.21^Gp_dh_N^Glyceraldehyde 3-phosphate dehydrogenase, NAD binding domain^6-156^E:1e-55`PF02800.17^Gp_dh_C^Glyceraldehyde 3-phosphate dehydrogenase, C-terminal domain^161-318^E:2.5e-72	1	-1,088	0,502	2,721	0,076	-2,289	0,017	-3,88	0	2,147	G3PC_SINAL	Glyceraldehyde-3-phosphate dehydrogenase, cytosolic (EC 1.2.1.12)	GAPC	Sinapis alba (White mustard) (Brassica hirta)	PATHWAY: Carbohydrate degradation; glycolysis; pyruvate from D-glyceraldehyde 3-phosphate: step 1/5.	Cytoplasm; Glycolysis; NAD; Oxidoreductase; S-nitrosylation
SRR924147_TR36549_c0_g1_i1_len=788	NA	NA	NA	NA	NA	PF02298.14^Cu_bind_like^Plastocyanin-like domain^40-145^E:1.1e-17	1	0	1	-2,339	1	-3,291	1	-3,291	0	7,416	.	.	.	.	.	.
SRR924147_TR37367_c3_g2_i6_len=5723	POLX_TOBAC	Q:95-3433,H:232-1323	43.8%ID	E:0	Retrovirus-related Pol polyprotein from transposon TNT 1-94	PF00098.20^zf-CCHC^Zinc knuckle^31-46^E:0.00067`PF13976.3^gag_pre-integrs^GAG-pre-integrase domain^201-268^E:2.8e-14`PF00665.23^rve^Integrase core domain^284-398^E:1e-25`PF07727.11^RVT_2^Reverse transcriptase (RNA-dependent DNA polymerase)^658-905^E:4.7e-92	1	-0,384	1	-0,291	1	-0,83	1	-1,248	0	6,459	POLX_TOBAC	Retrovirus-related Pol polyprotein from transposon TNT 1-94 [Includes: Protease (EC 3.4.23.-); Reverse transcriptase (EC 2.7.7.49); Endonuclease]		Nicotiana tabacum (Common tobacco)		Aspartyl protease; Endonuclease; Hydrolase; Metal-binding; Nuclease; Nucleotidyltransferase; Protease; RNA-directed DNA polymerase; Transferase; Transposable element; Zinc; Zinc-finger
SRR924148_TR5957_c0_g3_i4_len=2802	PS12A_ARATH	Q:846-2150,H:4-438	80.46%ID	E:0	26S proteasome non-ATPase regulatory subunit 12 homolog A	PF01399.24^PCI^PCI domain^294-403^E:1.3e-17	0,52	0,409	0,745	0,199	0,195	1,314	0,692	-1,139	0	1,798	PS12A_ARATH	26S proteasome non-ATPase regulatory subunit 12 homolog A (26S proteasome regulatory subunit RPN5a) (AtRPN5a) (26S proteasome regulatory subunit p55 homolog A) (Protein EMBRYO DEFECTIVE 2107) (Protein MARIPOSA)	RPN5A EMB2107 MSA At5g09900 MYH9.11	Arabidopsis thaliana (Mouse-ear cress)		Alternative splicing; Coiled coil; Complete proteome; Cytoplasm; Nucleus; Proteasome; Reference proteome
SRR924148_TR8145_c0_g1_i1_len=2036	NA	NA	NA	NA	NA	PF00013.26^KH_1^KH domain^167-226^E:7.1e-10`PF10469.6^AKAP7_NLS^AKAP7 2'5' RNA ligase-like domain^240-525^E:3.1e-72	1	0	1	0	1	0	1	0	0	7,759	.	.	.	.	.	.
SRR924148_TR8145_c0_g1_i2_len=2010	NA	NA	NA	NA	NA	PF00013.26^KH_1^KH domain^130-189^E:6.5e-10`PF10469.6^AKAP7_NLS^AKAP7 2'5' RNA ligase-like domain^203-488^E:2.6e-72	1	-0,847	1	-0,775	1	0	1	0	0	7,546	.	.	.	.	.	.
SRR924148_TR12881_c0_g1_i1_len=1813	DDC_CATRO	Q:1717-218,H:1-500	99.8%ID	E:0	Aromatic-L-amino-acid decarboxylase	PF00282.16^Pyridoxal_deC^Pyridoxal-dependent decarboxylase conserved domain^88-462^E:1e-117	1	0,342	1	1,504	1	0,764	1	-0,743	0	0,815	DDC_CATRO	Aromatic-L-amino-acid decarboxylase (AADC) (EC 4.1.1.28) (DOPA decarboxylase) (Tryptophan decarboxylase)	TDC	Catharanthus roseus (Madagascar periwinkle) (Vinca rosea)		Decarboxylase; Lyase; Pyridoxal phosphate
SRR924148_TR24540_c2_g1_i3_len=2477	MORC3_HUMAN	Q:1987-836,H:16-382	36.32%ID	E:2e-54	MORC family CW-type zinc finger protein 3	PF13589.3^HATPase_c_3^Histidine kinase-, DNA gyrase B-, and HSP90-like ATPase^179-272^E:5.3e-15	1	0,367	1	0,485	1	-0,788	1	-0,447	0,001	2,647	MORC3_HUMAN	MORC family CW-type zinc finger protein 3 (Zinc finger CW-type coiled-coil domain protein 3)	MORC3 KIAA0136 ZCWCC3	Homo sapiens (Human)		3D-structure; Coiled coil; Complete proteome; Isopeptide bond; Metal-binding; Phosphoprotein; Reference proteome; Ubl conjugation; Zinc; Zinc-finger
SRR924148_TR31052_c0_g1_i2_len=1340	NA	NA	NA	NA	NA	PF05004.10^IFRD^Interferon-related developmental regulator (IFRD)^36-343^E:4.6e-85	1	-0,256	1	-2,928	0,059	-0,987	0,352	-0,518	0	6,984	.	.	.	.	.	.
SRR924148_TR32136_c0_g1_i1_len=1524	PER42_ARATH	Q:1365-376,H:1-330	79.7%ID	E:0	Peroxidase 42	PF00141.20^peroxidase^Peroxidase^48-291^E:4.1e-60	0,219	-0,273	0	-2,176	0,492	-0,189	0,007	-0,786	0	0,948	PER42_ARATH	Peroxidase 42 (Atperox P42) (EC 1.11.1.7) (ATP1a/ATP1b) (PRXR1)	PER42 P42 At4g21960 F1N20.3 T8O5.170	Arabidopsis thaliana (Mouse-ear cress)		Biological rhythms; Calcium; Complete proteome; Disulfide bond; Glycoprotein; Heme; Hydrogen peroxide; Iron; Metal-binding; Oxidoreductase; Peroxidase; Reference proteome; Secreted; Signal
SRR924148_TR35408_c0_g1_i2_len=1037	SMD1_DROME	Q:342-614,H:1-91	71.43%ID	E:1e-38	Probable small nuclear ribonucleoprotein Sm D1	PF01423.19^LSM^LSM domain^5-69^E:1.5e-18	0,009	6,037	0,132	1,522	1	0,379	0,76	0,681	0,001	6,557	SMD1_DROME	Probable small nuclear ribonucleoprotein Sm D1 (Sm-D1) (snRNP core protein D1)	SmD1 snRNP69D CG10753	Drosophila melanogaster (Fruit fly)		Complete proteome; Methylation; Nucleus; Reference proteome; Repeat; Ribonucleoprotein; mRNA processing; mRNA splicing

Ms6h

Transcript (CDF97)	Uniprot homolog	BLASTX Position	BLASTX %Identity	BLASTX evalue	Uniprot descr	Pfam	PValue_Ms6h	logFC_Ms6h	PValue_Ms8h	logFC_Ms8h	PValue_MsDamaged24h	logFC_MsDamaged24h	PValue_MsIntact24h	logFC_MsIntact24h	PValue_Ms1wk	logFC_Ms1wk	Entry.name	Protein names	Gene names	Organism	Pathway	Keywords
SRR1144633_TR13606_c0_g2_i1_len=6994	NA	NA	NA	NA	NA	PF07529.10^HSA^HSA^575-656^E:1.4e-06`PF00249.28^Myb_DNA-binding^Myb-like DNA-binding domain^1045-1093^E:3.6e-06`PF13921.3^Myb_DNA-bind_6^Myb-like DNA-binding domain^1046-1093^E:3.1e-06	0	6,856	0,034	5,405	0,319	0,898	0,228	1,023	1	0,298	.	.	.	.	.	.
SRR1144633_TR17733_c0_g1_i2_len=3511	NA	NA	NA	NA	NA	.	0	6,903	0,009	5,944	0,129	-4,896	0,129	-4,644	1	-0,166	.	.	.	.	.	.
SRR1144633_TR21914_c0_g1_i2_len=4936	YCX91_PHAAO	Q:1288-1560,H:1-91	97.8%ID	E:1e-52	Uncharacterized protein ORF91	.	0	0,884	0,984	-0,005	0,01	-0,534	0,017	-0,497	0,155	-1,149	YCX91_PHAAO	Uncharacterized protein ORF91		Phalaenopsis aphrodite subsp. formosana (Moth orchid)		Chloroplast; Plastid
SRR1144633_TR22053_c0_g1_i2_len=1609	CYSP3_SOLLC	Q:310-1248,H:44-356	81.15%ID	E:0	Cysteine proteinase 3	PF00112.20^Peptidase_C1^Papain family cysteine protease^53-268^E:7.3e-79	0	2,194	0,032	0,518	0,083	0,782	0,148	0,665	0,075	0,5	CYSP3_SOLLC	Cysteine proteinase 3 (EC 3.4.22.-)	CYP-3	Solanum lycopersicum (Tomato) (Lycopersicon esculentum)		Complete proteome; Disulfide bond; Glycoprotein; Hydrolase; Protease; Reference proteome; Signal; Thiol protease; Vacuole; Zymogen
SRR1144633_TR32090_c0_g1_i1_len=633	CYT5_ARATH	Q:454-179,H:22-114	59.14%ID	E:5e-33	Cysteine proteinase inhibitor 5	PF00031.18^Cystatin^Cystatin domain^34-119^E:4.4e-15`PF16845.2^SQAPI^Aspartic acid proteinase inhibitor^39-119^E:7.2e-37`PF07430.8^PP1^Phloem filament protein PP1 cystatin-like domain^47-115^E:0.00013	0	1,25	0,071	0,523	0,417	0,191	0,783	0,066	1	0	CYT5_ARATH	Cysteine proteinase inhibitor 5 (AtCYS-5)	CYS5 At5g47550 MNJ7.14	Arabidopsis thaliana (Mouse-ear cress)		Complete proteome; Glycoprotein; Plant defense; Protease inhibitor; Reference proteome; Secreted; Signal; Thiol protease inhibitor
SRR1144634_TR13321_c0_g1_i3_len=881	NA	NA	NA	NA	NA	.	0	7,031	0,017	5,735	0,066	-5,324	0,066	-5,324	1	-0,671	.	.	.	.	.	.
SRR1144634_TR40497_c0_g2_i1_len=540	NA	NA	NA	NA	NA	.	0	6,674	0	-8,674	1	0	0,129	5,12	0,005	-6,183	.	.	.	.	.	.
SRR122237_TR19703_c0_g1_i1_len=876	NA	NA	NA	NA	NA	.	0	2,56	0,39	-0,193	0	-1,572	0	-1,033	0,001	0,939	.	.	.	.	.	.
SRR122238_TR5874_c0_g1_i8_len=812	NA	NA	NA	NA	NA	.	0	6,781	0,394	-0,494	0,057	1,381	0,381	0,78	1	0	.	.	.	.	.	.
SRR122238_TR41053_c1_g4_i10_len=623	NA	NA	NA	NA	NA	.	0	1,834	0,922	0,021	0,175	-0,281	0,254	-0,236	0,716	-0,081	.	.	.	.	.	.
SRR122238_TR71685_c0_g1_i2_len=532	NA	NA	NA	NA	NA	.	0	2,043	0,858	-0,087	0,294	0,279	0,597	0,146	0,471	0,564	.	.	.	.	.	.
SRR122238_TR72446_c0_g1_i1_len=1168	SWET3_ARATH	Q:128-775,H:1-216	62.04%ID	E:1e-93	Bidirectional sugar transporter SWEET3 {ECO:0000303|PubMed:21107422}	PF03083.13^MtN3_slv^Sugar efflux transporter for intercellular exchange^9-95^E:5.6e-16`PF03083.13^MtN3_slv^Sugar efflux transporter for intercellular exchange^133-216^E:1.6e-24	0,001	3,553	0,003	2,555	0,188	0,71	0,049	0,987	0,258	-0,996	SWET3_ARATH	Bidirectional sugar transporter SWEET3 (AtSWEET3) (Protein SUGARS WILL EVENTUALLY BE EXPORTED TRANSPORTERS 3)	SWEET3 At5g53190 MFH8.13	Arabidopsis thaliana (Mouse-ear cress)		Cell membrane; Complete proteome; Membrane; Reference proteome; Repeat; Sugar transport; Transmembrane; Transmembrane helix; Transport
SRR122238_TR73472_c0_g1_i1_len=648	NA	NA	NA	NA	NA	.	0	3,254	0,121	0,33	0,903	-0,027	0,858	0,042	0,822	0,363	.	.	.	.	.	.
SRR122238_TR75334_c0_g1_i3_len=856	CCB12_ORYSJ	Q:132-473,H:1-114	45.22%ID	E:3e-22	Cyclin-B1-2	PF05348.8^UMP1^Proteasome maturation factor UMP1^59-172^E:9.5e-30	0	1,927	0,026	-1,162	0,002	1,44	0,121	0,812	0,241	-0,388	CCB12_ORYSJ	Cyclin-B1-2 (G2/mitotic-specific cyclin-B1-2) (CycB1;2)	CYCB1-2 Os02g0801300 LOC_Os02g55720	Oryza sativa subsp. japonica (Rice)		Cell cycle; Cell division; Complete proteome; Cyclin; Reference proteome
SRR1271857_TR5020_c0_g1_i2_len=2456	RAP27_ARATH	Q:501-1841,H:1-406	46.09%ID	E:1e-88	Ethylene-responsive transcription factor RAP2-7	.	0,001	1,337	0,009	1,003	1	0,057	0,5	-0,732	0,407	-0,481	RAP27_ARATH	Ethylene-responsive transcription factor RAP2-7 (Protein RELATED TO APETALA2 7) (Protein TARGET OF EAT 1)	RAP2-7 TOE1 At2g28550 T17D12.11	Arabidopsis thaliana (Mouse-ear cress)		Activator; Alternative splicing; Complete proteome; DNA-binding; Ethylene signaling pathway; Nucleus; Reference proteome; Repeat; Transcription; Transcription regulation
SRR1271857_TR8332_c0_g1_i4_len=2679	NA	NA	NA	NA	NA	PF03547.15^Mem_trans^Membrane transport protein^11-155^E:5.2e-27	0	2,715	0,051	0,786	0,026	2,165	1	0,472	0,005	0,868	.	.	.	.	.	.
SRR1271857_TR23195_c2_g1_i16_len=1602	NA	NA	NA	NA	NA	.	0	2,506	0,763	0,065	0,175	0,289	0,101	0,35	0,664	0,101	.	.	.	.	.	.
SRR1271857_TR26787_c0_g1_i1_len=787	NA	NA	NA	NA	NA	.	0	6,87	0,003	-1,467	0,066	-5,35	0,732	-0,546	0,719	0,268	.	.	.	.	.	.
SRR1271857_TR32088_c1_g1_i1_len=3371	PHSL2_SOLTU	Q:2992-251,H:54-974	82.36%ID	E:0	Alpha-1,4 glucan phosphorylase L-2 isozyme, chloroplastic/amyloplastic	PF00343.17^Phosphorylase^Carbohydrate phosphorylase^179-970^E:7.1e-299	0	1,121	0,018	0,693	0,472	0,774	0,045	1,507	0,273	0,392	PHSL2_SOLTU	Alpha-1,4 glucan phosphorylase L-2 isozyme, chloroplastic/amyloplastic (EC 2.4.1.1) (Starch phosphorylase L-2)	STP-1	Solanum tuberosum (Potato)		Allosteric enzyme; Amyloplast; Carbohydrate metabolism; Chloroplast; Complete proteome; Glycosyltransferase; Plastid; Pyridoxal phosphate; Reference proteome; Transferase; Transit peptide
SRR1271858_TR54792_c0_g1_i5_len=834	NA	NA	NA	NA	NA	.	0	2,492	0,312	0,764	0,046	0,882	0,046	0,874	0,497	-0,234	.	.	.	.	.	.
SRR1271859_TR7049_c0_g1_i1_len=2169	TLP3_ARATH	Q:1510-353,H:30-406	62.5%ID	E:2e-162	Tubby-like F-box protein 3	PF00646.30^F-box^F-box domain^28-82^E:2.4e-06`PF01167.15^Tub^Tub family^101-383^E:5.9e-88	0	2,222	0,001	6,411	0,692	1,256	1	0,921	0,034	5,675	TLP3_ARATH	Tubby-like F-box protein 3 (AtTLP3)	TULP3 At2g47900 F17A22.29	Arabidopsis thaliana (Mouse-ear cress)		Alternative splicing; Cell membrane; Complete proteome; Cytoplasm; Membrane; Nucleus; Plastid; Reference proteome; Stress response
SRR1271859_TR28483_c1_g1_i1_len=2056	U74B1_ARATH	Q:1952-588,H:1-458	54.98%ID	E:2e-163	UDP-glycosyltransferase 74B1	PF00201.15^UDPGT^UDP-glucoronosyl and UDP-glucosyl transferase^251-417^E:3e-30	0	5,974	0,129	2,774	0,254	2,9	0,502	2,362	1	-1,004	U74B1_ARATH	UDP-glycosyltransferase 74B1 (EC 2.4.1.-) (N-hydroxythioamide S-beta-glucosyltransferase) (EC 2.4.1.195) (Thiohydroximate S-glucosyltransferase)	UGT74B1 At1g24100 F3I6.2	Arabidopsis thaliana (Mouse-ear cress)		Alternative splicing; Complete proteome; Glycosyltransferase; Reference proteome; Transferase
SRR1271859_TR32386_c6_g2_i1_len=1517	NA	NA	NA	NA	NA	.	0	1,484	0,117	0,335	0	-1,273	0	-1,148	0,75	-0,077	.	.	.	.	.	.
SRR1271859_TR33997_c0_g1_i1_len=1019	ACP1_CASGL	Q:168-578,H:1-136	67.88%ID	E:1e-48	Acyl carrier protein 1, chloroplastic	PF00550.22^PP-binding^Phosphopantetheine attachment site^61-128^E:6.3e-10	0	3,249	0,005	6,141	0,095	0,561	0,003	0,936	0,006	-0,745	ACP1_CASGL	Acyl carrier protein 1, chloroplastic (ACP 1)	ACP1	Casuarina glauca (Swamp oak)	PATHWAY: Lipid metabolism; fatty acid biosynthesis.	Chloroplast; Fatty acid biosynthesis; Fatty acid metabolism; Lipid biosynthesis; Lipid metabolism; Phosphopantetheine; Phosphoprotein; Plastid; Transit peptide
SRR1271859_TR36251_c0_g1_i2_len=1055	NA	NA	NA	NA	NA	.	0	2,081	0,712	0,216	0,836	0,074	0,915	-0,037	0,195	0,831	.	.	.	.	.	.
SRR1271859_TR37059_c0_g2_i1_len=561	NA	NA	NA	NA	NA	.	0	7,205	1	0,756	0,003	-2,06	0,025	-1,436	1	-0,024	.	.	.	.	.	.
SRR1271859_TR44596_c0_g3_i2_len=2228	BAM9_ARATH	Q:248-1915,H:1-521	51.24%ID	E:5e-171	Inactive beta-amylase 9	PF01373.14^Glyco_hydro_14^Glycosyl hydrolase family 14^13-210^E:1.6e-34	0,001	2,846	1	-0,47	0,298	0,775	0,848	0,334	0,124	-0,968	BAM9_ARATH	Inactive beta-amylase 9 (1,4-alpha-D-glucan maltohydrolase) (Inactive beta-amylase 3)	BAM9 BMY3 At5g18670 T1A4.50	Arabidopsis thaliana (Mouse-ear cress)		Carbohydrate metabolism; Complete proteome; Cytoplasm; Phosphoprotein; Polysaccharide degradation; Reference proteome
SRR1271859_TR45551_c1_g3_i3_len=2426	NA	NA	NA	NA	NA	.	0	1,19	0,399	-0,182	0,184	-0,288	0,46	-0,16	0,9	0,031	.	.	.	.	.	.
SRR342017_TR6268_c0_g4_i2_len=1128	WRK70_ARATH	Q:134-724,H:7-187	38.65%ID	E:3e-33	Probable WRKY transcription factor 70	PF03106.12^WRKY^WRKY DNA -binding domain^174-234^E:2.3e-23	0	3,227	0,001	4,394	0,282	1,234	0,56	0,852	1	-0,171	WRK70_ARATH	Probable WRKY transcription factor 70 (WRKY DNA-binding protein 70)	WRKY70 At3g56400 T5P19.50	Arabidopsis thaliana (Mouse-ear cress)		Complete proteome; DNA-binding; Nucleus; Reference proteome; Transcription; Transcription regulation
SRR342017_TR8859_c1_g4_i5_len=1394	10HGO_CATRO	Q:1256-198,H:3-359	62.18%ID	E:7e-119	8-hydroxygeraniol dehydrogenase	PF08240.9^ADH_N^Alcohol dehydrogenase GroES-like domain^82-190^E:5e-21`PF00107.23^ADH_zinc_N^Zinc-binding dehydrogenase^234-357^E:1.7e-16	0,001	0,827	0,003	1	0,77	-0,194	0,221	0,609	0,385	0,254	10HGO_CATRO	8-hydroxygeraniol dehydrogenase (Cr10HGO) (EC 1.1.1.324)	10HGO	Catharanthus roseus (Madagascar periwinkle) (Vinca rosea)		Metal-binding; NADP; Oxidoreductase; Zinc
SRR342017_TR11921_c0_g2_i1_len=693	SLU7A_ARATH	Q:642-25,H:20-225	81.55%ID	E:5e-120	Pre-mRNA-splicing factor SLU7-A	PF14392.3^zf-CCHC_4^Zinc knuckle^93-107^E:0.066`PF11708.5^Slu7^Pre-mRNA splicing Prp18-interacting factor^134-224^E:4.7e-15	0	7,041	1	0	0,005	-6,203	0,005	-6,203	1	0	SLU7A_ARATH	Pre-mRNA-splicing factor SLU7-A	At1g65660 F1E22.4 F5I14.19	Arabidopsis thaliana (Mouse-ear cress)		Complete proteome; Metal-binding; Nucleus; Phosphoprotein; Reference proteome; Spliceosome; Zinc; Zinc-finger; mRNA processing; mRNA splicing
SRR342017_TR19797_c0_g2_i1_len=818	KPK2_ARATH	Q:9-800,H:111-358	66.04%ID	E:1e-115	Serine/threonine-protein kinase AtPK2/AtPK19	PF00069.22^Pkinase^Protein kinase domain^39-249^E:2.1e-64`PF07714.14^Pkinase_Tyr^Protein tyrosine kinase^40-246^E:1.4e-36`PF14531.3^Kinase-like^Kinase-like^151-248^E:1e-09	0	6,798	1	0	1	-3,317	0,025	2,809	0,502	4,019	KPK2_ARATH	Serine/threonine-protein kinase AtPK2/AtPK19 (EC 2.7.11.1) (Ribosomal-protein S6 kinase homolog 2)	ATPK2 ATPK19 S6K2 At3g08720 F17O14.19	Arabidopsis thaliana (Mouse-ear cress)		ATP-binding; Complete proteome; Kinase; Nucleotide-binding; Phosphoprotein; Reference proteome; Serine/threonine-protein kinase; Transferase
SRR342019_TR1632_c1_g2_i1_len=1652	SUBL_ARATH	Q:1612-386,H:351-757	57.18%ID	E:5e-148	.	PF00082.19^Peptidase_S8^Subtilase family^32-246^E:2.5e-23`PF02225.19^PA^PA domain^60-121^E:1e-09	0	8,224	0,176	-0,429	0,356	0,397	0,004	-1,413	1	0	SBT17_ARATH	Subtilisin-like protease SBT1.7 (EC 3.4.21.-) (Cucumisin-like serine protease) (Subtilase subfamily 1 member 7) (AtSBT1.7) (Subtilisin-like serine protease 1) (At-SLP1)	SBT1.7 ARA12 ASP48 SLP1 At5g67360 K8K14.8	Arabidopsis thaliana (Mouse-ear cress)		Calcium; Cell wall; Complete proteome; Direct protein sequencing; Glycoprotein; Hydrolase; Protease; Reference proteome; Secreted; Serine protease; Signal; Zymogen
SRR342019_TR7401_c4_g2_i1_len=6090	PUR4_ARATH	Q:5873-2313,H:9-1192	78.69%ID	E:0	Probable phosphoribosylformylglycinamidine synthase, chloroplastic/mitochondrial	PF00586.21^AIRS^AIR synthase related protein, N-terminal domain^393-495^E:0.021`PF02769.19^AIRS_C^AIR synthase related protein, C-terminal domain^535-689^E:2.2e-21`PF02769.19^AIRS_C^AIR synthase related protein, C-terminal domain^945-1075^E:3.5e-16`PF13507.3^GATase_5^CobB/CobQ-like glutamine amidotransferase domain^1143-1196^E:2.4e-15	0	1,559	0,882	0,059	0,694	0,217	0,626	0,233	0,564	0,418	PUR4_ARATH	Probable phosphoribosylformylglycinamidine synthase, chloroplastic/mitochondrial (FGAM synthase) (FGAMS) (EC 6.3.5.3) (Formylglycinamide ribonucleotide amidotransferase) (FGAR amidotransferase) (FGAR-AT) (Formylglycinamide ribotide amidotransferase)	At1g74260 F1O17.7	Arabidopsis thaliana (Mouse-ear cress)	PATHWAY: Purine metabolism; IMP biosynthesis via de novo pathway; 5-amino-1-(5-phospho-D-ribosyl)imidazole from N(2)-formyl-N(1)-(5-phospho-D-ribosyl)glycinamide: step 1/2.	ATP-binding; Chloroplast; Complete proteome; Glutamine amidotransferase; Ligase; Magnesium; Metal-binding; Mitochondrion; Nucleotide-binding; Plastid; Purine biosynthesis; Reference proteome; Transit peptide
SRR342019_TR7886_c0_g1_i8_len=636	NA	NA	NA	NA	NA	PF01190.14^Pollen_Ole_e_I^Pollen proteins Ole e I like^29-110^E:1.2e-15	0	8,648	1	0	1	0	1	0	0,146	0,393	.	.	.	.	.	.
SRR342019_TR32367_c4_g5_i5_len=4034	NA	NA	NA	NA	NA	.	0	4,989	1	2,691	1	-0,327	0,781	-0,546	1	-1,17	.	.	.	.	.	.
SRR342019_TR38712_c0_g1_i2_len=768	NA	NA	NA	NA	NA	PF00304.17^Gamma-thionin^Gamma-thionin family^53-99^E:2.5e-16	0	0,792	0,121	-0,489	1	0,225	0,502	-2,065	0,712	0,08	.	.	.	.	.	.
SRR342022_TR641_c3_g5_i4_len=549	NA	NA	NA	NA	NA	.	0,001	1,321	0,563	-0,224	0,452	-0,613	0,235	-0,812	0,415	-0,365	.	.	.	.	.	.
SRR342022_TR737_c0_g1_i1_len=913	DNJ11_ARATH	Q:288-653,H:40-161	47.69%ID	E:1e-30	Chaperone protein dnaJ 11, chloroplastic	PF00226.28^DnaJ^DnaJ domain^58-125^E:4.1e-20	0,001	6,645	0,258	1,094	0,034	5,684	1	0	1	0	DNJ11_ARATH	Chaperone protein dnaJ 11, chloroplastic (AtDjC11) (AtJ11)	ATJ11 C11 J11 At4g36040 T19K4.170	Arabidopsis thaliana (Mouse-ear cress)		Chaperone; Chloroplast; Complete proteome; Direct protein sequencing; Plastid; Reference proteome; Transit peptide
SRR342022_TR9376_c1_g1_i1_len=5851	YCX91_PHAAO	Q:223-495,H:1-91	97.8%ID	E:2e-52	Uncharacterized protein ORF91	.	0	1,288	0,635	-0,103	0,239	-0,259	0,202	-0,28	0,581	-0,505	YCX91_PHAAO	Uncharacterized protein ORF91		Phalaenopsis aphrodite subsp. formosana (Moth orchid)		Chloroplast; Plastid

SRR342022_TR9794_c0_g20_i1_len=1639	STSY_RAUSE	Q:819-1544,H:83-324	40.91%ID	E:4e-59	Strictosidine synthase	PF03088.13^Str_synth^Strictosidine synthase^73-160^E:1.5e-25	0,001	1,704	0,103	1,523	0,692	0,563	1	-0,471	1	0,026	STSY_RAUSE	Strictosidine synthase (EC 4.3.3.2)	STR1	Rauvolfia serpentina (Serpentine wood) (Ophioxylon serpentinum)	PATHWAY: Alkaloid biosynthesis; 3alpha(S)-strictosidine biosynthesis; 3alpha(S)-strictosidine from secologanin and tryptamine: step 1/1.	3D-structure; Alkaloid metabolism; Direct protein sequencing; Glycoprotein; Lyase; Signal; Vacuole
SRR342022_TR9794_c0_g20_i3_len=1230	STSY_RAUMA	Q:278-1135,H:37-322	43.71%ID	E:1e-81	Strictosidine synthase	PF03088.13^Str_synth^Strictosidine synthase^177-264^E:3e-25	0	1,31	0,002	1,389	1	-0,037	0,076	-1,503	0,938	0,04	STSY_RAUMA	Strictosidine synthase (EC 4.3.3.2) (Fragment)	STR1	Rauvolfia mannii	PATHWAY: Alkaloid biosynthesis; 3alpha(S)-strictosidine biosynthesis; 3alpha(S)-strictosidine from secologanin and tryptamine: step 1/1.	Alkaloid metabolism; Glycoprotein; Lyase; Signal; Vacuole
SRR342022_TR22888_c2_g4_i1_len=1175	14335_SOLTU	Q:1081-317,H:1-256	93.75%ID	E:3e-172	14-3-3-like protein 16R	PF00244.17^14-3-3^14-3-3 protein^32-257^E:7.4e-105	0	3,144	0,029	-0,567	0,111	-0,519	0,004	-0,958	0,003	-0,77	14335_SOLTU	14-3-3-like protein 16R		Solanum tuberosum (Potato)		Complete proteome; Reference proteome
SRR342022_TR26103_c2_g5_i1_len=1165	NA	NA	NA	NA	NA	.	0	4,122	0,402	0,979	0,002	2,443	0,026	1,901	0,949	0,033	.	.	.	.	.	.
SRR342022_TR26348_c3_g6_i5_len=717	MECR_ARATH	Q:58-525,H:220-375	78.21%ID	E:3e-81	Probable trans-2-enoyl-CoA reductase, mitochondrial	.	0,001	6,615	0,623	-0,38	0,076	1,397	0,44	0,767	0,003	-1,47	MECR_ARATH	Probable trans-2-enoyl-CoA reductase, mitochondrial (EC 1.3.1.38)	At3g45770 T6D9.100	Arabidopsis thaliana (Mouse-ear cress)		Alternative splicing; Complete proteome; Fatty acid biosynthesis; Fatty acid metabolism; Lipid biosynthesis; Lipid metabolism; Mitochondrion; NADP; Oxidoreductase; Reference proteome; Transit peptide
SRR342023_TR2846_c0_g5_i2_len=1227	ABP20_PRUPE	Q:258-782,H:28-202	69.89%ID	E:3e-72	Auxin-binding protein ABP20	PF00190.19^Cupin_1^Cupin^54-199^E:4.1e-32	0	1,428	0	-3,732	0	-2,648	0	-1,69	0,615	0,105	ABP20_PRUPE	Auxin-binding protein ABP20	ABP20	Prunus persica (Peach) (Amygdalus persica)		Apoplast; Auxin signaling pathway; Cell wall; Disulfide bond; Glycoprotein; Manganese; Metal-binding; Receptor; Secreted; Signal
SRR342023_TR17123_c5_g1_i3_len=829	NA	NA	NA	NA	NA	.	0	1,079	0,724	-0,199	1	-0,424	0,381	-1,563	1	0	.	.	.	.	.	.
SRR342023_TR22890_c2_g1_i3_len=708	TBL21_ARATH	Q:634-5,H:179-391	62.91%ID	E:2e-100	Protein trichome birefringence-like 21	PF14416.3^PMR5N^PMR5 N terminal Domain^12-64^E:1.6e-22`PF13839.3^PC-Esterase^GDSL/SGNH-like Acyl-Esterase family found in Pmr5 and Cas1p^65-216^E:2.4e-44	0	6,951	0,282	1,239	0,564	-0,477	0,312	-0,817	1	0	TBL21_ARATH	Protein trichome birefringence-like 21	TBL21 At5g15890 F1N13.30	Arabidopsis thaliana (Mouse-ear cress)		Complete proteome; Membrane; Reference proteome; Signal-anchor; Transmembrane; Transmembrane helix
SRR342023_TR27467_c3_g4_i8_len=1394	NA	NA	NA	NA	NA	PF06108.9^DUF952^Protein of unknown function (DUF952)^19-54^E:7.6e-08	0	1,571	0	-2,315	0,132	0,762	0,496	0,388	1	-2,002	.	.	.	.	.	.
SRR646572_TR24195_c1_g6_i3_len=5788	NU5C_COFAR	Q:4474-2276,H:1-733	85.68%ID	E:0	NAD(P)H-quinone oxidoreductase subunit 5, chloroplastic	PF00662.17^Proton_antipo_N^NADH-Ubiquinone oxidoreductase (complex I), chain 5 N-terminus^72-129^E:5e-20`PF00361.17^Proton_antipo_M^Proton-conducting membrane transporter^141-442^E:1.9e-96`PF01010.16^Proton_antipo_C^NADH-dehyrogenase subunit F, TMs, (complex I) C-terminus^448-688^E:2.7e-112	0	1,62	0,927	0,099	0,203	-0,597	0,203	-0,581	0,363	-0,277	NU5C_COFAR	NAD(P)H-quinone oxidoreductase subunit 5, chloroplastic (EC 1.6.5.-) (NAD(P)H dehydrogenase subunit 5) (NADH-plastoquinone oxidoreductase subunit 5)	ndhF	Coffea arabica (Arabian coffee)		Chloroplast; Membrane; NAD; NADP; Oxidoreductase; Plastid; Plastoquinone; Quinone; Thylakoid; Transmembrane; Transmembrane helix; Transport
SRR646572_TR30031_c0_g1_i2_len=1681	NA	NA	NA	NA	NA	.	0	4,175	0,594	0,124	0,012	0,612	0,002	0,752	0,423	-0,174	.	.	.	.	.	.
SRR646572_TR30268_c0_g1_i1_len=737	NA	NA	NA	NA	NA	.	0	8,205	0	-10,998	1	0	1	0	1	0	.	.	.	.	.	.
SRR646596_TR11266_c4_g7_i3_len=553	NA	NA	NA	NA	NA	.	0	3,2	0,579	-0,117	0,745	0,071	0,383	0,187	0,708	0,192	.	.	.	.	.	.
SRR646596_TR32001_c0_g1_i1_len=2993	SIR1_TOBAC	Q:187-2271,H:1-691	82.21%ID	E:0	Sulfite reductase 1 [ferredoxin], chloroplastic	PF03460.14^NIR_SIR_ferr^Nitrite/Sulfite reductase ferredoxin-like half domain^195-252^E:2e-15`PF01077.19^NIR_SIR^Nitrite and sulphite reductase 4Fe-4S domain^289-467^E:2.1e-50`PF03460.14^NIR_SIR_ferr^Nitrite/Sulfite reductase ferredoxin-like half domain^485-538^E:6.1e-13`PF01077.19^NIR_SIR^Nitrite and sulphite reductase 4Fe-4S domain^563-701^E:1.2e-09	0	2,577	0,321	-0,289	0,339	-0,32	0,708	0,123	0,695	0,125	SIR1_TOBAC	Sulfite reductase 1 [ferredoxin], chloroplastic (NtSiR1) (EC 1.8.7.1)	SIR1	Nicotiana tabacum (Common tobacco)		4Fe-4S; Chloroplast; DNA-binding; Direct protein sequencing; Heme; Iron; Iron-sulfur; Metal-binding; Oxidoreductase; Plastid; Thioether bond; Transit peptide
SRR646604_TR17021_c2_g3_i3_len=2306	NA	NA	NA	NA	NA	.	0	1,632	1	-0,003	0,776	0,081	0,799	-0,092	0,29	-0,399	.	.	.	.	.	.
SRR646604_TR26908_c0_g1_i1_len=551	NA	NA	NA	NA	NA	.	0	1,357	0,129	0,4	0,045	0,505	0,004	0,724	0,001	-0,946	.	.	.	.	.	.
SRR648705_TR118_c0_g1_i1_len=935	NA	NA	NA	NA	NA	.	0	1,086	0,008	0,606	0,254	4,414	1	2,551	0,049	0,468	.	.	.	.	.	.
SRR648705_TR17279_c0_g2_i8_len=2030	TLP8_ORYSJ	Q:1602-313,H:1-445	73.66%ID	E:0	Tubby-like F-box protein 8	PF00646.30^F-box^F-box domain^52-105^E:7.7e-11`PF12937.4^F-box-like^F-box-like^53-95^E:1.2e-06`PF01167.15^Tub^Tub family^117-424^E:7.4e-97	0	6,996	0,03	-1,073	0,103	1,709	0,356	1,251	1	-0,398	TLP8_ORYSJ	Tubby-like F-box protein 8 (OsTLP8) (Tubby-like F-box protein 1) (OsTLP1)	TULP8 TULP1 Os05g0437900 LOC_Os05g36190 OJ1058_F05.11 OsJ_017918 OSJNBb0042J17.6	Oryza sativa subsp. japonica (Rice)		Complete proteome; Reference proteome
SRR648705_TR17428_c0_g2_i1_len=1299	SWT17_ARATH	Q:310-999,H:1-241	60.17%ID	E:1e-82	Bidirectional sugar transporter SWEET17 {ECO:0000303|PubMed:21107422}	PF03083.13^MtN3_slv^Sugar efflux transporter for intercellular exchange^7-92^E:2.6e-26`PF03083.13^MtN3_slv^Sugar efflux transporter for intercellular exchange^128-212^E:1.8e-25	0	1,755	0,057	1,42	0,282	0,398	0,562	-0,268	0,211	-0,466	SWT17_ARATH	Bidirectional sugar transporter SWEET17 (AtSWEET17) (Protein SUGARS WILL EVENTUALLY BE EXPORTED TRANSPORTERS 17)	SWEET17 At4g15920 dl4000c FCAALL.237	Arabidopsis thaliana (Mouse-ear cress)		Complete proteome; Membrane; Reference proteome; Repeat; Sugar transport; Transmembrane; Transmembrane helix; Transport; Vacuole
SRR648705_TR18459_c0_g2_i1_len=609	NA	NA	NA	NA	NA	.	0	1,644	0,165	-0,297	0,037	-0,461	0,612	-0,114	0,782	0,075	.	.	.	.	.	.
SRR648705_TR23045_c0_g2_i1_len=4443	AL221_ARATH	Q:142-1917,H:1-596	80.2%ID	E:0	Aldehyde dehydrogenase 22A1	PF00171.19^Aldedh^Aldehyde dehydrogenase family^59-523^E:1.4e-121	0,001	6,608	0,381	1,365	1	0	1	0	0,629	-1,623	AL221_ARATH	Aldehyde dehydrogenase 22A1 (EC 1.2.1.3) (Novel aldehyde dehydrogenase family 22 member A1)	ALDH22A1 At3g66658 T8E24.4	Arabidopsis thaliana (Mouse-ear cress)		Alternative splicing; Complete proteome; NAD; Oxidoreductase; Reference proteome; Secreted; Signal
SRR648705_TR25614_c3_g2_i6_len=1277	NA	NA	NA	NA	NA	.	0	1,722	0,554	-0,134	0,301	0,255	0,18	0,324	0,405	0,202	.	.	.	.	.	.
SRR648705_TR25781_c0_g1_i1_len=2293	GAE6_ARATH	Q:1864-539,H:4-452	79.33%ID	E:0	UDP-glucuronate 4-epimerase 6	PF04321.14^RmlD_sub_bind^RmlD substrate binding domain^118-302^E:1e-10`PF01370.18^Epimerase^NAD dependent epimerase/dehydratase family^120-357^E:5.1e-46`PF02719.12^Polysacc_synt_2^Polysaccharide biosynthesis protein^120-356^E:3.4e-08`PF16363.2^GDP_Man_Dehyd^GDP-mannose 4,6 dehydratase^121-440^E:3.6e-48`PF01073.16^3Beta_HSD^3-beta hydroxysteroid dehydrogenase/isomerase family^121-399^E:1.4e-12`PF07993.9^NAD_binding_4^Male sterility protein^158-299^E:3.9e-05	0	1,295	0,023	0,648	0,538	-0,203	0,611	-0,16	0,255	-0,539	GAE6_ARATH	UDP-glucuronate 4-epimerase 6 (EC 5.1.3.6) (UDP-glucuronic acid epimerase 6) (AtUGlcAE2)	GAE6 UGlcAE2 At3g23820 F14O13.1	Arabidopsis thaliana (Mouse-ear cress)		Carbohydrate metabolism; Complete proteome; Golgi apparatus; Isomerase; Membrane; NAD; Reference proteome; Transmembrane; Transmembrane helix
SRR648705_TR41790_c2_g1_i2_len=6940	NA	NA	NA	NA	NA	.	0	1,665	0,064	-0,383	0,967	0,009	1	0	0,764	-0,101	.	.	.	.	.	.
SRR648707_TR1151_c0_g3_i2_len=1066	NA	NA	NA	NA	NA	.	0	3,415	0,045	1,511	0,006	1,808	1	0,219	0,663	-0,443	.	.	.	.	.	.
SRR648707_TR2930_c2_g2_i5_len=3561	PSBA_CUCSA	Q:2927-1869,H:1-353	99.72%ID	E:0	Photosystem II protein D1 {ECO:0000255|HAMAP-Rule:MF_01379}	PF00124.16^Photo_RC^Photosynthetic reaction centre protein^28-327^E:3.7e-98	0	3,35	0,095	0,503	0,19	0,41	0,097	0,503	0,003	-0,777	PSBA_CUCSA	Photosystem II protein D1 (PSII D1 protein) (EC 1.10.3.9) (Photosystem II Q(B) protein)	psbA	Cucumis sativus (Cucumber)		Acetylation; Calcium; Chlorophyll; Chloroplast; Chromophore; Electron transport; Herbicide resistance; Iron; Magnesium; Manganese; Membrane; Metal-binding; Oxidoreductase; Phosphoprotein; Photosynthesis; Photosystem II; Plastid; Thylakoid; Transmembrane; Transmembrane helix; Transport
SRR648707_TR8102_c0_g1_i2_len=1197	FK151_ARATH	Q:672-379,H:57-153	80.61%ID	E:1e-45	Peptidyl-prolyl cis-trans isomerase FKBP15-1	PF00254.25^FKBP_C^FKBP-type peptidyl-prolyl cis-trans isomerase^6-84^E:5.7e-30	0,001	2,135	0,006	2,333	0,042	-2,103	0,042	-2,083	1	-0,204	FK151_ARATH	Peptidyl-prolyl cis-trans isomerase FKBP15-1 (PPIase FKBP15-1) (EC 5.2.1.8) (15 kDa FK506-binding protein) (15 kDa FKBP) (FK506-binding protein 15-1) (AtFKBP15-1) (FK506-binding protein 2-1) (Immunophilin FKBP15-1) (Rotamase)	FKBP15-1 At3g25220 MJL12.17	Arabidopsis thaliana (Mouse-ear cress)		Complete proteome; Endoplasmic reticulum; Isomerase; Reference proteome; Rotamase; Signal
SRR648707_TR35408_c0_g2_i3_len=1189	YCX91_PHAAO	Q:833-1105,H:1-91	97.8%ID	E:3e-56	Uncharacterized protein ORF91	.	0	2,777	0,211	-0,282	0,017	-0,581	0,01	-0,629	0,258	0,989	YCX91_PHAAO	Uncharacterized protein ORF91		Phalaenopsis aphrodite subsp. formosana (Moth orchid)		Chloroplast; Plastid
SRR648707_TR37954_c0_g3_i6_len=3641	NA	NA	NA	NA	NA	PF07800.9^DUF1644^Protein of unknown function (DUF1644)^22-250^E:7.2e-76	0	5,664	0,034	5,508	0,882	-0,163	0,763	-0,243	0,319	1,051	.	.	.	.	.	.
SRR648707_TR39808_c0_g1_i10_len=2016	ALMT9_ARATH	Q:476-1174,H:134-365	56.65%ID	E:1e-91	Aluminum-activated malate transporter 9	PF11744.5^ALMT^Aluminium activated malate transporter^2-216^E:1.2e-82	0,001	6,577	1	0,131	0,258	-1,05	0,679	-0,412	0,719	0,276	ALMT9_ARATH	Aluminum-activated malate transporter 9 (AtALMT9)	ALMT9 At3g18440 MYF24.16	Arabidopsis thaliana (Mouse-ear cress)		Chloride; Complete proteome; Ion channel; Ion transport; Membrane; Reference proteome; Transmembrane; Transmembrane helix; Transport; Vacuole
SRR924147_TR23104_c0_g1_i7_len=659	NA	NA	NA	NA	NA	.	0	2,54	0,705	-0,186	0,865	-0,144	0,865	-0,115	0,781	0,441	.	.	.	.	.	.

Ms8h

Transcript (CDF97)	Uniprot homolog	BLASTX Position	BLASTX %Identity	BLASTX evalue	Uniprot descr	Pfam	PValue_Ms6h	logFC_Ms6h	PValue_Ms8h	logFC_Ms8h	PValue_MsDamaged24h	logFC_MsDamaged24h	PValue_MsIntact24h	logFC_MsIntact24h	PValue_Ms1wk	logFC_Ms1wk	Entry.name	Protein names	Gene names	Organism	Pathway	Keywords
SRR1144633_TR2479_c0_g2_i1_len=507	RER1A_ARATH	Q:292-17,H:26-116	68.48%ID	E:2e-25	Protein RER1A	PF03248.10^Rer1^Rer1 family^27-126^E:6.7e-37	0,113	0,573	0	1,824	0,526	-0,242	0,678	-0,147	0,031	0,874	RER1A_ARATH	Protein RER1A (AtRER1A)	RER1A At4g39220 T22F8.120	Arabidopsis thaliana (Mouse-ear cress)		Acetylation; Complete proteome; Membrane; Reference proteome; Transmembrane; Transmembrane helix
SRR1144633_TR2604_c0_g1_i1_len=1488	PXN_ARATH	Q:335-1249,H:22-326	73.94%ID	E:8e-160	Peroxisomal nicotinamide adenine dinucleotide carrier	PF00153.24^Mito_carr^Mitochondrial carrier protein^7-90^E:7.9e-17`PF00153.24^Mito_carr^Mitochondrial carrier protein^109-217^E:3.1e-20`PF00153.24^Mito_carr^Mitochondrial carrier protein^228-318^E:1.1e-18	0,298	0,5	0	1,835	0,001	1,616	0,044	1,087	0,255	0,621	PXN_ARATH	Peroxisomal nicotinamide adenine dinucleotide carrier (Peroxisomal NAD carrier) (Peroxisomal membrane protein 38, (PMP36)) (AtPMP38) (Protein ABERRANT PEROXISOME MORPHOLOGY 3) (Solute carrier family 25 member 17)	PXN APEM3 At2g39970 T28M21.13	Arabidopsis thaliana (Mouse-ear cress)		Antiport; Complete proteome; Glyoxysome; Membrane; Peroxisome; Reference proteome; Repeat; Transmembrane; Transmembrane helix; Transport
SRR1144633_TR5687_c0_g2_i1_len=2190	ORYA_ORYSJ	Q:1916-639,H:22-444	66.36%ID	E:0	Oryzain alpha chain	PF08246.9^Inhibitor_I29^Cathepsin propeptide inhibitor domain (I29)^116-173^E:1.3e-16`PF00112.20^Peptidase_C1^Papain family cysteine protease^204-419^E:2.2e-84`PF00396.15^Granulin^Granulin^453-499^E:9.6e-11	0,002	0,695	0	1,422	0,002	1,301	0,528	0,327	0,076	-2,996	ORYA_ORYSJ	Oryzain alpha chain (EC 3.4.22.-)	Os04g0650000 LOC_Os04g55650 H0212B02.7 OSJNBb0059K02.8	Oryza sativa subsp. japonica (Rice)		Complete proteome; Direct protein sequencing; Disulfide bond; Glycoprotein; Hydrolase; Protease; Reference proteome; Signal; Thiol protease; Zymogen
SRR1144633_TR8390_c0_g2_i1_len=3017	LACS7_ARATH	Q:2389-320,H:4-697	75.79%ID	E:0	Long chain acyl-CoA synthetase 7, peroxisomal	PF00501.25^AMP-binding^AMP-binding enzyme^105-550^E:8.5e-109	0,698	0,253	0,001	2,496	0,471	0,568	0,044	1,2	0,524	0,577	LACS7_ARATH	Long chain acyl-CoA synthetase 7, peroxisomal (EC 6.2.1.3)	LACS7 At5g27600 F15A18.60	Arabidopsis thaliana (Mouse-ear cress)	PATHWAY: Lipid metabolism; fatty acid metabolism.	ATP-binding; Complete proteome; Fatty acid metabolism; Ligase; Lipid metabolism; Magnesium; Nucleotide-binding; Peroxisome; Reference proteome
SRR1144633_TR10084_c0_g2_i1_len=1911	NA	NA	NA	NA	NA	PF10250.6^O-FucT^GDP-fucose protein O-fucosyltransferase^97-379^E:7.9e-24	0,008	0,85	0	1,609	0,411	0,319	0,248	-0,46	0,524	0,229	.	.	.	.	.	.
SRR1144633_TR11177_c0_g1_i1_len=732	NA	NA	NA	NA	NA	.	1	2,971	0	5,782	1	0	1	1,45	1	2,622	.	.	.	.	.	.
SRR1144633_TR15026_c0_g1_i3_len=2476	DHQS_SOLLC	Q:1276-2472,H:40-439	87.25%ID	E:0	3-dehydroquinate synthase, chloroplastic	PF13685.3^Fe-ADH_2^Iron-containing alcohol dehydrogenase^98-318^E:1.8e-11`PF01761.17^DHQ_synthase^3-dehydroquinate synthase^150-408^E:5.4e-106	0,272	0,499	0	2,085	0,378	0,348	0,23	0,464	0,021	1,093	DHQS_SOLLC	3-dehydroquinate synthase, chloroplastic (EC 4.2.3.4)	DHQS AROB Solyc02g083590 LOC544273	Solanum lycopersicum (Tomato) (Lycopersicon esculentum)	PATHWAY: Metabolic intermediate biosynthesis; chorismate biosynthesis; chorismate from D-erythrose 4-phosphate and phosphoenolpyruvate: step 2/7.	Amino-acid biosynthesis; Aromatic amino acid biosynthesis; Chloroplast; Complete proteome; Lyase; Metal-binding; NAD; Plastid; Reference proteome; Transit peptide
SRR1144633_TR15043_c0_g1_i2_len=1758	GPX4_SPIOL	Q:682-356,H:61-169	81.65%ID	E:5e-58	Probable phospholipid hydroperoxide glutathione peroxidase	PF00255.16^GSHPx^Glutathione peroxidase^85-141^E:4.5e-16`PF00578.18^AhpC-TSA^AhpC/TSA family^85-141^E:2.7e-08	0,241	0,716	0	1,958	0,707	0,397	1	0,066	1	0,765	GPX4_SPIOL	Probable phospholipid hydroperoxide glutathione peroxidase (PHGPx) (EC 1.11.1.12)		Spinacia oleracea (Spinach)		Cytoplasm; Oxidoreductase; Peroxidase
SRR1144633_TR15250_c0_g1_i1_len=646	BI1L_ARATH	Q:528-37,H:24-190	68.86%ID	E:2e-66	BI1-like protein	PF01027.17^Bax1-I^Inhibitor of apoptosis-promoting Bax1^61-203^E:2.3e-23	0,064	0,507	0	1,45	0,076	0,483	0,115	0,431	1	-1,548	BI1L_ARATH	BI1-like protein	At4g15470 dl3775w FCAALL.58	Arabidopsis thaliana (Mouse-ear cress)		Complete proteome; Membrane; Reference proteome; Transmembrane; Transmembrane helix
SRR1144633_TR17118_c0_g1_i1_len=2301	HSP72_SOLLC	Q:2145-208,H:1-644	94.58%ID	E:0	Heat shock cognate 70 kDa protein 2	PF00012.17^HSP70^Hsp70 protein^9-618^E:1.3e-268`PF06723.10^MreB_Mbl^MreB/Mbl protein^127-380^E:8.4e-14	0,161	-0,362	0	5,349	0	-9,168	0	-2,364	0,663	0,476	HSP72_SOLLC	Heat shock cognate 70 kDa protein 2	HSC-2	Solanum lycopersicum (Tomato) (Lycopersicon esculentum)		ATP-binding; Complete proteome; Nucleotide-binding; Reference proteome; Stress response
SRR1144633_TR17118_c0_g1_i3_len=2377	HSP7C_PETHY	Q:2221-275,H:1-651	93.09%ID	E:0	Heat shock cognate 70 kDa protein	PF00012.17^HSP70^Hsp70 protein^9-618^E:7.2e-268`PF06723.10^MreB_Mbl^MreB/Mbl protein^127-380^E:5.9e-14	0,103	1,723	0	2,461	0,002	1,241	0,006	1,106	1	0,979	HSP7C_PETHY	Heat shock cognate 70 kDa protein	HSP70	Petunia hybrida (Petunia)		ATP-binding; Nucleotide-binding; Stress response
SRR1144633_TR19576_c0_g1_i1_len=2196	IMA_SOLLC	Q:334-1917,H:1-526	85.82%ID	E:0	Importin subunit alpha	PF01749.17^IBB^Importin beta binding domain^12-94^E:1.5e-22`PF00514.20^Arm^Armadillo/beta-catenin-like repeat^105-145^E:8.4e-11`PF13646.3^HEAT_2^HEAT repeats^118-187^E:3.5e-07`PF13513.3^HEAT_EZ^HEAT-like repeat^130-185^E:5.1e-08`PF00514.20^Arm^Armadillo/beta-catenin-like repeat^147-186^E:1.4e-13`PF02985.19^HEAT^HEAT repeat^160-186^E:0.00018`PF00514.20^Arm^Armadillo/beta-catenin-like repeat^189-230^E:5.4e-07`PF00514.20^Arm^Armadillo/beta-catenin-like repeat^243-270^E:1.8e-07`PF00514.20^Arm^Armadillo/beta-catenin-like repeat^274-312^E:3.8e-09`PF00514.20^Arm^Armadillo/beta-catenin-like repeat^318-356^E:1.7e-09`PF00514.20^Arm^Armadillo/beta-catenin-like repeat^358-397^E:3.4e-13`PF00514.20^Arm^Armadillo/beta-catenin-like repeat^402-439^E:1.4e-06`PF16186.2^Arm_3^Atypical Arm repeat^456-502^E:7e-21	1	-0,036	0,001	1,393	0,481	0,281	0,385	0,343	0,283	0,452	IMA_SOLLC	Importin subunit alpha (Karyopherin subunit alpha) (KAP alpha)		Solanum lycopersicum (Tomato) (Lycopersicon esculentum)		Complete proteome; Cytoplasm; Protein transport; Reference proteome; Repeat; Transport
SRR1144633_TR22199_c0_g3_i1_len=1959	STP13_ARATH	Q:266-1822,H:14-526	83.82%ID	E:0	Sugar transport protein 13	PF00083.21^Sugar_tr^Sugar (and other) transporter^29-491^E:5.8e-119`PF07690.13^MFS_1^Major Facilitator Superfamily^77-440^E:1.3e-25	0,267	0,648	0	2,688	0,019	1,555	0,112	1,161	1	-0,349	STP13_ARATH	Sugar transport protein 13 (Hexose transporter 13) (Multicopy suppressor of snf4 deficiency protein 1)	STP13 MSS1 At5g26340 F9D12.17	Arabidopsis thaliana (Mouse-ear cress)		Cell membrane; Complete proteome; Membrane; Reference proteome; Sugar transport; Symport; Transmembrane; Transmembrane helix; Transport
SRR1144633_TR22975_c0_g1_i1_len=2297	PAO4_ARATH	Q:1771-431,H:48-497	70.07%ID	E:0	Probable polyamine oxidase 4	PF01593.21^Amino_oxidase^Flavin containing amine oxidoreductase^12-416^E:1.1e-81`PF13450.3^NAD_binding_8^NAD(P)-binding Rossmann-like domain^12-58^E:1.5e-05	0,042	0,69	0	1,275	0,312	0,718	0,235	0,838	0,593	-0,646	PAO4_ARATH	Probable polyamine oxidase 4 (AtPAO4) (EC 1.5.3.16) (Amine oxidase 2)	PAO4 At1g65840 F1E22.18	Arabidopsis thaliana (Mouse-ear cress)		Complete proteome; FAD; Flavoprotein; Oxidoreductase; Peroxisome; Reference proteome
SRR1144633_TR23146_c0_g1_i3_len=3060	ACD10_MOUSE	Q:315-2402,H:282-962	47.35%ID	E:0	Acyl-CoA dehydrogenase family member 10	PF01636.20^APH^Phosphotransferase enzyme family^43-278^E:7.5e-41`PF02771.13^Acyl-CoA_dh_N^Acyl-CoA dehydrogenase, N-terminal domain^414-556^E:8.4e-11`PF02770.16^Acyl-CoA_dh_M^Acyl-CoA dehydrogenase, middle domain^561-662^E:5.4e-18`PF00441.21^Acyl-CoA_dh_1^Acyl-CoA dehydrogenase, C-terminal domain^674-733^E:7e-09	1	0,341	0	3,829	0,034	3,617	0,009	4,132	0,62	0,665	ACD10_MOUSE	Acyl-CoA dehydrogenase family member 10 (ACAD-10) (EC 1.3.99.-)	Acad10	Mus musculus (Mouse)		Acetylation; Complete proteome; FAD; Flavoprotein; Oxidoreductase; Reference proteome
SRR1144633_TR25914_c0_g1_i1_len=1386	YIPF6_XENTR	Q:773-177,H:25-230	36.67%ID	E:1e-30	Protein YIPF6	PF04893.14^Yip1^Yip1 domain^137-268^E:3.5e-13	0,593	0,681	0	2,811	0,611	0,408	1	0,104	1	-0,078	YIPF6_XENTR	Protein YIPF6 (YIP1 family member 6)	yipf6 TGas105o14.1	Xenopus tropicalis (Western clawed frog) (Silurana tropicalis)		Complete proteome; Glycoprotein; Membrane; Reference proteome; Transmembrane; Transmembrane helix
SRR1144633_TR29750_c0_g1_i1_len=1236	NA	NA	NA	NA	NA	PF02544.13^Steroid_dh^3-oxo-5-alpha-steroid 4-dehydrogenase^132-266^E:2.4e-28`PF06966.9^DUF1295^Protein of unknown function (DUF1295)^133-236^E:2.9e-05	0,691	0,224	0	2,021	0,609	0,304	0,793	0,179	0,407	0,633	.	.	.	.	.	.
SRR1144633_TR30547_c0_g1_i1_len=2085	BH062_ARATH	Q:160-1845,H:1-456	39.72%ID	E:3e-97	Transcription factor bHLH62	PF00010.23^HLH^Helix-loop-helix DNA-binding domain^381-428^E:3.2e-06	0,593	0,603	0	2,877	0,073	2,52	0,01	3,023	1	0,281	BH062_ARATH	Transcription factor bHLH62 (Basic helix-loop-helix protein 62) (AtbHLH62) (bHLH 62) (Transcription factor EN 85) (bHLH transcription factor bHLH062)	BHLH62 EN85 At3g07340 F21O3.5	Arabidopsis thaliana (Mouse-ear cress)		Complete proteome; DNA-binding; Nucleus; Reference proteome; Transcription; Transcription regulation
SRR1144633_TR33197_c0_g2_i1_len=3093	2A5B_ARATH	Q:1916-441,H:1-492	78.54%ID	E:0	Serine/threonine protein phosphatase 2A 57 kDa regulatory subunit B' beta isoform	PF01603.17^B56^Protein phosphatase 2A regulatory B subunit (B56 family)^74-485^E:8.2e-189	1	0,621	0	7,617	0,502	3,945	1	0,704	1	0	2A5B_ARATH	Serine/threonine protein phosphatase 2A 57 kDa regulatory subunit B' beta isoform (AtB' beta) (PP2A, B' subunit, beta isoform)	B'BETA At3g09880 F8A24.7	Arabidopsis thaliana (Mouse-ear cress)		Complete proteome; Reference proteome
SRR1144633_TR34904_c0_g1_i1_len=2150	C76B6_CATRO	Q:189-1664,H:6-490	50.61%ID	E:2e-163	Geraniol 8-hydroxylase	PF00067.19^p450^Cytochrome P450^35-484^E:9e-114	1	0,235	0	2,458	0	-1,505	0	-1,446	1	0,236	C76B6_CATRO	Geraniol 8-hydroxylase (EC 1.14.13.152) (Cytochrome P450 76B6) (Geraniol 10-hydroxylase) (CrG10H)	CYP76B6 G10H	Catharanthus roseus (Madagascar periwinkle) (Vinca rosea)		Direct protein sequencing; Endoplasmic reticulum; Heme; Iron; Membrane; Metal-binding; Monooxygenase; NADP; Oxidoreductase; Transmembrane; Transmembrane helix
SRR1144633_TR35396_c1_g5_i2_len=1933	CLPP_COFAR	Q:1860-1273,H:1-196	97.96%ID	E:6e-134	ATP-dependent Clp protease proteolytic subunit {ECO:0000255|HAMAP-Rule:MF_00444}	PF00574.20^CLP_protease^Clp protease^17-194^E:2.4e-67	0,028	0,801	0	2,117	0,904	0,067	0,753	-0,113	0,004	1,235	CLPP_COFAR	ATP-dependent Clp protease proteolytic subunit (EC 3.4.21.92) (Endopeptidase Clp)	clpP	Coffea arabica (Arabian coffee)		Chloroplast; Hydrolase; Plastid; Protease; Serine protease
SRR1144633_TR37078_c0_g1_i1_len=1691	CXE18_ARATH	Q:1369-404,H:17-333	57.23%ID	E:7e-129	Probable carboxylesterase 18	PF00135.25^COesterase^Carboxylesterase family^111-168^E:6.3e-08`PF07859.10^Abhydrolase_3^alpha/beta hydrolase fold^127-348^E:5e-59	0,496	0,406	0	1,704	0,376	0,443	0,053	0,872	1	0,083	CXE18_ARATH	Probable carboxylesterase 18 (AtCXE18) (EC 3.1.1.1)	CXE18 At5g23530 MQM1.21	Arabidopsis thaliana (Mouse-ear cress)		Acetylation; Complete proteome; Hydrolase; Reference proteome; Serine esterase
SRR1144634_TR1364_c0_g1_i1_len=1646	PUMP5_ARATH	Q:1167-193,H:1-311	76.31%ID	E:8e-153	Mitochondrial uncoupling protein 5	PF00153.24^Mito_carr^Mitochondrial carrier protein^6-123^E:1.5e-16`PF00153.24^Mito_carr^Mitochondrial carrier protein^131-225^E:3.4e-19`PF00153.24^Mito_carr^Mitochondrial carrier protein^233-323^E:5.2e-19	0,534	0,484	0	2,269	0,001	-0,968	0,014	-0,706	1	0,11	PUMP5_ARATH	Mitochondrial uncoupling protein 5 (AtPUMP5) (Mitochondrial dicarboxylate carrier 1)	PUMP5 DIC1 UCP5 At2g22500 F14M13.10	Arabidopsis thaliana (Mouse-ear cress)		Complete proteome; Membrane; Mitochondrion; Mitochondrion inner membrane; Reference proteome; Repeat; Stress response; Transmembrane; Transmembrane helix; Transport
SRR1144634_TR3109_c0_g1_i1_len=1655	NA	NA	NA	NA	NA	PF06999.9^Suc_Fer-like^Sucrase/ferredoxin-like^79-290^E:3.9e-44	0,83	0,141	0	1,392	0,219	0,646	0,878	0,162	0,842	0,139	.	.	.	.	.	.
SRR1144634_TR3598_c0_g3_i1_len=971	UGT2_GARJA	Q:880-2,H:1-290	64.85%ID	E:2e-140	7-deoxyloganetin glucosyltransferase	.	0,042	2,088	0	2,615	1	-1,321	1	0,743	0,629	-1,85	UGT2_GARJA	7-deoxyloganetin glucosyltransferase (EC 2.4.1.324) (Genipin glucosyltransferase) (UDP-glucose glucosyltransferase 2) (GjUGT2) (UDP-glycosyltransferase 85A24)	UGT85A24 UGT2	Gardenia jasminoides (Cape jasmine) (Gardenia augusta)		Glycosyltransferase; Transferase
SRR1144634_TR7175_c0_g3_i1_len=1256	NA	NA	NA	NA	NA	.	0,254	4,561	0	6,678	0,646	0,315	0,729	-0,247	0,56	-0,906	.	.	.	.	.	.
SRR1144634_TR17686_c0_g2_i1_len=2222	NA	NA	NA	NA	NA	PF02485.18^Branch^Core-2/I-Branching enzyme^107-336^E:4.8e-84	0,502	0,295	0	1,649	0,487	0,324	0,291	0,471	0,833	-0,136	.	.	.	.	.	.
SRR1144634_TR18250_c0_g6_i1_len=1967	KATAM_ORYSJ	Q:499-1704,H:117-517	42.65%ID	E:2e-107	Xyloglucan galactosyltransferase KATAMARI1 homolog	PF03016.12^Exostosin^Exostosin family^117-456^E:3.6e-74	0,02	1,194	0	1,918	0,739	0,162	0,653	-0,172	0,587	-0,284	KATAM_ORYSJ	Xyloglucan galactosyltransferase KATAMARI1 homolog (EC 2.4.1.-)	Os03g0144800 LOC_Os03g05110 OJ1172F09.5	Oryza sativa subsp. japonica (Rice)	PATHWAY: Protein modification; protein glycosylation.	Complete proteome; Glycosyltransferase; Golgi apparatus; Membrane; Reference proteome; Signal-anchor; Transferase; Transmembrane; Transmembrane helix
SRR1144634_TR19241_c1_g3_i3_len=927	CALM3_PETHY	Q:109-561,H:1-151	99.34%ID	E:2e-102	Calmodulin-related protein	PF00036.29^EF-hand_1^EF hand^12-40^E:3.3e-09`PF13499.3^EF-hand_7^EF-hand domain pair^12-73^E:3e-15`PF13405.3^EF-hand_6^EF-hand domain^12-41^E:1.5e-09`PF13202.3^EF-hand_5^EF hand^14-37^E:8.4e-05`PF13833.3^EF-hand_8^EF-hand domain pair^25-75^E:6.4e-14`PF00036.29^EF-hand_1^EF hand^48-75^E:2.4e-08`PF13202.3^EF-hand_5^EF hand^49-73^E:0.00019`PF13499.3^EF-hand_7^EF-hand domain pair^83-146^E:7.2e-18`PF00036.29^EF-hand_1^EF hand^85-112^E:2.7e-09`PF13405.3^EF-hand_6^EF-hand domain^85-114^E:2.7e-08`PF12763.4^EF-hand_4^Cytoskeletal-regulatory complex EF hand^85-147^E:3.6e-05`PF08976.8^EF-hand_11^EF-hand domain^85-151^E:0.00015`PF13202.3^EF-hand_5^EF hand^86-110^E:1.9e-06`PF13833.3^EF-hand_8^EF-hand domain pair^98-146^E:1.4e-14`PF00036.29^EF-hand_1^EF hand^121-148^E:4.5e-10`PF13202.3^EF-hand_5^EF hand^124-146^E:7.2e-05	1	0,093	0	1,717	0,839	0,13	0,196	0,528	0,592	0,22	CALM3_PETHY	Calmodulin-related protein	CAM53	Petunia hybrida (Petunia)		Calcium; Metal-binding; Methylation; Repeat
SRR1144634_TR21619_c0_g1_i1_len=1897	NA	NA	NA	NA	NA	.	0,429	0,476	0	1,981	0,09	-0,581	0,371	-0,31	0	-1,071	.	.	.	.	.	.
SRR1144634_TR22239_c0_g3_i3_len=1531	BIP5_TOBAC	Q:1531-125,H:178-646	97.87%ID	E:0	Luminal-binding protein 5	PF00012.17^HSP70^Hsp70 protein^1-468^E:5.8e-201`PF06723.10^MreB_Mbl^MreB/Mbl protein^2-228^E:1.6e-14	0,31	-0,291	0	1,499	0,816	0,111	0,101	-0,668	0,052	1,569	BIP5_TOBAC	Luminal-binding protein 5 (BiP 5) (78 kDa glucose-regulated protein homolog 5) (GRP-78-5)	BIP5	Nicotiana tabacum (Common tobacco)		ATP-binding; Endoplasmic reticulum; Glycoprotein; Nucleotide-binding; Signal
SRR1144634_TR23126_c3_g3_i1_len=696	PRS2_SOLTU	Q:597-130,H:1-155	38.75%ID	E:5e-25	Pathogenesis-related protein STH-2	PF00407.16^Bet_v_1^Pathogenesis-related protein Bet v I family^34-183^E:2.5e-11	0,593	0,672	0	2,099	1	0,017	0,067	0,646	0,407	0,666	PRS2_SOLTU	Pathogenesis-related protein STH-2	STH-2	Solanum tuberosum (Potato)		Complete proteome; Pathogenesis-related protein; Plant defense; Reference proteome
SRR1144634_TR25112_c0_g1_i1_len=1039	NA	NA	NA	NA	NA	.	0,219	0,678	0,001	1,624	0,001	1,842	0,031	1,352	0,042	-1,862	.	.	.	.	.	.
SRR1144634_TR26885_c0_g2_i4_len=1467	GEDH1_OCIBA	Q:280-1242,H:33-353	64.49%ID	E:8e-106	Geraniol dehydrogenase 1	PF08240.9^ADH_N^Alcohol dehydrogenase GroES-like domain^5-90^E:4.3e-18`PF02826.16^2-Hacid_dh_C^D-isomer specific 2-hydroxyacid dehydrogenase, NAD binding domain^113-164^E:8.4e-07`PF00107.23^ADH_zinc_N^Zinc-binding dehydrogenase^134-257^E:1.9e-19	0,034	0,832	0	3,162	0,2	0,658	0,068	-1,133	0,311	0,35	GEDH1_OCIBA	Geraniol dehydrogenase 1 (ObaGEDH1) (EC 1.1.1.183)	GEDH1	Ocimum basilicum (Sweet basil)		Metal-binding; NADP; Oxidoreductase; Zinc
SRR1144634_TR28979_c0_g2_i1_len=1098	CAMT4_ARATH	Q:956-222,H:15-259	91.84%ID	E:3e-170	Caffeoyl-CoA O-methyltransferase 1	PF01596.14^Methyltransf_3^O-methyltransferase^36-247^E:3.3e-105`PF13578.3^Methyltransf_24^Methyltransferase domain^85-191^E:1.5e-09	0,005	0,902	0	4,742	0,082	0,465	0,032	0,565	0,007	2,249	CAMT4_ARATH	Caffeoyl-CoA O-methyltransferase 1 (EC 2.1.1.104) (Trans-caffeoyl-CoA 3-O-methyltransferase) (CCoAMT) (CCoAOMT1)	CCOAOMT1 At4g34050 F28A23.190	Arabidopsis thaliana (Mouse-ear cress)	PATHWAY: Aromatic compound metabolism; phenylpropanoid biosynthesis.	Acetylation; Alternative splicing; Complete proteome; Lignin biosynthesis; Metal-binding; Methyltransferase; Reference proteome; S-adenosyl-L-methionine; Transferase
SRR1144634_TR36865_c0_g1_i1_len=1455	MTDH_FRAAN	Q:142-1200,H:4-356	69.12%ID	E:1e-147	Probable mannitol dehydrogenase	PF08240.9^ADH_N^Alcohol dehydrogenase GroES-like domain^39-152^E:1.2e-27`PF00107.23^ADH_zinc_N^Zinc-binding dehydrogenase^195-318^E:7.5e-19`PF13602.3^ADH_zinc_N_2^Zinc-binding dehydrogenase^228-347^E:3e-07	1	0,023	0	1,453	0,034	1,006	0,352	0,511	0,914	-0,038	MTDH_FRAAN	Probable mannitol dehydrogenase (EC 1.1.1.255) (NAD-dependent mannitol dehydrogenase)	CAD	Fragaria ananassa (Strawberry)		Metal-binding; NAD; Oxidoreductase; Zinc
SRR1144634_TR37091_c0_g2_i2_len=697	FABI_BRANA	Q:659-3,H:89-307	91.32%ID	E:1e-143	Enoyl-[acyl-carrier-protein] reductase [NADH], chloroplastic	.	0,803	-0,151	0	7,184	0,059	1,581	0,016	1,9	0,781	0,341	FABI_BRANA	Enoyl-[acyl-carrier-protein] reductase [NADH], chloroplastic (EC 1.3.1.9) (NADH-dependent enoyl-ACP reductase)		Brassica napus (Rape)	PATHWAY: Lipid metabolism; fatty acid biosynthesis.	3D-structure; Chloroplast; Direct protein sequencing; Fatty acid biosynthesis; Fatty acid metabolism; Lipid biosynthesis; Lipid metabolism; NAD; Oxidoreductase; Plastid; Transit peptide
SRR122236_TR3141_c0_g1_i1_len=2618	NUCL2_ORYSJ	Q:1082-492,H:435-630	56.28%ID	E:3e-62	Nucleolin 2	PF00076.19^RRM_1^RNA recognition motif. (a.k.a. RRM, RBD, or RNP domain)^30-100^E:1e-19`PF13893.3^RRM_5^RNA recognition motif. (a.k.a. RRM, RBD, or RNP domain)^69-103^E:0.17`PF00076.19^RRM_1^RNA recognition motif. (a.k.a. RRM, RBD, or RNP domain)^133-203^E:2.7e-08	0	-3,8	0	2,321	1	0	1	0	1	0,314	NUCL2_ORYSJ	Nucleolin 2 (Protein NUCLEOLIN LIKE 2)	Os04g0620700 LOC_Os04g52960 OSJNBa0058K23.21	Oryza sativa subsp. japonica (Rice)		Complete proteome; Nucleus; RNA-binding; Reference proteome; Repeat; rRNA processing
SRR122236_TR11769_c1_g1_i1_len=994	CFI3_ARATH	Q:754-161,H:6-204	68.34%ID	E:2e-82	Probable chalcone–flavonone isomerase 3	PF02431.12^Chalcone^Chalcone-flavanone isomerase^9-201^E:3.8e-30	1	0,023	0	1,732	0,09	-0,45	0	-1,022	0,599	-0,187	CFI3_ARATH	Probable chalcone–flavonone isomerase 3 (Chalcone isomerase 3) (EC 5.5.1.6) (Chalcone isomerase-like 1)	CHI3 CHI-L1 CHIL At5g05270 K18I23.7	Arabidopsis thaliana (Mouse-ear cress)	PATHWAY: Secondary metabolite biosynthesis; flavonoid biosynthesis.	3D-structure; Complete proteome; Flavonoid biosynthesis; Isomerase; Reference proteome
SRR122236_TR13314_c0_g1_i1_len=1104	STR15_ARATH	Q:958-440,H:4-181	50%ID	E:4e-52	Rhodanese-like domain-containing protein 15, chloroplastic	PF00581.17^Rhodanese^Rhodanese-like domain^81-174^E:1.3e-12	1	0	0	6,681	0,502	-4,007	1	-0,84	1	-2,05	STR15_ARATH	Rhodanese-like domain-containing protein 15, chloroplastic (Protein DARK INDUCIBLE 1) (Senescence-associated protein 1) (AtSEN1) (Sulfurtransferase 15) (AtStr15)	STR15 DIN1 SEN1 At4g35770 F4B14.3 F8D20.280	Arabidopsis thaliana (Mouse-ear cress)		Alternative splicing; Chloroplast; Complete proteome; Plastid; Reference proteome; Thylakoid; Transit peptide
SRR122236_TR13806_c0_g2_i1_len=682	VP321_ARATH	Q:294-611,H:1-107	86.92%ID	E:6e-49	Vacuolar protein sorting-associated protein 32 homolog 1	PF03357.18^Snf7^Snf7^20-116^E:7.6e-22	0,789	-0,104	0	1,192	0,175	0,437	0,211	0,418	0,112	0,553	VP321_ARATH	Vacuolar protein sorting-associated protein 32 homolog 1 (AtVPS32-1) (Charged multivesicular body protein 4 homolog 1) (ESCRT-III complex subunit VPS32 homolog 1)	VPS32.1 CHMP4-1 At2g19830 F6F22.14	Arabidopsis thaliana (Mouse-ear cress)		Coiled coil; Complete proteome; Endosome; Protein transport; Reference proteome; Transport
SRR122236_TR20709_c0_g2_i1_len=2081	HSP72_SOLLC	Q:3-1928,H:5-644	94.7%ID	E:0	Heat shock cognate 70 kDa protein 2	PF00012.17^HSP70^Hsp70 protein^5-614^E:6.6e-269`PF06723.10^MreB_Mbl^MreB/Mbl protein^138-376^E:1.5e-13	0,603	-0,227	0	2,08	0,798	0,087	0,202	0,373	0,271	0,26	HSP72_SOLLC	Heat shock cognate 70 kDa protein 2	HSC-2	Solanum lycopersicum (Tomato) (Lycopersicon esculentum)		ATP-binding; Complete proteome; Nucleotide-binding; Reference proteome; Stress response
SRR122236_TR27726_c0_g1_i1_len=887	CALM_LILLO	Q:766-320,H:1-149	100%ID	E:3e-101	Calmodulin	PF00036.29^EF-hand_1^EF hand^12-40^E:2.4e-09`PF13499.3^EF-hand_7^EF-hand domain pair^12-73^E:1.9e-15`PF13405.3^EF-hand_6^EF-hand domain^12-41^E:1e-09`PF13202.3^EF-hand_5^EF hand^14-37^E:6.2e-05`PF13833.3^EF-hand_8^EF-hand domain pair^25-75^E:4.2e-14`PF00036.29^EF-hand_1^EF hand^48-75^E:1.7e-08`PF13202.3^EF-hand_5^EF hand^49-73^E:0.00014`PF13499.3^EF-hand_7^EF-hand domain pair^83-146^E:4.6e-18`PF00036.29^EF-hand_1^EF hand^85-112^E:2e-09`PF13405.3^EF-hand_6^EF-hand domain^85-114^E:1.9e-08`PF12763.4^EF-hand_4^Cytoskeletal-regulatory complex EF hand^85-146^E:4.6e-05`PF13202.3^EF-hand_5^EF hand^86-110^E:1.4e-06`PF13833.3^EF-hand_8^EF-hand domain pair^98-146^E:9.8e-15`PF00036.29^EF-hand_1^EF hand^121-148^E:3.3e-10`PF13202.3^EF-hand_5^EF hand^124-146^E:5.3e-05	1	0,009	0	1,337	0,316	0,262	0,015	0,607	0,193	-0,343	CALM_LILLO	Calmodulin (CaM)		Lilium longiflorum (Trumpet lily)		Acetylation; Calcium; Metal-binding; Methylation; Repeat
SRR122237_TR1540_c1_g2_i1_len=1166	MTDH_FRAAN	Q:1052-3,H:4-353	69.14%ID	E:3e-149	Probable mannitol dehydrogenase	PF08240.9^ADH_N^Alcohol dehydrogenase GroES-like domain^39-152^E:1.2e-27`PF00107.23^ADH_zinc_N^Zinc-binding dehydrogenase^195-318^E:7.2e-19`PF13602.3^ADH_zinc_N_2^Zinc-binding dehydrogenase^228-347^E:2.9e-07	0,484	-0,408	0	3,349	0,299	1,308	0,189	1,526	0,065	1,357	MTDH_FRAAN	Probable mannitol dehydrogenase (EC 1.1.1.255) (NAD-dependent mannitol dehydrogenase)	CAD	Fragaria ananassa (Strawberry)		Metal-binding; NAD; Oxidoreductase; Zinc
SRR122237_TR1813_c0_g1_i2_len=1223	AKRC9_ARATH	Q:1125-181,H:1-315	73.65%ID	E:5e-168	Aldo-keto reductase family 4 member C9	PF00248.18^Aldo_ket_red^Aldo/keto reductase family^51-317^E:2.5e-46	0,822	0,281	0,001	2,924	0,017	5,897	0,034	5,675	1	-0,951	AKRC9_ARATH	Aldo-keto reductase family 4 member C9 (EC 1.1.1.-)	AKR4C9 At2g37770 F13M22 T8P21.32	Arabidopsis thaliana (Mouse-ear cress)		3D-structure; Acetylation; Alternative splicing; Complete proteome; Detoxification; NADP; Oxidoreductase; Reference proteome; Stress response
SRR122237_TR6005_c0_g2_i3_len=524	COPA2_ARATH	Q:244-32,H:71-141	94.37%ID	E:2e-73	Coatomer subunit alpha-2	PF00400.29^WD40^WD domain, G-beta repeat^35-72^E:6.6e-10	0,212	0,539	0	2,212	0,329	-0,515	0,123	-0,799	0,09	0,984	COPA2_ARATH	Coatomer subunit alpha-2 (Alpha-coat protein 2) (Alpha-COP 2)	At2g21390 F3K23.15	Arabidopsis thaliana (Mouse-ear cress)		Complete proteome; Cytoplasm; Cytoplasmic vesicle; ER-Golgi transport; Golgi apparatus; Membrane; Protein transport; Reference proteome; Repeat; Transport; WD repeat
SRR122237_TR12797_c0_g1_i1_len=1300	HIDM_GLYEC	Q:292-1170,H:19-327	43.59%ID	E:4e-71	2-hydroxyisoflavanone dehydratase	PF00135.25^COesterase^Carboxylesterase family^68-178^E:5.2e-06`PF07859.10^Abhydrolase_3^alpha/beta hydrolase fold^82-290^E:1.2e-42	0,326	0,503	0	1,951	1	0,155	1	-1,458	0,025	0,732	HIDM_GLYEC	2-hydroxyisoflavanone dehydratase (EC 3.1.1.1) (EC 4.2.1.105) (Carboxylesterase HIDM)	HIDM	Glycyrrhiza echinata (Licorice)	PATHWAY: Secondary metabolite biosynthesis; flavonoid biosynthesis.	Flavonoid biosynthesis; Hydrolase; Lyase
SRR122237_TR16659_c0_g1_i1_len=1142	NA	NA	NA	NA	NA	PF13419.3^HAD_2^Haloacid dehalogenase-like hydrolase^37-229^E:3.2e-14`PF00702.23^Hydrolase^haloacid dehalogenase-like hydrolase^126-223^E:7e-07`PF13242.3^Hydrolase_like^HAD-hyrolase-like^186-238^E:4.3e-08	0,299	1,364	0	4,344	0,692	1,223	0,692	1,196	1	0	.	.	.	.	.	.
SRR122237_TR18008_c0_g1_i2_len=2280	HSP7C_PETHY	Q:2218-272,H:1-651	93.09%ID	E:0	Heat shock cognate 70 kDa protein	PF00012.17^HSP70^Hsp70 protein^9-618^E:3.2e-268`PF06723.10^MreB_Mbl^MreB/Mbl protein^127-380^E:8.5e-14	0,518	-0,212	0	1,211	0,877	0,052	0,202	0,376	0,132	2,143	HSP7C_PETHY	Heat shock cognate 70 kDa protein	HSP70	Petunia hybrida (Petunia)		ATP-binding; Nucleotide-binding; Stress response
SRR122237_TR26495_c0_g1_i1_len=586	UBC35_ARATH	Q:452-3,H:1-150	97.33%ID	E:2e-103	Ubiquitin-conjugating enzyme E2 35	PF00179.23^UQ_con^Ubiquitin-conjugating enzyme^9-145^E:8.8e-50	0,753	-0,111	0	1,503	0,007	1,12	0,001	1,285	1	-0,894	UBC35_ARATH	Ubiquitin-conjugating enzyme E2 35 (EC 2.3.2.23) (E2 ubiquitin-conjugating enzyme 35) (Ubiquitin carrier protein 35)	UBC35 UBC13A UBG13A At1g78870 F9K20.8	Arabidopsis thaliana (Mouse-ear cress)	PATHWAY: Protein modification; protein ubiquitination. {ECO:0000255|PROSITE-ProRule:PRU00388}.	ATP-binding; Alternative splicing; Complete proteome; Nucleotide-binding; Reference proteome; Transferase; Ubl conjugation pathway
SRR122237_TR27921_c0_g1_i1_len=601	GLRX_SOLLC	Q:528-235,H:1-98	79.59%ID	E:2e-52	Glutaredoxin	PF00462.21^Glutaredoxin^Glutaredoxin^15-77^E:3.6e-23	0,475	0,167	0	1,151	0,037	0,507	0,027	0,533	0,016	-1,092	GLRX_SOLLC	Glutaredoxin		Solanum lycopersicum (Tomato) (Lycopersicon esculentum)		Complete proteome; Cytoplasm; Disulfide bond; Electron transport; Redox-active center; Reference proteome; Transport
SRR122237_TR29344_c0_g1_i2_len=2041	BXL1_ARATH	Q:1887-253,H:226-771	70.73%ID	E:0	Beta-D-xylosidase 1	PF00933.18^Glyco_hydro_3^Glycosyl hydrolase family 3 N terminal domain^1-100^E:1.8e-07`PF01915.19^Glyco_hydro_3_C^Glycosyl hydrolase family 3 C-terminal domain^140-370^E:8e-53`PF14310.3^Fn3-like^Fibronectin type III-like domain^440-502^E:1.1e-09	0,407	-0,768	0	2,449	1	0,562	1	-0,021	0,629	-2,007	BXL1_ARATH	Beta-D-xylosidase 1 (AtBXL1) (EC 3.2.1.-) (Alpha-L-arabinofuranosidase) (EC 3.2.1.55)	BXL1 XYL1 At5g49360 K7J8.3	Arabidopsis thaliana (Mouse-ear cress)		Complete proteome; Extracellular matrix; Glycoprotein; Glycosidase; Hydrolase; Reference proteome; Secreted; Signal
SRR122238_TR60_c0_g1_i1_len=1086	NA	NA	NA	NA	NA	.	0,706	0,151	0	1,917	0,92	-0,039	0,115	-0,488	0	-1,09	.	.	.	.	.	.
SRR122238_TR1325_c0_g1_i1_len=2082	MFSD5_HUMAN	Q:184-1446,H:6-419	37.76%ID	E:6e-68	Molybdate-anion transporter	PF05631.11^MFS_5^Sugar-tranasporters, 12 TM^4-358^E:1.7e-184`PF07690.13^MFS_1^Major Facilitator Superfamily^43-393^E:6.8e-15	0,464	0,228	0	1,019	0,231	-0,355	0,448	-0,232	0,911	-0,037	MFSD5_HUMAN	Molybdate-anion transporter (Major facilitator superfamily domain-containing protein 5) (Molybdate transporter 2 homolog) (hsMOT2)	MFSD5 UNQ832/PRO1759	Homo sapiens (Human)		Alternative splicing; Cell membrane; Complete proteome; Ion transport; Membrane; Reference proteome; Transmembrane; Transmembrane helix; Transport
SRR122238_TR1677_c0_g1_i1_len=1509	NA	NA	NA	NA	NA	PF00582.23^Usp^Universal stress protein family^5-157^E:2.5e-31	0,08	1,339	0	2,866	0,002	1,704	0,147	0,945	1	-0,565	.	.	.	.	.	.
SRR122238_TR2204_c0_g1_i1_len=2125	GH31_ARATH	Q:1990-197,H:1-589	83.64%ID	E:0	Probable indole-3-acetic acid-amido synthetase GH3.1	PF03321.10^GH3^GH3 auxin-responsive promoter^71-613^E:1.3e-209	0,356	1,116	0	6,26	0,132	1,878	0,025	2,427	0,226	-2,135	GH31_ARATH	Probable indole-3-acetic acid-amido synthetase GH3.1 (EC 6.3.2.-) (Auxin-responsive GH3-like protein 1) (AtGH3-1)	GH3.1 At2g14960 T26I20.12	Arabidopsis thaliana (Mouse-ear cress)		Complete proteome; Ligase; Reference proteome
SRR122238_TR3315_c0_g1_i7_len=2496	NA	NA	NA	NA	NA	PF11961.5^DUF3475^Domain of unknown function (DUF3475)^155-211^E:5.5e-22`PF05003.9^DUF668^Protein of unknown function (DUF668)^375-460^E:3.4e-32	0,407	0,739	0	2,321	0,239	1,367	0,517	0,938	1	-0,543	.	.	.	.	.	.
SRR122238_TR4682_c0_g1_i1_len=733	21KD_DAUCA	Q:549-79,H:28-187	35.62%ID	E:4e-22	21 kDa protein	PF04043.12^PMEI^Plant invertase/pectin methylesterase inhibitor^61-214^E:6.2e-28	1	-0,34	0	7,249	1	0,297	1	-0,973	1	0	21KD_DAUCA	21 kDa protein (1.2 protein)		Daucus carota (Wild carrot)		Signal
SRR122238_TR4696_c0_g3_i1_len=642	PRS1_SOLTU	Q:533-90,H:1-155	35.9%ID	E:7e-22	Pathogenesis-related protein STH-21	PF00407.16^Bet_v_1^Pathogenesis-related protein Bet v I family^4-142^E:1.1e-12	1	0,016	0	3,245	0,003	1,269	0,019	1,035	0,732	-0,571	PRS1_SOLTU	Pathogenesis-related protein STH-21	STH-21	Solanum tuberosum (Potato)		Complete proteome; Pathogenesis-related protein; Plant defense; Reference proteome
SRR122238_TR8715_c0_g2_i1_len=1514	LOR8_ARATH	Q:1370-747,H:1-196	48.13%ID	E:2e-54	Protein LURP-one-related 8	PF04525.9^LOR^LURP-one-related^80-247^E:1.3e-47	0,132	2,268	0	3,732	0,021	1,75	0,62	0,685	0,76	-0,413	LOR8_ARATH	Protein LURP-one-related 8	At2g38640 T6A23.16	Arabidopsis thaliana (Mouse-ear cress)		Complete proteome; Reference proteome
SRR122238_TR11100_c1_g1_i1_len=1541	UBC32_ARATH	Q:1416-514,H:1-307	63.23%ID	E:9e-130	Ubiquitin-conjugating enzyme E2 32	PF00179.23^UQ_con^Ubiquitin-conjugating enzyme^15-126^E:2.1e-20	0,331	0,348	0,001	1,022	0,06	0,875	0,142	0,715	0,156	0,655	UBC32_ARATH	Ubiquitin-conjugating enzyme E2 32 (EC 2.3.2.23) (E2 ubiquitin-conjugating enzyme 32) (Ubiquitin carrier protein 32)	UBC32 At3g17000 K14A17.7	Arabidopsis thaliana (Mouse-ear cress)	PATHWAY: Protein modification; protein ubiquitination. {ECO:0000255|PROSITE-ProRule:PRU00388}.	ATP-binding; Complete proteome; Membrane; Nucleotide-binding; Reference proteome; Transferase; Transmembrane; Transmembrane helix; Ubl conjugation pathway
SRR122238_TR12157_c0_g1_i2_len=733	NRPBC_ARATH	Q:242-394,H:1-51	90.2%ID	E:1e-26	DNA-directed RNA polymerases II, IV and V subunit 12	.	1	-0,016	0	1,252	0,006	0,946	0,111	0,575	0,84	-0,106	NRPBC_ARATH	DNA-directed RNA polymerases II, IV and V subunit 12 (DNA-directed RNA Polymerase II subunit K)	NRPB12 NRPD12 NRPE12 At5g41010 MEE6.8	Arabidopsis thaliana (Mouse-ear cress)		Complete proteome; DNA-directed RNA polymerase; Metal-binding; Nucleus; Reference proteome; Transcription; Zinc
SRR122238_TR13167_c0_g1_i1_len=1519	Y1571_ARATH	Q:447-1118,H:139-359	40.18%ID	E:5e-35	Probable leucine-rich repeat receptor-like protein kinase At1g35710	PF08263.9^LRRNT_2^Leucine rich repeat N-terminal domain^113-154^E:0.0024`PF00560.30^LRR_1^Leucine Rich Repeat^209-229^E:0.19`PF13516.3^LRR_6^Leucine Rich repeat^209-223^E:0.59`PF13855.3^LRR_8^Leucine rich repeat^224-268^E:1.1e-06`PF12799.4^LRR_4^Leucine Rich repeats (2 copies)^232-273^E:6.5e-07`PF00560.30^LRR_1^Leucine Rich Repeat^233-255^E:0.66`PF12799.4^LRR_4^Leucine Rich repeats (2 copies)^256-300^E:1.8e-06`PF13516.3^LRR_6^Leucine Rich repeat^280-292^E:2`PF00560.30^LRR_1^Leucine Rich Repeat^281-297^E:0.19`PF13855.3^LRR_8^Leucine rich repeat^307-366^E:2.3e-13`PF12799.4^LRR_4^Leucine Rich repeats (2 copies)^307-344^E:1.9e-06`PF00560.30^LRR_1^Leucine Rich Repeat^309-330^E:1.3	0,275	0,443	0,001	1,309	0,415	0,347	0,819	-0,148	0,38	0,379	Y1571_ARATH	Probable leucine-rich repeat receptor-like protein kinase At1g35710 (EC 2.7.11.1)	At1g35710 F14D7.1	Arabidopsis thaliana (Mouse-ear cress)		ATP-binding; Alternative splicing; Complete proteome; Glycoprotein; Kinase; Leucine-rich repeat; Membrane; Nucleotide-binding; Phosphoprotein; Receptor; Reference proteome; Repeat; Serine/threonine-protein kinase; Signal; Transferase; Transmembrane; Transmembrane helix
SRR122238_TR13468_c0_g1_i6_len=1804	CBWD1_HUMAN	Q:1316-249,H:41-377	36.97%ID	E:7e-65	COBW domain-containing protein 1	PF02492.16^cobW^CobW/HypB/UreG, nucleotide-binding domain^99-283^E:1.2e-56`PF07683.11^CobW_C^Cobalamin synthesis protein cobW C-terminal domain^358-452^E:5.3e-21	0,031	-0,584	0	7,264	1	-0,977	1	-0,697	0,734	-0,106	CBWD1_HUMAN	COBW domain-containing protein 1 (Cobalamin synthase W domain-containing protein 1) (COBP) (NPC-A-6 COBW domain-containing protein 1) (NPC-A-6)	CBWD1	Homo sapiens (Human)		ATP-binding; Alternative splicing; Complete proteome; Nucleotide-binding; Polymorphism; Reference proteome
SRR122238_TR14088_c0_g2_i1_len=871	NA	NA	NA	NA	NA	.	1	3,013	0	6,662	0,002	6,284	0,009	6,036	1	-0,346	.	.	.	.	.	.
SRR122238_TR14264_c0_g1_i1_len=1379	SAMT_CLABR	Q:67-1137,H:1-357	55.46%ID	E:1e-138	Salicylate carboxymethyltransferase	PF03492.12^Methyltransf_7^SAM dependent carboxyl methyltransferase^39-356^E:7.3e-126	1	0,533	0	8,601	1	2,093	1	-0,316	1	0,013	SAMT_CLABR	Salicylate carboxymethyltransferase (EC 2.1.1.274) (S-adenosyl-L-methionine:salicylate acid carboxylmethyltransferase) (CbSAMT) (Salicylate O-methyltransferase)	SAMT	Clarkia breweri (Fairy fans) (Eucharidium breweri)		3D-structure; Magnesium; Metal-binding; Methyltransferase; S-adenosyl-L-methionine; Transferase
SRR122238_TR15504_c0_g2_i1_len=1263	CX5B2_ARATH	Q:376-702,H:49-157	71.56%ID	E:4e-43	Cytochrome c oxidase subunit 5b-2, mitochondrial	PF01215.16^COX5B^Cytochrome c oxidase subunit Vb^151-241^E:4.6e-17`PF10276.6^zf-CHCC^Zinc-finger domain^194-229^E:2.4e-09	0,256	-0,336	0	4,996	1	0,687	1	0,234	0,094	-0,49	CX5B2_ARATH	Cytochrome c oxidase subunit 5b-2, mitochondrial (AtCOX5b-2)	COX5B-2 At1g80230 F18B13.29	Arabidopsis thaliana (Mouse-ear cress)		Complete proteome; Membrane; Metal-binding; Mitochondrion; Mitochondrion inner membrane; Reference proteome; Transit peptide; Zinc
SRR122238_TR17128_c0_g1_i1_len=967	DNJ20_ARATH	Q:625-215,H:66-197	57.25%ID	E:2e-44	Chaperone protein dnaJ 20, chloroplastic	PF00226.28^DnaJ^DnaJ domain^80-144^E:3.1e-21	0,341	-0,418	0	1,691	0,001	3,125	0,001	3,174	1	-1,551	DNJ20_ARATH	Chaperone protein dnaJ 20, chloroplastic (AtDjC20) (AtJ20)	ATJ20 C20 J20 At4g13830 F18A5.220	Arabidopsis thaliana (Mouse-ear cress)		Alternative splicing; Chaperone; Chloroplast; Complete proteome; Plastid; Reference proteome; Transit peptide

SRR122238_TR17315_c0_g1_i1_len=978	ISCAP_ARATH	Q:678-304,H:56-180	83.2%ID	E:6e-71	Iron-sulfur assembly protein IscA, chloroplastic	PF01521.17^Fe-S_biosyn^Iron-sulphur cluster biosynthesis^117-219^E:2.5e-21	0,87	0,229	0	2,264	0,049	1,206	1	-0,084	1	-0,046	ISCAP_ARATH	Iron-sulfur assembly protein IscA, chloroplastic (Plastid SufA-like protein) (Protein scaffold protein AtCpIscA)	ISCA At1g10500 T10O24.11	Arabidopsis thaliana (Mouse-ear cress)		Chloroplast; Complete proteome; Iron; Iron-sulfur; Metal-binding; Plastid; Reference proteome; Transit peptide
SRR122238_TR22165_c0_g1_i2_len=1106	SRP_VITRI	Q:129-866,H:1-241	65.04%ID	E:2e-89	Stress-related protein	PF05755.9^REF^Rubber elongation factor protein (REF)^60-274^E:4e-92	0,452	0,281	0,001	1,249	0,147	0,489	0,64	-0,177	0,256	0,379	SRP_VITRI	Stress-related protein	SRP	Vitis riparia (Frost grape) (Vitis vulpina)		Stress response
SRR122238_TR22541_c0_g1_i4_len=1860	NA	NA	NA	NA	NA	.	0,76	0,775	0,001	2,271	0,356	1,253	0,356	1,269	0,461	1,559	.	.	.	.	.	.
SRR122238_TR23102_c0_g1_i5_len=1620	LEU1A_SOLPN	Q:209-1507,H:23-454	85.22%ID	E:0	2-isopropylmalate synthase A	PF00682.16^HMGL-like^HMGL-like^73-352^E:6.6e-92	0,174	-0,56	0	2,632	0,547	-0,339	0,317	0,541	0,145	0,512	LEU1A_SOLPN	2-isopropylmalate synthase A (EC 2.3.3.13) (Alpha-IPM synthase A) (Alpha-isopropylmalate synthase A)	IPMSA	Solanum pennellii (Tomato) (Lycopersicon pennellii)	PATHWAY: Amino-acid biosynthesis; L-leucine biosynthesis; L-leucine from 3-methyl-2-oxobutanoate: step 1/4.	Amino-acid biosynthesis; Branched-chain amino acid biosynthesis; Leucine biosynthesis; Transferase
SRR122238_TR23144_c0_g2_i3_len=965	DNAJ8_ARATH	Q:483-157,H:55-162	69.09%ID	E:2e-35	Chaperone protein dnaJ 8, chloroplastic	PF00226.28^DnaJ^DnaJ domain^43-96^E:3.5e-15	0,326	-0,329	0	9,182	1	-1,482	1	-1,482	0,006	0,908	DNAJ8_ARATH	Chaperone protein dnaJ 8, chloroplastic (AtDjC8) (AtJ8)	ATJ8 C8 J8 At1g80920 F23A5.28	Arabidopsis thaliana (Mouse-ear cress)		Chaperone; Chloroplast; Complete proteome; Plastid; Reference proteome; Transit peptide
SRR122238_TR29113_c0_g1_i2_len=1378	NA	NA	NA	NA	NA	PF06232.8^ATS3^Embryo-specific protein 3, (ATS3)^43-161^E:7.4e-53	0,618	0,279	0,001	2,283	1	0,218	1	0,119	0,166	0,779	.	.	.	.	.	.
SRR122238_TR29256_c0_g1_i1_len=1272	RL5_CUCSA	Q:75-923,H:1-283	85.51%ID	E:1e-157	60S ribosomal protein L5	PF17144.1^Ribosomal_L5e^Ribosomal large subunit proteins 60S L5, and 50S L18^14-175^E:3.4e-83`PF14204.3^Ribosomal_L18_c^Ribosomal L18 C-terminal region^192-280^E:2.5e-36	0,178	-0,599	0	2,293	1	-0,087	1	-0,046	0,404	-0,187	RL5_CUCSA	60S ribosomal protein L5	RPL5	Cucumis sativus (Cucumber)		RNA-binding; Ribonucleoprotein; Ribosomal protein; rRNA-binding
SRR122238_TR29998_c0_g2_i2_len=1776	UGT6_CATRO	Q:74-1534,H:1-487	99.59%ID	E:0	7-deoxyloganetin glucosyltransferase	PF00201.15^UDPGT^UDP-glucoronosyl and UDP-glucosyl transferase^97-447^E:3.6e-30	0,066	4,062	0	7,142	0,129	3,661	0,502	2,764	1	0,509	UGT6_CATRO	7-deoxyloganetin glucosyltransferase (EC 2.4.1.324) (Iridoid 1-O-glucosyltransferase) (UDP-glucose glucosyltransferase 6) (CrUGT6) (UDP-glycosyltransferase 85A23)	UGT85A23 UGT6	Catharanthus roseus (Madagascar periwinkle) (Vinca rosea)		Transferase
SRR122238_TR33097_c0_g2_i2_len=2686	SFH8_ARATH	Q:2340-484,H:1-620	76%ID	E:0	Phosphatidylinositol/phosphatidylcholine transfer protein SFH8	PF03765.12^CRAL_TRIO_N^CRAL/TRIO, N-terminal domain^108-131^E:1.3e-05`PF00650.17^CRAL_TRIO^CRAL/TRIO domain^155-321^E:8e-34	0,154	0,459	0	1,418	0,037	0,672	0,037	0,661	0,108	-0,612	SFH8_ARATH	Phosphatidylinositol/phosphatidylcholine transfer protein SFH8 (Protein SEC FOURTEEN HOMOLOGS 8) (AtSFH8)	SFH8 At2g21520 F3K23.28	Arabidopsis thaliana (Mouse-ear cress)		Alternative splicing; Cell membrane; Coiled coil; Complete proteome; Golgi apparatus; Membrane; Protein transport; Reference proteome; Transport
SRR122238_TR34562_c0_g2_i2_len=1584	IDH1_ARATH	Q:1420-290,H:1-367	84.88%ID	E:0	Isocitrate dehydrogenase [NAD] regulatory subunit 1, mitochondrial	PF00180.17^Iso_dh^Isocitrate/isopropylmalate dehydrogenase^50-372^E:2.3e-78	0,921	-0,088	0	1,382	0,415	0,357	0,601	0,242	0,666	-0,347	IDH1_ARATH	Isocitrate dehydrogenase [NAD] regulatory subunit 1, mitochondrial (EC 1.1.1.41) (IDH-I) (Isocitric dehydrogenase 1) (NAD(+)-specific ICDH 1)	IDH1 At4g35260 F23E12.180	Arabidopsis thaliana (Mouse-ear cress)		Complete proteome; Magnesium; Manganese; Metal-binding; Mitochondrion; NAD; Oxidoreductase; Reference proteome; Transit peptide; Tricarboxylic acid cycle
SRR122238_TR35386_c0_g1_i18_len=2319	2AAB_ARATH	Q:265-2025,H:1-587	94.38%ID	E:0	Serine/threonine-protein phosphatase 2A 65 kDa regulatory subunit A beta isoform	PF02985.19^HEAT^HEAT repeat^242-270^E:0.00019`PF13646.3^HEAT_2^HEAT repeats^361-461^E:2.2e-11`PF02985.19^HEAT^HEAT repeat^440-465^E:0.0027	0,756	-0,13	0	1,707	0,732	0,147	0,613	0,2	0,048	0,685	2AAB_ARATH	Serine/threonine-protein phosphatase 2A 65 kDa regulatory subunit A beta isoform (AtA beta) (PP2A, subunit A, beta isoform)	PP2AA2 DF1 At3g25800 K13N2.14	Arabidopsis thaliana (Mouse-ear cress)		Acetylation; Alternative splicing; Complete proteome; Reference proteome; Repeat
SRR122238_TR35731_c0_g1_i3_len=604	CD48D_ARATH	Q:505-47,H:1-153	91.5%ID	E:6e-95	Cell division control protein 48 homolog D	PF02359.15^CDC48_N^Cell division protein 48 (CDC48), N-terminal domain^63-144^E:3.6e-24	0,955	-0,032	0	1,901	0,744	0,113	0,744	0,122	0,019	0,834	CD48D_ARATH	Cell division control protein 48 homolog D (AtCDC48d) (Transitional endoplasmic reticulum ATPase D)	CDC48D At3g53230 T4D2.160	Arabidopsis thaliana (Mouse-ear cress)		ATP-binding; Acetylation; Cell cycle; Cell division; Complete proteome; Cytoplasm; Cytoskeleton; Nucleotide-binding; Nucleus; Phosphoprotein; Protein transport; Reference proteome; Repeat; Transport; Ubl conjugation
SRR122238_TR36321_c1_g2_i1_len=724	ADF5_ARATH	Q:582-154,H:1-143	86.71%ID	E:2e-86	Actin-depolymerizing factor 5	PF00241.17^Cofilin_ADF^Cofilin/tropomyosin-type actin-binding protein^65-187^E:4.2e-32	0,407	-0,468	0	2,836	1	-0,02	0,623	-0,342	1	1,139	ADF5_ARATH	Actin-depolymerizing factor 5 (ADF-5) (AtADF5)	ADF5 At2g16700 T24I21.11	Arabidopsis thaliana (Mouse-ear cress)		Actin-binding; Alternative splicing; Complete proteome; Reference proteome
SRR122238_TR36833_c0_g1_i6_len=949	VATO2_ARATH	Q:828-304,H:4-178	90.86%ID	E:2e-87	V-type proton ATPase subunit c''2	PF00137.18^ATP-synt_C^ATP synthase subunit C^23-82^E:6.1e-15`PF00137.18^ATP-synt_C^ATP synthase subunit C^107-166^E:4.5e-10	0,886	0,049	0	7,143	1	-0,217	1	-1,113	0,462	-0,204	VATO2_ARATH	V-type proton ATPase subunit c''2 (V-ATPase subunit c''2) (Vacuolar H(+)-ATPase subunit c'' isoform 2) (Vacuolar proton pump subunit c''2)	VHA-c''2 VMA16 At2g25610 F3N11.6	Arabidopsis thaliana (Mouse-ear cress)		Complete proteome; Endoplasmic reticulum; Golgi apparatus; Hydrogen ion transport; Ion transport; Membrane; Reference proteome; Transmembrane; Transmembrane helix; Transport
SRR122238_TR37722_c0_g1_i2_len=1208	IF4E3_WHEAT	Q:881-324,H:48-232	84.41%ID	E:1e-114	Eukaryotic translation initiation factor NCBP	PF01652.15^IF4E^Eukaryotic initiation factor 4E^58-217^E:1.3e-51	0,129	5,063	0	7,775	0,017	5,915	1	0	0,154	0,818	IF4E3_WHEAT	Eukaryotic translation initiation factor NCBP (Novel cap-binding protein) (nCBP) (mRNA cap-binding protein)	NCBP	Triticum aestivum (Wheat)		Complete proteome; Initiation factor; Protein biosynthesis; RNA-binding; Reference proteome; Translation regulation
SRR122238_TR41245_c1_g2_i13_len=2585	NA	NA	NA	NA	NA	.	0,694	0,36	0	7,096	1	-0,307	1	0,239	1	-0,021	.	.	.	.	.	.
SRR122238_TR41386_c0_g1_i2_len=1936	TAL_RENSM	Q:442-1485,H:12-366	50.42%ID	E:1e-96	Transaldolase {ECO:0000255|HAMAP-Rule:MF_00493}	PF00923.16^Transaldolase^Transaldolase^110-433^E:2.5e-78	0,962	-0,029	0	1,398	0,072	0,599	0,035	0,686	0,234	0,421	TAL_RENSM	Transaldolase (EC 2.2.1.2)	tal RSal33209_2247	Renibacterium salmoninarum (strain ATCC 33209 / DSM 20767 / JCM 11484 / NBRC 15589 / NCIMB 2235)	PATHWAY: Carbohydrate degradation; pentose phosphate pathway; D-glyceraldehyde 3-phosphate and beta-D-fructose 6-phosphate from D-ribose 5-phosphate and D-xylulose 5-phosphate (non-oxidative stage): step 2/3. {ECO:0000255|HAMAP-Rule:MF_00493}.	Complete proteome; Cytoplasm; Pentose shunt; Reference proteome; Schiff base; Transferase
SRR122238_TR42679_c0_g1_i1_len=751	ERG3_ORYSJ	Q:109-543,H:1-144	66.21%ID	E:9e-61	Elicitor-responsive protein 3	PF00168.27^C2^C2 domain^4-104^E:4.5e-21	0,025	1,467	0	3,338	0,004	2,1	0,059	1,561	1	-0,192	ERG3_ORYSJ	Elicitor-responsive protein 3 (16 kDa phloem protein) (RPP16)	ERG3 Os04g0531100 LOC_Os04g44870 OsJ_014914 OSJNBa0081C01.13	Oryza sativa subsp. japonica (Rice)		Complete proteome; Reference proteome
SRR122238_TR43809_c0_g1_i1_len=832	SDF2_ARATH	Q:141-536,H:84-215	78.79%ID	E:5e-74	Stromal cell-derived factor 2-like protein	PF02815.16^MIR^MIR domain^3-91^E:4.4e-06	0,524	-0,623	0,001	5,911	1	-0,017	1	-0,456	0,438	-0,398	SDF2_ARATH	Stromal cell-derived factor 2-like protein (AtSDF2) (SDF2-like protein)	SDF2 At2g25110 F13D4.70	Arabidopsis thaliana (Mouse-ear cress)		3D-structure; Complete proteome; Endoplasmic reticulum; Glycoprotein; Plant defense; Reference proteome; Repeat; Signal
SRR122238_TR45965_c0_g1_i1_len=1251	NA	NA	NA	NA	NA	.	0,226	1,862	0	2,253	0,03	1,051	0,002	1,386	1	0,123	.	.	.	.	.	.
SRR122238_TR47400_c1_g1_i2_len=1835	C72A1_CATRO	Q:106-1674,H:1-523	97.13%ID	E:0	Secologanin synthase	PF00067.19^p450^Cytochrome P450^90-497^E:3.1e-87	0,037	0,668	0	2,033	0,819	-0,16	0,329	-0,546	0,427	0,27	C72A1_CATRO	Secologanin synthase (SLS) (EC 1.3.3.9) (CYPLXXII) (Cytochrome P450 72A1)	CYP72A1 CYP72 P450CR3	Catharanthus roseus (Madagascar periwinkle) (Vinca rosea)	PATHWAY: Alkaloid biosynthesis; secologanin biosynthesis.	Alkaloid metabolism; Heme; Iron; Membrane; Metal-binding; Monooxygenase; NADP; Oxidoreductase; Transmembrane; Transmembrane helix; Vacuole
SRR122238_TR47649_c1_g1_i4_len=1154	NA	NA	NA	NA	NA	.	0,352	0,267	0	1,476	0,031	0,655	0,01	0,772	0,195	-0,344	.	.	.	.	.	.
SRR122238_TR50801_c0_g1_i2_len=1006	TOM20_SOLTU	Q:122-703,H:1-192	73.71%ID	E:9e-92	Mitochondrial import receptor subunit TOM20	PF06552.9^TOM20_plant^Plant specific mitochondrial import receptor subunit TOM20^7-193^E:1.7e-94	1	-0,015	0,001	0,956	0,034	0,68	0,093	0,558	0,902	-0,048	TOM20_SOLTU	Mitochondrial import receptor subunit TOM20 (Translocase of outer membrane 20 kDa subunit)	TOM20	Solanum tuberosum (Potato)		Complete proteome; Direct protein sequencing; Membrane; Mitochondrion; Mitochondrion outer membrane; Protein transport; Reference proteome; TPR repeat; Transmembrane; Transmembrane helix; Transport
SRR122238_TR54170_c2_g7_i6_len=1422	LEA14_SOYBN	Q:167-613,H:4-152	72.48%ID	E:5e-72	Desiccation protectant protein Lea14 homolog	PF03168.10^LEA_2^Late embryogenesis abundant protein^96-192^E:5.4e-18	0,692	0,595	0	3,458	0,133	1,13	0,187	1,04	0,708	0,175	LEA14_SOYBN	Desiccation protectant protein Lea14 homolog		Glycine max (Soybean) (Glycine hispida)		Complete proteome; Reference proteome
SRR122238_TR57409_c0_g1_i2_len=939	NA	NA	NA	NA	NA	.	0,56	0,861	0	2,855	0,471	0,569	0,707	0,441	1	0,54	.	.	.	.	.	.
SRR122238_TR57866_c0_g2_i1_len=3327	CDKF4_ORYSJ	Q:1072-1833,H:1-252	71.09%ID	E:2e-117	Cyclin-dependent kinase F-4	PF00069.22^Pkinase^Protein kinase domain^4-203^E:1.6e-67`PF07714.14^Pkinase_Tyr^Protein tyrosine kinase^6-195^E:3.1e-38	0,081	0,689	0	1,589	0,859	0,074	0,683	0,145	0,006	1,348	CDKF4_ORYSJ	Cyclin-dependent kinase F-4 (CDKF;4) (EC 2.7.11.22) (EC 2.7.11.23) (Serine/threonine-protein kinase MHK-like protein 2)	CDKF-4 Os02g0700600 LOC_Os02g47220 OJ1111_E07.13-1 P0459B01.42-1	Oryza sativa subsp. japonica (Rice)		ATP-binding; Complete proteome; Kinase; Nucleotide-binding; Phosphoprotein; Reference proteome; Serine/threonine-protein kinase; Transferase
SRR122238_TR59227_c0_g1_i2_len=725	NDBAA_ARATH	Q:132-401,H:10-99	88.89%ID	E:2e-52	NADH dehydrogenase [ubiquinone] 1 beta subcomplex subunit 10-A	.	0,803	-0,1	0	1,266	0,336	0,328	0,255	0,389	0,742	0,108	NDBAA_ARATH	NADH dehydrogenase [ubiquinone] 1 beta subcomplex subunit 10-A	At1g49140 F27J15.9	Arabidopsis thaliana (Mouse-ear cress)		Complete proteome; Electron transport; Membrane; Mitochondrion; Mitochondrion inner membrane; Reference proteome; Respiratory chain; Transport
SRR122238_TR61114_c0_g7_i1_len=2620	AROC2_SOLLC	Q:102-1166,H:34-388	88.17%ID	E:0	Chorismate synthase 2, chloroplastic	PF01264.18^Chorismate_synt^Chorismate synthase^57-387^E:8.5e-132	0,545	0,46	0,001	1,909	0,298	0,589	0,034	1,039	1	-0,002	AROC2_SOLLC	Chorismate synthase 2, chloroplastic (EC 4.2.3.5) (5-enolpyruvylshikimate-3-phosphate phospholyase 2)	CS2	Solanum lycopersicum (Tomato) (Lycopersicon esculentum)	PATHWAY: Metabolic intermediate biosynthesis; chorismate biosynthesis; chorismate from D-erythrose 4-phosphate and phosphoenolpyruvate: step 7/7.	Amino-acid biosynthesis; Aromatic amino acid biosynthesis; Chloroplast; Complete proteome; Lyase; Plastid; Reference proteome; Transit peptide
SRR122238_TR65674_c0_g1_i3_len=1260	FK126_ARATH	Q:1222-149,H:1-359	48.07%ID	E:3e-108	F-box/kelch-repeat protein At1g15670	PF00646.30^F-box^F-box domain^16-58^E:0.0011`PF13964.3^Kelch_6^Kelch motif^119-168^E:1.3e-07`PF01344.22^Kelch_1^Kelch motif^125-165^E:1e-05`PF01344.22^Kelch_1^Kelch motif^179-214^E:8e-08	1	-0,01	0	3,563	0,017	-4,193	0,017	-4,342	0,153	0,69	FK126_ARATH	F-box/kelch-repeat protein At1g15670	At1g15670 F7H2.1	Arabidopsis thaliana (Mouse-ear cress)		Complete proteome; Kelch repeat; Reference proteome; Repeat
SRR122238_TR67135_c0_g1_i2_len=3431	SND1_HUMAN	Q:3321-442,H:21-902	34.7%ID	E:3e-147	Staphylococcal nuclease domain-containing protein 1	PF00565.14^SNase^Staphylococcal nuclease homologue^65-173^E:4.9e-11`PF00565.14^SNase^Staphylococcal nuclease homologue^295-387^E:6e-13`PF00565.14^SNase^Staphylococcal nuclease homologue^434-574^E:1.6e-06`PF00565.14^SNase^Staphylococcal nuclease homologue^636-733^E:3.2e-13`PF00567.21^TUDOR^Tudor domain^753-876^E:7.8e-21`PF00565.14^SNase^Staphylococcal nuclease homologue^884-988^E:9.4e-08	0,626	-0,223	0	1,513	1	-0,081	0,77	-0,228	1	-0,377	SND1_HUMAN	Staphylococcal nuclease domain-containing protein 1 (100 kDa coactivator) (EBNA2 coactivator p100) (Tudor domain-containing protein 11) (p100 co-activator)	SND1 TDRD11	Homo sapiens (Human)		3D-structure; Acetylation; Complete proteome; Cytoplasm; Direct protein sequencing; Host-virus interaction; Nucleus; Phosphoprotein; Reference proteome; Repeat; Transcription; Transcription regulation
SRR122238_TR68263_c1_g1_i1_len=1439	NA	NA	NA	NA	NA	PF00076.19^RRM_1^RNA recognition motif. (a.k.a. RRM, RBD, or RNP domain)^7-66^E:1.5e-08	0,254	4,746	0	6,665	1	0	1	0	1	-3,406	.	.	.	.	.	.
SRR122238_TR73330_c1_g1_i2_len=557	UBQ11_ARATH	Q:1-555,H:34-218	100%ID	E:1e-129	Polyubiquitin 11	PF00240.20^ubiquitin^Ubiquitin family^1-41^E:3.3e-17`PF11976.5^Rad60-SLD^Ubiquitin-2 like Rad60 SUMO-like^2-38^E:2.6e-07`PF11976.5^Rad60-SLD^Ubiquitin-2 like Rad60 SUMO-like^44-114^E:3.7e-16`PF00240.20^ubiquitin^Ubiquitin family^46-117^E:5.4e-34`PF13881.3^Rad60-SLD_2^Ubiquitin-2 like Rad60 SUMO-like^53-114^E:0.062`PF14560.3^Ubiquitin_2^Ubiquitin-like domain^55-112^E:0.00024`PF11976.5^Rad60-SLD^Ubiquitin-2 like Rad60 SUMO-like^120-185^E:3.9e-14`PF00240.20^ubiquitin^Ubiquitin family^122-185^E:2.3e-29`PF14560.3^Ubiquitin_2^Ubiquitin-like domain^131-180^E:0.0032	0,857	0,063	0	1,405	0,725	0,125	0,905	0,048	0,008	1,019	UBQ11_ARATH	Polyubiquitin 11 [Cleaved into: Ubiquitin]	UBQ11 At4g05050 C17L7.6 T32N4.13	Arabidopsis thaliana (Mouse-ear cress)		Alternative splicing; Complete proteome; Cytoplasm; Isopeptide bond; Nucleus; Reference proteome; Repeat; Ubl conjugation; Ubl conjugation pathway
SRR122238_TR74772_c1_g1_i2_len=983	SRP14_ARATH	Q:121-462,H:1-114	74.56%ID	E:3e-45	Signal recognition particle 14 kDa protein	PF02290.12^SRP14^Signal recognition particle 14kD protein^4-93^E:3e-26	1	0	0	7,787	1	0	1	0	0,444	-0,484	SRP14_ARATH	Signal recognition particle 14 kDa protein (SRP14)	SRP14 At2g43640 F18O19.25	Arabidopsis thaliana (Mouse-ear cress)		Alternative splicing; Complete proteome; Cytoplasm; RNA-binding; Reference proteome; Ribonucleoprotein; Signal recognition particle
SRR122238_TR76412_c0_g1_i2_len=1501	NA	NA	NA	NA	NA	PF02341.12^RcbX^RbcX protein^98-198^E:2.2e-18	0,109	1,11	0	1,882	0,29	0,243	0,803	0,059	0,73	0,138	.	.	.	.	.	.
SRR122238_TR78053_c0_g1_i2_len=996	NA	NA	NA	NA	NA	PF06364.9^DUF1068^Protein of unknown function (DUF1068)^10-174^E:2.2e-64	0,626	0,23	0	7,591	1	0	1	0,508	0,581	-0,449	.	.	.	.	.	.
SRR122238_TR78962_c0_g3_i1_len=765	SNAK2_SOLTU	Q:581-333,H:22-104	68.67%ID	E:2e-33	Snakin-2	PF02704.11^GASA^Gibberellin regulated protein^49-108^E:2.1e-21	0,629	1,675	0	3,547	0,76	0,458	0,282	1,042	0,732	-0,631	SNAK2_SOLTU	Snakin-2	SN2	Solanum tuberosum (Potato)		Antimicrobial; Cell wall; Complete proteome; Direct protein sequencing; Disulfide bond; Plant defense; Reference proteome; Secreted; Signal
SRR122238_TR78977_c0_g1_i1_len=851	DNJ11_ARATH	Q:575-285,H:65-161	67.35%ID	E:5e-38	Chaperone protein dnaJ 11, chloroplastic	PF00226.28^DnaJ^DnaJ domain^94-157^E:1.9e-21	0,08	1,109	0	1,759	0,704	-0,179	0,051	-0,89	1	0,08	DNJ11_ARATH	Chaperone protein dnaJ 11, chloroplastic (AtDjC11) (AtJ11)	ATJ11 C11 J11 At4g36040 T19K4.170	Arabidopsis thaliana (Mouse-ear cress)		Chaperone; Chloroplast; Complete proteome; Direct protein sequencing; Plastid; Reference proteome; Transit peptide
SRR122238_TR79291_c1_g2_i2_len=2169	PIF4_ARATH	Q:146-1603,H:6-413	33.27%ID	E:8e-46	Transcription factor PIF4	PF00010.23^HLH^Helix-loop-helix DNA-binding domain^326-372^E:2e-13	0,025	0,61	0	1,645	0,054	0,536	0,003	0,801	0,234	-0,475	PIF4_ARATH	Transcription factor PIF4 (Basic helix-loop-helix protein 9) (AtbHLH9) (bHLH 9) (Phytochrome-interacting factor 4) (Short under red-light 2) (Transcription factor EN 102) (bHLH transcription factor bHLH009)	PIF4 BHLH9 EN102 SRL2 At2g43010 MFL8.13	Arabidopsis thaliana (Mouse-ear cress)		Alternative splicing; Complete proteome; DNA-binding; Nucleus; Phytochrome signaling pathway; Reference proteome; Transcription; Transcription regulation
SRR122238_TR79711_c1_g2_i1_len=1153	SERA1_ARATH	Q:59-946,H:308-603	86.15%ID	E:3e-160	D-3-phosphoglycerate dehydrogenase 1, chloroplastic	PF02826.16^2-Hacid_dh_C^D-isomer specific 2-hydroxyacid dehydrogenase, NAD binding domain^7-38^E:1.7e-07`PF01842.22^ACT^ACT domain^229-289^E:2e-07	0,183	0,392	0	2,912	0,011	0,86	0,17	0,5	0,031	-0,629	SERA1_ARATH	D-3-phosphoglycerate dehydrogenase 1, chloroplastic (EC 1.1.1.95) (Protein EMBRYO SAC DEVELOPMENT ARREST 9)	PGDH1 EDA9 At4g34200 F10M10.7	Arabidopsis thaliana (Mouse-ear cress)	PATHWAY: Amino-acid biosynthesis; L-serine biosynthesis; L-serine from 3-phospho-D-glycerate: step 1/3.	Chloroplast; Complete proteome; NAD; Oxidoreductase; Plastid; Reference proteome; Transit peptide
SRR122238_TR80349_c0_g1_i1_len=2880	NA	NA	NA	NA	NA	.	0,333	0,636	0	1,82	0,121	0,514	0,108	0,518	0,031	1,392	.	.	.	.	.	.
SRR122238_TR81963_c0_g1_i1_len=1197	KAI2_ARATH	Q:1029-229,H:1-267	79.78%ID	E:7e-161	Probable esterase KAI2	PF12146.5^Hydrolase_4^Serine aminopeptidase, S33^17-124^E:1.4e-07`PF00561.17^Abhydrolase_1^alpha/beta hydrolase fold^19-156^E:2.4e-11`PF12697.4^Abhydrolase_6^Alpha/beta hydrolase family^20-257^E:2e-11	0,76	0,443	0	3,634	1	-0,145	0,132	-2,334	0,732	0,695	KAI2_ARATH	Probable esterase KAI2 (Protein DWARF-14-like) (Protein D14-like) (Protein HYPOSENSITIVE TO LIGHT) (Protein KARRIKIN INSENSITIVE 2)	KAI2 D14L HTL At4g37470 F6G17.120	Arabidopsis thaliana (Mouse-ear cress)		3D-structure; Complete proteome; Cytoplasm; Hydrolase; Nucleus; Reference proteome
SRR122238_TR85786_c0_g1_i1_len=1017	NA	NA	NA	NA	NA	.	1	3,673	0	8,31	0,254	4,721	0,502	4,255	1	0	.	.	.	.	.	.
SRR122238_TR86442_c0_g1_i1_len=609	NA	NA	NA	NA	NA	.	0,533	0,25	0,001	1,088	0,936	0,032	0,804	-0,079	0,015	0,765	.	.	.	.	.	.
SRR122238_TR89533_c0_g1_i1_len=804	NA	NA	NA	NA	NA	.	1	-0,207	0	4,603	0,005	4,918	0,017	4,506	1	0,123	.	.	.	.	.	.
SRR122238_TR90465_c0_g1_i5_len=2093	ACOX4_ARATH	Q:1941-754,H:1-396	80.05%ID	E:0	Acyl-coenzyme A oxidase 4, peroxisomal	PF02771.13^Acyl-CoA_dh_N^Acyl-CoA dehydrogenase, N-terminal domain^56-165^E:1.2e-23`PF02770.16^Acyl-CoA_dh_M^Acyl-CoA dehydrogenase, middle domain^171-263^E:9.5e-23`PF00441.21^Acyl-CoA_dh_1^Acyl-CoA dehydrogenase, C-terminal domain^280-398^E:7.8e-22	0,189	1,699	0	7,46	0,132	2,206	0,132	2,353	1	-0,201	ACOX4_ARATH	Acyl-coenzyme A oxidase 4, peroxisomal (AOX 4) (EC 1.3.3.6) (G6p) (Short-chain acyl-CoA oxidase) (AtCX4) (AtG6) (SAOX)	ACX4 G6 At3g51840 ATEM1.9	Arabidopsis thaliana (Mouse-ear cress)		3D-structure; Acetylation; Complete proteome; FAD; Fatty acid metabolism; Flavoprotein; Glyoxysome; Lipid metabolism; Oxidoreductase; Peroxisome; Reference proteome
SRR1271857_TR3205_c1_g5_i1_len=996	TBA2_GOSHI	Q:783-457,H:1-109	94.5%ID	E:9e-113	Tubulin alpha-2 chain	PF00091.22^Tubulin^Tubulin/FtsZ family, GTPase domain^3-111^E:7.6e-29	0,662	0,115	0	2,17	0,707	0,34	0,044	1,174	1	1,76	TBA2_GOSHI	Tubulin alpha-2 chain (Alpha-2-tubulin)		Gossypium hirsutum (Upland cotton) (Gossypium mexicanum)		Acetylation; Cytoplasm; Cytoskeleton; GTP-binding; Microtubule; Nucleotide-binding
SRR1271857_TR3504_c0_g1_i3_len=1075	RL71_ARATH	Q:95-829,H:1-247	55.87%ID	E:5e-86	60S ribosomal protein L7-1	PF08079.9^Ribosomal_L30_N^Ribosomal L30 N-terminal domain^13-75^E:1.4e-09`PF00327.17^Ribosomal_L30^Ribosomal protein L30p/L7e^87-137^E:1.3e-17	0,044	-0,628	0	6,476	1	-2,34	1	0,979	0,753	-0,112	RL71_ARATH	60S ribosomal protein L7-1	RPL7A At1g80750 F23A5.10	Arabidopsis thaliana (Mouse-ear cress)		Complete proteome; Reference proteome; Ribonucleoprotein; Ribosomal protein
SRR1271857_TR3680_c3_g1_i1_len=987	UBIQP_AVEFA	Q:95-985,H:1-297	100%ID	E:0	Polyubiquitin	PF11976.5^Rad60-SLD^Ubiquitin-2 like Rad60 SUMO-like^32-102^E:1.1e-15`PF14560.3^Ubiquitin_2^Ubiquitin-like domain^32-100^E:0.00037`PF00240.20^ubiquitin^Ubiquitin family^34-105^E:1.6e-33`PF13881.3^Rad60-SLD_2^Ubiquitin-2 like Rad60 SUMO-like^41-102^E:0.14`PF11976.5^Rad60-SLD^Ubiquitin-2 like Rad60 SUMO-like^108-178^E:1.1e-15`PF00240.20^ubiquitin^Ubiquitin family^110-181^E:1.6e-33`PF14560.3^Ubiquitin_2^Ubiquitin-like domain^119-176^E:0.00069`PF13881.3^Rad60-SLD_2^Ubiquitin-2 like Rad60 SUMO-like^120-178^E:0.16`PF11976.5^Rad60-SLD^Ubiquitin-2 like Rad60 SUMO-like^184-254^E:1.1e-15`PF00240.20^ubiquitin^Ubiquitin family^186-257^E:1.6e-33`PF14560.3^Ubiquitin_2^Ubiquitin-like domain^195-252^E:0.00069`PF13881.3^Rad60-SLD_2^Ubiquitin-2 like Rad60 SUMO-like^196-254^E:0.16`PF11976.5^Rad60-SLD^Ubiquitin-2 like Rad60 SUMO-like^260-327^E:8e-15`PF00240.20^ubiquitin^Ubiquitin family^262-328^E:4.3e-31`PF14560.3^Ubiquitin_2^Ubiquitin-like domain^271-327^E:0.0011	0,55	0,129	0	1,334	0,023	0,486	0,193	0,28	0,023	0,5	UBIQP_AVEFA	Polyubiquitin [Cleaved into: Ubiquitin]		Avena fatua (Wild oats)		Cytoplasm; Isopeptide bond; Nucleus; Repeat; Ubl conjugation
SRR1271857_TR7086_c0_g2_i1_len=2109	GAT1_ARATH	Q:110-1444,H:4-451	60.49%ID	E:0	GABA transporter 1	PF01490.15^Aa_trans^Transmembrane amino acid transporter protein^38-445^E:5.2e-64	0,502	1,768	0	5,424	0,066	4,588	0,502	3,137	1	0,304	GAT1_ARATH	GABA transporter 1 (AtGAT1) (Bidirectional amino acid transporter 1)	GAT1 BAT1 At1g08230 T23G18.9	Arabidopsis thaliana (Mouse-ear cress)		Amino-acid transport; Cell membrane; Complete proteome; Membrane; Reference proteome; Transmembrane; Transmembrane helix; Transport
SRR1271857_TR8549_c0_g2_i1_len=1927	P2C44_ORYSJ	Q:583-1431,H:37-319	78.95%ID	E:2e-153	Probable protein phosphatase 2C 44	PF00481.18^PP2C^Protein phosphatase 2C^58-286^E:2.8e-56	0,195	0,898	0	2,873	0,189	2,128	0,118	2,19	0,798	0,509	P2C44_ORYSJ	Probable protein phosphatase 2C 44 (OsPP2C44) (EC 3.1.3.16)	Os04g0609600 LOC_Os04g52000 OSJNBa0085I10.2	Oryza sativa subsp. japonica (Rice)		Complete proteome; Hydrolase; Magnesium; Manganese; Metal-binding; Protein phosphatase; Reference proteome
SRR1271857_TR13296_c0_g2_i3_len=2112	SR542_SOLLC	Q:1190-390,H:213-479	93.63%ID	E:0	Signal recognition particle 54 kDa protein 2	PF00448.19^SRP54^SRP54-type protein, GTPase domain^19-105^E:3.1e-29`PF02978.16^SRP_SPB^Signal peptide binding domain^137-236^E:8.3e-28	1	0,006	0	4,5	1	-0,078	1	0,018	0,874	-0,175	SR542_SOLLC	Signal recognition particle 54 kDa protein 2 (SRP54)		Solanum lycopersicum (Tomato) (Lycopersicon esculentum)		Complete proteome; Cytoplasm; GTP-binding; Nucleotide-binding; RNA-binding; Reference proteome; Ribonucleoprotein; Signal recognition particle
SRR1271857_TR14185_c0_g1_i1_len=1569	CK1_ARATH	Q:353-1360,H:7-344	65.98%ID	E:2e-165	Probable choline kinase 1	PF01633.17^Choline_kinase^Choline/ethanolamine kinase^63-264^E:1.1e-70`PF01636.20^APH^Phosphotransferase enzyme family^107-255^E:9.8e-14`PF02958.17^EcKinase^Ecdysteroid kinase^170-240^E:7.4e-05	1	1,332	0	7,601	0,502	4,166	0,005	6,147	0,188	-0,727	CK1_ARATH	Probable choline kinase 1 (AtCK1) (EC 2.7.1.32)	CK1 CK At1g71697 F14O23.8 F26A9.3	Arabidopsis thaliana (Mouse-ear cress)	PATHWAY: Phospholipid metabolism; phosphatidylcholine biosynthesis; phosphocholine from choline: step 1/1.	ATP-binding; Complete proteome; Kinase; Lipid biosynthesis; Lipid metabolism; Nucleotide-binding; Phospholipid biosynthesis; Phospholipid metabolism; Reference proteome; Transferase
SRR1271857_TR17880_c0_g1_i1_len=1707	MBD10_ARATH	Q:420-734,H:5-107	71.43%ID	E:2e-42	Methyl-CpG-binding domain-containing protein 10	PF01429.16^MBD^Methyl-CpG binding domain^10-74^E:1.1e-11	0,1	0,497	0	2,154	0,014	0,972	0,026	0,892	0,072	0,525	MBD10_ARATH	Methyl-CpG-binding domain-containing protein 10 (AtMBD10) (MBD10) (Methyl-CpG-binding protein MBD10)	MBD10 At1g15340 F9L1.28	Arabidopsis thaliana (Mouse-ear cress)		Alternative splicing; Coiled coil; Complete proteome; DNA-binding; Nucleus; Phosphoprotein; Reference proteome; Transcription; Transcription regulation
SRR1271857_TR18163_c0_g2_i1_len=2913	AB25B_ARATH	Q:1240-2520,H:292-718	85.95%ID	E:0	ABC transporter B family member 25, mitochondrial	PF00664.20^ABC_membrane^ABC transporter transmembrane region^2-102^E:7.1e-17`PF00005.24^ABC_tran^ABC transporter^165-314^E:2.4e-36	1	-0,252	0	6,92	1	0,13	1	0,069	1	0,047	AB25B_ARATH	ABC transporter B family member 25, mitochondrial (ABC transporter ABCB.25) (AtABCB25) (ABC transporter of the mitochondrion 3) (AtATM3) (Iron-sulfur clusters transporter ATM3) (Protein STARIK 1)	ABCB25 ATM3 STA1 At5g58270 MCK7.14	Arabidopsis thaliana (Mouse-ear cress)	PATHWAY: Cofactor biosynthesis; molybdopterin biosynthesis.	ATP-binding; Complete proteome; Ion transport; Iron; Iron transport; Membrane; Mitochondrion; Mitochondrion inner membrane; Nucleotide-binding; Reference proteome; Transit peptide; Transmembrane; Transmembrane helix; Transport
SRR1271857_TR20542_c0_g1_i2_len=1999	ALMT9_ARATH	Q:590-1831,H:134-566	52.64%ID	E:7e-134	Aluminum-activated malate transporter 9	PF11744.5^ALMT^Aluminium activated malate transporter^2-392^E:2.6e-123	0,004	1,103	0	1,507	0,673	0,235	0,673	0,201	0,641	0,214	ALMT9_ARATH	Aluminum-activated malate transporter 9 (AtALMT9)	ALMT9 At3g18440 MYF24.16	Arabidopsis thaliana (Mouse-ear cress)		Chloride; Complete proteome; Ion channel; Ion transport; Membrane; Reference proteome; Transmembrane; Transmembrane helix; Transport; Vacuole
SRR1271857_TR20924_c0_g1_i1_len=2018	OXA1_ARATH	Q:317-1432,H:1-352	51.61%ID	E:2e-116	Mitochondrial inner membrane protein OXA1	PF02096.17^60KD_IMP^60Kd inner membrane protein^172-365^E:2.9e-28	0,826	0,118	0,001	1,621	0,235	0,921	0,235	0,955	0,356	1,313	OXA1_ARATH	Mitochondrial inner membrane protein OXA1 (Oxidase assembly 1 protein) (AtOXA1)	OXA1 ATOXA1 At5g62050 MTG10.19	Arabidopsis thaliana (Mouse-ear cress)		Complete proteome; Membrane; Mitochondrion; Mitochondrion inner membrane; Reference proteome; Transit peptide; Transmembrane; Transmembrane helix
SRR1271857_TR21360_c0_g1_i1_len=624	NA	NA	NA	NA	NA	.	0,44	0,749	0	2,345	0,189	1,778	0,044	2,316	0,9	0,114	.	.	.	.	.	.
SRR1271857_TR21402_c0_g1_i1_len=2349	GLPK_ARATH	Q:1383-172,H:119-522	80.69%ID	E:0	Glycerol kinase	PF00370.18^FGGY_N^FGGY family of carbohydrate kinases, N-terminal domain^3-110^E:1.1e-25`PF02782.13^FGGY_C^FGGY family of carbohydrate kinases, C-terminal domain^118-317^E:8.7e-60	0,787	-0,121	0	5,701	1	0,692	1	0,334	0,601	0,251	GLPK_ARATH	Glycerol kinase (Glycerokinase) (EC 2.7.1.30) (ATP:glycerol 3-phosphotransferase) (Protein GLYCEROL INSENSITIVE 1) (Protein NONHOST RESISTANCE TO P. S. PHASEOLICOLA 1)	GLPK GLI1 NHO1 At1g80460 T21F11.21	Arabidopsis thaliana (Mouse-ear cress)	PATHWAY: Polyol metabolism; glycerol degradation via glycerol kinase pathway; sn-glycerol 3-phosphate from glycerol: step 1/1.	ATP-binding; Alternative splicing; Complete proteome; Cytoplasm; Glycerol metabolism; Kinase; Nucleotide-binding; Plant defense; Reference proteome; Transferase
SRR1271857_TR21681_c0_g1_i1_len=2200	SRPR_DROME	Q:1055-129,H:293-614	59.63%ID	E:7e-120	Signal recognition particle receptor subunit alpha homolog	PF04086.10^SRP-alpha_N^Signal recognition particle, alpha subunit, N-terminal^28-302^E:4.8e-66`PF02881.16^SRP54_N^SRP54-type protein, helical bundle domain^319-386^E:1.2e-06`PF00448.19^SRP54^SRP54-type protein, GTPase domain^418-622^E:5.1e-55	0,925	-0,062	0	2,829	0,807	0,124	0,555	0,288	1	2,912	SRPR_DROME	Signal recognition particle receptor subunit alpha homolog (DP-alpha) (Docking protein alpha) (SR-alpha) (GTP-binding protein)	Gtp-bp CG2522	Drosophila melanogaster (Fruit fly)		Complete proteome; Endoplasmic reticulum; GTP-binding; Membrane; Nucleotide-binding; Phosphoprotein; Receptor; Reference proteome
SRR1271857_TR27022_c0_g1_i8_len=4421	EXEC1_ARATH	Q:1377-2804,H:196-683	53.99%ID	E:2e-138	Protein EXECUTER 1, chloroplastic {ECO:0000305}	PF12014.5^DUF3506^Domain of unknown function (DUF3506)^327-455^E:4.7e-25	1	3,503	0	8,432	0,254	4,67	1	3,179	0,254	4,623	EXEC1_ARATH	Protein EXECUTER 1, chloroplastic (AtEX1)	EX1 At4g33630 T16L1.120	Arabidopsis thaliana (Mouse-ear cress)		Chloroplast; Complete proteome; Plastid; Reference proteome; Stress response; Transit peptide
SRR1271857_TR29183_c0_g1_i1_len=869	HA22E_ARATH	Q:226-546,H:1-106	75.7%ID	E:4e-37	HVA22-like protein e	PF03134.16^TB2_DP1_HVA22^TB2/DP1, HVA22 family^24-98^E:3.8e-26	0,381	1,609	0	4,836	0,356	1,367	1	0,443	1	1,571	HA22E_ARATH	HVA22-like protein e (AtHVA22e)	HVA22E At5g50720 MFB16.12	Arabidopsis thaliana (Mouse-ear cress)		Complete proteome; Membrane; Reference proteome; Stress response; Transmembrane; Transmembrane helix
SRR1271857_TR30166_c0_g1_i4_len=3392	POT11_ARATH	Q:820-3177,H:13-792	78.17%ID	E:0	Potassium transporter 11	PF02705.13^K_trans^K+ potassium transporter^53-624^E:5.6e-189	0,14	0,65	0	2,664	0,071	0,9	0,012	1,185	0,38	0,484	POT11_ARATH	Potassium transporter 11 (AtPOT11)	POT11 KUP11 At2g35060 F19I3.29	Arabidopsis thaliana (Mouse-ear cress)		Alternative splicing; Cell membrane; Complete proteome; Glycoprotein; Ion transport; Membrane; Potassium; Potassium transport; Reference proteome; Transmembrane; Transmembrane helix; Transport
SRR1271857_TR35199_c3_g5_i4_len=4852	PSBC_TOBAC	Q:3227-4645,H:1-473	99.79%ID	E:0	Photosystem II CP43 reaction center protein {ECO:0000255|HAMAP-Rule:MF_01496}	PF00421.16^PSII^Photosystem II protein^31-469^E:1.3e-150	0,068	1,052	0	3,085	0,112	-0,481	0,964	0,024	1	0	PSBC_TOBAC	Photosystem II CP43 reaction center protein (PSII 43 kDa protein) (Protein CP-43)	psbC	Nicotiana tabacum (Common tobacco)		Acetylation; Chlorophyll; Chloroplast; Chromophore; Manganese; Membrane; Metal-binding; Phosphoprotein; Photosynthesis; Photosystem II; Plastid; Thylakoid; Transmembrane; Transmembrane helix
SRR1271857_TR36799_c0_g1_i6_len=608	Y1864_ARATH	Q:588-28,H:140-320	55.85%ID	E:4e-65	Probable receptor-like protein kinase At1g80640	PF00069.22^Pkinase^Protein kinase domain^1-98^E:3.4e-21`PF07714.14^Pkinase_Tyr^Protein tyrosine kinase^1-101^E:1.3e-17	0,214	0,779	0	6,939	1	0	1	0	0,611	0,41	Y1864_ARATH	Probable receptor-like protein kinase At1g80640 (EC 2.7.11.1)	At1g80640 T21F11.3	Arabidopsis thaliana (Mouse-ear cress)		ATP-binding; Alternative splicing; Cell membrane; Complete proteome; Kinase; Membrane; Nucleotide-binding; Phosphoprotein; Reference proteome; Serine/threonine-protein kinase; Signal; Transferase; Transmembrane; Transmembrane helix
SRR1271857_TR36846_c0_g1_i2_len=1436	APG2_ARATH	Q:1183-203,H:27-353	72.78%ID	E:1e-164	GDSL esterase/lipase APG	PF00657.19^Lipase_GDSL^GDSL-like Lipase/Acylhydrolase^35-347^E:3e-17	0,357	0,596	0	7,239	0,517	0,75	0,002	2,416	0,129	5,037	APG2_ARATH	GDSL esterase/lipase APG (EC 3.1.1.-) (Extracellular lipase APG)	APG At3g16370 MYA6.18 T2O4.2	Arabidopsis thaliana (Mouse-ear cress)		Complete proteome; Glycoprotein; Hydrolase; Lipid degradation; Lipid metabolism; Reference proteome; Secreted; Signal
SRR1271857_TR38503_c0_g1_i1_len=2402	MPK9_ARATH	Q:529-2082,H:1-510	80.61%ID	E:0	Mitogen-activated protein kinase 9	PF00069.22^Pkinase^Protein kinase domain^106-397^E:3.6e-68`PF07714.14^Pkinase_Tyr^Protein tyrosine kinase^108-318^E:4.9e-31`PF06293.11^Kdo^Lipopolysaccharide kinase (Kdo/WaaP) family^139-251^E:0.00012	0,87	0,149	0,001	3,402	0,756	-0,193	0,756	-0,233	0,694	0,365	MPK9_ARATH	Mitogen-activated protein kinase 9 (AtMPK9) (MAP kinase 9) (EC 2.7.11.24)	MPK9 At3g18040 MRC8.2 MRC8.4	Arabidopsis thaliana (Mouse-ear cress)		ATP-binding; Alternative splicing; Complete proteome; Kinase; Nucleotide-binding; Phosphoprotein; Reference proteome; Serine/threonine-protein kinase; Transferase
SRR1271857_TR39898_c2_g1_i5_len=2263	GH35_ORYSJ	Q:413-2125,H:7-579	65.85%ID	E:0	Jasmonic acid-amido synthetase JAR1 {ECO:0000305}	PF03321.10^GH3^GH3 auxin-responsive promoter^19-560^E:4.4e-194	0,005	1,393	0	3,908	0,025	1,976	0,402	1,106	0,763	0,266	GH35_ORYSJ	Jasmonic acid-amido synthetase JAR1 (EC 6.3.2.-) (Auxin-responsive GH3-like protein 5) (OsGH3-5) (Indole-3-acetic acid-amido synthetase GH3.5) (Jasmonate-amino acid synthetase JAR1) (Protein JASMONATE RESISTANT 1) (OsJAR1)	GH3.5 JAR1 Os05g0586200 LOC_Os05g50890 OsJ_19714 OSJNBa0009C07.16	Oryza sativa subsp. japonica (Rice)		Complete proteome; Developmental protein; Growth regulation; Jasmonic acid signaling pathway; Ligase; Plant defense; Reference proteome; Stress response
SRR1271857_TR41074_c0_g2_i1_len=1167	C72A1_CATRO	Q:195-935,H:1-250	51.2%ID	E:1e-84	Secologanin synthase	PF00067.19^p450^Cytochrome P450^91-231^E:1.8e-13	0,704	0,162	0,001	2,545	0,059	1,099	0,298	0,65	0,002	1,45	C72A1_CATRO	Secologanin synthase (SLS) (EC 1.3.3.9) (CYPLXXII) (Cytochrome P450 72A1)	CYP72A1 CYP72 P450CR3	Catharanthus roseus (Madagascar periwinkle) (Vinca rosea)	PATHWAY: Alkaloid biosynthesis; secologanin biosynthesis.	Alkaloid metabolism; Heme; Iron; Membrane; Metal-binding; Monooxygenase; NADP; Oxidoreductase; Transmembrane; Transmembrane helix; Vacuole
SRR1271857_TR41235_c0_g1_i2_len=588	GRP1_SINAL	Q:81-335,H:1-85	87.06%ID	E:1e-45	Glycine-rich RNA-binding protein GRP1A	PF00076.19^RRM_1^RNA recognition motif. (a.k.a. RRM, RBD, or RNP domain)^37-105^E:2.2e-23`PF13893.3^RRM_5^RNA recognition motif. (a.k.a. RRM, RBD, or RNP domain)^47-107^E:0.013	0,008	-0,562	0	1,113	0,405	-0,317	0,627	-0,201	0,216	0,261	GRP1_SINAL	Glycine-rich RNA-binding protein GRP1A		Sinapis alba (White mustard) (Brassica hirta)		Nucleus; RNA-binding
SRR1271857_TR43774_c0_g1_i2_len=610	PRS2_SOLTU	Q:88-555,H:1-155	37.5%ID	E:9e-25	Pathogenesis-related protein STH-2	PF00407.16^Bet_v_1^Pathogenesis-related protein Bet v I family^1-150^E:1.6e-11	0,472	0,661	0	3,15	0,058	0,738	0,041	0,808	0,694	-0,345	PRS2_SOLTU	Pathogenesis-related protein STH-2	STH-2	Solanum tuberosum (Potato)		Complete proteome; Pathogenesis-related protein; Plant defense; Reference proteome
SRR1271858_TR1781_c0_g3_i3_len=1427	COMT1_CATRO	Q:171-977,H:1-269	97.77%ID	E:0	Caffeic acid 3-O-methyltransferase	PF08100.8^Dimerisation^Dimerisation domain^34-85^E:7e-23`PF00891.15^Methyltransf_2^O-methyltransferase^129-266^E:2e-54	0,86	0,231	0,001	2,099	0,071	0,876	0,219	0,644	0,133	-1,079	COMT1_CATRO	Caffeic acid 3-O-methyltransferase (CAOMT) (COMT) (EC 2.1.1.68) (S-adenosysl-L-methionine:caffeic acid 3-O-methyltransferase)	COMT1	Catharanthus roseus (Madagascar periwinkle) (Vinca rosea)	PATHWAY: Aromatic compound metabolism; phenylpropanoid biosynthesis.	Lignin biosynthesis; Methyltransferase; S-adenosyl-L-methionine; Transferase
SRR1271858_TR2709_c3_g4_i1_len=2025	EF1A_TOBAC	Q:140-1444,H:1-435	97.93%ID	E:0	Elongation factor 1-alpha	PF00009.24^GTP_EFTU^Elongation factor Tu GTP binding domain^52-267^E:1.3e-52`PF03144.22^GTP_EFTU_D2^Elongation factor Tu domain 2^294-359^E:8.4e-15`PF03143.14^GTP_EFTU_D3^Elongation factor Tu C-terminal domain^368-475^E:4.6e-38	0,093	-0,438	0	1,307	0,568	0,193	0,418	0,266	0,007	-0,722	EF1A_TOBAC	Elongation factor 1-alpha (EF-1-alpha) (Vitronectin-like adhesion protein 1) (PVN1)		Nicotiana tabacum (Common tobacco)		Cytoplasm; Elongation factor; GTP-binding; Nucleotide-binding; Phosphoprotein; Protein biosynthesis
SRR1271858_TR3122_c0_g1_i1_len=3235	EBF1_ARATH	Q:377-2380,H:1-627	56.74%ID	E:0	EIN3-binding F-box protein 1	PF00646.30^F-box^F-box domain^65-105^E:8.4e-06`PF13516.3^LRR_6^Leucine Rich repeat^265-288^E:0.79	1	0,007	0,001	1,394	0,497	0,24	0,42	0,278	0,02	1,109	EBF1_ARATH	EIN3-binding F-box protein 1 (F-box/LRR-repeat protein 6)	EBF1 FBL6 At2g25490 F13B15.15	Arabidopsis thaliana (Mouse-ear cress)	PATHWAY: Protein modification; protein ubiquitination.	Complete proteome; Ethylene signaling pathway; Nucleus; Reference proteome; Ubl conjugation pathway
SRR1271858_TR4206_c0_g1_i1_len=1374	FB119_ARATH	Q:1192-218,H:11-337	42.43%ID	E:1e-75	F-box protein At2g27310	PF00646.30^F-box^F-box domain^13-53^E:2.1e-05	0,226	2,086	0	3,5	0,076	3,293	0,001	4,195	1	-1,186	FB119_ARATH	F-box protein At2g27310	At2g27310 F12K2.11	Arabidopsis thaliana (Mouse-ear cress)		Complete proteome; Reference proteome
SRR1271858_TR5205_c0_g1_i1_len=2234	NA	NA	NA	NA	NA	PF00847.17^AP2^AP2 domain^244-293^E:4.3e-14	0,009	4,732	0	8,763	0,004	3,607	0,132	2,624	1	0	.	.	.	.	.	.
SRR1271858_TR6830_c0_g2_i2_len=1089	GSTX6_SOYBN	Q:267-911,H:1-216	50.46%ID	E:1e-67	Probable glutathione S-transferase	PF02798.17^GST_N^Glutathione S-transferase, N-terminal domain^6-77^E:7.2e-22`PF13417.3^GST_N_3^Glutathione S-transferase, N-terminal domain^9-84^E:4.7e-18`PF13409.3^GST_N_2^Glutathione S-transferase, N-terminal domain^15-78^E:2.9e-14`PF00043.22^GST_C^Glutathione S-transferase, C-terminal domain^105-194^E:2.2e-09`PF14497.3^GST_C_3^Glutathione S-transferase, C-terminal domain^112-201^E:8.2e-06`PF13410.3^GST_C_2^Glutathione S-transferase, C-terminal domain^123-190^E:1.8e-07	1	0,193	0	3,487	0,043	3,559	0,629	2,256	1	-2,625	GSTX6_SOYBN	Probable glutathione S-transferase (EC 2.5.1.18) (G2-4) (Heat shock protein 26A)	HSP26-A	Glycine max (Soybean) (Glycine hispida)		Complete proteome; Reference proteome; Stress response; Transferase
SRR1271858_TR6830_c0_g2_i3_len=577	GST23_MAIZE	Q:61-423,H:102-222	37.19%ID	E:3e-22	Glutathione transferase GST 23	PF00043.22^GST_C^Glutathione S-transferase, C-terminal domain^11-87^E:1.8e-09`PF14497.3^GST_C_3^Glutathione S-transferase, C-terminal domain^13-94^E:2.5e-06`PF13410.3^GST_C_2^Glutathione S-transferase, C-terminal domain^16-83^E:4.4e-08	1	1,25	0	4,728	0,034	3,912	0,034	3,77	1	-1,049	GST23_MAIZE	Glutathione transferase GST 23 (EC 2.5.1.18) (Glutathione transferase GST 36)		Zea mays (Maize)		Complete proteome; Plant defense; Reference proteome; Transferase
SRR1271858_TR7354_c0_g1_i1_len=2018	AROD6_ARATH	Q:567-1631,H:28-402	80.74%ID	E:0	Arogenate dehydratase/prephenate dehydratase 6, chloroplastic	PF00800.15^PDT^Prephenate dehydratase^149-327^E:1.5e-59	0,308	0,43	0	1,651	0,382	0,34	0,088	0,621	0,547	-0,323	AROD6_ARATH	Arogenate dehydratase/prephenate dehydratase 6, chloroplastic (AtADT6) (AtPDT6) (EC 4.2.1.51) (EC 4.2.1.91)	ADT6 PDT6 At1g08250 T23G18.10	Arabidopsis thaliana (Mouse-ear cress)	PATHWAY: Amino-acid biosynthesis; L-phenylalanine biosynthesis; L-phenylalanine from L-arogenate: step 1/1.; PATHWAY: Amino-acid biosynthesis; L-phenylalanine biosynthesis; phenylpyruvate from prephenate: step 1/1.	Amino-acid biosynthesis; Aromatic amino acid biosynthesis; Chloroplast; Complete proteome; Lyase; Phenylalanine biosynthesis; Plastid; Reference proteome; Transit peptide
SRR1271858_TR7861_c0_g2_i1_len=1022	NA	NA	NA	NA	NA	.	0,402	0,912	0	2,808	0,407	0,651	1	0,055	0,132	1,434	.	.	.	.	.	.
SRR1271858_TR8366_c1_g1_i1_len=2765	HSP80_SOLLC	Q:2637-544,H:1-699	92.85%ID	E:0	Heat shock cognate protein 80	PF13589.3^HATPase_c_3^Histidine kinase-, DNA gyrase B-, and HSP90-like ATPase^25-146^E:2.2e-10`PF02518.23^HATPase_c^Histidine kinase-, DNA gyrase B-, and HSP90-like ATPase^27-181^E:5.6e-16`PF00183.15^HSP90^Hsp90 protein^184-690^E:1.9e-239	0,266	-0,249	0	1,251	0,018	0,533	0,004	0,649	0,465	0,184	HSP80_SOLLC	Heat shock cognate protein 80	HSC80	Solanum lycopersicum (Tomato) (Lycopersicon esculentum)		ATP-binding; Chaperone; Complete proteome; Cytoplasm; Nucleotide-binding; Reference proteome; Stress response
SRR1271858_TR9519_c0_g2_i22_len=3787	ACOC_ORYSJ	Q:1869-3299,H:404-880	88.47%ID	E:0	Putative aconitate hydratase, cytoplasmic	PF00330.17^Aconitase^Aconitase family (aconitate hydratase)^185-514^E:8.2e-124	0,535	-0,238	0	1,933	0,52	0,419	0,623	0,356	0,195	0,614	ACOC_ORYSJ	Putative aconitate hydratase, cytoplasmic (Aconitase) (EC 4.2.1.3) (Citrate hydro-lyase)	Os08g0191100 LOC_Os08g09200 P0610E02.32	Oryza sativa subsp. japonica (Rice)	PATHWAY: Carbohydrate metabolism; glyoxylate and dicarboxylate metabolism.	4Fe-4S; Complete proteome; Cytoplasm; Glyoxylate bypass; Iron; Iron-sulfur; Lyase; Metal-binding; Reference proteome
SRR1271858_TR9833_c0_g1_i8_len=2562	SRG1_ARATH	Q:180-1187,H:16-358	39.48%ID	E:5e-68	Protein SRG1	PF14226.3^DIOX_N^non-haem dioxygenase in morphine synthesis N-terminal^53-158^E:1e-25`PF03171.17^2OG-FeII_Oxy^2OG-Fe(II) oxygenase superfamily^207-303^E:4.6e-23	0,381	2,179	0	5,252	0,003	3,412	0,226	2,032	1	-1,331	SRG1_ARATH	Protein SRG1 (AtSRG1) (Protein SENESCENCE-RELATED GENE 1)	SRG1 At1g17020 F20D23.28 F6I1.30	Arabidopsis thaliana (Mouse-ear cress)		Complete proteome; Iron; Metal-binding; Oxidoreductase; Reference proteome
SRR1271858_TR15896_c1_g1_i1_len=846	NA	NA	NA	NA	NA	.	0,732	0,761	0	3,904	0,118	1,642	0,189	1,369	0,663	-0,366	.	.	.	.	.	.
SRR1271858_TR16005_c0_g2_i1_len=3261	ENPL_CATRO	Q:490-2940,H:1-817	98.41%ID	E:0	Endoplasmin homolog	PF02518.23^HATPase_c^Histidine kinase-, DNA gyrase B-, and HSP90-like ATPase^101-256^E:2.1e-14`PF13589.3^HATPase_c_3^Histidine kinase-, DNA gyrase B-, and HSP90-like ATPase^103-221^E:1.4e-09`PF00183.15^HSP90^Hsp90 protein^259-799^E:1.9e-219	0,683	-0,126	0	1,539	0,513	0,201	0,12	-0,49	0,054	0,697	ENPL_CATRO	Endoplasmin homolog (Glucose-regulated protein 94 homolog) (GRP-94 homolog)	HSP90	Catharanthus roseus (Madagascar periwinkle) (Vinca rosea)		ATP-binding; Calcium; Chaperone; Endoplasmic reticulum; Glycoprotein; Nucleotide-binding; Signal
SRR1271858_TR16071_c0_g1_i23_len=3124	SUSY_SOLLC	Q:1415-2827,H:335-805	91.08%ID	E:0	Sucrose synthase	PF00862.16^Sucrose_synth^Sucrose synthase^1-167^E:2.3e-104`PF00534.17^Glycos_transf_1^Glycosyl transferases group 1^178-351^E:6.9e-34`PF13692.3^Glyco_trans_1_4^Glycosyl transferases group 1^185-338^E:2.2e-11	0,398	0,419	0	1,52	0,389	0,327	1	0,024	0,273	0,375	SUSY_SOLLC	Sucrose synthase (EC 2.4.1.13) (Sucrose-UDP glucosyltransferase)		Solanum lycopersicum (Tomato) (Lycopersicon esculentum)		Complete proteome; Glycosyltransferase; Reference proteome; Transferase
SRR1271858_TR18698_c0_g2_i1_len=2106	RETOL_ARATH	Q:1732-350,H:32-482	56.25%ID	E:3e-166	Reticuline oxidase-like protein	PF01565.20^FAD_binding_4^FAD binding domain^80-215^E:3.4e-26	0,319	1,063	0	2,794	0,593	0,73	0,111	1,479	1	0,089	RETOL_ARATH	Reticuline oxidase-like protein (EC 1.21.-.-)	At4g20830 F21C20.180	Arabidopsis thaliana (Mouse-ear cress)		Alternative splicing; Cell membrane; Complete proteome; FAD; Flavoprotein; GPI-anchor; Glycoprotein; Lipoprotein; Membrane; Oxidoreductase; Reference proteome; Secreted; Signal
SRR1271858_TR18769_c0_g1_i13_len=2006	ACOX4_ARATH	Q:1010-1726,H:193-431	86.19%ID	E:4e-148	Acyl-coenzyme A oxidase 4, peroxisomal	PF02771.13^Acyl-CoA_dh_N^Acyl-CoA dehydrogenase, N-terminal domain^56-147^E:7.9e-16	0,052	1,542	0	2,429	0,001	3,147	0,008	2,783	0,807	0,161	ACOX4_ARATH	Acyl-coenzyme A oxidase 4, peroxisomal (AOX 4) (EC 1.3.3.6) (G6p) (Short-chain acyl-CoA oxidase) (AtCX4) (AtG6) (SAOX)	ACX4 G6 At3g51840 ATEM1.9	Arabidopsis thaliana (Mouse-ear cress)		3D-structure; Acetylation; Complete proteome; FAD; Fatty acid metabolism; Flavoprotein; Glyoxysome; Lipid metabolism; Oxidoreductase; Peroxisome; Reference proteome
SRR1271858_TR19089_c2_g1_i25_len=5906	AB5C_ARATH	Q:4945-428,H:12-1514	79.15%ID	E:0	ABC transporter C family member 5	PF00664.20^ABC_membrane^ABC transporter transmembrane region^336-600^E:7.1e-29`PF00005.24^ABC_tran^ABC transporter^664-798^E:2.1e-18`PF00664.20^ABC_membrane^ABC transporter transmembrane region^983-1217^E:2.1e-26`PF00005.24^ABC_tran^ABC transporter^1310-1456^E:1.1e-28	0,115	0,729	0	3,686	0,233	0,748	0,032	1,231	0,057	1,372	AB5C_ARATH	ABC transporter C family member 5 (ABC transporter ABCC.5) (AtABCC5) (EC 3.6.3.44) (ATP-energized glutathione S-conjugate pump 5) (Glutathione S-conjugate-transporting ATPase 5) (Multidrug resistance-associated protein 5)	ABCC5 MRP5 At1g04120 F20D22.11	Arabidopsis thaliana (Mouse-ear cress)		ATP-binding; Alternative splicing; Coiled coil; Complete proteome; Hydrolase; Membrane; Nucleotide-binding; Reference proteome; Repeat; Transmembrane; Transmembrane helix; Transport
SRR1271858_TR19955_c2_g2_i4_len=966	NA	NA	NA	NA	NA	.	0,86	0,117	0	8,695	1	-1,468	1	-2,462	1	-0,039	.	.	.	.	.	.
SRR1271858_TR20550_c0_g1_i4_len=1155	PER25_ARATH	Q:298-1062,H:74-328	74.12%ID	E:2e-134	Peroxidase 25	.	1	3,342	0	4,806	0,502	4,187	0,017	5,769	1	-1,143	PER25_ARATH	Peroxidase 25 (Atperox P25) (EC 1.11.1.7)	PER25 P25 At2g41480 T26J13.7	Arabidopsis thaliana (Mouse-ear cress)		Calcium; Complete proteome; Disulfide bond; Glycoprotein; Heme; Hydrogen peroxide; Iron; Metal-binding; Oxidoreductase; Peroxidase; Reference proteome; Secreted; Signal
SRR1271858_TR21646_c0_g2_i1_len=1981	NA	NA	NA	NA	NA	.	0,321	0,384	0	6,352	0	-7,616	0	-7,616	0,164	-0,513	.	.	.	.	.	.
SRR1271858_TR21966_c4_g2_i2_len=634	UBQ3_ARATH	Q:1-633,H:53-263	99.53%ID	E:7e-146	Polyubiquitin 3	PF11976.5^Rad60-SLD^Ubiquitin-2 like Rad60 SUMO-like^25-95^E:4.9e-16`PF00240.20^ubiquitin^Ubiquitin family^27-98^E:7.1e-34`PF13881.3^Rad60-SLD_2^Ubiquitin-2 like Rad60 SUMO-like^35-95^E:0.08`PF14560.3^Ubiquitin_2^Ubiquitin-like domain^36-93^E:0.00031`PF11976.5^Rad60-SLD^Ubiquitin-2 like Rad60 SUMO-like^101-171^E:4.9e-16`PF00240.20^ubiquitin^Ubiquitin family^103-174^E:7.1e-34`PF13881.3^Rad60-SLD_2^Ubiquitin-2 like Rad60 SUMO-like^111-171^E:0.08`PF14560.3^Ubiquitin_2^Ubiquitin-like domain^112-169^E:0.00031`PF00240.20^ubiquitin^Ubiquitin family^179-211^E:1.7e-09	0,389	0,195	0	0,79	0,002	0,68	0,143	0,329	0,806	0,059	UBQ3_ARATH	Polyubiquitin 3 [Cleaved into: Ubiquitin]	UBQ3 At5g03240 F15A17_270 MOK16.15	Arabidopsis thaliana (Mouse-ear cress)		Complete proteome; Cytoplasm; Isopeptide bond; Nucleus; Reference proteome; Repeat; Ubl conjugation; Ubl conjugation pathway
SRR1271858_TR22444_c0_g1_i1_len=2791	TMN11_ARATH	Q:272-2242,H:1-658	84.5%ID	E:0	Transmembrane 9 superfamily member 11 {ECO:0000305}	PF02990.13^EMP70^Endomembrane protein 70^58-612^E:1.1e-183	0,457	0,231	0	1,411	0,564	0,192	0,782	-0,118	0,663	0,162	TMN11_ARATH	Transmembrane 9 superfamily member 11 (Endomembrane protein 6) (Transmembrane nine protein 11) (AtTMN11)	TMN11 EMP6 At5g35160 T13C12.17	Arabidopsis thaliana (Mouse-ear cress)		Alternative splicing; Complete proteome; Endosome; Golgi apparatus; Membrane; Reference proteome; Signal; Transmembrane; Transmembrane helix
SRR1271858_TR25959_c0_g1_i4_len=4795	NA	NA	NA	NA	NA	.	1	0,1	0,001	2,627	0,298	0,689	0,471	0,463	1	0	.	.	.	.	.	.
SRR1271858_TR25983_c0_g1_i1_len=1093	NA	NA	NA	NA	NA	PF14009.3^DUF4228^Domain of unknown function (DUF4228)^1-215^E:2.2e-27	0,076	2,811	0	3,767	0,103	1,478	0,004	2,226	0,356	0,958	.	.	.	.	.	.
SRR1271858_TR26578_c0_g1_i3_len=2678	NA	NA	NA	NA	NA	.	0,394	0,492	0	1,85	0,038	1,024	0,099	0,87	0,823	-0,152	.	.	.	.	.	.
SRR1271858_TR26622_c0_g1_i4_len=840	HIP26_ARATH	Q:365-793,H:2-153	48.03%ID	E:1e-41	Heavy metal-associated isoprenylated plant protein 26	PF00403.23^HMA^Heavy-metal-associated domain^28-84^E:1.5e-14	0,154	0,814	0	2,148	0,777	-0,157	0,266	-0,506	1	-0,025	HIP26_ARATH	Heavy metal-associated isoprenylated plant protein 26 (AtHIPP26) (Farnesylated protein 6) (AtFP6)	HIPP26 FP6 At4g38580 F20M13.140	Arabidopsis thaliana (Mouse-ear cress)		Cell membrane; Complete proteome; Lipoprotein; Membrane; Metal-binding; Methylation; Nucleus; Prenylation; Reference proteome
SRR1271858_TR28728_c0_g1_i1_len=871	NA	NA	NA	NA	NA	PF03168.10^LEA_2^Late embryogenesis abundant protein^77-177^E:5.8e-09	0,003	0,722	0	0,927	0,051	0,434	0,651	0,105	1	0,239	.	.	.	.	.	.
SRR1271858_TR30[illegible]	PDAT1_ARATH	Q:454-[illegible]	[illegible]	[illegible]	[illegible]	PF02450.12^LCAT^Lecithin:cholesterol acyltransferase^129-370^E:5.7e-45`PF02450.12^LCAT^Lecithin:cholesterol [illegible]	[illegible]	[illegible]	[illegible]	[illegible]	[illegible]	[illegible]	[illegible]	[illegible]	[illegible]	[illegible]	[illegible]	[illegible]	PDAT1 PDAT At5g13640 MSH12.10	[illegible]	[illegible]	Acyltransferase; Complete proteome; Glycerol metabolism; Glycoprotein; Lipid [illegible]

[illegible]	PDAT1_ARATH	[illegible]2340,H:47-671	[illegible]	E:0	Phospholipid:diacylglycerol acyltransferase 1	[illegible] acyltransferase^473-630^E:9.9e-19	[illegible]	[illegible]	0	[illegible]	[illegible]	[illegible]	[illegible]	[illegible]	[illegible]	[illegible]	PDAT1_ARATH	Phospholipid:diacylglycerol acyltransferase 1 (AtPDAT1) (EC 2.3.1.158)	[illegible] T6I14.2	Arabidopsis thaliana (Mouse-ear cress)	PATHWAY: Glycerolipid metabolism; triacylglycerol biosynthesis.	[illegible] biosynthesis; Lipid metabolism; Membrane; Reference proteome; Transferase; Transmembrane; Transmembrane helix
SRR1271858_TR32130_c0_g1_i1_len=748	CB12_SOLLC	Q:32-694,H:46-266	91.4%ID	E:1e-148	Chlorophyll a-b binding protein 7, chloroplastic	PF00504.18^Chloroa_b-bind^Chlorophyll A-B binding protein^39-205^E:6.8e-52	0	-6,679	0	7,321	0,001	-6,538	0,352	-1,041	1	0,048	CB12_SOLLC	Chlorophyll a-b binding protein 7, chloroplastic (LHCI type II CAB-7)	CAB7	Solanum lycopersicum (Tomato) (Lycopersicon esculentum)		Chlorophyll; Chloroplast; Chromophore; Complete proteome; Magnesium; Membrane; Metal-binding; Phosphoprotein; Photosynthesis; Photosystem I; Photosystem II; Plastid; Reference proteome; Thylakoid; Transit peptide; Transmembrane; Transmembrane helix
SRR1271858_TR34670_c0_g1_i3_len=1808	METK3_CATRO	Q:1588-419,H:1-390	99.49%ID	E:0	S-adenosylmethionine synthase 3	PF00438.17^S-AdoMet_synt_N^S-adenosylmethionine synthetase, N-terminal domain^4-101^E:1.5e-42`PF02772.13^S-AdoMet_synt_M^S-adenosylmethionine synthetase, central domain^117-238^E:6.3e-47`PF02773.13^S-AdoMet_synt_C^S-adenosylmethionine synthetase, C-terminal domain^240-381^E:6.8e-63	0,92	-0,044	0,001	1,086	0,221	-0,369	0,166	-0,421	0,152	-0,431	METK3_CATRO	S-adenosylmethionine synthase 3 (AdoMet synthase 3) (EC 2.5.1.6) (Methionine adenosyltransferase 3) (MAT 3)	SAMS3	Catharanthus roseus (Madagascar periwinkle) (Vinca rosea)	PATHWAY: Amino-acid biosynthesis; S-adenosyl-L-methionine biosynthesis; S-adenosyl-L-methionine from L-methionine: step 1/1.	ATP-binding; Cobalt; Cytoplasm; Magnesium; Manganese; Metal-binding; Nucleotide-binding; One-carbon metabolism; Potassium; Transferase
SRR1271858_TR36855_c0_g1_i1_len=1654	TRPB_CAMAC	Q:1654-584,H:110-466	93.84%ID	E:0	Tryptophan synthase beta chain 2, chloroplastic	PF00291.22^PALP^Pyridoxal-phosphate dependent enzyme^13-342^E:1.5e-49	0,118	-1,697	0	7,676	0,034	5,672	1	0	1	0	TRPB_CAMAC	Tryptophan synthase beta chain 2, chloroplastic (EC 4.2.1.20)	TSB	Camptotheca acuminata (Happy tree)	PATHWAY: Amino-acid biosynthesis; L-tryptophan biosynthesis; L-tryptophan from chorismate: step 5/5.	Amino-acid biosynthesis; Aromatic amino acid biosynthesis; Chloroplast; Lyase; Plastid; Pyridoxal phosphate; Transit peptide; Tryptophan biosynthesis
SRR1271858_TR41214_c0_g1_i1_len=2975	METE_CATRO	Q:143-2668,H:1-765	90.14%ID	E:0	5-methyltetrahydropteroyltriglutamate--homocysteine methyltransferase	PF08267.9^Meth_synt_1^Cobalamin-independent synthase, N-terminal domain^3-315^E:1.9e-121`PF01717.15^Meth_synt_2^Cobalamin-independent synthase, Catalytic domain^432-576^E:2.1e-68	0,42	0,274	0	2,46	0,51	-0,329	0,37	-0,43	0,05	0,473	METE_CATRO	5-methyltetrahydropteroyltriglutamate--homocysteine methyltransferase (EC 2.1.1.14) (Cobalamin-independent methionine synthase isozyme) (Vitamin-B12-independent methionine synthase isozyme)	METE	Catharanthus roseus (Madagascar periwinkle) (Vinca rosea)	PATHWAY: Amino-acid biosynthesis; L-methionine biosynthesis via de novo pathway; L-methionine from L-homocysteine (MetE route): step 1/1.	Amino-acid biosynthesis; Cytoplasm; Metal-binding; Methionine biosynthesis; Methyltransferase; Transferase; Zinc
SRR1271858_TR41214_c0_g1_i5_len=2963	METE_CATRO	Q:143-2656,H:1-765	90.57%ID	E:0	5-methyltetrahydropteroyltriglutamate--homocysteine methyltransferase	PF08267.9^Meth_synt_1^Cobalamin-independent synthase, N-terminal domain^3-315^E:1.2e-121`PF01717.15^Meth_synt_2^Cobalamin-independent synthase, Catalytic domain^432-492^E:6.5e-20	0,876	0,085	0	1,717	0,206	-0,638	0,6	-0,259	0,543	0,151	METE_CATRO	5-methyltetrahydropteroyltriglutamate--homocysteine methyltransferase (EC 2.1.1.14) (Cobalamin-independent methionine synthase isozyme) (Vitamin-B12-independent methionine synthase isozyme)	METE	Catharanthus roseus (Madagascar periwinkle) (Vinca rosea)	PATHWAY: Amino-acid biosynthesis; L-methionine biosynthesis via de novo pathway; L-methionine from L-homocysteine (MetE route): step 1/1.	Amino-acid biosynthesis; Cytoplasm; Metal-binding; Methionine biosynthesis; Methyltransferase; Transferase; Zinc
SRR1271858_TR41800_c0_g1_i3_len=3104	TLC1_SOLTU	Q:630-2153,H:1-511	85.52%ID	E:0	Plastidic ATP/ADP-transporter	PF03219.11^TLC^TLC ATP/ADP transporter^100-509^E:4.6e-170	0,634	0,225	0	1,946	1	0,9	1	0,259	0,155	0,472	TLC1_SOLTU	Plastidic ATP/ADP-transporter		Solanum tuberosum (Potato)		ATP-binding; Chloroplast; Complete proteome; Membrane; Nucleotide-binding; Plastid; Reference proteome; Transmembrane; Transmembrane helix; Transport
SRR1271858_TR42899_c0_g3_i1_len=3309	TPS11_ARATH	Q:579-3101,H:10-835	69.31%ID	E:0	Probable alpha,alpha-trehalose-phosphate synthase [UDP-forming] 11	PF00982.18^Glyco_transf_20^Glycosyltransferase family 20^63-549^E:2e-184`PF02358.13^Trehalose_PPase^Trehalose-phosphatase^599-832^E:3.3e-77`PF08282.9^Hydrolase_3^haloacid dehalogenase-like hydrolase^727-803^E:3e-05	0,564	0,361	0,001	2,391	0,189	1,669	0,118	1,815	0,472	0,708	TPS11_ARATH	Probable alpha,alpha-trehalose-phosphate synthase [UDP-forming] 11 (EC 2.4.1.15) (Trehalose-6-phosphate synthase 11) (AtTPS11)	TPS11 TPSB At2g18700 MSF3.8	Arabidopsis thaliana (Mouse-ear cress)		Alternative splicing; Complete proteome; Glycosyltransferase; Phosphoprotein; Reference proteome; Transferase
SRR1271858_TR43396_c1_g1_i3_len=2746	PTR28_ARATH	Q:2437-1532,H:4-302	63.58%ID	E:2e-122	Protein NRT1/ PTR FAMILY 5.6	PF00854.18^PTR2^POT family^112-275^E:3.2e-41	0,254	3,969	0,001	6,584	0,001	6,433	0,034	5,633	1	0	PTR28_ARATH	Protein NRT1/ PTR FAMILY 5.6 (AtNPF5.6)	NPF5.6 At2g37900 T8P21.19	Arabidopsis thaliana (Mouse-ear cress)		Complete proteome; Membrane; Phosphoprotein; Reference proteome; Transmembrane; Transmembrane helix; Transport
SRR1271858_TR43523_c0_g2_i1_len=1206	NB5R1_ARATH	Q:231-986,H:30-281	88.89%ID	E:2e-166	NADH--cytochrome b5 reductase 1	PF00970.21^FAD_binding_6^Oxidoreductase FAD-binding domain^46-144^E:8.3e-33`PF00175.18^NAD_binding_1^Oxidoreductase NAD-binding domain^154-260^E:3.4e-31	0,044	0,54	0	1,676	0,019	0,65	0,065	0,52	0,465	-0,21	NB5R1_ARATH	NADH--cytochrome b5 reductase 1 (EC 1.6.2.2)	CBR1 CBR At5g17770 MVA3.13	Arabidopsis thaliana (Mouse-ear cress)		Complete proteome; FAD; Flavoprotein; Membrane; Mitochondrion; Mitochondrion outer membrane; NAD; Oxidoreductase; Phosphoprotein; Reference proteome; Transmembrane; Transmembrane helix
SRR1271858_TR46029_c0_g1_i1_len=822	NA	NA	NA	NA	NA	.	0,254	3,758	0,001	5	0,043	3,142	0,381	2,394	1	-1,059	.	.	.	.	.	.
SRR1271858_TR48059_c0_g4_i3_len=1423	MTDH_FRAAN	Q:1257-220,H:8-353	64.74%ID	E:9e-119	Probable mannitol dehydrogenase	PF08240.9^ADH_N^Alcohol dehydrogenase GroES-like domain^32-144^E:5.2e-27`PF02826.16^2-Hacid_dh_C^D-isomer specific 2-hydroxyacid dehydrogenase, NAD binding domain^167-217^E:1.1e-06`PF00107.23^ADH_zinc_N^Zinc-binding dehydrogenase^188-311^E:2.8e-19	0,006	0,607	0	2,982	0,119	0,372	0,035	-0,518	0,34	0,461	MTDH_FRAAN	Probable mannitol dehydrogenase (EC 1.1.1.255) (NAD-dependent mannitol dehydrogenase)	CAD	Fragaria ananassa (Strawberry)		Metal-binding; NAD; Oxidoreductase; Zinc
SRR1271858_TR48059_c0_g4_i12_len=1243	10HGO_CATRO	Q:1074-889,H:11-72	74.19%ID	E:1e-23	8-hydroxygeraniol dehydrogenase	.	0,047	0,71	0	2,274	0,641	0,252	0,268	-0,647	1	0,004	10HGO_CATRO	8-hydroxygeraniol dehydrogenase (Cr10HGO) (EC 1.1.1.324)	10HGO	Catharanthus roseus (Madagascar periwinkle) (Vinca rosea)		Metal-binding; NADP; Oxidoreductase; Zinc
SRR1271858_TR48572_c0_g1_i1_len=2696	ABKC_DICDI	Q:1653-367,H:158-631	34.5%ID	E:9e-84	Probable serine/threonine-protein kinase abkC	PF03109.13^ABC1^ABC1 family^275-399^E:1.3e-31	0,505	-0,486	0	1,821	1	0,091	1	0,062	0,86	0,21	ABKC_DICDI	Probable serine/threonine-protein kinase abkC (EC 2.7.11.-)	abkC adckC DDB_G0267774	Dictyostelium discoideum (Slime mold)		ATP-binding; Coiled coil; Complete proteome; Kinase; Nucleotide-binding; Reference proteome; Serine/threonine-protein kinase; Transferase
SRR1271858_TR48586_c1_g1_i3_len=2124	DCE_PETHY	Q:283-1740,H:1-485	86.83%ID	E:0	Glutamate decarboxylase	PF00282.16^Pyridoxal_deC^Pyridoxal-dependent decarboxylase conserved domain^33-381^E:1.8e-110	0,195	1,352	0	3,95	0,402	1,045	0,195	1,309	0,76	0,631	DCE_PETHY	Glutamate decarboxylase (GAD) (EC 4.1.1.15)	GAD	Petunia hybrida (Petunia)		3D-structure; Calmodulin-binding; Decarboxylase; Lyase; Pyridoxal phosphate
SRR1271858_TR50158_c0_g1_i3_len=1789	B561J_ARATH	Q:1452-385,H:24-384	55.4%ID	E:5e-140	Cytochrome b561 and DOMON domain-containing protein At5g47530	PF04526.10^DUF568^Protein of unknown function (DUF568)^89-186^E:1.6e-23`PF03188.13^Cytochrom_B561^Eukaryotic cytochrome b561^207-332^E:8.4e-06	0,026	2,218	0	3,778	0,002	2,768	0,118	1,773	1	0,099	B561J_ARATH	Cytochrome b561 and DOMON domain-containing protein At5g47530 (Protein b561A.tha10)	At5g47530 MNJ7.12	Arabidopsis thaliana (Mouse-ear cress)		Complete proteome; Electron transport; Heme; Iron; Membrane; Metal-binding; Reference proteome; Signal; Transmembrane; Transmembrane helix; Transport
SRR1271858_TR51869_c0_g1_i1_len=1317	SGT1_ORYSJ	Q:1193-105,H:5-367	66.85%ID	E:1e-149	Protein SGT1 homolog {ECO:0000250|UniProtKB:Q08446}	PF13181.3^TPR_8^Tetratricopeptide repeat^37-69^E:0.00066`PF13432.3^TPR_16^Tetratricopeptide repeat^42-97^E:0.0004`PF13181.3^TPR_8^Tetratricopeptide repeat^71-101^E:0.0045`PF04969.13^CS^CS domain^167-242^E:2.3e-14`PF05002.12^SGS^SGS domain^282-362^E:4.4e-37	0,156	-0,572	0	1,927	0,758	0,159	0,139	0,604	1	0,668	SGT1_ORYSJ	Protein SGT1 homolog (OsSGT1) (Suppressor of G2 allele of SKP1 homolog)	SGT1 Os01g0624500 LOC_Os01g43540 OsJ_02663	Oryza sativa subsp. japonica (Rice)		Complete proteome; Cytoplasm; Nucleus; Plant defense; Reference proteome; Repeat; TPR repeat; Ubl conjugation pathway
SRR1271858_TR52271_c0_g1_i7_len=1364	AOC4_ARATH	Q:798-388,H:111-248	71.74%ID	E:6e-58	Allene oxide cyclase 4, chloroplastic	.	0,254	3,247	0	7,683	0,034	4,655	0,002	5,395	1	0,592	AOC4_ARATH	Allene oxide cyclase 4, chloroplastic (EC 5.3.99.6)	AOC4 At1g13280 T6J4.4 T6J4_23	Arabidopsis thaliana (Mouse-ear cress)		Chloroplast; Complete proteome; Isomerase; Plastid; Reference proteome; Transit peptide
SRR1271858_TR52573_c0_g1_i1_len=570	NA	NA	NA	NA	NA	.	1	1,397	0	3,865	0,629	2,311	1	0,742	1	-0,347	.	.	.	.	.	.
SRR1271858_TR52658_c0_g2_i1_len=949	NA	NA	NA	NA	NA	PF13664.3^DUF4149^Domain of unknown function (DUF4149)^47-144^E:3.1e-24	0,135	0,662	0	4,303	0,123	0,75	0,443	0,416	0,158	1,778	.	.	.	.	.	.
SRR1271858_TR54040_c0_g1_i3_len=4108	AROF_ARATH	Q:3912-3115,H:1-269	68.4%ID	E:6e-113	Phospho-2-dehydro-3-deoxyheptonate aldolase 1, chloroplastic	PF01474.13^DAHP_synth_2^Class-II DAHP synthetase family^137-331^E:9.5e-78	0,072	0,715	0	2,702	0,045	0,875	0,298	0,528	0,059	1,093	AROF_ARATH	Phospho-2-dehydro-3-deoxyheptonate aldolase 1, chloroplastic (EC 2.5.1.54) (3-deoxy-D-arabino-heptulosonate 7-phosphate synthase 1) (DAHP synthase 1) (Phospho-2-keto-3-deoxyheptonate aldolase 1)	DHS1 At4g39980 T5J17.150	Arabidopsis thaliana (Mouse-ear cress)	PATHWAY: Metabolic intermediate biosynthesis; chorismate biosynthesis; chorismate from D-erythrose 4-phosphate and phosphoenolpyruvate: step 1/7.	Amino-acid biosynthesis; Aromatic amino acid biosynthesis; Chloroplast; Complete proteome; Plastid; Reference proteome; Transferase; Transit peptide
SRR1271858_TR54152_c0_g1_i2_len=1045	NAC68_ORYSJ	Q:225-803,H:13-216	30.62%ID	E:4e-23	NAC domain-containing protein 68	PF02365.12^NAM^No apical meristem (NAM) protein^8-135^E:8e-21	1	1,52	0	6,119	0,066	3,313	0,254	2,739	1	0	NAC68_ORYSJ	NAC domain-containing protein 68 (ONAC068)	NAC68 NAC4 Os01g0816100 LOC_Os01g60020 B1148D12.1 OSJNBa0085D07.44	Oryza sativa subsp. japonica (Rice)		Complete proteome; DNA-binding; Nucleus; Reference proteome; Transcription; Transcription regulation
SRR1271858_TR55679_c0_g2_i2_len=2894	SERB2_ARATH	Q:2705-1827,H:18-305	75.59%ID	E:4e-153	Phosphoserine aminotransferase 2, chloroplastic	PF12146.5^Hydrolase_4^Serine aminopeptidase, S33^62-323^E:3.5e-13`PF00561.17^Abhydrolase_1^alpha/beta hydrolase fold^66-326^E:3.2e-13`PF12697.4^Abhydrolase_6^Alpha/beta hydrolase family^67-327^E:5.7e-15	0,555	0,258	0	2,367	0,832	0,174	0,545	0,418	0,025	3,089	SERB2_ARATH	Phosphoserine aminotransferase 2, chloroplastic (AtPSAT2) (EC 2.6.1.52)	PSAT2 At2g17630 T19E12.3	Arabidopsis thaliana (Mouse-ear cress)	PATHWAY: Amino-acid biosynthesis; L-serine biosynthesis; L-serine from 3-phospho-D-glycerate: step 2/3.	Acetylation; Amino-acid biosynthesis; Aminotransferase; Chloroplast; Complete proteome; Plastid; Pyridoxal phosphate; Reference proteome; Serine biosynthesis; Transferase; Transit peptide
SRR1271858_TR56848_c0_g1_i1_len=1619	NA	NA	NA	NA	NA	PF02270.12^TFIIF_beta^Transcription initiation factor IIF, beta subunit^15-111^E:9.8e-07`PF02270.12^TFIIF_beta^Transcription initiation factor IIF, beta subunit^109-259^E:4.4e-34	0,9	0,139	0	2,488	0,057	1,291	0,028	1,419	0,518	0,385	.	.	.	.	.	.
SRR1271858_TR57629_c1_g1_i1_len=1200	GEDH1_OCIBA	Q:7-1065,H:3-358	61.8%ID	E:4e-153	Geraniol dehydrogenase 1	PF08240.9^ADH_N^Alcohol dehydrogenase GroES-like domain^38-147^E:3.5e-23`PF00107.23^ADH_zinc_N^Zinc-binding dehydrogenase^191-314^E:1.3e-15	0,034	5,007	0	7,7	1	0,506	1	-1,506	1	0	GEDH1_OCIBA	Geraniol dehydrogenase 1 (ObaGEDH1) (EC 1.1.1.183)	GEDH1	Ocimum basilicum (Sweet basil)		Metal-binding; NADP; Oxidoreductase; Zinc
SRR1271858_TR59296_c0_g1_i2_len=1962	MDAR_SOLLC	Q:1875-484,H:1-433	80.82%ID	E:0	Monodehydroascorbate reductase	PF07992.11^Pyr_redox_2^Pyridine nucleotide-disulphide oxidoreductase^10-201^E:3.6e-39`PF00070.24^Pyr_redox^Pyridine nucleotide-disulphide oxidoreductase^44-126^E:4.8e-14	0,398	0,41	0	1,963	0,006	1,926	0,045	1,554	0,088	1,507	MDAR_SOLLC	Monodehydroascorbate reductase (MDAR) (EC 1.6.5.4) (Ascorbate free radical reductase) (AFR reductase)	AFRR	Solanum lycopersicum (Tomato) (Lycopersicon esculentum)		Complete proteome; Cytoplasm; FAD; Flavoprotein; NAD; Oxidoreductase; Redox-active center; Reference proteome
SRR1271858_TR59410_c0_g1_i2_len=1630	C7352_ARATH	Q:1357-59,H:88-510	37.61%ID	E:4e-79	Cytokinin hydroxylase	PF00067.19^p450^Cytochrome P450^15-426^E:4.9e-82	1	2,29	0	6,648	1	2,441	1	2,615	1	-1,098	C7352_ARATH	Cytokinin hydroxylase (EC 1.14.13.-) (Cytochrome P450 35A2)	CYP735A2 At1g67110 F5A8.3	Arabidopsis thaliana (Mouse-ear cress)		Complete proteome; Heme; Iron; Membrane; Metal-binding; Monooxygenase; NADP; Oxidoreductase; Reference proteome; Transmembrane; Transmembrane helix
SRR1271858_TR62188_c0_g1_i8_len=2292	SRPR_DROME	Q:1055-129,H:293-614	59.63%ID	E:2e-119	Signal recognition particle receptor subunit alpha homolog	PF04086.10^SRP-alpha_N^Signal recognition particle, alpha subunit, N-terminal^28-302^E:4.8e-66`PF02881.16^SRP54_N^SRP54-type protein, helical bundle domain^319-386^E:1.2e-06`PF00448.19^SRP54^SRP54-type protein, GTPase domain^418-622^E:5.1e-55	0,324	0,376	0	1,434	0,014	0,901	0,13	0,581	0,389	0,36	SRPR_DROME	Signal recognition particle receptor subunit alpha homolog (DP-alpha) (Docking protein alpha) (SR-alpha) (GTP-binding protein)	Gtp-bp CG2522	Drosophila melanogaster (Fruit fly)		Complete proteome; Endoplasmic reticulum; GTP-binding; Membrane; Nucleotide-binding; Phosphoprotein; Receptor; Reference proteome
SRR1271858_TR62488_c1_g1_i3_len=596	RS23_FRAAN	Q:445-44,H:1-134	98.51%ID	E:8e-91	40S ribosomal protein S23	PF00164.22^Ribosom_S12_S23^Ribosomal protein S12/S23^9-135^E:5.3e-57	0,004	-1,225	0	1,751	0,03	-1,059	0,008	-1,343	1	0	RS23_FRAAN	40S ribosomal protein S23 (S12)	RPS23	Fragaria ananassa (Strawberry)		Hydroxylation; Ribonucleoprotein; Ribosomal protein
SRR1271858_TR65065_c0_g2_i2_len=1798	ACCH1_ARATH	Q:1063-203,H:80-365	48.96%ID	E:2e-92	1-aminocyclopropane-1-carboxylate oxidase homolog 1	PF14226.3^DIOX_N^non-haem dioxygenase in morphine synthesis N-terminal^26-94^E:2.7e-07`PF03171.17^2OG-FeII_Oxy^2OG-Fe(II) oxygenase superfamily^153-245^E:1.4e-22	0,129	4,66	0	7,266	0,254	4,459	1	3,124	1	0	ACCH1_ARATH	1-aminocyclopropane-1-carboxylate oxidase homolog 1	At1g06620 F12K11.24 F12K11.6	Arabidopsis thaliana (Mouse-ear cress)		Complete proteome; Iron; Metal-binding; Oxidoreductase; Reference proteome
SRR1271858_TR66001_c0_g1_i2_len=2746	AIM1_ARATH	Q:1287-211,H:354-712	73.26%ID	E:2e-176	Peroxisomal fatty acid beta-oxidation multifunctional protein AIM1	PF00378.17^ECH_1^Enoyl-CoA hydratase/isomerase^80-282^E:1.6e-37`PF16113.2^ECH_2^Enoyl-CoA hydratase/isomerase^85-281^E:1.9e-20`PF02737.15^3HCDH_N^3-hydroxyacyl-CoA dehydrogenase, NAD binding domain^377-420^E:1.9e-07	0,226	2,263	0	2,576	0,517	0,963	0,356	1,315	1	0,032	AIM1_ARATH	Peroxisomal fatty acid beta-oxidation multifunctional protein AIM1 (Protein ABNORMAL INFLORESCENCE MERISTEM 1) (AtAIM1) [Includes: Enoyl-CoA hydratase/3-2-trans-enoyl-CoA isomerase/3-hydroxybutyryl-CoA epimerase (EC 4.2.1.17) (EC 5.1.2.3) (EC 5.3.3.8); 3-hydroxyacyl-CoA dehydrogenase (EC 1.1.1.35)]	AIM1 At4g29010 F19B15.40	Arabidopsis thaliana (Mouse-ear cress)	PATHWAY: Lipid metabolism; fatty acid beta-oxidation.	Complete proteome; Fatty acid metabolism; Isomerase; Lipid metabolism; Lyase; Multifunctional enzyme; NAD; Oxidoreductase; Peroxisome; Reference proteome
SRR1271858_TR66262_c2_g1_i19_len=3904	4CLL5_ARATH	Q:208-1839,H:3-542	70.77%ID	E:0	4-coumarate--CoA ligase-like 5	PF00501.25^AMP-binding^AMP-binding enzyme^53-461^E:8.2e-103`PF13193.3^AMP-binding_C^AMP-binding enzyme C-terminal domain^470-545^E:1.3e-16	0,032	1,216	0	2,728	0,046	0,897	0,108	0,762	0,147	0,892	4CLL5_ARATH	4-coumarate--CoA ligase-like 5 (EC 6.2.1.-) (4-coumarate--CoA ligase isoform 9) (At4CL9) (Peroxisomal OPC-8:0-CoA ligase 1)	4CLL5 OPCL1 At1g20510 F5M15.17	Arabidopsis thaliana (Mouse-ear cress)		ATP-binding; Alternative splicing; Complete proteome; Ligase; Nucleotide-binding; Peroxisome; Reference proteome
SRR1271858_TR68514_c0_g1_i1_len=713	NA	NA	NA	NA	NA	.	0,774	0,126	0	2,337	0,03	0,709	0,01	0,821	0	-1,268	.	.	.	.	.	.
SRR1271858_TR69965_c1_g1_i5_len=1187	CAMT4_ARATH	Q:139-981,H:15-259	79.72%ID	E:8e-158	Caffeoyl-CoA O-methyltransferase 1	PF01596.14^Methyltransf_3^O-methyltransferase^10-150^E:2.2e-65	0,239	1,278	0	3,189	0,407	0,579	0,407	0,652	0,072	0,72	CAMT4_ARATH	Caffeoyl-CoA O-methyltransferase 1 (EC 2.1.1.104) (Trans-caffeoyl-CoA 3-O-methyltransferase) (CCoAMT) (CCoAOMT1)	CCOAOMT1 At4g34050 F28A23.190	Arabidopsis thaliana (Mouse-ear cress)	PATHWAY: Aromatic compound metabolism; phenylpropanoid biosynthesis.	Acetylation; Alternative splicing; Complete proteome; Lignin biosynthesis; Metal-binding; Methyltransferase; Reference proteome; S-adenosyl-L-methionine; Transferase
SRR1271859_TR622_c0_g1_i1_len=3438	SND1_DANRE	Q:3210-1231,H:11-616	37.07%ID	E:9e-106	Staphylococcal nuclease domain-containing protein 1	PF00565.14^SNase^Staphylococcal nuclease homologue^40-148^E:2.7e-11`PF00565.14^SNase^Staphylococcal nuclease homologue^270-362^E:3.3e-13`PF00565.14^SNase^Staphylococcal nuclease homologue^409-549^E:8.3e-07	0,788	0,157	0	2,396	1	0,13	1	0,183	1	0,009	SND1_DANRE	Staphylococcal nuclease domain-containing protein 1 (100 kDa coactivator) (4SNc-Tudor domain protein) (p100 co-activator)	snd1 sn4tdr	Danio rerio (Zebrafish) (Brachydanio rerio)		Alternative splicing; Complete proteome; Cytoplasm; Reference proteome; Repeat
SRR1271859_TR815_c0_g1_i2_len=1144	NA	NA	NA	NA	NA	.	0,026	0,548	0	1,113	0,144	0,367	0,45	0,197	0,009	0,608	.	.	.	.	.	.
SRR1271859_TR1178_c0_g1_i1_len=1795	BZR1_ARATH	Q:448-1335,H:22-335	63.06%ID	E:2e-78	Protein BRASSINAZOLE-RESISTANT 1	PF05687.10^BES1_N^BES1/BZR1 plant transcription factor, N-terminal^34-159^E:6.2e-61	0,581	0,335	0	2,237	0,043	1,352	0,195	1,01	1	-0,069	BZR1_ARATH	Protein BRASSINAZOLE-RESISTANT 1 (Protein BIN2 SUBSTRATE 2)	BZR1 BIS2 At1g75080 F9E10_7	Arabidopsis thaliana (Mouse-ear cress)		Brassinosteroid signaling pathway; Complete proteome; DNA-binding; Growth regulation; Nucleus; Phosphoprotein; Reference proteome; Transcription; Transcription regulation
SRR1271859_TR1593_c0_g1_i1_len=1501	TRL2_ORYSJ	Q:421-930,H:36-202	72.51%ID	E:5e-83	Thioredoxin-like 2, chloroplastic	PF00085.17^Thioredoxin^Thioredoxin^96-182^E:2.7e-18	0,321	0,424	0	1,385	0,875	0,057	1	-0,02	0,389	0,398	TRL2_ORYSJ	Thioredoxin-like 2, chloroplastic (Lilium-type thioredoxin 2) (OsTrx19)	Os05g0200100 LOC_Os05g11090 OSJNBa0017O06.11	Oryza sativa subsp. japonica (Rice)		Chloroplast; Complete proteome; Disulfide bond; Electron transport; Plastid; Redox-active center; Reference proteome; Transit peptide; Transport
SRR1271859_TR2392_c0_g1_i1_len=1113	TCTP_ELAGV	Q:846-343,H:1-168	87.5%ID	E:6e-83	Translationally-controlled tumor protein homolog	PF00838.14^TCTP^Translationally controlled tumour protein^1-164^E:9.4e-59	1	-0,005	0	2,838	0,09	1,03	0,152	0,877	1	-0,033	TCTP_ELAGV	Translationally-controlled tumor protein homolog (TCTP)	TCTP	Elaeis guineensis var. tenera (Oil palm)		Calcium; Cytoplasm
SRR1271859_TR3405_c0_g1_i11_len=2129	Y5707_ARATH	Q:557-1630,H:40-389	52.09%ID	E:3e-117	Probable receptor-like protein kinase At5g47070	PF00069.22^Pkinase^Protein kinase domain^86-364^E:1.9e-47`PF07714.14^Pkinase_Tyr^Protein tyrosine kinase^88-365^E:2.8e-49	0,819	0,153	0	1,482	0,037	1,197	0,01	1,429	0,904	-0,108	Y5707_ARATH	Probable receptor-like protein kinase At5g47070 (EC 2.7.11.1)	At5g47070 K14A3.2	Arabidopsis thaliana (Mouse-ear cress)		ATP-binding; Cell membrane; Complete proteome; Kinase; Membrane; Nucleotide-binding; Phosphoprotein; Reference proteome; Serine/threonine-protein kinase; Transferase
SRR1271859_TR3793_c0_g4_i1_len=870	NA	NA	NA	NA	NA	.	0,043	1,256	0	2,542	0,044	2,224	0,027	2,357	1	0,948	.	.	.	.	.	.
SRR1271859_TR4407_c1_g1_i2_len=2549	NA	NA	NA	NA	NA	PF07765.9^KIP1^KIP1-like protein^21-94^E:9.6e-37	0,267	0,4	0	1,356	0,004	0,936	0,004	0,928	0,381	0,824	.	.	.	.	.	.
SRR1271859_TR5307_c0_g1_i2_len=2094	GSHRP_SPIOL	Q:1824-385,H:10-489	86.67%ID	E:0	Glutathione reductase, chloroplastic	PF07992.11^Pyr_redox_2^Pyridine nucleotide-disulphide oxidoreductase^26-352^E:7.1e-67`PF13738.3^Pyr_redox_3^Pyridine nucleotide-disulphide oxidoreductase^157-336^E:4.6e-13`PF00070.24^Pyr_redox^Pyridine nucleotide-disulphide oxidoreductase^204-278^E:1.2e-17`PF02852.19^Pyr_redox_dim^Pyridine nucleotide-disulphide oxidoreductase, dimerisation domain^372-481^E:4.1e-30	0,606	-0,196	0	1,426	0,004	1,476	0,016	1,305	0,272	0,502	GSHRP_SPIOL	Glutathione reductase, chloroplastic (GR) (GRase) (EC 1.8.1.7) (Fragment)		Spinacia oleracea (Spinach)		Chloroplast; Disulfide bond; FAD; Flavoprotein; NADP; Oxidoreductase; Plastid; Redox-active center; Transit peptide
SRR1271859_TR6013_c0_g1_i1_len=2112	NA	NA	NA	NA	NA	PF00403.23^HMA^Heavy-metal-associated domain^13-70^E:1.1e-15	0,662	0,19	0	1,361	0,02	1,406	0,027	1,36	0,887	0,067	.	.	.	.	.	.
SRR1271859_TR6020_c0_g2_i3_len=2090	PTR21_ARATH	Q:1188-157,H:274-617	52.74%ID	E:8e-179	Protein NRT1/ PTR FAMILY 2.13	PF00854.18^PTR2^POT family^150-305^E:2.1e-29	1	1,144	0	4,079	0,003	1,921	0,021	1,543	1	0,558	PTR21_ARATH	Protein NRT1/ PTR FAMILY 2.13 (AtNPF2.13) (Nitrate transporter 1.7)	NPF2.13 NRT1.7 At1g69870 T17F3.10	Arabidopsis thaliana (Mouse-ear cress)		Cell membrane; Complete proteome; Membrane; Nitrate assimilation; Reference proteome; Symport; Transmembrane; Transmembrane helix; Transport
SRR1271859_TR7382_c0_g1_i2_len=1349	NA	NA	NA	NA	NA	PF15365.3^PNRC^Proline-rich nuclear receptor coactivator^175-221^E:3.4e-06	0,319	0,939	0	2,429	0,005	1,281	0,123	0,789	1	-0,06	.	.	.	.	.	.
SRR1271859_TR9359_c0_g1_i1_len=1931	SNX1_ARATH	Q:418-1554,H:24-401	79.95%ID	E:0	Sorting nexin 1	PF00787.21^PX^PX domain^24-137^E:2.7e-25`PF09325.7^Vps5^Vps5 C terminal like^169-394^E:1.5e-23	1	-0,031	0,001	3,851	0,239	1,325	0,158	1,44	1	0,011	SNX1_ARATH	Sorting nexin 1 (AtSNX1) (Vacuolar protein sorting-associated protein 5 homolog)	SNX1 VPS5 At5g06140 K16F4.11 MBL20	Arabidopsis thaliana (Mouse-ear cress)		Complete proteome; Cytoplasm; Endosome; Golgi apparatus; Lipid-binding; Membrane; Phosphoprotein; Protein transport; Reference proteome; Transport
SRR1271859_TR12007_c0_g1_i1_len=2551	CP74A_ARATH	Q:782-2203,H:43-517	70.32%ID	E:0	Allene oxide synthase, chloroplastic	PF00067.19^p450^Cytochrome P450^372-518^E:1.7e-15	0,04	1,315	0	4,296	0,003	1,864	0,001	1,937	0,402	0,837	CP74A_ARATH	Allene oxide synthase, chloroplastic (EC 4.2.1.92) (Cytochrome P450 74A) (Hydroperoxide dehydrase)	CYP74A AOS At5g42650 MJB21.2	Arabidopsis thaliana (Mouse-ear cress)	PATHWAY: Lipid metabolism; oxylipin biosynthesis. {ECO:0000305}.	3D-structure; Chloroplast; Complete proteome; Fatty acid biosynthesis; Fatty acid metabolism; Heme; Iron; Lipid biosynthesis; Lipid metabolism; Lyase; Metal-binding; Oxylipin biosynthesis; Plastid; Reference proteome; Transit peptide
SRR1271859_TR12590_c1_g2_i1_len=1418	NA	NA	NA	NA	NA	.	0,094	0,614	0	1,524	0,039	0,643	0,005	0,846	0,736	0,127	.	.	.	.	.	.
SRR1271859_TR12638_c2_g1_i2_len=1704	GLNA1_VITVI	Q:1538-666,H:16-306	92.1%ID	E:3e-180	Glutamine synthetase cytosolic isozyme 1	PF03951.16^Gln-synt_N^Glutamine synthetase, beta-Grasp domain^62-137^E:1.1e-12`PF00120.21^Gln-synt_C^Glutamine synthetase, catalytic domain^207-295^E:1.6e-11	0,188	0,507	0,001	1,887	0,009	0,857	0,175	0,472	0,165	0,77	GLNA1_VITVI	Glutamine synthetase cytosolic isozyme 1 (EC 6.3.1.2) (Glutamate--ammonia ligase)	GS1-1 GS1;1	Vitis vinifera (Grape)		ATP-binding; Cytoplasm; Ligase; Nucleotide-binding
SRR1271859_TR13493_c1_g2_i2_len=1116	NAC10_ORYSJ	Q:221-685,H:29-200	58.14%ID	E:9e-58	NAC transcription factor ONAC010	PF02365.12^NAM^No apical meristem (NAM) protein^16-139^E:1.3e-27	0,129	1,131	0	2,444	0,189	0,523	0,836	0,115	0,679	0,413	NAC10_ORYSJ	NAC transcription factor ONAC010	ONAC010 Os07g0566500 LOC_Os07g37920 OJ1092_A07.104 OJ1773_H01.126 OsJ_24777	Oryza sativa subsp. japonica (Rice)		Complete proteome; DNA-binding; Nucleus; Reference proteome; Transcription; Transcription regulation
SRR1271859_TR17337_c1_g4_i9_len=2350	MAOX_VITVI	Q:122-1246,H:1-375	87.2%ID	E:0	NADP-dependent malic enzyme	PF00390.16^malic^Malic enzyme, N-terminal domain^117-296^E:3.2e-79`PF03949.12^Malic_M^Malic enzyme, NAD binding domain^307-375^E:5.8e-18	0,404	-0,281	0,001	1,153	0,76	0,597	0,282	1,126	0,086	-0,529	MAOX_VITVI	NADP-dependent malic enzyme (NADP-ME) (EC 1.1.1.40)		Vitis vinifera (Grape)		Cytoplasm; Metal-binding; NADP; Oxidoreductase
SRR1271859_TR18232_c0_g2_i1_len=1530	NA	NA	NA	NA	NA	.	0,628	-0,138	0	1,305	0,08	0,541	0,001	0,96	0,947	-0,042	.	.	.	.	.	.
SRR1271859_TR18550_c0_g2_i3_len=2041	CISY_DAUCA	Q:240-1655,H:1-472	87.29%ID	E:0	Citrate synthase, mitochondrial	PF00285.18^Citrate_synt^Citrate synthase, C-terminal domain^79-457^E:7.3e-105	0,606	0,195	0,001	1,152	0,002	1,1	0,013	0,922	0,618	-0,226	CISY_DAUCA	Citrate synthase, mitochondrial (EC 2.3.3.16)	CS	Daucus carota (Wild carrot)	PATHWAY: Carbohydrate metabolism; tricarboxylic acid cycle; isocitrate from oxaloacetate: step 1/2.	Mitochondrion; Transferase; Transit peptide; Tricarboxylic acid cycle
SRR1271859_TR18858_c0_g1_i2_len=2342	NA	NA	NA	NA	NA	.	0,559	0,182	0,001	1,021	0,607	0,13	0,015	0,575	0,072	0,513	.	.	.	.	.	.
SRR1271859_TR18858_c0_g1_i7_len=2417	NA	NA	NA	NA	NA	PF01852.16^START^START domain^154-305^E:3.7e-05	0,806	0,097	0	1,654	1	-0,005	0,092	0,439	0,004	0,872	.	.	.	.	.	.
SRR1271859_TR19578_c0_g1_i1_len=2377	ERF53_ARATH	Q:1368-679,H:147-313	49.57%ID	E:6e-44	Ethylene-responsive transcription factor ERF053	PF00847.17^AP2^AP2 domain^219-268^E:2.7e-12	0,56	0,778	0	2,82	0,009	1,445	0,059	1,14	1	0,162	ERF53_ARATH	Ethylene-responsive transcription factor ERF053	ERF053 At2g20880 F5H14.15	Arabidopsis thaliana (Mouse-ear cress)		Activator; Complete proteome; DNA-binding; Ethylene signaling pathway; Nucleus; Reference proteome; Transcription; Transcription regulation
SRR1271859_TR19956_c0_g2_i2_len=1478	NA	NA	NA	NA	NA	PF13419.3^HAD_2^Haloacid dehalogenase-like hydrolase^256-327^E:2.1e-06`PF13242.3^Hydrolase_like^HAD-hyrolase-like^284-330^E:3.2e-05	0,76	-0,387	0	7,78	0,187	0,587	0,793	-0,225	1	0	.	.	.	.	.	.
SRR1271859_TR20045_c0_g3_i4_len=1369	NA	NA	NA	NA	NA	.	0,409	0,24	0	7,377	0,027	-0,619	0,018	-0,66	1	0	.	.	.	.	.	.
SRR1271859_TR20162_c0_g1_i1_len=1009	PPSP2_ARATH	Q:28-792,H:7-274	60.97%ID	E:1e-114	Inorganic pyrophosphatase 2	PF06888.9^Put_Phosphatase^Putative Phosphatase^10-234^E:2.3e-93`PF12710.4^HAD^haloacid dehalogenase-like hydrolase^12-182^E:2.9e-12	1	1,027	0	5,359	0,01	2,3	0,016	2,27	0,76	-0,551	PPSP2_ARATH	Inorganic pyrophosphatase 2 (AtPPsPase2) (PPi phosphatase 2) (Pyrophosphate-specific phosphatase 2) (EC 3.6.1.1)	At1g17710 F11A6.5	Arabidopsis thaliana (Mouse-ear cress)		Alternative splicing; Complete proteome; Hydrolase; Magnesium; Metal-binding; Reference proteome
SRR1271859_TR21345_c0_g1_i2_len=2705	C3H66_ARATH	Q:466-1800,H:4-445	54.58%ID	E:4e-144	Zinc finger CCCH domain-containing protein 66	PF12796.4^Ank_2^Ankyrin repeats (3 copies)^68-146^E:1.5e-08`PF13637.3^Ank_4^Ankyrin repeats (many copies)^79-125^E:7.2e-05`PF00023.27^Ank^Ankyrin repeat^113-146^E:7.2e-06`PF00642.21^zf-CCCH^Zinc finger C-x8-C-x5-C-x3-H type (and similar)^269-289^E:3.8e-05	0,034	0,771	0	1,81	0,651	0,159	0,471	0,239	0,165	0,442	C3H66_ARATH	Zinc finger CCCH domain-containing protein 66 (AtC3H66)	At5g58620 MZN1.16 MZN1.8	Arabidopsis thaliana (Mouse-ear cress)		ANK repeat; Complete proteome; DNA-binding; Metal-binding; Reference proteome; Repeat; Zinc; Zinc-finger
SRR1271859_TR23255_c0_g2_i10_len=1897	P2C60_ARATH	Q:1339-323,H:1-339	82.6%ID	E:0	Probable protein phosphatase 2C 60	PF00481.18^PP2C^Protein phosphatase 2C^23-103^E:8.2e-10`PF00481.18^PP2C^Protein phosphatase 2C^157-313^E:1.4e-59	1	0,192	0,001	6,578	0,004	2,339	0,01	2,196	1	0	P2C60_ARATH	Probable protein phosphatase 2C 60 (AtPP2C60) (EC 3.1.3.16)	At4g31860 F11C18.60	Arabidopsis thaliana (Mouse-ear cress)		Alternative splicing; Complete proteome; Hydrolase; Magnesium; Manganese; Metal-binding; Protein phosphatase; Reference proteome
SRR1271859_TR27119_c0_g1_i1_len=1987	STAD_OLEEU	Q:322-1503,H:1-390	85.03%ID	E:0	Stearoyl-[acyl-carrier-protein] 9-desaturase, chloroplastic	PF03405.11^FA_desaturase_2^Fatty acid desaturase^66-388^E:4.9e-159	1	-0,088	0	2,448	0,352	0,475	1	0	0,343	0,377	STAD_OLEEU	Stearoyl-[acyl-carrier-protein] 9-desaturase, chloroplastic (Stearoyl-ACP desaturase) (EC 1.14.19.2) (Acyl-[acyl-carrier-protein] desaturase)		Olea europaea (Common olive)	PATHWAY: Lipid metabolism; fatty acid metabolism.	Chloroplast; Fatty acid biosynthesis; Fatty acid metabolism; Iron; Lipid biosynthesis; Lipid metabolism; Metal-binding; Oxidoreductase; Plastid; Transit peptide
SRR1271859_TR27564_c0_g1_i1_len=1929	AVT3_SCHPO	Q:1482-385,H:253-622	30.13%ID	E:4e-33	Vacuolar amino acid transporter 3	PF01490.15^Aa_trans^Transmembrane amino acid transporter protein^33-415^E:2.2e-75	0,735	0,162	0	2,252	0,44	0,642	1	-0,094	1	-0,042	AVT3_SCHPO	Vacuolar amino acid transporter 3	avt3 SPAC3H1.09c	Schizosaccharomyces pombe (strain 972 / ATCC 24843) (Fission yeast)		Amino-acid transport; Complete proteome; Endoplasmic reticulum; Membrane; Phosphoprotein; Reference proteome; Sporulation; Transmembrane; Transmembrane helix; Transport; Vacuole
SRR1271859_TR28211_c0_g1_i1_len=1116	FER3_ARATH	Q:819-364,H:1-155	56.77%ID	E:3e-56	Ferredoxin-3, chloroplastic	PF00111.24^Fer2^2Fe-2S iron-sulfur cluster binding domain^65-138^E:2.6e-21	0,643	0,599	0	3,542	0,016	2,112	0,01	2,254	0,195	1,336	FER3_ARATH	Ferredoxin-3, chloroplastic (AtFd3)	FD3 At2g27510 F10A12.19	Arabidopsis thaliana (Mouse-ear cress)		2Fe-2S; Acetylation; Chloroplast; Complete proteome; Electron transport; Iron; Iron-sulfur; Metal-binding; Plastid; Reference proteome; Transit peptide; Transport
SRR1271859_TR28211_c0_g1_i2_len=2423	FER3_ARATH	Q:819-364,H:1-155	56.77%ID	E:1e-53	Ferredoxin-3, chloroplastic	PF00111.24^Fer2^2Fe-2S iron-sulfur cluster binding domain^65-138^E:2.6e-21	0,692	0,879	0,001	3,255	0,118	2,085	0,01	2,817	1	0,323	FER3_ARATH	Ferredoxin-3, chloroplastic (AtFd3)	FD3 At2g27510 F10A12.19	Arabidopsis thaliana (Mouse-ear cress)		2Fe-2S; Acetylation; Chloroplast; Complete proteome; Electron transport; Iron; Iron-sulfur; Metal-binding; Plastid; Reference proteome; Transit peptide; Transport
SRR1271859_TR28319_c1_g3_i4_len=1230	QORH_ARATH	Q:16-1002,H:1-329	72.34%ID	E:4e-167	Putative quinone-oxidoreductase homolog, chloroplastic	PF08240.9^ADH_N^Alcohol dehydrogenase GroES-like domain^34-95^E:3.5e-07`PF00107.23^ADH_zinc_N^Zinc-binding dehydrogenase^167-248^E:2e-13`PF13602.3^ADH_zinc_N_2^Zinc-binding dehydrogenase^198-327^E:6e-22	0,043	3,1	0	6,696	0,132	0,78	0,458	0,478	0,629	-0,988	QORH_ARATH	Putative quinone-oxidoreductase homolog, chloroplastic (EC 1.-.-.-)	At4g13010 F25G13_100	Arabidopsis thaliana (Mouse-ear cress)		Chloroplast; Complete proteome; Membrane; NAD; Oxidoreductase; Plastid; Plastid inner membrane; Reference proteome
SRR1271859_TR28488_c0_g1_i2_len=1662	LPA3_ARATH	Q:1058-270,H:96-358	80.61%ID	E:2e-155	Protein LOW PSII ACCUMULATION 3, chloroplastic	PF09353.7^DUF1995^Domain of unknown function (DUF1995)^5-198^E:1e-35	0,489	-0,238	0	7,274	1	-1,175	1	-2,647	0,806	-0,098	LPA3_ARATH	Protein LOW PSII ACCUMULATION 3, chloroplastic	LPA3 At1g73060 F3N23.26	Arabidopsis thaliana (Mouse-ear cress)		Acetylation; Chloroplast; Complete proteome; Membrane; Plastid; Reference proteome; Thylakoid; Transit peptide
SRR1271859_TR28957_c0_g2_i1_len=836	NA	NA	NA	NA	NA	.	0,001	1,906	0	1,999	0,002	1,132	0,12	0,633	0,312	0,833	.	.	.	.	.	.
SRR1271859_TR30385_c0_g4_i11_len=4804	PDR2_NICPL	Q:225-3146,H:4-985	72.53%ID	E:0	Pleiotropic drug resistance protein 2	PF14510.3^ABC_trans_N^ABC-transporter extracellular N-terminal^91-150^E:4.3e-13`PF00005.24^ABC_tran^ABC transporter^176-358^E:4.2e-16`PF01061.21^ABC2_membrane^ABC-2 type transporter^512-724^E:1.6e-37`PF08370.8^PDR_assoc^Plant PDR ABC transporter associated^729-791^E:3.4e-25`PF00005.24^ABC_tran^ABC transporter^874-957^E:1.7e-09	0,241	0,65	0	2,07	0,076	1,46	0,015	1,769	0,611	-0,362	PDR2_NICPL	Pleiotropic drug resistance protein 2 (NpPDR2)	PDR2	Nicotiana plumbaginifolia (Leadwort-leaved tobacco) (Tex-Mex tobacco)		ATP-binding; Membrane; Nucleotide-binding; Repeat; Transmembrane; Transmembrane helix; Transport
SRR1271859_TR30654_c1_g1_i17_len=2188	NA	NA	NA	NA	NA	PF03097.15^BRO1^BRO1-like domain^36-334^E:1.2e-11	0,524	0,6	0	3,348	0,258	0,856	0,62	0,451	1	0,922	.	.	.	.	.	.
SRR1271859_TR31831_c0_g2_i6_len=822	YKT61_ARATH	Q:600-46,H:1-184	88.65%ID	E:6e-118	VAMP-like protein YKT61	PF13774.3^Longin^Regulated-SNARE-like domain^49-113^E:7.7e-11`PF00957.18^Synaptobrevin^Synaptobrevin^138-184^E:6e-11	0,807	-0,141	0	7,489	0,18	0,745	0,009	1,299	0,254	4,394	YKT61_ARATH	VAMP-like protein YKT61 (AtYKT61) (Geranylgeranylated protein 1) (AtGP1)	YKT61 At5g58060 K21L19.5 K21L19_40	Arabidopsis thaliana (Mouse-ear cress)		Alternative splicing; Cell membrane; Coiled coil; Complete proteome; Lipoprotein; Membrane; Methylation; Palmitate; Prenylation; Protein transport; Reference proteome; Transport
SRR1271859_TR33275_c0_g1_i1_len=771	NA	NA	NA	NA	NA	PF12023.5^DUF3511^Domain of unknown function (DUF3511)^67-111^E:3.5e-27	0,212	0,564	0	1,909	0,038	0,937	0,004	1,266	0,748	0,219	.	.	.	.	.	.
SRR1271859_TR33775_c0_g1_i1_len=1024	CHI6_ORYSJ	Q:189-926,H:26-271	66.26%ID	E:1e-103	Chitinase 6	PF00187.16^Chitin_bind_1^Chitin recognition protein^26-57^E:3.3e-07`PF00182.16^Glyco_hydro_19^Chitinase class I^70-270^E:4.7e-50	0,158	1,708	0,001	2,431	0,007	2,14	0,002	2,34	0,377	0,644	CHI6_ORYSJ	Chitinase 6 (EC 3.2.1.14) (Pathogenesis related (PR)-3 chitinase 6)	Cht6 Os02g0605900 LOC_Os02g39330 OJ1058_F07.19 OsJ_07447	Oryza sativa subsp. japonica (Rice)		Carbohydrate metabolism; Chitin degradation; Chitin-binding; Complete proteome; Disulfide bond; Glycoprotein; Glycosidase; Hydrolase; Plant defense; Polysaccharide degradation; Reference proteome; Signal
SRR1271859_TR33807_c0_g1_i3_len=3203	INVC_ARATH	Q:419-2416,H:11-664	66.42%ID	E:0	Alkaline/neutral invertase C, mitochondrial {ECO:0000305}	PF12899.4^Glyco_hydro_100^Alkaline and neutral invertase^199-639^E:2.4e-214	0,312	0,709	0,001	2,046	0,044	2,068	0,016	2,34	1	-0,167	INVC_ARATH	Alkaline/neutral invertase C, mitochondrial (A/N-INVC) (EC 3.2.1.26)	INVC At3g06500 F5E6.17	Arabidopsis thaliana (Mouse-ear cress)		Carbohydrate metabolism; Complete proteome; Glycosidase; Hydrolase; Mitochondrion; Phosphoprotein; Reference proteome; Transit peptide
SRR1271859_TR34060_c0_g2_i1_len=5990	CLAH1_ARATH	Q:278-5248,H:1-1657	93.06%ID	E:0	Clathrin heavy chain 1	PF01394.17^Clathrin_propel^Clathrin propeller repeat^20-56^E:1.6e-07`PF01394.17^Clathrin_propel^Clathrin propeller repeat^154-197^E:2.9e-10`PF09268.7^Clathrin-link^Clathrin, heavy-chain linker^344-367^E:2.5e-09`PF13838.3^Clathrin_H_link^Clathrin-H-link^369-434^E:2e-30`PF00637.17^Clathrin^Region in Clathrin and VPS^557-690^E:1.8e-21`PF00637.17^Clathrin^Region in Clathrin and VPS^701-840^E:2.1e-21`PF00637.17^Clathrin^Region in Clathrin and VPS^850-978^E:1.2e-28`PF00637.17^Clathrin^Region in Clathrin and VPS^993-1131^E:4.5e-31`PF00637.17^Clathrin^Region in Clathrin and VPS^1145-1281^E:2.6e-26`PF00637.17^Clathrin^Region in Clathrin and VPS^1289-1431^E:1.3e-28`PF00637.17^Clathrin^Region in Clathrin and VPS^1439-1579^E:1.2e-30	0,971	-0,019	0	1,482	0,448	-0,247	0,867	-0,065	0,101	-0,528	CLAH1_ARATH	Clathrin heavy chain 1	CHC1 At3g11130 F11B9.30 F9F8.6	Arabidopsis thaliana (Mouse-ear cress)		Acetylation; Coated pit; Complete proteome; Cytoplasmic vesicle; Endocytosis; Membrane; Reference proteome; Repeat
SRR1271859_TR38022_c0_g1_i9_len=1603	NA	NA	NA	NA	NA	.	0,142	0,511	0	4,263	1	0,237	1	-1,003	0,03	0,964	.	.	.	.	.	.
SRR1271859_TR38580_c1_g1_i1_len=2681	BXL1_ARATH	Q:2527-314,H:32-771	74.87%ID	E:0	Beta-D-xylosidase 1	PF00933.18^Glyco_hydro_3^Glycosyl hydrolase family 3 N terminal domain^135-379^E:1e-37`PF01915.19^Glyco_hydro_3_C^Glycosyl hydrolase family 3 C-terminal domain^419-649^E:2e-52`PF14310.3^Fn3-like^Fibronectin type III-like domain^719-781^E:1.9e-09	1	0	0	6,801	1	0	1	3,487	1	0	BXL1_ARATH	Beta-D-xylosidase 1 (AtBXL1) (EC 3.2.1.-) (Alpha-L-arabinofuranosidase) (EC 3.2.1.55)	BXL1 XYL1 At5g49360 K7J8.3	Arabidopsis thaliana (Mouse-ear cress)		Complete proteome; Extracellular matrix; Glycoprotein; Glycosidase; Hydrolase; Reference proteome; Secreted; Signal
SRR1271859_TR38879_c0_g2_i1_len=609	NA	NA	NA	NA	NA	.	0,254	2,823	0,001	6,593	0,002	4,276	0,502	2,334	1	-0,259	.	.	.	.	.	.
SRR1271859_TR39225_c0_g1_i1_len=554	IF1A_ONOVI	Q:219-554,H:1-112	97.32%ID	E:8e-60	Eukaryotic translation initiation factor 1A	.	0,343	-0,236	0	1,204	0,143	0,346	0,162	0,329	0,121	0,369	IF1A_ONOVI	Eukaryotic translation initiation factor 1A (eIF-1A) (Eukaryotic translation initiation factor 4C) (eIF-4C)		Onobrychis viciifolia (Common sainfoin)		Initiation factor; Protein biosynthesis
SRR1271859_TR39611_c0_g1_i2_len=818	DNJ20_ARATH	Q:375-743,H:66-189	59.68%ID	E:2e-42	Chaperone protein dnaJ 20, chloroplastic	PF00226.28^DnaJ^DnaJ domain^80-144^E:3.3e-21	0,001	1,538	0	8,932	1	-0,376	0,118	1,881	0,129	-5,132	DNJ20_ARATH	Chaperone protein dnaJ 20, chloroplastic (AtDjC20) (AtJ20)	ATJ20 C20 J20 At4g13830 F18A5.220	Arabidopsis thaliana (Mouse-ear cress)		Alternative splicing; Chaperone; Chloroplast; Complete proteome; Plastid; Reference proteome; Transit peptide
SRR1271859_TR41028_c0_g1_i1_len=1515	PR1B4_ARATH	Q:1232-636,H:8-206	68.5%ID	E:3e-77	PRA1 family protein B4	PF03208.16^PRA1^PRA1 family protein^50-193^E:7.4e-50	0,316	0,641	0	1,657	0,912	0,092	0,908	-0,108	0,854	-0,155	PR1B4_ARATH	PRA1 family protein B4 (AtPRA1.B4)	PRA1B4 At2g38360 T19C21.15	Arabidopsis thaliana (Mouse-ear cress)		Complete proteome; Endosome; Membrane; Reference proteome; Transmembrane; Transmembrane helix; Transport
SRR1271859_TR41867_c0_g1_i1_len=1200	NA	NA	NA	NA	NA	PF05514.8^HR_lesion^HR-like lesion-inducing^1-138^E:1.1e-50	1	-0,012	0	1,468	0,398	0,366	0,135	0,617	0,493	-0,237	.	.	.	.	.	.
SRR1271859_TR44244_c0_g2_i1_len=1353	VA722_ARATH	Q:352-1008,H:5-221	83.11%ID	E:2e-104	Vesicle-associated membrane protein 722 {ECO:0000303|PubMed:11115874}	PF13774.3^Longin^Regulated-SNARE-like domain^39-118^E:4.4e-23`PF00957.18^Synaptobrevin^Synaptobrevin^136-222^E:7.7e-34	0,868	0,08	0,001	1,248	0,007	1,128	0,115	0,73	0,941	0,051	VA722_ARATH	Vesicle-associated membrane protein 722 (AtVAMP722) (Synaptobrevin-related protein 1)	VAMP722 HAT24 SAR1 At2g33120 F25I18.14	Arabidopsis thaliana (Mouse-ear cress)		Alternative splicing; Cell membrane; Coiled coil; Complete proteome; Endosome; Membrane; Protein transport; Reference proteome; Transmembrane; Transmembrane helix; Transport
SRR1271859_TR44824_c0_g1_i11_len=1234	CHE1_CHEAL	Q:292-681,H:28-159	42.86%ID	E:4e-28	Pollen allergen Che a 1	PF01190.14^Pollen_Ole_e_I^Pollen proteins Ole e I like^29-110^E:3e-15	0,164	-0,48	0	1,208	0,004	0,822	0,126	0,465	1	0	CHE1_CHEAL	Pollen allergen Che a 1 (allergen Che a 1)		Chenopodium album (Fat-hen)		Allergen; Direct protein sequencing; Disulfide bond; Glycoprotein; Secreted; Signal
SRR1271859_TR44906_c0_g4_i19_len=3804	ZADH2_MOUSE	Q:1433-390,H:29-377	45.79%ID	E:5e-79	Zinc-binding alcohol dehydrogenase domain-containing protein 2	PF00106.22^adh_short^short chain dehydrogenase^4-69^E:9.4e-14`PF13561.3^adh_short_C2^Enoyl-(Acyl carrier protein) reductase^4-81^E:1.1e-09`PF08240.9^ADH_N^Alcohol dehydrogenase GroES-like domain^180-244^E:4.6e-08`PF00107.23^ADH_zinc_N^Zinc-binding dehydrogenase^304-433^E:3.6e-29`PF13602.3^ADH_zinc_N_2^Zinc-binding dehydrogenase^336-481^E:2.7e-21	0,781	0,483	0	2,735	0,461	1,439	1	0,665	1	-1,609	ZADH2_MOUSE	Zinc-binding alcohol dehydrogenase domain-containing protein 2 (EC 1.-.-.-)	Zadh2	Mus musculus (Mouse)		Acetylation; Complete proteome; NADP; Oxidoreductase; Peroxisome; Reference proteome
SRR1271859_TR45041_c0_g1_i7_len=779	KAD1_ARATH	Q:362-66,H:48-146	87.88%ID	E:2e-54	Adenylate kinase 1, chloroplastic	PF00406.19^ADK^Adenylate kinase^72-162^E:1.4e-25`PF13207.3^AAA_17^AAA domain^72-160^E:6.7e-17	0,419	0,433	0	2,144	0,164	0,91	0,214	0,837	0,524	0,616	KAD1_ARATH	Adenylate kinase 1, chloroplastic (AK 1) (AtPADK1) (EC 2.7.4.3) (ATP-AMP transphosphorylase 1) (ATP:AMP phosphotransferase) (Adenylate monophosphate kinase 1) (AMK1)	ADK At2g37250 F3G5.4	Arabidopsis thaliana (Mouse-ear cress)		ATP-binding; Chloroplast; Complete proteome; Kinase; Nucleotide-binding; Plastid; Reference proteome; Transferase; Transit peptide
SRR1271859_TR45218_c0_g2_i2_len=1264	NA	NA	NA	NA	NA	PF06749.9^DUF1218^Protein of unknown function (DUF1218)^58-153^E:2.6e-28	0,352	0,705	0	3,417	0,812	0,131	0,798	-0,177	0,412	0,519	.	.	.	.	.	.
SRR1271859_TR46477_c0_g2_i3_len=912	DNJ19_ARATH	Q:912-346,H:158-346	80.95%ID	E:4e-114	DnaJ protein ERDJ3B	PF01556.15^DnaJ_C^DnaJ C terminal domain^48-173^E:1.6e-31	0,944	-0,044	0	1,617	0,015	1,004	0,003	1,189	0,868	-0,074	DNJ19_ARATH	DnaJ protein ERDJ3B (Chaperone protein dnaJ 19) (AtDjB19) (AtJ19) (Endoplasmic reticulum dnaJ domain-containing protein 3B) (AtERdj3B) (Protein SCJ1 homolog ERDJ3B)	ERDJ3B B19 At3g62600 F26K9.30	Arabidopsis thaliana (Mouse-ear cress)		Chaperone; Complete proteome; Endoplasmic reticulum; Glycoprotein; Reference proteome; Signal; Stress response
SRR1271859_TR47126_c1_g2_i4_len=952	RAD23_ORYSJ	Q:210-809,H:1-198	52.5%ID	E:2e-51	Probable ubiquitin receptor RAD23	PF11976.5^Rad60-SLD^Ubiquitin-2 like Rad60 SUMO-like^1-73^E:3.1e-07`PF00240.20^ubiquitin^Ubiquitin family^3-76^E:2e-19`PF13881.3^Rad60-SLD_2^Ubiquitin-2 like Rad60 SUMO-like^9-69^E:0.00053`PF00627.28^UBA^UBA/TS-N domain^161-198^E:7.9e-15	0,678	-0,153	0,001	1,386	0,699	0,187	0,921	0,058	0,008	1,228	RAD23_ORYSJ	Probable ubiquitin receptor RAD23 (OsRAD23) (Probable DNA repair protein RAD23)	RAD23 Os09g0407200 LOC_Os09g24200 OJ1261_A08.49 P0465E03.1	Oryza sativa subsp. japonica (Rice)		Complete proteome; DNA damage; DNA repair; Nucleus; Reference proteome; Repeat
SRR342017_TR1625_c0_g1_i2_len=3321	TRPX_ARATH	Q:336-2057,H:26-607	74.4%ID	E:0	Anthranilate synthase alpha subunit 2, chloroplastic	PF04715.10^Anth_synt_I_N^Anthranilate synthase component I, N terminal region^104-258^E:1.9e-30`PF00425.15^Chorismate_bind^chorismate binding enzyme^319-590^E:3.9e-85	0,004	1,424	0	4,529	0,045	1,62	0,187	1,229	0,377	0,618	TRPX_ARATH	Anthranilate synthase alpha subunit 2, chloroplastic (EC 4.1.3.27) (Anthranilate synthase component 1-2) (Anthranilate synthase component I-2)	ASA2 At2g29690 T27A16.21	Arabidopsis thaliana (Mouse-ear cress)	PATHWAY: Amino-acid biosynthesis; L-tryptophan biosynthesis; L-tryptophan from chorismate: step 1/5.	Amino-acid biosynthesis; Aromatic amino acid biosynthesis; Chloroplast; Complete proteome; Lyase; Plastid; Reference proteome; Transit peptide; Tryptophan biosynthesis
SRR342017_TR1796_c3_g11_i1_len=3843	CYF_COFAR	Q:2947-3843,H:1-298	95.65%ID	E:0	Cytochrome f {ECO:0000255|HAMAP-Rule:MF_00610}	PF16639.2^Apocytochr_F_N^Apocytochrome F, N-terminal^36-190^E:3.6e-78`PF01333.16^Apocytochr_F_C^Apocytochrome F, C-terminal^202-299^E:2.6e-36	0,066	-4,133	0	6,817	0,066	-0,972	0,618	-0,244	0,502	3,416	CYF_COFAR	Cytochrome f	petA	Coffea arabica (Arabian coffee)		Chloroplast; Electron transport; Heme; Iron; Membrane; Metal-binding; Photosynthesis; Plastid; Signal; Thylakoid; Transmembrane; Transmembrane helix; Transport
SRR342017_TR1807_c0_g1_i2_len=1200	NA	NA	NA	NA	NA	PF13259.3^DUF4050^Protein of unknown function (DUF4050)^73-113^E:3.2e-11	0,729	0,269	0	3,156	0,155	1,055	0,057	1,25	0,692	0,955	.	.	.	.	.	.
SRR342017_TR1966_c0_g5_i1_len=618	RANA1_TOBAC	Q:67-618,H:8-191	99.46%ID	E:4e-136	GTP-binding nuclear protein Ran-A1	PF00071.19^Ras^Ras family^1-80^E:1.4e-20	0,603	-0,174	0	2,038	0,038	0,689	0,05	0,648	0,552	0,143	RANA1_TOBAC	GTP-binding nuclear protein Ran-A1	RAN-A1	Nicotiana tabacum (Common tobacco)		GTP-binding; Nucleotide-binding; Nucleus; Protein transport; Transport
SRR342017_TR2922_c1_g5_i1_len=796	PABP2_ARATH	Q:296-3,H:24-123	72%ID	E:3e-41	Polyadenylate-binding protein 2	PF00076.19^RRM_1^RNA recognition motif. (a.k.a. RRM, RBD, or RNP domain)^28-98^E:7.7e-19`PF13893.3^RRM_5^RNA recognition motif. (a.k.a. RRM, RBD, or RNP domain)^36-104^E:0.0048	0,78	-0,075	0	1,224	0,727	0,094	0,112	0,411	0,008	-0,595	PABP2_ARATH	Polyadenylate-binding protein 2 (PABP-2) (Poly(A)-binding protein 2)	PAB2 At4g34110 F28A23.130	Arabidopsis thaliana (Mouse-ear cress)		Complete proteome; Cytoplasm; Host-virus interaction; Nonsense-mediated mRNA decay; Nucleus; RNA-binding; Reference proteome; Repeat; Translation regulation
SRR342017_TR3458_c0_g1_i1_len=1449	SRP_VITRI	Q:405-1046,H:9-241	58.55%ID	E:2e-81	Stress-related protein	PF05755.9^REF^Rubber elongation factor protein (REF)^18-212^E:1.4e-78	0,117	0,611	0	1,497	0,149	0,533	0,149	0,54	0,181	0,472	SRP_VITRI	Stress-related protein	SRP	Vitis riparia (Frost grape) (Vitis vulpina)		Stress response
SRR342017_TR5448_c0_g4_i4_len=725	MTDH_PETCR	Q:699-214,H:174-335	68.52%ID	E:2e-72	Mannitol dehydrogenase	PF00107.23^ADH_zinc_N^Zinc-binding dehydrogenase^6-128^E:3.1e-19	0,197	0,326	0	1,991	0,019	0,767	0,522	-0,251	0,663	0,434	MTDH_PETCR	Mannitol dehydrogenase (EC 1.1.1.255) (NAD-dependent mannitol dehydrogenase) (Fragment)	ELI3	Petroselinum crispum (Parsley) (Petroselinum hortense)		Metal-binding; NAD; Oxidoreductase; Zinc
SRR342017_TR7835_c0_g1_i6_len=2826	NA	NA	NA	NA	NA	.	0,526	0,317	0	1,609	0,856	0,115	0,721	0,169	0,083	-0,59	.	.	.	.	.	.
SRR342017_TR10013_c0_g1_i1_len=2070	U92A1_ARATH	Q:368-1861,H:8-485	40.83%ID	E:6e-120	UDP-glycosyltransferase 92A1	PF00201.15^UDPGT^UDP-glucoronosyl and UDP-glucosyl transferase^287-453^E:2e-22	0,454	0,392	0	2,151	1	0,18	1	0,345	0,18	0,472	U92A1_ARATH	UDP-glycosyltransferase 92A1 (EC 2.4.1.-)	UGT92A1 At5g12890 T24H18.60	Arabidopsis thaliana (Mouse-ear cress)		Complete proteome; Glycosyltransferase; Reference proteome; Transferase
SRR342017_TR13432_c0_g1_i1_len=716	IAA17_ARATH	Q:604-29,H:3-218	50.69%ID	E:2e-60	Auxin-responsive protein IAA17	PF02309.13^AUX_IAA^AUX/IAA family^31-201^E:3.3e-58	1	-0,038	0	3,815	0	-2,94	0	-2,596	1	-0,12	IAA17_ARATH	Auxin-responsive protein IAA17 (Auxin response 3) (Indoleacetic acid-induced protein 17)	IAA17 AXR3 At1g04250 F19P19.31	Arabidopsis thaliana (Mouse-ear cress)		3D-structure; Auxin signaling pathway; Complete proteome; Nucleus; Phosphoprotein; Reference proteome; Repressor; Transcription; Transcription regulation
SRR342017_TR15152_c0_g5_i3_len=976	HT1_ARATH	Q:304-975,H:110-327	38.26%ID	E:4e-40	Serine/threonine-protein kinase HT1	PF07714.14^Pkinase_Tyr^Protein tyrosine kinase^95-325^E:4e-51`PF00069.22^Pkinase^Protein kinase domain^97-322^E:1.5e-47	0,357	0,663	0	3,184	0,065	1,388	0,001	2,021	0,56	-0,943	HT1_ARATH	Serine/threonine-protein kinase HT1 (EC 2.7.11.1) (High leaf temperature protein 1)	HT1 At1g62400 F24O1.13	Arabidopsis thaliana (Mouse-ear cress)		ATP-binding; Complete proteome; Kinase; Nucleotide-binding; Reference proteome; Serine/threonine-protein kinase; Transferase
SRR342017_TR15231_c3_g12_i1_len=684	NA	NA	NA	NA	NA	.	0,758	0,106	0	5,72	0,019	0,892	0,003	1,075	1	0,236	.	.	.	.	.	.
SRR342017_TR17457_c0_g2_i1_len=811	NA	NA	NA	NA	NA	.	0,646	0,308	0	2,188	0,086	0,913	0,283	0,661	0,545	0,623	.	.	.	.	.	.

SRR342017_TR18067_c0_g1_i3_len=1739	DNJH2_ALLPO	Q:1567-236,H:1-418	79.1%ID	E:0	DnaJ protein homolog 2	PF00226.28^DnaJ^DnaJ domain^13-71^E:3.9e-22`PF01556.15^DnaJ_C^DnaJ C terminal domain^121-369^E:1.3e-35`PF00684.16^DnaJ_CXXCXGXG^DnaJ central domain^147-213^E:5e-15	0,606	0,177	0	2,102	0,549	0,174	0,635	0,137	0,019	0,931	DNJH2_ALLPO	DnaJ protein homolog 2	LDJ2	Allium porrum (Leek) (Allium ampeloprasum var. porrum)		Chaperone; Lipoprotein; Membrane; Metal-binding; Methylation; Prenylation; Repeat; Zinc; Zinc-finger
SRR342017_TR19861_c0_g1_i1_len=1064	KCY4_ORYSJ	Q:232-837,H:1-208	78.85%ID	E:3e-112	UMP-CMP kinase 4 {ECO:0000255|HAMAP-Rule:MF_03172}	PF13671.3^AAA_33^AAA domain^23-156^E:3.4e-08`PF13238.3^AAA_18^AAA domain^24-150^E:7e-09`PF00406.19^ADK^Adenylate kinase^26-178^E:2e-48`PF13207.3^AAA_17^AAA domain^27-157^E:3.2e-37`PF13426.4^PAS_9^PAS domain^33-91^E:0.3	0,591	0,207	0	1,489	0,528	-0,272	0,528	-0,285	0,578	0,238	KCY4_ORYSJ	Probable UMP/CMP kinase 4 (EC 2.7.4.14)	Os02g0778400 LOC_Os02g53790 OJ1293_A01.3	Oryza sativa subsp. japonica (Rice)		ATP-binding; Complete proteome; Kinase; Nucleotide-binding; Pyrimidine biosynthesis; Reference proteome; Transferase
SRR342017_TR20576_c1_g5_i1_len=2757	THIK2_ARATH	Q:364-1701,H:1-449	78.32%ID	E:0	3-ketoacyl-CoA thiolase 2, peroxisomal	PF00108.20^Thiolase_N^Thiolase, N-terminal domain^49-304^E:3.3e-78`PF02803.15^Thiolase_C^Thiolase, C-terminal domain^313-435^E:4.5e-48	0,463	0,288	0	2,282	0,007	1,514	0,001	1,694	0,729	0,142	THIK2_ARATH	3-ketoacyl-CoA thiolase 2, peroxisomal (EC 2.3.1.16) (Acetyl-CoA acyltransferase 2) (Beta-ketothiolase 2) (Peroxisomal 3-oxoacyl-CoA thiolase 2) (Peroxisome defective protein 1)	PED1 KAT2 At2g33150 F25I18.11	Arabidopsis thaliana (Mouse-ear cress)	PATHWAY: Lipid metabolism; fatty acid metabolism.	3D-structure; Acyltransferase; Complete proteome; Fatty acid biosynthesis; Fatty acid metabolism; Glyoxysome; Lipid biosynthesis; Lipid metabolism; Oxylipin biosynthesis; Peroxisome; Reference proteome; Transferase; Transit peptide
SRR342017_TR20662_c1_g1_i2_len=3177	NDHK_EUCGG	Q:1710-1033,H:1-224	95.58%ID	E:7e-139	NAD(P)H-quinone oxidoreductase subunit K, chloroplastic {ECO:0000255|HAMAP-Rule:MF_01356}	PF01058.19^Oxidored_q6^NADH ubiquinone oxidoreductase, 20 Kd subunit^44-153^E:1.6e-23	1	0,037	0,001	2,023	0,917	0,088	0,189	0,544	1	-0,125	NDHK_EUCGG	NAD(P)H-quinone oxidoreductase subunit K, chloroplastic (EC 1.6.5.-) (NAD(P)H dehydrogenase subunit K) (NADH-plastoquinone oxidoreductase subunit K)	ndhK	Eucalyptus globulus subsp. globulus (Tasmanian blue gum)		4Fe-4S; Chloroplast; Iron; Iron-sulfur; Membrane; Metal-binding; NAD; NADP; Oxidoreductase; Plastid; Plastoquinone; Quinone; Thylakoid; Transport
SRR342017_TR20766_c0_g1_i2_len=788	NIFU1_ARATH	Q:509-18,H:66-230	77.58%ID	E:2e-64	NifU-like protein 1, chloroplastic	PF01106.14^NifU^NifU-like domain^71-131^E:6.3e-23`PF01106.14^NifU^NifU-like domain^154-209^E:7.3e-05	0,058	0,653	0	1,397	0,003	0,913	0,01	0,812	0,381	2,348	NIFU1_ARATH	NifU-like protein 1, chloroplastic (AtCNfu1) (AtCnfU-IVb)	NIFU1 CNFU1 NFU1 At4g01940 T7B11.20	Arabidopsis thaliana (Mouse-ear cress)		Chloroplast; Complete proteome; Disulfide bond; Plastid; Reference proteome; Transit peptide
SRR342017_TR20766_c0_g9_i1_len=1839	DREB3_ARATH	Q:1250-957,H:38-135	80.61%ID	E:6e-48	Dehydration-responsive element-binding protein 3	PF00847.17^AP2^AP2 domain^74-124^E:6.6e-12	0,129	1,133	0	3,283	0,117	0,606	0,022	0,854	0,381	-0,779	DREB3_ARATH	Dehydration-responsive element-binding protein 3 (Protein TINY 2)	DREB3 ERF041 TINY2 At5g11590 F15N18.180	Arabidopsis thaliana (Mouse-ear cress)		Activator; Complete proteome; DNA-binding; Ethylene signaling pathway; Nucleus; Reference proteome; Transcription; Transcription regulation
SRR342017_TR20986_c1_g1_i2_len=1210	GEDH1_OCIBA	Q:1136-441,H:11-242	65.95%ID	E:1e-71	Geraniol dehydrogenase 1	PF08240.9^ADH_N^Alcohol dehydrogenase GroES-like domain^32-144^E:2.4e-27`PF02826.16^2-Hacid_dh_C^D-isomer specific 2-hydroxyacid dehydrogenase, NAD binding domain^167-217^E:6.4e-07	0,317	-0,509	0	2,591	0,798	-0,23	0,663	0,488	0,04	0,514	GEDH1_OCIBA	Geraniol dehydrogenase 1 (ObaGEDH1) (EC 1.1.1.183)	GEDH1	Ocimum basilicum (Sweet basil)		Metal-binding; NADP; Oxidoreductase; Zinc
SRR342017_TR21527_c1_g1_i6_len=1845	NA	NA	NA	NA	NA	PF00790.16^VHS^VHS domain^138-269^E:1.4e-22`PF03127.11^GAT^GAT domain^325-399^E:3.2e-16	0,925	0,064	0	1,625	0,051	1,028	0,051	1,063	1	1,28	.	.	.	.	.	.
SRR342017_TR22495_c0_g2_i1_len=2497	KPYC_SOYBN	Q:1869-391,H:23-507	44.15%ID	E:2e-122	Pyruvate kinase, cytosolic isozyme	PF00224.18^PK^Pyruvate kinase, barrel domain^30-375^E:4.5e-93`PF02887.13^PK_C^Pyruvate kinase, alpha/beta domain^395-522^E:7.8e-25	0,713	0,141	0	1,719	0,877	0,06	0,356	0,285	0,377	0,573	KPYC_SOYBN	Pyruvate kinase, cytosolic isozyme (PK) (EC 2.7.1.40)		Glycine max (Soybean) (Glycine hispida)	PATHWAY: Carbohydrate degradation; glycolysis; pyruvate from D-glyceraldehyde 3-phosphate: step 5/5.	ATP-binding; Complete proteome; Cytoplasm; Glycolysis; Kinase; Magnesium; Metal-binding; Nucleotide-binding; Potassium; Pyruvate; Reference proteome; Transferase
SRR342017_TR23313_c2_g1_i3_len=2140	GLYK_ARATH	Q:443-1549,H:87-455	73.24%ID	E:0	D-glycerate 3-kinase, chloroplastic	.	0,451	0,328	0	4,222	0	-6,62	0	-6,674	0,099	-0,831	GLYK_ARATH	D-glycerate 3-kinase, chloroplastic (AtGLYK) (EC 2.7.1.31)	GLYK At1g80380 F5I6.13	Arabidopsis thaliana (Mouse-ear cress)	PATHWAY: Photosynthesis; photorespiration; 3-phospho-D-glycerate from glycine: step 4/4.	ATP-binding; Alternative splicing; Chloroplast; Complete proteome; Glycolate pathway; Kinase; Nucleotide-binding; Photorespiration; Plastid; Reference proteome; Transferase; Transit peptide
SRR342017_TR23421_c1_g2_i7_len=1492	RSSA_DAUCA	Q:166-981,H:1-269	78.02%ID	E:1e-139	40S ribosomal protein SA {ECO:0000255|HAMAP-Rule:MF_03015}	PF00318.17^Ribosomal_S2^Ribosomal protein S2^18-112^E:1.3e-12`PF00318.17^Ribosomal_S2^Ribosomal protein S2^117-182^E:1.8e-12	0,09	-0,734	0	8,258	1	0	1	0	0,009	-5,941	RSSA_DAUCA	40S ribosomal protein SA (p40)	179B	Daucus carota (Wild carrot)		Cytoplasm; Ribonucleoprotein; Ribosomal protein
SRR342017_TR24328_c0_g3_i3_len=574	NU50B_ARATH	Q:396-572,H:320-378	72.88%ID	E:5e-22	Nuclear pore complex protein NUP50B {ECO:0000303|PubMed:21189294}	PF00638.15^Ran_BP1^RanBP1 domain^121-173^E:1e-07	0,163	-0,689	0,001	6,638	0	-6,728	0	-6,728	0,196	0,517	NU50B_ARATH	Nuclear pore complex protein NUP50B (Nucleoporin 50B)	NUP50B At3g15970 MSL1.1	Arabidopsis thaliana (Mouse-ear cress)		Acetylation; Complete proteome; Nuclear pore complex; Nucleus; Phosphoprotein; Protein transport; Reference proteome; Repeat; Translocation; Transport; mRNA transport
SRR342017_TR24386_c3_g1_i4_len=1596	LEUNG_ARATH	Q:1276-1539,H:1-88	98.86%ID	E:6e-50	Transcriptional corepressor LEUNIG {ECO:0000303|PubMed:11058164}	PF06232.8^ATS3^Embryo-specific protein 3, (ATS3)^30-132^E:4.1e-07`PF01477.20^PLAT^PLAT/LH2 domain^36-153^E:2.2e-14	0,187	1,035	0	4,17	0,001	3,204	0,003	3,059	0,111	-0,915	LEUNG_ARATH	Transcriptional corepressor LEUNIG (Protein ROTUNDA2)	LUG RON2 At4g32551 L23H3.30	Arabidopsis thaliana (Mouse-ear cress)		Alternative splicing; Complete proteome; Developmental protein; Differentiation; Flowering; Nucleus; Reference proteome; Repeat; Repressor; Transcription; Transcription regulation; WD repeat
SRR342017_TR24619_c3_g5_i1_len=2646	PAO4_ARATH	Q:691-2139,H:11-497	68.79%ID	E:0	Probable polyamine oxidase 4	PF00890.21^FAD_binding_2^FAD binding domain^33-76^E:3.6e-05`PF13450.3^NAD_binding_8^NAD(P)-binding Rossmann-like domain^35-101^E:8.6e-15`PF01593.21^Amino_oxidase^Flavin containing amine oxidoreductase^40-459^E:3.3e-88	0,704	0,186	0	2,2	0,319	1,051	0,228	1,083	0,188	-0,452	PAO4_ARATH	Probable polyamine oxidase 4 (AtPAO4) (EC 1.5.3.16) (Amine oxidase 2)	PAO4 At1g65840 F1E22.18	Arabidopsis thaliana (Mouse-ear cress)		Complete proteome; FAD; Flavoprotein; Oxidoreductase; Peroxisome; Reference proteome
SRR342017_TR24903_c0_g4_i1_len=3970	PSAA_EUCGG	Q:3431-1182,H:1-750	99.33%ID	E:0	Photosystem I P700 chlorophyll a apoprotein A1 {ECO:0000255|HAMAP-Rule:MF_00458}	PF00223.16^PsaA_PsaB^Photosystem I psaA/psaB protein^38-750^E:0	0,058	0,819	0	2,022	0	-1,767	0	-1,43	1	-0,304	PSAA_EUCGG	Photosystem I P700 chlorophyll a apoprotein A1 (EC 1.97.1.12) (PSI-A) (PsaA)	psaA	Eucalyptus globulus subsp. globulus (Tasmanian blue gum)		4Fe-4S; Chlorophyll; Chloroplast; Chromophore; Electron transport; Iron; Iron-sulfur; Magnesium; Membrane; Metal-binding; Oxidoreductase; Photosynthesis; Photosystem I; Plastid; Thylakoid; Transmembrane; Transmembrane helix; Transport
SRR342017_TR30377_c3_g5_i5_len=2173	RP45C_ARATH	Q:389-87,H:261-361	71.29%ID	E:3e-41	Polyadenylate-binding protein RBP45C	PF01490.15^Aa_trans^Transmembrane amino acid transporter protein^33-415^E:1.9e-75	1	0,017	0	2,075	0,517	0,922	0,732	0,678	1	0,032	RP45C_ARATH	Polyadenylate-binding protein RBP45C (Poly(A)-binding protein RBP45C) (RNA-binding protein 45C) (AtRBP45C)	RBP45C At4g27000 F10M23.340	Arabidopsis thaliana (Mouse-ear cress)		Complete proteome; Nucleus; RNA-binding; Reference proteome; Repeat; mRNA processing
SRR342017_TR31799_c2_g4_i2_len=1294	CLC1_ARATH	Q:349-900,H:56-241	63.44%ID	E:1e-57	Clathrin light chain 1	PF01086.14^Clathrin_lg_ch^Clathrin light chain^131-271^E:4.4e-10	1	0,009	0	1,741	0,196	0,531	0,085	0,691	1	0,011	CLC1_ARATH	Clathrin light chain 1	At2g20760 F5H14.27	Arabidopsis thaliana (Mouse-ear cress)		Coated pit; Coiled coil; Complete proteome; Cytoplasmic vesicle; Membrane; Reference proteome
SRR342017_TR32897_c1_g10_i4_len=2602	TRPB_CAMAC	Q:1236-2600,H:1-451	83.99%ID	E:0	Tryptophan synthase beta chain 2, chloroplastic	PF00291.22^PALP^Pyridoxal-phosphate dependent enzyme^126-455^E:3.5e-49	0,154	0,522	0	3,095	0,895	-0,13	0,788	-0,159	0,011	0,663	TRPB_CAMAC	Tryptophan synthase beta chain 2, chloroplastic (EC 4.2.1.20)	TSB	Camptotheca acuminata (Happy tree)	PATHWAY: Amino-acid biosynthesis; L-tryptophan biosynthesis; L-tryptophan from chorismate: step 5/5.	Amino-acid biosynthesis; Aromatic amino acid biosynthesis; Chloroplast; Lyase; Plastid; Pyridoxal phosphate; Transit peptide; Tryptophan biosynthesis
SRR342017_TR33069_c2_g1_i2_len=3355	SPE1_PEA	Q:1075-3267,H:1-727	70.79%ID	E:0	Arginine decarboxylase	PF02784.13^Orn_Arg_deC_N^Pyridoxal-dependent decarboxylase, pyridoxal binding domain^140-410^E:2.1e-40`PF00278.19^Orn_DAP_Arg_deC^Pyridoxal-dependent decarboxylase, C-terminal sheet domain^498-612^E:7.4e-19	1	-0,072	0	2,609	0,656	0,344	0,114	0,82	0,536	-0,184	SPE1_PEA	Arginine decarboxylase (ADC) (ARGDC) (EC 4.1.1.19)		Pisum sativum (Garden pea)	PATHWAY: Amine and polyamine biosynthesis; agmatine biosynthesis; agmatine from L-arginine: step 1/1.	Decarboxylase; Lyase; Magnesium; Putrescine biosynthesis; Pyridoxal phosphate; Spermidine biosynthesis
SRR342017_TR33314_c2_g1_i3_len=802	NA	NA	NA	NA	NA	.	1	-0,132	0	7,425	1	0	1	0	0,528	-0,317	.	.	.	.	.	.
SRR342017_TR33422_c0_g1_i1_len=791	NA	NA	NA	NA	NA	PF04770.9^ZF-HD_dimer^ZF-HD protein dimerisation region^26-77^E:2.8e-28	1	0,238	0	4,093	0,597	0,368	1	-0,202	0,811	-0,317	.	.	.	.	.	.
SRR342019_TR881_c0_g5_i3_len=2314	WRK19_ARATH	Q:839-1309,H:91-297	56.52%ID	E:8e-60	Probable WRKY transcription factor 19	.	1	0,13	0,001	2,192	1	0,113	0,777	-0,186	0,874	0,198	WRK19_ARATH	Probable WRKY transcription factor 19 (WRKY DNA-binding protein 19)	WRKY19 At4g12020 F16J13.90	Arabidopsis thaliana (Mouse-ear cress)		ATP-binding; Alternative splicing; Complete proteome; DNA-binding; Kinase; Leucine-rich repeat; Nucleotide-binding; Nucleus; Plant defense; Reference proteome; Repeat; Serine/threonine-protein kinase; Transcription; Transcription regulation; Transferase
SRR342019_TR1150_c9_g1_i4_len=633	NA	NA	NA	NA	NA	.	0,934	-0,05	0	6,847	1	0	1	2,444	0,803	0,14	.	.	.	.	.	.
SRR342019_TR2208_c1_g2_i4_len=2113	ACOT8_MOUSE	Q:773-1633,H:24-310	44.18%ID	E:2e-62	Acyl-coenzyme A thioesterase 8	PF00027.26^cNMP_binding^Cyclic nucleotide-binding domain^34-112^E:9.2e-11`PF13622.3^4HBT_3^Thioesterase-like superfamily^161-414^E:5.4e-56`PF02551.12^Acyl_CoA_thio^Acyl-CoA thioesterase^307-414^E:2.2e-26	0,609	0,267	0	2,186	0,034	1,035	0,006	1,286	1	1,676	ACOT8_MOUSE	Acyl-coenzyme A thioesterase 8 (Acyl-CoA thioesterase 8) (EC 3.1.2.27) (Choloyl-coenzyme A thioesterase) (Peroxisomal acyl-CoA thioesterase 2) (PTE-2) (Peroxisomal acyl-coenzyme A thioester hydrolase 1) (PTE-1) (Peroxisomal long-chain acyl-CoA thioesterase 1)	Acot8 Pte1 Pte2	Mus musculus (Mouse)		Complete proteome; Hydrolase; Peroxisome; Peroxisome biogenesis; Reference proteome; Serine esterase
SRR342019_TR2813_c0_g4_i7_len=2773	MT878_ARATH	Q:1045-20,H:1-347	44.44%ID	E:6e-78	Probable S-adenosylmethionine-dependent methyltransferase At5g38780	PF03492.12^Methyltransf_7^SAM dependent carboxyl methyltransferase^69-350^E:4e-101	0,011	0,897	0	2,763	0,002	1,368	0,666	0,255	0,196	0,782	MT878_ARATH	Probable S-adenosylmethionine-dependent methyltransferase At5g38780 (EC 2.1.1.-)	At5g38780 MKD10.80	Arabidopsis thaliana (Mouse-ear cress)		Complete proteome; Methyltransferase; Reference proteome; S-adenosyl-L-methionine; Transferase
SRR342019_TR2947_c0_g1_i3_len=584	NA	NA	NA	NA	NA	.	0,766	-0,153	0	4,794	1	0,31	1	-1,505	0,419	-0,476	.	.	.	.	.	.
SRR342019_TR2947_c2_g4_i3_len=521	NA	NA	NA	NA	NA	.	0,704	0,212	0	7,203	1	-1,628	1	-1,628	0,079	-0,87	.	.	.	.	.	.
SRR342019_TR3224_c3_g3_i2_len=3520	AGLU_SPIOL	Q:2071-3369,H:471-902	59.73%ID	E:2e-172	Alpha-glucosidase	PF01055.23^Glyco_hydro_31^Glycosyl hydrolases family 31^1-168^E:5.9e-59	0,364	0,475	0	1,626	0,517	0,817	1	0,353	0,254	4,494	AGLU_SPIOL	Alpha-glucosidase (EC 3.2.1.20) (Maltase)		Spinacia oleracea (Spinach)		Direct protein sequencing; Glycoprotein; Glycosidase; Hydrolase; Signal
SRR342019_TR3806_c0_g1_i3_len=4254	TRPX_ARATH	Q:3320-1599,H:26-607	74.4%ID	E:0	Anthranilate synthase alpha subunit 2, chloroplastic	PF04715.10^Anth_synt_I_N^Anthranilate synthase component I, N terminal region^104-258^E:1.9e-30`PF00425.15^Chorismate_bind^chorismate binding enzyme^319-590^E:3.9e-85	0,155	1,125	0	4,855	1	-0,404	0,381	-1,909	1	3,219	TRPX_ARATH	Anthranilate synthase alpha subunit 2, chloroplastic (EC 4.1.3.27) (Anthranilate synthase component 1-2) (Anthranilate synthase component I-2)	ASA2 At2g29690 T27A16.21	Arabidopsis thaliana (Mouse-ear cress)	PATHWAY: Amino-acid biosynthesis; L-tryptophan biosynthesis; L-tryptophan from chorismate: step 1/5.	Amino-acid biosynthesis; Aromatic amino acid biosynthesis; Chloroplast; Complete proteome; Lyase; Plastid; Reference proteome; Transit peptide; Tryptophan biosynthesis
SRR342019_TR5968_c2_g10_i4_len=2743	Y005_SYNY3	Q:539-1831,H:107-520	35.86%ID	E:2e-83	Uncharacterized protein sll0005	PF03109.13^ABC1^ABC1 family^124-241^E:3.1e-31	0,025	3,442	0	2,739	0,039	1,086	0,255	0,653	0,839	0,111	Y005_SYNY3	Uncharacterized protein sll0005	sll0005	Synechocystis sp. (strain PCC 6803 / Kazusa)		Complete proteome; Reference proteome
SRR342019_TR7099_c4_g2_i7_len=2073	GEDH1_OCIBA	Q:429-953,H:68-242	62.86%ID	E:4e-48	Geraniol dehydrogenase 1	PF08240.9^ADH_N^Alcohol dehydrogenase GroES-like domain^4-82^E:4e-15`PF02826.16^2-Hacid_dh_C^D-isomer specific 2-hydroxyacid dehydrogenase, NAD binding domain^105-155^E:3.2e-07	0,254	4,397	0	6,832	0,001	-6,492	0,001	-6,492	0,005	-6,197	GEDH1_OCIBA	Geraniol dehydrogenase 1 (ObaGEDH1) (EC 1.1.1.183)	GEDH1	Ocimum basilicum (Sweet basil)		Metal-binding; NADP; Oxidoreductase; Zinc
SRR342019_TR7176_c0_g1_i1_len=1811	DDC_CATRO	Q:1360-194,H:112-500	98.46%ID	E:0	Aromatic-L-amino-acid decarboxylase	PF00282.16^Pyridoxal_deC^Pyridoxal-dependent decarboxylase conserved domain^4-333^E:5e-102	0,011	0,59	0	3,432	0,937	0,028	0	-1,39	1	0,323	DDC_CATRO	Aromatic-L-amino-acid decarboxylase (AADC) (EC 4.1.1.28) (DOPA decarboxylase) (Tryptophan decarboxylase)	TDC	Catharanthus roseus (Madagascar periwinkle) (Vinca rosea)		Decarboxylase; Lyase; Pyridoxal phosphate
SRR342019_TR8002_c0_g2_i1_len=1154	ARGE_ARATH	Q:1084-26,H:1-357	77.31%ID	E:0	Acetylornithine deacetylase	PF01546.25^Peptidase_M20^Peptidase family M20/M25/M40^94-282^E:5.8e-20`PF07687.11^M20_dimer^Peptidase dimerisation domain^202-315^E:2.3e-13	0,316	0,634	0	3,52	0,08	1,206	0,057	1,315	0,502	3,977	ARGE_ARATH	Acetylornithine deacetylase (EC 3.5.1.16) (N-acetylornithinase) (AO) (Acetylornithinase) (NAO)	At4g17830 dl4945w T6K21	Arabidopsis thaliana (Mouse-ear cress)	PATHWAY: Amino-acid biosynthesis; L-arginine biosynthesis; L-ornithine from N(2)-acetyl-L-ornithine (linear): step 1/1.	Alternative splicing; Amino-acid biosynthesis; Arginine biosynthesis; Cobalt; Complete proteome; Hydrolase; Metal-binding; Reference proteome; Zinc
SRR342019_TR8266_c0_g2_i1_len=1510	MY1R1_SOLTU	Q:413-886,H:24-188	57.89%ID	E:9e-39	Transcription factor MYB1R1	PF00249.28^Myb_DNA-binding^Myb-like DNA-binding domain^107-151^E:4.6e-11	0,008	1,289	0	2,197	0,168	0,459	0,722	0,143	0,025	1,171	MY1R1_SOLTU	Transcription factor MYB1R1 (Myb-related protein R1) (StMYB1R-1)		Solanum tuberosum (Potato)		Complete proteome; Cytoplasm; DNA-binding; Nucleus; Reference proteome; Stress response; Transcription; Transcription regulation
SRR342019_TR8733_c0_g1_i4_len=2110	ASPG1_ARATH	Q:1530-337,H:177-500	28.64%ID	E:3e-26	Protein ASPARTIC PROTEASE IN GUARD CELL 1	PF14543.3^TAXi_N^Xylanase inhibitor N-terminal^83-265^E:3.6e-28`PF00026.20^Asp^Eukaryotic aspartyl protease^90-490^E:6e-07`PF14541.3^TAXi_C^Xylanase inhibitor C-terminal^309-489^E:1.8e-28	0,004	2,399	0	4,149	0,002	1,894	0,057	1,293	1	-0,93	ASPG1_ARATH	Protein ASPARTIC PROTEASE IN GUARD CELL 1 (AtASPG1) (EC 3.4.23.-)	ASPG1 At3g18490 MYF24.22	Arabidopsis thaliana (Mouse-ear cress)		Aspartyl protease; Complete proteome; DNA-binding; Disulfide bond; Endoplasmic reticulum; Hydrolase; Protease; Reference proteome; Signal
SRR342019_TR8982_c2_g3_i3_len=977	MTDH_APIGR	Q:648-4,H:2-216	62.79%ID	E:7e-84	Mannitol dehydrogenase	PF08240.9^ADH_N^Alcohol dehydrogenase GroES-like domain^39-151^E:2.5e-22	1	0,575	0	6,872	0,066	5,362	0,009	5,984	1	-0,318	MTDH_APIGR	Mannitol dehydrogenase (EC 1.1.1.255) (NAD-dependent mannitol dehydrogenase)	MTD	Apium graveolens (Celery)		Cytoplasm; Metal-binding; NAD; Oxidoreductase; Zinc
SRR342019_TR9643_c2_g2_i3_len=2153	STSY_RAUMA	Q:1165-2031,H:35-322	43.34%ID	E:2e-72	Strictosidine synthase	PF02121.15^IP_trans^Phosphatidylinositol transfer protein^4-218^E:2.4e-82	0,001	6,53	0	6,787	0,025	1,83	0,038	1,732	1	0	STSY_RAUMA	Strictosidine synthase (EC 4.3.3.2) (Fragment)	STR1	Rauvolfia mannii	PATHWAY: Alkaloid biosynthesis; 3alpha(S)-strictosidine biosynthesis; 3alpha(S)-strictosidine from secologanin and tryptamine: step 1/1.	Alkaloid metabolism; Glycoprotein; Lyase; Signal; Vacuole
SRR342019_TR9656_c0_g2_i1_len=712	PR1B4_ARATH	Q:253-708,H:8-159	64.05%ID	E:9e-49	PRA1 family protein B4	.	0,547	0,318	0	2,81	0,695	-0,174	0,878	-0,088	1	-0,004	PR1B4_ARATH	PRA1 family protein B4 (AtPRA1.B4)	PRA1B4 At2g38360 T19C21.15	Arabidopsis thaliana (Mouse-ear cress)		Complete proteome; Endosome; Membrane; Reference proteome; Transmembrane; Transmembrane helix; Transport
SRR342019_TR10001_c1_g1_i1_len=984	NA	NA	NA	NA	NA	.	0,006	1,834	0	6,924	0,005	6,125	0,005	6,164	1	0	.	.	.	.	.	.
SRR342019_TR10238_c0_g2_i2_len=782	NA	NA	NA	NA	NA	PF04588.10^HIG_1_N^Hypoxia induced protein conserved region^21-71^E:5.4e-10	1	-0,063	0	2,091	0,472	0,755	0,352	0,889	0,832	-0,291	.	.	.	.	.	.
SRR342019_TR11670_c0_g7_i1_len=2195	P2A13_ARATH	Q:950-114,H:1-286	61.89%ID	E:2e-120	F-box protein PP2-A13	PF00646.30^F-box^F-box domain^56-97^E:0.0054`PF14299.3^PP2^Phloem protein 2^152-310^E:1.3e-36	0,079	1,1	0	2,954	0,004	0,758	0,013	0,651	0,007	1,163	P2A13_ARATH	F-box protein PP2-A13 (Protein PHLOEM PROTEIN 2-LIKE A13) (AtPP2-A13) (SKP1-interacting partner 9)	PP2A13 SKIP9 At3g61060 T27I15.150	Arabidopsis thaliana (Mouse-ear cress)	PATHWAY: Protein modification; protein ubiquitination.	Alternative splicing; Complete proteome; Nucleus; Reference proteome; Ubl conjugation pathway
SRR342019_TR13074_c0_g1_i1_len=2903	RFS_PEA	Q:2645-327,H:4-798	68.72%ID	E:0	Galactinol–sucrose galactosyltransferase	PF05691.9^Raffinose_syn^Raffinose synthase or seed imbibition protein Sip1^25-758^E:0	0,077	0,788	0	1,638	0,07	-0,504	0,016	-0,673	0,005	1,186	RFS_PEA	Galactinol–sucrose galactosyltransferase (EC 2.4.1.82) (Raffinose synthase)	RFS	Pisum sativum (Garden pea)		Carbohydrate metabolism; Glycosyltransferase; Transferase
SRR342019_TR13756_c1_g1_i1_len=1526	MOMT_CATRO	Q:311-1354,H:1-348	93.97%ID	E:0	Myricetin O-methyltransferase	PF08100.8^Dimerisation^Dimerisation domain^27-74^E:8.4e-20`PF00891.15^Methyltransf_2^O-methyltransferase^117-334^E:1.7e-58	1	-0,067	0	2,773	0,56	0,859	1	0,486	0,745	-0,179	MOMT_CATRO	Myricetin O-methyltransferase (EC 2.1.1.267) (CrOMT2) (Flavonoid O-methyltransferase)		Catharanthus roseus (Madagascar periwinkle) (Vinca rosea)		Acetylation; Direct protein sequencing; Methyltransferase; S-adenosyl-L-methionine; Transferase
SRR342019_TR15806_c0_g2_i1_len=1588	UBQ4_ARATH	Q:95-1240,H:1-382	100%ID	E:0	Polyubiquitin 4	PF11976.5^Rad60-SLD^Ubiquitin-2 like Rad60 SUMO-like^1-71^E:1.4e-15`PF00240.20^ubiquitin^Ubiquitin family^3-74^E:2.1e-33`PF14560.3^Ubiquitin_2^Ubiquitin-like domain^14-69^E:0.00096`PF11976.5^Rad60-SLD^Ubiquitin-2 like Rad60 SUMO-like^77-147^E:1.4e-15`PF00240.20^ubiquitin^Ubiquitin family^79-150^E:2.1e-33`PF14560.3^Ubiquitin_2^Ubiquitin-like domain^88-145^E:0.00088`PF11976.5^Rad60-SLD^Ubiquitin-2 like Rad60 SUMO-like^153-223^E:1.4e-15`PF00240.20^ubiquitin^Ubiquitin family^155-226^E:2.1e-33`PF14560.3^Ubiquitin_2^Ubiquitin-like domain^164-221^E:0.00088`PF11976.5^Rad60-SLD^Ubiquitin-2 like Rad60 SUMO-like^229-299^E:1.4e-15`PF00240.20^ubiquitin^Ubiquitin family^231-302^E:2.1e-33`PF14560.3^Ubiquitin_2^Ubiquitin-like domain^240-297^E:0.00088`PF11976.5^Rad60-SLD^Ubiquitin-2 like Rad60 SUMO-like^305-375^E:1.4e-15`PF00240.20^ubiquitin^Ubiquitin family^307-378^E:2.1e-33`PF14560.3^Ubiquitin_2^Ubiquitin-like domain^316-373^E:0.00088	0,788	0,092	0	1,385	0,025	0,81	0,001	1,114	0,269	0,308	UBQ4_ARATH	Polyubiquitin 4 [Cleaved into: Ubiquitin]	UBQ4 At5g20620 T1M15.20	Arabidopsis thaliana (Mouse-ear cress)		Complete proteome; Cytoplasm; Isopeptide bond; Nucleus; Reference proteome; Repeat; Ubl conjugation; Ubl conjugation pathway
SRR342019_TR16674_c1_g1_i1_len=1211	NA	NA	NA	NA	NA	PF00010.23^HLH^Helix-loop-helix DNA-binding domain^14-60^E:5.1e-09	0,175	0,917	0	3,766	0,003	2,942	0,01	2,774	1	0,216	.	.	.	.	.	.
SRR342019_TR18903_c8_g6_i2_len=4370	ADS3_ARATH	Q:3244-4218,H:54-367	71.78%ID	E:1e-147	Palmitoyl-monogalactosyldiacylglycerol delta-7 desaturase, chloroplastic {ECO:0000303|PubMed:15579662}	PF00487.21^FA_desaturase^Fatty acid desaturase^142-354^E:4e-17	1	-0,002	0	3,337	1	-0,188	0,56	0,636	0,705	0,18	ADS3_ARATH	Palmitoyl-monogalactosyldiacylglycerol delta-7 desaturase, chloroplastic (EC 1.14.19.-) (FAD5) (Monogalactosyldiacylglycerol-specific palmitic acid desaturase)	ADS3 At3g15850 MSJ11.25	Arabidopsis thaliana (Mouse-ear cress)	PATHWAY: Lipid metabolism; oxylipin biosynthesis.; PATHWAY: Lipid metabolism; polyunsaturated fatty acid biosynthesis.	Chloroplast; Complete proteome; Fatty acid biosynthesis; Fatty acid metabolism; Iron; Lipid biosynthesis; Lipid metabolism; Membrane; Oxidoreductase; Oxylipin biosynthesis; Plastid; Reference proteome; Transit peptide; Transmembrane; Transmembrane helix
SRR342019_TR20560_c4_g2_i7_len=2144	BAT1_ORYSJ	Q:1615-116,H:19-518	82.8%ID	E:0	Amino-acid permease BAT1 homolog	PF13520.3^AA_permease_2^Amino acid permease^43-499^E:2.4e-61`PF00324.18^AA_permease^Amino acid permease^67-518^E:2e-24	0,044	1,148	0	2,091	0,548	-0,277	0,13	-0,67	1	-0,136	BAT1_ORYSJ	Amino-acid permease BAT1 homolog	BAT1 Os01g0607200 LOC_Os01g42234 OsJ_02547	Oryza sativa subsp. japonica (Rice)		Amino-acid transport; Complete proteome; Membrane; Reference proteome; Transmembrane; Transmembrane helix; Transport
SRR342019_TR20794_c0_g2_i1_len=563	GAT1_ARATH	Q:282-563,H:13-106	71.28%ID	E:2e-29	GABA transporter 1	PF01490.15^Aa_trans^Transmembrane amino acid transporter protein^41-117^E:4.7e-13	0,002	6,299	0	3,381	1	0,093	1	0,18	0,692	-0,742	GAT1_ARATH	GABA transporter 1 (AtGAT1) (Bidirectional amino acid transporter 1)	GAT1 BAT1 At1g08230 T23G18.9	Arabidopsis thaliana (Mouse-ear cress)		Amino-acid transport; Cell membrane; Complete proteome; Membrane; Reference proteome; Transmembrane; Transmembrane helix; Transport
SRR342019_TR21130_c0_g1_i1_len=674	10HGO_CATRO	Q:674-120,H:176-360	72.43%ID	E:1e-71	8-hydroxygeraniol dehydrogenase	PF00107.23^ADH_zinc_N^Zinc-binding dehydrogenase^19-142^E:3.7e-18	0,226	1,935	0	4,179	0,066	5,39	0,034	5,525	1	-0,106	10HGO_CATRO	8-hydroxygeraniol dehydrogenase (Cr10HGO) (EC 1.1.1.324)	10HGO	Catharanthus roseus (Madagascar periwinkle) (Vinca rosea)		Metal-binding; NADP; Oxidoreductase; Zinc
SRR342019_TR21911_c0_g1_i5_len=1886	BT2_ARATH	Q:1171-1593,H:213-362	59.33%ID	E:1e-46	BTB/POZ and TAZ domain-containing protein 2	PF02135.13^zf-TAZ^TAZ zinc finger^1-84^E:3.6e-12	0,017	1,555	0	1,832	1	0,918	1	0,8	0,938	-0,055	BT2_ARATH	BTB/POZ and TAZ domain-containing protein 2 (BTB and TAZ domain protein 2)	BT2 At3g48360 T29H11.120	Arabidopsis thaliana (Mouse-ear cress)	PATHWAY: Protein modification; protein ubiquitination.	Complete proteome; Cytoplasm; Metal-binding; Nucleus; Reference proteome; Ubl conjugation pathway; Zinc; Zinc-finger
SRR342019_TR22699_c0_g1_i2_len=2163	GH31_ARATH	Q:1580-201,H:139-589	81.78%ID	E:0	Probable indole-3-acetic acid-amido synthetase GH3.1	PF03321.10^GH3^GH3 auxin-responsive promoter^8-438^E:5.2e-165	1	2,635	0	7,091	0,502	4,176	1	3,148	1	-1,043	GH31_ARATH	Probable indole-3-acetic acid-amido synthetase GH3.1 (EC 6.3.2.-) (Auxin-responsive GH3-like protein 1) (AtGH3-1)	GH3.1 At2g14960 T26I20.12	Arabidopsis thaliana (Mouse-ear cress)		Complete proteome; Ligase; Reference proteome
SRR342019_TR22855_c4_g10_i3_len=2051	BAS1_ORYSJ	Q:863-276,H:66-261	95.41%ID	E:8e-130	2-Cys peroxiredoxin BAS1, chloroplastic	PF00578.18^AhpC-TSA^AhpC/TSA family^78-211^E:5e-41`PF08534.7^Redoxin^Redoxin^78-213^E:7.1e-15`PF10417.6^1-cysPrx_C^C-terminal domain of 1-Cys peroxiredoxin^232-267^E:5e-11	0,239	1,242	0	6,191	0,034	5,645	0,502	3,841	1	0	BAS1_ORYSJ	2-Cys peroxiredoxin BAS1, chloroplastic (EC 1.11.1.15) (Thiol-specific antioxidant protein)	BAS1 Os02g0537700 LOC_Os02g33450 OSJNBa0014M17.2 P0508B05.23	Oryza sativa subsp. japonica (Rice)		Antioxidant; Chloroplast; Complete proteome; Direct protein sequencing; Disulfide bond; Hydrogen peroxide; Oxidoreductase; Peroxidase; Plastid; Redox-active center; Reference proteome; Transit peptide
SRR342019_TR24464_c0_g10_i2_len=652	GSHRC_PEA	Q:117-650,H:239-416	81.46%ID	E:3e-103	Glutathione reductase, cytosolic	PF07992.11^Pyr_redox_2^Pyridine nucleotide-disulphide oxidoreductase^14-127^E:4.6e-28`PF00070.24^Pyr_redox^Pyridine nucleotide-disulphide oxidoreductase^14-53^E:7.6e-05	0,326	-0,366	0	1,403	0,054	0,906	0,132	0,717	0,512	0,186	GSHRC_PEA	Glutathione reductase, cytosolic (GR) (GRase) (EC 1.8.1.7) (GOR2)		Pisum sativum (Garden pea)		Cytoplasm; Disulfide bond; FAD; Flavoprotein; NADP; Oxidoreductase; Redox-active center
SRR342019_TR24849_c2_g4_i2_len=2320	NA	NA	NA	NA	NA	PF01699.21^Na_Ca_ex^Sodium/calcium exchanger protein^74-250^E:1.8e-08`PF13833.3^EF-hand_8^EF-hand domain pair^278-316^E:0.0007`PF13499.3^EF-hand_7^EF-hand domain pair^291-356^E:7.1e-11`PF13202.3^EF-hand_5^EF hand^294-318^E:2.4e-05`PF01699.21^Na_Ca_ex^Sodium/calcium exchanger protein^422-496^E:2.2e-05	0,513	-0,221	0	1,91	0,1	-0,79	0,007	0,995	0,555	0,327	.	.	.	.	.	.
SRR342019_TR26415_c0_g1_i1_len=1209	PS12A_ARATH	Q:1042-62,H:4-330	79.51%ID	E:5e-179	26S proteasome non-ATPase regulatory subunit 12 homolog A	.	0,415	-0,386	0	3,262	0,496	-0,414	0,691	-0,211	0,692	0,921	PS12A_ARATH	26S proteasome non-ATPase regulatory subunit 12 homolog A (26S proteasome regulatory subunit RPN5a) (AtRPN5a) (26S proteasome regulatory subunit p55 homolog A) (Protein EMBRYO DEFECTIVE 2107) (Protein MARIPOSA)	RPN5A EMB2107 MSA At5g09900 MYH9.11	Arabidopsis thaliana (Mouse-ear cress)		Alternative splicing; Coiled coil; Complete proteome; Cytoplasm; Nucleus; Proteasome; Reference proteome
SRR342019_TR30583_c1_g2_i7_len=1140	DNJH2_ALLPO	Q:153-905,H:168-418	84.86%ID	E:7e-136	DnaJ protein homolog 2	PF00684.16^DnaJ_CXXCXGXG^DnaJ central domain^9-69^E:1.6e-12`PF01556.15^DnaJ_C^DnaJ C terminal domain^28-198^E:1.9e-32	0,427	0,265	0	1,57	0,91	0,049	0,908	-0,038	0,878	0,046	DNJH2_ALLPO	DnaJ protein homolog 2	LDJ2	Allium porrum (Leek) (Allium ampeloprasum var. porrum)		Chaperone; Lipoprotein; Membrane; Metal-binding; Methylation; Prenylation; Repeat; Zinc; Zinc-finger
SRR342019_TR31285_c1_g3_i1_len=722	NA	NA	NA	NA	NA	PF04061.11^ORMDL^ORMDL family^15-112^E:4.1e-37	0,049	-1,238	0	4,118	0,502	3,775	1	0	0,719	0,326	.	.	.	.	.	.
SRR342019_TR31366_c0_g2_i1_len=724	21KD_DAUCA	Q:174-659,H:28-189	56.17%ID	E:7e-65	21 kDa protein	PF04043.12^PMEI^Plant invertase/pectin methylesterase inhibitor^57-213^E:5.3e-35	0,002	0,727	0	2,15	0,006	0,725	0,26	0,311	0,596	-0,123	21KD_DAUCA	21 kDa protein (1.2 protein)		Daucus carota (Wild carrot)		Signal
SRR342019_TR34950_c0_g1_i1_len=1157	ERF4_NICSY	Q:886-239,H:15-226	57.27%ID	E:7e-40	Ethylene-responsive transcription factor 4	PF00847.17^AP2^AP2 domain^25-74^E:2.2e-14	0,037	1,33	0	3,118	0,04	0,695	0,359	0,346	0,306	0,541	ERF4_NICSY	Ethylene-responsive transcription factor 4 (Ethylene-responsive element-binding factor 3) (EREBP-3) (Ethylene-responsive element-binding factor 4 homolog) (NsERF3)	ERF4 ERF-3 ERF3	Nicotiana sylvestris (Wood tobacco) (South American tobacco)		DNA-binding; Ethylene signaling pathway; Nucleus; Plant defense; Repressor; Transcription; Transcription regulation
SRR342019_TR35374_c1_g1_i3_len=646	CYT5_ARATH	Q:455-198,H:28-114	62.07%ID	E:5e-33	Cysteine proteinase inhibitor 5	PF00031.18^Cystatin^Cystatin domain^34-119^E:4.4e-15`PF16845.2^SQAPI^Aspartic acid proteinase inhibitor^39-119^E:7.2e-37`PF07430.8^PP1^Phloem filament protein PP1 cystatin-like domain^47-115^E:0.00013	0,707	-0,301	0	2,713	1	0,006	0,746	-0,128	0,502	3,201	CYT5_ARATH	Cysteine proteinase inhibitor 5 (AtCYS-5)	CYS5 At5g47550 MNJ7.14	Arabidopsis thaliana (Mouse-ear cress)		Complete proteome; Glycoprotein; Plant defense; Protease inhibitor; Reference proteome; Secreted; Signal; Thiol protease inhibitor
SRR342019_TR36227_c6_g7_i9_len=2492	C7BL3_CICIN	Q:1627-245,H:33-486	38.53%ID	E:3e-114	Costunolide synthase	PF00067.19^p450^Cytochrome P450^62-508^E:6.2e-86	0,002	0,908	0	2,746	0,102	0,588	0,439	-0,362	0,597	0,397	C7BL3_CICIN	Costunolide synthase (CiCOS) (EC 1.14.13.120) (Cytochrome P450 71BL3)	CYP71BL3 COS	Cichorium intybus (Chicory)		Heme; Iron; Membrane; Metal-binding; Monooxygenase; NADP; Oxidoreductase; Transmembrane; Transmembrane helix
SRR342019_TR36910_c0_g2_i1_len=1187	NA	NA	NA	NA	NA	PF00612.24^IQ^IQ calmodulin-binding motif^68-86^E:3.5e-05`PF02179.13^BAG^BAG domain^91-163^E:4.7e-11	0,44	0,686	0	3,16	1	0,505	0,517	0,972	0,732	-0,62	.	.	.	.	.	.
SRR342019_TR37006_c3_g3_i1_len=754	TBA4_ARATH	Q:510-1,H:1-170	98.82%ID	E:7e-104	Tubulin alpha-4 chain	PF00091.22^Tubulin^Tubulin/FtsZ family, GTPase domain^3-168^E:3.8e-52	0	-1,042	0	4,269	0,044	-0,759	0,206	-0,465	0,026	0,602	TBA4_ARATH	Tubulin alpha-4 chain	TUBA4 TUA4 At1g04820 F13M7.19	Arabidopsis thaliana (Mouse-ear cress)		Acetylation; Complete proteome; Cytoplasm; Cytoskeleton; GTP-binding; Microtubule; Nucleotide-binding; Phosphoprotein; Reference proteome
SRR342019_TR37278_c0_g1_i5_len=2170	LAL5_ARATH	Q:290-1687,H:8-464	59.87%ID	E:7e-168	.	PF01554.15^MatE^MatE^57-216^E:1.4e-29`PF01554.15^MatE^MatE^278-439^E:1.1e-25	0,594	0,232	0	5,566	0,117	0,928	0,196	0,809	0,002	1,855	DTX18_ARATH	Protein DETOXIFICATION 18 (AtDTX18) (Multidrug and toxic compound extrusion protein 18) (MATE protein 18) (Protein LIKE ALF5)	DTX18 LAL5 At3g23550 MDB19.3	Arabidopsis thaliana (Mouse-ear cress)		Complete proteome; Membrane; Reference proteome; Transmembrane; Transmembrane helix; Transport
SRR342019_TR37281_c0_g2_i1_len=1210	MSBP2_ARATH	Q:212-718,H:1-168	77.51%ID	E:6e-78	Membrane steroid-binding protein 2	PF00173.25^Cyt-b5^Cytochrome b5-like Heme/Steroid binding domain^73-167^E:1.6e-13	0,276	0,25	0	1,577	0,027	0,561	0,05	0,502	0,738	0,079	MSBP2_ARATH	Membrane steroid-binding protein 2 (AtMP2) (Membrane-associated progesterone-binding protein 3) (AtMAPR3)	MSBP2 MAPR3 MP2 At3g48890 T21J18.160	Arabidopsis thaliana (Mouse-ear cress)		Cell membrane; Complete proteome; Lipid-binding; Membrane; Phosphoprotein; Reference proteome; Steroid-binding; Transmembrane; Transmembrane helix
SRR342019_TR37498_c2_g6_i1_len=2647	INT1_ARATH	Q:1936-398,H:3-496	69.75%ID	E:0	Inositol transporter 1	PF00083.21^Sugar_tr^Sugar (and other) transporter^11-300^E:8.6e-80`PF07690.13^MFS_1^Major Facilitator Superfamily^11-249^E:8.2e-12	0,211	0,688	0	2,723	0,045	1,359	0,004	1,814	1	-0,498	INT1_ARATH	Inositol transporter 1	INT1 At2g43330 T1O24.7	Arabidopsis thaliana (Mouse-ear cress)		Complete proteome; Membrane; Reference proteome; Symport; Transmembrane; Transmembrane helix; Transport; Vacuole
SRR342019_TR38071_c4_g2_i20_len=2287	ARGE_ARATH	Q:260-1075,H:1-275	69.2%ID	E:3e-130	Acetylornithine deacetylase	PF01546.25^Peptidase_M20^Peptidase family M20/M25/M40^95-228^E:4e-19`PF07687.11^M20_dimer^Peptidase dimerisation domain^203-243^E:0.00013	0,076	2,943	0	4,384	0,017	3,533	0,129	2,627	1	0,798	ARGE_ARATH	Acetylornithine deacetylase (EC 3.5.1.16) (N-acetylornithinase) (AO) (Acetylornithinase) (NAO)	At4g17830 dl4945w T6K21	Arabidopsis thaliana (Mouse-ear cress)	PATHWAY: Amino-acid biosynthesis; L-arginine biosynthesis; L-ornithine from N(2)-acetyl-L-ornithine (linear): step 1/1.	Alternative splicing; Amino-acid biosynthesis; Arginine biosynthesis; Cobalt; Complete proteome; Hydrolase; Metal-binding; Reference proteome; Zinc
SRR342019_TR38616_c0_g1_i2_len=910	CPC_CUCSA	Q:778-455,H:4-111	48.65%ID	E:2e-24	Cucumber peeling cupredoxin	PF02298.14^Cu_bind_like^Plastocyanin-like domain^35-121^E:6.5e-23	1	-1,495	0,001	3,443	0,241	0,668	0,121	0,87	1	0,298	CPC_CUCSA	Cucumber peeling cupredoxin (CPC) (Stellacyanin)		Cucumis sativus (Cucumber)		3D-structure; Copper; Direct protein sequencing; Disulfide bond; Electron transport; Glycoprotein; Hydroxylation; Metal-binding; Pyrrolidone carboxylic acid; Transport
SRR342019_TR38836_c2_g1_i5_len=2869	DCE_SOLLC	Q:2258-2869,H:1-205	84.88%ID	E:3e-105	Glutamate decarboxylase	PF06507.10^Auxin_resp^Auxin response factor^1-40^E:3e-15	0,002	1,133	0	1,397	0,014	1,044	0,032	0,935	0,001	1,014	DCE_SOLLC	Glutamate decarboxylase (GAD) (EC 4.1.1.15) (ERT D1)		Solanum lycopersicum (Tomato) (Lycopersicon esculentum)		Calmodulin-binding; Complete proteome; Decarboxylase; Lyase; Pyridoxal phosphate; Reference proteome
SRR342022_TR98_c1_g13_i2_len=1686	E1313_ARATH	Q:256-1563,H:30-446	43.28%ID	E:2e-120	Glucan endo-1,3-beta-glucosidase 13	PF00332.15^Glyco_hydro_17^Glycosyl hydrolases family 17^35-351^E:7.2e-99`PF07983.10^X8^X8 domain^395-466^E:7.6e-22	0,77	-0,196	0	9,567	0,502	4,19	1	0	0,547	0,37	E1313_ARATH	Glucan endo-1,3-beta-glucosidase 13 (EC 3.2.1.39) ((1->3)-beta-glucan endohydrolase 13) ((1->3)-beta-glucanase 13) (Beta-1,3-endoglucanase 13) (Beta-1,3-glucanase 13)	At5g56590 MIK19.3	Arabidopsis thaliana (Mouse-ear cress)		Cell membrane; Cell wall; Cell wall biogenesis/degradation; Complete proteome; Disulfide bond; GPI-anchor; Glycoprotein; Glycosidase; Hydrolase; Lipoprotein; Membrane; Plant defense; Reference proteome; Secreted; Signal
SRR342022_TR447_c1_g4_i2_len=1712	CESA2_ARATH	Q:146-1711,H:1-507	73.61%ID	E:0	Cellulose synthase A catalytic subunit 2 [UDP-forming]	PF14569.3^zf-UDP^Zinc-binding RING-finger^30-105^E:2.5e-41`PF03552.11^Cellulose_synt^Cellulose synthase^371-522^E:9.7e-89	0,002	0,851	0	3,06	0,15	-0,418	0,55	-0,175	1	0,842	CESA2_ARATH	Cellulose synthase A catalytic subunit 2 [UDP-forming] (AtCesA2) (Ath-A) (EC 2.4.1.12)	CESA2 ATHA At4g39350 T22F8.250	Arabidopsis thaliana (Mouse-ear cress)	PATHWAY: Glycan metabolism; plant cellulose biosynthesis.	Acetylation; Cell membrane; Cell wall biogenesis/degradation; Cellulose biosynthesis; Coiled coil; Complete proteome; Glycoprotein; Glycosyltransferase; Membrane; Metal-binding; Reference proteome; Transferase; Transmembrane; Transmembrane helix; Zinc; Zinc-finger
SRR342022_TR1355_c0_g2_i1_len=989	DNJ20_ARATH	Q:387-797,H:66-197	57.25%ID	E:2e-44	Chaperone protein dnaJ 20, chloroplastic	PF00226.28^DnaJ^DnaJ domain^80-144^E:3.1e-21	1	0,004	0	2,555	0,461	1,669	0,016	2,809	1	0,009	DNJ20_ARATH	Chaperone protein dnaJ 20, chloroplastic (AtDjC20) (AtJ20)	ATJ20 C20 J20 At4g13830 F18A5.220	Arabidopsis thaliana (Mouse-ear cress)		Alternative splicing; Chaperone; Chloroplast; Complete proteome; Plastid; Reference proteome; Transit peptide
SRR342022_TR1586_c2_g8_i3_len=3492	METK1_CATRO	Q:1836-3014,H:1-393	99.75%ID	E:0	S-adenosylmethionine synthase 1	PF00438.17^S-AdoMet_synt_N^S-adenosylmethionine synthetase, N-terminal domain^4-101^E:8.4e-43`PF02772.13^S-AdoMet_synt_M^S-adenosylmethionine synthetase, central domain^117-238^E:1.7e-46`PF02773.13^S-AdoMet_synt_C^S-adenosylmethionine synthetase, C-terminal domain^240-381^E:2e-62	0,182	0,507	0	1,685	0,306	0,503	0,306	0,517	0,91	-0,029	METK1_CATRO	S-adenosylmethionine synthase 1 (AdoMet synthase 1) (EC 2.5.1.6) (Methionine adenosyltransferase 1) (MAT 1)	SAMS1	Catharanthus roseus (Madagascar periwinkle) (Vinca rosea)	PATHWAY: Amino-acid biosynthesis; S-adenosyl-L-methionine biosynthesis; S-adenosyl-L-methionine from L-methionine: step 1/1.	ATP-binding; Cobalt; Cytoplasm; Magnesium; Manganese; Metal-binding; Nucleotide-binding; One-carbon metabolism; Potassium; Transferase
SRR342022_TR1813_c1_g9_i2_len=1262	NA	NA	NA	NA	NA	.	0,012	0,929	0	1,261	1	0,168	0,593	0,741	0,915	-0,069	.	.	.	.	.	.
SRR342022_TR1898_c4_g2_i3_len=1676	PSAB_SOLTU	Q:263-1585,H:294-734	99.32%ID	E:0	Photosystem I P700 chlorophyll a apoprotein A2 {ECO:0000255|HAMAP-Rule:MF_00482}	PF00223.16^PsaA_PsaB^Photosystem I psaA/psaB protein^1-427^E:1.6e-217	0,159	0,507	0	1,965	0	-2,292	0	-1,49	1	0	PSAB_SOLTU	Photosystem I P700 chlorophyll a apoprotein A2 (EC 1.97.1.12) (PSI-B) (PsaB)	psaB	Solanum tuberosum (Potato)		4Fe-4S; Chlorophyll; Chloroplast; Chromophore; Complete proteome; Electron transport; Iron; Iron-sulfur; Magnesium; Membrane; Metal-binding; Oxidoreductase; Photosynthesis; Photosystem I; Plastid; Reference proteome; Thylakoid; Transmembrane; Transmembrane helix; Transport
SRR342022_TR1981_c1_g1_i1_len=636	UBIQP_AVEFA	Q:1-570,H:53-242	99.47%ID	E:1e-130	Polyubiquitin	PF11976.5^Rad60-SLD^Ubiquitin-2 like Rad60 SUMO-like^25-95^E:4.2e-16`PF00240.20^ubiquitin^Ubiquitin family^27-98^E:6.2e-34`PF13881.3^Rad60-SLD_2^Ubiquitin-2 like Rad60 SUMO-like^34-95^E:0.07`PF14560.3^Ubiquitin_2^Ubiquitin-like domain^36-93^E:0.00028`PF11976.5^Rad60-SLD^Ubiquitin-2 like Rad60 SUMO-like^101-171^E:4.2e-16`PF00240.20^ubiquitin^Ubiquitin family^103-174^E:6.2e-34`PF13881.3^Rad60-SLD_2^Ubiquitin-2 like Rad60 SUMO-like^110-171^E:0.065`PF14560.3^Ubiquitin_2^Ubiquitin-like domain^112-169^E:0.00028	0,823	0,086	0	1,469	0,627	-0,155	0,874	-0,05	0,003	1,143	UBIQP_AVEFA	Polyubiquitin [Cleaved into: Ubiquitin]		Avena fatua (Wild oats)		Cytoplasm; Isopeptide bond; Nucleus; Repeat; Ubl conjugation
SRR342022_TR2467_c1_g7_i4_len=1549	RNLE_SOLLC	Q:762-103,H:11-230	63.8%ID	E:3e-99	Extracellular ribonuclease LE	PF00445.15^Ribonuclease_T2^Ribonuclease T2 family^28-202^E:1.1e-59	0,066	3,76	0	8,179	0,043	3,707	0,381	2,642	1	0	RNLE_SOLLC	Extracellular ribonuclease LE (RNase LE) (EC 3.1.27.1)		Solanum lycopersicum (Tomato) (Lycopersicon esculentum)		3D-structure; Cell wall; Complete proteome; Direct protein sequencing; Disulfide bond; Endonuclease; Hydrolase; Nuclease; Reference proteome; Secreted; Signal; Stress response
SRR342022_TR3657_c1_g2_i2_len=709	RAE1C_ARATH	Q:135-668,H:15-192	95.51%ID	E:1e-103	Ras-related protein RABE1c	PF00025.18^Arf^ADP-ribosylation factor family^16-163^E:1.7e-15`PF00071.19^Ras^Ras family^17-178^E:1.1e-66`PF08477.10^Roc^Ras of Complex, Roc, domain of DAPkinase^17-132^E:3.4e-35`PF04670.9^Gtr1_RagA^Gtr1/RagA G protein conserved region^17-132^E:3.4e-05`PF01926.20^MMR_HSR1^50S ribosome-binding GTPase^18-128^E:3.6e-05	0,87	-0,081	0	1,624	0,312	0,318	0,244	0,353	0,651	0,124	RAE1C_ARATH	Ras-related protein RABE1c (AtRABE1c) (Ras-related protein Ara-3) (Ras-related protein Rab8A) (AtRab8A)	RABE1C ARA-3 RAB8A At3g46060 F12M12.30	Arabidopsis thaliana (Mouse-ear cress)		Cell membrane; Complete proteome; GTP-binding; Golgi apparatus; Lipoprotein; Membrane; Nucleotide-binding; Prenylation; Protein transport; Reference proteome; Transport
SRR342022_TR4468_c0_g1_i1_len=1311	NA	NA	NA	NA	NA	PF07223.8^DUF1421^Protein of unknown function (DUF1421)^242-435^E:5.1e-35	0,826	0,11	0,001	1,751	0,24	0,508	0,204	0,542	0,484	0,435	.	.	.	.	.	.
SRR342022_TR4652_c0_g1_i2_len=1303	MER_ERYCB	Q:84-1052,H:1-327	56.67%ID	E:7e-122	Methylecgonone reductase	PF00248.18^Aldo_ket_red^Aldo/keto reductase family^35-294^E:2.1e-49	0,199	0,328	0	1,573	0,353	0,258	0,454	-0,226	0,038	0,522	MER_ERYCB	Methylecgonone reductase (MecgoR) (EC 1.1.1.334)		Erythroxylum coca (Coca plant)		Alkaloid metabolism; NADP; Oxidoreductase
SRR342022_TR5089_c0_g3_i4_len=514	NA	NA	NA	NA	NA	PF00582.23^Usp^Universal stress protein family^50-170^E:7.4e-20	0,484	0,385	0	2,389	0,004	0,961	0,268	0,409	0,526	-0,309	.	.	.	.	.	.
SRR342022_TR5735_c0_g2_i2_len=1591	CALX_SOYBN	Q:1322-81,H:20-438	80.67%ID	E:0	Calnexin homolog	PF00262.15^Calreticulin^Calreticulin family^34-389^E:1.9e-147	0,083	-0,476	0	2,741	0,898	-0,064	0,898	-0,064	0,115	0,463	CALX_SOYBN	Calnexin homolog		Glycine max (Soybean) (Glycine hispida)		Calcium; Chaperone; Complete proteome; Disulfide bond; Endoplasmic reticulum; Glycoprotein; Lectin; Membrane; Metal-binding; Reference proteome; Repeat; Signal; Transmembrane; Transmembrane helix
SRR342022_TR6753_c0_g1_i3_len=749	DV4H_CATRO	Q:743-264,H:240-401	51.23%ID	E:1e-52	Deacetoxyvindoline 4-hydroxylase	PF03171.17^2OG-FeII_Oxy^2OG-Fe(II) oxygenase superfamily^14-106^E:8e-24	0,009	3,938	0	7,841	1	2,306	1	3,032	1	-1,656	DV4H_CATRO	Deacetoxyvindoline 4-hydroxylase (EC 1.14.11.20)	D4H	Catharanthus roseus (Madagascar periwinkle) (Vinca rosea)	PATHWAY: Alkaloid biosynthesis; vindoline biosynthesis; vindoline from tabersonine: step 5/6.	Cytoplasm; Dioxygenase; Direct protein sequencing; Iron; Metal-binding; Oxidoreductase; Vitamin C
SRR342022_TR6797_c3_g1_i1_len=2919	RFS2_ARATH	Q:2692-368,H:1-773	77.96%ID	E:0	Probable galactinol–sucrose galactosyltransferase 2	PF05691.9^Raffinose_syn^Raffinose synthase or seed imbibition protein Sip1^7-759^E:0	0,283	0,297	0	3,336	0,015	0,526	0,356	0,203	0,017	5,778	RFS2_ARATH	Probable galactinol–sucrose galactosyltransferase 2 (EC 2.4.1.82) (Protein SEED IMBIBITION 2) (Raffinose synthase 2)	RFS2 RS2 SIP2 At3g57520 T8H10.120	Arabidopsis thaliana (Mouse-ear cress)		Alternative splicing; Carbohydrate metabolism; Complete proteome; Glycosyltransferase; Reference proteome; Transferase
SRR342022_TR8568_c0_g1_i2_len=1934	NA	NA	NA	NA	NA	PF00403.23^HMA^Heavy-metal-associated domain^13-70^E:1.1e-15	0,237	0,451	0	1,482	0,011	1,55	0,011	1,526	0,016	-1,975	.	.	.	.	.	.
SRR342022_TR8639_c2_g3_i2_len=2205	BIP5_TOBAC	Q:139-2076,H:1-646	96.28%ID	E:0	Luminal-binding protein 5	PF00012.17^HSP70^Hsp70 protein^38-645^E:5e-269`PF06723.10^MreB_Mbl^MreB/Mbl protein^153-405^E:4.7e-16	0,316	-0,333	0	1,524	0,807	0,189	0,255	-0,611	0,066	1,739	BIP5_TOBAC	Luminal-binding protein 5 (BiP 5) (78 kDa glucose-regulated protein homolog 5) (GRP-78-5)	BIP5	Nicotiana tabacum (Common tobacco)		ATP-binding; Endoplasmic reticulum; Glycoprotein; Nucleotide-binding; Signal
SRR342022_TR8939_c1_g2_i3_len=1921	C76BA_SWEMU	Q:180-1514,H:45-491	52.13%ID	E:3e-170	Geraniol 8-hydroxylase	PF00067.19^p450^Cytochrome P450^48-492^E:9.6e-109	0,051	1,149	0	3,666	0,163	-0,409	0,082	-0,528	1	0,028	C76BA_SWEMU	Geraniol 8-hydroxylase (EC 1.14.13.152) (Cytochrome P450 76B10) (Geraniol 10-hydroxylase) (SmG10H)	CYP76B10 G10H	Swertia mussotii (Felwort)		Endoplasmic reticulum; Heme; Iron; Membrane; Metal-binding; Monooxygenase; NADP; Oxidoreductase; Transmembrane; Transmembrane helix
SRR342022_TR9396_c5_g12_i5_len=680	GEDH1_OCIBA	Q:566-12,H:3-186	58.38%ID	E:4e-61	Geraniol dehydrogenase 1	PF08240.9^ADH_N^Alcohol dehydrogenase GroES-like domain^40-152^E:2.2e-24	0,753	-0,1	0	2,795	0,123	-0,705	0,766	-0,151	0,832	0,186	GEDH1_OCIBA	Geraniol dehydrogenase 1 (ObaGEDH1) (EC 1.1.1.183)	GEDH1	Ocimum basilicum (Sweet basil)		Metal-binding; NADP; Oxidoreductase; Zinc
SRR342022_TR9505_c0_g2_i1_len=3064	PDAT1_ARATH	Q:2428-542,H:47-671	80.45%ID	E:0	Phospholipid:diacylglycerol acyltransferase 1	PF02450.12^LCAT^Lecithin:cholesterol acyltransferase^129-370^E:5.7e-45`PF02450.12^LCAT^Lecithin:cholesterol acyltransferase^473-630^E:9.9e-19	1	-0,06	0	2,515	0,026	0,93	0,048	0,857	0,878	0,158	PDAT1_ARATH	Phospholipid:diacylglycerol acyltransferase 1 (AtPDAT) (EC 2.3.1.158)	PDAT1 PDAT At5g13640 MSH12.10 T6I14.2	Arabidopsis thaliana (Mouse-ear cress)	PATHWAY: Glycerolipid metabolism; triacylglycerol biosynthesis.	Acyltransferase; Complete proteome; Glycerol metabolism; Glycoprotein; Lipid biosynthesis; Lipid metabolism; Membrane; Reference proteome; Transferase; Transmembrane; Transmembrane helix
SRR342022_TR10275_c3_g3_i4_len=3102	NUCL2_ORYSJ	Q:1953-2543,H:435-630	56.28%ID	E:7e-62	Nucleolin 2	PF00076.19^RRM_1^RNA recognition motif. (a.k.a. RRM, RBD, or RNP domain)^412-482^E:3.8e-19`PF00076.19^RRM_1^RNA recognition motif. (a.k.a. RRM, RBD, or RNP domain)^515-585^E:9.9e-08	0,001	-1,074	0	1,678	0,381	2,056	1	1,035	0,619	0,134	NUCL2_ORYSJ	Nucleolin 2 (Protein NUCLEOLIN LIKE 2)	Os04g0620700 LOC_Os04g52960 OSJNBa0058K23.21	Oryza sativa subsp. japonica (Rice)		Complete proteome; Nucleus; RNA-binding; Reference proteome; Repeat; rRNA processing
SRR342022_TR11768_c3_g4_i2_len=4069	CESA1_ARATH	Q:3800-492,H:1-1066	74.55%ID	E:0	Cellulose synthase A catalytic subunit 1 [UDP-forming]	PF14569.3^zf-UDP^Zinc-binding RING-finger^29-106^E:2.1e-44`PF03552.11^Cellulose_synt^Cellulose synthase^355-1071^E:0`PF13632.3^Glyco_trans_2_3^Glycosyl transferase family group 2^773-880^E:1e-06	0,007	0,759	0	1,565	0,885	0,057	1	-0,001	1	-0,15	CESA1_ARATH	Cellulose synthase A catalytic subunit 1 [UDP-forming] (AtCesA1) (EC 2.4.1.12) (Protein RADIALLY SWOLLEN 1) (AtRSW1)	CESA1 RSW1 At4g32410 F8B4.110	Arabidopsis thaliana (Mouse-ear cress)	PATHWAY: Glycan metabolism; plant cellulose biosynthesis.	Acetylation; Cell membrane; Cell wall biogenesis/degradation; Cellulose biosynthesis; Coiled coil; Complete proteome; Glycoprotein; Glycosyltransferase; Membrane; Metal-binding; Reference proteome; Transferase; Transmembrane; Transmembrane helix; Zinc; Zinc-finger
SRR342022_TR13523_c2_g2_i1_len=1898	NA	NA	NA	NA	NA	.	0,43	0,579	0	2,001	0,073	-0,694	0,256	-0,418	1	0,021	.	.	.	.	.	.
SRR342022_TR13754_c0_g1_i1_len=921	RAE1C_ARATH	Q:232-837,H:15-216	93.07%ID	E:6e-119	Ras-related protein RABE1c	PF00025.18^Arf^ADP-ribosylation factor family^16-163^E:2.1e-15`PF00071.19^Ras^Ras family^17-178^E:1.6e-66`PF08477.10^Roc^Ras of Complex, Roc, domain of DAPkinase^17-132^E:4.6e-35`PF04670.9^Gtr1_RagA^Gtr1/RagA G protein conserved region^17-132^E:4.5e-05`PF01926.20^MMR_HSR1^50S ribosome-binding GTPase^18-128^E:4.7e-05	0,854	0,13	0	6,797	0	-2,668	0	-7,493	0,007	1,084	RAE1C_ARATH	Ras-related protein RABE1c (AtRABE1c) (Ras-related protein Ara-3) (Ras-related protein Rab8A) (AtRab8A)	RABE1C ARA-3 RAB8A At3g46060 F12M12.30	Arabidopsis thaliana (Mouse-ear cress)		Cell membrane; Complete proteome; GTP-binding; Golgi apparatus; Lipoprotein; Membrane; Nucleotide-binding; Prenylation; Protein transport; Reference proteome; Transport
SRR342022_TR13973_c1_g9_i2_len=1403	MTDH_FRAAN	Q:1307-267,H:10-356	57.59%ID	E:4e-116	Probable mannitol dehydrogenase	PF08240.9^ADH_N^Alcohol dehydrogenase GroES-like domain^59-172^E:5.8e-26`PF00107.23^ADH_zinc_N^Zinc-binding dehydrogenase^216-336^E:3.7e-12	0,019	0,761	0	5,802	0,158	1,633	0,254	-2,483	0,298	0,47	MTDH_FRAAN	Probable mannitol dehydrogenase (EC 1.1.1.255) (NAD-dependent mannitol dehydrogenase)	CAD	Fragaria ananassa (Strawberry)		Metal-binding; NAD; Oxidoreductase; Zinc
SRR342022_TR15185_c0_g1_i1_len=678	NA	NA	NA	NA	NA	.	0,009	4,371	0	8,406	0,502	2,278	0,254	2,513	0,282	-1,214	.	.	.	.	.	.
SRR342022_TR15284_c2_g2_i1_len=2271	C72A1_CATRO	Q:259-1821,H:1-522	54.58%ID	E:0	Secologanin synthase	PF00067.19^p450^Cytochrome P450^91-497^E:1.2e-74	0,001	0,86	0	2,365	0,009	0,732	0,193	0,388	0,029	0,618	C72A1_CATRO	Secologanin synthase (SLS) (EC 1.3.3.9) (CYPLXXII) (Cytochrome P450 72A1)	CYP72A1 CYP72 P450CR3	Catharanthus roseus (Madagascar periwinkle) (Vinca rosea)	PATHWAY: Alkaloid biosynthesis; secologanin biosynthesis.	Alkaloid metabolism; Heme; Iron; Membrane; Metal-binding; Monooxygenase; NADP; Oxidoreductase; Transmembrane; Transmembrane helix; Vacuole
SRR342022_TR15834_c0_g2_i1_len=819	SUI1_MAIZE	Q:645-310,H:1-115	79.13%ID	E:8e-57	Protein translation factor SUI1 homolog	PF01253.19^SUI1^Translation initiation factor SUI1^77-151^E:8.1e-25	0,716	0,112	0	1,078	0,001	0,737	0,001	0,749	0,679	-0,39	SUI1_MAIZE	Protein translation factor SUI1 homolog (Protein GOS2)	TIF	Zea mays (Maize)		Complete proteome; Protein biosynthesis; Reference proteome; Translation regulation
SRR342022_TR16350_c5_g1_i4_len=2640	PIF4_ARATH	Q:571-2028,H:6-413	33.27%ID	E:3e-45	Transcription factor PIF4	PF00010.23^HLH^Helix-loop-helix DNA-binding domain^326-372^E:2e-13	0,017	0,796	0	1,733	0,201	0,511	0,054	0,731	0,679	0,507	PIF4_ARATH	Transcription factor PIF4 (Basic helix-loop-helix protein 9) (AtbHLH9) (bHLH 9) (Phytochrome-interacting factor 4) (Short under red-light 2) (Transcription factor EN 102) (bHLH transcription factor bHLH009)	PIF4 BHLH9 EN102 SRL2 At2g43010 MFL8.13	Arabidopsis thaliana (Mouse-ear cress)		Alternative splicing; Complete proteome; DNA-binding; Nucleus; Phytochrome signaling pathway; Reference proteome; Transcription; Transcription regulation
SRR342022_TR16831_c0_g2_i1_len=604	NA	NA	NA	NA	NA	.	0,002	4,406	0	5,62	0,189	2,078	1	0,406	1	0	.	.	.	.	.	.
SRR342022_TR19090_c3_g1_i6_len=1784	CATB_PONAB	Q:1465-563,H:26-334	46.58%ID	E:8e-74	Cathepsin B	PF08127.10^Propeptide_C1^Peptidase family C1 propeptide^42-82^E:5.2e-10`PF00112.20^Peptidase_C1^Papain family cysteine protease^102-336^E:2.7e-64	0,729	-0,17	0	5,583	0,502	4,149	1	2,795	1	1,065	CATB_PONAB	Cathepsin B (EC 3.4.22.1) [Cleaved into: Cathepsin B light chain; Cathepsin B heavy chain]	CTSB	Pongo abelii (Sumatran orangutan) (Pongo pygmaeus abelii)		Acetylation; Complete proteome; Disulfide bond; Glycoprotein; Hydrolase; Lysosome; Protease; Reference proteome; Secreted; Signal; Thiol protease; Zymogen
SRR342022_TR19350_c0_g1_i2_len=860	10HGO_CATRO	Q:63-860,H:1-264	84.59%ID	E:2e-132	8-hydroxygeraniol dehydrogenase	PF08240.9^ADH_N^Alcohol dehydrogenase GroES-like domain^39-153^E:4.8e-26`PF02826.16^2-Hacid_dh_C^D-isomer specific 2-hydroxyacid dehydrogenase, NAD binding domain^179-228^E:4.2e-06`PF01262.18^AlaDh_PNT_C^Alanine dehydrogenase/PNT, C-terminal domain^187-258^E:8e-06`PF00107.23^ADH_zinc_N^Zinc-binding dehydrogenase^196-	0,226	2,346	0	3,789	0,226	2,62	0,381	2,414	1	0,164	10HGO_CATRO	8-hydroxygeraniol dehydrogenase (Cr10HGO) (EC 1.1.1.324)	10HGO	Catharanthus roseus (Madagascar periwinkle) (Vinca rosea)		Metal-binding; NADP; Oxidoreductase; Zinc

						258^E:1.7e-08																
SRR342022_TR20398_c2_g1_i3_len=619	NA	NA	NA	NA	NA	.	0,187	1,108	0	3,01	0,034	1,063	0,142	0,793	1	0,057	.	.	.	.	.	.
SRR342022_TR20837_c4_g8_i1_len=644	SCE1_ARATH	Q:98-577,H:1-160	84.38%ID	E:6e-85	SUMO-conjugating enzyme SCE1	PF00179.23^UQ_con^Ubiquitin-conjugating enzyme^9-151^E:4.9e-44	0,821	0,079	0	1,649	0,001	1,026	0,031	0,716	0,07	0,583	SCE1_ARATH	SUMO-conjugating enzyme SCE1 (EC 6.3.2.-) (Protein EMBRYO DEFECTIVE 1637) (Protein hus5 homolog) (SUMO-conjugating enzyme 1) (AtSCE1)	SCE1 AHUS5 EMB1637 At3g57870 T10K17.80	Arabidopsis thaliana (Mouse-ear cress)	PATHWAY: Protein modification; protein sumoylation.	ATP-binding; Acetylation; Complete proteome; Ligase; Nucleotide-binding; Reference proteome; Ubl conjugation pathway
SRR342022_TR20959_c3_g3_i5_len=1508	SDC1_ARATH	Q:1318-182,H:77-453	60.16%ID	E:2e-170	Serine decarboxylase	PF00282.16^Pyridoxal_deC^Pyridoxal-dependent decarboxylase conserved domain^48-302^E:1.5e-23	0,052	1,5	0,001	6,607	0,007	2,193	0,025	1,956	0,112	1,113	SDC1_ARATH	Serine decarboxylase (AtSDC) (EC 4.1.1.-) (Protein EMBRYO DEFECTIVE 1075) (Serine decarboxylase 1) (AtSDC1)	SDC EMB1075 SDC1 At1g43710 F2J6.7	Arabidopsis thaliana (Mouse-ear cress)		Complete proteome; Cytoplasm; Decarboxylase; Lyase; Pyridoxal phosphate; Reference proteome
SRR342022_TR21029_c2_g2_i2_len=3334	MACP2_ARATH	Q:1199-2902,H:15-598	66.1%ID	E:0	MACPF domain-containing protein At4g24290	PF01823.16^MACPF^MAC/Perforin domain^110-311^E:2.1e-36	0,319	0,882	0	2,337	0,042	1,775	0,158	1,408	0,732	-0,853	MACP2_ARATH	MACPF domain-containing protein At4g24290	At4g24290 T22A6.120	Arabidopsis thaliana (Mouse-ear cress)		Alternative splicing; Complete proteome; Hypersensitive response; Immunity; Innate immunity; Plant defense; Reference proteome
SRR342022_TR21370_c4_g3_i1_len=2810	SPE1_PEA	Q:2286-94,H:1-727	70.79%ID	E:0	Arginine decarboxylase	PF02784.13^Orn_Arg_deC_N^Pyridoxal-dependent decarboxylase, pyridoxal binding domain^140-410^E:2.1e-40`PF00278.19^Orn_DAP_Arg_deC^Pyridoxal-dependent decarboxylase, C-terminal sheet domain^498-612^E:7.4e-19	0,723	0,123	0	3,17	0,664	0,122	0,017	0,614	1	0,176	SPE1_PEA	Arginine decarboxylase (ADC) (ARGDC) (EC 4.1.1.19)		Pisum sativum (Garden pea)	PATHWAY: Amine and polyamine biosynthesis; agmatine biosynthesis; agmatine from L-arginine: step 1/1.	Decarboxylase; Lyase; Magnesium; Putrescine biosynthesis; Pyridoxal phosphate; Spermidine biosynthesis
SRR342022_TR21436_c1_g5_i1_len=1437	VPS2A_ARATH	Q:429-1097,H:2-225	91.07%ID	E:3e-108	Vacuolar protein sorting-associated protein 2 homolog 1	PF03357.18^Snf7^Snf7^17-186^E:6.1e-45	0,419	0,368	0	1,701	0,017	1,252	0,012	1,278	1	1,2	VPS2A_ARATH	Vacuolar protein sorting-associated protein 2 homolog 1 (AtVPS2-1) (Charged multivesicular body protein 2 homolog 1) (ESCRT-III complex subunit VPS2 homolog 1) (SNF7-like protein)	VPS2.1 CHMP2-1 SLP At2g06530 T12H3.8	Arabidopsis thaliana (Mouse-ear cress)		Coiled coil; Complete proteome; Endosome; Protein transport; Reference proteome; Transport
SRR342022_TR22371_c0_g2_i4_len=796	NA	NA	NA	NA	NA	.	0,004	1,902	0	2,852	0,487	-0,323	0,541	0,244	1	-1,24	.	.	.	.	.	.
SRR342022_TR24124_c0_g1_i5_len=1744	10HGO_CATRO	Q:1703-786,H:11-303	57.52%ID	E:5e-95	8-hydroxygeraniol dehydrogenase	PF08240.9^ADH_N^Alcohol dehydrogenase GroES-like domain^41-164^E:2.7e-23`PF02826.16^2-Hacid_dh_C^D-isomer specific 2-hydroxyacid dehydrogenase, NAD binding domain^195-244^E:3.2e-07`PF00107.23^ADH_zinc_N^Zinc-binding dehydrogenase^211-318^E:2.7e-12	0,034	0,489	0	1,198	0,016	0,659	0,787	0,096	0,007	0,639	10HGO_CATRO	8-hydroxygeraniol dehydrogenase (Cr10HGO) (EC 1.1.1.324)	10HGO	Catharanthus roseus (Madagascar periwinkle) (Vinca rosea)		Metal-binding; NADP; Oxidoreductase; Zinc
SRR342022_TR26056_c0_g2_i2_len=710	RL192_ARATH	Q:67-648,H:1-196	86.22%ID	E:7e-90	60S ribosomal protein L19-2	PF01280.17^Ribosomal_L19e^Ribosomal protein L19e^4-146^E:2.6e-68	0,009	-0,836	0	1,559	0,102	0,565	0,684	-0,155	0,13	0,493	RL192_ARATH	60S ribosomal protein L19-2	RPL19B At3g16780 MGL6.25	Arabidopsis thaliana (Mouse-ear cress)		Complete proteome; Reference proteome; Ribonucleoprotein; Ribosomal protein
SRR342022_TR26073_c3_g1_i13_len=567	NA	NA	NA	NA	NA	PF00226.28^DnaJ^DnaJ domain^12-77^E:1e-24	0	-1,521	0	1,794	1	-1,477	1	-1,477	1	1,187	.	.	.	.	.	.
SRR342022_TR27588_c4_g1_i1_len=2560	PPI1_ARATH	Q:305-1459,H:25-412	56.01%ID	E:6e-133	Proton pump-interactor 1	.	0,903	-0,051	0	1,611	0,61	0,186	0,654	0,17	0,674	0,126	PPI1_ARATH	Proton pump-interactor 1	PPI1 At4g27500 F27G19.100	Arabidopsis thaliana (Mouse-ear cress)		Cell membrane; Coiled coil; Complete proteome; Endoplasmic reticulum; Membrane; Phosphoprotein; Reference proteome; Transmembrane; Transmembrane helix
SRR342022_TR28482_c0_g2_i1_len=540	VATL_GOSHI	Q:422-39,H:1-128	96.88%ID	E:3e-43	V-type proton ATPase 16 kDa proteolipid subunit	PF00137.18^ATP-synt_C^ATP synthase subunit C^17-76^E:1.3e-13`PF00137.18^ATP-synt_C^ATP synthase subunit C^97-128^E:6.3e-08	0,448	-0,183	0	0,922	0,918	0,03	0,65	0,113	0,76	0,336	VATL_GOSHI	V-type proton ATPase 16 kDa proteolipid subunit (V-ATPase 16 kDa proteolipid subunit) (Vacuolar proton pump 16 kDa proteolipid subunit)	CVA16-2; CVA16-4	Gossypium hirsutum (Upland cotton) (Gossypium mexicanum)		Hydrogen ion transport; Ion transport; Membrane; Transmembrane; Transmembrane helix; Transport; Vacuole
SRR342022_TR28725_c0_g1_i2_len=1839	BEBT_TOBAC	Q:121-1452,H:3-445	77.25%ID	E:0	Benzyl alcohol O-benzoyltransferase	PF02458.12^Transferase^Transferase family^14-440^E:4.9e-81	0,402	1,223	0	2,705	0,003	1,335	0,002	1,372	0,094	1,43	BEBT_TOBAC	Benzyl alcohol O-benzoyltransferase (EC 2.3.1.196) (Benzoyl coenzyme A:benzyl alcohol benzoyl transferase)	HSR201	Nicotiana tabacum (Common tobacco)		Acyltransferase; Transferase
SRR342022_TR28792_c1_g1_i3_len=1799	PTR21_ARATH	Q:551-1429,H:322-617	52.03%ID	E:3e-106	Protein NRT1/ PTR FAMILY 2.13	PF00854.18^PTR2^POT family^1-161^E:1.1e-29	0,002	1,654	0	2,736	0,002	1,435	0,007	1,298	0,335	0,624	PTR21_ARATH	Protein NRT1/ PTR FAMILY 2.13 (AtNPF2.13) (Nitrate transporter 1.7)	NPF2.13 NRT1.7 At1g69870 T17F3.10	Arabidopsis thaliana (Mouse-ear cress)		Cell membrane; Complete proteome; Membrane; Nitrate assimilation; Reference proteome; Symport; Transmembrane; Transmembrane helix; Transport
SRR342022_TR28792_c1_g1_i7_len=3186	PTR21_ARATH	Q:1368-2816,H:136-617	53.91%ID	E:4e-177	Protein NRT1/ PTR FAMILY 2.13	PF00854.18^PTR2^POT family^22-440^E:4.2e-80	1	1,325	0	4,253	0,038	1,708	0,282	1,147	0,319	1,09	PTR21_ARATH	Protein NRT1/ PTR FAMILY 2.13 (AtNPF2.13) (Nitrate transporter 1.7)	NPF2.13 NRT1.7 At1g69870 T17F3.10	Arabidopsis thaliana (Mouse-ear cress)		Cell membrane; Complete proteome; Membrane; Nitrate assimilation; Reference proteome; Symport; Transmembrane; Transmembrane helix; Transport
SRR342022_TR29142_c3_g2_i2_len=2476	ATHB6_ARATH	Q:1134-1610,H:34-204	60.23%ID	E:3e-50	Homeobox-leucine zipper protein ATHB-6	PF00046.26^Homeobox^Homeobox domain^20-73^E:2.7e-16`PF02183.15^HALZ^Homeobox associated leucine zipper^75-117^E:1.2e-18	0,111	0,382	0	1,502	0,019	0,679	0,747	0,12	0,167	0,396	ATHB6_ARATH	Homeobox-leucine zipper protein ATHB-6 (HD-ZIP protein ATHB-6) (Homeodomain transcription factor ATHB-6)	ATHB-6 At2g22430 F14M13.17	Arabidopsis thaliana (Mouse-ear cress)		Abscisic acid signaling pathway; Complete proteome; DNA-binding; Homeobox; Nucleus; Reference proteome; Stress response; Transcription; Transcription regulation
SRR342022_TR29250_c1_g2_i1_len=3164	CIPK9_ARATH	Q:247-1551,H:8-443	78.44%ID	E:0	CBL-interacting serine/threonine-protein kinase 9	PF00069.22^Pkinase^Protein kinase domain^15-270^E:4.2e-73`PF07714.14^Pkinase_Tyr^Protein tyrosine kinase^16-266^E:3.4e-38`PF14531.3^Kinase-like^Kinase-like^110-220^E:2.3e-07`PF03822.11^NAF^NAF domain^309-369^E:2.1e-24	0,694	-0,427	0	2,542	1	-0,056	0,182	0,519	1	-0,555	CIPK9_ARATH	CBL-interacting serine/threonine-protein kinase 9 (EC 2.7.11.1) (SNF1-related kinase 3.12) (SOS2-like protein kinase PKS6)	CIPK9 PKS6 SnRK3.12 At1g01140 F6F3.28 T25K16.13	Arabidopsis thaliana (Mouse-ear cress)		ATP-binding; Alternative splicing; Complete proteome; Cytoplasm; Kinase; Manganese; Nucleotide-binding; Nucleus; Phosphoprotein; Reference proteome; Serine/threonine-protein kinase; Transferase
SRR342022_TR30274_c0_g1_i1_len=557	NA	NA	NA	NA	NA	.	0,517	0,867	0	7,671	0,066	5,342	1	0	0,407	0,482	.	.	.	.	.	.
SRR342022_TR30555_c0_g1_i1_len=2499	AROA_PETHY	Q:1858-311,H:6-516	84.3%ID	E:0	3-phosphoshikimate 1-carboxyvinyltransferase, chloroplastic	PF00275.17^EPSP_synthase^EPSP synthase (3-phosphoshikimate 1-carboxyvinyltransferase)^79-509^E:1.7e-149	0,911	0,049	0	1,187	0,28	0,295	0,098	0,444	1	0,789	AROA_PETHY	3-phosphoshikimate 1-carboxyvinyltransferase, chloroplastic (EC 2.5.1.19) (5-enolpyruvylshikimate-3-phosphate synthase) (EPSP synthase)		Petunia hybrida (Petunia)	PATHWAY: Metabolic intermediate biosynthesis; chorismate biosynthesis; chorismate from D-erythrose 4-phosphate and phosphoenolpyruvate: step 6/7.	Amino-acid biosynthesis; Aromatic amino acid biosynthesis; Chloroplast; Herbicide resistance; Plastid; Transferase; Transit peptide
SRR342022_TR30829_c0_g2_i1_len=1950	THCAS_CANSA	Q:272-1762,H:32-540	43.95%ID	E:3e-133	Tetrahydrocannabinolic acid synthase	PF01565.20^FAD_binding_4^FAD binding domain^74-210^E:5.6e-23`PF08031.9^BBE^Berberine and berberine like^486-521^E:1.1e-13	0,472	0,32	0	3,208	0,277	0,579	0,777	-0,176	0,203	0,555	THCAS_CANSA	Tetrahydrocannabinolic acid synthase (THCAS) (EC 1.21.3.7) (Delta(1)-tetrahydrocannabinolic acid synthase) (THCA synthase)		Cannabis sativa (Hemp) (Marijuana)		3D-structure; Apoplast; Direct protein sequencing; Disulfide bond; FAD; Flavoprotein; Glycoprotein; Oxidoreductase; Secreted; Signal
SRR342022_TR33517_c0_g1_i1_len=542	NA	NA	NA	NA	NA	.	0,593	0,794	0	3,37	0,026	1,983	0,004	2,392	1	0,365	.	.	.	.	.	.
SRR342022_TR34117_c0_g3_i2_len=900	TRXH_RICCO	Q:798-466,H:3-113	83.78%ID	E:4e-63	Thioredoxin H-type	PF00085.17^Thioredoxin^Thioredoxin^42-144^E:3.8e-33	1	0,777	0	6,041	0,502	4,048	0,502	3,763	1	0	TRXH_RICCO	Thioredoxin H-type (Trx-H)		Ricinus communis (Castor bean)		Cytoplasm; Disulfide bond; Electron transport; Redox-active center; Transport
SRR342022_TR34371_c0_g3_i1_len=691	NA	NA	NA	NA	NA	.	0,058	0,986	0,001	1,547	0,003	1,448	0,365	0,529	0,129	3,921	.	.	.	.	.	.
SRR342022_TR34629_c0_g1_i1_len=857	NA	NA	NA	NA	NA	PF01190.14^Pollen_Ole_e_I^Pollen proteins Ole e I like^35-122^E:5.4e-10	0,184	0,474	0	1,221	0,008	0,904	0,067	0,641	0,597	0,208	.	.	.	.	.	.
SRR342022_TR34968_c0_g1_i1_len=731	NA	NA	NA	NA	NA	.	0,56	0,874	0	2,774	0,008	1,64	0,663	0,521	1	-0,114	.	.	.	.	.	.
SRR342023_TR361_c0_g1_i1_len=1290	NA	NA	NA	NA	NA	.	0,653	0,198	0,001	1,203	0,03	0,748	0,008	0,896	0,266	-0,519	.	.	.	.	.	.
SRR342023_TR417_c0_g2_i5_len=2137	NA	NA	NA	NA	NA	PF06101.8^DUF946^Plant protein of unknown function (DUF946)^13-551^E:1.2e-243	0,201	0,493	0	1,333	0,258	1,123	0,472	0,853	1	-0,094	.	.	.	.	.	.
SRR342023_TR823_c0_g2_i1_len=1317	TAF7_ARATH	Q:310-753,H:1-148	75%ID	E:4e-66	Transcription initiation factor TFIID subunit 7	PF04658.10^TAFII55_N^TAFII55 protein conserved region^1-139^E:1.5e-33	1	-0,001	0,001	6,63	1	-0,102	1	0,326	0,904	0,099	TAF7_ARATH	Transcription initiation factor TFIID subunit 7 (TBP-associated factor 7) (AtTAF7)	TAF7 At1g55300 F7A10.3	Arabidopsis thaliana (Mouse-ear cress)		Activator; Alternative splicing; Complete proteome; Nucleus; Reference proteome; Transcription; Transcription regulation
SRR342023_TR1754_c1_g5_i2_len=986	AB19I_ARATH	Q:886-47,H:13-290	77.5%ID	E:4e-147	ABC transporter I family member 19	PF00005.24^ABC_tran^ABC transporter^27-170^E:2.3e-13	0,712	-0,248	0	2,158	0,889	-0,118	0,793	0,187	0,017	5,848	AB19I_ARATH	ABC transporter I family member 19 (ABC transporter ABCI.19) (AtABCI19) (GCN-related protein 2) (Non-intrinsic ABC protein 4)	ABCI19 NAP4 At1g03905 F21M11.17	Arabidopsis thaliana (Mouse-ear cress)		ATP-binding; Complete proteome; Cytoplasm; Nucleotide-binding; Reference proteome; Transport
SRR342023_TR2711_c0_g2_i1_len=2040	AOC4_ARATH	Q:1611-2039,H:111-254	72.22%ID	E:3e-60	Allene oxide cyclase 4, chloroplastic	PF12049.5^DUF3531^Protein of unknown function (DUF3531)^1-141^E:1.1e-55	0,004	1,393	0	3,161	0,01	1,758	0,035	1,528	0,212	-1,05	AOC4_ARATH	Allene oxide cyclase 4, chloroplastic (EC 5.3.99.6)	AOC4 At1g13280 T6J4.4 T6J4_23	Arabidopsis thaliana (Mouse-ear cress)		Chloroplast; Complete proteome; Isomerase; Plastid; Reference proteome; Transit peptide
SRR342023_TR3089_c0_g1_i1_len=783	STAD_OLEEU	Q:781-332,H:241-390	85.33%ID	E:1e-91	Stearoyl-[acyl-carrier-protein] 9-desaturase, chloroplastic	PF03405.11^FA_desaturase_2^Fatty acid desaturase^1-144^E:3.4e-62	1	0,014	0	1,421	0,156	0,6	0,915	0,09	1	1,141	STAD_OLEEU	Stearoyl-[acyl-carrier-protein] 9-desaturase, chloroplastic (Stearoyl-ACP desaturase) (EC 1.14.19.2) (Acyl-[acyl-carrier-protein] desaturase)		Olea europaea (Common olive)	PATHWAY: Lipid metabolism; fatty acid metabolism.	Chloroplast; Fatty acid biosynthesis; Fatty acid metabolism; Iron; Lipid biosynthesis; Lipid metabolism; Metal-binding; Oxidoreductase; Plastid; Transit peptide
SRR342023_TR3168_c2_g1_i1_len=1254	LOR12_ARATH	Q:883-281,H:19-220	64.85%ID	E:8e-88	Protein LURP-one-related 12	PF04525.9^LOR^LURP-one-related^6-183^E:5.5e-53	0,074	0,922	0	3,363	0,005	0,77	0,042	0,56	0,175	0,537	LOR12_ARATH	Protein LURP-one-related 12	At3g15810 MSJ11.21	Arabidopsis thaliana (Mouse-ear cress)		Complete proteome; Reference proteome
SRR342023_TR3544_c0_g2_i2_len=563	DCE_PETHY	Q:50-562,H:277-447	87.13%ID	E:8e-108	Glutamate decarboxylase	PF00282.16^Pyridoxal_deC^Pyridoxal-dependent decarboxylase conserved domain^5-109^E:2.4e-28	1	0	0	5,532	0,034	5,665	0,017	5,917	1	0,365	DCE_PETHY	Glutamate decarboxylase (GAD) (EC 4.1.1.15)	GAD	Petunia hybrida (Petunia)		3D-structure; Calmodulin-binding; Decarboxylase; Lyase; Pyridoxal phosphate
SRR342023_TR5587_c2_g1_i2_len=1204	INT1_ARATH	Q:597-49,H:3-186	80.43%ID	E:3e-60	Inositol transporter 1	PF00083.21^Sugar_tr^Sugar (and other) transporter^32-185^E:3.7e-41`PF07690.13^MFS_1^Major Facilitator Superfamily^37-182^E:5.4e-13	0,666	0,308	0	3,06	0,052	1,46	0,052	1,455	1	0,971	INT1_ARATH	Inositol transporter 1	INT1 At2g43330 T1O24.7	Arabidopsis thaliana (Mouse-ear cress)		Complete proteome; Membrane; Reference proteome; Symport; Transmembrane; Transmembrane helix; Transport; Vacuole
SRR342023_TR5798_c3_g3_i1_len=522	NA	NA	NA	NA	NA	.	0,185	-0,41	0	3,13	0,028	-0,85	0,814	-0,109	1	0	.	.	.	.	.	.
SRR342023_TR6044_c0_g1_i9_len=1904	P2C06_ORYSJ	Q:805-2,H:126-391	57.35%ID	E:2e-99	Probable protein phosphatase 2C 6	PF00481.18^PP2C^Protein phosphatase 2C^134-363^E:3.6e-49	0,319	0,947	0	6,709	1	-0,013	0,781	-0,475	0,004	2,322	P2C06_ORYSJ	Probable protein phosphatase 2C 6 (OsPP2C06) (EC 3.1.3.16)	Os01g0583100 LOC_Os01g40094	Oryza sativa subsp. japonica (Rice)		3D-structure; Complete proteome; Hydrolase; Magnesium; Manganese; Metal-binding; Protein phosphatase; Reference proteome
SRR342023_TR6962_c0_g6_i1_len=1484	SPSA4_ARATH	Q:1052-3,H:205-555	82.91%ID	E:0	Probable sucrose-phosphate synthase 4	PF13579.3^Glyco_trans_4_4^Glycosyl transferase 4-like domain^10-203^E:1.5e-07`PF13439.3^Glyco_transf_4^Glycosyltransferase Family 4^10-159^E:1.5e-06`PF00862.16^Sucrose_synth^Sucrose synthase^68-214^E:8.1e-08	0,212	0,915	0	3,555	0,117	0,977	0,117	0,968	0,008	0,889	SPSA4_ARATH	Probable sucrose-phosphate synthase 4 (EC 2.4.1.14) (Sucrose phosphate synthase 4F) (AtSPS4F) (UDP-glucose-fructose-phosphate glucosyltransferase)	SPS4 SPSC At4g10120 F28M11.40	Arabidopsis thaliana (Mouse-ear cress)	PATHWAY: Glycan biosynthesis; sucrose biosynthesis; sucrose from D-fructose 6-phosphate and UDP-alpha-D-glucose: step 1/2.	Complete proteome; Glycosyltransferase; Phosphoprotein; Reference proteome; Transferase
SRR342023_TR7033_c4_g6_i3_len=1039	FLS_PETHY	Q:911-96,H:11-279	74.73%ID	E:3e-138	Flavonol synthase/flavanone 3-hydroxylase	PF14226.3^DIOX_N^non-haem dioxygenase in morphine synthesis N-terminal^43-154^E:1e-25`PF03171.17^2OG-FeII_Oxy^2OG-Fe(II) oxygenase superfamily^204-270^E:1.7e-16	0,945	-0,059	0	1,897	0,004	-0,713	0,009	-0,639	1	0,288	FLS_PETHY	Flavonol synthase/flavanone 3-hydroxylase (FLS) (EC 1.14.11.23) (EC 1.14.11.9)	FL	Petunia hybrida (Petunia)	PATHWAY: Secondary metabolite biosynthesis; flavonoid biosynthesis.	Cytoplasm; Dioxygenase; Flavonoid biosynthesis; Iron; Metal-binding; Oxidoreductase; Vitamin C
SRR342023_TR7067_c0_g1_i1_len=769	NA	NA	NA	NA	NA	.	0,035	0,661	0	1,6	0,001	0,743	0,002	0,703	0,874	-0,105	.	.	.	.	.	.
SRR342023_TR10321_c1_g2_i1_len=935	NA	NA	NA	NA	NA	PF05004.10^IFRD^Interferon-related developmental regulator (IFRD)^32-195^E:9.1e-42	0,921	0,072	0,001	1,536	0,486	0,237	0,169	0,439	1	1,053	.	.	.	.	.	.
SRR342023_TR10433_c1_g1_i2_len=2969	P5CS_ACTDE	Q:2486-345,H:1-713	86.69%ID	E:0	Delta-1-pyrroline-5-carboxylate synthase	PF00696.25^AA_kinase^Amino acid kinase family^16-261^E:1.1e-38`PF00171.19^Aldedh^Aldehyde dehydrogenase family^294-559^E:6.4e-08	0,41	-0,324	0	2,252	0,511	0,204	0,256	-0,343	0,381	1,865	P5CS_ACTDE	Delta-1-pyrroline-5-carboxylate synthase (P5CS) [Includes: Glutamate 5-kinase (GK) (EC 2.7.2.11) (Gamma-glutamyl kinase); Gamma-glutamyl phosphate reductase (GPR) (EC 1.2.1.41) (Glutamate-5-semialdehyde dehydrogenase) (Glutamyl-gamma-semialdehyde dehydrogenase)]		Actinidia deliciosa (Kiwi)	PATHWAY: Amino-acid biosynthesis; L-proline biosynthesis; L-glutamate 5-semialdehyde from L-glutamate: step 1/2.; PATHWAY: Amino-acid biosynthesis; L-proline biosynthesis; L-glutamate 5-semialdehyde from L-glutamate: step 2/2.	ATP-binding; Amino-acid biosynthesis; Kinase; Multifunctional enzyme; NADP; Nucleotide-binding; Oxidoreductase; Proline biosynthesis; Transferase
SRR342023_TR12131_c0_g3_i1_len=605	UBL5_ARATH	Q:494-276,H:1-73	98.63%ID	E:2e-43	Ubiquitin-like protein 5	.	0,005	0,739	0	1,402	0,047	0,496	0,094	0,421	0,034	5,726	UBL5_ARATH	Ubiquitin-like protein 5	UBL5 At5g42300 K5J14.10	Arabidopsis thaliana (Mouse-ear cress)		Complete proteome; Cytoplasm; Reference proteome; Ubl conjugation pathway
SRR342023_TR12510_c1_g2_i1_len=1214	ALL8_OLEEU	Q:490-960,H:8-161	67.52%ID	E:2e-66	Calcium-binding allergen Ole e 8	PF10591.6^SPARC_Ca_bdg^Secreted protein acidic and rich in cysteine Ca binding region^20-82^E:0.00011`PF13833.3^EF-hand_8^EF-hand domain pair^22-49^E:0.00063`PF00036.29^EF-hand_1^EF hand^23-51^E:2.7e-08`PF13405.3^EF-hand_6^EF-hand domain^23-52^E:1.1e-06`PF13499.3^EF-hand_7^EF-hand domain pair^24-84^E:5e-14`PF13202.3^EF-hand_5^EF hand^25-47^E:5.3e-09`PF13833.3^EF-hand_8^EF-hand domain pair^55-82^E:0.00014`PF00036.29^EF-hand_1^EF hand^59-85^E:6.7e-08`PF13202.3^EF-hand_5^EF hand^61-83^E:9.2e-05`PF13499.3^EF-hand_7^EF-hand domain pair^101-163^E:3.5e-16`PF00036.29^EF-hand_1^EF hand^102-130^E:1.9e-07`PF13405.3^EF-hand_6^EF-hand domain^102-131^E:1.2e-07`PF13202.3^EF-hand_5^EF hand^104-125^E:7.5e-05`PF13833.3^EF-hand_8^EF-hand domain pair^114-164^E:5.5e-13`PF00036.29^EF-hand_1^EF hand^140-165^E:1.4e-07`PF13202.3^EF-hand_5^EF hand^145-163^E:7.5e-05	0,679	0,408	0	3,026	1	0,006	0,011	0,733	0,044	-0,802	ALL8_OLEEU	Calcium-binding allergen Ole e 8 (PCA18/PCA23) (allergen Ole e 8)		Olea europaea (Common olive)		Allergen; Calcium; Metal-binding; Polymorphism; Repeat
SRR342023_TR12885_c0_g1_i1_len=1070	COPT5_ARATH	Q:844-365,H:1-146	53.42%ID	E:2e-34	Copper transporter 5	PF04145.12^Ctr^Ctr copper transporter family^4-143^E:1.5e-24	0,031	0,874	0	2,008	0,051	0,805	0,019	0,941	1	0,637	COPT5_ARATH	Copper transporter 5 (AtCOPT5)	COPT5 At5g20650 T1M15.50	Arabidopsis thaliana (Mouse-ear cress)		Complete proteome; Copper; Copper transport; Ion transport; Membrane; Reference proteome; Transmembrane; Transmembrane helix; Transport
SRR342023_TR14316_c0_g1_i5_len=1804	NA	NA	NA	NA	NA	PF00226.28^DnaJ^DnaJ domain^11-76^E:3.7e-26	0,005	1,039	0,001	1,078	1	-1,309	1	-1,56	0,156	0,591	.	.	.	.	.	.
SRR342023_TR14686_c0_g1_i2_len=2205	FAP2_ARATH	Q:729-1865,H:24-398	53.87%ID	E:9e-124	Fatty-acid-binding protein 2	PF16035.2^Chalcone_2^Chalcone isomerase like^234-406^E:2.4e-16`PF16036.2^Chalcone_3^Chalcone isomerase-like^289-405^E:5.2e-07	0,822	0,296	0	2,702	0,088	1,349	0,001	2,237	1	-0,008	FAP2_ARATH	Fatty-acid-binding protein 2 (AtFAP2) (Chalcone-flavanone isomerase family protein 2)	FAP2 At2g26310 T1D16.5	Arabidopsis thaliana (Mouse-ear cress)		Alternative splicing; Chloroplast; Complete proteome; Plastid; Reference proteome
SRR342023_TR15920_c0_g1_i1_len=818	IMP3_MOUSE	Q:85-621,H:2-181	57.78%ID	E:1e-72	U3 small nucleolar ribonucleoprotein protein IMP3	PF00163.16^Ribosomal_S4^Ribosomal protein S4/S9 N-terminal domain^3-62^E:3.1e-10`PF01479.22^S4^S4 domain^107-153^E:3e-10	0,226	2,335	0	6,978	0,066	3,352	0,129	3,079	0	-7,671	IMP3_MOUSE	U3 small nucleolar ribonucleoprotein protein IMP3 (U3 snoRNP protein IMP3)	Imp3	Mus musculus (Mouse)		Complete proteome; Nucleus; RNA-binding; Reference proteome; Ribonucleoprotein; Ribosome biogenesis; rRNA processing; rRNA-binding
SRR342023_TR15973_c0_g3_i1_len=3335	Y3475_ARATH	Q:3118-185,H:46-998	35.92%ID	E:1e-180	Probable LRR receptor-like serine/threonine-protein kinase At3g47570	PF08263.9^LRRNT_2^Leucine rich repeat N-terminal domain^42-88^E:2.1e-07`PF13855.3^LRR_8^Leucine rich repeat^165-225^E:6.4e-08`PF13516.3^LRR_6^Leucine Rich repeat^385-400^E:0.42`PF00560.30^LRR_1^Leucine Rich Repeat^388-410^E:0.028`PF13516.3^LRR_6^Leucine Rich repeat^460-475^E:1.2`PF00560.30^LRR_1^Leucine Rich Repeat^607-629^E:0.0034`PF13516.3^LRR_6^Leucine Rich repeat^607-620^E:1.1`PF00069.22^Pkinase^Protein kinase domain^750-950^E:1.5e-44`PF07714.14^Pkinase_Tyr^Protein tyrosine kinase^754-952^E:6.4e-42	0,004	0,989	0	1,7	0,195	1,149	0,004	2,026	0,245	-0,513	Y3475_ARATH	Probable LRR receptor-like serine/threonine-protein kinase At3g47570 (EC 2.7.11.1)	At3g47570 F1P2.120	Arabidopsis thaliana (Mouse-ear cress)		ATP-binding; Cell membrane; Complete proteome; Glycoprotein; Kinase; Leucine-rich repeat; Membrane; Nucleotide-binding; Phosphoprotein; Receptor; Reference proteome; Repeat; Serine/threonine-protein kinase; Signal; Transferase; Transmembrane; Transmembrane helix
SRR342023_TR16547_c0_g1_i1_len=1366	NA	NA	NA	NA	NA	.	0,126	0,633	0	1,457	0,52	0,147	0,945	-0,02	0,852	0,068	.	.	.	.	.	.
SRR342023_TR18138_c2_g3_i3_len=2780	DRP2A_ARATH	Q:1304-3,H:361-794	81.01%ID	E:0	Dynamin-2A	PF02469.19^Fasciclin^Fasciclin domain^129-258^E:1.2e-13	0,071	0,549	0	1,631	0,005	0,902	0,004	0,928	1	0,701	DRP2A_ARATH	Dynamin-2A (EC 3.6.5.5) (Dynamin-like protein 6) (Dynamin-related protein 2A)	DRP2A ADL6 At1g10290 F14N23.17	Arabidopsis thaliana (Mouse-ear cress)		Acetylation; Coiled coil; Complete proteome; Cytoplasm; Cytoplasmic vesicle; Cytoskeleton; GTP-binding; Golgi apparatus; Hydrolase; Lipid-binding; Membrane; Microtubule; Motor protein; Nucleotide-binding; Reference proteome
SRR342023_TR19692_c2_g6_i1_len=2356	CAP10_ARATH	Q:1969-536,H:11-479	70.54%ID	E:0	Putative clathrin assembly protein At5g35200	PF07651.13^ANTH^ANTH domain^34-301^E:7.9e-91	0,255	0,623	0	1,839	0,093	0,788	0,075	0,829	1	0,917	CAP10_ARATH	Putative clathrin assembly protein At5g35200	At5g35200 K3D20.4 T25C13.80	Arabidopsis thaliana (Mouse-ear cress)		Coated pit; Complete proteome; Cytoplasmic vesicle; Endocytosis; Golgi apparatus; Membrane; Phosphoprotein; Reference proteome
SRR342023_TR20699_c2_g3_i2_len=2023	AROF_SOLTU	Q:107-1720,H:1-536	83.27%ID	E:0	Phospho-2-dehydro-3-deoxyheptonate aldolase 1, chloroplastic	PF01474.13^DAHP_synth_2^Class-II DAHP synthetase family^96-532^E:3.7e-197	0,455	0,23	0	1,349	0,003	-0,803	0,071	-0,485	1	-0,117	AROF_SOLTU	Phospho-2-dehydro-3-deoxyheptonate aldolase 1, chloroplastic (EC 2.5.1.54) (3-deoxy-D-arabino-heptulosonate 7-phosphate synthase 1) (DAHP synthase 1) (Phospho-2-keto-3-deoxyheptonate aldolase 1)	SHKA ARO1	Solanum tuberosum (Potato)	PATHWAY: Metabolic intermediate biosynthesis; chorismate biosynthesis; chorismate from D-erythrose 4-phosphate and phosphoenolpyruvate: step 1/7.	Amino-acid biosynthesis; Aromatic amino acid biosynthesis; Chloroplast; Complete proteome; Direct protein sequencing; Plastid; Reference proteome; Transferase; Transit peptide
SRR342023_TR20776_c0_g3_i1_len=805	NA	NA	NA	NA	NA	PF00403.23^HMA^Heavy-metal-associated domain^13-70^E:1.9e-16	0,839	-0,121	0	3,076	0,038	1,897	0,025	2,016	0,24	0,413	.	.	.	.	.	.
SRR342023_TR20862_c0_g2_i1_len=2097	DCR_ARATH	Q:1729-344,H:3-448	32.24%ID	E:1e-62	BAHD acyltransferase DCR	PF02458.12^Transferase^Transferase family^5-466^E:6.5e-109`PF01396.16^zf-C4_Topoisom^Topoisomerase DNA binding C4 zinc finger^12-24^E:2	0,02	1,143	0	2,143	0,044	1,046	0,059	1,001	1	-0,745	DCR_ARATH	BAHD acyltransferase DCR (EC 2.3.1.-) (Protein DEFECTIVE IN CUTICULAR RIDGES) (Protein PERMEABLE LEAVES 3)	DCR PEL3 At5g23940 MRO11.2	Arabidopsis thaliana (Mouse-ear cress)		Acyltransferase; Complete proteome; Cytoplasm; Reference proteome; Transferase
SRR342023_TR22238_c1_g1_i2_len=1056	AGL8_SOLTU	Q:284-988,H:1-249	60.87%ID	E:4e-90	Agamous-like MADS-box protein AGL8 homolog	PF00319.15^SRF-TF^SRF-type transcription factor (DNA-binding and dimerisation domain)^104-151^E:2.4e-25`PF01486.14^K-box^K-box region^184-262^E:1.9e-23	0,21	-0,494	0	2,533	0,018	0,844	0,127	0,571	0,268	0,589	AGL8_SOLTU	Agamous-like MADS-box protein AGL8 homolog (POTM1-1)		Solanum tuberosum (Potato)		Complete proteome; DNA-binding; Nucleus; Reference proteome; Transcription; Transcription regulation
SRR342023_TR23059_c0_g3_i2_len=3077	PMTE_ARATH	Q:2384-576,H:1-605	74.09%ID	E:0	Probable methyltransferase PMT14	PF03141.13^Methyltransf_29^Putative S-adenosyl-L-methionine-dependent methyltransferase^93-598^E:1.2e-231`PF08241.9^Methyltransf_11^Methyltransferase domain^210-304^E:8.1e-06	0,13	0,485	0	1,344	0,721	0,176	0,721	0,184	0,555	-0,277	PMTE_ARATH	Probable methyltransferase PMT14 (EC 2.1.1.-)	At4g18030 T6K21.210	Arabidopsis thaliana (Mouse-ear cress)		Complete proteome; Glycoprotein; Golgi apparatus; Membrane; Methyltransferase; Reference proteome; Signal-anchor; Transferase; Transmembrane; Transmembrane helix
SRR342023_TR23812_c0_g3_i1_len=2056	PDI_DATGL	Q:145-1539,H:26-492	69.16%ID	E:0	Protein disulfide-isomerase	PF00085.17^Thioredoxin^Thioredoxin^33-139^E:1.4e-27`PF13848.3^Thioredoxin_6^Thioredoxin-like domain^170-352^E:1.3e-19`PF00085.17^Thioredoxin^Thioredoxin^375-478^E:6.6e-26	0,593	0,15	0	1,426	0,003	0,85	0,013	0,725	0,013	0,689	PDI_DATGL	Protein disulfide-isomerase (PDI) (EC 5.3.4.1)	PDI	Datisca glomerata (Durango root)		Disulfide bond; Endoplasmic reticulum; Glycoprotein; Isomerase; Redox-active center; Repeat; Signal
SRR342023_TR24547_c0_g1_i1_len=933	GGPPS_HEVBR	Q:167-811,H:90-308	34.25%ID	E:5e-22	Geranylgeranyl pyrophosphate synthase, chloroplastic	PF00348.14^polyprenyl_synt^Polyprenyl synthetase^74-253^E:1.9e-09	0,355	0,418	0	2,789	0,009	0,997	0,001	1,237	0,848	0,25	GGPPS_HEVBR	Geranylgeranyl pyrophosphate synthase, chloroplastic (GGPP synthase) (GGPS) (EC 2.5.1.-) ((2E,6E)-farnesyl diphosphate synthase) (Dimethylallyltranstransferase) (EC 2.5.1.1) (Farnesyl diphosphate synthase) (Farnesyltranstransferase) (EC 2.5.1.29) (Geranyltranstransferase) (EC 2.5.1.10)	GGPS GG GGPPS1	Hevea brasiliensis (Para rubber tree) (Siphonia brasiliensis)	PATHWAY: Isoprenoid biosynthesis; farnesyl diphosphate biosynthesis; farnesyl diphosphate from geranyl diphosphate and isopentenyl diphosphate: step 1/1.; PATHWAY: Isoprenoid biosynthesis; geranyl diphosphate biosynthesis; geranyl diphosphate from dimethylallyl diphosphate and isopentenyl diphosphate: step 1/1.; PATHWAY: Isoprenoid biosynthesis; geranylgeranyl diphosphate biosynthesis; geranylgeranyl diphosphate from farnesyl diphosphate and isopentenyl diphosphate: step 1/1.	Carotenoid biosynthesis; Chloroplast; Isoprene biosynthesis; Magnesium; Metal-binding; Plastid; Transferase; Transit peptide
SRR342023_TR25191_c7_g5_i1_len=3144	NIA_PETHY	Q:3012-265,H:1-909	84.42%ID	E:0	Nitrate reductase [NADH]	PF00174.16^Oxidored_molyb^Oxidoreductase molybdopterin binding domain^144-323^E:2.5e-55`PF03404.13^Mo-co_dimer^Mo-co oxidoreductase dimerisation domain^351-481^E:1e-53`PF00173.25^Cyt-b5^Cytochrome b5-like Heme/Steroid binding domain^543-611^E:1.7e-22`PF00970.21^FAD_binding_6^Oxidoreductase FAD-binding domain^663-769^E:1.3e-33`PF00175.18^NAD_binding_1^Oxidoreductase NAD-binding domain^789-897^E:5.8e-35	0,067	0,662	0	2,593	0,295	-0,232	0,003	0,651	1	-0,373	NIA_PETHY	Nitrate reductase [NADH] (NR) (EC 1.7.1.1)	NIA	Petunia hybrida (Petunia)		Disulfide bond; FAD; Flavoprotein; Heme; Iron; Metal-binding; Molybdenum; NAD; Nitrate assimilation; Oxidoreductase
SRR342023_TR27467_c3_g4_i3_len=6071	POLX_TOBAC	Q:6071-2694,H:221-1323	43.99%ID	E:0	Retrovirus-related Pol polyprotein from transposon TNT 1-94	PF13976.3^gag_pre-integrs^GAG-pre-integrase domain^183-250^E:2.8e-14`PF00665.23^rve^Integrase core domain^266-380^E:6e-26`PF07727.11^RVT_2^Reverse transcriptase (RNA-dependent DNA polymerase)^640-887^E:4.5e-93	0,215	0,367	0	8,263	0,003	-0,974	0,001	-1,086	1	1,661	POLX_TOBAC	Retrovirus-related Pol polyprotein from transposon TNT 1-94 [Includes: Protease (EC 3.4.23.-); Reverse transcriptase (EC 2.7.7.49); Endonuclease]		Nicotiana tabacum (Common tobacco)		Aspartyl protease; Endonuclease; Hydrolase; Metal-binding; Nuclease; Nucleotidyltransferase; Protease; RNA-directed DNA polymerase; Transferase; Transposable element; Zinc; Zinc-finger
SRR342023_TR28675_c0_g1_i2_len=627	CHAC2_MOUSE	Q:481-65,H:2-134	49.29%ID	E:1e-33	Putative glutathione-specific gamma-glutamylcyclotransferase 2 {ECO:0000250|UniProtKB:Q9BUX1}	PF04752.9^ChaC^ChaC-like protein^4-142^E:9e-39	0,14	-0,659	0,001	6,572	0,756	-0,272	0,635	-0,409	0,502	-4,252	CHAC2_MOUSE	Putative glutathione-specific gamma-glutamylcyclotransferase 2 (Gamma-GCG 2) (EC 2.3.2.-) (Cation transport regulator-like protein 2)	Chac2	Mus musculus (Mouse)		Acyltransferase; Alternative splicing; Complete proteome; Reference proteome; Transferase
SRR342023_TR28996_c1_g3_i2_len=700	AGLU_SPIOL	Q:389-3,H:43-181	53.19%ID	E:3e-28	Alpha-glucosidase	PF16863.2^NtCtMGAM_N^N-terminal barrel of NtMGAM and CtMGAM, maltase-glucoamylase^115-225^E:3.2e-28	0,01	1,289	0	4,759	1	-0,616	0,502	1,861	1	1,631	AGLU_SPIOL	Alpha-glucosidase (EC 3.2.1.20) (Maltase)		Spinacia oleracea (Spinach)		Direct protein sequencing; Glycoprotein; Glycosidase; Hydrolase; Signal
SRR342023_TR29320_c0_g3_i3_len=876	ERD15_ARATH	Q:142-630,H:1-163	44.07%ID	E:6e-25	Protein EARLY RESPONSIVE TO DEHYDRATION 15	.	0,035	0,531	0	1,158	0,003	1,076	0,011	0,934	0,818	-0,067	ERD15_ARATH	Protein EARLY RESPONSIVE TO DEHYDRATION 15 (PAM2-containing protein CID1) (Polyadenylate-binding protein-interacting protein 1) (PABP-interacting protein 1) (Poly(A)-binding protein-interacting protein 1) (Protein CTC-INTERACTING DOMAIN 1) (Protein LIGHT STRESS-REGULATED 1)	ERD15 CID1 LSR1 At2g41430 F13H10.2 T26J13.2	Arabidopsis thaliana (Mouse-ear cress)		Alternative splicing; Complete proteome; Cytoplasm; Reference proteome
SRR342023_TR29950_c1_g5_i2_len=2744	PCP_PONAB	Q:914-2311,H:25-488	44.4%ID	E:9e-136	Lysosomal Pro-X carboxypeptidase	PF05577.9^Peptidase_S28^Serine carboxypeptidase S28^66-488^E:2.6e-86	0,356	1,475	0	3,356	0,189	1,866	1	0,753	1	-1,152	PCP_PONAB	Lysosomal Pro-X carboxypeptidase (EC 3.4.16.2) (Proline carboxypeptidase) (Prolylcarboxypeptidase) (PRCP)	PRCP	Pongo abelii (Sumatran orangutan) (Pongo pygmaeus abelii)		Carboxypeptidase; Complete proteome; Disulfide bond; Glycoprotein; Hydrolase; Lysosome; Protease; Reference proteome; Signal; Zymogen
SRR342023_TR30512_c0_g1_i4_len=2442	SERA3_ARATH	Q:941-3,H:46-358	87.54%ID	E:6e-166	D-3-phosphoglycerate dehydrogenase 3, chloroplastic	PF00389.27^2-Hacid_dh^D-isomer specific 2-hydroxyacid dehydrogenase, catalytic domain^109-418^E:7e-35`PF02826.16^2-Hacid_dh_C^D-isomer specific 2-hydroxyacid dehydrogenase, NAD binding domain^211-386^E:4.8e-63`PF00670.18^AdoHcyase_NAD^S-adenosyl-L-homocysteine hydrolase, NAD binding domain^241-350^E:1e-06`PF07991.9^IlvN^Acetohydroxy acid isomeroreductase, NADPH-binding domain^243-346^E:7.9e-07	0,325	0,429	0	3,596	0,394	0,48	0,763	0,206	1	0,501	SERA3_ARATH	D-3-phosphoglycerate dehydrogenase 3, chloroplastic (EC 1.1.1.95)	PGDH3 3-PGDH At3g19480 MLD14.22	Arabidopsis thaliana (Mouse-ear cress)	PATHWAY: Amino-acid biosynthesis; L-serine biosynthesis; L-serine from 3-phospho-D-glycerate: step 1/3.	Chloroplast; Complete proteome; NAD; Oxidoreductase; Plastid; Reference proteome; Transit peptide
SRR342023_TR31127_c1_g9_i6_len=2527	CGS1_ARATH	Q:1570-365,H:163-563	85.57%ID	E:0	Cystathionine gamma-synthase 1, chloroplastic {ECO:0000305}	PF01053.17^Cys_Met_Meta_PP^Cys/Met metabolism PLP-dependent enzyme^157-540^E:1.1e-140	0,387	-0,446	0	1,887	0,787	-0,123	0,799	0,143	1	-1,405	CGS1_ARATH	Cystathionine gamma-synthase 1, chloroplastic (AtCGS1) (EC 2.5.1.48) (METHIONINE OVERACCUMULATION 1) (O-succinylhomoserine (thiol)-lyase)	CGS1 CYS1 MTO1 At3g01120 T4P13.19	Arabidopsis thaliana (Mouse-ear cress)	PATHWAY: Amino-acid biosynthesis; L-methionine biosynthesis via de novo pathway; L-cystathionine from O-succinyl-L-homoserine: step 1/1. {ECO:0000305}.	Amino-acid biosynthesis; Chloroplast; Complete proteome; Direct protein sequencing; Methionine biosynthesis; Plastid; Polymorphism; Pyridoxal phosphate; Reference proteome; Transferase; Transit peptide
SRR342023_TR31490_c0_g2_i1_len=535	NA	NA	NA	NA	NA	.	0,692	0,692	0	4,743	1	3,268	0,502	3,841	1	-0,589	.	.	.	.	.	.
SRR646572_TR6401_c0_g1_i1_len=556	10HGO_CATRO	Q:354-4,H:2-118	69.23%ID	E:4e-53	8-hydroxygeraniol dehydrogenase	PF08240.9^ADH_N^Alcohol dehydrogenase GroES-like domain^39-119^E:5.2e-22	0,017	5,772	0	5,08	0,005	6,165	0,009	6,005	0,732	0,701	10HGO_CATRO	8-hydroxygeraniol dehydrogenase (Cr10HGO) (EC 1.1.1.324)	10HGO	Catharanthus roseus (Madagascar periwinkle) (Vinca rosea)		Metal-binding; NADP; Oxidoreductase; Zinc
SRR646572_TR6509_c0_g1_i3_len=2551	CSE_ARATH	Q:1177-266,H:24-323	31.8%ID	E:9e-36	Caffeoylshikimate esterase	PF12146.5^Hydrolase_4^Serine aminopeptidase, S33^58-301^E:1.6e-56`PF00561.17^Abhydrolase_1^alpha/beta hydrolase fold^59-173^E:2.1e-06`PF12697.4^Abhydrolase_6^Alpha/beta hydrolase family^63-299^E:1e-09	0,129	0,53	0	2,067	0,58	0,268	0,11	0,636	0,555	-0,332	CSE_ARATH	Caffeoylshikimate esterase (EC 3.1.1.-) (Lysophospholipase 2) (LysoPL2)	CSE At1g52760 F14G24.3	Arabidopsis thaliana (Mouse-ear cress)		Cell membrane; Complete proteome; Hydrolase; Lignin biosynthesis; Membrane; Reference proteome
SRR646572_TR13586_c0_g1_i1_len=1078	EXPA8_ARATH	Q:956-261,H:20-252	84.55%ID	E:8e-143	Expansin-A8	PF03330.15^DPBB_1^Lytic transglycolase^86-171^E:1.4e-21`PF01357.18^Pollen_allerg_1^Pollen allergen^182-259^E:1.2e-29	0,298	0,637	0	2,421	0	-3,456	0	-1,555	0,56	-0,749	EXPA8_ARATH	Expansin-A8 (AtEXPA8) (Alpha-expansin-8) (At-EXP8) (AtEx8) (Ath-ExpAlpha-1.11)	EXPA8 EXP8 At2g40610 T2P4.4	Arabidopsis thaliana (Mouse-ear cress)		Cell wall; Cell wall biogenesis/degradation; Complete proteome; Disulfide bond; Membrane; Reference proteome; Secreted; Signal
SRR646572_TR18397_c0_g2_i1_len=519	ARF_MAIZE	Q:170-502,H:1-111	99.1%ID	E:4e-75	ADP-ribosylation factor	PF00025.18^Arf^ADP-ribosylation factor family^5-112^E:5.4e-52`PF09439.7^SRPRB^Signal recognition particle receptor beta subunit^16-106^E:9.6e-11`PF08477.10^Roc^Ras of Complex, Roc, domain of DAPkinase^19-100^E:2.2e-12`PF04670.9^Gtr1_RagA^Gtr1/RagA G protein conserved region^19-110^E:6.5e-09`PF01926.20^MMR_HSR1^50S ribosome-binding GTPase^19-106^E:0.00011`PF00071.19^Ras^Ras family^20-99^E:1.9e-09	0,927	0,027	0	1,21	0,806	-0,063	0,695	-0,094	0	-10,197	ARF_MAIZE	ADP-ribosylation factor	ARF1	Zea mays (Maize)		Complete proteome; ER-Golgi transport; GTP-binding; Golgi apparatus; Lipoprotein; Myristate; Nucleotide-binding; Protein transport; Reference proteome; Transport
SRR646572_TR18608_c0_g1_i1_len=1877	ENO_SOLLC	Q:157-1485,H:1-444	93.24%ID	E:0	Enolase	PF03952.13^Enolase_N^Enolase, N-terminal domain^56-191^E:3.3e-56`PF00113.19^Enolase_C^Enolase, C-terminal TIM barrel domain^200-491^E:5.1e-161`PF13378.3^MR_MLE_C^Enolase C-terminal domain-like^327-455^E:1.1e-08`PF07476.8^MAAL_C^Methylaspartate ammonia-lyase C-terminus^349-452^E:1.6e-05	0,052	-0,586	0	1,714	0,562	0,199	0,194	0,442	0,558	-0,187	ENO_SOLLC	Enolase (EC 4.2.1.11) (2-phospho-D-glycerate hydro-lyase) (2-phosphoglycerate dehydratase)	PGH1	Solanum lycopersicum (Tomato) (Lycopersicon esculentum)	PATHWAY: Carbohydrate degradation; glycolysis; pyruvate from D-glyceraldehyde 3-phosphate: step 4/5.	Complete proteome; Cytoplasm; Glycolysis; Lyase; Magnesium; Metal-binding; Reference proteome
SRR646572_TR23378_c1_g1_i1_len=680	PRS1_SOLTU	Q:113-556,H:1-155	35.9%ID	E:9e-22	Pathogenesis-related protein STH-21	PF00407.16^Bet_v_1^Pathogenesis-related protein Bet v I family^4-142^E:1.1e-12	1	0	0	4,213	0,02	0,843	0,01	0,923	0,249	-0,542	PRS1_SOLTU	Pathogenesis-related protein STH-21	STH-21	Solanum tuberosum (Potato)		Complete proteome; Pathogenesis-related protein; Plant defense; Reference proteome
SRR646572_TR24688_c0_g2_i4_len=2579	SYFB_ARATH	Q:2406-628,H:1-598	71.33%ID	E:0	Phenylalanine--tRNA ligase beta subunit, cytoplasmic {ECO:0000305}	PF03483.14^B3_4^B3/4 domain^120-281^E:4.3e-24`PF03484.12^B5^tRNA synthetase B5 domain^309-379^E:3.6e-16	0,234	0,484	0,001	1,38	0,021	1,082	0,107	0,768	1	-0,275	SYFB_ARATH	Phenylalanine--tRNA ligase beta subunit, cytoplasmic (EC 6.1.1.20) (Phenylalanyl-tRNA synthetase beta subunit) (PheRS)	At1g72550 F28P22.26	Arabidopsis thaliana (Mouse-ear cress)		ATP-binding; Alternative splicing; Aminoacyl-tRNA synthetase; Complete proteome; Cytoplasm; Ligase; Magnesium; Metal-binding; Nucleotide-binding; Protein biosynthesis; Reference proteome
SRR646572_TR24916_c0_g1_i2_len=1599	TRL2_ORYSJ	Q:388-897,H:36-202	72.51%ID	E:1e-82	Thioredoxin-like 2, chloroplastic	PF00085.17^Thioredoxin^Thioredoxin^96-182^E:2.7e-18	0,162	0,586	0	1,461	0,935	0,052	0,749	0,134	0,882	-0,068	TRL2_ORYSJ	Thioredoxin-like 2, chloroplastic (Lilium-type thioredoxin 2) (OsTrx19)	Os05g0200100 LOC_Os05g11090 OSJNBa0017O06.11	Oryza sativa subsp. japonica (Rice)		Chloroplast; Complete proteome; Disulfide bond; Electron transport; Plastid; Redox-active center; Reference proteome; Transit peptide; Transport
SRR646572_TR26413_c0_g1_i1_len=2152	MPI1_ARATH	Q:405-1712,H:10-429	56.19%ID	E:5e-163	Mannose-6-phosphate isomerase 1	PF01238.18^PMI_typeI^Phosphomannose isomerase type I^15-399^E:1e-105	0,452	0,603	0,001	1,947	0,028	1,464	0,028	1,506	0,874	0,167	MPI1_ARATH	Mannose-6-phosphate isomerase 1 (EC 5.3.1.8) (Phosphohexomutase 1) (Phosphomannose isomerase 1) (PMI1) (Protein MATERNAL EFFECT EMBRYO ARREST 31)	PMI1 MEE31 At3g02570 F16B3.20	Arabidopsis thaliana (Mouse-ear cress)	PATHWAY: Nucleotide-sugar biosynthesis; GDP-alpha-D-mannose biosynthesis; alpha-D-mannose 1-phosphate from D-fructose 6-phosphate: step 1/2.	Acetylation; Complete proteome; Isomerase; Metal-binding; Reference proteome; Zinc
SRR646572_TR28058_c0_g1_i3_len=2050	SG1_RAUSE	Q:1976-342,H:1-531	71.98%ID	E:0	Strictosidine-O-beta-D-glucosidase	PF00232.15^Glyco_hydro_1^Glycosyl hydrolase family 1^49-520^E:4.2e-155	1	1,78	0	4,355	0,76	0,582	0,129	-4,955	1	-0,033	SG1_RAUSE	Strictosidine-O-beta-D-glucosidase (EC 3.2.1.105)	SGR1	Rauvolfia serpentina (Serpentine wood) (Ophioxylon serpentinum)		3D-structure; Alkaloid metabolism; Glycosidase; Hydrolase
SRR646572_TR32239_c0_g1_i1_len=798	NA	NA	NA	NA	NA	.	1	-0,038	0	1,898	0,06	0,754	0,266	0,482	1	3,396	.	.	.	.	.	.
SRR646572_TR32671_c0_g1_i1_len=1425	ORP4B_ARATH	Q:1223-90,H:4-382	53.02%ID	E:2e-127	Oxysterol-binding protein-related protein 4B	PF01237.15^Oxysterol_BP^Oxysterol-binding protein^101-426^E:9.1e-53	0,044	1,983	0	3,405	1	0,268	1	-0,733	1	1,236	ORP4B_ARATH	Oxysterol-binding protein-related protein 4B (OSBP-related protein 4B)	ORP4B At4g25850 F14M19.130	Arabidopsis thaliana (Mouse-ear cress)		Alternative splicing; Complete proteome; Lipid transport; Lipid-binding; Reference proteome; Transport
SRR646572_TR37244_c0_g4_i1_len=760	NA	NA	NA	NA	NA	.	0,555	0,263	0	6,125	1	-0,842	1	-0,93	0,231	-0,576	.	.	.	.	.	.
SRR646572_TR38156_c0_g1_i1_len=1592	NA	NA	NA	NA	NA	.	0,298	0,617	0	1,607	0,335	0,735	0,147	0,99	1	-2,34	.	.	.	.	.	.
SRR646572_TR38513_c0_g1_i2_len=1805	ADT1_SOLTU	Q:1585-410,H:1-386	86.22%ID	E:0	ADP,ATP carrier protein, mitochondrial	PF00153.24^Mito_carr^Mitochondrial carrier protein^91-187^E:2.5e-23`PF00153.24^Mito_carr^Mitochondrial carrier protein^194-290^E:2.1e-24`PF00153.24^Mito_carr^Mitochondrial carrier protein^296-380^E:5.8e-16	0,844	0,052	0	1,088	0,257	0,27	0,075	0,422	0,306	0,422	ADT1_SOLTU	ADP,ATP carrier protein, mitochondrial (ADP/ATP translocase) (Adenine nucleotide translocator) (ANT)	ANT	Solanum tuberosum (Potato)		Complete proteome; Membrane; Mitochondrion; Mitochondrion inner membrane; Reference proteome; Repeat; Transit peptide; Transmembrane; Transmembrane helix; Transport
SRR646596_TR704_c0_g1_i3_len=2629	AIM1_ARATH	Q:463-1911,H:95-577	74.74%ID	E:0	Peroxisomal fatty acid beta-oxidation multifunctional protein AIM1	PF16113.2^ECH_2^Enoyl-CoA hydratase/isomerase^13-133^E:1.1e-12`PF00378.17^ECH_1^Enoyl-CoA hydratase/isomerase^25-148^E:6.3e-26`PF02737.15^3HCDH_N^3-hydroxyacyl-CoA dehydrogenase, NAD binding domain^243-421^E:3.9e-58`PF00725.19^3HCDH^3-hydroxyacyl-CoA dehydrogenase, C-terminal domain^424-508^E:1.2e-15	0,356	1,155	0	3,842	0,076	2,311	0,025	2,648	0,547	0,406	AIM1_ARATH	Peroxisomal fatty acid beta-oxidation multifunctional protein AIM1 (Protein ABNORMAL INFLORESCENCE MERISTEM 1) (AtAIM1) [Includes: Enoyl-CoA hydratase/3-2-trans-enoyl-CoA isomerase/3-hydroxybutyryl-CoA epimerase (EC 4.2.1.17) (EC 5.1.2.3) (EC 5.3.3.8); 3-hydroxyacyl-CoA dehydrogenase (EC 1.1.1.35)]	AIM1 At4g29010 F19B15.40	Arabidopsis thaliana (Mouse-ear cress)	PATHWAY: Lipid metabolism; fatty acid beta-oxidation.	Complete proteome; Fatty acid metabolism; Isomerase; Lipid metabolism; Lyase; Multifunctional enzyme; NAD; Oxidoreductase; Peroxisome; Reference proteome
SRR646596_TR2176_c0_g1_i1_len=536	NA	NA	NA	NA	NA	.	0,471	0,455	0,001	2,987	0,502	-1,814	1	0,69	0,254	-2,165	.	.	.	.	.	.
SRR646596_TR5222_c0_g1_i3_len=2858	SUT31_ARATH	Q:2207-222,H:1-657	74.36%ID	E:0	Sulfate transporter 3.1	PF00916.17^Sulfate_transp^Sulfate permease family^83-463^E:1.6e-133`PF01740.18^STAS^STAS domain^515-633^E:1.1e-31	0,003	1,559	0	2,799	0,183	-0,442	0,102	-0,561	1	0,078	SUT31_ARATH	Sulfate transporter 3.1 (AST12) (AtST1)	SULTR3;1 ST1 At3g51895 ATEM1.15 F4F15.10	Arabidopsis thaliana (Mouse-ear cress)		Complete proteome; Membrane; Reference proteome; Stress response; Sulfate transport; Symport; Transmembrane; Transmembrane helix; Transport
SRR646596_TR6115_c0_g1_i3_len=2827	C7BL3_CICIN	Q:1266-2648,H:33-486	38.53%ID	E:4e-113	Costunolide synthase	PF00067.19^p450^Cytochrome P450^34-480^E:5.1e-86	0,483	0,264	0	2,09	0,026	0,985	0,897	0,146	1	0	C7BL3_CICIN	Costunolide synthase (CiCOS) (EC 1.14.13.120) (Cytochrome P450 71BL3)	CYP71BL3 COS	Cichorium intybus (Chicory)		Heme; Iron; Membrane; Metal-binding; Monooxygenase; NADP; Oxidoreductase; Transmembrane; Transmembrane helix
SRR646596_TR7168_c2_g6_i1_len=529	COPA1_ARATH	Q:1-528,H:268-443	85.8%ID	E:3e-102	Coatomer subunit alpha-1	PF04053.11^Coatomer_WDAD^Coatomer WD associated region^77-176^E:4e-12	1	-0,002	0,001	2,376	0,046	-0,902	0,108	-0,701	0,029	1,213	COPA1_ARATH	Coatomer subunit alpha-1 (Alpha-coat protein 1) (Alpha-COP 1)	At1g62020 F8K4.21	Arabidopsis thaliana (Mouse-ear cress)		Complete proteome; Cytoplasm; Cytoplasmic vesicle; ER-Golgi transport; Golgi apparatus; Membrane; Protein transport; Reference proteome; Repeat; Transport; WD repeat
SRR646596_TR10511_c0_g1_i2_len=1191	MY108_ARATH	Q:1012-611,H:19-152	71.64%ID	E:4e-60	Transcription factor MYB108	PF00249.28^Myb_DNA-binding^Myb-like DNA-binding domain^30-77^E:2.4e-17`PF13921.3^Myb_DNA-bind_6^Myb-like DNA-binding domain^33-90^E:6.4e-13`PF00249.28^Myb_DNA-binding^Myb-like DNA-binding domain^83-126^E:1.4e-14`PF13921.3^Myb_DNA-bind_6^Myb-like DNA-binding domain^87-130^E:9.1e-06	0,692	0,999	0,001	4,543	0,014	4,018	0,076	3,417	1	0,546	MY108_ARATH	Transcription factor MYB108 (Myb-related protein 108) (AtMYB108) (Protein BOTRYTIS-SUSCEPTIBLE 1)	MYB108 BOS1 At3g06490 F5E6.18	Arabidopsis thaliana (Mouse-ear cress)		Activator; Complete proteome; DNA-binding; Nucleus; Plant defense; Reference proteome; Repeat; Transcription; Transcription regulation; Ubl conjugation
SRR646596_TR12166_c0_g1_i1_len=2773	PDAT1_ARATH	Q:2326-440,H:47-671	80.45%ID	E:0	Phospholipid:diacylglycerol acyltransferase 1	PF02450.12^LCAT^Lecithin:cholesterol acyltransferase^129-370^E:5.7e-45`PF02450.12^LCAT^Lecithin:cholesterol acyltransferase^473-630^E:9.9e-19	0,707	0,368	0	2,25	0,013	1,137	0,043	0,962	1	0	PDAT1_ARATH	Phospholipid:diacylglycerol acyltransferase 1 (AtPDAT) (EC 2.3.1.158)	PDAT1 PDAT At5g13640 MSH12.10 T6I14.2	Arabidopsis thaliana (Mouse-ear cress)	PATHWAY: Glycerolipid metabolism; triacylglycerol biosynthesis.	Acyltransferase; Complete proteome; Glycerol metabolism; Glycoprotein; Lipid biosynthesis; Lipid metabolism; Membrane; Reference proteome; Transferase; Transmembrane; Transmembrane helix
SRR646596_TR12597_c0_g1_i1_len=3507	INT1_ARATH	Q:2165-3085,H:185-496	69.23%ID	E:1e-123	Inositol transporter 1	PF00083.21^Sugar_tr^Sugar (and other) transporter^7-297^E:8.9e-80`PF07690.13^MFS_1^Major Facilitator Superfamily^8-246^E:8.2e-12	0,14	0,486	0	1,275	0,175	0,473	0,496	0,269	0,497	-0,247	INT1_ARATH	Inositol transporter 1	INT1 At2g43330 T1O24.7	Arabidopsis thaliana (Mouse-ear cress)		Complete proteome; Membrane; Reference proteome; Symport; Transmembrane; Transmembrane helix; Transport; Vacuole
SRR646596_TR12597_c0_g2_i1_len=2654	INT1_ARATH	Q:769-2232,H:3-496	73.28%ID	E:0	Inositol transporter 1	PF00083.21^Sugar_tr^Sugar (and other) transporter^32-475^E:9e-125`PF07690.13^MFS_1^Major Facilitator Superfamily^37-424^E:5.5e-30	0,655	0,232	0	2,417	0,105	1,029	0,105	1,027	0,882	0,154	INT1_ARATH	Inositol transporter 1	INT1 At2g43330 T1O24.7	Arabidopsis thaliana (Mouse-ear cress)		Complete proteome; Membrane; Reference proteome; Symport; Transmembrane; Transmembrane helix; Transport; Vacuole
SRR646596_TR15196_c0_g1_i1_len=2351	AROF_ARATH	Q:2156-597,H:1-523	79.16%ID	E:0	Phospho-2-dehydro-3-deoxyheptonate aldolase 1, chloroplastic	PF01474.13^DAHP_synth_2^Class-II DAHP synthetase family^137-573^E:6.5e-198	0,312	0,619	0	1,916	0,175	1,007	0,663	0,487	0,352	0,828	AROF_ARATH	Phospho-2-dehydro-3-deoxyheptonate aldolase 1, chloroplastic (EC 2.5.1.54) (3-deoxy-D-arabino-heptulosonate 7-phosphate synthase 1) (DAHP synthase 1) (Phospho-2-keto-3-deoxyheptonate aldolase 1)	DHS1 At4g39980 T5J17.150	Arabidopsis thaliana (Mouse-ear cress)	PATHWAY: Metabolic intermediate biosynthesis; chorismate biosynthesis; chorismate from D-erythrose 4-phosphate and phosphoenolpyruvate: step 1/7.	Amino-acid biosynthesis; Aromatic amino acid biosynthesis; Chloroplast; Complete proteome; Plastid; Reference proteome; Transferase; Transit peptide
SRR646596_TR16794_c0_g1_i2_len=1994	HIBC8_ARATH	Q:395-1540,H:1-383	75.98%ID	E:0	3-hydroxyisobutyryl-CoA hydrolase-like protein 5	PF00378.17^ECH_1^Enoyl-CoA hydratase/isomerase^19-251^E:5.5e-28`PF16113.2^ECH_2^Enoyl-CoA hydratase/isomerase^21-353^E:1.7e-110	0,156	0,61	0	2,219	0,283	0,651	0,52	0,452	0,471	0,412	HIBC8_ARATH	3-hydroxyisobutyryl-CoA hydrolase-like protein 5 (EC 3.1.2.-)	At1g06550 F12K11.12	Arabidopsis thaliana (Mouse-ear cress)		Acetylation; Complete proteome; Hydrolase; Reference proteome
SRR646596_TR21262_c0_g1_i2_len=1890	AAP6_ARATH	Q:1667-249,H:6-479	77.89%ID	E:0	Amino acid permease 6	PF01490.15^Aa_trans^Transmembrane amino acid transporter protein^104-536^E:2.2e-116	0,01	0,805	0,001	1,015	0,649	0,128	0,176	0,364	0,746	-0,141	AAP6_ARATH	Amino acid permease 6 (Amino acid transporter AAP6)	AAP6 At5g49630 MNI5.1	Arabidopsis thaliana (Mouse-ear cress)		Amino-acid transport; Cell membrane; Complete proteome; Membrane; Reference proteome; Symport; Transmembrane; Transmembrane helix; Transport
SRR646596_TR24774_c0_g1_i1_len=1047	GSTU9_ARATH	Q:823-215,H:9-214	42.72%ID	E:7e-46	Glutathione S-transferase U9	PF02798.17^GST_N^Glutathione S-transferase, N-terminal domain^25-96^E:1.5e-16`PF13417.3^GST_N_3^Glutathione S-transferase, N-terminal domain^28-103^E:1.2e-15`PF13409.3^GST_N_2^Glutathione S-transferase, N-terminal domain^34-97^E:1.5e-07	0,019	0,623	0	1,342	0,001	0,956	0,244	0,37	0,635	0,342	GSTU9_ARATH	Glutathione S-transferase U9 (AtGSTU9) (EC 2.5.1.18) (GST class-tau member 9) (Glutathione S-transferase 14)	GSTU9 GST14 GST14B At5g62480 K19B1.9	Arabidopsis thaliana (Mouse-ear cress)		Alternative splicing; Complete proteome; Cytoplasm; Detoxification; Phosphoprotein; Reference proteome; Transferase
SRR646596_TR25083_c0_g1_i1_len=2052	CAAT1_ARATH	Q:240-1946,H:9-579	73.73%ID	E:0	Cationic amino acid transporter 1	PF13520.3^AA_permease_2^Amino acid permease^74-475^E:1.2e-43`PF00324.18^AA_permease^Amino acid permease^78-443^E:6.7e-32`PF13906.3^AA_permease_C^C-terminus of AA_permease^513-563^E:1.7e-16	0,692	1,128	0	4,242	0,629	1,491	1	0,331	0,226	-2,02	CAAT1_ARATH	Cationic amino acid transporter 1 (Amino acid transporter 1)	CAT1 AAT1 At4g21120 F7J7.60	Arabidopsis thaliana (Mouse-ear cress)		Acetylation; Amino-acid transport; Complete proteome; Glycoprotein; Membrane; Reference proteome; Transmembrane; Transmembrane helix; Transport
SRR646596_TR26282_c0_g1_i2_len=1753	ACR11_ARATH	Q:442-1167,H:45-290	71.54%ID	E:6e-122	ACT domain-containing protein ACR11 {ECO:0000305}	.	0,037	0,515	0	1,144	0,907	0,061	0,324	0,306	0,003	0,707	ACR11_ARATH	ACT domain-containing protein ACR11 (Protein ACT DOMAIN REPEATS 11)	ACR11 At1g16880 F6I1.12	Arabidopsis thaliana (Mouse-ear cress)		Alternative splicing; Chloroplast; Complete proteome; Plastid; Reference proteome; Repeat; Transit peptide
SRR646596_TR26309_c0_g3_i1_len=2296	SAHH_CATRO	Q:473-1927,H:1-485	99.59%ID	E:0	Adenosylhomocysteinase	PF05221.14^AdoHcyase^S-adenosyl-L-homocysteine hydrolase^13-484^E:1e-145`PF00670.18^AdoHcyase_NAD^S-adenosyl-L-homocysteine hydrolase, NAD binding domain^240-403^E:1.2e-83`PF02826.16^2-Hacid_dh_C^D-isomer specific 2-hydroxyacid dehydrogenase, NAD binding domain^259-349^E:5.1e-09`PF07991.9^IlvN^Acetohydroxy acid isomeroreductase, NADPH-binding domain^260-325^E:5.4e-05	0,454	-0,165	0	1,161	0,609	0,134	0,619	-0,129	0,333	0,211	SAHH_CATRO	Adenosylhomocysteinase (AdoHcyase) (EC 3.3.1.1) (S-adenosyl-L-homocysteine hydrolase)	SAHH	Catharanthus roseus (Madagascar periwinkle) (Vinca rosea)	PATHWAY: Amino-acid biosynthesis; L-homocysteine biosynthesis; L-homocysteine from S-adenosyl-L-homocysteine: step 1/1.	Hydrolase; NAD; One-carbon metabolism; Stress response
[illegible]	PEX13_ARATH	Q:139-[illegible]	66.67%ID	E:1e-36	Peroxisomal membrane protein 13	[illegible]	[illegible]	[illegible]	0	[illegible]	[illegible]	[illegible]	[illegible]	[illegible]	[illegible]	[illegible]	PEX13_ARATH	Peroxisomal membrane protein 13 (Peroxin-13) (AtPEX13) (Peroxisome biogenesis protein 13)	[illegible]	[illegible]		Alternative splicing; Complete proteome; Membrane; Peroxisome; Peroxisome [illegible]

SRR646596_TR26498_c0_g1_i1_len=[illegible]	PEX13_ARATH	[illegible]513,H:180-303	[illegible]	[illegible]	[illegible]	.	[illegible]	[illegible]	[illegible]	[illegible]	[illegible]	[illegible]	[illegible]	[illegible]	[illegible]	[illegible] PEX13_ARATH	[illegible] (Pex13p) (Protein ABERRANT PEROXISOME MORPHOLOGY 2)	[illegible]	Arabidopsis thaliana (Mouse-ear cress)		[illegible] biogenesis; Protein transport; Reference proteome; Translocation; Transport
SRR646596_TR26527_c0_g2_i2_len=931	NA	NA	NA	NA	NA	.	0,175	0,942	0	2,208	0,017	2,36	0,001	2,794	1	-0,46	.	.	.	.	.	.
SRR646596_TR27458_c0_g1_i1_len=881	NA	NA	NA	NA	NA	PF05678.11^VQ^VQ motif^96-122^E:2.4e-12	0,18	0,775	0	1,574	0,004	1,958	0,004	1,985	0,328	0,473	.	.	.	.	.	.
SRR646596_TR27682_c0_g1_i2_len=2140	ODB2_ARATH	Q:1870-515,H:45-476	60.61%ID	E:0	Lipoamide acyltransferase component of branched-chain alpha-keto acid dehydrogenase complex, mitochondrial	PF00364.19^Biotin_lipoyl^Biotin-requiring enzyme^127-198^E:1.5e-16`PF02817.14^E3_binding^e3 binding domain^239-274^E:3.4e-16`PF00198.20^2-oxoacid_dh^2-oxoacid dehydrogenases acyltransferase (catalytic domain)^317-546^E:1.9e-77	0,23	0,464	0	1,367	0,005	1,606	0,007	1,547	1	-0,011	ODB2_ARATH	Lipoamide acyltransferase component of branched-chain alpha-keto acid dehydrogenase complex, mitochondrial (EC 2.3.1.168) (Branched-chain alpha-keto acid dehydrogenase complex component E2) (BCE2) (BCKAD-E2) (BCKADE2) (Dihydrolipoamide acetyltransferase component of branched-chain alpha-keto acid dehydrogenase complex) (Dihydrolipoamide branched chain transacylase) (Dihydrolipoyllysine-residue (2-methylpropanoyl)transferase) (Protein DARK INDUCIBLE 3)	BCE2 DIN3 LTA1 At3g06850 F3E22.1	Arabidopsis thaliana (Mouse-ear cress)		Acyltransferase; Complete proteome; Fatty acid biosynthesis; Fatty acid metabolism; Lipid biosynthesis; Lipid metabolism; Lipoyl; Mitochondrion; Reference proteome; Transferase; Transit peptide
SRR646596_TR28941_c0_g2_i4_len=2045	NA	NA	NA	NA	NA	PF05212.9^DUF707^Protein of unknown function (DUF707)^74-375^E:3.1e-132	0,471	0,598	0	2,728	0,043	2,5	0,003	3,125	0,189	-1,9	.	.	.	.	.	.
SRR646596_TR28962_c0_g1_i4_len=2457	C72A1_CATRO	Q:2338-770,H:1-523	99.24%ID	E:0	Secologanin synthase	PF00067.19^p450^Cytochrome P450^90-497^E:8e-88	0,269	0,442	0	5,087	1	0,121	0,534	-0,425	0,732	0,779	C72A1_CATRO	Secologanin synthase (SLS) (EC 1.3.3.9) (CYPLXXII) (Cytochrome P450 72A1)	CYP72A1 CYP72 P450CR3	Catharanthus roseus (Madagascar periwinkle) (Vinca rosea)	PATHWAY: Alkaloid biosynthesis; secologanin biosynthesis.	Alkaloid metabolism; Heme; Iron; Membrane; Metal-binding; Monooxygenase; NADP; Oxidoreductase; Transmembrane; Transmembrane helix; Vacuole
SRR646596_TR28962_c0_g1_i5_len=2543	C72A1_CATRO	Q:1606-770,H:245-523	97.49%ID	E:0	Secologanin synthase	PF00067.19^p450^Cytochrome P450^1-236^E:1.1e-63	0,034	0,826	0	3,226	0,059	1,192	0,257	0,831	0,129	2,867	C72A1_CATRO	Secologanin synthase (SLS) (EC 1.3.3.9) (CYPLXXII) (Cytochrome P450 72A1)	CYP72A1 CYP72 P450CR3	Catharanthus roseus (Madagascar periwinkle) (Vinca rosea)	PATHWAY: Alkaloid biosynthesis; secologanin biosynthesis.	Alkaloid metabolism; Heme; Iron; Membrane; Metal-binding; Monooxygenase; NADP; Oxidoreductase; Transmembrane; Transmembrane helix; Vacuole
SRR646596_TR29927_c0_g1_i1_len=1056	NA	NA	NA	NA	NA	PF00226.28^DnaJ^DnaJ domain^58-126^E:7.6e-19	0,031	1,299	0	1,632	0,001	1,757	0,043	1,233	1	-0,015	.	.	.	.	.	.
SRR646596_TR33051_c0_g1_i1_len=1104	PER25_ARATH	Q:1018-119,H:29-328	74.33%ID	E:3e-164	Peroxidase 25	PF00141.20^peroxidase^Peroxidase^45-286^E:4.9e-76	0,502	2,751	0	5,525	0,189	1,976	0,118	2,157	1	1,533	PER25_ARATH	Peroxidase 25 (Atperox P25) (EC 1.11.1.7)	PER25 P25 At2g41480 T26J13.7	Arabidopsis thaliana (Mouse-ear cress)		Calcium; Complete proteome; Disulfide bond; Glycoprotein; Heme; Hydrogen peroxide; Iron; Metal-binding; Oxidoreductase; Peroxidase; Reference proteome; Secreted; Signal
SRR646604_TR355_c0_g1_i1_len=1464	NA	NA	NA	NA	NA	PF04862.9^DUF642^Protein of unknown function (DUF642)^53-214^E:2e-66`PF04862.9^DUF642^Protein of unknown function (DUF642)^225-391^E:1e-17	1	-0,014	0	7,719	1	-1,927	1	-1,228	0,798	0,541	.	.	.	.	.	.
SRR646604_TR1619_c0_g1_i1_len=1903	PHT17_ARATH	Q:1902-727,H:130-507	78.32%ID	E:0	Probable inorganic phosphate transporter 1-7	PF00083.21^Sugar_tr^Sugar (and other) transporter^1-396^E:7.6e-37`PF07690.13^MFS_1^Major Facilitator Superfamily^1-338^E:1.4e-10	1	0,724	0	3,864	0,694	0,418	1	-0,082	0,76	0,612	PHT17_ARATH	Probable inorganic phosphate transporter 1-7 (AtPht1;7) (H(+)/Pi cotransporter)	PHT1-7 At3g54700 T5N23.60	Arabidopsis thaliana (Mouse-ear cress)		Complete proteome; Membrane; Phosphate transport; Phosphoprotein; Reference proteome; Symport; Transmembrane; Transmembrane helix; Transport
SRR646604_TR2491_c0_g1_i3_len=2295	MATE4_ARATH	Q:193-1863,H:5-559	63.88%ID	E:0	.	PF01554.15^MatE^MatE^131-285^E:5.6e-17`PF14667.3^Polysacc_synt_C^Polysaccharide biosynthesis C-terminal domain^247-384^E:5e-07	0,009	1,315	0	2,707	0,012	0,809	0,052	0,643	0,38	0,433	DTX46_ARATH	Protein DETOXIFICATION 46, chloroplastic (AtDTX46) (Multidrug and toxic compound extrusion protein 46) (MATE protein 46) (Protein EDS5 HOMOLOGUE)	DTX46 EDS5H At2g21340 F3K23.10	Arabidopsis thaliana (Mouse-ear cress)		Alternative splicing; Chloroplast; Complete proteome; Membrane; Plastid; Reference proteome; Transit peptide; Transmembrane; Transmembrane helix; Transport
SRR646604_TR8166_c0_g2_i1_len=2140	TCMO_CATRO	Q:235-1749,H:1-505	98.81%ID	E:0	Trans-cinnamate 4-monooxygenase	PF00067.19^p450^Cytochrome P450^34-495^E:3.7e-116	0,774	0,143	0	2,841	0,684	0,124	0,041	0,553	0,204	0,551	TCMO_CATRO	Trans-cinnamate 4-monooxygenase (EC 1.14.13.11) (Cinnamic acid 4-hydroxylase) (C4H) (CA4H) (Cytochrome P450 73) (Cytochrome P450C4H)	CYP73A4	Catharanthus roseus (Madagascar periwinkle) (Vinca rosea)	PATHWAY: Phenylpropanoid metabolism; trans-4-coumarate biosynthesis; trans-4-coumarate from trans-cinnamate: step 1/1.	Heme; Iron; Metal-binding; Monooxygenase; NADP; Oxidoreductase
SRR646604_TR8543_c0_g1_i2_len=2070	MGDG_TOBAC	Q:1682-498,H:6-413	71.15%ID	E:0	Probable monogalactosyldiacylglycerol synthase, chloroplastic	PF06925.8^MGDG_synth^Monogalactosyldiacylglycerol (MGDG) synthase^143-311^E:1.5e-59	0,126	0,619	0	2,655	0,083	0,587	0,005	0,901	1	0,061	MGDG_TOBAC	Probable monogalactosyldiacylglycerol synthase, chloroplastic (NtMGD1) (EC 2.4.1.46) (MGDG synthase type A)	MGD A	Nicotiana tabacum (Common tobacco)		Chloroplast; Glycosyltransferase; Membrane; Plastid; Transferase; Transit peptide
SRR646604_TR9361_c0_g1_i1_len=933	TCP10_ARATH	Q:566-787,H:14-87	91.89%ID	E:7e-40	Transcription factor TCP10	PF03634.10^TCP^TCP family transcription factor^28-132^E:1.7e-34	0,848	0,232	0	3,165	0,043	1,335	0,001	1,858	0,282	0,91	TCP10_ARATH	Transcription factor TCP10	TCP10 At2g31070 T16B12.12	Arabidopsis thaliana (Mouse-ear cress)		Complete proteome; DNA-binding; Developmental protein; Nucleus; Reference proteome; Transcription; Transcription regulation
SRR646604_TR11233_c0_g1_i2_len=1666	ACOX4_ARATH	Q:1540-620,H:1-307	79.15%ID	E:0	Acyl-coenzyme A oxidase 4, peroxisomal	PF02771.13^Acyl-CoA_dh_N^Acyl-CoA dehydrogenase, N-terminal domain^56-165^E:7.7e-24`PF02770.16^Acyl-CoA_dh_M^Acyl-CoA dehydrogenase, middle domain^171-263^E:6.5e-23	0,038	0,987	0	2,527	0,166	0,83	0,034	1,204	1	0,076	ACOX4_ARATH	Acyl-coenzyme A oxidase 4, peroxisomal (AOX 4) (EC 1.3.3.6) (G6p) (Short-chain acyl-CoA oxidase) (AtCX4) (AtG6) (SAOX)	ACX4 G6 At3g51840 ATEM1.9	Arabidopsis thaliana (Mouse-ear cress)		3D-structure; Acetylation; Complete proteome; FAD; Fatty acid metabolism; Flavoprotein; Glyoxysome; Lipid metabolism; Oxidoreductase; Peroxisome; Reference proteome
SRR646604_TR11484_c0_g2_i1_len=1111	RS141_MAIZE	Q:984-568,H:1-138	94.24%ID	E:4e-77	40S ribosomal protein S14	PF00411.16^Ribosomal_S11^Ribosomal protein S11^70-188^E:1.1e-47	0,502	-4,307	0	2,602	0,461	1,106	0,692	0,898	1	0,01	RS141_MAIZE	40S ribosomal protein S14 (Clone MCH1)		Zea mays (Maize)		Complete proteome; Reference proteome; Ribonucleoprotein; Ribosomal protein
SRR646604_TR12699_c0_g1_i3_len=2823	GGAP1_ARATH	Q:1935-715,H:1-403	74.69%ID	E:0	GDP-L-galactose phosphorylase 1	.	0,292	0,307	0	1,704	0,653	0,107	0,875	-0,042	0,001	0,842	GGAP1_ARATH	GDP-L-galactose phosphorylase 1 (EC 2.7.7.69) (Protein VITAMIN C DEFECTIVE 2)	VTC2 At4g26850 F10M23.190	Arabidopsis thaliana (Mouse-ear cress)	PATHWAY: Cofactor biosynthesis; L-ascorbate biosynthesis via GDP-alpha-D-mannose pathway; L-ascorbate from GDP-alpha-D-mannose: step 2/5.	Ascorbate biosynthesis; Complete proteome; Cytoplasm; Guanine-nucleotide releasing factor; Hydrolase; Nucleotide-binding; Nucleotidyltransferase; Nucleus; Reference proteome; Transferase
SRR646604_TR17922_c0_g1_i1_len=3216	SERB2_ARATH	Q:1676-2902,H:18-421	79.81%ID	E:0	Phosphoserine aminotransferase 2, chloroplastic	.	0,568	0,278	0	2,25	0,748	0,265	0,87	0,162	0,431	0,331	SERB2_ARATH	Phosphoserine aminotransferase 2, chloroplastic (AtPSAT2) (EC 2.6.1.52)	PSAT2 At2g17630 T19E12.3	Arabidopsis thaliana (Mouse-ear cress)	PATHWAY: Amino-acid biosynthesis; L-serine biosynthesis; L-serine from 3-phospho-D-glycerate: step 2/3.	Acetylation; Amino-acid biosynthesis; Aminotransferase; Chloroplast; Complete proteome; Plastid; Pyridoxal phosphate; Reference proteome; Serine biosynthesis; Transferase; Transit peptide
SRR646604_TR19937_c0_g3_i1_len=4044	YSL7_ARATH	Q:3621-1543,H:1-687	77.06%ID	E:0	Probable metal-nicotianamine transporter YSL7	PF03169.12^OPT^OPT oligopeptide transporter protein^60-680^E:1.1e-152	0,129	1,098	0	3,304	0,044	2,291	0,692	1,018	0,732	-0,516	YSL7_ARATH	Probable metal-nicotianamine transporter YSL7 (Protein YELLOW STRIPE LIKE 7) (AtYSL7)	YSL7 At1g65730 F1E22.10	Arabidopsis thaliana (Mouse-ear cress)		Complete proteome; Membrane; Reference proteome; Transmembrane; Transmembrane helix; Transport
SRR646604_TR21373_c0_g1_i1_len=1971	NA	NA	NA	NA	NA	PF07250.8^Glyoxal_oxid_N^Glyoxal oxidase N-terminus^40-280^E:1.5e-100`PF09118.8^DUF1929^Domain of unknown function (DUF1929)^432-534^E:8e-25	0,643	0,477	0	3,292	0,76	0,532	1	0,404	1	0,032	.	.	.	.	.	.
SRR646604_TR23664_c0_g1_i1_len=812	NA	NA	NA	NA	NA	.	0,581	0,47	0	3,4	0,073	1,889	0,002	2,656	0,337	-0,466	.	.	.	.	.	.
SRR646604_TR27892_c0_g1_i7_len=4401	NES1_FRAAN	Q:4081-2468,H:14-515	50.74%ID	E:1e-157	(3S,6E)-nerolidol synthase 1	PF01535.17^PPR^PPR repeat^160-185^E:0.00039`PF17177.1^PPR_long^Pentacotripeptide-repeat region of PRORP^167-276^E:1.1e-07`PF13812.3^PPR_3^Pentatricopeptide repeat domain^175-233^E:2.8e-09`PF01535.17^PPR^PPR repeat^191-217^E:0.09`PF12854.4^PPR_1^PPR repeat^221-252^E:1.8e-12`PF13041.3^PPR_2^PPR repeat family^223-270^E:4.9e-16`PF01535.17^PPR^PPR repeat^226-256^E:6e-07`PF13812.3^PPR_3^Pentatricopeptide repeat domain^247-302^E:1.8e-07`PF12854.4^PPR_1^PPR repeat^255-287^E:8.9e-06`PF13041.3^PPR_2^PPR repeat family^258-305^E:8.8e-08`PF01535.17^PPR^PPR repeat^261-290^E:0.0007`PF13041.3^PPR_2^PPR repeat family^294-340^E:9.6e-11`PF01535.17^PPR^PPR repeat^297-326^E:0.0036`PF13812.3^PPR_3^Pentatricopeptide repeat domain^317-371^E:4e-11`PF13041.3^PPR_2^PPR repeat family^329-373^E:3.9e-11`PF01535.17^PPR^PPR repeat^331-361^E:1.8e-06	0,066	5,324	0,001	4,77	1	3,141	0,066	5,477	1	2,102	NES1_FRAAN	(3S,6E)-nerolidol synthase 1 (FaNES1) (EC 4.2.3.48)		Fragaria ananassa (Strawberry)	PATHWAY: Secondary metabolite biosynthesis; terpenoid biosynthesis.	Cytoplasm; Lyase; Magnesium; Manganese; Metal-binding
SRR646604_TR27892_c0_g1_i8_len=4323	NES1_FRAAN	Q:4003-2468,H:14-515	53.32%ID	E:2e-161	(3S,6E)-nerolidol synthase 1	PF01397.18^Terpene_synth^Terpene synthase, N-terminal domain^67-224^E:7.8e-42`PF03936.13^Terpene_synth_C^Terpene synthase family, metal binding domain^258-528^E:1.4e-72	0,003	3,075	0	4,541	1	2,464	1	0	1	0,954	NES1_FRAAN	(3S,6E)-nerolidol synthase 1 (FaNES1) (EC 4.2.3.48)		Fragaria ananassa (Strawberry)	PATHWAY: Secondary metabolite biosynthesis; terpenoid biosynthesis.	Cytoplasm; Lyase; Magnesium; Manganese; Metal-binding
SRR646604_TR28367_c0_g1_i1_len=2132	G6PD_SOLTU	Q:471-1868,H:46-511	89.7%ID	E:0	Glucose-6-phosphate 1-dehydrogenase, cytoplasmic isoform	PF00479.19^G6PD_N^Glucose-6-phosphate dehydrogenase, NAD binding domain^6-61^E:2.6e-23`PF02781.13^G6PD_C^Glucose-6-phosphate dehydrogenase, C-terminal domain^63-342^E:9e-116	0,594	0,258	0	1,957	0,183	0,659	0,6	0,273	0,389	0,38	G6PD_SOLTU	Glucose-6-phosphate 1-dehydrogenase, cytoplasmic isoform (G6PD) (EC 1.1.1.49)	G6PDH	Solanum tuberosum (Potato)	PATHWAY: Carbohydrate degradation; pentose phosphate pathway; D-ribulose 5-phosphate from D-glucose 6-phosphate (oxidative stage): step 1/3.	Carbohydrate metabolism; Complete proteome; Cytoplasm; Glucose metabolism; NADP; Oxidoreductase; Reference proteome
SRR646604_TR28486_c1_g1_i1_len=1134	NA	NA	NA	NA	NA	PF07223.8^DUF1421^Protein of unknown function (DUF1421)^5-206^E:2.8e-51	0,429	0,52	0	1,868	0,086	0,85	0,163	0,715	1	-0,079	.	.	.	.	.	.
SRR646604_TR32269_c0_g1_i1_len=1949	UFOG6_FRAAN	Q:1611-220,H:6-475	55.77%ID	E:7e-177	UDP-glucose flavonoid 3-O-glucosyltransferase 6 {ECO:0000303|PubMed:18487633}	PF00201.15^UDPGT^UDP-glucoronosyl and UDP-glucosyl transferase^268-395^E:6.3e-23	1	1,674	0	6,591	0,129	3,523	0,502	2,376	1	1,211	UFOG6_FRAAN	UDP-glucose flavonoid 3-O-glucosyltransferase 6 (EC 2.4.1.91) (Flavonol 3-O-glucosyltransferase 6) (FaGT6)	GT6	Fragaria ananassa (Strawberry)		Glycosyltransferase; Transferase
SRR646604_TR32970_c2_g1_i3_len=3560	HMDH_CATRO	Q:3425-2199,H:1-407	98.29%ID	E:0	3-hydroxy-3-methylglutaryl-coenzyme A reductase	PF00368.15^HMG-CoA_red^Hydroxymethylglutaryl-coenzyme A reductase^254-446^E:2.4e-64	0,352	0,866	0,001	3,229	0,077	1,023	0,505	0,521	1	0,337	HMDH_CATRO	3-hydroxy-3-methylglutaryl-coenzyme A reductase (HMG-CoA reductase) (EC 1.1.1.34)	HMGR	Catharanthus roseus (Madagascar periwinkle) (Vinca rosea)	PATHWAY: Metabolic intermediate biosynthesis; (R)-mevalonate biosynthesis; (R)-mevalonate from acetyl-CoA: step 3/3.	Endoplasmic reticulum; Glycoprotein; Isoprene biosynthesis; Membrane; NADP; Oxidoreductase; Transmembrane; Transmembrane helix
SRR646604_TR34575_c0_g2_i1_len=1237	NA	NA	NA	NA	NA	PF14364.3^DUF4408^Domain of unknown function (DUF4408)^10-41^E:2.2e-06`PF05553.8^DUF761^Cotton fibre expressed protein^180-215^E:2.9e-18	0,16	1,126	0,001	3,858	0,003	2,042	0,228	1,016	1	-1,707	.	.	.	.	.	.
SRR646604_TR34583_c0_g1_i4_len=3032	SUSY_SOLLC	Q:2812-398,H:1-805	91.06%ID	E:0	Sucrose synthase	PF00862.16^Sucrose_synth^Sucrose synthase^8-553^E:1e-282`PF00534.17^Glycos_transf_1^Glycosyl transferases group 1^564-737^E:2.5e-33`PF13692.3^Glyco_trans_1_4^Glycosyl transferases group 1^571-724^E:7.2e-11	0,325	0,407	0	2,913	0,91	-0,062	0,785	0,094	1	0,717	SUSY_SOLLC	Sucrose synthase (EC 2.4.1.13) (Sucrose-UDP glucosyltransferase)		Solanum lycopersicum (Tomato) (Lycopersicon esculentum)		Complete proteome; Glycosyltransferase; Reference proteome; Transferase
SRR646604_TR34878_c0_g1_i1_len=1243	CSPL7_SOYBN	Q:198-680,H:16-173	56.17%ID	E:5e-42	CASP-like protein 1D2	PF04535.9^DUF588^Domain of unknown function (DUF588)^42-190^E:1.7e-39	0,066	4,543	0	5,86	0,129	5,152	0,502	3,733	1	-2,595	CSPL7_SOYBN	CASP-like protein 1D2 (GmCASPL1D2)		Glycine max (Soybean) (Glycine hispida)		Cell membrane; Complete proteome; Glycoprotein; Membrane; Reference proteome; Transmembrane; Transmembrane helix
SRR648705_TR567_c0_g1_i1_len=1422	NA	NA	NA	NA	NA	PF00076.19^RRM_1^RNA recognition motif. (a.k.a. RRM, RBD, or RNP domain)^7-66^E:1.4e-08	0,493	0,34	0,001	1,446	0,056	0,954	0,005	1,326	1	-0,159	.	.	.	.	.	.
SRR648705_TR1034_c0_g3_i1_len=1629	MTDH_FRAAN	Q:1418-357,H:4-357	67.8%ID	E:8e-152	Probable mannitol dehydrogenase	PF08240.9^ADH_N^Alcohol dehydrogenase GroES-like domain^39-151^E:6.3e-28`PF00107.23^ADH_zinc_N^Zinc-binding dehydrogenase^195-317^E:2e-18	0,454	-0,407	0	2,99	0,016	2,376	0,044	2,219	0,692	1,001	MTDH_FRAAN	Probable mannitol dehydrogenase (EC 1.1.1.255) (NAD-dependent mannitol dehydrogenase)	CAD	Fragaria ananassa (Strawberry)		Metal-binding; NAD; Oxidoreductase; Zinc
SRR648705_TR3034_c0_g1_i3_len=4755	SERA1_ARATH	Q:399-2066,H:42-603	84.52%ID	E:0	D-3-phosphoglycerate dehydrogenase 1, chloroplastic	PF00389.27^2-Hacid_dh^D-isomer specific 2-hydroxyacid dehydrogenase, catalytic domain^60-369^E:2e-34`PF02826.16^2-Hacid_dh_C^D-isomer specific 2-hydroxyacid dehydrogenase, NAD binding domain^162-337^E:1e-62`PF00670.18^AdoHcyase_NAD^S-adenosyl-L-homocysteine hydrolase, NAD binding domain^192-301^E:2e-06`PF07991.9^IlvN^Acetohydroxy acid isomeroreductase, NADPH-binding domain^194-297^E:1.6e-06`PF01842.22^ACT^ACT domain^528-588^E:5.5e-07	0,756	-0,242	0	2,15	0,505	-0,445	0,666	0,303	0,394	0,476	SERA1_ARATH	D-3-phosphoglycerate dehydrogenase 1, chloroplastic (EC 1.1.1.95) (Protein EMBRYO SAC DEVELOPMENT ARREST 9)	PGDH1 EDA9 At4g34200 F10M10.7	Arabidopsis thaliana (Mouse-ear cress)	PATHWAY: Amino-acid biosynthesis; L-serine biosynthesis; L-serine from 3-phospho-D-glycerate: step 1/3.	Chloroplast; Complete proteome; NAD; Oxidoreductase; Plastid; Reference proteome; Transit peptide
SRR648705_TR4584_c0_g2_i1_len=1742	MYB44_ARATH	Q:408-719,H:3-106	80.77%ID	E:3e-51	Transcription factor MYB44	PF00249.28^Myb_DNA-binding^Myb-like DNA-binding domain^39-84^E:7.9e-19`PF13921.3^Myb_DNA-bind_6^Myb-like DNA-binding domain^42-100^E:1.4e-17`PF00249.28^Myb_DNA-binding^Myb-like DNA-binding domain^93-135^E:3.4e-14`PF13921.3^Myb_DNA-bind_6^Myb-like DNA-binding domain^94-152^E:3.4e-09	0,004	3,104	0	4,038	0,002	5,601	0,017	5,201	1	-0,19	MYB44_ARATH	Transcription factor MYB44 (Myb-related protein 44) (AtMYB44) (Myb-related protein R1) (AtMYBR1)	MYB44 MYBR1 At5g67300 K8K14.2	Arabidopsis thaliana (Mouse-ear cress)		Abscisic acid signaling pathway; Complete proteome; DNA-binding; Nucleus; Reference proteome; Repeat; Transcription; Transcription regulation
SRR648705_TR4794_c0_g1_i4_len=1868	AGD9_ARATH	Q:1542-337,H:1-402	67.64%ID	E:2e-178	Probable ADP-ribosylation factor GTPase-activating protein AGD9	PF01412.15^ArfGap^Putative GTPase activating protein for Arf^12-113^E:8.4e-35	1	0,044	0	1,597	0,102	0,716	0,085	0,743	0,707	-0,3	AGD9_ARATH	Probable ADP-ribosylation factor GTPase-activating protein AGD9 (ARF GAP AGD9) (Protein ARF-GAP DOMAIN 9) (AtAGD9)	AGD9 At5g46750 MZA15.17	Arabidopsis thaliana (Mouse-ear cress)		Acetylation; Complete proteome; GTPase activation; Metal-binding; Phosphoprotein; Reference proteome; Zinc; Zinc-finger
SRR648705_TR4868_c0_g1_i1_len=2051	NA	NA	NA	NA	NA	PF12146.5^Hydrolase_4^Serine aminopeptidase, S33^125-366^E:4.8e-10`PF00561.17^Abhydrolase_1^alpha/beta hydrolase fold^129-239^E:1.6e-10`PF12697.4^Abhydrolase_6^Alpha/beta hydrolase family^130-377^E:2.5e-11	0,865	-0,135	0	3,357	0,593	-0,713	0,798	-0,48	1	0,016	.	.	.	.	.	.
SRR648705_TR6257_c0_g2_i1_len=1567	NIFU2_ARATH	Q:1164-508,H:7-225	68.78%ID	E:3e-88	NifU-like protein 2, chloroplastic	PF01106.14^NifU^NifU-like domain^95-160^E:1.1e-25`PF01106.14^NifU^NifU-like domain^176-230^E:1.5e-10	0,661	0,263	0	1,465	0,095	0,72	0,095	0,724	1	-0,122	NIFU2_ARATH	NifU-like protein 2, chloroplastic (AtCNfu2) (AtCnfU-V)	NIFU2 CNFU2 NFU2 At5g49940 K9P8.16	Arabidopsis thaliana (Mouse-ear cress)		2Fe-2S; 3D-structure; Alternative splicing; Chloroplast; Complete proteome; Disulfide bond; Iron; Iron-sulfur; Metal-binding; Plastid; Reference proteome; Transit peptide
SRR648705_TR7060_c0_g2_i1_len=6120	TPL_ARATH	Q:222-3548,H:1-1109	82.69%ID	E:0	Protein TOPLESS	PF00400.29^WD40^WD domain, G-beta repeat^456-490^E:1.8e-05`PF00400.29^WD40^WD domain, G-beta repeat^915-948^E:0.0081	0,568	0,251	0	3,571	1	0,06	1	0,327	0,34	0,535	TPL_ARATH	Protein TOPLESS (WUS-interacting protein 1)	TPL WSIP1 At1g15750 F7H2.9	Arabidopsis thaliana (Mouse-ear cress)		Complete proteome; Developmental protein; Jasmonic acid signaling pathway; Nucleus; Phosphoprotein; Reference proteome; Repeat; Repressor; Transcription; Transcription regulation; WD repeat
SRR648705_TR7900_c0_g1_i3_len=1553	ERGI3_DANRE	Q:314-1312,H:4-334	47.38%ID	E:8e-107	Endoplasmic reticulum-Golgi intermediate compartment protein 3	PF13850.3^ERGIC_N^Endoplasmic Reticulum-Golgi Intermediate Compartment (ERGIC)^8-97^E:1.1e-31`PF07970.9^COPIIcoated_ERV^Endoplasmic reticulum vesicle transporter^149-336^E:8.2e-69	0,258	0,928	0,001	3,786	0,286	0,827	0,811	0,182	0,152	0,875	ERGI3_DANRE	Endoplasmic reticulum-Golgi intermediate compartment protein 3	ergic3 zgc:113959 zgc:55762	Danio rerio (Zebrafish) (Brachydanio rerio)		Alternative splicing; Complete proteome; ER-Golgi transport; Endoplasmic reticulum; Golgi apparatus; Membrane; Reference proteome; Transmembrane; Transmembrane helix; Transport
SRR648705_TR8110_c0_g1_i1_len=1031	IAA17_ARATH	Q:245-820,H:3-218	51.15%ID	E:1e-59	Auxin-responsive protein IAA17	PF02309.13^AUX_IAA^AUX/IAA family^31-202^E:6.3e-59	1	0,038	0	7,058	0	-7,593	0	-9,345	0,786	0,1	IAA17_ARATH	Auxin-responsive protein IAA17 (Auxin response 3) (Indoleacetic acid-induced protein 17)	IAA17 AXR3 At1g04250 F19P19.31	Arabidopsis thaliana (Mouse-ear cress)		3D-structure; Auxin signaling pathway; Complete proteome; Nucleus; Phosphoprotein; Reference proteome; Repressor; Transcription; Transcription regulation
SRR648705_TR8125_c0_g2_i2_len=2472	2AAB_ARATH	Q:363-2123,H:1-587	89.44%ID	E:0	Serine/threonine-protein phosphatase 2A 65 kDa regulatory subunit A beta isoform	PF12755.4^Vac14_Fab1_bd^Vacuolar 14 Fab1-binding region^235-300^E:1.7e-05`PF02985.19^HEAT^HEAT repeat^242-270^E:0.0008`PF13646.3^HEAT_2^HEAT repeats^361-459^E:7.1e-11	0,547	0,335	0	1,833	0,777	0,26	0,777	0,204	0,419	0,391	2AAB_ARATH	Serine/threonine-protein phosphatase 2A 65 kDa regulatory subunit A beta isoform (AtA beta) (PP2A, subunit A, beta isoform)	PP2AA2 DF1 At3g25800 K13N2.14	Arabidopsis thaliana (Mouse-ear cress)		Acetylation; Alternative splicing; Complete proteome; Reference proteome; Repeat
SRR648705_TR8541_c2_g1_i3_len=1354	RSSA_DAUCA	Q:163-978,H:1-269	76.92%ID	E:2e-138	40S ribosomal protein SA {ECO:0000255|HAMAP-Rule:MF_03015}	PF00318.17^Ribosomal_S2^Ribosomal protein S2^18-112^E:1.4e-12`PF00318.17^Ribosomal_S2^Ribosomal protein S2^117-182^E:1.9e-12	0	-1,173	0	10,892	1	0	1	0	0,353	-0,229	RSSA_DAUCA	40S ribosomal protein SA (p40)	179B	Daucus carota (Wild carrot)		Cytoplasm; Ribonucleoprotein; Ribosomal protein
SRR648705_TR9327_c0_g1_i1_len=2293	MLO6_ARATH	Q:1732-257,H:1-539	44.22%ID	E:4e-144	MLO-like protein 6	PF03094.12^Mlo^Mlo family^11-107^E:4e-38`PF03094.12^Mlo^Mlo family^105-440^E:1.2e-141	0,517	1,091	0	4,131	0,073	2,338	0,027	2,538	1	-0,188	MLO6_ARATH	MLO-like protein 6 (AtMlo6)	MLO6 At1g61560 T25B24.9	Arabidopsis thaliana (Mouse-ear cress)		Alternative splicing; Calmodulin-binding; Complete proteome; Membrane; Pathogenesis-related protein; Plant defense; Reference proteome; Transmembrane; Transmembrane helix
SRR648705_TR9782_c1_g4_i1_len=2063	WTR18_ARATH	Q:816-1886,H:14-365	60.55%ID	E:1e-121	WAT1-related protein At3g28050	PF00892.17^EamA^EamA-like transporter family^20-151^E:2e-08`PF00892.17^EamA^EamA-like transporter family^205-328^E:8.3e-08	0,025	1,887	0	4,117	0,025	2,634	0,076	2,191	0,629	-1,428	WTR18_ARATH	WAT1-related protein At3g28050	At3g28050 MMG15.6	Arabidopsis thaliana (Mouse-ear cress)		Complete proteome; Membrane; Reference proteome; Repeat; Transmembrane; Transmembrane helix
SRR648705_TR10167_c1_g1_i1_len=1637	MOMT_CATRO	Q:1318-275,H:1-348	100%ID	E:0	Myricetin O-methyltransferase	PF08100.8^Dimerisation^Dimerisation domain^27-74^E:6.1e-19`PF00891.15^Methyltransf_2^O-methyltransferase^117-334^E:5.2e-58	0,341	0,402	0	2,197	0,01	1,436	0,093	1,059	0,798	-0,412	MOMT_CATRO	Myricetin O-methyltransferase (EC 2.1.1.267) (CrOMT2) (Flavonoid O-methyltransferase)		Catharanthus roseus (Madagascar periwinkle) (Vinca rosea)		Acetylation; Direct protein sequencing; Methyltransferase; S-adenosyl-L-methionine; Transferase
SRR648705_TR10690_c0_g2_i2_len=1030	NA	NA	NA	NA	NA	PF14368.3^LTP_2^Probable lipid transfer^13-104^E:1.8e-19	1	0,224	0	3,362	0,076	2,373	0,076	2,313	0,822	-0,421	.	.	.	.	.	.
SRR648705_TR11339_c0_g2_i1_len=2032	PAT_PETHY	Q:482-1711,H:70-479	89.02%ID	E:0	Bifunctional aspartate aminotransferase and glutamate/aspartate-prephenate aminotransferase	PF00155.18^Aminotran_1_2^Aminotransferase class I and II^195-557^E:7.1e-71	0,68	0,185	0	1,838	0,108	0,673	0,187	0,594	0,58	0,26	PAT_PETHY	Bifunctional aspartate aminotransferase and glutamate/aspartate-prephenate aminotransferase (PhPPA-AT) (EC 2.6.1.1) (EC 2.6.1.78) (EC 2.6.1.79)		Petunia hybrida (Petunia)	PATHWAY: Amino-acid biosynthesis; L-phenylalanine biosynthesis; L-arogenate from prephenate (L-Asp route): step 1/1.; PATHWAY: Amino-acid biosynthesis; L-phenylalanine biosynthesis; L-arogenate from prephenate (L-Glu route): step 1/1.	Amino-acid biosynthesis; Aminotransferase; Chloroplast; Plastid; Pyridoxal phosphate; Transferase; Transit peptide
SRR648705_TR11458_c2_g2_i2_len=1353	NA	NA	NA	NA	NA	.	0,002	-6,299	0	7,041	1	0	1	3,582	0,111	-0,499	.	.	.	.	.	.
SRR648705_TR12335_c0_g1_i2_len=2429	KPYC_SOLTU	Q:1935-406,H:1-510	90.78%ID	E:0	Pyruvate kinase, cytosolic isozyme	PF00224.18^PK^Pyruvate kinase, barrel domain^19-362^E:2.7e-159`PF02887.13^PK_C^Pyruvate kinase, alpha/beta domain^380-504^E:1.4e-25	0,566	0,197	0,001	1,22	0,004	1,156	0,009	1,056	0,684	0,172	KPYC_SOLTU	Pyruvate kinase, cytosolic isozyme (PK) (EC 2.7.1.40)		Solanum tuberosum (Potato)	PATHWAY: Carbohydrate degradation; glycolysis; pyruvate from D-glyceraldehyde 3-phosphate: step 5/5.	ATP-binding; Complete proteome; Cytoplasm; Glycolysis; Kinase; Magnesium; Metal-binding; Nucleotide-binding; Potassium; Pyruvate; Reference proteome; Transferase
SRR648705_TR13569_c0_g2_i6_len=2015	EF1A_TOBAC	Q:1791-484,H:1-436	97.94%ID	E:0	Elongation factor 1-alpha	PF00009.24^GTP_EFTU^Elongation factor Tu GTP binding domain^6-221^E:1e-52`PF03144.22^GTP_EFTU_D2^Elongation factor Tu domain 2^248-313^E:7.3e-15`PF03143.14^GTP_EFTU_D3^Elongation factor Tu C-terminal domain^322-429^E:3.9e-38	0,002	-0,75	0	1,406	0,676	0,123	0,932	0,04	0,066	-4,445	EF1A_TOBAC	Elongation factor 1-alpha (EF-1-alpha) (Vitronectin-like adhesion protein 1) (PVN1)		Nicotiana tabacum (Common tobacco)		Cytoplasm; Elongation factor; GTP-binding; Nucleotide-binding; Phosphoprotein; Protein biosynthesis
SRR648705_TR13752_c1_g1_i1_len=2469	HEMH_CUCSA	Q:328-1812,H:1-491	68.01%ID	E:0	Ferrochelatase-2, chloroplastic	PF00762.16^Ferrochelatase^Ferrochelatase^116-439^E:1.5e-109	0,2	0,655	0	2,29	0,109	1,135	0,257	0,841	0,623	0,299	HEMH_CUCSA	Ferrochelatase-2, chloroplastic (EC 4.99.1.1) (Ferrochelatase II) (Heme synthase 2) (Protoheme ferro-lyase 2)	HEMH	Cucumis sativus (Cucumber)	PATHWAY: Porphyrin-containing compound metabolism; protoheme biosynthesis; protoheme from protoporphyrin-IX: step 1/1.	Chloroplast; Heme biosynthesis; Iron; Lyase; Plastid; Porphyrin biosynthesis; Transit peptide
SRR648705_TR14391_c0_g2_i9_len=2536	NEK6_ARATH	Q:309-1139,H:1-276	75.45%ID	E:1e-134	Serine/threonine-protein kinase Nek6	PF00069.22^Pkinase^Protein kinase domain^15-268^E:1.7e-67`PF07714.14^Pkinase_Tyr^Protein tyrosine kinase^16-265^E:2.8e-42	0,132	0,751	0	2,407	0,147	1,028	0,257	0,847	0,906	-0,121	NEK6_ARATH	Serine/threonine-protein kinase Nek6 (EC 2.7.11.1) (NimA-related protein kinase 6) (AtNek6)	NEK6 At3g20860 MOE17.17	Arabidopsis thaliana (Mouse-ear cress)		ATP-binding; Complete proteome; Kinase; Nucleotide-binding; Reference proteome; Serine/threonine-protein kinase; Transferase
SRR648705_TR14643_c0_g1_i1_len=571	NA	NA	NA	NA	NA	.	0,623	0,308	0	2,452	0,195	1,326	0,003	2,361	0,781	-0,352	.	.	.	.	.	.
SRR648705_TR15040_c0_g1_i1_len=3233	SDL5A_SOYBN	Q:531-2357,H:1-610	85.25%ID	E:0	Dynamin-related protein 5A	PF00350.20^Dynamin_N^Dynamin family^37-212^E:1.9e-54`PF01031.17^Dynamin_M^Dynamin central region^221-489^E:1.8e-65`PF02212.15^GED^Dynamin GTPase effector domain^513-605^E:5.5e-23	1	0,045	0,001	1,852	0,683	0,269	0,591	0,351	0,705	0,275	SDL5A_SOYBN	Dynamin-related protein 5A (Soybean dynamin-like protein 5A) (SDL5A)		Glycine max (Soybean) (Glycine hispida)		Cell cycle; Cell division; Complete proteome; Cytoplasm; Cytoskeleton; GTP-binding; Hydrolase; Microtubule; Motor protein; Nucleotide-binding; Reference proteome
SRR648705_TR17092_c0_g4_i1_len=1630	ADK2_ARATH	Q:1481-456,H:3-344	80.99%ID	E:0	Adenosine kinase 2	PF00294.21^PfkB^pfkB family carbohydrate kinase^26-335^E:3.3e-75	0,338	-0,223	0	1,036	0,321	-0,249	0,65	-0,117	0,848	-0,174	ADK2_ARATH	Adenosine kinase 2 (AK 2) (EC 2.7.1.20) (Adenosine 5'-phosphotransferase 2)	ADK2 At5g03300 F12E4.30 MOK16.21	Arabidopsis thaliana (Mouse-ear cress)	PATHWAY: Purine metabolism; AMP biosynthesis via salvage pathway; AMP from adenosine: step 1/1.	ATP-binding; Complete proteome; Host-virus interaction; Kinase; Magnesium; Nucleotide-binding; Purine salvage; Reference proteome; Transferase
SRR648705_TR17142_c0_g2_i1_len=4604	PAP14_ARATH	Q:648-2441,H:72-666	66.94%ID	E:0	Probable plastid-lipid-associated protein 14, chloroplastic	PF04755.9^PAP_fibrillin^PAP_fibrillin^444-564^E:4.7e-05	0,073	2,372	0	7,007	0,502	4,11	0,254	4,457	0,001	6,532	PAP14_ARATH	Probable plastid-lipid-associated protein 14, chloroplastic (AtPap14) (Fibrillin-11) (OBP3-responsive protein 1)	PAP14 FBN11 FIB11 ORG1 At5g53450	Arabidopsis thaliana (Mouse-ear cress)		Alternative splicing; Chloroplast; Complete proteome; Plastid; Reference proteome; Transit peptide
SRR648705_TR17281_c0_g1_i2_len=2445	RD21A_ARATH	Q:2176-923,H:42-458	63.18%ID	E:2e-171	Cysteine proteinase RD21a	PF08246.9^Inhibitor_I29^Cathepsin propeptide inhibitor domain (I29)^38-95^E:2.5e-15`PF00112.20^Peptidase_C1^Papain family cysteine protease^126-341^E:3.9e-80`PF00396.15^Granulin^Granulin^375-421^E:1.1e-11	0,076	2,82	0,001	5,051	0,502	2,73	0,502	2,605	0,84	0,308	RD21A_ARATH	Cysteine proteinase RD21a (RD21) (EC 3.4.22.-)	RD21A At1g47128 F2G19.31	Arabidopsis thaliana (Mouse-ear cress)		Complete proteome; Disulfide bond; Glycoprotein; Hydrolase; Protease; Reference proteome; Signal; Thiol protease; Zymogen
SRR648705_TR18288_c0_g1_i1_len=1412	NA	NA	NA	NA	NA	PF14144.3^DOG1^Seed dormancy control^28-101^E:1.3e-26	0,129	3,37	0	7,5	0,076	2,42	0,381	1,534	1	-1,296	.	.	.	.	.	.
SRR648705_TR18930_c0_g1_i2_len=1868	NAC8_ARATH	Q:1625-360,H:1-447	57.05%ID	E:2e-163	NAC domain-containing protein 8	PF02365.12^NAM^No apical meristem (NAM) protein^59-197^E:1.2e-13	0,429	0,511	0,001	2,354	0,068	1,192	0,129	0,978	1	0,006	NAC8_ARATH	NAC domain-containing protein 8 (ANAC008)	NAC008 At1g25580 F14G11.2 F2J7.1	Arabidopsis thaliana (Mouse-ear cress)		Complete proteome; DNA-binding; Nucleus; Reference proteome; Transcription; Transcription regulation
SRR648705_TR20783_c0_g1_i3_len=4009	ADS3_ARATH	Q:915-517,H:54-175	54.48%ID	E:1e-61	Palmitoyl-monogalactosyldiacylglycerol delta-7 desaturase, chloroplastic {ECO:0000303|PubMed:15579662}	.	0,031	0,837	0	4,529	0,629	-1,264	0,629	-0,995	0,401	0,24	ADS3_ARATH	Palmitoyl-monogalactosyldiacylglycerol delta-7 desaturase, chloroplastic (EC 1.14.19.-) (FAD5) (Monogalactosyldiacylglycerol-specific palmitic acid desaturase)	ADS3 At3g15850 MSJ11.25	Arabidopsis thaliana (Mouse-ear cress)	PATHWAY: Lipid metabolism; oxylipin biosynthesis.; PATHWAY: Lipid metabolism; polyunsaturated fatty acid biosynthesis.	Chloroplast; Complete proteome; Fatty acid biosynthesis; Fatty acid metabolism; Iron; Lipid biosynthesis; Lipid metabolism; Membrane; Oxidoreductase; Oxylipin biosynthesis; Plastid; Reference proteome; Transit peptide; Transmembrane; Transmembrane helix
SRR648705_TR21250_c0_g1_i2_len=778	ARF2_ORYSJ	Q:205-747,H:1-181	98.9%ID	E:4e-130	ADP-ribosylation factor 2	PF00025.18^Arf^ADP-ribosylation factor family^5-177^E:3e-80`PF09439.7^SRPRB^Signal recognition particle receptor beta subunit^16-143^E:8.5e-14`PF08477.10^Roc^Ras of Complex, Roc, domain of DAPkinase^19-129^E:1.1e-13`PF04670.9^Gtr1_RagA^Gtr1/RagA G protein conserved region^19-142^E:2.5e-10`PF01926.20^MMR_HSR1^50S ribosome-binding GTPase^19-122^E:4.7e-05`PF00071.19^Ras^Ras family^20-172^E:1e-12	0,65	-0,113	0	1,582	0,619	0,122	0,473	-0,176	0,073	0,436	ARF2_ORYSJ	ADP-ribosylation factor 2	ARF Os05g0489600 LOC_Os05g41060 OJ1119_H02.17	Oryza sativa subsp. japonica (Rice)		Complete proteome; ER-Golgi transport; GTP-binding; Golgi apparatus; Lipoprotein; Myristate; Nucleotide-binding; Protein transport; Reference proteome; Transport
SRR648705_TR21900_c1_g1_i1_len=974	NA	NA	NA	NA	NA	.	0,006	-1,023	0	6,728	1	0	1	0	0,203	-0,515	.	.	.	.	.	.
SRR648705_TR22018_c0_g1_i1_len=2076	CAAT1_ARATH	Q:1826-117,H:3-579	72.27%ID	E:0	Cationic amino acid transporter 1	PF13520.3^AA_permease_2^Amino acid permease^5-406^E:8.1e-44`PF00324.18^AA_permease^Amino acid permease^9-374^E:1.3e-31`PF13906.3^AA_permease_C^C-terminus of AA_permease^444-494^E:1.4e-16	0,461	1,101	0	3,446	0,254	2,713	0,129	2,974	1	-0,75	CAAT1_ARATH	Cationic amino acid transporter 1 (Amino acid transporter 1)	CAT1 AAT1 At4g21120 F7J7.60	Arabidopsis thaliana (Mouse-ear cress)		Acetylation; Amino-acid transport; Complete proteome; Glycoprotein; Membrane; Reference proteome; Transmembrane; Transmembrane helix; Transport
SRR648705_TR23032_c0_g1_i8_len=2384	RH56_ARATH	Q:347-1513,H:39-427	95.63%ID	E:0	DEAD-box ATP-dependent RNA helicase 56	PF00270.26^DEAD^DEAD/DEAH box helicase^65-230^E:9.2e-38`PF04851.12^ResIII^Type III restriction enzyme, res subunit^81-227^E:2.1e-06`PF00271.28^Helicase_C^Helicase conserved C-terminal domain^271-377^E:2.2e-27	0,429	0,535	0,001	1,569	0,472	0,562	1	-0,076	0,745	0,191	RH56_ARATH	DEAD-box ATP-dependent RNA helicase 56 (EC 3.6.4.13) (UAP56 homolog B)	RH56 UAP56B At5g11200 F2I11.90	Arabidopsis thaliana (Mouse-ear cress)		ATP-binding; Alternative splicing; Complete proteome; Helicase; Hydrolase; Nucleotide-binding; Nucleus; RNA-binding; Reference proteome; Transport; mRNA processing; mRNA splicing; mRNA transport
SRR648705_TR23290_c0_g1_i1_len=2230	BGL40_ARATH	Q:1865-414,H:25-506	70.66%ID	E:0	Beta-glucosidase 40	PF00232.15^Glyco_hydro_1^Glycosyl hydrolase family 1^38-512^E:2.6e-159	0,076	2,308	0	5,213	0,103	1,853	1	0,232	0,226	-1,744	BGL40_ARATH	Beta-glucosidase 40 (AtBGLU40) (EC 3.2.1.21)	BGLU40 At1g26560 T1K7.7	Arabidopsis thaliana (Mouse-ear cress)		Complete proteome; Disulfide bond; Glycoprotein; Glycosidase; Hydrolase; Reference proteome; Signal
SRR648705_TR23916_c1_g2_i3_len=1259	GL52_ORYSJ	Q:352-942,H:24-220	79.19%ID	E:1e-108	Germin-like protein 5-1	PF00190.19^Cupin_1^Cupin^64-210^E:5.6e-50`PF07883.8^Cupin_2^Cupin domain^100-171^E:1.5e-11	0,623	0,384	0	2,785	0,086	0,983	0,623	0,406	0,283	0,617	GL52_ORYSJ	Germin-like protein 5-1	Os05g0277500 LOC_Os05g19670 OsJ_017118 OSJNBa0055E23.9	Oryza sativa subsp. japonica (Rice)		Apoplast; Complete proteome; Disulfide bond; Glycoprotein; Manganese; Metal-binding; Reference proteome; Secreted; Signal
SRR648705_TR24245_c1_g1_i4_len=867	NA	NA	NA	NA	NA	.	0,594	0,174	0	1,856	0,099	0,831	0,153	0,748	0,381	0,845	.	.	.	.	.	.
SRR648705_TR24558_c0_g1_i1_len=3561	NRAM6_ARATH	Q:283-1437,H:7-391	82.34%ID	E:0	Metal transporter Nramp6	PF01566.15^Nramp^Natural resistance-associated macrophage protein^64-396^E:9.2e-114	0,017	1,076	0	8,422	1	0,38	1	0,43	1	0,701	NRAM6_ARATH	Metal transporter Nramp6 (AtNramp6)	NRAMP6 At1g15960 T24D18.6	Arabidopsis thaliana (Mouse-ear cress)		Alternative splicing; Complete proteome; Ion transport; Iron; Iron transport; Membrane; Reference proteome; Transmembrane; Transmembrane helix; Transport
SRR648705_TR25855_c0_g1_i1_len=1517	NA	NA	NA	NA	NA	.	0,544	-0,211	0	4,09	1	1,056	1	0,86	1	-0,01	.	.	.	.	.	.
SRR648705_TR26775_c0_g1_i1_len=2734	PAL1_TOBAC	Q:2460-337,H:4-715	88.48%ID	E:0	Phenylalanine ammonia-lyase	PF00221.16^Lyase_aromatic^Aromatic amino acid lyase^62-540^E:3.3e-154	0,25	0,42	0	2,554	0,026	0,491	0,003	0,644	0	-1,871	PAL1_TOBAC	Phenylalanine ammonia-lyase (EC 4.3.1.24)	TPA1 PALB	Nicotiana tabacum (Common tobacco)	PATHWAY: Phenylpropanoid metabolism; trans-cinnamate biosynthesis; trans-cinnamate from L-phenylalanine: step 1/1.	Cytoplasm; Lyase; Phenylpropanoid metabolism
SRR648705_TR27157_c0_g1_i1_len=2229	ORP3A_ARATH	Q:602-1912,H:22-452	76.2%ID	E:0	Oxysterol-binding protein-related protein 3A	PF01237.15^Oxysterol_BP^Oxysterol-binding protein^72-426^E:6.1e-102	1	-0,004	0	2,309	0,044	2,406	0,016	2,669	0,611	-0,429	ORP3A_ARATH	Oxysterol-binding protein-related protein 3A (OSBP-related protein 3A) (Protein UNFERTILIZED EMBRYO SAC 18)	ORP3A UNE18 At5g02100 T7H20.150	Arabidopsis thaliana (Mouse-ear cress)		Complete proteome; Endoplasmic reticulum; Fertilization; Golgi apparatus; Lipid transport; Lipid-binding; Reference proteome; Steroid-binding; Transport
SRR648705_TR28402_c0_g1_i8_len=2308	KC1D_ARATH	Q:1668-613,H:1-365	72.4%ID	E:0	Casein kinase I isoform delta-like	PF00069.22^Pkinase^Protein kinase domain^10-232^E:1.4e-27`PF07714.14^Pkinase_Tyr^Protein tyrosine kinase^10-270^E:3.7e-15	0,387	0,435	0	7,746	1	-0,093	0,115	0,677	0,093	0,846	KC1D_ARATH	Casein kinase I isoform delta-like (CKI-delta) (EC 2.7.11.1)	At4g26100 F20B18.210	Arabidopsis thaliana (Mouse-ear cress)		ATP-binding; Complete proteome; Cytoplasm; Kinase; Nucleotide-binding; Phosphoprotein; Reference proteome; Serine/threonine-protein kinase; Transferase
SRR648705_TR29523_c0_g2_i1_len=1570	GOLS1_AJURE	Q:200-1216,H:1-333	83.78%ID	E:0	Galactinol synthase 1	PF01501.17^Glyco_transf_8^Glycosyl transferase family 8^32-274^E:3.6e-43	0,002	1,226	0	1,54	0,061	0,467	0,078	0,44	0,001	-0,763	GOLS1_AJURE	Galactinol synthase 1 (ArGolS1) (GolS-1) (EC 2.4.1.123)	GOLS1	Ajuga reptans (Bugle)		Carbohydrate metabolism; Cytoplasm; Galactose metabolism; Glycosyltransferase; Manganese; Metal-binding; Transferase
SRR648705_TR29889_c0_g1_i1_len=1289	ABIL2_ARATH	Q:91-921,H:55-312	54.64%ID	E:6e-74	Protein ABIL2	.	1	-0,072	0	2,912	0,073	1,965	0,118	1,881	0,822	-0,304	ABIL2_ARATH	Protein ABIL2 (Abl interactor-like protein 2) (AtABIL2)	ABIL2 At3g49290 F2K15.150	Arabidopsis thaliana (Mouse-ear cress)		Alternative splicing; Complete proteome; Cytoplasm; Cytoskeleton; Reference proteome
SRR648705_TR31810_c0_g1_i1_len=1031	HA22E_ARATH	Q:703-383,H:1-106	75.7%ID	E:1e-36	HVA22-like protein e	PF03134.16^TB2_DP1_HVA22^TB2/DP1, HVA22 family^24-98^E:3.8e-26	0,593	0,632	0	3,563	0,008	1,381	0,196	0,768	1	-1,883	HA22E_ARATH	HVA22-like protein e (AtHVA22e)	HVA22E At5g50720 MFB16.12	Arabidopsis thaliana (Mouse-ear cress)		Complete proteome; Membrane; Reference proteome; Stress response; Transmembrane; Transmembrane helix
SRR648705_TR32123_c0_g2_i1_len=2142	AROG_SOLTU	Q:304-1782,H:30-510	84.79%ID	E:0	Phospho-2-dehydro-3-deoxyheptonate aldolase 2, chloroplastic	PF01474.13^DAHP_synth_2^Class-II DAHP synthetase family^2-384^E:1.3e-180	0,356	1,004	0,001	3,368	1	0,454	0,692	0,824	0	-1,824	AROG_SOLTU	Phospho-2-dehydro-3-deoxyheptonate aldolase 2, chloroplastic (EC 2.5.1.54) (3-deoxy-D-arabino-heptulosonate 7-phosphate synthase 2) (DAHP synthase 2) (Phospho-2-keto-3-deoxyheptonate aldolase 2)	SHKB ARO2	Solanum tuberosum (Potato)	PATHWAY: Metabolic intermediate biosynthesis; chorismate biosynthesis; chorismate from D-erythrose 4-phosphate and phosphoenolpyruvate: step 1/7.	Amino-acid biosynthesis; Aromatic amino acid biosynthesis; Chloroplast; Complete proteome; Plastid; Reference proteome; Transferase; Transit peptide
SRR648705_TR33278_c0_g1_i1_len=1304	NA	NA	NA	NA	NA	.	0,748	-0,206	0	3,068	0,412	0,508	0,01	1,276	1	0	.	.	.	.	.	.
SRR648705_TR33627_c0_g2_i2_len=1496	MY1R1_SOLTU	Q:1344-571,H:23-257	57.36%ID	E:3e-65	Transcription factor MYB1R1	PF00249.28^Myb_DNA-binding^Myb-like DNA-binding domain^133-177^E:5.1e-11	0,256	0,414	0	1,57	0,031	0,638	0,104	0,491	0,555	0,272	MY1R1_SOLTU	Transcription factor MYB1R1 (Myb-related protein R1) (StMYB1R-1)		Solanum tuberosum (Potato)		Complete proteome; Cytoplasm; DNA-binding; Nucleus; Reference proteome; Stress response; Transcription; Transcription regulation
SRR648705_TR34636_c0_g1_i1_len=2294	CIPK6_ARATH	Q:1700-468,H:18-438	77.91%ID	E:0	CBL-interacting serine/threonine-protein kinase 6	PF00069.22^Pkinase^Protein kinase domain^16-270^E:5.7e-75`PF07714.14^Pkinase_Tyr^Protein tyrosine kinase^17-265^E:4.3e-35`PF14531.3^Kinase-like^Kinase-like^111-257^E:8.6e-08`PF03822.11^NAF^NAF domain^296-355^E:3.5e-22	0,127	0,372	0	1,355	0,009	0,642	0,694	0,105	0,104	0,434	CIPK6_ARATH	CBL-interacting serine/threonine-protein kinase 6 (EC 2.7.11.1) (SNF1-related kinase 3.14) (SOS2-like protein kinase PKS4) (SOS3-interacting protein 3)	CIPK6 PKS4 SIP3 SnRK3.14 At4g30960 F6I18.130	Arabidopsis thaliana (Mouse-ear cress)		ATP-binding; Complete proteome; Endoplasmic reticulum; Kinase; Manganese; Nucleotide-binding; Phosphoprotein; Reference proteome; Serine/threonine-protein kinase; Transferase
SRR648705_TR35196_c0_g3_i1_len=2965	AVP_VIGRR	Q:257-2563,H:1-766	90.77%ID	E:0	Pyrophosphate-energized vacuolar membrane proton pump	PF03030.13^H_PPase^Inorganic H+ pyrophosphatase^21-754^E:1.5e-260	0,208	-0,363	0	8,959	1	-2,393	1	-2,927	0,546	-0,202	AVP_VIGRR	Pyrophosphate-energized vacuolar membrane proton pump (EC 3.6.1.1) (Pyrophosphate-energized inorganic pyrophosphatase) (H(+)-PPase) (Vacuolar H(+)-pyrophosphatase)		Vigna radiata var. radiata (Mung bean) (Phaseolus aureus)		3D-structure; Direct protein sequencing; Disulfide bond; Hydrogen ion transport; Hydrolase; Ion transport; Magnesium; Membrane; Metal-binding; Transmembrane; Transmembrane helix; Transport; Vacuole
SRR648705_TR36700_c0_g2_i1_len=1096	TAF10_ARATH	Q:401-808,H:1-134	73.53%ID	E:2e-57	Transcription initiation factor TFIID subunit 10	PF03540.10^TFIID_30kDa^Transcription initiation factor TFIID 23-30kDa subunit^39-88^E:2.4e-22	1	0,569	0	3,574	1	0,549	1	-0,097	0,452	-0,544	TAF10_ARATH	Transcription initiation factor TFIID subunit 10 (Protein SALT TOLERANCE DURING GERMINATION 1) (TBP-associated factor 10) (AtTAF10) (TBP-associated factor II 15)	TAF10 SGT1 TAFII15 At4g31720 F28M20.90	Arabidopsis thaliana (Mouse-ear cress)		Activator; Complete proteome; Nucleus; Reference proteome; Transcription; Transcription regulation
SRR648705_TR37292_c1_g1_i2_len=2917	NCPR_CATRO	Q:2553-427,H:6-714	99.72%ID	E:0	NADPH–cytochrome P450 reductase {ECO:0000255|HAMAP-Rule:MF_03212}	PF00258.22^Flavodoxin_1^Flavodoxin^106-249^E:1.5e-32`PF00667.17^FAD_binding_1^FAD binding domain^310-531^E:2e-68`PF00175.18^NAD_binding_1^Oxidoreductase NAD-binding domain^568-678^E:1.6e-16	0,002	0,902	0	2,898	0,778	0,065	0,723	0,084	1	0,013	NCPR_CATRO	NADPH–cytochrome P450 reductase (CPR) (P450R) (EC 1.6.2.4)	CPR	Catharanthus roseus (Madagascar periwinkle) (Vinca rosea)		Endoplasmic reticulum; FAD; FMN; Flavoprotein; Membrane; NADP; Oxidoreductase
SRR648705_TR37367_c0_g2_i3_len=1695	LHT1_ARATH	Q:275-1597,H:5-446	82.84%ID	E:0	Lysine histidine transporter 1	PF01490.15^Aa_trans^Transmembrane amino acid transporter protein^32-426^E:1.7e-108`PF03222.10^Trp_Tyr_perm^Tryptophan/tyrosine permease family^36-166^E:5.2e-09	0,093	1,101	0	2,755	1	-0,118	1	0,029	0,034	-4,869	LHT1_ARATH	Lysine histidine transporter 1	LHT1 At5g40780 K1B16.3	Arabidopsis thaliana (Mouse-ear cress)		Alternative splicing; Amino-acid transport; Cell membrane; Complete proteome; Membrane; Reference proteome; Symport; Transmembrane; Transmembrane helix; Transport
SRR648705_TR39730_c0_g1_i2_len=2695	DNAJ_THEEB	Q:475-1512,H:2-342	54.08%ID	E:4e-111	Chaperone protein DnaJ {ECO:0000255|HAMAP-Rule:MF_01152}	PF00226.28^DnaJ^DnaJ domain^74-135^E:1.5e-26`PF01556.15^DnaJ_C^DnaJ C terminal domain^194-411^E:3.8e-32`PF00684.16^DnaJ_CXXCXGXG^DnaJ central domain^220-284^E:2.5e-12	0,93	-0,074	0	2,884	1	0,26	1	-0,659	0,766	0,151	DNAJ_THEEB	Chaperone protein DnaJ	dnaJ tll0790	Thermosynechococcus elongatus (strain BP-1)		Chaperone; Complete proteome; Cytoplasm; DNA replication; Metal-binding; Reference proteome; Repeat; Stress response; Zinc; Zinc-finger
SRR648705_TR40618_c0_g2_i1_len=1906	PROD2_ARATH	Q:1552-281,H:42-467	57.04%ID	E:3e-180	Proline dehydrogenase 2, mitochondrial	PF01619.15^Pro_dh^Proline dehydrogenase^144-480^E:3.4e-76	0,035	1,623	0,001	2,903	0,017	0,979	0,06	0,781	0,004	-2,086	PROD2_ARATH	Proline dehydrogenase 2, mitochondrial (EC 1.5.5.2) (Osmotic stress-induced proline dehydrogenase) (Proline oxidase)	POX2 PRODH2 At5g38710 MKD10.10	Arabidopsis thaliana (Mouse-ear cress)	PATHWAY: Amino-acid degradation; L-proline degradation into L-glutamate; L-glutamate from L-proline: step 1/2.	Complete proteome; FAD; Flavoprotein; Mitochondrion; Oxidoreductase; Proline metabolism; Reference proteome; Stress response; Transit peptide
SRR648705_TR40655_c1_g3_i5_len=2382	MAOX_VITVI	Q:2222-996,H:1-409	86.8%ID	E:0	NADP-dependent malic enzyme	PF00390.16^malic^Malic enzyme, N-terminal domain^117-296^E:4e-79`PF03949.12^Malic_M^Malic enzyme, NAD binding domain^307-407^E:1.8e-26	0,085	0,629	0	2,364	1	2,387	0,502	4,342	1	-1,421	MAOX_VITVI	NADP-dependent malic enzyme (NADP-ME) (EC 1.1.1.40)		Vitis vinifera (Grape)		Cytoplasm; Metal-binding; NADP; Oxidoreductase
SRR648705_TR40800_c0_g1_i1_len=351	NA	NA	NA	NA	NA	.	0,86	-0,05	0	1,422	0,153	0,738	0,188	0,708	0,509	0,318	.	.	.	.	.	.
SRR648705_TR42241_c0_g1_i6_len=1717	PIP11_ORYSJ	Q:818-1351,H:112-289	85.39%ID	E:5e-102	Aquaporin PIP1-1	PF00230.17^MIP^Major intrinsic protein^1-136^E:1.1e-47	0,356	1,136	0	3,899	0,014	1,677	0,031	1,565	1	-1,102	PIP11_ORYSJ	Aquaporin PIP1-1 (OsPIP1;1) (Plasma membrane intrinsic protein 1-1) (Plasma membrane intrinsic protein 1a) (PIP1a) (Water channel protein RWC1) (RWC-1)	PIP1-1 PIP1A RWC1 Os02g0666200 LOC_Os02g44630 OJ1486_E07.10 OsJ_007594 P0461B08.25	Oryza sativa subsp. japonica (Rice)		Cell membrane; Complete proteome; Membrane; Reference proteome; Repeat; Transmembrane; Transmembrane helix; Transport
SRR648705_TR42436_c0_g1_i2_len=1575	OP162_ARATH	Q:826-317,H:7-178	67.05%ID	E:1e-63	Outer envelope pore protein 16-2, chloroplastic	PF02466.16^Tim17^Tim17/Tim22/Tim23/Pmp24 family^34-171^E:7e-17	1	1,321	0	6,831	0,132	1,404	1	0,271	0,299	1,816	OP162_ARATH	Outer envelope pore protein 16-2, chloroplastic (Chloroplastic outer envelope pore protein of 16 kDa 2) (AtOEP16-2) (OEP16-2) (Outer plastid envelope protein 16-S) (AtOEP16-S) (Seeds outer plastid envelope protein 16)	OEP162 At4g16160 dl4120w FCAALL.207	Arabidopsis thaliana (Mouse-ear cress)		Alternative splicing; Chloroplast; Complete proteome; Ion transport; Membrane; Plastid; Plastid outer membrane; Porin; Reference proteome; Transmembrane; Transmembrane beta strand; Transmembrane helix; Transport
SRR648705_TR42535_c0_g1_i1_len=923	NA	NA	NA	NA	NA	.	0,254	2,666	0	6,583	0,066	4,088	0,066	4,049	1	-0,507	.	.	.	.	.	.
SRR648705_TR43148_c1_g2_i1_len=2238	GH31_ARATH	Q:2104-353,H:16-589	76.41%ID	E:0	Probable indole-3-acetic acid-amido synthetase GH3.1	PF03321.10^GH3^GH3 auxin-responsive promoter^32-575^E:1.4e-203	1	0,385	0	7,83	1	1,372	0,502	2,814	1	-0,735	GH31_ARATH	Probable indole-3-acetic acid-amido synthetase GH3.1 (EC 6.3.2.-) (Auxin-responsive GH3-like protein 1) (AtGH3-1)	GH3.1 At2g14960 T26I20.12	Arabidopsis thaliana (Mouse-ear cress)		Complete proteome; Ligase; Reference proteome
SRR648705_TR43618_c0_g1_i9_len=1272	RS12_HORVU	Q:299-670,H:20-143	83.06%ID	E:3e-59	40S ribosomal protein S12	PF01248.23^Ribosomal_L7Ae^Ribosomal protein L7Ae/L30e/S12e/Gadd45 family^27-120^E:1.9e-26	1	0,064	0,001	3,517	1	0,442	1	-0,836	0,335	-0,811	RS12_HORVU	40S ribosomal protein S12	RPS12	Hordeum vulgare (Barley)		Ribonucleoprotein; Ribosomal protein
SRR648705_TR43618_c0_g1_i16_len=876	RS12_HORVU	Q:225-596,H:20-143	82.26%ID	E:4e-69	40S ribosomal protein S12	PF01248.23^Ribosomal_L7Ae^Ribosomal protein L7Ae/L30e/S12e/Gadd45 family^27-120^E:4e-27	0	-1,036	0	9,862	1	0	1	0	0,67	-0,115	RS12_HORVU	40S ribosomal protein S12	RPS12	Hordeum vulgare (Barley)		Ribonucleoprotein; Ribosomal protein
SRR648705_TR44156_c0_g1_i1_len=2243	BAM1_ARATH	Q:238-1971,H:1-575	73.51%ID	E:0	Beta-amylase 1, chloroplastic	PF01373.14^Glyco_hydro_14^Glycosyl hydrolase family 14^114-537^E:7.9e-139	0,394	0,495	0	2,748	0,01	0,855	0,005	0,929	0,014	1,168	BAM1_ARATH	Beta-amylase 1, chloroplastic (EC 3.2.1.2) (1,4-alpha-D-glucan maltohydrolase) (Beta-amylase 7) (Thioredoxin-regulated beta-amylase) (TR-BAMY)	BAM1 BMY7 TRBAMY At3g23920 F14O13.12	Arabidopsis thaliana (Mouse-ear cress)		Carbohydrate metabolism; Chloroplast; Complete proteome; Disulfide bond; Glycosidase; Hydrolase; Phosphoprotein; Plastid; Polysaccharide degradation; Reference proteome; Transit peptide
SRR648705_TR45155_c0_g1_i1_len=1815	UFOG3_FRAAN	Q:155-1567,H:3-478	51.65%ID	E:4e-165	Putative UDP-glucose flavonoid 3-O-glucosyltransferase 3 {ECO:0000250|UniProtKB:Q2V6K0}	PF00201.15^UDPGT^UDP-glucoronosyl and UDP-glucosyl transferase^321-447^E:9.7e-22	0,266	0,594	0	1,951	0,001	2,35	0,007	2,029	0,793	-0,214	UFOG3_FRAAN	Putative UDP-glucose flavonoid 3-O-glucosyltransferase 3 (FaGT3) (EC 2.4.1.-) (Flavonol 3-O-glucosyltransferase 3)	GT3	Fragaria ananassa (Strawberry)		Glycosyltransferase; Transferase
SRR648707_TR192_c0_g2_i1_len=1782	BEATH_CLABR	Q:167-1456,H:3-432	33.18%ID	E:1e-60	Acetyl-CoA-benzylalcohol acetyltransferase	PF02458.12^Transferase^Transferase family^55-480^E:5.4e-69	0,419	0,427	0,001	1,918	0,243	0,577	0,419	0,467	1	-0,15	BEATH_CLABR	Acetyl-CoA-benzylalcohol acetyltransferase (CbBEAT-1) (EC 2.3.1.224)	BEAT	Clarkia breweri (Fairy fans) (Eucharidium breweri)		Acyltransferase; Direct protein sequencing; Transferase
SRR648707_TR238_c0_g1_i2_len=3001	TMN9_ARATH	Q:2512-638,H:16-644	82.83%ID	E:0	Transmembrane 9 superfamily member 9 {ECO:0000305}	PF02990.13^EMP70^Endomembrane protein 70^61-598^E:3.4e-220	0,275	0,442	0	1,141	0,103	0,543	0,082	0,584	0,027	-0,855	TMN9_ARATH	Transmembrane 9 superfamily member 9 (Endomembrane protein 2) (Transmembrane nine protein 9) (AtTMN9)	TMN9 EMP2 At5g25100 T11H3_110	Arabidopsis thaliana (Mouse-ear cress)		Alternative splicing; Complete proteome; Endosome; Golgi apparatus; Membrane; Reference proteome; Signal; Transmembrane; Transmembrane helix
SRR648707_TR1407_c0_g1_i1_len=1308	EXO_ARATH	Q:1104-262,H:23-314	65.54%ID	E:1e-130	Protein EXORDIUM	PF04674.9^Phi_1^Phosphate-induced protein 1 conserved region^44-310^E:2.2e-121	0,692	0,957	0	3,625	1	0,045	1	-0,157	1	-0,135	EXO_ARATH	Protein EXORDIUM	EXO At4g08950 T3H13.3	Arabidopsis thaliana (Mouse-ear cress)		Apoplast; Complete proteome; Reference proteome; Secreted; Signal
SRR648707_TR2884_c2_g2_i5_len=1821	MTDH_FRAAN	Q:497-1555,H:4-356	66.86%ID	E:3e-141	Probable mannitol dehydrogenase	PF08240.9^ADH_N^Alcohol dehydrogenase GroES-like domain^39-151^E:6.3e-28`PF00107.23^ADH_zinc_N^Zinc-binding dehydrogenase^195-318^E:7.5e-19`PF13602.3^ADH_zinc_N_2^Zinc-binding dehydrogenase^228-347^E:3e-07	0,098	0,46	0	1,281	0,01	0,871	0,165	0,503	0,565	0,199	MTDH_FRAAN	Probable mannitol dehydrogenase (EC 1.1.1.255) (NAD-dependent mannitol dehydrogenase)	CAD	Fragaria ananassa (Strawberry)		Metal-binding; NAD; Oxidoreductase; Zinc
SRR648707_TR5359_c0_g2_i1_len=1529	EXOL2_ARATH	Q:138-1013,H:8-305	71.76%ID	E:5e-148	Protein EXORDIUM-like 2	PF04674.9^Phi_1^Phosphate-induced protein 1 conserved region^39-301^E:1.7e-122	0,025	3,693	0	7,202	0,066	3,893	1	1,337	0,712	0,24	EXOL2_ARATH	Protein EXORDIUM-like 2	EXL2 At5g64260 MSJ1.10	Arabidopsis thaliana (Mouse-ear cress)		Apoplast; Complete proteome; Glycoprotein; Reference proteome; Secreted; Signal
SRR648707_TR5635_c2_g3_i2_len=1841	MTDH_FRAAN	Q:988-1788,H:90-356	55.02%ID	E:2e-85	Probable mannitol dehydrogenase	PF08240.9^ADH_N^Alcohol dehydrogenase GroES-like domain^3-53^E:0.00013`PF00107.23^ADH_zinc_N^Zinc-binding dehydrogenase^97-217^E:1.6e-12	1	0,603	0	5,522	1	1,432	1	-0,795	0,718	0,25	MTDH_FRAAN	Probable mannitol dehydrogenase (EC 1.1.1.255) (NAD-dependent mannitol dehydrogenase)	CAD	Fragaria ananassa (Strawberry)		Metal-binding; NAD; Oxidoreductase; Zinc
SRR648707_TR5635_c2_g3_i6_len=2259	MTDH_FRAAN	Q:463-1509,H:10-356	58.17%ID	E:3e-119	Probable mannitol dehydrogenase	PF08240.9^ADH_N^Alcohol dehydrogenase GroES-like domain^39-152^E:9.5e-27`PF00107.23^ADH_zinc_N^Zinc-binding dehydrogenase^196-320^E:1.5e-16	0,079	0,444	0	2,554	0,008	0,742	1	-0,006	0,425	0,206	MTDH_FRAAN	Probable mannitol dehydrogenase (EC 1.1.1.255) (NAD-dependent mannitol dehydrogenase)	CAD	Fragaria ananassa (Strawberry)		Metal-binding; NAD; Oxidoreductase; Zinc

SRR648707_TR5635_c2_g3_i8_len=1332	GEDH1_OCIBA	Q:225-1097,H:11-299	54.64%ID	E:1e-89	Geraniol dehydrogenase 1	PF08240.9^ADH_N^Alcohol dehydrogenase GroES-like domain^39-152^E:2.1e-27`PF00107.23^ADH_zinc_N^Zinc-binding dehydrogenase^196-299^E:6.1e-10	1	-0,102	0	5,775	0,629	0,868	1	-0,229	0,718	0,217	GEDH1_OCIBA	Geraniol dehydrogenase 1 (ObaGEDH1) (EC 1.1.1.183)	GEDH1	Ocimum basilicum (Sweet basil)		Metal-binding; NADP; Oxidoreductase; Zinc
SRR648707_TR5814_c0_g2_i4_len=1929	BZW2_CHICK	Q:258-1484,H:1-411	32.77%ID	E:8e-72	Basic leucine zipper and W2 domain-containing protein 2	PF02020.15^W2^eIF4-gamma/eIF5/eIF2-epsilon^337-410^E:2.1e-19	0,456	0,246	0	1,445	0,034	1,145	0,1	0,937	0,908	0,04	BZW2_CHICK	Basic leucine zipper and W2 domain-containing protein 2	BZW2 RCJMB04_7o12	Gallus gallus (Chicken)		Complete proteome; Developmental protein; Differentiation; Neurogenesis; Reference proteome
SRR648707_TR6209_c0_g1_i1_len=1737	GST23_MAIZE	Q:366-1028,H:1-222	49.55%ID	E:8e-71	Glutathione transferase GST 23	PF02798.17^GST_N^Glutathione S-transferase, N-terminal domain^5-77^E:2.4e-19`PF13417.3^GST_N_3^Glutathione S-transferase, N-terminal domain^8-79^E:2.8e-16`PF13409.3^GST_N_2^Glutathione S-transferase, N-terminal domain^13-77^E:1.4e-12	1	0	0,001	6,643	0,254	4,734	1	3,215	1	2,871	GST23_MAIZE	Glutathione transferase GST 23 (EC 2.5.1.18) (Glutathione transferase GST 36)		Zea mays (Maize)		Complete proteome; Plant defense; Reference proteome; Transferase
SRR648707_TR6306_c0_g1_i1_len=1846	METK1_CATRO	Q:1729-551,H:1-393	99.75%ID	E:0	S-adenosylmethionine synthase 1	PF00438.17^S-AdoMet_synt_N^S-adenosylmethionine synthetase, N-terminal domain^4-101^E:8.4e-43`PF02772.13^S-AdoMet_synt_M^S-adenosylmethionine synthetase, central domain^117-238^E:1.7e-46`PF02773.13^S-AdoMet_synt_C^S-adenosylmethionine synthetase, C-terminal domain^240-381^E:2e-62	0,018	0,506	0	1,914	0,045	0,445	0,014	0,547	0,507	-0,388	METK1_CATRO	S-adenosylmethionine synthase 1 (AdoMet synthase 1) (EC 2.5.1.6) (Methionine adenosyltransferase 1) (MAT 1)	SAMS1	Catharanthus roseus (Madagascar periwinkle) (Vinca rosea)	PATHWAY: Amino-acid biosynthesis; S-adenosyl-L-methionine biosynthesis; S-adenosyl-L-methionine from L-methionine: step 1/1.	ATP-binding; Cobalt; Cytoplasm; Magnesium; Manganese; Metal-binding; Nucleotide-binding; One-carbon metabolism; Potassium; Transferase
SRR648707_TR7957_c0_g2_i1_len=2226	DHQS_SOLLC	Q:1873-992,H:40-339	85.33%ID	E:7e-179	3-dehydroquinate synthase, chloroplastic	PF13685.3^Fe-ADH_2^Iron-containing alcohol dehydrogenase^98-318^E:1.2e-11`PF01761.17^DHQ_synthase^3-dehydroquinate synthase^150-335^E:8.3e-82	0,089	0,73	0	1,511	0,573	0,235	0,349	0,358	0,043	2,606	DHQS_SOLLC	3-dehydroquinate synthase, chloroplastic (EC 4.2.3.4)	DHQS AROB Solyc02g083590 LOC544273	Solanum lycopersicum (Tomato) (Lycopersicon esculentum)	PATHWAY: Metabolic intermediate biosynthesis; chorismate biosynthesis; chorismate from D-erythrose 4-phosphate and phosphoenolpyruvate: step 2/7.	Amino-acid biosynthesis; Aromatic amino acid biosynthesis; Chloroplast; Complete proteome; Lyase; Metal-binding; NAD; Plastid; Reference proteome; Transit peptide
SRR648707_TR8248_c0_g2_i1_len=1563	TT12_ARATH	Q:275-1468,H:107-504	48.99%ID	E:5e-118	.	PF01554.15^MatE^MatE^7-121^E:1.8e-25`PF01554.15^MatE^MatE^183-344^E:1.2e-30	0,239	1,225	0	3,88	0,132	1,94	0,381	1,317	0,27	0,296	DTX41_ARATH	Protein DETOXIFICATION 41 (AtDTX41) (Multidrug and toxic compound extrusion protein 41) (MATE protein 41) (Protein TANNIN-DEFICIENT SEED 3) (Protein TRANSPARENT TESTA 12)	DTX41 TDS3 TT12 At3g59030 F17J16_80	Arabidopsis thaliana (Mouse-ear cress)	PATHWAY: Secondary metabolite biosynthesis; flavonoid biosynthesis.	Antiport; Complete proteome; Flavonoid biosynthesis; Membrane; Reference proteome; Transmembrane; Transmembrane helix; Transport; Vacuole
SRR648707_TR8391_c0_g1_i1_len=2404	SGRW_PEA	Q:2106-1384,H:1-236	71.07%ID	E:4e-112	Protein STAY-GREEN, chloroplastic	.	1	0,985	0,001	4,573	0,076	2,808	0,014	3,301	1	-1,584	SGRW_PEA	Protein STAY-GREEN, chloroplastic (Protein STAYGREEN)	SGR	Pisum sativum (Garden pea)		Chloroplast; Plastid; Transit peptide
SRR648707_TR8550_c0_g1_i1_len=1443	PPSP2_ARATH	Q:1193-366,H:7-274	65.22%ID	E:1e-127	Inorganic pyrophosphatase 2	PF06888.9^Put_Phosphatase^Putative Phosphatase^5-237^E:9.6e-97`PF12710.4^HAD^haloacid dehalogenase-like hydrolase^7-185^E:4.8e-09	0,502	4,157	0	7,512	0,011	1,502	0,016	1,436	0,629	1,898	PPSP2_ARATH	Inorganic pyrophosphatase 2 (AtPPsPase2) (PPi phosphatase 2) (Pyrophosphate-specific phosphatase 2) (EC 3.6.1.1)	At1g17710 F11A6.5	Arabidopsis thaliana (Mouse-ear cress)		Alternative splicing; Complete proteome; Hydrolase; Magnesium; Metal-binding; Reference proteome
SRR648707_TR8559_c2_g1_i1_len=2840	METE_CATRO	Q:2685-391,H:1-765	99.22%ID	E:0	5-methyltetrahydropteroyltriglutamate--homocysteine methyltransferase	PF08267.9^Meth_synt_1^Cobalamin-independent synthase, N-terminal domain^3-315^E:3.8e-121`PF01717.15^Meth_synt_2^Cobalamin-independent synthase, Catalytic domain^432-755^E:1.1e-157`PF08267.9^Meth_synt_1^Cobalamin-independent synthase, N-terminal domain^551-690^E:0.00019	0,138	0,318	0	2,233	0,002	-0,689	0,024	-0,506	0,176	-0,409	METE_CATRO	5-methyltetrahydropteroyltriglutamate--homocysteine methyltransferase (EC 2.1.1.14) (Cobalamin-independent methionine synthase isozyme) (Vitamin-B12-independent methionine synthase isozyme)	METE	Catharanthus roseus (Madagascar periwinkle) (Vinca rosea)	PATHWAY: Amino-acid biosynthesis; L-methionine biosynthesis via de novo pathway; L-methionine from L-homocysteine (MetE route): step 1/1.	Amino-acid biosynthesis; Cytoplasm; Metal-binding; Methionine biosynthesis; Methyltransferase; Transferase; Zinc
SRR648707_TR9041_c1_g1_i2_len=2978	CUL1_ARATH	Q:2703-517,H:4-732	79.29%ID	E:0	Cullin-1	PF00888.19^Cullin^Cullin family^24-644^E:1.7e-178`PF10557.6^Cullin_Nedd8^Cullin protein neddylation domain^671-733^E:5.4e-26	0,178	-0,598	0	7,685	0,057	1,241	0,04	1,325	1	0	CUL1_ARATH	Cullin-1	CUL1 At4g02570 T10P11.26	Arabidopsis thaliana (Mouse-ear cress)	PATHWAY: Protein modification; protein ubiquitination.	Auxin signaling pathway; Cell cycle; Complete proteome; Cytoplasm; Cytoskeleton; Developmental protein; Ethylene signaling pathway; Isopeptide bond; Nucleus; Reference proteome; Ubl conjugation; Ubl conjugation pathway
SRR648707_TR10329_c0_g1_i1_len=2485	KPRS2_SPIOL	Q:1824-763,H:39-395	84.87%ID	E:0	Ribose-phosphate pyrophosphokinase 2, chloroplastic	PF13793.3^Pribosyltran_N^N-terminal domain of ribose phosphate pyrophosphokinase^2-98^E:9.5e-43`PF00156.24^Pribosyltran^Phosphoribosyl transferase domain^133-229^E:1.7e-15`PF14572.3^Pribosyl_synth^Phosphoribosyl synthetase-associated domain^184-291^E:2.1e-23	1	-0,06	0	3,27	0,035	1,74	0,023	1,819	1	-0,109	KPRS2_SPIOL	Ribose-phosphate pyrophosphokinase 2, chloroplastic (EC 2.7.6.1) (Phosphoribosyl pyrophosphate synthase 2)	PRS2	Spinacia oleracea (Spinach)		ATP-binding; Chloroplast; Kinase; Magnesium; Metal-binding; Nucleotide biosynthesis; Nucleotide-binding; Plastid; Transferase; Transit peptide
SRR648707_TR11016_c0_g3_i1_len=1459	GSTXC_TOBAC	Q:1070-396,H:1-221	53.1%ID	E:6e-70	Probable glutathione S-transferase parC	PF13417.3^GST_N_3^Glutathione S-transferase, N-terminal domain^11-87^E:1.3e-14`PF02798.17^GST_N^Glutathione S-transferase, N-terminal domain^11-81^E:6.9e-12`PF13409.3^GST_N_2^Glutathione S-transferase, N-terminal domain^16-82^E:1.9e-10`PF00043.22^GST_C^Glutathione S-transferase, C-terminal domain^123-199^E:5.9e-10`PF13410.3^GST_C_2^Glutathione S-transferase, C-terminal domain^128-194^E:1.7e-09	0,356	1,292	0	3,531	0,002	1,803	0,109	1,101	1	0,419	GSTXC_TOBAC	Probable glutathione S-transferase parC (EC 2.5.1.18) (Auxin-regulated protein parC)	PARC	Nicotiana tabacum (Common tobacco)		Auxin signaling pathway; Transferase
SRR648707_TR11446_c0_g2_i1_len=1674	C7D55_HYOMU	Q:1437-82,H:47-495	45.58%ID	E:1e-149	Premnaspirodiene oxygenase	PF00067.19^p450^Cytochrome P450^66-511^E:4.5e-90	0,629	2,092	0	6,027	1	0,562	1	1,461	1	-0,075	C7D55_HYOMU	Premnaspirodiene oxygenase (HPO) (EC 1.14.13.121) (Cytochrome P450 71D55)	CYP71D55	Hyoscyamus muticus (Egyptian henbane)		Heme; Iron; Membrane; Metal-binding; Monooxygenase; NADP; Oxidoreductase; Transmembrane; Transmembrane helix
SRR648707_TR11517_c0_g1_i1_len=1879	VINSY_RAUSE	Q:1568-366,H:1-401	70.79%ID	E:0	Vinorine synthase	PF02458.12^Transferase^Transferase family^6-411^E:5.9e-80	0,61	0,184	0,001	1,501	0,206	-0,464	0,176	-0,506	0,121	0,795	VINSY_RAUSE	Vinorine synthase (EC 2.3.1.160)	ACT	Rauvolfia serpentina (Serpentine wood) (Ophioxylon serpentinum)		3D-structure; Acyltransferase; Alkaloid metabolism; Direct protein sequencing; Transferase
SRR648707_TR11691_c0_g1_i1_len=1632	CLC1_ARATH	Q:494-1045,H:56-241	63.44%ID	E:1e-56	Clathrin light chain 1	PF01086.14^Clathrin_lg_ch^Clathrin light chain^131-271^E:4.4e-10	1	0,048	0	1,795	1	0,044	0,86	-0,204	1	-0,018	CLC1_ARATH	Clathrin light chain 1	At2g20760 F5H14.27	Arabidopsis thaliana (Mouse-ear cress)		Coated pit; Coiled coil; Complete proteome; Cytoplasmic vesicle; Membrane; Reference proteome
SRR648707_TR11891_c2_g3_i9_len=1871	MTDH_APIGR	Q:281-1345,H:3-357	63.66%ID	E:4e-151	Mannitol dehydrogenase	PF08240.9^ADH_N^Alcohol dehydrogenase GroES-like domain^39-151^E:8.1e-22`PF00107.23^ADH_zinc_N^Zinc-binding dehydrogenase^195-318^E:1.8e-15	0,798	0,154	0	1,969	0,006	1,857	0,003	1,991	0,317	-0,469	MTDH_APIGR	Mannitol dehydrogenase (EC 1.1.1.255) (NAD-dependent mannitol dehydrogenase)	MTD	Apium graveolens (Celery)		Cytoplasm; Metal-binding; NAD; Oxidoreductase; Zinc
SRR648707_TR12055_c0_g1_i1_len=3210	AGLU_SPIOL	Q:302-2848,H:43-902	63.21%ID	E:0	Alpha-glucosidase	PF16863.2^NtCtMGAM_N^N-terminal barrel of NtMGAM and CtMGAM, maltase-glucoamylase^70-203^E:2.9e-35`PF13802.3^Gal_mutarotas_2^Galactose mutarotase-like^208-272^E:1.2e-10`PF01055.23^Glyco_hydro_31^Glycosyl hydrolases family 31^293-760^E:1.4e-156	0,175	1,074	0	8,685	1	-3,198	1	0,666	0,44	-0,83	AGLU_SPIOL	Alpha-glucosidase (EC 3.2.1.20) (Maltase)		Spinacia oleracea (Spinach)		Direct protein sequencing; Glycoprotein; Glycosidase; Hydrolase; Signal
SRR648707_TR13003_c0_g1_i1_len=2774	Y5262_ARATH	Q:915-2483,H:5-524	51.5%ID	E:4e-152	Ankyrin repeat-containing protein At5g02620	PF12796.4^Ank_2^Ankyrin repeats (3 copies)^70-140^E:1.9e-08`PF12796.4^Ank_2^Ankyrin repeats (3 copies)^101-176^E:8.4e-09`PF13637.3^Ank_4^Ankyrin repeats (many copies)^151-203^E:5.3e-06`PF12796.4^Ank_2^Ankyrin repeats (3 copies)^153-240^E:8.7e-18`PF13637.3^Ank_4^Ankyrin repeats (many copies)^184-236^E:9.6e-11`PF00023.27^Ank^Ankyrin repeat^185-209^E:0.025`PF00023.27^Ank^Ankyrin repeat^216-242^E:0.0007`PF13637.3^Ank_4^Ankyrin repeats (many copies)^219-271^E:2.1e-11`PF12796.4^Ank_2^Ankyrin repeats (3 copies)^248-314^E:9.5e-13`PF00023.27^Ank^Ankyrin repeat^251-271^E:0.017`PF13857.3^Ank_5^Ankyrin repeats (many copies)^278-326^E:1.4e-09`PF00023.27^Ank^Ankyrin repeat^285-317^E:6.7e-06`PF13962.3^PGG^Domain of unknown function^399-509^E:1.6e-24	0,183	0,68	0,001	1,871	0,547	0,421	0,547	0,43	1	0,076	Y5262_ARATH	Ankyrin repeat-containing protein At5g02620	At5g02620 T22P11.210	Arabidopsis thaliana (Mouse-ear cress)		ANK repeat; Complete proteome; Membrane; Phosphoprotein; Reference proteome; Repeat; Transmembrane; Transmembrane helix
SRR648707_TR13703_c0_g2_i1_len=1723	C94C1_ARATH	Q:1564-185,H:31-493	62.5%ID	E:0	Cytochrome P450 94C1	PF00067.19^p450^Cytochrome P450^89-488^E:1e-66	0,002	5,898	0	8,53	0,129	2,82	0,129	2,608	1	1,198	C94C1_ARATH	Cytochrome P450 94C1 (EC 1.14.-.-)	CYP94C1 At2g27690 F15K20.21	Arabidopsis thaliana (Mouse-ear cress)		Complete proteome; Heme; Iron; Jasmonic acid signaling pathway; Membrane; Metal-binding; Monooxygenase; Oxidoreductase; Reference proteome; Transmembrane; Transmembrane helix
SRR648707_TR17560_c0_g2_i1_len=1994	DHEB_NICPL	Q:1624-392,H:1-411	90.51%ID	E:0	Glutamate dehydrogenase B	PF02812.15^ELFV_dehydrog_N^Glu/Leu/Phe/Val dehydrogenase, dimerisation domain^33-159^E:6e-49`PF00208.18^ELFV_dehydrog^Glutamate/Leucine/Phenylalanine/Valine dehydrogenase^176-408^E:1.1e-72	0,08	1,347	0	1,77	1	-0,28	0,822	-0,355	1	-0,069	DHEB_NICPL	Glutamate dehydrogenase B (GDH B) (EC 1.4.1.3)	GDHB	Nicotiana plumbaginifolia (Leadwort-leaved tobacco) (Tex-Mex tobacco)		NAD; Oxidoreductase
SRR648707_TR17598_c0_g1_i1_len=1129	NA	NA	NA	NA	NA	.	0,381	1,738	0	3,153	0,006	1,876	0,004	1,91	1	-0,045	.	.	.	.	.	.
SRR648707_TR19071_c1_g2_i1_len=965	NA	NA	NA	NA	NA	.	0,581	0,385	0	1,461	1	0	1	0	0,059	1,224	.	.	.	.	.	.
SRR648707_TR19558_c0_g2_i2_len=1965	C72A1_CATRO	Q:1888-317,H:1-524	99.05%ID	E:0	Secologanin synthase	PF00067.19^p450^Cytochrome P450^93-497^E:1.6e-87	0,131	0,464	0	2,337	0,844	0,077	0,1	-0,765	0,01	0,957	C72A1_CATRO	Secologanin synthase (SLS) (EC 1.3.3.9) (CYPLXXII) (Cytochrome P450 72A1)	CYP72A1 CYP72 P450CR3	Catharanthus roseus (Madagascar periwinkle) (Vinca rosea)	PATHWAY: Alkaloid biosynthesis; secologanin biosynthesis.	Alkaloid metabolism; Heme; Iron; Membrane; Metal-binding; Monooxygenase; NADP; Oxidoreductase; Transmembrane; Transmembrane helix; Vacuole
SRR648707_TR19720_c0_g1_i1_len=1244	DNJ19_ARATH	Q:935-39,H:22-322	76.82%ID	E:2e-164	DnaJ protein ERDJ3B	PF00226.28^DnaJ^DnaJ domain^27-88^E:5e-29`PF01556.15^DnaJ_C^DnaJ C terminal domain^137-284^E:5.2e-22	0,194	-0,496	0	1,855	0,093	0,805	0,093	0,805	0,243	0,606	DNJ19_ARATH	DnaJ protein ERDJ3B (Chaperone protein dnaJ 19) (AtDjB19) (AtJ19) (Endoplasmic reticulum dnaJ domain-containing protein 3B) (AtERdj3B) (Protein SCJ1 homolog ERDJ3B)	ERDJ3B B19 At3g62600 F26K9.30	Arabidopsis thaliana (Mouse-ear cress)		Chaperone; Complete proteome; Endoplasmic reticulum; Glycoprotein; Reference proteome; Signal; Stress response
SRR648707_TR20275_c1_g1_i1_len=2708	S38A6_XENTR	Q:548-1687,H:14-405	27.98%ID	E:4e-22	Probable sodium-coupled neutral amino acid transporter 6	PF01490.15^Aa_trans^Transmembrane amino acid transporter protein^45-447^E:3.3e-58`PF03222.10^Trp_Tyr_perm^Tryptophan/tyrosine permease family^46-447^E:1.8e-11	0,211	0,792	0	3,523	0,043	1,179	0,031	1,277	1	0,332	S38A6_XENTR	Probable sodium-coupled neutral amino acid transporter 6 (Na(+)-coupled neutral amino acid transporter 6) (Solute carrier family 38 member 6)	slc38a6 TEgg038h13.1	Xenopus tropicalis (Western clawed frog) (Silurana tropicalis)		Amino-acid transport; Cell membrane; Complete proteome; Glycoprotein; Ion transport; Membrane; Reference proteome; Sodium; Sodium transport; Transmembrane; Transmembrane helix; Transport
SRR648707_TR20795_c0_g1_i1_len=3296	ZADH2_MOUSE	Q:1070-2113,H:29-377	45.79%ID	E:2e-79	Zinc-binding alcohol dehydrogenase domain-containing protein 2	PF00106.22^adh_short^short chain dehydrogenase^9-209^E:2.7e-39`PF13561.3^adh_short_C2^Enoyl-(Acyl carrier protein) reductase^13-221^E:1.2e-31`PF08240.9^ADH_N^Alcohol dehydrogenase GroES-like domain^320-385^E:6.3e-08`PF00107.23^ADH_zinc_N^Zinc-binding dehydrogenase^444-573^E:5.6e-29`PF13602.3^ADH_zinc_N_2^Zinc-binding dehydrogenase^476-621^E:4.2e-21	0,91	0,122	0	2,396	0,019	1,514	0,112	1,089	0,36	0,434	ZADH2_MOUSE	Zinc-binding alcohol dehydrogenase domain-containing protein 2 (EC 1.-.-.-)	Zadh2	Mus musculus (Mouse)		Acetylation; Complete proteome; NADP; Oxidoreductase; Peroxisome; Reference proteome
SRR648707_TR21122_c0_g3_i1_len=1311	RAD2A_ARATH	Q:260-868,H:1-203	90.15%ID	E:3e-133	Ras-related protein RABD2a	PF00025.18^Arf^ADP-ribosylation factor family^9-129^E:4.3e-17`PF00071.19^Ras^Ras family^10-170^E:2.1e-68`PF08477.10^Roc^Ras of Complex, Roc, domain of DAPkinase^10-125^E:8.1e-37`PF04670.9^Gtr1_RagA^Gtr1/RagA G protein conserved region^10-125^E:2.1e-05`PF09439.7^SRPRB^Signal recognition particle receptor beta subunit^11-131^E:2.5e-05`PF01926.20^MMR_HSR1^50S ribosome-binding GTPase^11-113^E:6.3e-05	0,374	0,286	0	1,457	0,069	0,51	0,042	0,565	0,828	-0,079	RAD2A_ARATH	Ras-related protein RABD2a (AtRABD2a) (Ras-related protein Ara-5) (Ras-related protein Rab1B) (AtRab1B)	RABD2A ARA-5 RAB1B At1g02130 T7I23.6	Arabidopsis thaliana (Mouse-ear cress)		Complete proteome; ER-Golgi transport; GTP-binding; Golgi apparatus; Lipoprotein; Membrane; Nucleotide-binding; Prenylation; Protein transport; Reference proteome; Transport
SRR648707_TR22512_c0_g3_i15_len=1245	RS12_HORVU	Q:965-594,H:20-143	82.26%ID	E:6e-59	40S ribosomal protein S12	PF01248.23^Ribosomal_L7Ae^Ribosomal protein L7Ae/L30e/S12e/Gadd45 family^27-120^E:4.8e-26	0,04	-1,36	0	4,449	0,254	-4,413	0,629	-0,975	1	0,197	RS12_HORVU	40S ribosomal protein S12	RPS12	Hordeum vulgare (Barley)		Ribonucleoprotein; Ribosomal protein
SRR648707_TR22679_c0_g1_i1_len=1138	PROF1_RICCO	Q:281-673,H:1-131	88.55%ID	E:4e-79	Profilin-1	PF00235.16^Profilin^Profilin^1-130^E:2.3e-44	0,677	-0,119	0	1,043	0,121	0,391	0,087	0,429	0,466	0,207	PROF1_RICCO	Profilin-1	PRO1	Ricinus communis (Castor bean)		Actin-binding; Cytoplasm; Cytoskeleton
SRR648707_TR24099_c1_g2_i1_len=2134	EF1A_TOBAC	Q:1860-556,H:1-435	97.93%ID	E:0	Elongation factor 1-alpha	PF00009.24^GTP_EFTU^Elongation factor Tu GTP binding domain^6-221^E:1e-52`PF03144.22^GTP_EFTU_D2^Elongation factor Tu domain 2^248-313^E:7.3e-15`PF03143.14^GTP_EFTU_D3^Elongation factor Tu C-terminal domain^322-429^E:3.9e-38	0	-0,894	0	0,911	0,689	-0,103	0,316	-0,253	1	0,022	EF1A_TOBAC	Elongation factor 1-alpha (EF-1-alpha) (Vitronectin-like adhesion protein 1) (PVN1)		Nicotiana tabacum (Common tobacco)		Cytoplasm; Elongation factor; GTP-binding; Nucleotide-binding; Phosphoprotein; Protein biosynthesis
SRR648707_TR24774_c0_g3_i1_len=1552	DV4H_CATRO	Q:1414-308,H:23-400	66.14%ID	E:9e-170	Deacetoxyvindoline 4-hydroxylase	PF14226.3^DIOX_N^non-haem dioxygenase in morphine synthesis N-terminal^59-162^E:1.1e-20`PF03171.17^2OG-FeII_Oxy^2OG-Fe(II) oxygenase superfamily^216-309^E:6.1e-23	1	0	0	6,852	1	1,217	1	2,351	1	0	DV4H_CATRO	Deacetoxyvindoline 4-hydroxylase (EC 1.14.11.20)	D4H	Catharanthus roseus (Madagascar periwinkle) (Vinca rosea)	PATHWAY: Alkaloid biosynthesis; vindoline biosynthesis; vindoline from tabersonine: step 5/6.	Cytoplasm; Dioxygenase; Direct protein sequencing; Iron; Metal-binding; Oxidoreductase; Vitamin C
SRR648707_TR25271_c0_g1_i2_len=1919	RA212_ARATH	Q:305-1507,H:1-358	42.26%ID	E:9e-63	Ethylene-responsive transcription factor RAP2-12	PF00847.17^AP2^AP2 domain^127-174^E:3.2e-11	0,883	0,073	0	1,745	0,009	0,913	0,085	0,628	0,107	0,602	RA212_ARATH	Ethylene-responsive transcription factor RAP2-12 (Protein RELATED TO APETALA2 12)	RAP2-12 ERF074 At1g53910 T18A20.14	Arabidopsis thaliana (Mouse-ear cress)		Activator; Alternative splicing; Cell membrane; Complete proteome; DNA-binding; Ethylene signaling pathway; Membrane; Nucleus; Reference proteome; Transcription; Transcription regulation
SRR648707_TR25398_c0_g1_i1_len=2477	METK2_CATRO	Q:1710-532,H:1-393	100%ID	E:0	S-adenosylmethionine synthase 2	PF00438.17^S-AdoMet_synt_N^S-adenosylmethionine synthetase, N-terminal domain^16-113^E:6.8e-43`PF02772.13^S-AdoMet_synt_M^S-adenosylmethionine synthetase, central domain^129-250^E:4.8e-46`PF02773.13^S-AdoMet_synt_C^S-adenosylmethionine synthetase, C-terminal domain^252-393^E:1.4e-62	0,004	0,866	0	2,635	0,359	-0,288	0,055	-0,591	0,912	-0,087	METK2_CATRO	S-adenosylmethionine synthase 2 (AdoMet synthase 2) (EC 2.5.1.6) (Methionine adenosyltransferase 2) (MAT 2)	SAMS2	Catharanthus roseus (Madagascar periwinkle) (Vinca rosea)	PATHWAY: Amino-acid biosynthesis; S-adenosyl-L-methionine biosynthesis; S-adenosyl-L-methionine from L-methionine: step 1/1.	ATP-binding; Cobalt; Cytoplasm; Magnesium; Manganese; Metal-binding; Nucleotide-binding; One-carbon metabolism; Potassium; Transferase
SRR648707_TR25740_c0_g1_i3_len=846	NA	NA	NA	NA	NA	.	0,002	-1,261	0	7,548	1	-0,849	1	-1,551	0,001	-1,079	.	.	.	.	.	.
SRR648707_TR26428_c1_g2_i5_len=1903	GCL1_ARATH	Q:1678-395,H:1-433	63.86%ID	E:0	LanC-like protein GCL1	PF05147.10^LANC_like^Lanthionine synthetase C-like protein^71-427^E:1.2e-85	0,076	2,889	0	6,683	1	3,354	0,502	4,006	0,001	-4,472	GCL1_ARATH	LanC-like protein GCL1 (G protein-coupled receptor 2-like protein 1) (Protein GCR2-like 1)	GCL1 At5g65280 MQN23.23	Arabidopsis thaliana (Mouse-ear cress)		Complete proteome; Reference proteome
SRR648707_TR26579_c0_g2_i2_len=3527	PMA4_NICPL	Q:339-2402,H:3-690	91.28%ID	E:0	Plasma membrane ATPase 4	PF00690.23^Cation_ATPase_N^Cation transporter/ATPase, N-terminus^21-84^E:1.7e-14`PF00122.17^E1-E2_ATPase^E1-E2 ATPase^103-323^E:8e-61`PF00702.23^Hydrolase^haloacid dehalogenase-like hydrolase^328-605^E:1.2e-18	0,219	0,396	0	1,284	0,59	-0,153	0,085	-0,465	0,139	-0,575	PMA4_NICPL	Plasma membrane ATPase 4 (EC 3.6.3.6) (Proton pump 4)	PMA4	Nicotiana plumbaginifolia (Leadwort-leaved tobacco) (Tex-Mex tobacco)		ATP-binding; Cell membrane; Hydrogen ion transport; Hydrolase; Ion transport; Magnesium; Membrane; Metal-binding; Nucleotide-binding; Phosphoprotein; Transmembrane; Transmembrane helix; Transport
SRR648707_TR27191_c0_g1_i1_len=1699	C8D11_ARATH	Q:720-1307,H:304-497	55.84%ID	E:3e-70	Cytochrome P450 81D11	PF00067.19^p450^Cytochrome P450^14-191^E:1.5e-62	0,002	2,482	0,001	2,813	0,254	2,681	0,254	2,395	0,76	-0,574	C8D11_ARATH	Cytochrome P450 81D11 (EC 1.14.-.-)	CYP81D11 At3g28740 T19N8.3	Arabidopsis thaliana (Mouse-ear cress)		Complete proteome; Heme; Iron; Membrane; Metal-binding; Monooxygenase; Oxidoreductase; Reference proteome; Transmembrane; Transmembrane helix
SRR648707_TR27191_c0_g1_i2_len=1985	C81D1_ARATH	Q:166-1617,H:25-502	41.75%ID	E:2e-128	Cytochrome P450 81D1	PF00067.19^p450^Cytochrome P450^31-479^E:8.9e-98	0,015	0,931	0	3,529	0,132	0,751	0,675	0,32	0,287	0,355	C81D1_ARATH	Cytochrome P450 81D1 (EC 1.14.-.-)	CYP81D1 CYP91A1 At5g36220 T30G6.3	Arabidopsis thaliana (Mouse-ear cress)		Complete proteome; Heme; Iron; Membrane; Metal-binding; Monooxygenase; Oxidoreductase; Reference proteome; Transmembrane; Transmembrane helix
SRR648707_TR28453_c0_g1_i2_len=1993	PYRD_ARATH	Q:479-1642,H:70-458	84.32%ID	E:0	Dihydroorotate dehydrogenase (quinone), mitochondrial	PF01180.18^DHO_dh^Dihydroorotate dehydrogenase^124-442^E:2.5e-99	0,472	-0,741	0	6,784	0,381	1,379	1	0,017	0,189	-2,039	PYRD_ARATH	Dihydroorotate dehydrogenase (quinone), mitochondrial (DHOdehase) (EC 1.3.5.2) (Dihydroorotate oxidase)	PYRD At5g23300 MKD15.16	Arabidopsis thaliana (Mouse-ear cress)	PATHWAY: Pyrimidine metabolism; UMP biosynthesis via de novo pathway; orotate from (S)-dihydroorotate (quinone route): step 1/1.	Complete proteome; FMN; Flavoprotein; Membrane; Mitochondrion; Mitochondrion inner membrane; Oxidoreductase; Pyrimidine biosynthesis; Reference proteome; Transit peptide; Transmembrane; Transmembrane helix
SRR648707_TR28521_c0_g2_i3_len=1909	GUAA_HELHP	Q:1103-1483,H:846-972	42.52%ID	E:5e-37	Probable GMP synthase [glutamine-hydrolyzing]	PF03352.10^Adenine_glyco^Methyladenine glycosylase^179-228^E:6.9e-17	1	0,078	0	7,253	1	0	1	0	0,698	0,222	GUAA_HELHP	Probable GMP synthase [glutamine-hydrolyzing] (EC 6.3.5.2) (GMP synthetase) (Glutamine amidotransferase)	guaA HH_1444	Helicobacter hepaticus (strain ATCC 51449 / 3B1)	PATHWAY: Purine metabolism; GMP biosynthesis; GMP from XMP (L-Gln route): step 1/1.	ATP-binding; Complete proteome; GMP biosynthesis; Glutamine amidotransferase; Ligase; Nucleotide-binding; Purine biosynthesis; Reference proteome; Repeat
SRR648707_TR28569_c0_g2_i2_len=667	NA	NA	NA	NA	NA	.	0,189	1,685	0	4,815	0,001	3,909	0,629	1,695	1	-0,23	.	.	.	.	.	.
SRR648707_TR28758_c0_g2_i1_len=1680	CDA1_ARATH	Q:371-1369,H:3-298	53.29%ID	E:2e-103	Cytidine deaminase 1	PF00383.20^dCMP_cyt_deam_1^Cytidine and deoxycytidylate deaminase zinc-binding region^38-117^E:9.3e-11`PF08211.9^dCMP_cyt_deam_2^Cytidine and deoxycytidylate deaminase zinc-binding region^153-319^E:2.4e-48	0,5	0,599	0	2,372	1	-0,504	0,692	-0,898	1	-0,329	CDA1_ARATH	Cytidine deaminase 1 (At-CDA1) (EC 3.5.4.5)	CDA1 CDD DESZ At2g19570 F3P11	Arabidopsis thaliana (Mouse-ear cress)		Complete proteome; Hydrolase; Metal-binding; Reference proteome; Zinc
SRR648707_TR30433_c0_g1_i2_len=1559	10HGO_CATRO	Q:138-1229,H:10-360	59.89%ID	E:3e-130	8-hydroxygeraniol dehydrogenase	PF08240.9^ADH_N^Alcohol dehydrogenase GroES-like domain^74-197^E:4e-23`PF02826.16^2-Hacid_dh_C^D-isomer specific 2-hydroxyacid dehydrogenase, NAD binding domain^228-277^E:4.3e-07`PF00107.23^ADH_zinc_N^Zinc-binding dehydrogenase^244-366^E:1.1e-17	0,049	0,575	0	1,556	0,065	0,708	0,918	0,091	0,003	0,822	10HGO_CATRO	8-hydroxygeraniol dehydrogenase (Cr10HGO) (EC 1.1.1.324)	10HGO	Catharanthus roseus (Madagascar periwinkle) (Vinca rosea)		Metal-binding; NADP; Oxidoreductase; Zinc
SRR648707_TR30984_c0_g2_i1_len=710	NA	NA	NA	NA	NA	PF00582.23^Usp^Universal stress protein family^5-134^E:4.8e-18	0,357	0,694	0	2,607	0,006	1,709	0,019	1,492	0,01	1,735	.	.	.	.	.	.
SRR648707_TR31917_c0_g2_i4_len=1953	KPRS3_SPIOL	Q:376-1425,H:40-392	79.15%ID	E:0	Ribose-phosphate pyrophosphokinase 3, mitochondrial	PF13793.3^Pribosyltran_N^N-terminal domain of ribose phosphate pyrophosphokinase^93-206^E:1.1e-06`PF00156.24^Pribosyltran^Phosphoribosyl transferase domain^298-342^E:6.9e-10`PF14572.3^Pribosyl_synth^Phosphoribosyl synthetase-associated domain^301-353^E:1.7e-06	0,298	0,547	0	2,927	0,5	-0,614	0,5	-0,618	0,886	0,183	KPRS3_SPIOL	Ribose-phosphate pyrophosphokinase 3, mitochondrial (EC 2.7.6.1) (Phosphoribosyl pyrophosphate synthase 3)	PRS3	Spinacia oleracea (Spinach)		ATP-binding; Kinase; Magnesium; Metal-binding; Mitochondrion; Nucleotide biosynthesis; Nucleotide-binding; Transferase; Transit peptide
SRR648707_TR32826_c2_g6_i5_len=753	TRPA2_ARATH	Q:753-16,H:74-290	71.54%ID	E:9e-109	Tryptophan synthase alpha chain, chloroplastic	PF00290.17^Trp_syntA^Tryptophan synthase alpha chain^1-197^E:1.3e-76	0,18	0,935	0,001	1,797	0,84	-0,192	0,489	0,511	1	0,316	TRPA2_ARATH	Tryptophan synthase alpha chain, chloroplastic (EC 4.2.1.20) (Indole-3-glycerol-phosphate lyase, chloroplastic) (EC 4.1.2.8) (Protein TRYPTOPHAN-REQUIRING 3)	TSA1 TRP3 TSA2 At3g54640 T14E10.210	Arabidopsis thaliana (Mouse-ear cress)	PATHWAY: Amino-acid biosynthesis; L-tryptophan biosynthesis; L-tryptophan from chorismate: step 5/5.	Amino-acid biosynthesis; Aromatic amino acid biosynthesis; Auxin biosynthesis; Chloroplast; Complete proteome; Lyase; Plastid; Reference proteome; Transit peptide; Tryptophan biosynthesis
SRR648707_TR33272_c1_g1_i10_len=4156	TRPX_ARATH	Q:250-1674,H:26-508	71.64%ID	E:0	Anthranilate synthase alpha subunit 2, chloroplastic	PF04715.10^Anth_synt_I_N^Anthranilate synthase component I, N terminal region^104-258^E:1.4e-30`PF00425.15^Chorismate_bind^chorismate binding enzyme^319-502^E:1.8e-60	0,066	1,731	0	3,272	0,002	4,064	0,009	3,766	1	0,071	TRPX_ARATH	Anthranilate synthase alpha subunit 2, chloroplastic (EC 4.1.3.27) (Anthranilate synthase component 1-2) (Anthranilate synthase component I-2)	ASA2 At2g29690 T27A16.21	Arabidopsis thaliana (Mouse-ear cress)	PATHWAY: Amino-acid biosynthesis; L-tryptophan biosynthesis; L-tryptophan from chorismate: step 1/5.	Amino-acid biosynthesis; Aromatic amino acid biosynthesis; Chloroplast; Complete proteome; Lyase; Plastid; Reference proteome; Transit peptide; Tryptophan biosynthesis
SRR648707_TR33880_c0_g1_i1_len=1560	NA	NA	NA	NA	NA	PF03168.10^LEA_2^Late embryogenesis abundant protein^115-218^E:1.6e-14	0,178	0,662	0	1,637	0,614	0,176	0,438	0,271	0,464	-0,339	.	.	.	.	.	.
SRR648707_TR34356_c0_g1_i5_len=1944	NA	NA	NA	NA	NA	PF01494.16^FAD_binding_3^FAD binding domain^7-325^E:1e-18`PF05834.9^Lycopene_cycl^Lycopene cyclase protein^7-171^E:2.5e-08`PF00890.21^FAD_binding_2^FAD binding domain^7-42^E:3.4e-05`PF08491.7^SE^Squalene epoxidase^238-328^E:4.7e-05	0,282	1,028	0	3,086	0,189	1,776	0,692	1,153	1	-0,777	.	.	.	.	.	.
SRR648707_TR34828_c0_g1_i1_len=2647	NA	NA	NA	NA	NA	.	0,407	0,686	0	2,858	0,01	2,053	0,282	1,12	0,132	-2,299	.	.	.	.	.	.
SRR648707_TR35326_c0_g1_i1_len=688	NA	NA	NA	NA	NA	.	0,56	0,932	0	2,779	1	0,224	0,257	0,9	0,694	-0,329	.	.	.	.	.	.
SRR648707_TR37824_c0_g1_i1_len=988	NA	NA	NA	NA	NA	PF00582.23^Usp^Universal stress protein family^76-217^E:2.4e-20	0,381	1,534	0	4,715	0,003	4,282	0,629	2,075	0,629	0,994	.	.	.	.	.	.
SRR648707_TR39531_c0_g1_i1_len=1067	WRK75_ARATH	Q:426-701,H:54-145	90.22%ID	E:1e-54	Probable WRKY transcription factor 75	PF03106.12^WRKY^WRKY DNA -binding domain^109-166^E:1.2e-26	1	3,447	0	5,982	0,009	5,373	0,129	4,195	1	0,042	WRK75_ARATH	Probable WRKY transcription factor 75 (WRKY DNA-binding protein 75)	WRKY75 At5g13080 T19L5.40	Arabidopsis thaliana (Mouse-ear cress)		Complete proteome; DNA-binding; Nucleus; Reference proteome; Transcription; Transcription regulation
SRR648707_TR40343_c0_g1_i1_len=5818	CLU_ARATH	Q:2306-3595,H:788-1258	38.87%ID	E:5e-89	Clustered mitochondria protein	PF15044.3^CLU_N^Mitochondrial function, CLU-N-term^49-120^E:9.3e-08`PF12807.4^eIF3_p135^Translation initiation factor eIF3 subunit 135^726-867^E:5.8e-25`PF13424.3^TPR_12^Tetratricopeptide repeat^907-968^E:2.2e-08`PF13424.3^TPR_12^Tetratricopeptide repeat^937-1007^E:5.5e-13`PF13374.3^TPR_10^Tetratricopeptide repeat^937-978^E:9.3e-09`PF13181.3^TPR_8^Tetratricopeptide repeat^938-969^E:0.11`PF13424.3^TPR_12^Tetratricopeptide repeat^1021-1095^E:1e-10`PF13374.3^TPR_10^Tetratricopeptide repeat^1021-1060^E:1e-06	0,18	-0,407	0	6,151	1	-0,34	1	-0,385	0,149	0,415	CLU_ARATH	Clustered mitochondria protein (Friendly mitochondria protein)	FMT CLU At3g52140 F4F15.250	Arabidopsis thaliana (Mouse-ear cress)		Alternative splicing; Complete proteome; Cytoplasm; Reference proteome; Repeat; TPR repeat
SRR924147_TR951_c0_g1_i1_len=1152	NA	NA	NA	NA	NA	.	0,798	0,524	0,001	1,635	0,044	2,222	0,118	1,932	1	-1,182	.	.	.	.	.	.
SRR924147_TR951_c0_g1_i2_len=1075	NA	NA	NA	NA	NA	PF02162.14^XYPPX^XYPPX repeat (two copies)^44-57^E:0.066`PF02162.14^XYPPX^XYPPX repeat (two copies)^59-73^E:0.0033	0,29	0,464	0,001	1,053	0,002	1,645	0,009	1,421	1	-0,447	.	.	.	.	.	.
SRR924147_TR1368_c0_g3_i1_len=2107	ARGD_ALNGL	Q:400-1740,H:7-448	72.83%ID	E:0	Acetylornithine aminotransferase, mitochondrial	PF00202.18^Aminotran_3^Aminotransferase class-III^88-462^E:1e-113	0,833	0,11	0,001	1,448	0,269	0,436	0,12	0,601	0,926	-0,068	ARGD_ALNGL	Acetylornithine aminotransferase, mitochondrial (ACOAT) (EC 2.6.1.11) (Acetylornithine transaminase) (AOTA)	AG118	Alnus glutinosa (European alder) (Betula alnus var. glutinosa)	PATHWAY: Amino-acid biosynthesis; L-arginine biosynthesis; N(2)-acetyl-L-ornithine from L-glutamate: step 4/4.	Amino-acid biosynthesis; Aminotransferase; Arginine biosynthesis; Mitochondrion; Pyridoxal phosphate; Transferase; Transit peptide
SRR924147_TR1444_c0_g1_i1_len=530	NA	NA	NA	NA	NA	PF17060.2^MPS2^Monopolar spindle protein 2^37-102^E:3e-06	1	-0,022	0	5,437	0,38	-0,581	0,077	0,766	0,692	1,182	.	.	.	.	.	.
SRR924147_TR2166_c0_g1_i2_len=2233	GDI1_ARATH	Q:280-1587,H:1-436	85.78%ID	E:0	Guanosine nucleotide diphosphate dissociation inhibitor 1	PF00996.15^GDI^GDP dissociation inhibitor^1-433^E:6.1e-232	1	-0,011	0,001	1,104	0,204	0,4	0,267	0,352	0,04	0,585	GDI1_ARATH	Guanosine nucleotide diphosphate dissociation inhibitor 1 (AtGDI1)	GDI1 At2g44100 F6E13.23	Arabidopsis thaliana (Mouse-ear cress)		Alternative splicing; Complete proteome; GTPase activation; Reference proteome
SRR924147_TR2487_c0_g1_i1_len=3547	ISPH_ARATH	Q:3334-1949,H:1-466	78.16%ID	E:0	4-hydroxy-3-methylbut-2-enyl diphosphate reductase, chloroplastic	PF01554.15^MatE^MatE^45-205^E:2.4e-29`PF01554.15^MatE^MatE^266-429^E:2.7e-26	0,015	0,726	0	1,85	0,068	0,434	0,003	0,697	0,62	0,485	ISPH_ARATH	4-hydroxy-3-methylbut-2-enyl diphosphate reductase, chloroplastic (EC 1.17.1.2) (Protein CHLOROPLAST BIOGENESIS 6)	ISPH CLB6 HDR At4g34350 F10M10.120	Arabidopsis thaliana (Mouse-ear cress)	PATHWAY: Isoprenoid biosynthesis; dimethylallyl diphosphate biosynthesis; dimethylallyl diphosphate from (2E)-4-hydroxy-3-methylbutenyl diphosphate: step 1/1.; PATHWAY: Isoprenoid biosynthesis; isopentenyl diphosphate biosynthesis via DXP pathway; isopentenyl diphosphate from 1-deoxy-D-xylulose 5-phosphate: step 6/6.	3Fe-4S; Chloroplast; Complete proteome; Iron; Iron-sulfur; Isoprene biosynthesis; Metal-binding; NADP; Oxidoreductase; Plastid; Reference proteome; Transit peptide
SRR924147_TR2631_c0_g1_i1_len=1272	ADF1_PETHY	Q:888-472,H:1-139	92.09%ID	E:7e-75	Actin-depolymerizing factor 1	PF00241.17^Cofilin_ADF^Cofilin/tropomyosin-type actin-binding protein^14-136^E:4.9e-36	0,617	0,186	0	1,211	0,24	0,539	0,328	0,458	0,366	0,277	ADF1_PETHY	Actin-depolymerizing factor 1 (ADF-1)	ADF1	Petunia hybrida (Petunia)		Actin-binding
SRR924147_TR2824_c0_g1_i1_len=2585	PTR18_ARATH	Q:1479-2348,H:295-590	74.5%ID	E:4e-147	Protein NRT1/ PTR FAMILY 3.1	PF00854.18^PTR2^POT family^6-231^E:1.1e-49	0,006	1,937	0	2,474	0,487	0,284	0,212	-0,55	1	0,007	PTR18_ARATH	Protein NRT1/ PTR FAMILY 3.1 (AtNPF3.1)	NPF3.1 NITR At1g68570 F24J5.19	Arabidopsis thaliana (Mouse-ear cress)		Complete proteome; Membrane; Nitrate assimilation; Reference proteome; Transmembrane; Transmembrane helix; Transport
SRR924147_TR2926_c0_g3_i1_len=1249	RS12_HORVU	Q:443-802,H:24-143	82.5%ID	E:7e-56	40S ribosomal protein S12	PF01248.23^Ribosomal_L7Ae^Ribosomal protein L7Ae/L30e/S12e/Gadd45 family^25-118^E:3.6e-28	0,001	-1,096	0	1,623	0,84	0,267	1	-0,047	0,066	-0,56	RS12_HORVU	40S ribosomal protein S12	RPS12	Hordeum vulgare (Barley)		Ribonucleoprotein; Ribosomal protein
SRR924147_TR3939_c0_g2_i2_len=2395	MAOX_VITVI	Q:318-2090,H:1-591	85.11%ID	E:0	NADP-dependent malic enzyme	PF00390.16^malic^Malic enzyme, N-terminal domain^117-296^E:8.3e-79`PF03949.12^Malic_M^Malic enzyme, NAD binding domain^307-560^E:2.3e-99	0,798	0,1	0,001	0,986	0,042	1,839	0,158	1,407	0,909	0,058	MAOX_VITVI	NADP-dependent malic enzyme (NADP-ME) (EC 1.1.1.40)		Vitis vinifera (Grape)		Cytoplasm; Metal-binding; NADP; Oxidoreductase
SRR924147_TR7686_c0_g1_i1_len=2673	HSP7M_PHAVU	Q:2371-398,H:21-675	87.86%ID	E:0	Heat shock 70 kDa protein, mitochondrial	PF00012.17^HSP70^Hsp70 protein^59-651^E:5.8e-274`PF06723.10^MreB_Mbl^MreB/Mbl protein^189-425^E:7.2e-11	0,03	-0,773	0	1,641	0,34	0,561	0,77	0,227	0,066	0,687	HSP7M_PHAVU	Heat shock 70 kDa protein, mitochondrial		Phaseolus vulgaris (Kidney bean) (French bean)		ATP-binding; Mitochondrion; Nucleotide-binding; Stress response; Transit peptide
SRR924147_TR9531_c0_g2_i4_len=1714	UPTG2_SOLTU	Q:302-1375,H:4-361	89.11%ID	E:0	Alpha-1,4-glucan-protein synthase [UDP-forming] 2	PF03214.10^RGP^Reversibly glycosylated polypeptide^10-344^E:4.3e-180	0,918	0,049	0	1,396	0,218	0,403	1	0,02	1	0,002	UPTG2_SOLTU	Alpha-1,4-glucan-protein synthase [UDP-forming] 2 (EC 2.4.1.-) (Reversibly glycosylated polypeptide 2) (RGP2) (UDP-glucose:protein transglucosylase 2) (UPTG 2)	UPTG2	Solanum tuberosum (Potato)		Cell junction; Cell wall; Cell wall biogenesis/degradation; Cellulose biosynthesis; Complete proteome; Direct protein sequencing; Glycoprotein; Glycosyltransferase; Golgi apparatus; Reference proteome; Secreted; Transferase
SRR924147_TR10322_c0_g2_i1_len=2784	C94A1_VICSA	Q:2568-1165,H:45-512	66.67%ID	E:0	Cytochrome P450 94A1	PF00067.19^p450^Cytochrome P450^86-491^E:6.1e-65	1	1,86	0	6,579	0,502	2,323	0,502	2,714	1	0,577	C94A1_VICSA	Cytochrome P450 94A1 (EC 1.14.-.-) (P450-dependent fatty acid omega-hydroxylase)	CYP94A1 VAGH111	Vicia sativa (Spring vetch) (Tare)		Endoplasmic reticulum; Heme; Iron; Membrane; Metal-binding; Monooxygenase; Oxidoreductase; Transmembrane; Transmembrane helix
SRR924147_TR10387_c0_g1_i1_len=1888	ELO3L_ARATH	Q:1373-522,H:5-288	63.99%ID	E:1e-119	Elongation of fatty acids protein 3-like	PF01151.15^ELO^GNS1/SUR4 family^31-278^E:4.6e-32	0,133	1,091	0	2,461	0,094	1,372	0,187	1,221	0,692	1,005	ELO3L_ARATH	Elongation of fatty acids protein 3-like (Protein ELO3-like) (EC 2.3.1.-) (Protein HIGH EXPRESSION OF OSMOTICALLY RESPONSIVE GENES 3) (Very long-chain fatty acid condensing enzyme HOS3) (VLCFA condensing enzyme HOS3)	HOS3 At4g36830 AP22.81 C7A10.530	Arabidopsis thaliana (Mouse-ear cress)		Complete proteome; Fatty acid biosynthesis; Fatty acid metabolism; Lipid biosynthesis; Lipid metabolism; Membrane; Reference proteome; Stress response; Transferase; Transmembrane; Transmembrane helix
SRR924147_TR15342_c0_g1_i3_len=992	CYB5_BRAOB	Q:613-215,H:2-134	60.9%ID	E:2e-42	Cytochrome b5	PF00173.25^Cyt-b5^Cytochrome b5-like Heme/Steroid binding domain^10-81^E:7.1e-27	0,017	1,347	0	3,586	0,016	1,808	0,038	1,681	0,321	0,395	CYB5_BRAOB	Cytochrome b5	CYB5	Brassica oleracea var. botrytis (Cauliflower)		Direct protein sequencing; Electron transport; Endoplasmic reticulum; Heme; Iron; Membrane; Metal-binding; Microsome; Transmembrane; Transmembrane helix; Transport
SRR924147_TR19983_c3_g5_i1_len=2871	NA	NA	NA	NA	NA	PF03222.10^Trp_Tyr_perm^Tryptophan/tyrosine permease family^28-264^E:2.3e-05`PF01490.15^Aa_trans^Transmembrane amino acid transporter protein^31-427^E:2.5e-50	0,572	0,264	0	1,834	0,57	0,36	0,882	0,138	0,294	0,554	.	.	.	.	.	.
SRR924147_TR20336_c0_g1_i1_len=2313	MTHR2_ARATH	Q:2139-358,H:1-594	81.48%ID	E:0	Methylenetetrahydrofolate reductase 2	PF02219.14^MTHFR^Methylenetetrahydrofolate reductase^6-301^E:3.1e-125	0,033	0,941	0	1,695	1	-0,031	0,897	0,104	0,299	1,228	MTHR2_ARATH	Methylenetetrahydrofolate reductase 2 (AtMTHFR2) (EC 1.5.1.20)	MTHFR2 At2g44160 F6E13.29	Arabidopsis thaliana (Mouse-ear cress)	PATHWAY: One-carbon metabolism; tetrahydrofolate interconversion.	Complete proteome; FAD; Flavoprotein; NAD; Oxidoreductase; Reference proteome
SRR924147_TR20576_c0_g2_i1_len=3385	SD18_ARATH	Q:212-2707,H:23-850	58.11%ID	E:0	Receptor-like serine/threonine-protein kinase SD1-8	PF01453.21^B_lectin^D-mannose binding lectin^70-177^E:2.6e-37`PF00954.17^S_locus_glycop^S-locus glycoprotein domain^208-316^E:1.9e-38`PF08276.8^PAN_2^PAN-like domain^338-403^E:6.7e-22`PF12398.5^DUF3660^Receptor serine/threonine kinase^487-518^E:1.6e-05`PF00069.22^Pkinase^Protein kinase domain^529-729^E:2.1e-45`PF07714.14^Pkinase_Tyr^Protein tyrosine kinase^531-797^E:5.1e-51`PF11883.5^DUF3403^Domain of unknown function (DUF3403)^801-847^E:1.5e-20	0,395	0,592	0	2,264	0,112	1,129	0,381	0,623	0,643	0,54	SD18_ARATH	Receptor-like serine/threonine-protein kinase SD1-8 (EC 2.7.11.1) (Arabidopsis thaliana receptor kinase 3) (S-domain-1 (SD1) receptor kinase 8) (SD1-8)	SD18 ARK3 At4g21380 T6K22.110	Arabidopsis thaliana (Mouse-ear cress)		ATP-binding; Cell membrane; Complete proteome; Disulfide bond; EGF-like domain; Glycoprotein; Kinase; Lectin; Membrane; Nucleotide-binding; Receptor; Reference proteome; Serine/threonine-protein kinase; Signal; Transferase; Transmembrane; Transmembrane helix
SRR924147_TR21288_c0_g2_i1_len=1253	GSTF_HYOMU	Q:795-298,H:48-212	68.67%ID	E:2e-79	Glutathione S-transferase	PF00043.22^GST_C^Glutathione S-transferase, C-terminal domain^9-103^E:2.9e-16`PF13410.3^GST_C_2^Glutathione S-transferase, C-terminal domain^31-97^E:4.5e-09`PF14497.3^GST_C_3^Glutathione S-transferase, C-terminal domain^37-104^E:9.6e-06	0,027	1,249	0	3,733	0,564	0,415	0,694	0,406	1	1,932	GSTF_HYOMU	Glutathione S-transferase (EC 2.5.1.18) (25 kDa auxin-binding protein) (GST class-phi)		Hyoscyamus muticus (Egyptian henbane)		Auxin signaling pathway; Direct protein sequencing; Transferase
SRR924147_TR21288_c0_g2_i3_len=1328	GSTF_HYOMU	Q:870-298,H:48-212	59.69%ID	E:1e-73	Glutathione S-transferase	PF00043.22^GST_C^Glutathione S-transferase, C-terminal domain^48-141^E:7e-16`PF13410.3^GST_C_2^Glutathione S-transferase, C-terminal domain^69-135^E:9.3e-09`PF14497.3^GST_C_3^Glutathione S-transferase, C-terminal domain^75-142^E:2.1e-05	0,732	0,934	0	7,1	0,066	5,212	0,034	5,687	1	-3,061	GSTF_HYOMU	Glutathione S-transferase (EC 2.5.1.18) (25 kDa auxin-binding protein) (GST class-phi)		Hyoscyamus muticus (Egyptian henbane)		Auxin signaling pathway; Direct protein sequencing; Transferase
SRR924147_TR21851_c0_g1_i1_len=874	GOLS2_SOLLC	Q:708-1,H:1-235	84.75%ID	E:2e-148	Galactinol synthase 2	PF01501.17^Glyco_transf_8^Glycosyl transferase family 8^28-236^E:6.4e-31	0,027	1,301	0	3,19	0,001	1,503	0,007	1,295	0,035	1,69	GOLS2_SOLLC	Galactinol synthase 2 (GolS-2) (SlGolS2) (EC 2.4.1.123)	GOLS2	Solanum lycopersicum (Tomato) (Lycopersicon esculentum)		Carbohydrate metabolism; Complete proteome; Cytoplasm; Galactose metabolism; Glycosyltransferase; Manganese; Metal-binding; Reference proteome; Transferase
SRR924147_TR22490_c0_g3_i1_len=2880	TLC1_SOLTU	Q:2283-409,H:1-631	83.68%ID	E:0	Plastidic ATP/ADP-transporter	PF03219.11^TLC^TLC ATP/ADP transporter^100-569^E:5.5e-196	0,361	0,255	0	1,904	0,195	1,302	0,282	1,144	1	-0,271	TLC1_SOLTU	Plastidic ATP/ADP-transporter		Solanum tuberosum (Potato)		ATP-binding; Chloroplast; Complete proteome; Membrane; Nucleotide-binding; Plastid; Reference proteome; Transmembrane; Transmembrane helix; Transport
SRR924147_TR26041_c0_g1_i1_len=1004	NA	NA	NA	NA	NA	PF00847.17^AP2^AP2 domain^72-121^E:2.2e-14	1	3,59	0	9,052	0,005	6,181	0,129	5,14	1	0	.	.	.	.	.	.
SRR924147_TR27129_c0_g1_i1_len=956	NA	NA	NA	NA	NA	PF07816.8^DUF1645^Protein of unknown function (DUF1645)^138-244^E:3.1e-12	0,239	1,294	0	5,093	0,002	3,166	0,118	2,14	1	-0,025	.	.	.	.	.	.
SRR924147_TR27325_c0_g1_i1_len=1436	IX15L_ARATH	Q:242-1150,H:1-303	51.76%ID	E:3e-96	Protein IRX15-LIKE	PF04669.10^Polysacc_synt_4^Polysaccharide biosynthesis^104-295^E:1.2e-67	0,882	0,149	0	2,099	0,593	0,73	0,228	1,176	0,545	-0,511	IX15L_ARATH	Protein IRX15-LIKE	IRX15-L At5g67210 K21H1.17	Arabidopsis thaliana (Mouse-ear cress)		Complete proteome; Golgi apparatus; Membrane; Reference proteome; Transmembrane; Transmembrane helix
SRR924147_TR27992_c1_g2_i13_len=2026	RD23D_ARATH	Q:1751-729,H:1-340	61.96%ID	E:2e-119	Ubiquitin receptor RAD23d	PF11976.5^Rad60-SLD^Ubiquitin-2 like Rad60 SUMO-like^1-73^E:1.5e-05`PF00240.20^ubiquitin^Ubiquitin family^3-76^E:2e-19`PF13881.3^Rad60-SLD_2^Ubiquitin-2 like Rad60 SUMO-like^3-77^E:0.0012`PF00627.28^UBA^UBA/TS-N domain^146-181^E:1.8e-12`PF09280.8^XPC-binding^XPC-binding domain^252-307^E:1.7e-23	0,312	0,593	0	2,174	0,402	0,88	0,56	0,815	1	0	RD23D_ARATH	Ubiquitin receptor RAD23d (AtRAD23d) (Putative DNA repair protein RAD23-4) (RAD23-like protein 4) (AtRAD23-4)	RAD23D RAD23 RAD23-4 At5g38470 MXI10.20	Arabidopsis thaliana (Mouse-ear cress)		Alternative splicing; Complete proteome; Cytoplasm; DNA damage; DNA repair; Nucleus; Reference proteome; Repeat
SRR924147_TR28083_c0_g1_i1_len=1834	NA	NA	NA	NA	NA	PF06813.10^Nodulin-like^Nodulin-like^1-155^E:2.8e-47	1	-0,002	0	3,791	1	0,315	1	0,471	0,694	-0,389	.	.	.	.	.	.
SRR924147_TR29398_c0_g1_i1_len=2346	YTHD2_MOUSE	Q:1362-1922,H:382-565	48.17%ID	E:8e-53	YTH domain-containing family protein 2 {ECO:0000305}	PF04146.12^YTH^YT521-B-like domain^346-487^E:2.4e-35	0,517	-1,202	0	7,361	1	0	1	0	0,04	-0,901	YTHD2_MOUSE	YTH domain-containing family protein 2	Ythdf2	Mus musculus (Mouse)		Acetylation; Complete proteome; Cytoplasm; Phosphoprotein; RNA-binding; Reference proteome
SRR924147_TR29753_c0_g1_i1_len=1829	SERB2_ARATH	Q:1582-356,H:18-421	79.81%ID	E:0	Phosphoserine aminotransferase 2, chloroplastic	PF00266.16^Aminotran_5^Aminotransferase class-V^65-412^E:1.1e-56	0,272	0,496	0	2,259	0,319	1,012	0,593	0,668	0,182	-0,508	SERB2_ARATH	Phosphoserine aminotransferase 2, chloroplastic (AtPSAT2) (EC 2.6.1.52)	PSAT2 At2g17630 T19E12.3	Arabidopsis thaliana (Mouse-ear cress)	PATHWAY: Amino-acid biosynthesis; L-serine biosynthesis; L-serine from 3-phospho-D-glycerate: step 2/3.	Acetylation; Amino-acid biosynthesis; Aminotransferase; Chloroplast; Complete proteome; Plastid; Pyridoxal phosphate; Reference proteome; Serine biosynthesis; Transferase; Transit peptide
SRR924147_TR30885_c0_g1_i2_len=3400	SKI11_ARATH	Q:1496-2836,H:1-467	64.74%ID	E:0	F-box/kelch-repeat protein SKIP11	PF01344.22^Kelch_1^Kelch motif^228-274^E:7.2e-10`PF13418.3^Kelch_4^Galactose oxidase, central domain^228-273^E:1.2e-06`PF01344.22^Kelch_1^Kelch motif^276-323^E:1.1e-11`PF13964.3^Kelch_6^Kelch motif^276-322^E:5.8e-06	0,663	0,508	0	2,564	0,195	0,911	0,195	0,927	1	0	SKI11_ARATH	F-box/kelch-repeat protein SKIP11 (SKP1-interacting partner 11)	SKIP11 At2g02870 T17M13.4	Arabidopsis thaliana (Mouse-ear cress)	PATHWAY: Protein modification; protein ubiquitination.	Complete proteome; Kelch repeat; Nucleus; Reference proteome; Repeat; Ubl conjugation pathway
SRR924147_TR31368_c0_g1_i1_len=3900	PLDA1_TOBAC	Q:3737-1311,H:1-808	85.17%ID	E:0	Phospholipase D alpha 1	PF00168.27^C2^C2 domain^10-126^E:2.3e-13`PF00614.19^PLDc^Phospholipase D Active site motif^327-365^E:1.4e-10`PF13091.3^PLDc_2^PLD-like domain^506-690^E:3.4e-07`PF00614.19^PLDc^Phospholipase D Active site motif^656-682^E:1.6e-07`PF12357.5^PLD_C^Phospholipase D C terminal^727-799^E:5.2e-28	0,876	0,056	0	1,139	0,931	0,052	0,717	-0,172	1	-0,02	PLDA1_TOBAC	Phospholipase D alpha 1 (PLD alpha 1) (EC 3.1.4.4) (Choline phosphatase 1) (Phosphatidylcholine-hydrolyzing phospholipase D 1)	PLD1	Nicotiana tabacum (Common tobacco)		Calcium; Hydrolase; Lipid degradation; Lipid metabolism; Repeat
SRR924147_TR31625_c0_g1_i1_len=5143	EDR1_ARATH	Q:1385-483,H:649-930	37.5%ID	E:8e-48	Serine/threonine-protein kinase EDR1	PF00564.21^PB1^PB1 domain^213-296^E:4.1e-19`PF00069.22^Pkinase^Protein kinase domain^1001-1261^E:6.1e-49`PF07714.14^Pkinase_Tyr^Protein tyrosine kinase^1002-1263^E:7.6e-64	0,152	0,894	0	7,602	0,683	-0,255	1	0,063	0,017	2,186	EDR1_ARATH	Serine/threonine-protein kinase EDR1 (EC 2.7.11.1) (MAPKK kinase EDR1) (Protein ENHANCED DISEASE RESISTANCE 1) (AtEDR1) (Serine/threonine/tyrosine-protein kinase 10)	EDR1 STY10 At1g08720 F22O13.20	Arabidopsis thaliana (Mouse-ear cress)		ATP-binding; Abscisic acid signaling pathway; Complete proteome; Endoplasmic reticulum; Endosome; Ethylene signaling pathway; Golgi apparatus; Kinase; Nucleotide-binding; Nucleus; Phosphoprotein; Plant defense; Reference proteome; Serine/threonine-protein kinase; Transferase
SRR924147_TR33837_c0_g1_i2_len=2666	AB1F_ARATH	Q:2353-686,H:40-595	85.61%ID	E:0	ABC transporter F family member 1	PF00005.24^ABC_tran^ABC transporter^100-254^E:2.3e-22`PF12848.4^ABC_tran_Xtn^ABC transporter^293-365^E:2.9e-23`PF00005.24^ABC_tran^ABC transporter^409-540^E:1.5e-22`PF13304.3^AAA_21^AAA domain, putative AbiEii toxin, Type IV TA system^474-570^E:3.2e-06	0,648	-0,229	0	1,633	0,082	0,859	0,756	0,246	1	0,511	AB1F_ARATH	ABC transporter F family member 1 (ABC transporter ABCF.1) (AtABCF1) (GCN20-type ATP-binding cassette protein GCN1)	ABCF1 GCN1 At5g60790 MAE1.10	Arabidopsis thaliana (Mouse-ear cress)		ATP-binding; Complete proteome; Nucleotide-binding; Reference proteome; Repeat; Transport; Ubl conjugation
SRR924147_TR35628_c2_g1_i1_len=1609	MOMT_CATRO	Q:1222-179,H:1-348	80.17%ID	E:0	Myricetin O-methyltransferase	PF08100.8^Dimerisation^Dimerisation domain^27-74^E:4.9e-20`PF00891.15^Methyltransf_2^O-methyltransferase^116-334^E:3.2e-56	0,611	0,353	0	3,238	0,56	0,686	1	0,209	1	-0,223	MOMT_CATRO	Myricetin O-methyltransferase (EC 2.1.1.267) (CrOMT2) (Flavonoid O-methyltransferase)		Catharanthus roseus (Madagascar periwinkle) (Vinca rosea)		Acetylation; Direct protein sequencing; Methyltransferase; S-adenosyl-L-methionine; Transferase
SRR924147_TR37762_c0_g2_i1_len=1712	PUMP5_ARATH	Q:734-1324,H:115-311	81.22%ID	E:7e-120	Mitochondrial uncoupling protein 5	PF00153.24^Mito_carr^Mitochondrial carrier protein^2-95^E:2.3e-19`PF00153.24^Mito_carr^Mitochondrial carrier protein^103-193^E:1.7e-19	0,732	0,798	0,001	2,474	0,044	-0,864	0,182	-0,585	0,637	0,211	PUMP5_ARATH	Mitochondrial uncoupling protein 5 (AtPUMP5) (Mitochondrial dicarboxylate carrier 1)	PUMP5 DIC1 UCP5 At2g22500 F14M13.10	Arabidopsis thaliana (Mouse-ear cress)		Complete proteome; Membrane; Mitochondrion; Mitochondrion inner membrane; Reference proteome; Repeat; Stress response; Transmembrane; Transmembrane helix; Transport
SRR924147_TR38371_c0_g3_i1_len=668	U91A1_ARATH	Q:666-223,H:313-466	51.92%ID	E:2e-48	UDP-glycosyltransferase 91A1	PF00201.15^UDPGT^UDP-glucoronosyl and UDP-glucosyl transferase^19-131^E:9.8e-14	0,009	6,052	0,001	3,289	0,043	3,256	0,132	2,955	0,299	-1,393	U91A1_ARATH	UDP-glycosyltransferase 91A1 (EC 2.4.1.-)	UGT91A1 At2g22590 T9I22.3	Arabidopsis thaliana (Mouse-ear cress)		Complete proteome; Glycosyltransferase; Reference proteome; Transferase
SRR924147_TR38826_c0_g1_i1_len=1249	SDF2_ARATH	Q:948-313,H:1-215	77.21%ID	E:1e-119	Stromal cell-derived factor 2-like protein	PF02815.16^MIR^MIR domain^66-198^E:1.9e-14	1	-0,028	0	5,744	1	-1,022	1	-1,113	1	0,23	SDF2_ARATH	Stromal cell-derived factor 2-like protein (AtSDF2) (SDF2-like protein)	SDF2 At2g25110 F13D4.70	Arabidopsis thaliana (Mouse-ear cress)		3D-structure; Complete proteome; Endoplasmic reticulum; Glycoprotein; Plant defense; Reference proteome; Repeat; Signal
SRR924147_TR38993_c0_g2_i1_len=4095	ACA10_ARATH	Q:3633-415,H:10-1069	72.44%ID	E:0	Calcium-transporting ATPase 10, plasma membrane-type	PF12515.5^CaATP_NAI^Ca2+-ATPase N terminal autoinhibitory domain^41-82^E:7.1e-16`PF00690.23^Cation_ATPase_N^Cation transporter/ATPase, N-terminus^152-219^E:5.7e-12`PF00122.17^E1-E2_ATPase^E1-E2 ATPase^239-485^E:4.4e-44`PF00702.23^Hydrolase^haloacid dehalogenase-like hydrolase^491-816^E:5.3e-19`PF13246.3^Cation_ATPase^Cation transport ATPase (P-type)^554-628^E:1.4e-14`PF00689.18^Cation_ATPase_C^Cation transporting ATPase, C-terminus^887-1064^E:2e-48	1	-0,011	0	1,936	0,666	0,28	0,153	0,685	1	-0,514	ACA10_ARATH	Calcium-transporting ATPase 10, plasma membrane-type (EC 3.6.3.8) (Ca(2+)-ATPase isoform 10)	ACA10 At4g29900 F27B13.140	Arabidopsis thaliana (Mouse-ear cress)		ATP-binding; Acetylation; Calcium; Calcium transport; Calmodulin-binding; Complete proteome; Hydrolase; Ion transport; Magnesium; Membrane; Metal-binding; Nucleotide-binding; Reference proteome; Transmembrane; Transmembrane helix; Transport
SRR924148_TR263_c0_g3_i1_len=1052	PRX2B_ARATH	Q:232-717,H:1-162	81.48%ID	E:1e-94	Peroxiredoxin-2B	PF08534.7^Redoxin^Redoxin^83-236^E:2.3e-34`PF00578.18^AhpC-TSA^AhpC/TSA family^83-198^E:3.7e-12	0,7	-0,092	0,001	0,807	0,055	0,526	0,08	0,489	0,036	-0,543	PRX2B_ARATH	Peroxiredoxin-2B (EC 1.11.1.15) (Peroxiredoxin IIB) (Peroxiredoxin TPx1) (Thioredoxin reductase 2B) (Thioredoxin-dependent peroxidase 1)	PRXIIB TPX1 At1g65980 F12P19.14	Arabidopsis thaliana (Mouse-ear cress)		Alternative splicing; Antioxidant; Complete proteome; Cytoplasm; Disulfide bond; Oxidoreductase; Peroxidase; Redox-active center; Reference proteome
						PF11976.5^Rad60-SLD^Ubiquitin-2 like Rad60 SUMO-like^1-71^E:9.9e-16`PF00240.20^ubiquitin^Ubiquitin family^3-74^E:1.5e-33`PF14560.3^Ubiquitin_2^Ubiquitin-like domain^14-69^E:0.00068`PF13881.3^Rad60-SLD_2^Ubiquitin-2 like Rad60 SUMO-like^14-71^E:0.16`PF11976.5^Rad60-SLD^Ubiquitin-2 like Rad60 SUMO-like^77-147^E:9.9e-16`PF00240.20^ubiquitin^Ubiquitin family^79-150^E:1.5e-33`PF14560.3^Ubiquitin_2^Ubiquitin-like domain^88-145^E:0.00061`PF13881.3^Rad60-SLD_2^Ubiquitin-																Alternative splicing; Complete proteome; Cytoplasm; Isopeptide bond;

SRR924148_TR4911_c1_g2_i3_len=1404	UBQ14_ARATH	Q:201-1115,H:1-305	100%ID	E:0	Polyubiquitin 14	2 like Rad60 SUMO-like^89-147^E:0.15`PF11976.5^Rad60-SLD^Ubiquitin-2 like Rad60 SUMO-like^153-223^E:9.9e-16`PF00240.20^ubiquitin^Ubiquitin family^155-226^E:1.5e-33`PF14560.3^Ubiquitin_2^Ubiquitin-like domain^164-221^E:0.00061`PF13881.3^Rad60-SLD_2^Ubiquitin-2 like Rad60 SUMO-like^165-223^E:0.15`PF11976.5^Rad60-SLD^Ubiquitin-2 like Rad60 SUMO-like^229-299^E:9.9e-16`PF00240.20^ubiquitin^Ubiquitin family^231-302^E:1.5e-33`PF14560.3^Ubiquitin_2^Ubiquitin-like domain^240-297^E:0.00061`PF13881.3^Rad60-SLD_2^Ubiquitin-2 like Rad60 SUMO-like^241-299^E:0.15	0,774	0,072	0	0,984	0,003	0,701	0,089	0,416	0,118	0,413	UBQ14_ARATH	Polyubiquitin 14 [Cleaved into: Ubiquitin]	UBQ14 At4g02890 T5J8.21	Arabidopsis thaliana (Mouse-ear cress)		Alternative splicing; Complete proteome; Cytoplasm; Isopeptide bond; Nucleus; Reference proteome; Repeat; Ubl conjugation; Ubl conjugation pathway
SRR924148_TR7529_c0_g1_i4_len=1635	MTDH_FRAAN	Q:193-1251,H:4-356	70.25%ID	E:1e-156	Probable mannitol dehydrogenase	PF08240.9^ADH_N^Alcohol dehydrogenase GroES-like domain^39-152^E:1.2e-27`PF00107.23^ADH_zinc_N^Zinc-binding dehydrogenase^195-317^E:2e-18	0,448	0,182	0	3,707	0,852	0,064	0,122	-0,472	0,012	0,605	MTDH_FRAAN	Probable mannitol dehydrogenase (EC 1.1.1.255) (NAD-dependent mannitol dehydrogenase)	CAD	Fragaria ananassa (Strawberry)		Metal-binding; NAD; Oxidoreductase; Zinc
SRR924148_TR12807_c0_g7_i1_len=994	NA	NA	NA	NA	NA	.	0,257	0,346	0	2,851	0,003	1,165	0,135	0,62	0,552	-0,146	.	.	.	.	.	.
SRR924148_TR13374_c0_g2_i3_len=2798	TCMO_CATRO	Q:2157-1645,H:137-307	98.25%ID	E:8e-166	Trans-cinnamate 4-monooxygenase	PF00067.19^p450^Cytochrome P450^34-137^E:5.6e-24	1	-0,144	0	3,974	0,431	0,322	0,139	0,574	1	0,122	TCMO_CATRO	Trans-cinnamate 4-monooxygenase (EC 1.14.13.11) (Cinnamic acid 4-hydroxylase) (C4H) (CA4H) (Cytochrome P450 73) (Cytochrome P450C4H)	CYP73A4	Catharanthus roseus (Madagascar periwinkle) (Vinca rosea)	PATHWAY: Phenylpropanoid metabolism; trans-4-coumarate biosynthesis; trans-4-coumarate from trans-cinnamate: step 1/1.	Heme; Iron; Metal-binding; Monooxygenase; NADP; Oxidoreductase
SRR924148_TR14715_c0_g1_i2_len=1791	MOMT_CATRO	Q:1303-263,H:1-348	73.85%ID	E:1e-178	Myricetin O-methyltransferase	PF08100.8^Dimerisation^Dimerisation domain^27-74^E:8.4e-20`PF00891.15^Methyltransf_2^O-methyltransferase^116-333^E:5.1e-56	0,431	0,409	0	2,323	0,402	1,059	0,56	0,997	1	-0,139	MOMT_CATRO	Myricetin O-methyltransferase (EC 2.1.1.267) (CrOMT2) (Flavonoid O-methyltransferase)		Catharanthus roseus (Madagascar periwinkle) (Vinca rosea)		Acetylation; Direct protein sequencing; Methyltransferase; S-adenosyl-L-methionine; Transferase
SRR924148_TR15049_c0_g2_i1_len=1789	WTR18_ARATH	Q:690-1607,H:64-365	58.6%ID	E:7e-109	WAT1-related protein At3g28050	PF00892.17^EamA^EamA-like transporter family^137-260^E:5.3e-08	0,027	2,315	0	2,941	0,014	3,386	0,132	2,734	1	0,025	WTR18_ARATH	WAT1-related protein At3g28050	At3g28050 MMG15.6	Arabidopsis thaliana (Mouse-ear cress)		Complete proteome; Membrane; Reference proteome; Repeat; Transmembrane; Transmembrane helix
SRR924148_TR15095_c0_g1_i2_len=1072	NCPR_CATRO	Q:66-860,H:450-714	99.62%ID	E:0	NADPH–cytochrome P450 reductase {ECO:0000255|HAMAP-Rule:MF_03212}	PF00667.17^FAD_binding_1^FAD binding domain^1-64^E:4.7e-21`PF00175.18^NAD_binding_1^Oxidoreductase NAD-binding domain^101-211^E:2.2e-17	0,111	0,843	0	1,519	0,001	0,959	1	0,022	0,008	-0,836	NCPR_CATRO	NADPH–cytochrome P450 reductase (CPR) (P450R) (EC 1.6.2.4)	CPR	Catharanthus roseus (Madagascar periwinkle) (Vinca rosea)		Endoplasmic reticulum; FAD; FMN; Flavoprotein; Membrane; NADP; Oxidoreductase
SRR924148_TR15241_c0_g1_i1_len=1429	ARR3_ARATH	Q:1050-514,H:1-175	66.67%ID	E:9e-67	Two-component response regulator ARR3	PF00072.21^Response_reg^Response regulator receiver domain^34-154^E:1.6e-21	0,729	0,242	0,001	1,648	0,798	-0,2	0,904	0,121	1	-0,208	ARR3_ARATH	Two-component response regulator ARR3	ARR3 At1g59940 F23H11.25	Arabidopsis thaliana (Mouse-ear cress)		Complete proteome; Cytokinin signaling pathway; Nucleus; Phosphoprotein; Reference proteome; Transcription; Transcription regulation; Two-component regulatory system
SRR924148_TR16153_c1_g1_i1_len=3622	COB22_ARATH	Q:3333-562,H:1-926	84.02%ID	E:0	Coatomer subunit beta'-2	PF00400.29^WD40^WD domain, G-beta repeat^91-127^E:8e-06`PF00400.29^WD40^WD domain, G-beta repeat^133-171^E:0.00013`PF00400.29^WD40^WD domain, G-beta repeat^177-215^E:5.6e-06`PF00400.29^WD40^WD domain, G-beta repeat^220-256^E:6.6e-09`PF04053.11^Coatomer_WDAD^Coatomer WD associated region^319-763^E:7.2e-167	1	0,006	0	2,883	0	-2,642	0	-2,99	0,547	-0,414	COB22_ARATH	Coatomer subunit beta'-2 (Beta'-coat protein 2) (Beta'-COP 2)	At1g52360 F19K6.16	Arabidopsis thaliana (Mouse-ear cress)		Alternative splicing; Complete proteome; Cytoplasm; Cytoplasmic vesicle; ER-Golgi transport; Golgi apparatus; Membrane; Protein transport; Reference proteome; Repeat; Transport; WD repeat
SRR924148_TR16153_c1_g1_i4_len=3587	COB22_ARATH	Q:3298-527,H:1-926	84.02%ID	E:0	Coatomer subunit beta'-2	PF00400.29^WD40^WD domain, G-beta repeat^91-127^E:8e-06`PF00400.29^WD40^WD domain, G-beta repeat^133-171^E:0.00013`PF00400.29^WD40^WD domain, G-beta repeat^177-215^E:5.6e-06`PF00400.29^WD40^WD domain, G-beta repeat^220-256^E:6.6e-09`PF04053.11^Coatomer_WDAD^Coatomer WD associated region^319-763^E:7.2e-167	0,724	-0,203	0,001	5,566	0	-5,874	0	-4,508	0,679	-0,432	COB22_ARATH	Coatomer subunit beta'-2 (Beta'-coat protein 2) (Beta'-COP 2)	At1g52360 F19K6.16	Arabidopsis thaliana (Mouse-ear cress)		Alternative splicing; Complete proteome; Cytoplasm; Cytoplasmic vesicle; ER-Golgi transport; Golgi apparatus; Membrane; Protein transport; Reference proteome; Repeat; Transport; WD repeat
SRR924148_TR17798_c0_g1_i1_len=2057	ODPA1_ARATH	Q:207-1391,H:1-389	81.27%ID	E:0	Pyruvate dehydrogenase E1 component subunit alpha-1, mitochondrial	PF00676.17^E1_dh^Dehydrogenase E1 component^139-434^E:4.4e-122`PF02775.18^TPP_enzyme_C^Thiamine pyrophosphate enzyme, C-terminal TPP binding domain^225-340^E:3.8e-05`PF13292.3^DXP_synthase_N^1-deoxy-D-xylulose-5-phosphate synthase^237-301^E:7.1e-05	0,79	0,15	0	1,951	0,739	0,143	0,802	0,117	0,317	0,534	ODPA1_ARATH	Pyruvate dehydrogenase E1 component subunit alpha-1, mitochondrial (PDHE1-A) (EC 1.2.4.1)	E1 ALPHA At1g59900 F23H11.21	Arabidopsis thaliana (Mouse-ear cress)		Complete proteome; Glycolysis; Mitochondrion; Oxidoreductase; Pyruvate; Reference proteome; Thiamine pyrophosphate; Transit peptide
SRR924148_TR19161_c0_g1_i2_len=3665	MACP2_ARATH	Q:643-2346,H:15-598	66.1%ID	E:0	MACPF domain-containing protein At4g24290	PF01823.16^MACPF^MAC/Perforin domain^110-311^E:2.1e-36	0,87	0,163	0	3,923	0,038	1,698	0,038	1,619	0,025	3,307	MACP2_ARATH	MACPF domain-containing protein At4g24290	At4g24290 T22A6.120	Arabidopsis thaliana (Mouse-ear cress)		Alternative splicing; Complete proteome; Hypersensitive response; Immunity; Innate immunity; Plant defense; Reference proteome
SRR924148_TR20718_c0_g2_i1_len=2035	COBRA_ARATH	Q:1784-588,H:35-435	83.29%ID	E:0	Protein COBRA	PF04833.12^COBRA^COBRA-like protein^56-219^E:1.1e-73	0,099	0,473	0	1,211	0,847	0,06	0,881	-0,066	0,781	-0,541	COBRA_ARATH	Protein COBRA (Cell expansion protein)	COB At5g60920 MSL3.40	Arabidopsis thaliana (Mouse-ear cress)		Cell membrane; Complete proteome; GPI-anchor; Glycoprotein; Lipoprotein; Membrane; Reference proteome; Signal
SRR924148_TR21272_c0_g1_i2_len=1671	UPTG2_SOLTU	Q:1388-348,H:2-348	93.37%ID	E:0	Alpha-1,4-glucan-protein synthase [UDP-forming] 2	PF03214.10^RGP^Reversibly glycosylated polypeptide^13-347^E:4.4e-180	0,522	0,274	0	2,007	0,019	1,277	0,505	0,45	1	-0,47	UPTG2_SOLTU	Alpha-1,4-glucan-protein synthase [UDP-forming] 2 (EC 2.4.1.-) (Reversibly glycosylated polypeptide 2) (RGP2) (UDP-glucose:protein transglucosylase 2) (UPTG 2)	UPTG2	Solanum tuberosum (Potato)		Cell junction; Cell wall; Cell wall biogenesis/degradation; Cellulose biosynthesis; Complete proteome; Direct protein sequencing; Glycoprotein; Glycosyltransferase; Golgi apparatus; Reference proteome; Secreted; Transferase
SRR924148_TR21806_c0_g1_i1_len=1148	RNLE_SOLLC	Q:324-983,H:11-230	63.8%ID	E:8e-101	Extracellular ribonuclease LE	PF00445.15^Ribonuclease_T2^Ribonuclease T2 family^28-202^E:1.1e-59	0,066	5,267	0	8,773	0,009	4	0,066	3,173	1	-0,543	RNLE_SOLLC	Extracellular ribonuclease LE (RNase LE) (EC 3.1.27.1)		Solanum lycopersicum (Tomato) (Lycopersicon esculentum)		3D-structure; Cell wall; Complete proteome; Direct protein sequencing; Disulfide bond; Endonuclease; Hydrolase; Nuclease; Reference proteome; Secreted; Signal; Stress response
SRR924148_TR25216_c0_g1_i1_len=2005	6GPD3_ARATH	Q:1829-384,H:5-486	89.42%ID	E:0	6-phosphogluconate dehydrogenase, decarboxylating 3	PF03446.12^NAD_binding_2^NAD binding domain of 6-phosphogluconate dehydrogenase^6-178^E:9.7e-47`PF00393.16^6PGD^6-phosphogluconate dehydrogenase, C-terminal domain^183-476^E:3.1e-126	0,771	0,081	0	1,216	0,052	0,566	0,018	0,679	0,511	0,264	6GPD3_ARATH	6-phosphogluconate dehydrogenase, decarboxylating 3 (EC 1.1.1.44)	At3g02360 F11A12.5	Arabidopsis thaliana (Mouse-ear cress)	PATHWAY: Carbohydrate degradation; pentose phosphate pathway; D-ribulose 5-phosphate from D-glucose 6-phosphate (oxidative stage): step 3/3.	Complete proteome; Cytoplasm; Gluconate utilization; NADP; Oxidoreductase; Pentose shunt; Reference proteome
SRR924148_TR25216_c0_g1_i2_len=2009	6GPD3_ARATH	Q:1829-384,H:5-486	89.42%ID	E:0	6-phosphogluconate dehydrogenase, decarboxylating 3	PF03446.12^NAD_binding_2^NAD binding domain of 6-phosphogluconate dehydrogenase^6-178^E:9.7e-47`PF00393.16^6PGD^6-phosphogluconate dehydrogenase, C-terminal domain^183-476^E:3.1e-126	0,76	0,599	0	5,792	0,129	3,912	0,034	4,336	0,132	-2,569	6GPD3_ARATH	6-phosphogluconate dehydrogenase, decarboxylating 3 (EC 1.1.1.44)	At3g02360 F11A12.5	Arabidopsis thaliana (Mouse-ear cress)	PATHWAY: Carbohydrate degradation; pentose phosphate pathway; D-ribulose 5-phosphate from D-glucose 6-phosphate (oxidative stage): step 3/3.	Complete proteome; Cytoplasm; Gluconate utilization; NADP; Oxidoreductase; Pentose shunt; Reference proteome
SRR924148_TR26351_c1_g1_i1_len=2411	HSP7C_PETHY	Q:165-2111,H:1-651	92.2%ID	E:0	Heat shock cognate 70 kDa protein	PF00012.17^HSP70^Hsp70 protein^9-618^E:7.6e-270`PF06723.10^MreB_Mbl^MreB/Mbl protein^142-380^E:4.4e-14	0,065	1,441	0	4,376	0,006	1,045	0,003	1,116	0,001	1,974	HSP7C_PETHY	Heat shock cognate 70 kDa protein	HSP70	Petunia hybrida (Petunia)		ATP-binding; Nucleotide-binding; Stress response
SRR924148_TR27417_c0_g3_i1_len=1194	YKT61_ARATH	Q:828-229,H:1-199	86.5%ID	E:3e-126	VAMP-like protein YKT61	PF13774.3^Longin^Regulated-SNARE-like domain^49-112^E:1.3e-10`PF00957.18^Synaptobrevin^Synaptobrevin^138-195^E:2.5e-15	0,211	0,787	0	1,986	0,268	0,419	0,337	0,369	0,679	0,383	YKT61_ARATH	VAMP-like protein YKT61 (AtYKT61) (Geranylgeranylated protein 1) (AtGP1)	YKT61 At5g58060 K21L19.5 K21L19_40	Arabidopsis thaliana (Mouse-ear cress)		Alternative splicing; Cell membrane; Coiled coil; Complete proteome; Lipoprotein; Membrane; Methylation; Palmitate; Prenylation; Protein transport; Reference proteome; Transport
SRR924148_TR27519_c0_g3_i2_len=1047	PXG4_ARATH	Q:288-818,H:19-189	68.36%ID	E:6e-83	Probable peroxygenase 4	PF05042.10^Caleosin^Caleosin related protein^23-195^E:1.5e-66	0,832	0,327	0	3,164	0,402	1,177	0,402	1,091	0,317	0,519	PXG4_ARATH	Probable peroxygenase 4 (AtPXG4) (EC 1.11.2.3) (Caleosin-4)	PXG4 CLO4 At1g70670 F5A18.15	Arabidopsis thaliana (Mouse-ear cress)		Calcium; Complete proteome; Iron; Lipid droplet; Metal-binding; Oxidoreductase; Phosphoprotein; Reference proteome
SRR924148_TR28419_c0_g3_i1_len=1569	PIP11_ORYSJ	Q:353-1207,H:2-289	85.42%ID	E:1e-178	Aquaporin PIP1-1	PF00230.17^MIP^Major intrinsic protein^45-274^E:8.8e-84	0,889	0,116	0	9,664	0,18	0,801	0,032	1,166	0,254	-3,804	PIP11_ORYSJ	Aquaporin PIP1-1 (OsPIP1;1) (Plasma membrane intrinsic protein 1-1) (Plasma membrane intrinsic protein 1a) (PIP1a) (Water channel protein RWC1) (RWC-1)	PIP1-1 PIP1A RWC1 Os02g0666200 LOC_Os02g44630 OJ1486_E07.10 OsJ_007594 P0461B08.25	Oryza sativa subsp. japonica (Rice)		Cell membrane; Complete proteome; Membrane; Reference proteome; Repeat; Transmembrane; Transmembrane helix; Transport
SRR924148_TR30000_c0_g1_i1_len=1212	PGR5_ARATH	Q:303-677,H:1-133	69.17%ID	E:4e-53	Protein PROTON GRADIENT REGULATION 5, chloroplastic	.	0,268	0,39	0	1,164	0,915	0,027	0,57	0,129	0,622	0,124	PGR5_ARATH	Protein PROTON GRADIENT REGULATION 5, chloroplastic	PGR5 At2g05620 T20G20.3	Arabidopsis thaliana (Mouse-ear cress)		Chloroplast; Complete proteome; Disulfide bond; Electron transport; Membrane; Plastid; Reference proteome; Thylakoid; Transit peptide; Transport
SRR924148_TR30106_c0_g1_i1_len=1893	NA	NA	NA	NA	NA	PF05542.8^DUF760^Protein of unknown function (DUF760)^148-273^E:9.7e-23`PF05542.8^DUF760^Protein of unknown function (DUF760)^337-418^E:0.00052	0,337	0,286	0	1,397	0,1	0,449	0,075	0,484	0,418	0,249	.	.	.	.	.	.
SRR924148_TR30611_c0_g1_i1_len=1898	NA	NA	NA	NA	NA	.	0,025	1,172	0	2,042	0,028	1,567	0,212	1,084	0,377	0,599	.	.	.	.	.	.
SRR924148_TR31965_c0_g1_i1_len=2718	EDR2L_ARATH	Q:390-2510,H:6-712	29.48%ID	E:4e-99	Protein ENHANCED DISEASE RESISTANCE 2-like	PF01852.16^START^START domain^251-416^E:4e-09`PF07059.9^DUF1336^Protein of unknown function (DUF1336)^538-743^E:8e-69	0,438	0,363	0	2,463	0,021	0,973	0,026	0,929	0,419	0,43	EDR2L_ARATH	Protein ENHANCED DISEASE RESISTANCE 2-like	EDR2L At5g45560 MFC19.23	Arabidopsis thaliana (Mouse-ear cress)		Cell membrane; Complete proteome; Endoplasmic reticulum; Endosome; Membrane; Plant defense; Reference proteome; Transmembrane; Transmembrane helix
SRR924148_TR35150_c0_g1_i4_len=1434	QKIL5_ARATH	Q:257-1120,H:11-296	60%ID	E:8e-95	KH domain-containing protein At1g09660/At1g09670	PF16544.2^STAR_dimer^Homodimerisation region of STAR domain protein^34-68^E:2.9e-09	0,038	0,956	0,001	2,095	0,663	0,462	0,049	1,248	0,129	-5,106	QKIL5_ARATH	KH domain-containing protein At1g09660/At1g09670 (Quaking-like protein 5)	At1g09660 F21M12.5 F21M12.6	Arabidopsis thaliana (Mouse-ear cress)		Alternative splicing; Complete proteome; Nucleus; Phosphoprotein; RNA-binding; Reference proteome
SRR924148_TR35505_c0_g1_i1_len=3045	NA	NA	NA	NA	NA	.	0,013	0,784	0	1,261	0,136	0,511	0,046	0,678	1	1,755	.	.	.	.	.	.
SRR924148_TR35571_c0_g1_i1_len=2095	UGDH1_SOYBN	Q:286-1725,H:1-480	92.29%ID	E:0	UDP-glucose 6-dehydrogenase 1	PF03721.11^UDPG_MGDP_dh_N^UDP-glucose/GDP-mannose dehydrogenase family, NAD binding domain^3-184^E:5.3e-66`PF00984.16^UDPG_MGDP_dh^UDP-glucose/GDP-mannose dehydrogenase family, central domain^210-304^E:6.8e-32`PF03720.12^UDPG_MGDP_dh_C^UDP-glucose/GDP-mannose dehydrogenase family, UDP binding domain^328-451^E:1.4e-37	0,265	0,314	0	1,704	0,267	-0,307	0,322	-0,277	0,004	-0,848	UGDH1_SOYBN	UDP-glucose 6-dehydrogenase 1 (UDP-Glc dehydrogenase 1) (UDP-GlcDH 1) (UDPGDH 1) (EC 1.1.1.22) (Gm-UGD1)	UGD1	Glycine max (Soybean) (Glycine hispida)	PATHWAY: Nucleotide-sugar biosynthesis; UDP-alpha-D-glucuronate biosynthesis; UDP-alpha-D-glucuronate from UDP-alpha-D-glucose: step 1/1.	Complete proteome; NAD; Oxidoreductase; Phosphoprotein; Reference proteome
SRR924148_TR37769_c0_g1_i6_len=2562	AGT23_ARATH	Q:228-1733,H:20-481	69.72%ID	E:0	Alanine–glyoxylate aminotransferase 2 homolog 3, mitochondrial	PF00202.18^Aminotran_3^Aminotransferase class-III^3-321^E:2.5e-75	0,189	1,826	0	5,652	0,254	2,918	1	1,843	0,228	1,024	AGT23_ARATH	Alanine–glyoxylate aminotransferase 2 homolog 3, mitochondrial (EC 2.6.1.44) (Beta-alanine-pyruvate aminotransferase 3)	At3g08860 T16O11.21	Arabidopsis thaliana (Mouse-ear cress)		Aminotransferase; Complete proteome; Mitochondrion; Photorespiration; Pyridoxal phosphate; Reference proteome; Transferase; Transit peptide
SRR924148_TR37788_c0_g1_i3_len=1429	NDK4_ARATH	Q:1108-404,H:1-235	76.37%ID	E:2e-123	Nucleoside diphosphate kinase IV, chloroplastic/mitochondrial	PF00334.16^NDK^Nucleoside diphosphate kinase^88-221^E:3.1e-52	0,364	0,272	0	1,475	0,144	0,41	0,213	0,347	0,449	0,225	NDK4_ARATH	Nucleoside diphosphate kinase IV, chloroplastic/mitochondrial (NDK IV) (NDP kinase IV) (NDPK IV) (EC 2.7.4.6) (Nucleoside diphosphate kinase 4)	NDK4 At4g23900 T32A16.70	Arabidopsis thaliana (Mouse-ear cress)		ATP-binding; Chloroplast; Complete proteome; Direct protein sequencing; Kinase; Magnesium; Metal-binding; Mitochondrion; Nucleotide metabolism; Nucleotide-binding; Plastid; Reference proteome; Thylakoid; Transferase; Transit peptide
SRR924148_TR38585_c0_g1_i2_len=2773	NA	NA	NA	NA	NA	.	0,346	0,425	0	1,503	0,429	0,246	0,627	0,161	0,692	1,17	.	.	.	.	.	.
SRR924148_TR39344_c1_g1_i1_len=1978	CALM_LILLO	Q:209-655,H:1-149	100%ID	E:2e-96	Calmodulin	PF00036.29^EF-hand_1^EF hand^12-40^E:2.4e-09`PF13499.3^EF-hand_7^EF-hand domain pair^12-73^E:1.9e-15`PF13405.3^EF-hand_6^EF-hand domain^12-41^E:1e-09`PF13202.3^EF-hand_5^EF hand^14-37^E:6.2e-05`PF13833.3^EF-hand_8^EF-hand domain pair^25-75^E:4.2e-14`PF00036.29^EF-hand_1^EF hand^48-75^E:1.7e-08`PF13202.3^EF-hand_5^EF hand^49-73^E:0.00014`PF13499.3^EF-hand_7^EF-hand domain pair^83-146^E:4.6e-18`PF00036.29^EF-hand_1^EF hand^85-112^E:2e-09`PF13405.3^EF-hand_6^EF-hand domain^85-114^E:1.9e-08`PF12763.4^EF-hand_4^Cytoskeletal-regulatory complex EF hand^85-146^E:4.6e-05`PF13202.3^EF-hand_5^EF hand^86-110^E:1.4e-06`PF13833.3^EF-hand_8^EF-hand domain pair^98-146^E:9.8e-15`PF00036.29^EF-hand_1^EF hand^121-148^E:3.3e-10`PF13202.3^EF-hand_5^EF hand^124-146^E:5.3e-05	0,315	-0,286	0	0,983	0,542	0,164	0,221	0,315	0,541	-0,146	CALM_LILLO	Calmodulin (CaM)		Lilium longiflorum (Trumpet lily)		Acetylation; Calcium; Metal-binding; Methylation; Repeat

MsDamaged24h

Transcript (CDF97)	Uniprot homolog	BLASTX Position	BLASTX %Identity	BLASTX evalue	Uniprot descr	Pfam	PValue_Ms6h	logFC_Ms6h	PValue_Ms8h	logFC_Ms8h	PValue_MsDamaged24h	logFC_MsDamaged24h	PValue_MsIntact24h	logFC_MsIntact24h	PValue_Ms1wk	logFC_Ms1wk	Entry.name	Protein names	Gene names	Organism	Pathway	Keywords
SRR1144633_TR4555_c1_g1_i1_len=968	LOR6_ARATH	Q:841-263,H:4-191	54.64%ID	E:4e-65	Protein LURP-one-related 6	PF04525.9^LOR^LURP-one-related^15-196^E:4.2e-44	1	0,254	1	-1,093	0	6,715	0,129	5,118	1	0	LOR6_ARATH	Protein LURP-one-related 6	At2g05910 T6P5.11	Arabidopsis thaliana (Mouse-ear cress)		Complete proteome; Reference proteome
SRR1144633_TR5319_c0_g1_i2_len=1495	PER12_ARATH	Q:246-1187,H:26-339	63.38%ID	E:3e-147	Peroxidase 12	PF00141.20^peroxidase^Peroxidase^62-303^E:9.4e-70	0,064	0,425	0,002	0,725	0	2,162	0,001	1,296	1	-0,991	PER12_ARATH	Peroxidase 12 (Atperox P12) (EC 1.11.1.7) (ATP4a) (PRXR6)	PER12 P12 At1g71695 F14O23.6 F26A9.5	Arabidopsis thaliana (Mouse-ear cress)		Calcium; Complete proteome; Disulfide bond; Glycoprotein; Heme; Hydrogen peroxide; Iron; Metal-binding; Oxidoreductase; Peroxidase; Reference proteome; Secreted; Signal; Vacuole
SRR1144633_TR18843_c0_g1_i1_len=769	RL361_ARATH	Q:203-484,H:8-101	88.3%ID	E:4e-50	60S ribosomal protein L36-1	.	0,024	-0,583	0,393	0,218	0	1,486	0,006	0,995	0,399	-0,275	RL361_ARATH	60S ribosomal protein L36-1	RPL36A At2g37600 F13M22.10	Arabidopsis thaliana (Mouse-ear cress)		Complete proteome; Reference proteome; Ribonucleoprotein; Ribosomal protein
SRR1144633_TR23923_c0_g1_i2_len=991	QCR7_SOLTU	Q:224-592,H:1-123	82.93%ID	E:3e-55	Cytochrome b-c1 complex subunit 7	PF02271.13^UCR_14kD^Ubiquinol-cytochrome C reductase complex 14kD subunit^17-110^E:2.1e-26	0,472	0,64	0,042	1,699	0	1,475	0,021	0,927	1	1,879	QCR7_SOLTU	Cytochrome b-c1 complex subunit 7 (CR14) (Complex III subunit 7) (Complex III subunit VII) (Ubiquinol-cytochrome c reductase complex 14 kDa protein)		Solanum tuberosum (Potato)		Complete proteome; Direct protein sequencing; Electron transport; Membrane; Mitochondrion; Mitochondrion inner membrane; Reference proteome; Respiratory chain; Transport
SRR1144633_TR24183_c0_g1_i1_len=1194	E134_MAIZE	Q:983-486,H:58-219	37.95%ID	E:2e-30	Endo-1,3	PF01738.15^DLH^Dienelactone hydrolase family^19-227^E:1.3e-28	0,946	0,045	0,319	0,377	0,001	1,221	0,001	1,17	1	-0,614	E134_MAIZE	Endo-1,3;1,4-beta-D-glucanase (EC 3.2.1.-)		Zea mays (Maize)		Complete proteome; Direct protein sequencing; Glycoprotein; Hydrolase; Reference proteome; Secreted; Signal
SRR1144633_TR27508_c1_g2_i1_len=1881	CASL2_CANSA	Q:373-1608,H:127-540	51.44%ID	E:1e-132	Cannabidiolic acid synthase-like 2	PF01565.20^FAD_binding_4^FAD binding domain^16-104^E:1.6e-16`PF08031.9^BBE^Berberine and berberine like^365-423^E:2.2e-21	0,781	0,586	0,066	1,721	0	3,745	0,299	1,772	1	0,362	CASL2_CANSA	Cannabidiolic acid synthase-like 2	CBDAS3	Cannabis sativa (Hemp) (Marijuana)		Disulfide bond; FAD; Flavoprotein; Glycoprotein; Secreted; Signal
SRR1144634_TR3033_c0_g2_i1_len=975	FB237_ARATH	Q:963-484,H:223-380	90.62%ID	E:2e-95	F-box protein At4g18380	.	1	0,147	0,707	0,267	0	7,133	0,001	6,462	0,643	-0,415	FB237_ARATH	F-box protein At4g18380	At4g18380 F28J12.40	Arabidopsis thaliana (Mouse-ear cress)		Complete proteome; Reference proteome
SRR1144634_TR3385_c0_g1_i1_len=875	NA	NA	NA	NA	NA	PF04554.10^Extensin_2^Extensin-like region^126-174^E:0.00016	0,502	2,687	1	2,807	0	3,646	0,001	3,307	1	0	.	.	.	.	.	.
SRR1144634_TR10120_c0_g1_i2_len=950	NA	NA	NA	NA	NA	PF00582.23^Usp^Universal stress protein family^29-174^E:2.1e-26	1	0	0,502	3,788	0	7,095	0,017	5,879	0,412	-0,506	.	.	.	.	.	.
SRR1144634_TR23319_c0_g1_i1_len=2062	APA1_ARATH	Q:268-1806,H:1-506	75.49%ID	E:0	Aspartic proteinase A1	PF00026.20^Asp^Eukaryotic aspartyl protease^131-554^E:4e-136`PF14543.3^TAXi_N^Xylanase inhibitor N-terminal^132-290^E:3.1e-13`PF03489.14^SapB_2^Saposin-like type B, region 2^365-398^E:8.4e-13`PF05184.12^SapB_1^Saposin-like type B, region 1^428-464^E:1.2e-14	0,054	0,584	0,501	0,218	0	1,107	0,022	0,696	0,062	0,608	APA1_ARATH	Aspartic proteinase A1 (EC 3.4.23.-)	APA1 PRA1 At1g11910 F12F1.24	Arabidopsis thaliana (Mouse-ear cress)		Aspartyl protease; Complete proteome; Disulfide bond; Glycoprotein; Hydrolase; Protease; Reference proteome; Repeat; Signal; Vacuole; Zymogen
SRR1144634_TR27168_c0_g1_i1_len=1196	TPRL3_ERYCB	Q:327-1073,H:10-258	80.32%ID	E:1e-138	Tropinone reductase-like 3	PF00106.22^adh_short^short chain dehydrogenase^15-203^E:5.1e-42`PF08659.7^KR^KR domain^18-182^E:7.5e-09`PF13561.3^adh_short_C2^Enoyl-(Acyl carrier protein) reductase^24-253^E:6.1e-59	0,834	0,098	0,001	1,042	0	1,409	0,002	1,165	0,423	-0,373	TPRL3_ERYCB	Tropinone reductase-like 3 (EC 1.1.1.-)		Erythroxylum coca (Coca plant)		Oxidoreductase
SRR1144634_TR38172_c0_g2_i1_len=1307	PUP3_ARATH	Q:111-1052,H:15-331	44.48%ID	E:5e-62	Purine permease 3	PF16913.2^PUNUT^Purine nucleobase transmembrane transport^14-333^E:4.8e-91`PF00892.17^EamA^EamA-like transporter family^69-156^E:2.4e-07	0,022	0,787	0,043	0,687	0	1,393	0,039	0,688	1	0,971	PUP3_ARATH	Purine permease 3 (AtPUP3)	PUP3 At1g28220 F3H9.12	Arabidopsis thaliana (Mouse-ear cress)		Complete proteome; Membrane; Reference proteome; Transmembrane; Transmembrane helix; Transport
SRR122236_TR13132_c1_g1_i1_len=1818	COMT1_CATRO	Q:1222-197,H:19-360	100%ID	E:0	Caffeic acid 3-O-methyltransferase	PF08100.8^Dimerisation^Dimerisation domain^40-91^E:1e-22`PF00891.15^Methyltransf_2^O-methyltransferase^135-351^E:8.2e-90	1	0	0,002	6,365	0	7,382	0,001	6,479	1	0	COMT1_CATRO	Caffeic acid 3-O-methyltransferase (CAOMT) (COMT) (EC 2.1.1.68) (S-adenosysl-L-methionine:caffeic acid 3-O-methyltransferase)	COMT1	Catharanthus roseus (Madagascar periwinkle) (Vinca rosea)	PATHWAY: Aromatic compound metabolism; phenylpropanoid biosynthesis.	Lignin biosynthesis; Methyltransferase; S-adenosyl-L-methionine; Transferase
SRR122236_TR23485_c0_g2_i1_len=1184	PIP23_ARATH	Q:1096-254,H:1-281	82.21%ID	E:2e-168	Aquaporin PIP2-3	PF00230.17^MIP^Major intrinsic protein^58-293^E:2.3e-84	0,066	5,224	0,155	1,102	0	2,025	0,001	1,697	1	0	PIP23_ARATH	Aquaporin PIP2-3 (Plasma membrane intrinsic protein 2-3) (AtPIP2;3) (Plasma membrane intrinsic protein 2c) (PIP2c) (RD28-PIP) (TMP2C) (Water stress-induced tonoplast intrinsic protein) (WSI-TIP) [Cleaved into: Aquaporin PIP2-3, N-terminally processed]	PIP2-3 PIP2C RD28 At2g37180 T2N18.6	Arabidopsis thaliana (Mouse-ear cress)		Acetylation; Cell membrane; Complete proteome; Membrane; Phosphoprotein; Reference proteome; Repeat; Stress response; Transmembrane; Transmembrane helix; Transport
SRR122236_TR25666_c0_g2_i1_len=1473	PTR8_ARATH	Q:1469-207,H:174-585	55.11%ID	E:1e-162	Protein NRT1/ PTR FAMILY 2.9	PF00854.18^PTR2^POT family^5-354^E:2.3e-66	0,502	2,871	0,001	6,507	0	5,055	0,025	3,046	1	1,42	PTR8_ARATH	Protein NRT1/ PTR FAMILY 2.9 (AtNPF2.9) (Nitrate transporter 1.9)	NPF2.9 NRT1.9 At1g18880 F6A14.2	Arabidopsis thaliana (Mouse-ear cress)		Cell membrane; Complete proteome; Membrane; Reference proteome; Transmembrane; Transmembrane helix; Transport
SRR122237_TR2245_c0_g1_i2_len=666	OEP16_PEA	Q:572-135,H:1-146	76.71%ID	E:8e-61	Outer envelope pore protein 16, chloroplastic	PF02466.16^Tim17^Tim17/Tim22/Tim23/Pmp24 family^59-133^E:8.7e-11	0,253	0,328	0,093	-0,476	0,001	0,954	0,018	0,697	0,139	-0,412	OEP16_PEA	Outer envelope pore protein 16, chloroplastic (Chloroplastic outer envelope pore protein of 16 kDa)	OEP16	Pisum sativum (Garden pea)		Chloroplast; Ion transport; Membrane; Plastid; Plastid outer membrane; Porin; Transmembrane; Transmembrane beta strand; Transmembrane helix; Transport
SRR122237_TR27186_c0_g2_i1_len=1066	NA	NA	NA	NA	NA	.	1	0,04	0,265	-0,388	0	1,853	0,1	0,947	0,003	-0,953	.	.	.	.	.	.
SRR122238_TR1242_c0_g1_i2_len=845	NA	NA	NA	NA	NA	PF12023.5^DUF3511^Domain of unknown function (DUF3511)^66-109^E:3.7e-23	0,793	0,185	0,763	0,24	0	1,751	0,019	1,197	0,729	0,34	.	.	.	.	.	.
SRR122238_TR3208_c0_g1_i2_len=884	NA	NA	NA	NA	NA	PF05678.11^VQ^VQ motif^78-104^E:2.1e-12	0,524	0,644	0,226	0,735	0	6,785	0,017	5,871	1	0,577	.	.	.	.	.	.
SRR122238_TR5046_c0_g1_i2_len=1073	UEV1C_ARATH	Q:209-610,H:12-145	92.54%ID	E:6e-84	Ubiquitin-conjugating enzyme E2 variant 1C	PF00179.23^UQ_con^Ubiquitin-conjugating enzyme^43-136^E:2.6e-14	0,93	0,029	0,301	0,263	0	1,126	0,014	0,81	0,917	-0,038	UEV1C_ARATH	Ubiquitin-conjugating enzyme E2 variant 1C (Ubc enzyme variant 1C) (Protein MMS ZWEI HOMOLOG 3)	UEV1C MMZ3 At2g36060 F11F19.3	Arabidopsis thaliana (Mouse-ear cress)		Alternative splicing; Complete proteome; Reference proteome
SRR122238_TR5736_c0_g1_i1_len=569	SBP3_ARATH	Q:521-225,H:1-99	73.74%ID	E:1e-47	Probable steroid-binding protein 3	PF00173.25^Cyt-b5^Cytochrome b5-like Heme/Steroid binding domain^20-101^E:6.2e-15	0,528	0,288	0,474	-0,298	0	1,616	0,003	1,167	0	-2,714	SBP3_ARATH	Probable steroid-binding protein 3 (AtMP3) (Membrane-associated progesterone-binding protein 2) (AtMAPR2)	MP3 MAPR2 At2g24940 F27C12.14	Arabidopsis thaliana (Mouse-ear cress)		3D-structure; Acetylation; Complete proteome; Lipid-binding; Nucleus; Reference proteome; Steroid-binding
SRR122238_TR6557_c0_g1_i2_len=1217	FK201_ARATH	Q:1027-593,H:1-145	80.69%ID	E:6e-79	Peptidyl-prolyl cis-trans isomerase FKBP20-1	PF00254.25^FKBP_C^FKBP-type peptidyl-prolyl cis-trans isomerase^97-181^E:3e-26	1	-0,015	0,931	0,056	0,001	1,311	0,004	1,128	1	-0,055	FK201_ARATH	Peptidyl-prolyl cis-trans isomerase FKBP20-1 (PPIase FKBP20-1) (EC 5.2.1.8) (FK506-binding protein 20-1) (AtFKBP20-1) (Immunophilin FKBP20-1) (Rotamase)	FKBP20-1 FKBP20 At3g55520 T22E16.180	Arabidopsis thaliana (Mouse-ear cress)		Acetylation; Complete proteome; Isomerase; Reference proteome; Rotamase
SRR122238_TR8779_c1_g1_i1_len=697	NA	NA	NA	NA	NA	.	1	0	1	2,147	0,001	5,595	1	1,818	1	0	.	.	.	.	.	.
SRR122238_TR16898_c0_g1_i8_len=517	NA	NA	NA	NA	NA	.	0,015	0,653	0,461	-0,215	0	0,914	0,487	-0,208	0	-0,985	.	.	.	.	.	.
SRR122238_TR17468_c0_g1_i2_len=723	UBQ11_ARATH	Q:1-435,H:85-229	98.62%ID	E:6e-96	Polyubiquitin 11	PF00240.20^ubiquitin^Ubiquitin family^2-66^E:2.1e-29`PF11976.5^Rad60-SLD^Ubiquitin-2 like Rad60 SUMO-like^2-63^E:2.8e-12`PF13881.3^Rad60-SLD_2^Ubiquitin-2 like Rad60 SUMO-like^4-63^E:0.039`PF14560.3^Ubiquitin_2^Ubiquitin-like domain^7-61^E:0.0002`PF11976.5^Rad60-SLD^Ubiquitin-2 like Rad60 SUMO-like^69-139^E:2.1e-16`PF00240.20^ubiquitin^Ubiquitin family^71-142^E:3.1e-34`PF13881.3^Rad60-SLD_2^Ubiquitin-2 like Rad60 SUMO-like^78-139^E:0.036`PF14560.3^Ubiquitin_2^Ubiquitin-like domain^80-137^E:0.00014	0,474	0,153	0,369	0,19	0	0,99	0,022	0,489	0,097	-0,354	UBQ11_ARATH	Polyubiquitin 11 [Cleaved into: Ubiquitin]	UBQ11 At4g05050 C17L7.6 T32N4.13	Arabidopsis thaliana (Mouse-ear cress)		Alternative splicing; Complete proteome; Cytoplasm; Isopeptide bond; Nucleus; Reference proteome; Repeat; Ubl conjugation; Ubl conjugation pathway
SRR122238_TR18052_c0_g1_i2_len=655	NLTP_VIGUN	Q:495-253,H:20-99	57.32%ID	E:4e-23	Probable non-specific lipid-transfer protein AKCS9	.	0,005	0,685	0	-1,142	0,001	0,83	0,001	-0,902	0,003	0,719	NLTP_VIGUN	Probable non-specific lipid-transfer protein AKCS9 (LTP)		Vigna unguiculata (Cowpea)		Disulfide bond; Lipid-binding; Signal; Transport
SRR122238_TR18135_c1_g1_i4_len=532	NA	NA	NA	NA	NA	.	0,403	-0,347	0,004	-0,903	0	7,496	0,001	6,429	0	-8,324	.	.	.	.	.	.
SRR122238_TR19349_c0_g1_i1_len=698	RS212_ARATH	Q:218-460,H:1-81	88.89%ID	E:2e-47	40S ribosomal protein S21-2	.	0,094	-0,497	0,3	0,283	0	1,45	0,007	1,06	0,334	-0,278	RS212_ARATH	40S ribosomal protein S21-2	RPS21C At5g27700 T1G16.30	Arabidopsis thaliana (Mouse-ear cress)		Acetylation; Complete proteome; Reference proteome; Ribonucleoprotein; Ribosomal protein
SRR122238_TR20450_c1_g1_i1_len=1321	PIP1_SOLLC	Q:1165-317,H:1-283	87.28%ID	E:0	Probable aquaporin PIP-type pTOM75	PF00230.17^MIP^Major intrinsic protein^45-274^E:8.9e-85	0,35	0,368	0,015	0,95	0	1,476	0,007	0,84	0,043	-2,743	PIP1_SOLLC	Probable aquaporin PIP-type pTOM75 (Ripening-associated membrane protein) (RAMP)		Solanum lycopersicum (Tomato) (Lycopersicon esculentum)		Cell membrane; Complete proteome; Membrane; Reference proteome; Repeat; Stress response; Transmembrane; Transmembrane helix; Transport
SRR122238_TR22058_c0_g1_i4_len=685	NDUB2_ARATH	Q:605-465,H:11-57	82.98%ID	E:6e-22	NADH dehydrogenase [ubiquinone] 1 beta subcomplex subunit 2	.	0,325	0,286	1	-0,022	0	1,007	0,061	0,566	0,86	0,19	NDUB2_ARATH	NADH dehydrogenase [ubiquinone] 1 beta subcomplex subunit 2	At1g76200 T23E18.30	Arabidopsis thaliana (Mouse-ear cress)		Complete proteome; Electron transport; Membrane; Mitochondrion; Mitochondrion inner membrane; Reference proteome; Respiratory chain; Transport
SRR122238_TR22754_c3_g1_i4_len=793	KIRO_ACTDE	Q:37-486,H:1-150	65.33%ID	E:6e-63	Kirola {ECO:0000303|PubMed:21309790}	PF00407.16^Bet_v_1^Pathogenesis-related protein Bet v I family^14-161^E:5.3e-45	0,519	0,154	0,607	-0,137	0	1,369	0,026	0,583	0,163	0,332	KIRO_ACTDE	Kirola (allergen Act d 11)		Actinidia deliciosa (Kiwi)		3D-structure; Acetylation; Allergen; Direct protein sequencing; Polymorphism
SRR122238_TR22801_c0_g1_i2_len=914	ML423_ARATH	Q:718-278,H:8-152	37.41%ID	E:3e-27	MLP-like protein 423	PF00407.16^Bet_v_1^Pathogenesis-related protein Bet v I family^3-153^E:3.8e-31	0,13	0,652	0,039	1,03	0	3,208	0,042	1,936	1	-0,04	ML423_ARATH	MLP-like protein 423	MLP423 At1g24020 T23E23.17 T23E23_22	Arabidopsis thaliana (Mouse-ear cress)		Complete proteome; Reference proteome
SRR122238_TR22801_c0_g1_i3_len=773	ML423_ARATH	Q:610-170,H:8-152	37.41%ID	E:2e-27	MLP-like protein 423	PF00407.16^Bet_v_1^Pathogenesis-related protein Bet v I family^3-153^E:3.8e-31	0,925	-0,055	0,153	0,776	0	1,987	0,707	0,426	0,004	1,17	ML423_ARATH	MLP-like protein 423	MLP423 At1g24020 T23E23.17 T23E23_22	Arabidopsis thaliana (Mouse-ear cress)		Complete proteome; Reference proteome
SRR122238_TR23534_c0_g1_i4_len=951	ASPRX_ORYSJ	Q:1-690,H:280-496	59.13%ID	E:6e-94	Aspartic proteinase	PF00026.20^Asp^Eukaryotic aspartyl protease^1-229^E:2e-30`PF03489.14^SapB_2^Saposin-like type B, region 2^41-73^E:4.2e-13`PF05184.12^SapB_1^Saposin-like type B, region 1^103-139^E:7.7e-13	0,024	0,799	0,011	-0,728	0	2,241	0,129	0,898	0,464	0,208	ASPRX_ORYSJ	Aspartic proteinase (EC 3.4.23.-)	RAP Os05g0137400 LOC_Os05g04630 OJ1127_B08.13	Oryza sativa subsp. japonica (Rice)		Aspartyl protease; Complete proteome; Disulfide bond; Glycoprotein; Hydrolase; Protease; Reference proteome; Signal; Vacuole; Zymogen
SRR122238_TR27031_c0_g2_i2_len=786	YLS8_ARATH	Q:155-580,H:1-142	97.18%ID	E:9e-101	Thioredoxin-like protein YLS8	PF02966.13^DIM1^Mitosis protein DIM1^41-173^E:8.4e-67`PF00085.17^Thioredoxin^Thioredoxin^54-138^E:6e-06	0,528	-0,191	0,156	0,411	0	1,143	0,006	0,861	0,566	-0,167	YLS8_ARATH	Thioredoxin-like protein YLS8 (Protein YELLOW-LEAF-SPECIFIC GENE 8)	YLS8 At5g08290 F8L15.20	Arabidopsis thaliana (Mouse-ear cress)		Complete proteome; Reference proteome
SRR122238_TR34894_c0_g1_i1_len=505	NA	NA	NA	NA	NA	.	0,502	4,211	1	1,089	0	9,001	0,129	5,044	0,502	2,054	.	.	.	.	.	.
SRR122238_TR40800_c0_g1_i4_len=867	NA	NA	NA	NA	NA	.	0,044	0,978	1	0,075	0	2,11	0,039	1,036	0,298	0,593	.	.	.	.	.	.
SRR122238_TR43175_c3_g3_i1_len=1468	MTDH_PETCR	Q:1226-249,H:10-335	65.95%ID	E:4e-128	Mannitol dehydrogenase	PF00107.23^ADH_zinc_N^Zinc-binding dehydrogenase^69-192^E:2.8e-19`PF13602.3^ADH_zinc_N_2^Zinc-binding dehydrogenase^102-221^E:1.1e-07	0,496	0,272	0,01	0,788	0	1,59	0,02	1,125	0,732	-0,792	MTDH_PETCR	Mannitol dehydrogenase (EC 1.1.1.255) (NAD-dependent mannitol dehydrogenase) (Fragment)	ELI3	Petroselinum crispum (Parsley) (Petroselinum hortense)		Metal-binding; NAD; Oxidoreductase; Zinc
SRR122238_TR43543_c0_g1_i6_len=861	PABN2_ARATH	Q:129-599,H:64-219	86.62%ID	E:2e-82	Polyadenylate-binding protein 2 {ECO:0000303|PubMed:18479511}	PF00076.19^RRM_1^RNA recognition motif. (a.k.a. RRM, RBD, or RNP domain)^50-118^E:1.5e-17`PF13893.3^RRM_5^RNA recognition motif. (a.k.a. RRM, RBD, or RNP domain)^63-121^E:0.019	0,377	-0,576	0,243	-0,566	0	2,692	0,065	1,321	0,468	-0,219	PABN2_ARATH	Polyadenylate-binding protein 2 (AtPabN2) (AtPabN3) (Poly(A)-binding protein 2) (Nuclear poly(A)-binding protein 2) (Poly(A)-binding protein II) (PABII)	PABN2 At5g65260 MQN23.20	Arabidopsis thaliana (Mouse-ear cress)		Coiled coil; Complete proteome; Cytoplasm; Nucleus; RNA-binding; Reference proteome
SRR122238_TR51655_c0_g1_i1_len=902	NA	NA	NA	NA	NA	PF14368.3^LTP_2^Probable lipid transfer^40-135^E:2e-18`PF00234.19^Tryp_alpha_amyl^Protease inhibitor/seed storage/LTP family^59-135^E:0.00018	1	2,411	1	2,922	0	6,443	0,254	3,52	1	1,769	.	.	.	.	.	.
SRR122238_TR52620_c0_g3_i1_len=926	HSP21_SOLPE	Q:190-660,H:1-155	84.81%ID	E:4e-69	17.3 kDa class II heat shock protein	PF00011.18^HSP20^Hsp20/alpha crystallin family^51-154^E:1.8e-30	1	1,259	0,044	2,135	0	1,7	0,086	0,963	1	-1,989	HSP21_SOLPE	17.3 kDa class II heat shock protein (Hsp17.3) (Hsp20.2)		Solanum peruvianum (Peruvian tomato) (Lycopersicon peruvianum)		Cytoplasm; Stress response
SRR122238_TR58412_c1_g1_i2_len=962	NA	NA	NA	NA	NA	PF05340.9^DUF740^Protein of unknown function (DUF740)^8-96^E:1.4e-06	1	0,477	0,005	4,34	0,001	4,527	0,254	2,432	1	1,632	.	.	.	.	.	.
SRR122238_TR58480_c0_g1_i1_len=1072	TIP13_ARATH	Q:121-876,H:1-252	78.97%ID	E:2e-122	Aquaporin TIP1-3	PF00230.17^MIP^Major intrinsic protein^14-234^E:4.2e-74	0,461	1,233	0,001	4,223	0	3,596	0,239	1,264	0,56	0,935	TIP13_ARATH	Aquaporin TIP1-3 (Gamma-tonoplast intrinsic protein 3) (Gamma-TIP3) (Tonoplast intrinsic protein 1-3) (AtTIP1;3) [Cleaved into: Aquaporin TIP1-3, N-terminally processed]	TIP1-3 At4g01470 F11O4.1	Arabidopsis thaliana (Mouse-ear cress)		Acetylation; Complete proteome; Membrane; Reference proteome; Repeat; Stress response; Transmembrane; Transmembrane helix; Transport; Vacuole
SRR122238_TR62477_c0_g2_i1_len=925	MYB4_ORYSJ	Q:76-399,H:1-108	69.44%ID	E:2e-49	Myb-related protein Myb4	PF00249.28^Myb_DNA-binding^Myb-like DNA-binding domain^14-61^E:4.9e-14`PF13921.3^Myb_DNA-bind_6^Myb-like DNA-binding domain^17-76^E:2.5e-12`PF00249.28^Myb_DNA-binding^Myb-like DNA-binding domain^67-110^E:4.1e-16`PF13921.3^Myb_DNA-bind_6^Myb-like DNA-binding domain^71-120^E:2.5e-09	1	0,97	0,066	5,48	0,001	6,653	0,009	6,104	1	-1,044	MYB4_ORYSJ	Myb-related protein Myb4 (OsMyb4) (Transcription factor RLTR1)	MYB4 LTR1 Os04g0517100 LOC_Os04g43680 OSJNBa0073E02.6	Oryza sativa subsp. japonica (Rice)		Complete proteome; DNA-binding; Nucleus; Reference proteome; Repeat; Stress response; Transcription; Transcription regulation
SRR122238_TR63091_c0_g1_i1_len=649	VAS_ARATH	Q:570-235,H:7-117	41.96%ID	E:4e-26	Lipid transfer-like protein VAS	PF14368.3^LTP_2^Probable lipid transfer^32-127^E:1.8e-15`PF00234.19^Tryp_alpha_amyl^Protease inhibitor/seed storage/LTP family^49-124^E:9.4e-07	1	2,794	1	0	0	5,517	1	2,405	1	0	VAS_ARATH	Lipid transfer-like protein VAS	VAS At5g13900 MAC12.14	Arabidopsis thaliana (Mouse-ear cress)		Complete proteome; Lipid-binding; Reference proteome; Signal; Transport
SRR122238_TR67949_c0_g1_i1_len=703	NA	NA	NA	NA	NA	PF06522.8^B12D^NADH-ubiquinone reductase complex 1 MLRQ subunit^63-130^E:1.6e-24	0,427	0,186	0,2	0,304	0,001	0,767	0,003	0,681	0,576	0,155	.	.	.	.	.	.
SRR122238_TR69076_c0_g1_i5_len=833	NA	NA	NA	NA	NA	PF14368.3^LTP_2^Probable lipid transfer^23-104^E:9e-17`PF00234.19^Tryp_alpha_amyl^Protease inhibitor/seed storage/LTP family^27-104^E:4.3e-10	1	0	0,017	5,829	0	9,309	0,017	5,745	0,865	-0,153	.	.	.	.	.	.
SRR122238_TR74472_c0_g1_i8_len=984	NA	NA	NA	NA	NA	PF03061.19^4HBT^Thioesterase superfamily^87-157^E:1.3e-10	0,798	0,364	0,076	1,521	0	3,8	0,076	2,419	1	-0,444	.	.	.	.	.	.
SRR122238_TR74622_c0_g1_i12_len=1853	WSD1_ARATH	Q:1642-293,H:7-468	30.11%ID	E:7e-57	O-acyltransferase WSD1	PF03007.13^WES_acyltransf^Wax ester synthase-like Acyl-CoA acyltransferase domain^105-162^E:2.8e-05`PF06974.10^DUF1298^Protein of unknown function (DUF1298)^305-450^E:4e-40	0,692	1,049	0,066	1,957	0	2,278	0,003	1,998	1	0,646	WSD1_ARATH	O-acyltransferase WSD1 (Diacylglycerol O-acyltransferase) (DGAT) (EC 2.3.1.20) (Long-chain-alcohol O-fatty-acyltransferase) (EC 2.3.1.75) (Wax synthase) (WS)	WSD1 At5g37300 MNJ8.9	Arabidopsis thaliana (Mouse-ear cress)	PATHWAY: Glycerolipid metabolism; triacylglycerol biosynthesis.	Acyltransferase; Complete proteome; Endoplasmic reticulum; Membrane; Reference proteome; Transferase; Transmembrane; Transmembrane helix
SRR122238_TR77088_c0_g1_i1_len=727	H2B_GOSHI	Q:225-533,H:45-147	96.12%ID	E:9e-59	Histone H2B	PF00125.21^Histone^Core histone H2A/H2B/H3/H4^9-124^E:7.3e-23`PF00808.20^CBFD_NFYB_HMF^Histone-like transcription factor (CBF/NF-Y) and archaeal histone^66-122^E:4.4e-05	0,002	-0,732	0,347	-0,236	0	0,988	0,006	0,75	0,034	-0,459	H2B_GOSHI	Histone H2B	HIS2B	Gossypium hirsutum (Upland cotton) (Gossypium mexicanum)		Acetylation; Chromosome; DNA-binding; Isopeptide bond; Nucleosome core; Nucleus; Ubl conjugation
SRR122238_TR77575_c2_g1_i1_len=627	NA	NA	NA	NA	NA	.	0,039	0,781	0,008	1,441	0	2,801	0,006	1,938	0,255	0,686	.	.	.	.	.	.
SRR122238_TR78792_c0_g1_i1_len=890	SCE1_ARATH	Q:802-323,H:1-160	84.38%ID	E:1e-83	SUMO-conjugating enzyme SCE1	PF00179.23^UQ_con^Ubiquitin-conjugating enzyme^9-151^E:4.9e-44	0,769	-0,118	0,007	0,836	0	1,175	0,002	1,039	0,692	0,924	SCE1_ARATH	SUMO-conjugating enzyme SCE1 (EC 6.3.2.-) (Protein EMBRYO DEFECTIVE 1637) (Protein hus5 homolog) (SUMO-conjugating enzyme 1) (AtSCE1)	SCE1 AHUS5 EMB1637 At3g57870 T10K17.80	Arabidopsis thaliana (Mouse-ear cress)	PATHWAY: Protein modification; protein sumoylation.	ATP-binding; Acetylation; Complete proteome; Ligase; Nucleotide-binding; Reference proteome; Ubl conjugation pathway
SRR122238_TR85122_c0_g1_i1_len=882	MTND1_VITVI	Q:90-650,H:1-188	81.38%ID	E:1e-110	1,2-dihydroxy-3-keto-5-methylthiopentene dioxygenase 1 {ECO:0000255|HAMAP-Rule:MF_03154}	PF03079.11^ARD^ARD/ARD' family^34-187^E:1.4e-58`PF02311.16^AraC_binding^AraC-like ligand binding domain^114-186^E:3.2e-07`PF07883.8^Cupin_2^Cupin domain^117-175^E:1.8e-10	0,16	0,612	0,316	0,333	0	0,876	0,001	0,792	0,084	0,6	MTND1_VITVI	1,2-dihydroxy-3-keto-5-methylthiopentene dioxygenase 1 (EC 1.13.11.54) (Acireductone dioxygenase (Fe(2+)-requiring) 1) (ARD 1) (Fe-ARD 1)	VIT_05s0020g04070	Vitis vinifera (Grape)	PATHWAY: Amino-acid biosynthesis; L-methionine biosynthesis via salvage pathway; L-methionine from S-methyl-5-thio-alpha-D-ribose 1-phosphate: step 5/6. {ECO:0000255|HAMAP-Rule:MF_03154}.	Amino-acid biosynthesis; Complete proteome; Cytoplasm; Dioxygenase; Iron; Metal-binding; Methionine biosynthesis; Nucleus; Oxidoreductase; Reference proteome
SRR122238_TR85742_c0_g1_i1_len=681	NA	NA	NA	NA	NA	.	1	-0,576	1	1,036	0	4,059	0,461	1,001	0,517	-0,933	.	.	.	.	.	.
SRR122238_TR87627_c2_g1_i1_len=778	TCTP_ELAGV	Q:699-196,H:1-168	84.52%ID	E:2e-90	Translationally-controlled tumor protein homolog	PF00838.14^TCTP^Translationally controlled tumour protein^27-190^E:2.5e-57	0,763	-0,066	0,257	0,247	0,001	0,711	0,003	0,632	0,729	0,113	TCTP_ELAGV	Translationally-controlled tumor protein homolog (TCTP)	TCTP	Elaeis guineensis var. tenera (Oil palm)		Calcium; Cytoplasm
SRR122238_TR88708_c0_g1_i1_len=899	ALL13_OLEEU	Q:847-176,H:1-226	78.76%ID	E:2e-129	Thaumatin-like protein	PF00314.14^Thaumatin^Thaumatin family^48-241^E:1e-59	0,629	1,12	0,381	2,04	0	5,003	0,043	3,284	1	1,031	ALL13_OLEEU	Thaumatin-like protein (allergen Ole e 13)		Olea europaea (Common olive)		Allergen; Direct protein sequencing; Disulfide bond; Pathogenesis-related protein; Plant defense; Secreted; Signal
SRR1271857_TR5342_c0_g1_i2_len=622	IDHC_SOYBN	Q:620-9,H:238-412	80.88%ID	E:1e-111	Isocitrate dehydrogenase [NADP]	PF00180.17^Iso_dh^Isocitrate/isopropylmalate dehydrogenase^5-145^E:1e-39	0,021	1,078	0,81	-0,098	0,001	6,592	0,502	3,833	0,611	0,418	IDHC_SOYBN	Isocitrate dehydrogenase [NADP] (IDH) (EC 1.1.1.42) (IDP) (NADP(+)-specific ICDH) (Oxalosuccinate decarboxylase)	IDH1	Glycine max (Soybean) (Glycine hispida)		Complete proteome; Cytoplasm; Glyoxylate bypass; Magnesium; Manganese; Metal-binding; NADP; Oxidoreductase; Reference proteome; Tricarboxylic acid cycle
SRR1271857_TR15065_c0_g1_i1_len=1662	DV4H_CATRO	Q:1644-445,H:2-401	100%ID	E:0	Deacetoxyvindoline 4-hydroxylase	PF14226.3^DIOX_N^non-haem dioxygenase in morphine synthesis N-terminal^66-172^E:1.8e-18`PF03171.17^2OG-FeII_Oxy^2OG-Fe(II) oxygenase superfamily^224-314^E:9.5e-23	0,26	0,252	0,065	0,429	0	1,151	0,002	0,717	0,489	0,182	DV4H_CATRO	Deacetoxyvindoline 4-hydroxylase (EC 1.14.11.20)	D4H	Catharanthus roseus (Madagascar periwinkle) (Vinca rosea)	PATHWAY: Alkaloid biosynthesis; vindoline biosynthesis; vindoline from tabersonine: step 5/6.	Cytoplasm; Dioxygenase; Direct protein sequencing; Iron; Metal-binding; Oxidoreductase; Vitamin C
SRR1271857_TR16492_c0_g2_i1_len=545	NA	NA	NA	NA	NA	PF06697.9^DUF1191^Protein of unknown function (DUF1191)^18-91^E:6.2e-18	1	0,126	0,008	-1,304	0,001	6,592	1	0	0,288	-0,404	.	.	.	.	.	.
SRR1271857_TR20330_c0_g3_i1_len=595	NA	NA	NA	NA	NA	.	0,133	1,318	0,203	-0,6	0	7,586	1	0	1	0	.	.	.	.	.	.
SRR1271857_TR25421_c0_g2_i7_len=579	CCC11_ARATH	Q:468-127,H:1-114	76.32%ID	E:2e-57	Cyclin-C1-1	PF00134.20^Cyclin_N^Cyclin, N-terminal domain^44-112^E:2.8e-13	0,177	0,723	0,014	1,723	0	6,685	0,005	6,209	0,267	0,679	CCC11_ARATH	Cyclin-C1-1 (CycC1;1)	CYCC1-1 At5g48640 K15N18.11	Arabidopsis thaliana (Mouse-ear cress)		Cell cycle; Cell division; Complete proteome; Cyclin; Reference proteome
SRR1271857_TR26900_c0_g1_i1_len=1743	C7D55_HYOMU	Q:1513-116,H:46-500	48.18%ID	E:1e-152	Premnaspirodiene oxygenase	PF00067.19^p450^Cytochrome P450^31-477^E:5.7e-104	0,045	0,654	0,009	0,84	0	1,338	0,008	0,986	0,002	0,924	C7D55_HYOMU	Premnaspirodiene oxygenase (HPO) (EC 1.14.13.121) (Cytochrome P450 71D55)	CYP71D55	Hyoscyamus muticus (Egyptian henbane)		Heme; Iron; Membrane; Metal-binding; Monooxygenase; NADP; Oxidoreductase; Transmembrane; Transmembrane helix
SRR1271857_TR30444_c1_g1_i27_len=1848	VESTR_MEDSA	Q:1479-517,H:5-324	51.71%ID	E:8e-118	Vestitone reductase	PF01370.18^Epimerase^NAD dependent epimerase/dehydratase family^10-251^E:7e-19`PF05368.10^NmrA^NmrA-like family^10-134^E:5.1e-05`PF01073.16^3Beta_HSD^3-beta hydroxysteroid dehydrogenase/isomerase family^12-200^E:2.3e-15`PF07993.9^NAD_binding_4^Male sterility protein^14-213^E:3.3e-10`PF13460.3^NAD_binding_10^NAD(P)H-binding^14-135^E:9.3e-08	1	-0,482	1	0,32	0	3,934	0,076	2,59	0,692	0,575	VESTR_MEDSA	Vestitone reductase (EC 1.1.1.348)		Medicago sativa (Alfalfa)		3D-structure; Direct protein sequencing; Flavonoid biosynthesis; NADP; Oxidoreductase; Plant defense
SRR1271857_TR38373_c0_g1_i1_len=2130	PCS1L_ARATH	Q:739-1791,H:82-442	37.4%ID	E:4e-68	Aspartic proteinase PCS1	PF14543.3^TAXi_N^Xylanase inhibitor N-terminal^184-343^E:1.5e-35`PF00026.20^Asp^Eukaryotic aspartyl protease^186-236^E:5.2e-05`PF14541.3^TAXi_C^Xylanase inhibitor C-terminal^381-538^E:6.2e-37	0,188	0,736	0,002	1,529	0	1,367	0,003	0,881	0,007	-1,017	PCS1L_ARATH	Aspartic proteinase PCS1 (EC 3.4.23.-) (Aspartic protease 38) (AtASP38) (Protein EMBRYO DEFECTIVE 24) (Protein PROMOTION OF CELL SURVIVAL 1)	PCS1 EMB24 At5g02190 T7H20.240	Arabidopsis thaliana (Mouse-ear cress)		Aspartyl protease; Complete proteome; Endoplasmic reticulum; Glycoprotein; Hydrolase; Protease; Reference proteome; Signal; Zymogen
SRR1271857_TR43038_c0_g2_i2_len=1432	HMGB1_ARATH	Q:126-500,H:1-123	73.6%ID	E:5e-24	High mobility group B protein 1	PF09011.7^HMG_box_2^HMG-box domain^93-165^E:8.1e-15`PF00505.16^HMG_box^HMG (high mobility group) box^96-165^E:1.9e-22	0,681	0,163	0,211	0,471	0	1,854	0,009	1,36	0,211	0,446	HMGB1_ARATH	High mobility group B protein 1 (High mobility group protein A) (AtHMGalpha) (HMG alpha) (Nucleosome/chromatin assembly factor group D 01) (Nucleosome/chromatin assembly factor group D 1)	HMGB1 HMGA HMGALPHA NFD01 NFD1 ORF13 At3g51880 ATEM1.13	Arabidopsis thaliana (Mouse-ear cress)		Alternative splicing; Complete proteome; DNA-binding; Nucleus; Phosphoprotein; Reference proteome
SRR1271858_TR2879_c0_g3_i3_len=603	ACR12_ARATH	Q:550-200,H:185-301	79.49%ID	E:1e-62	ACT domain-containing protein ACR12 {ECO:0000305}	.	0,464	0,417	0,36	-0,431	0	1,858	0,021	1,224	1	1,545	ACR12_ARATH	ACT domain-containing protein ACR12 (Protein ACT DOMAIN REPEATS 12)	ACR12 At5g04740 MUK11.6 T1E3.100	Arabidopsis thaliana (Mouse-ear cress)		Alternative splicing; Chloroplast; Complete proteome; Plastid; Reference proteome; Repeat; Transit peptide
SRR1271858_TR3134_c0_g1_i1_len=576	PTR53_ARATH	Q:2-574,H:33-223	64.4%ID	E:2e-84	Protein NRT1/ PTR FAMILY 2.11	PF00854.18^PTR2^POT family^84-188^E:2.4e-13	1	0,862	0,254	2,476	0	4,378	0,043	2,929	1	1,528	PTR53_ARATH	Protein NRT1/ PTR FAMILY 2.11 (AtNPF2.11) (Nitrate transporter 1.10) (Protein GLUCOSINOLATE TRANSPORTER-2)	NPF2.11 GTR2 NRT1.10 At5g62680 MRG21.10	Arabidopsis thaliana (Mouse-ear cress)		Cell membrane; Complete proteome; Membrane; Reference proteome; Transmembrane; Transmembrane helix; Transport
SRR1271858_TR9422_c0_g1_i2_len=852	IDD6_ARATH	Q:368-141,H:121-196	73.68%ID	E:1e-49	Protein indeterminate-domain 6, chloroplastic {ECO:0000303|PubMed:16784536}	.	1	-0,275	0,502	4,145	0	5,24	0,034	3,799	1	1,298	IDD6_ARATH	Protein indeterminate-domain 6, chloroplastic	IDD6 At1g14580 T5E21.8	Arabidopsis thaliana (Mouse-ear cress)		Chloroplast; Complete proteome; DNA-binding; Metal-binding; Phosphoprotein; Plastid; Reference proteome; Repeat; Transcription; Transcription regulation; Transit peptide; Zinc; Zinc-finger
SRR1271858_TR12676_c1_g1_i5_len=702	NA	NA	NA	NA	NA	.	1	0	1	1,968	0,001	6,567	1	1,624	1	-2,604	.	.	.	.	.	.
SRR1271858_TR16798_c0_g2_i1_len=706	TTM3_ARATH	Q:626-300,H:99-210	63.39%ID	E:3e-35	Triphosphate tunel metalloenzyme 3	PF01928.18^CYTH^CYTH domain^21-116^E:1.5e-12	0,471	-0,544	1	-0,049	0	6,727	1	0	0,581	-0,481	TTM3_ARATH	Triphosphate tunel metalloenzyme 3 (Adenosinetriphosphatase) (ATPase) (EC 3.6.1.3) (Triphosphatase) (PPPase) (EC 3.6.1.25)	TTM3 At2g11890 F23M2.5	Arabidopsis thaliana (Mouse-ear cress)		3D-structure; ATP-binding; Alternative splicing; Complete proteome; Hydrolase; Nucleotide-binding; Nucleus; Reference proteome
SRR1271858_TR21130_c1_g1_i4_len=1463	PIP22_ORYSJ	Q:1365-613,H:1-252	79.05%ID	E:4e-125	Probable aquaporin PIP2-2	PF00230.17^MIP^Major intrinsic protein^34-251^E:7e-72	1	3,242	0,001	6,415	0	4,828	0,001	4,015	1	-0,447	PIP22_ORYSJ	Probable aquaporin PIP2-2 (OsPIP2;2) (Plasma membrane intrinsic protein 2-2)	PIP2-2 Os02g0629200 LOC_Os02g41860 B1469H02.22-1	Oryza sativa subsp. japonica (Rice)		Cell membrane; Complete proteome; Membrane; Reference proteome; Repeat; Transmembrane; Transmembrane helix; Transport
SRR1271858_TR27329_c3_g4_i3_len=1188	NA	NA	NA	NA	NA	PF05922.13^Inhibitor_I9^Peptidase inhibitor I9^41-117^E:3.7e-16	0,946	0,05	0,331	0,35	0	1,479	0,004	1,063	0,583	-0,213	.	.	.	.	.	.
SRR1271858_TR33638_c0_g1_i1_len=560	NA	NA	NA	NA	NA	.	1	0,437	0,043	3,182	0	3,133	0,118	1,559	1	0,102	.	.	.	.	.	.

SRR1271858_TR34960_c0_g2_i1_len=1074	XTH28_ARATH	Q:139-867,H:66-308	69.55%ID	E:1e-129	Probable xyloglucan endotransglucosylase/hydrolase protein 28	PF00722.18^Glyco_hydro_16^Glycosyl hydrolases family 16^2-120^E:1.1e-39`PF06955.9^XET_C^Xyloglucan endo-transglycosylase (XET) C-terminus^160-195^E:1.4e-13	1	0,248	1	0,031	0	1,612	0,013	1,176	0,024	-0,974	XTH28_ARATH	Probable xyloglucan endotransglucosylase/hydrolase protein 28 (At-XTH28) (XTH-28) (EC 2.4.1.207)	XTH28 EXGT-A2 XTR2 At1g14720 F10B6.12 F10B6.29	Arabidopsis thaliana (Mouse-ear cress)		Apoplast; Cell wall; Cell wall biogenesis/degradation; Complete proteome; Disulfide bond; Glycoprotein; Glycosidase; Hydrolase; Reference proteome; Secreted; Signal; Transferase
SRR1271858_TR35881_c1_g1_i1_len=707	NA	NA	NA	NA	NA	.	1	2,839	1	1,414	0	9,008	0,001	6,475	0,502	-3,533	.	.	.	.	.	.
SRR1271858_TR38866_c0_g1_i1_len=1447	GILT_HUMAN	Q:927-379,H:62-243	37.23%ID	E:4e-29	Gamma-interferon-inducible lysosomal thiol reductase	PF03227.13^GILT^Gamma interferon inducible lysosomal thiol reductase (GILT)^41-143^E:4e-29	0,507	0,237	0,507	-0,248	0	1,232	0,028	0,789	1	0,219	GILT_HUMAN	Gamma-interferon-inducible lysosomal thiol reductase (EC 1.8.-.-) (Gamma-interferon-inducible protein IP-30) (Legumaturain)	IFI30 GILT IP30	Homo sapiens (Human)		Complete proteome; Direct protein sequencing; Disulfide bond; Glycoprotein; Immunity; Lysosome; Oxidoreductase; Redox-active center; Reference proteome; Secreted; Signal
SRR1271858_TR40126_c0_g3_i4_len=1150	ACCO5_ARATH	Q:240-746,H:109-285	43.5%ID	E:8e-42	1-aminocyclopropane-1-carboxylate oxidase 5	PF03171.17^2OG-FeII_Oxy^2OG-Fe(II) oxygenase superfamily^6-104^E:8.5e-30	0,034	3,582	0,017	3,974	0	3,697	0,003	3,239	0,034	-3,347	ACCO5_ARATH	1-aminocyclopropane-1-carboxylate oxidase 5 (ACC oxidase 5) (AtACO5) (EC 1.14.17.4)	At1g77330 F2P24.4	Arabidopsis thaliana (Mouse-ear cress)	PATHWAY: Alkene biosynthesis; ethylene biosynthesis via S-adenosyl-L-methionine; ethylene from S-adenosyl-L-methionine: step 2/2.	Coiled coil; Complete proteome; Ethylene biosynthesis; Iron; Metal-binding; Oxidoreductase; Plant defense; Reference proteome; Vitamin C
SRR1271858_TR47901_c0_g1_i1_len=790	NA	NA	NA	NA	NA	PF04398.9^DUF538^Protein of unknown function, DUF538^29-140^E:2.9e-29	1	0,521	0,034	5,649	0	7,351	0,254	4,732	1	-0,787	.	.	.	.	.	.
SRR1271858_TR61015_c1_g3_i3_len=5104	AB36G_ARATH	Q:634-5019,H:34-1469	73.53%ID	E:0	ABC transporter G family member 36	PF14510.3^ABC_trans_N^ABC-transporter extracellular N-terminal^95-155^E:9.2e-12`PF00005.24^ABC_tran^ABC transporter^180-362^E:9.5e-14`PF01061.21^ABC2_membrane^ABC-2 type transporter^516-727^E:2.6e-45`PF08370.8^PDR_assoc^Plant PDR ABC transporter associated^733-796^E:1.1e-25`PF00005.24^ABC_tran^ABC transporter^913-1065^E:1.6e-20`PF01061.21^ABC2_membrane^ABC-2 type transporter^1211-1424^E:1.8e-57	0,502	2,716	0,502	3,91	0	3,753	0,027	2,694	1	-0,75	AB36G_ARATH	ABC transporter G family member 36 (ABC transporter ABCG.36) (AtABCG36) (Pleiotropic drug resistance protein 8) (Protein PENETRATION 3)	ABCG36 PDR8 PEN3 At1g59870 F23H11.19	Arabidopsis thaliana (Mouse-ear cress)		ATP-binding; Acetylation; Cell membrane; Complete proteome; Membrane; Nucleotide-binding; Phosphoprotein; Plant defense; Reference proteome; Repeat; Transmembrane; Transmembrane helix; Transport
SRR1271859_TR2892_c1_g1_i4_len=1308	ULT1_ARATH	Q:315-989,H:14-237	75.11%ID	E:2e-121	Protein ULTRAPETALA 1	PF01342.18^SAND^SAND domain^65-102^E:3.3e-05	0,692	-1,131	0,103	1,56	0	3,465	0,132	2,155	1	0	ULT1_ARATH	Protein ULTRAPETALA 1	ULT1 At4g28190 F26K10.70	Arabidopsis thaliana (Mouse-ear cress)		Alternative splicing; Complete proteome; Cytoplasm; DNA-binding; Developmental protein; Nucleus; Reference proteome; Transcription; Transcription regulation
SRR1271859_TR4140_c0_g2_i1_len=1050	NA	NA	NA	NA	NA	.	0,629	1,16	0,014	2,842	0	3,379	0,073	2,005	1	0,198	.	.	.	.	.	.
SRR1271859_TR4946_c0_g3_i5_len=638	CB2D_SOLLC	Q:608-276,H:6-116	93.69%ID	E:4e-70	Chlorophyll a-b binding protein 1D	.	0,458	0,155	0	-2,667	0	1,036	0,004	0,613	0	-3,844	CB2D_SOLLC	Chlorophyll a-b binding protein 1D (LHCII type I CAB-1D) (LHCP) (Fragment)	CAB1D	Solanum lycopersicum (Tomato) (Lycopersicon esculentum)		Chlorophyll; Chloroplast; Chromophore; Complete proteome; Magnesium; Membrane; Metal-binding; Phosphoprotein; Photosynthesis; Photosystem I; Photosystem II; Plastid; Reference proteome; Thylakoid; Transmembrane; Transmembrane helix
SRR1271859_TR6881_c0_g2_i2_len=3130	FPP3_ARATH	Q:446-2428,H:1-615	43.79%ID	E:4e-92	Filament-like plant protein 3	PF05911.8^FPP^Filament-like plant protein, long coiled-coil^83-189^E:1.2e-36`PF05911.8^FPP^Filament-like plant protein, long coiled-coil^196-267^E:8e-20`PF05911.8^FPP^Filament-like plant protein, long coiled-coil^319-371^E:3.3e-13`PF05911.8^FPP^Filament-like plant protein, long coiled-coil^480-655^E:1.9e-23	1	-0,015	0,096	0,674	0	2,694	0,132	1,495	0,708	0,176	FPP3_ARATH	Filament-like plant protein 3 (AtFPP3)	FPP3 At3g05270 T12H1.24	Arabidopsis thaliana (Mouse-ear cress)		Coiled coil; Complete proteome; Reference proteome
SRR1271859_TR12778_c0_g1_i1_len=2171	PTR3_ARATH	Q:2043-328,H:9-579	66.43%ID	E:0	Protein NRT1/ PTR FAMILY 5.2	PF00854.18^PTR2^POT family^101-530^E:1.8e-99	0,263	0,359	0,356	0,34	0	1,35	0,012	0,917	0,037	0,914	PTR3_ARATH	Protein NRT1/ PTR FAMILY 5.2 (AtNPF5.2) (Peptide transporter PTR3-A) (AtPTR3)	NPF5.2 PTR3-A At5g46050 MCL19.10	Arabidopsis thaliana (Mouse-ear cress)		Complete proteome; Membrane; Reference proteome; Transmembrane; Transmembrane helix
SRR1271859_TR13981_c0_g2_i1_len=608	HSP12_MEDSA	Q:1-441,H:12-158	85.71%ID	E:1e-67	18.2 kDa class I heat shock protein	PF04969.13^CS^CS domain^41-132^E:0.0089`PF00011.18^HSP20^Hsp20/alpha crystallin family^43-146^E:7.5e-33	0,086	-0,855	0,017	-1,025	0	2,181	0,034	1,103	0,901	-0,049	HSP12_MEDSA	18.2 kDa class I heat shock protein	HSP18.2	Medicago sativa (Alfalfa)		Cytoplasm; Stress response
SRR1271859_TR15741_c0_g3_i1_len=861	NA	NA	NA	NA	NA	.	1	0,019	1	3,345	0,001	5,61	0,066	4,263	1	-0,874	.	.	.	.	.	.
SRR1271859_TR16572_c0_g1_i3_len=2193	GSTFA_ARATH	Q:316-936,H:1-207	62.8%ID	E:1e-88	Glutathione S-transferase F10	PF02798.17^GST_N^Glutathione S-transferase, N-terminal domain^2-75^E:3.7e-16`PF13417.3^GST_N_3^Glutathione S-transferase, N-terminal domain^13-78^E:1.5e-14`PF13409.3^GST_N_2^Glutathione S-transferase, N-terminal domain^14-75^E:8.7e-12`PF00043.22^GST_C^Glutathione S-transferase, C-terminal domain^127-202^E:1e-16`PF14497.3^GST_C_3^Glutathione S-transferase, C-terminal domain^134-203^E:6.5e-07	0,189	-0,521	0,522	-0,298	0,001	2,021	0,006	1,703	0,568	0,27	GSTFA_ARATH	Glutathione S-transferase F10 (AtGSTF10) (EC 2.5.1.18) (AtGSTF4) (GST class-phi member 10) (Protein EARLY RESPONSE TO DEHYDRATION 13)	GSTF10 ERD13 GSTF4 At2g30870 F7F1.8	Arabidopsis thaliana (Mouse-ear cress)		Complete proteome; Cytoplasm; Detoxification; Direct protein sequencing; Reference proteome; Stress response; Transferase
SRR1271859_TR21191_c0_g1_i1_len=1093	NA	NA	NA	NA	NA	.	1	1,694	0,502	4,346	0	8,377	0,017	5,885	1	-3,571	.	.	.	.	.	.
SRR1271859_TR21383_c0_g2_i1_len=683	CSPL2_RICCO	Q:97-549,H:12-163	59.87%ID	E:3e-41	CASP-like protein 1C2	PF04535.9^DUF588^Domain of unknown function (DUF588)^7-141^E:1.8e-37	0,502	3,225	0,009	5,325	0,001	4,425	0,629	2,294	0,502	-2,716	CSPL2_RICCO	CASP-like protein 1C2 (RcCASPL1C2)	RCOM_0936380	Ricinus communis (Castor bean)		Cell membrane; Complete proteome; Glycoprotein; Membrane; Reference proteome; Transmembrane; Transmembrane helix
SRR1271859_TR22448_c0_g1_i1_len=717	H2B_GOSHI	Q:232-540,H:45-147	96.12%ID	E:1e-58	Histone H2B	PF00125.21^Histone^Core histone H2A/H2B/H3/H4^9-124^E:9.9e-23`PF00808.20^CBFD_NFYB_HMF^Histone-like transcription factor (CBF/NF-Y) and archaeal histone^66-122^E:4.4e-05	0	-0,953	0,205	-0,355	0	1,025	0,003	0,883	1	-2,277	H2B_GOSHI	Histone H2B	HIS2B	Gossypium hirsutum (Upland cotton) (Gossypium mexicanum)		Acetylation; Chromosome; DNA-binding; Isopeptide bond; Nucleosome core; Nucleus; Ubl conjugation
SRR1271859_TR25424_c0_g4_i1_len=1358	NA	NA	NA	NA	NA	PF09032.8^Siah-Interact_N^Siah interacting protein, N terminal^3-63^E:5.7e-08`PF04969.13^CS^CS domain^73-149^E:1.9e-15	0,226	-0,511	0,389	-0,41	0	1,27	0,023	0,809	1	0,015	.	.	.	.	.	.
SRR1271859_TR26970_c0_g2_i1_len=1951	NA	NA	NA	NA	NA	.	0,129	3,623	0,005	4,182	0	3,496	0,025	2,733	0,732	0,891	.	.	.	.	.	.
SRR1271859_TR43498_c0_g1_i4_len=2228	INT2_ARATH	Q:194-1951,H:1-579	67.63%ID	E:0	Probable inositol transporter 2	PF00083.21^Sugar_tr^Sugar (and other) transporter^30-379^E:1.9e-94`PF07690.13^MFS_1^Major Facilitator Superfamily^35-368^E:2e-33`PF06609.10^TRI12^Fungal trichothecene efflux pump (TRI12)^74-228^E:4.2e-07`PF00083.21^Sugar_tr^Sugar (and other) transporter^461-563^E:4.4e-28	0,073	1,898	0,132	2,964	0	7,051	0,034	5,66	1	0,127	INT2_ARATH	Probable inositol transporter 2	INT2 At1g30220 F12P21.2	Arabidopsis thaliana (Mouse-ear cress)		Cell membrane; Complete proteome; Membrane; Reference proteome; Symport; Transmembrane; Transmembrane helix; Transport
SRR1271859_TR45268_c0_g1_i1_len=685	NA	NA	NA	NA	NA	.	0,254	4,534	0,129	5,148	0	7,344	0,129	4,865	1	0	.	.	.	.	.	.
SRR1271859_TR47365_c0_g3_i1_len=2084	APA1_ARATH	Q:1765-332,H:27-506	67.36%ID	E:0	Aspartic proteinase A1	PF00026.20^Asp^Eukaryotic aspartyl protease^121-544^E:6.3e-134`PF14543.3^TAXi_N^Xylanase inhibitor N-terminal^122-279^E:2.8e-14`PF03489.14^SapB_2^Saposin-like type B, region 2^354-387^E:1.5e-12`PF05184.12^SapB_1^Saposin-like type B, region 1^418-454^E:1e-10	0,934	0,03	0,935	-0,026	0,001	1,1	0,014	0,829	0,08	0,547	APA1_ARATH	Aspartic proteinase A1 (EC 3.4.23.-)	APA1 PRA1 At1g11910 F12F1.24	Arabidopsis thaliana (Mouse-ear cress)		Aspartyl protease; Complete proteome; Disulfide bond; Glycoprotein; Hydrolase; Protease; Reference proteome; Repeat; Signal; Vacuole; Zymogen
SRR342017_TR987_c0_g9_i1_len=1009	AB9B_ARATH	Q:2-697,H:994-1225	81.9%ID	E:7e-124	ABC transporter B family member 9	PF00005.24^ABC_tran^ABC transporter^19-168^E:2.5e-35	0,781	0,504	0,316	0,633	0	6,797	0,034	5,534	0,175	-0,966	AB9B_ARATH	ABC transporter B family member 9 (ABC transporter ABCB.9) (AtABCB9) (Multidrug resistance protein 9) (P-glycoprotein 9)	ABCB9 MDR9 PGP9 At4g18050 F15J5.20	Arabidopsis thaliana (Mouse-ear cress)		ATP-binding; Complete proteome; Glycoprotein; Membrane; Nucleotide-binding; Reference proteome; Repeat; Transmembrane; Transmembrane helix; Transport
SRR342017_TR1512_c0_g3_i1_len=960	CH10C_ARATH	Q:6-674,H:31-253	77.58%ID	E:7e-119	20 kDa chaperonin, chloroplastic	PF00166.18^Cpn10^Chaperonin 10 Kd subunit^33-122^E:5.6e-28`PF00166.18^Cpn10^Chaperonin 10 Kd subunit^131-222^E:3.9e-28	0	-3,334	0	-2,451	0	4,284	0,004	3,434	0	-0,897	CH10C_ARATH	20 kDa chaperonin, chloroplastic (Chaperonin 10) (Ch-CPN10) (Cpn10) (Chaperonin 20) (Protein Cpn21)	CPN21 CHCPN10 CPN20 At5g20720 T1M15.120	Arabidopsis thaliana (Mouse-ear cress)		Chaperone; Chloroplast; Complete proteome; Phosphoprotein; Plastid; Reference proteome; Repeat; Transit peptide
SRR342017_TR2746_c0_g1_i2_len=3059	SUBL_ARATH	Q:2585-378,H:31-757	58.63%ID	E:0	.	PF05922.13^Inhibitor_I9^Peptidase inhibitor I9^29-107^E:1.4e-17`PF00082.19^Peptidase_S8^Subtilase family^137-588^E:1.3e-49`PF02225.19^PA^PA domain^403-463^E:2.4e-09	0	-1,437	0,357	0,649	0	1,743	0,739	0,338	1	0,022	SBT17_ARATH	Subtilisin-like protease SBT1.7 (EC 3.4.21.-) (Cucumisin-like serine protease) (Subtilase subfamily 1 member 7) (AtSBT1.7) (Subtilisin-like serine protease 1) (At-SLP1)	SBT1.7 ARA12 ASP48 SLP1 At5g67360 K8K14.8	Arabidopsis thaliana (Mouse-ear cress)		Calcium; Cell wall; Complete proteome; Direct protein sequencing; Glycoprotein; Hydrolase; Protease; Reference proteome; Secreted; Serine protease; Signal; Zymogen
SRR342017_TR3199_c0_g5_i1_len=1156	NA	NA	NA	NA	NA	.	0,365	0,568	0	-1,631	0,001	6,552	1	0	0	-1,919	.	.	.	.	.	.
SRR342017_TR4266_c1_g9_i1_len=1532	PDI_DATGL	Q:22-1086,H:136-492	68.07%ID	E:1e-159	Protein disulfide-isomerase	PF13848.3^Thioredoxin_6^Thioredoxin-like domain^44-226^E:6.6e-20`PF00085.17^Thioredoxin^Thioredoxin^249-352^E:4e-26	0,01	-2,201	0,005	-0,937	0	2,809	0,282	1,209	0,004	-0,889	PDI_DATGL	Protein disulfide-isomerase (PDI) (EC 5.3.4.1)	PDI	Datisca glomerata (Durango root)		Disulfide bond; Endoplasmic reticulum; Glycoprotein; Isomerase; Redox-active center; Repeat; Signal
SRR342017_TR4309_c0_g2_i1_len=809	NA	NA	NA	NA	NA	PF07172.8^GRP^Glycine rich protein family^1-100^E:4.6e-10	0,102	-0,422	0,15	-0,387	0	2,973	0,042	1,842	0,009	-0,698	.	.	.	.	.	.
SRR342017_TR7232_c0_g1_i1_len=525	CID9_ARATH	Q:525-355,H:267-323	75.44%ID	E:2e-21	Polyadenylate-binding protein-interacting protein 9	.	0,017	5,772	0,105	-1,013	0	7,451	1	0	1	0	CID9_ARATH	Polyadenylate-binding protein-interacting protein 9 (PABP-interacting protein 9) (Poly(A)-binding protein-interacting protein 9) (PAM2-containing protein CID9) (Protein CTC-INTERACTING DOMAIN 9)	CID9 At3g14450 MOA2.5	Arabidopsis thaliana (Mouse-ear cress)		Complete proteome; Nucleus; RNA-binding; Reference proteome; Repeat
SRR342017_TR8315_c2_g6_i1_len=967	DCAM_CATRO	Q:1-429,H:208-350	78.32%ID	E:5e-75	S-adenosylmethionine decarboxylase proenzyme	PF01536.13^SAM_decarbox^Adenosylmethionine decarboxylase^1-125^E:3.7e-33`PF08315.9^cwf18^cwf18 pre-mRNA splicing factor^143-237^E:2.4e-26	0,235	-0,891	0,031	-1,638	0,001	6,58	0,017	5,851	0,443	-0,441	DCAM_CATRO	S-adenosylmethionine decarboxylase proenzyme (AdoMetDC) (SAMDC) (EC 4.1.1.50) [Cleaved into: S-adenosylmethionine decarboxylase alpha chain; S-adenosylmethionine decarboxylase beta chain]	SAMDC	Catharanthus roseus (Madagascar periwinkle) (Vinca rosea)	PATHWAY: Amine and polyamine biosynthesis; S-adenosylmethioninamine biosynthesis; S-adenosylmethioninamine from S-adenosyl-L-methionine: step 1/1.	Autocatalytic cleavage; Decarboxylase; Lyase; Polyamine biosynthesis; Pyruvate; S-adenosyl-L-methionine; Schiff base; Spermidine biosynthesis; Zymogen
SRR342017_TR10528_c0_g1_i3_len=2256	KTNA1_ARATH	Q:321-1856,H:8-523	85.11%ID	E:0	Katanin p60 ATPase-containing subunit A1 {ECO:0000255|HAMAP-Rule:MF_03023}	PF05496.9^RuvB_N^Holliday junction DNA helicase ruvB N-terminus^268-337^E:8.2e-05`PF00004.26^AAA^ATPase family associated with various cellular activities (AAA)^269-406^E:1e-37`PF09336.7^Vps4_C^Vps4 C terminal oligomerisation domain^470-515^E:4e-11	0,629	-1,177	0,068	-1,055	0,001	3,35	0,001	3,262	0,629	1,893	KTNA1_ARATH	Katanin p60 ATPase-containing subunit A1 (Katanin p60 subunit A1) (EC 3.6.4.3) (CAD ATPase) (Katanin-1) (Atp60) (Protein BOTERO 1) (Protein ECTOPIC ROOT HAIR 3) (Protein FAT ROOT) (Protein FRAGILE FIBER 2) (AtAAA1) (p60 katanin)	AAA1 BOT1 ERH3 FRA2 FTR KSS KTN1 LUE1 At1g80350 F5I6.10	Arabidopsis thaliana (Mouse-ear cress)		ATP-binding; Complete proteome; Cytoplasm; Cytoskeleton; Hydrolase; Microtubule; Nucleotide-binding; Reference proteome
SRR342017_TR11113_c0_g7_i6_len=1759	TC120_ARATH	Q:1597-632,H:763-1085	71.52%ID	E:8e-152	Translocase of chloroplast 120, chloroplastic	PF11886.5^TOC159_MAD^Translocase of chloroplast 159/132, membrane anchor domain^43-305^E:8.5e-116	0,781	0,41	0,075	-0,751	0	7,187	0,009	5,934	0,719	-0,31	TC120_ARATH	Translocase of chloroplast 120, chloroplastic (AtToc120) (EC 3.6.5.-) (120 kDa chloroplast outer envelope protein)	TOC120 At3g16620 MGL6.15	Arabidopsis thaliana (Mouse-ear cress)		Acetylation; Chloroplast; Coiled coil; Complete proteome; Cytoplasm; GTP-binding; Hydrolase; Magnesium; Membrane; Metal-binding; Nucleotide-binding; Phosphoprotein; Plastid; Plastid outer membrane; Protein transport; Receptor; Reference proteome; Transmembrane; Transmembrane helix; Transport
SRR342017_TR12681_c0_g1_i1_len=538	NA	NA	NA	NA	NA	.	0,548	-0,249	0,384	-0,307	0	1,752	0,006	1,27	0,041	-0,563	.	.	.	.	.	.
SRR342017_TR14705_c0_g2_i3_len=1792	Y5188_ARATH	Q:969-151,H:577-851	55.23%ID	E:5e-98	Probable LRR receptor-like serine/threonine-protein kinase At1g51880	PF00069.22^Pkinase^Protein kinase domain^1-200^E:6.6e-33`PF07714.14^Pkinase_Tyr^Protein tyrosine kinase^2-144^E:7.2e-28	1	-0,08	0,025	1,206	0,001	1,996	0,009	1,617	1	-0,284	Y5188_ARATH	Probable LRR receptor-like serine/threonine-protein kinase At1g51880 (EC 2.7.11.1)	At1g51880 T14L22.9	Arabidopsis thaliana (Mouse-ear cress)		ATP-binding; Complete proteome; Glycoprotein; Kinase; Leucine-rich repeat; Membrane; Nucleotide-binding; Phosphoprotein; Receptor; Reference proteome; Repeat; Serine/threonine-protein kinase; Signal; Transferase; Transmembrane; Transmembrane helix
SRR342017_TR16388_c0_g1_i1_len=863	PSAG_ARATH	Q:207-629,H:27-160	72.34%ID	E:8e-67	Photosystem I reaction center subunit V, chloroplastic	PF01241.15^PSI_PSAK^Photosystem I psaG / psaK^53-144^E:2.5e-26	0,388	-0,239	0,4	0,305	0	1,468	0,019	0,942	0,433	0,21	PSAG_ARATH	Photosystem I reaction center subunit V, chloroplastic (PSI-G)	PSAG At1g55670 F20N2.21 F20N2.33 F20N2_3	Arabidopsis thaliana (Mouse-ear cress)		Chloroplast; Complete proteome; Membrane; Photosynthesis; Photosystem I; Plastid; Reference proteome; Thylakoid; Transit peptide; Transmembrane; Transmembrane helix
SRR342017_TR17600_c3_g1_i1_len=728	P5CS2_ARATH	Q:673-314,H:596-715	87.5%ID	E:4e-65	Delta-1-pyrroline-5-carboxylate synthase B	.	0,005	6,16	0,77	-0,162	0	8,246	0,502	4,373	1	0	P5CS2_ARATH	Delta-1-pyrroline-5-carboxylate synthase B (P5CS B) [Includes: Glutamate 5-kinase (GK) (EC 2.7.2.11) (Gamma-glutamyl kinase); Gamma-glutamyl phosphate reductase (GPR) (EC 1.2.1.41) (Glutamate-5-semialdehyde dehydrogenase) (Glutamyl-gamma-semialdehyde dehydrogenase)]	P5CSB P5CS2 At3g55610 F1I16_20	Arabidopsis thaliana (Mouse-ear cress)	PATHWAY: Amino-acid biosynthesis; L-proline biosynthesis; L-glutamate 5-semialdehyde from L-glutamate: step 1/2.; PATHWAY: Amino-acid biosynthesis; L-proline biosynthesis; L-glutamate 5-semialdehyde from L-glutamate: step 2/2.	ATP-binding; Alternative splicing; Amino-acid biosynthesis; Complete proteome; Kinase; Multifunctional enzyme; NADP; Nucleotide-binding; Oxidoreductase; Proline biosynthesis; Reference proteome; Transferase
SRR342017_TR19058_c0_g5_i2_len=973	NA	NA	NA	NA	NA	PF14279.3^HNH_5^HNH endonuclease^147-198^E:2.3e-17`PF01844.20^HNH^HNH endonuclease^147-189^E:7e-13`PF13395.3^HNH_4^HNH endonuclease^153-190^E:3.1e-07	0,059	0,618	0,306	0,339	0,001	1,932	0,001	1,826	0,355	-0,399	.	.	.	.	.	.
SRR342017_TR23868_c0_g1_i1_len=903	NA	NA	NA	NA	NA	.	0,051	1,09	0,848	0,201	0	2,531	0,147	1,107	1	0,289	.	.	.	.	.	.
SRR342017_TR27898_c3_g4_i1_len=505	ACO2M_ARATH	Q:46-258,H:920-990	85.92%ID	E:3e-36	Aconitate hydratase 2, mitochondrial	.	0,271	-0,333	1	0,002	0	1,366	0,08	0,703	0,005	-0,857	ACO2M_ARATH	Aconitate hydratase 2, mitochondrial (Aconitase 2) (EC 4.2.1.3) (Citrate hydro-lyase 2)	ACO2 At2g05710 T3P4.5	Arabidopsis thaliana (Mouse-ear cress)	PATHWAY: Carbohydrate metabolism; tricarboxylic acid cycle; isocitrate from oxaloacetate: step 2/2.	4Fe-4S; Complete proteome; Glyoxylate bypass; Iron; Iron-sulfur; Lyase; Metal-binding; Mitochondrion; Reference proteome; Transit peptide; Tricarboxylic acid cycle
SRR342017_TR30393_c0_g3_i1_len=1333	FLS_PETCR	Q:1230-559,H:44-271	36.8%ID	E:9e-40	Flavonol synthase/flavanone 3-hydroxylase	PF14226.3^DIOX_N^non-haem dioxygenase in morphine synthesis N-terminal^35-139^E:9.7e-23`PF03171.17^2OG-FeII_Oxy^2OG-Fe(II) oxygenase superfamily^188-255^E:1.3e-13	0,01	1,961	0,009	4,594	0	3,049	0,001	2,57	0,02	1,353	FLS_PETCR	Flavonol synthase/flavanone 3-hydroxylase (EC 1.14.11.23) (EC 1.14.11.9)	FLS	Petroselinum crispum (Parsley) (Petroselinum hortense)	PATHWAY: Secondary metabolite biosynthesis; flavonoid biosynthesis.	Dioxygenase; Flavonoid biosynthesis; Iron; Metal-binding; Oxidoreductase; Vitamin C
SRR342017_TR31911_c1_g3_i4_len=1511	FKB62_ARATH	Q:337-1071,H:307-550	82.45%ID	E:2e-123	Peptidyl-prolyl cis-trans isomerase FKBP62	PF00254.25^FKBP_C^FKBP-type peptidyl-prolyl cis-trans isomerase^30-96^E:2.2e-15`PF14559.3^TPR_19^Tetratricopeptide repeat^176-236^E:3.5e-06`PF00515.25^TPR_1^Tetratricopeptide repeat^199-232^E:1.6e-08`PF07719.14^TPR_2^Tetratricopeptide repeat^200-232^E:1.7e-06`PF13181.3^TPR_8^Tetratricopeptide repeat^200-232^E:1.1e-06	0,84	-0,357	0,793	-0,211	0	2,35	0,04	1,509	0,728	-0,122	FKB62_ARATH	Peptidyl-prolyl cis-trans isomerase FKBP62 (PPIase FKBP62) (EC 5.2.1.8) (70 kDa peptidyl-prolyl isomerase) (FK506-binding protein 62) (AtFKBP62) (Immunophilin FKBP62) (Peptidylprolyl isomerase ROF1) (Protein ROTAMASE FKBP 1) (Rotamase)	FKBP62 ROF1 At3g25230 MJL12.19	Arabidopsis thaliana (Mouse-ear cress)		Acetylation; Alternative splicing; Calmodulin-binding; Chaperone; Complete proteome; Cytoplasm; Isomerase; Nucleus; Reference proteome; Repeat; Rotamase; TPR repeat
SRR342019_TR508_c0_g1_i2_len=1062	NA	NA	NA	NA	NA	PF00582.23^Usp^Universal stress protein family^8-153^E:4.6e-33	0,811	0,293	0,006	2,228	0	4,237	0,003	3,899	0,629	-0,949	.	.	.	.	.	.
SRR342019_TR14652_c1_g1_i3_len=1999	WTR41_ARATH	Q:1747-578,H:13-356	41.77%ID	E:1e-80	WAT1-related protein At5g40230	PF00892.17^EamA^EamA-like transporter family^17-156^E:1.1e-13	0,003	1,385	0,012	1,03	0	2,694	0,014	1,72	1	0,971	WTR41_ARATH	WAT1-related protein At5g40230	At5g40230 MSN9.130	Arabidopsis thaliana (Mouse-ear cress)		Complete proteome; Membrane; Reference proteome; Repeat; Transmembrane; Transmembrane helix
SRR342019_TR17901_c0_g1_i1_len=903	NA	NA	NA	NA	NA	.	0,836	-0,127	0,586	0,238	0	1,54	0,027	1,054	1	0	.	.	.	.	.	.
SRR342019_TR18099_c0_g1_i1_len=842	NA	NA	NA	NA	NA	.	0,063	0,694	0,001	1,525	0,001	1,416	0,38	-0,468	0,005	1,034	.	.	.	.	.	.
SRR342019_TR18305_c3_g2_i2_len=2465	BGH3B_BACO1	Q:305-2065,H:35-658	31.32%ID	E:5e-83	Beta-glucosidase BoGH3B	PF00933.18^Glyco_hydro_3^Glycosyl hydrolase family 3 N terminal domain^51-379^E:3.7e-71`PF01915.19^Glyco_hydro_3_C^Glycosyl hydrolase family 3 C-terminal domain^416-625^E:1.7e-33	0,652	-0,13	0,663	0,149	0	2,294	0,043	1,242	0,098	-0,487	BGH3B_BACO1	Beta-glucosidase BoGH3B (EC 3.2.1.21) (Glycosyl hydrolase family protein 3B) (BoGH3B)	BACOVA_02659	Bacteroides ovatus (strain ATCC 8483 / DSM 1896 / JCM 5824 / NCTC 11153)	PATHWAY: Glucan metabolism; xyloglucan degradation. {ECO:0000269|PubMed:24463512}.	Carbohydrate metabolism; Complete proteome; Glycosidase; Hydrolase; Periplasm; Polysaccharide degradation; Signal
SRR342019_TR18596_c0_g1_i2_len=787	14KD_DAUCA	Q:313-561,H:54-136	71.08%ID	E:5e-32	14 kDa proline-rich protein DC2.15	.	0,093	-0,393	0,015	-0,546	0,001	1,232	0,003	1,112	1	0,034	14KD_DAUCA	14 kDa proline-rich protein DC2.15		Daucus carota (Wild carrot)		Membrane; Signal; Transmembrane; Transmembrane helix
SRR342019_TR18824_c6_g1_i4_len=727	NLTP2_TOBAC	Q:131-481,H:1-114	55.93%ID	E:4e-26	Non-specific lipid-transfer protein 2	PF14368.3^LTP_2^Probable lipid transfer^15-105^E:1.6e-06`PF00234.19^Tryp_alpha_amyl^Protease inhibitor/seed storage/LTP family^30-113^E:9.6e-08	0,054	0,485	0,606	0,139	0	0,916	0,078	0,415	0,157	0,303	NLTP2_TOBAC	Non-specific lipid-transfer protein 2 (LTP 2)		Nicotiana tabacum (Common tobacco)		Disulfide bond; Lipid-binding; Signal; Transport
SRR342019_TR25523_c1_g1_i3_len=2015	TCP13_ARATH	Q:374-640,H:61-147	82.02%ID	E:2e-39	Transcription factor TCP13	PF03634.10^TCP^TCP family transcription factor^60-180^E:1.8e-30	0,107	0,574	0,008	0,894	0	1,408	0,026	0,889	0,496	-0,255	TCP13_ARATH	Transcription factor TCP13 (Plastid transcription factor 1) (TFPD)	TCP13 PTF1 At3g02150 F14P3.20 F1C9.6	Arabidopsis thaliana (Mouse-ear cress)		Alternative splicing; Chloroplast; Complete proteome; DNA-binding; Developmental protein; Nucleus; Plastid; Reference proteome; Transcription; Transcription regulation
SRR342019_TR27500_c1_g2_i3_len=1179	EF1A_TOBAC	Q:1113-517,H:238-436	97.99%ID	E:3e-135	Elongation factor 1-alpha	PF03144.22^GTP_EFTU_D2^Elongation factor Tu domain 2^7-50^E:2.2e-07`PF03143.14^GTP_EFTU_D3^Elongation factor Tu C-terminal domain^59-166^E:5.5e-39	0,003	-0,893	0	-1,413	0	0,967	0,576	0,17	0	-2,412	EF1A_TOBAC	Elongation factor 1-alpha (EF-1-alpha) (Vitronectin-like adhesion protein 1) (PVN1)		Nicotiana tabacum (Common tobacco)		Cytoplasm; Elongation factor; GTP-binding; Nucleotide-binding; Phosphoprotein; Protein biosynthesis
SRR342019_TR29283_c0_g1_i1_len=324	NA	NA	NA	NA	NA	.	0,831	-0,097	0,939	-0,028	0	2,22	1	0,099	0,066	-0,663	.	.	.	.	.	.
SRR342019_TR30815_c0_g1_i1_len=1278	NA	NA	NA	NA	NA	.	0,073	1,968	1	0,999	0	3,613	0,025	2,747	0,692	1,034	.	.	.	.	.	.
SRR342019_TR39208_c0_g1_i3_len=1250	C72A1_CATRO	Q:91-768,H:253-470	53.98%ID	E:3e-81	Secologanin synthase	PF00067.19^p450^Cytochrome P450^6-206^E:1.2e-38	0,026	0,932	0,001	1,338	0	1,65	0,268	0,56	0,793	-0,191	C72A1_CATRO	Secologanin synthase (SLS) (EC 1.3.3.9) (CYPLXXII) (Cytochrome P450 72A1)	CYP72A1 CYP72 P450CR3	Catharanthus roseus (Madagascar periwinkle) (Vinca rosea)	PATHWAY: Alkaloid biosynthesis; secologanin biosynthesis.	Alkaloid metabolism; Heme; Iron; Membrane; Metal-binding; Monooxygenase; NADP; Oxidoreductase; Transmembrane; Transmembrane helix; Vacuole
SRR342022_TR253_c0_g4_i4_len=2723	PLT5_ARATH	Q:1152-2657,H:21-525	68.18%ID	E:0	Polyol transporter 5	PF00083.21^Sugar_tr^Sugar (and other) transporter^118-576^E:3.8e-109`PF07690.13^MFS_1^Major Facilitator Superfamily^124-519^E:3.5e-28	0,278	0,769	0,001	2,179	0	1,319	0,002	1,144	0,147	-0,971	PLT5_ARATH	Polyol transporter 5 (Protein POLYOL TRANSPORTER 5) (AtPLT5) (Sugar-proton symporter PLT5)	PLT5 At3g18830 MVE11.10 MVE11.22	Arabidopsis thaliana (Mouse-ear cress)		Cell membrane; Complete proteome; Membrane; Reference proteome; Sugar transport; Symport; Transmembrane; Transmembrane helix; Transport
SRR342022_TR735_c0_g4_i4_len=1202	TET3_ARATH	Q:228-947,H:46-285	69.17%ID	E:9e-109	Tetraspanin-3	PF00335.17^Tetraspannin^Tetraspanin family^1-202^E:4.9e-19	0,2	0,672	0,206	0,748	0	2,39	0,016	1,532	1	-0,023	TET3_ARATH	Tetraspanin-3	TET3 At3g45600 F9K21.18	Arabidopsis thaliana (Mouse-ear cress)		Complete proteome; Glycoprotein; Membrane; Reference proteome; Transmembrane; Transmembrane helix
SRR342022_TR4752_c0_g1_i2_len=688	SGT1B_ARATH	Q:642-205,H:213-358	73.29%ID	E:2e-61	Protein SGT1 homolog B {ECO:0000250|UniProtKB:Q08446}	PF04969.13^CS^CS domain^15-39^E:0.032`PF05002.12^SGS^SGS domain^79-159^E:8.1e-38	0,793	0,197	0,315	-0,358	0	1,707	0,489	0,415	0,254	-4,768	SGT1B_ARATH	Protein SGT1 homolog B (AtSGT1b) (Protein ENHANCED DOWNY MILDEW 1) (Protein ENHANCER OF TIR1-1 AUXIN RESISTANCE 3) (Suppressor of G2 allele of SKP1 homolog B)	SGT1B EDM1 ETA3 RPR1 At4g11260 F8L21.50	Arabidopsis thaliana (Mouse-ear cress)		Complete proteome; Cytoplasm; Immunity; Innate immunity; Nucleus; Plant defense; Reference proteome; Repeat; TPR repeat; Ubl conjugation pathway
SRR342022_TR6416_c1_g3_i1_len=954	DIR1_ARATH	Q:788-360,H:37-182	55.41%ID	E:4e-49	Dirigent protein 1	PF03018.11^Dirigent^Dirigent-like protein^49-187^E:4.7e-49	0,905	-0,048	0,001	-1,405	0	1,377	0,507	-0,377	0,127	0,485	DIR1_ARATH	Dirigent protein 1 (AtDIR1)	DIR1 At5g42510 MDH9.21	Arabidopsis thaliana (Mouse-ear cress)		Apoplast; Complete proteome; Glycoprotein; Reference proteome; Secreted; Signal
SRR342022_TR6635_c2_g3_i2_len=1531	COPE1_ARATH	Q:332-1153,H:1-274	82.12%ID	E:4e-171	Coatomer subunit epsilon-1	PF04733.11^Coatomer_E^Coatomer epsilon subunit^7-291^E:5.3e-136	0,129	-5,13	0,001	-3,787	0,001	6,634	1	0	1	0	COPE1_ARATH	Coatomer subunit epsilon-1 (Epsilon-coat protein 1) (Epsilon-COP 1)	At1g30630 T5I8.8	Arabidopsis thaliana (Mouse-ear cress)		Complete proteome; Cytoplasm; Cytoplasmic vesicle; ER-Golgi transport; Golgi apparatus; Membrane; Protein transport; Reference proteome; Transport
SRR342022_TR6870_c0_g16_i2_len=2343	NPC1_ARATH	Q:1651-167,H:32-524	81.21%ID	E:0	Non-specific phospholipase C1	PF04185.11^Phosphoesterase^Phosphoesterase family^42-402^E:4.9e-106	0,244	0,441	0,363	0,352	0	1,753	0,004	1,056	0,032	0,813	NPC1_ARATH	Non-specific phospholipase C1 (EC 3.1.-.-)	NPC1 At1g07230 F10K1.6	Arabidopsis thaliana (Mouse-ear cress)		Complete proteome; Hydrolase; Reference proteome; Secreted; Signal
SRR342022_TR8332_c0_g1_i1_len=751	Y4844_ARATH	Q:120-494,H:17-142	68.25%ID	E:4e-53	Uncharacterized protein At4g28440	.	0,749	-0,149	0,79	-0,131	0	2,486	0,015	1,899	0,356	-0,372	Y4844_ARATH	Uncharacterized protein At4g28440	At4g28440 F20O9.120	Arabidopsis thaliana (Mouse-ear cress)		3D-structure; Acetylation; Complete proteome; Reference proteome
SRR342022_TR8880_c0_g1_i2_len=709	NA	NA	NA	NA	NA	PF00505.16^HMG_box^HMG (high mobility group) box^2-54^E:1.9e-13`PF09011.7^HMG_box_2^HMG-box domain^3-54^E:3.4e-07	0,258	0,867	0,034	-0,856	0	7,044	1	0	0,528	-0,217	.	.	.	.	.	.
SRR342022_TR9797_c6_g3_i2_len=1391	14337_SOLLC	Q:1106-369,H:8-252	91.06%ID	E:2e-160	14-3-3 protein 7	PF00244.17^14-3-3^14-3-3 protein^11-233^E:8.5e-107	0,836	-0,06	0,659	0,128	0	0,966	0,003	0,825	1	-0,323	14337_SOLLC	14-3-3 protein 7	TFT7	Solanum lycopersicum (Tomato) (Lycopersicon esculentum)		Complete proteome; Reference proteome
SRR342022_TR11933_c0_g2_i1_len=521	RPBAL_ARATH	Q:120-326,H:1-69	91.3%ID	E:1e-40	DNA-directed RNA polymerase subunit 10-like protein {ECO:0000303|PubMed:19110459}	.	0	-1,465	1	0,02	0	1,34	0,003	1,032	0,559	-0,174	RPBAL_ARATH	DNA-directed RNA polymerase subunit 10-like protein (ABC10) (DNA-directed RNA polymerase III subunit L) (DNA-directed RNA polymerases I, II, and III subunit RPABC5) (RNA polymerases I, II, and III subunit ABC5) (RPB10 homolog)	NRPB10L At1g61700 T13M11.6	Arabidopsis thaliana (Mouse-ear cress)		Complete proteome; DNA-directed RNA polymerase; Metal-binding; Nucleus; Reference proteome; Transcription; Zinc
SRR342022_TR12779_c1_g5_i1_len=1741	GYAR_THEPD	Q:1124-318,H:58-316	31.54%ID	E:9e-33	Glyoxylate reductase {ECO:0000255|HAMAP-Rule:MF_00776}	PF00389.27^2-Hacid_dh^D-isomer specific 2-hydroxyacid dehydrogenase, catalytic domain^31-338^E:1.5e-14`PF02826.16^2-Hacid_dh_C^D-isomer specific 2-hydroxyacid dehydrogenase, NAD binding domain^125-311^E:4e-45	0,38	-0,492	0,001	-1,5	0,001	2,01	0,001	1,988	0,57	-0,349	GYAR_THEPD	Glyoxylate reductase (EC 1.1.1.26)	gyaR Tpen_0823	Thermofilum pendens (strain Hrk 5)		Complete proteome; Cytoplasm; NAD; Oxidoreductase; Reference proteome
SRR342022_TR13987_c1_g1_i1_len=5352	AB9C_ARATH	Q:4725-583,H:70-1506	62.79%ID	E:0	ABC transporter C family member 9	PF00664.20^ABC_membrane^ABC transporter transmembrane region^324-590^E:3.8e-30`PF00005.24^ABC_tran^ABC transporter^656-790^E:4.5e-19`PF00664.20^ABC_membrane^ABC transporter transmembrane region^960-1041^E:1.6e-08`PF00005.24^ABC_tran^ABC transporter^1217-1365^E:1e-29	0,729	0,306	1	0,165	0	2,174	0,003	1,808	1	-0,15	AB9C_ARATH	ABC transporter C family member 9 (ABC transporter ABCC.9) (AtABCC9) (EC 3.6.3.44) (ATP-energized glutathione S-conjugate pump 9) (Glutathione S-conjugate-transporting ATPase 9) (Multidrug resistance-associated protein 9)	ABCC9 MRP9 At3g60160 T2O9.140	Arabidopsis thaliana (Mouse-ear cress)		ATP-binding; Complete proteome; Hydrolase; Membrane; Nucleotide-binding; Reference proteome; Repeat; Transmembrane; Transmembrane helix; Transport
SRR342022_TR15491_c0_g1_i3_len=813	NA	NA	NA	NA	NA	.	0,015	1,842	0,003	2,847	0	4,205	0,008	3,603	0,692	1,089	.	.	.	.	.	.
SRR342022_TR19399_c0_g1_i2_len=1771	PCP_MOUSE	Q:1266-367,H:181-490	37.34%ID	E:1e-62	Lysosomal Pro-X carboxypeptidase	PF05577.9^Peptidase_S28^Serine carboxypeptidase S28^34-169^E:6.7e-19	0,076	0,709	0,64	0,154	0	1,801	0,022	0,841	0,278	-0,786	PCP_MOUSE	Lysosomal Pro-X carboxypeptidase (EC 3.4.16.2) (Proline carboxypeptidase) (Prolylcarboxypeptidase) (PRCP)	Prcp	Mus musculus (Mouse)		Alternative splicing; Carboxypeptidase; Complete proteome; Disulfide bond; Glycoprotein; Hydrolase; Lysosome; Protease; Reference proteome; Signal; Zymogen
SRR342022_TR19923_c4_g11_i3_len=1420	IDHC_SOLTU	Q:89-1291,H:10-411	90.8%ID	E:0	Isocitrate dehydrogenase [NADP]	PF00180.17^Iso_dh^Isocitrate/isopropylmalate dehydrogenase^6-392^E:8.8e-90	0,443	-0,231	0,036	0,591	0	3,01	0,01	2,179	0,39	0,215	IDHC_SOLTU	Isocitrate dehydrogenase [NADP] (IDH) (EC 1.1.1.42) (IDP) (NADP(+)-specific ICDH) (Oxalosuccinate decarboxylase)	ICDH-1	Solanum tuberosum (Potato)		Complete proteome; Cytoplasm; Glyoxylate bypass; Magnesium; Manganese; Metal-binding; NADP; Oxidoreductase; Reference proteome; Tricarboxylic acid cycle
SRR342022_TR20507_c0_g2_i1_len=558	SODC2_ORYSJ	Q:83-508,H:1-152	80.26%ID	E:2e-82	Superoxide dismutase [Cu-Zn] 2	PF00080.17^Sod_Cu^Copper/zinc superoxide dismutase (SODC)^14-138^E:4.2e-41	0,635	0,36	0,87	0,169	0	1,823	0,002	1,403	0,643	0,43	SODC2_ORYSJ	Superoxide dismutase [Cu-Zn] 2 (EC 1.15.1.1)	SODCC2 SODCC.2 Os07g0665200 LOC_Os07g46990 OJ1343_D04.132 P0450A04.103	Oryza sativa subsp. japonica (Rice)		Antioxidant; Complete proteome; Copper; Cytoplasm; Direct protein sequencing; Disulfide bond; Metal-binding; Oxidoreductase; Reference proteome; Zinc
SRR342022_TR20584_c2_g3_i1_len=2296	CALR3_ARATH	Q:1463-876,H:29-224	82.14%ID	E:1e-155	Calreticulin-3	PF00262.15^Calreticulin^Calreticulin family^30-222^E:4.9e-38	0,824	-0,09	0,55	0,18	0,001	1,33	0,036	0,874	0,101	-0,64	CALR3_ARATH	Calreticulin-3 (Protein PRIORITY IN SWEET LIFE 1)	CRT3 At1g08450 T27G7.13	Arabidopsis thaliana (Mouse-ear cress)		Alternative splicing; Calcium; Chaperone; Complete proteome; Disulfide bond; Endoplasmic reticulum; Glycoprotein; Lectin; Metal-binding; Reference proteome; Repeat; Signal; Zinc
SRR342022_TR20959_c3_g3_i7_len=1300	SDC1_ARATH	Q:1222-182,H:109-453	63.11%ID	E:1e-164	Serine decarboxylase	PF00282.16^Pyridoxal_deC^Pyridoxal-dependent decarboxylase conserved domain^57-291^E:1.6e-23	1	0,024	1	-0,18	0	7,139	1	0	0,452	0,611	SDC1_ARATH	Serine decarboxylase (AtSDC) (EC 4.1.1.-) (Protein EMBRYO DEFECTIVE 1075) (Serine decarboxylase 1) (AtSDC1)	SDC EMB1075 SDC1 At1g43710 F2J6.7	Arabidopsis thaliana (Mouse-ear cress)		Complete proteome; Cytoplasm; Decarboxylase; Lyase; Pyridoxal phosphate; Reference proteome
SRR342022_TR21139_c0_g3_i2_len=2361	SLK2_ARATH	Q:119-919,H:285-551	79.03%ID	E:8e-150	Probable transcriptional regulator SLK2	PF01803.13^LIM_bind^LIM-domain binding protein^50-306^E:1.2e-58	0,074	0,99	0,196	0,792	0	1,737	0,014	1,101	0,377	0,642	SLK2_ARATH	Probable transcriptional regulator SLK2 (AtSLK2) (Protein SEUSS-like 2)	SLK2 At5g62090 MTG10.12	Arabidopsis thaliana (Mouse-ear cress)		Complete proteome; Developmental protein; Differentiation; Flowering; Nucleus; Reference proteome; Transcription; Transcription regulation
SRR342022_TR21139_c0_g3_i3_len=2103	SLK2_ARATH	Q:731-1027,H:498-593	63.64%ID	E:1e-32	Probable transcriptional regulator SLK2	.	0,593	0,784	0,286	0,849	0	1,921	0,031	1,3	0,352	-0,858	SLK2_ARATH	Probable transcriptional regulator SLK2 (AtSLK2) (Protein SEUSS-like 2)	SLK2 At5g62090 MTG10.12	Arabidopsis thaliana (Mouse-ear cress)		Complete proteome; Developmental protein; Differentiation; Flowering; Nucleus; Reference proteome; Transcription; Transcription regulation
SRR342022_TR24993_c0_g1_i1_len=1259	APX2_ARATH	Q:184-927,H:4-251	85.89%ID	E:6e-144	L-ascorbate peroxidase 2, cytosolic	PF00141.20^peroxidase^Peroxidase^25-226^E:1.3e-45	0,075	0,832	0,058	1,008	0	2,068	0,001	1,01	1	-0,182	APX2_ARATH	L-ascorbate peroxidase 2, cytosolic (EC 1.11.1.11) (L-ascorbate peroxidase 1b) (APX1b) (AtAPx02)	APX2 APX1B At3g09640 F11F8_23	Arabidopsis thaliana (Mouse-ear cress)		Calcium; Complete proteome; Cytoplasm; Heme; Hydrogen peroxide; Iron; Metal-binding; Oxidoreductase; Peroxidase; Potassium; Reference proteome
SRR342022_TR25652_c0_g1_i1_len=607	NA	NA	NA	NA	NA	.	0,915	0,039	0,882	0,041	0	1,382	0,011	0,964	0,373	0,326	.	.	.	.	.	.
SRR342022_TR26335_c1_g2_i1_len=1785	KIRO_ACTDE	Q:1732-1283,H:1-150	64.67%ID	E:7e-59	Kirola {ECO:0000303|PubMed:21309790}	PF00407.16^Bet_v_1^Pathogenesis-related protein Bet v I family^19-166^E:1.1e-44	0,167	0,397	0,786	0,097	0	1,183	0,189	0,459	0,043	0,692	KIRO_ACTDE	Kirola (allergen Act d 11)		Actinidia deliciosa (Kiwi)		3D-structure; Acetylation; Allergen; Direct protein sequencing; Polymorphism
SRR342022_TR26881_c0_g1_i1_len=851	XTH28_ARATH	Q:711-202,H:139-308	64.12%ID	E:5e-79	Probable xyloglucan endotransglucosylase/hydrolase protein 28	PF00722.18^Glyco_hydro_16^Glycosyl hydrolases family 16^3-34^E:1.1e-05`PF06955.9^XET_C^Xyloglucan endo-transglycosylase (XET) C-terminus^73-109^E:7e-14	0,195	1,183	0,597	0,421	0	1,833	0,02	1,338	1	-1,175	XTH28_ARATH	Probable xyloglucan endotransglucosylase/hydrolase protein 28 (At-XTH28) (XTH-28) (EC 2.4.1.207)	XTH28 EXGT-A2 XTR2 At1g14720 F10B6.12 F10B6.29	Arabidopsis thaliana (Mouse-ear cress)		Apoplast; Cell wall; Cell wall biogenesis/degradation; Complete proteome; Disulfide bond; Glycoprotein; Glycosidase; Hydrolase; Reference proteome; Secreted; Signal; Transferase
SRR342022_TR28339_c0_g1_i1_len=838	PHF5B_ARATH	Q:184-477,H:1-98	98.98%ID	E:1e-62	PHD finger-like domain-containing protein 5B	PF03660.11^PHF5^PHF5-like protein^1-104^E:2.2e-50	0,941	0,048	0,081	0,666	0	1,409	0,002	1,24	0,331	0,358	PHF5B_ARATH	PHD finger-like domain-containing protein 5B	At1g07170 F10K1.12	Arabidopsis thaliana (Mouse-ear cress)		Complete proteome; Reference proteome
SRR342022_TR28808_c0_g2_i1_len=746	IPRT1_CATRO	Q:41-520,H:1-157	70%ID	E:2e-68	Probable intracellular pathogenesis-related protein T1	PF00407.16^Bet_v_1^Pathogenesis-related protein Bet v I family^5-157^E:1.4e-18	1	2,049	1	2,98	0	6,661	1	1,791	1	0	IPRT1_CATRO	Probable intracellular pathogenesis-related protein T1	PCKR3	Catharanthus roseus (Madagascar periwinkle) (Vinca rosea)		Direct protein sequencing; Glycoprotein; Pathogenesis-related protein; Plant defense
SRR342022_TR32961_c0_g1_i1_len=1309	NA	NA	NA	NA	NA	.	1	0,055	0,793	0,196	0,001	1,506	0,007	1,234	1	0,029	.	.	.	.	.	.
SRR342022_TR33610_c0_g1_i2_len=1726	C7D55_HYOMU	Q:1586-165,H:26-497	53.67%ID	E:2e-158	Premnaspirodiene oxygenase	PF00067.19^p450^Cytochrome P450^54-505^E:7.2e-100	0,009	1,142	0,316	0,677	0	1,687	0,011	1,102	1	0,388	C7D55_HYOMU	Premnaspirodiene oxygenase (HPO) (EC 1.14.13.121) (Cytochrome P450 71D55)	CYP71D55	Hyoscyamus muticus (Egyptian henbane)		Heme; Iron; Membrane; Metal-binding; Monooxygenase; NADP; Oxidoreductase; Transmembrane; Transmembrane helix
SRR342023_TR1002_c0_g1_i2_len=1069	NA	NA	NA	NA	NA	.	0,196	-0,767	0,298	-0,614	0,001	6,656	0,009	6,012	0,1	-0,75	.	.	.	.	.	.
SRR342023_TR5701_c4_g3_i3_len=2131	NA	NA	NA	NA	NA	PF03879.11^Cgr1^Cgr1 family^73-177^E:6.9e-07	0,604	0,118	0	-0,865	0,001	1,262	0,044	0,832	0,548	-0,297	.	.	.	.	.	.
SRR342023_TR12049_c0_g1_i1_len=1863	GLNA1_VITVI	Q:456-1478,H:16-356	93.26%ID	E:0	Glutamine synthetase cytosolic isozyme 1	PF03951.16^Gln-synt_N^Glutamine synthetase, beta-Grasp domain^22-97^E:1.2e-12`PF00120.21^Gln-synt_C^Glutamine synthetase, catalytic domain^167-347^E:3.6e-15	0,017	0,87	0,04	0,867	0,001	0,997	0,106	0,51	0,163	0,418	GLNA1_VITVI	Glutamine synthetase cytosolic isozyme 1 (EC 6.3.1.2) (Glutamate–ammonia ligase)	GS1-1 GS1;1	Vitis vinifera (Grape)		ATP-binding; Cytoplasm; Ligase; Nucleotide-binding
SRR342023_TR12198_c1_g3_i7_len=968	AMYB_SOYBN	Q:48-911,H:204-495	68.49%ID	E:3e-143	Beta-amylase	PF01373.14^Glyco_hydro_14^Glycosyl hydrolase family 14^1-132^E:8.7e-23	0,009	0,578	0,698	0,093	0	1,38	0,463	0,189	1	0,718	AMYB_SOYBN	Beta-amylase (EC 3.2.1.2) (1,4-alpha-D-glucan maltohydrolase)	BMY1	Glycine max (Soybean) (Glycine hispida)		3D-structure; Acetylation; Carbohydrate metabolism; Complete proteome; Direct protein sequencing; Glycosidase; Hydrolase; Polysaccharide degradation; Reference proteome
SRR342023_TR12287_c4_g5_i2_len=736	NLTP_HELAN	Q:736-338,H:10-116	44.36%ID	E:3e-22	Non-specific lipid-transfer protein	PF00234.19^Tryp_alpha_amyl^Protease inhibitor/seed storage/LTP family^21-129^E:1.1e-08	0,064	0,49	0,022	0,623	0	2,075	0,032	0,749	0,856	0,111	NLTP_HELAN	Non-specific lipid-transfer protein (LTP) (NsLTP) (SDI-9)		Helianthus annuus (Common sunflower)		Disulfide bond; Lipid-binding; Signal; Transport
SRR342023_TR17904_c3_g4_i1_len=1093	CSPLF_POPTR	Q:796-326,H:34-187	41.4%ID	E:3e-31	CASP-like protein 1F3	PF04535.9^DUF588^Domain of unknown function (DUF588)^16-161^E:1.8e-22	1	0	1	0	0	7,868	1	0	1	0	CSPLF_POPTR	CASP-like protein 1F3 (PtCASPL1F3)	POPTRDRAFT_752786	Populus trichocarpa (Western balsam poplar) (Populus balsamifera subsp. trichocarpa)		Cell membrane; Complete proteome; Membrane; Reference proteome; Transmembrane; Transmembrane helix
SRR342023_TR20015_c0_g3_i1_len=559	NA	NA	NA	NA	NA	.	0,005	1,226	0,392	0,335	0	1,204	0,063	0,689	1	-1,948	.	.	.	.	.	.
SRR342023_TR24805_c2_g5_i3_len=2384	GLGB_SOLTU	Q:1919-336,H:247-768	85.23%ID	E:0	1,4-alpha-glucan-branching enzyme	PF00128.21^Alpha-amylase^Alpha amylase, catalytic domain^93-191^E:8.6e-11`PF02806.15^Alpha-amylase_C^Alpha amylase, C-terminal all-beta domain^463-564^E:1.5e-24	0,371	0,369	0,679	0,231	0	1,78	0,034	1,106	0,402	0,78	GLGB_SOLTU	1,4-alpha-glucan-branching enzyme (EC 2.4.1.18) (Q-enzyme) (Starch-branching enzyme)	SBE1 SBE	Solanum tuberosum (Potato)	PATHWAY: Glycan biosynthesis; starch biosynthesis.	Amyloplast; Chloroplast; Complete proteome; Glycosyltransferase; Plastid; Reference proteome; Starch biosynthesis; Transferase
SRR342023_TR26191_c0_g1_i2_len=1283	PPCE_MOUSE	Q:1282-578,H:482-709	62.98%ID	E:2e-102	Prolyl endopeptidase	PF00326.18^Peptidase_S9^Prolyl oligopeptidase family^2-231^E:2.6e-67	0,173	0,674	0,055	0,738	0,001	1,389	0,001	1,353	0,258	0,923	PPCE_MOUSE	Prolyl endopeptidase (PE) (EC 3.4.21.26) (Post-proline cleaving enzyme)	Prep Pep	Mus musculus (Mouse)		Acetylation; Complete proteome; Cytoplasm; Hydrolase; Protease; Reference proteome; Serine protease
SRR342023_TR26608_c0_g1_i1_len=783	PRB1_TOBAC	Q:54-524,H:4-159	62.66%ID	E:9e-61	Basic form of pathogenesis-related protein 1	PF00188.23^CAP^Cysteine-rich secretory protein family^44-162^E:5.1e-22	1	3,255	1	1,467	0	6,232	0,129	4,139	1	1,445	PRB1_TOBAC	Basic form of pathogenesis-related protein 1 (PRP 1)		Nicotiana tabacum (Common tobacco)		Disulfide bond; Pathogenesis-related protein; Plant defense; Pyrrolidone carboxylic acid; Signal
SRR342023_TR28516_c2_g2_i5_len=759	KIRO_ACTDE	Q:272-709,H:3-148	46.58%ID	E:1e-33	Kirola {ECO:0000303|PubMed:21309790}	PF00407.16^Bet_v_1^Pathogenesis-related protein Bet v I family^5-141^E:5e-32	0,19	-0,356	0,009	1,04	0	1,505	0,079	0,583	1	0,474	KIRO_ACTDE	Kirola (allergen Act d 11)		Actinidia deliciosa (Kiwi)		3D-structure; Acetylation; Allergen; Direct protein sequencing; Polymorphism
SRR342023_TR29130_c0_g1_i1_len=925	NA	NA	NA	NA	NA	PF00643.21^zf-B_box^B-box zinc finger^35-74^E:6.1e-06	1	0,392	0,502	2,878	0	1,524	0,516	0,236	1	-0,319	.	.	.	.	.	.
SRR646572_TR11666_c0_g1_i1_len=724	EGC_CITJA	Q:97-477,H:1-129	50.77%ID	E:1e-33	EG45-like domain containing protein	PF03330.15^DPBB_1^Lytic transglycolase^50-126^E:2.5e-15	1	3,108	1	1,795	0	6,956	1	3,7	1	0,876	EGC_CITJA	EG45-like domain containing protein (Blight-associated protein p12) (Plant natriuretic peptide) (PNP)	CjBAp12	Citrus jambhiri (Rough lemon)		Direct protein sequencing; Secreted; Signal
SRR646596_TR3041_c0_g1_i8_len=737	PSB6_ARATH	Q:163-468,H:137-233	74.51%ID	E:2e-45	Proteasome subunit beta type-6	.	0,913	-0,048	0,762	-0,104	0	1,258	0,018	0,835	0,552	-0,156	PSB6_ARATH	Proteasome subunit beta type-6 (EC 3.4.25.1) (20S proteasome beta subunit A-1) (Proteasome component D) (Proteasome subunit beta type-1)	PBA1 PRCD At4g31300 F8F16.120	Arabidopsis thaliana (Mouse-ear cress)		Alternative splicing; Complete proteome; Cytoplasm; Hydrolase; Nucleus; Protease; Proteasome; Reference proteome; Threonine protease; Zymogen
SRR646596_TR7023_c0_g1_i1_len=576	NA	NA	NA	NA	NA	.	0,003	2,109	0,003	2,61	0	2,889	0,016	2,138	0,083	0,782	.	.	.	.	.	.
SRR646596_TR12660_c0_g1_i3_len=750	PIN4_BOVIN	Q:498-187,H:33-131	49.09%ID	E:2e-23	Peptidyl-prolyl cis-trans isomerase NIMA-interacting 4	PF13616.3^Rotamase_3^PPIC-type PPIASE domain^46-138^E:4.4e-19`PF00639.18^Rotamase^PPIC-type PPIASE domain^50-139^E:6.4e-18	0,78	0,091	0,57	0,174	0,001	1,017	0,008	0,823	0,008	0,907	PIN4_BOVIN	Peptidyl-prolyl cis-trans isomerase NIMA-interacting 4 (EC 5.2.1.8) (Parvulin-14) (Par14) (Peptidyl-prolyl cis-trans isomerase Pin4) (PPIase Pin4) (Rotamase Pin4)	PIN4	Bos taurus (Bovine)		Complete proteome; Cytoplasm; Cytoskeleton; DNA-binding; Isomerase; Nucleus; Phosphoprotein; Reference proteome; Rotamase
SRR646596_TR13781_c0_g1_i1_len=1344	Y8359_ORYSI	Q:1246-947,H:1-100	68%ID	E:4e-33	Uncharacterized protein OsI_027940	PF04969.13^CS^CS domain^6-81^E:4.6e-09	0,078	-0,428	0,053	0,479	0	1,038	0,016	0,69	1	0,072	Y8359_ORYSI	Uncharacterized protein OsI_027940	OsI_027940 OsI_28951	Oryza sativa subsp. indica (Rice)		Complete proteome; Reference proteome
SRR646596_TR14059_c0_g1_i1_len=385	NA	NA	NA	NA	NA	.	0,339	-0,298	0,323	-0,293	0,001	1,092	0,002	0,986	0,054	-0,627	.	.	.	.	.	.
SRR646596_TR16896_c1_g3_i1_len=1134	PIP1_SOLLC	Q:42-881,H:1-280	85.36%ID	E:7e-176	Probable aquaporin PIP-type pTOM75	PF00230.17^MIP^Major intrinsic protein^58-286^E:4.6e-83	1	-0,059	0,754	-0,192	0	1,386	0,022	0,851	0,133	-0,355	PIP1_SOLLC	Probable aquaporin PIP-type pTOM75 (Ripening-associated membrane protein) (RAMP)		Solanum lycopersicum (Tomato) (Lycopersicon esculentum)		Cell membrane; Complete proteome; Membrane; Reference proteome; Repeat; Stress response; Transmembrane; Transmembrane helix; Transport
SRR646596_TR19537_c0_g1_i5_len=2111	IDHC_SOLTU	Q:1842-613,H:1-411	91.48%ID	E:0	Isocitrate dehydrogenase [NADP]	PF00180.17^Iso_dh^Isocitrate/isopropylmalate dehydrogenase^7-402^E:4.7e-92	0,493	0,173	0,005	0,696	0	2,198	0,029	1,294	0,932	0,036	IDHC_SOLTU	Isocitrate dehydrogenase [NADP] (IDH) (EC 1.1.1.42) (IDP) (NADP(+)-specific ICDH) (Oxalosuccinate decarboxylase)	ICDH-1	Solanum tuberosum (Potato)		Complete proteome; Cytoplasm; Glyoxylate bypass; Magnesium; Manganese; Metal-binding; NADP; Oxidoreductase; Reference proteome; Tricarboxylic acid cycle
SRR646596_TR32608_c0_g2_i1_len=1141	CATB_PONAB	Q:1053-157,H:28-334	46.56%ID	E:4e-75	Cathepsin B	PF08127.10^Propeptide_C1^Peptidase family C1 propeptide^4-43^E:2.7e-09`PF00112.20^Peptidase_C1^Papain family cysteine protease^63-297^E:1.9e-64	0,34	0,473	0,038	-0,697	0,001	6,621	0,254	4,759	1	2,641	CATB_PONAB	Cathepsin B (EC 3.4.22.1) [Cleaved into: Cathepsin B light chain; Cathepsin B heavy chain]	CTSB	Pongo abelii (Sumatran orangutan) (Pongo pygmaeus abelii)		Acetylation; Complete proteome; Disulfide bond; Glycoprotein; Hydrolase; Lysosome; Protease; Reference proteome; Secreted; Signal; Thiol protease; Zymogen
SRR646604_TR1237_c0_g1_i1_len=776	NA	NA	NA	NA	NA	.	1	1,781	0,129	4,419	0	4,835	1	1,144	1	-1,759	.	.	.	.	.	.
SRR646604_TR5547_c0_g2_i6_len=1697	S35F2_HUMAN	Q:422-1318,H:38-345	34.09%ID	E:2e-43	Solute carrier family 35 member F2	PF06027.9^SLC35F^Solute carrier family 35^15-301^E:1.3e-90`PF00892.17^EamA^EamA-like transporter family^48-149^E:1.3e-06`PF08449.8^UAA^UAA transporter family^54-302^E:5.1e-10`PF08627.7^CRT-like^CRT-like, chloroquine-resistance transporter-like^72-215^E:1.6e-09`PF00892.17^EamA^EamA-like transporter family^165-300^E:4e-08	1	0,512	0,002	6,277	0,001	6,558	0,002	6,283	0,257	0,873	S35F2_HUMAN	Solute carrier family 35 member F2	SLC35F2	Homo sapiens (Human)		Acetylation; Alternative splicing; Complete proteome; Membrane; Phosphoprotein; Reference proteome; Transmembrane; Transmembrane helix; Transport
SRR646604_TR8057_c0_g1_i35_len=5058	LOX21_SOLTU	Q:115-2094,H:1-650	63.61%ID	E:0	Linoleate 13S-lipoxygenase 2-1, chloroplastic	PF01477.20^PLAT^PLAT/LH2 domain^144-212^E:4e-07`PF00305.16^Lipoxygenase^Lipoxygenase^225-659^E:1.7e-194	0,017	5,925	0,002	2,921	0	3,477	0,003	2,996	1	0,65	LOX21_SOLTU	Linoleate 13S-lipoxygenase 2-1, chloroplastic (EC 1.13.11.12) (Lipoxygenase 2-1)	LOX2.1 LOX-H1	Solanum tuberosum (Potato)	PATHWAY: Lipid metabolism; oxylipin biosynthesis. {ECO:0000255|PROSITE-ProRule:PRU00726}.	Chloroplast; Complete proteome; Dioxygenase; Fatty acid biosynthesis; Fatty acid metabolism; Iron; Lipid biosynthesis; Lipid metabolism; Metal-binding; Oxidoreductase; Oxylipin biosynthesis; Plastid; Reference proteome; Thylakoid; Transit peptide
SRR646604_TR16195_c0_g1_i1_len=624	PTR53_ARATH	Q:77-622,H:27-208	62.64%ID	E:2e-75	Protein NRT1/ PTR FAMILY 2.11	PF00854.18^PTR2^POT family^89-181^E:1.7e-10	1	2,705	0,034	5,668	0,001	4,309	0,014	3,671	1	0	PTR53_ARATH	Protein NRT1/ PTR FAMILY 2.11 (AtNPF2.11) (Nitrate transporter 1.10) (Protein GLUCOSINOLATE TRANSPORTER-2)	NPF2.11 GTR2 NRT1.10 At5g62680 MRG21.10	Arabidopsis thaliana (Mouse-ear cress)		Cell membrane; Complete proteome; Membrane; Reference proteome; Transmembrane; Transmembrane helix; Transport
SRR646604_TR16508_c0_g1_i1_len=1761	AVP_VIGRR	Q:245-1336,H:403-766	98.08%ID	E:0	Pyrophosphate-energized vacuolar membrane proton pump	PF03030.13^H_PPase^Inorganic H+ pyrophosphatase^1-273^E:5e-130	0,578	-0,166	0,203	-0,322	0	2,119	0,376	0,421	0	-1,701	AVP_VIGRR	Pyrophosphate-energized vacuolar membrane proton pump (EC 3.6.1.1) (Pyrophosphate-energized inorganic pyrophosphatase) (H(+)-PPase) (Vacuolar H(+)-pyrophosphatase)		Vigna radiata var. radiata (Mung bean) (Phaseolus aureus)		3D-structure; Direct protein sequencing; Disulfide bond; Hydrogen ion transport; Hydrolase; Ion transport; Magnesium; Membrane; Metal-binding; Transmembrane; Transmembrane helix; Transport; Vacuole
SRR646604_TR29352_c0_g1_i7_len=2095	ACCO3_PETHY	Q:1919-1596,H:4-111	81.48%ID	E:5e-56	1-aminocyclopropane-1-carboxylate oxidase 3	PF14226.3^DIOX_N^non-haem dioxygenase in morphine synthesis N-terminal^60-149^E:1.1e-12	0,066	-5,283	1	0	0	6,965	1	0	0,129	-4,934	ACCO3_PETHY	1-aminocyclopropane-1-carboxylate oxidase 3 (ACC oxidase 3) (EC 1.14.17.4) (Ethylene-forming enzyme) (EFE)	ACO3	Petunia hybrida (Petunia)	PATHWAY: Alkene biosynthesis; ethylene biosynthesis via S-adenosyl-L-methionine; ethylene from S-adenosyl-L-methionine: step 2/2.	Ethylene biosynthesis; Iron; Metal-binding; Oxidoreductase; Vitamin C
SRR646604_TR30811_c0_g1_i1_len=669	NA	NA	NA	NA	NA	.	1	0,207	0,132	2,323	0	5,419	0,005	4,32	1	-0,037	.	.	.	.	.	.
SRR646604_TR38185_c0_g1_i2_len=1744	NAC72_ARATH	Q:400-963,H:62-241	68.59%ID	E:6e-96	NAC domain-containing protein 72	.	1	0,304	0,009	5,978	0	6,854	0,005	6,138	0,254	-4,846	NAC72_ARATH	NAC domain-containing protein 72 (ANAC072) (Protein RESPONSIVE TO DESICCATION 26)	NAC072 RD26 At4g27410 F27G19.10	Arabidopsis thaliana (Mouse-ear cress)		Alternative splicing; Complete proteome; DNA-binding; Nucleus; Reference proteome; Transcription; Transcription regulation
SRR648705_TR5819_c1_g1_i1_len=409	NA	NA	NA	NA	NA	.	0,001	-6,657	0,283	0,63	0,001	2,969	0,066	2,09	0,692	-1,004	.	.	.	.	.	.
SRR648705_TR15564_c0_g1_i2_len=2040	APA1_ARATH	Q:1767-271,H:5-505	64.61%ID	E:0	Aspartic proteinase A1	PF00026.20^Asp^Eukaryotic aspartyl protease^78-501^E:9e-128`PF14543.3^TAXi_N^Xylanase inhibitor N-terminal^79-237^E:1.1e-13`PF03489.14^SapB_2^Saposin-like type B, region 2^313-345^E:1.2e-12`PF05184.12^SapB_1^Saposin-like type B, region 1^375-411^E:2.3e-12	0,5	0,185	0,002	0,944	0	1,256	0,03	0,743	0,014	1,252	APA1_ARATH	Aspartic proteinase A1 (EC 3.4.23.-)	APA1 PRA1 At1g11910 F12F1.24	Arabidopsis thaliana (Mouse-ear cress)		Aspartyl protease; Complete proteome; Disulfide bond; Glycoprotein; Hydrolase; Protease; Reference proteome; Repeat; Signal; Vacuole; Zymogen
SRR648705_TR15715_c0_g1_i2_len=1693	WTR7_ARATH	Q:1532-564,H:4-317	43.45%ID	E:2e-75	WAT1-related protein At1g43650	PF00892.17^EamA^EamA-like transporter family^195-333^E:1.2e-17	1	1,812	1	1,6	0	4,208	0,025	3,262	1	0,637	WTR7_ARATH	WAT1-related protein At1g43650	At1g43650 F2J6.1	Arabidopsis thaliana (Mouse-ear cress)		Alternative splicing; Complete proteome; Membrane; Reference proteome; Repeat; Transmembrane; Transmembrane helix

SRR648705_TR18488_c0_g3_i1_len=1982	NA	NA	NA	NA	NA	PF04640.11^PLATZ^PLATZ transcription factor^48-119^E:5.7e-30	0,692	1,272	0,008	2,839	0	2,646	0,002	2,474	1	3,044	.	.	.	.	.	.
SRR648705_TR19299_c0_g3_i2_len=590	IF5A2_NICPL	Q:242-415,H:102-159	89.66%ID	E:1e-30	Eukaryotic translation initiation factor 5A-2	.	0,424	-0,209	0,707	0,093	0	1,232	0,054	0,629	0,088	-0,45	IF5A2_NICPL	Eukaryotic translation initiation factor 5A-2 (eIF-5A-2) (eIF-4D)	EIF-5A2	Nicotiana plumbaginifolia (Leadwort-leaved tobacco) (Tex-Mex tobacco)		Hypusine; Initiation factor; Protein biosynthesis
SRR648705_TR24298_c0_g2_i1_len=1980	ORYA_ORYSJ	Q:1735-449,H:32-448	46.06%ID	E:1e-114	Oryzain alpha chain	PF00112.20^Peptidase_C1^Papain family cysteine protease^62-277^E:2.2e-78`PF00396.15^Granulin^Granulin^319-365^E:1.7e-10	0,027	0,803	0,631	-0,204	0	1,764	0,053	0,904	0,319	0,918	ORYA_ORYSJ	Oryzain alpha chain (EC 3.4.22.-)	Os04g0650000 LOC_Os04g55650 H0212B02.7 OSJNBb0059K02.8	Oryza sativa subsp. japonica (Rice)		Complete proteome; Direct protein sequencing; Disulfide bond; Glycoprotein; Hydrolase; Protease; Reference proteome; Signal; Thiol protease; Zymogen
SRR648705_TR27003_c0_g1_i1_len=1817	NA	NA	NA	NA	NA	PF05678.11^VQ^VQ motif^157-184^E:5.2e-11	0,299	1,661	0,003	3,952	0	2,89	0,017	2,16	1	-0,013	.	.	.	.	.	.
SRR648705_TR34506_c0_g2_i2_len=1540	RBG3_ARATH	Q:1350-991,H:1-120	68.33%ID	E:6e-50	Glycine-rich RNA-binding protein 3, mitochondrial	PF00076.19^RRM_1^RNA recognition motif. (a.k.a. RRM, RBD, or RNP domain)^42-112^E:2.2e-23`PF13893.3^RRM_5^RNA recognition motif. (a.k.a. RRM, RBD, or RNP domain)^58-116^E:0.031	0,013	-0,629	0,205	0,306	0	1,478	0,056	0,851	0,312	-0,327	RBG3_ARATH	Glycine-rich RNA-binding protein 3, mitochondrial (AtGR-RBP3) (AtRBG3) (Mitochondrial RNA-binding protein 2a) (At-mRBP2a)	RBG3 GR-RBP3 MRBP2A At5g61030 MAF19_30	Arabidopsis thaliana (Mouse-ear cress)		Complete proteome; Mitochondrion; RNA-binding; Reference proteome; Transit peptide
SRR648705_TR35323_c0_g2_i1_len=514	NA	NA	NA	NA	NA	.	1	-3,66	0	-1,375	0	6,869	0,001	6,413	0	-2,501	.	.	.	.	.	.
SRR648705_TR38889_c0_g3_i1_len=1558	P2C21_ARATH	Q:211-1200,H:1-332	73.8%ID	E:0	Probable protein phosphatase 2C 21	PF00481.18^PP2C^Protein phosphatase 2C^23-102^E:1.2e-12`PF00481.18^PP2C^Protein phosphatase 2C^139-312^E:2.7e-61	0,917	0,102	0,918	-0,078	0	1,758	0,008	1,263	0,908	-0,099	P2C21_ARATH	Probable protein phosphatase 2C 21 (AtPP2C21) (EC 3.1.3.16) (AtPPC4;2)	PPC4-2 At2g25070 F13D4.1 F27C12	Arabidopsis thaliana (Mouse-ear cress)		Complete proteome; Hydrolase; Magnesium; Manganese; Metal-binding; Protein phosphatase; Reference proteome
SRR648705_TR44621_c0_g1_i1_len=915	CCB12_ORYSJ	Q:747-406,H:1-114	45.22%ID	E:2e-22	Cyclin-B1-2	PF05348.8^UMP1^Proteasome maturation factor UMP1^72-185^E:1.2e-29	1	-0,056	0,608	0,27	0	2,047	0,002	1,786	1	0,383	CCB12_ORYSJ	Cyclin-B1-2 (G2/mitotic-specific cyclin-B1-2) (CycB1;2)	CYCB1-2 Os02g0801300 LOC_Os02g55720	Oryza sativa subsp. japonica (Rice)		Cell cycle; Cell division; Complete proteome; Cyclin; Reference proteome
SRR648705_TR45105_c0_g1_i3_len=1616	FB330_ARATH	Q:260-1270,H:19-353	69.14%ID	E:3e-157	F-box protein At3g58530	PF13516.3^LRR_6^Leucine Rich repeat^118-136^E:0.0078`PF13516.3^LRR_6^Leucine Rich repeat^170-188^E:1.6`PF13516.3^LRR_6^Leucine Rich repeat^270-294^E:0.0087	0,62	-0,459	0,189	1,595	0,001	6,611	0,005	6,152	0,811	0,365	FB330_ARATH	F-box protein At3g58530	At3g58530 F14P22.120	Arabidopsis thaliana (Mouse-ear cress)		Complete proteome; Reference proteome
SRR648707_TR1542_c0_g1_i1_len=804	PIR7A_ORYSJ	Q:38-667,H:52-262	35.98%ID	E:2e-41	Probable esterase PIR7A	.	0,897	0,152	0,042	-0,936	0	2,157	0,001	1,933	0,518	-0,337	PIR7A_ORYSJ	Probable esterase PIR7A (EC 3.1.-.-)	PIR7A Os01g0934900 LOC_Os01g70860 P0423A12.27 P0492G09.8	Oryza sativa subsp. japonica (Rice)		Complete proteome; Hydrolase; Reference proteome; Serine esterase
SRR648707_TR10326_c0_g2_i2_len=2205	ORYA_ORYSJ	Q:177-1463,H:32-448	46.06%ID	E:4e-114	Oryzain alpha chain	PF08246.9^Inhibitor_I29^Cathepsin propeptide inhibitor domain (I29)^49-108^E:6.5e-12`PF00112.20^Peptidase_C1^Papain family cysteine protease^142-357^E:3.4e-78`PF00396.15^Granulin^Granulin^399-445^E:2.1e-10	0,396	0,242	0,278	0,357	0	1,232	0,026	0,7	0,007	0,707	ORYA_ORYSJ	Oryzain alpha chain (EC 3.4.22.-)	Os04g0650000 LOC_Os04g55650 H0212B02.7 OSJNBb0059K02.8	Oryza sativa subsp. japonica (Rice)		Complete proteome; Direct protein sequencing; Disulfide bond; Glycoprotein; Hydrolase; Protease; Reference proteome; Signal; Thiol protease; Zymogen
SRR648707_TR11505_c0_g2_i1_len=1892	SCP19_ARATH	Q:177-1577,H:3-465	52.67%ID	E:2e-169	Serine carboxypeptidase-like 19	PF00450.19^Peptidase_S10^Serine carboxypeptidase^35-468^E:6.9e-121	1	-0,01	1	0	0	2,747	0,006	2,175	1	0	SCP19_ARATH	Serine carboxypeptidase-like 19 (Protein SINAPOYLGLUCOSE ACCUMULATOR 2) (Sinapoylglucose--choline O-sinapoyltransferase) (SCT) (EC 2.3.1.91) [Cleaved into: Serine carboxypeptidase-like 19 chain A; Serine carboxypeptidase-like 19 chain B]	SCPL19 SCT SNG2 At5g09640 F17I14.170	Arabidopsis thaliana (Mouse-ear cress)		3D-structure; Acyltransferase; Complete proteome; Disulfide bond; Glycoprotein; Reference proteome; Secreted; Signal; Transferase
SRR648707_TR14283_c1_g2_i5_len=779	CGS1_ARATH	Q:97-345,H:481-563	84.34%ID	E:2e-40	Cystathionine gamma-synthase 1, chloroplastic {ECO:0000305}	.	0,691	0,208	0	-1,307	0	6,697	1	0	0	-3,012	CGS1_ARATH	Cystathionine gamma-synthase 1, chloroplastic (AtCGS1) (EC 2.5.1.48) (METHIONINE OVERACCUMULATION 1) (O-succinylhomoserine (thiol)-lyase)	CGS1 CYS1 MTO1 At3g01120 T4P13.19	Arabidopsis thaliana (Mouse-ear cress)	PATHWAY: Amino-acid biosynthesis; L-methionine biosynthesis via de novo pathway; L-cystathionine from O-succinyl-L-homoserine: step 1/1. {ECO:0000305}.	Amino-acid biosynthesis; Chloroplast; Complete proteome; Direct protein sequencing; Methionine biosynthesis; Plastid; Polymorphism; Pyridoxal phosphate; Reference proteome; Transferase; Transit peptide
SRR648707_TR16194_c0_g1_i1_len=984	ISAM1_ARATH	Q:804-412,H:1-134	73.88%ID	E:5e-65	Iron-sulfur assembly protein IscA-like 1, mitochondrial	PF01521.17^Fe-S_biosyn^Iron-sulphur cluster biosynthesis^80-179^E:2.3e-19	0,842	-0,146	0,042	0,906	0,001	1,313	0,002	1,244	0,882	-0,144	ISAM1_ARATH	Iron-sulfur assembly protein IscA-like 1, mitochondrial	At2g16710 T24I21.12	Arabidopsis thaliana (Mouse-ear cress)		Alternative splicing; Complete proteome; Iron; Iron-sulfur; Metal-binding; Mitochondrion; Reference proteome; Transit peptide
SRR648707_TR17590_c0_g2_i9_len=1614	GSTZ_EUPES	Q:301-927,H:11-219	65.55%ID	E:8e-95	Glutathione S-transferase zeta class	PF02798.17^GST_N^Glutathione S-transferase, N-terminal domain^13-86^E:2.9e-17`PF13417.3^GST_N_3^Glutathione S-transferase, N-terminal domain^16-92^E:2.1e-18`PF13409.3^GST_N_2^Glutathione S-transferase, N-terminal domain^22-87^E:2.9e-17`PF14497.3^GST_C_3^Glutathione S-transferase, C-terminal domain^139-208^E:0.0001	0,547	0,318	0,242	0,549	0	1,703	0,008	1,333	0,937	-0,052	GSTZ_EUPES	Glutathione S-transferase zeta class (EC 2.5.1.18)		Euphorbia esula (Leafy spurge)		Cytoplasm; Transferase
SRR648707_TR19558_c0_g2_i9_len=2095	C72A1_CATRO	Q:1156-317,H:245-524	97.86%ID	E:0	Secologanin synthase	PF00067.19^p450^Cytochrome P450^93-235^E:1.4e-16	0,039	0,999	0,013	1,59	0	1,972	0,005	1,37	0,075	0,857	C72A1_CATRO	Secologanin synthase (SLS) (EC 1.3.3.9) (CYPLXXII) (Cytochrome P450 72A1)	CYP72A1 CYP72 P450CR3	Catharanthus roseus (Madagascar periwinkle) (Vinca rosea)	PATHWAY: Alkaloid biosynthesis; secologanin biosynthesis.	Alkaloid metabolism; Heme; Iron; Membrane; Metal-binding; Monooxygenase; NADP; Oxidoreductase; Transmembrane; Transmembrane helix; Vacuole
SRR648707_TR22887_c0_g3_i1_len=1374	DHAR2_ARATH	Q:1012-383,H:3-213	72.99%ID	E:2e-107	Glutathione S-transferase DHAR2	PF13417.3^GST_N_3^Glutathione S-transferase, N-terminal domain^21-89^E:5.3e-19`PF02798.17^GST_N^Glutathione S-transferase, N-terminal domain^21-79^E:1.5e-06`PF13409.3^GST_N_2^Glutathione S-transferase, N-terminal domain^22-84^E:1e-14`PF13410.3^GST_C_2^Glutathione S-transferase, C-terminal domain^121-184^E:6.2e-08	0,11	0,483	0,031	0,668	0	1,152	0,003	0,884	0,708	0,125	DHAR2_ARATH	Glutathione S-transferase DHAR2 (EC 2.5.1.18) (Chloride intracellular channel homolog 2) (CLIC homolog 2) (Glutathione-dependent dehydroascorbate reductase 2) (AtDHAR2) (CytDHAR) (GSH-dependent dehydroascorbate reductase 2)	DHAR2 DHAR At1g75270 F22H5.1	Arabidopsis thaliana (Mouse-ear cress)		Complete proteome; Cytoplasm; Detoxification; Glutathionylation; Reference proteome; Stress response; Transferase
SRR648707_TR28023_c0_g8_i1_len=844	NA	NA	NA	NA	NA	.	0,538	-0,189	0,053	0,524	0	1,085	0,003	0,919	0,87	-0,059	.	.	.	.	.	.
SRR648707_TR28049_c0_g2_i1_len=2065	DAT_CATRO	Q:96-761,H:11-237	46.72%ID	E:1e-69	Deacetylvindoline O-acetyltransferase	PF02458.12^Transferase^Transferase family^6-192^E:2.7e-32	0,057	0,63	0,027	0,817	0	1,491	0,026	0,983	0,011	0,828	DAT_CATRO	Deacetylvindoline O-acetyltransferase (EC 2.3.1.107) (Acetyl-coenzyme A:deacetylvindoline 4-O-acetyltransferase)	DAT	Catharanthus roseus (Madagascar periwinkle) (Vinca rosea)	PATHWAY: Alkaloid biosynthesis; vindoline biosynthesis; vindoline from tabersonine: step 6/6.	Acyltransferase; Coiled coil; Direct protein sequencing; Transferase
SRR648707_TR32373_c0_g1_i1_len=831	H2B_GOSHI	Q:507-199,H:45-147	96.12%ID	E:3e-58	Histone H2B	PF00125.21^Histone^Core histone H2A/H2B/H3/H4^9-124^E:7.3e-23`PF00808.20^CBFD_NFYB_HMF^Histone-like transcription factor (CBF/NF-Y) and archaeal histone^66-122^E:4.4e-05	0	-1,06	0,032	-0,574	0	1,197	0,018	0,751	0,317	-0,514	H2B_GOSHI	Histone H2B	HIS2B	Gossypium hirsutum (Upland cotton) (Gossypium mexicanum)		Acetylation; Chromosome; DNA-binding; Isopeptide bond; Nucleosome core; Nucleus; Ubl conjugation
SRR648707_TR33512_c0_g2_i1_len=1573	RD19A_ARATH	Q:1420-392,H:27-364	75.51%ID	E:0	Cysteine proteinase RD19a	PF08246.9^Inhibitor_I29^Cathepsin propeptide inhibitor domain (I29)^51-107^E:1.8e-10`PF00112.20^Peptidase_C1^Papain family cysteine protease^138-361^E:5.6e-75	0,152	0,42	0,539	0,219	0	1,091	0,099	0,553	0,173	0,659	RD19A_ARATH	Cysteine proteinase RD19a (RD19) (EC 3.4.22.-)	RD19A At4g39090 F19H22.190	Arabidopsis thaliana (Mouse-ear cress)		Complete proteome; Disulfide bond; Glycoprotein; Hydrolase; Protease; Reference proteome; Signal; Thiol protease; Zymogen
SRR648707_TR35315_c0_g1_i1_len=2024	BADH2_ARATH	Q:175-1683,H:1-503	78.33%ID	E:0	Betaine aldehyde dehydrogenase 2, mitochondrial	PF00171.19^Aldedh^Aldehyde dehydrogenase family^16-485^E:1.9e-172	0,555	0,299	0,003	1,59	0	2,27	0,094	1,26	0,049	1,355	BADH2_ARATH	Betaine aldehyde dehydrogenase 2, mitochondrial (BADH) (EC 1.2.1.8) (Aldehyde dehydrogenase family 10 member A9)	ALDH10A9 At3g48170 T24C20.50	Arabidopsis thaliana (Mouse-ear cress)		Complete proteome; Mitochondrion; NAD; Oxidoreductase; Reference proteome; Transit peptide
SRR648707_TR38345_c0_g1_i1_len=768	NA	NA	NA	NA	NA	.	0,212	0,979	0,031	1,326	0	1,877	0,002	1,719	1	0,095	.	.	.	.	.	.
SRR924147_TR13760_c0_g1_i1_len=953	MTND1_VITVI	Q:836-276,H:1-188	81.38%ID	E:1e-110	1,2-dihydroxy-3-keto-5-methylthiopentene dioxygenase 1 {ECO:0000255|HAMAP-Rule:MF_03154}	PF03079.11^ARD^ARD/ARD' family^44-197^E:1.6e-58`PF02311.16^AraC_binding^AraC-like ligand binding domain^124-196^E:3.5e-07`PF07883.8^Cupin_2^Cupin domain^127-185^E:1.9e-10	0,206	0,706	0,784	-0,148	0	1,017	0,026	0,628	1	-0,296	MTND1_VITVI	1,2-dihydroxy-3-keto-5-methylthiopentene dioxygenase 1 (EC 1.13.11.54) (Acireductone dioxygenase (Fe(2+)-requiring) 1) (ARD 1) (Fe-ARD 1)	VIT_05s0020g04070	Vitis vinifera (Grape)	PATHWAY: Amino-acid biosynthesis; L-methionine biosynthesis via salvage pathway; L-methionine from S-methyl-5-thio-alpha-D-ribose 1-phosphate: step 5/6. {ECO:0000255|HAMAP-Rule:MF_03154}.	Amino-acid biosynthesis; Complete proteome; Cytoplasm; Dioxygenase; Iron; Metal-binding; Methionine biosynthesis; Nucleus; Oxidoreductase; Reference proteome
SRR924147_TR17644_c1_g1_i3_len=824	SAP8_ORYSJ	Q:751-257,H:1-171	61.4%ID	E:1e-67	Zinc finger A20 and AN1 domain-containing stress-associated protein 8	PF01754.13^zf-A20^A20-like zinc finger^16-38^E:5.4e-13`PF01428.13^zf-AN1^AN1-like Zinc finger^106-142^E:1.4e-08	0,017	-5,84	0,022	1,149	0	7,245	0,005	6,264	1	2,295	SAP8_ORYSJ	Zinc finger A20 and AN1 domain-containing stress-associated protein 8 (OsSAP8) (Stress-associated protein 3)	SAP8 SAP3 Os06g0612800 LOC_Os06g41010 OsJ_021110 P0417G12.11 P0429G06.28	Oryza sativa subsp. japonica (Rice)		Complete proteome; Metal-binding; Reference proteome; Stress response; Zinc; Zinc-finger
SRR924147_TR27043_c0_g1_i1_len=771	NA	NA	NA	NA	NA	.	1	0	0,013	-1,701	0	7,767	0,001	6,427	1	1,925	.	.	.	.	.	.
SRR924147_TR27246_c0_g1_i1_len=1043	RRAA3_ARATH	Q:752-255,H:1-166	76.51%ID	E:2e-89	Putative 4-hydroxy-4-methyl-2-oxoglutarate aldolase 3	PF03737.12^Methyltransf_6^Demethylmenaquinone methyltransferase^6-159^E:2.1e-43	1	0,008	0,532	-0,191	0	1,195	0,004	0,938	0,429	-0,232	RRAA3_ARATH	Putative 4-hydroxy-4-methyl-2-oxoglutarate aldolase 3 (HMG aldolase 3) (EC 4.1.3.17) (Oxaloacetate decarboxylase) (OAA decarboxylase) (EC 4.1.1.3) (Regulator of ribonuclease activity homolog 3) (RraA-like protein 3)	At5g56260 K24C1.7	Arabidopsis thaliana (Mouse-ear cress)		Acetylation; Complete proteome; Lyase; Metal-binding; Reference proteome
SRR924147_TR30185_c0_g1_i1_len=1522	TET3_ARATH	Q:1147-293,H:1-285	72.63%ID	E:7e-141	Tetraspanin-3	PF00335.17^Tetraspannin^Tetraspanin family^5-255^E:5.8e-32	0,073	0,702	0,002	1,302	0	1,574	0,019	0,933	0,438	0,343	TET3_ARATH	Tetraspanin-3	TET3 At3g45600 F9K21.18	Arabidopsis thaliana (Mouse-ear cress)		Complete proteome; Glycoprotein; Membrane; Reference proteome; Transmembrane; Transmembrane helix
SRR924147_TR36395_c0_g2_i1_len=1343	NUDT2_ARATH	Q:1052-285,H:19-272	57.42%ID	E:2e-104	Nudix hydrolase 2	PF00293.25^NUDIX^NUDIX domain^109-228^E:3.2e-23	0,783	0,242	0,319	0,825	0	7,782	0,001	6,481	0,77	-0,188	NUDT2_ARATH	Nudix hydrolase 2 (AtNUDT2) (EC 3.6.1.-) (ADP-ribose pyrophosphatase) (EC 3.6.1.13) (NADH pyrophosphatase) (EC 3.6.1.22)	NUDT2 NUDX2 At5g47650 MNJ7.24	Arabidopsis thaliana (Mouse-ear cress)		Complete proteome; Hydrolase; Magnesium; Manganese; Metal-binding; NAD; Reference proteome
SRR924147_TR37526_c0_g2_i1_len=2103	CYSP_PEA	Q:1924-761,H:24-362	67.1%ID	E:0	Cysteine proteinase 15A	PF08246.9^Inhibitor_I29^Cathepsin propeptide inhibitor domain (I29)^84-140^E:2.5e-09`PF00112.20^Peptidase_C1^Papain family cysteine protease^171-312^E:6.7e-46	0,217	0,321	0,009	0,682	0	1,843	0,011	1,316	1	0,181	CYSP_PEA	Cysteine proteinase 15A (EC 3.4.22.-) (Turgor-responsive protein 15A)		Pisum sativum (Garden pea)		Disulfide bond; Glycoprotein; Hydrolase; Protease; Signal; Stress response; Thiol protease; Zymogen
SRR924148_TR490_c0_g2_i1_len=656	NA	NA	NA	NA	NA	.	0,011	0,596	0,835	0,051	0,001	0,801	0,001	0,756	0,57	0,345	.	.	.	.	.	.
SRR924148_TR972_c0_g1_i1_len=717	NA	NA	NA	NA	NA	.	0,312	-0,334	0,612	0,144	0	1,136	0,041	0,602	0,143	-0,375	.	.	.	.	.	.
SRR924148_TR9339_c0_g1_i5_len=1298	RAN3_ARATH	Q:1115-453,H:1-221	98.64%ID	E:3e-143	GTP-binding nuclear protein Ran-3	PF00025.18^Arf^ADP-ribosylation factor family^13-139^E:3.1e-11`PF00071.19^Ras^Ras family^15-171^E:4.5e-50`PF08477.10^Roc^Ras of Complex, Roc, domain of DAPkinase^15-128^E:1.7e-29`PF00910.19^RNA_helicase^RNA helicase^17-139^E:3.4e-05	0,251	-0,289	0,001	0,8	0	1,002	0,006	0,723	0,937	-0,034	RAN3_ARATH	GTP-binding nuclear protein Ran-3 (Ras-related nuclear protein 3)	RAN3 At5g55190 MCO15.14	Arabidopsis thaliana (Mouse-ear cress)		Complete proteome; GTP-binding; Nucleotide-binding; Nucleus; Protein transport; Reference proteome; Transport
SRR924148_TR14151_c0_g1_i2_len=957	HSP12_MEDSA	Q:736-263,H:1-158	86.71%ID	E:4e-75	18.2 kDa class I heat shock protein	PF04969.13^CS^CS domain^52-143^E:0.011`PF00011.18^HSP20^Hsp20/alpha crystallin family^54-157^E:9.3e-33	1	0,297	0,118	1,802	0,001	2,939	0,066	1,982	1	0,366	HSP12_MEDSA	18.2 kDa class I heat shock protein	HSP18.2	Medicago sativa (Alfalfa)		Cytoplasm; Stress response
SRR924148_TR14205_c0_g2_i3_len=1070	SMD2_DROME	Q:851-582,H:23-112	87.78%ID	E:3e-41	Probable small nuclear ribonucleoprotein Sm D2	PF01423.19^LSM^LSM domain^84-159^E:4.1e-19	0,025	-0,612	0,775	-0,089	0	1,726	0,006	1,318	0,022	-0,614	SMD2_DROME	Probable small nuclear ribonucleoprotein Sm D2 (Sm-D2) (snRNP core protein D2)	SmD2 snRNP2 CG1249	Drosophila melanogaster (Fruit fly)		Complete proteome; Nucleus; Reference proteome; Ribonucleoprotein; mRNA processing; mRNA splicing
SRR924148_TR15158_c0_g1_i2_len=530	NA	NA	NA	NA	NA	PF01323.17^DSBA^DSBA-like thioredoxin domain^16-69^E:4.5e-12	1	-0,003	0,034	3,252	0	6,915	0,017	5,731	1	-2,251	.	.	.	.	.	.
SRR924148_TR16062_c2_g1_i5_len=660	NA	NA	NA	NA	NA	.	0,502	-4,265	1	0	0	3,907	1	-3,208	0,034	5,633	.	.	.	.	.	.
SRR924148_TR28419_c0_g3_i2_len=1336	PIP1_SOLLC	Q:203-1048,H:1-282	86.52%ID	E:3e-179	Probable aquaporin PIP-type pTOM75	PF00230.17^MIP^Major intrinsic protein^45-274^E:3.2e-84	0,907	-0,045	0	-1,305	0	1,49	0,001	0,818	0,444	0,317	PIP1_SOLLC	Probable aquaporin PIP-type pTOM75 (Ripening-associated membrane protein) (RAMP)		Solanum lycopersicum (Tomato) (Lycopersicon esculentum)		Cell membrane; Complete proteome; Membrane; Reference proteome; Repeat; Stress response; Transmembrane; Transmembrane helix; Transport
SRR924148_TR28419_c0_g3_i3_len=1425	PIP1_SOLLC	Q:203-1051,H:1-283	86.93%ID	E:7e-180	Probable aquaporin PIP-type pTOM75	PF00230.17^MIP^Major intrinsic protein^45-274^E:3.7e-84	0,272	-0,484	0,274	0,479	0	1,373	0,054	0,706	0,792	-0,064	PIP1_SOLLC	Probable aquaporin PIP-type pTOM75 (Ripening-associated membrane protein) (RAMP)		Solanum lycopersicum (Tomato) (Lycopersicon esculentum)		Cell membrane; Complete proteome; Membrane; Reference proteome; Repeat; Stress response; Transmembrane; Transmembrane helix; Transport
SRR924148_TR29013_c0_g1_i1_len=855	MIRA_SYNDU	Q:221-754,H:34-217	54.84%ID	E:1e-56	Miraculin	PF00197.15^Kunitz_legume^Trypsin and protease inhibitor^31-205^E:4.7e-61	1	-0,04	0,226	0,778	0	4,265	0,073	1,735	0,377	-0,628	MIRA_SYNDU	Miraculin (MIR)		Synsepalum dulcificum (Miracle fruit) (Richadella dulcifica)		Direct protein sequencing; Disulfide bond; Glycoprotein; Signal; Taste-modifying protein
SRR924148_TR31476_c1_g4_i1_len=1224	RL211_ARATH	Q:270-761,H:1-164	84.76%ID	E:9e-98	60S ribosomal protein L21-1	PF01157.15^Ribosomal_L21e^Ribosomal protein L21e^5-98^E:4.7e-44	0,017	-0,643	0,609	-0,145	0,001	1,074	0,083	0,591	0,248	-0,268	RL211_ARATH	60S ribosomal protein L21-1	RPL21A At1g09590 F14J9.25; RPL21C At1g09690 F21M12.8	Arabidopsis thaliana (Mouse-ear cress)		Complete proteome; Reference proteome; Ribonucleoprotein; Ribosomal protein
SRR924148_TR34514_c0_g2_i1_len=1927	C7A29_PANGI	Q:1571-39,H:10-515	60.55%ID	E:0	Cytochrome P450 CYP72A219	PF00067.19^p450^Cytochrome P450^100-515^E:3.2e-89	1	1,864	0,254	4,657	0	6,759	0,066	5,319	1	0,942	C7A29_PANGI	Cytochrome P450 CYP72A219 (EC 1.14.-.-) (Cytochrome P450 CYP72A129)		Panax ginseng (Korean ginseng)		Heme; Iron; Membrane; Metal-binding; Monooxygenase; Oxidoreductase; Transmembrane; Transmembrane helix
SRR924148_TR35468_c0_g2_i1_len=1310	CFIS1_ARATH	Q:279-905,H:13-221	65.55%ID	E:2e-82	Pre-mRNA cleavage factor Im 25 kDa subunit 1 {ECO:0000303|PubMed:18479511}	PF13869.3^NUDIX_2^Nucleotide hydrolase^33-219^E:1.2e-75	1	0,858	0,073	1,995	0	1,314	0,069	0,73	0,258	0,994	CFIS1_ARATH	Pre-mRNA cleavage factor Im 25 kDa subunit 1 (Cleavage and polyadenylation specificity factor 25 kDa subunit) (AtCFI-25) (CFIm-25) (CPSF 25 kDa subunit) (Cleavage and polyadenylation specificity factor subunit 25)	CFIS1 CFIM25 At4g29820 F27B13.60	Arabidopsis thaliana (Mouse-ear cress)		Complete proteome; Metal-binding; Nucleus; RNA-binding; Reference proteome; mRNA processing
SRR924148_TR35599_c0_g2_i1_len=1604	FTSZ1_ARATH	Q:1353-295,H:36-397	87.29%ID	E:5e-171	Cell division protein FtsZ homolog 1, chloroplastic	PF00091.22^Tubulin^Tubulin/FtsZ family, GTPase domain^64-224^E:2.4e-42`PF12327.5^FtsZ_C^FtsZ family, C-terminal domain^273-366^E:6.8e-30	0	-7,253	0	-6,68	0	6,834	1	0	0,37	0,432	FTSZ1_ARATH	Cell division protein FtsZ homolog 1, chloroplastic (AtFtsZ1) (AtFtsZ1-1) (Chloroplast FtsZ) (CpFtsZ) (Protein ACCUMULATION AND REPLICATION OF CHLOROPLASTS 10) (Protein PLASTID MOVEMENT IMPAIRED4)	FTSZ1 ARC10 FTSZ1-1 PMI4 At5g55280 MCO15.23	Arabidopsis thaliana (Mouse-ear cress)		Acetylation; Cell cycle; Chloroplast; Complete proteome; GTP-binding; Membrane; Nucleotide-binding; Plastid; Reference proteome; Thylakoid; Transit peptide

MsDamaged24h_MsIntact24h

Transcript (CDF97)	Uniprot homolog	BLASTX Position	BLASTX %Identity	BLASTX evalue	Uniprot descr	Pfam	PValue_Ms6h	logFC_Ms6h	PValue_Ms8h	logFC_Ms8h	PValue_MsDamaged24h	logFC_MsDamaged24h	PValue_MsIntact24h	logFC_MsIntact24h	PValue_Ms1wk	logFC_Ms1wk	Entry.name	Protein names	Gene names	Organism	Pathway	Keywords
SRR1144633_TR4067_c0_g3_i1_len=2840	BGAL_SOLLC	Q:28-2487,H:16-835	84.27%ID	E:0	Beta-galactosidase	PF01301.16^Glyco_hydro_35^Glycosyl hydrolases family 35^24-329^E:6e-118`PF02449.12^Glyco_hydro_42^Beta-galactosidase^45-193^E:6e-10`PF02140.15^Gal_Lectin^Galactose binding lectin domain^752-828^E:1.3e-20	0,277	0,517	0,214	0,769	0	1,698	0,001	1,541	0,16	-1,171	BGAL_SOLLC	Beta-galactosidase (EC 3.2.1.23) (Acid beta-galactosidase) (Lactase) (Exo-(1-->4)-beta-D-galactanase)		Solanum lycopersicum (Tomato) (Lycopersicon esculentum)		Complete proteome; Direct protein sequencing; Glycosidase; Hydrolase; Reference proteome; Signal
SRR1144633_TR7251_c0_g1_i1_len=1269	CBSX3_ARATH	Q:221-994,H:1-206	64.48%ID	E:4e-109	CBS domain-containing protein CBSX3, mitochondrial	PF00571.25^CBS^CBS domain^34-67^E:0.00063`PF00571.25^CBS^CBS domain^79-134^E:1.9e-14	0,91	0,041	0,001	-1,009	0	1,34	0	1,347	1	0,86	CBSX3_ARATH	CBS domain-containing protein CBSX3, mitochondrial	CBSX3 At5g10860 T30N20.130	Arabidopsis thaliana (Mouse-ear cress)		CBS domain; Complete proteome; Direct protein sequencing; Mitochondrion; Reference proteome; Repeat; Transit peptide
SRR1144633_TR9803_c1_g4_i3_len=1550	MED6_ARATH	Q:526-1203,H:32-298	59.7%ID	E:8e-88	Mediator of RNA polymerase II transcription subunit 6	PF04934.11^Med6^MED6 mediator sub complex component^36-161^E:5.2e-42	0,326	0,559	0,37	-0,316	0	2,735	0	2,836	0,854	0,147	MED6_ARATH	Mediator of RNA polymerase II transcription subunit 6	MED6 MED6_1 At3g21350 MHC9	Arabidopsis thaliana (Mouse-ear cress)		Alternative splicing; Complete proteome; Nucleus; Reference proteome; Transcription; Transcription regulation
SRR1144633_TR20268_c0_g1_i2_len=981	RFS6_ARATH	Q:178-942,H:1-263	73%ID	E:1e-133	Probable galactinol--sucrose galactosyltransferase 6	PF05691.9^Raffinose_syn^Raffinose synthase or seed imbibition protein Sip1^7-264^E:2.6e-107	0,002	2,404	0,002	3,187	0	1,602	0	1,723	0,16	0,481	RFS6_ARATH	Probable galactinol--sucrose galactosyltransferase 6 (EC 2.4.1.82) (Protein DARK INDUCIBLE 10) (Raffinose synthase 6)	RFS6 DIN10 RS6 At5g20250 F5O24.140	Arabidopsis thaliana (Mouse-ear cress)		Alternative splicing; Carbohydrate metabolism; Complete proteome; Glycosyltransferase; Reference proteome; Transferase
SRR1144633_TR29859_c0_g1_i1_len=1115	MTND3_ARATH	Q:113-709,H:1-199	85.43%ID	E:8e-126	1,2-dihydroxy-3-keto-5-methylthiopentene dioxygenase 3 {ECO:0000255|HAMAP-Rule:MF_03154}	PF03079.11^ARD^ARD/ARD' family^14-168^E:1.5e-65`PF02311.16^AraC_binding^AraC-like ligand binding domain^94-152^E:0.00015`PF07883.8^Cupin_2^Cupin domain^97-152^E:1.2e-07	0,183	0,36	0,018	0,617	0	1,205	0	1,175	1	-1,473	MTND3_ARATH	1,2-dihydroxy-3-keto-5-methylthiopentene dioxygenase 3 (EC 1.13.11.54) (Acireductone dioxygenase (Fe(2+)-requiring) 3) (ARD 3) (Fe-ARD 3)	ARD3 At4g14710 dl3395c FCAALL.141	Arabidopsis thaliana (Mouse-ear cress)	PATHWAY: Amino-acid biosynthesis; L-methionine biosynthesis via salvage pathway; L-methionine from S-methyl-5-thio-alpha-D-ribose 1-phosphate: step 5/6. {ECO:0000255|HAMAP-Rule:MF_03154}.	Alternative splicing; Amino-acid biosynthesis; Complete proteome; Cytoplasm; Dioxygenase; Iron; Metal-binding; Methionine biosynthesis; Nucleus; Oxidoreductase; Reference proteome
SRR1144633_TR30186_c0_g1_i1_len=1016	CYPH_CATRO	Q:105-620,H:1-172	99.42%ID	E:8e-111	Peptidyl-prolyl cis-trans isomerase	PF00160.18^Pro_isomerase^Cyclophilin type peptidyl-prolyl cis-trans isomerase/CLD^8-169^E:4.2e-50	0,83	0,046	0,004	0,613	0	1,704	0	1,393	1	-0,053	CYPH_CATRO	Peptidyl-prolyl cis-trans isomerase (PPIase) (EC 5.2.1.8) (Cyclophilin) (Cyclosporin A-binding protein) (Rotamase)	PCKR1	Catharanthus roseus (Madagascar periwinkle) (Vinca rosea)		3D-structure; Cyclosporin; Cytoplasm; Isomerase; Rotamase
SRR1144633_TR33254_c0_g1_i1_len=528	NA	NA	NA	NA	NA	.	0,517	0,751	0,52	0,422	0	2,935	0	2,287	1	-0,649	.	.	.	.	.	.
SRR1144634_TR3610_c0_g1_i1_len=1176	E134_MAIZE	Q:937-371,H:28-219	47.18%ID	E:5e-42	Endo-1,3	PF01738.15^DLH^Dienelactone hydrolase family^32-236^E:3e-29	0,581	0,379	0,082	0,935	0	2,604	0	1,902	1	0	E134_MAIZE	Endo-1,3;1,4-beta-D-glucanase (EC 3.2.1.-)		Zea mays (Maize)		Complete proteome; Direct protein sequencing; Glycoprotein; Hydrolase; Reference proteome; Secreted; Signal
SRR1144634_TR8815_c0_g1_i1_len=724	PIP1_SOLLC	Q:348-719,H:110-233	87.9%ID	E:4e-71	Probable aquaporin PIP-type pTOM75	.	0,744	0,084	0,514	0,167	0	1,518	0	0,859	0,796	0,073	PIP1_SOLLC	Probable aquaporin PIP-type pTOM75 (Ripening-associated membrane protein) (RAMP)		Solanum lycopersicum (Tomato) (Lycopersicon esculentum)		Cell membrane; Complete proteome; Membrane; Reference proteome; Repeat; Stress response; Transmembrane; Transmembrane helix; Transport
SRR1144634_TR9972_c0_g1_i1_len=513	Y3669_ARATH	Q:46-384,H:74-186	64.6%ID	E:2e-50	Putative acyl-coenzyme A oxidase At3g06690	.	0,048	0,83	0,633	-0,18	0	2,209	0	2,312	0,01	-1,376	Y3669_ARATH	Putative acyl-coenzyme A oxidase At3g06690 (Acyl-CoA oxidase) (EC 1.3.3.6)	At3g06690 F3E22.17 T8E24.15	Arabidopsis thaliana (Mouse-ear cress)		Complete proteome; Fatty acid metabolism; Lipid metabolism; Oxidoreductase; Reference proteome
SRR1144634_TR15347_c0_g1_i1_len=770	GPX2_ARATH	Q:1-444,H:20-167	77.7%ID	E:7e-81	Probable glutathione peroxidase 2	PF00255.16^GSHPx^Glutathione peroxidase^2-98^E:5.4e-40`PF00578.18^AhpC-TSA^AhpC/TSA family^2-125^E:1.3e-06	0,064	0,614	0,411	0,319	0	2,367	0,001	1,859	0,555	-0,277	GPX2_ARATH	Probable glutathione peroxidase 2 (EC 1.11.1.9)	GPX2 At2g31570 T9H9.9	Arabidopsis thaliana (Mouse-ear cress)		Complete proteome; Cytoplasm; Nucleus; Oxidoreductase; Peroxidase; Reference proteome; Stress response
SRR1144634_TR16808_c0_g3_i1_len=630	NA	NA	NA	NA	NA	.	0,472	-0,74	0,094	1,363	0	6,923	0	7,173	1	0	.	.	.	.	.	.
SRR1144634_TR27482_c0_g1_i1_len=730	SFR2_ORYSJ	Q:605-6,H:184-384	77.11%ID	E:4e-112	Beta-glucosidase-like SFR2, chloroplastic	PF00232.15^Glyco_hydro_1^Glycosyl hydrolase family 1^2-169^E:7.7e-31	1	-0,051	0,593	0,773	0	2,333	0	2,252	0,381	2,402	SFR2_ORYSJ	Beta-glucosidase-like SFR2, chloroplastic (Beta-glucosidase 36) (Os11bglu36) (Protein SENSITIVE TO FREEZING 2) (OsSFR2)	SFR2 BGLU36 Os11g0683500 LOC_Os11g45710 OsJ_34793	Oryza sativa subsp. japonica (Rice)		Chloroplast; Complete proteome; Glycoprotein; Glycosidase; Hydrolase; Membrane; Plastid; Plastid outer membrane; Reference proteome
SRR1144634_TR33866_c0_g2_i1_len=1767	DNJH_CUCSA	Q:1469-216,H:1-413	90.91%ID	E:0	DnaJ protein homolog	PF00226.28^DnaJ^DnaJ domain^13-71^E:5.7e-23`PF01556.15^DnaJ_C^DnaJ C terminal domain^123-343^E:7.6e-39`PF00684.16^DnaJ_CXXCXGXG^DnaJ central domain^148-214^E:2.6e-15	0,065	0,425	0,038	0,538	0	0,912	0	1,088	1	0,485	DNJH_CUCSA	DnaJ protein homolog (DNAJ-1)	DNAJ1	Cucumis sativus (Cucumber)		Cell membrane; Chaperone; Lipoprotein; Membrane; Metal-binding; Methylation; Prenylation; Repeat; Stress response; Zinc; Zinc-finger
SRR1144634_TR34115_c0_g1_i1_len=1773	DES_CAPAN	Q:1562-147,H:6-476	52.74%ID	E:7e-179	9-divinyl ether synthase	PF00067.19^p450^Cytochrome P450^324-435^E:2.7e-19	0,001	1,21	0,34	0,504	0	2,143	0	2,242	0,029	1,234	DES_CAPAN	9-divinyl ether synthase (StDES) (EC 4.2.1.121) (Colneleate synthase) (Cytochrome P450 74D4)	DES CYP74D4	Capsicum annuum (Bell pepper)		Heme; Iron; Lyase; Metal-binding
SRR1144634_TR37899_c0_g1_i1_len=1388	H12_ARATH	Q:342-554,H:62-132	80.28%ID	E:2e-21	Histone H1.2	PF00538.16^Linker_histone^linker histone H1 and H5 family^61-127^E:1.7e-18	0,55	-0,148	0,208	-0,314	0	1,049	0	1,152	0,426	-0,196	H12_ARATH	Histone H1.2	At2g30620 T06B20.3	Arabidopsis thaliana (Mouse-ear cress)		Acetylation; Alternative splicing; Chromosome; Complete proteome; DNA-binding; Isopeptide bond; Nucleus; Phosphoprotein; Reference proteome; Ubl conjugation
SRR1144634_TR38651_c0_g1_i1_len=1540	NA	NA	NA	NA	NA	.	0,274	0,396	0,003	1,078	0	1,241	0	1,086	1	-0,323	.	.	.	.	.	.
SRR1144634_TR40502_c0_g1_i1_len=2682	RH7_SPIOL	Q:1169-2236,H:285-640	70.22%ID	E:2e-164	DEAD-box ATP-dependent RNA helicase 7	PF00271.28^Helicase_C^Helicase conserved C-terminal domain^53-151^E:7.5e-27`PF08152.9^GUCT^GUCT (NUC152) domain^240-335^E:3e-29	0,484	-0,359	1	0,05	0,001	2,23	0	2,545	0,009	-0,979	RH7_SPIOL	DEAD-box ATP-dependent RNA helicase 7 (EC 3.6.4.13)	RH7 PRH75	Spinacia oleracea (Spinach)		ATP-binding; Helicase; Hydrolase; Nucleotide-binding; Nucleus; RNA-binding
SRR122238_TR1559_c1_g1_i2_len=922	IF5A3_SOLTU	Q:84-554,H:1-157	92.99%ID	E:1e-102	Eukaryotic translation initiation factor 5A-3	PF01287.17^eIF-5a^Eukaryotic elongation factor 5A hypusine, DNA-binding OB fold^85-154^E:1.3e-27	0,041	0,565	0,006	0,747	0	1,876	0	1,941	0,835	0,06	IF5A3_SOLTU	Eukaryotic translation initiation factor 5A-3 (eIF-5A-3) (eIF-4D)	EIF5A3	Solanum tuberosum (Potato)		Complete proteome; Hypusine; Initiation factor; Protein biosynthesis; Reference proteome
SRR122238_TR1890_c0_g1_i2_len=1608	DV4H_CATRO	Q:71-1270,H:2-401	100%ID	E:0	Deacetoxyvindoline 4-hydroxylase	PF14226.3^DIOX_N^non-haem dioxygenase in morphine synthesis N-terminal^66-172^E:1.8e-18`PF03171.17^2OG-FeII_Oxy^2OG-Fe(II) oxygenase superfamily^224-314^E:9.5e-23	0,376	0,197	0,102	0,378	0	1,197	0	0,821	0,003	0,652	DV4H_CATRO	Deacetoxyvindoline 4-hydroxylase (EC 1.14.11.20)	D4H	Catharanthus roseus (Madagascar periwinkle) (Vinca rosea)	PATHWAY: Alkaloid biosynthesis; vindoline biosynthesis; vindoline from tabersonine: step 5/6.	Cytoplasm; Dioxygenase; Direct protein sequencing; Iron; Metal-binding; Oxidoreductase; Vitamin C
SRR122238_TR3746_c0_g1_i3_len=1800	PT504_ARATH	Q:1513-1079,H:6-150	84.83%ID	E:9e-80	Probable sugar phosphate/phosphate translocator At5g04160	PF03151.13^TPT^Triose-phosphate Transporter family^20-160^E:2e-22	1	0,197	0,381	2,487	0,001	4,18	0	5,439	1	-0,414	PT504_ARATH	Probable sugar phosphate/phosphate translocator At5g04160	At5g04160 F21E1.80	Arabidopsis thaliana (Mouse-ear cress)		Complete proteome; Membrane; Pyruvate; Reference proteome; Sugar transport; Transmembrane; Transmembrane helix; Transport
SRR122238_TR4783_c0_g1_i4_len=1296	RTNLH_ARATH	Q:311-1027,H:1-247	56.97%ID	E:2e-90	Reticulon-like protein B8	PF02453.14^Reticulon^Reticulon^157-311^E:2.6e-50	0,572	0,191	0,005	0,918	0	1,024	0	1,048	1	0,013	RTNLH_ARATH	Reticulon-like protein B8 (AtRTNLB8)	RTNLB8 At3g10260 F14P13.14	Arabidopsis thaliana (Mouse-ear cress)		Alternative splicing; Complete proteome; Endoplasmic reticulum; Membrane; Reference proteome; Transmembrane; Transmembrane helix
SRR122238_TR5220_c0_g1_i7_len=1631	NA	NA	NA	NA	NA	PF05184.12^SapB_1^Saposin-like type B, region 1^61-96^E:1.9e-11`PF03489.14^SapB_2^Saposin-like type B, region 2^101-133^E:4.8e-05`PF05184.12^SapB_1^Saposin-like type B, region 1^148-181^E:1.1e-08`PF03489.14^SapB_2^Saposin-like type B, region 2^188-220^E:3.1e-08	0,13	0,558	0,139	0,496	0	1,43	0	1,221	0,87	0,061	.	.	.	.	.	.
SRR122238_TR5755_c0_g1_i3_len=1677	OXR1_XENTR	Q:836-327,H:702-869	38.6%ID	E:3e-24	Oxidation resistance protein 1	PF07534.13^TLD^TLD^208-345^E:8.8e-31	0,833	0,13	1	0,065	0,001	2,226	0	2,931	0,195	-0,957	OXR1_XENTR	Oxidation resistance protein 1	oxr1	Xenopus tropicalis (Western clawed frog) (Silurana tropicalis)		Alternative splicing; Complete proteome; Mitochondrion; Reference proteome; Stress response
SRR122238_TR18192_c0_g1_i3_len=601	NA	NA	NA	NA	NA	.	0,504	-0,153	0,871	-0,04	0	0,924	0	0,914	0,917	-0,072	.	.	.	.	.	.
SRR122238_TR20385_c0_g1_i1_len=753	NA	NA	NA	NA	NA	.	0,828	0,06	0,021	0,553	0	1,505	0	1,192	0,174	-0,338	.	.	.	.	.	.
SRR122238_TR20757_c0_g1_i4_len=983	NA	NA	NA	NA	NA	PF11460.5^DUF3007^Protein of unknown function (DUF3007)^102-197^E:3.9e-34	0,91	-0,031	0,027	-0,528	0	1,484	0	1,747	0,56	0,144	.	.	.	.	.	.
SRR122238_TR20797_c0_g1_i1_len=1690	XTH28_ARATH	Q:355-1209,H:24-308	69.12%ID	E:2e-150	Probable xyloglucan endotransglucosylase/hydrolase protein 28	PF00722.18^Glyco_hydro_16^Glycosyl hydrolases family 16^33-214^E:1.9e-55`PF06955.9^XET_C^Xyloglucan endo-transglycosylase (XET) C-terminus^254-289^E:2.3e-13	0,165	0,716	0,022	1,141	0	1,154	0	1,183	0,882	0,174	XTH28_ARATH	Probable xyloglucan endotransglucosylase/hydrolase protein 28 (At-XTH28) (XTH-28) (EC 2.4.1.207)	XTH28 EXGT-A2 XTR2 At1g14720 F10B6.12 F10B6.29	Arabidopsis thaliana (Mouse-ear cress)		Apoplast; Cell wall; Cell wall biogenesis/degradation; Complete proteome; Disulfide bond; Glycoprotein; Glycosidase; Hydrolase; Reference proteome; Secreted; Signal; Transferase
SRR122238_TR22189_c0_g1_i1_len=994	HS157_ARATH	Q:248-682,H:3-137	60.27%ID	E:2e-45	15.7 kDa heat shock protein, peroxisomal	PF00011.18^HSP20^Hsp20/alpha crystallin family^30-141^E:4.8e-22`PF04969.13^CS^CS domain^30-126^E:0.0036	0,454	-0,372	0,154	-0,827	0	2,444	0,001	1,832	0,698	0,235	HS157_ARATH	15.7 kDa heat shock protein, peroxisomal (AtHsp15.7)	HSP15.7 At5g37670 K12B20.120	Arabidopsis thaliana (Mouse-ear cress)		Complete proteome; Peroxisome; Reference proteome; Stress response
SRR122238_TR23422_c0_g1_i1_len=2433	CCX1_ARATH	Q:229-1728,H:51-569	51.24%ID	E:2e-141	Cation/calcium exchanger 1	PF01699.21^Na_Ca_ex^Sodium/calcium exchanger protein^106-249^E:3.7e-26`PF01699.21^Na_Ca_ex^Sodium/calcium exchanger protein^387-541^E:3e-25	1	0,064	0,643	0,456	0	3,952	0	3,567	0,472	-0,664	CCX1_ARATH	Cation/calcium exchanger 1 (AtCCX1) (Protein CATION EXCHANGER 7)	CCX1 CAX7 At5g17860 MPI7.2 MVA3_210	Arabidopsis thaliana (Mouse-ear cress)		Antiport; Complete proteome; Ion transport; Membrane; Potassium; Potassium transport; Reference proteome; Sodium; Sodium transport; Transmembrane; Transmembrane helix; Transport; Vacuole
SRR122238_TR25933_c0_g1_i3_len=5610	MNS3_ARATH	Q:310-2166,H:1-624	68.68%ID	E:0	Mannosyl-oligosaccharide 1,2-alpha-mannosidase MNS3	PF00520.28^Ion_trans^Ion transport protein^60-307^E:7.7e-37`PF07885.13^Ion_trans_2^Ion channel^208-300^E:2e-11`PF00027.26^cNMP_binding^Cyclic nucleotide-binding domain^398-482^E:8.7e-15`PF11834.5^KHA^KHA, dimerisation domain of potassium ion channel^698-739^E:3.5e-07	0,09	0,983	0,031	1,416	0	1,593	0	2,142	0,226	2,23	MNS3_ARATH	Mannosyl-oligosaccharide 1,2-alpha-mannosidase MNS3 (EC 3.2.1.113)	MNS3 At1g30000 T1P2.10	Arabidopsis thaliana (Mouse-ear cress)	PATHWAY: Protein modification; protein glycosylation.	Calcium; Complete proteome; Disulfide bond; Glycoprotein; Golgi apparatus; Hydrolase; Magnesium; Manganese; Membrane; Reference proteome; Signal-anchor; Transmembrane; Transmembrane helix
SRR122238_TR26284_c0_g1_i2_len=1075	ATG8F_ARATH	Q:730-380,H:1-117	92.31%ID	E:9e-60	Autophagy-related protein 8f	PF02991.13^Atg8^Autophagy protein Atg8 ubiquitin like^14-117^E:5.1e-51`PF04110.10^APG12^Ubiquitin-like autophagy protein Apg12^40-117^E:3.6e-07	0,568	0,188	0,84	-0,077	0	1,674	0	1,791	1	-0,026	ATG8F_ARATH	Autophagy-related protein 8f (Autophagy-related ubiquitin-like modifier ATG8f) (AtAPG8f) (Protein autophagy 8f)	ATG8F APG8F At4g16520 dl4285c FCAALL.383	Arabidopsis thaliana (Mouse-ear cress)		Autophagy; Complete proteome; Cytoplasm; Cytoplasmic vesicle; Cytoskeleton; Lipoprotein; Membrane; Microtubule; Protein transport; Reference proteome; Transport; Ubl conjugation pathway; Vacuole
SRR122238_TR27743_c0_g1_i1_len=1029	HS21C_SOLLC	Q:629-123,H:87-235	43.79%ID	E:2e-38	Small heat shock protein, chloroplastic	PF00011.18^HSP20^Hsp20/alpha crystallin family^144-247^E:2.9e-22	1	0,74	1	0,479	0	4,649	0	4,64	0,356	1,059	HS21C_SOLLC	Small heat shock protein, chloroplastic	HSP21 TOM111	Solanum lycopersicum (Tomato) (Lycopersicon esculentum)		Chloroplast; Complete proteome; Plastid; Reference proteome; Stress response; Transit peptide
SRR122238_TR29842_c1_g6_i2_len=761	KIRO_ACTDE	Q:79-522,H:1-148	47.3%ID	E:2e-35	Kirola {ECO:0000303|PubMed:21309790}	PF00407.16^Bet_v_1^Pathogenesis-related protein Bet v I family^30-173^E:7.6e-33	0,11	0,337	0,552	-0,129	0	1,347	0	0,951	0,016	0,503	KIRO_ACTDE	Kirola (allergen Act d 11)		Actinidia deliciosa (Kiwi)		3D-structure; Acetylation; Allergen; Direct protein sequencing; Polymorphism
SRR122238_TR31182_c0_g1_i1_len=737	CH10_ARATH	Q:516-226,H:1-97	78.35%ID	E:7e-49	10 kDa chaperonin	.	0	-1,134	0,964	-0,027	0	2,643	0	1,849	0,692	-0,806	CH10_ARATH	10 kDa chaperonin (Chaperonin 10) (CPN10) (Protein groES)	CPN10 At1g14980 T15D22.2	Arabidopsis thaliana (Mouse-ear cress)		Chaperone; Complete proteome; Cytoplasm; Reference proteome
SRR122238_TR31835_c0_g1_i2_len=524	PLPD1_ARATH	Q:2-265,H:478-565	85.23%ID	E:1e-42	Dihydrolipoyl dehydrogenase 1, chloroplastic	.	0,86	0,231	0,048	-0,702	0	1,726	0	2,342	0,014	-0,813	PLPD1_ARATH	Dihydrolipoyl dehydrogenase 1, chloroplastic (ptLPD1) (EC 1.8.1.4) (Dihydrolipoamide dehydrogenase 1) (Protein LIPOAMIDE DEHYDROGENASE 1) (Pyruvate dehydrogenase complex E3 subunit 1) (E3-1) (PDC-E3 1)	LPD1 At3g16950 K14A17.6	Arabidopsis thaliana (Mouse-ear cress)		Alternative splicing; Chloroplast; Complete proteome; Disulfide bond; FAD; Flavoprotein; NAD; Oxidoreductase; Plastid; Redox-active center; Reference proteome; Transit peptide
SRR122238_TR32080_c0_g1_i3_len=1351	CXE6_ARATH	Q:140-1087,H:23-329	54.23%ID	E:1e-116	Probable carboxylesterase 6	PF00135.25^COesterase^Carboxylesterase family^77-138^E:1.5e-08`PF10340.6^DUF2424^Protein of unknown function (DUF2424)^77-235^E:9.8e-07`PF12740.4^Chlorophyllase2^Chlorophyllase enzyme^86-231^E:1.5e-07`PF07859.10^Abhydrolase_3^alpha/beta hydrolase fold^97-323^E:2.6e-58	1	1,03	0,066	4,383	0	7,245	0	8,549	1	-1,14	CXE6_ARATH	Probable carboxylesterase 6 (AtCXE6) (EC 3.1.1.1)	CXE6 At1g68620 F24J5.14	Arabidopsis thaliana (Mouse-ear cress)		Complete proteome; Hydrolase; Reference proteome; Serine esterase
SRR122238_TR36414_c0_g1_i1_len=1467	PTR8_ARATH	Q:3-1229,H:163-568	55.96%ID	E:4e-155	Protein NRT1/ PTR FAMILY 2.9	PF00854.18^PTR2^POT family^1-369^E:9.6e-69	1	0,616	0,008	3,995	0	4,775	0	3,579	1	0,977	PTR8_ARATH	Protein NRT1/ PTR FAMILY 2.9 (AtNPF2.9) (Nitrate transporter 1.9)	NPF2.9 NRT1.9 At1g18880 F6A14.2	Arabidopsis thaliana (Mouse-ear cress)		Cell membrane; Complete proteome; Membrane; Reference proteome; Transmembrane; Transmembrane helix; Transport
SRR122238_TR36974_c1_g1_i1_len=1014	NA	NA	NA	NA	NA	.	0,044	1,042	0,077	0,826	0	3,099	0	3,221	1	0,21	.	.	.	.	.	.
SRR122238_TR36974_c1_g1_i2_len=1162	NA	NA	NA	NA	NA	.	0,017	5,839	0,798	0,338	0	7,096	0,001	6,596	1	-2,43	.	.	.	.	.	.
SRR122238_TR37291_c0_g1_i1_len=628	PH1_ARATH	Q:590-165,H:1-142	77.46%ID	E:1e-76	Pleckstrin homology domain-containing protein 1	PF00169.26^PH^PH domain^40-137^E:3.3e-22	0,58	0,291	0,288	0,496	0	1,947	0	1,528	1	-0,087	PH1_ARATH	Pleckstrin homology domain-containing protein 1 (AtPH1)	PH1 At2g29700 T27A16.20	Arabidopsis thaliana (Mouse-ear cress)		Complete proteome; Cytoplasm; Lipid-binding; Reference proteome
SRR122238_TR37377_c0_g2_i1_len=1477	Y4967_ARATH	Q:1301-231,H:4-359	59.66%ID	E:3e-157	Uncharacterized oxidoreductase At4g09670	PF01408.19^GFO_IDH_MocA^Oxidoreductase family, NAD-binding Rossmann fold^6-125^E:1.1e-18	0,893	-0,056	0,727	0,12	0	2,137	0	2,309	0,308	0,504	Y4967_ARATH	Uncharacterized oxidoreductase At4g09670 (EC 1.-.-.-)	At4g09670 F17A8.20	Arabidopsis thaliana (Mouse-ear cress)		3D-structure; Acetylation; Complete proteome; Oxidoreductase; Reference proteome
SRR122238_TR40444_c0_g1_i5_len=1134	ACFR1_ARATH	Q:288-785,H:65-238	64.94%ID	E:1e-65	Transmembrane ascorbate ferrireductase 1	PF03188.13^Cytochrom_B561^Eukaryotic cytochrome b561^2-115^E:1.1e-37	0,56	0,646	0,132	-1,395	0	2,074	0	2,119	0,278	-0,803	ACFR1_ARATH	Transmembrane ascorbate ferrireductase 1 (EC 1.16.5.1) (Cytochrome b561) (Artb561-1) (AtCytb561) (Protein b561A.tha5) (Tonoplast Cyt-b561) (TCytb)	CYB561A ACYB-2 CYB561B1 CYBASC1 At4g25570 M7J2.60	Arabidopsis thaliana (Mouse-ear cress)		Complete proteome; Electron transport; Heme; Iron; Membrane; Metal-binding; Oxidoreductase; Reference proteome; Transmembrane; Transmembrane helix; Transport; Vacuole
SRR122238_TR44116_c0_g1_i1_len=653	MIFH_BRUMA	Q:582-238,H:1-114	46.09%ID	E:3e-25	Macrophage migration inhibitory factor homolog	PF01187.15^MIF^Macrophage migration inhibitory factor (MIF)^25-138^E:3.1e-25	0,84	-0,074	0	-1,331	0	1,311	0	1,484	0,517	-0,783	MIFH_BRUMA	Macrophage migration inhibitory factor homolog (Bm-MIF-1) (BmMIF) (MIF) (EC 5.3.2.1) (L-dopachrome isomerase) (L-dopachrome tautomerase) (EC 5.3.3.12) (Phenylpyruvate tautomerase)	Bm1_28435 BMBAC01P19.1	Brugia malayi (Filarial nematode worm)		3D-structure; Complete proteome; Cytokine; Isomerase; Secreted
SRR122238_TR45476_c0_g1_i4_len=1633	NA	NA	NA	NA	NA	PF07207.8^Lir1^Light regulated protein Lir1^27-162^E:1.6e-41	0,117	0,352	0	-1,119	0	3,286	0	2,535	0,702	0,114	.	.	.	.	.	.
SRR122238_TR45975_c1_g1_i1_len=3425	LOX31_SOLTU	Q:619-3108,H:114-914	80.63%ID	E:0	Linoleate 13S-lipoxygenase 3-1, chloroplastic	PF00305.16^Lipoxygenase^Lipoxygenase^1-303^E:3.3e-145	1	2,426	0,692	1,419	0	8,23	0	7,641	1	-3,613	LOX31_SOLTU	Linoleate 13S-lipoxygenase 3-1, chloroplastic (EC 1.13.11.12)	LOX3.1 LOX-H3	Solanum tuberosum (Potato)	PATHWAY: Lipid metabolism; oxylipin biosynthesis. {ECO:0000255|PROSITE-ProRule:PRU00726}.	Chloroplast; Complete proteome; Dioxygenase; Fatty acid biosynthesis; Fatty acid metabolism; Iron; Lipid biosynthesis; Lipid metabolism; Metal-binding; Oxidoreductase; Oxylipin biosynthesis; Plastid; Reference proteome; Thylakoid; Transit peptide
SRR122238_TR46684_c3_g1_i1_len=1388	NA	NA	NA	NA	NA	PF04434.14^SWIM^SWIM zinc finger^2-26^E:0.00073	1	0,098	0,517	-0,73	0	2,963	0	3,667	1	-0,176	.	.	.	.	.	.
SRR122238_TR47542_c0_g1_i2_len=679	UBL5_ARATH	Q:533-315,H:1-73	98.63%ID	E:3e-43	Ubiquitin-like protein 5	.	0,08	-0,476	0,461	-0,196	0	1,259	0	0,987	0,798	-0,385	UBL5_ARATH	Ubiquitin-like protein 5	UBL5 At5g42300 K5J14.10	Arabidopsis thaliana (Mouse-ear cress)		Complete proteome; Cytoplasm; Reference proteome; Ubl conjugation pathway
SRR122238_TR50041_c1_g2_i7_len=4327	XLG1_ARATH	Q:3619-986,H:11-871	48%ID	E:0	Extra-large guanine nucleotide-binding protein 1	PF00503.17^G-alpha^G-protein alpha subunit^509-899^E:2.2e-60	0,56	0,973	0,118	1,765	0	3,438	0	3,789	1	0,216	XLG1_ARATH	Extra-large guanine nucleotide-binding protein 1 (Extra-large GTP-binding protein 1) (Extra-large G-protein 1)	XLG1 XLG At2g23460 F26B6.11	Arabidopsis thaliana (Mouse-ear cress)		Calcium; Complete proteome; GTP-binding; Metal-binding; Nucleotide-binding; Nucleus; Reference proteome; TonB box; Transducer; Zinc; Zinc-finger
SRR122238_TR54205_c1_g1_i3_len=852	NA	NA	NA	NA	NA	PF00505.16^HMG_box^HMG (high mobility group) box^2-54^E:1.9e-13`PF09011.7^HMG_box_2^HMG-box domain^3-54^E:3.4e-07	0,049	0,556	0,665	-0,13	0	1,781	0	1,551	0,085	0,621	.	.	.	.	.	.
SRR122238_TR57053_c2_g2_i2_len=721	MLP31_ARATH	Q:647-195,H:18-171	37.66%ID	E:2e-29	MLP-like protein 31	PF00407.16^Bet_v_1^Pathogenesis-related protein Bet v I family^6-150^E:5.4e-41	0,545	0,566	0,004	1,056	0,001	1,218	0	1,301	1	-0,092	MLP31_ARATH	MLP-like protein 31	MLP31 At1g70840 F15H11.9	Arabidopsis thaliana (Mouse-ear cress)		Complete proteome; Reference proteome
SRR122238_TR59858_c0_g1_i1_len=1263	PER4_VITVI	Q:89-1024,H:12-321	72.76%ID	E:1e-161	Peroxidase 4 {ECO:0000250|UniProtKB:Q42578}	PF00141.20^peroxidase^Peroxidase^41-283^E:1.2e-80	0,593	0,734	0,629	2,11	0	4,283	0	2,617	1	0,521	PER4_VITVI	Peroxidase 4 (EC 1.11.1.7)	GSVIVT00023967001 LOC100257005	Vitis vinifera (Grape)		Calcium; Direct protein sequencing; Disulfide bond; Glycoprotein; Heme; Hydrogen peroxide; Iron; Metal-binding; Oxidoreductase; Peroxidase; Pyrrolidone carboxylic acid; Secreted; Signal
SRR122238_TR62903_c0_g2_i5_len=672	NA	NA	NA	NA	NA	PF07172.8^GRP^Glycine rich protein family^1-86^E:9.9e-11	0,68	-0,139	0,251	-0,38	0	4,999	0	3,928	0,059	-1,208	.	.	.	.	.	.
SRR122238_TR68717_c0_g1_i1_len=623	MPC4_ARATH	Q:98-421,H:1-108	81.48%ID	E:4e-60	Mitochondrial pyruvate carrier 4 {ECO:0000305}	PF03650.10^MPC^Uncharacterised protein family (UPF0041)^6-104^E:4.4e-41	0,615	0,165	0,228	0,358	0	1,313	0,001	1,141	0,384	-0,257	MPC4_ARATH	Mitochondrial pyruvate carrier 4	MPC4 At4g22310 T10I14_140	Arabidopsis thaliana (Mouse-ear cress)		Complete proteome; Membrane; Mitochondrion; Mitochondrion inner membrane; Reference proteome; Transmembrane; Transmembrane helix; Transport
SRR122238_TR68965_c0_g1_i2_len=808	H33_VITVI	Q:80-487,H:1-136	100%ID	E:1e-91	Histone H3.3	.	0,87	-0,165	0,679	-0,308	0	3,729	0	4,057	0,921	0,088	H33_VITVI	Histone H3.3		Vitis vinifera (Grape)		Acetylation; Chromosome; DNA-binding; Methylation; Nucleosome core; Nucleus; Phosphoprotein
SRR122238_TR71775_c0_g1_i1_len=856	ERF99_ARATH	Q:325-606,H:74-169	66.67%ID	E:2e-29	Ethylene-responsive transcription factor 13	PF00847.17^AP2^AP2 domain^9-149^E:1.7e-14	0,502	2,504	0,017	5,773	0	6,199	0	6,005	0,066	-3,41	ERF99_ARATH	Ethylene-responsive transcription factor 13 (AtERF13) (Ethylene-responsive element-binding factor 13) (EREBP-13)	ERF13 ERF-13 ERF099 At2g44840 T13E15.15	Arabidopsis thaliana (Mouse-ear cress)		Activator; Complete proteome; DNA-binding; Ethylene signaling pathway; Nucleus; Plant defense; Reference proteome; Transcription; Transcription regulation
SRR122238_TR74308_c0_g1_i1_len=1721	ATL60_ARATH	Q:752-1309,H:95-300	44.76%ID	E:2e-41	RING-H2 finger protein ATL60	PF13639.3^zf-RING_2^Ring finger domain^116-159^E:1.2e-14`PF17123.2^zf-RING_11^RING-like zinc finger^116-145^E:3.9e-11`PF13923.3^zf-C3HC4_2^Zinc finger, C3HC4 type (RING finger)^116-158^E:8.9e-09`PF00097.22^zf-C3HC4^Zinc finger, C3HC4 type (RING finger)^117-158^E:5.4e-06`PF13445.3^zf-RING_UBOX^RING-type zinc-finger^117-156^E:0.00013`PF12678.4^zf-rbx1^RING-H2 zinc finger domain^125-159^E:8e-08`PF12861.4^zf-ANAPC11^Anaphase-promoting complex subunit 11 RING-H2 finger^125-162^E:6e-05	0,629	0,995	0,692	1,213	0	4,895	0	5,28	0,629	0,758	ATL60_ARATH	RING-H2 finger protein ATL60	ATL60 At1g53820 T18A20.16	Arabidopsis thaliana (Mouse-ear cress)	PATHWAY: Protein modification; protein ubiquitination.	Complete proteome; Membrane; Metal-binding; Reference proteome; Transmembrane; Transmembrane helix; Ubl conjugation pathway; Zinc; Zinc-finger
SRR122238_TR75852_c1_g2_i1_len=1099	NA	NA	NA	NA	NA	PF00407.16^Bet_v_1^Pathogenesis-related protein Bet v I family^3-166^E:1.2e-14	0,298	0,29	1	0,001	0	0,976	0	0,89	0,882	-0,183	.	.	.	.	.	.
SRR122238_TR81300_c0_g1_i1_len=996	ATL80_ARATH	Q:773-249,H:23-197	58.15%ID	E:6e-44	RING-H2 finger protein ATL80	PF13639.3^zf-RING_2^Ring finger domain^111-154^E:7.3e-15`PF17123.2^zf-RING_11^RING-like zinc finger^111-140^E:1.5e-11`PF13923.3^zf-C3HC4_2^Zinc finger, C3HC4 type (RING finger)^112-153^E:2.2e-10`PF00097.22^zf-C3HC4^Zinc finger, C3HC4 type (RING finger)^112-153^E:2.1e-07`PF13445.3^zf-RING_UBOX^RING-type zinc-finger^112-151^E:0.00013`PF12678.4^zf-rbx1^RING-H2 zinc finger domain^116-154^E:2.2e-08`PF12861.4^zf-ANAPC11^Anaphase-promoting complex subunit 11 RING-H2 finger^120-160^E:4.8e-07	0,505	0,286	0,19	0,442	0	1,557	0	1,842	0,712	0,23	ATL80_ARATH	RING-H2 finger protein ATL80	ATL80 At1g20823 F2D10.34	Arabidopsis thaliana (Mouse-ear cress)	PATHWAY: Protein modification; protein ubiquitination.	Complete proteome; Membrane; Metal-binding; Plant defense; Reference proteome; Transmembrane; Transmembrane helix; Ubl conjugation pathway; Zinc; Zinc-finger
SRR1271857_TR2460_c0_g1_i1_len=1056	NA	NA	NA	NA	NA	.	0,381	1,332	0,692	1,148	0	3,672	0	3,828	0,629	1,089	.	.	.	.	.	.
SRR1271857_TR3142_c0_g1_i12_len=2328	PIP1_SOLLC	Q:2038-1409,H:1-210	87.62%ID	E:1e-126	Probable aquaporin PIP-type pTOM75	PF00230.17^MIP^Major intrinsic protein^45-218^E:2.9e-57	0,557	0,158	0,007	0,688	0	1,468	0,001	0,81	0,066	3,501	PIP1_SOLLC	Probable aquaporin PIP-type pTOM75 (Ripening-associated membrane protein) (RAMP)		Solanum lycopersicum (Tomato) (Lycopersicon esculentum)		Cell membrane; Complete proteome; Membrane; Reference proteome; Repeat; Stress response; Transmembrane; Transmembrane helix; Transport
SRR1271857_TR11665_c0_g2_i1_len=604	NA	NA	NA	NA	NA	.	0,5	-0,732	0,466	0,233	0	2,192	0	2,112	0,01	-0,663	.	.	.	.	.	.
SRR1271857_TR17375_c0_g2_i1_len=2181	RCA1_LARTR	Q:241-1650,H:1-475	80.08%ID	E:0	Ribulose bisphosphate carboxylase/oxygenase activase 1, chloroplastic	PF00004.26^AAA^ATPase family associated with various cellular activities (AAA)^160-301^E:3.2e-15	1	-0,003	0,025	-2,656	0	1,334	0	1,275	0,694	0,348	RCA1_LARTR	Ribulose bisphosphate carboxylase/oxygenase activase 1, chloroplastic (RA 1) (RuBisCO activase 1) (RuBisCO activase alpha form)	RCA1	Larrea tridentata (Creosote bush) (Zygophyllum tridentatum)		3D-structure; ATP-binding; Chloroplast; Nucleotide-binding; Plastid; Transit peptide

SRR1271857_TR20249_c0_g1_i1_len=812	NA	NA	NA	NA	NA	.	0,895	-0,101	0,008	-1,238	0	1,386	0	1,288	1	0,858	.	.	.	.	.	.
SRR1271857_TR26300_c2_g2_i2_len=688	NA	NA	NA	NA	NA	PF14878.3^DLD^Death-like domain of SPT6^3-67^E:7.2e-13`PF14633.3^SH2_2^SH2 domain^161-208^E:1.4e-07	1	0,007	0,109	1,038	0	6,678	0	6,86	0,517	0,879	.	.	.	.	.	.
SRR1271857_TR31278_c0_g1_i3_len=1262	RAG3F_ARATH	Q:270-887,H:1-206	92.72%ID	E:4e-139	Ras-related protein RABG3f	PF00025.18^Arf^ADP-ribosylation factor family^9-175^E:3.5e-14`PF00071.19^Ras^Ras family^10-176^E:4.8e-56`PF08477.10^Roc^Ras of Complex, Roc, domain of DAPkinase^10-128^E:4.7e-33`PF01926.20^MMR_HSR1^50S ribosome-binding GTPase^10-109^E:2.1e-07`PF04670.9^Gtr1_RagA^Gtr1/RagA G protein conserved region^10-130^E:4.9e-06	0,325	0,266	0,284	0,314	0	1,251	0	1,22	0,874	0,056	RAG3F_ARATH	Ras-related protein RABG3f (AtRABG3f) (Ras-related protein Rab71) (AtRab71) (Ras-related protein Rab7B) (AtRab7B)	RABG3F RAB71 RAB7B At3g18820 MVE11.21	Arabidopsis thaliana (Mouse-ear cress)		Cell membrane; Complete proteome; GTP-binding; Lipoprotein; Membrane; Methylation; Nucleotide-binding; Prenylation; Protein transport; Reference proteome; Transport
SRR1271857_TR33931_c0_g1_i11_len=2240	NA	NA	NA	NA	NA	PF00892.17^EamA^EamA-like transporter family^55-109^E:3.8e-09	0,059	1,618	0,187	0,996	0	2,82	0	2,55	0,593	0,731	.	.	.	.	.	.
SRR1271857_TR33931_c0_g1_i13_len=1989	NA	NA	NA	NA	NA	PF00892.17^EamA^EamA-like transporter family^4-120^E:3.5e-08`PF00892.17^EamA^EamA-like transporter family^130-279^E:1.1e-13	0,065	1,281	0,027	1,354	0	3,086	0	3,219	0,882	-0,188	.	.	.	.	.	.
SRR1271857_TR33994_c0_g1_i1_len=1179	OP24B_ARATH	Q:1002-367,H:3-213	67.92%ID	E:1e-100	Outer envelope pore protein 24B, chloroplastic	.	0,18	0,792	0,001	1,417	0	3,067	0	3,394	1	0,318	OP24B_ARATH	Outer envelope pore protein 24B, chloroplastic (Chloroplastic outer envelope pore protein of 24 kDa B)	OEP24B At5g42960 MBD2.16	Arabidopsis thaliana (Mouse-ear cress)		Chloroplast; Complete proteome; Ion transport; Membrane; Plastid; Plastid outer membrane; Porin; Reference proteome; Transmembrane; Transmembrane beta strand; Transport
SRR1271857_TR41363_c0_g1_i1_len=750	NA	NA	NA	NA	NA	.	0,629	1,304	0,739	0,306	0	2,875	0	3,101	0,004	2,371	.	.	.	.	.	.
SRR1271857_TR41398_c0_g1_i1_len=942	NA	NA	NA	NA	NA	PF06521.8^PAR1^PAR1 protein^35-185^E:1.7e-73	0,044	2,472	0,226	2,577	0	8,523	0	6,949	1	-0,303	.	.	.	.	.	.
SRR1271858_TR3359_c0_g1_i1_len=775	ELOF1_ORYSJ	Q:648-385,H:1-89	92.13%ID	E:1e-41	Transcription elongation factor 1 homolog	.	0,009	0,928	0,035	0,598	0	2,462	0	2,979	0,808	-0,074	ELOF1_ORYSJ	Transcription elongation factor 1 homolog	Os07g0631100 LOC_Os07g43730 OsJ_25233 P0519E12.3	Oryza sativa subsp. japonica (Rice)		Complete proteome; Metal-binding; Nucleus; Reference proteome; Transcription; Transcription regulation; Zinc; Zinc-finger
SRR1271858_TR6554_c0_g1_i2_len=1263	NA	NA	NA	NA	NA	PF00561.17^Abhydrolase_1^alpha/beta hydrolase fold^53-156^E:1.6e-15`PF12697.4^Abhydrolase_6^Alpha/beta hydrolase family^54-170^E:2.2e-09	1	2,944	0,017	5,141	0	8,022	0	8,427	1	0	.	.	.	.	.	.
SRR1271858_TR11582_c0_g2_i1_len=1292	NA	NA	NA	NA	NA	PF14009.3^DUF4228^Domain of unknown function (DUF4228)^71-240^E:3e-26	1	2,231	0,034	4,872	0	4,794	0	5,568	0,822	-0,273	.	.	.	.	.	.
SRR1271858_TR19955_c1_g2_i2_len=1765	ENL2_ARATH	Q:422-730,H:30-132	59.22%ID	E:2e-35	Early nodulin-like protein 2	PF02298.14^Cu_bind_like^Plastocyanin-like domain^44-127^E:1.8e-21	0,288	0,501	0,021	0,983	0	1,961	0	1,984	0,908	-0,083	ENL2_ARATH	Early nodulin-like protein 2 (Phytocyanin-like protein)	At4g27520 T29A15.10	Arabidopsis thaliana (Mouse-ear cress)		Cell membrane; Complete proteome; GPI-anchor; Glycoprotein; Lipoprotein; Membrane; Reference proteome; Signal
SRR1271858_TR26202_c0_g1_i1_len=377	NA	NA	NA	NA	NA	.	0,35	0,582	0,611	-0,457	0	3,216	0	2,622	0,011	-1,253	.	.	.	.	.	.
SRR1271858_TR28353_c0_g3_i2_len=1308	NA	NA	NA	NA	NA	PF12146.5^Hydrolase_4^Serine aminopeptidase, S33^34-148^E:7.2e-34`PF00561.17^Abhydrolase_1^alpha/beta hydrolase fold^39-144^E:5.5e-09`PF12697.4^Abhydrolase_6^Alpha/beta hydrolase family^39-144^E:1.9e-07	0,502	2,827	0,034	4,777	0,001	5,957	0	6,218	1	1,135	.	.	.	.	.	.
SRR1271858_TR28609_c0_g4_i1_len=3258	IDN2_ARATH	Q:459-1886,H:21-501	51.56%ID	E:9e-148	Protein INVOLVED IN DE NOVO 2 {ECO:0000303|PubMed:19915591}	PF03470.11^zf-XS^XS zinc finger domain^48-91^E:6.4e-19`PF03468.11^XS^XS domain^120-231^E:3e-39	1	0,079	0,103	1,771	0	3,598	0	3,708	1	0,046	IDN2_ARATH	Protein INVOLVED IN DE NOVO 2 (Protein RNA-DIRECTED DNA METHYLATION 12)	IDN2 RDM12 At3g48670 T8P19.180	Arabidopsis thaliana (Mouse-ear cress)		3D-structure; Coiled coil; Complete proteome; RNA-binding; RNA-mediated gene silencing; Reference proteome
SRR1271858_TR28987_c0_g1_i4_len=1753	NA	NA	NA	NA	NA	PF04117.9^Mpv17_PMP22^Mpv17 / PMP22 family^250-311^E:1.7e-17	1	0,444	0,043	3,445	0	5,539	0	6,165	1	0,246	.	.	.	.	.	.
SRR1271858_TR31243_c0_g1_i6_len=2333	DOX1_ARATH	Q:88-1275,H:1-368	68.51%ID	E:0	Alpha-dioxygenase 1	PF03098.12^An_peroxidase^Animal haem peroxidase^2-221^E:1.8e-41	1	-0,086	0,001	5,21	0	8,504	0	9,913	0,008	3,99	DOX1_ARATH	Alpha-dioxygenase 1 (Alpha DOX1) (EC 1.14.99.-) (Fatty acid dioxygenase AlphaDOX1) (Pathogen-induced oxygenase) (Plant alpha dioxygenase 1)	DOX1 DIOX1 PADOX-1 PIOX At3g01420 T13O15.6	Arabidopsis thaliana (Mouse-ear cress)		3D-structure; Complete proteome; Dioxygenase; Fatty acid biosynthesis; Fatty acid metabolism; Glycoprotein; Hypersensitive response; Iron; Lipid biosynthesis; Lipid metabolism; Metal-binding; Oxidoreductase; Oxylipin biosynthesis; Peroxidase; Plant defense; Reference proteome; Signal
SRR1271858_TR37116_c0_g1_i3_len=2160	NA	NA	NA	NA	NA	PF00106.22^adh_short^short chain dehydrogenase^17-197^E:1.4e-37`PF08659.7^KR^KR domain^18-195^E:5.7e-07`PF13561.3^adh_short_C2^Enoyl-(Acyl carrier protein) reductase^23-197^E:7.3e-24	1	0,558	0,034	3,772	0	6,997	0	7,202	1	-0,977	.	.	.	.	.	.
SRR1271858_TR37746_c0_g3_i1_len=783	KIRO_ACTDE	Q:174-608,H:1-145	48.97%ID	E:2e-35	Kirola {ECO:0000303|PubMed:21309790}	PF00407.16^Bet_v_1^Pathogenesis-related protein Bet v I family^4-147^E:1.5e-32	0,015	0,647	0,816	-0,069	0	1,393	0	1,203	0,162	0,457	KIRO_ACTDE	Kirola (allergen Act d 11)		Actinidia deliciosa (Kiwi)		3D-structure; Acetylation; Allergen; Direct protein sequencing; Polymorphism
SRR1271858_TR42752_c1_g1_i1_len=1899	CYSP_PEA	Q:127-1179,H:15-362	74.15%ID	E:0	Cysteine proteinase 15A	PF08246.9^Inhibitor_I29^Cathepsin propeptide inhibitor domain (I29)^76-132^E:3.3e-09`PF00112.20^Peptidase_C1^Papain family cysteine protease^163-386^E:3.7e-74	0,002	0,709	0,045	0,442	0	1,915	0	1,335	0,115	0,346	CYSP_PEA	Cysteine proteinase 15A (EC 3.4.22.-) (Turgor-responsive protein 15A)		Pisum sativum (Garden pea)		Disulfide bond; Glycoprotein; Hydrolase; Protease; Signal; Stress response; Thiol protease; Zymogen
SRR1271858_TR43413_c0_g1_i1_len=1182	NA	NA	NA	NA	NA	PF00847.17^AP2^AP2 domain^139-188^E:1.3e-14	1	0,454	0,254	4,481	0	3,981	0	4,145	0,129	-2,887	.	.	.	.	.	.
SRR1271858_TR46311_c2_g2_i1_len=2190	C81E8_MEDTR	Q:245-1699,H:6-493	57.14%ID	E:0	Cytochrome P450 81E8 {ECO:0000303|PubMed:14617078}	PF00067.19^p450^Cytochrome P450^34-477^E:2.3e-90	0,534	0,403	0,001	1,88	0	2,407	0	2,845	0,629	1,184	C81E8_MEDTR	Cytochrome P450 81E8 (EC 1.14.13.-)	CYP81E8	Medicago truncatula (Barrel medic) (Medicago tribuloides)		Heme; Iron; Membrane; Metal-binding; Monooxygenase; NADP; Oxidoreductase; Transmembrane; Transmembrane helix
SRR1271858_TR46468_c0_g1_i1_len=344	NA	NA	NA	NA	NA	.	0,431	-0,443	1	0,012	0	1,804	0,001	1,43	1	0,118	.	.	.	.	.	.
SRR1271858_TR53596_c0_g1_i2_len=1448	PEX13_ARATH	Q:664-1026,H:179-304	67.46%ID	E:2e-49	Peroxisomal membrane protein 13	.	1	0,031	0,005	1,08	0	1,562	0	1,714	0,511	-0,222	PEX13_ARATH	Peroxisomal membrane protein 13 (Peroxin-13) (AtPEX13) (Peroxisome biogenesis protein 13) (Pex13p) (Protein ABERRANT PEROXISOME MORPHOLOGY 2)	PEX13 APM2 At3g07560 F21O3.27	Arabidopsis thaliana (Mouse-ear cress)		Alternative splicing; Complete proteome; Membrane; Peroxisome; Peroxisome biogenesis; Protein transport; Reference proteome; Translocation; Transport
SRR1271858_TR54104_c0_g4_i1_len=1244	EXLB1_ARATH	Q:156-890,H:1-247	54.66%ID	E:9e-92	Expansin-like B1	PF03330.15^DPBB_1^Lytic transglycolase^114-188^E:4.8e-12`PF01357.18^Pollen_allerg_1^Pollen allergen^202-283^E:2.3e-14	0,008	2,897	0,038	1,89	0	3,186	0	3,255	0,005	1,138	EXLB1_ARATH	Expansin-like B1 (At-EXPR1) (AtEXLB1) (AtEXPR1) (Ath-ExpBeta-3.1)	EXLB1 EXPR1 At4g17030 dl4545c	Arabidopsis thaliana (Mouse-ear cress)		Complete proteome; Glycoprotein; Reference proteome; Secreted; Signal
SRR1271858_TR54335_c0_g1_i1_len=2840	SERC_ARATH	Q:2653-2192,H:1-155	61.29%ID	E:1e-51	Phosphoserine phosphatase, chloroplastic	.	0,059	1,189	0,003	1,917	0	2,133	0	2,249	0,381	-2,009	SERC_ARATH	Phosphoserine phosphatase, chloroplastic (PSP) (PSPase) (EC 3.1.3.3) (O-phosphoserine phosphohydrolase)	PSP PSP1 At1g18640 F26I16.2	Arabidopsis thaliana (Mouse-ear cress)	PATHWAY: Amino-acid biosynthesis; L-serine biosynthesis; L-serine from 3-phospho-D-glycerate: step 3/3.	Amino-acid biosynthesis; Chloroplast; Complete proteome; Hydrolase; Magnesium; Metal-binding; Phosphoprotein; Plastid; Reference proteome; Serine biosynthesis; Transit peptide
SRR1271858_TR56671_c1_g1_i1_len=1490	GATLA_ARATH	Q:212-1168,H:37-352	67.08%ID	E:4e-146	Probable galacturonosyltransferase-like 10	PF01501.17^Glyco_transf_8^Glycosyl transferase family 8^70-330^E:6.3e-52	1	0,447	0,005	5,056	0	4,519	0	5,363	0,044	2,373	GATLA_ARATH	Probable galacturonosyltransferase-like 10 (EC 2.4.1.-) (Galactinol synthase 8) (AtGolS8) (GolS-8)	GATL10 GOLS8 At3g28340 MZF16.17	Arabidopsis thaliana (Mouse-ear cress)	PATHWAY: Glycan metabolism; pectin biosynthesis.	Cell wall biogenesis/degradation; Complete proteome; Glycoprotein; Glycosyltransferase; Golgi apparatus; Membrane; Reference proteome; Signal-anchor; Transferase; Transmembrane; Transmembrane helix
SRR1271858_TR68167_c0_g1_i1_len=806	NA	NA	NA	NA	NA	.	0,004	1,956	0,01	2,567	0	3,876	0	3,628	1	0,515	.	.	.	.	.	.
SRR1271858_TR68715_c1_g1_i2_len=2103	CKX5_ARATH	Q:1778-219,H:10-521	71.81%ID	E:0	Cytokinin dehydrogenase 5	PF01565.20^FAD_binding_4^FAD binding domain^60-208^E:1.5e-15`PF09265.7^Cytokin-bind^Cytokinin dehydrogenase 1, FAD and cytokinin binding^240-523^E:3e-121	1	-0,111	0,066	3,842	0	2,668	0	3,445	1	0,205	CKX5_ARATH	Cytokinin dehydrogenase 5 (EC 1.5.99.12) (Cytokinin oxidase 5) (AtCKX5) (AtCKX6) (CKO5)	CKX5 CKX6 At1g75450 F1B16.2	Arabidopsis thaliana (Mouse-ear cress)		Alternative splicing; Complete proteome; FAD; Flavoprotein; Glycoprotein; Oxidoreductase; Reference proteome; Secreted; Signal
SRR1271858_TR69362_c0_g4_i1_len=712	BGL14_ORYSJ	Q:119-538,H:376-513	52.14%ID	E:3e-40	Probable inactive beta-glucosidase 14	PF00232.15^Glyco_hydro_1^Glycosyl hydrolase family 1^6-125^E:1.4e-40	1	0	0,489	-0,53	0	2,483	0,001	2,062	0,239	-1,458	BGL14_ORYSJ	Probable inactive beta-glucosidase 14 (Os4bglu14)	BGLU14 Os04g0513100 LOC_Os04g43360 OSJNBa0004N05.21	Oryza sativa subsp. japonica (Rice)		Complete proteome; Disulfide bond; Glycoprotein; Hydrolase; Reference proteome; Signal
SRR1271859_TR446_c3_g11_i15_len=2775	RBOHC_SOLTU	Q:41-2194,H:249-937	78.64%ID	E:0	Respiratory burst oxidase homolog protein C	PF01794.16^Ferric_reduct^Ferric reductase like transmembrane component^111-267^E:2.3e-23`PF08022.9^FAD_binding_8^FAD-binding domain^310-426^E:1.2e-35`PF08030.9^NAD_binding_6^Ferric reductase NAD binding domain^433-625^E:1.7e-50	0,517	1,262	0,003	1,968	0	5,676	0	7,234	0,458	-0,402	RBOHC_SOLTU	Respiratory burst oxidase homolog protein C (EC 1.11.1.-) (EC 1.6.3.-) (NADPH oxidase RBOHC) (StRBOHC)	RBOHC	Solanum tuberosum (Potato)		Calcium; Coiled coil; Complete proteome; FAD; Flavoprotein; Membrane; Metal-binding; NADP; Oxidoreductase; Peroxidase; Phosphoprotein; Reference proteome; Repeat; Transmembrane; Transmembrane helix
SRR1271859_TR2483_c1_g1_i9_len=1301	RAE1C_ARATH	Q:193-798,H:15-216	93.07%ID	E:7e-117	Ras-related protein RABE1c	PF00025.18^Arf^ADP-ribosylation factor family^16-163^E:2.1e-15`PF00071.19^Ras^Ras family^17-178^E:1.6e-66`PF08477.10^Roc^Ras of Complex, Roc, domain of DAPkinase^17-132^E:4.6e-35`PF04670.9^Gtr1_RagA^Gtr1/RagA G protein conserved region^17-132^E:4.5e-05`PF01926.20^MMR_HSR1^50S ribosome-binding GTPase^18-128^E:4.7e-05	0,811	0,335	0,16	-0,589	0	8,307	0	7,859	0	-3,996	RAE1C_ARATH	Ras-related protein RABE1c (AtRABE1c) (Ras-related protein Ara-3) (Ras-related protein Rab8A) (AtRab8A)	RABE1C ARA-3 RAB8A At3g46060 F12M12.30	Arabidopsis thaliana (Mouse-ear cress)		Cell membrane; Complete proteome; GTP-binding; Golgi apparatus; Lipoprotein; Membrane; Nucleotide-binding; Prenylation; Protein transport; Reference proteome; Transport
SRR1271859_TR4799_c0_g2_i1_len=947	RAC5_ORYSJ	Q:947-483,H:50-174	77.42%ID	E:2e-76	Rac-like GTP-binding protein 5	.	0,661	-0,247	0,044	-0,774	0	8,351	0	7,771	1	0	RAC5_ORYSJ	Rac-like GTP-binding protein 5 (GTPase protein RacD) (OsRac5)	RAC5 RACD Os02g0834000 LOC_Os02g58730 OJ1282_E10.23-1 OJ1282_E10.23-2 P0264G11.2-1 P0264G11.2-2	Oryza sativa subsp. japonica (Rice)		Alternative splicing; Complete proteome; Cytoplasm; GTP-binding; Lipoprotein; Membrane; Methylation; Nucleotide-binding; Prenylation; Reference proteome
SRR1271859_TR15473_c0_g2_i7_len=3276	BAM3_ARATH	Q:3107-1404,H:1-548	65.67%ID	E:0	Beta-amylase 3, chloroplastic	PF01373.14^Glyco_hydro_14^Glycosyl hydrolase family 14^109-526^E:9e-167	0,196	0,855	0,277	0,554	0	1,65	0	1,529	0,739	-0,254	BAM3_ARATH	Beta-amylase 3, chloroplastic (EC 3.2.1.2) (1,4-alpha-D-glucan maltohydrolase) (Beta-amylase 8) (Chloroplast beta-amylase) (CT-BMY)	BAM3 BMY8 CTBMY At4g17090 dl4575c FCAALL.5	Arabidopsis thaliana (Mouse-ear cress)		Carbohydrate metabolism; Chloroplast; Complete proteome; Glycosidase; Hydrolase; Plastid; Polysaccharide degradation; Reference proteome; Transit peptide
SRR1271859_TR15652_c1_g2_i2_len=2722	TM1L2_MOUSE	Q:650-1429,H:17-303	29.17%ID	E:1e-26	TOM1-like protein 2	PF00790.16^VHS^VHS domain^22-132^E:4e-34`PF03127.11^GAT^GAT domain^216-288^E:4.4e-16	0,388	0,362	0,023	0,907	0	1,466	0,001	1,43	0,803	-0,2	TM1L2_MOUSE	TOM1-like protein 2 (Target of Myb-like protein 2)	Tom1l2	Mus musculus (Mouse)		Alternative splicing; Complete proteome; Phosphoprotein; Protein transport; Reference proteome; Transport
SRR1271859_TR24395_c0_g1_i7_len=1072	UBC4_SOLLC	Q:123-566,H:1-148	97.97%ID	E:1e-101	Ubiquitin-conjugating enzyme E2-17 kDa	PF00179.23^UQ_con^Ubiquitin-conjugating enzyme^5-141^E:3.2e-54	0,362	0,302	0,715	0,124	0	1,016	0	1,018	0,416	0,201	UBC4_SOLLC	Ubiquitin-conjugating enzyme E2-17 kDa (EC 2.3.2.23) (E2 ubiquitin-conjugating enzyme) (Ubiquitin carrier protein) (Ubiquitin-protein ligase)		Solanum lycopersicum (Tomato) (Lycopersicon esculentum)	PATHWAY: Protein modification; protein ubiquitination. {ECO:0000255|PROSITE-ProRule:PRU00388}.	ATP-binding; Complete proteome; Nucleotide-binding; Reference proteome; Transferase; Ubl conjugation pathway
SRR1271859_TR24419_c0_g2_i1_len=1845	TF2B_DICDI	Q:470-1042,H:122-312	32.98%ID	E:7e-28	Transcription initiation factor IIB	PF00382.16^TFIIB^Transcription factor TFIIB repeat^149-217^E:1.8e-06`PF00382.16^TFIIB^Transcription factor TFIIB repeat^243-313^E:3.6e-12	1	-0,092	0,257	0,817	0	2,384	0	2,795	0,643	0,44	TF2B_DICDI	Transcription initiation factor IIB (General transcription factor TFIIB)	gtf2b tfiib DDB_G0290929	Dictyostelium discoideum (Slime mold)		Complete proteome; Metal-binding; Nucleus; Reference proteome; Repeat; Transcription; Transcription regulation; Zinc; Zinc-finger
SRR1271859_TR27683_c0_g2_i1_len=1352	TET8_ARATH	Q:316-1050,H:20-267	68.55%ID	E:4e-92	Tetraspanin-8	PF00335.17^Tetraspannin^Tetraspanin family^6-253^E:2.7e-38	0,014	0,742	0,016	0,63	0,001	1,667	0	1,73	0,384	0,263	TET8_ARATH	Tetraspanin-8	TET8 At2g23810 F27L4.1	Arabidopsis thaliana (Mouse-ear cress)		Complete proteome; Glycoprotein; Membrane; Reference proteome; Transmembrane; Transmembrane helix
SRR1271859_TR28455_c0_g3_i1_len=775	EF1A_TOBAC	Q:774-616,H:383-435	96.23%ID	E:9e-26	Elongation factor 1-alpha	.	0,15	-0,414	0,633	-0,114	0	2,607	0	2,1	1	-0,253	EF1A_TOBAC	Elongation factor 1-alpha (EF-1-alpha) (Vitronectin-like adhesion protein 1) (PVN1)		Nicotiana tabacum (Common tobacco)		Cytoplasm; Elongation factor; GTP-binding; Nucleotide-binding; Phosphoprotein; Protein biosynthesis
SRR1271859_TR30604_c0_g1_i3_len=722	NA	NA	NA	NA	NA	.	1	0,76	0,254	3,565	0	5,278	0	4,784	1	-0,295	.	.	.	.	.	.
SRR1271859_TR30921_c0_g1_i1_len=1568	RIC1_ARATH	Q:447-620,H:1-58	81.03%ID	E:4e-26	CRIB domain-containing protein RIC1	PF00786.25^PBD^P21-Rho-binding domain^28-59^E:1.9e-08	0,01	2,343	0,239	1,373	0	3,646	0	3,945	1	-0,062	RIC1_ARATH	CRIB domain-containing protein RIC1 (ROP-interactive CRIB motif-containing protein 1) (Target of ROP protein RIC1)	RIC1 At2g33460 F4P9.23	Arabidopsis thaliana (Mouse-ear cress)		Complete proteome; Cytoplasm; Cytoskeleton; Developmental protein; Growth regulation; Reference proteome
SRR1271859_TR35858_c0_g1_i6_len=537	SODC2_MAIZE	Q:57-437,H:25-151	90.55%ID	E:3e-77	Superoxide dismutase [Cu-Zn] 2	PF00080.17^Sod_Cu^Copper/zinc superoxide dismutase (SODC)^19-141^E:3.6e-44	0,673	0,225	0,528	0,259	0	1,2	0	1,346	0,037	-1,32	SODC2_MAIZE	Superoxide dismutase [Cu-Zn] 2 (EC 1.15.1.1)	SODCC.1 SOD2	Zea mays (Maize)		Antioxidant; Complete proteome; Copper; Cytoplasm; Disulfide bond; Metal-binding; Oxidoreductase; Reference proteome; Zinc
SRR1271859_TR37955_c0_g1_i1_len=4292	Y2182_ARATH	Q:832-2970,H:57-784	35.68%ID	E:6e-112	.	PF08263.9^LRRNT_2^Leucine rich repeat N-terminal domain^47-71^E:0.0002`PF13855.3^LRR_8^Leucine rich repeat^101-162^E:1.9e-07`PF00560.30^LRR_1^Leucine Rich Repeat^102-119^E:1.7`PF13516.3^LRR_6^Leucine Rich repeat^175-188^E:1.1`PF13855.3^LRR_8^Leucine rich repeat^413-471^E:4.7e-10`PF00560.30^LRR_1^Leucine Rich Repeat^460-482^E:0.018`PF13516.3^LRR_6^Leucine Rich repeat^529-544^E:1.2`PF00560.30^LRR_1^Leucine Rich Repeat^530-550^E:0.29`PF00069.22^Pkinase^Protein kinase domain^687-851^E:1.4e-11`PF07714.14^Pkinase_Tyr^Protein tyrosine kinase^701-851^E:1.7e-09	1	0,325	0,008	3,033	0	3,392	0	4,284	1	-0,591	PXC3_ARATH	Leucine-rich repeat receptor-like tyrosine-protein kinase PXC3 (EC 2.7.10.1) (Protein PXY/TDR-CORRELATED 3)	PXC3 At2g41820 T11A7.8	Arabidopsis thaliana (Mouse-ear cress)		ATP-binding; Cell membrane; Complete proteome; Glycoprotein; Kinase; Leucine-rich repeat; Membrane; Nucleotide-binding; Receptor; Reference proteome; Repeat; Signal; Transferase; Transmembrane; Transmembrane helix; Tyrosine-protein kinase
SRR1271859_TR43882_c2_g5_i3_len=2275	PTR53_ARATH	Q:1158-256,H:313-615	54.1%ID	E:0	Protein NRT1/ PTR FAMILY 2.11	PF00854.18^PTR2^POT family^5-247^E:8.2e-47	0,692	1,264	0,026	1,912	0	4,971	0	4,649	1	0,816	PTR53_ARATH	Protein NRT1/ PTR FAMILY 2.11 (AtNPF2.11) (Nitrate transporter 1.10) (Protein GLUCOSINOLATE TRANSPORTER-2)	NPF2.11 GTR2 NRT1.10 At5g62680 MRG21.10	Arabidopsis thaliana (Mouse-ear cress)		Cell membrane; Complete proteome; Membrane; Reference proteome; Transmembrane; Transmembrane helix; Transport
SRR1271859_TR44909_c1_g1_i2_len=994	ARC5A_ARATH	Q:119-511,H:2-132	86.26%ID	E:3e-75	Actin-related protein 2/3 complex subunit 5A	PF04699.11^P16-Arc^ARP2/3 complex 16 kDa subunit (p16-Arc)^13-129^E:2.4e-21	0,559	0,363	0,316	0,64	0	2,186	0	2,44	0,52	0,462	ARC5A_ARATH	Actin-related protein 2/3 complex subunit 5A (Actin-related protein C5A) (Arp2/3 complex 16 kDa subunit) (p16-ARC) (Protein CROOKED)	ARPC5A CRK At4g01710 T15B16.22	Arabidopsis thaliana (Mouse-ear cress)		Acetylation; Actin-binding; Cell projection; Complete proteome; Cytoplasm; Cytoskeleton; Reference proteome
SRR1271859_TR44971_c0_g2_i6_len=809	NOP10_ARATH	Q:525-334,H:1-64	84.38%ID	E:2e-30	H/ACA ribonucleoprotein complex subunit 3-like protein	.	0,402	0,814	0,283	0,645	0	7,444	0	7,357	1	-0,818	NOP10_ARATH	H/ACA ribonucleoprotein complex subunit 3-like protein (Nucleolar protein 10)	At2g20490 T13C7.8	Arabidopsis thaliana (Mouse-ear cress)		Alternative splicing; Complete proteome; Nucleus; Reference proteome; Ribonucleoprotein; Ribosome biogenesis; rRNA processing
SRR1271859_TR46177_c0_g2_i22_len=2978	ACL5_ARATH	Q:1185-256,H:29-338	76.13%ID	E:2e-171	Thermospermine synthase ACAULIS5	PF01564.14^Spermine_synth^Spermine/spermidine synthase^41-274^E:7.9e-55	1	0,032	0,381	-0,678	0	2,559	0,001	2,438	0,34	0,551	ACL5_ARATH	Thermospermine synthase ACAULIS5 (EC 2.5.1.79)	ACL5 TKV At5g19530 T20D1.50	Arabidopsis thaliana (Mouse-ear cress)		Alternative splicing; Complete proteome; Polyamine biosynthesis; Reference proteome; Transferase
SRR342017_TR620_c1_g7_i7_len=2956	NA	NA	NA	NA	NA	PF07887.8^Calmodulin_bind^Calmodulin binding protein-like^91-380^E:7.9e-117	1	0,105	0,068	0,985	0	1,398	0	1,494	0,692	1,46	.	.	.	.	.	.
SRR342017_TR2079_c0_g4_i3_len=1967	PP273_ARATH	Q:1078-434,H:394-610	53%ID	E:5e-49	Pentatricopeptide repeat-containing protein At3g49240	PF04117.9^Mpv17_PMP22^Mpv17 / PMP22 family^355-413^E:2.8e-15	0,298	-0,788	0,048	0,956	0	2,879	0	3,526	0,524	-0,594	PP273_ARATH	Pentatricopeptide repeat-containing protein At3g49240 (Protein EMBRYO DEFECTIVE 1796)	EMB1796 At3g49240 F2K15.100	Arabidopsis thaliana (Mouse-ear cress)		Complete proteome; Reference proteome; Repeat
SRR342017_TR5846_c0_g1_i2_len=690	NRPBC_ARATH	Q:602-450,H:1-51	90.2%ID	E:3e-26	DNA-directed RNA polymerases II, IV and V subunit 12	.	1	-0,173	0,074	0,925	0	7,409	0	6,759	0,461	-1,046	NRPBC_ARATH	DNA-directed RNA polymerases II, IV and V subunit 12 (DNA-directed RNA Polymerase II subunit K)	NRPB12 NRPD12 NRPE12 At5g41010 MEE6.8	Arabidopsis thaliana (Mouse-ear cress)		Complete proteome; DNA-directed RNA polymerase; Metal-binding; Nucleus; Reference proteome; Transcription; Zinc
SRR342017_TR7809_c1_g1_i3_len=1867	CXE1_ACTER	Q:1848-970,H:44-333	47.12%ID	E:5e-92	Carboxylesterase 1	PF10340.6^DUF2424^Protein of unknown function (DUF2424)^45-155^E:5.1e-05`PF07859.10^Abhydrolase_3^alpha/beta hydrolase fold^52-271^E:1.2e-45	0,06	0,827	0,252	0,538	0	2,066	0	1,89	1	0,056	CXE1_ACTER	Carboxylesterase 1 (AeCXE1) (EC 3.1.1.1)	CXE1	Actinidia eriantha (Velvet vine) (Actinidia fulvicoma var. lanata)		3D-structure; Hydrolase; Serine esterase
SRR342017_TR9822_c1_g1_i1_len=845	UEV1C_ARATH	Q:624-187,H:1-145	89.73%ID	E:2e-88	Ubiquitin-conjugating enzyme E2 variant 1C	PF00179.23^UQ_con^Ubiquitin-conjugating enzyme^43-136^E:8.3e-15	0,407	0,375	0,881	0,053	0	2,757	0	3,479	0	-4,15	UEV1C_ARATH	Ubiquitin-conjugating enzyme E2 variant 1C (Ubc enzyme variant 1C) (Protein MMS ZWEI HOMOLOG 3)	UEV1C MMZ3 At2g36060 F11F19.3	Arabidopsis thaliana (Mouse-ear cress)		Alternative splicing; Complete proteome; Reference proteome
SRR342017_TR10372_c0_g8_i1_len=1923	TT12_ARATH	Q:1826-381,H:1-494	39.52%ID	E:5e-108	.	PF01554.15^MatE^MatE^49-209^E:1e-29`PF01554.15^MatE^MatE^272-432^E:6e-26	0,009	0,796	0,037	0,723	0	1,567	0	1,776	0,949	0,05	DTX41_ARATH	Protein DETOXIFICATION 41 (AtDTX41) (Multidrug and toxic compound extrusion protein 41) (MATE protein 41) (Protein TANNIN-DEFICIENT SEED 3) (Protein TRANSPARENT TESTA 12)	DTX41 TDS3 TT12 At3g59030 F17J16_80	Arabidopsis thaliana (Mouse-ear cress)	PATHWAY: Secondary metabolite biosynthesis; flavonoid biosynthesis.	Antiport; Complete proteome; Flavonoid biosynthesis; Membrane; Reference proteome; Transmembrane; Transmembrane helix; Transport; Vacuole
SRR342017_TR16264_c0_g2_i1_len=409	NA	NA	NA	NA	NA	.	0,032	0,511	0,609	-0,132	0	1,805	0	1,086	0,001	0,789	.	.	.	.	.	.
SRR342017_TR16783_c0_g1_i1_len=607	C81E8_MEDTR	Q:76-606,H:184-366	42.19%ID	E:5e-39	Cytochrome P450 81E8 {ECO:0000303|PubMed:14617078}	PF00067.19^p450^Cytochrome P450^24-175^E:9e-26	1	0	0,001	6,424	0	7,691	0	8,766	0,009	-6,1	C81E8_MEDTR	Cytochrome P450 81E8 (EC 1.14.13.-)	CYP81E8	Medicago truncatula (Barrel medic) (Medicago tribuloides)		Heme; Iron; Membrane; Metal-binding; Monooxygenase; NADP; Oxidoreductase; Transmembrane; Transmembrane helix
SRR342017_TR16981_c0_g5_i1_len=582	CHMP1_DICDI	Q:222-581,H:3-122	54.17%ID	E:2e-30	Charged multivesicular body protein 1	PF03357.18^Snf7^Snf7^11-125^E:2.5e-08	0,648	-0,22	0,138	0,911	0	7,662	0	6,834	0,125	0,516	CHMP1_DICDI	Charged multivesicular body protein 1 (Developmental gene 1118 protein) (Vacuolar protein-sorting-associated protein 46)	chmp1 DG1118 vps46 DDB_G0267394	Dictyostelium discoideum (Slime mold)		Coiled coil; Complete proteome; Endosome; Membrane; Protein transport; Reference proteome; Transport
SRR342017_TR17600_c2_g1_i1_len=1330	NA	NA	NA	NA	NA	.	0,691	-0,215	1	-0,001	0	2,18	0	2,169	1	0,031	.	.	.	.	.	.
SRR342017_TR19411_c0_g1_i1_len=535	NA	NA	NA	NA	NA	PF02519.11^Auxin_inducible^Auxin responsive protein^25-92^E:2.8e-15	1	0,465	0,066	3,329	0	6,816	0	7,413	0,629	-1,969	.	.	.	.	.	.
SRR342017_TR23073_c0_g2_i2_len=2424	SPEA_BACHD	Q:1548-577,H:162-483	38.07%ID	E:7e-49	Arginine decarboxylase	PF01276.17^OKR_DC_1^Orn/Lys/Arg decarboxylase, major domain^11-157^E:1.3e-26`PF03711.12^OKR_DC_1_C^Orn/Lys/Arg decarboxylase, C-terminal domain^257-318^E:2.9e-10	0,517	-0,777	0,228	1,086	0	3,842	0	4,902	1	-0,314	SPEA_BACHD	Arginine decarboxylase (EC 4.1.1.19)	speA BH2640	Bacillus halodurans (strain ATCC BAA-125 / DSM 18197 / FERM 7344 / JCM 9153 / C-125)	PATHWAY: Amine and polyamine biosynthesis; agmatine biosynthesis; agmatine from L-arginine: step 1/1.	Complete proteome; Cytoplasm; Decarboxylase; Lyase; Polyamine biosynthesis; Putrescine biosynthesis; Pyridoxal phosphate; Spermidine biosynthesis
SRR342017_TR23753_c0_g1_i1_len=750	ATG8C_ORYSJ	Q:614-264,H:1-117	96.58%ID	E:7e-66	Autophagy-related protein 8C	PF02991.13^Atg8^Autophagy protein Atg8 ubiquitin like^14-117^E:7.7e-52`PF04110.10^APG12^Ubiquitin-like autophagy protein Apg12^40-117^E:5.4e-06	0,43	0,213	0,213	0,355	0	1,514	0	1,193	0,091	0,577	ATG8C_ORYSJ	Autophagy-related protein 8C (Autophagy-related ubiquitin-like modifier ATG8C)	ATG8C APG8C Os08g0191600 LOC_Os08g09240 OSJNBa0056O06.5	Oryza sativa subsp. japonica (Rice)		Autophagy; Complete proteome; Cytoplasm; Cytoplasmic vesicle; Cytoskeleton; Lipoprotein; Membrane; Microtubule; Protein transport; Reference proteome; Transport; Ubl conjugation pathway; Vacuole
SRR342017_TR30411_c0_g3_i1_len=968	Y2416_ARATH	Q:738-403,H:1-111	78.57%ID	E:2e-55	Uncharacterized protein At2g34160	.	0,193	-0,782	0,165	-0,749	0,001	6,577	0,001	6,628	1	-1,883	Y2416_ARATH	Uncharacterized protein At2g34160	At2g34160 T14G11.28	Arabidopsis thaliana (Mouse-ear cress)		3D-structure; Acetylation; Complete proteome; Reference proteome
SRR342017_TR32809_c1_g2_i1_len=549	NA	NA	NA	NA	NA	.	1	0	0,066	-5,334	0	8,052	0	7,299	1	0	.	.	.	.	.	.
SRR342019_TR2632_c0_g3_i1_len=584	NA	NA	NA	NA	NA	.	0,718	-0,196	0,193	0,711	0	1,94	0	1,799	0,656	-0,318	.	.	.	.	.	.
SRR342019_TR3802_c1_g2_i1_len=1205	CHID_SOLLC	Q:170-892,H:5-245	75.93%ID	E:5e-128	Basic endochitinase	PF00182.16^Glyco_hydro_19^Chitinase class I^25-256^E:3.6e-132	0,034	3,762	0,132	2,59	0	6,023	0	3,87	0,103	1,773	CHID_SOLLC	Basic endochitinase (EC 3.2.1.14) (Fragment)	CHI14	Solanum lycopersicum (Tomato) (Lycopersicon esculentum)		Carbohydrate metabolism; Chitin degradation; Complete proteome; Disulfide bond; Glycosidase; Hydrolase; Plant defense; Polysaccharide degradation; Reference proteome; Secreted
SRR342019_TR3921_c0_g1_i2_len=706	NA	NA	NA	NA	NA	.	0,132	3,202	0,004	2,265	0	5,83	0	6,02	1	-0,905	.	.	.	.	.	.
SRR342019_TR4291_c0_g2_i2_len=1728	HAT4_ARATH	Q:988-1254,H:145-240	69.79%ID	E:2e-31	Homeobox-leucine zipper protein HAT4	.	0,781	-0,518	0,021	1,542	0	3,992	0	3,881	1	-1,018	HAT4_ARATH	Homeobox-leucine zipper protein HAT4 (HD-ZIP protein ATHB-2) (Homeodomain transcription factor ATHB-2) (Homeodomain-leucine zipper protein HAT4) (HD-ZIP protein 4)	HAT4 ATHB-2 At4g16780 dl4415w FCAALL.101	Arabidopsis thaliana (Mouse-ear cress)		Complete proteome; DNA-binding; Developmental protein; Homeobox; Nucleus; Phytochrome signaling pathway; Reference proteome; Repressor; Transcription; Transcription regulation
SRR342019_TR8720_c0_g6_i1_len=959	NA	NA	NA	NA	NA	PF02221.12^E1_DerP2_DerF2^ML domain^27-146^E:5.9e-21	0,868	0,078	1	-0,002	0	1,305	0	1,278	0,816	0,074	.	.	.	.	.	.
SRR342019_TR10770_c0_g1_i3_len=1427	APT5_ARATH	Q:210-761,H:1-184	79.35%ID	E:7e-97	Adenine phosphoribosyltransferase 5	PF00156.24^Pribosyltran^Phosphoribosyl transferase domain^38-163^E:4.4e-18	1	-0,072	0,278	0,757	0	2,407	0	2,687	0,298	-0,624	APT5_ARATH	Adenine phosphoribosyltransferase 5 (AtAPT5) (EC 2.4.2.7)	APT5 At5g11160 F2I11.50	Arabidopsis thaliana (Mouse-ear cress)	PATHWAY: Purine metabolism; AMP biosynthesis via salvage pathway; AMP from adenine: step 1/1.	Complete proteome; Cytoplasm; Glycosyltransferase; Purine salvage; Reference proteome; Transferase
SRR342019_TR10890_c5_g1_i1_len=1226	NA	NA	NA	NA	NA	.	0,712	-0,246	0,729	0,205	0	2,261	0	3,226	0,299	-1,402	.	.	.	.	.	.
SRR342019_TR14536_c0_g1_i1_len=562	NA	NA	NA	NA	NA	PF00280.15^potato_inhibit^Potato inhibitor I family^55-117^E:1.2e-22	0,009	4,712	0,254	4,697	0	11,523	0	7,627	1	2,407	.	.	.	.	.	.
SRR342019_TR15088_c0_g2_i2_len=1051	PEX13_ARATH	Q:787-422,H:178-304	66.93%ID	E:3e-49	Peroxisomal membrane protein 13	.	0,62	0,227	0,356	0,366	0	1,689	0	1,936	0,781	-0,448	PEX13_ARATH	Peroxisomal membrane protein 13 (Peroxin-13) (AtPEX13) (Peroxisome biogenesis protein 13) (Pex13p) (Protein ABERRANT PEROXISOME MORPHOLOGY 2)	PEX13 APM2 At3g07560 F21O3.27	Arabidopsis thaliana (Mouse-ear cress)		Alternative splicing; Complete proteome; Membrane; Peroxisome; Peroxisome biogenesis; Protein transport; Reference proteome; Translocation; Transport
SRR342019_TR18690_c4_g2_i1_len=2431	NA	NA	NA	NA	NA	.	0,447	-0,372	0,075	-0,749	0	1,803	0	2,301	0,87	-0,09	.	.	.	.	.	.
SRR342019_TR18705_c0_g2_i5_len=2086	FIP2_ARATH	Q:436-212,H:224-298	84%ID	E:2e-30	FH protein interacting protein FIP2	.	0,052	1,563	0,002	2,801	0	3,329	0	2,756	0,502	-2,641	FIP2_ARATH	FH protein interacting protein FIP2 (BTB/POZ domain-containing protein At5g55000)	FIP2 At5g55000 MBG8.27	Arabidopsis thaliana (Mouse-ear cress)	PATHWAY: Protein modification; protein ubiquitination.	Alternative splicing; Complete proteome; Reference proteome; Repeat; Ubl conjugation pathway
SRR342019_TR19616_c0_g5_i1_len=758	NA	NA	NA	NA	NA	.	0,692	0,996	0,158	1,534	0	2,83	0	2,663	0,732	0,46	.	.	.	.	.	.
SRR342019_TR20229_c0_g1_i1_len=584	NA	NA	NA	NA	NA	.	0,231	-0,608	0,008	3,275	0	6,926	0	7,265	0,277	-0,565	.	.	.	.	.	.
SRR342019_TR23814_c0_g2_i2_len=605	KIRO_ACTDE	Q:49-498,H:1-150	65.33%ID	E:8e-64	Kirola {ECO:0000303|PubMed:21309790}	PF00407.16^Bet_v_1^Pathogenesis-related protein Bet v I family^2-149^E:7.3e-45	1	0,005	0,699	-0,156	0	1,31	0,001	1,012	0,002	0,737	KIRO_ACTDE	Kirola (allergen Act d 11)		Actinidia deliciosa (Kiwi)		3D-structure; Acetylation; Allergen; Direct protein sequencing; Polymorphism
SRR342019_TR25167_c3_g2_i1_len=628	KIRO_ACTDE	Q:6-320,H:44-148	50.48%ID	E:8e-23	Kirola {ECO:0000303|PubMed:21309790}	PF00407.16^Bet_v_1^Pathogenesis-related protein Bet v I family^2-105^E:2.1e-24	0,01	0,586	0,495	-0,172	0	1,498	0	0,985	0,358	0,22	KIRO_ACTDE	Kirola (allergen Act d 11)		Actinidia deliciosa (Kiwi)		3D-structure; Acetylation; Allergen; Direct protein sequencing; Polymorphism
SRR342019_TR31099_c1_g2_i2_len=2133	JAR1_ARATH	Q:1966-146,H:13-575	47.45%ID	E:0	Jasmonic acid-amido synthetase JAR1	PF03321.10^GH3^GH3 auxin-responsive promoter^32-615^E:3.4e-180	0,868	0,074	0,055	-0,805	0	1,133	0	1,139	0,001	-1,197	JAR1_ARATH	Jasmonic acid-amido synthetase JAR1 (EC 6.3.2.-) (Jasmonate-amino acid synthetase JAR1) (Protein FAR-RED INSENSITIVE 219) (Protein JASMONATE RESISTANT 1)	JAR1 FIN219 At2g46370 F11C10.6	Arabidopsis thaliana (Mouse-ear cress)		3D-structure; Alternative splicing; Coiled coil; Complete proteome; Cytoplasm; Immunity; Innate immunity; Jasmonic acid signaling pathway; Ligase; Plant defense; Reference proteome
SRR342019_TR31660_c0_g1_i2_len=964	NA	NA	NA	NA	NA	.	1	1,341	0,034	5,705	0	7,368	0	8,805	1	0,77	.	.	.	.	.	.
SRR342019_TR32889_c2_g2_i6_len=1863	NA	NA	NA	NA	NA	PF00717.20^Peptidase_S24^Peptidase S24-like^111-168^E:2.5e-07	1	0,643	0,086	-0,969	0	7,318	0	7,173	1	-0,679	.	.	.	.	.	.
SRR342019_TR35359_c0_g3_i3_len=853	NA	NA	NA	NA	NA	PF01221.15^Dynein_light^Dynein light chain type 1^5-89^E:5.5e-30	1	-0,013	1	-0,076	0	1,549	0	1,196	0,807	-0,168	.	.	.	.	.	.
SRR342019_TR36508_c0_g3_i1_len=975	DNAJ8_ARATH	Q:516-767,H:55-137	64.71%ID	E:6e-29	Chaperone protein dnaJ 8, chloroplastic	PF00226.28^DnaJ^DnaJ domain^43-96^E:2.1e-15	1	-0,806	0	-5,83	0	2,313	0	1,3	1	1,373	DNAJ8_ARATH	Chaperone protein dnaJ 8, chloroplastic (AtDjC8) (AtJ8)	ATJ8 C8 J8 At1g80920 F23A5.28	Arabidopsis thaliana (Mouse-ear cress)		Chaperone; Chloroplast; Complete proteome; Plastid; Reference proteome; Transit peptide
SRR342019_TR37176_c0_g2_i1_len=1337	NA	NA	NA	NA	NA	PF03168.10^LEA_2^Late embryogenesis abundant protein^165-250^E:1.7e-09	1	-0,87	0,286	-0,86	0	4,78	0	4,365	1	-0,296	.	.	.	.	.	.
SRR342019_TR39145_c0_g1_i1_len=3556	NAA15_PONAB	Q:3364-1001,H:3-785	43.3%ID	E:0	N-alpha-acetyltransferase 15, NatA auxiliary subunit	PF07719.14^TPR_2^Tetratricopeptide repeat^79-110^E:4.2e-06`PF13181.3^TPR_8^Tetratricopeptide repeat^80-110^E:8.4e-05`PF13414.3^TPR_11^TPR repeat^84-123^E:2.2e-07`PF13176.3^TPR_7^Tetratricopeptide repeat^84-110^E:0.0023`PF12569.5^NARP1^NMDA receptor-regulated protein 1^187-696^E:3.9e-202	1	-0,085	0,142	-0,74	0	4,405	0	4,383	1	0,217	NAA15_PONAB	N-alpha-acetyltransferase 15, NatA auxiliary subunit (NMDA receptor-regulated protein 1)	NAA15 NARG1	Pongo abelii (Sumatran orangutan) (Pongo pygmaeus abelii)		Acetylation; Complete proteome; Cytoplasm; Nucleus; Phosphoprotein; Reference proteome; Repeat; TPR repeat
SRR342022_TR482_c2_g4_i1_len=1348	PDCB3_ARATH	Q:487-687,H:20-86	71.64%ID	E:5e-30	PLASMODESMATA CALLOSE-BINDING PROTEIN 3	PF07983.10^X8^X8 domain^21-92^E:1.8e-19	0,586	-0,154	0,901	0,051	0	1,2	0	1,491	0,23	-0,35	PDCB3_ARATH	PLASMODESMATA CALLOSE-BINDING PROTEIN 3 (AtPDCB3)	PDCB3 At1g18650 F25I16.1	Arabidopsis thaliana (Mouse-ear cress)		Cell junction; Cell membrane; Complete proteome; Disulfide bond; GPI-anchor; Glycoprotein; Lipoprotein; Membrane; Reference proteome; Signal
SRR342022_TR492_c0_g2_i1_len=802	NA	NA	NA	NA	NA	.	0,56	0,677	0,01	2,106	0	2,293	0	2,311	1	-0,027	.	.	.	.	.	.
SRR342022_TR585_c0_g7_i3_len=2282	THIK2_ARATH	Q:204-1595,H:1-462	84.05%ID	E:0	3-ketoacyl-CoA thiolase 2, peroxisomal	PF00108.20^Thiolase_N^Thiolase, N-terminal domain^54-309^E:3.2e-79`PF02803.15^Thiolase_C^Thiolase, C-terminal domain^318-440^E:2.2e-48	0,674	0,124	0,106	0,44	0	1,438	0	1,281	1	-0,094	THIK2_ARATH	3-ketoacyl-CoA thiolase 2, peroxisomal (EC 2.3.1.16) (Acetyl-CoA acyltransferase 2) (Beta-ketothiolase 2) (Peroxisomal 3-oxoacyl-CoA thiolase 2) (Peroxisome defective protein 1)	PED1 KAT2 At2g33150 F25I18.11	Arabidopsis thaliana (Mouse-ear cress)	PATHWAY: Lipid metabolism; fatty acid metabolism.	3D-structure; Acyltransferase; Complete proteome; Fatty acid biosynthesis; Fatty acid metabolism; Glyoxysome; Lipid biosynthesis; Lipid metabolism; Oxylipin biosynthesis; Peroxisome; Reference proteome; Transferase; Transit peptide
SRR342022_TR1398_c2_g4_i7_len=2375	SERC_ARATH	Q:184-951,H:1-257	68.09%ID	E:4e-114	Phosphoserine phosphatase, chloroplastic	PF00702.23^Hydrolase^haloacid dehalogenase-like hydrolase^83-253^E:7.3e-16`PF12710.4^HAD^haloacid dehalogenase-like hydrolase^86-253^E:3.1e-12	1	0,478	0,226	2,493	0	2,699	0	2,893	0,461	1,15	SERC_ARATH	Phosphoserine phosphatase, chloroplastic (PSP) (PSPase) (EC 3.1.3.3) (O-phosphoserine phosphohydrolase)	PSP PSP1 At1g18640 F26I16.2	Arabidopsis thaliana (Mouse-ear cress)	PATHWAY: Amino-acid biosynthesis; L-serine biosynthesis; L-serine from 3-phospho-D-glycerate: step 3/3.	Amino-acid biosynthesis; Chloroplast; Complete proteome; Hydrolase; Magnesium; Metal-binding; Phosphoprotein; Plastid; Reference proteome; Serine biosynthesis; Transit peptide
SRR342022_TR2139_c0_g1_i1_len=865	NA	NA	NA	NA	NA	.	1	0,007	0,377	0,221	0	1,478	0	1,357	1	0,199	.	.	.	.	.	.
SRR342022_TR3621_c2_g8_i14_len=4963	COB22_ARATH	Q:284-3055,H:1-926	83.91%ID	E:0	Coatomer subunit beta'-2	PF00400.29^WD40^WD domain, G-beta repeat^91-127^E:8e-06`PF00400.29^WD40^WD domain, G-beta repeat^133-171^E:0.00013`PF00400.29^WD40^WD domain, G-beta repeat^177-215^E:5.6e-06`PF00400.29^WD40^WD domain, G-beta repeat^220-256^E:6.6e-09`PF04053.11^Coatomer_WDAD^Coatomer WD associated region^319-763^E:7.2e-167	1	0,304	0,847	-0,089	0	5,957	0	5,97	1	-1,127	COB22_ARATH	Coatomer subunit beta'-2 (Beta'-coat protein 2) (Beta'-COP 2)	At1g52360 F19K6.16	Arabidopsis thaliana (Mouse-ear cress)		Alternative splicing; Complete proteome; Cytoplasm; Cytoplasmic vesicle; ER-Golgi transport; Golgi apparatus; Membrane; Protein transport; Reference proteome; Repeat; Transport; WD repeat
SRR342022_TR5920_c1_g1_i4_len=3954	ISPH_ARATH	Q:144-1529,H:1-466	78.16%ID	E:0	4-hydroxy-3-methylbut-2-enyl diphosphate reductase, chloroplastic	PF02401.15^LYTB^LytB protein^109-449^E:2.6e-82	0,004	0,822	0,012	0,73	0	1,005	0	0,923	0,843	0,056	ISPH_ARATH	4-hydroxy-3-methylbut-2-enyl diphosphate reductase, chloroplastic (EC 1.17.1.2) (Protein CHLOROPLAST BIOGENESIS 6)	ISPH CLB6 HDR At4g34350 F10M10.120	Arabidopsis thaliana (Mouse-ear cress)	PATHWAY: Isoprenoid biosynthesis; dimethylallyl diphosphate biosynthesis; dimethylallyl diphosphate from (2E)-4-hydroxy-3-methylbutenyl diphosphate: step 1/1.; PATHWAY: Isoprenoid biosynthesis; isopentenyl diphosphate biosynthesis via DXP pathway; isopentenyl diphosphate from 1-deoxy-D-xylulose 5-phosphate: step 6/6.	3Fe-4S; Chloroplast; Complete proteome; Iron; Iron-sulfur; Isoprene biosynthesis; Metal-binding; NADP; Oxidoreductase; Plastid; Reference proteome; Transit peptide
SRR342022_TR9018_c2_g2_i7_len=2553	NA	NA	NA	NA	NA	PF14735.3^HAUS4^HAUS augmin-like complex subunit 4^181-416^E:2e-79	0,694	-0,418	0,007	1,998	0	2,492	0	3,309	1	-0,154	.	.	.	.	.	.
SRR342022_TR9584_c0_g1_i1_len=1986	IP5PB_ARATH	Q:1091-99,H:10-333	54.82%ID	E:3e-129	Type IV inositol polyphosphate 5-phosphatase 11 {ECO:0000303|PubMed:15181205}	PF03372.20^Exo_endo_phos^Endonuclease/Exonuclease/phosphatase family^56-321^E:9.4e-28	1	0,135	0,006	2,665	0	2,526	0	2,945	1	-0,367	IP5PB_ARATH	Type IV inositol polyphosphate 5-phosphatase 11 (At5PTase11) (EC 3.1.3.36) (EC 3.1.3.86)	IP5P11 At1g47510 F16N3.22	Arabidopsis thaliana (Mouse-ear cress)		Alternative splicing; Cell membrane; Complete proteome; Hydrolase; Membrane; Reference proteome
SRR342022_TR9587_c2_g5_i2_len=1495	QORH_ARATH	Q:1195-209,H:1-329	79.33%ID	E:0	Putative quinone-oxidoreductase homolog, chloroplastic	PF08240.9^ADH_N^Alcohol dehydrogenase GroES-like domain^34-95^E:1.2e-08`PF00107.23^ADH_zinc_N^Zinc-binding dehydrogenase^167-272^E:8.2e-12`PF13602.3^ADH_zinc_N_2^Zinc-binding dehydrogenase^198-327^E:4e-24	0,854	-0,246	0,316	0,595	0	2,302	0	1,89	0,298	0,619	QORH_ARATH	Putative quinone-oxidoreductase homolog, chloroplastic (EC 1.-.-.-)	At4g13010 F25G13_100	Arabidopsis thaliana (Mouse-ear cress)		Chloroplast; Complete proteome; Membrane; NAD; Oxidoreductase; Plastid; Plastid inner membrane; Reference proteome
SRR342022_TR11743_c1_g10_i1_len=544	NA	NA	NA	NA	NA	.	0,433	-0,281	0,009	-0,775	0	8,627	0	8,142	1	3,673	.	.	.	.	.	.
SRR342022_TR13399_c3_g6_i1_len=638	C71B1_THLAR	Q:536-171,H:373-494	45.08%ID	E:3e-35	Cytochrome P450 71B1	PF00067.19^p450^Cytochrome P450^21-125^E:2.5e-28	0,286	0,297	0,062	0,469	0	10,147	0	7,867	1	0	C71B1_THLAR	Cytochrome P450 71B1 (EC 1.14.-.-) (CYPLXXIB1)	CYP71B1	Thlaspi arvense (Field penny-cress)		Heme; Iron; Metal-binding; Monooxygenase; Oxidoreductase
SRR342022_TR15235_c0_g1_i2_len=1587	NA	NA	NA	NA	NA	.	1	-0,557	0,142	-0,856	0	7,621	0	7,499	0,034	5,592	.	.	.	.	.	.
SRR342022_TR17193_c0_g3_i3_len=607	RL291_ARATH	Q:95-277,H:1-61	81.97%ID	E:8e-27	60S ribosomal protein L29-1	.	0	-0,77	0,656	0,115	0	1,244	0	0,85	0,019	-0,604	RL291_ARATH	60S ribosomal protein L29-1	RPL29A At3g06700 F3E22.16 T8E24.8	Arabidopsis thaliana (Mouse-ear cress)		Complete proteome; Phosphoprotein; Reference proteome; Ribonucleoprotein; Ribosomal protein
SRR342022_TR18075_c0_g1_i1_len=859	IAA17_ARATH	Q:123-698,H:3-218	51.61%ID	E:4e-61	Auxin-responsive protein IAA17	PF02309.13^AUX_IAA^AUX/IAA family^31-202^E:8.1e-59	1	-1,296	0,145	0,528	0	8,341	0	8,359	1	0,999	IAA17_ARATH	Auxin-responsive protein IAA17 (Auxin response 3) (Indoleacetic acid-induced protein 17)	IAA17 AXR3 At1g04250 F19P19.31	Arabidopsis thaliana (Mouse-ear cress)		3D-structure; Auxin signaling pathway; Complete proteome; Nucleus; Phosphoprotein; Reference proteome; Repressor; Transcription; Transcription regulation
SRR342022_TR20959_c3_g3_i1_len=1188	SDC1_ARATH	Q:998-333,H:77-297	62.61%ID	E:2e-95	Serine decarboxylase	PF00282.16^Pyridoxal_deC^Pyridoxal-dependent decarboxylase conserved domain^47-194^E:7.4e-13	0,074	0,928	0,02	1,362	0	3,493	0	3,07	0,25	0,788	SDC1_ARATH	Serine decarboxylase (AtSDC) (EC 4.1.1.-) (Protein EMBRYO DEFECTIVE 1075) (Serine decarboxylase 1) (AtSDC1)	SDC EMB1075 SDC1 At1g43710 F2J6.7	Arabidopsis thaliana (Mouse-ear cress)		Complete proteome; Cytoplasm; Decarboxylase; Lyase; Pyridoxal phosphate; Reference proteome
SRR342022_TR20959_c3_g3_i2_len=980	SDC1_ARATH	Q:902-336,H:109-296	68.78%ID	E:2e-89	Serine decarboxylase	PF00282.16^Pyridoxal_deC^Pyridoxal-dependent decarboxylase conserved domain^37-183^E:6.9e-13	0,002	1,347	0,02	1,12	0	1,846	0	2,132	0,2	0,64	SDC1_ARATH	Serine decarboxylase (AtSDC) (EC 4.1.1.-) (Protein EMBRYO DEFECTIVE 1075) (Serine decarboxylase 1) (AtSDC1)	SDC EMB1075 SDC1 At1g43710 F2J6.7	Arabidopsis thaliana (Mouse-ear cress)		Complete proteome; Cytoplasm; Decarboxylase; Lyase; Pyridoxal phosphate; Reference proteome
SRR342022_TR21116_c0_g4_i1_len=598	DNAJ8_ARATH	Q:596-330,H:75-162	68.89%ID	E:1e-25	Chaperone protein dnaJ 8, chloroplastic	.	1	-0,37	0	-8,236	0	2,338	0,001	1,127	1	-1,279	DNAJ8_ARATH	Chaperone protein dnaJ 8, chloroplastic (AtDjC8) (AtJ8)	ATJ8 C8 J8 At1g80920 F23A5.28	Arabidopsis thaliana (Mouse-ear cress)		Chaperone; Chloroplast; Complete proteome; Plastid; Reference proteome; Transit peptide
SRR342022_TR21224_c2_g5_i4_len=2745	DGAT2_ARATH	Q:1669-860,H:46-314	71.85%ID	E:7e-138	Diacylglycerol O-acyltransferase 2	PF03982.10^DAGAT^Diacylglycerol acyltransferase^4-254^E:2.1e-79	0,318	0,475	0,881	-0,078	0	1,513	0	1,733	0,231	-0,598	DGAT2_ARATH	Diacylglycerol O-acyltransferase 2 (EC 2.3.1.20)	DGAT2 At3g51520 F26O13.160	Arabidopsis thaliana (Mouse-ear cress)	PATHWAY: Glycerolipid metabolism; triacylglycerol biosynthesis.	Acyltransferase; Complete proteome; Endoplasmic reticulum; Glycerol metabolism; Glycoprotein; Lipid biosynthesis; Lipid metabolism; Membrane; Reference proteome; Transferase; Transmembrane; Transmembrane helix
SRR342022_TR27684_c0_g1_i1_len=848	Y2416_ARATH	Q:130-516,H:1-130	73.08%ID	E:3e-56	Uncharacterized protein At2g34160	PF01918.18^Alba^Alba^20-82^E:3.7e-12	0,459	-0,203	0,416	-0,206	0	1,397	0	1,315	1	0,416	Y2416_ARATH	Uncharacterized protein At2g34160	At2g34160 T14G11.28	Arabidopsis thaliana (Mouse-ear cress)		3D-structure; Acetylation; Complete proteome; Reference proteome
SRR342022_TR27863_c4_g2_i6_len=4886	GLR35_ARATH	Q:3431-1800,H:102-649	68.43%ID	E:0	Glutamate receptor 3.5	PF01094.25^ANF_receptor^Receptor family ligand binding region^2-255^E:9.4e-49`PF10613.6^Lig_chan-Glu_bd^Ligated ion channel L-glutamate- and glycine-binding site^321-419^E:4.9e-11`PF00497.17^SBP_bac_3^Bacterial extracellular solute-binding proteins, family 3^329-423^E:2.4e-09	0,692	0,994	0,517	1,164	0	3,643	0	3,513	1	-1,425	GLR35_ARATH	Glutamate receptor 3.5 (Ionotropic glutamate receptor GLR6) (Ligand-gated ion channel 3.5)	GLR3.5 GLR6 At2g32390 T32F6.9	Arabidopsis thaliana (Mouse-ear cress)		Alternative splicing; Complete proteome; Glycoprotein; Ion channel; Ion transport; Ligand-gated ion channel; Membrane; Receptor; Reference proteome; Signal; Transmembrane; Transmembrane helix; Transport
SRR342022_TR30302_c1_g3_i1_len=2189	ODO1_PETHY	Q:1267-881,H:1-129	90.7%ID	E:1e-76	Protein ODORANT1	PF00249.28^Myb_DNA-binding^Myb-like DNA-binding domain^14-61^E:2.9e-14`PF13921.3^Myb_DNA-bind_6^Myb-like DNA-binding domain^17-76^E:6.7e-08`PF00249.28^Myb_DNA-binding^Myb-like DNA-binding domain^71-111^E:4.7e-14`PF13921.3^Myb_DNA-bind_6^Myb-like DNA-binding domain^73-118^E:5.6e-07	1	1,321	0,003	3,777	0	3,98	0	4,333	1	0,975	ODO1_PETHY	Protein ODORANT1 (MYB-like protein ODO1)	ODO1	Petunia hybrida (Petunia)		Activator; DNA-binding; Nucleus; Repeat; Transcription; Transcription regulation
SRR342022_TR30799_c2_g2_i7_len=1663	AB25B_ARATH	Q:572-345,H:643-718	86.84%ID	E:9e-35	ABC transporter B family member 25, mitochondrial	PF06232.8^ATS3^Embryo-specific protein 3, (ATS3)^30-132^E:1.5e-08`PF01477.20^PLAT^PLAT/LH2 domain^36-153^E:2e-13	0,76	0,786	0,003	1,913	0	7,453	0	6,882	1	0,298	AB25B_ARATH	ABC transporter B family member 25, mitochondrial (ABC transporter ABCB.25) (AtABCB25) (ABC transporter of the mitochondrion 3) (AtATM3) (Iron-sulfur clusters transporter ATM3) (Protein STARIK 1)	ABCB25 ATM3 STA1 At5g58270 MCK7.14	Arabidopsis thaliana (Mouse-ear cress)	PATHWAY: Cofactor biosynthesis; molybdopterin biosynthesis.	ATP-binding; Complete proteome; Ion transport; Iron; Iron transport; Membrane; Mitochondrion; Mitochondrion inner membrane; Nucleotide-binding; Reference proteome; Transit peptide; Transmembrane; Transmembrane helix; Transport
SRR342022_TR31131_c0_g4_i2_len=532	NA	NA	NA	NA	NA	.	0,023	1,109	0,865	0,186	0	1,77	0	1,719	1	0,016	.	.	.	.	.	.
SRR342022_TR32499_c1_g1_i4_len=1644	ACCH1_ARATH	Q:311-1363,H:20-365	48.01%ID	E:3e-112	1-aminocyclopropane-1-carboxylate oxidase homolog 1	PF14226.3^DIOX_N^non-haem dioxygenase in morphine synthesis N-terminal^113-214^E:1.7e-18`PF03171.17^2OG-FeII_Oxy^2OG-Fe(II) oxygenase superfamily^273-365^E:6.4e-22	0,044	0,514	0,226	0,301	0	2,655	0	2,997	0,104	0,83	ACCH1_ARATH	1-aminocyclopropane-1-carboxylate oxidase homolog 1	At1g06620 F12K11.24 F12K11.6	Arabidopsis thaliana (Mouse-ear cress)		Complete proteome; Iron; Metal-binding; Oxidoreductase; Reference proteome
SRR342022_TR32635_c2_g1_i8_len=1404	AGL19_ARATH	Q:310-930,H:1-207	56.02%ID	E:3e-68	Agamous-like MADS-box protein AGL19	PF00319.15^SRF-TF^SRF-type transcription factor (DNA-binding and dimerisation domain)^10-57^E:8.9e-27`PF01486.14^K-box^K-box region^90-174^E:6.2e-26	0,237	0,504	0,077	0,691	0	1,457	0	1,833	1	-0,088	AGL19_ARATH	Agamous-like MADS-box protein AGL19	AGL19 At4g22950 F7H19.130	Arabidopsis thaliana (Mouse-ear cress)		Complete proteome; DNA-binding; Developmental protein; Flowering; Nucleus; Reference proteome; Transcription; Transcription regulation
SRR342023_TR1188_c3_g15_i1_len=745	TRXM4_ARATH	Q:401-42,H:70-191	59.84%ID	E:7e-47	Thioredoxin M4, chloroplastic	PF00085.17^Thioredoxin^Thioredoxin^82-181^E:2.2e-28	0,003	0,966	0,25	-0,421	0	2,875	0	3,253	1	0	TRXM4_ARATH	Thioredoxin M4, chloroplastic (AtTrxm4)	At3g15360 MJK13.2	Arabidopsis thaliana (Mouse-ear cress)		Chloroplast; Complete proteome; Disulfide bond; Electron transport; Plastid; Redox-active center; Reference proteome; Transit peptide; Transport
SRR342023_TR2829_c0_g4_i2_len=1353	PSA2A_ARATH	Q:275-979,H:1-235	94.47%ID	E:4e-159	Proteasome subunit alpha type-2-A	PF10584.6^Proteasome_A_N^Proteasome subunit A N-terminal signature^6-28^E:1.1e-08`PF00227.23^Proteasome^Proteasome subunit^31-213^E:1.7e-62	0,963	-0,023	0,034	0,676	0,001	1,244	0,001	1,229	0,927	-0,035	PSA2A_ARATH	Proteasome subunit alpha type-2-A (EC 3.4.25.1) (20S proteasome alpha subunit B-1) (Proteasome component 3)	PAB1 PRC3 At1g16470 F3O9.27	Arabidopsis thaliana (Mouse-ear cress)		Alternative splicing; Complete proteome; Cytoplasm; Hydrolase; Nucleus; Protease; Proteasome; Reference proteome; Threonine protease
SRR342023_TR3321_c1_g2_i1_len=1008	CYP22_ARATH	Q:297-833,H:21-199	86.59%ID	E:1e-111	Peptidyl-prolyl cis-trans isomerase CYP22	PF00160.18^Pro_isomerase^Cyclophilin type peptidyl-prolyl cis-trans isomerase/CLD^30-191^E:9.2e-48	0,558	-0,233	0,897	-0,141	0	7,786	0	7,546	0,728	0,15	CYP22_ARATH	Peptidyl-prolyl cis-trans isomerase CYP22 (PPIase CYP22) (EC 5.2.1.8) (Cyclophilin of 22 kDa) (Cyclophilin-22)	CYP22 CYP22-1 At2g38730 T6A23.7	Arabidopsis thaliana (Mouse-ear cress)		Chaperone; Complete proteome; Cyclosporin; Isomerase; Reference proteome; Rotamase
SRR342023_TR3389_c0_g1_i3_len=762	NA	NA	NA	NA	NA	.	0,692	1,381	1	0,937	0	10,604	0	6,002	0,502	-1,828	.	.	.	.	.	.
SRR342023_TR3965_c0_g1_i2_len=2802	SUF4_ARATH	Q:1574-462,H:1-367	62.66%ID	E:5e-128	Protein SUPPRESSOR OF FRI 4	.	0,675	0,317	0,094	1,152	0	1,685	0	1,879	1	-0,684	SUF4_ARATH	Protein SUPPRESSOR OF FRI 4	SUF4 At1g30970 F17F8.14	Arabidopsis thaliana (Mouse-ear cress)		Alternative splicing; Complete proteome; DNA-binding; Developmental protein; Differentiation; Flowering; Metal-binding; Nucleus; Reference proteome; Zinc; Zinc-

		567																		cress)		finger
SRR342023_TR3970_c2_g2_i1_len=2037	C81F1_ARATH	Q:316-1656,H:38-495	44.16%ID	E:9e-132	Cytochrome P450 81F1 {ECO:0000305}	PF00067.19^p450^Cytochrome P450^37-481^E:2.5e-92	0,93	-0,089	0,019	0,83	0	2,093	0	3,433	0,008	1,156	C81F1_ARATH	Cytochrome P450 81F1 (EC 1.14.-.-)	CYP81F1 CYP91A2 At4g37430 F6G17.80	Arabidopsis thaliana (Mouse-ear cress)		Complete proteome; Heme; Iron; Membrane; Metal-binding; Monooxygenase; Oxidoreductase; Reference proteome; Transmembrane; Transmembrane helix
SRR342023_TR4400_c0_g3_i2_len=2180	LACS7_ARATH	Q:2180-390,H:101-697	79.06%ID	E:0	Long chain acyl-CoA synthetase 7, peroxisomal	PF00501.25^AMP-binding^AMP-binding enzyme^14-455^E:8.4e-109	0,684	0,212	0,05	0,711	0	2,003	0,001	1,917	0,594	-0,258	LACS7_ARATH	Long chain acyl-CoA synthetase 7, peroxisomal (EC 6.2.1.3)	LACS7 At5g27600 F15A18.60	Arabidopsis thaliana (Mouse-ear cress)	PATHWAY: Lipid metabolism; fatty acid metabolism.	ATP-binding; Complete proteome; Fatty acid metabolism; Ligase; Lipid metabolism; Magnesium; Nucleotide-binding; Peroxisome; Reference proteome
SRR342023_TR6699_c0_g1_i2_len=1346	RP45C_ARATH	Q:3-179,H:301-359	77.97%ID	E:1e-23	Polyadenylate-binding protein RBP45C	PF00076.19^RRM_1^RNA recognition motif. (a.k.a. RRM, RBD, or RNP domain)^1-44^E:1.1e-08`PF13893.3^RRM_5^RNA recognition motif. (a.k.a. RRM, RBD, or RNP domain)^1-47^E:0.0057	0,257	0,849	0,748	0,193	0	1,524	0,001	1,471	0,214	-0,79	RP45C_ARATH	Polyadenylate-binding protein RBP45C (Poly(A)-binding protein RBP45C) (RNA-binding protein 45C) (AtRBP45C)	RBP45C At4g27000 F10M23.340	Arabidopsis thaliana (Mouse-ear cress)		Complete proteome; Nucleus; RNA-binding; Reference proteome; Repeat; mRNA processing
SRR342023_TR6857_c1_g4_i4_len=919	HSP13_SOYBN	Q:741-274,H:1-153	80.77%ID	E:1e-85	17.5 kDa class I heat shock protein	PF04969.13^CS^CS domain^50-141^E:0.022`PF00011.18^HSP20^Hsp20/alpha crystallin family^52-155^E:1.4e-32	0,545	0,462	0,121	0,78	0	2,661	0	2,176	0,44	0,648	HSP13_SOYBN	17.5 kDa class I heat shock protein (HSP 17.5-M)	HSP17.5-M	Glycine max (Soybean) (Glycine hispida)		Complete proteome; Cytoplasm; Reference proteome; Stress response
SRR342023_TR7599_c1_g2_i3_len=1661	Y5133_ARATH	Q:1-1158,H:69-456	62.47%ID	E:7e-170	BTB/POZ domain-containing protein At5g41330	.	0,611	0,347	0,124	0,936	0	2,353	0	2,19	0,635	0,325	Y5133_ARATH	BTB/POZ domain-containing protein At5g41330	At5g41330 MYC6.4	Arabidopsis thaliana (Mouse-ear cress)	PATHWAY: Protein modification; protein ubiquitination.	Complete proteome; Reference proteome; Repeat; Ubl conjugation pathway; WD repeat
SRR342023_TR8361_c1_g2_i1_len=2988	NA	NA	NA	NA	NA	PF09405.7^Btz^CASC3/Barentsz eIF4AIII binding^142-249^E:2e-20	1	0,102	0,177	-0,73	0	5,995	0	5,936	1	-0,55	.	.	.	.	.	.
SRR342023_TR8667_c0_g1_i1_len=1115	EDR2L_ARATH	Q:880-176,H:489-711	44.68%ID	E:1e-50	Protein ENHANCED DISEASE RESISTANCE 2-like	PF07059.9^DUF1336^Protein of unknown function (DUF1336)^93-308^E:1.2e-68	0,581	0,364	0,002	1,704	0	2,077	0	2,18	1	0,009	EDR2L_ARATH	Protein ENHANCED DISEASE RESISTANCE 2-like	EDR2L At5g45560 MFC19.23	Arabidopsis thaliana (Mouse-ear cress)		Cell membrane; Complete proteome; Endoplasmic reticulum; Endosome; Membrane; Plant defense; Reference proteome; Transmembrane; Transmembrane helix
SRR342023_TR10547_c3_g1_i1_len=1517	PTR27_ARATH	Q:1517-429,H:196-555	78.79%ID	E:7e-168	Protein NRT1/ PTR FAMILY 6.2	PF00854.18^PTR2^POT family^2-328^E:3.2e-75	0,732	0,795	0,017	1,256	0	1,587	0	1,142	0,748	-0,299	PTR27_ARATH	Protein NRT1/ PTR FAMILY 6.2 (AtNPF6.2) (Nitrate transporter 1.4)	NPF6.2 NRT1.4 NTP2 At2g26690 F18A8.6	Arabidopsis thaliana (Mouse-ear cress)		Complete proteome; Membrane; Nitrate assimilation; Reference proteome; Symport; Transmembrane; Transmembrane helix; Transport
SRR342023_TR10607_c1_g1_i1_len=619	RS273_ARATH	Q:502-251,H:1-84	95.24%ID	E:2e-52	40S ribosomal protein S27-3	.	1	0,048	0,007	-2,2	0	2,471	0	3,552	0,502	-3,798	RS273_ARATH	40S ribosomal protein S27-3	RPS27D ARS27B At5g47930 K16F13.1	Arabidopsis thaliana (Mouse-ear cress)		Complete proteome; Metal-binding; Reference proteome; Ribonucleoprotein; Ribosomal protein; Zinc; Zinc-finger
SRR342023_TR12080_c0_g1_i1_len=1012	AB7I_ARATH	Q:62-883,H:201-475	61.09%ID	E:3e-121	Protein ABCI7, chloroplastic	PF01458.14^UPF0051^Uncharacterized protein family (UPF0051)^4-229^E:3.4e-60	0,078	0,545	0,601	0,16	0	1,272	0,001	1,005	0,978	0,015	AB7I_ARATH	Protein ABCI7, chloroplastic (ABC transporter I family member 7) (ABC transporter ABCI.7) (AtABCI7) (Non-intrinsic ABC protein 6) (Plastid SufD-like protein)	ABCI7 NAP6 At1g32500 F5D14.28	Arabidopsis thaliana (Mouse-ear cress)		Chloroplast; Complete proteome; Plastid; Reference proteome; Transit peptide
SRR342023_TR19508_c3_g10_i1_len=931	NA	NA	NA	NA	NA	.	0,034	5,518	1	-0,065	0	7,363	0,001	6,582	0,381	2,011	.	.	.	.	.	.
SRR342023_TR20611_c2_g3_i1_len=552	CHID_SOLLC	Q:157-552,H:4-135	73.48%ID	E:3e-59	Basic endochitinase	PF00182.16^Glyco_hydro_19^Chitinase class I^25-152^E:1.3e-66	1	0	1	0	0	7,973	0	6,953	0,103	1,588	CHID_SOLLC	Basic endochitinase (EC 3.2.1.14) (Fragment)	CHI14	Solanum lycopersicum (Tomato) (Lycopersicon esculentum)		Carbohydrate metabolism; Chitin degradation; Complete proteome; Disulfide bond; Glycosidase; Hydrolase; Plant defense; Polysaccharide degradation; Reference proteome; Secreted
SRR342023_TR24545_c0_g1_i2_len=906	ISAM1_ARATH	Q:203-589,H:1-132	75%ID	E:4e-65	Iron-sulfur assembly protein IscA-like 1, mitochondrial	PF01521.17^Fe-S_biosyn^Iron-sulphur cluster biosynthesis^20-119^E:8.5e-20	0,513	0,207	0,359	0,323	0	1,232	0	1,179	0,424	0,316	ISAM1_ARATH	Iron-sulfur assembly protein IscA-like 1, mitochondrial	At2g16710 T24I21.12	Arabidopsis thaliana (Mouse-ear cress)		Alternative splicing; Complete proteome; Iron; Iron-sulfur; Metal-binding; Mitochondrion; Reference proteome; Transit peptide
SRR342023_TR26043_c3_g3_i3_len=1890	SKIP5_ARATH	Q:332-565,H:53-130	78.21%ID	E:4e-34	F-box protein SKIP5	.	1	-1,58	0,175	-0,96	0	8,619	0	9,318	1	0,26	SKIP5_ARATH	F-box protein SKIP5 (SKP1-interacting partner 5)	SKIP5 At3g54480 T14E10.50	Arabidopsis thaliana (Mouse-ear cress)	PATHWAY: Protein modification; protein ubiquitination.	Alternative splicing; Complete proteome; Reference proteome; Ubl conjugation pathway
SRR342023_TR26203_c0_g2_i2_len=2408	PTR18_ARATH	Q:518-2236,H:12-590	74.18%ID	E:0	Protein NRT1/ PTR FAMILY 3.1	PF00854.18^PTR2^POT family^88-512^E:1.1e-101	0,064	0,984	0,034	0,998	0	1,967	0	1,624	0,281	-0,564	PTR18_ARATH	Protein NRT1/ PTR FAMILY 3.1 (AtNPF3.1)	NPF3.1 NITR At1g68570 F24J5.19	Arabidopsis thaliana (Mouse-ear cress)		Complete proteome; Membrane; Nitrate assimilation; Reference proteome; Transmembrane; Transmembrane helix; Transport
SRR342023_TR27607_c1_g7_i1_len=2563	ACOX3_ARATH	Q:431-2338,H:41-675	75.47%ID	E:0	Acyl-coenzyme A oxidase 3, peroxisomal	PF02770.16^Acyl-CoA_dh_M^Acyl-CoA dehydrogenase, middle domain^192-300^E:8.7e-14`PF00441.21^Acyl-CoA_dh_1^Acyl-CoA dehydrogenase, C-terminal domain^334-489^E:1.3e-08`PF01756.16^ACOX^Acyl-CoA oxidase^539-666^E:1.3e-17	0,415	0,38	0,021	1,053	0	1,884	0	1,959	0,626	0,297	ACOX3_ARATH	Acyl-coenzyme A oxidase 3, peroxisomal (AOX 3) (Acyl-CoA oxidase 3) (EC 1.3.3.6) (Medium-chain acyl-CoA oxidase) (AtCX3)	ACX3 At1g06290 At1g06300 F9P14.15 F9P14_11 T2D23.1	Arabidopsis thaliana (Mouse-ear cress)	PATHWAY: Lipid metabolism; peroxisomal fatty acid beta-oxidation.	Complete proteome; FAD; Fatty acid metabolism; Flavoprotein; Lipid metabolism; Oxidoreductase; Peroxisome; Reference proteome; Transit peptide
SRR646572_TR2358_c0_g1_i5_len=1447	NA	NA	NA	NA	NA	PF06200.11^tify^tify domain^88-119^E:1.2e-16`PF09425.7^CCT_2^Divergent CCT motif^226-239^E:1.2e-05	0,052	1,488	0,034	5,697	0	3,166	0	3,68	0,335	0,661	.	.	.	.	.	.
SRR646572_TR6469_c0_g2_i13_len=1024	SAC8_ARATH	Q:158-454,H:76-174	49.49%ID	E:4e-21	Phosphoinositide phosphatase SAC8	.	0,221	0,418	0,005	0,921	0	1,06	0	1,058	1	-0,025	SAC8_ARATH	Phosphoinositide phosphatase SAC8 (AtSAC8) (EC 3.1.3.-) (Protein SUPPRESSOR OF ACTIN 1A) (AtSAC1a) (Protein SUPPRESSOR OF ACTIN 8) (Putative transmembrane protein G5p) (AtG5) (SAC domain protein 8) (SAC1-like protein AtSAC1a)	SAC8 G5 SAC1A At3g51830 ATEM1.8	Arabidopsis thaliana (Mouse-ear cress)		Alternative splicing; Complete proteome; Endoplasmic reticulum; Hydrolase; Membrane; Reference proteome; Transmembrane; Transmembrane helix
SRR646572_TR11019_c0_g2_i1_len=792	NA	NA	NA	NA	NA	.	0,679	0,178	0,008	0,975	0	2,307	0	2,539	0,487	0,262	.	.	.	.	.	.
SRR646572_TR13497_c0_g1_i1_len=1218	IN21B_ORYSJ	Q:785-153,H:29-241	68.08%ID	E:9e-99	Protein IN2-1 homolog B	PF13417.3^GST_N_3^Glutathione S-transferase, N-terminal domain^111-188^E:1.6e-20`PF13409.3^GST_N_2^Glutathione S-transferase, N-terminal domain^116-182^E:3.6e-13`PF16865.2^GST_C_5^Glutathione S-transferase, C-terminal domain^199-285^E:3.7e-06`PF00043.22^GST_C^Glutathione S-transferase, C-terminal domain^224-292^E:8.4e-05`PF13410.3^GST_C_2^Glutathione S-transferase, C-terminal domain^226-288^E:2e-10	0,494	0,287	0,021	0,837	0	1,7	0	1,421	0,608	-0,216	IN21B_ORYSJ	Protein IN2-1 homolog B (Glutathione S-transferase GSTZ5)	GSTZ5 Os03g0283100 LOC_Os03g17470	Oryza sativa subsp. japonica (Rice)		Complete proteome; Reference proteome
SRR646572_TR20127_c0_g1_i2_len=532	TCP10_ARATH	Q:165-386,H:14-87	91.89%ID	E:1e-41	Transcription factor TCP10	PF03634.10^TCP^TCP family transcription factor^68-172^E:3.7e-34	0,017	5,757	0,01	2,281	0	7,623	0	7,824	0,402	-1,106	TCP10_ARATH	Transcription factor TCP10	TCP10 At2g31070 T16B12.12	Arabidopsis thaliana (Mouse-ear cress)		Complete proteome; DNA-binding; Developmental protein; Nucleus; Reference proteome; Transcription; Transcription regulation
SRR646572_TR24001_c1_g1_i2_len=3205	SLK2_ARATH	Q:2012-1077,H:285-593	74.04%ID	E:4e-164	Probable transcriptional regulator SLK2	PF01803.13^LIM_bind^LIM-domain binding protein^291-551^E:9.6e-59	0,196	0,814	0,027	2,129	0	1,912	0	2,109	1	0,699	SLK2_ARATH	Probable transcriptional regulator SLK2 (AtSLK2) (Protein SEUSS-like 2)	SLK2 At5g62090 MTG10.12	Arabidopsis thaliana (Mouse-ear cress)		Complete proteome; Developmental protein; Differentiation; Flowering; Nucleus; Reference proteome; Transcription; Transcription regulation
SRR646572_TR30597_c0_g1_i1_len=1332	NA	NA	NA	NA	NA	PF00168.27^C2^C2 domain^1-64^E:1e-13`PF08372.7^PRT_C^Plant phosphoribosyltransferase C-terminal^209-364^E:6e-81	0,356	1,134	0,028	1,525	0	2,451	0	3,087	0,282	1,129	.	.	.	.	.	.
SRR646596_TR1244_c0_g1_i6_len=3997	SYK_ARATH	Q:292-1899,H:80-615	81.34%ID	E:0	.	PF01336.22^tRNA_anti-codon^OB-fold nucleic acid binding domain^88-166^E:2.9e-13`PF00152.17^tRNA-synt_2^tRNA synthetases class II (D, K and N)^206-555^E:2.8e-82	0,781	0,508	0,212	0,966	0	3,153	0	2,942	1	0,04	SYKC_ARATH	Lysine--tRNA ligase, cytoplasmic (EC 6.1.1.6) (Lysyl-tRNA synthetase) (LysRS) (Lysyl-tRNA synthetase 1) (AtKRS-1)	At3g11710 T19F11.11	Arabidopsis thaliana (Mouse-ear cress)		ATP-binding; Acetylation; Aminoacyl-tRNA synthetase; Calcium; Coiled coil; Complete proteome; Cytoplasm; DNA-binding; Ligase; Metal-binding; Nucleotide-binding; Protein biosynthesis; Reference proteome
SRR646596_TR1359_c0_g1_i8_len=3891	GLR36_ARATH	Q:2973-265,H:17-901	60.69%ID	E:0	Glutamate receptor 3.6	PF13458.3^Peripla_BP_6^Periplasmic binding protein^43-409^E:2.3e-18`PF01094.25^ANF_receptor^Receptor family ligand binding region^56-414^E:1.7e-82`PF10613.6^Lig_chan-Glu_bd^Ligated ion channel L-glutamate- and glycine-binding site^490-576^E:4.7e-09`PF00497.17^SBP_bac_3^Bacterial extracellular solute-binding proteins, family 3^531-814^E:6.6e-20`PF00060.23^Lig_chan^Ligand-gated ion channel^595-847^E:9.7e-35	0,071	0,945	0,004	1,716	0	2,468	0	2,832	1	0,279	GLR36_ARATH	Glutamate receptor 3.6 (Ligand-gated ion channel 3.6)	GLR3.6 At3g51480 F26O13.120	Arabidopsis thaliana (Mouse-ear cress)		Complete proteome; Glycoprotein; Ion channel; Ion transport; Ligand-gated ion channel; Membrane; Receptor; Reference proteome; Signal; Transmembrane; Transmembrane helix; Transport
SRR646596_TR2684_c0_g1_i15_len=2068	NA	NA	NA	NA	NA	PF06200.11^tify^tify domain^88-111^E:5.6e-12	0,008	3,385	0,008	2,82	0,001	2,445	0	2,741	1	-0,461	.	.	.	.	.	.
SRR646596_TR11097_c0_g1_i1_len=1054	IF5A3_SOLTU	Q:809-339,H:1-157	92.99%ID	E:7e-102	Eukaryotic translation initiation factor 5A-3	PF01287.17^eIF-5a^Eukaryotic elongation factor 5A hypusine, DNA-binding OB fold^85-154^E:1.3e-27	0,895	0,173	0,077	0,781	0	2,511	0	2,2	0,52	0,489	IF5A3_SOLTU	Eukaryotic translation initiation factor 5A-3 (eIF-5A-3) (eIF-4D)	EIF5A3	Solanum tuberosum (Potato)		Complete proteome; Hypusine; Initiation factor; Protein biosynthesis; Reference proteome
SRR646596_TR11837_c0_g1_i1_len=1040	NA	NA	NA	NA	NA	.	0,059	1,641	0,008	4,157	0	7,082	0	7,591	1	1,116	.	.	.	.	.	.
SRR646596_TR18518_c0_g1_i1_len=1873	DR100_ARATH	Q:1717-782,H:26-345	36.22%ID	E:5e-52	DNA-damage-repair/toleration protein DRT100	PF08263.9^LRRNT_2^Leucine rich repeat N-terminal domain^56-95^E:2e-10`PF13855.3^LRR_8^Leucine rich repeat^203-262^E:8.2e-11`PF00560.30^LRR_1^Leucine Rich Repeat^227-246^E:0.32`PF13855.3^LRR_8^Leucine rich repeat^277-335^E:4.8e-09`PF13516.3^LRR_6^Leucine Rich repeat^299-313^E:1.8`PF08263.9^LRRNT_2^Leucine rich repeat N-terminal domain^355-369^E:960	0,727	0,114	0,054	0,64	0	1,779	0	1,482	0,214	-0,386	DR100_ARATH	DNA-damage-repair/toleration protein DRT100	DRT100 At3g12610 MMF12.5 T2E22.8 T2E22_107	Arabidopsis thaliana (Mouse-ear cress)		Complete proteome; DNA damage; DNA repair; Leucine-rich repeat; Reference proteome; Repeat; Signal
SRR646596_TR19575_c0_g1_i1_len=913	HS22C_PETHY	Q:85-744,H:1-227	62.77%ID	E:1e-87	Small heat shock protein, chloroplastic	PF00011.18^HSP20^Hsp20/alpha crystallin family^153-251^E:1.2e-28	0,629	1,094	1	0,34	0	4,984	0,001	2,915	1	-0,333	HS22C_PETHY	Small heat shock protein, chloroplastic	HSP22	Petunia hybrida (Petunia)		Chloroplast; Plastid; Stress response; Transit peptide
SRR646596_TR20016_c1_g1_i1_len=7314	SDP1_ARATH	Q:1030-3477,H:1-784	72.31%ID	E:0	Triacylglycerol lipase SDP1	PF11815.5^DUF3336^Domain of unknown function (DUF3336)^99-229^E:6.9e-29`PF01734.19^Patatin^Patatin-like phospholipase^236-430^E:3.4e-18	1	0,706	0,629	1,74	0	4,919	0	5,137	1	0,331	SDP1_ARATH	Triacylglycerol lipase SDP1 (EC 3.1.1.3) (Protein SUGAR-DEPENDENT 1)	SDP1 At5g04040 F8F6.10 F8F6.250	Arabidopsis thaliana (Mouse-ear cress)		Complete proteome; Glycerol metabolism; Glycoprotein; Hydrolase; Lipid degradation; Lipid droplet; Lipid metabolism; Membrane; Reference proteome; Transmembrane; Transmembrane helix
SRR646596_TR21231_c0_g1_i1_len=545	NA	NA	NA	NA	NA	.	0,949	0,042	0,057	0,571	0	2,2	0	1,754	0,061	-0,729	.	.	.	.	.	.
SRR646596_TR22122_c0_g1_i3_len=1945	DCAM_CATRO	Q:1437-367,H:1-357	100%ID	E:0	S-adenosylmethionine decarboxylase proenzyme	PF01536.13^SAM_decarbox^Adenosylmethionine decarboxylase^9-332^E:9.8e-110	0,352	0,468	0,014	1,126	0	1,371	0	1,462	1	-0,264	DCAM_CATRO	S-adenosylmethionine decarboxylase proenzyme (AdoMetDC) (SAMDC) (EC 4.1.1.50) [Cleaved into: S-adenosylmethionine decarboxylase alpha chain; S-adenosylmethionine decarboxylase beta chain]	SAMDC	Catharanthus roseus (Madagascar periwinkle) (Vinca rosea)	PATHWAY: Amine and polyamine biosynthesis; S-adenosylmethioninamine biosynthesis; S-adenosylmethioninamine from S-adenosyl-L-methionine: step 1/1.	Autocatalytic cleavage; Decarboxylase; Lyase; Polyamine biosynthesis; Pyruvate; S-adenosyl-L-methionine; Schiff base; Spermidine biosynthesis; Zymogen
SRR646596_TR24381_c0_g1_i1_len=1227	MSRB3_ORYSJ	Q:644-1033,H:98-227	86.92%ID	E:3e-77	Peptide methionine sulfoxide reductase B3, chloroplastic	PF01641.15^SelR^SelR domain^74-192^E:1.7e-47	1	0	0,034	5,532	0	7,497	0	8,71	1	-3,497	MSRB3_ORYSJ	Peptide methionine sulfoxide reductase B3, chloroplastic (OsMSRB3) (EC 1.8.4.12) (Peptide-methionine (R)-S-oxide reductase)	MSRB3 Os05g0404200 LOC_Os05g33510 OSJNBb0006J12.16	Oryza sativa subsp. japonica (Rice)		Chloroplast; Complete proteome; Disulfide bond; Electron transport; Metal-binding; Oxidoreductase; Plastid; Redox-active center; Reference proteome; Transit peptide; Transport; Zinc
SRR646596_TR25673_c0_g1_i1_len=665	NA	NA	NA	NA	NA	.	0,006	1,747	0,282	1,057	0	4,239	0	4,333	0,319	1,107	.	.	.	.	.	.
SRR646596_TR25676_c0_g1_i1_len=689	PRS1_SOLTU	Q:124-567,H:1-155	35.9%ID	E:9e-22	Pathogenesis-related protein STH-21	PF00407.16^Bet_v_1^Pathogenesis-related protein Bet v I family^4-142^E:1.1e-12	0,629	2,416	0,068	1,061	0	3,508	0	2,519	0,083	-0,744	PRS1_SOLTU	Pathogenesis-related protein STH-21	STH-21	Solanum tuberosum (Potato)		Complete proteome; Pathogenesis-related protein; Plant defense; Reference proteome
SRR646604_TR1107_c0_g1_i1_len=4751	PR5_ARATH	Q:1058-366,H:7-239	55.27%ID	E:2e-63	Pathogenesis-related protein 5	PF01764.22^Lipase_3^Lipase (class 3)^412-548^E:4.6e-26	1	-1,67	0,034	4,734	0	6,245	0	6,978	1	1,614	PR5_ARATH	Pathogenesis-related protein 5 (PR-5)	At1g75040 F9E10.11	Arabidopsis thaliana (Mouse-ear cress)		Apoplast; Complete proteome; Direct protein sequencing; Disulfide bond; Pathogenesis-related protein; Plant defense; Reference proteome; Secreted; Signal
SRR646604_TR1303_c1_g1_i2_len=1834	TT12_ARATH	Q:1699-197,H:5-501	48.21%ID	E:3e-136	.	PF01554.15^MatE^MatE^64-224^E:2e-33`PF01554.15^MatE^MatE^286-447^E:8.6e-30	1	3,261	0,009	4,322	0	4,299	0	3,586	1	0,451	DTX41_ARATH	Protein DETOXIFICATION 41 (AtDTX41) (Multidrug and toxic compound extrusion protein 41) (MATE protein 41) (Protein TANNIN-DEFICIENT SEED 3) (Protein TRANSPARENT TESTA 12)	DTX41 TDS3 TT12 At3g59030 F17J16_80	Arabidopsis thaliana (Mouse-ear cress)	PATHWAY: Secondary metabolite biosynthesis; flavonoid biosynthesis.	Antiport; Complete proteome; Flavonoid biosynthesis; Membrane; Reference proteome; Transmembrane; Transmembrane helix; Transport; Vacuole
SRR646604_TR1685_c0_g1_i1_len=2325	NA	NA	NA	NA	NA	.	1	-0,295	0,034	4,098	0	5,734	0	6,989	1	3,377	.	.	.	.	.	.
SRR646604_TR1973_c0_g2_i2_len=2046	VPE_CITSI	Q:1910-444,H:1-494	73.95%ID	E:0	Vacuolar-processing enzyme	PF01650.15^Peptidase_C13^Peptidase C13 family^54-324^E:7.1e-116	0,037	0,567	0,366	-0,282	0	1,234	0	1,072	1	2,896	VPE_CITSI	Vacuolar-processing enzyme (VPE) (EC 3.4.22.-)		Citrus sinensis (Sweet orange) (Citrus aurantium var. sinensis)		Glycoprotein; Hydrolase; Protease; Signal; Thiol protease
SRR646604_TR3235_c0_g1_i1_len=1234	RFS6_ARATH	Q:1178-300,H:261-549	83.28%ID	E:3e-177	Probable galactinol--sucrose galactosyltransferase 6	PF05691.9^Raffinose_syn^Raffinose synthase or seed imbibition protein Sip1^8-311^E:8.4e-148	1	0,139	0,044	2,055	0	1,987	0	2,024	0,811	0,273	RFS6_ARATH	Probable galactinol--sucrose galactosyltransferase 6 (EC 2.4.1.82) (Protein DARK INDUCIBLE 10) (Raffinose synthase 6)	RFS6 DIN10 RS6 At5g20250 F5O24.140	Arabidopsis thaliana (Mouse-ear cress)		Alternative splicing; Carbohydrate metabolism; Complete proteome; Glycosyltransferase; Reference proteome; Transferase
SRR646604_TR7114_c0_g1_i1_len=900	NA	NA	NA	NA	NA	PF13639.3^zf-RING_2^Ring finger domain^119-162^E:6.7e-12`PF17123.2^zf-RING_11^RING-like zinc finger^119-148^E:1.3e-07	1	0,351	0,005	5,028	0	9,099	0	10,06	1	0	.	.	.	.	.	.
SRR646604_TR7319_c0_g1_i6_len=3664	NA	NA	NA	NA	NA	PF00106.22^adh_short^short chain dehydrogenase^6-100^E:4.2e-07`PF13561.3^adh_short_C2^Enoyl-(Acyl carrier protein) reductase^17-99^E:3.5e-07	1	0,915	0,002	5,571	0	6,979	0	7,11	1	-0,302	.	.	.	.	.	.
SRR646604_TR8057_c0_g1_i34_len=5115	LOXC1_ORYSJ	Q:469-1821,H:119-583	53.76%ID	E:0	Lipoxygenase 7, chloroplastic	PF01477.20^PLAT^PLAT/LH2 domain^144-212^E:3.3e-07`PF00305.16^Lipoxygenase^Lipoxygenase^225-569^E:1.3e-138	0,732	0,798	0,001	3,361	0	7,084	0	6,886	1	-0,489	LOXC1_ORYSJ	Lipoxygenase 7, chloroplastic (EC 1.13.11.12)	CM-LOX1 LOX2.1 Os08g0508800 LOC_Os08g39840 B1168A08.24	Oryza sativa subsp. japonica (Rice)	PATHWAY: Lipid metabolism; oxylipin biosynthesis. {ECO:0000255|PROSITE-ProRule:PRU00726}.	Chloroplast; Complete proteome; Dioxygenase; Fatty acid biosynthesis; Fatty acid metabolism; Iron; Lipid biosynthesis; Lipid metabolism; Metal-binding; Oxidoreductase; Oxylipin biosynthesis; Plastid; Reference proteome; Transit peptide
SRR646604_TR11161_c0_g1_i1_len=2489	PLT5_ARATH	Q:219-1709,H:26-525	69.86%ID	E:0	Polyol transporter 5	PF00083.21^Sugar_tr^Sugar (and other) transporter^36-493^E:3.3e-109`PF07690.13^MFS_1^Major Facilitator Superfamily^41-431^E:1.8e-28	0,118	1,706	0,001	4,77	0	2,077	0	2,014	1	-0,69	PLT5_ARATH	Polyol transporter 5 (Protein POLYOL TRANSPORTER 5) (AtPLT5) (Sugar-proton symporter PLT5)	PLT5 At3g18830 MVE11.10 MVE11.22	Arabidopsis thaliana (Mouse-ear cress)		Cell membrane; Complete proteome; Membrane; Reference proteome; Sugar transport; Symport; Transmembrane; Transmembrane helix; Transport
SRR646604_TR12279_c0_g2_i1_len=655	NA	NA	NA	NA	NA	.	0,232	-0,316	0,001	-0,926	0	1,237	0,001	1,067	0,86	-0,191	.	.	.	.	.	.
SRR646604_TR12815_c1_g1_i3_len=4404	POLX_TOBAC	Q:1648-1208,H:884-1033	42.38%ID	E:6e-42	Retrovirus-related Pol polyprotein from transposon TNT 1-94	.	0,006	2,539	0,001	4,946	0	3,668	0	4,099	1	0,837	POLX_TOBAC	Retrovirus-related Pol polyprotein from transposon TNT 1-94 [Includes: Protease (EC 3.4.23.-); Reverse transcriptase (EC 2.7.7.49); Endonuclease]		Nicotiana tabacum (Common tobacco)		Aspartyl protease; Endonuclease; Hydrolase; Metal-binding; Nuclease; Nucleotidyltransferase; Protease; RNA-directed DNA polymerase; Transferase; Transposable element; Zinc; Zinc-finger
SRR646604_TR13798_c0_g2_i1_len=811	SUI1_ORYSJ	Q:560-222,H:1-115	92.17%ID	E:4e-70	Protein translation factor SUI1 homolog	PF01253.19^SUI1^Translation initiation factor SUI1^28-102^E:5.4e-26	0,04	0,467	0,02	0,537	0	1,442	0	1,329	0	-1,392	SUI1_ORYSJ	Protein translation factor SUI1 homolog (Protein GOS2) (Protein eIF1) (Translation initiation factor 1)	GOS2 Os07g0529800 LOC_Os07g34589	Oryza sativa subsp. japonica (Rice)		Complete proteome; Initiation factor; Protein biosynthesis; Reference proteome; Translation regulation
SRR646604_TR17589_c0_g1_i1_len=1951	WRKY7_ARATH	Q:1553-468,H:1-345	48.57%ID	E:1e-90	Probable WRKY transcription factor 7	PF10533.6^Plant_zn_clust^Plant zinc cluster domain^237-286^E:1.2e-17`PF03106.12^WRKY^WRKY DNA -binding domain^290-346^E:3.4e-27	1	0,035	0,707	0,36	0	1,897	0	2,749	0,254	2,35	WRKY7_ARATH	Probable WRKY transcription factor 7 (WRKY DNA-binding protein 7)	WRKY7 At4g24240 T22A6.70	Arabidopsis thaliana (Mouse-ear cress)		Complete proteome; DNA-binding; Nucleus; Reference proteome; Transcription; Transcription regulation
SRR646604_TR17596_c1_g1_i2_len=1658	PRSP1_SPIOL	Q:590-1312,H:56-302	70.97%ID	E:9e-110	Ribosome-binding factor PSRP1, chloroplastic	PF02482.16^Ribosomal_S30AE^Sigma 54 modulation protein / S30EA ribosomal protein^85-181^E:2.2e-21`PF16321.2^Ribosom_S30AE_C^Sigma 54 modulation/S30EA ribosomal protein C terminus^223-277^E:4.4e-25	0,306	-0,506	0,6	-0,338	0	1,284	0	1,208	0,041	-0,902	PRSP1_SPIOL	Ribosome-binding factor PSRP1, chloroplastic (30S ribosomal protein 1) (CS-S5) (CS5) (Plastid-specific 30S ribosomal protein 1) (PSRP-1) (Ribosomal protein 1) (S22)	PSRP1 RPS22 RPS30	Spinacia oleracea (Spinach)		Chloroplast; Direct protein sequencing; Plastid; RNA-binding; Transit peptide; rRNA-binding
SRR646604_TR20006_c1_g1_i4_len=1523	ACCH1_ARATH	Q:1235-183,H:20-365	47.44%ID	E:2e-111	1-aminocyclopropane-1-carboxylate oxidase homolog 1	PF14226.3^DIOX_N^non-haem dioxygenase in morphine synthesis N-terminal^113-214^E:1.7e-18`PF03171.17^2OG-FeII_Oxy^2OG-Fe(II) oxygenase superfamily^273-365^E:1.8e-22	0,221	0,606	0,009	1,066	0	2,878	0	2,777	1	0,691	ACCH1_ARATH	1-aminocyclopropane-1-carboxylate oxidase homolog 1	At1g06620 F12K11.24 F12K11.6	Arabidopsis thaliana (Mouse-ear cress)		Complete proteome; Iron; Metal-binding; Oxidoreductase; Reference proteome
SRR646604_TR21469_c0_g1_i2_len=1215	SFR2_ORYSJ	Q:92-934,H:342-633	71.58%ID	E:5e-149	Beta-glucosidase-like SFR2, chloroplastic	PF00232.15^Glyco_hydro_1^Glycosyl hydrolase family 1^78-226^E:1.1e-23	0,507	0,36	0,87	0,174	0	2,164	0	1,762	1	-1,614	SFR2_ORYSJ	Beta-glucosidase-like SFR2, chloroplastic (Beta-glucosidase 36) (Os11bglu36) (Protein SENSITIVE TO FREEZING 2) (OsSFR2)	SFR2 BGLU36 Os11g0683500 LOC_Os11g45710 OsJ_34793	Oryza sativa subsp. japonica (Rice)		Chloroplast; Complete proteome; Glycoprotein; Glycosidase; Hydrolase; Membrane; Plastid; Plastid outer membrane; Reference proteome
SRR646604_TR22369_c0_g2_i47_len=2247	FAD3E_TOBAC	Q:953-249,H:146-374	69.07%ID	E:4e-104	Omega-3 fatty acid desaturase, endoplasmic reticulum	PF00487.21^FA_desaturase^Fatty acid desaturase^25-149^E:2.1e-17	1	1,719	0,001	5,19	0	4,891	0	5,358	1	0,481	FAD3E_TOBAC	Omega-3 fatty acid desaturase, endoplasmic reticulum (EC 1.14.19.-)	FAD3	Nicotiana tabacum (Common tobacco)	PATHWAY: Lipid metabolism; polyunsaturated fatty acid biosynthesis.	Endoplasmic reticulum; Fatty acid biosynthesis; Fatty acid metabolism; Lipid biosynthesis; Lipid metabolism; Membrane; Oxidoreductase; Transmembrane; Transmembrane helix
SRR646604_TR26889_c0_g1_i1_len=1402	ERF92_ARATH	Q:1054-470,H:36-213	55.05%ID	E:6e-45	Ethylene-responsive transcription factor 1B	PF00847.17^AP2^AP2 domain^110-159^E:4.8e-14	1	0,083	0,025	2,757	0	6,936	0	7,357	1	0,483	ERF92_ARATH	Ethylene-responsive transcription factor 1B (AtERF1B) (Ethylene-responsive element-binding factor 1B) (EREBP-1B)	ERF1B ERF092 ERF1 At3g23240 K14B15.15	Arabidopsis thaliana (Mouse-ear cress)		Activator; Complete proteome; DNA-binding; Ethylene signaling pathway; Nucleus; Plant defense; Reference proteome; Transcription; Transcription regulation
SRR646604_TR28128_c0_g1_i3_len=947	DOX1_ARATH	Q:512-291,H:565-638	79.73%ID	E:2e-33	Alpha-dioxygenase 1	.	1	1,554	0,014	3,443	0	8,042	0	8,553	1	-1,225	DOX1_ARATH	Alpha-dioxygenase 1 (Alpha DOX1) (EC 1.14.99.-) (Fatty acid dioxygenase AlphaDOX1) (Pathogen-induced oxygenase) (Plant alpha dioxygenase 1)	DOX1 DIOX1 PADOX-1 PIOX At3g01420 T13O15.6	Arabidopsis thaliana (Mouse-ear cress)		3D-structure; Complete proteome; Dioxygenase; Fatty acid biosynthesis; Fatty acid metabolism; Glycoprotein; Hypersensitive response; Iron; Lipid biosynthesis; Lipid metabolism; Metal-binding; Oxidoreductase; Oxylipin biosynthesis; Peroxidase; Plant defense; Reference proteome; Signal
SRR646604_TR28506_c0_g1_i7_len=2388	LACE1_HUMAN	Q:1010-2026,H:118-472	41.62%ID	E:4e-75	Lactation elevated protein 1	PF03969.13^AFG1_ATPase^AFG1-like ATPase^1-260^E:2.2e-78	0,352	0,475	0,069	1,149	0	1,853	0	1,789	1	0,008	LACE1_HUMAN	Lactation elevated protein 1 (Protein AFG1 homolog)	LACE1 AFG1	Homo sapiens (Human)		ATP-binding; Complete proteome; Nucleotide-binding; Reference proteome
SRR646604_TR28750_c0_g1_i1_len=2523	LRKS4_ARATH	Q:280-2169,H:19-657	62.21%ID	E:0	L-type lectin-domain containing receptor kinase S.4	PF00139.16^Lectin_legB^Legume lectin domain^29-281^E:2.7e-78`PF00069.22^Pkinase^Protein kinase domain^350-616^E:9e-47`PF07714.14^Pkinase_Tyr^Protein tyrosine kinase^352-618^E:1.2e-44	0,258	0,939	0,118	2,338	0	2,568	0	2,852	0,517	1,054	LRKS4_ARATH	L-type lectin-domain containing receptor kinase S.4 (LecRK-S.4) (EC 2.7.11.1)	LECRKS4 At3g55550 T22E16.210	Arabidopsis thaliana (Mouse-ear cress)		ATP-binding; Cell membrane; Complete proteome; Glycoprotein; Kinase; Lectin; Membrane; Nucleotide-binding; Receptor; Reference proteome; Serine/threonine-protein kinase; Signal; Transferase; Transmembrane; Transmembrane helix
SRR646604_TR30204_c0_g1_i2_len=918	NA	NA	NA	NA	NA	PF00076.19^RRM_1^RNA recognition motif. (a.k.a. RRM, RBD, or RNP domain)^19-79^E:1.5e-06`PF13893.3^RRM_5^RNA recognition motif. (a.k.a. RRM, RBD, or RNP domain)^37-83^E:0.3	1	0	0,076	2,875	0	3,624	0	3,461	1	-1,015	.	.	.	.	.	.
SRR646604_TR30204_c0_g1_i5_len=2698	NA	NA	NA	NA	NA	PF00076.19^RRM_1^RNA recognition motif. (a.k.a. RRM, RBD, or RNP domain)^19-84^E:3e-08`PF13893.3^RRM_5^RNA recognition motif. (a.k.a. RRM, RBD, or RNP domain)^37-83^E:0.073	1	0,018	0,043	2,921	0	2,704	0	2,837	1	-0,338	.	.	.	.	.	.
SRR646604_TR30321_c0_g1_i1_len=2309	Y3028_ARATH	Q:2232-883,H:4-440	32.62%ID	E:9e-68	Uncharacterized acetyltransferase At3g50280	PF02458.12^Transferase^Transferase family^27-474^E:2.5e-121	1	-0,305	1	1,693	0	4,359	0	4,445	1	0,365	Y3028_ARATH	Uncharacterized acetyltransferase At3g50280 (EC 2.3.1.-)	At3g50280 F11C1.120	Arabidopsis thaliana (Mouse-ear cress)		Acyltransferase; Complete proteome; Reference proteome; Transferase
SRR646604_TR33327_c0_g1_i2_len=2182	CIPKC_ARATH	Q:1846-500,H:5-459	81.76%ID	E:0	CBL-interacting serine/threonine-protein kinase 12	PF00069.22^Pkinase^Protein kinase domain^46-300^E:8e-77`PF07714.14^Pkinase_Tyr^Protein tyrosine kinase^47-296^E:2.2e-40`PF14531.3^Kinase-like^Kinase-like^140-288^E:1.6e-09`PF03822.11^NAF^NAF domain^352-408^E:5.9e-24	1	-0,48	0,254	3,895	0	6,963	0	6,985	1	-0,311	CIPKC_ARATH	CBL-interacting serine/threonine-protein kinase 12 (EC 2.7.11.1) (SNF1-related kinase 3.9) (SOS2-like protein kinase PKS8)	CIPK12 PKS8 SnRK3.9 At4g18700 F28A21.110	Arabidopsis thaliana (Mouse-ear cress)		ATP-binding; Complete proteome; Kinase; Manganese; Nucleotide-binding; Phosphoprotein; Reference proteome; Serine/threonine-protein kinase; Transferase
SRR646604_TR35022_c0_g1_i1_len=3882	SSL6_ARATH	Q:247-1308,H:10-361	54.65%ID	E:7e-132	Protein STRICTOSIDINE SYNTHASE-LIKE 6 {ECO:0000303|PubMed:10777701}	PF01554.15^MatE^MatE^122-289^E:3.3e-11`PF14667.3^Polysacc_synt_C^Polysaccharide biosynthesis C-terminal domain^247-338^E:3.3e-07	0,073	1,917	0,043	2,574	0	4,027	0	3,772	0,692	-0,688	SSL6_ARATH	Protein STRICTOSIDINE SYNTHASE-LIKE 6 (AtSSL6)	SSL6 At3g51440 F26O13.80	Arabidopsis thaliana (Mouse-ear cress)		Complete proteome; Glycoprotein; Phosphoprotein; Reference proteome; Signal; Vacuole
SRR646604_TR36368_c0_g2_i1_len=2094	C76BA_SWEMU	Q:1502-147,H:46-495	51.32%ID	E:2e-152	Geraniol 8-hydroxylase	PF00067.19^p450^Cytochrome P450^38-493^E:4.7e-107	0,042	1,912	0,132	1,486	0	0,901	0	1,271	0,019	1,492	C76BA_SWEMU	Geraniol 8-hydroxylase (EC 1.14.13.152) (Cytochrome P450 76B10) (Geraniol 10-hydroxylase) (SmG10H)	CYP76B10 G10H	Swertia mussotii (Felwort)		Endoplasmic reticulum; Heme; Iron; Membrane; Metal-binding; Monooxygenase; NADP; Oxidoreductase; Transmembrane; Transmembrane helix
SRR646604_TR37182_c0_g2_i1_len=2338	NSL1_ARATH	Q:2180-357,H:5-611	64.22%ID	E:0	MACPF domain-containing protein NSL1	PF01823.16^MACPF^MAC/Perforin domain^108-324^E:2.4e-33	0,781	0,519	0,003	2,785	0,001	2,099	0	2,462	1	0,775	NSL1_ARATH	MACPF domain-containing protein NSL1 (Protein NECROTIC SPOTTED LESIONS 1) (Protein NSL1)	NSL1 At1g28380 F3M18.18	Arabidopsis thaliana (Mouse-ear cress)		Complete proteome; Hypersensitive response; Immunity; Innate immunity; Plant defense; Reference proteome
SRR646604_TR38644_c1_g1_i5_len=4811	AFS1_MALDO	Q:1298-681,H:274-478	57.77%ID	E:8e-81	(E,E)-alpha-farnesene synthase	.	1	0	0,017	5,894	0	5,651	0	6,287	1	0	AFS1_MALDO	(E,E)-alpha-farnesene synthase (MdASF1) (EC 4.2.3.46) (Prenyltransferase) (EC 2.5.1.-)	AFS1 AFAR	Malus domestica (Apple) (Pyrus malus)		Cytoplasm; Lyase; Magnesium; Manganese; Metal-binding; Potassium; Transferase
SRR648705_TR1188_c0_g2_i1_len=1919	CBDAS_CANSA	Q:183-1037,H:25-311	48.44%ID	E:2e-120	Cannabidiolic acid synthase	PF01565.20^FAD_binding_4^FAD binding domain^79-215^E:1.3e-30	0,854	0,189	0,111	1,31	0	3,584	0	3,837	1	-1,331	CBDAS_CANSA	Cannabidiolic acid synthase (EC 1.21.3.8) (CBDA synthase.)	CBDAS	Cannabis sativa (Hemp) (Marijuana)		Apoplast; Direct protein sequencing; Disulfide bond; FAD; Flavoprotein; Glycoprotein; Oxidoreductase; Secreted; Signal
SRR648705_TR1599_c0_g1_i1_len=2132	COL12_ARATH	Q:1696-419,H:1-357	42.86%ID	E:1e-95	Zinc finger protein CONSTANS-LIKE 12	PF06203.11^CCT^CCT motif^376-418^E:4.3e-16	1	0,624	0,254	3,479	0	2,153	0	2,304	1	-0,803	COL12_ARATH	Zinc finger protein CONSTANS-LIKE 12	COL12 At3g21880 MEK6.3 MEK6_2	Arabidopsis thaliana (Mouse-ear cress)		Complete proteome; Metal-binding; Nucleus; Reference proteome; Repeat; Zinc; Zinc-finger
SRR648705_TR6174_c0_g2_i5_len=1363	EF1D2_ARATH	Q:993-307,H:2-231	81.74%ID	E:8e-82	Elongation factor 1-delta 2	PF00736.16^EF1_GNE^EF-1 guanine nucleotide exchange domain^144-231^E:1.1e-33	0,014	-0,793	1	0,022	0	2,41	0	2,335	1	-0,084	EF1D2_ARATH	Elongation factor 1-delta 2 (EF-1-delta 2) (Elongation factor 1B-beta 2) (eEF-1B beta 2)	At2g18110 F8D23.11 T27K22.2	Arabidopsis thaliana (Mouse-ear cress)		Acetylation; Complete proteome; Elongation factor; Protein biosynthesis; Reference proteome
SRR648705_TR6360_c0_g2_i1_len=627	NA	NA	NA	NA	NA	PF07939.8^DUF1685^Protein of unknown function (DUF1685)^110-163^E:5.6e-27	0,196	0,785	0,195	1,272	0	7,786	0	6,709	0,029	0,925	.	.	.	.	.	.
SRR648705_TR8953_c0_g2_i1_len=2099	H33_VITVI	Q:1826-1419,H:1-136	100%ID	E:1e-86	Histone H3.3	PF02297.14^COX6B^Cytochrome oxidase c subunit VIb^123-182^E:1.1e-16	0,226	-0,455	0,043	0,701	0	1,3	0	1,351	0,684	-0,183	H33_VITVI	Histone H3.3		Vitis vinifera (Grape)		Acetylation; Chromosome; DNA-binding; Methylation; Nucleosome core; Nucleus; Phosphoprotein
SRR648705_TR13253_c0_g1_i1_len=1881	ALFC3_ARATH	Q:1593-409,H:1-391	83.54%ID	E:0	Probable fructose-bisphosphate aldolase 3, chloroplastic	PF00274.16^Glycolytic^Fructose-bisphosphate aldolase class-I^51-395^E:1.1e-158	0,884	-0,048	0,004	0,857	0	1,307	0	1,533	0,785	-0,097	ALFC3_ARATH	Probable fructose-bisphosphate aldolase 3, chloroplastic (EC 4.1.2.13)	FBA3 At2g01140 F10A8.2	Arabidopsis thaliana (Mouse-ear cress)	PATHWAY: Carbohydrate degradation; glycolysis; D-glyceraldehyde 3-phosphate and glycerone phosphate from D-glucose: step 4/4.	Chloroplast; Complete proteome; Glycolysis; Lyase; Methylation; Phosphoprotein; Plastid; Reference proteome; Schiff base; Transit peptide
SRR648705_TR13457_c0_g3_i2_len=2131	BGL46_ARATH	Q:299-1711,H:24-498	57.98%ID	E:0	Beta-glucosidase 46	PF00232.15^Glyco_hydro_1^Glycosyl hydrolase family 1^1-396^E:2.1e-113	0,844	0,14	0,193	0,605	0	1,458	0,001	1,373	0,306	0,549	BGL46_ARATH	Beta-glucosidase 46 (AtBGLU46) (EC 3.2.1.21)	BGLU46 At1g61820 F8K4.3	Arabidopsis thaliana (Mouse-ear cress)		Alternative splicing; Complete proteome; Disulfide bond; Glycoprotein; Glycosidase; Hydrolase; Reference proteome; Signal
SRR648705_TR14763_c0_g1_i1_len=2353	PTR5_ARATH	Q:2021-309,H:2-567	65.97%ID	E:0	Protein NRT1/ PTR FAMILY 8.2	PF07690.13^MFS_1^Major Facilitator Superfamily^44-512^E:5.9e-13`PF00854.18^PTR2^POT family^101-531^E:8.4e-127	0,195	1,365	0,006	2,541	0	4,348	0	4,213	0,517	-1,13	PTR5_ARATH	Protein NRT1/ PTR FAMILY 8.2 (AtNPF8.2) (Peptide transporter PTR5)	NPF8.2 PTR5 At5g01180 F7J8.160	Arabidopsis thaliana (Mouse-ear cress)		Cell membrane; Complete proteome; Membrane; Peptide transport; Phosphoprotein; Protein transport; Reference proteome; Transmembrane; Transmembrane helix; Transport
SRR648705_TR16302_c0_g1_i2_len=1327	PIP27_MAIZE	Q:1243-380,H:1-287	81.66%ID	E:5e-150	Aquaporin PIP2-7	PF00230.17^MIP^Major intrinsic protein^34-269^E:3.2e-83	1	3,491	0,034	5,494	0	7,175	0	7,054	1	0,883	PIP27_MAIZE	Aquaporin PIP2-7 (Plasma membrane intrinsic protein 2-7) (ZmPIP2-7) (ZmPIP2;7)	PIP2-7	Zea mays (Maize)		Cell membrane; Complete proteome; Membrane; Reference proteome; Repeat; Transmembrane; Transmembrane helix; Transport
SRR648705_TR18311_c1_g1_i2_len=1978	PREA_ECOLI	Q:634-1647,H:5-329	42.01%ID	E:8e-77	NAD-dependent dihydropyrimidine dehydrogenase subunit PreA	PF01180.18^DHO_dh^Dihydroorotate dehydrogenase^54-365^E:6.8e-33	0,312	0,324	0,056	0,671	0,001	1,187	0,001	1,17	0,767	0,121	PREA_ECOLI	NAD-dependent dihydropyrimidine dehydrogenase subunit PreA (DPD) (EC 1.3.1.1) (Dihydrothymine dehydrogenase) (Dihydrouracil dehydrogenase)	preA yeiA b2147 JW2134	Escherichia coli (strain K12)		4Fe-4S; Complete proteome; Direct protein sequencing; Iron; Iron-sulfur; Metal-binding; NAD; Oxidoreductase; Reference proteome; Repeat
SRR648705_TR18889_c0_g2_i1_len=1169	NA	NA	NA	NA	NA	.	1	-0,562	0,226	3,128	0	5,685	0	6,459	1	0,072	.	.	.	.	.	.
SRR648705_TR19703_c0_g1_i2_len=1636	NA	NA	NA	NA	NA	.	1	0,309	0,006	1,818	0	1,868	0	2,132	1	0,006	.	.	.	.	.	.
SRR648705_TR21740_c0_g1_i1_len=1291	DAG_ANTMA	Q:846-532,H:80-184	54.29%ID	E:4e-36	DAG protein, chloroplastic	.	0,117	-0,36	0,59	-0,124	0	1,259	0	1,211	1	-0,005	DAG_ANTMA	DAG protein, chloroplastic	DAG	Antirrhinum majus (Garden snapdragon)		Chloroplast; Plastid; Transit peptide
SRR648705_TR22910_c0_g1_i1_len=890	RLA31_ARATH	Q:715-506,H:1-69	70%ID	E:8e-24	60S acidic ribosomal protein P3-1	.	0,151	-0,514	0,719	0,122	0	1,854	0,001	1,538	0,407	-0,714	RLA31_ARATH	60S acidic ribosomal protein P3-1	RPP3A At4g25890 F14M19.170	Arabidopsis thaliana (Mouse-ear cress)		Complete proteome; Phosphoprotein; Reference proteome; Ribonucleoprotein; Ribosomal protein
SRR648705_TR24450_c0_g1_i1_len=3380	NAGS2_ARATH	Q:2947-1337,H:68-610	70.38%ID	E:0	Probable amino-acid acetyltransferase NAGS2, chloroplastic	PF00696.25^AA_kinase^Amino acid kinase family^107-302^E:5e-08`PF13673.4^Acetyltransf_10^Acetyltransferase (GNAT) domain^485-563^E:2.8e-05`PF13508.4^Acetyltransf_7^Acetyltransferase (GNAT) domain^486-563^E:2.4e-08`PF00583.22^Acetyltransf_1^Acetyltransferase (GNAT) family^490-561^E:1.6e-10	1	0,007	0,5	0,609	0	2,003	0,001	1,796	0,095	-0,859	NAGS2_ARATH	Probable amino-acid acetyltransferase NAGS2, chloroplastic (EC 2.3.1.1) (N-acetylglutamate synthase 2)	NAGS2 At4g37670 F19F18.160	Arabidopsis thaliana (Mouse-ear cress)	PATHWAY: Amino-acid biosynthesis; L-arginine biosynthesis; N(2)-acetyl-L-ornithine from L-glutamate: step 1/4.	Acyltransferase; Alternative splicing; Amino-acid biosynthesis; Arginine biosynthesis; Chloroplast; Complete proteome; Plastid; Reference proteome; Transferase; Transit peptide
SRR648705_TR30487_c0_g2_i2_len=1724	CYSP3_SOLLC	Q:1450-512,H:44-356	80.83%ID	E:0	Cysteine proteinase 3	PF00112.20^Peptidase_C1^Papain family cysteine protease^53-268^E:5.7e-79	0	-3,322	1	0,069	0	4,575	0	4,311	0,894	-0,082	CYSP3_SOLLC	Cysteine proteinase 3 (EC 3.4.22.-)	CYP-3	Solanum lycopersicum (Tomato) (Lycopersicon esculentum)		Complete proteome; Disulfide bond; Glycoprotein; Hydrolase; Protease; Reference proteome; Signal; Thiol protease; Vacuole; Zymogen
SRR648705_TR34486_c0_g4_i1_len=2929	Y2242_ARATH	Q:2038-554,H:328-848	36.52%ID	E:6e-84	Probable LRR receptor-like serine/threonine-protein kinase At2g24230	PF13855.3^LRR_8^Leucine rich repeat^55-113^E:4.5e-08`PF00069.22^Pkinase^Protein kinase domain^402-574^E:3e-14`PF07714.14^Pkinase_Tyr^Protein tyrosine kinase^403-572^E:2.7e-22	0,489	0,52	0,06	0,84	0	3,247	0	3,564	0,798	0,17	Y2242_ARATH	Probable LRR receptor-like serine/threonine-protein kinase At2g24230 (EC 2.7.11.1)	At2g24230 F27D4.14	Arabidopsis thaliana (Mouse-ear cress)		ATP-binding; Complete proteome; Glycoprotein; Kinase; Leucine-rich repeat; Membrane; Nucleotide-binding; Phosphoprotein; Receptor; Reference proteome; Repeat; Serine/threonine-protein kinase; Signal; Transferase; Transmembrane; Transmembrane helix
SRR648705_TR36981_c0_g1_i1_len=723	NA	NA	NA	NA	NA	.	0,357	0,275	0,376	-0,261	0	1,843	0	1,367	0,171	0,32	.	.	.	.	.	.
SRR648705_TR41754_c0_g1_i1_len=539	NA	NA	NA	NA	NA	.	0,687	-0,107	0,012	0,602	0	0,909	0	1,044	0	-1,056	.	.	.	.	.	.
SRR648705_TR42919_c0_g1_i2_len=1073	HS21C_SOLLC	Q:550-44,H:87-235	43.79%ID	E:3e-38	Small heat shock protein, chloroplastic	PF00011.18^HSP20^Hsp20/alpha crystallin family^144-247^E:2.9e-22	0,278	-0,795	1	-0,034	0	3,285	0	3,341	0,129	-4,971	HS21C_SOLLC	Small heat shock protein, chloroplastic	HSP21 TOM111	Solanum lycopersicum (Tomato) (Lycopersicon esculentum)		Chloroplast; Complete proteome; Plastid; Reference proteome; Stress response; Transit peptide
SRR648705_TR43500_c1_g2_i4_len=4099	EXEC1_ARATH	Q:385-2235,H:64-683	52.86%ID	E:7e-180	Protein EXECUTER 1, chloroplastic {ECO:0000305}	PF12014.5^DUF3506^Domain of unknown function (DUF3506)^538-666^E:8.6e-25	1	0,081	0,042	1,808	0	6,894	0	7,256	0,005	6,256	EXEC1_ARATH	Protein EXECUTER 1, chloroplastic (AtEX1)	EX1 At4g33630 T16L1.120	Arabidopsis thaliana (Mouse-ear cress)		Chloroplast; Complete proteome; Plastid; Reference proteome; Stress response; Transit peptide
SRR648705_TR43708_c0_g3_i5_len=615	PERR_RAUSE	Q:553-2,H:10-193	83.7%ID	E:2e-111	Perakine reductase	PF00248.18^Aldo_ket_red^Aldo/keto reductase family^7-172^E:3.1e-46	0,701	0,122	0,008	0,87	0	1,185	0	1,236	0,077	0,984	PERR_RAUSE	Perakine reductase (EC 1.1.1.317)	PR	Rauvolfia serpentina (Serpentine wood) (Ophioxylon serpentinum)		3D-structure; Alkaloid metabolism; NADP; Oxidoreductase
SRR648707_TR2562_c0_g1_i4_len=1304	UBC28_ARATH	Q:825-382,H:1-148	97.3%ID	E:2e-100	Ubiquitin-conjugating enzyme E2 28	PF00179.23^UQ_con^Ubiquitin-conjugating enzyme^5-141^E:5.6e-54	0,544	0,157	0,23	0,331	0,001	0,89	0,001	0,884	0,41	0,338	UBC28_ARATH	Ubiquitin-conjugating enzyme E2 28 (EC 2.3.2.23) (E2 ubiquitin-conjugating enzyme 28) (Ubiquitin carrier protein 28)	UBC28 UBC9A At1g64230 F22C12.2	Arabidopsis thaliana (Mouse-ear cress)	PATHWAY: Protein modification; protein ubiquitination. {ECO:0000255|PROSITE-ProRule:PRU00388}.	ATP-binding; Alternative splicing; Complete proteome; Nucleotide-binding; Reference proteome; Transferase; Ubl conjugation pathway
SRR648707_TR6045_c0_g1_i3_len=700	UBC5_ARATH	Q:645-223,H:47-185	74.47%ID	E:2e-65	Ubiquitin-conjugating enzyme E2 5	.	1	0,049	0,395	-0,555	0	6,666	0	6,686	0,822	-0,388	UBC5_ARATH	Ubiquitin-conjugating enzyme E2 5 (EC 2.3.2.23) (E2 ubiquitin-conjugating enzyme 5) (Ubiquitin carrier protein 5) (Ubiquitin-conjugating enzyme E2-21 kDa 2) (Ubiquitin-protein ligase 5)	UBC5 At1g63800 F24D7.1 T12P18.18	Arabidopsis thaliana (Mouse-ear cress)	PATHWAY: Protein modification; protein ubiquitination. {ECO:0000255|PROSITE-ProRule:PRU00388}.	ATP-binding; Complete proteome; Nucleotide-binding; Reference proteome; Transferase; Ubl conjugation pathway
SRR648707_TR7874_c0_g2_i1_len=724	NA	NA	NA	NA	NA	.	1	-0,221	0,009	5,052	0,001	5,367	0	5,891	1	0,713	.	.	.	.	.	.
SRR648707_TR11426_c0_g1_i2_len=1012	NA	NA	NA	NA	NA	PF05620.8^DUF788^Protein of unknown function (DUF788)^1-163^E:3.6e-53	0,73	0,132	0,006	0,885	0	1,629	0	1,665	0,469	-0,299	.	.	.	.	.	.
SRR648707_TR13304_c0_g2_i1_len=1087	RS17_SOLLC	Q:189-581,H:1-131	86.26%ID	E:9e-73	40S ribosomal protein S17	PF00833.15^Ribosomal_S17e^Ribosomal S17^1-116^E:2e-57	0	-1,196	0,943	-0,023	0	1,204	0,001	1,004	0,041	-0,482	RS17_SOLLC	40S ribosomal protein S17	RPS17 GI1	Solanum lycopersicum (Tomato) (Lycopersicon esculentum)		Complete proteome; Reference proteome; Ribonucleoprotein; Ribosomal protein
SRR648707_TR13620_c0_g2_i1_len=1150	YDR2_SCHPO	Q:804-280,H:4-168	40.68%ID	E:2e-25	Uncharacterized endoplasmic reticulum membrane protein C16E8.02	PF06127.8^DUF962^Protein of unknown function (DUF962)^5-162^E:7.2e-32	0,533	0,248	1	0,02	0	1,645	0,001	1,338	0,052	1,633	YDR2_SCHPO	Uncharacterized endoplasmic reticulum membrane protein C16E8.02	SPAC16E8.02	Schizosaccharomyces pombe (strain 972 / ATCC 24843) (Fission yeast)		Complete proteome; Endoplasmic reticulum; Membrane; Reference proteome; Transmembrane; Transmembrane helix
SRR648707_TR19799_c0_g1_i1_len=2332	FKB62_ARATH	Q:2106-460,H:1-550	79.71%ID	E:0	Peptidyl-prolyl cis-trans isomerase FKBP62	PF00254.25^FKBP_C^FKBP-type peptidyl-prolyl cis-trans isomerase^51-140^E:2.4e-34`PF00254.25^FKBP_C^FKBP-type peptidyl-prolyl cis-trans isomerase^166-257^E:8.5e-19`PF00254.25^FKBP_C^FKBP-type peptidyl-prolyl cis-trans isomerase^282-379^E:3.3e-20`PF14559.3^TPR_19^Tetratricopeptide repeat^459-519^E:7.8e-06`PF00515.25^TPR_1^Tetratricopeptide repeat^482-515^E:3.9e-08`PF07719.14^TPR_2^Tetratricopeptide repeat^483-515^E:4.2e-06`PF13181.3^TPR_8^Tetratricopeptide repeat^483-515^E:2.8e-06	0,181	-0,425	0,002	1,036	0	1,168	0,001	0,966	0,041	0,728	FKB62_ARATH	Peptidyl-prolyl cis-trans isomerase FKBP62 (PPIase FKBP62) (EC 5.2.1.8) (70 kDa peptidyl-prolyl isomerase) (FK506-binding protein 62) (AtFKBP62) (Immunophilin FKBP62) (Peptidylprolyl isomerase ROF1) (Protein ROTAMASE FKBP 1) (Rotamase)	FKBP62 ROF1 At3g25230 MJL12.19	Arabidopsis thaliana (Mouse-ear cress)		Acetylation; Alternative splicing; Calmodulin-binding; Chaperone; Complete proteome; Cytoplasm; Isomerase; Nucleus; Reference proteome; Repeat; Rotamase; TPR repeat
SRR648707_TR21079_c0_g1_i3_len=1039	CRPM2_ARATH	Q:171-776,H:1-203	65.52%ID	E:2e-87	Cold-regulated 413 plasma membrane protein 2	PF05562.8^WCOR413^Cold acclimation protein WCOR413^9-189^E:8.3e-85	0,019	1,077	0,001	1,377	0	2,142	0	1,905	1	0,066	CRPM2_ARATH	Cold-regulated 413 plasma membrane protein 2 (AtCOR413-PM2)	COR413PM2 At3g50830 F18B3.110	Arabidopsis thaliana (Mouse-ear cress)		Cell membrane; Complete proteome; Membrane; Reference proteome; Transmembrane; Transmembrane helix
SRR648707_TR28964_c0_g2_i1_len=828	GCSH2_ARATH	Q:115-567,H:1-152	71.71%ID	E:2e-73	Glycine cleavage system H protein 2, mitochondrial	PF01597.16^GCV_H^Glycine cleavage H-protein^69-188^E:6.1e-49`PF00364.19^Biotin_lipoyl^Biotin-requiring enzyme^107-146^E:9.1e-06	0,886	-0,143	0,02	1,169	0	3,05	0	3,44	0,233	-0,453	GCSH2_ARATH	Glycine cleavage system H protein 2, mitochondrial	GDH2 At2g35120 T4C15.21	Arabidopsis thaliana (Mouse-ear cress)		Complete proteome; Lipoyl; Mitochondrion; Phosphoprotein; Reference proteome; Transit peptide
SRR648707_TR29191_c0_g2_i1_len=767	FKB12_ARATH	Q:104-439,H:1-112	84.82%ID	E:3e-64	Peptidyl-prolyl cis-trans isomerase FKBP12	PF00254.25^FKBP_C^FKBP-type peptidyl-prolyl cis-trans isomerase^48-143^E:1.2e-26	0,712	-0,086	0,217	-0,284	0	1,403	0	1,267	0,927	-0,021	FKB12_ARATH	Peptidyl-prolyl cis-trans isomerase FKBP12 (PPIase FKBP12) (EC 5.2.1.8) (12 kDa FK506-binding protein) (12 kDa FKBP) (FK506-binding protein 12) (AtFKBP12) (FKBP-12) (Immunophilin FKBP12) (Rotamase)	FKBP12 FKP12 At5g64350 MSJ1.19	Arabidopsis thaliana (Mouse-ear cress)		Complete proteome; Cytoplasm; Disulfide bond; Isomerase; Reference proteome; Rotamase
SRR648707_TR30821_c0_g2_i1_len=1906	CBDAS_CANSA	Q:1796-264,H:25-537	47.31%ID	E:1e-148	Cannabidiolic acid synthase	PF01565.20^FAD_binding_4^FAD binding domain^2-111^E:2.6e-21`PF08031.9^BBE^Berberine and berberine like^371-428^E:2.4e-22	1	-0,029	0,025	1,391	0	4,396	0	4,817	1	0	CBDAS_CANSA	Cannabidiolic acid synthase (EC 1.21.3.8) (CBDA synthase.)	CBDAS	Cannabis sativa (Hemp) (Marijuana)		Apoplast; Direct protein sequencing; Disulfide bond; FAD; Flavoprotein; Glycoprotein; Oxidoreductase; Secreted; Signal
SRR924147_TR595_c0_g1_i2_len=670	TRL33_ORYSJ	Q:594-220,H:4-129	63.49%ID	E:2e-53	Thioredoxin-like 3-3	PF00085.17^Thioredoxin^Thioredoxin^62-142^E:1e-13	0,454	0,308	0,721	0,165	0	3,101	0	2,782	0,021	1,222	TRL33_ORYSJ	Thioredoxin-like 3-3 (Thioredoxin-like 1)	Os04g0560200 LOC_Os04g47260 OsJ_15765 OSJNBa0084K11.5	Oryza sativa subsp. japonica (Rice)		Complete proteome; Disulfide bond; Electron transport; Redox-active center; Reference proteome; Transport
SRR924147_TR3405_c0_g1_i2_len=1493	PERR_RAUSE	Q:218-1225,H:1-336	83.93%ID	E:0	Perakine reductase	PF00248.18^Aldo_ket_red^Aldo/keto reductase family^20-311^E:3.3e-77	1	0,037	0,784	0,125	0	1,668	0	1,504	0,876	0,087	PERR_RAUSE	Perakine reductase (EC 1.1.1.317)	PR	Rauvolfia serpentina (Serpentine wood) (Ophioxylon serpentinum)		3D-structure; Alkaloid metabolism; NADP; Oxidoreductase
SRR924147_TR10126_c0_g2_i1_len=1006	UBC5_ARATH	Q:209-655,H:1-149	91.95%ID	E:1e-96	Ubiquitin-conjugating enzyme E2 5	PF00179.23^UQ_con^Ubiquitin-conjugating enzyme^10-142^E:1.3e-38	0,77	0,156	0,009	1,165	0	1,687	0,001	1,597	0,37	-0,309	UBC5_ARATH	Ubiquitin-conjugating enzyme E2 5 (EC 2.3.2.23) (E2 ubiquitin-conjugating enzyme 5) (Ubiquitin carrier protein 5) (Ubiquitin-conjugating enzyme E2-21 kDa 2) (Ubiquitin-protein ligase 5)	UBC5 At1g63800 F24D7.1 T12P18.18	Arabidopsis thaliana (Mouse-ear cress)	PATHWAY: Protein modification; protein ubiquitination. {ECO:0000255|PROSITE-ProRule:PRU00388}.	ATP-binding; Complete proteome; Nucleotide-binding; Reference proteome; Transferase; Ubl conjugation pathway
SRR924147_TR15306_c0_g1_i2_len=1509	NA	NA	NA	NA	NA	PF00582.23^Usp^Universal stress protein family^5-157^E:2.5e-31	0,43	0,661	0,01	2,121	0	2,862	0	2,547	0,878	0,124	.	.	.	.	.	.
SRR924147_TR15394_c0_g1_i1_len=2081	LACE1_HUMAN	Q:1588-386,H:74-475	39.52%ID	E:2e-78	Lactation elevated protein 1	PF03969.13^AFG1_ATPase^AFG1-like ATPase^88-432^E:6.9e-94	0,454	0,388	0,151	0,685	0	2,06	0	1,685	0,5	0,658	LACE1_HUMAN	Lactation elevated protein 1 (Protein AFG1 homolog)	LACE1 AFG1	Homo sapiens (Human)		ATP-binding; Complete proteome; Nucleotide-binding; Reference proteome
SRR924147_TR20586_c0_g1_i3_len=1362	NA	NA	NA	NA	NA	PF07685.11^GATase_3^CobB/CobQ-like glutamine amidotransferase domain^51-127^E:0.00017`PF00117.25^GATase^Glutamine amidotransferase class-I^55-154^E:4.1e-07	0,094	1,168	0,035	1,411	0	2,977	0	3,105	0,545	-0,569	.	.	.	.	.	.
SRR924147_TR20586_c0_g1_i4_len=2119	NA	NA	NA	NA	NA	PF00117.25^GATase^Glutamine amidotransferase class-I^3-93^E:8.8e-07	0,059	1,859	0,593	0,581	0	3,913	0,001	3,619	0,319	-1	.	.	.	.	.	.
SRR924147_TR30062_c0_g1_i1_len=1556	GILT_HUMAN	Q:444-992,H:62-243	37.23%ID	E:6e-29	Gamma-interferon-inducible lysosomal thiol reductase	PF03227.13^GILT^Gamma interferon inducible lysosomal thiol reductase (GILT)^41-143^E:4e-29	0,644	0,162	0,746	0,128	0	1,529	0	1,226	0,506	-0,182	GILT_HUMAN	Gamma-interferon-inducible lysosomal thiol reductase (EC 1.8.-.-) (Gamma-interferon-inducible protein IP-30) (Legumaturain)	IFI30 GILT IP30	Homo sapiens (Human)		Complete proteome; Direct protein sequencing; Disulfide bond; Glycoprotein; Immunity; Lysosome; Oxidoreductase; Redox-active center; Reference proteome; Secreted; Signal
SRR924147_TR34505_c0_g5_i1_len=1405	PSA7_SOLLC	Q:229-885,H:1-219	94.98%ID	E:2e-149	Proteasome subunit alpha type-7	PF10584.6^Proteasome_A_N^Proteasome subunit A N-terminal signature^4-26^E:7.7e-14`PF00227.23^Proteasome^Proteasome subunit^27-210^E:5.7e-59	0,594	-0,153	0,467	0,221	0,001	1,105	0,001	1,117	0,596	0,156	PSA7_SOLLC	Proteasome subunit alpha type-7 (EC 3.4.25.1) (20S proteasome alpha subunit D) (20S proteasome subunit alpha-4)	PAD1 PSR5	Solanum lycopersicum (Tomato) (Lycopersicon esculentum)		Complete proteome; Cytoplasm; Hydrolase; Nucleus; Protease; Proteasome; Reference proteome; Threonine protease
SRR924147_TR36086_c0_g1_i1_len=1593	HAT4_ARATH	Q:538-1119,H:55-242	74.49%ID	E:2e-66	Homeobox-leucine zipper protein HAT4	PF04618.9^HD-ZIP_N^HD-ZIP protein N terminus^4-141^E:4.9e-20`PF00046.26^Homeobox^Homeobox domain^164-218^E:3.6e-16`PF02183.15^HALZ^Homeobox associated leucine zipper^220-254^E:5.1e-11	0,732	-0,643	0,026	2,306	0	3,82	0	3,902	0,76	-0,565	HAT4_ARATH	Homeobox-leucine zipper protein HAT4 (HD-ZIP protein ATHB-2) (Homeodomain transcription factor ATHB-2) (Homeodomain-leucine zipper protein HAT4) (HD-ZIP protein 4)	HAT4 ATHB-2 At4g16780 dl4415w FCAALL.101	Arabidopsis thaliana (Mouse-ear cress)		Complete proteome; DNA-binding; Developmental protein; Homeobox; Nucleus; Phytochrome signaling pathway; Reference proteome; Repressor; Transcription; Transcription regulation

SRR924148_TR5782_c0_g1_i1_len=1026	NA	NA	NA	NA	NA	PF02466.16^Tim17^Tim17/Tim22/Tim23/Pmp24 family^35-127^E:1e-09	0	-6,81	0,277	-0,503	0	2,002	0	1,85	1	-0,055	.	.	.	.	.	.
SRR924148_TR6159_c1_g1_i1_len=2250	CBP22_HORVU	Q:1605-466,H:54-436	69.97%ID	E:0	Serine carboxypeptidase II-2	PF00450.19^Peptidase_S10^Serine carboxypeptidase^1-242^E:1.6e-57	0,783	0,202	0,016	1,299	0	2,879	0	2,944	1	0,681	CBP22_HORVU	Serine carboxypeptidase II-2 (EC 3.4.16.6) (CP-MII.2) [Cleaved into: Serine carboxypeptidase II-2 chain A; Serine carboxypeptidase II-2 chain B] (Fragment)	CXP;2-2	Hordeum vulgare (Barley)		Carboxypeptidase; Direct protein sequencing; Disulfide bond; Glycoprotein; Hydrolase; Protease; Zymogen
SRR924148_TR6556_c0_g3_i1_len=1355	NA	NA	NA	NA	NA	PF04640.11^PLATZ^PLATZ transcription factor^113-188^E:9.1e-27	1	1,544	0,034	4,074	0	4,865	0	5,728	1	-0,144	.	.	.	.	.	.
SRR924148_TR12937_c0_g1_i1_len=1992	MASY_RICCO	Q:1910-249,H:13-567	88.65%ID	E:0	Malate synthase, glyoxysomal	PF01274.19^Malate_synthase^Malate synthase^41-571^E:1.1e-220	0,76	-0,437	0,402	1,113	0	2,784	0	3,572	0,732	-0,739	MASY_RICCO	Malate synthase, glyoxysomal (EC 2.3.3.9)		Ricinus communis (Castor bean)	PATHWAY: Carbohydrate metabolism; glyoxylate cycle; (S)-malate from isocitrate: step 2/2.	Glyoxylate bypass; Glyoxysome; Peroxisome; Transferase; Tricarboxylic acid cycle
SRR924148_TR17236_c0_g2_i3_len=1407	APX1_ARATH	Q:294-1043,H:1-250	85.6%ID	E:4e-145	L-ascorbate peroxidase 1, cytosolic	PF00141.20^peroxidase^Peroxidase^22-227^E:4.7e-49	0,986	0,007	0,005	0,638	0	1,123	0	1,015	0	-1,554	APX1_ARATH	L-ascorbate peroxidase 1, cytosolic (AP) (AtAPx01) (EC 1.11.1.11)	APX1 At1g07890 F24B9.2	Arabidopsis thaliana (Mouse-ear cress)		Alternative splicing; Calcium; Complete proteome; Cytoplasm; Direct protein sequencing; Heme; Hydrogen peroxide; Iron; Metal-binding; Oxidoreductase; Peroxidase; Phosphoprotein; Potassium; Reference proteome
SRR924148_TR20364_c1_g4_i6_len=4616	GATLA_ARATH	Q:866-1717,H:37-317	65.14%ID	E:1e-111	Probable galacturonosyltransferase-like 10	PF01501.17^Glyco_transf_8^Glycosyl transferase family 8^70-301^E:5.8e-46	1	0,434	0,004	2,671	0	2,967	0	3,488	1	-0,191	GATLA_ARATH	Probable galacturonosyltransferase-like 10 (EC 2.4.1.-) (Galactinol synthase 8) (AtGolS8) (GolS-8)	GATL10 GOLS8 At3g28340 MZF16.17	Arabidopsis thaliana (Mouse-ear cress)	PATHWAY: Glycan metabolism; pectin biosynthesis.	Cell wall biogenesis/degradation; Complete proteome; Glycoprotein; Glycosyltransferase; Golgi apparatus; Membrane; Reference proteome; Signal-anchor; Transferase; Transmembrane; Transmembrane helix
SRR924148_TR20791_c0_g2_i1_len=878	NA	NA	NA	NA	NA	.	0,941	-0,03	0,302	0,358	0	1,664	0	1,562	1	0,082	.	.	.	.	.	.
SRR924148_TR28513_c0_g1_i1_len=780	NA	NA	NA	NA	NA	.	1	0,006	0,537	0,157	0	1,066	0,001	0,901	0,383	-0,252	.	.	.	.	.	.
SRR924148_TR30094_c0_g4_i1_len=1108	NA	NA	NA	NA	NA	.	0,228	1,015	0,043	3,051	0	3,439	0	3,452	1	0,322	.	.	.	.	.	.
SRR924148_TR32919_c1_g1_i1_len=1921	WRK11_ARATH	Q:1051-1308,H:224-309	83.72%ID	E:2e-43	Probable WRKY transcription factor 11	.	0,729	0,276	0,822	0,396	0	2,048	0	2,696	0,895	0,082	WRK11_ARATH	Probable WRKY transcription factor 11 (WRKY DNA-binding protein 11)	WRKY11 At4g31550 F3L17.120	Arabidopsis thaliana (Mouse-ear cress)		Alternative splicing; Complete proteome; DNA-binding; Nucleus; Reference proteome; Transcription; Transcription regulation
SRR924148_TR35978_c0_g1_i2_len=968	NA	NA	NA	NA	NA	.	1	3,267	0,629	1,626	0	3,425	0,001	3,327	1	-0,022	.	.	.	.	.	.

MsIntact24h

Transcript (CDF97)	Uniprot homolog	BLASTX Position	BLASTX %Identity	BLASTX evalue	Uniprot descr	Pfam	PValue_Ms6h	logFC_Ms6h	PValue_Ms8h	logFC_Ms8h	PValue_MsDamaged24h	logFC_MsDamaged24h	PValue_MsIntact24h	logFC_MsIntact24h	PValue_Ms1wk	logFC_Ms1wk	Entry.name	Protein names	Gene names	Organism	Pathway	Keywords
SRR1144633_TR1121_c0_g1_i1_len=1878	PP1_ORYSJ	Q:361-1263,H:15-316	89.74%ID	E:0	Serine/threonine-protein phosphatase PP1	PF16891.2^STPPase_N^Serine-threonine protein phosphatase N-terminal domain^6-52^E:1.4e-22`PF00149.25^Metallophos^Calcineurin-like phosphoesterase^54-245^E:2e-40	0,87	0,08	0,041	0,692	0,004	1,287	0	1,577	0,931	-0,07	PP1_ORYSJ	Serine/threonine-protein phosphatase PP1 (EC 3.1.3.16)	Os03g0268000 LOC_Os03g16110 OJ1364E02.17 OJA1364E02.7	Oryza sativa subsp. japonica (Rice)		Complete proteome; Hydrolase; Manganese; Metal-binding; Protein phosphatase; Reference proteome
SRR1144633_TR3195_c0_g1_i1_len=3680	XPO2_ARATH	Q:232-3087,H:1-950	70.29%ID	E:0	Exportin-2	PF03810.16^IBN_N^Importin-beta N-terminal domain^29-108^E:2.3e-13`PF08506.7^Cse1^Cse1^167-536^E:3.8e-139`PF03378.12^CAS_CSE1^CAS/CSE protein, C-terminus^537-964^E:1.2e-152	0,312	-0,572	0,017	1,373	0,08	1,232	0,001	1,99	1	0,421	XPO2_ARATH	Exportin-2 (Exp2) (Cellular apoptosis susceptibility protein homolog) (Importin-alpha re-exporter)	CAS At2g46520 F11C10 F13A10.5	Arabidopsis thaliana (Mouse-ear cress)		Complete proteome; Cytoplasm; Nucleus; Protein transport; Reference proteome; Transport
SRR1144633_TR11716_c0_g2_i1_len=1771	PAP3_BRACM	Q:1372-506,H:85-359	63.79%ID	E:2e-115	Plastid lipid-associated protein 3, chloroplastic	PF04755.9^PAP_fibrillin^PAP_fibrillin^153-368^E:1.5e-67	0,897	0,041	0,177	0,405	0,004	0,988	0	1,234	0,317	0,374	PAP3_BRACM	Plastid lipid-associated protein 3, chloroplastic	PAP3	Brassica campestris (Field mustard)		Chloroplast; Plastid; Transit peptide
SRR1144633_TR14448_c0_g1_i2_len=860	GSTFB_ARATH	Q:103-750,H:1-211	53.24%ID	E:1e-77	Glutathione S-transferase F11	PF02798.17^GST_N^Glutathione S-transferase, N-terminal domain^2-75^E:1.6e-17`PF13409.3^GST_N_2^Glutathione S-transferase, N-terminal domain^13-75^E:1.3e-07`PF13417.3^GST_N_3^Glutathione S-transferase, N-terminal domain^15-80^E:8.1e-11`PF00043.22^GST_C^Glutathione S-transferase, C-terminal domain^115-208^E:7.5e-12`PF13410.3^GST_C_2^Glutathione S-transferase, C-terminal domain^133-189^E:1.1e-06	1	0	0,254	4,716	0,066	5,392	0	6,783	1	-1,579	GSTFB_ARATH	Glutathione S-transferase F11 (AtGSTF11) (EC 2.5.1.18) (AtGSTF6) (GST class-phi) (GST class-phi member 11)	GSTF11 GSTF6 At3g03190 T17B22.12	Arabidopsis thaliana (Mouse-ear cress)		Complete proteome; Cytoplasm; Detoxification; Reference proteome; Transferase
SRR1144633_TR15197_c0_g2_i1_len=2404	ASPG1_ARATH	Q:1342-188,H:106-500	37.93%ID	E:5e-70	Protein ASPARTIC PROTEASE IN GUARD CELL 1	PF00026.20^Asp^Eukaryotic aspartyl protease^177-513^E:4.2e-23`PF14543.3^TAXi_N^Xylanase inhibitor N-terminal^178-347^E:1.1e-53`PF14541.3^TAXi_C^Xylanase inhibitor C-terminal^367-513^E:2.5e-34	1	-0,005	0,043	-0,64	0,002	1,142	0	1,363	0,5	-0,642	ASPG1_ARATH	Protein ASPARTIC PROTEASE IN GUARD CELL 1 (AtASPG1) (EC 3.4.23.-)	ASPG1 At3g18490 MYF24.22	Arabidopsis thaliana (Mouse-ear cress)		Aspartyl protease; Complete proteome; DNA-binding; Disulfide bond; Endoplasmic reticulum; Hydrolase; Protease; Reference proteome; Signal
SRR1144633_TR19956_c0_g2_i1_len=2208	TAL_SHEAM	Q:1923-949,H:5-312	35.47%ID	E:1e-46	Transaldolase {ECO:0000255|HAMAP-Rule:MF_00492}	PF00923.16^Transaldolase^Transaldolase^79-386^E:4.2e-49	0,741	0,119	0,381	0,3	0,012	1,103	0	1,457	0,502	2,638	TAL_SHEAM	Transaldolase (EC 2.2.1.2)	tal Sama_0913	Shewanella amazonensis (strain ATCC BAA-1098 / SB2B)	PATHWAY: Carbohydrate degradation; pentose phosphate pathway; D-glyceraldehyde 3-phosphate and beta-D-fructose 6-phosphate from D-ribose 5-phosphate and D-xylulose 5-phosphate (non-oxidative stage): step 2/3. {ECO:0000255|HAMAP-Rule:MF_00492}.	Complete proteome; Cytoplasm; Pentose shunt; Reference proteome; Schiff base; Transferase
SRR1144633_TR22423_c0_g1_i1_len=2171	GSHB_SOLLC	Q:302-1825,H:42-546	74.21%ID	E:0	Glutathione synthetase, chloroplastic	PF03917.14^GSH_synth_ATP^Eukaryotic glutathione synthase, ATP binding domain^103-564^E:4.1e-139`PF03199.12^GSH_synthase^Eukaryotic glutathione synthase^298-397^E:9.5e-35	0,683	0,292	0,233	0,739	0,02	0,858	0	1,337	0,849	0,077	GSHB_SOLLC	Glutathione synthetase, chloroplastic (GSH synthetase) (GSH-S) (Glutathione synthase) (EC 6.3.2.3)	GSH2	Solanum lycopersicum (Tomato) (Lycopersicon esculentum)	PATHWAY: Sulfur metabolism; glutathione biosynthesis; glutathione from L-cysteine and L-glutamate: step 2/2.	ATP-binding; Chloroplast; Complete proteome; Glutathione biosynthesis; Ligase; Magnesium; Metal-binding; Nucleotide-binding; Plastid; Reference proteome; Transit peptide
SRR1144633_TR25443_c0_g1_i1_len=2147	SCL15_ARATH	Q:1680-235,H:25-486	46.75%ID	E:4e-124	Scarecrow-like protein 15	PF03514.11^GRAS^GRAS domain family^210-571^E:1.5e-89	1	0,443	0,088	1,537	0,01	1,497	0	2,025	1	0,251	SCL15_ARATH	Scarecrow-like protein 15 (AtSCL15) (GRAS family protein 25) (AtGRAS-25)	SCL15 At4g36710 C7A10.650	Arabidopsis thaliana (Mouse-ear cress)		Complete proteome; Nucleus; Reference proteome; Transcription; Transcription regulation
SRR1144634_TR2142_c0_g1_i1_len=1943	BCS1B_DICDI	Q:597-1460,H:181-457	33.45%ID	E:9e-31	Probable mitochondrial chaperone BCS1-B	PF00004.26^AAA^ATPase family associated with various cellular activities (AAA)^35-190^E:1.8e-14`PF05496.9^RuvB_N^Holliday junction DNA helicase ruvB N-terminus^35-91^E:0.00014	0,049	1,413	0,014	1,823	0,004	1,401	0	1,793	1	0,411	BCS1B_DICDI	Probable mitochondrial chaperone BCS1-B (BCS1-like protein 2)	bcsl1b DDB_G0291910	Dictyostelium discoideum (Slime mold)		ATP-binding; Chaperone; Complete proteome; Membrane; Mitochondrion; Mitochondrion inner membrane; Nucleotide-binding; Reference proteome; Transmembrane; Transmembrane helix
SRR1144634_TR17530_c0_g1_i4_len=1302	GSHB_SOLLC	Q:1187-123,H:192-546	77.46%ID	E:0	Glutathione synthetase, chloroplastic	PF03917.14^GSH_synth_ATP^Eukaryotic glutathione synthase, ATP binding domain^3-357^E:8.2e-105`PF03199.12^GSH_synthase^Eukaryotic glutathione synthase^91-190^E:4.3e-35	0,848	0,182	0,854	-0,282	0,001	1,395	0	1,499	1	-0,55	GSHB_SOLLC	Glutathione synthetase, chloroplastic (GSH synthetase) (GSH-S) (Glutathione synthase) (EC 6.3.2.3)	GSH2	Solanum lycopersicum (Tomato) (Lycopersicon esculentum)	PATHWAY: Sulfur metabolism; glutathione biosynthesis; glutathione from L-cysteine and L-glutamate: step 2/2.	ATP-binding; Chloroplast; Complete proteome; Glutathione biosynthesis; Ligase; Magnesium; Metal-binding; Nucleotide-binding; Plastid; Reference proteome; Transit peptide
SRR1144634_TR18582_c0_g1_i1_len=930	NA	NA	NA	NA	NA	PF00847.17^AP2^AP2 domain^73-123^E:8.9e-13	0,132	1,349	0,003	2,409	0,014	3,479	0	4,404	1	-0,604	.	.	.	.	.	.
SRR1144634_TR21850_c0_g2_i1_len=1765	DGK5_ARATH	Q:1682-726,H:1-320	74.92%ID	E:0	Diacylglycerol kinase 5	PF00781.21^DAGK_cat^Diacylglycerol kinase catalytic domain^65-192^E:7.6e-26`PF00609.16^DAGK_acc^Diacylglycerol kinase accessory domain^258-308^E:7.1e-14	0,807	0,188	0,115	0,754	0,017	1,196	0	1,922	1	0,004	DGK5_ARATH	Diacylglycerol kinase 5 (AtDGK5) (DAG kinase 5) (EC 2.7.1.107) (Diglyceride kinase 5) (DGK 5)	DGK5 At2g20900 F5H14.13	Arabidopsis thaliana (Mouse-ear cress)		ATP-binding; Alternative splicing; Complete proteome; Kinase; Nucleotide-binding; Plant defense; Reference proteome; Stress response; Transferase
SRR1144634_TR37303_c0_g2_i4_len=4925	LOX31_SOLTU	Q:2459-4924,H:1-826	79.21%ID	E:0	Linoleate 13S-lipoxygenase 3-1, chloroplastic	PF12146.5^Hydrolase_4^Serine aminopeptidase, S33^68-184^E:1e-08`PF01477.20^PLAT^PLAT/LH2 domain^592-676^E:1.4e-12`PF00305.16^Lipoxygenase^Lipoxygenase^689-1285^E:9.5e-273	0,112	1,27	0,001	2,401	0,001	6,457	0	7,338	1	0	LOX31_SOLTU	Linoleate 13S-lipoxygenase 3-1, chloroplastic (EC 1.13.11.12)	LOX3.1 LOX-H3	Solanum tuberosum (Potato)	PATHWAY: Lipid metabolism; oxylipin biosynthesis. {ECO:0000255|PROSITE-ProRule:PRU00726}.	Chloroplast; Complete proteome; Dioxygenase; Fatty acid biosynthesis; Fatty acid metabolism; Iron; Lipid biosynthesis; Lipid metabolism; Metal-binding; Oxidoreductase; Oxylipin biosynthesis; Plastid; Reference proteome; Thylakoid; Transit peptide
SRR122236_TR4154_c0_g1_i1_len=1464	MTDH_FRAAN	Q:121-1182,H:4-357	70.34%ID	E:2e-159	Probable mannitol dehydrogenase	PF08240.9^ADH_N^Alcohol dehydrogenase GroES-like domain^39-152^E:1.2e-27`PF00107.23^ADH_zinc_N^Zinc-binding dehydrogenase^195-317^E:2e-18	0,009	6,062	0,349	0,395	0,066	5,245	0	7,238	0	-9,752	MTDH_FRAAN	Probable mannitol dehydrogenase (EC 1.1.1.255) (NAD-dependent mannitol dehydrogenase)	CAD	Fragaria ananassa (Strawberry)		Metal-binding; NAD; Oxidoreductase; Zinc
SRR122236_TR5311_c0_g1_i1_len=699	GRXC9_ARATH	Q:607-221,H:11-137	54.2%ID	E:8e-40	Glutaredoxin-C9	PF00462.21^Glutaredoxin^Glutaredoxin^47-115^E:2.5e-09	0,381	2,207	0,003	3,114	0,004	2,199	0	3,715	1	-3,216	GRXC9_ARATH	Glutaredoxin-C9 (AtGrxC9) (Protein ROXY 19)	GRXC9 GRX480 ROXY19 At1g28480 F3M18.8	Arabidopsis thaliana (Mouse-ear cress)		Complete proteome; Cytoplasm; Disulfide bond; Electron transport; Nucleus; Redox-active center; Reference proteome; Transport
SRR122237_TR10367_c0_g1_i1_len=958	NA	NA	NA	NA	NA	PF02519.11^Auxin_inducible^Auxin responsive protein^17-115^E:1.1e-15	0,502	-1,643	0,254	3,942	0,017	5,761	0	6,797	0,059	-1,799	.	.	.	.	.	.
SRR122238_TR1035_c0_g1_i6_len=2350	SLK2_ARATH	Q:391-1254,H:309-593	73.61%ID	E:7e-150	Probable transcriptional regulator SLK2	PF01803.13^LIM_bind^LIM-domain binding protein^9-204^E:7.6e-46	0,163	0,719	0,407	0,435	0,003	1,371	0	1,585	1	0,073	SLK2_ARATH	Probable transcriptional regulator SLK2 (AtSLK2) (Protein SEUSS-like 2)	SLK2 At5g62090 MTG10.12	Arabidopsis thaliana (Mouse-ear cress)		Complete proteome; Developmental protein; Differentiation; Flowering; Nucleus; Reference proteome; Transcription; Transcription regulation
SRR122238_TR2305_c0_g1_i3_len=1328	NA	NA	NA	NA	NA	PF14009.3^DUF4228^Domain of unknown function (DUF4228)^1-171^E:5e-35	0,461	0,932	0,003	3,56	0,023	1,634	0	2,487	1	0,111	.	.	.	.	.	.
SRR122238_TR8025_c0_g1_i1_len=977	NA	NA	NA	NA	NA	PF04520.10^Senescence_reg^Senescence regulator^81-204^E:9.9e-37	0,62	0,53	0,01	1,882	0,006	2,547	0	3,33	0,811	0,233	.	.	.	.	.	.
SRR122238_TR11364_c1_g2_i2_len=1783	BGL45_ARATH	Q:778-1527,H:259-510	53.36%ID	E:2e-153	Beta-glucosidase 45	PF00232.15^Glyco_hydro_1^Glycosyl hydrolase family 1^6-228^E:2e-78	0,917	0,052	0,008	1,338	0,006	2,385	0,001	2,785	0,262	0,464	BGL45_ARATH	Beta-glucosidase 45 (AtBGLU45) (EC 3.2.1.21)	BGLU45 At1g61810 F8K4.2 T13M11.19	Arabidopsis thaliana (Mouse-ear cress)		Alternative splicing; Complete proteome; Disulfide bond; Glycoprotein; Glycosidase; Hydrolase; Reference proteome; Signal
SRR122238_TR11402_c0_g1_i1_len=912	MES10_ARATH	Q:890-129,H:22-275	50.79%ID	E:1e-91	Methylesterase 10	PF12146.5^Hydrolase_4^Serine aminopeptidase, S33^5-137^E:1.5e-09`PF00561.17^Abhydrolase_1^alpha/beta hydrolase fold^7-116^E:6.7e-14`PF12697.4^Abhydrolase_6^Alpha/beta hydrolase family^9-248^E:8.4e-13	1	2,089	0,129	2,915	0,034	4,277	0	5,425	1	-2,167	MES10_ARATH	Methylesterase 10 (AtMES10) (EC 3.1.1.-)	MES10 At3g50440 T20E23_40	Arabidopsis thaliana (Mouse-ear cress)		Complete proteome; Hydrolase; Reference proteome
SRR122238_TR11783_c0_g1_i1_len=1725	MIOX_ORYSJ	Q:531-1361,H:32-308	71.84%ID	E:4e-144	Probable inositol oxygenase	PF05153.12^MIOX^Myo-inositol oxygenase^101-350^E:2.1e-124	0,017	1,205	0,132	1,394	0,01	2,086	0	3,36	0,692	0,956	MIOX_ORYSJ	Probable inositol oxygenase (EC 1.13.99.1) (Myo-inositol oxygenase) (MI oxygenase)	Os06g0561000 LOC_Os06g36560 P0456F09.5 P0528E12.30	Oryza sativa subsp. japonica (Rice)	PATHWAY: Polyol metabolism; myo-inositol degradation into D-glucuronate; D-glucuronate from myo-inositol: step 1/1.	Ascorbate biosynthesis; Complete proteome; Cytoplasm; Iron; Metal-binding; Oxidoreductase; Reference proteome
SRR122238_TR15254_c0_g3_i4_len=908	APX3_ARATH	Q:875-351,H:111-287	78.53%ID	E:5e-74	L-ascorbate peroxidase 3, peroxisomal	PF00141.20^peroxidase^Peroxidase^28-125^E:3.8e-20	0,506	-0,199	0,046	-0,549	0,007	0,723	0,001	0,884	0,009	-5,96	APX3_ARATH	L-ascorbate peroxidase 3, peroxisomal (AtAPx03) (EC 1.11.1.11)	APX3 APX APXIII At4g35000 M4E13.60	Arabidopsis thaliana (Mouse-ear cress)		Acetylation; Calcium; Complete proteome; Glyoxysome; Heme; Hydrogen peroxide; Iron; Membrane; Metal-binding; Oxidoreductase; Peroxidase; Peroxisome; Potassium; Reference proteome; Transit peptide; Transmembrane; Transmembrane helix
SRR122238_TR16864_c0_g1_i2_len=2560	KSG1_ARATH	Q:272-1492,H:2-405	81.42%ID	E:0	Shaggy-related protein kinase alpha	PF00069.22^Pkinase^Protein kinase domain^73-357^E:3e-64`PF07714.14^Pkinase_Tyr^Protein tyrosine kinase^76-279^E:1.7e-24	0,865	0,211	0,021	1,59	0,003	2,041	0	2,344	0,739	-0,207	KSG1_ARATH	Shaggy-related protein kinase alpha (EC 2.7.11.1) (ASK-alpha)	ASK1 At5g26751 F2P16.21	Arabidopsis thaliana (Mouse-ear cress)		ATP-binding; Acetylation; Complete proteome; Kinase; Nucleotide-binding; Phosphoprotein; Reference proteome; Serine/threonine-protein kinase; Transferase
SRR122238_TR20547_c0_g1_i2_len=1209	NA	NA	NA	NA	NA	.	1	-0,379	0,129	4,962	0,034	3,883	0	5,488	1	-0,91	.	.	.	.	.	.
SRR122238_TR22324_c0_g1_i1_len=1347	FB237_ARATH	Q:1300-245,H:6-380	36.9%ID	E:8e-64	F-box protein At4g18380	PF00646.30^F-box^F-box domain^11-49^E:0.0099`PF12937.4^F-box-like^F-box-like^12-46^E:5.4e-08	0,502	2,312	0,132	3,233	0,034	4,753	0	5,798	1	-1,114	FB237_ARATH	F-box protein At4g18380	At4g18380 F28J12.40	Arabidopsis thaliana (Mouse-ear cress)		Complete proteome; Reference proteome
SRR122238_TR22745_c0_g1_i4_len=2323	MLO6_ARATH	Q:2152-344,H:1-583	60.59%ID	E:0	MLO-like protein 6	PF03094.12^Mlo^Mlo family^8-500^E:5.7e-250	0,44	0,748	0,663	0,397	0,037	1,295	0	2,206	0,299	-1,75	MLO6_ARATH	MLO-like protein 6 (AtMlo6)	MLO6 At1g61560 T25B24.9	Arabidopsis thaliana (Mouse-ear cress)		Alternative splicing; Calmodulin-binding; Complete proteome; Membrane; Pathogenesis-related protein; Plant defense; Reference proteome; Transmembrane; Transmembrane helix
SRR122238_TR23432_c0_g2_i1_len=878	NA	NA	NA	NA	NA	.	0,506	-0,179	0,218	-0,34	0,009	0,794	0	1,16	0,764	-0,103	.	.	.	.	.	.
SRR122238_TR38821_c0_g1_i2_len=1232	DNJ72_ARATH	Q:184-396,H:2-72	63.38%ID	E:2e-23	Chaperone protein dnaJ 72	PF00226.28^DnaJ^DnaJ domain^63-130^E:2e-24	0,409	0,431	0,376	0,473	0,002	1,297	0	1,51	1	0,132	DNJ72_ARATH	Chaperone protein dnaJ 72 (AtDjC72) (AtJ72)	ATJ72 C72 LCR51 At2g41000 T3K9.23	Arabidopsis thaliana (Mouse-ear cress)		Alternative splicing; Chaperone; Complete proteome; Membrane; Reference proteome; Transmembrane; Transmembrane helix
SRR122238_TR44501_c0_g3_i2_len=2188	PME3_PHAVU	Q:2089-335,H:1-578	57.55%ID	E:0	Pectinesterase 3	PF04043.12^PMEI^Plant invertase/pectin methylesterase inhibitor^74-229^E:1.6e-25`PF01095.16^Pectinesterase^Pectinesterase^281-574^E:1.9e-134	0,444	0,319	0,086	0,86	0,032	0,752	0	1,343	0,779	-0,121	PME3_PHAVU	Pectinesterase 3 (PE 3) (EC 3.1.1.11) (Pectin methylesterase 3)	MPE3	Phaseolus vulgaris (Kidney bean) (French bean)	PATHWAY: Glycan metabolism; pectin degradation; 2-dehydro-3-deoxy-D-gluconate from pectin: step 1/5.	Aspartyl esterase; Cell wall; Cell wall biogenesis/degradation; Disulfide bond; Glycoprotein; Hydrolase; Secreted; Signal
SRR122238_TR45975_c1_g1_i4_len=3521	LOX31_SOLTU	Q:619-3204,H:114-914	77.64%ID	E:0	Linoleate 13S-lipoxygenase 3-1, chloroplastic	PF00305.16^Lipoxygenase^Lipoxygenase^1-262^E:9.2e-100	1	2,176	0,254	4,593	0,009	3,945	0	4,699	1	0,608	LOX31_SOLTU	Linoleate 13S-lipoxygenase 3-1, chloroplastic (EC 1.13.11.12)	LOX3.1 LOX-H3	Solanum tuberosum (Potato)	PATHWAY: Lipid metabolism; oxylipin biosynthesis. {ECO:0000255|PROSITE-ProRule:PRU00726}.	Chloroplast; Complete proteome; Dioxygenase; Fatty acid biosynthesis; Fatty acid metabolism; Iron; Lipid biosynthesis; Lipid metabolism; Metal-binding; Oxidoreductase; Oxylipin biosynthesis; Plastid; Reference proteome; Thylakoid; Transit peptide
SRR122238_TR49386_c0_g2_i3_len=1508	FPPS1_LUPAL	Q:1310-285,H:1-342	79.53%ID	E:0	Farnesyl pyrophosphate synthase 1	PF00348.14^polyprenyl_synt^Polyprenyl synthetase^33-296^E:2e-94	1	0,007	0,034	0,87	0,003	1,055	0	1,548	0,555	-0,271	FPPS1_LUPAL	Farnesyl pyrophosphate synthase 1 (FPP synthase 1) (FPS 1) (EC 2.5.1.10) ((2E,6E)-farnesyl diphosphate synthase 1) (Dimethylallyltranstransferase 1) (EC 2.5.1.1) (Farnesyl diphosphate synthase 1) (Geranyltranstransferase 1)	FPS1	Lupinus albus (White lupin) (Lupinus termis)	PATHWAY: Isoprenoid biosynthesis; farnesyl diphosphate biosynthesis; farnesyl diphosphate from geranyl diphosphate and isopentenyl diphosphate: step 1/1.; PATHWAY: Isoprenoid biosynthesis; geranyl diphosphate biosynthesis; geranyl diphosphate from dimethylallyl diphosphate and isopentenyl diphosphate: step 1/1.	Cholesterol biosynthesis; Cholesterol metabolism; Cytoplasm; Isoprene biosynthesis; Lipid biosynthesis; Lipid metabolism; Magnesium; Metal-binding; Steroid biosynthesis; Steroid metabolism; Sterol biosynthesis; Sterol metabolism; Transferase
SRR122238_TR52070_c1_g5_i2_len=1384	EXPA1_ARATH	Q:155-898,H:1-250	84.4%ID	E:1e-114	Expansin-A1	PF03330.15^DPBB_1^Lytic transglycolase^60-145^E:3.7e-22`PF01357.18^Pollen_allerg_1^Pollen allergen^156-233^E:1.2e-30	0,3	0,329	0,024	0,601	0,175	0,548	0	1,761	0,545	-0,45	EXPA1_ARATH	Expansin-A1 (AtEXPA1) (Alpha-expansin-1) (At-EXP1) (AtEx1) (Ath-ExpAlpha-1.2)	EXPA1 EXP1 At1g69530 F10D13_18	Arabidopsis thaliana (Mouse-ear cress)		Alternative splicing; Cell wall; Cell wall biogenesis/degradation; Complete proteome; Disulfide bond; Membrane; Reference proteome; Secreted; Signal
SRR122238_TR60656_c1_g1_i1_len=1153	ERD2_PETHY	Q:774-130,H:1-215	92.09%ID	E:5e-133	ER lumen protein-retaining receptor	PF00810.15^ER_lumen_recept^ER lumen protein retaining receptor^28-171^E:2.2e-51	0,816	-0,11	0,001	1,367	0,045	0,861	0	1,404	1	-0,563	ERD2_PETHY	ER lumen protein-retaining receptor (HDEL receptor) (PGP169-12)	ERD2	Petunia hybrida (Petunia)		ER-Golgi transport; Endoplasmic reticulum; Membrane; Protein transport; Receptor; Transmembrane; Transmembrane helix; Transport
SRR122238_TR62934_c0_g1_i2_len=652	RPD6A_ARATH	Q:495-259,H:61-139	94.94%ID	E:1e-46	DNA-directed RNA polymerases II, IV and V subunit 6A	PF01192.19^RNA_pol_Rpb6^RNA polymerase Rpb6^53-104^E:6.3e-17	0	-2,234	0,014	-1,012	1	0	0	6,919	1	-1,817	RPD6A_ARATH	DNA-directed RNA polymerases II, IV and V subunit 6A (RNA polymerase Rpb6)	NRPB6A NRPD6A NRPE6A At5g51940 MSG15.2	Arabidopsis thaliana (Mouse-ear cress)		Complete proteome; DNA-directed RNA polymerase; Nucleus; Reference proteome; Transcription
SRR122238_TR69049_c2_g1_i3_len=1046	SUI11_ARATH	Q:270-608,H:1-113	90.27%ID	E:2e-68	Protein translation factor SUI1 homolog 1	PF01253.19^SUI1^Translation initiation factor SUI1^28-102^E:4.3e-26	0,672	-0,132	0,914	-0,056	0,005	0,985	0,001	1,148	1	0,009	SUI11_ARATH	Protein translation factor SUI1 homolog 1	At4g27130 F20D21.11 T24A18.80	Arabidopsis thaliana (Mouse-ear cress)		Acetylation; Complete proteome; Protein biosynthesis; Reference proteome; Translation regulation
SRR122238_TR77058_c1_g1_i6_len=612	F16P1_SPIOL	Q:368-84,H:51-147	65.98%ID	E:9e-32	Fructose-1,6-bisphosphatase, chloroplastic	PF00316.17^FBPase^Fructose-1-6-bisphosphatase, N-terminal domain^117-176^E:2.7e-12	0,001	-6,56	1	0	0,034	5,65	0	6,729	1	1,191	F16P1_SPIOL	Fructose-1,6-bisphosphatase, chloroplastic (FBPase) (EC 3.1.3.11) (D-fructose-1,6-bisphosphate 1-phosphohydrolase)		Spinacia oleracea (Spinach)	PATHWAY: Carbohydrate biosynthesis; Calvin cycle.	3D-structure; Calvin cycle; Carbohydrate metabolism; Chloroplast; Direct protein sequencing; Disulfide bond; Hydrolase; Magnesium; Metal-binding; Plastid; Transit peptide
SRR122238_TR80913_c0_g1_i1_len=1036	VA711_ARATH	Q:63-620,H:34-219	84.95%ID	E:4e-103	Vesicle-associated membrane protein 711 {ECO:0000303|PubMed:11115874}	PF13774.3^Longin^Regulated-SNARE-like domain^21-95^E:6.6e-23`PF00957.18^Synaptobrevin^Synaptobrevin^111-198^E:4.5e-31	0,471	-0,451	0,034	-1,127	0,001	6,426	0	7,066	0,151	-0,683	VA711_ARATH	Vesicle-associated membrane protein 711 (AtVAMP711) (v-SNARE synaptobrevin 7C) (AtVAMP7C)	VAMP711 VAMP7C At4g32150 F10N7.40	Arabidopsis thaliana (Mouse-ear cress)		Acetylation; Coiled coil; Complete proteome; Membrane; Protein transport; Reference proteome; Transmembrane; Transmembrane helix; Transport; Vacuole
SRR122238_TR89616_c0_g1_i2_len=2252	NA	NA	NA	NA	NA	.	0,407	0,728	0,014	1,647	0,01	2,22	0	2,759	1	0,105	.	.	.	.	.	.
SRR1271857_TR593_c3_g1_i1_len=1988	NA	NA	NA	NA	NA	PF04434.14^SWIM^SWIM zinc finger^144-174^E:3.5e-06	1	0,012	0,692	0,614	0,001	2,475	0	3,568	0,402	1,211	.	.	.	.	.	.
SRR1271857_TR11022_c0_g1_i1_len=2516	NA	NA	NA	NA	NA	PF00881.21^Nitroreductase^Nitroreductase family^173-295^E:1.1e-06	1	0,029	0,01	1,663	0,006	2,572	0	3,095	1	0,199	.	.	.	.	.	.
SRR1271857_TR13742_c0_g1_i2_len=2734	NA	NA	NA	NA	NA	PF07534.13^TLD^TLD^207-288^E:4e-12	0,798	0,328	1	0,085	0,076	2,332	0,001	3,31	0,878	-0,213	.	.	.	.	.	.
SRR1271857_TR15657_c0_g1_i1_len=2414	NCED1_PHAVU	Q:2206-356,H:6-615	71.15%ID	E:0	9-cis-epoxycarotenoid dioxygenase NCED1, chloroplastic	PF03055.12^RPE65^Retinal pigment epithelial membrane protein^150-616^E:8.6e-102	0,502	2,183	0,004	3,422	0,004	2,362	0,001	2,639	0,381	1,422	NCED1_PHAVU	9-cis-epoxycarotenoid dioxygenase NCED1, chloroplastic (EC 1.13.11.51) (PvNCED1)	NCED1	Phaseolus vulgaris (Kidney bean) (French bean)		Abscisic acid biosynthesis; Chloroplast; Coiled coil; Dioxygenase; Iron; Membrane; Metal-binding; Oxidoreductase; Plastid; Stress response; Thylakoid; Transit peptide
SRR1271857_TR22691_c0_g2_i1_len=2796	NA	NA	NA	NA	NA	PF00400.29^WD40^WD domain, G-beta repeat^129-155^E:0.15`PF00400.29^WD40^WD domain, G-beta repeat^239-267^E:0.002`PF00400.29^WD40^WD domain, G-beta repeat^273-308^E:0.02`PF00400.29^WD40^WD domain, G-beta repeat^367-398^E:0.027`PF00400.29^WD40^WD domain, G-beta repeat^408-457^E:0.00022	1	1,198	0,008	3,778	0,094	1,072	0	1,978	0,027	2,678	.	.	.	.	.	.
SRR1271857_TR23244_c0_g1_i1_len=2966	Y1491_ARATH	Q:2309-1380,H:72-368	33.23%ID	E:2e-43	Uncharacterized protein At1g04910	PF10250.6^O-FucT^GDP-fucose protein O-fucosyltransferase^94-382^E:3.1e-45	1	0,077	0,5	0,747	0,016	1,885	0	2,601	1	0,106	Y1491_ARATH	Uncharacterized protein At1g04910	At1g04910 F13M7.10	Arabidopsis thaliana (Mouse-ear cress)		Complete proteome; Golgi apparatus; Membrane; Reference proteome; Transmembrane; Transmembrane helix
SRR1271857_TR29018_c0_g3_i5_len=2164	NA	NA	NA	NA	NA	.	0,517	0,706	0,517	-0,867	1	-0,304	0	3,906	1	-0,061	.	.	.	.	.	.
SRR1271857_TR32733_c0_g1_i6_len=2084	PT504_ARATH	Q:1022-1792,H:6-262	81.71%ID	E:2e-147	Probable sugar phosphate/phosphate translocator At5g04160	PF03151.13^TPT^Triose-phosphate Transporter family^20-270^E:8.5e-36	1	1,541	0,629	1,79	0,066	3,211	0	4,543	1	-1,24	PT504_ARATH	Probable sugar phosphate/phosphate translocator At5g04160	At5g04160 F21E1.80	Arabidopsis thaliana (Mouse-ear cress)		Complete proteome; Membrane; Pyruvate; Reference proteome; Sugar transport; Transmembrane; Transmembrane helix; Transport
SRR1271857_TR37545_c0_g1_i1_len=1367	7SB1_SOYBN	Q:114-1343,H:26-424	35.48%ID	E:6e-56	Basic 7S globulin	PF14543.3^TAXi_N^Xylanase inhibitor N-terminal^61-236^E:1.6e-36`PF14541.3^TAXi_C^Xylanase inhibitor C-terminal^277-432^E:4.9e-46	1	0,228	0,629	1,351	0,066	4,144	0,001	5,378	0,502	2,04	7SB1_SOYBN	Basic 7S globulin (SBg7S) (Bg) [Cleaved into: Basic 7S globulin high kDa subunit; Basic 7S globulin low kDa subunit]	BG G7S	Glycine max (Soybean) (Glycine hispida)		3D-structure; Complete proteome; Direct protein sequencing; Disulfide bond; Reference proteome; Seed storage protein; Signal; Storage protein
SRR1271857_TR38570_c0_g1_i17_len=2110	BON1_ARATH	Q:142-1878,H:1-576	70.69%ID	E:0	Protein BONZAI 1	PF00168.27^C2^C2 domain^53-157^E:1.6e-12`PF00168.27^C2^C2 domain^199-291^E:1.7e-10`PF07002.13^Copine^Copine^363-576^E:2.2e-80	0,908	-0,072	0,493	0,354	0,022	1,362	0,001	1,902	1	-0,033	BON1_ARATH	Protein BONZAI 1 (COPINE 1)	BON1 CPN1 At5g61900 K22G18.2	Arabidopsis thaliana (Mouse-ear cress)		Cell membrane; Complete proteome; Lipoprotein; Membrane; Myristate; Plant defense; Reference proteome; Repeat
SRR1271858_TR9953_c0_g1_i2_len=1742	BH112_ARATH	Q:1254-1658,H:258-389	70.07%ID	E:4e-49	Transcription factor bHLH112	.	1	0,417	0,402	0,959	0,014	1,817	0	2,971	0,43	0,61	BH112_ARATH	Transcription factor bHLH112 (Basic helix-loop-helix protein 112) (AtbHLH112) (bHLH 112) (Transcription factor EN 64) (bHLH transcription factor bHLH112)	BHLH112 EN64 At1g61660 T13M11.1	Arabidopsis thaliana (Mouse-ear cress)		Alternative splicing; Complete proteome; DNA-binding; Nucleus; Reference proteome; Transcription; Transcription regulation
SRR1271858_TR10360_c0_g1_i1_len=1736	ERF80_ARATH	Q:1481-1272,H:29-98	88.57%ID	E:1e-22	Ethylene-responsive transcription factor 9	PF00847.17^AP2^AP2 domain^23-72^E:5.5e-14	1	0,026	0,026	2,049	0,007	1,232	0	1,788	0,257	-0,858	ERF80_ARATH	Ethylene-responsive transcription factor 9 (AtERF9) (Ethylene-responsive element-binding factor 9) (EREBP-9)	ERF9 ERF-9 ERF080 At5g44210 MLN1.14	Arabidopsis thaliana (Mouse-ear cress)		Complete proteome; DNA-binding; Ethylene signaling pathway; Nucleus; Plant defense; Reference proteome; Repressor; Transcription; Transcription regulation
SRR1271858_TR11121_c0_g1_i1_len=932	NA	NA	NA	NA	NA	.	0,739	-0,292	0,233	0,806	0,035	1,692	0,001	2,33	1	-1,93	.	.	.	.	.	.
SRR1271858_TR15208_c0_g1_i29_len=7385	AB10C_ARATH	Q:1142-3499,H:2-766	59.17%ID	E:0	ABC transporter C family member 10	PF00664.20^ABC_membrane^ABC transporter transmembrane region^310-569^E:2.4e-23`PF00005.24^ABC_tran^ABC transporter^644-778^E:6.7e-25	1	0,125	0,402	1,072	0,029	1,137	0	1,946	0,079	1,025	AB10C_ARATH	ABC transporter C family member 10 (ABC transporter ABCC.10) (AtABCC10) (EC 3.6.3.44) (ATP-energized glutathione S-conjugate pump 14) (Glutathione S-conjugate-transporting ATPase 14) (Multidrug resistance-associated protein 14)	ABCC10 MRP10 MRP14 At3g59140 F17J16.190	Arabidopsis thaliana (Mouse-ear cress)		ATP-binding; Complete proteome; Hydrolase; Membrane; Nucleotide-binding; Reference proteome; Repeat; Transmembrane; Transmembrane helix; Transport
SRR1271858_TR21091_c0_g1_i1_len=3472	MPK7_ARATH	Q:2027-2710,H:141-368	76.32%ID	E:1e-117	Mitogen-activated protein kinase 7	PF00069.22^Pkinase^Protein kinase domain^1-130^E:7.7e-22	0,732	0,403	0,002	2,663	0,025	2,737	0	3,758	0,502	-3,456	MPK7_ARATH	Mitogen-activated protein kinase 7 (AtMPK7) (MAP kinase 7) (EC 2.7.11.24)	MPK7 At2g18170 F8D23	Arabidopsis thaliana (Mouse-ear cress)		ATP-binding; Complete proteome; Kinase; Nucleotide-binding; Phosphoprotein; Plant defense; Reference proteome; Serine/threonine-protein kinase; Transferase
SRR1271858_TR21511_c0_g1_i1_len=1610	NA	NA	NA	NA	NA	.	1	-0,117	0,004	2,083	0,007	1,405	0,001	1,686	1	0,162	.	.	.	.	.	.
SRR1271858_TR21658_c2_g1_i3_len=714	NA	NA	NA	NA	NA	.	0,502	2,261	0,01	2,946	0,015	0,719	0	1,718	1	0,923	.	.	.	.	.	.
SRR1271858_TR25802_c0_g2_i1_len=848	NA	NA	NA	NA	NA	.	1	0,198	0,381	2,394	0,01	2,736	0	3,33	1	0,315	.	.	.	.	.	.
SRR1271858_TR25881_c0_g4_i7_len=3003	NA	NA	NA	NA	NA	.	0,593	0,509	0,112	1,136	0,059	1,825	0,001	2,613	0,86	0,249	.	.	.	.	.	.
SRR1271858_TR27375_c1_g1_i1_len=2462	Y5392_ARATH	Q:1342-311,H:448-787	43.27%ID	E:5e-90	Probable receptor-like protein kinase At5g39020	PF13947.3^GUB_WAK_bind^Wall-associated receptor kinase galacturonan-binding^32-97^E:6.8e-08`PF14380.3^WAK_assoc^Wall-associated receptor kinase C-terminal^152-244^E:2.5e-06`PF07714.14^Pkinase_Tyr^Protein tyrosine kinase^339-607^E:6.1e-46`PF00069.22^Pkinase^Protein kinase domain^340-605^E:2.9e-46	1	-0,025	1	0,074	0,071	0,962	0	2,042	1	-0,383	Y5392_ARATH	Probable receptor-like protein kinase At5g39020 (EC 2.7.11.-)	At5g39020 MXF12.30	Arabidopsis thaliana (Mouse-ear cress)		ATP-binding; Complete proteome; Glycoprotein; Kinase; Membrane; Nucleotide-binding; Reference proteome; Serine/threonine-protein kinase; Signal; Transferase; Transmembrane; Transmembrane helix
SRR1271858_TR33514_c0_g1_i3_len=1675	BEBT_TOBAC	Q:1135-248,H:150-445	79.05%ID	E:1e-170	Benzyl alcohol O-benzoyltransferase	PF02458.12^Transferase^Transferase family^1-270^E:5.8e-41	1	0,141	0,299	1,515	0,132	1,479	0	2,583	0,528	0,329	BEBT_TOBAC	Benzyl alcohol O-benzoyltransferase (EC 2.3.1.196) (Benzoyl coenzyme A:benzyl alcohol benzoyl transferase)	HSR201	Nicotiana tabacum (Common tobacco)		Acyltransferase; Transferase
SRR1271858_TR44750_c0_g1_i6_len=2825	NA	NA	NA	NA	NA	.	0,381	1,887	0,381	2,511	0,026	1,935	0	2,874	0,798	-0,533	.	.	.	.	.	.
SRR1271858_TR44807_c0_g2_i6_len=1042	NA	NA	NA	NA	NA	.	0,732	0,923	0,005	4,491	0,043	2,86	0	4,03	0,56	0,773	.	.	.	.	.	.
SRR1271858_TR52796_c0_g1_i2_len=996	NA	NA	NA	NA	NA	PF10601.6^zf-LITAF-like^LITAF-like zinc ribbon domain^65-129^E:7.9e-12	0,618	0,298	0,001	1,555	0,003	1,33	0,001	1,449	0,882	0,159	.	.	.	.	.	.
SRR1271858_TR53101_c0_g2_i4_len=1945	NA	NA	NA	NA	NA	.	1	0,213	1	0,706	0,017	4,117	0	5,247	1	0,022	.	.	.	.	.	.
SRR1271858_TR60281_c0_g2_i1_len=583	NA	NA	NA	NA	NA	.	0,458	-0,382	1	0	1	0	0	7,232	1	0	.	.	.	.	.	.
SRR1271858_TR64995_c1_g2_i2_len=1783	E1314_ARATH	Q:546-1544,H:28-361	70.36%ID	E:3e-171	Glucan endo-1,3-beta-glucosidase 14	PF00332.15^Glyco_hydro_17^Glycosyl hydrolases family 17^28-344^E:3.1e-94	0,239	1,513	0,001	2,187	0,015	1,834	0	2,454	0,356	-1,138	E1314_ARATH	Glucan endo-1,3-beta-glucosidase 14 (EC 3.2.1.39) ((1->3)-beta-glucan endohydrolase 14) ((1->3)-beta-glucanase 14) (Beta-1,3-endoglucanase 14) (Beta-1,3-glucanase 14)	At2g27500 F10A12.18	Arabidopsis thaliana (Mouse-ear cress)		Alternative splicing; Cell membrane; Cell wall; Cell wall biogenesis/degradation; Complete proteome; Cytoplasm; GPI-anchor; Glycoprotein; Glycosidase; Hydrolase; Lipoprotein; Membrane; Plant defense; Reference proteome; Secreted; Signal
SRR1271858_TR65916_c0_g1_i3_len=1129	NATAL_ARATH	Q:215-871,H:21-236	68.49%ID	E:6e-101	Probable acetyltransferase NATA1-like	PF00583.22^Acetyltransf_1^Acetyltransferase (GNAT) family^135-215^E:1.3e-14`PF13508.4^Acetyltransf_7^Acetyltransferase (GNAT) domain^139-214^E:1.9e-07`PF13673.4^Acetyltransf_10^Acetyltransferase (GNAT) domain^142-214^E:1.2e-08`PF08445.7^FR47^FR47-like protein^153-217^E:8.7e-06	1	-0,031	0,029	0,898	0,013	1,426	0,001	1,758	0,2	-0,677	NATAL_ARATH	Probable acetyltransferase NATA1-like (EC 2.3.1.-)	At2g39020 T7F6.19	Arabidopsis thaliana (Mouse-ear cress)		Acyltransferase; Complete proteome; Reference proteome; Transferase
SRR1271858_TR69422_c1_g1_i1_len=494	NA	NA	NA	NA	NA	.	1	0,973	0,017	3,785	0,003	1,765	0	1,955	1	-0,936	.	.	.	.	.	.
SRR1271858_TR69458_c0_g1_i2_len=1398	RIN4_ARATH	Q:157-945,H:3-208	36.84%ID	E:5e-35	RPM1-interacting protein 4	PF05627.8^AvrRpt-cleavage^Cleavage site for pathogenic type III effector avirulence factor Avr^199-235^E:2.6e-22	0,066	-4,495	1	-0,609	0,004	3,873	0	4,73	1	-0,072	RIN4_ARATH	RPM1-interacting protein 4	RIN4 At3g25070 MJL12_1	Arabidopsis thaliana (Mouse-ear cress)		3D-structure; Complete proteome; Direct protein sequencing; Hypersensitive response; Immunity; Innate immunity; Lipoprotein; Membrane; Palmitate; Phosphoprotein; Plant defense; Reference proteome
SRR1271859_TR3351_c0_g1_i1_len=960	NA	NA	NA	NA	NA	PF06108.9^DUF952^Protein of unknown function (DUF952)^19-112^E:1.9e-24	0,718	0,223	0,712	0,189	0,004	1,818	0	2,179	1	-0,429	.	.	.	.	.	.
SRR1271859_TR6287_c0_g1_i1_len=2208	ERDL6_ARATH	Q:1928-981,H:1-315	82.28%ID	E:2e-162	Sugar transporter ERD6-like 6	PF00083.21^Sugar_tr^Sugar (and other) transporter^52-316^E:5.9e-58`PF07690.13^MFS_1^Major Facilitator Superfamily^61-292^E:1.4e-19	1	0,094	0,001	2,601	0,147	1,026	0	2,178	0,629	-0,73	ERDL6_ARATH	Sugar transporter ERD6-like 6	At1g75220 F22H5.6	Arabidopsis thaliana (Mouse-ear cress)		Acetylation; Complete proteome; Membrane; Reference proteome; Sugar transport; Transmembrane; Transmembrane helix; Transport
SRR1271859_TR6287_c0_g1_i4_len=2520	ERDL6_ARATH	Q:1730-981,H:67-315	82.4%ID	E:4e-128	Sugar transporter ERD6-like 6	PF07690.13^MFS_1^Major Facilitator Superfamily^7-242^E:1.7e-20`PF00083.21^Sugar_tr^Sugar (and other) transporter^33-266^E:5.1e-57	1	-0,18	0,781	0,489	0,001	6,453	0,001	6,607	1	2,374	ERDL6_ARATH	Sugar transporter ERD6-like 6	At1g75220 F22H5.6	Arabidopsis thaliana (Mouse-ear cress)		Acetylation; Complete proteome; Membrane; Reference proteome; Sugar transport; Transmembrane; Transmembrane helix; Transport
SRR1271859_TR7000_c2_g1_i6_len=1206	NA	NA	NA	NA	NA	PF10457.6^MENTAL^Cholesterol-capturing domain^114-201^E:2.1e-05	0,211	0,807	0,009	1,202	0,004	1,709	0	2,696	0,138	-0,872	.	.	.	.	.	.
SRR1271859_TR7368_c0_g2_i1_len=1075	NA	NA	NA	NA	NA	.	0,842	0,139	0,069	-0,71	0,001	1,538	0	1,693	1	0,08	.	.	.	.	.	.
SRR1271859_TR15713_c0_g1_i22_len=4218	FB92_ARATH	Q:3589-1544,H:85-758	61.1%ID	E:0	F-box protein At1g78280	PF13621.3^Cupin_8^Cupin-like domain^11-195^E:4.1e-16`PF02373.19^JmjC^JmjC domain, hydroxylase^80-193^E:8.7e-15	1	1,044	0,014	3,132	0,076	3,118	0	4,545	1	-2,495	FB92_ARATH	F-box protein At1g78280	At1g78280 F3F9.18	Arabidopsis thaliana (Mouse-ear cress)		Complete proteome; Reference proteome
SRR1271859_TR15770_c0_g1_i7_len=2206	PNC1_SOYBN	Q:1151-1942,H:48-310	76.14%ID	E:2e-110	Peroxisomal adenine nucleotide carrier 1	PF00153.24^Mito_carr^Mitochondrial carrier protein^69-162^E:6.6e-17	0,629	1,308	0,025	2,665	0,004	3,789	0	4,432	1	0,271	PNC1_SOYBN	Peroxisomal adenine nucleotide carrier 1 (GmPNC1)	PNC1 Glyma02g11800	Glycine max (Soybean) (Glycine hispida)		Antiport; Complete proteome; Membrane; Peroxisome; Reference proteome; Repeat; Transmembrane; Transmembrane helix; Transport
SRR1271859_TR17688_c0_g2_i12_len=1795	MPK3_ARATH	Q:259-1365,H:1-370	83.78%ID	E:0	Mitogen-activated protein kinase 3	PF00069.22^Pkinase^Protein kinase domain^45-326^E:2.1e-73`PF07714.14^Pkinase_Tyr^Protein tyrosine kinase^46-237^E:4.2e-33`PF06293.11^Kdo^Lipopolysaccharide kinase (Kdo/WaaP) family^79-194^E:4.8e-06	0,282	1,18	0,007	2,029	0,175	0,875	0	2,117	1	0,009	MPK3_ARATH	Mitogen-activated protein kinase 3 (AtMPK3) (MAP kinase 3) (EC 2.7.11.24)	MPK3 At3g45640 F9K21.220 T6D9.4	Arabidopsis thaliana (Mouse-ear cress)		ATP-binding; Complete proteome; Cytoplasm; Hypersensitive response; Kinase; Nucleotide-binding; Nucleus; Phosphoprotein; Plant defense; Reference proteome; Serine/threonine-protein kinase; Stress response; Transferase
SRR1271859_TR20608_c1_g2_i1_len=1739	Y8948_DICDI	Q:934-1698,H:15-261	32.32%ID	E:3e-30	Putative methyltransferase DDB_G0268948	PF13649.3^Methyltransf_25^Methyltransferase domain^37-128^E:9.1e-10`PF13847.3^Methyltransf_31^Methyltransferase domain^37-138^E:4.5e-08`PF08241.9^Methyltransf_11^Methyltransferase domain^39-132^E:1.3e-10`PF08242.9^Methyltransf_12^Methyltransferase domain^39-132^E:2.2e-08	1	0,23	0,132	2,481	0,076	2,734	0	3,942	0,848	-0,158	Y8948_DICDI	Putative methyltransferase DDB_G0268948 (EC 2.1.1.-)	DDB_G0268948	Dictyostelium discoideum (Slime mold)		Complete proteome; Methyltransferase; Reference proteome; S-adenosyl-L-methionine; Transferase
SRR1271859_TR22096_c0_g1_i1_len=802	NA	NA	NA	NA	NA	.	0,546	-0,182	0,014	0,705	0,036	0,564	0	1,442	0,097	-0,562	.	.	.	.	.	.
SRR1271859_TR27652_c0_g1_i1_len=517	NA	NA	NA	NA	NA	.	0,44	-0,914	0,732	0,584	0,005	6,146	0	7,215	0,001	-6,625	.	.	.	.	.	.
SRR1271859_TR29110_c0_g1_i1_len=1921	ZOG_PHALU	Q:1696-314,H:13-459	55.53%ID	E:2e-168	Zeatin O-glucosyltransferase	PF00201.15^UDPGT^UDP-glucoronosyl and UDP-glucosyl transferase^231-391^E:5.9e-22	1	2,17	0,129	5,177	0,005	6,173	0	7,542	1	0	ZOG_PHALU	Zeatin O-glucosyltransferase (EC 2.4.1.203) (Trans-zeatin O-beta-D-glucosyltransferase)	ZOG1	Phaseolus lunatus (Lima bean) (Phaseolus limensis)		Glycosyltransferase; Transferase
SRR1271859_TR29831_c0_g1_i1_len=564	NA	NA	NA	NA	NA	.	0,062	0,793	0,77	-0,153	0,014	1,097	0,001	1,415	0,438	-0,351	.	.	.	.	.	.
SRR1271859_TR32785_c0_g1_i6_len=2776	SCP29_ARATH	Q:403-1458,H:30-381	68.18%ID	E:4e-176	Serine carboxypeptidase-like 29	PF00450.19^Peptidase_S10^Serine carboxypeptidase^45-384^E:1.3e-116	0,62	0,693	0,002	2,477	0,003	3,563	0	3,873	0,692	-0,594	SCP29_ARATH	Serine carboxypeptidase-like 29 (EC 3.4.16.-)	SCPL29 At4g30810 F6I18.280 T10C21.3	Arabidopsis thaliana (Mouse-ear cress)		Carboxypeptidase; Complete proteome; Disulfide bond; Glycoprotein; Hydrolase; Protease; Reference proteome; Secreted; Signal
SRR1271859_TR33139_c0_g2_i4_len=1489	NA	NA	NA	NA	NA	PF00226.28^DnaJ^DnaJ domain^66-128^E:8.8e-18`PF13459.3^Fer4_15^4Fe-4S single cluster domain^151-207^E:3e-10`PF13370.3^Fer4_13^4Fe-4S single cluster domain of Ferredoxin I^153-207^E:2e-14`PF12800.4^Fer4_4^4Fe-4S binding domain^157-170^E:0.0048	1	-1,096	1	0	0,014	0,866	0	1,241	0,009	0,691	.	.	.	.	.	.
SRR1271859_TR33648_c0_g5_i31_len=1176	IPCS2_ARATH	Q:586-795,H:1-70	78.57%ID	E:3e-31	Phosphatidylinositol:ceramide inositolphosphotransferase 2	.	0,756	0,256	0,059	1,806	0,298	0,654	0,001	1,742	0,132	1,317	IPCS2_ARATH	Phosphatidylinositol:ceramide inositolphosphotransferase 2 (EC 2.7.8.-) (Inositol-phosphorylceramide synthase 2) (AtIPCS2) (IPC synthase 2) (Protein ENHANCING RPW8-MEDIATED HR-LIKE CELL DEATH 1) (Sphingolipid synthase 2)	IPCS2 ERH1 At2g37940 T8P21.15	Arabidopsis thaliana (Mouse-ear cress)		Complete proteome; Golgi apparatus; Lipid biosynthesis; Lipid metabolism; Membrane; Plant defense; Reference proteome; Sphingolipid metabolism; Transferase; Transmembrane; Transmembrane helix
SRR1271859_TR37381_c0_g1_i2_len=2460	NRAM2_ARATH	Q:465-1685,H:45-454	79.02%ID	E:0	Metal transporter Nramp2	PF01566.15^Nramp^Natural resistance-associated macrophage protein^92-453^E:7.8e-117	1	0,506	1	0,625	0,014	0,729	0,001	0,971	1	-0,861	NRAM2_ARATH	Metal transporter Nramp2 (AtNramp2)	NRAMP2 At1g47240 F8G22.4	Arabidopsis thaliana (Mouse-ear cress)		Complete proteome; Ion transport; Iron; Iron transport; Membrane; Reference proteome; Transmembrane; Transmembrane helix; Transport
SRR1271859_TR38441_c0_g1_i1_len=1240	TSN_CHICK	Q:908-309,H:19-222	41.83%ID	E:7e-41	Translin	PF01997.13^Translin^Translin family^99-291^E:7.8e-46	1	0	0,034	5,699	1	0	0	8,084	1	0	TSN_CHICK	Translin (EC 3.1.-.-) (Component 3 of promoter of RISC) (C3PO)	TSN	Gallus gallus (Chicken)		Complete proteome; DNA-binding; Endonuclease; Hydrolase; Nuclease; Nucleus; RNA-binding; Reference proteome
SRR1271859_TR43978_c0_g3_i26_len=5083	NA	NA	NA	NA	NA	PF07712.9^SURNod19^Stress up-regulated Nod 19^34-333^E:2.6e-142	1	0,661	0,01	2,57	0,01	2,276	0,001	2,738	1	-0,105	.	.	.	.	.	.
SRR1271859_TR44349_c0_g3_i1_len=1114	NA	NA	NA	NA	NA	.	1	0,153	0,132	1,247	0,017	3,862	0,001	4,607	1	1,469	.	.	.	.	.	.
SRR1271859_TR44809_c0_g2_i1_len=1519	NA	NA	NA	NA	NA	PF00364.19^Biotin_lipoyl^Biotin-requiring enzyme^204-276^E:1.4e-07	0,137	-0,448	0,624	-0,163	0,13	0,525	0,001	1,09	0,682	-0,124	.	.	.	.	.	.
SRR1271859_TR45075_c0_g1_i1_len=1421	G2OX2_PEA	Q:150-1154,H:1-340	54.73%ID	E:2e-123	Gibberellin 2-beta-dioxygenase 2	PF14226.3^DIOX_N^non-haem dioxygenase in morphine synthesis N-terminal^17-88^E:1e-16`PF03171.17^2OG-FeII_Oxy^2OG-Fe(II) oxygenase superfamily^174-275^E:2.2e-25	1	1,063	0,017	4,582	0,001	2,122	0	3,303	1	-1,916	G2OX2_PEA	Gibberellin 2-beta-dioxygenase 2 (EC 1.14.11.13) (GA 2-oxidase 2) (Gibberellin 2-beta-hydroxylase 2) (Gibberellin 2-oxidase 2)	GA2OX2	Pisum sativum (Garden pea)	PATHWAY: Plant hormone biosynthesis; gibberellin biosynthesis.	Dioxygenase; Iron; Metal-binding; Oxidoreductase
SRR342017_TR14705_c0_g2_i2_len=1218	Y5188_ARATH	Q:1056-151,H:551-851	57.1%ID	E:2e-119	Probable LRR receptor-like serine/threonine-protein kinase At1g51880	PF00069.22^Pkinase^Protein kinase domain^1-200^E:6.6e-33`PF07714.14^Pkinase_Tyr^Protein tyrosine kinase^2-144^E:7.2e-28	0,524	-0,713	1	-0,1	1	3,321	0	7,088	1	-1,403	Y5188_ARATH	Probable LRR receptor-like serine/threonine-protein kinase At1g51880 (EC 2.7.11.1)	At1g51880 T14L22.9	Arabidopsis thaliana (Mouse-ear cress)		ATP-binding; Complete proteome; Glycoprotein; Kinase; Leucine-rich repeat; Membrane; Nucleotide-binding; Phosphoprotein; Receptor; Reference proteome; Repeat; Serine/threonine-protein kinase; Signal; Transferase; Transmembrane; Transmembrane helix
SRR342017_TR15423_c0_g2_i1_len=659	ZN363_MOUSE	Q:564-301,H:161-247	51.14%ID	E:6e-24	RING finger and CHY zinc finger domain-containing protein 1	.	1	-0,331	0,017	5,879	0,066	5,484	0	6,923	1	0	ZN363_MOUSE	RING finger and CHY zinc finger domain-containing protein 1 (EC 6.3.2.-) (Androgen receptor N-terminal-interacting protein) (CH-rich-interacting match with PLAG1) (E3 ubiquitin-protein ligase Pirh2) (Zinc finger protein 363)	Rchy1 Arnip Chimp Zfp363 Znf363	Mus musculus (Mouse)	PATHWAY: Protein modification; protein ubiquitination.	3D-structure; Complete proteome; Cytoplasm; Ligase; Metal-binding; Nucleus; Reference proteome; Ubl conjugation; Ubl conjugation pathway; Zinc; Zinc-finger
SRR342017_TR20330_c2_g1_i2_len=1059	GLB3_ARATH	Q:214-714,H:1-173	76.88%ID	E:8e-93	Two-on-two hemoglobin-3	PF01152.18^Bac_globin^Bacterial-like globin^26-142^E:1.3e-43	0,535	0,283	0,441	0,309	0,279	0,374	0,001	1,059	0,081	-0,706	GLB3_ARATH	Two-on-two hemoglobin-3 (AtGLB3) (2-on-2 hemoglobin-3)	GLB3 At4g32690 F4D11.110	Arabidopsis thaliana (Mouse-ear cress)		3D-structure; Complete proteome; Heme; Iron; Metal-binding; Oxygen transport; Reference proteome; Transport
SRR342017_TR21297_c0_g1_i1_len=1119	UMP1_ARATH	Q:250-639,H:72-201	64.12%ID	E:2e-49	.	.	1	-2,211	0,017	4,118	1	-3,463	0	3,522	1	0,005	MORF8_ARATH	Multiple organellar RNA editing factor 8, chloroplastic/mitochondrial (RNA editing-interacting protein 1)	MORF8 RIP1 At3g15000 K15M2.14	Arabidopsis thaliana (Mouse-ear cress)		Chloroplast; Complete proteome; Direct protein sequencing; Mitochondrion; Plastid; RNA editing; Reference proteome; Transit peptide
SRR342017_TR23423_c0_g6_i2_len=2301	P2C25_ARATH	Q:1538-363,H:1-396	56.76%ID	E:2e-128	Probable protein phosphatase 2C 25	PF00481.18^PP2C^Protein phosphatase 2C^137-381^E:2.4e-58	1	0,18	0,014	2,991	0,187	1,068	0	2,726	1	-0,194	P2C25_ARATH	Probable protein phosphatase 2C 25 (AtPP2C25) (EC 3.1.3.16) (Protein phosphatase AP2C1)	At2g30020 F23F1.6	Arabidopsis thaliana (Mouse-ear cress)		Complete proteome; Cytoplasm; Hydrolase; Magnesium; Manganese; Metal-binding; Nucleus; Protein phosphatase; Reference proteome
SRR342017_TR24288_c0_g1_i1_len=1587	FABG_CUPLA	Q:1369-[illegible]	77.58%ID	E:1e-148	3-oxoacyl-[acyl-carrier-protein] reductase, [illegible]	PF00106.22^adh_short^short chain dehydrogenase^83-275^E:1.4e-65`PF08659.7^KR^KR domain^84-237^E:2.7e-20`PF13561.3^adh_short_C2^Enoyl-[illegible]	0,316	-0,266	0,717	-0,11	0,006	0,769	0	1,598	0,895	0,038	FABG_CUPLA	3-oxoacyl-[acyl-carrier-protein] reductase, chloroplastic (EC 1.1.1.100) (3-ketoacyl-acyl carrier protein [illegible]	CLKR27	Cuphea lanceolata (Cigar flower)	PATHWAY: Lipid metabolism; fatty acid biosynthesis.	Chloroplast; Fatty acid biosynthesis; Fatty acid metabolism; Lipid biosynthesis; Lipid metabolism; [illegible]

[illegible]	[illegible]	595,H:1-320	[illegible]	[illegible]	chloroplastic	(Acyl carrier protein) reductase^88-322^E:4.3e-67	[illegible]	[illegible]	[illegible]	[illegible]	[illegible]	[illegible]	[illegible]	[illegible]	[illegible]	[illegible]	[illegible]	reductase)	[illegible]	[illegible]	[illegible]	NADP; Oxidoreductase; Plastid; Transit peptide
SRR342017_TR31846_c0_g2_i1_len=776	NA	NA	NA	NA	NA	.	0,593	0,615	0,031	1,28	0,007	2,21	0	2,64	1	0,392	.	.	.	.	.	.
SRR342019_TR4570_c1_g1_i1_len=1375	CAP13_ARATH	Q:121-1230,H:1-332	42.47%ID	E:3e-94	Putative clathrin assembly protein At1g25240	PF07651.13^ANTH^ANTH domain^33-293^E:2.4e-49	1	0,574	0,239	1,352	0,017	2,376	0	3,117	1	-0,021	CAP13_ARATH	Putative clathrin assembly protein At1g25240	At1g25240 F4F7.37	Arabidopsis thaliana (Mouse-ear cress)		Coated pit; Complete proteome; Cytoplasmic vesicle; Endocytosis; Golgi apparatus; Membrane; Reference proteome
SRR342019_TR4682_c0_g1_i2_len=2781	MFPA_CUCSA	Q:166-2298,H:4-714	80.59%ID	E:0	Glyoxysomal fatty acid beta-oxidation multifunctional protein MFP-a	PF00378.17^ECH_1^Enoyl-CoA hydratase/isomerase^68-263^E:8.3e-40`PF16113.2^ECH_2^Enoyl-CoA hydratase/isomerase^80-246^E:4e-24`PF02737.15^3HCDH_N^3-hydroxyacyl-CoA dehydrogenase, NAD binding domain^366-544^E:1.9e-60`PF00725.19^3HCDH^3-hydroxyacyl-CoA dehydrogenase, C-terminal domain^547-640^E:1.1e-20	1	-0,423	0,118	2,113	0,009	3,854	0	4,531	1	0,451	MFPA_CUCSA	Glyoxysomal fatty acid beta-oxidation multifunctional protein MFP-a [Includes: Enoyl-CoA hydratase/3-2-trans-enoyl-CoA isomerase/3-hydroxybutyryl-CoA epimerase (EC 4.2.1.17) (EC 5.1.2.3) (EC 5.3.3.8); 3-hydroxyacyl-CoA dehydrogenase (EC 1.1.1.35)]		Cucumis sativus (Cucumber)	PATHWAY: Lipid metabolism; fatty acid beta-oxidation.	Fatty acid metabolism; Glyoxysome; Isomerase; Lipid metabolism; Lyase; Multifunctional enzyme; NAD; Oxidoreductase; Peroxisome
SRR342019_TR10250_c0_g1_i4_len=2875	NA	NA	NA	NA	NA	.	0,5	0,691	0,299	1,413	0,004	3,328	0	3,982	0,461	-1,111	.	.	.	.	.	.
SRR342019_TR10559_c0_g4_i2_len=2559	TT12_ARATH	Q:958-2340,H:45-505	39.83%ID	E:6e-93	.	PF01554.15^MatE^MatE^51-211^E:6.5e-29`PF01554.15^MatE^MatE^274-433^E:6.7e-28	0,798	0,287	1	0,063	0,002	1,561	0	2,129	1	-0,078	DTX41_ARATH	Protein DETOXIFICATION 41 (AtDTX41) (Multidrug and toxic compound extrusion protein 41) (MATE protein 41) (Protein TANNIN-DEFICIENT SEED 3) (Protein TRANSPARENT TESTA 12)	DTX41 TDS3 TT12 At3g59030 F17J16_80	Arabidopsis thaliana (Mouse-ear cress)	PATHWAY: Secondary metabolite biosynthesis; flavonoid biosynthesis.	Antiport; Complete proteome; Flavonoid biosynthesis; Membrane; Reference proteome; Transmembrane; Transmembrane helix; Transport; Vacuole
SRR342019_TR10890_c5_g1_i2_len=1480	PDCB3_ARATH	Q:1027-827,H:20-86	71.64%ID	E:4e-30	PLASMODESMATA CALLOSE-BINDING PROTEIN 3	PF07983.10^X8^X8 domain^21-92^E:1.8e-19	0,122	-0,411	0,541	0,2	0,001	1,113	0,001	1,17	0,732	-0,549	PDCB3_ARATH	PLASMODESMATA CALLOSE-BINDING PROTEIN 3 (AtPDCB3)	PDCB3 At1g18650 F25I16.1	Arabidopsis thaliana (Mouse-ear cress)		Cell junction; Cell membrane; Complete proteome; Disulfide bond; GPI-anchor; Glycoprotein; Lipoprotein; Membrane; Reference proteome; Signal
SRR342019_TR18451_c0_g1_i2_len=1723	F10AL_ARATH	Q:1470-736,H:1-243	65.85%ID	E:6e-87	FAM10 family protein At4g22670	PF13181.3^TPR_8^Tetratricopeptide repeat^134-157^E:0.064`PF00515.25^TPR_1^Tetratricopeptide repeat^161-191^E:1.2e-06`PF13181.3^TPR_8^Tetratricopeptide repeat^161-191^E:0.0012`PF13432.3^TPR_16^Tetratricopeptide repeat^164-215^E:0.0048	0,223	-0,401	0,558	-0,233	0,002	1,022	0,001	1,125	1	-0,705	F10AL_ARATH	FAM10 family protein At4g22670	At4g22670 T12H17.60	Arabidopsis thaliana (Mouse-ear cress)		Coiled coil; Complete proteome; Direct protein sequencing; Phosphoprotein; Reference proteome; Repeat; TPR repeat
SRR342019_TR18877_c0_g1_i4_len=2587	AZG1_ARATH	Q:2519-837,H:10-567	77.9%ID	E:0	Adenine/guanine permease AZG1	PF00860.17^Xan_ur_permease^Permease family^141-545^E:2.9e-24	1	0,067	0,643	0,61	0,021	1,576	0,001	2,119	1	-0,174	AZG1_ARATH	Adenine/guanine permease AZG1 (AzgA-homolog protein) (Protein AZAGUANINE RESISTANT 1) (AtAzg1)	AZG1 At3g10960 F9F8.22	Arabidopsis thaliana (Mouse-ear cress)		Complete proteome; Membrane; Reference proteome; Transmembrane; Transmembrane helix; Transport
SRR342019_TR31828_c0_g1_i2_len=1317	SDR1_PICAB	Q:926-162,H:17-271	60.55%ID	E:6e-102	Short-chain type dehydrogenase/reductase	PF00106.22^adh_short^short chain dehydrogenase^19-213^E:7.6e-42`PF08659.7^KR^KR domain^21-191^E:7.3e-11`PF13561.3^adh_short_C2^Enoyl-(Acyl carrier protein) reductase^24-266^E:1.1e-58	0,528	0,327	0,005	1,116	0,002	1,563	0	1,874	1	0,381	SDR1_PICAB	Short-chain type dehydrogenase/reductase (EC 1.-.-.-)		Picea abies (Norway spruce) (Picea excelsa)		Oxidoreductase
SRR342019_TR35444_c0_g2_i1_len=1748	NA	NA	NA	NA	NA	.	0,505	-0,491	0,679	-0,461	0,049	1,34	0,001	1,967	0,319	0,9	.	.	.	.	.	.
SRR342019_TR38237_c0_g1_i1_len=515	NA	NA	NA	NA	NA	.	1	0,671	0,017	4,02	0,017	4,423	0	5,787	0,629	1,99	.	.	.	.	.	.
SRR342019_TR39237_c0_g2_i1_len=2418	NA	NA	NA	NA	NA	PF00168.27^C2^C2 domain^16-68^E:5.7e-06`PF00168.27^C2^C2 domain^140-246^E:2.7e-17`PF00168.27^C2^C2 domain^302-373^E:5.1e-16	1	0,345	0,008	2,962	0,002	2,017	0	2,961	1	1,988	.	.	.	.	.	.
SRR342022_TR667_c1_g1_i1_len=2615	NA	NA	NA	NA	NA	.	0,317	-0,498	1	0,037	0,029	1,21	0	1,822	0,77	0,26	.	.	.	.	.	.
SRR342022_TR985_c10_g1_i1_len=1176	MYC2_ARATH	Q:256-849,H:64-253	37.37%ID	E:3e-40	Transcription factor MYC2	PF14215.3^bHLH-MYC_N^bHLH-MYC and R2R3-MYB transcription factors N-terminal^16-208^E:2.9e-52	0,037	0,927	0,01	1,734	0,002	1,014	0	1,402	1	0,668	MYC2_ARATH	Transcription factor MYC2 (AtMYC2) (Basic helix-loop-helix protein 6) (AtbHLH6) (bHLH 6) (Protein JASMONATE INSENSITIVE 1) (R-homologous Arabidopsis protein 1) (RAP-1) (Transcription factor EN 38) (Z-box binding factor 1 protein) (bHLH transcription factor bHLH006) (rd22BP1)	MYC2 BHLH6 EN38 JAI1 JIN1 RAP1 RD22BP1 ZBF1 At1g32640 F6N18.4	Arabidopsis thaliana (Mouse-ear cress)		Abscisic acid signaling pathway; Activator; Complete proteome; DNA-binding; Jasmonic acid signaling pathway; Nucleus; Phosphoprotein; Reference proteome; Transcription; Transcription regulation
SRR342022_TR3295_c2_g3_i1_len=3147	BGAL_SOLLC	Q:2717-219,H:3-835	83.43%ID	E:0	Beta-galactosidase	PF01301.16^Glyco_hydro_35^Glycosyl hydrolases family 35^36-341^E:6.2e-118`PF02449.12^Glyco_hydro_42^Beta-galactosidase^57-205^E:6.2e-10`PF02140.15^Gal_Lectin^Galactose binding lectin domain^764-840^E:1.4e-20	0,52	0,428	0,239	1,554	0,005	1,527	0,001	1,797	0,629	0,981	BGAL_SOLLC	Beta-galactosidase (EC 3.2.1.23) (Acid beta-galactosidase) (Lactase) (Exo-(1-->4)-beta-D-galactanase)		Solanum lycopersicum (Tomato) (Lycopersicon esculentum)		Complete proteome; Direct protein sequencing; Glycosidase; Hydrolase; Reference proteome; Signal
SRR342022_TR10678_c0_g1_i1_len=1206	NA	NA	NA	NA	NA	.	0,502	4,179	1	0	0,009	6,028	0	6,795	1	-0,142	.	.	.	.	.	.
SRR342022_TR11701_c0_g1_i1_len=1560	NA	NA	NA	NA	NA	PF02485.18^Branch^Core-2/I-Branching enzyme^127-321^E:3.1e-71	0,461	1,501	1	-0,394	0,005	5,352	0	5,94	1	0,044	.	.	.	.	.	.
SRR342022_TR12749_c1_g9_i1_len=2782	SPEA_BACSU	Q:221-1231,H:148-474	34.41%ID	E:2e-43	Arginine decarboxylase	PF05889.10^SepSecS^O-phosphoseryl-tRNA(Sec) selenium transferase, SepSecS^8-77^E:0.00021`PF01276.17^OKR_DC_1^Orn/Lys/Arg decarboxylase, major domain^11-218^E:2.3e-27`PF03711.12^OKR_DC_1_C^Orn/Lys/Arg decarboxylase, C-terminal domain^259-321^E:6.7e-09	1	1,051	0,129	5,144	0,017	5,755	0	7,079	1	2,41	SPEA_BACSU	Arginine decarboxylase (EC 4.1.1.19)	speA cad BSU14630	Bacillus subtilis (strain 168)	PATHWAY: Amine and polyamine biosynthesis; agmatine biosynthesis; agmatine from L-arginine: step 1/1.	Complete proteome; Cytoplasm; Decarboxylase; Lyase; Polyamine biosynthesis; Putrescine biosynthesis; Pyridoxal phosphate; Reference proteome; Spermidine biosynthesis
SRR342022_TR13042_c0_g2_i1_len=1816	PP145_ARATH	Q:77-1783,H:31-603	59.93%ID	E:0	Pentatricopeptide repeat-containing protein At2g02980, chloroplastic {ECO:0000305}	PF13812.3^PPR_3^Pentatricopeptide repeat domain^188-228^E:0.011`PF13041.3^PPR_2^PPR repeat family^189-236^E:1.2e-10`PF01535.17^PPR^PPR repeat^192-220^E:6.7e-07`PF01535.17^PPR^PPR repeat^265-287^E:0.00052`PF01535.17^PPR^PPR repeat^294-321^E:3.1e-05`PF01535.17^PPR^PPR repeat^437-460^E:0.014`PF14432.3^DYW_deaminase^DYW family of nucleic acid deaminases^464-584^E:4.8e-28	1	-0,04	1	0,449	0,005	3,941	0	6,025	1	0,285	PP145_ARATH	Pentatricopeptide repeat-containing protein At2g02980, chloroplastic (Protein ORGANELLE TRANSCRIPT PROCESSING 85)	PCMP-H26 OTP85 At2g02980 T17M13.15	Arabidopsis thaliana (Mouse-ear cress)		Chloroplast; Complete proteome; Plastid; Reference proteome; Repeat; Transit peptide; mRNA processing
SRR342022_TR15143_c0_g1_i2_len=1152	VPS25_ARATH	Q:344-856,H:7-179	75.14%ID	E:6e-92	Vacuolar protein sorting-associated protein 25	.	0,864	0,096	0,088	0,597	0,014	1,184	0,001	1,511	0,866	0,065	VPS25_ARATH	Vacuolar protein sorting-associated protein 25 (AtVPS25) (ESCRT-II complex subunit VPS25)	VPS25 At4g19003 F13C5	Arabidopsis thaliana (Mouse-ear cress)		Complete proteome; Endosome; Protein transport; Reference proteome; Transport
SRR342022_TR18873_c0_g3_i2_len=1092	NA	NA	NA	NA	NA	.	0,075	0,828	0,069	0,491	0,016	0,716	0	1,356	0,842	0,137	.	.	.	.	.	.
SRR342022_TR21147_c3_g1_i6_len=1877	IPCS2_ARATH	Q:596-1525,H:1-305	77.74%ID	E:3e-176	Phosphatidylinositol:ceramide inositolphosphotransferase 2	PF14360.3^PAP2_C^PAP2 superfamily C-terminal^172-240^E:1.3e-22	1	0,016	0,133	1,155	0,152	0,816	0,001	1,705	0,707	0,443	IPCS2_ARATH	Phosphatidylinositol:ceramide inositolphosphotransferase 2 (EC 2.7.8.-) (Inositol-phosphorylceramide synthase 2) (AtIPCS2) (IPC synthase 2) (Protein ENHANCING RPW8-MEDIATED HR-LIKE CELL DEATH 1) (Sphingolipid synthase 2)	IPCS2 ERH1 At2g37940 T8P21.15	Arabidopsis thaliana (Mouse-ear cress)		Complete proteome; Golgi apparatus; Lipid biosynthesis; Lipid metabolism; Membrane; Plant defense; Reference proteome; Sphingolipid metabolism; Transferase; Transmembrane; Transmembrane helix
SRR342022_TR23948_c0_g1_i1_len=1758	ITPK1_MAIZE	Q:1404-481,H:18-324	58.58%ID	E:3e-130	Inositol-tetrakisphosphate 1-kinase 1	PF05770.8^Ins134_P3_kin^Inositol 1, 3, 4-trisphosphate 5/6-kinase^4-311^E:2.8e-125	0,005	1,47	0,043	1,283	0,003	2,241	0	2,589	0,822	-0,219	ITPK1_MAIZE	Inositol-tetrakisphosphate 1-kinase 1 (EC 2.7.1.134) (Inositol 1,3,4-trisphosphate 5/6-kinase 1) (Inositol-triphosphate 5/6-kinase 1) (Ins(1,3,4)P(3) 5/6-kinase 1) (EC 2.7.1.159) (Low phytic acid protein 2) (ZmIpk)	ITPK1 LPA2	Zea mays (Maize)		ATP-binding; Complete proteome; Kinase; Magnesium; Metal-binding; Nucleotide-binding; Reference proteome; Transferase
SRR342022_TR26348_c3_g6_i3_len=1381	MECR_ARATH	Q:26-1189,H:11-375	70.1%ID	E:0	Probable trans-2-enoyl-CoA reductase, mitochondrial	PF08240.9^ADH_N^Alcohol dehydrogenase GroES-like domain^74-135^E:1.8e-07`PF00107.23^ADH_zinc_N^Zinc-binding dehydrogenase^199-334^E:6.9e-17	0,77	0,129	0,044	1,19	0,001	1,451	0,001	1,505	0,666	-0,304	MECR_ARATH	Probable trans-2-enoyl-CoA reductase, mitochondrial (EC 1.3.1.38)	At3g45770 T6D9.100	Arabidopsis thaliana (Mouse-ear cress)		Alternative splicing; Complete proteome; Fatty acid biosynthesis; Fatty acid metabolism; Lipid biosynthesis; Lipid metabolism; Mitochondrion; NADP; Oxidoreductase; Reference proteome; Transit peptide
SRR342022_TR26609_c1_g19_i2_len=4014	NA	NA	NA	NA	NA	PF07724.11^AAA_2^AAA domain (Cdc48 subfamily)^744-880^E:9.1e-07	0,87	0,159	0,025	1,435	0,009	1,64	0	2,085	0,147	0,999	.	.	.	.	.	.
SRR342022_TR27537_c0_g3_i1_len=1748	NA	NA	NA	NA	NA	.	0,731	0,115	0,085	0,464	0,004	0,881	0	1,095	0,41	0,264	.	.	.	.	.	.
SRR342022_TR28115_c4_g2_i2_len=3376	ACCA_ARATH	Q:424-2499,H:14-717	62.75%ID	E:0	Acetyl-coenzyme A carboxylase carboxyl transferase subunit alpha, chloroplastic	PF03255.11^ACCA^Acetyl co-enzyme A carboxylase carboxyltransferase alpha subunit^99-241^E:6e-54`PF01039.19^Carboxyl_trans^Carboxyl transferase domain^178-339^E:6.8e-09	0,24	-0,334	0,19	0,41	0,281	0,337	0	1,101	0,619	0,16	ACCA_ARATH	Acetyl-coenzyme A carboxylase carboxyl transferase subunit alpha, chloroplastic (ACCase subunit alpha) (Acetyl-CoA carboxylase carboxyltransferase subunit alpha) (EC 6.4.1.2)	CAC3 At2g38040 T8P21.5	Arabidopsis thaliana (Mouse-ear cress)	PATHWAY: Lipid metabolism; malonyl-CoA biosynthesis; malonyl-CoA from acetyl-CoA: step 1/1.	ATP-binding; Chloroplast; Coiled coil; Complete proteome; Disulfide bond; Fatty acid biosynthesis; Fatty acid metabolism; Ligase; Lipid biosynthesis; Lipid metabolism; Membrane; Nucleotide-binding; Phosphoprotein; Plastid; Plastid inner membrane; Reference proteome; Transit peptide
SRR342022_TR30015_c0_g1_i2_len=725	NA	NA	NA	NA	NA	.	0,945	-0,043	0,07	-0,642	0,057	0,771	0	1,31	0,471	-0,561	.	.	.	.	.	.
SRR342022_TR33011_c0_g1_i1_len=591	NA	NA	NA	NA	NA	.	1	0,32	0,254	3,427	0,158	1,767	0	2,995	0,502	3,19	.	.	.	.	.	.
SRR342023_TR5185_c1_g1_i1_len=1438	CLPP4_ARATH	Q:1167-487,H:67-292	81.94%ID	E:1e-125	ATP-dependent Clp protease proteolytic subunit 4, chloroplastic {ECO:0000303|PubMed:11299370}	PF00574.20^CLP_protease^Clp protease^102-276^E:3.8e-70	0,2	-0,403	0,406	-0,316	0,004	1,193	0	1,417	1	-0,318	CLPP4_ARATH	ATP-dependent Clp protease proteolytic subunit 4, chloroplastic (EC 3.4.21.92) (Endopeptidase ClpP4) (nClpP4) (nClpP3)	CLPP4 NCLPP3 NCLPP4 At5g45390 MFC19.5	Arabidopsis thaliana (Mouse-ear cress)		3D-structure; Acetylation; Chloroplast; Complete proteome; Direct protein sequencing; Hydrolase; Plastid; Protease; Reference proteome; Serine protease; Transit peptide; Ubl conjugation
SRR342023_TR11224_c0_g2_i1_len=1170	PNAE_RAUSE	Q:223-774,H:5-189	45.41%ID	E:4e-52	Polyneuridine-aldehyde esterase	PF00561.17^Abhydrolase_1^alpha/beta hydrolase fold^45-167^E:2.9e-14`PF12146.5^Hydrolase_4^Serine aminopeptidase, S33^45-158^E:4.7e-12`PF12697.4^Abhydrolase_6^Alpha/beta hydrolase family^46-150^E:1.4e-11	0,788	0,221	1	0,048	0,003	1,768	0,001	1,907	0,195	0,856	PNAE_RAUSE	Polyneuridine-aldehyde esterase (EC 3.1.1.78) (Polyneuridine aldehyde esterase)	PNAE	Rauvolfia serpentina (Serpentine wood) (Ophioxylon serpentinum)	PATHWAY: Alkaloid biosynthesis; ajmaline biosynthesis.	3D-structure; Alkaloid metabolism; Direct protein sequencing; Hydrolase; Serine esterase
SRR342023_TR11881_c0_g2_i1_len=719	RD19A_ARATH	Q:186-716,H:25-198	73.45%ID	E:1e-88	Cysteine proteinase RD19a	PF08246.9^Inhibitor_I29^Cathepsin propeptide inhibitor domain (I29)^57-113^E:1.1e-09`PF00112.20^Peptidase_C1^Papain family cysteine protease^144-202^E:5.2e-24	0,11	-0,469	1	2,449	0,502	3,844	0	7,075	0,502	2,48	RD19A_ARATH	Cysteine proteinase RD19a (RD19) (EC 3.4.22.-)	RD19A At4g39090 F19H22.190	Arabidopsis thaliana (Mouse-ear cress)		Complete proteome; Disulfide bond; Glycoprotein; Hydrolase; Protease; Reference proteome; Signal; Thiol protease; Zymogen
SRR342023_TR15682_c2_g8_i8_len=3256	NA	NA	NA	NA	NA	.	0,502	3,949	0,502	4,237	0,066	4,027	0,001	5,221	1	1,928	.	.	.	.	.	.
SRR342023_TR20558_c0_g1_i3_len=2993	ADO3_ARATH	Q:747-2486,H:46-618	83.48%ID	E:0	Adagio protein 3	PF00989.22^PAS^PAS fold^61-166^E:6.3e-06`PF13426.4^PAS_9^PAS domain^66-167^E:1.3e-14`PF00646.30^F-box^F-box domain^216-257^E:5.4e-05`PF13418.3^Kelch_4^Galactose oxidase, central domain^310-360^E:3.8e-11`PF13415.3^Kelch_3^Galactose oxidase, central domain^319-368^E:4.7e-07`PF13418.3^Kelch_4^Galactose oxidase, central domain^361-411^E:8.2e-09`PF13415.3^Kelch_3^Galactose oxidase, central domain^375-421^E:1e-11`PF13418.3^Kelch_4^Galactose oxidase, central domain^413-461^E:1.8e-07`PF13415.3^Kelch_3^Galactose oxidase, central domain^424-475^E:1e-09`PF07646.12^Kelch_2^Kelch motif^534-581^E:1.2e-05	0,699	-0,157	0,336	-0,329	0,502	3,119	0	5,937	0,411	0,301	ADO3_ARATH	Adagio protein 3 (F-box only protein 2a) (FBX2a) (Flavin-binding kelch repeat F-box protein 1)	ADO3 FKF1 At1g68050 T23K23.10	Arabidopsis thaliana (Mouse-ear cress)	PATHWAY: Protein modification; protein ubiquitination.	Biological rhythms; Chromophore; Complete proteome; Cytoplasm; FMN; Flavoprotein; Flowering; Kelch repeat; Nucleus; Photoreceptor protein; Receptor; Reference proteome; Repeat; Sensory transduction; Ubl conjugation pathway
SRR342023_TR23730_c2_g6_i1_len=1528	ASIL2_ARATH	Q:1528-926,H:1-206	56.76%ID	E:2e-52	Trihelix transcription factor ASIL2 {ECO:0000305}	PF13837.3^Myb_DNA-bind_4^Myb/SANT-like DNA-binding domain^67-158^E:7.2e-26	0,729	0,272	0,278	0,75	0,01	1,302	0,001	1,616	0,066	3,821	ASIL2_ARATH	Trihelix transcription factor ASIL2 (6B-interacting protein 1-like 2) (Trihelix DNA-binding protein ASIL2)	ASIL2 At3g14180 MAG2.16	Arabidopsis thaliana (Mouse-ear cress)		Coiled coil; Complete proteome; DNA-binding; Nucleus; Reference proteome; Repressor; Transcription; Transcription regulation
SRR342023_TR31226_c2_g9_i9_len=7814	AB10C_ARATH	Q:5608-1187,H:2-1451	65.72%ID	E:0	ABC transporter C family member 10	PF00664.20^ABC_membrane^ABC transporter transmembrane region^310-569^E:7.2e-23`PF00005.24^ABC_tran^ABC transporter^644-778^E:1.6e-24`PF00664.20^ABC_membrane^ABC transporter transmembrane region^918-1178^E:1.3e-25`PF00005.24^ABC_tran^ABC transporter^1252-1399^E:9.6e-30	0,763	0,245	0,068	1,019	0,01	0,867	0	1,29	1	0,037	AB10C_ARATH	ABC transporter C family member 10 (ABC transporter ABCC.10) (AtABCC10) (EC 3.6.3.44) (ATP-energized glutathione S-conjugate pump 14) (Glutathione S-conjugate-transporting ATPase 14) (Multidrug resistance-associated protein 14)	ABCC10 MRP10 MRP14 At3g59140 F17J16.190	Arabidopsis thaliana (Mouse-ear cress)		ATP-binding; Complete proteome; Hydrolase; Membrane; Nucleotide-binding; Reference proteome; Repeat; Transmembrane; Transmembrane helix; Transport
SRR646572_TR13187_c0_g1_i1_len=1315	M3K2_ARATH	Q:1172-333,H:68-347	39.06%ID	E:2e-54	Mitogen-activated protein kinase kinase kinase 2	PF00069.22^Pkinase^Protein kinase domain^6-266^E:1.4e-55`PF07714.14^Pkinase_Tyr^Protein tyrosine kinase^8-261^E:1.7e-35	0,629	1,916	0,025	3,685	0,01	2,102	0	3,226	1	0,28	M3K2_ARATH	Mitogen-activated protein kinase kinase kinase 2 (EC 2.7.11.25) (Arabidopsis NPK1-related protein kinase 2)	ANP2 At1g54960 F14C21.49 T24C10.7	Arabidopsis thaliana (Mouse-ear cress)		ATP-binding; Coiled coil; Complete proteome; Cytoplasm; Cytoskeleton; Kinase; Microtubule; Nucleotide-binding; Reference proteome; Serine/threonine-protein kinase; Transferase
SRR646572_TR20908_c0_g2_i1_len=1888	GME1_ORYSI	Q:1632-505,H:1-378	92.59%ID	E:0	GDP-mannose 3,5-epimerase 1	PF01370.18^Epimerase^NAD dependent epimerase/dehydratase family^30-269^E:6.2e-46`PF16363.2^GDP_Man_Dehyd^GDP-mannose 4,6 dehydratase^32-329^E:9.7e-28`PF01073.16^3Beta_HSD^3-beta hydroxysteroid dehydrogenase/isomerase family^32-185^E:1.6e-09	0,212	-0,396	0,942	-0,042	0,001	1,427	0	1,595	0,807	-0,149	GME1_ORYSI	GDP-mannose 3,5-epimerase 1 (GDP-Man 3,5-epimerase 1) (EC 5.1.3.18) (OsGME-1)	OsI_032456	Oryza sativa subsp. indica (Rice)	PATHWAY: Cofactor biosynthesis; L-ascorbate biosynthesis via GDP-alpha-D-mannose pathway; L-ascorbate from GDP-alpha-D-mannose: step 1/5. {ECO:0000269|PubMed:9620799}.	Ascorbate biosynthesis; Complete proteome; Isomerase; NAD; Reference proteome
SRR646572_TR21312_c0_g1_i1_len=829	NA	NA	NA	NA	NA	.	0,648	0,196	0,182	0,535	0,002	1,209	0,001	1,317	0,388	-0,351	.	.	.	.	.	.
SRR646572_TR32716_c0_g1_i2_len=2150	ACCC1_POPTR	Q:134-1708,H:9-528	89.9%ID	E:0	Biotin carboxylase 1, chloroplastic	PF00289.19^Biotin_carb_N^Biotin carboxylase, N-terminal domain^71-178^E:1.3e-40`PF02786.14^CPSase_L_D2^Carbamoyl-phosphate synthase L chain, ATP binding domain^183-390^E:6e-83`PF02222.19^ATP-grasp^ATP-grasp domain^205-360^E:6e-10`PF07478.10^Dala_Dala_lig_C^D-ala D-ala ligase C-terminus^209-359^E:9.5e-08`PF02785.16^Biotin_carb_C^Biotin carboxylase C-terminal domain^404-509^E:4.2e-39	0,134	-0,406	0,031	-0,628	0,157	0,445	0	1,398	0,935	-0,029	ACCC1_POPTR	Biotin carboxylase 1, chloroplastic (EC 6.3.4.14) (Acetyl-CoA carboxylase subunit A 1) (ACC) (EC 6.4.1.2)	POPTRDRAFT_831870	Populus trichocarpa (Western balsam poplar) (Populus balsamifera subsp. trichocarpa)	PATHWAY: Lipid metabolism; malonyl-CoA biosynthesis; malonyl-CoA from acetyl-CoA: step 1/1.	ATP-binding; Biotin; Chloroplast; Complete proteome; Fatty acid biosynthesis; Fatty acid metabolism; Ligase; Lipid biosynthesis; Lipid metabolism; Magnesium; Manganese; Metal-binding; Nucleotide-binding; Plastid; Reference proteome; Transit peptide
SRR646596_TR1350_c2_g1_i1_len=2436	HTSF1_HUMAN	Q:550-1524,H:48-355	34.43%ID	E:3e-39	HIV Tat-specific factor 1	PF14237.3^DUF4339^Domain of unknown function (DUF4339)^29-73^E:1.8e-13`PF00076.19^RRM_1^RNA recognition motif. (a.k.a. RRM, RBD, or RNP domain)^249-321^E:4.2e-09`PF13893.3^RRM_5^RNA recognition motif. (a.k.a. RRM, RBD, or RNP domain)^294-336^E:0.56`PF00076.19^RRM_1^RNA recognition motif. (a.k.a. RRM, RBD, or RNP domain)^402-457^E:7.6e-10`PF13893.3^RRM_5^RNA recognition motif. (a.k.a. RRM, RBD, or RNP domain)^413-457^E:0.55	1	0,484	1	1,205	0,034	4,855	0	5,966	0,692	-1,257	HTSF1_HUMAN	HIV Tat-specific factor 1 (Tat-SF1)	HTATSF1	Homo sapiens (Human)		3D-structure; Acetylation; Activator; Complete proteome; Direct protein sequencing; Nucleus; Phosphoprotein; Polymorphism; RNA-binding; Reference proteome; Repeat; Transcription; Transcription regulation
SRR646596_TR5568_c0_g1_i1_len=913	PTI5_SOLLC	Q:176-622,H:1-143	63.46%ID	E:2e-42	Pathogenesis-related genes transcriptional activator PTI5	PF00847.17^AP2^AP2 domain^66-116^E:1.2e-14	1	1,573	0,502	3,511	0,009	6,032	0	6,75	1	0,595	PTI5_SOLLC	Pathogenesis-related genes transcriptional activator PTI5 (PTO-interacting protein 5)	PTI5	Solanum lycopersicum (Tomato) (Lycopersicon esculentum)		Activator; Complete proteome; DNA-binding; Nucleus; Plant defense; Reference proteome; Transcription; Transcription regulation
SRR646596_TR7456_c0_g1_i2_len=1909	NAC2_ARATH	Q:543-1283,H:54-289	59.22%ID	E:2e-76	NAC domain-containing protein 2	PF02365.12^NAM^No apical meristem (NAM) protein^9-77^E:2e-14	0,629	0,937	0,025	3,066	0,025	3,647	0	4,685	0,692	-0,682	NAC2_ARATH	NAC domain-containing protein 2 (ANAC002)	NAC002 ATAF1 At1g01720 T1N6.12	Arabidopsis thaliana (Mouse-ear cress)		Complete proteome; DNA-binding; Nucleus; Reference proteome; Transcription; Transcription regulation
SRR646596_TR15471_c0_g1_i1_len=5321	MNS3_ARATH	Q:233-2089,H:1-624	68.84%ID	E:0	Mannosyl-oligosaccharide 1,2-alpha-mannosidase MNS3	PF00520.28^Ion_trans^Ion transport protein^60-307^E:9.1e-37`PF07885.13^Ion_trans_2^Ion channel^208-300^E:2.1e-11`PF00027.26^cNMP_binding^Cyclic nucleotide-binding domain^398-482^E:9.3e-15`PF11834.5^KHA^KHA, dimerisation domain of potassium ion channel^698-767^E:4.2e-22	0,16	1,202	0,212	0,924	0,003	1,796	0	2,173	0,014	-2,735	MNS3_ARATH	Mannosyl-oligosaccharide 1,2-alpha-mannosidase MNS3 (EC 3.2.1.113)	MNS3 At1g30000 T1P2.10	Arabidopsis thaliana (Mouse-ear cress)	PATHWAY: Protein modification; protein glycosylation.	Calcium; Complete proteome; Disulfide bond; Glycoprotein; Golgi apparatus; Hydrolase; Magnesium; Manganese; Membrane; Reference proteome; Signal-anchor; Transmembrane; Transmembrane helix
SRR646596_TR16289_c0_g1_i1_len=3534	PP377_ARATH	Q:3133-710,H:25-822	55.87%ID	E:0	Putative pentatricopeptide repeat-containing protein At5g13230, mitochondrial	PF01535.17^PPR^PPR repeat^98-125^E:0.0034`PF01535.17^PPR^PPR repeat^127-156^E:0.0021`PF01535.17^PPR^PPR repeat^200-223^E:0.37`PF13041.3^PPR_2^PPR repeat family^225-271^E:1.5e-07`PF01535.17^PPR^PPR repeat^228-257^E:0.00016`PF13041.3^PPR_2^PPR repeat family^327-375^E:2e-07`PF01535.17^PPR^PPR repeat^331-356^E:0.014`PF01535.17^PPR^PPR repeat^402-427^E:0.0012`PF13041.3^PPR_2^PPR repeat family^428-475^E:1.3e-11`PF01535.17^PPR^PPR repeat^430-459^E:1.3e-05`PF01535.17^PPR^PPR repeat^503-529^E:0.00098`PF13041.3^PPR_2^PPR repeat family^528-576^E:1.9e-10`PF13812.3^PPR_3^Pentatricopeptide repeat domain^528-577^E:8.1e-05`PF01535.17^PPR^PPR repeat^531-558^E:4.1e-07`PF01535.17^PPR^PPR repeat^603-627^E:0.0023`PF14432.3^DYW_deaminase^DYW family of nucleic acid deaminases^702-826^E:7.8e-35	1	0,007	0,132	2,615	0,017	4,834	0	5,714	1	-0,595	PP377_ARATH	Putative pentatricopeptide repeat-containing protein At5g13230, mitochondrial	PCMP-H89 At5g13230 T31B5_50	Arabidopsis thaliana (Mouse-ear cress)		Complete proteome; Mitochondrion; Reference proteome; Repeat; Transit peptide
SRR646596_TR30964_c0_g2_i5_len=2491	NA	NA	NA	NA	NA	PF01699.21^Na_Ca_ex^Sodium/calcium exchanger protein^76-244^E:4.6e-07`PF13499.3^EF-hand_7^EF-hand domain pair^293-357^E:5.3e-10`PF13202.3^EF-hand_5^EF hand^295-318^E:4.9e-05`PF01699.21^Na_Ca_ex^Sodium/calcium exchanger protein^418-556^E:4.2e-05	0,814	0,116	0,892	-0,052	0,038	0,97	0	2,128	0,472	-0,738	.	.	.	.	.	.
SRR646596_TR33708_c1_g1_i5_len=501	NA	NA	NA	NA	NA	.	0,013	0,656	0,021	0,513	0,005	0,636	0	1,083	0,022	0,569	.	.	.	.	.	.
SRR646604_TR3399_c0_g1_i1_len=1474	BUP1_DICDI	Q:209-1360,H:5-390	62.69%ID	E:2e-167	Beta-ureidopropionase	PF00795.19^CN_hydrolase^Carbon-nitrogen hydrolase^146-417^E:1.8e-52	0,066	5,226	0,003	2,881	0,002	2,489	0	2,89	0,56	0,727	BUP1_DICDI	Beta-ureidopropionase (EC 3.5.1.6) (Beta-alanine synthase) (N-carbamoyl-beta-alanine amidohydrolase)	pyd3 bup1 upb1 DDB_G0274123	Dictyostelium discoideum (Slime mold)	PATHWAY: Amino-acid biosynthesis; beta-alanine biosynthesis.	Acetylation; Complete proteome; Direct protein sequencing; Hydrolase; Reference proteome
SRR646604_TR3972_c0_g1_i1_len=1748	ZOG_PHALU	Q:90-1472,H:13-459	55.53%ID	E:3e-169	Zeatin O-glucosyltransferase	PF00201.15^UDPGT^UDP-glucoronosyl and UDP-glucosyl transferase^231-391^E:5.9e-22	1	0,541	0,254	4,356	0,009	5,201	0	6,974	1	-1,478	ZOG_PHALU	Zeatin O-glucosyltransferase (EC 2.4.1.203) (Trans-zeatin O-beta-D-glucosyltransferase)	ZOG1	Phaseolus lunatus (Lima bean) (Phaseolus limensis)		Glycosyltransferase; Transferase
SRR646604_TR9559_c0_g1_i3_len=2157	AAP3_ARATH	Q:1733-306,H:3-476	79.62%ID	E:0	Amino acid permease 3	PF01490.15^Aa_trans^Transmembrane amino acid transporter protein^40-473^E:7.7e-118	0,013	1,454	0,076	2,46	0,007	1,379	0	1,756	1	-0,006	AAP3_ARATH	Amino acid permease 3 (Amino acid transporter AAP3)	AAP3 At1g77380 F2P24.9	Arabidopsis thaliana (Mouse-ear cress)		Amino-acid transport; Cell membrane; Complete proteome; Membrane; Nucleus; Reference proteome; Symport; Transmembrane; Transmembrane helix; Transport
SRR646604_TR10280_c1_g4_i2_len=2417	C81E8_MEDTR	Q:1123-2415,H:43-475	45.52%ID	E:4e-126	Cytochrome P450 81E8 {ECO:0000303|PubMed:14617078}	PF00067.19^p450^Cytochrome P450^25-277^E:2.3e-66	0,629	1,065	0,132	2,038	0,002	4,25	0	4,78	1	1,291	C81E8_MEDTR	Cytochrome P450 81E8 (EC 1.14.13.-)	CYP81E8	Medicago truncatula (Barrel medic) (Medicago tribuloides)		Heme; Iron; Membrane; Metal-binding; Monooxygenase; NADP; Oxidoreductase; Transmembrane; Transmembrane helix
SRR646604_TR12851_c0_g1_i1_len=2260	MASY_RICCO	Q:1837-176,H:13-567	88.47%ID	E:0	Malate synthase, glyoxysomal	PF01274.19^Malate_synthase^Malate synthase^26-556^E:8.6e-221	1	-0,572	1	-0,574	0,226	1,802	0,001	3,268	1	0,83	MASY_RICCO	Malate synthase, glyoxysomal (EC 2.3.3.9)		Ricinus communis (Castor bean)	PATHWAY: Carbohydrate metabolism; glyoxylate cycle; (S)-malate from isocitrate: step 2/2.	Glyoxylate bypass; Glyoxysome; Peroxisome; Transferase; Tricarboxylic acid cycle
SRR646604_TR14310_c0_g3_i3_len=2558	COP1_ARATH	Q:393-2288,H:46-674	77.06%ID	E:0	E3 ubiquitin-protein ligase COP1	PF13920.3^zf-C3HC4_3^Zinc finger, C3HC4 type (RING finger)^54-96^E:9.8e-07`PF13923.3^zf-C3HC4_2^Zinc finger, C3HC4 type (RING finger)^57-94^E:5.1e-10`PF00097.22^zf-C3HC4^Zinc finger, C3HC4 type (RING finger)^57-94^E:8.4e-08`PF13445.3^zf-RING_UBOX^RING-type zinc-finger^57-92^E:3.5e-05`PF00400.29^WD40^WD domain, G-beta repeat^462-499^E:0.0022`PF00400.29^WD40^WD domain, G-beta repeat^549-584^E:0.00014	0,365	0,518	0,84	-0,326	0,025	1,987	0,001	2,533	0,267	0,73	COP1_ARATH	E3 ubiquitin-protein ligase COP1 (EC 6.3.2.-) (Constitutive photomorphogenesis protein 1)	COP1 At2g32950 T21L14.11	Arabidopsis thaliana (Mouse-ear cress)	PATHWAY: Protein modification; protein ubiquitination.	Coiled coil; Complete proteome; Cytoplasm; Ligase; Metal-binding; Nucleus; Phytochrome signaling pathway; Reference proteome; Repeat; Ubl conjugation; Ubl conjugation pathway; WD repeat; Zinc; Zinc-finger
SRR646604_TR17882_c0_g1_i1_len=1257	NA	NA	NA	NA	NA	.	1	0	1	0,413	0,008	3,65	0	4,2	1	0	.	.	.	.	.	.
SRR646604_TR28128_c0_g1_i5_len=2344	DOX1_ARATH	Q:2306-288,H:1-639	69.58%ID	E:0	Alpha-dioxygenase 1	PF03098.12^An_peroxidase^Animal haem peroxidase^106-498^E:4.1e-65	1	0,108	0,254	3,723	0,005	6,188	0	6,865	1	0,112	DOX1_ARATH	Alpha-dioxygenase 1 (Alpha DOX1) (EC 1.14.99.-) (Fatty acid dioxygenase AlphaDOX1) (Pathogen-induced oxygenase) (Plant alpha dioxygenase 1)	DOX1 DIOX1 PADOX-1 PIOX At3g01420 T13O15.6	Arabidopsis thaliana (Mouse-ear cress)		3D-structure; Complete proteome; Dioxygenase; Fatty acid biosynthesis; Fatty acid metabolism; Glycoprotein; Hypersensitive response; Iron; Lipid biosynthesis; Lipid metabolism; Metal-binding; Oxidoreductase; Oxylipin biosynthesis; Peroxidase; Plant defense; Reference proteome; Signal
SRR646604_TR35852_c0_g1_i2_len=2419	WRK33_ARATH	Q:1357-431,H:179-467	60.06%ID	E:1e-93	Probable WRKY transcription factor 33	PF03106.12^WRKY^WRKY DNA -binding domain^71-126^E:7e-25`PF03106.12^WRKY^WRKY DNA -binding domain^250-307^E:7.5e-26	0,381	1,658	0,008	3,777	0,027	2,342	0	3,228	1	-0,207	WRK33_ARATH	Probable WRKY transcription factor 33 (WRKY DNA-binding protein 33)	WRKY33 At2g38470 T19C21.4	Arabidopsis thaliana (Mouse-ear cress)		Complete proteome; DNA-binding; Nucleus; Phosphoprotein; Plant defense; Reference proteome; Repeat; Stress response; Transcription; Transcription regulation
SRR646604_TR36869_c0_g1_i1_len=1591	NA	NA	NA	NA	NA	.	1	-0,214	1	1,488	0,016	1,834	0	2,471	1	-0,371	.	.	.	.	.	.
SRR648705_TR7203_c0_g1_i1_len=1907	PLA14_ARATH	Q:201-1730,H:7-502	60.66%ID	E:0	Phospholipase A1-Ibeta2, chloroplastic	PF01764.22^Lipase_3^Lipase (class 3)^316-463^E:3.2e-36	1	0,626	0,004	3,408	0,008	3,388	0	3,991	1	-0,351	PLA14_ARATH	Phospholipase A1-Ibeta2, chloroplastic (EC 3.1.1.-)	At4g16820 dl4435w FCAALL.42	Arabidopsis thaliana (Mouse-ear cress)		Chloroplast; Complete proteome; Hydrolase; Isopeptide bond; Lipid degradation; Lipid metabolism; Plastid; Reference proteome; Transit peptide; Ubl conjugation
SRR648705_TR12223_c0_g1_i1_len=1374	LSF2_ARATH	Q:1184-387,H:19-282	69.74%ID	E:8e-131	Phosphoglucan phosphatase LSF2, chloroplastic	PF00782.17^DSPc^Dual specificity phosphatase, catalytic domain^116-235^E:2.3e-15	0,183	0,651	0,387	0,476	0,007	1,148	0	1,422	0,356	-1,185	LSF2_ARATH	Phosphoglucan phosphatase LSF2, chloroplastic (EC 3.1.3.-) (Phosphoglucan phosphatase like sex Four2) (Protein LIKE SEX4 2)	LSF2 At3g10940 F9F8.24	Arabidopsis thaliana (Mouse-ear cress)		3D-structure; Carbohydrate metabolism; Chloroplast; Complete proteome; Hydrolase; Plastid; Reference proteome; Transit peptide
SRR648705_TR17005_c0_g1_i3_len=4112	Y2317_ARATH	Q:576-3872,H:2-1112	65.77%ID	E:0	Probable leucine-rich repeat receptor-like protein kinase At2g33170	PF08263.9^LRRNT_2^Leucine rich repeat N-terminal domain^34-71^E:7e-10`PF00560.30^LRR_1^Leucine Rich Repeat^510-532^E:0.94`PF13855.3^LRR_8^Leucine rich repeat^558-616^E:1.1e-08`PF00069.22^Pkinase^Protein kinase domain^813-1080^E:1.2e-49`PF07714.14^Pkinase_Tyr^Protein tyrosine kinase^818-1081^E:1.6e-43`PF08263.9^LRRNT_2^Leucine rich repeat N-terminal domain^1017-1025^E:11000	0,781	0,611	0,299	1,309	0,004	2,68	0	3,303	1	0,066	Y2317_ARATH	Probable leucine-rich repeat receptor-like protein kinase At2g33170 (EC 2.7.11.1)	At2g33170 F25I18.9	Arabidopsis thaliana (Mouse-ear cress)		ATP-binding; Complete proteome; Glycoprotein; Kinase; Leucine-rich repeat; Membrane; Nucleotide-binding; Phosphoprotein; Receptor; Reference proteome; Repeat; Serine/threonine-protein kinase; Signal; Transferase; Transmembrane; Transmembrane helix
SRR648705_TR19464_c0_g2_i1_len=962	ATG8I_ARATH	Q:259-549,H:19-115	78.35%ID	E:7e-51	Autophagy-related protein 8i	PF02991.13^Atg8^Autophagy protein Atg8 ubiquitin like^17-120^E:4.1e-43`PF04110.10^APG12^Ubiquitin-like autophagy protein Apg12^44-120^E:2.3e-06	0,4	0,285	0,349	0,42	0,001	1,306	0,001	1,321	0,108	0,598	ATG8I_ARATH	Autophagy-related protein 8i (Autophagy-related ubiquitin-like modifier ATG8i) (AtAPG8i) (Protein autophagy 8i)	ATG8I APG8I At3g15580 MQD17.3	Arabidopsis thaliana (Mouse-ear cress)		Autophagy; Complete proteome; Cytoplasm; Cytoplasmic vesicle; Cytoskeleton; Lipoprotein; Membrane; Microtubule; Protein transport; Reference proteome; Transport; Ubl conjugation pathway; Vacuole
SRR648705_TR20594_c0_g2_i5_len=4055	ASPL2_ARATH	Q:3464-2127,H:21-453	48.43%ID	E:2e-139	Aspartic proteinase-like protein 2	PF14543.3^TAXi_N^Xylanase inhibitor N-terminal^83-267^E:1.1e-40`PF00026.20^Asp^Eukaryotic aspartyl protease^83-439^E:1.6e-29`PF14541.3^TAXi_C^Xylanase inhibitor C-terminal^286-438^E:8.4e-24	0,781	0,354	0,003	2,325	0,044	1,119	0	1,771	1	-0,101	ASPL2_ARATH	Aspartic proteinase-like protein 2 (EC 3.4.23.-)	At1g65240 T23K8.15	Arabidopsis thaliana (Mouse-ear cress)		Aspartyl protease; Cell membrane; Complete proteome; GPI-anchor; Glycoprotein; Hydrolase; Lipoprotein; Membrane; Protease; Reference proteome; Signal
SRR648705_TR20832_c0_g1_i1_len=1063	MGDP1_HUMAN	Q:199-627,H:4-159	41.14%ID	E:1e-31	Magnesium-dependent phosphatase 1	PF12689.4^Acid_PPase^Acid Phosphatase^20-162^E:8.1e-37`PF13419.3^HAD_2^Haloacid dehalogenase-like hydrolase^42-144^E:4.8e-09	1	-0,047	0,812	0,179	0,002	1,517	0	1,922	1	-0,018	MGDP1_HUMAN	Magnesium-dependent phosphatase 1 (MDP-1) (EC 3.1.3.-) (EC 3.1.3.48)	MDP1	Homo sapiens (Human)		3D-structure; Alternative splicing; Complete proteome; Hydrolase; Magnesium; Metal-binding; Protein phosphatase; Reference proteome
SRR648705_TR24450_c0_g1_i3_len=3403	NAGS2_ARATH	Q:2338-1337,H:272-610	75.51%ID	E:0	Probable amino-acid acetyltransferase NAGS2, chloroplastic	PF13673.4^Acetyltransf_10^Acetyltransferase (GNAT) domain^223-302^E:1.1e-05`PF13508.4^Acetyltransf_7^Acetyltransferase (GNAT) domain^224-301^E:9.9e-09`PF00583.22^Acetyltransf_1^Acetyltransferase (GNAT) family^228-299^E:6.6e-11	1	0	1	0	0,066	5,379	0	6,894	1	0	NAGS2_ARATH	Probable amino-acid acetyltransferase NAGS2, chloroplastic (EC 2.3.1.1) (N-acetylglutamate synthase 2)	NAGS2 At4g37670 F19F18.160	Arabidopsis thaliana (Mouse-ear cress)	PATHWAY: Amino-acid biosynthesis; L-arginine biosynthesis; N(2)-acetyl-L-ornithine from L-glutamate: step 1/4.	Acyltransferase; Alternative splicing; Amino-acid biosynthesis; Arginine biosynthesis; Chloroplast; Complete proteome; Plastid; Reference proteome; Transferase; Transit peptide
SRR648705_TR30701_c0_g1_i5_len=2113	TM53B_XENLA	Q:1144-1938,H:29-269	28.84%ID	E:1e-23	Transmembrane protein 53-B	PF05705.11^DUF829^Eukaryotic protein of unknown function (DUF829)^168-429^E:1.1e-67	0,811	-0,356	0,5	0,638	0,068	1,012	0	2,3	1	0,111	TM53B_XENLA	Transmembrane protein 53-B	tmem53-b	Xenopus laevis (African clawed frog)		Membrane; Transmembrane; Transmembrane helix
SRR648705_TR34874_c3_g1_i1_len=612	NA	NA	NA	NA	NA	PF03061.19^4HBT^Thioesterase superfamily^57-129^E:2.5e-12	0,066	5,364	0,282	1,217	0,008	3,063	0	3,579	0,254	4,664	.	.	.	.	.	.
SRR648705_TR34891_c0_g1_i1_len=932	NA	NA	NA	NA	NA	PF04398.9^DUF538^Protein of unknown function, DUF538^41-148^E:2.1e-28	1	0,07	1	-0,101	0,008	3,779	0	4,648	1	0,035	.	.	.	.	.	.
SRR648705_TR35196_c0_g2_i1_len=2964	AVP_VIGRR	Q:257-2563,H:1-766	91.16%ID	E:0	Pyrophosphate-energized vacuolar membrane proton pump	PF03030.13^H_PPase^Inorganic H+ pyrophosphatase^21-754^E:5.9e-261	0,034	0,764	0,012	-0,941	0,171	0,352	0	1	0,034	0,769	AVP_VIGRR	Pyrophosphate-energized vacuolar membrane proton pump (EC 3.6.1.1) (Pyrophosphate-energized inorganic pyrophosphatase) (H(+)-PPase) (Vacuolar H(+)-pyrophosphatase)		Vigna radiata var. radiata (Mung bean) (Phaseolus aureus)		3D-structure; Direct protein sequencing; Disulfide bond; Hydrogen ion transport; Hydrolase; Ion transport; Magnesium; Membrane; Metal-binding; Transmembrane; Transmembrane helix; Transport; Vacuole
SRR648705_TR39974_c0_g1_i2_len=3466	NA	NA	NA	NA	NA	PF01918.18^Alba^Alba^19-83^E:1.9e-21	1	0,28	0,524	0,666	0,076	2,356	0	3,706	0,489	0,43	.	.	.	.	.	.
SRR648707_TR11715_c0_g1_i1_len=2834	NA	NA	NA	NA	NA	.	1	2,16	0,005	4,586	0,059	1,782	0	2,653	0,381	2,544	.	.	.	.	.	.
SRR648707_TR13466_c0_g1_i3_len=3962	Y5181_ARATH	Q:1171-3720,H:8-856	45.68%ID	E:0	Probable LRR receptor-like serine/threonine-protein kinase At1g51810	PF12819.4^Malectin_like^Carbohydrate-binding protein of the ER^33-362^E:1.9e-96`PF00560.30^LRR_1^Leucine Rich Repeat^444-462^E:0.63`PF07714.14^Pkinase_Tyr^Protein tyrosine kinase^584-781^E:1.6e-43`PF00069.22^Pkinase^Protein kinase domain^585-849^E:1.6e-47	0,902	0,069	0,031	1,287	0,004	2,383	0	3,013	0,832	0,204	Y5181_ARATH	Probable LRR receptor-like serine/threonine-protein kinase At1g51810 (EC 2.7.11.1)	At1g51810 F19C24.1 T14L22.2	Arabidopsis thaliana (Mouse-ear cress)		ATP-binding; Alternative splicing; Complete proteome; Glycoprotein; Kinase; Leucine-rich repeat; Membrane; Nucleotide-binding; Phosphoprotein; Receptor; Reference proteome; Repeat; Serine/threonine-protein kinase; Signal; Transferase; Transmembrane; Transmembrane helix
SRR648707_TR14270_c0_g1_i1_len=1053	ARC5A_ARATH	Q:856-464,H:2-132	86.26%ID	E:5e-75	Actin-related protein 2/3 complex subunit 5A	PF04699.11^P16-Arc^ARP2/3 complex 16 kDa subunit (p16-Arc)^13-129^E:2.4e-21	0,002	6,283	0,545	0,525	1	0	0	7,167	1	0,64	ARC5A_ARATH	Actin-related protein 2/3 complex subunit 5A (Actin-related protein C5A) (Arp2/3 complex 16 kDa subunit) (p16-ARC) (Protein CROOKED)	ARPC5A CRK At4g01710 T15B16.22	Arabidopsis thaliana (Mouse-ear cress)		Acetylation; Actin-binding; Cell projection; Complete proteome; Cytoplasm; Cytoskeleton; Reference proteome
SRR648707_TR14568_c3_g4_i6_len=2373	MTNA_TOBAC	Q:258-1367,H:1-369	78.11%ID	E:7e-178	Methylthioribose-1-phosphate isomerase {ECO:0000255|HAMAP-Rule:MF_03119}	PF01008.14^IF-2B^Initiation factor 2 subunit family^53-354^E:1.3e-76	0,493	-0,322	0,064	0,956	0,004	2,098	0	3,196	1	-0,051	MTNA_TOBAC	Methylthioribose-1-phosphate isomerase (M1Pi) (MTR-1-P isomerase) (EC 5.3.1.23) (S-methyl-5-thioribose-1-phosphate isomerase) (Translation initiation factor eIF-2B subunit alpha/beta/delta-like protein)	CIG2	Nicotiana tabacum (Common tobacco)	PATHWAY: Amino-acid biosynthesis; L-methionine biosynthesis via salvage pathway; L-methionine from S-methyl-5-thio-alpha-D-ribose 1-phosphate: step 1/6. {ECO:0000255|HAMAP-Rule:MF_03119}.	Amino-acid biosynthesis; Cytoplasm; Isomerase; Methionine biosynthesis; Nucleus
SRR648707_TR19203_c0_g1_i3_len=1048	SABP2_TOBAC	Q:101-868,H:6-260	67.58%ID	E:2e-123	Salicylic acid-binding protein 2	PF12146.5^Hydrolase_4^Serine aminopeptidase, S33^5-107^E:1.4e-08`PF00561.17^Abhydrolase_1^alpha/beta hydrolase fold^7-128^E:3.7e-13`PF12697.4^Abhydrolase_6^Alpha/beta hydrolase family^8-249^E:2.5e-19	0,189	1,554	0,278	0,75	0,018	1,098	0	1,971	0,195	1,206	SABP2_TOBAC	Salicylic acid-binding protein 2 (NtSABP2) (EC 3.1.1.-) (Methyl salicylate esterase)	SABP2	Nicotiana tabacum (Common tobacco)		3D-structure; Hydrolase; Immunity; Innate immunity; Plant defense
SRR648707_TR21246_c1_g1_i1_len=1736	GGPPS_HEVBR	Q:1488-844,H:90-308	34.25%ID	E:7e-21	Geranylgeranyl pyrophosphate synthase, chloroplastic	PF00348.14^polyprenyl_synt^Polyprenyl synthetase^101-280^E:2.3e-09	0,05	0,801	0,003	1,461	0,011	0,894	0	1,232	0,461	-1,485	GGPPS_HEVBR	Geranylgeranyl pyrophosphate synthase, chloroplastic (GGPP synthase) (GGPS) (EC 2.5.1.-) ((2E,6E)-farnesyl diphosphate synthase) (Dimethylallyltranstransferase) (EC 2.5.1.1) (Farnesyl diphosphate synthase) (Farnesyltranstransferase) (EC 2.5.1.29) (Geranyltranstransferase) (EC 2.5.1.10)	GGPS GG GGPPS1	Hevea brasiliensis (Para rubber tree) (Siphonia brasiliensis)	PATHWAY: Isoprenoid biosynthesis; farnesyl diphosphate biosynthesis; farnesyl diphosphate from geranyl diphosphate and isopentenyl diphosphate: step 1/1.; PATHWAY: Isoprenoid biosynthesis; geranyl diphosphate biosynthesis; geranyl diphosphate from dimethylallyl diphosphate and isopentenyl diphosphate: step 1/1.; PATHWAY: Isoprenoid biosynthesis; geranylgeranyl diphosphate biosynthesis; geranylgeranyl diphosphate from farnesyl diphosphate and isopentenyl diphosphate: step 1/1.	Carotenoid biosynthesis; Chloroplast; Isoprene biosynthesis; Magnesium; Metal-binding; Plastid; Transferase; Transit peptide
SRR648707_TR24142_c0_g2_i6_len=2127	ASB1_ARATH	Q:806-576,H:197-273	79.22%ID	E:1e-35	Anthranilate synthase beta subunit 1, chloroplastic	.	0,066	1,904	0,002	3,103	0,014	3,566	0,001	4,051	1	-0,419	ASB1_ARATH	Anthranilate synthase beta subunit 1, chloroplastic (EC 4.1.3.27) (Anthranilate synthase component 2-1) (Anthranilate synthase, glutamine amidotransferase component 2-1) (Protein TRYPTOPHAN BIOSYNTHESIS 4) (Protein WEAK ETHYLENE INSENSITIVE 7)	ASB1 TRP4 WEI7 At1g25220 F4F7.39	Arabidopsis thaliana (Mouse-ear cress)	PATHWAY: Amino-acid biosynthesis; L-tryptophan biosynthesis; L-tryptophan from chorismate: step 1/5.	Alternative splicing; Amino-acid biosynthesis; Aromatic amino acid biosynthesis; Chloroplast; Complete proteome; Glutamine amidotransferase; Lyase; Plastid; Reference proteome; Transit peptide; Tryptophan biosynthesis
SRR648707_TR24143_c1_g1_i2_len=2985	Y2913_ARATH	Q:2804-468,H:12-794	59.22%ID	E:0	G-type lectin S-receptor-like serine/threonine-protein kinase At2g19130	PF01453.21^B_lectin^D-mannose binding lectin^121-229^E:2.4e-29`PF00954.17^S_locus_glycop^S-locus glycoprotein domain^260-368^E:6.3e-35`PF08276.8^PAN_2^PAN-like domain^397-455^E:1.9e-16`PF14295.3^PAN_4^PAN domain^418-446^E:0.003`PF07714.14^Pkinase_Tyr^Protein tyrosine kinase^544-811^E:2e-42`PF00069.22^Pkinase^Protein kinase domain^545-809^E:3.5e-49	0,093	1,096	0,257	0,908	0,026	2,186	0,001	2,825	1	-0,171	Y2913_ARATH	G-type lectin S-receptor-like serine/threonine-protein kinase At2g19130 (EC 2.7.11.1)	At2g19130 T20K24.15	Arabidopsis thaliana (Mouse-ear cress)		ATP-binding; Cell membrane; Complete proteome; Disulfide bond; EGF-like domain; Glycoprotein; Kinase; Lectin; Membrane; Nucleotide-binding; Phosphoprotein; Receptor; Reference proteome; Serine/threonine-protein kinase; Signal; Transferase; Transmembrane; Transmembrane helix
SRR648707_TR27941_c0_g2_i1_len=1183	AB19I_ARATH	Q:1029-304,H:51-290	79.34%ID	E:6e-123	ABC transporter I family member 19	.	0,066	5,278	0,014	1,688	0,017	5,799	0	7,071	1	0	AB19I_ARATH	ABC transporter I family member 19 (ABC transporter ABCI.19) (AtABCI19) (GCN-related protein 2) (Non-intrinsic ABC protein 4)	ABCI19 NAP4 At1g03905 F21M11.17	Arabidopsis thaliana (Mouse-ear cress)		ATP-binding; Complete proteome; Cytoplasm; Nucleotide-binding; Reference proteome; Transport
SRR648707_TR28476_c1_g2_i1_len=726	NA	NA	NA	NA	NA	.	0,299	1,499	0,017	2,261	0,001	1,954	0	3,298	0,133	1,155	.	.	.	.	.	.
SRR648707_TR37562_c0_g1_i1_len=1266	PABN2_ARATH	Q:1044-421,H:13-219	84.13%ID	E:1e-85	Polyadenylate-binding protein 2 {ECO:0000303|PubMed:18479511}	PF00076.19^RRM_1^RNA recognition motif. (a.k.a. RRM, RBD, or RNP domain)^95-163^E:2.5e-17`PF13893.3^RRM_5^RNA recognition motif. (a.k.a. RRM, RBD, or RNP domain)^108-166^E:0.03	0,957	0,041	0	-3,299	0,608	0,21	0	1,175	0,129	4,873	PABN2_ARATH	Polyadenylate-binding protein 2 (AtPabN2) (AtPabN3) (Poly(A)-binding protein 2) (Nuclear poly(A)-binding protein 2) (Poly(A)-binding protein II) (PABII)	PABN2 At5g65260 MQN23.20	Arabidopsis thaliana (Mouse-ear cress)		Coiled coil; Complete proteome; Cytoplasm; Nucleus; RNA-binding; Reference proteome
SRR924147_TR1558_c0_g1_i1_len=2487	Y5262_ARATH	Q:1638-172,H:5-497	63.69%ID	E:5e-179	Ankyrin repeat-containing protein At5g02620	PF12796.4^Ank_2^Ankyrin repeats (3 copies)^19-88^E:5.2e-07`PF12796.4^Ank_2^Ankyrin repeats (3 copies)^98-157^E:2.4e-12`PF13637.3^Ank_4^Ankyrin repeats (many copies)^104-155^E:2.3e-09`PF13637.3^Ank_4^Ankyrin repeats (many copies)^135-189^E:6.5e-12`PF12796.4^Ank_2^Ankyrin repeats (3 copies)^166-226^E:1.3e-13`PF00023.27^Ank^Ankyrin repeat^168-190^E:2.3e-05`PF13606.3^Ank_3^Ankyrin repeat^168-190^E:0.00055`PF00023.27^Ank^Ankyrin repeat^203-235^E:0.0066`PF13637.3^Ank_4^Ankyrin repeats (many copies)^205-257^E:8.4e-09`PF13857.3^Ank_5^Ankyrin repeats (many copies)^225-278^E:6.1e-09`PF12796.4^Ank_2^Ankyrin repeats (3 copies)^233-295^E:7.8e-08`PF00023.27^Ank^Ankyrin repeat^237-261^E:0.00016`PF13606.3^Ank_3^Ankyrin repeat^237-260^E:0.005`PF13962.3^PGG^Domain of unknown function^350-	1	0,204	0,461	1,586	0,003	2,867	0	3,991	1	0,564	Y5262_ARATH	Ankyrin repeat-containing protein At5g02620	At5g02620 T22P11.210	Arabidopsis thaliana (Mouse-ear cress)		ANK repeat; Complete proteome; Membrane; Phosphoprotein; Reference proteome; Repeat; Transmembrane; Transmembrane helix

						465^E:6.2e-27															
SRR924147_TR34893_c0_g1_i1_len=934	Y5278_ARATH	Q:545-267,H:76-168	53.76%ID	E:1e-31	Uncharacterized protein PAM68-like	PF11947.5^DUF3464^Protein of unknown function (DUF3464)^66-187^E:5.1e-39	0,252	-0,272	0,001	-0,795	0,003	0,922	0	1,276	0,572	-0,14	Y5278_ARATH	Uncharacterized protein PAM68-like	At5g52780 F6N7.27	Arabidopsis thaliana (Mouse-ear cress)		Complete proteome; Membrane; Reference proteome; Transmembrane; Transmembrane helix
SRR924148_TR589_c0_g1_i1_len=2266	KELP_ARATH	Q:120-596,H:13-165	40.85%ID	E:2e-27	RNA polymerase II transcriptional coactivator KELP	PF14223.3^Retrotran_gag_2^gag-polypeptide of LTR copia-type^175-310^E:5.6e-16`PF00098.20^zf-CCHC^Zinc knuckle^341-356^E:0.00023	1	-0,393	0,239	1,3	0,299	1,587	0	3,232	1	0,364	KELP_ARATH	RNA polymerase II transcriptional coactivator KELP	KELP At4g10920 F25I24.130 F8M12.6	Arabidopsis thaliana (Mouse-ear cress)		Activator; Complete proteome; DNA-binding; Nucleus; Reference proteome; Transcription; Transcription regulation
SRR924148_TR8016_c0_g1_i1_len=2514	SBIR1_ARATH	Q:420-2258,H:35-639	60.62%ID	E:0	Leucine-rich repeat receptor-like serine/threonine/tyrosine-protein kinase SOBIR1	PF13855.3^LRR_8^Leucine rich repeat^177-236^E:1.4e-06`PF00560.30^LRR_1^Leucine Rich Repeat^201-223^E:1.8`PF00560.30^LRR_1^Leucine Rich Repeat^272-289^E:1.9`PF00069.22^Pkinase^Protein kinase domain^425-703^E:3.5e-44`PF07714.14^Pkinase_Tyr^Protein tyrosine kinase^425-654^E:3.2e-35	0,444	0,473	0,016	1,46	0,017	2,344	0	2,976	1	0,108	SBIR1_ARATH	Leucine-rich repeat receptor-like serine/threonine/tyrosine-protein kinase SOBIR1 (EC 2.7.10.1) (EC 2.7.11.1) (Protein EVERSHED) (Protein SUPPRESSOR OF BIR1-1)	SOBIR1 EVR At2g31880 F20M17.8	Arabidopsis thaliana (Mouse-ear cress)		ATP-binding; Cell membrane; Complete proteome; Glycoprotein; Kinase; Leucine-rich repeat; Membrane; Nucleotide-binding; Plant defense; Receptor; Reference proteome; Repeat; Serine/threonine-protein kinase; Signal; Transferase; Transmembrane; Transmembrane helix; Tyrosine-protein kinase
SRR924148_TR21803_c1_g1_i1_len=726	DV4H_CATRO	Q:104-517,H:24-159	70.21%ID	E:3e-58	Deacetoxyvindoline 4-hydroxylase	PF14226.3^DIOX_N^non-haem dioxygenase in morphine synthesis N-terminal^68-124^E:2e-12	0,003	1,25	0,001	1,69	0,011	1,13	0	1,689	0,389	0,371	DV4H_CATRO	Deacetoxyvindoline 4-hydroxylase (EC 1.14.11.20)	D4H	Catharanthus roseus (Madagascar periwinkle) (Vinca rosea)	PATHWAY: Alkaloid biosynthesis; vindoline biosynthesis; vindoline from tabersonine: step 5/6.	Cytoplasm; Dioxygenase; Direct protein sequencing; Iron; Metal-binding; Oxidoreductase; Vitamin C
SRR924148_TR31225_c1_g2_i1_len=829	NA	NA	NA	NA	NA	.	1	0,199	0,299	1,327	1	0,69	0	4,497	0	-3,286	.	.	.	.	.	.
SRR924148_TR32921_c0_g1_i2_len=1481	RCE1_ARATH	Q:362-910,H:1-184	83.7%ID	E:2e-110	NEDD8-conjugating enzyme Ubc12	PF00179.23^UQ_con^Ubiquitin-conjugating enzyme^33-167^E:4.2e-38	0,887	-0,062	0,136	0,528	0,002	1,277	0,001	1,357	0,883	-0,07	RCE1_ARATH	NEDD8-conjugating enzyme Ubc12 (EC 6.3.2.-) (RUB1 carrier protein 1) (RUB1-conjugating enzyme 1) (RUB1-protein ligase 1)	RCE1 At4g36800 C7A10.560	Arabidopsis thaliana (Mouse-ear cress)	PATHWAY: Protein modification; protein neddylation.	ATP-binding; Alternative splicing; Complete proteome; Ligase; Nucleotide-binding; Reference proteome; Ubl conjugation pathway
SRR924148_TR37722_c0_g1_i1_len=957	NA	NA	NA	NA	NA	.	0,559	0,418	0,839	0,098	0,001	1,588	0	2,459	0,178	-0,628	.	.	.	.	.	.
SRR924148_TR37795_c0_g1_i1_len=952	PCR2_ARATH	Q:639-208,H:6-152	63.27%ID	E:2e-59	Protein PLANT CADMIUM RESISTANCE 2	PF04749.14^PLAC8^PLAC8 family^55-153^E:9.7e-29	0,298	0,737	0,326	0,55	0,01	1,16	0	1,757	1	-0,201	PCR2_ARATH	Protein PLANT CADMIUM RESISTANCE 2 (AtPCR2)	PCR2 At1g14870 F10B6.27	Arabidopsis thaliana (Mouse-ear cress)		Cell membrane; Complete proteome; Membrane; Reference proteome; Transmembrane; Transmembrane helix

Supplemental Table S2

Description of transcripts down-regulated upon challenge with M. sexta found in the intersections between lists of each experiment.

Core
Ms1wk
Ms6h
Ms8h
MsDamaged24h
MsDamaged24h_MsIntact24h
MsIntact24h

Core

Transcript (CDF97)	Uniprot homolog	BLASTX Position	BLASTX %Identity	BLASTX evalue	Uniprot descr	Pfam	PValue_Ms6h	logFC_Ms6h	PValue_Ms8h	logFC_Ms8h	PValue_MsDamaged24h	logFC_MsDamaged24h	PValue_MsIntact24h	logFC_MsIntact24h	PValue_Ms1wk	logFC_Ms1wk	Entry.name	Protein names	Gene names	Organism	Pathway	Keywords
SRR1144634_TR39822_c0_g2_i1_len=510	RUXG_ARATH	Q:107-346,H:1-80	90%ID	E:3e-45	Probable small nuclear ribonucleoprotein G	.	0	-1,001	0,781	-0,092	0,349	0,365	0,793	-0,107	0	-1,015	RUXG_ARATH	Probable small nuclear ribonucleoprotein G (snRNP-G) (Sm protein G) (Sm-G) (SmG)	At2g23930 T29E15.13	Arabidopsis thaliana (Mouse-ear cress)		Alternative splicing; Complete proteome; Nucleus; RNA-binding; Reference proteome; Ribonucleoprotein; Spliceosome; mRNA processing; mRNA splicing
SRR122238_TR62384_c3_g1_i4_len=752	RL321_ARATH	Q:605-213,H:3-133	86.26%ID	E:1e-70	60S ribosomal protein L32-1	PF01655.15^Ribosomal_L32e^Ribosomal protein L32^16-122^E:2.1e-50	0	-1,235	0,391	-0,22	0,639	0,151	0,256	-0,402	0	-1,025	RL321_ARATH	60S ribosomal protein L32-1	RPL32A At4g18100 F15J5.70	Arabidopsis thaliana (Mouse-ear cress)		Complete proteome; Reference proteome; Ribonucleoprotein; Ribosomal protein
SRR122238_TR75230_c0_g1_i1_len=855	RL12_PRUAR	Q:767-270,H:1-166	92.17%ID	E:5e-104	60S ribosomal protein L12	PF03946.11^Ribosomal_L11_N^Ribosomal protein L11, N-terminal domain^15-70^E:6.1e-23`PF00298.16^Ribosomal_L11^Ribosomal protein L11, RNA binding domain^75-144^E:1.5e-19	0	-0,932	0,687	-0,103	0,4	0,24	0,904	0,046	0	-1,243	RL12_PRUAR	60S ribosomal protein L12	RPL12	Prunus armeniaca (Apricot) (Armeniaca vulgaris)		RNA-binding; Ribonucleoprotein; Ribosomal protein; rRNA-binding
SRR122238_TR83917_c0_g1_i1_len=621	RL37A_ORYSJ	Q:498-223,H:1-92	96.74%ID	E:3e-57	60S ribosomal protein L37a	PF01780.16^Ribosomal_L37ae^Ribosomal L37ae protein family^45-129^E:2.9e-39	0	-1,058	0,386	0,223	1	-0,003	0,633	-0,167	0	-1,357	RL37A_ORYSJ	60S ribosomal protein L37a	Os01g0679700 LOC_Os01g48770 B1144G04.40-1; Os05g0557000 LOC_Os05g48320 OJ1214_E03.13 OsJ_018715	Oryza sativa subsp. japonica (Rice)		3D-structure; Complete proteome; Metal-binding; Reference proteome; Ribonucleoprotein; Ribosomal protein; Zinc; Zinc-finger
SRR342019_TR27032_c0_g1_i1_len=1711	NA	NA	NA	NA	NA	PF00627.28^UBA^UBA/TS-N domain^180-216^E:2.7e-08`PF09409.7^PUB^PUB domain^387-457^E:2.2e-25	0	-6,648	1	-0,019	1	-1,533	1	-1,978	0	-5,597	.	.	.	.	.	.
SRR342022_TR21159_c0_g3_i1_len=2114	EP1G_DAUCA	Q:386-1501,H:5-373	48.58%ID	E:3e-98	Epidermis-specific secreted glycoprotein EP1	PF01453.21^B_lectin^D-mannose binding lectin^112-208^E:6.1e-26`PF08276.8^PAN_2^PAN-like domain^424-453^E:3.4e-05`PF14295.3^PAN_4^PAN domain^427-445^E:0.38	0	-1,159	0,01	-0,875	0,507	0,256	0,003	0,952	0	-1,18	EP1G_DAUCA	Epidermis-specific secreted glycoprotein EP1 (52/54 kDa medium protein)	EP1	Daucus carota (Wild carrot)		Direct protein sequencing; Glycoprotein; Lectin; Secreted; Signal
SRR342023_TR8921_c1_g1_i2_len=778	R27A3_ARATH	Q:131-568,H:1-146	89.04%ID	E:3e-89	60S ribosomal protein L27a-3	PF00828.16^Ribosomal_L27A^Ribosomal proteins 50S-L15, 50S-L18e, 60S-L27A^71-186^E:6.6e-23	0	-1,097	0,117	-0,368	0,657	0,122	0,528	-0,18	0	-1,271	R27A3_ARATH	60S ribosomal protein L27a-3	RPL27AC At1g70600 F24J13.17 F5A18.22	Arabidopsis thaliana (Mouse-ear cress)		Complete proteome; Reference proteome; Ribonucleoprotein; Ribosomal protein
SRR648707_TR10026_c0_g2_i1_len=2229	PMGI_MESCR	Q:210-1880,H:1-557	89.05%ID	E:0	2,3-bisphosphoglycerate-independent phosphoglycerate mutase	PF01676.15^Metalloenzyme^Metalloenzyme superfamily^22-545^E:4.7e-101`PF06415.10^iPGM_N^BPG-independent PGAM N-terminus (iPGM_N)^102-322^E:6.4e-59	0,001	-2,264	0	2,743	0,006	1,921	0,001	2,244	0	-2,869	PMGI_MESCR	2,3-bisphosphoglycerate-independent phosphoglycerate mutase (BPG-independent PGAM) (Phosphoglyceromutase) (EC 5.4.2.12) (PGAM-I)	PGM1	Mesembryanthemum crystallinum (Common ice plant) (Cryophytum crystallinum)	PATHWAY: Carbohydrate degradation; glycolysis; pyruvate from D-glyceraldehyde 3-phosphate: step 3/5.	Cytoplasm; Glycolysis; Isomerase; Manganese; Metal-binding; Stress response
SRR648707_TR24657_c0_g1_i1_len=1073	R10A3_ARATH	Q:171-821,H:1-217	91.71%ID	E:3e-129	60S ribosomal protein L10a-3	PF00687.18^Ribosomal_L1^Ribosomal protein L1p/L10e family^21-211^E:1.1e-44	0	-1,091	0,608	-0,127	0,463	0,211	0,259	-0,33	0	-11,816	R10A3_ARATH	60S ribosomal protein L10a-3	RPL10AC At5g22440 MWD9.24	Arabidopsis thaliana (Mouse-ear cress)		Complete proteome; Reference proteome; Ribonucleoprotein; Ribosomal protein
SRR924147_TR1969_c1_g1_i6_len=904	RL31_NICGU	Q:178-537,H:1-120	91.67%ID	E:1e-55	60S ribosomal protein L31	.	0,001	-0,822	0,287	-0,257	0,367	0,252	0,796	-0,085	0	-0,942	RL31_NICGU	60S ribosomal protein L31	RPL31	Nicotiana glutinosa (Tobacco)		Ribonucleoprotein; Ribosomal protein
SRR1144634_TR3773_c0_g2_i6_len=2346	THIC_ARATH	Q:136-1770,H:99-643	91.01%ID	E:0	Phosphomethylpyrimidine synthase, chloroplastic	PF01964.15^ThiC_Rad_SAM^Radical SAM ThiC family^79-499^E:4.5e-198	0,68	0,14	0	-1,564	1	0	1	1,674	0	-1,232	THIC_ARATH	Phosphomethylpyrimidine synthase, chloroplastic (EC 4.1.99.17) (Hydroxymethylpyrimidine phosphate synthase) (HMP-P synthase) (HMP-phosphate synthase) (HMPP synthase) (Protein PYRIMIDINE REQUIRING) (Thiamine biosynthesis protein ThiC) (Protein THIAMINE C)	THIC PY At2g29630 T27A16.27	Arabidopsis thaliana (Mouse-ear cress)	PATHWAY: Cofactor biosynthesis; thiamine diphosphate biosynthesis.	3D-structure; 4Fe-4S; Chloroplast; Complete proteome; Iron; Iron-sulfur; Lyase; Metal-binding; Plastid; Reference proteome; S-adenosyl-L-methionine; Thiamine biosynthesis; Transit peptide; Zinc
SRR122238_TR7314_c0_g1_i4_len=1143	XTH9_ARATH	Q:122-994,H:10-289	72.16%ID	E:3e-158	Xyloglucan endotransglucosylase/hydrolase protein 9	PF00722.18^Glyco_hydro_16^Glycosyl hydrolases family 16^63-239^E:3.2e-57`PF06955.9^XET_C^Xyloglucan endo-transglycosylase (XET) C-terminus^281-326^E:1.8e-17	0,002	-0,822	0	-3,848	0,611	0,417	1	0,148	0	-0,848	XTH9_ARATH	Xyloglucan endotransglucosylase/hydrolase protein 9 (At-XTH9) (XTH-9) (EC 2.4.1.207)	XTH9 EXGT-A6 XTR16 At4g03210 F4C21.14	Arabidopsis thaliana (Mouse-ear cress)		Alternative splicing; Apoplast; Cell wall; Cell wall biogenesis/degradation; Complete proteome; Disulfide bond; Glycoprotein; Glycosidase; Hydrolase; Reference proteome; Secreted; Signal; Transferase
SRR122238_TR14630_c0_g2_i2_len=1271	GLYM2_FLAPR	Q:1233-289,H:209-517	86.98%ID	E:0	Serine hydroxymethyltransferase 2, mitochondrial	PF00464.16^SHMT^Serine hydroxymethyltransferase^2-240^E:2.9e-122	1	-0,006	0	-0,861	0,034	-0,476	0,005	-0,633	0	-1,614	GLYM2_FLAPR	Serine hydroxymethyltransferase 2, mitochondrial (SHMT2) (EC 2.1.2.1) (Glycine hydroxymethyltransferase 2) (Serine methylase 2)		Flaveria pringlei	PATHWAY: One-carbon metabolism; tetrahydrofolate interconversion.	Mitochondrion; One-carbon metabolism; Pyridoxal phosphate; Transferase; Transit peptide
SRR122238_TR41140_c0_g1_i1_len=1134	RK3_TOBAC	Q:971-216,H:8-259	82.42%ID	E:2e-123	50S ribosomal protein L3, chloroplastic	PF00297.19^Ribosomal_L3^Ribosomal protein L3^185-272^E:2.2e-17	0,095	-0,738	0,001	-1,614	0,683	0,285	1	0,118	0	-1,172	RK3_TOBAC	50S ribosomal protein L3, chloroplastic (Fragment)	RPL3	Nicotiana tabacum (Common tobacco)		Chloroplast; Plastid; RNA-binding; Ribonucleoprotein; Ribosomal protein; Transit peptide; rRNA-binding
SRR122238_TR45252_c0_g1_i1_len=1771	RR1_SPIOL	Q:1648-380,H:1-408	81.84%ID	E:0	30S ribosomal protein S1, chloroplastic	PF00575.20^S1^S1 RNA binding domain^109-181^E:1.3e-05`PF00575.20^S1^S1 RNA binding domain^197-261^E:1.8e-08`PF00575.20^S1^S1 RNA binding domain^274-344^E:3.9e-22	0,307	-0,243	0	-0,907	0,013	-0,603	0,083	-0,417	0,001	-0,842	RR1_SPIOL	30S ribosomal protein S1, chloroplastic (CS1)	RPS1	Spinacia oleracea (Spinach)		Chloroplast; Direct protein sequencing; Plastid; RNA-binding; Repeat; Ribonucleoprotein; Ribosomal protein; Transit peptide
SRR122238_TR45790_c0_g1_i7_len=1429	COL5_ARATH	Q:1243-545,H:149-355	40.59%ID	E:7e-33	Zinc finger protein CONSTANS-LIKE 5	PF06203.11^CCT^CCT motif^182-224^E:4.2e-18	1	0	0	-7,224	1	0	1	0	0	-8,413	COL5_ARATH	Zinc finger protein CONSTANS-LIKE 5	COL5 At5g57660 MRI1.1	Arabidopsis thaliana (Mouse-ear cress)		Complete proteome; Metal-binding; Nucleus; Reference proteome; Repeat; Zinc; Zinc-finger
SRR122238_TR50273_c0_g1_i13_len=1803	HACD2_BOVIN	Q:1474-545,H:149-201	39.55%ID	E:2e-21	Very-long-chain (3R)-3-hydroxyacyl-CoA dehydratase 2 {ECO:0000305}	PF04387.11^PTPLA^Protein tyrosine phosphatase-like protein, PTPLA^1-135^E:3.7e-33	0,132	-2,183	0	-6,151	1	-0,768	1	1,252	0	-3,199	HACD2_BOVIN	Very-long-chain (3R)-3-hydroxyacyl-CoA dehydratase 2 (EC 4.2.1.134) (3-hydroxyacyl-CoA dehydratase 2) (HACD2) (Protein-tyrosine phosphatase-like member B)	HACD2 PTPLB	Bos taurus (Bovine)	PATHWAY: Lipid metabolism; fatty acid biosynthesis. {ECO:0000250|UniProtKB:Q6Y1H2}.	Acetylation; Complete proteome; Endoplasmic reticulum; Fatty acid biosynthesis; Fatty acid metabolism; Glycoprotein; Lipid biosynthesis; Lipid metabolism; Lyase; Membrane; Reference proteome; Transmembrane; Transmembrane helix
SRR122238_TR64958_c0_g1_i3_len=853	CPNB1_ARATH	Q:731-387,H:486-600	86.96%ID	E:1e-59	Chaperonin 60 subunit beta 1, chloroplastic	PF00118.21^Cpn60_TCP1^TCP-1/cpn60 chaperonin family^8-97^E:1.2e-23	0,006	-0,685	0	-1,173	0,11	0,537	0,046	0,646	0	-0,849	CPNB1_ARATH	Chaperonin 60 subunit beta 1, chloroplastic (CPN-60 beta 1) (60 kDa chaperonin subunit beta 1) (RuBisCO large subunit-binding protein subunit beta, chloroplastic)	CPN60B1 Cpn60-B(3) LEN1 At1g55490 T5A14.11	Arabidopsis thaliana (Mouse-ear cress)		ATP-binding; Chaperone; Chloroplast; Complete proteome; Direct protein sequencing; Nucleotide-binding; Phosphoprotein; Plastid; Reference proteome; Transit peptide
SRR122238_TR83367_c0_g1_i1_len=716	NSA2_HUMAN	Q:661-311,H:144-260	82.91%ID	E:2e-64	Ribosome biogenesis protein NSA2 homolog	.	0,461	1,518	0	-2,445	1	0	1	0	0	-5,232	NSA2_HUMAN	Ribosome biogenesis protein NSA2 homolog (Hairy cell leukemia protein 1) (TGF-beta-inducible nuclear protein 1)	NSA2 TINP1 HUSSY-29	Homo sapiens (Human)		Complete proteome; Nucleus; Phosphoprotein; Polymorphism; Reference proteome; Ribonucleoprotein; Ribosome biogenesis; rRNA processing
SRR1271857_TR18303_c0_g1_i1_len=1310	RUB1_BRANA	Q:1131-361,H:292-546	86.38%ID	E:2e-135	RuBisCO large subunit-binding protein subunit alpha, chloroplastic	PF00118.21^Cpn60_TCP1^TCP-1/cpn60 chaperonin family^25-242^E:1.8e-42	0,133	-1,161	0	-3,912	1	-1,038	1	-1,083	0	-3,863	RUB1_BRANA	RuBisCO large subunit-binding protein subunit alpha, chloroplastic (60 kDa chaperonin subunit alpha) (CPN-60 alpha) (Fragment)		Brassica napus (Rape)		ATP-binding; Chaperone; Chloroplast; Nucleotide-binding; Phosphoprotein; Plastid; Transit peptide
SRR1271858_TR414_c0_g1_i1_len=1581	DHEB_NICPL	Q:1369-356,H:74-411	89.94%ID	E:0	Glutamate dehydrogenase B	PF02812.15^ELFV_dehydrog_N^Glu/Leu/Phe/Val dehydrogenase, dimerisation domain^6-91^E:7.1e-31`PF00208.18^ELFV_dehydrog^Glutamate/Leucine/Phenylalanine/Valine dehydrogenase^108-340^E:6.3e-73	0,147	0,906	0	-1,543	0,781	0,47	1	0,287	0,001	-1,893	DHEB_NICPL	Glutamate dehydrogenase B (GDH B) (EC 1.4.1.3)	GDHB	Nicotiana plumbaginifolia (Leadwort-leaved tobacco) (Tex-Mex tobacco)		NAD; Oxidoreductase
SRR1271858_TR55352_c0_g1_i2_len=1138	PI5K9_ARATH	Q:94-867,H:554-812	75.57%ID	E:9e-129	Phosphatidylinositol 4-phosphate 5-kinase 9	PF01504.15^PIP5K^Phosphatidylinositol-4-phosphate 5-Kinase^1-247^E:1.4e-50	0,017	-5,869	0,001	-1,884	1	-0,404	0,129	-4,919	0	-2,721	PI5K9_ARATH	Phosphatidylinositol 4-phosphate 5-kinase 9 (AtPIP5K9) (EC 2.7.1.68) (1-phosphatidylinositol 4-phosphate kinase 9) (Diphosphoinositide kinase 9) (PtdIns(4)P-5-kinase 9)	PIP5K9 At3g09920 F8A24.2	Arabidopsis thaliana (Mouse-ear cress)		ATP-binding; Complete proteome; Kinase; Membrane; Nucleotide-binding; Nucleus; Reference proteome; Repeat; Transferase
SRR1271858_TR60176_c0_g1_i1_len=1053	VATA_ARATH	Q:390-593,H:556-623	89.71%ID	E:5e-31	V-type proton ATPase catalytic subunit A	.	1	0,417	0	-3,54	1	-0,953	1	-0,769	0	-2,964	VATA_ARATH	V-type proton ATPase catalytic subunit A (V-ATPase subunit A) (EC 3.6.3.14) (V-ATPase 69 kDa subunit) (Vacuolar H(+)-ATPase subunit A) (Vacuolar proton pump subunit alpha)	VHA-A At1g78900 F9K20.5	Arabidopsis thaliana (Mouse-ear cress)		ATP-binding; Complete proteome; Hydrogen ion transport; Hydrolase; Ion transport; Membrane; Nucleotide-binding; Reference proteome; Transport; Vacuole
SRR1271859_TR4946_c0_g3_i5_len=638	CB2D_SOLLC	Q:608-276,H:6-116	93.69%ID	E:4e-70	Chlorophyll a-b binding protein 1D	.	0,458	0,155	0	-2,667	0	1,036	0,004	0,613	0	-3,844	CB2D_SOLLC	Chlorophyll a-b binding protein 1D (LHCII type I CAB-1D) (LHCP) (Fragment)	CAB1D	Solanum lycopersicum (Tomato) (Lycopersicon esculentum)		Chlorophyll; Chloroplast; Chromophore; Complete proteome; Magnesium; Membrane; Metal-binding; Phosphoprotein; Photosynthesis; Photosystem I; Photosystem II; Plastid; Reference proteome; Thylakoid; Transmembrane; Transmembrane helix
SRR1271859_TR5902_c0_g2_i1_len=668	CLPP6_ARATH	Q:143-334,H:208-271	87.5%ID	E:8e-33	ATP-dependent Clp protease proteolytic subunit 6, chloroplastic {ECO:0000303|PubMed:11299370}	.	0,068	1,067	0	-1,94	0,177	0,747	1	0,052	0	-1,577	CLPP6_ARATH	ATP-dependent Clp protease proteolytic subunit 6, chloroplastic (EC 3.4.21.92) (Endopeptidase ClpP6) (nClpP6) (nClpP1)	CLPP6 NCLPP1 NCLPP6 At1g11750 F25C20.10	Arabidopsis thaliana (Mouse-ear cress)		3D-structure; Alternative splicing; Chloroplast; Complete proteome; Direct protein sequencing; Hydrolase; Plastid; Protease; Reference proteome; Serine protease; Transit peptide
SRR1271859_TR21277_c0_g2_i5_len=870	EDL16_ARATH	Q:157-534,H:386-479	58.73%ID	E:4e-35	Sugar transporter ERD6-like 16	PF00083.21^Sugar_tr^Sugar (and other) transporter^38-115^E:2.5e-13	0,043	-1,279	0	-2,46	0,001	-3,175	0,001	-3,684	0	-1,642	EDL16_ARATH	Sugar transporter ERD6-like 16	At5g18840 F17K4.90	Arabidopsis thaliana (Mouse-ear cress)		Complete proteome; Membrane; Reference proteome; Sugar transport; Transmembrane; Transmembrane helix; Transport
SRR1271859_TR28887_c0_g1_i4_len=1649	GDI2_ARATH	Q:263-1054,H:173-436	87.12%ID	E:2e-167	Guanosine nucleotide diphosphate dissociation inhibitor 2	PF00996.15^GDI^GDP dissociation inhibitor^1-226^E:1.9e-113	1	0,893	0	-3,533	1	0,088	1	-0,57	0	-2,56	GDI2_ARATH	Guanosine nucleotide diphosphate dissociation inhibitor 2 (AtGDI2)	GDI2 At3g59920 F24G16.190	Arabidopsis thaliana (Mouse-ear cress)		Complete proteome; GTPase activation; Reference proteome
SRR342017_TR756_c1_g3_i3_len=811	PSBP_SPIOL	Q:76-567,H:104-267	83.54%ID	E:1e-96	Oxygen-evolving enhancer protein 2, chloroplastic	PF01789.13^PsbP^PsbP^26-188^E:1.6e-38	1	-0,31	0	-7,903	0,629	1,248	0,043	2,619	0	-2,658	PSBP_SPIOL	Oxygen-evolving enhancer protein 2, chloroplastic (OEE2) (23 kDa subunit of oxygen evolving system of photosystem II) (23 kDa thylakoid membrane protein) (OEC 23 kDa subunit)	PSBP	Spinacia oleracea (Spinach)		3D-structure; Chloroplast; Direct protein sequencing; Membrane; Photosynthesis; Photosystem II; Plastid; Thylakoid; Transit peptide
SRR342017_TR3199_c0_g5_i1_len=1156	NA	NA	NA	NA	NA	.	0,365	0,568	0	-1,631	0,001	6,552	1	0	0	-1,919	.	.	.	.	.	.
SRR342017_TR24038_c0_g1_i3_len=1877	XTH9_ARATH	Q:639-211,H:157-289	71.33%ID	E:9e-87	Xyloglucan endotransglucosylase/hydrolase protein 9	.	0,001	-1,162	0	-3,189	0,299	-1,786	0,034	-3,573	0	-1,069	XTH9_ARATH	Xyloglucan endotransglucosylase/hydrolase protein 9 (At-XTH9) (XTH-9) (EC 2.4.1.207)	XTH9 EXGT-A6 XTR16 At4g03210 F4C21.14	Arabidopsis thaliana (Mouse-ear cress)		Alternative splicing; Apoplast; Cell wall; Cell wall biogenesis/degradation; Complete proteome; Disulfide bond; Glycoprotein; Glycosidase; Hydrolase; Reference proteome; Secreted; Signal; Transferase
SRR342017_TR26185_c0_g2_i3_len=833	CBSX5_ARATH	Q:90-659,H:193-389	52.79%ID	E:2e-42	CBS domain-containing protein CBSX5	.	0,129	-5,153	0	-8,119	1	0	1	0	0	-1,632	CBSX5_ARATH	CBS domain-containing protein CBSX5	CBSX5 At4g27460 F27G19.60	Arabidopsis thaliana (Mouse-ear cress)		CBS domain; Complete proteome; Reference proteome; Repeat
SRR342017_TR30787_c0_g2_i2_len=1214	EP1G_DAUCA	Q:333-629,H:263-373	48.65%ID	E:2e-25	Epidermis-specific secreted glycoprotein EP1	PF08276.8^PAN_2^PAN-like domain^59-88^E:2.2e-06`PF00024.23^PAN_1^PAN domain^60-88^E:0.00014	1	-1,051	0	-5,341	1	-1,282	1	1,492	0	-3,325	EP1G_DAUCA	Epidermis-specific secreted glycoprotein EP1 (52/54 kDa medium protein)	EP1	Daucus carota (Wild carrot)		Direct protein sequencing; Glycoprotein; Lectin; Secreted; Signal
SRR342019_TR19968_c3_g12_i11_len=818	VICHY_VICSN	Q:148-525,H:384-509	61.11%ID	E:9e-46	Vicianin hydrolase {ECO:0000312|EMBL:ABD03937.1}	PF00232.15^Glyco_hydro_1^Glycosyl hydrolase family 1^3-128^E:3e-38	0,76	0,747	0	-3,14	1	-3,165	1	-3,165	0	-1,648	VICHY_VICSN	Vicianin hydrolase (VH) (EC 3.2.1.119) (Fragment)		Vicia sativa subsp. nigra (Common vetch) (Vicia angustifolia)		Direct protein sequencing; Disulfide bond; Glycoprotein; Glycosidase; Hydrolase; Signal
SRR342019_TR25267_c2_g1_i2_len=663	PGKH_WHEAT	Q:610-371,H:390-469	96.25%ID	E:3e-33	Phosphoglycerate kinase, chloroplastic	PF00162.16^PGK^Phosphoglycerate kinase^18-89^E:5.7e-29	1	0,164	0	-1,913	0,892	-0,152	0,675	-0,305	0	-4,904	PGKH_WHEAT	Phosphoglycerate kinase, chloroplastic (EC 2.7.2.3)		Triticum aestivum (Wheat)	PATHWAY: Carbohydrate biosynthesis; Calvin cycle.	ATP-binding; Calvin cycle; Chloroplast; Complete proteome; Kinase; Nucleotide-binding; Plastid; Reference proteome; Transferase; Transit peptide
SRR342019_TR27500_c1_g2_i3_len=1179	EF1A_TOBAC	Q:1113-517,H:238-436	97.99%ID	E:3e-135	Elongation factor 1-alpha	PF03144.22^GTP_EFTU_D2^Elongation factor Tu domain 2^7-50^E:2.2e-07`PF03143.14^GTP_EFTU_D3^Elongation factor Tu C-terminal domain^59-166^E:5.5e-39	0,003	-0,893	0	-1,413	0	0,967	0,576	0,17	0	-2,412	EF1A_TOBAC	Elongation factor 1-alpha (EF-1-alpha) (Vitronectin-like adhesion protein 1) (PVN1)		Nicotiana tabacum (Common tobacco)		Cytoplasm; Elongation factor; GTP-binding; Nucleotide-binding; Phosphoprotein; Protein biosynthesis
SRR342019_TR35318_c0_g1_i1_len=928	NA	NA	NA	NA	NA	PF02325.14^YGGT^YGGT family^131-200^E:2.5e-15	0,791	-0,087	0	-1,183	0,18	-0,472	0,31	-0,37	0	-6,178	.	.	.	.	.	.
SRR342019_TR38162_c5_g11_i3_len=817	TIP11_ARATH	Q:765-373,H:121-251	82.44%ID	E:8e-62	Aquaporin TIP1-1	PF00230.17^MIP^Major intrinsic protein^1-84^E:5.9e-31	0,798	0,49	0	-3,051	0,206	0,752	0,458	0,493	0	-4,155	TIP11_ARATH	Aquaporin TIP1-1 (Aquaporin TIP) (Gamma-tonoplast intrinsic protein) (Gamma-TIP) (Tonoplast intrinsic protein 1-1) (AtTIP1;1) (Tonoplast intrinsic protein, root-specific RB7) [Cleaved into: Aquaporin TIP1-1, N-terminally processed]	TIP1-1 AQP.1 At2g36830 T1J8.1	Arabidopsis thaliana (Mouse-ear cress)		Acetylation; Complete proteome; Membrane; Reference proteome; Repeat; Transmembrane; Transmembrane helix; Transport; Vacuole
SRR342022_TR24849_c2_g7_i1_len=3139	GLGB1_PEA	Q:2542-1037,H:342-846	85.74%ID	E:0	1,4-alpha-glucan-branching enzyme 1, chloroplastic/amyloplastic	PF00128.21^Alpha-amylase^Alpha amylase, catalytic domain^15-86^E:9.1e-13`PF02806.15^Alpha-amylase_C^Alpha amylase, C-terminal all-beta domain^404-498^E:1.9e-25	0,534	-0,402	0	-2,129	0,593	-0,671	0,239	-1,135	0	-4,28	GLGB1_PEA	1,4-alpha-glucan-branching enzyme 1, chloroplastic/amyloplastic (EC 2.4.1.18) (Starch branching enzyme I)	SBEI	Pisum sativum (Garden pea)	PATHWAY: Glycan biosynthesis; starch biosynthesis.	Amyloplast; Chloroplast; Direct protein sequencing; Plastid; Transferase; Transit peptide
SRR342022_TR24889_c4_g1_i1_len=565	NA	NA	NA	NA	NA	.	0,812	-0,187	0	-5,712	0,131	-0,496	0,011	-0,861	0	-3,916	.	.	.	.	.	.
SRR342022_TR27316_c4_g3_i1_len=1414	SYNC1_ARATH	Q:532-1218,H:342-572	74.46%ID	E:2e-119	Asparagine–tRNA ligase, cytoplasmic 1 {ECO:0000305}	PF00152.17^tRNA-synt_2^tRNA synthetases class II (D, K and N)^1-199^E:2e-41	0,629	1,285	0	-2,681	1	-0,719	1	0,282	0	-2,469	SYNC1_ARATH	Asparagine–tRNA ligase, cytoplasmic 1 (EC 6.1.1.22) (Asparaginyl-tRNA synthetase 1) (AsnRS 1) (Protein EMBRYO DEFECTIVE 2755)	SYNC1 EMB2755 At5g56680 MIK19.13	Arabidopsis thaliana (Mouse-ear cress)		ATP-binding; Acetylation; Aminoacyl-tRNA synthetase; Complete proteome; Cytoplasm; DNA-binding; Ligase; Nucleotide-binding; Protein biosynthesis; Reference proteome
SRR342022_TR28853_c0_g3_i2_len=1326	UBC2_WHEAT	Q:532-987,H:1-152	96.05%ID	E:2e-103	Ubiquitin-conjugating enzyme E2 2	PF00179.23^UQ_con^Ubiquitin-conjugating enzyme^8-144^E:1.9e-52	0,03	0,73	0,001	-1,217	0,074	0,535	0,067	0,546	0	-2,916	UBC2_WHEAT	Ubiquitin-conjugating enzyme E2 2 (EC 2.3.2.23) (E2 ubiquitin-conjugating enzyme 2) (Ubiquitin carrier protein 2) (Ubiquitin-conjugating enzyme E2-16 kDa) (Ubiquitin-protein ligase 2)	UBC2 UBC1	Triticum aestivum (Wheat)	PATHWAY: Protein modification; protein ubiquitination. {ECO:0000255|PROSITE-ProRule:PRU00388}.	ATP-binding; Complete proteome; Nucleotide-binding; Reference proteome; Transferase; Ubl conjugation pathway
SRR342023_TR10339_c0_g2_i1_len=1669	FLS2_ARATH	Q:238-1338,H:25-467	31.07%ID	E:1e-34	LRR receptor-like serine/threonine-protein kinase FLS2	PF08263.9^LRRNT_2^Leucine rich repeat N-terminal domain^33-66^E:3.6e-05`PF00560.30^LRR_1^Leucine Rich Repeat^114-134^E:0.046`PF13855.3^LRR_8^Leucine rich repeat^161-219^E:9.2e-09`PF00560.30^LRR_1^Leucine Rich Repeat^162-183^E:0.07`PF13516.3^LRR_6^Leucine Rich repeat^184-199^E:1.7`PF13855.3^LRR_8^Leucine rich repeat^230-289^E:5.8e-09`PF13516.3^LRR_6^Leucine Rich repeat^254-267^E:1.7`PF00560.30^LRR_1^Leucine Rich Repeat^255-273^E:0.27	0,147	-0,458	0	-1,895	0,352	-0,951	0,5	-0,701	0	-1,855	FLS2_ARATH	LRR receptor-like serine/threonine-protein kinase FLS2 (EC 2.7.11.1) (Protein FLAGELLIN-SENSING 2) (Protein FLAGELLIN-SENSITIVE 2)	FLS2 At5g46330 MPL12.13 MPL12.8	Arabidopsis thaliana (Mouse-ear cress)		3D-structure; ATP-binding; Cell membrane; Complete proteome; Disulfide bond; Endosome; Glycoprotein; Kinase; Leucine-rich repeat; Membrane; Nucleotide-binding; Phosphoprotein; Plant defense; Receptor; Reference proteome; Repeat; Serine/threonine-protein kinase; Signal; Transferase; Transmembrane; Transmembrane helix; Ubl conjugation; Ubl conjugation pathway
SRR342023_TR15713_c0_g2_i5_len=1971	XTH33_ARATH	Q:1010-1852,H:26-309	61.19%ID	E:5e-128	Probable xyloglucan endotransglucosylase/hydrolase protein 33	PF00722.18^Glyco_hydro_16^Glycosyl hydrolases family 16^4-170^E:6.1e-51`PF06955.9^XET_C^Xyloglucan endo-transglycosylase (XET) C-terminus^212-249^E:1.6e-12	0,793	0,141	0	-1,897	0,146	-0,509	0,008	-0,958	0	-3,05	XTH33_ARATH	Probable xyloglucan endotransglucosylase/hydrolase protein 33 (At-XTH33) (XTH-33) (EC 2.4.1.207)	XTH33 At1g10550 T10O24.17	Arabidopsis thaliana (Mouse-ear cress)		Apoplast; Cell wall; Cell wall biogenesis/degradation; Complete proteome; Disulfide bond; Glycoprotein; Glycosidase; Hydrolase; Reference proteome; Secreted; Signal; Transferase
SRR342023_TR20256_c0_g2_i1_len=506	EFGC_ARATH	Q:40-504,H:511-665	94.84%ID	E:2e-97	Elongation factor G, chloroplastic {ECO:0000255|HAMAP-Rule:MF_03063}	PF14492.3^EFG_II^Elongation Factor G, domain II^1-24^E:8.9e-08`PF03764.15^EFG_IV^Elongation factor G, domain IV^26-125^E:9.1e-40	0,007	-0,71	0	-1,651	0,006	-0,616	0,461	-0,165	0	-5,803	EFGC_ARATH	Elongation factor G, chloroplastic (cEF-G) (Elongation factor EF-G/SCO1) (Protein SNOWY COTYLEDON 1, chloroplastic) (AtSCO1)	CPEFG SCO1 At1g62750 F23N19.11	Arabidopsis thaliana (Mouse-ear cress)	PATHWAY: Protein biosynthesis; polypeptide chain elongation. {ECO:0000255|HAMAP-Rule:MF_03063}.	Chloroplast; Complete proteome; Elongation factor; GTP-binding; Nucleotide-binding; Plastid; Protein biosynthesis; Reference proteome; Transit peptide
SRR342023_TR29686_c4_g4_i4_len=2952	EDA2_ARATH	Q:269-1621,H:37-489	66%ID	E:0	Probable serine protease EDA2	PF05577.9^Peptidase_S28^Serine carboxypeptidase S28^58-445^E:6.3e-77	0,367	-0,254	0	-1,179	0,02	-0,494	0,158	-0,299	0	-7,569	EDA2_ARATH	Probable serine protease EDA2 (EC 3.4.-.-) (Protein EMBRYO SAC DEVELOPMENT ARREST 2)	EDA2 At2g18080 T27K22.5	Arabidopsis thaliana (Mouse-ear cress)		Complete proteome; Glycoprotein; Hydrolase; Protease; Reference proteome; Secreted; Serine protease; Signal
SRR646572_TR16443_c0_g1_i1_len=1014	PSAL_ARATH	Q:213-803,H:23-219	82.74%ID	E:3e-93	Photosystem I reaction center subunit XI, chloroplastic	PF02605.12^PsaL^Photosystem I reaction centre subunit XI^60-212^E:1.1e-59	0,601	0,111	0	-0,919	0,036	-0,437	0,106	-0,336	0	-6,196	PSAL_ARATH	Photosystem I reaction center subunit XI, chloroplastic (PSI-L) (PSI subunit V)	PSAL At4g12800 T20K18.150	Arabidopsis thaliana (Mouse-ear cress)		3D-structure; Chloroplast; Complete proteome; Membrane; Photosynthesis; Photosystem I; Plastid; Reference proteome; Thylakoid; Transit peptide; Transmembrane; Transmembrane helix
SRR646604_TR4091_c0_g1_i1_len=1704	AT74_ARATH	Q:264-1271,H:9-275	43.15%ID	E:1e-80	Phosphoglycerate mutase-like protein AT74 {ECO:0000305}	PF00300.19^His_Phos_1^Histidine phosphatase superfamily (branch 1)^73-173^E:2.9e-13	0,745	-0,153	0	-2,097	0,534	0,43	1	0,106	0	-1,981	AT74_ARATH	Phosphoglycerate mutase-like protein AT74 (At-74)	At3g05170 T12H1.14	Arabidopsis thaliana (Mouse-ear cress)		Complete proteome; Reference proteome
SRR646604_TR18082_c0_g1_i10_len=3201	RSH1L_ARATH	Q:2766-301,H:1-882	64.83%ID	E:0	Putative GTP diphosphokinase RSH1, chloroplastic	PF13328.3^HD_4^HD domain^91-248^E:1.3e-42`PF01966.19^HD^HD domain^111-217^E:1.3e-12`PF04607.14^RelA_SpoT^Region found in RelA / SpoT proteins^316-433^E:1.4e-22`PF02824.18^TGS^TGS domain^504-563^E:3.4e-19	0,189	-1,521	0	-2,482	0,034	5,678	1	0	0	-6,839	RSH1L_ARATH	Putative GTP diphosphokinase RSH1, chloroplastic (EC 2.7.6.5) (RelA/SpoT homolog 1) (AtRSH1) (ppGpp synthetase RSH1)	RSH1	Arabidopsis thaliana (Mouse-ear cress)		ATP-binding; Chloroplast; GTP-binding; Kinase; Nucleotide-binding; Plastid; Stress response; Transferase; Transit peptide
SRR646604_TR21591_c0_g1_i1_len=1410	NA	NA	NA	NA	NA	PF03168.10^LEA_2^Late embryogenesis abundant protein^97-197^E:2.1e-05	0,76	-0,106	0	-2,626	1	0,017	1	-0,029	0	-1,972	.	.	.	.	.	.
SRR648705_TR867_c0_g1_i1_len=914	NA	NA	NA	NA	NA	.	0,326	0,488	0	-1,685	0,474	-0,35	0,211	-0,636	0	-1,509	.	.	.	.	.	.
SRR648705_TR5023_c0_g1_i1_len=743	NDK4_ARATH	Q:634-407,H:160-235	96.05%ID	E:4e-45	Nucleoside diphosphate kinase IV, chloroplastic/mitochondrial	.	0,335	0,723	0	-1,598	0,01	2,353	0,027	2,156	0	-3,673	NDK4_ARATH	Nucleoside diphosphate kinase IV, chloroplastic/mitochondrial (NDK IV) (NDP kinase IV) (NDPK IV) (EC 2.7.4.6) (Nucleoside diphosphate kinase 4)	NDK4 At4g23900 T32A16.70	Arabidopsis thaliana (Mouse-ear cress)		ATP-binding; Chloroplast; Complete proteome; Direct protein sequencing; Kinase; Magnesium; Metal-binding; Mitochondrion; Nucleotide metabolism; Nucleotide-binding; Plastid; Reference proteome; Thylakoid; Transferase; Transit peptide
SRR648705_TR8275_c0_g2_i1_len=677	PMTQ_ARATH	Q:142-369,H:754-829	67.11%ID	E:5e-28	Probable methyltransferase PMT26	.	1	-0,298	0	-1,722	0,044	2,186	0,502	-4,194	0	-5,534	PMTQ_ARATH	Probable methyltransferase PMT26 (EC 2.1.1.-)	At5g64030 MBM17.13	Arabidopsis thaliana (Mouse-ear cress)		Complete proteome; Glycoprotein; Golgi apparatus; Membrane; Methyltransferase; Reference proteome; Signal-anchor; Transferase; Transmembrane; Transmembrane helix
SRR648705_TR18576_c0_g1_i1_len=417	NA	NA	NA	NA	NA	.	0,818	-0,062	0	-1,657	0,05	0,721	0,522	0,252	0	-0,882	.	.	.	.	.	.
SRR648705_TR22426_c0_g1_i1_len=1622	PANK2_ARATH	Q:282-1202,H:592-898	88.27%ID	E:6e-173	Pantothenate kinase 2	PF01937.16^DUF89^Protein of unknown function DUF89^16-292^E:3.6e-43	1	0	0	-6,953	1	0	1	0	0	-6,889	PANK2_ARATH	Pantothenate kinase 2 (AtPANK2) (EC 2.7.1.33) (Pantothenic acid kinase 2)	PANK2 At4g32180 F10M6.180 F10N7.10	Arabidopsis thaliana (Mouse-ear cress)	PATHWAY: Cofactor biosynthesis; coenzyme A biosynthesis; CoA from (R)-pantothenate: step 1/5.	ATP-binding; Alternative splicing; Coenzyme A biosynthesis; Complete proteome; Kinase; Nucleotide-binding; Reference proteome; Transferase
SRR648705_TR35082_c0_g1_i2_len=823	NA	NA	NA	NA	NA	.	1	0,089	0	-3,121	0,254	-2,367	1	-0,654	0,001	-0,976	.	.	.	.	.	.
SRR648705_TR35323_c0_g2_i1_len=514	NA	NA	NA	NA	NA	.	1	-3,66	0	-1,375	0	6,869	0,001	6,413	0	-2,501	.	.	.	.	.	.
SRR648705_TR36841_c1_g1_i3_len=2314	GPDL4_ARATH	Q:573-1772,H:319-716	51.86%ID	E:7e-126	Glycerophosphodiester phosphodiesterase GDPDL4 {ECO:0000305}	PF03009.14^GDPD^Glycerophosphoryl diester phosphodiesterase family^18-307^E:2.5e-22	1	0,732	0	-5,175	1	-0,863	0,502	-4,14	0	-4,988	GPDL4_ARATH	Glycerophosphodiester phosphodiesterase GDPDL4 (EC 3.1.4.46) (Glycerophosphodiester phosphodiesterase-like 4) (ATGDPDL4) (Glycerophosphodiesterase-like 1) (Protein SHV3-LIKE 1)	GDPDL4 GPDL1 SVL1 At5g55480 MTE17.19	Arabidopsis thaliana (Mouse-ear cress)		Cell membrane; Cell wall biogenesis/degradation; Complete proteome; GPI-anchor; Glycerol metabolism; Glycoprotein; Hydrolase; Lipoprotein; Membrane; Reference proteome; Repeat; Signal
SRR648707_TR14283_c1_g2_i5_len=779	CGS1_ARATH	Q:97-345,H:481-563	84.34%ID	E:2e-40	Cystathionine gamma-synthase 1, chloroplastic {ECO:0000305}	.	0,691	0,208	0	-1,307	0	6,697	1	0	0	-3,012	CGS1_ARATH	Cystathionine gamma-synthase 1, chloroplastic (AtCGS1) (EC 2.5.1.48) (METHIONINE OVERACCUMULATION 1) (O-succinylhomoserine (thiol)-lyase)	CGS1 CYS1 MTO1 At3g01120 T4P13.19	Arabidopsis thaliana (Mouse-ear cress)	PATHWAY: Amino-acid biosynthesis; L-methionine biosynthesis via de novo pathway; L-cystathionine from O-succinyl-L-homoserine: step 1/1. {ECO:0000305}.	Amino-acid biosynthesis; Chloroplast; Complete proteome; Direct protein sequencing; Methionine biosynthesis; Plastid; Polymorphism; Pyridoxal phosphate; Reference proteome; Transferase; Transit peptide
SRR648707_TR32126_c1_g2_i1_len=1187	CSK2B_ARATH	Q:252-653,H:153-286	85.07%ID	E:2e-80	Casein kinase II subunit beta	PF01214.15^CK_II_beta^Casein kinase II regulatory subunit^3-129^E:4.7e-57	0,017	5,758	0	-3,233	0,066	1,725	0,239	1,352	0	-3,556	CSK2B_ARATH	Casein kinase II subunit beta (CK II beta)	CKB1 At5g47080 K14A3.3	Arabidopsis thaliana (Mouse-ear cress)		Alternative splicing; Complete proteome; Phosphoprotein; Reference proteome
SRR924147_TR22586_c8_g1_i2_len=1185	NA	NA	NA	NA	NA	.	0,643	-0,487	0,001	-1,644	0,545	0,561	0,545	0,481	0	-3,019	.	.	.	.	.	.
SRR924148_TR8190_c0_g1_i3_len=1388	GLNA_VIGAC	Q:100-1053,H:39-356	91.82%ID	E:0	Glutamine synthetase nodule isozyme	PF00120.21^Gln-synt_C^Glutamine synthetase, catalytic domain^34-257^E:9.9e-17	0,017	-5,729	0	-2,058	0,798	0,332	0,034	-5,634	0	-6,866	GLNA_VIGAC	Glutamine synthetase nodule isozyme (GS) (EC 6.3.1.2) (Glutamate–ammonia ligase)		Vigna aconitifolia (Moth bean) (Phaseolus aconitifolius)		ATP-binding; Cytoplasm; Ligase; Nitrogen fixation; Nucleotide-binding
SRR924148_TR15002_c0_g1_i1_len=653	NA	NA	NA	NA	NA	PF14547.3^Hydrophob_seed^Hydrophobic seed protein^56-139^E:1.2e-27	0,017	-5,763	0	-8,197	1	1,328	1	-2,809	0	-1,667	.	.	.	.	.	.
SRR1144634_TR22741_c0_g1_i1_len=620	TBA1_ANEPH	Q:1-396,H:301-432	99.24%ID	E:5e-92	Tubulin alpha-1 chain	PF03953.14^Tubulin_C^Tubulin C-terminal domain^1-91^E:6.9e-35	0	-0,815	0	-1,481	0,591	0,177	1	0,005	0	-1,105	TBA1_ANEPH	Tubulin alpha-1 chain	TUBA1	Anemia phyllitidis (Fern) (Osmunda phyllitidis)		Acetylation; Cytoplasm; Cytoskeleton; GTP-binding; Microtubule; Nucleotide-binding
SRR1144634_TR24801_c1_g1_i1_len=506	LUT1_ARATH	Q:505-299,H:471-539	82.61%ID	E:3e-33	Carotene epsilon-monooxygenase, chloroplastic	.	0	-7,934	0	-9,837	1	0	1	0	0	-1,707	LUT1_ARATH	Carotene epsilon-monooxygenase, chloroplastic (EC 1.14.99.45) (Cytochrome P450 97C1) (Protein LUTEIN DEFICIENT 1)	CYP97C1 LUT1 At3g53130 T4D2.60	Arabidopsis thaliana (Mouse-ear cress)		Carotenoid biosynthesis; Chloroplast; Complete proteome; Heme; Iron; Membrane; Metal-binding; Monooxygenase; Oxidoreductase; Plastid; Reference proteome; Transit peptide
SRR122238_TR12177_c3_g1_i2_len=608	H32_WHEAT	Q:2-400,H:4-136	99.25%ID	E:3e-75	Histone H3.2	PF00125.21^Histone^Core histone H2A/H2B/H3/H4^1-129^E:3.5e-53	0	-1,579	0	-3,274	0,781	-0,345	0,781	-0,349	0	-0,939	H32_WHEAT	Histone H3.2		Triticum aestivum (Wheat)		Acetylation; Chromosome; Complete proteome; DNA-binding; Direct protein sequencing; Methylation; Nucleosome core; Nucleus; Phosphoprotein; Reference proteome
SRR1271857_TR9636_c0_g2_i1_len=800	CP20B_ARATH	Q:188-475,H:161-256	79.17%ID	E:1e-49	.	.	0,001	-0,772	0	-1,757	0,086	-0,87	0,044	-0,963	0	-2,47	PNSL5_ARATH	Photosynthetic NDH subunit of lumenal location 5, chloroplastic (Cyclophilin of 20 kDa 2) (Peptidyl-prolyl cis-trans isomerase CYP20-2) (PPIase CYP20-2) (EC 5.2.1.8) (Rotamase CYP20-2) (Thylakoid lumen PPIase of 20 kDa) (TLP20)	PNSL5 CYP20-2 At5g13120 T19L5_80	Arabidopsis thaliana (Mouse-ear cress)		Alternative splicing; Chaperone; Chloroplast; Complete proteome; Cyclosporin; Direct protein sequencing; Disulfide bond; Isomerase; Membrane; Plastid; Reference proteome; Rotamase; Thylakoid; Transit peptide; Transport
SRR1271858_TR7760_c0_g2_i1_len=563	H4_SOYBN	Q:438-193,H:22-103	100%ID	E:1e-49	Histone H4	PF15511.3^CENP-T_C^Centromere kinetochore component CENP-T histone fold^35-96^E:1.4e-08`PF02969.14^TAF^TATA box binding protein associated factor (TAF)^35-92^E:0.00016	0	-1,301	0	-1,657	0,201	0,413	0,782	0,095	0	-0,862	H4_SOYBN	Histone H4		Glycine max (Soybean) (Glycine hispida)		Acetylation; Chromosome; Complete proteome; DNA-binding; Methylation; Nucleosome core; Nucleus; Reference proteome
SRR342017_TR1512_c0_g3_i1_len=960	CH10C_ARATH	Q:6-674,H:31-253	77.58%ID	E:7e-119	20 kDa chaperonin, chloroplastic	PF00166.18^Cpn10^Chaperonin 10 Kd subunit^33-122^E:5.6e-28`PF00166.18^Cpn10^Chaperonin 10 Kd subunit^131-222^E:3.9e-28	0	-3,334	0	-2,451	0	4,284	0,004	3,434	0	-0,897	CH10C_ARATH	20 kDa chaperonin, chloroplastic (Chaperonin 10) (Ch-CPN10) (Cpn10) (Chaperonin 20) (Protein Cpn21)	CPN21 CHCPN10 CPN20 At5g20720 T1M15.120	Arabidopsis thaliana (Mouse-ear cress)		Chaperone; Chloroplast; Complete proteome; Phosphoprotein; Plastid; Reference proteome; Repeat; Transit peptide
SRR342017_TR7679_c0_g1_i1_len=1564	ASPG2_ARATH	Q:365-1384,H:128-470	32.66%ID	E:1e-32	Protein ASPARTIC PROTEASE IN GUARD CELL 2	PF14543.3^TAXi_N^Xylanase inhibitor N-terminal^125-286^E:2e-33`PF00026.20^Asp^Eukaryotic aspartyl protease^125-458^E:6.7e-19`PF14541.3^TAXi_C^Xylanase inhibitor C-terminal^309-455^E:4.6e-33	0	-0,911	0	-1,38	0,52	-0,389	0,52	-0,46	0,001	-0,922	ASPG2_ARATH	Protein ASPARTIC PROTEASE IN GUARD CELL 2 (AtASPG2) (EC 3.4.23.-)	ASPG2 At3g20015 MZE19.7	Arabidopsis thaliana (Mouse-ear cress)		Aspartyl protease; Complete proteome; DNA-binding; Disulfide bond; Hydrolase; Protease; Reference proteome; Signal
SRR342017_TR24942_c1_g1_i1_len=518	H4_SOYBN	Q:351-106,H:22-103	100%ID	E:7e-50	Histone H4	PF15511.3^CENP-T_C^Centromere kinetochore component CENP-T histone fold^35-96^E:1.4e-08`PF02969.14^TAF^TATA box binding protein associated factor (TAF)^35-92^E:0.00016	0,001	-0,844	0	-1,484	0,539	0,239	1	-0,015	0	-0,961	H4_SOYBN	Histone H4		Glycine max (Soybean) (Glycine hispida)		Acetylation; Chromosome; Complete proteome; DNA-binding; Methylation; Nucleosome core; Nucleus; Reference proteome
SRR342023_TR3286_c0_g2_i1_len=712	LSM5_ARATH	Q:602-345,H:1-85	93.02%ID	E:5e-49	Sm-like protein LSM5 {ECO:0000305}	PF01423.19^LSM^LSM domain^49-117^E:3.9e-20	0	-7,066	0	-1,357	0,839	0,136	0,439	0,33	0	-8,488	LSM5_ARATH	Sm-like protein LSM5 (AtLSM5) (Protein SUPERSENSITIVE TO ABA AND DROUGHT 1) (AtSAD1) (U6 snRNA-associated Sm-like protein LSM5)	LSM5 SAD1 At5g48870 K24G6.21	Arabidopsis thaliana (Mouse-ear cress)		Acetylation; Complete proteome; Cytoplasm; Nucleus; RNA-binding; Reference proteome; Ribonucleoprotein; Spliceosome; Stress response; mRNA processing; mRNA splicing
SRR646596_TR9498_c0_g1_i1_len=670	H32_WHEAT	Q:130-537,H:1-136	99.26%ID	E:1e-77	Histone H3.2	PF00125.21^Histone^Core histone H2A/H2B/H3/H4^1-132^E:8.3e-55	0	-1,697	0	-5,152	0,524	-0,603	0,381	-0,812	0	-1,06	H32_WHEAT	Histone H3.2		Triticum aestivum (Wheat)		Acetylation; Chromosome; Complete proteome; DNA-binding; Direct protein sequencing; Methylation; Nucleosome core; Nucleus; Phosphoprotein; Reference proteome
SRR648707_TR24165_c0_g1_i1_len=857	H4_SOYBN	Q:151-396,H:22-103	100%ID	E:2e-48	Histone H4	.	0	-1,237	0	-1,212	0,099	0,623	0,474	0,293	0	-1,859	H4_SOYBN	Histone H4		Glycine max (Soybean) (Glycine hispida)		Acetylation; Chromosome; Complete proteome; DNA-binding; Methylation; Nucleosome core; Nucleus; Reference proteome
SRR924147_TR27514_c1_g1_i2_len=734	H4_SOYBN	Q:246-479,H:22-99	100%ID	E:6e-47	Histone H4	PF15511.3^CENP-T_C^Centromere kinetochore component CENP-T histone fold^35-96^E:1.4e-08`PF02969.14^TAF^TATA box binding protein associated factor (TAF)^35-92^E:0.00016	0	-1,264	0	-2,604	1	-0,402	1	-0,104	0	-1,131	H4_SOYBN	Histone H4		Glycine max (Soybean) (Glycine hispida)		Acetylation; Chromosome; Complete proteome; DNA-binding; Methylation; Nucleosome core; Nucleus; Reference proteome
SRR924148_TR21098_c0_g1_i2_len=1165	RSI1_SOLLC	Q:249-536,H:1-96	81.25%ID	E:2e-44	Protein RSI-1	.	0	-1,239	0	-3,589	1	0,043	0,84	-0,243	0	-1,51	RSI1_SOLLC	Protein RSI-1 (TR132)	RSI-1	Solanum lycopersicum (Tomato) (Lycopersicon esculentum)		Complete proteome; Disulfide bond; Reference proteome; Secreted; Signal
SRR924148_TR23429_c0_g1_i1_len=1029	NA	NA	NA	NA	NA	.	0	-1,604	0	-4,517	1	0,036	0,732	-0,873	0	-1,105	.	.	.	.	.	.
SRR342023_TR11828_c0_g1_i2_len=558	NA	NA	NA	NA	NA	.	0	-7,281	1	-1,066	0,286	-0,835	0	-6,739	0,299	1,788	.	.	.	.	.	.
SRR1144634_TR34776_c0_g2_i3_len=2152	POLX_TOBAC	Q:2144-1356,H:1065-1325	53.23%ID	E:1e-75	Retrovirus-related Pol polyprotein from transposon TNT 1-94	.	0,113	0,447	0	-2,413	0,051	-0,646	0	-1,581	0,603	0,238	POLX_TOBAC	Retrovirus-related Pol polyprotein from transposon TNT 1-94 [Includes: Protease (EC 3.4.23.-); Reverse transcriptase (EC 2.7.7.49); Endonuclease]		Nicotiana tabacum (Common tobacco)		Aspartyl protease; Endonuclease; Hydrolase; Metal-binding; Nuclease; Nucleotidyltransferase; Protease; RNA-directed DNA polymerase; Transferase; Transposable element; Zinc; Zinc-finger
SRR122238_TR9506_c0_g1_i1_len=641	PSAK_ARATH	Q:556-155,H:1-130	80.6%ID	E:2e-51	Photosystem I reaction center subunit psaK, chloroplastic	PF01241.15^PSI_PSAK^Photosystem I psaG / psaK^57-132^E:2.4e-24	0,917	0,023	0,001	-0,729	0,002	-0,652	0	-0,75	0,722	0,08	PSAK_ARATH	Photosystem I reaction center subunit psaK, chloroplastic (PSI-K) (Photosystem I subunit X)	PSAK At1g30380 T4K22.2	Arabidopsis thaliana (Mouse-ear cress)		Chloroplast; Complete proteome; Membrane; Photosynthesis; Photosystem I; Plastid; Reference proteome; Thylakoid; Transit peptide; Transmembrane; Transmembrane helix
SRR122238_TR34725_c0_g1_i1_len=1085	SABP2_TOBAC	Q:981-223,H:5-259	56.08%ID	E:6e-97	Salicylic acid-binding protein 2	PF00561.17^Abhydrolase_1^alpha/beta hydrolase fold^36-142^E:5.9e-10`PF12146.5^Hydrolase_4^Serine aminopeptidase, S33^36-270^E:2.4e-09`PF12697.4^Abhydrolase_6^Alpha/beta hydrolase family^38-276^E:3.8e-15	0,252	0,25	0	-0,987	0,003	-0,689	0	-2,387	0,826	-0,051	SABP2_TOBAC	Salicylic acid-binding protein 2 (NtSABP2) (EC 3.1.1.-) (Methyl salicylate esterase)	SABP2	Nicotiana tabacum (Common tobacco)		3D-structure; Hydrolase; Immunity; Innate immunity; Plant defense
SRR122238_TR73778_c0_g1_i1_len=1257	NA	NA	NA	NA	NA	.	0,539	0,176	0,001	-0,993	0,002	-0,877	0,001	-0,933	0,001	1,12	.	.	.	.	.	.
SRR122238_TR85664_c0_g1_i2_len=611	NA	NA	NA	NA	NA	.	1	0	0	-8,08	1	-0,025	0	-9,042	1	-1,134	.	.	.	.	.	.
SRR1271857_TR37022_c0_g2_i2_len=1129	NA	NA	NA	NA	NA	.	0,235	-1	0	-2,671	0,003	-2,301	0,001	-2,456	1	-0,129	.	.	.	.	.	.
SRR1271857_TR43256_c0_g2_i3_len=857	PSAL_SPIOL	Q:682-191,H:53-216	88.41%ID	E:6e-79	Photosystem I reaction center subunit XI, chloroplastic	.	0,454	0,348	0	-1,822	0,003	-1,222	0	-2,1	1	-0,045	PSAL_SPIOL	Photosystem I reaction center subunit XI, chloroplastic (PSI-L) (PSI subunit V)	PSAL	Spinacia oleracea (Spinach)		3D-structure; Chloroplast; Direct protein sequencing; Membrane; Photosynthesis; Photosystem I; Plastid; Thylakoid; Transit peptide; Transmembrane; Transmembrane helix
SRR1271858_TR44225_c0_g2_i1_len=659	NLTP_VIGUN	Q:507-265,H:20-99	57.32%ID	E:4e-23	Probable non-specific lipid-transfer protein AKCS9	.	0,15	-0,528	0	-1,224	0,023	0,822	0	-8,349	0	2,598	NLTP_VIGUN	Probable non-specific lipid-transfer protein AKCS9 (LTP)		Vigna unguiculata (Cowpea)		Disulfide bond; Lipid-binding; Signal; Transport
SRR342017_TR26374_c1_g2_i2_len=997	NA	NA	NA	NA	NA	.	0,005	-1,656	0	-4,982	0,004	-1,846	0	-3,502	0,229	-0,525	.	.	.	.	.	.
SRR342022_TR15102_c0_g1_i1_len=1095	NA	NA	NA	NA	NA	.	0,007	-0,837	0,001	-1,529	0,003	-1,291	0	-1,697	0,061	-0,648	.	.	.	.	.	.
SRR342023_TR1771_c4_g6_i1_len=894	NA	NA	NA	NA	NA	.	0,931	-0,071	0	-2,312	0,001	-1,576	0	-1,848	1	-0,649	.	.	.	.	.	.
SRR342023_TR9971_c4_g6_i8_len=1569	EXOL5_ARATH	Q:1487-558,H:54-363	76.04%ID	E:2e-157	Protein EXORDIUM-like 5	PF04674.9^Phi_1^Phosphate-induced protein 1 conserved region^51-332^E:8.4e-105	0,073	-0,634	0	-1,748	0,01	-1,274	0,001	-1,817	1	0,253	EXOL5_ARATH	Protein EXORDIUM-like 5	EXL5 At2g17230 T23A1.9	Arabidopsis thaliana (Mouse-ear cress)		Apoplast; Complete proteome; Glycoprotein; Reference proteome; Secreted; Signal
SRR646604_TR11901_c0_g1_i4_len=2137	PSY_CAPAN	Q:481-1758,H:1-419	79.34%ID	E:0	Bifunctional 15-cis-phytoene synthase, chromoplastic {ECO:0000303|PubMed:16578835}	PF00494.16^SQS_PSY^Squalene/phytoene synthase^150-405^E:5.1e-75	0,656	-0,156	0	-1,63	0,15	-0,385	0	-1,028	0,189	1,58	PSY_CAPAN	Bifunctional 15-cis-phytoene synthase, chromoplastic (EC 2.5.1.32) (Geranylgeranyl pyrophosphate geranylgeranyltransferase) (Phytoene synthase)	PSY1	Capsicum annuum (Bell pepper)	PATHWAY: Carotenoid biosynthesis; phytoene biosynthesis; all-trans-phytoene from geranylgeranyl diphosphate: step 1/1. {ECO:0000305}.	Carotenoid biosynthesis; Chromoplast; Isoprene biosynthesis; Multifunctional enzyme; Plastid; Transferase; Transit peptide
SRR646604_TR15068_c0_g1_i2_len=899	NA	NA	NA	NA	NA	PF03080.12^DUF239^Domain of unknown function (DUF239)^16-201^E:1.1e-73	1	0,009	0	-2,037	0,002	-1,303	0	-2,947	0,563	0,27	.	.	.	.	.	.
SRR646604_TR17949_c0_g1_i2_len=1275	CB4B_ARATH	Q:979-626,H:167-284	93.22%ID	E:2e-69	Chlorophyll a-b binding protein CP29.2, chloroplastic	.	0,77	0,149	0	-2,831	0,008	-1,035	0	-1,876	0,003	-0,652	CB4B_ARATH	Chlorophyll a-b binding protein CP29.2, chloroplastic (LHCB4.2) (LHCII protein 4.2)	LHCB4.2 At3g08940 T16O11.12	Arabidopsis thaliana (Mouse-ear cress)		Acetylation; Alternative splicing; Chlorophyll; Chloroplast; Chromophore; Complete proteome; Direct protein sequencing; Magnesium; Membrane; Metal-binding; Phosphoprotein; Photosynthesis; Photosystem I; Photosystem II; Plastid; Reference proteome; Thylakoid; Transit peptide; Transmembrane; Transmembrane helix
SRR648705_TR11177_c0_g2_i1_len=2099	NEP1_NEPGR	Q:1250-225,H:93-434	33.62%ID	E:6e-39	Aspartic proteinase nepenthesin-1	PF14543.3^TAXi_N^Xylanase inhibitor N-terminal^99-262^E:6.6e-34`PF00026.20^Asp^Eukaryotic aspartyl protease^99-434^E:3e-11`PF14541.3^TAXi_C^Xylanase inhibitor C-terminal^285-434^E:1.8e-32	0,647	-0,138	0	-3,536	0,002	-1,614	0	-2,065	0,875	0,041	NEP1_NEPGR	Aspartic proteinase nepenthesin-1 (EC 3.4.23.12) (Nepenthesin-I)	nep1	Nepenthes gracilis (Slender pitcher plant)		Aspartyl protease; Disulfide bond; Glycoprotein; Hydrolase; Polymorphism; Protease; Secreted; Signal; Zymogen
SRR648707_TR18946_c0_g1_i1_len=978	NA	NA	NA	NA	NA	PF00026.20^Asp^Eukaryotic aspartyl protease^14-176^E:1.4e-13`PF14541.3^TAXi_C^Xylanase inhibitor C-terminal^17-176^E:3.2e-40	0,502	-0,255	0	-2,275	0,002	-1,776	0,001	-1,913	0,656	0,197	.	.	.	.	.	.

SRR648707_TR22461_c0_g1_i2_len=1674	DXS2_ORYSJ	Q:102-1394,H:283-713	82.6%ID	E:0	Probable 1-deoxy-D-xylulose-5-phosphate synthase 2, chloroplastic	PF13292.3^DXP_synthase_N^1-deoxy-D-xylulose-5-phosphate synthase^6-51^E:6.9e-11`PF02779.21^Transket_pyr^Transketolase, pyrimidine binding domain^88-249^E:2.8e-43`PF02780.17^Transketolase_C^Transketolase, C-terminal domain^267-390^E:2.2e-31	0,107	0,576	0,001	-1,757	0,326	-0,556	0	-3,052	0,257	-0,308	DXS2_ORYSJ	Probable 1-deoxy-D-xylulose-5-phosphate synthase 2, chloroplastic (1-deoxyxylulose-5-phosphate synthase) (DXP synthase) (DXPS) (EC 2.2.1.7)	Os07g0190000 LOC_Os07g09190 OsJ_23394 OSJNBb0002L09.31 OSJNBb0003E08.7	Oryza sativa subsp. japonica (Rice)	PATHWAY: Metabolic intermediate biosynthesis; 1-deoxy-D-xylulose 5-phosphate biosynthesis; 1-deoxy-D-xylulose 5-phosphate from D-glyceraldehyde 3-phosphate and pyruvate: step 1/1.	Chloroplast; Complete proteome; Isoprene biosynthesis; Magnesium; Metal-binding; Plastid; Reference proteome; Thiamine biosynthesis; Thiamine pyrophosphate; Transferase; Transit peptide
SRR924147_TR21947_c2_g1_i1_len=1264	MTDH_APIGR	Q:1262-243,H:17-359	57.73%ID	E:4e-130	Mannitol dehydrogenase	PF08240.9^ADH_N^Alcohol dehydrogenase GroES-like domain^22-131^E:3e-23`PF02826.16^2-Hacid_dh_C^D-isomer specific 2-hydroxyacid dehydrogenase, NAD binding domain^162-212^E:2.8e-06`PF00107.23^ADH_zinc_N^Zinc-binding dehydrogenase^175-298^E:5e-14	0,48	-0,153	0	-1,666	0,006	-0,617	0	-0,912	0,021	-0,922	MTDH_APIGR	Mannitol dehydrogenase (EC 1.1.1.255) (NAD-dependent mannitol dehydrogenase)	MTD	Apium graveolens (Celery)		Cytoplasm; Metal-binding; NAD; Oxidoreductase; Zinc
SRR342017_TR10068_c0_g1_i1_len=877	R10A1_ARATH	Q:235-876,H:1-214	90.19%ID	E:5e-126	60S ribosomal protein L10a-1	PF00687.18^Ribosomal_L1^Ribosomal protein L1p/L10e family^23-210^E:5.6e-44	0	-8,353	1	3,301	0	-6,845	0,854	-0,25	0,502	-4,155	R10A1_ARATH	60S ribosomal protein L10a-1	RPL10AA At1g08360 T27G7.6	Arabidopsis thaliana (Mouse-ear cress)		Complete proteome; Reference proteome; Ribonucleoprotein; Ribosomal protein
SRR342017_TR11303_c0_g1_i1_len=1129	NA	NA	NA	NA	NA	.	0,001	-0,896	0,681	-0,165	0,001	-0,983	0,026	-0,631	0,001	1,294	.	.	.	.	.	.
SRR342023_TR12969_c4_g2_i2_len=506	G3PA_TOBAC	Q:200-505,H:9-109	86.27%ID	E:3e-45	Glyceraldehyde-3-phosphate dehydrogenase A, chloroplastic	PF00044.21^Gp_dh_N^Glyceraldehyde 3-phosphate dehydrogenase, NAD binding domain^69-119^E:1.9e-13	0	-1,22	0,08	0,627	0	-1,45	0,026	-0,505	0,009	0,557	G3PA_TOBAC	Glyceraldehyde-3-phosphate dehydrogenase A, chloroplastic (EC 1.2.1.13) (NADP-dependent glyceraldehydephosphate dehydrogenase subunit A) (Fragment)	GAPA	Nicotiana tabacum (Common tobacco)	PATHWAY: Carbohydrate biosynthesis; Calvin cycle.	Calvin cycle; Chloroplast; NADP; Oxidoreductase; Plastid; Transit peptide
SRR646572_TR8074_c0_g1_i1_len=820	NA	NA	NA	NA	NA	.	0	-2,078	0,704	0,161	0,001	-1,145	0,328	-0,299	0,642	0,222	.	.	.	.	.	.
SRR122236_TR9961_c0_g2_i2_len=816	CB23_TOBAC	Q:319-627,H:163-265	99.03%ID	E:5e-64	Chlorophyll a-b binding protein 36, chloroplastic	.	0,053	-0,49	0	-1,301	0	-1,127	0,01	-0,673	0,001	0,681	CB23_TOBAC	Chlorophyll a-b binding protein 36, chloroplastic (LHCII type I CAB-36) (LHCP)	CAB36	Nicotiana tabacum (Common tobacco)		Acetylation; Chlorophyll; Chloroplast; Chromophore; Magnesium; Membrane; Metal-binding; Phosphoprotein; Photosynthesis; Photosystem I; Photosystem II; Plastid; Thylakoid; Transit peptide; Transmembrane; Transmembrane helix
SRR122238_TR54680_c0_g1_i2_len=1203	BAS1_ORYSJ	Q:815-228,H:66-261	95.41%ID	E:1e-133	2-Cys peroxiredoxin BAS1, chloroplastic	PF00578.18^AhpC-TSA^AhpC/TSA family^78-211^E:5e-41`PF08534.7^Redoxin^Redoxin^78-213^E:7.1e-15`PF10417.6^1-cysPrx_C^C-terminal domain of 1-Cys peroxiredoxin^232-267^E:5e-11	0,021	-0,503	0	-0,75	0	-0,762	0,014	-0,535	0,062	-0,458	BAS1_ORYSJ	2-Cys peroxiredoxin BAS1, chloroplastic (EC 1.11.1.15) (Thiol-specific antioxidant protein)	BAS1 Os02g0537700 LOC_Os02g33450 OSJNBa0014M17.2 P0508B05.23	Oryza sativa subsp. japonica (Rice)		Antioxidant; Chloroplast; Complete proteome; Direct protein sequencing; Disulfide bond; Hydrogen peroxide; Oxidoreductase; Peroxidase; Plastid; Redox-active center; Reference proteome; Transit peptide
SRR122238_TR55852_c0_g1_i2_len=930	RBS_MALSP	Q:851-312,H:1-183	81.42%ID	E:2e-107	Ribulose bisphosphate carboxylase small chain, chloroplastic	PF12338.5^RbcS^Ribulose-1,5-bisphosphate carboxylase small subunit^2-42^E:6.5e-21`PF00101.17^RuBisCO_small^Ribulose bisphosphate carboxylase, small chain^69-177^E:1.2e-39	0,097	-0,427	0	-1,073	0	-0,821	0,007	-0,612	0,551	-0,154	RBS_MALSP	Ribulose bisphosphate carboxylase small chain, chloroplastic (RuBisCO small subunit) (EC 4.1.1.39)	RBCS	Malus sp. (Crab apple)		Calvin cycle; Carbon dioxide fixation; Chloroplast; Lyase; Monooxygenase; Oxidoreductase; Photorespiration; Photosynthesis; Plastid; Transit peptide
SRR122238_TR76493_c0_g1_i2_len=816	PSAN_ARATH	Q:163-666,H:1-168	69.19%ID	E:9e-61	Photosystem I reaction center subunit N, chloroplastic	PF05479.8^PsaN^Photosystem I reaction centre subunit N (PSAN or PSI-N)^97-225^E:6.7e-68	0,796	-0,055	0	-0,999	0	-0,725	0,005	-0,587	0,981	0,006	PSAN_ARATH	Photosystem I reaction center subunit N, chloroplastic (PSI-N)	PSAN At5g64040 MHJ24.2	Arabidopsis thaliana (Mouse-ear cress)		Acetylation; Alternative splicing; Chloroplast; Complete proteome; Direct protein sequencing; Membrane; Photosynthesis; Photosystem I; Plastid; Reference proteome; Thylakoid; Transit peptide
SRR1271857_TR634_c0_g1_i3_len=2761	CP41B_ARATH	Q:1417-2550,H:1-378	85.19%ID	E:0	Chloroplast stem-loop binding protein of 41 kDa b, chloroplastic	PF04321.14^RmlD_sub_bind^RmlD substrate binding domain^55-299^E:9e-05`PF01370.18^Epimerase^NAD dependent epimerase/dehydratase family^56-273^E:1.2e-13`PF13460.3^NAD_binding_10^NAD(P)H-binding^60-212^E:9.6e-09	0,252	-0,299	0	-0,93	0	-0,828	0,01	-0,61	0,324	-0,239	CP41B_ARATH	Chloroplast stem-loop binding protein of 41 kDa b, chloroplastic (CSP41-b) (Heteroglycan-interacting protein 1.3) (Protein CHLOROPLAST RNA BINDING) (Protein Gb5f)	CSP41B CRB HIP1.3 At1g09340 T31J12.6	Arabidopsis thaliana (Mouse-ear cress)		Carbohydrate metabolism; Chloroplast; Complete proteome; Cytoplasm; Phosphoprotein; Plastid; Polysaccharide degradation; RNA-binding; Reference proteome; Transit peptide; rRNA processing; rRNA-binding
SRR1271857_TR8897_c0_g1_i2_len=905	LOG1_ARATH	Q:207-602,H:80-211	90.15%ID	E:3e-80	Cytokinin riboside 5'-monophosphate phosphoribohydrolase LOG1	PF03641.11^Lysine_decarbox^Possible lysine decarboxylase^1-90^E:5.6e-35	0,13	-0,642	0	-3,578	0,001	-1,722	0,003	-1,416	0,62	-0,259	LOG1_ARATH	Cytokinin riboside 5'-monophosphate phosphoribohydrolase LOG1 (EC 3.2.2.n1) (Protein LONELY GUY 1)	LOG1 At2g28305 T1B3.18 T3B23.2	Arabidopsis thaliana (Mouse-ear cress)		Complete proteome; Cytokinin biosynthesis; Cytoplasm; Hydrolase; Nucleus; Reference proteome
SRR1271857_TR18439_c1_g1_i3_len=1505	NA	NA	NA	NA	NA	PF14215.3^bHLH-MYC_N^bHLH-MYC and R2R3-MYB transcription factors N-terminal^13-198^E:5.2e-27	0,24	-0,408	0	-3,185	0,001	-1,687	0,013	-1,224	1	-0,043	.	.	.	.	.	.
SRR1271857_TR20316_c0_g1_i2_len=1988	PSBO_TOBAC	Q:949-188,H:79-332	91.34%ID	E:6e-164	Oxygen-evolving enhancer protein 1, chloroplastic	PF01716.15^MSP^Manganese-stabilising protein / photosystem II polypeptide^1-168^E:1.3e-76	0,923	-0,021	0	-1,165	0	-0,787	0,002	-0,642	0,672	0,091	PSBO_TOBAC	Oxygen-evolving enhancer protein 1, chloroplastic (OEE1) (33 kDa subunit of oxygen evolving system of photosystem II) (33 kDa thylakoid membrane protein) (OEC 33 kDa subunit)	PSBO	Nicotiana tabacum (Common tobacco)		Chloroplast; Manganese; Membrane; Photosynthesis; Photosystem II; Plastid; Thylakoid; Transit peptide
SRR1271857_TR25644_c0_g1_i1_len=1865	CHLI_SOYBN	Q:210-1475,H:1-421	85.82%ID	E:0	Magnesium-chelatase subunit ChlI, chloroplastic	PF01078.18^Mg_chelatase^Magnesium chelatase, subunit ChlI^198-268^E:2.9e-07`PF00493.20^MCM^MCM2/3/5 family^201-388^E:4.4e-06	0,023	-0,529	0	-1,046	0	-0,936	0,004	-0,664	0,82	0,055	CHLI_SOYBN	Magnesium-chelatase subunit ChlI, chloroplastic (Mg-chelatase subunit I-1) (EC 6.6.1.1) (Mg-protoporphyrin IX chelatase subunit ChlI)	CHLI	Glycine max (Soybean) (Glycine hispida)	PATHWAY: Porphyrin-containing compound metabolism; chlorophyll biosynthesis.	ATP-binding; Chlorophyll biosynthesis; Chloroplast; Complete proteome; Disulfide bond; Ligase; Nucleotide-binding; Photosynthesis; Plastid; Reference proteome; Transit peptide
SRR1271857_TR30200_c1_g3_i3_len=1157	CAHC_SPIOL	Q:1074-547,H:144-319	89.2%ID	E:5e-110	Carbonic anhydrase, chloroplastic	PF00484.16^Pro_CA^Carbonic anhydrase^1-158^E:5.6e-40	1	0,005	0	-3,577	0	-1,996	0,095	-0,741	0,118	-1,842	CAHC_SPIOL	Carbonic anhydrase, chloroplastic (EC 4.2.1.1) (Carbonate dehydratase)		Spinacia oleracea (Spinach)		Chloroplast; Direct protein sequencing; Lyase; Plastid; Transit peptide; Zinc
SRR1271858_TR28861_c0_g1_i1_len=1089	APT3_ARATH	Q:896-372,H:7-181	82.86%ID	E:1e-99	Adenine phosphoribosyltransferase 3	PF00156.24^Pribosyltran^Phosphoribosyl transferase domain^44-167^E:6e-20	0,746	-0,11	0	-2,138	0	-2,322	0,002	-1,38	0,444	0,239	APT3_ARATH	Adenine phosphoribosyltransferase 3 (EC 2.4.2.7)	APT3 At4g22570 F7K2.150	Arabidopsis thaliana (Mouse-ear cress)	PATHWAY: Purine metabolism; AMP biosynthesis via salvage pathway; AMP from adenine: step 1/1.	Complete proteome; Cytoplasm; Glycosyltransferase; Purine salvage; Reference proteome; Transferase
SRR342017_TR8859_c1_g4_i3_len=1797	MTDH_APIGR	Q:1174-203,H:33-359	57.49%ID	E:2e-118	Mannitol dehydrogenase	PF08240.9^ADH_N^Alcohol dehydrogenase GroES-like domain^5-92^E:2.8e-17`PF02826.16^2-Hacid_dh_C^D-isomer specific 2-hydroxyacid dehydrogenase, NAD binding domain^123-173^E:2.3e-06`PF00107.23^ADH_zinc_N^Zinc-binding dehydrogenase^136-259^E:3.8e-14	0,294	0,523	0	-2,758	0,001	-1,793	0,004	-1,403	0,147	-0,938	MTDH_APIGR	Mannitol dehydrogenase (EC 1.1.1.255) (NAD-dependent mannitol dehydrogenase)	MTD	Apium graveolens (Celery)		Cytoplasm; Metal-binding; NAD; Oxidoreductase; Zinc
SRR342017_TR8869_c0_g2_i1_len=978	NDADA_ARATH	Q:779-351,H:1-143	87.41%ID	E:1e-86	NADH dehydrogenase [ubiquinone] 1 alpha subcomplex subunit 13-A	PF06212.9^GRIM-19^GRIM-19 protein^20-138^E:2.2e-42	0,43	0,688	0	-2,208	0	-2,772	0,019	-1,271	1	0	NDADA_ARATH	NADH dehydrogenase [ubiquinone] 1 alpha subcomplex subunit 13-A (Protein MATERNAL EFFECT EMBRYO ARREST 4)	MEE4 At1g04630 T1G11.12	Arabidopsis thaliana (Mouse-ear cress)		Complete proteome; Electron transport; Membrane; Mitochondrion; Mitochondrion inner membrane; Reference proteome; Respiratory chain; Transmembrane; Transmembrane helix; Transport
SRR342017_TR10762_c4_g2_i5_len=3443	LACS2_ARATH	Q:91-2145,H:3-661	64.97%ID	E:0	Long chain acyl-CoA synthetase 2	PF00501.25^AMP-binding^AMP-binding enzyme^52-521^E:3.3e-99	0,451	-0,31	0,001	-1,954	0	-1,742	0,007	-1,141	0,095	0,729	LACS2_ARATH	Long chain acyl-CoA synthetase 2 (EC 6.2.1.3) (Protein Botrytis resistant 1) (Protein LATERAL ROOT DEVELOPMENT 2)	LACS2 BRE1 LRD2 SMA4 At1g49430 F13F21.14	Arabidopsis thaliana (Mouse-ear cress)	PATHWAY: Lipid metabolism; fatty acid metabolism.	ATP-binding; Complete proteome; Endoplasmic reticulum; Fatty acid metabolism; Ligase; Lipid metabolism; Magnesium; Nucleotide-binding; Reference proteome
SRR342017_TR27230_c0_g2_i1_len=846	NA	NA	NA	NA	NA	.	0,816	-0,109	0	-3,51	0,001	-2,094	0,021	-1,242	0,258	-0,499	.	.	.	.	.	.
SRR342017_TR32028_c2_g3_i3_len=2023	HEM3_PEA	Q:651-1649,H:40-369	83.53%ID	E:0	Porphobilinogen deaminase, chloroplastic	PF01379.17^Porphobil_deam^Porphobilinogen deaminase, dipyromethane cofactor binding domain^73-284^E:1.5e-72`PF03900.12^Porphobil_deamC^Porphobilinogen deaminase, C-terminal domain^298-370^E:3.6e-13	0,005	-0,725	0	-1,189	0,001	-1,151	0,024	-0,733	0,666	-0,113	HEM3_PEA	Porphobilinogen deaminase, chloroplastic (PBG) (EC 2.5.1.61) (Hydroxymethylbilane synthase) (HMBS) (Pre-uroporphyrinogen synthase)	HEMC	Pisum sativum (Garden pea)	PATHWAY: Porphyrin-containing compound metabolism; protoporphyrin-IX biosynthesis; coproporphyrinogen-III from 5-aminolevulinate: step 2/4.; PATHWAY: Porphyrin-containing compound metabolism; chlorophyll biosynthesis.	Chlorophyll biosynthesis; Chloroplast; Direct protein sequencing; Plastid; Porphyrin biosynthesis; Transferase; Transit peptide
SRR342019_TR17507_c0_g10_i1_len=1488	NA	NA	NA	NA	NA	.	0,395	-0,306	0	-2,395	0	-1,889	0,002	-1,71	0,004	-1,214	.	.	.	.	.	.
SRR342019_TR19760_c0_g1_i1_len=1781	DCVR_CUCSA	Q:211-1455,H:1-419	75.18%ID	E:0	Divinyl chlorophyllide a 8-vinyl-reductase, chloroplastic	PF05368.10^NmrA^NmrA-like family^84-323^E:4.3e-13`PF01370.18^Epimerase^NAD dependent epimerase/dehydratase family^84-205^E:1e-08`PF01073.16^3Beta_HSD^3-beta hydroxysteroid dehydrogenase/isomerase family^85-207^E:8e-06`PF13460.3^NAD_binding_10^NAD(P)H-binding^88-290^E:4.6e-19	0,158	-0,458	0	-1,203	0	-1,082	0,003	-0,81	0,337	0,28	DCVR_CUCSA	Divinyl chlorophyllide a 8-vinyl-reductase, chloroplastic (EC 1.3.1.75)	DVR	Cucumis sativus (Cucumber)	PATHWAY: Porphyrin-containing compound metabolism; chlorophyll biosynthesis.	Chlorophyll biosynthesis; Chloroplast; NADP; Oxidoreductase; Plastid; Transit peptide
SRR342019_TR21621_c2_g1_i1_len=1884	Y4744_ARATH	Q:1447-401,H:43-387	42.13%ID	E:4e-86	Uncharacterized protein At4g06744	PF13516.3^LRR_6^Leucine Rich repeat^279-293^E:1.2	0,068	-0,436	0	-2,041	0	-1,178	0,003	-0,927	0,012	-0,621	Y4744_ARATH	Uncharacterized protein At4g06744	At4g06744 F15N16	Arabidopsis thaliana (Mouse-ear cress)		Complete proteome; Leucine-rich repeat; Reference proteome; Repeat; Secreted; Signal
SRR342019_TR30363_c1_g4_i2_len=3294	HEM6_TOBAC	Q:1378-164,H:1-397	76.85%ID	E:0	Oxygen-dependent coproporphyrinogen-III oxidase, chloroplastic	.	0,033	-0,641	0	-1,164	0	-1,102	0,003	-0,889	0,377	-0,29	HEM6_TOBAC	Oxygen-dependent coproporphyrinogen-III oxidase, chloroplastic (Coprogen oxidase) (Coproporphyrinogenase) (EC 1.3.3.3)	CPX	Nicotiana tabacum (Common tobacco)	PATHWAY: Porphyrin-containing compound metabolism; protoporphyrin-IX biosynthesis; protoporphyrinogen-IX from coproporphyrinogen-III (O2 route): step 1/1.	Chlorophyll biosynthesis; Chloroplast; Heme biosynthesis; Oxidoreductase; Plastid; Porphyrin biosynthesis; Transit peptide
SRR342019_TR38174_c0_g1_i6_len=904	RR13_ARATH	Q:163-615,H:4-154	74.17%ID	E:1e-71	30S ribosomal protein S13, chloroplastic	PF00416.19^Ribosomal_S13^Ribosomal protein S13/S18^45-149^E:2.8e-32	0,366	-0,21	0	-1,006	0	-0,824	0,003	-0,704	0,032	-0,472	RR13_ARATH	30S ribosomal protein S13, chloroplastic (CS13)	RPS13 At5g14320 F18O22_110	Arabidopsis thaliana (Mouse-ear cress)		Alternative splicing; Chloroplast; Complete proteome; Plastid; RNA-binding; Reference proteome; Ribonucleoprotein; Ribosomal protein; Transit peptide; rRNA-binding
SRR342022_TR25676_c0_g3_i1_len=2187	PLY18_ARATH	Q:1579-434,H:27-408	86.13%ID	E:0	Probable pectate lyase 18	PF00544.16^Pec_lyase_C^Pectate lyase^233-413^E:5.6e-20`PF13229.3^Beta_helix^Right handed beta helix region^249-367^E:2.2e-05	0,862	0,067	0	-1,839	0	-1,018	0,032	-0,586	0,226	2,285	PLY18_ARATH	Probable pectate lyase 18 (EC 4.2.2.2) (Pectate lyase A10)	At4g24780 F22K18.20	Arabidopsis thaliana (Mouse-ear cress)	PATHWAY: Glycan metabolism; pectin degradation; 2-dehydro-3-deoxy-D-gluconate from pectin: step 2/5.	Calcium; Complete proteome; Glycoprotein; Lyase; Metal-binding; Reference proteome; Signal
SRR342022_TR28271_c1_g1_i1_len=6054	CLU_ARATH	Q:2492-3781,H:788-1258	38.87%ID	E:3e-89	Clustered mitochondria protein	PF15044.3^CLU_N^Mitochondrial function, CLU-N-term^49-120^E:6.3e-08`PF12807.4^eIF3_p135^Translation initiation factor eIF3 subunit 135^726-867^E:5.6e-25`PF13424.3^TPR_12^Tetratricopeptide repeat^907-968^E:2.2e-08`PF13424.3^TPR_12^Tetratricopeptide repeat^937-1007^E:5.5e-13`PF13374.3^TPR_10^Tetratricopeptide repeat^937-978^E:9.3e-09`PF13181.3^TPR_8^Tetratricopeptide repeat^938-969^E:0.11`PF13424.3^TPR_12^Tetratricopeptide repeat^1021-1095^E:1e-10`PF13374.3^TPR_10^Tetratricopeptide repeat^1021-1060^E:1e-06	1	-0,128	0	-8,831	0	-0,906	0,006	-0,687	1	0	CLU_ARATH	Clustered mitochondria protein (Friendly mitochondria protein)	FMT CLU At3g52140 F4F15.250	Arabidopsis thaliana (Mouse-ear cress)		Alternative splicing; Complete proteome; Cytoplasm; Reference proteome; Repeat; TPR repeat
SRR342023_TR3519_c2_g1_i1_len=1064	PLAS_SOLLC	Q:219-695,H:11-170	76.54%ID	E:3e-67	Plastocyanin, chloroplastic	PF00127.17^Copper-bind^Copper binding proteins, plastocyanin/azurin family^73-170^E:4.2e-36	0,249	-0,244	0	-1,228	0	-0,788	0,007	-0,563	0	0,951	PLAS_SOLLC	Plastocyanin, chloroplastic	PETE	Solanum lycopersicum (Tomato) (Lycopersicon esculentum)		Chloroplast; Complete proteome; Copper; Electron transport; Membrane; Metal-binding; Plastid; Reference proteome; Thylakoid; Transit peptide; Transport
SRR342023_TR8761_c0_g3_i1_len=1004	NA	NA	NA	NA	NA	.	0,074	-0,942	0	-1,785	0	-3,297	0,004	-2,038	1	-0,064	.	.	.	.	.	.
SRR342023_TR18283_c3_g6_i1_len=860	NA	NA	NA	NA	NA	.	0,807	0,167	0	-1,723	0,001	-1,478	0,025	-0,893	0,848	0,333	.	.	.	.	.	.
SRR646596_TR18300_c0_g1_i1_len=1635	FENR1_TOBAC	Q:344-1348,H:29-362	89.85%ID	E:0	Ferredoxin--NADP reductase, leaf-type isozyme, chloroplastic	PF00970.21^FAD_binding_6^Oxidoreductase FAD-binding domain^118-204^E:0.00016`PF00175.18^NAD_binding_1^Oxidoreductase NAD-binding domain^218-333^E:8e-30	0,618	-0,114	0	-0,835	0	-0,777	0,006	-0,584	0,019	-0,502	FENR1_TOBAC	Ferredoxin--NADP reductase, leaf-type isozyme, chloroplastic (FNR) (EC 1.18.1.2)	PETH	Nicotiana tabacum (Common tobacco)	PATHWAY: Energy metabolism; photosynthesis.	Chloroplast; Electron transport; FAD; Flavoprotein; Membrane; NADP; Oxidoreductase; Photosynthesis; Plastid; Thylakoid; Transit peptide; Transport
SRR646604_TR2290_c0_g1_i1_len=1657	CP41A_ARATH	Q:1329-346,H:78-405	82.93%ID	E:0	Chloroplast stem-loop binding protein of 41 kDa a, chloroplastic	PF01370.18^Epimerase^NAD dependent epimerase/dehydratase family^88-298^E:5.9e-08`PF13460.3^NAD_binding_10^NAD(P)H-binding^88-236^E:2.4e-06	0,008	-1,343	0	-2,682	0	-1,471	0,005	-1,038	1	0,066	CP41A_ARATH	Chloroplast stem-loop binding protein of 41 kDa a, chloroplastic (CSP41-a)	CSP41A At3g63140 T20O10.240	Arabidopsis thaliana (Mouse-ear cress)		Chloroplast; Complete proteome; Phosphoprotein; Plastid; RNA-binding; Reference proteome; Transit peptide; rRNA processing; rRNA-binding
SRR646604_TR5735_c0_g1_i1_len=2479	PSAO_ARATH	Q:258-596,H:28-140	87.61%ID	E:4e-61	Photosystem I subunit O	.	0,832	-0,332	0	-2,81	0	-2,475	0,001	-2,035	0,581	0,395	PSAO_ARATH	Photosystem I subunit O (PSI-O)	PSAO At1g08380 T27G7	Arabidopsis thaliana (Mouse-ear cress)		Chloroplast; Complete proteome; Direct protein sequencing; Membrane; Photosynthesis; Photosystem I; Plastid; Reference proteome; Thylakoid; Transit peptide; Transmembrane; Transmembrane helix
SRR646604_TR5839_c0_g1_i12_len=3706	PHOT2_ARATH	Q:2929-335,H:64-915	74.89%ID	E:0	Phototropin-2	PF13426.4^PAS_9^PAS domain^176-272^E:4.1e-19`PF00989.22^PAS^PAS fold^182-267^E:1.5e-08`PF08447.9^PAS_3^PAS fold^183-266^E:8e-08`PF13426.4^PAS_9^PAS domain^446-538^E:2e-19`PF00989.22^PAS^PAS fold^449-538^E:2.2e-06`PF00069.22^Pkinase^Protein kinase domain^626-910^E:4e-60`PF07714.14^Pkinase_Tyr^Protein tyrosine kinase^628-774^E:2.8e-16`PF07714.14^Pkinase_Tyr^Protein tyrosine kinase^815-894^E:7.5e-06	0,341	-0,419	0	-2,464	0	-1,09	0,002	-0,818	0,24	0,398	PHOT2_ARATH	Phototropin-2 (EC 2.7.11.1) (Defective in chloroplast avoidance protein 1) (Non-phototropic hypocotyl 1-like protein 1) (AtKin7) (NPH1-like protein 1)	PHOT2 CAV1 KIN7 NPL1 At5g58140 K21L19.6	Arabidopsis thaliana (Mouse-ear cress)		3D-structure; ATP-binding; Alternative splicing; Cell membrane; Chromophore; Complete proteome; FMN; Flavoprotein; Kinase; Membrane; Nucleotide-binding; Phosphoprotein; Photoreceptor protein; Receptor; Reference proteome; Repeat; Sensory transduction; Serine/threonine-protein kinase; Transferase
SRR646604_TR16056_c0_g4_i3_len=1773	PX24B_DICDI	Q:855-1439,H:1-182	31.28%ID	E:2e-24	PXMP2/4 family protein 2	PF04117.9^Mpv17_PMP22^Mpv17 / PMP22 family^129-189^E:1.2e-15	0,33	-0,366	0	-2,718	0,001	-1,987	0,008	-1,451	0,23	0,464	PX24B_DICDI	PXMP2/4 family protein 2	DDB_G0278529	Dictyostelium discoideum (Slime mold)		Complete proteome; Membrane; Reference proteome; Transmembrane; Transmembrane helix
SRR646604_TR17949_c0_g1_i1_len=1768	CB4A_ARATH	Q:1441-626,H:14-287	86.5%ID	E:1e-161	Chlorophyll a-b binding protein CP29.1, chloroplastic	PF00504.18^Chloroa_b-bind^Chlorophyll A-B binding protein^60-256^E:1.5e-49	0,953	0,014	0	-1,479	0	-0,816	0,002	-0,652	0,274	0,253	CB4A_ARATH	Chlorophyll a-b binding protein CP29.1, chloroplastic (LHCB4.1) (LHCII protein 4.1)	LHCB4.1 At5g01530 F7A7_50	Arabidopsis thaliana (Mouse-ear cress)		Chlorophyll; Chloroplast; Chromophore; Complete proteome; Magnesium; Membrane; Metal-binding; Phosphoprotein; Photosynthesis; Photosystem I; Photosystem II; Plastid; Reference proteome; Thylakoid; Transit peptide; Transmembrane; Transmembrane helix
SRR648707_TR19129_c1_g1_i1_len=1022	RBS_MALSP	Q:795-256,H:1-183	81.42%ID	E:5e-107	Ribulose bisphosphate carboxylase small chain, chloroplastic	PF12338.5^RbcS^Ribulose-1,5-bisphosphate carboxylase small subunit^2-42^E:6.5e-21`PF00101.17^RuBisCO_small^Ribulose bisphosphate carboxylase, small chain^69-177^E:1.2e-39	0,513	-0,135	0	-0,724	0	-0,87	0,002	-0,654	0,213	-0,257	RBS_MALSP	Ribulose bisphosphate carboxylase small chain, chloroplastic (RuBisCO small subunit) (EC 4.1.1.39)	RBCS	Malus sp. (Crab apple)		Calvin cycle; Carbon dioxide fixation; Chloroplast; Lyase; Monooxygenase; Oxidoreductase; Photorespiration; Photosynthesis; Plastid; Transit peptide
SRR924147_TR37530_c0_g2_i2_len=2375	AFC2_ARATH	Q:1186-542,H:212-425	80.09%ID	E:3e-116	Serine/threonine-protein kinase AFC2	PF00069.22^Pkinase^Protein kinase domain^1-211^E:3e-29`PF07714.14^Pkinase_Tyr^Protein tyrosine kinase^2-123^E:5.3e-08	0,155	-1,15	0	-6,876	0	-7,317	0,014	-1,801	1	0	AFC2_ARATH	Serine/threonine-protein kinase AFC2 (EC 2.7.12.1)	AFC2 At4g24740 F22K18.60	Arabidopsis thaliana (Mouse-ear cress)		ATP-binding; Alternative splicing; Complete proteome; Kinase; Nucleotide-binding; Reference proteome; Serine/threonine-protein kinase; Transferase
SRR924148_TR23282_c0_g1_i1_len=1579	PRXQ_GENTR	Q:1264-620,H:1-217	77.42%ID	E:1e-112	Peroxiredoxin Q, chloroplastic	PF08534.7^Redoxin^Redoxin^74-206^E:3.4e-26`PF00578.18^AhpC-TSA^AhpC/TSA family^75-193^E:4.5e-41	0,583	-0,174	0	-1,177	0	-0,972	0,006	-0,748	0,635	0,387	PRXQ_GENTR	Peroxiredoxin Q, chloroplastic (EC 1.11.1.15) (Thioredoxin reductase)	AFP1	Gentiana triflora (Clustered gentian)		Antioxidant; Chloroplast; Disulfide bond; Oxidoreductase; Peroxidase; Plastid; Redox-active center; Thylakoid; Transit peptide
SRR342022_TR6161_c1_g4_i7_len=1903	PGKH_TOBAC	Q:1723-308,H:1-472	91.31%ID	E:0	Phosphoglycerate kinase, chloroplastic	PF00162.16^PGK^Phosphoglycerate kinase^146-524^E:3.9e-161	0,421	-0,182	0,107	-0,35	0	-1,044	0,001	-0,692	0	-3,32	PGKH_TOBAC	Phosphoglycerate kinase, chloroplastic (EC 2.7.2.3)		Nicotiana tabacum (Common tobacco)	PATHWAY: Carbohydrate biosynthesis; Calvin cycle.	ATP-binding; Calvin cycle; Chloroplast; Kinase; Nucleotide-binding; Plastid; Transferase; Transit peptide
SRR342022_TR28030_c0_g3_i1_len=1265	CB23_TOBAC	Q:161-955,H:1-265	92.45%ID	E:4e-180	Chlorophyll a-b binding protein 36, chloroplastic	PF00504.18^Chloroa_b-bind^Chlorophyll A-B binding protein^66-231^E:5.2e-50	0,249	0,374	0,027	-0,619	0	-1,777	0,02	-0,638	0	-3,315	CB23_TOBAC	Chlorophyll a-b binding protein 36, chloroplastic (LHCII type I CAB-36) (LHCP)	CAB36	Nicotiana tabacum (Common tobacco)		Acetylation; Chlorophyll; Chloroplast; Chromophore; Magnesium; Membrane; Metal-binding; Phosphoprotein; Photosynthesis; Photosystem I; Photosystem II; Plastid; Thylakoid; Transit peptide; Transmembrane; Transmembrane helix
SRR122238_TR32454_c2_g1_i1_len=738	GASA4_ARATH	Q:154-411,H:20-106	70.45%ID	E:3e-34	Gibberellin-regulated protein 4	PF02704.11^GASA^Gibberellin regulated protein^47-106^E:3.7e-22	0,001	-0,872	0	-3,035	0	-1,456	0,003	-1,054	0,016	-0,672	GASA4_ARATH	Gibberellin-regulated protein 4 (GAST1 protein homolog 4)	GASA4 At5g15230 F8M21.120	Arabidopsis thaliana (Mouse-ear cress)		Alternative splicing; Complete proteome; Disulfide bond; Gibberellin signaling pathway; Reference proteome; Secreted; Signal
SRR342019_TR36750_c2_g2_i1_len=2441	DCUP_TOBAC	Q:2128-1028,H:23-391	86.18%ID	E:0	Uroporphyrinogen decarboxylase, chloroplastic	PF01208.14^URO-D^Uroporphyrinogen decarboxylase (URO-D)^50-387^E:4.6e-116	0,001	-0,923	0	-1,438	0	-1,442	0,003	-0,874	1	-0,549	DCUP_TOBAC	Uroporphyrinogen decarboxylase, chloroplastic (UPD) (URO-D) (EC 4.1.1.37)	DCUP	Nicotiana tabacum (Common tobacco)	PATHWAY: Porphyrin-containing compound metabolism; protoporphyrin-IX biosynthesis; coproporphyrinogen-III from 5-aminolevulinate: step 4/4.	3D-structure; Chlorophyll biosynthesis; Chloroplast; Decarboxylase; Lyase; Plastid; Porphyrin biosynthesis; Transit peptide
SRR342019_TR36856_c2_g3_i7_len=4101	EFTS_SYNR3	Q:2157-2801,H:2-217	61.57%ID	E:2e-87	Elongation factor Ts {ECO:0000255|HAMAP-Rule:MF_00050}	PF00575.20^S1^S1 RNA binding domain^149-218^E:1.1e-15`PF00575.20^S1^S1 RNA binding domain^263-327^E:6.9e-09`PF00889.16^EF_TS^Elongation factor TS^672-814^E:5.9e-30`PF00889.16^EF_TS^Elongation factor TS^912-1054^E:3.9e-30	0,001	-0,966	0	-1,348	0	-1,198	0,162	-0,46	0,624	0,151	EFTS_SYNR3	Elongation factor Ts (EF-Ts)	tsf SynRCC307_1273	Synechococcus sp. (strain RCC307)		Complete proteome; Cytoplasm; Elongation factor; Protein biosynthesis; Reference proteome
SRR648705_TR21225_c0_g1_i2_len=1540	NA	NA	NA	NA	NA	PF13181.3^TPR_8^Tetratricopeptide repeat^231-262^E:0.11`PF13432.3^TPR_16^Tetratricopeptide repeat^236-290^E:3.2e-07`PF07719.14^TPR_2^Tetratricopeptide repeat^266-298^E:3.6e-07`PF13181.3^TPR_8^Tetratricopeptide repeat^266-298^E:6e-06`PF00515.25^TPR_1^Tetratricopeptide repeat^267-298^E:8.3e-08`PF13176.3^TPR_7^Tetratricopeptide repeat^267-297^E:0.0081`PF01535.17^PPR^PPR repeat^267-290^E:0.84	1	-0,613	0	-2,19	0,011	-1,46	0	-7,706	0	-4,06	.	.	.	.	.	.
SRR122238_TR49280_c0_g1_i1_len=1038	CYB5_TOBAC	Q:762-358,H:1-135	84.44%ID	E:4e-80	Cytochrome b5	PF00173.25^Cyt-b5^Cytochrome b5-like Heme/Steroid binding domain^9-80^E:5e-26	0	-10,138	0,55	0,189	0	-8,069	0	-8,069	0,56	-0,666	CYB5_TOBAC	Cytochrome b5		Nicotiana tabacum (Common tobacco)		Electron transport; Endoplasmic reticulum; Heme; Iron; Membrane; Metal-binding; Microsome; Transmembrane; Transmembrane helix; Transport
SRR1271857_TR28344_c0_g4_i1_len=847	ATPG_TOBAC	Q:173-847,H:1-223	80.89%ID	E:4e-98	ATP synthase gamma chain, chloroplastic	PF00231.16^ATP-synt^ATP synthase^59-225^E:8.2e-42	0	-2,597	1	0	0	-5,607	0	-2,452	0,254	4,628	ATPG_TOBAC	ATP synthase gamma chain, chloroplastic (F-ATPase gamma subunit)	ATPC	Nicotiana tabacum (Common tobacco)		ATP synthesis; CF(1); Chloroplast; Direct protein sequencing; Disulfide bond; Hydrogen ion transport; Ion transport; Membrane; Plastid; Thylakoid; Transit peptide; Transport
SRR1271859_TR26429_c0_g3_i1_len=1023	CRD1_ARATH	Q:427-897,H:1-158	72.96%ID	E:1e-62	Magnesium-protoporphyrin IX monomethyl ester [oxidative] cyclase, chloroplastic	.	0	-1,843	0,009	6,097	0	-5,267	0	-1,996	0	3,481	CRD1_ARATH	Magnesium-protoporphyrin IX monomethyl ester [oxidative] cyclase, chloroplastic (Mg-protoporphyrin IX monomethyl ester oxidative cyclase) (EC 1.14.13.81) (Copper response defect 1 protein) (Dicarboxylate diiron protein) (AtZIP) (MPE-cyclase)	CRD1 ACSF AT103 CHL27 ZIP At3g56940 F24I3.20	Arabidopsis thaliana (Mouse-ear cress)	PATHWAY: Porphyrin-containing compound metabolism; chlorophyll biosynthesis.	Alternative splicing; Chlorophyll biosynthesis; Chloroplast; Complete proteome; Iron; Membrane; Metal-binding; NADP; Oxidoreductase; Photosynthesis; Plastid; Plastid inner membrane; Reference proteome; Thylakoid; Transit peptide
SRR1271859_TR27972_c6_g5_i3_len=1278	FER_MASLA	Q:456-734,H:3-98	50%ID	E:2e-21	Ferredoxin	PF00111.24^Fer2^2Fe-2S iron-sulfur cluster binding domain^58-130^E:6.2e-13	0	-3,808	0,16	-1,303	0	-6,095	0	-4,959	0,337	0,445	FER_MASLA	Ferredoxin	petF	Mastigocladus laminosus (Fischerella sp.)		2Fe-2S; 3D-structure; Direct protein sequencing; Electron transport; Iron; Iron-sulfur; Metal-binding; Transport
SRR342017_TR991_c3_g2_i2_len=561	CYB5_TOBAC	Q:220-561,H:1-114	85.09%ID	E:6e-69	Cytochrome b5	PF00173.25^Cyt-b5^Cytochrome b5-like Heme/Steroid binding domain^82-153^E:1.1e-25	0	-10,978	0,187	0,392	0	-8,687	0	-8,687	0,614	0,127	CYB5_TOBAC	Cytochrome b5		Nicotiana tabacum (Common tobacco)		Electron transport; Endoplasmic reticulum; Heme; Iron; Membrane; Metal-binding; Microsome; Transmembrane; Transmembrane helix; Transport
SRR342023_TR6839_c0_g1_i1_len=813	CYB5_TOBAC	Q:222-626,H:1-135	84.44%ID	E:2e-81	Cytochrome b5	PF00173.25^Cyt-b5^Cytochrome b5-like Heme/Steroid binding domain^9-80^E:5e-26	0	-5,884	0,472	0,34	0	-7,431	0	-4,812	1	-0,014	CYB5_TOBAC	Cytochrome b5		Nicotiana tabacum (Common tobacco)		Electron transport; Endoplasmic reticulum; Heme; Iron; Membrane; Metal-binding; Microsome; Transmembrane; Transmembrane helix; Transport
SRR342023_TR19867_c0_g3_i3_len=632	NA	NA	NA	NA	NA	PF06596.8^PsbX^Photosystem II reaction centre X protein (PsbX)^89-126^E:1.4e-16	0	-1,557	0,361	-0,228	0	-1,503	0	-1,348	1	2,838	.	.	.	.	.	.
SRR1144633_TR24528_c0_g2_i1_len=766	NA	NA	NA	NA	NA	PF04570.11^zf-FLZ^zinc-finger of the FCS-type, C2-C2^73-123^E:3.4e-26	0,064	-0,435	0	-1,175	0	-0,935	0	-1,105	0,892	0,13	.	.	.	.	.	.
SRR1144633_TR35976_c0_g2_i1_len=1302	NA	NA	NA	NA	NA	.	0,151	-0,586	0	-1,659	0,001	-1,729	0	-2,53	1	1,696	.	.	.	.	.	.
SRR1144634_TR22153_c0_g2_i1_len=1013	PSAD_CUCSA	Q:200-934,H:1-207	67.35%ID	E:7e-100	Photosystem I reaction center subunit II, chloroplastic	PF02531.13^PsaD^PsaD^113-243^E:3.7e-68	0,586	-0,113	0	-0,859	0	-0,885	0,001	-0,692	0,611	-0,39	PSAD_CUCSA	Photosystem I reaction center subunit II, chloroplastic (PS I subunit 5) (Photosystem I 20 kDa subunit) (PSI-D)	psaD	Cucumis sativus (Cucumber)		Chloroplast; Direct protein sequencing; Membrane; Photosynthesis; Photosystem I; Plastid; Thylakoid; Transit peptide
SRR122236_TR6354_c0_g1_i1_len=630	PSAH1_ARATH	Q:576-208,H:1-123	81.3%ID	E:2e-51	Photosystem I reaction center subunit VI-1, chloroplastic	PF03244.11^PSI_PsaH^Photosystem I reaction centre subunit VI^25-163^E:3e-71	0,956	0,012	0	-1,252	0	-0,731	0,001	-0,708	0,447	-0,227	PSAH1_ARATH	Photosystem I reaction center subunit VI-1, chloroplastic (PSI-H1)	PSAH1 At3g16140 MSL1.18	Arabidopsis thaliana (Mouse-ear cress)		Chloroplast; Complete proteome; Membrane; Photosynthesis; Photosystem I; Plastid; Reference proteome; Thylakoid; Transit peptide; Transmembrane; Transmembrane helix
SRR122236_TR6924_c0_g1_i1_len=817	NA	NA	NA	NA	NA	.	0,143	-0,324	0	-1,971	0	-1,472	0	-1,272	0,429	-0,221	.	.	.	.	.	.
SRR122236_TR21773_c0_g1_i1_len=664	RK34_SPIOL	Q:597-127,H:1-150	53.7%ID	E:2e-33	50S ribosomal protein L34, chloroplastic	PF00468.14^Ribosomal_L34^Ribosomal protein L34^111-146^E:4.6e-13	0,026	-0,506	0	-1,592	0	-0,969	0	-0,95	0,016	-0,534	RK34_SPIOL	50S ribosomal protein L34, chloroplastic (CL34)	RPL34	Spinacia oleracea (Spinach)		3D-structure; Chloroplast; Direct protein sequencing; Plastid; RNA-binding; Ribonucleoprotein; Ribosomal protein; Transit peptide; rRNA-binding
SRR122236_TR24817_c0_g2_i1_len=1068	TLPH_ARATH	Q:894-220,H:17-241	77.33%ID	E:1e-125	Thaumatin-like protein	PF00314.14^Thaumatin^Thaumatin family^69-281^E:1.1e-82	0,065	-0,511	0	-5,063	0	-2,46	0	-3,281	1	-0,283	TLPH_ARATH	Thaumatin-like protein	At1g18250 T10O22.21	Arabidopsis thaliana (Mouse-ear cress)		Alternative splicing; Complete proteome; Disulfide bond; Reference proteome; Signal
SRR122237_TR14226_c0_g1_i1_len=656	NA	NA	NA	NA	NA	PF08041.8^PetM^PetM family of cytochrome b6f complex subunit 7^123-150^E:4.3e-11	0,474	0,163	0	-1,539	0	-1	0	-1,146	0,108	-0,353	.	.	.	.	.	.
SRR122238_TR2593_c0_g1_i2_len=922	NA	NA	NA	NA	NA	PF09713.7^A_thal_3526^Plant protein 1589 of unknown function (A_thal_3526)^51-101^E:2.5e-24	1	-0,021	0	-1,645	0	-1,793	0	-1,43	0,852	0,068	.	.	.	.	.	.
SRR122238_TR31553_c0_g4_i1_len=628	ARG7_VIGRR	Q:339-52,H:1-90	57.14%ID	E:4e-30	Indole-3-acetic acid-induced protein ARG7	.	0,002	-1,31	0	-3,937	0,001	-1,804	0	-1,937	1	0,061	ARG7_VIGRR	Indole-3-acetic acid-induced protein ARG7	ARG7	Vigna radiata var. radiata (Mung bean) (Phaseolus aureus)		
SRR122238_TR37137_c0_g3_i1_len=995	CB13_SOLLC	Q:112-801,H:44-273	90.87%ID	E:1e-152	Chlorophyll a-b binding protein 8, chloroplastic	PF00504.18^Chloroa_b-bind^Chlorophyll A-B binding protein^58-236^E:1.4e-49	0,554	-0,125	0	-1,589	0	-0,923	0	-0,857	0,846	0,041	CB13_SOLLC	Chlorophyll a-b binding protein 8, chloroplastic (LHCI type III CAB-8)	CAB8	Solanum lycopersicum (Tomato) (Lycopersicon esculentum)		Acetylation; Chlorophyll; Chloroplast; Chromophore; Complete proteome; Magnesium; Membrane; Metal-binding; Phosphoprotein; Photosynthesis; Photosystem I; Photosystem II; Plastid; Reference proteome; Thylakoid; Transit peptide; Transmembrane; Transmembrane helix
SRR122238_TR39804_c0_g1_i2_len=840	NA	NA	NA	NA	NA	PF05512.8^AWPM-19^AWPM-19-like family^15-155^E:8.9e-63	0,68	0,178	0	-1,711	0	-1,904	0	-2,244	1	0,041	.	.	.	.	.	.
SRR122238_TR40315_c0_g4_i2_len=1105	CB24_PEA	Q:1018-272,H:1-249	86.75%ID	E:2e-157	Chlorophyll a-b binding protein P4, chloroplastic	PF00504.18^Chloroa_b-bind^Chlorophyll A-B binding protein^63-222^E:7.4e-48	0,232	-0,25	0	-1,5	0	-1,027	0	-0,764	0,021	0,481	CB24_PEA	Chlorophyll a-b binding protein P4, chloroplastic (LHCI type III CAB-P4)	lhcA-P4	Pisum sativum (Garden pea)		3D-structure; Chlorophyll; Chloroplast; Chromophore; Direct protein sequencing; Magnesium; Membrane; Metal-binding; Phosphoprotein; Photosynthesis; Photosystem I; Photosystem II; Plastid; Thylakoid; Transit peptide; Transmembrane; Transmembrane helix
SRR122238_TR42031_c0_g1_i2_len=1366	APG2_ARATH	Q:1244-264,H:27-353	72.78%ID	E:4e-165	GDSL esterase/lipase APG	PF00657.19^Lipase_GDSL^GDSL-like Lipase/Acylhydrolase^45-357^E:3.3e-17	0,112	-0,394	0	-0,9	0	-1,23	0,001	-0,83	0,072	0,448	APG2_ARATH	GDSL esterase/lipase APG (EC 3.1.1.-) (Extracellular lipase APG)	APG At3g16370 MYA6.18 T2O4.2	Arabidopsis thaliana (Mouse-ear cress)		Complete proteome; Glycoprotein; Hydrolase; Lipid degradation; Lipid metabolism; Reference proteome; Secreted; Signal
SRR122238_TR53068_c1_g1_i1_len=822	PSAO_ARATH	Q:282-620,H:28-140	87.61%ID	E:1e-65	Photosystem I subunit O	.	0,584	0,115	0	-1,265	0	-1,014	0	-0,901	0,303	0,214	PSAO_ARATH	Photosystem I subunit O (PSI-O)	PSAO At1g08380 T27G7	Arabidopsis thaliana (Mouse-ear cress)		Chloroplast; Complete proteome; Direct protein sequencing; Membrane; Photosynthesis; Photosystem I; Plastid; Reference proteome; Thylakoid; Transit peptide; Transmembrane; Transmembrane helix
SRR122238_TR56703_c0_g1_i1_len=1420	GAT22_ARATH	Q:257-961,H:117-352	39.85%ID	E:1e-31	Putative GATA transcription factor 22	PF00320.24^GATA^GATA zinc finger^174-207^E:1.1e-16	0,077	-0,685	0	-1,785	0	-2,131	0	-2,737	0,321	0,352	GAT22_ARATH	Putative GATA transcription factor 22	GATA22 At4g26150 F20B18.260	Arabidopsis thaliana (Mouse-ear cress)		Complete proteome; DNA-binding; Metal-binding; Nucleus; Reference proteome; Transcription; Transcription regulation; Zinc; Zinc-finger
SRR122238_TR57396_c0_g1_i1_len=553	ARG7_VIGRR	Q:353-162,H:23-87	61.54%ID	E:5e-21	Indole-3-acetic acid-induced protein ARG7	PF02519.11^Auxin_inducible^Auxin responsive protein^53-132^E:1.3e-28	0,15	-0,393	0	-2,749	0	-3,087	0	-3,225	0,131	0,452	ARG7_VIGRR	Indole-3-acetic acid-induced protein ARG7	ARG7	Vigna radiata var. radiata (Mung bean) (Phaseolus aureus)		
SRR122238_TR64038_c0_g1_i3_len=2583	NA	NA	NA	NA	NA	PF12452.5^DUF3685^Protein of unknown function (DUF3685)^313-402^E:1.5e-07`PF12452.5^DUF3685^Protein of unknown function (DUF3685)^422-500^E:4.1e-13	0,571	-0,221	0	-1,474	0,001	-1,159	0	-1,247	0,635	-0,318	.	.	.	.	.	.
SRR122238_TR67591_c0_g1_i1_len=1165	PSAF_FLATR	Q:862-401,H:79-232	95.45%ID	E:2e-93	Photosystem I reaction center subunit III, chloroplastic	PF02507.12^PSI_PsaF^Photosystem I reaction centre subunit III^57-221^E:4.3e-82	0,538	-0,128	0	-0,874	0	-1,024	0	-0,838	0,15	-0,308	PSAF_FLATR	Photosystem I reaction center subunit III, chloroplastic (Light-harvesting complex I 17 kDa protein) (PSI-F)	PSAF	Flaveria trinervia (Clustered yellowtops) (Oedera trinervia)		Chloroplast; Photosynthesis; Photosystem I; Plastid; Thylakoid; Transit peptide
SRR122238_TR67591_c0_g1_i2_len=1708	PSAF_FLATR	Q:1405-944,H:79-232	95.45%ID	E:5e-91	Photosystem I reaction center subunit III, chloroplastic	PF02507.12^PSI_PsaF^Photosystem I reaction centre subunit III^57-221^E:4.3e-82	0,481	0,255	0	-1,849	0,001	-1,096	0	-1,211	0,541	-0,25	PSAF_FLATR	Photosystem I reaction center subunit III, chloroplastic (Light-harvesting complex I 17 kDa protein) (PSI-F)	PSAF	Flaveria trinervia (Clustered yellowtops) (Oedera trinervia)		Chloroplast; Photosynthesis; Photosystem I; Plastid; Thylakoid; Transit peptide
SRR122238_TR70905_c0_g1_i1_len=1419	GDL79_ARATH	Q:188-1198,H:26-362	78.93%ID	E:0	GDSL esterase/lipase At5g33370	PF00657.19^Lipase_GDSL^GDSL-like Lipase/Acylhydrolase^68-379^E:8.8e-14	0,051	-0,451	0,001	-1,13	0	-1,761	0	-1,752	0,646	0,109	GDL79_ARATH	GDSL esterase/lipase At5g33370 (EC 3.1.1.-) (Extracellular lipase At5g33370)	At5g33370 F19N2.90	Arabidopsis thaliana (Mouse-ear cress)		Alternative splicing; Complete proteome; Glycoprotein; Hydrolase; Lipid degradation; Lipid metabolism; Reference proteome; Secreted; Signal
SRR122238_TR71771_c0_g1_i4_len=1738	HPR1_ARATH	Q:1494-337,H:1-386	90.93%ID	E:0	Glycerate dehydrogenase HPR, peroxisomal	PF00389.27^2-Hacid_dh^D-isomer specific 2-hydroxyacid dehydrogenase, catalytic domain^35-346^E:2.7e-17`PF02826.16^2-Hacid_dh_C^D-isomer specific 2-hydroxyacid dehydrogenase, NAD binding domain^130-322^E:5e-47	0,259	-0,324	0,001	-0,914	0	-1,127	0	-1,138	0,811	0,252	HPR1_ARATH	Glycerate dehydrogenase HPR, peroxisomal (GDH) (EC 1.1.1.29) (NADH-dependent hydroxypyruvate reductase 1) (AtHPR1) (HPR 1)	HPR At1g68010 T23K23.14	Arabidopsis thaliana (Mouse-ear cress)	PATHWAY: Photosynthesis; photorespiration; 3-phospho-D-glycerate from glycine: step 3/4.	Alternative splicing; Complete proteome; Direct protein sequencing; Glycolate pathway; NAD; Oxidoreductase; Peroxisome; Photorespiration; Pyruvate; Reference proteome
SRR122238_TR82037_c0_g1_i2_len=749	NA	NA	NA	NA	NA	.	0,518	-0,152	0	-2,285	0	-1,276	0	-1,086	0,026	-0,475	.	.	.	.	.	.
SRR122238_TR82446_c0_g1_i2_len=1893	WSD1_ARATH	Q:1577-414,H:60-446	25.12%ID	E:8e-28	O-acyltransferase WSD1	PF03007.13^WES_acyltransf^Wax ester synthase-like Acyl-CoA acyltransferase domain^104-267^E:3.1e-12`PF06974.10^DUF1298^Protein of unknown function (DUF1298)^320-466^E:4.5e-34	0,007	-0,782	0	-1,845	0,001	-2,042	0,001	-2,123	0,801	0,075	WSD1_ARATH	O-acyltransferase WSD1 (Diacylglycerol O-acyltransferase) (DGAT) (EC 2.3.1.20) (Long-chain-alcohol O-fatty-acyltransferase) (EC 2.3.1.75) (Wax synthase) (WS)	WSD1 At5g37300 MNJ8.9	Arabidopsis thaliana (Mouse-ear cress)	PATHWAY: Glycerolipid metabolism; triacylglycerol biosynthesis.	Acyltransferase; Complete proteome; Endoplasmic reticulum; Membrane; Reference proteome; Transferase; Transmembrane; Transmembrane helix
SRR122238_TR88044_c0_g1_i7_len=2117	CYP38_ARATH	Q:336-1598,H:23-437	76.92%ID	E:0	Peptidyl-prolyl cis-trans isomerase CYP38, chloroplastic	.	0,093	-0,622	0	-1,63	0	-2,079	0	-1,67	0,522	-0,256	CYP38_ARATH	Peptidyl-prolyl cis-trans isomerase CYP38, chloroplastic (PPIase CYP38) (EC 5.2.1.8) (Rotamase CYP38) (Thylakoid lumen PPIase)	CYP38 At3g01480 F4P13.3	Arabidopsis thaliana (Mouse-ear cress)		3D-structure; Alternative splicing; Chloroplast; Complete proteome; Direct protein sequencing; Isomerase; Plastid; Reference proteome; Rotamase; Thylakoid; Transit peptide
SRR122238_TR88088_c1_g1_i1_len=720	NA	NA	NA	NA	NA	.	0,015	-0,581	0	-1,799	0	-1,338	0	-0,888	0,002	-0,693	.	.	.	.	.	.
SRR1271857_TR8068_c0_g2_i2_len=1540	BH096_ARATH	Q:1182-397,H:80-319	49.62%ID	E:4e-62	Transcription factor bHLH96	PF00010.23^HLH^Helix-loop-helix DNA-binding domain^163-214^E:2.9e-11	0,319	-0,274	0	-1,788	0	-1,41	0	-1,282	0,321	-0,298	BH096_ARATH	Transcription factor bHLH96 (Basic helix-loop-helix protein 96) (AtbHLH96) (bHLH 96) (Transcription factor EN 15) (bHLH transcription factor bHLH096)	BHLH96 EN15 At1g72210 T9N14.4	Arabidopsis thaliana (Mouse-ear cress)		Complete proteome; DNA-binding; Nucleus; Reference proteome; Transcription; Transcription regulation
SRR1271857_TR10347_c3_g1_i3_len=4570	NA	NA	NA	NA	NA	.	0,515	-0,239	0	-1,581	0	-2,657	0	-2,679	0,443	-0,182	.	.	.	.	.	.
SRR1271857_TR15428_c0_g1_i4_len=1346	CUT1B_ARATH	Q:400-816,H:35-174	65.52%ID	E:8e-51	Protein CURVATURE THYLAKOID 1B, chloroplastic	PF14159.3^CAAD^CAAD domains of cyanobacterial aminoacyl-tRNA synthetase^95-177^E:5.5e-30	0,713	0,081	0,001	-0,747	0	-0,945	0	-0,848	0,565	-0,13	CUT1B_ARATH	Protein CURVATURE THYLAKOID 1B, chloroplastic (Photosystem I protein P) (Thylakoid membrane phosphoprotein 14 kDa)	CURT1B PSAP PSI-P TMP14 At2g46820 F19D11.10	Arabidopsis thaliana (Mouse-ear cress)		Acetylation; Chloroplast; Complete proteome; Direct protein sequencing; Membrane; Phosphoprotein; Plastid; Reference proteome; Thylakoid; Transit peptide; Transmembrane; Transmembrane helix
SRR1271857_TR15597_c0_g2_i1_len=6870	CLU_ARATH	Q:4342-3044,H:787-1258	38.9%ID	E:2e-94	Clustered mitochondria protein	PF15044.3^CLU_N^Mitochondrial function, CLU-N-term^48-117^E:1.5e-08`PF12807.4^eIF3_p135^Translation initiation factor eIF3 subunit 135^734-877^E:2.5e-23`PF13424.3^TPR_12^Tetratricopeptide repeat^947-1017^E:2.8e-13`PF13374.3^TPR_10^Tetratricopeptide repeat^947-988^E:2.5e-08`PF13181.3^TPR_8^Tetratricopeptide repeat^949-979^E:0.19`PF13424.3^TPR_12^Tetratricopeptide repeat^1031-1105^E:1.6e-11`PF13374.3^TPR_10^Tetratricopeptide repeat^1031-1070^E:1.9e-06	0,044	-0,717	0	-2,019	0	-1,752	0	-1,326	0,188	0,501	CLU_ARATH	Clustered mitochondria protein (Friendly mitochondria protein)	FMT CLU At3g52140 F4F15.250	Arabidopsis thaliana (Mouse-ear cress)		Alternative splicing; Complete proteome; Cytoplasm; Reference proteome; Repeat; TPR repeat
SRR1271857_TR19276_c2_g1_i1_len=1665	ALF1_PEA	Q:1391-315,H:1-357	85.52%ID	E:0	Fructose-bisphosphate aldolase, cytoplasmic isozyme 1	PF00274.16^Glycolytic^Fructose-bisphosphate aldolase class-I^11-359^E:1.1e-173	0,06	-0,604	0	-2,704	0	-3,037	0	-3,879	0,061	0,515	ALF1_PEA	Fructose-bisphosphate aldolase, cytoplasmic isozyme 1 (EC 4.1.2.13)		Pisum sativum (Garden pea)	PATHWAY: Carbohydrate degradation; glycolysis; D-glyceraldehyde 3-phosphate and glycerone phosphate from D-glucose: step 4/4.	Cytoplasm; Glycolysis; Lyase; Schiff base
SRR1271857_TR32438_c0_g1_i2_len=1794	DAAA_ARATH	Q:679-1578,H:73-373	59.47%ID	E:1e-122	D-amino-acid transaminase, chloroplastic {ECO:0000303|PubMed:18318836}	PF01063.16^Aminotran_4^Amino-transferase class IV^30-254^E:1.2e-40	0,155	-1,121	0	-3,178	0	-1,986	0,001	-1,819	0,503	0,208	DAAA_ARATH	D-amino-acid transaminase, chloroplastic (EC 2.6.1.21) (EC 4.1.3.38) (Aminodeoxychorismate lyase) (AtADCL) (Branched-chain-amino-acid aminotransferase-like protein 3)	DAAT BCAL3 At5g57850 MTI20.10	Arabidopsis thaliana (Mouse-ear cress)	PATHWAY: Cofactor biosynthesis; tetrahydrofolate biosynthesis; 4-aminobenzoate from chorismate: step 2/2.	Aminotransferase; Chloroplast; Complete proteome; Lyase; Plastid; Pyridoxal phosphate; Reference proteome; Transferase; Transit peptide
SRR1271857_TR37789_c0_g1_i3_len=2638	CA4_ARATH	Q:952-404,H:76-250	45.9%ID	E:4e-44	Chlorophyll a-b binding protein 4, chloroplastic {ECO:0000303|PubMed:10366881}	PF00504.18^Chloroa_b-bind^Chlorophyll A-B binding protein^66-234^E:5.1e-48	0,275	-0,304	0	-1,549	0	-1,511	0	-1,356	0,331	0,342	CA4_ARATH	Chlorophyll a-b binding protein 4, chloroplastic (LHCI type III CAB-4)	LHCA4 CAB4 At3g47470 F1P2.20	Arabidopsis thaliana (Mouse-ear cress)		Chlorophyll; Chloroplast; Chromophore; Complete proteome; Magnesium; Membrane; Metal-binding; Phosphoprotein; Photosynthesis; Photosystem I; Photosystem II; Plastid; Reference proteome; Thylakoid; Transit peptide; Transmembrane; Transmembrane helix
SRR1271857_TR42253_c0_g1_i1_len=1539	SLD1_BOROF	Q:1348-680,H:1-223	63.23%ID	E:2e-100	Delta(8)-fatty-acid desaturase	PF00173.25^Cyt-b5^Cytochrome b5-like Heme/Steroid binding domain^9-77^E:6.5e-24`PF00487.21^FA_desaturase^Fatty acid desaturase^137-223^E:4.8e-11	0,025	-0,721	0	-2,136	0	-1,644	0	-1,422	0,332	0,27	SLD1_BOROF	Delta(8)-fatty-acid desaturase (EC 1.14.19.4) (Delta(8)-sphingolipid desaturase)	sld1	Borago officinalis (Bourrache) (Borage)		Electron transport; Heme; Iron; Lipid metabolism; Membrane; Metal-binding; Oxidoreductase; Sphingolipid metabolism; Transmembrane; Transmembrane helix; Transport
SRR1271858_TR18941_c0_g1_i8_len=1970	NA	NA	NA	NA	NA	PF09713.7^A_thal_3526^Plant protein 1589 of unknown function (A_thal_3526)^25-42^E:2e-06	0,564	-0,532	0	-2,986	0	-3,097	0	-3,663	0,882	0,154	.	.	.	.	.	.
SRR1271859_TR4946_c0_g3_i6_len=1085	CB22_TOBAC	Q:972-178,H:1-265	93.96%ID	E:0	Chlorophyll a-b binding protein 21, chloroplastic	PF00504.18^Chloroa_b-bind^Chlorophyll A-B binding protein^65-231^E:1.6e-50	0,586	-0,112	0	-1,197	0	-1,307	0	-1,14	0,006	0,576	CB22_TOBAC	Chlorophyll a-b binding protein 21, chloroplastic (LHCII type I CAB-21) (LHCP)	CAB21	Nicotiana tabacum (Common tobacco)		Acetylation; Chlorophyll; Chloroplast; Chromophore; Magnesium; Membrane; Metal-binding; Phosphoprotein; Photosynthesis; Photosystem I; Photosystem II; Plastid; Thylakoid; Transit peptide; Transmembrane; Transmembrane helix
SRR1271859_TR9590_c0_g1_i4_len=1071	BH063_ARATH	Q:189-761,H:149-331	52.74%ID	E:2e-53	Transcription factor bHLH63	.	0,122	-0,701	0	-3,388	0	-3,368	0	-2,943	0,803	-0,181	BH063_ARATH	Transcription factor bHLH63 (Basic helix-loop-helix protein 63) (AtbHLH63) (bHLH 63) (Protein CRYPTOCHROME INTERACTING BASIC-HELIX-LOOP-HELIX 1) (Transcription factor EN 84) (bHLH transcription factor bHLH063)	BHLH63 CIB1 EN84 At4g34530 T4L20.110	Arabidopsis thaliana (Mouse-ear cress)		Complete proteome; DNA-binding; Nucleus; Reference proteome; Transcription; Transcription regulation
SRR1271859_TR10640_c0_g1_i3_len=2408	NA	NA	NA	NA	NA	PF05368.10^NmrA^NmrA-like family^97-210^E:5.9e-06`PF13460.3^NAD_binding_10^NAD(P)H-binding^101-284^E:1.8e-13	0,282	-0,378	0	-1,24	0	-1,16	0	-1,296	0,881	0,099	.	.	.	.	.	.
SRR1271859_TR10640_c0_g1_i4_len=2605	NA	NA	NA	NA	NA	PF05368.10^NmrA^NmrA-like family^97-210^E:5.9e-06`PF13460.3^NAD_binding_10^NAD(P)H-binding^101-284^E:1.8e-13	0,537	-0,336	0	-1,753	0	-1,477	0,001	-1,363	0,902	0,137	.	.	.	.	.	.
SRR1271859_TR26429_c0_g1_i4_len=2808	CRD1_GOSHI	Q:427-1650,H:1-405	85.68%ID	E:0	Magnesium-protoporphyrin IX monomethyl ester [oxidative] cyclase, chloroplastic	PF02915.14^Rubrerythrin^Rubrerythrin^133-266^E:2.5e-41	0,162	-0,306	0	-1,335	0	-0,995	0	-0,763	0,136	0,326	CRD1_GOSHI	Magnesium-protoporphyrin IX monomethyl ester [oxidative] cyclase, chloroplastic (Mg-protoporphyrin IX monomethyl ester oxidative cyclase) (EC 1.14.13.81)	CRD1 ZIP	Gossypium hirsutum (Upland cotton) (Gossypium mexicanum)	PATHWAY: Porphyrin-containing compound metabolism; chlorophyll biosynthesis.	Chlorophyll biosynthesis; Chloroplast; Iron; Metal-binding; NADP; Oxidoreductase; Photosynthesis; Plastid; Transit peptide
SRR342017_TR2783_c0_g1_i1_len=945	NA	NA	NA	NA	NA	.	0,006	-0,82	0	-1,778	0	-1,701	0	-1,773	0,642	-0,156	.	.	.	.	.	.
SRR342017_TR7269_c3_g1_i2_len=1531	CAHC_TOBAC	Q:131-964,H:43-321	82.8%ID	E:3e-154	Carbonic anhydrase, chloroplastic	PF00484.16^Pro_CA^Carbonic anhydrase^148-305^E:2.4e-39	0,312	-0,285	0	-1,38	0	-1,368	0	-1,378	0,002	-0,661	CAHC_TOBAC	Carbonic anhydrase, chloroplastic (EC 4.2.1.1) (Carbonate dehydratase)		Nicotiana tabacum (Common tobacco)		Chloroplast; Lyase; Plastid; Transit peptide; Zinc
SRR342017_TR7269_c3_g1_i3_len=1220	BCA1_ARATH	Q:119-994,H:53-347	78.64%ID	E:3e-158	Beta carbonic anhydrase 1, chloroplastic {ECO:0000303|PubMed:17407539}	PF00484.16^Pro_CA^Carbonic anhydrase^147-304^E:2.6e-39	0,112	-0,464	0	-0,917	0	-1,48	0	-1,386	0,558	0,126	BCA1_ARATH	Beta carbonic anhydrase 1, chloroplastic (AtbCA1) (AtbetaCA1) (EC 4.2.1.1) (Beta carbonate dehydratase 1) (Protein SALICYLIC ACID-BINDING PROTEIN 3) (AtSABP3)	BCA1 CA1 SABP3 At3g01500 F4P13.5	Arabidopsis thaliana (Mouse-ear cress)		Acetylation; Alternative splicing; Cell membrane; Chloroplast; Complete proteome; Lyase; Membrane; Phosphoprotein; Plastid; Reference proteome; S-nitrosylation; Transit peptide; Zinc
SRR342017_TR9020_c2_g1_i1_len=905	RBS_MALSP	Q:779-240,H:1-183	81.42%ID	E:1e-107	Ribulose bisphosphate carboxylase small chain, chloroplastic	PF12338.5^RbcS^Ribulose-1,5-bisphosphate carboxylase small subunit^2-42^E:6.5e-21`PF00101.17^RuBisCO_small^Ribulose bisphosphate carboxylase, small chain^69-177^E:1.2e-39	0,236	-0,244	0	-1,042	0	-0,93	0	-0,827	0,135	-0,307	RBS_MALSP	Ribulose bisphosphate carboxylase small chain, chloroplastic (RuBisCO small subunit) (EC 4.1.1.39)	RBCS	Malus sp. (Crab apple)		Calvin cycle; Carbon dioxide fixation; Chloroplast; Lyase; Monooxygenase; Oxidoreductase; Photorespiration; Photosynthesis; Plastid; Transit peptide
SRR342017_TR9143_c0_g2_i1_len=757	SNAK1_SOLTU	Q:536-324,H:18-88	71.83%ID	E:2e-28	Snakin-1	.	0,281	-0,565	0,001	-6,575	0,001	-2,549	0	-3,577	0,44	-0,776	SNAK1_SOLTU	Snakin-1	SN1	Solanum tuberosum (Potato)		Antimicrobial; Cell wall; Complete proteome; Direct protein sequencing; Disulfide bond; Plant defense; Reference proteome; Secreted; Signal
SRR342017_TR9676_c1_g1_i2_len=1230	GDL79_ARATH	Q:1230-247,H:35-362	79.57%ID	E:0	GDSL esterase/lipase At5g33370	PF00657.19^Lipase_GDSL^GDSL-like Lipase/Acylhydrolase^1-308^E:4.9e-12	0,002	-0,782	0	-1,707	0	-1,528	0	-1,549	1	-0,089	GDL79_ARATH	GDSL esterase/lipase At5g33370 (EC 3.1.1.-) (Extracellular lipase At5g33370)	At5g33370 F19N2.90	Arabidopsis thaliana (Mouse-ear cress)		Alternative splicing; Complete proteome; Glycoprotein; Hydrolase; Lipid degradation; Lipid metabolism; Reference proteome; Secreted; Signal
SRR342017_TR18200_c0_g3_i1_len=1211	CB22_TOBAC	Q:1079-285,H:1-265	93.96%ID	E:0	Chlorophyll a-b binding protein 21, chloroplastic	PF00504.18^Chloroa_b-bind^Chlorophyll A-B binding protein^65-231^E:1.6e-50	0,971	0,008	0	-0,794	0	-1,31	0	-1,022	0,121	0,359	CB22_TOBAC	Chlorophyll a-b binding protein 21, chloroplastic (LHCII type I CAB-21) (LHCP)	CAB21	Nicotiana tabacum (Common tobacco)		Acetylation; Chlorophyll; Chloroplast; Chromophore; Magnesium; Membrane; Metal-binding; Phosphoprotein; Photosynthesis; Photosystem I; Photosystem II; Plastid; Thylakoid; Transit peptide; Transmembrane; Transmembrane helix
SRR342017_TR18707_c0_g1_i1_len=1596	CB23_TOBAC	Q:1136-342,H:1-265	92.45%ID	E:2e-178	Chlorophyll a-b binding protein 36, chloroplastic	PF00504.18^Chloroa_b-bind^Chlorophyll A-B binding protein^66-231^E:5.2e-50	0,546	-0,126	0	-0,817	0	-1,207	0	-0,981	0	0,8	CB23_TOBAC	Chlorophyll a-b binding protein 36, chloroplastic (LHCII type I CAB-36) (LHCP)	CAB36	Nicotiana tabacum (Common tobacco)		Acetylation; Chlorophyll; Chloroplast; Chromophore; Magnesium; Membrane; Metal-binding; Phosphoprotein; Photosynthesis; Photosystem I; Photosystem II; Plastid; Thylakoid; Transit peptide; Transmembrane; Transmembrane helix
SRR342017_TR25843_c0_g1_i1_len=542	GAST1_SOLLC	Q:285-494,H:43-112	84.29%ID	E:4e-37	Protein GAST1	PF02704.11^GASA^Gibberellin regulated protein^62-121^E:1.9e-22	0,022	-0,678	0	-2,666	0	-1,551	0	-1,351	0,002	1,112	GAST1_SOLLC	Protein GAST1	GAST1	Solanum lycopersicum (Tomato) (Lycopersicon esculentum)		Complete proteome; Disulfide bond; Reference proteome; Secreted; Signal
SRR342017_TR28272_c0_g1_i1_len=736	GASA6_ARATH	Q:594-346,H:21-101	75.9%ID	E:4e-37	Gibberellin-regulated protein 6	.	0,98	-0,009	0	-6,979	0	-4,513	0	-5,589	0,008	0,77	GASA6_ARATH	Gibberellin-regulated protein 6 (GAST1 protein homolog 6)	GASA6 At1g74670 F1M20.35	Arabidopsis thaliana (Mouse-ear cress)		Complete proteome; Disulfide bond; Gibberellin signaling pathway; Reference proteome; Secreted; Signal
SRR342017_TR28592_c0_g1_i2_len=768	NA	NA	NA	NA	NA	.	0,856	0,098	0	-1,632	0	-1,185	0	-1,579	0,159	0,56	.	.	.	.	.	.
SRR342017_TR33135_c1_g5_i1_len=902	PST2_PETHY	Q:520-200,H:1-105	54.63%ID	E:1e-25	Photosystem II 5 kDa protein, chloroplastic {ECO:0000303|PubMed:22700981}	.	0,595	-0,113	0	-1,085	0	-0,86	0	-0,736	0,535	0,131	PST2_PETHY	Photosystem II 5 kDa protein, chloroplastic		Petunia hybrida (Petunia)		Chloroplast; Direct protein sequencing; Disulfide bond; Membrane; Photosynthesis; Photosystem II; Plastid; Thylakoid; Transit peptide
SRR342017_TR33135_c1_g5_i2_len=521	NA	NA	NA	NA	NA	.	0,666	-0,094	0	-1,698	0	-0,87	0	-0,802	0,318	-0,212	.	.	.	.	.	.

SRR342019_TR1262_c0_g1_i1_len=411	NA	NA	NA	NA	NA	.	1	0,056	0	-3,196	0	-2,375	0	-1,935	0,094	1,107	.	.	.	.	.	.
SRR342019_TR1943_c7_g2_i6_len=1601	CAHC_TOBAC	Q:1496-510,H:1-321	77.27%ID	E:2e-157	Carbonic anhydrase, chloroplastic	PF00484.16^Pro_CA^Carbonic anhydrase^156-313^E:2.5e-39	0,285	-0,239	0	-0,957	0	-1,496	0	-1,577	0,018	-0,796	CAHC_TOBAC	Carbonic anhydrase, chloroplastic (EC 4.2.1.1) (Carbonate dehydratase)		Nicotiana tabacum (Common tobacco)		Chloroplast; Lyase; Plastid; Transit peptide; Zinc
SRR342019_TR17300_c1_g4_i2_len=668	PSAO_ARATH	Q:388-137,H:28-111	84.52%ID	E:3e-43	Photosystem I subunit O	.	0,805	0,065	0	-1,054	0	-1,073	0	-0,952	0,101	0,461	PSAO_ARATH	Photosystem I subunit O (PSI-O)	PSAO At1g08380 T27G7	Arabidopsis thaliana (Mouse-ear cress)		Chloroplast; Complete proteome; Direct protein sequencing; Membrane; Photosynthesis; Photosystem I; Plastid; Reference proteome; Thylakoid; Transit peptide; Transmembrane; Transmembrane helix
SRR342019_TR18410_c0_g8_i1_len=573	CB4A_SOLLC	Q:346-2,H:1-111	82.61%ID	E:2e-49	Chlorophyll a-b binding protein CP24 10A, chloroplastic	PF00504.18^Chloroa_b-bind^Chlorophyll A-B binding protein^72-115^E:2.4e-19	0,082	-0,428	0	-1,127	0	-1,532	0	-1,29	0,016	0,672	CB4A_SOLLC	Chlorophyll a-b binding protein CP24 10A, chloroplastic (CAB-10A) (LHCP)	CAP10A	Solanum lycopersicum (Tomato) (Lycopersicon esculentum)		Chlorophyll; Chloroplast; Chromophore; Complete proteome; Membrane; Photosynthesis; Photosystem II; Plastid; Reference proteome; Thylakoid; Transit peptide; Transmembrane; Transmembrane helix
SRR342019_TR20493_c2_g1_i1_len=2112	NA	NA	NA	NA	NA	.	0,352	0,313	0	-1,261	0,001	-1,022	0	-1,302	1	1,059	.	.	.	.	.	.
SRR342019_TR21154_c4_g3_i1_len=943	PB27A_ARATH	Q:638-246,H:41-174	70.37%ID	E:3e-58	Photosystem II repair protein PSB27-H1, chloroplastic	PF13326.3^PSII_Pbs27^Photosystem II Pbs27^42-171^E:5.7e-31	0,039	-0,505	0	-1,573	0	-0,958	0,001	-0,761	0,798	-0,47	PB27A_ARATH	Photosystem II repair protein PSB27-H1, chloroplastic (Psb27-H1) (Thylakoid lumenal protein PSB27-H1)	PSB27-1 At1g03600 F21B7.14 F21B7.21 F21B7.22 F21B7_140	Arabidopsis thaliana (Mouse-ear cress)		Chloroplast; Complete proteome; Direct protein sequencing; Membrane; Phosphoprotein; Plastid; Reference proteome; Thylakoid; Transit peptide
SRR342019_TR22648_c0_g2_i2_len=1741	DAAA_ARATH	Q:1164-265,H:73-373	59.47%ID	E:9e-123	D-amino-acid transaminase, chloroplastic {ECO:0000303|PubMed:18318836}	PF01063.16^Aminotran_4^Amino-transferase class IV^77-301^E:1.9e-40	0,002	-1,271	0	-2,057	0	-2,34	0	-1,979	1	0,097	DAAA_ARATH	D-amino-acid transaminase, chloroplastic (EC 2.6.1.21) (EC 4.1.3.38) (Aminodeoxychorismate lyase) (AtADCL) (Branched-chain-amino-acid aminotransferase-like protein 3)	DAAT BCAL3 At5g57850 MTI20.10	Arabidopsis thaliana (Mouse-ear cress)	PATHWAY: Cofactor biosynthesis; tetrahydrofolate biosynthesis; 4-aminobenzoate from chorismate: step 2/2.	Aminotransferase; Chloroplast; Complete proteome; Lyase; Plastid; Pyridoxal phosphate; Reference proteome; Transferase; Transit peptide
SRR342019_TR23916_c4_g1_i2_len=1152	CB22_TOBAC	Q:90-884,H:1-265	93.96%ID	E:0	Chlorophyll a-b binding protein 21, chloroplastic	PF00504.18^Chloroa_b-bind^Chlorophyll A-B binding protein^65-231^E:1.6e-50	0,531	-0,139	0	-2,022	0	-0,804	0	-0,937	0,447	0,157	CB22_TOBAC	Chlorophyll a-b binding protein 21, chloroplastic (LHCII type I CAB-21) (LHCP)	CAB21	Nicotiana tabacum (Common tobacco)		Acetylation; Chlorophyll; Chloroplast; Chromophore; Magnesium; Membrane; Metal-binding; Phosphoprotein; Photosynthesis; Photosystem I; Photosystem II; Plastid; Thylakoid; Transit peptide; Transmembrane; Transmembrane helix
SRR342019_TR23916_c4_g1_i3_len=1251	CB22_TOBAC	Q:90-884,H:1-265	93.96%ID	E:0	Chlorophyll a-b binding protein 21, chloroplastic	PF00504.18^Chloroa_b-bind^Chlorophyll A-B binding protein^65-231^E:1.6e-50	0,211	-0,257	0	-0,902	0	-0,967	0	-0,771	0,384	0,178	CB22_TOBAC	Chlorophyll a-b binding protein 21, chloroplastic (LHCII type I CAB-21) (LHCP)	CAB21	Nicotiana tabacum (Common tobacco)		Acetylation; Chlorophyll; Chloroplast; Chromophore; Magnesium; Membrane; Metal-binding; Phosphoprotein; Photosynthesis; Photosystem I; Photosystem II; Plastid; Thylakoid; Transit peptide; Transmembrane; Transmembrane helix
SRR342019_TR27542_c2_g1_i1_len=1526	CB5_ARATH	Q:460-1038,H:5-194	78.87%ID	E:1e-106	Chlorophyll a-b binding protein CP26, chloroplastic	PF00504.18^Chloroa_b-bind^Chlorophyll A-B binding protein^94-202^E:2.4e-24	1	0,004	0	-2,127	0	-1,339	0	-1,796	0,337	0,357	CB5_ARATH	Chlorophyll a-b binding protein CP26, chloroplastic (LHCB5) (LHCIIc) (Light-harvesting complex II protein 5)	LHCB5 At4g10340 F24G24.140	Arabidopsis thaliana (Mouse-ear cress)		Chlorophyll; Chloroplast; Chromophore; Complete proteome; Magnesium; Membrane; Metal-binding; Phosphoprotein; Photosynthesis; Photosystem I; Photosystem II; Plastid; Reference proteome; Thylakoid; Transit peptide; Transmembrane; Transmembrane helix
SRR342019_TR27542_c2_g1_i5_len=1425	CB5_ARATH	Q:460-1296,H:5-280	82.86%ID	E:7e-138	Chlorophyll a-b binding protein CP26, chloroplastic	PF00504.18^Chloroa_b-bind^Chlorophyll A-B binding protein^94-255^E:5.4e-46	0,983	0,005	0	-1,089	0	-0,929	0	-0,845	0,556	-0,123	CB5_ARATH	Chlorophyll a-b binding protein CP26, chloroplastic (LHCB5) (LHCIIc) (Light-harvesting complex II protein 5)	LHCB5 At4g10340 F24G24.140	Arabidopsis thaliana (Mouse-ear cress)		Chlorophyll; Chloroplast; Chromophore; Complete proteome; Magnesium; Membrane; Metal-binding; Phosphoprotein; Photosynthesis; Photosystem I; Photosystem II; Plastid; Reference proteome; Thylakoid; Transit peptide; Transmembrane; Transmembrane helix
SRR342019_TR27782_c0_g3_i1_len=1930	CHLP_TOBAC	Q:204-1580,H:1-460	87.83%ID	E:0	Geranylgeranyl diphosphate reductase, chloroplastic	PF01134.19^GIDA^Glucose inhibited division protein A^121-155^E:0.00011	0,09	-0,411	0	-1,771	0	-1,299	0	-0,937	0,025	0,495	CHLP_TOBAC	Geranylgeranyl diphosphate reductase, chloroplastic (EC 1.3.1.83) (Geranylgeranyl reductase)	CHLP	Nicotiana tabacum (Common tobacco)	PATHWAY: Porphyrin-containing compound metabolism; chlorophyll biosynthesis.; PATHWAY: Cofactor biosynthesis; tocopherol biosynthesis.	Chlorophyll biosynthesis; Chloroplast; NADP; Oxidoreductase; Photosynthesis; Plastid; Transit peptide
SRR342019_TR27808_c0_g1_i3_len=1457	CRR60_ARATH	Q:1218-526,H:35-259	62.77%ID	E:8e-80	Cysteine-rich repeat secretory protein 60	PF01657.14^Stress-antifung^Salt stress response/antifungal^31-125^E:5e-20`PF01657.14^Stress-antifung^Salt stress response/antifungal^174-231^E:9.5e-09	0,059	-0,623	0	-2,201	0	-1,674	0	-1,754	0,843	-0,102	CRR60_ARATH	Cysteine-rich repeat secretory protein 60 (Plasmodesmata-located protein 7) (PDLP7)	CRRSP60 At5g37660 K12B20.12	Arabidopsis thaliana (Mouse-ear cress)		Alternative splicing; Cell junction; Complete proteome; Disulfide bond; Host-virus interaction; Membrane; Reference proteome; Repeat; Signal; Transmembrane; Transmembrane helix; Transport
SRR342019_TR30724_c0_g2_i2_len=1607	PSAK_ARATH	Q:970-1275,H:29-130	87.25%ID	E:3e-41	Photosystem I reaction center subunit psaK, chloroplastic	.	0,922	0,071	0	-1,675	0	-1,454	0	-1,45	0,147	0,453	PSAK_ARATH	Photosystem I reaction center subunit psaK, chloroplastic (PSI-K) (Photosystem I subunit X)	PSAK At1g30380 T4K22.2	Arabidopsis thaliana (Mouse-ear cress)		Chloroplast; Complete proteome; Membrane; Photosynthesis; Photosystem I; Plastid; Reference proteome; Thylakoid; Transit peptide; Transmembrane; Transmembrane helix
SRR342019_TR31144_c1_g1_i2_len=775	FER_CAPAN	Q:119-556,H:1-144	73.29%ID	E:7e-60	Ferredoxin, chloroplastic	PF00111.24^Fer2^2Fe-2S iron-sulfur cluster binding domain^99-170^E:1.4e-19	0,051	-0,409	0	-1,084	0	-1,125	0	-1,055	0,233	-0,251	FER_CAPAN	Ferredoxin, chloroplastic (PFLP)	AP1	Capsicum annuum (Bell pepper)		2Fe-2S; Chloroplast; Electron transport; Iron; Iron-sulfur; Metal-binding; Phosphoprotein; Plastid; Transit peptide; Transport
SRR342019_TR37176_c0_g1_i3_len=1330	NA	NA	NA	NA	NA	PF14547.3^Hydrophob_seed^Hydrophobic seed protein^167-250^E:4.6e-27	0,019	-0,555	0	-2,672	0	-1,624	0	-1,2	0,92	-0,035	.	.	.	.	.	.
SRR342019_TR37354_c0_g1_i9_len=867	NA	NA	NA	NA	NA	.	0,729	0,208	0	-2,962	0	-2,914	0	-2,679	0,655	-0,237	.	.	.	.	.	.
SRR342022_TR2829_c2_g1_i1_len=2246	G3PB_PEA	Q:450-1874,H:10-451	81.47%ID	E:0	Glyceraldehyde-3-phosphate dehydrogenase B, chloroplastic	PF00044.21^Gp_dh_N^Glyceraldehyde 3-phosphate dehydrogenase, NAD binding domain^2-105^E:3.3e-33`PF02800.17^Gp_dh_C^Glyceraldehyde 3-phosphate dehydrogenase, C-terminal domain^110-267^E:1.1e-65`PF02672.12^CP12^CP12 domain^296-317^E:2.3e-05	0,543	-0,201	0	-1,039	0	-1,25	0,001	-0,734	0,285	0,263	G3PB_PEA	Glyceraldehyde-3-phosphate dehydrogenase B, chloroplastic (EC 1.2.1.13) (NADP-dependent glyceraldehydephosphate dehydrogenase subunit B)	GAPB GPB1	Pisum sativum (Garden pea)	PATHWAY: Carbohydrate biosynthesis; Calvin cycle.	Calvin cycle; Chloroplast; NADP; Oxidoreductase; Plastid; Transit peptide
SRR342022_TR4115_c1_g2_i2_len=2017	POR_DAUCA	Q:173-1366,H:1-398	79.45%ID	E:0	Protochlorophyllide reductase, chloroplastic	PF08659.7^KR^KR domain^86-189^E:9.7e-07`PF00106.22^adh_short^short chain dehydrogenase^88-230^E:3.4e-19`PF13561.3^adh_short_C2^Enoyl-(Acyl carrier protein) reductase^92-229^E:6.9e-11	0,019	-0,544	0	-1,645	0	-1,254	0	-0,841	1	-0,819	POR_DAUCA	Protochlorophyllide reductase, chloroplastic (PCR) (EC 1.3.1.33) (NADPH-protochlorophyllide oxidoreductase) (POR)	POR1	Daucus carota (Wild carrot)	PATHWAY: Porphyrin-containing compound metabolism; chlorophyll biosynthesis.	Chlorophyll biosynthesis; Chloroplast; NADP; Oxidoreductase; Photosynthesis; Plastid; Transit peptide
SRR342022_TR4209_c2_g2_i2_len=1887	PLY1_ARATH	Q:1052-1885,H:62-339	91.73%ID	E:0	Probable pectate lyase 1	PF00544.16^Pec_lyase_C^Pectate lyase^8-177^E:7e-22`PF13229.3^Beta_helix^Right handed beta helix region^27-146^E:3.7e-07	0,886	-0,062	0	-2,087	0	-2,443	0	-1,983	1	-0,996	PLY1_ARATH	Probable pectate lyase 1 (EC 4.2.2.2) (Pectate lyase A1)	At1g04680 T1G11.7 T1G11_6	Arabidopsis thaliana (Mouse-ear cress)	PATHWAY: Glycan metabolism; pectin degradation; 2-dehydro-3-deoxy-D-gluconate from pectin: step 2/5.	Calcium; Complete proteome; Glycoprotein; Lyase; Metal-binding; Reference proteome; Signal
SRR342022_TR7524_c6_g4_i8_len=1411	CB12_PETHY	Q:267-1037,H:1-268	60.45%ID	E:2e-105	Chlorophyll a-b binding protein, chloroplastic	PF00504.18^Chloroa_b-bind^Chlorophyll A-B binding protein^63-229^E:4.1e-50	0,05	-0,485	0	-1,423	0	-1,431	0	-1,281	0,282	-0,259	CB12_PETHY	Chlorophyll a-b binding protein, chloroplastic (LHCI type II CAB)		Petunia hybrida (Petunia)		Chlorophyll; Chloroplast; Chromophore; Magnesium; Membrane; Metal-binding; Phosphoprotein; Photosynthesis; Photosystem I; Photosystem II; Plastid; Thylakoid; Transit peptide; Transmembrane; Transmembrane helix
SRR342022_TR7773_c0_g1_i2_len=870	NA	NA	NA	NA	NA	.	0,175	-0,987	0,001	-2,473	0	-2,832	0	-2,874	1	-0,029	.	.	.	.	.	.
SRR342022_TR13446_c0_g2_i1_len=809	NA	NA	NA	NA	NA	.	0,875	0,064	0	-5,807	0,001	-2,644	0	-3,435	0,25	-0,393	.	.	.	.	.	.
SRR342022_TR16646_c3_g3_i4_len=1490	YAB1_ARATH	Q:970-353,H:21-229	65.12%ID	E:2e-81	Axial regulator YABBY 1	PF04690.10^YABBY^YABBY protein^32-195^E:7.7e-72	0,024	-0,721	0	-2,327	0	-1,883	0	-1,676	0,038	-0,679	YAB1_ARATH	Axial regulator YABBY 1 (FI-54) (Protein ABNORMAL FLORAL ORGANS) (Protein FILAMENTOUS FLOWER) (Protein antherless)	YAB1 AFO AT FIL At2g45190 F4L23.30	Arabidopsis thaliana (Mouse-ear cress)		Complete proteome; DNA-binding; Developmental protein; Metal-binding; Nucleus; Reference proteome; Zinc; Zinc-finger
SRR342022_TR18094_c3_g1_i1_len=617	NA	NA	NA	NA	NA	.	0,088	-0,595	0	-3,132	0	-1,447	0	-1,615	0,907	-0,04	.	.	.	.	.	.
SRR342022_TR18117_c1_g3_i1_len=1173	PDF1_ARATH	Q:698-1021,H:191-302	68.75%ID	E:2e-21	Protodermal factor 1	.	0,086	-0,512	0	-4,895	0	-2,809	0	-3,93	0,799	0,083	PDF1_ARATH	Protodermal factor 1	PDF1 At2g42840 F7D19.16	Arabidopsis thaliana (Mouse-ear cress)		Complete proteome; Reference proteome; Signal
SRR342022_TR18636_c4_g4_i3_len=2040	NA	NA	NA	NA	NA	.	0,089	-0,558	0	-1,545	0	-1,857	0	-1,913	1	0,251	.	.	.	.	.	.
SRR342022_TR20089_c0_g1_i1_len=1263	NA	NA	NA	NA	NA	.	0,932	-0,037	0	-2,302	0	-2,051	0	-1,369	1	0,219	.	.	.	.	.	.
SRR342022_TR20209_c3_g5_i2_len=2978	PP213_ARATH	Q:1168-2742,H:76-599	63.05%ID	E:0	Pentatricopeptide repeat-containing protein At3g04760, chloroplastic	PF01535.17^PPR^PPR repeat^71-94^E:0.25`PF13812.3^PPR_3^Pentatricopeptide repeat domain^87-144^E:0.0004`PF12854.4^PPR_1^PPR repeat^131-161^E:3.8e-09`PF13812.3^PPR_3^Pentatricopeptide repeat domain^131-180^E:3.5e-08`PF13041.3^PPR_2^PPR repeat family^132-181^E:4.2e-18`PF01535.17^PPR^PPR repeat^136-165^E:1.6e-06`PF13812.3^PPR_3^Pentatricopeptide repeat domain^157-213^E:7.1e-12`PF12854.4^PPR_1^PPR repeat^163-194^E:2.4e-10`PF13041.3^PPR_2^PPR repeat family^167-213^E:4.3e-15`PF01535.17^PPR^PPR repeat^170-200^E:4.3e-06`PF12854.4^PPR_1^PPR repeat^199-230^E:2.3e-06`PF01535.17^PPR^PPR repeat^205-235^E:0.00061`PF13041.3^PPR_2^PPR repeat family^218-251^E:1.2e-10`PF13812.3^PPR_3^Pentatricopeptide repeat domain^225-282^E:4.9e-10`PF12854.4^PPR_1^PPR repeat^233-261^E:2e-12`PF01535.17^PPR^PPR repeat^240-269^E:3.8e-06`PF13041.3^PPR_2^PPR repeat family^241-284^E:3.2e-12`PF13041.3^PPR_2^PPR repeat family^272-321^E:1e-13`PF01535.17^PPR^PPR repeat^275-302^E:0.0092`PF17177.1^PPR_long^Pentacotripeptide-repeat region of PROPR^291-416^E:2.9e-12`PF13812.3^PPR_3^Pentatricopeptide repeat domain^295-354^E:9.5e-08`PF12854.4^PPR_1^PPR repeat^304-335^E:1.8e-11`PF01535.17^PPR^PPR repeat^310-340^E:1.9e-07`PF13041.3^PPR_2^PPR repeat family^316-356^E:1.7e-09`PF13812.3^PPR_3^Pentatricopeptide repeat domain^332-391^E:4.2e-08`PF12854.4^PPR_1^PPR repeat^338-370^E:1.5e-09`PF01535.17^PPR^PPR repeat^346-375^E:0.00041`PF13812.3^PPR_3^Pentatricopeptide repeat domain^370-424^E:1.3e-10`PF12854.4^PPR_1^PPR repeat^374-404^E:1.3e-10`PF13041.3^PPR_2^PPR repeat family^377-424^E:3.8e-15`PF01535.17^PPR^PPR repeat^381-410^E:1.4e-06`PF13812.3^PPR_3^Pentatricopeptide repeat domain^401-458^E:8.1e-08`PF13041.3^PPR_2^PPR repeat family^412-461^E:4.7e-11`PF01535.17^PPR^PPR repeat^415-444^E:0.088`PF17177.1^PPR_long^Pentacotripeptide-repeat region of PROPR^437-526^E:6e-10`PF12854.4^PPR_1^PPR repeat^443-476^E:2.1e-14`PF13041.3^PPR_2^PPR repeat family^447-496^E:1.5e-15`PF01535.17^PPR^PPR repeat^450-480^E:2.1e-10`PF13812.3^PPR_3^Pentatricopeptide repeat domain^471-528^E:3e-09`PF12854.4^PPR_1^PPR repeat^479-511^E:1.5e-08`PF13041.3^PPR_2^PPR repeat family^483-528^E:1.2e-13`PF01535.17^PPR^PPR repeat^485-515^E:3.7e-07	0,125	-0,513	0,001	-1,302	0,001	-1,031	0,001	-1,016	0,089	0,574	PP213_ARATH	Pentatricopeptide repeat-containing protein At3g04760, chloroplastic	At3g04760 F7O18.25	Arabidopsis thaliana (Mouse-ear cress)		Chloroplast; Complete proteome; Plastid; Reference proteome; Repeat; Transit peptide
SRR342022_TR28986_c0_g2_i1_len=745	NA	NA	NA	NA	NA	PF14368.3^LTP_2^Probable lipid transfer^9-99^E:2.5e-11`PF00234.19^Tryp_alpha_amyl^Protease inhibitor/seed storage/LTP family^27-101^E:2.3e-07	0,003	1,147	0	-5,396	0,001	-1,085	0	-1,999	0,832	-0,254	.	.	.	.	.	.
SRR342022_TR32151_c0_g5_i4_len=749	NA	NA	NA	NA	NA	.	0,016	-0,546	0	-2,767	0	-2,51	0	-3,065	1	-0,927	.	.	.	.	.	.
SRR342023_TR161_c2_g2_i7_len=2055	RUS5_ARATH	Q:1612-362,H:80-504	64.32%ID	E:4e-136	Protein root UVB sensitive 5 {ECO:0000303|PubMed:19515790}	PF04884.11^DUF647^Vitamin B6 photo-protection and homoeostasis^44-282^E:7.3e-67	0,237	-0,471	0	-1,41	0	-1,718	0	-1,855	0,832	-0,258	RUS5_ARATH	Protein root UVB sensitive 5	RUS5 At5g01510 F7A7.30	Arabidopsis thaliana (Mouse-ear cress)		Complete proteome; Reference proteome
SRR342023_TR2846_c0_g5_i2_len=1227	ABP20_PRUPE	Q:258-782,H:28-202	69.89%ID	E:3e-72	Auxin-binding protein ABP20	PF00190.19^Cupin_1^Cupin^54-199^E:4.1e-32	0	1,428	0	-3,732	0	-2,648	0	-1,69	0,615	0,105	ABP20_PRUPE	Auxin-binding protein ABP20	ABP20	Prunus persica (Peach) (Amygdalus persica)		Apoplast; Auxin signaling pathway; Cell wall; Disulfide bond; Glycoprotein; Manganese; Metal-binding; Receptor; Secreted; Signal
SRR342023_TR7356_c3_g3_i3_len=1225	CB11_SOLLC	Q:907-200,H:1-236	86.02%ID	E:5e-135	Chlorophyll a-b binding protein 6A, chloroplastic	PF00504.18^Chloroa_b-bind^Chlorophyll A-B binding protein^56-210^E:3.5e-46	0,142	-0,323	0	-1,327	0	-1,12	0	-0,915	0,82	0,054	CB11_SOLLC	Chlorophyll a-b binding protein 6A, chloroplastic (LHCI type I CAB-6A) (Light-harvesting complex I 26 kDa protein)	CAB6A	Solanum lycopersicum (Tomato) (Lycopersicon esculentum)		Chlorophyll; Chloroplast; Chromophore; Complete proteome; Magnesium; Membrane; Metal-binding; Phosphoprotein; Photosynthesis; Photosystem I; Photosystem II; Plastid; Reference proteome; Thylakoid; Transit peptide; Transmembrane; Transmembrane helix
SRR342023_TR8133_c1_g4_i1_len=1447	PUMP3_ARATH	Q:183-521,H:15-129	54.7%ID	E:2e-27	Mitochondrial uncoupling protein 3	.	0,11	-0,533	0	-1,485	0	-1,686	0	-2,145	0,204	-0,423	PUMP3_ARATH	Mitochondrial uncoupling protein 3 (AtPUMP3)	PUMP3 UCP3 At1g14140 F7A19.22	Arabidopsis thaliana (Mouse-ear cress)		Complete proteome; Membrane; Mitochondrion; Mitochondrion inner membrane; Reference proteome; Repeat; Stress response; Transmembrane; Transmembrane helix; Transport
SRR342023_TR8775_c0_g2_i4_len=634	ARG7_VIGRR	Q:159-446,H:1-90	56.25%ID	E:4e-30	Indole-3-acetic acid-induced protein ARG7	.	0,099	-0,849	0	-3,356	0	-2,431	0	-2,01	0,003	-1,032	ARG7_VIGRR	Indole-3-acetic acid-induced protein ARG7	ARG7	Vigna radiata var. radiata (Mung bean) (Phaseolus aureus)		
SRR342023_TR9121_c7_g10_i2_len=1422	HIDM_GLYEC	Q:202-1140,H:16-327	45.34%ID	E:1e-87	2-hydroxyisoflavanone dehydratase	PF00135.25^COesterase^Carboxylesterase family^63-119^E:3.8e-06`PF07859.10^Abhydrolase_3^alpha/beta hydrolase fold^77-291^E:3.1e-33	0,008	-0,577	0	-2,663	0	-2,058	0	-1,793	0,126	0,339	HIDM_GLYEC	2-hydroxyisoflavanone dehydratase (EC 3.1.1.1) (EC 4.2.1.105) (Carboxylesterase HIDM)	HIDM	Glycyrrhiza echinata (Licorice)	PATHWAY: Secondary metabolite biosynthesis; flavonoid biosynthesis.	Flavonoid biosynthesis; Hydrolase; Lyase
SRR342023_TR10990_c0_g5_i2_len=981	CB12_SOLLC	Q:979-230,H:21-270	89.2%ID	E:5e-166	Chlorophyll a-b binding protein 7, chloroplastic	PF00504.18^Chloroa_b-bind^Chlorophyll A-B binding protein^54-220^E:7.8e-52	0,673	-0,089	0	-1,648	0	-0,951	0	-0,859	0,581	0,371	CB12_SOLLC	Chlorophyll a-b binding protein 7, chloroplastic (LHCI type II CAB-7)	CAB7	Solanum lycopersicum (Tomato) (Lycopersicon esculentum)		Chlorophyll; Chloroplast; Chromophore; Complete proteome; Magnesium; Membrane; Metal-binding; Phosphoprotein; Photosynthesis; Photosystem I; Photosystem II; Plastid; Reference proteome; Thylakoid; Transit peptide; Transmembrane; Transmembrane helix
SRR342023_TR12272_c0_g1_i1_len=1032	NA	NA	NA	NA	NA	.	0,01	-1,13	0	-3,367	0	-2,44	0	-2,466	0,193	-0,652	.	.	.	.	.	.
SRR342023_TR12898_c7_g3_i9_len=1759	PAP12_ARATH	Q:1428-394,H:62-407	68.5%ID	E:2e-173	Probable plastid-lipid-associated protein 12, chloroplastic	PF04755.9^PAP_fibrillin^PAP_fibrillin^85-259^E:9.3e-43	0,699	0,141	0	-1,273	0	-1,303	0	-1,097	0,443	0,287	PAP12_ARATH	Probable plastid-lipid-associated protein 12, chloroplastic (Fibrillin-10)	PAP12 FBN10 FIB10 At1g51110/At1g51115 F23H24.16/F23H24.17	Arabidopsis thaliana (Mouse-ear cress)		Chloroplast; Complete proteome; Plastid; Reference proteome; Thylakoid; Transit peptide
SRR342023_TR15719_c0_g2_i1_len=1696	CUT1A_ARATH	Q:647-303,H:50-164	73.04%ID	E:6e-51	Protein CURVATURE THYLAKOID 1A, chloroplastic	PF14159.3^CAAD^CAAD domains of cyanobacterial aminoacyl-tRNA synthetase^36-119^E:1.6e-32	0,374	-0,192	0	-0,84	0	-1,002	0	-0,928	0,6	0,135	CUT1A_ARATH	Protein CURVATURE THYLAKOID 1A, chloroplastic	CURT1A At4g01150 A_IG002N01.18 F2N1.18	Arabidopsis thaliana (Mouse-ear cress)		Acetylation; Alternative splicing; Chloroplast; Coiled coil; Complete proteome; Membrane; Plastid; Reference proteome; Thylakoid; Transit peptide; Transmembrane; Transmembrane helix
SRR342023_TR16454_c2_g4_i2_len=1411	GEDH1_OCIBA	Q:778-1404,H:33-244	57.55%ID	E:2e-72	Geraniol dehydrogenase 1	PF08240.9^ADH_N^Alcohol dehydrogenase GroES-like domain^5-92^E:9.8e-18`PF02826.16^2-Hacid_dh_C^D-isomer specific 2-hydroxyacid dehydrogenase, NAD binding domain^123-172^E:1e-06	0,521	-0,148	0	-1,065	0	-1,198	0	-1,328	0,089	0,363	GEDH1_OCIBA	Geraniol dehydrogenase 1 (ObaGEDH1) (EC 1.1.1.183)	GEDH1	Ocimum basilicum (Sweet basil)		Metal-binding; NADP; Oxidoreductase; Zinc
SRR342023_TR18007_c0_g1_i1_len=1626	SR43C_ARATH	Q:1169-291,H:83-369	67.01%ID	E:4e-134	Signal recognition particle 43 kDa protein, chloroplastic	PF12796.4^Ank_2^Ankyrin repeats (3 copies)^130-218^E:6.8e-10`PF13637.3^Ank_4^Ankyrin repeats (many copies)^165-196^E:6.5e-07`PF13857.3^Ank_5^Ankyrin repeats (many copies)^173-228^E:8.6e-12`PF13606.3^Ank_3^Ankyrin repeat^188-214^E:0.0049`PF00023.27^Ank^Ankyrin repeat^188-213^E:0.005`PF00385.21^Chromo^Chromo (CHRromatin Organisation MOdifier) domain^316-360^E:2.2e-09	0,084	-0,468	0	-1,323	0	-1,155	0	-1,177	0,482	0,197	SR43C_ARATH	Signal recognition particle 43 kDa protein, chloroplastic (Chromo protein SRP43) (CpSRP43)	CAO CPSRP43 At2g47450 T30B22.25	Arabidopsis thaliana (Mouse-ear cress)		3D-structure; ANK repeat; Chloroplast; Coiled coil; Complete proteome; Metal-binding; Plastid; Reference proteome; Repeat; Ribonucleoprotein; Signal recognition particle; Transit peptide
SRR342023_TR18091_c1_g1_i1_len=763	NA	NA	NA	NA	NA	.	0,004	-0,657	0	-2,205	0	-2,698	0	-2,35	1	-0,472	.	.	.	.	.	.
SRR342023_TR19865_c1_g1_i3_len=1788	NA	NA	NA	NA	NA	.	0,729	-0,209	0	-2,276	0	-2,29	0	-3,21	1	-0,211	.	.	.	.	.	.
SRR342023_TR19865_c1_g1_i7_len=1482	NA	NA	NA	NA	NA	PF00403.23^HMA^Heavy-metal-associated domain^36-92^E:5.1e-10`PF00403.23^HMA^Heavy-metal-associated domain^142-194^E:1.6e-12	0,009	-0,654	0	-2,328	0	-1,041	0	-1,157	1	0	.	.	.	.	.	.
SRR342023_TR20116_c0_g1_i1_len=534	NA	NA	NA	NA	NA	.	0,534	0,186	0	-1,758	0	-2,049	0	-2,412	0,001	1,176	.	.	.	.	.	.
SRR342023_TR20176_c2_g5_i1_len=761	CB4A_ARATH	Q:648-1,H:57-272	92.13%ID	E:4e-145	Chlorophyll a-b binding protein CP29.1, chloroplastic	PF00504.18^Chloroa_b-bind^Chlorophyll A-B binding protein^45-241^E:9.9e-50	0,863	-0,037	0	-1,15	0	-1,036	0	-0,932	0,794	0,056	CB4A_ARATH	Chlorophyll a-b binding protein CP29.1, chloroplastic (LHCB4.1) (LHCII protein 4.1)	LHCB4.1 At5g01530 F7A7_50	Arabidopsis thaliana (Mouse-ear cress)		Chlorophyll; Chloroplast; Chromophore; Complete proteome; Magnesium; Membrane; Metal-binding; Phosphoprotein; Photosynthesis; Photosystem I; Photosystem II; Plastid; Reference proteome; Thylakoid; Transit peptide; Transmembrane; Transmembrane helix
SRR342023_TR24219_c2_g1_i3_len=527	GDL79_ARATH	Q:3-266,H:275-362	79.55%ID	E:6e-48	GDSL esterase/lipase At5g33370	.	0,047	-0,43	0	-2,098	0	-1,424	0	-1,775	1	-0,031	GDL79_ARATH	GDSL esterase/lipase At5g33370 (EC 3.1.1.-) (Extracellular lipase At5g33370)	At5g33370 F19N2.90	Arabidopsis thaliana (Mouse-ear cress)		Alternative splicing; Complete proteome; Glycoprotein; Hydrolase; Lipid degradation; Lipid metabolism; Reference proteome; Secreted; Signal
SRR342023_TR27072_c0_g1_i1_len=2757	3MG1_ECOLI	Q:1717-1187,H:3-179	42.46%ID	E:7e-41	DNA-3-methyladenine glycosylase 1	PF03352.10^Adenine_glyco^Methyladenine glycosylase^219-390^E:2.7e-62	0,077	-0,468	0	-2,977	0	-1,764	0	-1,617	1	0,103	3MG1_ECOLI	DNA-3-methyladenine glycosylase 1 (EC 3.2.2.20) (3-methyladenine-DNA glycosylase I, constitutive) (TAG I) (DNA-3-methyladenine glycosidase I) (DNA-3-methyladenine glycosylase I)	tag b3549 JW3518	Escherichia coli (strain K12)		3D-structure; Complete proteome; DNA damage; DNA repair; Direct protein sequencing; Hydrolase; Metal-binding; Reference proteome; Zinc
SRR342023_TR28132_c0_g1_i2_len=646	NA	NA	NA	NA	NA	.	0,18	-0,805	0	-1,702	0	-2,787	0	-2,646	1	-2,774	.	.	.	.	.	.
SRR342023_TR28931_c0_g1_i1_len=696	NA	NA	NA	NA	NA	.	0,969	-0,021	0	-1,135	0,001	-1,04	0	-1,265	0,268	0,313	.	.	.	.	.	.
SRR646572_TR22428_c0_g1_i1_len=1365	FER_CAPAN	Q:352-789,H:1-144	73.29%ID	E:1e-57	Ferredoxin, chloroplastic	PF00111.24^Fer2^2Fe-2S iron-sulfur cluster binding domain^60-131^E:7.8e-20	0,026	-2,118	0	-4,129	0	-5,084	0	-3,752	1	-1,08	FER_CAPAN	Ferredoxin, chloroplastic (PFLP)	AP1	Capsicum annuum (Bell pepper)		2Fe-2S; Chloroplast; Electron transport; Iron; Iron-sulfur; Metal-binding; Phosphoprotein; Plastid; Transit peptide; Transport
SRR646596_TR7673_c0_g1_i2_len=4622	CHLH_ARATH	Q:4005-442,H:193-1381	89.57%ID	E:0	Magnesium-chelatase subunit ChlH, chloroplastic	PF02514.13^CobN-Mg_chel^CobN/Magnesium Chelatase^31-1148^E:0	0,165	-0,373	0	-1,62	0	-1,178	0	-0,902	0,047	0,465	CHLH_ARATH	Magnesium-chelatase subunit ChlH, chloroplastic (Mg-chelatase subunit H) (EC 6.6.1.1) (ABA-binding protein) (Mg-protoporphyrin IX chelatase subunit ChlH) (Protein CONDITIONAL CHLORINA) (Protein GENOMES UNCOUPLED 5) (Protein RAPID TRANSPIRATION IN DETACHED LEAVES 1)	CHLH ABAR CCH GUN5 RTL1 At5g13630 MSH12.9	Arabidopsis thaliana (Mouse-ear cress)	PATHWAY: Porphyrin-containing compound metabolism; chlorophyll biosynthesis.	ATP-binding; Acetylation; Alternative splicing; Chlorophyll biosynthesis; Chloroplast; Complete proteome; Ligase; Membrane; Nucleotide-binding; Photosynthesis; Plastid; Reference proteome; Transit peptide
SRR646596_TR9962_c1_g1_i1_len=679	PDF1_ARATH	Q:499-176,H:191-302	70.54%ID	E:2e-24	Protodermal factor 1	.	0,007	-0,668	0	-8,881	0	-2,43	0	-4,12	0,673	-0,11	PDF1_ARATH	Protodermal factor 1	PDF1 At2g42840 F7D19.16	Arabidopsis thaliana (Mouse-ear cress)		Complete proteome; Reference proteome; Signal
SRR646596_TR21625_c0_g2_i1_len=833	PSAO_ARATH	Q:304-654,H:24-140	86.32%ID	E:5e-67	Photosystem I subunit O	.	0,014	0,59	0	-1,359	0	-0,886	0	-0,933	0,931	0,027	PSAO_ARATH	Photosystem I subunit O (PSI-O)	PSAO At1g08380 T27G7	Arabidopsis thaliana (Mouse-ear cress)		Chloroplast; Complete proteome; Direct protein sequencing; Membrane; Photosynthesis; Photosystem I; Plastid; Reference proteome; Thylakoid; Transit peptide; Transmembrane; Transmembrane helix
SRR646596_TR23255_c2_g1_i1_len=683	PQL1_ARATH	Q:104-484,H:63-189	65.35%ID	E:8e-57	.	PF05757.8^PsbQ^Oxygen evolving enhancer protein 3 (PsbQ)^38-158^E:2.4e-27	0,2	-0,667	0	-1,972	0	-2,58	0	-2,127	0,005	-0,58	PNSL2_ARATH	Photosynthetic NDH subunit of lumenal location 2, chloroplastic (PsbQ-like protein 1)	PNSL2 PQL1 PQL2 At1g14150 F7A19.23	Arabidopsis thaliana (Mouse-ear cress)		Alternative splicing; Chloroplast; Coiled coil; Complete proteome; Direct protein sequencing; Membrane; Plastid; Reference proteome; Thylakoid; Transit peptide; Transport
SRR646596_TR31173_c0_g1_i2_len=682	NA	NA	NA	NA	NA	.	0,044	0,453	0,001	-0,728	0	-1,088	0	-0,837	0,275	-0,244	.	.	.	.	.	.
SRR646604_TR8465_c0_g2_i3_len=1255	CB11_SOLLC	Q:368-1075,H:1-236	86.02%ID	E:7e-135	Chlorophyll a-b binding protein 6A, chloroplastic	PF00504.18^Chloroa_b-bind^Chlorophyll A-B binding protein^56-210^E:3.5e-46	0,684	-0,086	0	-1,495	0	-0,929	0	-0,852	0,081	0,368	CB11_SOLLC	Chlorophyll a-b binding protein 6A, chloroplastic (LHCI type I CAB-6A) (Light-harvesting complex I 26 kDa protein)	CAB6A	Solanum lycopersicum (Tomato) (Lycopersicon esculentum)		Chlorophyll; Chloroplast; Chromophore; Complete proteome; Magnesium; Membrane; Metal-binding; Phosphoprotein; Photosynthesis; Photosystem I; Photosystem II; Plastid; Reference proteome; Thylakoid; Transit peptide; Transmembrane; Transmembrane helix
SRR646604_TR16238_c0_g1_i2_len=1070	CB4A_SOLLC	Q:782-237,H:75-256	92.31%ID	E:4e-109	Chlorophyll a-b binding protein CP24 10A, chloroplastic	PF00504.18^Chloroa_b-bind^Chlorophyll A-B binding protein^1-137^E:3.3e-22	0,587	-0,279	0	-2,517	0	-1,705	0	-2,122	0,58	0,27	CB4A_SOLLC	Chlorophyll a-b binding protein CP24 10A, chloroplastic (CAB-10A) (LHCP)	CAP10A	Solanum lycopersicum (Tomato) (Lycopersicon esculentum)		Chlorophyll; Chloroplast; Chromophore; Complete proteome; Membrane; Photosynthesis; Photosystem II; Plastid; Reference proteome; Thylakoid; Transit peptide; Transmembrane; Transmembrane helix
SRR646604_TR19153_c0_g2_i1_len=1168	CAPP1_ARATH	Q:3-926,H:663-967	86.36%ID	E:0	Phosphoenolpyruvate carboxylase 1	PF00311.14^PEPcase^Phosphoenolpyruvate carboxylase^2-308^E:3.7e-109	0,671	0,171	0	-1,482	0	-1,435	0	-1,159	0,096	-0,54	CAPP1_ARATH	Phosphoenolpyruvate carboxylase 1 (AtPPC1) (PEPC 1) (PEPCase 1) (EC 4.1.1.31) (107-kDa PEPC polypeptide)	PPC1 p107 At1g53310 F12M16.21	Arabidopsis thaliana (Mouse-ear cress)		Allosteric enzyme; Carbon dioxide fixation; Complete proteome; Cytoplasm; Lyase; Magnesium; Manganese; Phosphoprotein; Photosynthesis; Reference proteome; Ubl conjugation
SRR646604_TR28714_c0_g1_i2_len=945	CB23_TOBAC	Q:45-734,H:38-265	92.17%ID	E:1e-151	Chlorophyll a-b binding protein 36, chloroplastic	PF00504.18^Chloroa_b-bind^Chlorophyll A-B binding protein^1-126^E:4e-30	0,099	-0,347	0	-1,9	0	-1,076	0	-0,855	0	1,744	CB23_TOBAC	Chlorophyll a-b binding protein 36, chloroplastic (LHCII type I CAB-36) (LHCP)	CAB36	Nicotiana tabacum (Common tobacco)		Acetylation; Chlorophyll; Chloroplast; Chromophore; Magnesium; Membrane; Metal-binding; Phosphoprotein; Photosynthesis; Photosystem I; Photosystem II; Plastid; Thylakoid; Transit peptide; Transmembrane; Transmembrane helix
SRR646604_TR33658_c0_g1_i1_len=1118	TL16_ARATH	Q:618-265,H:99-216	70.34%ID	E:8e-47	Thylakoid lumenal 16.5 kDa protein, chloroplastic	.	0,015	-0,661	0	-1,965	0	-1,17	0	-0,855	0,866	-0,086	TL16_ARATH	Thylakoid lumenal 16.5 kDa protein, chloroplastic	At4g02530 T10P11.17	Arabidopsis thaliana (Mouse-ear cress)		Chloroplast; Complete proteome; Direct protein sequencing; Plastid; Reference proteome; Thylakoid; Transit peptide
SRR646604_TR36319_c0_g1_i1_len=1605	CAS_ARATH	Q:265-1365,H:15-386	70.16%ID	E:5e-157	Calcium sensing receptor, chloroplastic	.	0,304	-0,273	0	-1,239	0	-1,038	0	-0,868	0,593	0,125	CAS_ARATH	Calcium sensing receptor, chloroplastic (Sulfurtransferase 3) (AtStr3)	CAS STR3 At5g23060 MYJ24.5	Arabidopsis thaliana (Mouse-ear cress)		Chloroplast; Complete proteome; Membrane; Phosphoprotein; Plastid; Receptor; Reference proteome; Thylakoid; Transit peptide; Transmembrane; Transmembrane helix
SRR648705_TR1714_c1_g1_i4_len=1624	ILR3_ARATH	Q:1433-1005,H:90-234	60%ID	E:4e-48	Transcription factor ILR3	.	0,86	-0,127	0	-1,743	0	-1,177	0	-1,188	1	-0,779	ILR3_ARATH	Transcription factor ILR3 (Basic helix-loop-helix protein 105) (AtbHLH105) (bHLH 105) (Protein IAA-LEUCINE RESISTANT 3) (Transcription factor EN 133) (bHLH transcription factor bHLH105)	ILR3 BHLH105 EN133 At5g54680 K5F14.2	Arabidopsis thaliana (Mouse-ear cress)		Complete proteome; DNA-binding; Nucleus; Reference proteome; Transcription; Transcription regulation
SRR648705_TR13676_c0_g1_i1_len=1034	PSBQ2_ARATH	Q:851-159,H:1-230	70.26%ID	E:3e-69	Oxygen-evolving enhancer protein 3-2, chloroplastic	PF05757.8^PsbQ^Oxygen evolving enhancer protein 3 (PsbQ)^39-231^E:7.5e-79	0,692	-0,085	0	-0,975	0	-0,939	0	-0,745	0,337	0,326	PSBQ2_ARATH	Oxygen-evolving enhancer protein 3-2, chloroplastic (OEE3) (16 kDa subunit of oxygen evolving system of photosystem II) (OEC 16 kDa subunit)	PSBQ2 PSBQB At4g05180 C17L7.100	Arabidopsis thaliana (Mouse-ear cress)		Chloroplast; Complete proteome; Direct protein sequencing; Membrane; Phosphoprotein; Photosynthesis; Photosystem II; Plastid; Reference proteome; Thylakoid; Transit peptide
SRR648705_TR16311_c0_g1_i1_len=991	NA	NA	NA	NA	NA	.	0,158	-0,319	0	-2,605	0	-1,457	0	-1,37	0,739	-0,079	.	.	.	.	.	.
SRR648705_TR16543_c0_g1_i1_len=1355	GUN4C_ARATH	Q:796-239,H:82-265	63.49%ID	E:4e-71	Tetrapyrrole-binding protein, chloroplastic	PF05419.9^GUN4^GUN4-like^79-227^E:1.4e-50	0,151	-0,362	0	-1,206	0	-1,155	0	-1,003	0,569	-0,147	GUN4C_ARATH	Tetrapyrrole-binding protein, chloroplastic (Genomes uncoupled 4)	GUN4 At3g59400 F25L23_260	Arabidopsis thaliana (Mouse-ear cress)		Chlorophyll biosynthesis; Chloroplast; Complete proteome; Membrane; Plastid; Reference proteome; Transit peptide
SRR648705_TR26881_c0_g2_i1_len=1051	FLA6_ARATH	Q:164-625,H:37-190	63.64%ID	E:6e-52	Fasciclin-like arabinogalactan protein 6	PF02469.19^Fasciclin^Fasciclin domain^50-186^E:3.1e-20	0,002	-1,006	0	-2,46	0	-2,264	0	-1,248	0,002	-0,944	FLA6_ARATH	Fasciclin-like arabinogalactan protein 6	FLA6 At2g20520 T13C7.11	Arabidopsis thaliana (Mouse-ear cress)		Cell membrane; Complete proteome; GPI-anchor; Glycoprotein; Lipoprotein; Membrane; Proteoglycan; Reference proteome; Signal
SRR648705_TR39029_c0_g1_i1_len=2241	GCST_SOLTU	Q:566-1783,H:1-406	91.38%ID	E:0	Aminomethyltransferase, mitochondrial	PF01571.18^GCV_T^Aminomethyltransferase folate-binding domain^40-295^E:1.3e-87`PF08669.8^GCV_T_C^Glycine cleavage T-protein C-terminal barrel domain^304-396^E:5.2e-22	0,317	-0,24	0	-1,017	0	-0,998	0,001	-0,763	0,746	-0,076	GCST_SOLTU	Aminomethyltransferase, mitochondrial (EC 2.1.2.10) (Glycine cleavage system T protein) (GCVT)	GDCST	Solanum tuberosum (Potato)		Aminotransferase; Complete proteome; Mitochondrion; Reference proteome; Transferase; Transit peptide
SRR648705_TR43496_c0_g1_i1_len=1098	CB4A_SOLLC	Q:226-1005,H:1-256	87.31%ID	E:1e-143	Chlorophyll a-b binding protein CP24 10A, chloroplastic	PF00504.18^Chloroa_b-bind^Chlorophyll A-B binding protein^72-248^E:1.6e-46	0,191	-0,273	0	-1,97	0	-1,417	0	-1,252	0,017	0,493	CB4A_SOLLC	Chlorophyll a-b binding protein CP24 10A, chloroplastic (CAB-10A) (LHCP)	CAP10A	Solanum lycopersicum (Tomato) (Lycopersicon esculentum)		Chlorophyll; Chloroplast; Chromophore; Complete proteome; Membrane; Photosynthesis; Photosystem II; Plastid; Reference proteome; Thylakoid; Transit peptide; Transmembrane; Transmembrane helix
SRR648707_TR3137_c0_g1_i1_len=1128	CB23_SOLLC	Q:879-136,H:18-265	93.95%ID	E:4e-171	Chlorophyll a-b binding protein 13, chloroplastic	PF00504.18^Chloroa_b-bind^Chlorophyll A-B binding protein^130-297^E:5.3e-50	0,262	-0,242	0	-0,858	0	-1	0	-0,777	1	2,59	CB23_SOLLC	Chlorophyll a-b binding protein 13, chloroplastic (LHCII type III CAB-13)	CAB13 LHBC1	Solanum lycopersicum (Tomato) (Lycopersicon esculentum)		Chlorophyll; Chloroplast; Chromophore; Complete proteome; Direct protein sequencing; Magnesium; Membrane; Metal-binding; Phosphoprotein; Photosynthesis; Photosystem I; Photosystem II; Plastid; Reference proteome; Thylakoid; Transit peptide; Transmembrane; Transmembrane helix
SRR648707_TR13974_c0_g3_i1_len=1132	PR1_MEDTR	Q:744-340,H:39-173	68.89%ID	E:7e-64	Pathogenesis-related protein PR-1	PF00188.23^CAP^Cysteine-rich secretory protein family^46-162^E:1.9e-19	0,193	-0,694	0	-3,509	0	-1,426	0,001	-1,268	0,463	-0,269	PR1_MEDTR	Pathogenesis-related protein PR-1	PR-1	Medicago truncatula (Barrel medic) (Medicago tribuloides)		Disulfide bond; Pathogenesis-related protein; Plant defense; Signal
SRR648707_TR22833_c0_g1_i1_len=615	NA	NA	NA	NA	NA	.	0,398	-0,416	0	-2,605	0	-3,021	0	-3,038	0,021	-0,945	.	.	.	.	.	.
SRR924147_TR30328_c1_g1_i1_len=1044	PSBQ2_ARATH	Q:97-789,H:1-230	70.26%ID	E:2e-69	Oxygen-evolving enhancer protein 3-2, chloroplastic	PF05757.8^PsbQ^Oxygen evolving enhancer protein 3 (PsbQ)^71-263^E:1.2e-78	0,316	-0,214	0	-0,781	0	-0,945	0	-0,779	0,117	0,328	PSBQ2_ARATH	Oxygen-evolving enhancer protein 3-2, chloroplastic (OEE3) (16 kDa subunit of oxygen evolving system of photosystem II) (OEC 16 kDa subunit)	PSBQ2 PSBQB At4g05180 C17L7.100	Arabidopsis thaliana (Mouse-ear cress)		Chloroplast; Complete proteome; Direct protein sequencing; Membrane; Phosphoprotein; Photosynthesis; Photosystem II; Plastid; Reference proteome; Thylakoid; Transit peptide
SRR924148_TR14170_c0_g1_i1_len=1238	CB23_TOBAC	Q:218-1012,H:1-265	92.45%ID	E:3e-180	Chlorophyll a-b binding protein 36, chloroplastic	PF00504.18^Chloroa_b-bind^Chlorophyll A-B binding protein^66-231^E:5.2e-50	0,418	0,177	0	-0,84	0	-1,104	0	-1,02	0,179	-0,276	CB23_TOBAC	Chlorophyll a-b binding protein 36, chloroplastic (LHCII type I CAB-36) (LHCP)	CAB36	Nicotiana tabacum (Common tobacco)		Acetylation; Chlorophyll; Chloroplast; Chromophore; Magnesium; Membrane; Metal-binding; Phosphoprotein; Photosynthesis; Photosystem I; Photosystem II; Plastid; Thylakoid; Transit peptide; Transmembrane; Transmembrane helix
SRR924148_TR39337_c0_g1_i1_len=1503	ATPD_TOBAC	Q:1091-339,H:1-247	73.71%ID	E:7e-126	ATP synthase delta chain, chloroplastic	PF00213.15^OSCP^ATP synthase delta (OSCP) subunit^71-243^E:2.3e-43	0,418	-0,19	0	-0,881	0	-0,985	0	-0,778	1	-0,101	ATPD_TOBAC	ATP synthase delta chain, chloroplastic (F-ATPase delta chain)	ATPD	Nicotiana tabacum (Common tobacco)		ATP synthesis; CF(1); Chloroplast; Hydrogen ion transport; Ion transport; Membrane; Plastid; Thylakoid; Transit peptide; Transport
SRR122238_TR31153_c0_g1_i1_len=618	SNAK1_SOLTU	Q:141-353,H:18-88	71.83%ID	E:6e-29	Snakin-1	.	0	-1,101	0	-8,822	0	-2,718	0	-3,092	0,033	-0,544	SNAK1_SOLTU	Snakin-1	SN1	Solanum tuberosum (Potato)		Antimicrobial; Cell wall; Complete proteome; Direct protein sequencing; Disulfide bond; Plant defense; Reference proteome; Secreted; Signal
SRR342017_TR11290_c1_g1_i1_len=917	ABP20_PRUPE	Q:739-215,H:28-202	69.89%ID	E:3e-74	Auxin-binding protein ABP20	PF00190.19^Cupin_1^Cupin^90-235^E:6.5e-32	0	-2,411	0	-2,846	0	-3,08	0	-2,058	0,812	-0,068	ABP20_PRUPE	Auxin-binding protein ABP20	ABP20	Prunus persica (Peach) (Amygdalus persica)		Apoplast; Auxin signaling pathway; Cell wall; Disulfide bond; Glycoprotein; Manganese; Metal-binding; Receptor; Secreted; Signal
SRR342017_TR28944_c1_g1_i3_len=946	ACP1_CASGL	Q:659-246,H:1-135	55.71%ID	E:8e-29	Acyl carrier protein 1, chloroplastic	PF00550.22^PP-binding^Phosphopantetheine attachment site^63-130^E:7.4e-13	0	-1,148	0	-3,21	0	-1,93	0	-1,42	0,214	-0,449	ACP1_CASGL	Acyl carrier protein 1, chloroplastic (ACP 1)	ACP1	Casuarina glauca (Swamp oak)	PATHWAY: Lipid metabolism; fatty acid biosynthesis.	Chloroplast; Fatty acid biosynthesis; Fatty acid metabolism; Lipid biosynthesis; Lipid metabolism; Phosphopantetheine; Phosphoprotein; Plastid; Transit peptide
SRR342022_TR25175_c0_g1_i1_len=724	NA	NA	NA	NA	NA	.	0	-0,925	0	-4,596	0	-2,64	0	-2,33	0,143	-0,316	.	.	.	.	.	.
SRR342023_TR7730_c4_g1_i9_len=2380	AB19A_PRUPE	Q:853-284,H:19-208	73.16%ID	E:2e-89	Auxin-binding protein ABP19a	PF00190.19^Cupin_1^Cupin^59-197^E:2.5e-33`PF07883.8^Cupin_2^Cupin domain^92-163^E:1.6e-08	0,001	-0,878	0	-2,986	0	-1,684	0	-1,107	0,377	-0,68	AB19A_PRUPE	Auxin-binding protein ABP19a	ABP19A	Prunus persica (Peach) (Amygdalus persica)		Apoplast; Auxin signaling pathway; Cell wall; Disulfide bond; Glycoprotein; Manganese; Metal-binding; Receptor; Secreted; Signal
SRR122238_TR26719_c0_g1_i1_len=1857	AB19A_PRUPE	Q:983-1552,H:19-208	73.16%ID	E:4e-91	Auxin-binding protein ABP19a	PF00190.19^Cupin_1^Cupin^101-239^E:4.2e-33`PF07883.8^Cupin_2^Cupin domain^134-205^E:2.3e-08	0,004	-0,76	0	-3,639	0	-1,593	0	-1,119	0,001	-0,733	AB19A_PRUPE	Auxin-binding protein ABP19a	ABP19A	Prunus persica (Peach) (Amygdalus persica)		Apoplast; Auxin signaling pathway; Cell wall; Disulfide bond; Glycoprotein; Manganese; Metal-binding; Receptor; Secreted; Signal
SRR342017_TR9584_c0_g1_i1_len=1102	TIP21_ARATH	Q:176-913,H:1-247	82.19%ID	E:4e-92	Aquaporin TIP2-1	PF00230.17^MIP^Major intrinsic protein^16-232^E:1.7e-73	0,168	-0,424	0	-1,861	0	-1,93	0	-2,173	0	-10,205	TIP21_ARATH	Aquaporin TIP2-1 (Delta-tonoplast intrinsic protein) (Delta-TIP) (Tonoplast intrinsic protein 2-1) (AtTIP2;1) [Cleaved into: Aquaporin TIP2-1, N-terminally processed]	TIP2-1 At3g16240 MYA6.10	Arabidopsis thaliana (Mouse-ear cress)		Acetylation; Complete proteome; Membrane; Reference proteome; Repeat; Transmembrane; Transmembrane helix; Transport; Vacuole
SRR342017_TR22430_c1_g1_i15_len=965	RL291_ARATH	Q:139-321,H:1-61	81.97%ID	E:6e-26	60S ribosomal protein L29-1	.	1	-0,235	0	-3,62	0	-5,244	0	-6,24	0	-1,599	RL291_ARATH	60S ribosomal protein L29-1	RPL29A At3g06700 F3E22.16 T8E24.8	Arabidopsis thaliana (Mouse-ear cress)		Complete proteome; Phosphoprotein; Reference proteome; Ribonucleoprotein; Ribosomal protein
SRR342019_TR7401_c0_g1_i1_len=768	CB23_TOBAC	Q:643-17,H:1-209	89.95%ID	E:8e-137	Chlorophyll a-b binding protein 36, chloroplastic	PF00504.18^Chloroa_b-bind^Chlorophyll A-B binding protein^66-208^E:1e-34	0,077	-0,368	0	-0,792	0	-1,742	0	-1,131	0	-5,702	CB23_TOBAC	Chlorophyll a-b binding protein 36, chloroplastic (LHCII type I CAB-36) (LHCP)	CAB36	Nicotiana tabacum (Common tobacco)		Acetylation; Chlorophyll; Chloroplast; Chromophore; Magnesium; Membrane; Metal-binding; Phosphoprotein; Photosynthesis; Photosystem I; Photosystem II; Plastid; Thylakoid; Transit peptide; Transmembrane; Transmembrane helix
SRR342019_TR37176_c0_g1_i1_len=1844	NA	NA	NA	NA	NA	PF14547.3^Hydrophob_seed^Hydrophobic seed protein^167-250^E:4.6e-27	0,176	-0,479	0	-4,167	0	-2,11	0	-2,696	0	-1,187	.	.	.	.	.	.
SRR342019_TR37176_c0_g1_i2_len=1295	NA	NA	NA	NA	NA	PF14547.3^Hydrophob_seed^Hydrophobic seed protein^167-250^E:4.6e-27	0,01	-0,549	0	-3,626	0	-1,196	0	-1,139	0	-1,363	.	.	.	.	.	.
SRR122236_TR10225_c1_g1_i1_len=772	H32_WHEAT	Q:660-253,H:1-136	99.26%ID	E:6e-77	Histone H3.2	PF00125.21^Histone^Core histone H2A/H2B/H3/H4^38-169^E:2e-54	0	-1,557	0	-3,262	1	-0,031	0,124	-1,026	0,196	-0,791	H32_WHEAT	Histone H3.2		Triticum aestivum (Wheat)		Acetylation; Chromosome; Complete proteome; DNA-binding; Direct protein sequencing; Methylation; Nucleosome core; Nucleus; Phosphoprotein; Reference proteome
SRR122238_TR7106_c0_g1_i14_len=886	SERK1_ARATH	Q:119-709,H:6-204	62.31%ID	E:5e-61	Somatic embryogenesis receptor kinase 1	PF08263.9^LRRNT_2^Leucine rich repeat N-terminal domain^21-60^E:1.3e-11`PF13855.3^LRR_8^Leucine rich repeat^88-147^E:4.2e-07`PF00560.30^LRR_1^Leucine Rich Repeat^112-134^E:0.23`PF13855.3^LRR_8^Leucine rich repeat^113-171^E:8.3e-07`PF12799.4^LRR_4^Leucine Rich repeats (2 copies)^136-171^E:9.7e-07`PF00560.30^LRR_1^Leucine Rich Repeat^136-157^E:1`PF13516.3^LRR_6^Leucine Rich repeat^159-172^E:1.8`PF00560.30^LRR_1^Leucine Rich Repeat^160-176^E:0.066	0	-1,111	0	-3,993	0,019	-1,043	0,454	-0,327	0,051	-0,555	SERK1_ARATH	Somatic embryogenesis receptor kinase 1 (AtSERK1) (EC 2.7.10.1) (EC 2.7.11.1) (Somatic embryogenesis receptor-like kinase 1)	SERK1 At1g71830 F14O23.21 F14O23_24	Arabidopsis thaliana (Mouse-ear cress)		3D-structure; ATP-binding; Cell membrane; Complete proteome; Endoplasmic reticulum; Glycoprotein; Kinase; Leucine-rich repeat; Membrane; Nucleotide-binding; Phosphoprotein; Receptor; Reference proteome; Repeat; Serine/threonine-protein kinase; Signal; Transferase; Transmembrane; Transmembrane helix; Tyrosine-protein kinase
SRR122238_TR15261_c0_g2_i1_len=576	NA	NA	NA	NA	NA	.	0	-1,262	0	-8,669	0,017	-2,263	0,001	-3,516	0,233	-0,738	.	.	.	.	.	.
SRR122238_TR22981_c1_g1_i3_len=901	RS242_ARATH	Q:798-433,H:1-122	90.98%ID	E:1e-74	40S ribosomal protein S24-2	PF01282.16^Ribosomal_S24e^Ribosomal protein S24e^25-103^E:2.6e-38	0	-2,417	0	-1,529	0,675	-0,289	0,57	-0,305	0,502	4,3	RS242_ARATH	40S ribosomal protein S24-2	RPS24B At5g28060 F15F15.130	Arabidopsis thaliana (Mouse-ear cress)		Complete proteome; Reference proteome; Ribonucleoprotein; Ribosomal protein
SRR122238_TR38252_c0_g1_i2_len=898	H2AV1_ORYSJ	Q:627-301,H:30-138	97.25%ID	E:5e-68	Probable histone H2A variant 1	PF00125.21^Histone^Core histone H2A/H2B/H3/H4^16-106^E:5.3e-21`PF16211.2^Histone_H2A_C^C-terminus of histone H2A^107-136^E:7.2e-11	0	-1,11	0	-3,617	1	-0,252	0,781	-0,53	0,005	-0,726	H2AV1_ORYSJ	Probable histone H2A variant 1	Os03g0162200 LOC_Os03g06670 OJ1607A12.10	Oryza sativa subsp. japonica (Rice)		Chromosome; Complete proteome; DNA-binding; Nucleosome core; Nucleus; Reference proteome
SRR122238_TR61213_c1_g1_i1_len=887	H2A1_SOLLC	Q:403-660,H:56-146	75.82%ID	E:5e-35	Histone H2A.1	.	0	-1,616	0	-3,009	0,62	-0,53	0,282	-0,997	0,053	-0,503	H2A1_SOLLC	Histone H2A.1 (LeH2A-1)		Solanum lycopersicum (Tomato) (Lycopersicon esculentum)		Acetylation; Chromosome; Complete proteome; DNA-binding; Nucleosome core; Nucleus; Reference proteome
SRR122238_TR65198_c0_g1_i1_len=590	RLA1_MAIZE	Q:510-184,H:1-109	74.31%ID	E:2e-23	60S acidic ribosomal protein P1	PF00428.16^Ribosomal_60s^60s Acidic ribosomal protein^47-134^E:5e-27	0	-0,947	0	-0,949	0,022	0,707	0,493	0,231	1	-0,134	RLA1_MAIZE	60S acidic ribosomal protein P1 (L12)	RPP1A	Zea mays (Maize)		Complete proteome; Direct protein sequencing; Phosphoprotein; Reference proteome; Ribonucleoprotein; Ribosomal protein
SRR122238_TR68839_c0_g1_i3_len=1094	H2AX_CICAR	Q:982-560,H:1-139	84.51%ID	E:1e-62	Histone H2AX	PF00125.21^Histone^Core histone H2A/H2B/H3/H4^55-133^E:2.7e-13`PF00808.20^CBFD_NFYB_HMF^Histone-like transcription factor (CBF/NF-Y) and archaeal histone^69-132^E:0.00012`PF16211.2^Histone_H2A_C^C-terminus of histone H2A^136-169^E:3.1e-19	0	-1,508	0	-3,495	1	0,279	0,56	-0,64	0,001	-0,938	H2AX_CICAR	Histone H2AX	HIS2A	Cicer arietinum (Chickpea) (Garbanzo)		Chromosome; DNA-binding; Nucleosome core; Nucleus; Phosphoprotein
SRR122238_TR68839_c0_g1_i4_len=1169	H2AX_CICAR	Q:1057-560,H:1-139	72.46%ID	E:4e-69	Histone H2AX	.	0	-1,451	0	-2,553	0,76	-0,29	0,517	-0,955	0,008	-0,743	H2AX_CICAR	Histone H2AX	HIS2A	Cicer arietinum (Chickpea) (Garbanzo)		Chromosome; DNA-binding; Nucleosome core; Nucleus; Phosphoprotein
SRR122238_TR72645_c1_g4_i5_len=974	H2A1_MEDTR	Q:379-702,H:26-132	93.52%ID	E:1e-48	Probable histone H2A.1	PF00125.21^Histone^Core histone H2A/H2B/H3/H4^20-97^E:6.8e-13`PF16211.2^Histone_H2A_C^C-terminus of histone H2A^100-132^E:2.7e-18	0	-0,989	0	-1,492	0,458	0,292	0,936	-0,049	0,017	-0,574	H2A1_MEDTR	Probable histone H2A.1	MtrDRAFT_AC149210g1v1	Medicago truncatula (Barrel medic) (Medicago tribuloides)		Chromosome; DNA-binding; Nucleosome core; Nucleus
SRR122238_TR73275_c0_g1_i2_len=1098	GDL79_ARATH	Q:1094-294,H:98-364	65.92%ID	E:1e-125	GDSL esterase/lipase At5g33370	PF00657.19^Lipase_GDSL^GDSL-like Lipase/Acylhydrolase^57-244^E:5.3e-12	0	-1,411	0	-2,962	0,001	-1,737	0,002	-1,549	0,5	-0,554	GDL79_ARATH	GDSL esterase/lipase At5g33370 (EC 3.1.1.-) (Extracellular lipase At5g33370)	At5g33370 F19N2.90	Arabidopsis thaliana (Mouse-ear cress)		Alternative splicing; Complete proteome; Glycoprotein; Hydrolase; Lipid degradation; Lipid metabolism; Reference proteome; Secreted; Signal
SRR1271858_TR26724_c0_g1_i1_len=643	H4_SOYBN	Q:471-238,H:22-99	100%ID	E:2e-46	Histone H4	PF15511.3^CENP-T_C^Centromere kinetochore component CENP-T histone fold^35-96^E:1.4e-08`PF02969.14^TAF^TATA box binding protein associated factor (TAF)^35-92^E:0.00016	0	-1,351	0	-2,035	0,886	0,14	0,233	-0,75	0,306	-0,498	H4_SOYBN	Histone H4		Glycine max (Soybean) (Glycine hispida)		Acetylation; Chromosome; Complete proteome; DNA-binding; Methylation; Nucleosome core; Nucleus; Reference proteome

SRR1271858_TR48478_c0_g1_i1_len=650	H32_WHEAT	Q:528-121,H:1-136	99.26%ID	E:3e-77	Histone H3.2	PF00125.21^Histone^Core histone H2A/H2B/H3/H4^1-132^E:8.3e-55	0	-1,684	0	-4,424	0,377	-0,692	0,045	-1,482	1	-0,46	H32_WHEAT	Histone H3.2		Triticum aestivum (Wheat)		Acetylation; Chromosome; Complete proteome; DNA-binding; Direct protein sequencing; Methylation; Nucleosome core; Nucleus; Phosphoprotein; Reference proteome
SRR1271859_TR26446_c2_g4_i4_len=945	H2A1_MEDTR	Q:717-394,H:26-132	93.52%ID	E:1e-47	Probable histone H2A.1	PF00125.21^Histone^Core histone H2A/H2B/H3/H4^20-97^E:6.9e-13`PF16211.2^Histone_H2A_C^C-terminus of histone H2A^100-132^E:2.7e-18	0	-1,019	0	-1,505	0,505	0,438	0,86	0,2	0,025	0,812	H2A1_MEDTR	Probable histone H2A.1	MtrDRAFT_AC149210g1v1	Medicago truncatula (Barrel medic) (Medicago tribuloides)		Chromosome; DNA-binding; Nucleosome core; Nucleus
SRR1271859_TR26579_c1_g1_i5_len=1242	XTH9_ARATH	Q:101-544,H:10-156	72.97%ID	E:1e-118	Xyloglucan endotransglucosylase/hydrolase protein 9	PF00722.18^Glyco_hydro_16^Glycosyl hydrolases family 16^56-180^E:8.7e-39	0	-1,227	0	-3,151	1	0,057	0,581	-0,493	0,038	-1,756	XTH9_ARATH	Xyloglucan endotransglucosylase/hydrolase protein 9 (At-XTH9) (XTH-9) (EC 2.4.1.207)	XTH9 EXGT-A6 XTR16 At4g03210 F4C21.14	Arabidopsis thaliana (Mouse-ear cress)		Alternative splicing; Apoplast; Cell wall; Cell wall biogenesis/degradation; Complete proteome; Disulfide bond; Glycoprotein; Glycosidase; Hydrolase; Reference proteome; Secreted; Signal; Transferase
SRR1271859_TR33596_c0_g1_i2_len=560	H4_SOYBN	Q:247-480,H:22-99	100%ID	E:2e-47	Histone H4	PF15511.3^CENP-T_C^Centromere kinetochore component CENP-T histone fold^35-96^E:1.4e-08`PF02969.14^TAF^TATA box binding protein associated factor (TAF)^35-92^E:0.00016	0	-1,58	0	-1,809	0,312	0,556	0,874	0,108	0,076	-0,396	H4_SOYBN	Histone H4		Glycine max (Soybean) (Glycine hispida)		Acetylation; Chromosome; Complete proteome; DNA-binding; Methylation; Nucleosome core; Nucleus; Reference proteome
SRR342017_TR4062_c1_g6_i2_len=913	NA	NA	NA	NA	NA	PF12049.5^DUF3531^Protein of unknown function (DUF3531)^87-227^E:4.7e-55	0	-3,566	0	-3,933	1	-0,073	1	-2,396	0,418	0,317	.	.	.	.	.	.
SRR342017_TR9810_c0_g2_i1_len=881	RUBB_PEA	Q:881-60,H:318-591	93.07%ID	E:1e-159	RuBisCO large subunit-binding protein subunit beta, chloroplastic	PF00118.21^Cpn60_TCP1^TCP-1/cpn60 chaperonin family^16-258^E:2.9e-49	0,001	-0,958	0	-1,143	0,099	0,553	0,192	0,447	1	0,071	RUBB_PEA	RuBisCO large subunit-binding protein subunit beta, chloroplastic (60 kDa chaperonin subunit beta) (CPN-60 beta)		Pisum sativum (Garden pea)		ATP-binding; Chaperone; Chloroplast; Direct protein sequencing; Nucleotide-binding; Plastid; Transit peptide
SRR342017_TR16448_c0_g1_i6_len=793	H2B1_MEDTR	Q:505-230,H:57-148	98.91%ID	E:2e-57	Probable histone H2B.1	PF00125.21^Histone^Core histone H2A/H2B/H3/H4^2-128^E:1.3e-22`PF00808.20^CBFD_NFYB_HMF^Histone-like transcription factor (CBF/NF-Y) and archaeal histone^70-126^E:0.00028	0	-1,228	0	-2,359	1	0,08	0,34	0,47	0,52	0,45	H2B1_MEDTR	Probable histone H2B.1		Medicago truncatula (Barrel medic) (Medicago tribuloides)		Acetylation; Chromosome; DNA-binding; Isopeptide bond; Nucleosome core; Nucleus; Ubl conjugation
SRR342017_TR16982_c0_g1_i1_len=533	H32_LILLO	Q:389-216,H:1-58	100%ID	E:5e-31	Histone H3.2	PF00125.21^Histone^Core histone H2A/H2B/H3/H4^49-109^E:1.4e-10	0	-1,865	0	-3,354	0,732	0,666	1	0,462	0,671	-0,133	H32_LILLO	Histone H3.2	YAH3	Lilium longiflorum (Trumpet lily)		Acetylation; Chromosome; DNA-binding; Direct protein sequencing; Methylation; Nucleosome core; Nucleus; Phosphoprotein
SRR342017_TR20766_c0_g1_i3_len=679	NIFU1_ARATH	Q:509-18,H:66-230	77.58%ID	E:4e-65	NifU-like protein 1, chloroplastic	PF01106.14^NifU^NifU-like domain^2-59^E:7.4e-23`PF01106.14^NifU^NifU-like domain^81-137^E:3.2e-05	0	-6,96	0	-2,823	0,152	-0,803	0,037	0,958	0,502	-3,927	NIFU1_ARATH	NifU-like protein 1, chloroplastic (AtCNfu1) (AtCnfU-IVb)	NIFU1 CNFU1 NFU1 At4g01940 T7B11.20	Arabidopsis thaliana (Mouse-ear cress)		Chloroplast; Complete proteome; Disulfide bond; Plastid; Reference proteome; Transit peptide
SRR342017_TR22430_c1_g1_i21_len=674	RL291_ARATH	Q:202-384,H:1-61	81.97%ID	E:1e-26	60S ribosomal protein L29-1	.	0	-1,675	0	-2,104	0,592	-0,205	0,226	-0,466	0,12	-0,441	RL291_ARATH	60S ribosomal protein L29-1	RPL29A At3g06700 F3E22.16 T8E24.8	Arabidopsis thaliana (Mouse-ear cress)		Complete proteome; Phosphoprotein; Reference proteome; Ribonucleoprotein; Ribosomal protein
SRR342017_TR24038_c0_g1_i2_len=1157	XTH9_ARATH	Q:936-211,H:58-289	76.45%ID	E:7e-138	Xyloglucan endotransglucosylase/hydrolase protein 9	PF00722.18^Glyco_hydro_16^Glycosyl hydrolases family 16^3-116^E:5.4e-39`PF06955.9^XET_C^Xyloglucan endo-transglycosylase (XET) C-terminus^158-203^E:8.7e-18	0	-0,929	0	-3,345	0,133	0,617	0,754	0,194	0,452	-0,56	XTH9_ARATH	Xyloglucan endotransglucosylase/hydrolase protein 9 (At-XTH9) (XTH-9) (EC 2.4.1.207)	XTH9 EXGT-A6 XTR16 At4g03210 F4C21.14	Arabidopsis thaliana (Mouse-ear cress)		Alternative splicing; Apoplast; Cell wall; Cell wall biogenesis/degradation; Complete proteome; Disulfide bond; Glycoprotein; Glycosidase; Hydrolase; Reference proteome; Secreted; Signal; Transferase
SRR342019_TR18511_c2_g5_i3_len=1003	H32_WHEAT	Q:826-419,H:1-136	99.26%ID	E:1e-75	Histone H3.2	PF00125.21^Histone^Core histone H2A/H2B/H3/H4^1-132^E:8.3e-55	0	-1,493	0	-3,562	1	-0,465	0,299	-1,391	0,006	-0,807	H32_WHEAT	Histone H3.2		Triticum aestivum (Wheat)		Acetylation; Chromosome; Complete proteome; DNA-binding; Direct protein sequencing; Methylation; Nucleosome core; Nucleus; Phosphoprotein; Reference proteome
SRR342019_TR24957_c0_g2_i1_len=897	NA	NA	NA	NA	NA	.	0	-1,889	0	-4,213	1	0,439	1	0,413	1	0	.	.	.	.	.	.
SRR342019_TR28229_c0_g1_i1_len=1357	SODF3_ARATH	Q:997-341,H:45-263	77.63%ID	E:2e-128	Superoxide dismutase [Fe] 3, chloroplastic	PF00081.19^Sod_Fe_N^Iron/manganese superoxide dismutases, alpha-hairpin domain^55-140^E:2.9e-21`PF02777.15^Sod_Fe_C^Iron/manganese superoxide dismutases, C-terminal domain^150-250^E:1.2e-35	0,001	-0,931	0	-1,816	0,5	-0,792	0,832	-0,36	0,281	-0,299	SODF3_ARATH	Superoxide dismutase [Fe] 3, chloroplastic (EC 1.15.1.1) (Protein FE SUPEROXIDE DISMUTASE 3)	FSD3 At5g23310 MKD15.17	Arabidopsis thaliana (Mouse-ear cress)		Chloroplast; Complete proteome; Iron; Metal-binding; Oxidoreductase; Plastid; Reference proteome; Thylakoid; Transit peptide
SRR342019_TR28386_c1_g1_i1_len=1347	EP1G_DAUCA	Q:1181-294,H:8-309	48.71%ID	E:7e-84	Epidermis-specific secreted glycoprotein EP1	PF01453.21^B_lectin^D-mannose binding lectin^86-179^E:1.8e-26	0	-0,918	0	-1,669	1	0,065	1	0,135	0,126	-0,35	EP1G_DAUCA	Epidermis-specific secreted glycoprotein EP1 (52/54 kDa medium protein)	EP1	Daucus carota (Wild carrot)		Direct protein sequencing; Glycoprotein; Lectin; Secreted; Signal
SRR342019_TR33798_c0_g1_i2_len=846	PSAEA_NICSY	Q:604-242,H:21-141	69.92%ID	E:4e-36	Photosystem I reaction center subunit IV A, chloroplastic	PF02427.14^PSI_PsaE^Photosystem I reaction centre subunit IV / PsaE^141-200^E:4.7e-30	0	-1,596	0	-1,187	0,009	-0,583	0,02	-0,519	0,891	0,058	PSAEA_NICSY	Photosystem I reaction center subunit IV A, chloroplastic (PSI-E A) [Cleaved into: Photosystem I reaction center subunit IV A isoform 2]	PSAEA	Nicotiana sylvestris (Wood tobacco) (South American tobacco)		Chloroplast; Direct protein sequencing; Membrane; Photosynthesis; Photosystem I; Plastid; Thylakoid; Transit peptide
SRR342022_TR4553_c9_g3_i4_len=812	H2B1_MEDTR	Q:525-250,H:57-148	98.91%ID	E:6e-58	Probable histone H2B.1	PF00125.21^Histone^Core histone H2A/H2B/H3/H4^37-163^E:2.5e-22	0	-1,132	0	-1,996	0,429	0,466	0,623	0,35	0,005	-1,05	H2B1_MEDTR	Probable histone H2B.1		Medicago truncatula (Barrel medic) (Medicago tribuloides)		Acetylation; Chromosome; DNA-binding; Isopeptide bond; Nucleosome core; Nucleus; Ubl conjugation
SRR342022_TR6932_c1_g2_i3_len=1899	GSA_TOBAC	Q:1671-268,H:18-478	83.55%ID	E:0	Glutamate-1-semialdehyde 2,1-aminomutase, chloroplastic	PF00202.18^Aminotran_3^Aminotransferase class-III^84-466^E:1.5e-69	0	-0,917	0	-1,074	0,029	-0,65	0,485	-0,211	1	3,367	GSA_TOBAC	Glutamate-1-semialdehyde 2,1-aminomutase, chloroplastic (GSA) (EC 5.4.3.8) (Glutamate-1-semialdehyde aminotransferase) (GSA-AT)	GSA	Nicotiana tabacum (Common tobacco)	PATHWAY: Porphyrin-containing compound metabolism; protoporphyrin-IX biosynthesis; 5-aminolevulinate from L-glutamyl-tRNA(Glu): step 2/2.; PATHWAY: Porphyrin-containing compound metabolism; chlorophyll biosynthesis.	Chlorophyll biosynthesis; Chloroplast; Isomerase; Plastid; Porphyrin biosynthesis; Pyridoxal phosphate; Transit peptide
SRR342022_TR15174_c6_g3_i1_len=643	CP20B_ARATH	Q:486-37,H:1-153	55.48%ID	E:3e-33	.	PF00160.18^Pro_isomerase^Cyclophilin type peptidyl-prolyl cis-trans isomerase/CLD^146-203^E:1.1e-14	0	-0,868	0	-0,974	0,02	-1,099	0,14	-0,68	1	-0,287	PNSL5_ARATH	Photosynthetic NDH subunit of lumenal location 5, chloroplastic (Cyclophilin of 20 kDa 2) (Peptidyl-prolyl cis-trans isomerase CYP20-2) (PPIase CYP20-2) (EC 5.2.1.8) (Rotamase CYP20-2) (Thylakoid lumen PPIase of 20 kDa) (TLP20)	PNSL5 CYP20-2 At5g13120 T19L5_80	Arabidopsis thaliana (Mouse-ear cress)		Alternative splicing; Chaperone; Chloroplast; Complete proteome; Cyclosporin; Direct protein sequencing; Disulfide bond; Isomerase; Membrane; Plastid; Reference proteome; Rotamase; Thylakoid; Transit peptide; Transport
SRR342022_TR18762_c3_g4_i2_len=1984	CCNAL_DAUCA	Q:441-1436,H:11-331	61.26%ID	E:5e-136	G2/mitotic-specific cyclin C13-1	PF00134.20^Cyclin_N^Cyclin, N-terminal domain^107-235^E:3e-43`PF02984.16^Cyclin_C^Cyclin, C-terminal domain^238-360^E:8.9e-32	0	-1,569	0	-4,589	1	-0,307	1	-1,123	1	0,61	CCNAL_DAUCA	G2/mitotic-specific cyclin C13-1 (A-like cyclin) (Fragment)		Daucus carota (Wild carrot)		Cell cycle; Cell division; Cyclin; Mitosis
SRR342022_TR32140_c0_g1_i1_len=822	CCD32_ARATH	Q:107-727,H:168-367	46.76%ID	E:2e-57	Cyclin-D3-2	PF02984.16^Cyclin_C^Cyclin, C-terminal domain^16-125^E:1.1e-23	0	-1,85	0	-5,184	1	0	0,254	4,691	0,07	-0,728	CCD32_ARATH	Cyclin-D3-2 (G1/S-specific cyclin-D3-2) (CycD3;2)	CYCD3-2 At5g67260 K3G17.2	Arabidopsis thaliana (Mouse-ear cress)		Cell cycle; Cell division; Complete proteome; Cyclin; Reference proteome
SRR342023_TR8541_c0_g3_i1_len=664	RL111_ARATH	Q:160-663,H:1-168	95.83%ID	E:7e-107	60S ribosomal protein L11-1	PF00281.16^Ribosomal_L5^Ribosomal protein L5^62-115^E:1.4e-20`PF00673.18^Ribosomal_L5_C^ribosomal L5P family C-terminus^119-217^E:1.4e-20	0	-1,485	0	-1,737	0,317	-0,487	0,634	-0,274	0,051	-0,839	RL111_ARATH	60S ribosomal protein L11-1 (L16A)	RPL11A RPL16A At2g42740 F7D19.26	Arabidopsis thaliana (Mouse-ear cress)		Complete proteome; Cytoplasm; RNA-binding; Reference proteome; Ribonucleoprotein; Ribosomal protein; rRNA-binding
SRR342023_TR11135_c0_g1_i4_len=607	H2B1_MEDTR	Q:301-576,H:57-148	98.91%ID	E:2e-58	Probable histone H2B.1	PF00125.21^Histone^Core histone H2A/H2B/H3/H4^2-128^E:1.3e-22`PF00808.20^CBFD_NFYB_HMF^Histone-like transcription factor (CBF/NF-Y) and archaeal histone^70-126^E:0.00028	0	-1,341	0	-0,99	1	-0,027	0,037	-0,676	0,014	-0,542	H2B1_MEDTR	Probable histone H2B.1		Medicago truncatula (Barrel medic) (Medicago tribuloides)		Acetylation; Chromosome; DNA-binding; Isopeptide bond; Nucleosome core; Nucleus; Ubl conjugation
SRR342023_TR12524_c0_g5_i1_len=2310	CCD31_ARATH	Q:1845-1093,H:53-300	55.43%ID	E:3e-80	Cyclin-D3-1	PF00134.20^Cyclin_N^Cyclin, N-terminal domain^76-203^E:2.5e-26`PF02984.16^Cyclin_C^Cyclin, C-terminal domain^205-331^E:2.3e-19	0	-1,372	0	-3,874	0,132	-1,451	0,008	-2,897	0,285	-0,345	CCD31_ARATH	Cyclin-D3-1 (Cyclin-delta-3) (Cyclin-d3) (G1/S-specific cyclin-D3-1) (CycD3;1)	CYCD3-1 CYCD3 At4g34160 F28A23.80	Arabidopsis thaliana (Mouse-ear cress)		Cell cycle; Cell division; Complete proteome; Cyclin; Mitosis; Reference proteome
SRR646572_TR17837_c0_g1_i1_len=3147	POR_DAUCA	Q:2971-1796,H:1-398	84.67%ID	E:0	Protochlorophyllide reductase, chloroplastic	PF08659.7^KR^KR domain^80-173^E:8e-09`PF00106.22^adh_short^short chain dehydrogenase^82-224^E:6.5e-21`PF13561.3^adh_short_C2^Enoyl-(Acyl carrier protein) reductase^86-223^E:1.5e-12	0	-0,812	0	-2,568	0,037	-1,189	0,241	-0,697	0,016	0,585	POR_DAUCA	Protochlorophyllide reductase, chloroplastic (PCR) (EC 1.3.1.33) (NADPH-protochlorophyllide oxidoreductase) (POR)	POR1	Daucus carota (Wild carrot)	PATHWAY: Porphyrin-containing compound metabolism; chlorophyll biosynthesis.	Chlorophyll biosynthesis; Chloroplast; NADP; Oxidoreductase; Photosynthesis; Plastid; Transit peptide
SRR648705_TR25739_c0_g2_i1_len=813	H32_WHEAT	Q:206-613,H:1-136	99.26%ID	E:2e-76	Histone H3.2	PF00125.21^Histone^Core histone H2A/H2B/H3/H4^1-132^E:8.3e-55	0,001	-1,252	0	-3,548	1	-0,328	1	-0,518	0,008	-0,712	H32_WHEAT	Histone H3.2		Triticum aestivum (Wheat)		Acetylation; Chromosome; Complete proteome; DNA-binding; Direct protein sequencing; Methylation; Nucleosome core; Nucleus; Phosphoprotein; Reference proteome
SRR648705_TR34697_c0_g1_i1_len=1268	NA	NA	NA	NA	NA	PF00538.16^Linker_histone^linker histone H1 and H5 family^27-87^E:5.8e-09`PF02178.16^AT_hook^AT hook motif^122-130^E:25`PF02178.16^AT_hook^AT hook motif^151-162^E:0.18`PF02178.16^AT_hook^AT hook motif^188-199^E:0.55	0	-1,424	0	-3,574	0,073	-2,034	0,025	-2,74	0,033	-0,753	.	.	.	.	.	.
SRR648705_TR37418_c0_g2_i3_len=1382	KITH_ORYSJ	Q:935-579,H:67-186	74.17%ID	E:3e-76	Thymidine kinase	PF00265.15^TK^Thymidine kinase^22-145^E:2e-31	0,001	-1,211	0	-3,473	0,732	0,574	1	0,012	0,021	-0,937	KITH_ORYSJ	Thymidine kinase (EC 2.7.1.21)	TK Os03g0113100 LOC_Os03g02200 OSJNBa0090O10.3	Oryza sativa subsp. japonica (Rice)		ATP-binding; Alternative splicing; Complete proteome; DNA synthesis; Kinase; Metal-binding; Nucleotide-binding; Reference proteome; Transferase; Zinc
SRR648707_TR18549_c0_g2_i1_len=649	H32_WHEAT	Q:622-215,H:1-136	99.26%ID	E:1e-77	Histone H3.2	PF00125.21^Histone^Core histone H2A/H2B/H3/H4^10-141^E:1.1e-54	0	-1,447	0	-4,24	0,564	0,29	0,798	-0,226	0,004	-0,671	H32_WHEAT	Histone H3.2		Triticum aestivum (Wheat)		Acetylation; Chromosome; Complete proteome; DNA-binding; Direct protein sequencing; Methylation; Nucleosome core; Nucleus; Phosphoprotein; Reference proteome
SRR648707_TR26172_c0_g1_i1_len=745	H2B1_MEDTR	Q:303-578,H:57-148	98.91%ID	E:4e-58	Probable histone H2B.1	PF00125.21^Histone^Core histone H2A/H2B/H3/H4^46-168^E:2.6e-22	0	-1,239	0	-1,853	0,173	0,461	0,316	0,355	0,005	-0,645	H2B1_MEDTR	Probable histone H2B.1		Medicago truncatula (Barrel medic) (Medicago tribuloides)		Acetylation; Chromosome; DNA-binding; Isopeptide bond; Nucleosome core; Nucleus; Ubl conjugation
SRR924147_TR4550_c0_g2_i2_len=845	H32_WHEAT	Q:722-315,H:1-136	99.26%ID	E:4e-77	Histone H3.2	PF00125.21^Histone^Core histone H2A/H2B/H3/H4^42-173^E:2.1e-54	0	-1,383	0	-8,521	1	-0,216	0,066	-5,389	0,01	-1,135	H32_WHEAT	Histone H3.2		Triticum aestivum (Wheat)		Acetylation; Chromosome; Complete proteome; DNA-binding; Direct protein sequencing; Methylation; Nucleosome core; Nucleus; Phosphoprotein; Reference proteome
SRR924147_TR7895_c0_g1_i1_len=880	NA	NA	NA	NA	NA	PF12049.5^DUF3531^Protein of unknown function (DUF3531)^40-180^E:2.4e-55	0	-3,12	0	-5,242	1	1,538	1	0	0,004	-3,021	.	.	.	.	.	.
SRR924147_TR27771_c0_g2_i2_len=810	H2A4_ORYSJ	Q:184-507,H:30-137	92.59%ID	E:1e-48	Probable histone H2A.4	PF00125.21^Histone^Core histone H2A/H2B/H3/H4^20-97^E:6.9e-13`PF16211.2^Histone_H2A_C^C-terminus of histone H2A^100-132^E:1.6e-18	0	-1,214	0	-1,784	0,299	0,44	1	-0,006	0,006	-0,692	H2A4_ORYSJ	Probable histone H2A.4	Os05g0461400 LOC_Os05g38640 OJ1281_H05.14 OJ1525_A02.1 OsJ_018061	Oryza sativa subsp. japonica (Rice)		Chromosome; Complete proteome; DNA-binding; Nucleosome core; Nucleus; Reference proteome
SRR924148_TR4114_c0_g3_i1_len=1065	H2A1_SOLLC	Q:723-358,H:22-146	86.4%ID	E:3e-50	Histone H2A.1	PF00125.21^Histone^Core histone H2A/H2B/H3/H4^17-94^E:9.2e-13`PF16211.2^Histone_H2A_C^C-terminus of histone H2A^97-129^E:2e-18	0	-1,332	0	-2,811	0,811	-0,312	0,44	-0,811	0,337	-0,282	H2A1_SOLLC	Histone H2A.1 (LeH2A-1)		Solanum lycopersicum (Tomato) (Lycopersicon esculentum)		Acetylation; Chromosome; Complete proteome; DNA-binding; Nucleosome core; Nucleus; Reference proteome
SRR924148_TR26974_c1_g1_i1_len=814	H4_SOYBN	Q:627-382,H:22-103	100%ID	E:6e-49	Histone H4	PF15511.3^CENP-T_C^Centromere kinetochore component CENP-T histone fold^35-96^E:1.4e-08`PF02969.14^TAF^TATA box binding protein associated factor (TAF)^35-92^E:0.00016	0	-1,401	0	-2,463	1	0,001	0,306	-0,442	0,004	-0,782	H4_SOYBN	Histone H4		Glycine max (Soybean) (Glycine hispida)		Acetylation; Chromosome; Complete proteome; DNA-binding; Methylation; Nucleosome core; Nucleus; Reference proteome
SRR924148_TR35599_c0_g2_i1_len=1604	FTSZ1_ARATH	Q:1353-295,H:36-397	87.29%ID	E:5e-171	Cell division protein FtsZ homolog 1, chloroplastic	PF00091.22^Tubulin^Tubulin/FtsZ family, GTPase domain^64-224^E:2.4e-42`PF12327.5^FtsZ_C^FtsZ family, C-terminal domain^273-366^E:6.8e-30	0	-7,253	0	-6,68	0	6,834	1	0	0,37	0,432	FTSZ1_ARATH	Cell division protein FtsZ homolog 1, chloroplastic (AtFtsZ1) (AtFtsZ1-1) (Chloroplast FtsZ) (CpFtsZ) (Protein ACCUMULATION AND REPLICATION OF CHLOROPLASTS 10) (Protein PLASTID MOVEMENT IMPAIRED4)	FTSZ1 ARC10 FTSZ1-1 PMI4 At5g55280 MCO15.23	Arabidopsis thaliana (Mouse-ear cress)		Acetylation; Cell cycle; Chloroplast; Complete proteome; GTP-binding; Membrane; Nucleotide-binding; Plastid; Reference proteome; Thylakoid; Transit peptide

Ms1wk

Transcript (CDF97)	Uniprot homolog	BLASTX Position	BLASTX %Identity	BLASTX evalue	Uniprot descr	Pfam	PValue_Ms6h	logFC_Ms6h	PValue_Ms8h	logFC_Ms8h	PValue_MsDamaged24h	logFC_MsDamaged24h	PValue_MsIntact24h	logFC_MsIntact24h	PValue_Ms1wk	logFC_Ms1wk	Entry.name	Protein names	Gene names	Organism	Pathway	Keywords
SRR1144633_TR5470_c0_g1_i1_len=2288	YHKF_SCHPO	Q:784-1293,H:159-325	38.01%ID	E:3e-32	Uncharacterized RNA-binding protein C660.15	PF00076.19^RRM_1^RNA recognition motif. (a.k.a. RRM, RBD, or RNP domain)^6-66^E:4e-13`PF00076.19^RRM_1^RNA recognition motif. (a.k.a. RRM, RBD, or RNP domain)^94-151^E:4.4e-11`PF00076.19^RRM_1^RNA recognition motif. (a.k.a. RRM, RBD, or RNP domain)^244-297^E:3.6e-13	1	-0,487	0,76	0,59	1	-0,259	0,017	-5,782	0	-3,19	YHKF_SCHPO	Uncharacterized RNA-binding protein C660.15	SPBC660.15	Schizosaccharomyces pombe (strain 972 / ATCC 24843) (Fission yeast)		Complete proteome; Nucleus; RNA-binding; Reference proteome; Repeat
SRR1144633_TR5687_c0_g2_i2_len=2266	RD21A_ARATH	Q:1992-763,H:23-434	68.67%ID	E:0	Cysteine proteinase RD21a	PF08246.9^Inhibitor_I29^Cathepsin propeptide inhibitor domain (I29)^116-173^E:1.2e-16`PF00112.20^Peptidase_C1^Papain family cysteine protease^204-419^E:2e-84`PF00396.15^Granulin^Granulin^453-499^E:9.2e-11	0,123	0,792	0,204	0,586	0,629	2,121	1	1,322	0	-7,253	RD21A_ARATH	Cysteine proteinase RD21a (RD21) (EC 3.4.22.-)	RD21A At1g47128 F2G19.31	Arabidopsis thaliana (Mouse-ear cress)		Complete proteome; Disulfide bond; Glycoprotein; Hydrolase; Protease; Reference proteome; Signal; Thiol protease; Zymogen
SRR1144633_TR13566_c0_g2_i3_len=680	Y4757_DICDI	Q:609-166,H:608-766	37.11%ID	E:4e-23	OTU domain-containing protein DDB_G0284757	PF02338.16^OTU^OTU-like cysteine protease^53-142^E:7.9e-11	1	0,627	1	-0,16	1	0	1	0	0,001	-5,554	Y4757_DICDI	OTU domain-containing protein DDB_G0284757 (EC 3.1.2.-)	DDB_G0284757	Dictyostelium discoideum (Slime mold)		Complete proteome; Hydrolase; Protease; Reference proteome; Thiol protease
SRR1144633_TR19396_c0_g2_i1_len=1200	HS154_ARATH	Q:854-447,H:1-134	51.45%ID	E:3e-37	15.4 kDa class V heat shock protein	PF00011.18^HSP20^Hsp20/alpha crystallin family^33-112^E:1.6e-15	0,803	-0,085	0,909	-0,048	0,798	0,512	0,187	1,221	0,001	-1,438	HS154_ARATH	15.4 kDa class V heat shock protein (15.4 kDa heat shock protein) (AtHsp15.4)	HSP15.4 At4g21870 T8O5.80	Arabidopsis thaliana (Mouse-ear cress)		Complete proteome; Cytoplasm; Reference proteome; Stress response
SRR1144633_TR23247_c0_g3_i1_len=610	NA	NA	NA	NA	NA	.	0,454	0,326	0,555	0,315	0,626	0,286	1	0,018	0	-1,426	.	.	.	.	.	.
SRR1144633_TR27825_c0_g4_i7_len=5814	POLX_TOBAC	Q:2260-5787,H:1-1161	40.27%ID	E:0	Retrovirus-related Pol polyprotein from transposon TNT 1-94	PF14223.3^Retrotran_gag_2^gag-polypeptide of LTR copia-type^59-187^E:1.8e-33`PF00098.20^zf-CCHC^Zinc knuckle^227-242^E:0.00069`PF13976.3^gag_pre-integrs^GAG-pre-integrase domain^397-464^E:2.9e-14`PF00665.23^rve^Integrase core domain^480-594^E:6.3e-26`PF07727.11^RVT_2^Reverse transcriptase (RNA-dependent DNA polymerase)^854-1101^E:1.2e-91	0,502	3,981	1	-2,771	1	1,641	1	0	0	-6,243	POLX_TOBAC	Retrovirus-related Pol polyprotein from transposon TNT 1-94 [Includes: Protease (EC 3.4.23.-); Reverse transcriptase (EC 2.7.7.49); Endonuclease]		Nicotiana tabacum (Common tobacco)		Aspartyl protease; Endonuclease; Hydrolase; Metal-binding; Nuclease; Nucleotidyltransferase; Protease; RNA-directed DNA polymerase; Transferase; Transposable element; Zinc; Zinc-finger
SRR1144633_TR27911_c0_g1_i1_len=851	NDK1_TOBAC	Q:196-639,H:1-148	95.95%ID	E:3e-89	Nucleoside diphosphate kinase 1	.	1	0	1	0	1	0	1	0	0	-0,871	NDK1_TOBAC	Nucleoside diphosphate kinase 1 (EC 2.7.4.6) (Nucleoside diphosphate kinase I) (NDK I) (NDP kinase I) (NDPK I)		Nicotiana tabacum (Common tobacco)		ATP-binding; Kinase; Magnesium; Metal-binding; Nucleotide metabolism; Nucleotide-binding; Phosphoprotein; Transferase
SRR1144634_TR11817_c0_g1_i2_len=864	PSB3B_ARATH	Q:172-735,H:17-204	93.09%ID	E:2e-127	Proteasome subunit beta type-3-B	PF00227.23^Proteasome^Proteasome subunit^3-108^E:8.1e-22	1	-0,023	1	-0,354	0,461	1,315	1	-0,013	0	-3,971	PSB3B_ARATH	Proteasome subunit beta type-3-B (EC 3.4.25.1) (20S proteasome beta subunit C-2)	PBC2 At1g77440 T5M16.3	Arabidopsis thaliana (Mouse-ear cress)		Complete proteome; Cytoplasm; Hydrolase; Nucleus; Protease; Proteasome; Reference proteome; Threonine protease
SRR1144634_TR21619_c0_g1_i1_len=1897	NA	NA	NA	NA	NA	.	0,429	0,476	0	1,981	0,09	-0,581	0,371	-0,31	0	-1,071	.	.	.	.	.	.
SRR1144634_TR22915_c0_g1_i1_len=571	CVIF1_ARATH	Q:163-501,H:26-138	41.59%ID	E:2e-22	Cell wall / vacuolar inhibitor of fructosidase 1	PF04043.12^PMEI^Plant invertase/pectin methylesterase inhibitor^54-187^E:1e-13	0	1,217	0	1,991	0	1,233	0,932	-0,046	0	-7,8	CVIF1_ARATH	Cell wall / vacuolar inhibitor of fructosidase 1 (AtC/VIF1)	C/VIF1 CIF1 VIF1 At1g47960 T2J15.13	Arabidopsis thaliana (Mouse-ear cress)		Alternative splicing; Complete proteome; Disulfide bond; Glycoprotein; Reference proteome; Signal; Vacuole
SRR1144634_TR23693_c0_g1_i1_len=980	NA	NA	NA	NA	NA	PF00415.15^RCC1^Regulator of chromosome condensation (RCC1) repeat^2-42^E:3.1e-05`PF13540.3^RCC1_2^Regulator of chromosome condensation (RCC1) repeat^29-58^E:3.5e-09`PF00415.15^RCC1^Regulator of chromosome condensation (RCC1) repeat^45-103^E:1.8e-10`PF13540.3^RCC1_2^Regulator of chromosome condensation (RCC1) repeat^91-120^E:4.1e-08	1	-0,145	0,133	-1,124	0,118	2,087	0,502	-3,875	0,001	-6,586	.	.	.	.	.	.
SRR1144634_TR38687_c0_g1_i2_len=645	SP1L1_ARATH	Q:113-433,H:1-110	67.57%ID	E:1e-29	Protein SPIRAL1-like 1	.	0,08	-0,613	0,552	-0,242	0,594	0,27	0,826	0,162	0	-1,059	SP1L1_ARATH	Protein SPIRAL1-like 1	SP1L1 At1g26355 T1K7.26	Arabidopsis thaliana (Mouse-ear cress)		Complete proteome; Microtubule; Phosphoprotein; Reference proteome
SRR1144634_TR38813_c0_g1_i1_len=549	NA	NA	NA	NA	NA	.	1	0	1	0	1	0	1	0	0	-6,781	.	.	.	.	.	.
SRR122236_TR4154_c0_g1_i1_len=1464	MTDH_FRAAN	Q:121-1182,H:4-357	70.34%ID	E:2e-159	Probable mannitol dehydrogenase	PF08240.9^ADH_N^Alcohol dehydrogenase GroES-like domain^39-152^E:1.2e-27`PF00107.23^ADH_zinc_N^Zinc-binding dehydrogenase^195-317^E:2e-18	0,009	6,062	0,349	0,395	0,066	5,245	0	7,238	0	-9,752	MTDH_FRAAN	Probable mannitol dehydrogenase (EC 1.1.1.255) (NAD-dependent mannitol dehydrogenase)	CAD	Fragaria ananassa (Strawberry)		Metal-binding; NAD; Oxidoreductase; Zinc
SRR122236_TR10709_c1_g1_i2_len=1023	ARF2_ORYSJ	Q:915-373,H:1-181	99.45%ID	E:3e-129	ADP-ribosylation factor 2	PF00025.18^Arf^ADP-ribosylation factor family^5-177^E:3e-80`PF09439.7^SRPRB^Signal recognition particle receptor beta subunit^16-143^E:8.5e-14`PF08477.10^Roc^Ras of Complex, Roc, domain of DAPkinase^19-129^E:1.1e-13`PF04670.9^Gtr1_RagA^Gtr1/RagA G protein conserved region^19-142^E:2.5e-10`PF01926.20^MMR_HSR1^50S ribosome-binding GTPase^19-122^E:4.7e-05`PF00071.19^Ras^Ras family^20-172^E:1e-12	0,781	-0,45	1	0,318	0,189	1,557	0,299	1,336	0	-2,712	ARF2_ORYSJ	ADP-ribosylation factor 2	ARF Os05g0489600 LOC_Os05g41060 OJ1119_H02.17	Oryza sativa subsp. japonica (Rice)		Complete proteome; ER-Golgi transport; GTP-binding; Golgi apparatus; Lipoprotein; Myristate; Nucleotide-binding; Protein transport; Reference proteome; Transport
SRR122236_TR27651_c0_g1_i5_len=3650	CD27B_ARATH	Q:14-1435,H:173-634	67.93%ID	E:0	Cell division cycle protein 27 homolog B	PF00515.25^TPR_1^Tetratricopeptide repeat^363-395^E:4.7e-09`PF07719.14^TPR_2^Tetratricopeptide repeat^363-394^E:6e-06`PF13181.3^TPR_8^Tetratricopeptide repeat^363-393^E:0.00063`PF13432.3^TPR_16^Tetratricopeptide repeat^403-445^E:0.00035	0,854	-0,095	0,226	-0,507	0,438	-0,373	0,667	-0,254	0	-1,92	CD27B_ARATH	Cell division cycle protein 27 homolog B (CDC27 homolog B) (Protein HOBBIT)	CDC27B HBT At2g20000 T2G17.20	Arabidopsis thaliana (Mouse-ear cress)	PATHWAY: Protein modification; protein ubiquitination.	Cell cycle; Cell division; Complete proteome; Mitosis; Nucleus; Reference proteome; Repeat; TPR repeat; Ubl conjugation pathway
SRR122237_TR3203_c0_g2_i1_len=1640	CCD8A_ORYSJ	Q:551-114,H:405-550	80.14%ID	E:2e-77	Carotenoid cleavage dioxygenase 8 homolog A, chloroplastic	PF03055.12^RPE65^Retinal pigment epithelial membrane protein^2-115^E:1.9e-31	1	3,011	1	-0,641	1	0,585	1	2,545	0	-6,98	CCD8A_ORYSJ	Carotenoid cleavage dioxygenase 8 homolog A, chloroplastic (OsCCD8a) (EC 1.13.11.-) (Protein D10-like)	CCD8A D10L Os01g0566500 LOC_Os01g38580 OsJ_02260 P0002B05.5 P0697C12.32	Oryza sativa subsp. japonica (Rice)		Chloroplast; Complete proteome; Dioxygenase; Iron; Metal-binding; Oxidoreductase; Plastid; Reference proteome; Transit peptide
SRR122237_TR13269_c1_g1_i1_len=851	PSAG_ARATH	Q:77-499,H:27-160	72.34%ID	E:1e-66	Photosystem I reaction center subunit V, chloroplastic	PF01241.15^PSI_PSAK^Photosystem I psaG / psaK^53-144^E:2.5e-26	0,066	-5,347	0,282	-1,041	0,629	0,796	0,025	2,574	0	-6,292	PSAG_ARATH	Photosystem I reaction center subunit V, chloroplastic (PSI-G)	PSAG At1g55670 F20N2.21 F20N2.33 F20N2_3	Arabidopsis thaliana (Mouse-ear cress)		Chloroplast; Complete proteome; Membrane; Photosynthesis; Photosystem I; Plastid; Reference proteome; Thylakoid; Transit peptide; Transmembrane; Transmembrane helix
SRR122237_TR13364_c0_g1_i1_len=2449	EXLA2_ARATH	Q:1584-1826,H:184-264	70.37%ID	E:9e-46	Expansin-like A2	PF03330.15^DPBB_1^Lytic transglycolase^58-132^E:6.4e-12	1	0,347	1	0,839	0,629	-2,009	0,629	-1,43	0	-1,903	EXLA2_ARATH	Expansin-like A2 (At-EXPL2) (AtEXLA2) (AtEXPL2) (Ath-ExpBeta-2.2)	EXLA2 EXPL2 At4g38400 F22I13.170	Arabidopsis thaliana (Mouse-ear cress)		Complete proteome; Glycoprotein; Reference proteome; Secreted; Signal
SRR122238_TR60_c0_g1_i1_len=1086	NA	NA	NA	NA	NA	.	0,706	0,151	0	1,917	0,92	-0,039	0,115	-0,488	0	-1,09	.	.	.	.	.	.
SRR122238_TR3465_c0_g1_i1_len=964	ML423_ARATH	Q:143-580,H:7-150	55.48%ID	E:9e-43	MLP-like protein 423	PF00407.16^Bet_v_1^Pathogenesis-related protein Bet v I family^1-152^E:8.9e-40`PF10604.6^Polyketide_cyc2^Polyketide cyclase / dehydrase and lipid transport^5-139^E:1.3e-06	1	0,097	0,129	-3,587	0,066	-1,967	0,228	-0,958	0	-4,196	ML423_ARATH	MLP-like protein 423	MLP423 At1g24020 T23E23.17 T23E23_22	Arabidopsis thaliana (Mouse-ear cress)		Complete proteome; Reference proteome
SRR122238_TR3803_c0_g1_i1_len=948	WUN1_SOLTU	Q:281-607,H:1-105	67.89%ID	E:2e-43	Wound-induced protein 1	PF07107.8^WI12^Wound-induced protein WI12^94-202^E:2.8e-44	0,062	0,603	0	2,072	0	1,436	0,004	1,018	0	-1,487	WUN1_SOLTU	Wound-induced protein 1	WUN1	Solanum tuberosum (Potato)		Complete proteome; Reference proteome
SRR122238_TR4063_c0_g1_i3_len=1097	PSD10_DICDI	Q:958-284,H:11-231	35.11%ID	E:1e-30	26S proteasome non-ATPase regulatory subunit 10	PF12796.4^Ank_2^Ankyrin repeats (3 copies)^17-114^E:2.8e-14`PF13637.3^Ank_4^Ankyrin repeats (many copies)^48-104^E:1.1e-08`PF13857.3^Ank_5^Ankyrin repeats (many copies)^78-124^E:7.7e-10`PF13606.3^Ank_3^Ankyrin repeat^83-111^E:1.3e-05`PF00023.27^Ank^Ankyrin repeat^84-115^E:3.2e-07`PF13637.3^Ank_4^Ankyrin repeats (many copies)^86-128^E:7.8e-11`PF13857.3^Ank_5^Ankyrin repeats (many copies)^103-157^E:1.2e-10`PF12796.4^Ank_2^Ankyrin repeats (3 copies)^116-178^E:4e-14`PF00023.27^Ank^Ankyrin repeat^117-148^E:1.4e-06`PF13606.3^Ank_3^Ankyrin repeat^117-144^E:0.00016`PF13637.3^Ank_4^Ankyrin repeats (many copies)^118-170^E:2.2e-13`PF12796.4^Ank_2^Ankyrin repeats (3 copies)^150-213^E:3.7e-12`PF00023.27^Ank^Ankyrin repeat^150-180^E:4.7e-06`PF13606.3^Ank_3^Ankyrin repeat^150-177^E:1.3e-05`PF13637.3^Ank_4^Ankyrin repeats (many copies)^161-203^E:7.5e-06`PF13857.3^Ank_5^Ankyrin repeats (many copies)^169-220^E:1.8e-08`PF00023.27^Ank^Ankyrin repeat^183-214^E:0.0001`PF13606.3^Ank_3^Ankyrin repeat^183-211^E:0.00047	1	3,674	1	-3,014	1	2,606	1	3,192	0	-4,863	PSD10_DICDI	26S proteasome non-ATPase regulatory subunit 10 (26S proteasome regulatory subunit p28)	psmD10 DDB_G0289189	Dictyostelium discoideum (Slime mold)		ANK repeat; Chaperone; Complete proteome; Reference proteome; Repeat
SRR122238_TR5403_c0_g1_i1_len=1075	NA	NA	NA	NA	NA	.	0,656	0,352	0,195	-0,901	1	-0,679	1	-0,707	0	-1,768	.	.	.	.	.	.
SRR122238_TR5736_c0_g1_i1_len=569	SBP3_ARATH	Q:521-225,H:1-99	73.74%ID	E:1e-47	Probable steroid-binding protein 3	PF00173.25^Cyt-b5^Cytochrome b5-like Heme/Steroid binding domain^20-101^E:6.2e-15	0,528	0,288	0,474	-0,298	0	1,616	0,003	1,167	0	-2,714	SBP3_ARATH	Probable steroid-binding protein 3 (AtMP3) (Membrane-associated progesterone-binding protein 2) (AtMAPR2)	MP3 MAPR2 At2g24940 F27C12.14	Arabidopsis thaliana (Mouse-ear cress)		3D-structure; Acetylation; Complete proteome; Lipid-binding; Nucleus; Reference proteome; Steroid-binding
SRR122238_TR14039_c1_g1_i5_len=516	PSB7A_ARATH	Q:50-316,H:185-273	78.65%ID	E:4e-44	Proteasome subunit beta type-7-A	.	1	0	1	0	1	0	1	0	0	-1,161	PSB7A_ARATH	Proteasome subunit beta type-7-A (EC 3.4.25.1) (20S proteasome beta subunit B-1) (Proteasome component FA) (Proteasome component FB) (Proteasome subunit beta type-2)	PBB1 PRCFA PRCFB At3g27430 K1G2.15	Arabidopsis thaliana (Mouse-ear cress)		Alternative splicing; Complete proteome; Cytoplasm; Hydrolase; Nucleus; Protease; Proteasome; Reference proteome; Threonine protease; Zymogen
SRR122238_TR16558_c0_g3_i1_len=1747	NA	NA	NA	NA	NA	PF06219.9^DUF1005^Protein of unknown function (DUF1005)^15-247^E:5.4e-104	1	-0,066	1	-1,119	1	-1,932	1	0,169	0	-3,771	.	.	.	.	.	.
SRR122238_TR16558_c0_g4_i1_len=2018	NA	NA	NA	NA	NA	PF06219.9^DUF1005^Protein of unknown function (DUF1005)^1-443^E:7.4e-170	0,18	-0,817	0,43	-0,595	0,038	-1,752	0,312	-0,814	0	-3,062	.	.	.	.	.	.
SRR122238_TR16898_c0_g1_i1_len=553	NA	NA	NA	NA	NA	.	0	8,597	0,688	0,124	0	1,353	0,108	0,508	0	-3,847	.	.	.	.	.	.
SRR122238_TR16898_c0_g1_i8_len=517	NA	NA	NA	NA	NA	.	0,015	0,653	0,461	-0,215	0	0,914	0,487	-0,208	0	-0,985	.	.	.	.	.	.
SRR122238_TR17465_c0_g1_i1_len=1458	CHAC2_RAT	Q:1253-636,H:2-175	35.58%ID	E:5e-27	Putative glutathione-specific gamma-glutamylcyclotransferase 2 {ECO:0000250|UniProtKB:Q9BUX1}	PF04752.9^ChaC^ChaC-like protein^3-124^E:1.8e-21	1	1,326	1	-1,785	1	-2,714	1	-1,592	0	-1,942	CHAC2_RAT	Putative glutathione-specific gamma-glutamylcyclotransferase 2 (Gamma-GCG 2) (EC 2.3.2.-) (Cation transport regulator-like protein 2)	Chac2	Rattus norvegicus (Rat)		Acyltransferase; Complete proteome; Reference proteome; Transferase
SRR122238_TR18135_c1_g1_i4_len=532	NA	NA	NA	NA	NA	.	0,403	-0,347	0,004	-0,903	0	7,496	0,001	6,429	0	-8,324	.	.	.	.	.	.
SRR122238_TR19971_c0_g2_i3_len=1352	RAP23_ARATH	Q:153-932,H:1-245	42.25%ID	E:2e-30	Ethylene-responsive transcription factor RAP2-3	PF00847.17^AP2^AP2 domain^91-140^E:1.4e-14	0,095	-0,59	0,006	-1,232	0,76	0,695	0,282	1,256	0	-1,385	RAP23_ARATH	Ethylene-responsive transcription factor RAP2-3 (Cadmium-induced protein AS30) (Ethylene response factor 72) (ERF72) (Ethylene-responsive element binding protein) (AtEBP) (Protein RELATED TO APETALA2 3) (Related to AP2 3)	RAP2-3 EBP ERF072 At3g16770 MGL6.1 MGL6.24	Arabidopsis thaliana (Mouse-ear cress)		Cadmium; Cell membrane; Complete proteome; Cytoplasm; DNA-binding; Membrane; Nucleus; Reference proteome; Transcription; Transcription regulation
SRR122238_TR25223_c0_g2_i2_len=916	RR10_MESCR	Q:598-176,H:59-197	82.27%ID	E:3e-78	30S ribosomal protein S10, chloroplastic	PF00338.19^Ribosomal_S10^Ribosomal protein S10p/S20e^100-194^E:3e-33	0,798	-0,44	0,189	-1,703	1	0,381	1	0,359	0	-0,978	RR10_MESCR	30S ribosomal protein S10, chloroplastic	RPS10	Mesembryanthemum crystallinum (Common ice plant) (Cryophytum crystallinum)		Chloroplast; Plastid; Ribonucleoprotein; Ribosomal protein; Transit peptide
SRR122238_TR32215_c0_g1_i1_len=1183	DREB3_ARATH	Q:370-888,H:47-221	61.36%ID	E:3e-46	Dehydration-responsive element-binding protein 3	PF00847.17^AP2^AP2 domain^60-110^E:9.6e-13	0,57	0,372	0,257	-0,888	1	0,027	1	-0,289	0	-4,184	DREB3_ARATH	Dehydration-responsive element-binding protein 3 (Protein TINY 2)	DREB3 ERF041 TINY2 At5g11590 F15N18.180	Arabidopsis thaliana (Mouse-ear cress)		Activator; Complete proteome; DNA-binding; Ethylene signaling pathway; Nucleus; Reference proteome; Transcription; Transcription regulation
SRR122238_TR35269_c1_g6_i18_len=710	NA	NA	NA	NA	NA	.	1	-0,21	1	0,207	1	-1,459	0,502	-4,182	0,001	-4,464	.	.	.	.	.	.
SRR122238_TR35386_c0_g1_i4_len=1802	2AAB_ARATH	Q:138-1508,H:131-587	94.31%ID	E:0	Serine/threonine-protein phosphatase 2A 65 kDa regulatory subunit A beta isoform	PF12755.4^Vac14_Fab1_bd^Vacuolar 14 Fab1-binding region^77-142^E:0.00015`PF02985.19^HEAT^HEAT repeat^84-112^E:0.00013`PF13646.3^HEAT_2^HEAT repeats^203-303^E:1.3e-11`PF02985.19^HEAT^HEAT repeat^282-307^E:0.0018	0,629	1,099	0,006	-1,426	0,517	1,125	1	0,513	0	-2,848	2AAB_ARATH	Serine/threonine-protein phosphatase 2A 65 kDa regulatory subunit A beta isoform (AtA beta) (PP2A, subunit A, beta isoform)	PP2AA2 DF1 At3g25800 K13N2.14	Arabidopsis thaliana (Mouse-ear cress)		Acetylation; Alternative splicing; Complete proteome; Reference proteome; Repeat
SRR122238_TR38537_c0_g1_i1_len=1142	CML18_ORYSJ	Q:143-535,H:5-147	53.15%ID	E:9e-44	Probable calcium-binding protein CML18	PF10591.6^SPARC_Ca_bdg^Secreted protein acidic and rich in cysteine Ca binding region^32-106^E:0.00023`PF13833.3^EF-hand_8^EF-hand domain pair^46-68^E:0.00045`PF00036.29^EF-hand_1^EF hand^51-75^E:3.1e-07`PF13499.3^EF-hand_7^EF-hand domain pair^52-109^E:7.3e-16`PF13405.3^EF-hand_6^EF-hand domain^52-77^E:5.6e-06`PF13202.3^EF-hand_5^EF hand^52-71^E:1.1e-07`PF13833.3^EF-hand_8^EF-hand domain pair^69-107^E:5.8e-07`PF00036.29^EF-hand_1^EF hand^84-108^E:1.7e-07`PF13202.3^EF-hand_5^EF hand^91-108^E:3e-06`PF13833.3^EF-hand_8^EF-hand domain pair^98-147^E:1e-07`PF13499.3^EF-hand_7^EF-hand domain pair^120-182^E:1.1e-14`PF00036.29^EF-hand_1^EF hand^121-149^E:3.2e-08`PF13405.3^EF-hand_6^EF-hand domain^121-150^E:5.8e-09`PF13202.3^EF-hand_5^EF hand^122-145^E:6.7e-06`PF13833.3^EF-hand_8^EF-hand domain pair^135-182^E:1.3e-08`PF00036.29^EF-hand_1^EF hand^157-183^E:5.1e-08`PF13202.3^EF-hand_5^EF hand^163-182^E:0.0054	0,027	1,303	0,008	1,417	0,399	0,278	0,312	0,314	0	-1,251	CML18_ORYSJ	Probable calcium-binding protein CML18 (Calmodulin-like protein 18)	CML18 Os05g0223000 LOC_Os05g13580 OJ1532_D06.3	Oryza sativa subsp. japonica (Rice)		Calcium; Complete proteome; Metal-binding; Reference proteome; Repeat
SRR122238_TR38621_c0_g1_i1_len=1282	EXO_ARATH	Q:192-1106,H:16-314	66.67%ID	E:2e-115	Protein EXORDIUM	PF04674.9^Phi_1^Phosphate-induced protein 1 conserved region^41-323^E:1.7e-121	0,692	-0,77	1	-0,977	0,103	-1,81	0,103	-1,653	0	-2,03	EXO_ARATH	Protein EXORDIUM	EXO At4g08950 T3H13.3	Arabidopsis thaliana (Mouse-ear cress)		Apoplast; Complete proteome; Reference proteome; Secreted; Signal
SRR122238_TR42512_c0_g1_i9_len=1150	LGUL_CICAR	Q:953-396,H:1-186	80.65%ID	E:7e-111	Lactoylglutathione lyase	PF00903.22^Glyoxalase^Glyoxalase/Bleomycin resistance protein/Dioxygenase superfamily^78-220^E:1.2e-22	0,506	-0,176	0,412	-0,215	0,032	-0,573	0,183	-0,349	0	-1,485	LGUL_CICAR	Lactoylglutathione lyase (EC 4.4.1.5) (Aldoketomutase) (Glyoxalase I) (Glx I) (Ketone-aldehyde mutase) (Methylglyoxalase) (S-D-lactoylglutathione methylglyoxal lyase)		Cicer arietinum (Chickpea) (Garbanzo)	PATHWAY: Secondary metabolite metabolism; methylglyoxal degradation; (R)-lactate from methylglyoxal: step 1/2.	Lyase; Metal-binding; Zinc
SRR122238_TR45032_c0_g2_i4_len=1279	ERDL7_ARATH	Q:102-1073,H:139-462	79.01%ID	E:0	Sugar transporter ERD6-like 7	PF07690.13^MFS_1^Major Facilitator Superfamily^5-247^E:9.5e-11`PF00083.21^Sugar_tr^Sugar (and other) transporter^16-298^E:2.1e-66	0,629	-1,733	0,629	-1,628	0,56	0,856	0,76	0,606	0	-2,761	ERDL7_ARATH	Sugar transporter ERD6-like 7	At2g48020 T9J23.17	Arabidopsis thaliana (Mouse-ear cress)		Complete proteome; Membrane; Reference proteome; Sugar transport; Transmembrane; Transmembrane helix; Transport
SRR122238_TR48178_c0_g1_i5_len=791	FRL3_ARATH	Q:52-633,H:353-550	56.87%ID	E:3e-59	FRIGIDA-like protein 3	.	1	0	1	0	1	0	1	0	0	-8,909	FRL3_ARATH	FRIGIDA-like protein 3	FRL3 At5g48385 MJE7.2	Arabidopsis thaliana (Mouse-ear cress)		Coiled coil; Complete proteome; Developmental protein; Differentiation; Flowering; Reference proteome
SRR122238_TR50189_c0_g1_i2_len=808	NA	NA	NA	NA	NA	PF14009.3^DUF4228^Domain of unknown function (DUF4228)^1-165^E:1.8e-30	1	-0,49	1	-0,732	0,629	-1,951	0,629	-2,183	0	-2,497	.	.	.	.	.	.
SRR122238_TR50272_c2_g1_i1_len=1847	WRKY7_ARATH	Q:1735-644,H:1-345	48.32%ID	E:3e-90	Probable WRKY transcription factor 7	PF10533.6^Plant_zn_clust^Plant zinc cluster domain^239-288^E:1.2e-17`PF03106.12^WRKY^WRKY DNA -binding domain^292-348^E:3.4e-27	1	0,265	1	0,725	1	0,673	1	0,886	0,001	-6,64	WRKY7_ARATH	Probable WRKY transcription factor 7 (WRKY DNA-binding protein 7)	WRKY7 At4g24240 T22A6.70	Arabidopsis thaliana (Mouse-ear cress)		Complete proteome; DNA-binding; Nucleus; Reference proteome; Transcription; Transcription regulation
SRR122238_TR54054_c0_g1_i1_len=561	NA	NA	NA	NA	NA	.	1	0,703	1	-1,006	1	-2,667	1	0,61	0	-9,325	.	.	.	.	.	.
SRR122238_TR58319_c0_g1_i1_len=1317	ATCA1_ARATH	Q:360-1070,H:29-265	46.84%ID	E:5e-82	Alpha carbonic anhydrase 1, chloroplastic	PF00194.18^Carb_anhydrase^Eukaryotic-type carbonic anhydrase^35-267^E:4.7e-45	1	-0,144	0,286	-0,859	0,009	-0,74	0,005	-0,806	0	-5,311	ATCA1_ARATH	Alpha carbonic anhydrase 1, chloroplastic (AtaCA1) (AtalphaCA1) (EC 4.2.1.1) (Alpha carbonate dehydratase 1)	ACA1 CAH1 At3g52720 F3C22.120	Arabidopsis thaliana (Mouse-ear cress)		Alternative splicing; Chloroplast; Complete proteome; Disulfide bond; Endoplasmic reticulum; Glycoprotein; Lyase; Metal-binding; Plastid; Reference proteome; Signal; Zinc
SRR122238_TR58532_c0_g1_i1_len=988	RL24_PRUAV	Q:119-421,H:1-101	97.03%ID	E:7e-56	60S ribosomal protein L24	PF01246.17^Ribosomal_L24e^Ribosomal protein L24e^3-67^E:6e-30	0,001	-0,774	0,143	-0,352	0,091	0,454	0,388	0,244	0	-9,434	RL24_PRUAV	60S ribosomal protein L24	RPL24	Prunus avium (Cherry) (Cerasus avium)		Ribonucleoprotein; Ribosomal protein
SRR122238_TR58532_c0_g1_i6_len=1042	RL24_PRUAV	Q:119-421,H:1-101	97.03%ID	E:1e-55	60S ribosomal protein L24	PF01246.17^Ribosomal_L24e^Ribosomal protein L24e^3-67^E:6e-30	1	-1,373	1	-0,921	1	0	1	0	0	-5,166	RL24_PRUAV	60S ribosomal protein L24	RPL24	Prunus avium (Cherry) (Cerasus avium)		Ribonucleoprotein; Ribosomal protein
SRR122238_TR62384_c2_g1_i1_len=888	NA	NA	NA	NA	NA	PF11493.5^TSP9^Thylakoid soluble phosphoprotein TSP9^27-105^E:3.5e-30	0,874	-0,052	0,01	-0,677	0,19	-0,287	0,298	-0,228	0	-1,12	.	.	.	.	.	.
SRR122238_TR62412_c0_g1_i1_len=665	RUXG_ARATH	Q:555-316,H:1-80	90%ID	E:1e-44	Probable small nuclear ribonucleoprotein G	.	0,436	-0,234	0,52	-0,187	0,002	1,248	0,005	1,137	0	-3,673	RUXG_ARATH	Probable small nuclear ribonucleoprotein G (snRNP-G) (Sm protein G) (Sm-G) (SmG)	At2g23930 T29E15.13	Arabidopsis thaliana (Mouse-ear cress)		Alternative splicing; Complete proteome; Nucleus; RNA-binding; Reference proteome; Ribonucleoprotein; Spliceosome; mRNA processing; mRNA splicing
SRR122238_TR63567_c0_g1_i1_len=816	PSAG_ARATH	Q:558-136,H:27-160	72.34%ID	E:8e-67	Photosystem I reaction center subunit V, chloroplastic	PF01241.15^PSI_PSAK^Photosystem I psaG / psaK^53-144^E:2.5e-26	1	-0,04	0,76	-0,501	1	-0,191	1	-0,509	0	-3,603	PSAG_ARATH	Photosystem I reaction center subunit V, chloroplastic (PSI-G)	PSAG At1g55670 F20N2.21 F20N2.33 F20N2_3	Arabidopsis thaliana (Mouse-ear cress)		Chloroplast; Complete proteome; Membrane; Photosynthesis; Photosystem I; Plastid; Reference proteome; Thylakoid; Transit peptide; Transmembrane; Transmembrane helix
SRR122238_TR64719_c0_g2_i1_len=821	TIG_ARATH	Q:783-268,H:378-547	61.05%ID	E:3e-57	Trigger factor-like protein TIG, Chloroplastic	.	1	-0,258	0,381	-2,233	1	1,568	1	0	0	-1,667	TIG_ARATH	Trigger factor-like protein TIG, Chloroplastic (EC 5.2.1.8) (Immunophilin TIG) (Peptidyl-prolyl cis-trans isomerase TIG) (PPIase TIG) (Rotamase)	TIG At5g55220 MCO15_17	Arabidopsis thaliana (Mouse-ear cress)		Acetylation; Chaperone; Chloroplast; Complete proteome; Isomerase; Plastid; Reference proteome; Rotamase; Transit peptide
SRR122238_TR68262_c0_g1_i1_len=2996	CBF5_ARATH	Q:2712-1342,H:16-460	82.71%ID	E:0	H/ACA ribonucleoprotein complex subunit 4	PF08068.9^DKCLD^DKCLD (NUC011) domain^50-107^E:5.5e-31`PF01509.15^TruB_N^TruB family pseudouridylate synthase (N terminal domain)^111-227^E:2.1e-19`PF16198.2^TruB_C_2^tRNA pseudouridylate synthase B C-terminal domain^228-294^E:2.7e-22`PF01472.17^PUA^PUA domain^298-370^E:1.8e-21	1	0	1	-0,892	1	0	1	0	0	-1,687	CBF5_ARATH	H/ACA ribonucleoprotein complex subunit 4 (EC 5.4.99.-) (CBF5 homolog) (Dyskerin) (Nopp-140-associated protein of 57 kDa homolog) (AtNAP57) (Nucleolar protein NAP57 homolog)	CBF5 NAP57 At3g57150 F24I3.230	Arabidopsis thaliana (Mouse-ear cress)		Complete proteome; Isomerase; Nucleus; Phosphoprotein; RNA-binding; Reference proteome; Ribonucleoprotein; Ribosome biogenesis; rRNA processing
SRR122238_TR72015_c0_g1_i8_len=594	CAHC_TOBAC	Q:196-594,H:59-192	67.91%ID	E:4e-59	Carbonic anhydrase, chloroplastic	PF00484.16^Pro_CA^Carbonic anhydrase^105-143^E:3.4e-12	1	0	1	0	0,075	-0,876	0,038	-0,992	0	-2,763	CAHC_TOBAC	Carbonic anhydrase, chloroplastic (EC 4.2.1.1) (Carbonate dehydratase)		Nicotiana tabacum (Common tobacco)		Chloroplast; Lyase; Plastid; Transit peptide; Zinc
SRR122238_TR72645_c1_g4_i2_len=987	H2A4_ORYSJ	Q:379-702,H:30-137	92.59%ID	E:7e-49	Probable histone H2A.4	PF00125.21^Histone^Core histone H2A/H2B/H3/H4^20-97^E:6.8e-13`PF16211.2^Histone_H2A_C^C-terminus of histone H2A^100-132^E:1.6e-18	0,564	-0,524	0,16	-1,265	0,002	6,406	1	0	0	-1,371	H2A4_ORYSJ	Probable histone H2A.4	Os05g0461400 LOC_Os05g38640 OJ1281_H05.14 OJ1525_A02.1 OsJ_018061	Oryza sativa subsp. japonica (Rice)		Chromosome; Complete proteome; DNA-binding; Nucleosome core; Nucleus; Reference proteome
SRR122238_TR75012_c0_g1_i1_len=827	NA	NA	NA	NA	NA	.	0,407	-0,376	1	0	0,019	1,093	0,06	0,919	0	-8,263	.	.	.	.	.	.
SRR122238_TR84275_c0_g1_i1_len=700	NA	NA	NA	NA	NA	PF14368.3^LTP_2^Probable lipid transfer^18-102^E:2e-11`PF00234.19^Tryp_alpha_amyl^Protease inhibitor/seed storage/LTP family^30-102^E:1.3e-05	1	0	1	-3,388	1	0	1	2,931	0	-1,924	.	.	.	.	.	.
SRR122238_TR85957_c0_g1_i2_len=618	NA	NA	NA	NA	NA	.	0,01	0,813	0	2,288	0	1,403	0,502	0,323	0	-0,914	.	.	.	.	.	.
SRR122238_TR86861_c0_g2_i1_len=2227	NA	NA	NA	NA	NA	PF00847.17^AP2^AP2 domain^147-197^E:4.1e-11	1	-0,022	0,042	-2,106	0,132	-3,257	0,132	-2,004	0	-2,226	.	.	.	.	.	.
SRR122238_TR87584_c0_g1_i5_len=927	FER3_ARATH	Q:149-604,H:1-155	56.77%ID	E:5e-57	Ferredoxin-3, chloroplastic	PF00111.24^Fer2^2Fe-2S iron-sulfur cluster binding domain^65-138^E:2.6e-21	0,108	0,728	0	2,29	0,004	1,159	0	1,829	0	-8,72	FER3_ARATH	Ferredoxin-3, chloroplastic (AtFd3)	FD3 At2g27510 F10A12.19	Arabidopsis thaliana (Mouse-ear cress)		2Fe-2S; Acetylation; Chloroplast; Complete proteome; Electron transport; Iron; Iron-sulfur; Metal-binding; Plastid; Reference proteome; Transit peptide; Transport
SRR122238_TR87979_c0_g1_i1_len=1736	PP153_ARATH	Q:345-1484,H:206-579	52.37%ID	E:8e-140	Pentatricopeptide repeat-containing protein At2g15690	PF01535.17^PPR^PPR repeat^166-187^E:0.048`PF01535.17^PPR^PPR repeat^192-220^E:0.00032`PF01535.17^PPR^PPR repeat^227-253^E:1.3`PF14432.3^DYW_deaminase^DYW family of nucleic acid deaminases^348-462^E:5.3e-34	0,255	-0,457	0,077	-0,699	0,692	-0,927	0,76	0,604	0	-7,859	PP153_ARATH	Pentatricopeptide repeat-containing protein At2g15690	PCMP-H66 At2g15690 F9O13.24	Arabidopsis thaliana (Mouse-ear cress)		Complete proteome; Reference proteome; Repeat
SRR122238_TR89260_c0_g1_i1_len=1774	AOP1C_ARATH	Q:124-1029,H:13-311	36.57%ID	E:7e-59	Probable 2-oxoglutarate-dependent dioxygenase AOP1	PF14226.3^DIOX_N^non-haem dioxygenase in morphine synthesis N-terminal^27-87^E:7.8e-09`PF03171.17^2OG-FeII_Oxy^2OG-Fe(II) oxygenase superfamily^166-261^E:1.1e-16	1	0,433	1	-1,77	1	-1,208	1	-3,144	0,001	-2,87	AOP1C_ARATH	Probable 2-oxoglutarate-dependent dioxygenase AOP1 (EC 1.14.11.-)	AOP1 AOP1.1 At4g03070 T4I9.5	Arabidopsis thaliana (Mouse-ear cress)		Complete proteome; Dioxygenase; Iron; Metal-binding; Oxidoreductase; Reference proteome
SRR122238_TR89839_c0_g1_i2_len=1239	PURU1_ARATH	Q:449-1165,H:85-323	76.99%ID	E:6e-137	Formyltetrahydrofolate deformylase 1, mitochondrial	PF00551.16^Formyl_trans_N^Formyl transferase^29-206^E:7.4e-35	1	-0,688	0,692	-1,3	0,132	-1,542	0,066	-1,896	0,001	-1,625	PURU1_ARATH	Formyltetrahydrofolate deformylase 1, mitochondrial (EC 3.5.1.10)	PURU1 At5g47435 MNJ7.1	Arabidopsis thaliana (Mouse-ear cress)		Complete proteome; Hydrolase; Mitochondrion; One-carbon metabolism; Photorespiration; Reference proteome; Transit peptide
SRR122238_TR89859_c0_g1_i1_len=1203	NA	NA	NA	NA	NA	.	0,196	-0,578	0,114	-0,791	0,524	-0,613	0,679	-0,408	0	-1,171	.	.	.	.	.	.
SRR1271857_TR1778_c0_g1_i1_len=1301	RS92_ARATH	Q:1046-510,H:1-179	89.94%ID	E:2e-113	40S ribosomal protein S9-2	PF00163.16^Ribosomal_S4^Ribosomal protein S4/S9 N-terminal domain^8-64^E:2.6e-09`PF01479.22^S4^S4 domain^109-152^E:2.7e-11	0,012	-0,647	0,402	-0,217	0,032	0,624	0,247	0,353	0	-1,251	RS92_ARATH	40S ribosomal protein S9-2	RPS9C At5g39850 MYH19.1 MYH19.10	Arabidopsis thaliana (Mouse-ear cress)		Complete proteome; RNA-binding; Reference proteome; Ribonucleoprotein; Ribosomal protein; rRNA-binding
SRR1271857_TR5151_c0_g1_i1_len=696	EP1G_DAUCA	Q:547-74,H:26-183	67.09%ID	E:1e-71	Epidermis-specific secreted glycoprotein EP1	PF01453.21^B_lectin^D-mannose binding lectin^87-172^E:1.1e-26	0,007	-0,998	0	9,106	0	4,411	0	2,312	0	-1,524	EP1G_DAUCA	Epidermis-specific secreted glycoprotein EP1 (52/54 kDa medium protein)	EP1	Daucus carota (Wild carrot)		Direct protein sequencing; Glycoprotein; Lectin; Secreted; Signal
SRR1271857_TR5749_c0_g1_i5_len=2140	ERG24_ARATH	Q:668-303,H:248-369	84.43%ID	E:3e-52	Delta(14)-sterol reductase	PF01222.14^ERG4_ERG24^Ergosterol biosynthesis ERG4/ERG24 family^11-148^E:3.9e-22	1	-0,079	0,694	-0,402	1	0,25	1	-0,239	0,001	-4,455	ERG24_ARATH	Delta(14)-sterol reductase (EC 1.3.1.70) (C-14 sterol reductase) (Protein FACKEL) (Sterol C14-reductase)	FK At3g52940 F8J2_111	Arabidopsis thaliana (Mouse-ear cress)	PATHWAY: Steroid biosynthesis; zymosterol biosynthesis; zymosterol from lanosterol: step 2/6.	Alternative splicing; Complete proteome; Lipid biosynthesis; Lipid metabolism; Membrane; NADP; Oxidoreductase; Reference proteome; Steroid biosynthesis; Steroid metabolism; Sterol biosynthesis; Sterol metabolism; Transmembrane; Transmembrane helix
SRR1271857_TR10879_c1_g2_i1_len=1178	XTH2_SOYBN	Q:1009-230,H:30-282	67.31%ID	E:1e-123	Xyloglucan endotransglucosylase/hydrolase 2 {ECO:0000250|UniProtKB:Q38857}	PF00722.18^Glyco_hydro_16^Glycosyl hydrolases family 16^24-204^E:1.8e-62`PF06955.9^XET_C^Xyloglucan endo-transglycosylase (XET) C-terminus^237-279^E:1.1e-16	0,629	-1,246	1	0,196	0,01	-2,139	0,001	-2,725	0	-2,414	XTH2_SOYBN	Xyloglucan endotransglucosylase/hydrolase 2 (EC 2.4.1.207) (Brassinosteroid-regulated protein BRU1)		Glycine max (Soybean) (Glycine hispida)		Apoplast; Cell wall; Complete proteome; Disulfide bond; Glycoprotein; Glycosidase; Hydrolase; Reference proteome; Secreted; Signal; Transferase

SRR1271857_TR12669_c0_g2_i1_len=503	NA	NA	NA	NA	NA	.	1	0	1	0	1	0	1	0	0	-5,232	.	.	.	.	.	.
SRR1271857_TR15938_c3_g1_i1_len=1535	NA	NA	NA	NA	NA	.	1	-0,635	1	-0,467	1	0	1	0	0	-7,091	.	.	.	.	.	.
SRR1271857_TR18439_c1_g1_i2_len=1600	NA	NA	NA	NA	NA	PF14215.3^bHLH-MYC_N^bHLH-MYC and R2R3-MYB transcription factors N-terminal^65-196^E:5.2e-20	1	0	1	0	1	0	1	0	0	-7,442	.	.	.	.	.	.
SRR1271857_TR18439_c1_g1_i6_len=1589	NA	NA	NA	NA	NA	PF14215.3^bHLH-MYC_N^bHLH-MYC and R2R3-MYB transcription factors N-terminal^13-136^E:2.7e-14	1	-0,189	0,017	-5,88	0,129	-4,976	0,129	-3,804	0	-7,07	.	.	.	.	.	.
SRR1271857_TR21426_c0_g2_i2_len=2739	DADIS_PANGI	Q:72-2456,H:1-763	71.21%ID	E:0	Dammarenediol II synthase	PF13249.3^SQHop_cyclase_N^Squalene-hopene cyclase N-terminal domain^103-361^E:3.8e-39`PF00432.18^Prenyltrans^Prenyltransferase and squalene oxidase repeat^149-191^E:3.4e-09`PF13243.3^SQHop_cyclase_C^Squalene-hopene cyclase C-terminal domain^415-722^E:9.3e-38`PF00432.18^Prenyltrans^Prenyltransferase and squalene oxidase repeat^592-632^E:7.6e-09`PF00432.18^Prenyltrans^Prenyltransferase and squalene oxidase repeat^641-691^E:1.7e-07	0,62	-0,501	1	-0,464	0,798	-0,42	0,239	-1,429	0	-2,351	DADIS_PANGI	Dammarenediol II synthase (EC 4.2.1.125)	PNA DDS	Panax ginseng (Korean ginseng)		Isomerase; Lyase; Membrane; Repeat; Transmembrane; Transmembrane helix
SRR1271857_TR21502_c0_g3_i5_len=1074	VATL_KALDA	Q:837-343,H:1-165	98.18%ID	E:3e-69	V-type proton ATPase 16 kDa proteolipid subunit	PF00137.18^ATP-synt_C^ATP synthase subunit C^17-76^E:6.3e-14`PF00137.18^ATP-synt_C^ATP synthase subunit C^97-155^E:6.7e-21	1	-0,125	1	0,557	1	0,567	1	0,732	0	-6,984	VATL_KALDA	V-type proton ATPase 16 kDa proteolipid subunit (V-ATPase 16 kDa proteolipid subunit) (V-type H(+)-ATPase 16 kDa subunit) (Vacuolar proton pump 16 kDa proteolipid subunit)		Kalanchoe daigremontiana (Devil's backbone) (Bryophyllum daigremontianum)		Hydrogen ion transport; Ion transport; Membrane; Transmembrane; Transmembrane helix; Transport; Vacuole
SRR1271857_TR22544_c0_g3_i1_len=725	NA	NA	NA	NA	NA	.	0,719	0,138	0,108	-0,542	0,352	-0,745	1	-0,005	0	-4,11	.	.	.	.	.	.
SRR1271857_TR27540_c2_g2_i1_len=649	NA	NA	NA	NA	NA	.	0,732	-0,741	0,254	-3,635	1	1,419	1	0,767	0	-3,109	.	.	.	.	.	.
SRR1271857_TR35081_c0_g2_i1_len=2246	GSH1_SOLLC	Q:248-1984,H:1-523	74.31%ID	E:0	Glutamate--cysteine ligase, chloroplastic	PF04107.10^GCS2^Glutamate-cysteine ligase family 2(GCS2)^147-391^E:7.3e-81	0,793	-0,201	0,707	0,371	0,395	0,587	0,25	0,782	0	-8,02	GSH1_SOLLC	Glutamate--cysteine ligase, chloroplastic (EC 6.3.2.2) (Gamma-ECS) (GCS) (Gamma-glutamylcysteine synthetase)	GSH1	Solanum lycopersicum (Tomato) (Lycopersicon esculentum)	PATHWAY: Sulfur metabolism; glutathione biosynthesis; glutathione from L-cysteine and L-glutamate: step 1/2.	ATP-binding; Chloroplast; Complete proteome; Disulfide bond; Glutathione biosynthesis; Ligase; Nucleotide-binding; Plastid; Reference proteome; Transit peptide
SRR1271857_TR38062_c0_g3_i1_len=523	NA	NA	NA	NA	NA	.	0,121	-0,845	0,029	-1,097	1	0,347	1	0,069	0	-1,392	.	.	.	.	.	.
SRR1271857_TR43231_c0_g3_i4_len=3639	RVE6_ARATH	Q:3555-2605,H:1-314	58.01%ID	E:7e-84	Protein REVEILLE 6	.	0,729	-0,162	0,906	0,119	1	0,02	0,783	-0,176	0	-2,546	RVE6_ARATH	Protein REVEILLE 6	RVE6 At5g52660 F6N7.15	Arabidopsis thaliana (Mouse-ear cress)		Alternative splicing; Complete proteome; DNA-binding; Nucleus; Reference proteome; Transcription; Transcription regulation
SRR1271858_TR1911_c0_g2_i2_len=609	NA	NA	NA	NA	NA	PF13650.3^Asp_protease_2^Aspartyl protease^97-192^E:5.4e-06	0,798	0,544	1	-0,584	1	1,001	1	0,09	0,001	-3,822	.	.	.	.	.	.
SRR1271858_TR2802_c0_g1_i1_len=1319	POL3_DROME	Q:1312-644,H:370-590	39.56%ID	E:3e-37	Retrovirus-related Pol polyprotein from transposon 17.6	.	0,38	0,49	1	-0,776	0,629	1,004	0,629	1,168	0,001	-4,458	POL3_DROME	Retrovirus-related Pol polyprotein from transposon 17.6 [Includes: Protease (EC 3.4.23.-); Reverse transcriptase (EC 2.7.7.49); Endonuclease]	pol	Drosophila melanogaster (Fruit fly)		Aspartyl protease; Endonuclease; Hydrolase; Nuclease; Nucleotidyltransferase; Protease; RNA-directed DNA polymerase; Transferase; Transposable element
SRR1271858_TR4441_c0_g1_i1_len=626	NA	NA	NA	NA	NA	.	0,013	-1,544	0,009	-5,963	1	0,845	1	1,009	0,001	-1,18	.	.	.	.	.	.
SRR1271858_TR25211_c0_g2_i1_len=675	NA	NA	NA	NA	NA	.	0	1,292	0	6,744	0	8,539	0,129	4,207	0	-2,021	.	.	.	.	.	.
SRR1271858_TR30562_c1_g1_i7_len=3536	GCL1_ARATH	Q:2318-3229,H:164-433	64.14%ID	E:4e-124	LanC-like protein GCL1	PF05147.10^LANC_like^Lanthionine synthetase C-like protein^71-156^E:1.6e-14	1	0,4	1	0,586	1	0	1	0	0	-7,855	GCL1_ARATH	LanC-like protein GCL1 (G protein-coupled receptor 2-like protein 1) (Protein GCR2-like 1)	GCL1 At5g65280 MQN23.23	Arabidopsis thaliana (Mouse-ear cress)		Complete proteome; Reference proteome
SRR1271858_TR32754_c0_g2_i1_len=840	NA	NA	NA	NA	NA	.	0,62	0,732	0	3,277	0	3,166	0,001	2,819	0	-2,698	.	.	.	.	.	.
SRR1271858_TR41562_c0_g2_i1_len=2082	ASPG1_ARATH	Q:594-1787,H:177-500	28.07%ID	E:2e-26	Protein ASPARTIC PROTEASE IN GUARD CELL 1	PF14543.3^TAXi_N^Xylanase inhibitor N-terminal^81-263^E:4.1e-28`PF00026.20^Asp^Eukaryotic aspartyl protease^88-488^E:2.5e-07`PF14541.3^TAXi_C^Xylanase inhibitor C-terminal^307-487^E:5.7e-29	1	0,789	1	2,294	1	0,4	1	0,61	0	-2,923	ASPG1_ARATH	Protein ASPARTIC PROTEASE IN GUARD CELL 1 (AtASPG1) (EC 3.4.23.-)	ASPG1 At3g18490 MYF24.22	Arabidopsis thaliana (Mouse-ear cress)		Aspartyl protease; Complete proteome; DNA-binding; Disulfide bond; Endoplasmic reticulum; Hydrolase; Protease; Reference proteome; Signal
SRR1271858_TR46393_c0_g2_i1_len=720	NA	NA	NA	NA	NA	.	0,221	-0,594	0,644	-0,182	0,154	0,767	0,87	0,16	0,001	-1,321	.	.	.	.	.	.
SRR1271858_TR55841_c0_g2_i5_len=1175	SC31B_ARATH	Q:171-773,H:903-1104	65.85%ID	E:2e-60	Protein transport protein SEC31 homolog B {ECO:0000305}	PF07304.8^SRA1^Steroid receptor RNA activator (SRA1)^49-187^E:9.8e-06	0,719	-0,327	0,014	-0,892	0,052	1,478	0,111	1,204	0	-1,493	SC31B_ARATH	Protein transport protein SEC31 homolog B (SEC31-like protein B)	SEC31B At3g63460 MAA21.90	Arabidopsis thaliana (Mouse-ear cress)		Alternative splicing; Complete proteome; ER-Golgi transport; Endoplasmic reticulum; Golgi apparatus; Phosphoprotein; Protein transport; Reference proteome; Repeat; Transport; WD repeat
SRR1271858_TR57655_c0_g1_i1_len=1188	PPSP1_ARATH	Q:959-306,H:16-236	56.56%ID	E:3e-94	Inorganic pyrophosphatase 1	PF06888.9^Put_Phosphatase^Putative Phosphatase^7-235^E:8e-90`PF12710.4^HAD^haloacid dehalogenase-like hydrolase^7-183^E:4.5e-09	0,895	0,134	0,105	0,988	0,006	1,429	0,025	1,215	0	-7,217	PPSP1_ARATH	Inorganic pyrophosphatase 1 (AtPPsPase1) (PPi phosphatase 1) (Pyrophosphate-specific phosphatase 1) (EC 3.6.1.1) (Protein PHOSPHATE STARVATION-INDUCED GENE 2) (AtPS2)	PS2 At1g73010 F3N23.21	Arabidopsis thaliana (Mouse-ear cress)		Complete proteome; Hydrolase; Magnesium; Metal-binding; Reference proteome
SRR1271858_TR58517_c0_g1_i1_len=528	NA	NA	NA	NA	NA	.	1	0	1	0	1	1,335	1	0	0	-2,087	.	.	.	.	.	.
SRR1271858_TR68514_c0_g1_i1_len=713	NA	NA	NA	NA	NA	.	0,774	0,126	0	2,337	0,03	0,709	0,01	0,821	0	-1,268	.	.	.	.	.	.
SRR1271859_TR156_c0_g1_i2_len=788	RUXG_ARATH	Q:249-488,H:1-80	90%ID	E:5e-44	Probable small nuclear ribonucleoprotein G	.	0,581	-0,456	0,412	-0,52	0,189	1,841	1	0,771	0	-8,63	RUXG_ARATH	Probable small nuclear ribonucleoprotein G (snRNP-G) (Sm protein G) (Sm-G) (SmG)	At2g23930 T29E15.13	Arabidopsis thaliana (Mouse-ear cress)		Alternative splicing; Complete proteome; Nucleus; RNA-binding; Reference proteome; Ribonucleoprotein; Spliceosome; mRNA processing; mRNA splicing
SRR1271859_TR1028_c0_g1_i2_len=1019	NA	NA	NA	NA	NA	.	1	0	1	0,716	1	0	1	0	0	-6,707	.	.	.	.	.	.
SRR1271859_TR2483_c1_g1_i9_len=1301	RAE1C_ARATH	Q:193-798,H:15-216	93.07%ID	E:7e-117	Ras-related protein RABE1c	PF00025.18^Arf^ADP-ribosylation factor family^16-163^E:2.1e-15`PF00071.19^Ras^Ras family^17-178^E:1.6e-66`PF08477.10^Roc^Ras of Complex, Roc, domain of DAPkinase^17-132^E:4.6e-35`PF04670.9^Gtr1_RagA^Gtr1/RagA G protein conserved region^17-132^E:4.5e-05`PF01926.20^MMR_HSR1^50S ribosome-binding GTPase^18-128^E:4.7e-05	0,811	0,335	0,16	-0,589	0	8,307	0	7,859	0	-3,996	RAE1C_ARATH	Ras-related protein RABE1c (AtRABE1c) (Ras-related protein Ara-3) (Ras-related protein Rab8A) (AtRab8A)	RABE1C ARA-3 RAB8A At3g46060 F12M12.30	Arabidopsis thaliana (Mouse-ear cress)		Cell membrane; Complete proteome; GTP-binding; Golgi apparatus; Lipoprotein; Membrane; Nucleotide-binding; Prenylation; Protein transport; Reference proteome; Transport
SRR1271859_TR6453_c0_g2_i1_len=768	PDCB3_ARATH	Q:535-762,H:15-90	69.74%ID	E:1e-34	PLASMODESMATA CALLOSE-BINDING PROTEIN 3	.	0,282	-0,969	0,623	0,434	0,226	2,062	0,629	1,041	0	-2,266	PDCB3_ARATH	PLASMODESMATA CALLOSE-BINDING PROTEIN 3 (AtPDCB3)	PDCB3 At1g18650 F25I16.1	Arabidopsis thaliana (Mouse-ear cress)		Cell junction; Cell membrane; Complete proteome; Disulfide bond; GPI-anchor; Glycoprotein; Lipoprotein; Membrane; Reference proteome; Signal
SRR1271859_TR9840_c0_g1_i2_len=2451	ENO_SOLLC	Q:1605-277,H:1-444	93.24%ID	E:0	Enolase	PF03952.13^Enolase_N^Enolase, N-terminal domain^4-139^E:2.7e-56`PF00113.19^Enolase_C^Enolase, C-terminal TIM barrel domain^148-439^E:3.7e-161`PF13378.3^MR_MLE_C^Enolase C-terminal domain-like^275-403^E:9.1e-09`PF07476.8^MAAL_C^Methylaspartate ammonia-lyase C-terminus^297-400^E:1.3e-05	0,161	-0,344	0,531	-0,167	0,14	0,414	0,218	0,352	0	-2,1	ENO_SOLLC	Enolase (EC 4.2.1.11) (2-phospho-D-glycerate hydro-lyase) (2-phosphoglycerate dehydratase)	PGH1	Solanum lycopersicum (Tomato) (Lycopersicon esculentum)	PATHWAY: Carbohydrate degradation; glycolysis; pyruvate from D-glyceraldehyde 3-phosphate: step 4/5.	Complete proteome; Cytoplasm; Glycolysis; Lyase; Magnesium; Metal-binding; Reference proteome
SRR1271859_TR10078_c0_g1_i3_len=1503	DPM1_HUMAN	Q:697-1215,H:88-260	69.94%ID	E:9e-79	Dolichol-phosphate mannosyltransferase subunit 1	PF00535.23^Glycos_transf_2^Glycosyl transferase family 2^17-124^E:1.7e-19`PF13641.3^Glyco_tranf_2_3^Glycosyltransferase like family 2^23-155^E:1.4e-06`PF13506.3^Glyco_transf_21^Glycosyl transferase family 21^29-124^E:3.7e-07	1	0	1	-0,356	1	0	1	0	0	-7,094	DPM1_HUMAN	Dolichol-phosphate mannosyltransferase subunit 1 (EC 2.4.1.83) (Dolichol-phosphate mannose synthase subunit 1) (DPM synthase subunit 1) (Dolichyl-phosphate beta-D-mannosyltransferase subunit 1) (Mannose-P-dolichol synthase subunit 1) (MPD synthase subunit 1)	DPM1	Homo sapiens (Human)	PATHWAY: Protein modification; protein glycosylation.	Acetylation; Complete proteome; Congenital disorder of glycosylation; Congenital muscular dystrophy; Disease mutation; Dystroglycanopathy; Endoplasmic reticulum; Glycosyltransferase; Phosphoprotein; Reference proteome; Transferase
SRR1271859_TR12356_c0_g2_i1_len=584	MIRA_SYNDU	Q:44-379,H:103-216	59.13%ID	E:6e-37	Miraculin	PF00197.15^Kunitz_legume^Trypsin and protease inhibitor^2-108^E:1.3e-26	0,003	0,755	0	1,59	0	2,085	0	1,819	0	-1,733	MIRA_SYNDU	Miraculin (MIR)		Synsepalum dulcificum (Miracle fruit) (Richadella dulcifica)		Direct protein sequencing; Disulfide bond; Glycoprotein; Signal; Taste-modifying protein
SRR1271859_TR17834_c0_g1_i1_len=512	NA	NA	NA	NA	NA	.	0	1,376	0	8,288	0,066	5,219	1	2,21	0	-2,11	.	.	.	.	.	.
SRR1271859_TR20089_c0_g1_i3_len=1800	SPDE_COFAR	Q:1064-657,H:116-251	94.12%ID	E:3e-84	Spermidine synthase	PF01564.14^Spermine_synth^Spermine/spermidine synthase^1-120^E:6.6e-39	1	-0,498	0,692	0,921	1	-0,169	1	-0,65	0	-4,839	SPDE_COFAR	Spermidine synthase (SPDSY) (EC 2.5.1.16) (Putrescine aminopropyltransferase)		Coffea arabica (Arabian coffee)	PATHWAY: Amine and polyamine biosynthesis; spermidine biosynthesis; spermidine from putrescine: step 1/1.	Polyamine biosynthesis; Spermidine biosynthesis; Transferase
SRR1271859_TR20141_c0_g3_i2_len=1412	SKP1A_ARATH	Q:1224-760,H:2-160	77.99%ID	E:5e-69	SKP1-like protein 1A	PF03931.12^Skp1_POZ^Skp1 family, tetramerisation domain^5-64^E:1.5e-30`PF01466.16^Skp1^Skp1 family, dimerisation domain^108-155^E:2e-30	0,933	0,025	0,873	0,061	0,004	0,869	0,028	0,678	0	-2,409	SKP1A_ARATH	SKP1-like protein 1A (SKP1-like 1) (UFO-binding protein 1)	SKP1A ASK1 SKP1 UIP1 At1g75950 T4O12.17	Arabidopsis thaliana (Mouse-ear cress)	PATHWAY: Protein modification; protein ubiquitination.	3D-structure; Auxin signaling pathway; Chromosome partition; Complete proteome; Cytoplasm; Cytoskeleton; Developmental protein; Ethylene signaling pathway; Host-virus interaction; Nucleus; Reference proteome; Ubl conjugation pathway
SRR1271859_TR23354_c0_g2_i1_len=1562	NA	NA	NA	NA	NA	PF00257.16^Dehydrin^Dehydrin^10-178^E:2e-43	0	2,197	0	4,675	0	5,109	0	2,946	0	-2,756	.	.	.	.	.	.
SRR1271859_TR24255_c0_g1_i2_len=1652	G3PC_TOBAC	Q:1442-468,H:1-325	93.54%ID	E:0	Glyceraldehyde-3-phosphate dehydrogenase, cytosolic	PF00044.21^Gp_dh_N^Glyceraldehyde 3-phosphate dehydrogenase, NAD binding domain^6-156^E:1.1e-55`PF02800.17^Gp_dh_C^Glyceraldehyde 3-phosphate dehydrogenase, C-terminal domain^161-318^E:2.5e-72	0,152	-0,31	0,44	0,171	0,826	0,048	0,54	0,131	0	-7,135	G3PC_TOBAC	Glyceraldehyde-3-phosphate dehydrogenase, cytosolic (EC 1.2.1.12) (Fragment)	GAPC	Nicotiana tabacum (Common tobacco)	PATHWAY: Carbohydrate degradation; glycolysis; pyruvate from D-glyceraldehyde 3-phosphate: step 1/5.	Cytoplasm; Glycolysis; NAD; Oxidoreductase
SRR1271859_TR27396_c0_g1_i2_len=1056	Y575_SYNY3	Q:538-981,H:40-183	44%ID	E:6e-29	Thylakoid membrane protein slr0575	PF11016.5^DUF2854^Protein of unknown function (DUF2854)^114-264^E:5.9e-50	1	1,691	1	0	1	1,397	1	-0,243	0	-8,037	Y575_SYNY3	Thylakoid membrane protein slr0575	slr0575	Synechocystis sp. (strain PCC 6803 / Kazusa)		Complete proteome; Membrane; Reference proteome; Thylakoid; Transmembrane; Transmembrane helix
SRR1271859_TR27652_c0_g1_i1_len=517	NA	NA	NA	NA	NA	.	0,44	-0,914	0,732	0,584	0,005	6,146	0	7,215	0,001	-6,625	.	.	.	.	.	.
SRR1271859_TR29534_c0_g1_i11_len=2746	FLA1_ARATH	Q:178-1107,H:22-331	67.52%ID	E:2e-139	Fasciclin-like arabinogalactan protein 1	PF02469.19^Fasciclin^Fasciclin domain^73-143^E:0.0002`PF02469.19^Fasciclin^Fasciclin domain^235-363^E:1.9e-13	0,917	-0,068	0,623	-0,318	0,014	-1,067	0,01	-1,173	0,001	-3,044	FLA1_ARATH	Fasciclin-like arabinogalactan protein 1	FLA1 At5g55730 MDF20.17	Arabidopsis thaliana (Mouse-ear cress)		Cell membrane; Complete proteome; GPI-anchor; Glycoprotein; Lipoprotein; Membrane; Proteoglycan; Reference proteome; Repeat; Signal
SRR1271859_TR33494_c0_g1_i2_len=749	URM12_ARATH	Q:616-362,H:15-99	81.18%ID	E:1e-42	Ubiquitin-related modifier 1 homolog 2 {ECO:0000255|HAMAP-Rule:MF_03048}	PF09138.8^Urm1^Urm1 (Ubiquitin related modifier)^33-129^E:5.3e-36	0,718	-0,24	0,833	-0,113	0,43	0,706	0,545	0,602	0	-6,475	URM12_ARATH	Ubiquitin-related modifier 1 homolog 2	URM1-2 At3g61113 T20K12	Arabidopsis thaliana (Mouse-ear cress)	PATHWAY: tRNA modification; 5-methoxycarbonylmethyl-2-thiouridine-tRNA biosynthesis. {ECO:0000255|HAMAP-Rule:MF_03048}.	Complete proteome; Cytoplasm; Isopeptide bond; Reference proteome; Ubl conjugation pathway; tRNA processing
SRR1271859_TR35239_c0_g1_i1_len=917	SYP_PLAF7	Q:96-755,H:516-746	44.16%ID	E:7e-52	Proline--tRNA ligase	PF03129.17^HGTP_anticodon^Anticodon binding domain^21-117^E:4.8e-22`PF09180.8^ProRS-C_1^Prolyl-tRNA synthetase, C-terminal^144-217^E:2.8e-26	1	1,263	1	0	1	0,637	1	0	0,001	-1,6	SYP_PLAF7	Proline--tRNA ligase (EC 6.1.1.15) (Prolyl-tRNA synthetase) (ProRS)	proRS PFL0670c	Plasmodium falciparum (isolate 3D7)		3D-structure; ATP-binding; Aminoacyl-tRNA synthetase; Complete proteome; Cytoplasm; Ligase; Nucleotide-binding; Protein biosynthesis; Reference proteome
SRR1271859_TR36883_c0_g1_i9_len=911	DEF1A_SOLLC	Q:827-231,H:68-275	79.33%ID	E:7e-112	Peptide deformylase 1A, chloroplastic	PF01327.18^Pep_deformylase^Polypeptide deformylase^34-189^E:1.3e-46	0,299	-1,484	1	-0,133	0,517	0,946	1	-0,249	0,001	-6,633	DEF1A_SOLLC	Peptide deformylase 1A, chloroplastic (PDF 1A) (EC 3.5.1.88) (Polypeptide deformylase)	PDF1A	Solanum lycopersicum (Tomato) (Lycopersicon esculentum)		Chloroplast; Complete proteome; Hydrolase; Metal-binding; Plastid; Protein biosynthesis; Reference proteome; Transit peptide; Zinc
SRR1271859_TR37765_c1_g2_i1_len=2034	ARF2_ORYSJ	Q:1865-1323,H:1-181	99.45%ID	E:2e-124	ADP-ribosylation factor 2	PF00025.18^Arf^ADP-ribosylation factor family^5-177^E:3e-80`PF09439.7^SRPRB^Signal recognition particle receptor beta subunit^16-143^E:8.5e-14`PF08477.10^Roc^Ras of Complex, Roc, domain of DAPkinase^19-129^E:1.1e-13`PF04670.9^Gtr1_RagA^Gtr1/RagA G protein conserved region^19-142^E:2.5e-10`PF01926.20^MMR_HSR1^50S ribosome-binding GTPase^19-122^E:4.7e-05`PF00071.19^Ras^Ras family^20-172^E:1e-12	1	-0,404	1	0,653	1	0,059	1	-0,095	0	-3,934	ARF2_ORYSJ	ADP-ribosylation factor 2	ARF Os05g0489600 LOC_Os05g41060 OJ1119_H02.17	Oryza sativa subsp. japonica (Rice)		Complete proteome; ER-Golgi transport; GTP-binding; Golgi apparatus; Lipoprotein; Myristate; Nucleotide-binding; Protein transport; Reference proteome; Transport
SRR1271859_TR41148_c1_g1_i1_len=697	NA	NA	NA	NA	NA	.	0,721	-0,178	0,002	-1,203	0,249	-0,521	0,058	-0,838	0,001	-1,221	.	.	.	.	.	.
SRR1271859_TR42956_c0_g3_i8_len=1513	XTH2_SOYBN	Q:310-1089,H:30-282	67.31%ID	E:2e-122	Xyloglucan endotransglucosylase/hydrolase 2 {ECO:0000250|UniProtKB:Q38857}	PF00722.18^Glyco_hydro_16^Glycosyl hydrolases family 16^29-209^E:4.7e-63`PF06955.9^XET_C^Xyloglucan endo-transglycosylase (XET) C-terminus^242-284^E:1.2e-16	1	-1,022	1	0,135	0,226	-2,007	0,226	-1,834	0	-2,025	XTH2_SOYBN	Xyloglucan endotransglucosylase/hydrolase 2 (EC 2.4.1.207) (Brassinosteroid-regulated protein BRU1)		Glycine max (Soybean) (Glycine hispida)		Apoplast; Cell wall; Complete proteome; Disulfide bond; Glycoprotein; Glycosidase; Hydrolase; Reference proteome; Secreted; Signal; Transferase
SRR1271859_TR43217_c0_g2_i4_len=1598	NA	NA	NA	NA	NA	.	0,847	-0,114	0,35	0,557	0,763	0,24	1	0,134	0	-5,573	.	.	.	.	.	.
SRR1271859_TR44622_c0_g3_i1_len=596	NA	NA	NA	NA	NA	.	0,524	-0,524	0,333	0,574	0,748	0,223	0,86	-0,276	0	-7,403	.	.	.	.	.	.
SRR1271859_TR47131_c1_g2_i3_len=1606	NA	NA	NA	NA	NA	.	1	-0,007	0,25	-0,676	0,299	1,457	0,692	0,876	0	-1,944	.	.	.	.	.	.
SRR342017_TR4651_c0_g2_i3_len=1236	CUL1_ARATH	Q:2-985,H:411-738	78.05%ID	E:2e-173	Cullin-1	PF00888.19^Cullin^Cullin family^2-231^E:6.8e-77`PF10557.6^Cullin_Nedd8^Cullin protein neddylation domain^258-320^E:3.1e-26	1	-0,015	0,025	-1,717	0,226	1,775	0,132	2,036	0	-1,995	CUL1_ARATH	Cullin-1	CUL1 At4g02570 T10P11.26	Arabidopsis thaliana (Mouse-ear cress)	PATHWAY: Protein modification; protein ubiquitination.	Auxin signaling pathway; Cell cycle; Complete proteome; Cytoplasm; Cytoskeleton; Developmental protein; Ethylene signaling pathway; Isopeptide bond; Nucleus; Reference proteome; Ubl conjugation; Ubl conjugation pathway
SRR342017_TR7034_c0_g1_i6_len=853	NA	NA	NA	NA	NA	.	1	0	1	0	1	0	1	0	0	-2,316	.	.	.	.	.	.
SRR342017_TR7330_c1_g4_i2_len=896	BT3L4_DANRE	Q:799-434,H:1-123	58.54%ID	E:3e-41	Transcription factor BTF3 homolog 4	PF01849.15^NAC^NAC domain^36-91^E:5.1e-19	0,629	1,912	1	0,431	0,502	2,802	1	0,948	0	-6,43	BT3L4_DANRE	Transcription factor BTF3 homolog 4 (Basic transcription factor 3-like 4)	btf3l4	Danio rerio (Zebrafish) (Brachydanio rerio)		Complete proteome; Reference proteome
SRR342017_TR7809_c1_g2_i1_len=2995	TCPD_ARATH	Q:1704-2741,H:184-528	92.2%ID	E:0	T-complex protein 1 subunit delta {ECO:0000303|PubMed:11599560}	PF00118.21^Cpn60_TCP1^TCP-1/cpn60 chaperonin family^10-361^E:3.3e-97	0,24	-0,508	0,005	-1,633	0,211	-0,714	0,043	-1,244	0	-1,099	TCPD_ARATH	T-complex protein 1 subunit delta (TCP-1-delta) (CCT-delta) (Chaperonin CCT4)	CCT4 At3g18190 MRC8	Arabidopsis thaliana (Mouse-ear cress)		ATP-binding; Chaperone; Complete proteome; Cytoplasm; Nucleotide-binding; Reference proteome
SRR342017_TR8701_c1_g2_i2_len=1867	HMG13_ARATH	Q:383-1537,H:58-442	56.85%ID	E:4e-94	High mobility group B protein 13	PF09011.7^HMG_box_2^HMG-box domain^196-257^E:1.1e-09`PF00505.16^HMG_box^HMG (high mobility group) box^198-259^E:4e-17`PF09011.7^HMG_box_2^HMG-box domain^312-379^E:1e-13`PF00505.16^HMG_box^HMG (high mobility group) box^314-380^E:1.4e-18`PF09011.7^HMG_box_2^HMG-box domain^439-510^E:1.8e-11`PF00505.16^HMG_box^HMG (high mobility group) box^442-510^E:1.3e-17`PF08073.9^CHDNT^CHDNT (NUC034) domain^451-485^E:0.00027	0,058	-0,875	0,016	-2,704	0,692	-0,738	0,692	-0,627	0	-0,998	HMG13_ARATH	High mobility group B protein 13 (Nucleosome/chromatin assembly factor group D 13)	HMGB13 NFD13 At4g11080 F2P3.3 T22B4.60	Arabidopsis thaliana (Mouse-ear cress)		Complete proteome; DNA-binding; Nucleus; Reference proteome; Repeat
SRR342017_TR9822_c1_g1_i1_len=845	UEV1C_ARATH	Q:624-187,H:1-145	89.73%ID	E:2e-88	Ubiquitin-conjugating enzyme E2 variant 1C	PF00179.23^UQ_con^Ubiquitin-conjugating enzyme^43-136^E:8.3e-15	0,407	0,375	0,881	0,053	0	2,757	0	3,479	0	-4,15	UEV1C_ARATH	Ubiquitin-conjugating enzyme E2 variant 1C (Ubc enzyme variant 1C) (Protein MMS ZWEI HOMOLOG 3)	UEV1C MMZ3 At2g36060 F11F19.3	Arabidopsis thaliana (Mouse-ear cress)		Alternative splicing; Complete proteome; Reference proteome
SRR342017_TR12675_c1_g2_i3_len=886	GH33_ARATH	Q:288-1,H:446-541	79.17%ID	E:8e-43	Indole-3-acetic acid-amido synthetase GH3.3	.	1	0	1	-1,432	1	0	1	0	0	-2,419	GH33_ARATH	Indole-3-acetic acid-amido synthetase GH3.3 (EC 6.3.2.-) (Auxin-responsive GH3-like protein 3) (AtGH3-3)	GH3.3 At2g23170 T20D16.20	Arabidopsis thaliana (Mouse-ear cress)		Complete proteome; Ligase; Reference proteome
SRR342017_TR13518_c0_g1_i1_len=574	NUD25_ARATH	Q:51-362,H:71-175	72.64%ID	E:3e-49	Nudix hydrolase 25	.	0,16	1,24	0,002	6,311	0,066	5,298	0,002	6,299	0,001	-6,547	NUD25_ARATH	Nudix hydrolase 25 (AtNUDT25) (EC 3.6.1.17) (Bis(5'-nucleosyl)-tetraphosphatase (asymmetrical))	NUDT25 NUDX25 At1g30110 T2H7.9	Arabidopsis thaliana (Mouse-ear cress)		Complete proteome; Hydrolase; Manganese; Metal-binding; Reference proteome
SRR342017_TR17264_c1_g2_i1_len=762	14KD_DAUCA	Q:277-528,H:54-137	70.24%ID	E:2e-22	14 kDa proline-rich protein DC2.15	.	1	-1,659	1	-1,942	0,129	5,045	0,502	3,937	0	-0,961	14KD_DAUCA	14 kDa proline-rich protein DC2.15		Daucus carota (Wild carrot)		Membrane; Signal; Transmembrane; Transmembrane helix
SRR342017_TR17850_c1_g1_i1_len=1349	FLA8_ARATH	Q:1349-627,H:94-334	75.93%ID	E:5e-115	Fasciclin-like arabinogalactan protein 8	PF02469.19^Fasciclin^Fasciclin domain^105-235^E:5.2e-20	1	-0,346	0,692	-0,907	1	-0,478	1	0,158	0	-8,788	FLA8_ARATH	Fasciclin-like arabinogalactan protein 8 (AtAGP8)	FLA8 At2g45470 F4L23.2	Arabidopsis thaliana (Mouse-ear cress)		Cell membrane; Complete proteome; GPI-anchor; Glycoprotein; Lipoprotein; Membrane; Proteoglycan; Reference proteome; Repeat; Signal
SRR342017_TR22832_c0_g1_i1_len=694	RS212_ARATH	Q:544-302,H:1-81	81.48%ID	E:4e-44	40S ribosomal protein S21-2	.	0,729	0,091	0,042	-0,506	0,11	0,396	1	-0,005	0	-1,545	RS212_ARATH	40S ribosomal protein S21-2	RPS21C At5g27700 T1G16.30	Arabidopsis thaliana (Mouse-ear cress)		Acetylation; Complete proteome; Reference proteome; Ribonucleoprotein; Ribosomal protein
SRR342017_TR23003_c0_g5_i3_len=1212	DLDH1_ARATH	Q:554-1105,H:324-507	88.04%ID	E:2e-109	Dihydrolipoyl dehydrogenase 1, mitochondrial	PF11543.5^UN_NPL4^Nuclear pore localisation protein NPL4^1-84^E:2.5e-07	0,5	0,744	0,886	-0,214	0,84	0,253	1	0,058	0	-3,074	DLDH1_ARATH	Dihydrolipoyl dehydrogenase 1, mitochondrial (AtmLPD1) (mtLPD1) (EC 1.8.1.4) (Dihydrolipoamide dehydrogenase 1) (Glycine cleavage system L protein 1) (Pyruvate dehydrogenase complex E3 subunit 1) (E3-1) (PDC-E3 1)	LPD1 At1g48030 F21D18.28 T2J15.6	Arabidopsis thaliana (Mouse-ear cress)		Complete proteome; Disulfide bond; FAD; Flavoprotein; Mitochondrion; NAD; Oxidoreductase; Redox-active center; Reference proteome; Transit peptide
SRR342017_TR23398_c0_g3_i1_len=3171	ZDHC1_ARATH	Q:2350-578,H:1-594	61.86%ID	E:3e-172	Probable protein S-acyltransferase 22	PF01529.17^zf-DHHC^DHHC palmitoyltransferase^180-312^E:1.3e-29	0,719	-0,307	0,066	-1,694	0,517	-1,052	0,517	-0,989	0,001	-2,162	ZDHC1_ARATH	Probable protein S-acyltransferase 22 (EC 2.3.1.225) (Probable palmitoyltransferase At1g69420) (Zinc finger DHHC domain-containing protein At1g69420)	PAT22 At1g69420 F10D13.9 F23O10.1	Arabidopsis thaliana (Mouse-ear cress)		Acyltransferase; Cell membrane; Complete proteome; Cytoplasmic vesicle; Membrane; Metal-binding; Reference proteome; Transferase; Transmembrane; Transmembrane helix; Zinc; Zinc-finger
SRR342017_TR23485_c1_g3_i9_len=1475	7OMT_PAPSO	Q:225-1262,H:2-354	38.72%ID	E:2e-66	(R,S)-reticuline 7-O-methyltransferase {ECO:0000303|PubMed:14675446}	PF08100.8^Dimerisation^Dimerisation domain^32-76^E:1.8e-08`PF00891.15^Methyltransf_2^O-methyltransferase^114-336^E:1.4e-57	0,262	0,282	0,109	-0,469	0,283	-0,606	0,043	-1,252	0	-1,039	7OMT_PAPSO	(R,S)-reticuline 7-O-methyltransferase (7OMT) (EC 2.1.1.291)	7OMT PSOMT1	Papaver somniferum (Opium poppy)		Alkaloid metabolism; Direct protein sequencing; Methyltransferase; S-adenosyl-L-methionine; Transferase
SRR342017_TR27836_c0_g4_i5_len=601	NA	NA	NA	NA	NA	.	0,016	-1,498	0,611	0,363	0,748	0,223	0,534	0,423	0	-6,708	.	.	.	.	.	.
SRR342017_TR29615_c1_g4_i1_len=1526	TBL44_ARATH	Q:203-913,H:15-262	54.44%ID	E:2e-90	Protein PMR5	PF14416.3^PMR5N^PMR5 N terminal Domain^56-108^E:1e-22`PF13839.3^PC-Esterase^GDSL/SGNH-like Acyl-Esterase family found in Pmr5 and Cas1p^109-224^E:1.6e-40	1	-0,213	0,381	-2,223	1	-0,185	1	-0,166	0	-1,635	TBL44_ARATH	Protein PMR5 (Powdery mildew resistance protein 5) (Protein trichome birefringence-like 44)	PMR5 TBL44 At5g58600 MZN1.6	Arabidopsis thaliana (Mouse-ear cress)		Alternative splicing; Complete proteome; Membrane; Plant defense; Reference proteome; Signal-anchor; Transmembrane; Transmembrane helix
SRR342017_TR33136_c0_g3_i4_len=3634	FTSHC_ARATH	Q:1107-3089,H:348-1008	86.84%ID	E:0	ATP-dependent zinc metalloprotease FTSH 12, chloroplastic	PF00004.26^AAA^ATPase family associated with various cellular activities (AAA)^140-276^E:6.2e-32`PF07724.11^AAA_2^AAA domain (Cdc48 subfamily)^140-255^E:0.00026`PF01434.15^Peptidase_M41^Peptidase family M41^365-570^E:2.4e-23	0,62	-0,4	0,352	-0,912	1	-0,59	1	-0,545	0	-3,428	FTSHC_ARATH	ATP-dependent zinc metalloprotease FTSH 12, chloroplastic (AtFTSH12) (EC 3.4.24.-)	FTSH12 At1g79560 T8K14.2	Arabidopsis thaliana (Mouse-ear cress)		ATP-binding; Chloroplast; Complete proteome; Hydrolase; Membrane; Metal-binding; Metalloprotease; Nucleotide-binding; Plastid; Protease; Reference proteome; Thylakoid; Transit peptide; Transmembrane; Transmembrane helix; Zinc
SRR342017_TR33224_c4_g1_i1_len=2813	NA	NA	NA	NA	NA	PF00575.20^S1^S1 RNA binding domain^551-621^E:3.7e-11	1	0,139	1	-0,339	1	-0,233	1	-0,28	0	-9,653	.	.	.	.	.	.
SRR342019_TR5634_c1_g2_i3_len=846	NA	NA	NA	NA	NA	.	1	1,773	1	1,738	0,502	3,881	0,502	3,755	0	-1,005	.	.	.	.	.	.
SRR342019_TR7361_c1_g1_i1_len=767	SP1L1_ARATH	Q:131-451,H:1-110	67.57%ID	E:1e-28	Protein SPIRAL1-like 1	.	0,159	-0,418	0,321	-0,335	0,433	-0,262	0,838	-0,074	0	-1,285	SP1L1_ARATH	Protein SPIRAL1-like 1	SP1L1 At1g26355 T1K7.26	Arabidopsis thaliana (Mouse-ear cress)		Complete proteome; Microtubule; Phosphoprotein; Reference proteome
SRR342019_TR12159_c0_g1_i3_len=1145	NOG2_MOUSE	Q:305-595,H:382-480	52.53%ID	E:2e-25	Nucleolar GTP-binding protein 2	.	0,692	1,102	0,038	-1,211	0,76	-0,582	0,299	-1,517	0	-2,03	NOG2_MOUSE	Nucleolar GTP-binding protein 2	Gnl2	Mus musculus (Mouse)		Acetylation; Complete proteome; GTP-binding; Nucleotide-binding; Nucleus; Phosphoprotein; Reference proteome; Ribosome biogenesis
SRR342019_TR12347_c1_g12_i3_len=2335	GLYM_SOLTU	Q:1978-317,H:1-518	84.86%ID	E:0	Serine hydroxymethyltransferase, mitochondrial	PF00464.16^SHMT^Serine hydroxymethyltransferase^58-437^E:8.3e-202	1	1,237	1	-0,713	1	-0,468	1	0,523	0	-8,068	GLYM_SOLTU	Serine hydroxymethyltransferase, mitochondrial (SHMT) (EC 2.1.2.1) (Glycine hydroxymethyltransferase) (Serine methylase)		Solanum tuberosum (Potato)	PATHWAY: One-carbon metabolism; tetrahydrofolate interconversion.	Complete proteome; Mitochondrion; One-carbon metabolism; Pyridoxal phosphate; Reference proteome; Transferase; Transit peptide
SRR342019_TR14783_c0_g1_i1_len=1550	RK18_ARATH	Q:135-587,H:11-170	73.75%ID	E:4e-73	50S ribosomal protein L18, chloroplastic	PF02900.15^LigB^Catalytic LigB subunit of aromatic ring-opening dioxygenase^38-143^E:6.4e-05`PF00861.19^Ribosomal_L18p^Ribosomal L18 of archaea, bacteria, mitoch. and chloroplast^51-170^E:3.1e-47	0,107	-0,795	0,068	-0,902	1	-0,135	0,848	0,236	0	-1,038	RK18_ARATH	50S ribosomal protein L18, chloroplastic (CL18)	RPL18 At1g48350 F11A17.10	Arabidopsis thaliana (Mouse-ear cress)		Chloroplast; Complete proteome; Plastid; RNA-binding; Reference proteome; Ribonucleoprotein; Ribosomal protein; Transit peptide; rRNA-binding
SRR342019_TR16331_c0_g1_i1_len=1840	NA	NA	NA	NA	NA	PF02620.14^DUF177^Uncharacterized ACR, COG1399^175-318^E:3.7e-27	0,201	-0,359	0,03	-0,726	0,367	0,325	0,367	0,327	0	-5,368	.	.	.	.	.	.
SRR342019_TR16600_c1_g2_i1_len=634	MLO1_ARATH	Q:620-378,H:393-473	65.43%ID	E:2e-27	MLO-like protein 1	PF03094.12^Mlo^Mlo family^11-101^E:6.9e-27	1	0	0,076	-2,775	1	0,446	1	-1,735	0	-7,14	MLO1_ARATH	MLO-like protein 1 (AtMlo1) (MLO protein homolog 1) (AtMlo-H1)	MLO1 MLO-H1 At4g02600 T10P11.12	Arabidopsis thaliana (Mouse-ear cress)		Calmodulin-binding; Complete proteome; Membrane; Pathogenesis-related protein; Plant defense; Reference proteome; Transmembrane; Transmembrane helix
SRR342019_TR21676_c0_g2_i2_len=1344	NA	NA	NA	NA	NA	PF04949.10^Transcrip_act^Transcriptional activator^23-149^E:7e-63`PF08641.9^Mis14^Kinetochore protein Mis14 like^23-137^E:9.2e-05	0,444	-0,275	0,01	-0,989	1	-0,021	0,748	-0,303	0	-4,988	.	.	.	.	.	.
SRR342019_TR25937_c0_g2_i1_len=867	DAPB2_ARATH	Q:165-539,H:225-349	84%ID	E:4e-72	4-hydroxy-tetrahydrodipicolinate reductase 2, chloroplastic	PF05173.11^DapB_C^Dihydrodipicolinate reductase, C-terminus^1-115^E:4.5e-16	0,44	0,684	0,235	-0,993	1	0,116	1	-0,479	0,001	-3,748	DAPB2_ARATH	4-hydroxy-tetrahydrodipicolinate reductase 2, chloroplastic (HTPA reductase 2) (EC 1.17.1.8)	DAPB2 At3g59890 F24G16.160	Arabidopsis thaliana (Mouse-ear cress)	PATHWAY: Amino-acid biosynthesis; L-lysine biosynthesis via DAP pathway; (S)-tetrahydrodipicolinate from L-aspartate: step 4/4.	Alternative splicing; Amino-acid biosynthesis; Chloroplast; Complete proteome; Diaminopimelate biosynthesis; Lysine biosynthesis; NAD; NADP; Oxidoreductase; Plastid; Reference proteome; Transit peptide
SRR342019_TR27898_c0_g2_i4_len=2658	GLIP5_ARATH	Q:227-1123,H:47-343	62.33%ID	E:2e-124	GDSL esterase/lipase 5	PF00657.19^Lipase_GDSL^GDSL-like Lipase/Acylhydrolase^78-320^E:1.7e-08	0,783	-0,223	0,34	-0,48	0,278	-0,816	0,278	-0,734	0	-6,024	GLIP5_ARATH	GDSL esterase/lipase 5 (EC 3.1.1.-) (Extracellular lipase 5)	GLIP5 At1g53920 T18A20.15	Arabidopsis thaliana (Mouse-ear cress)		Complete proteome; Glycoprotein; Hydrolase; Lipid degradation; Lipid metabolism; Reference proteome; Secreted; Signal
SRR342019_TR28654_c1_g3_i1_len=991	R10A1_ARATH	Q:841-257,H:22-216	93.33%ID	E:9e-115	60S ribosomal protein L10a-1	PF00687.18^Ribosomal_L1^Ribosomal protein L1p/L10e family^2-149^E:2.5e-34	0,012	-0,935	0,011	-0,79	0,74	0,166	0,91	0,076	0	-7,076	R10A1_ARATH	60S ribosomal protein L10a-1	RPL10AA At1g08360 T27G7.6	Arabidopsis thaliana (Mouse-ear cress)		Complete proteome; Reference proteome; Ribonucleoprotein; Ribosomal protein
SRR342019_TR30363_c2_g2_i1_len=2308	NA	NA	NA	NA	NA	.	0,562	-0,179	0,69	0,139	0,732	0,171	1	-0,022	0	-5,281	.	.	.	.	.	.
SRR342019_TR31099_c1_g2_i2_len=2133	JAR1_ARATH	Q:1966-146,H:13-575	47.45%ID	E:0	Jasmonic acid-amido synthetase JAR1	PF03321.10^GH3^GH3 auxin-responsive promoter^32-615^E:3.4e-180	0,868	0,074	0,055	-0,805	0	1,133	0	1,139	0,001	-1,197	JAR1_ARATH	Jasmonic acid-amido synthetase JAR1 (EC 6.3.2.-) (Jasmonate-amino acid synthetase JAR1) (Protein FAR-RED INSENSITIVE 219) (Protein JASMONATE RESISTANT 1)	JAR1 FIN219 At2g46370 F11C10.6	Arabidopsis thaliana (Mouse-ear cress)		3D-structure; Alternative splicing; Coiled coil; Complete proteome; Cytoplasm; Immunity; Innate immunity; Jasmonic acid signaling pathway; Ligase; Plant defense; Reference proteome
SRR342019_TR34407_c0_g1_i1_len=808	NA	NA	NA	NA	NA	.	1	0,062	0,025	-2,951	0,629	-1,346	0,629	-1,698	0	-5,66	.	.	.	.	.	.
SRR342019_TR36227_c0_g1_i1_len=768	VTI12_ARATH	Q:714-274,H:53-199	70.75%ID	E:2e-66	Vesicle transport v-SNARE 12	PF05008.12^V-SNARE^Vesicle transport v-SNARE protein N-terminus^19-57^E:6.3e-11`PF12352.5^V-SNARE_C^Snare region anchored in the vesicle membrane C-terminus^95-159^E:2.9e-24	0,001	6,637	0,783	0,201	0	6,948	1	0	0	-7,499	VTI12_ARATH	Vesicle transport v-SNARE 12 (AtVTI12) (Vesicle soluble NSF attachment protein receptor VTI1b) (AtVTI1b) (Vesicle transport v-SNARE protein VTI1b)	VTI12 VTI1B At1g26670 T24P13.5	Arabidopsis thaliana (Mouse-ear cress)		Acetylation; Coiled coil; Complete proteome; Golgi apparatus; Membrane; Protein transport; Reference proteome; Transmembrane; Transmembrane helix; Transport
SRR342022_TR6858_c0_g1_i1_len=684	PGKY_TOBAC	Q:2-364,H:281-401	90.91%ID	E:2e-68	Phosphoglycerate kinase, cytosolic	PF00162.16^PGK^Phosphoglycerate kinase^4-110^E:3.2e-45	0,299	-0,454	0,001	-1,06	0,006	1,034	0,614	0,238	0	-1,757	PGKY_TOBAC	Phosphoglycerate kinase, cytosolic (EC 2.7.2.3)		Nicotiana tabacum (Common tobacco)	PATHWAY: Carbohydrate degradation; glycolysis; pyruvate from D-glyceraldehyde 3-phosphate: step 2/5.	ATP-binding; Cytoplasm; Glycolysis; Kinase; Nucleotide-binding; Transferase
SRR342022_TR12890_c0_g1_i1_len=1677	KCS1_ARATH	Q:190-1461,H:103-526	48.71%ID	E:2e-148	3-ketoacyl-CoA synthase 1 {ECO:0000303|PubMed:10074711}	PF08392.9^FAE1_CUT1_RppA^FAE1/Type III polyketide synthase-like protein^27-310^E:4.3e-105`PF08541.7^ACP_syn_III_C^3-Oxoacyl-[acyl-carrier-protein (ACP)] synthase III C terminal^327-409^E:6.9e-12`PF02797.12^Chal_sti_synt_C^Chalcone and stilbene synthases, C-terminal domain^333-410^E:4.9e-07	0,059	-1,088	0,017	-5,826	1	0,674	1	-2,597	0,001	-2,108	KCS1_ARATH	3-ketoacyl-CoA synthase 1 (KCS-1) (EC 2.3.1.199) (Very long-chain fatty acid condensing enzyme 1) (VLCFA condensing enzyme 1)	KCS1 EL1 At1g01120 T25K16.11	Arabidopsis thaliana (Mouse-ear cress)	PATHWAY: Lipid metabolism; fatty acid biosynthesis.	Acyltransferase; Complete proteome; Endoplasmic reticulum; Membrane; NAD; NADP; Reference proteome; Transferase; Transmembrane; Transmembrane helix
SRR342022_TR15891_c0_g1_i3_len=890	NA	NA	NA	NA	NA	.	0,623	0,333	0,004	1,214	0,003	1,819	0,035	1,403	0	-7,029	.	.	.	.	.	.
SRR342022_TR24136_c2_g2_i8_len=1688	BT3L4_DANRE	Q:783-1112,H:13-123	59.46%ID	E:4e-36	Transcription factor BTF3 homolog 4	PF01849.15^NAC^NAC domain^16-71^E:3.8e-19	0,76	-0,775	1	-0,252	0,692	0,645	0,692	0,811	0,001	-1,786	BT3L4_DANRE	Transcription factor BTF3 homolog 4 (Basic transcription factor 3-like 4)	btf3l4	Danio rerio (Zebrafish) (Brachydanio rerio)		Complete proteome; Reference proteome
SRR342022_TR29290_c1_g3_i1_len=1346	NA	NA	NA	NA	NA	PF03763.10^Remorin_C^Remorin, C-terminal region^255-343^E:1.3e-22	0,502	2,518	1	1,032	1	0,101	1	-0,219	0,001	-6,614	.	.	.	.	.	.
SRR342022_TR30032_c0_g1_i11_len=1911	KRR1_SCHPO	Q:1398-436,H:3-322	61.49%ID	E:4e-105	KRR1 small subunit processome component homolog	.	0,641	0,226	0,163	0,742	0,18	0,742	0,623	0,354	0	-6,864	KRR1_SCHPO	KRR1 small subunit processome component homolog (KRR-R motif-containing protein 1) (Ribosomal RNA assembly protein mis3)	mis3 krr1 SPBC25B2.05	Schizosaccharomyces pombe (strain 972 / ATCC 24843) (Fission yeast)		Complete proteome; Nucleus; RNA-binding; Reference proteome; Ribonucleoprotein; Ribosome biogenesis; rRNA processing
SRR342022_TR33327_c0_g2_i1_len=1122	GTE8_ARATH	Q:317-517,H:629-695	73.13%ID	E:2e-21	Transcription factor GTE8	.	1	-0,21	0,231	-0,653	0,076	2,498	1	-0,224	0,001	-1,911	GTE8_ARATH	Transcription factor GTE8 (Bromodomain-containing protein GTE8) (Protein GLOBAL TRANSCRIPTION FACTOR GROUP E8)	GTE8 At3g27260 K17E12.8	Arabidopsis thaliana (Mouse-ear cress)		Alternative splicing; Bromodomain; Coiled coil; Complete proteome; Nucleus; Phosphoprotein; Reference proteome; Transcription; Transcription regulation
SRR342022_TR34978_c2_g2_i2_len=1234	RALF_TOBAC	Q:911-669,H:25-115	68.13%ID	E:9e-31	Rapid alkalinization factor	PF05498.8^RALF^Rapid ALkalinization Factor (RALF)^126-187^E:4.6e-30	0,868	0,069	0,608	-0,226	0,548	0,271	0,291	0,435	0,001	-1,254	RALF_TOBAC	Rapid alkalinization factor (NtRALF)	RALF	Nicotiana tabacum (Common tobacco)		Direct protein sequencing; Disulfide bond; Hormone; Secreted; Signal
SRR342023_TR5742_c0_g1_i4_len=1677	IMP1L_MOUSE	Q:975-1394,H:18-154	42.55%ID	E:1e-31	Mitochondrial inner membrane protease subunit 1	PF00717.20^Peptidase_S24^Peptidase S24-like^45-113^E:1.6e-08	0,798	-0,26	0,009	-4,637	1	0	1	0	0	-7,007	IMP1L_MOUSE	Mitochondrial inner membrane protease subunit 1 (EC 3.4.21.-) (IMP1-like protein)	Immp1l	Mus musculus (Mouse)		Complete proteome; Hydrolase; Membrane; Mitochondrion; Mitochondrion inner membrane; Protease; Reference proteome
SRR342023_TR9759_c0_g2_i4_len=1908	NA	NA	NA	NA	NA	PF00013.26^KH_1^KH domain^167-226^E:2.6e-10	0,906	-0,066	0,071	0,833	0,278	0,72	0,564	0,499	0	-5,571	.	.	.	.	.	.
SRR342023_TR15920_c0_g1_i1_len=818	IMP3_MOUSE	Q:85-621,H:2-181	57.78%ID	E:1e-72	U3 small nucleolar ribonucleoprotein protein IMP3	PF00163.16^Ribosomal_S4^Ribosomal protein S4/S9 N-terminal domain^3-62^E:3.1e-10`PF01479.22^S4^S4 domain^107-153^E:3e-10	0,226	2,335	0	6,978	0,066	3,352	0,129	3,079	0	-7,671	IMP3_MOUSE	U3 small nucleolar ribonucleoprotein protein IMP3 (U3 snoRNP protein IMP3)	Imp3	Mus musculus (Mouse)		Complete proteome; Nucleus; RNA-binding; Reference proteome; Ribonucleoprotein; Ribosome biogenesis; rRNA processing; rRNA-binding
SRR342023_TR18138_c2_g8_i1_len=734	DRP2A_ARATH	Q:51-332,H:790-882	76.84%ID	E:2e-24	Dynamin-2A	.	0,407	0,515	0,93	0,079	0,132	0,835	0,874	0,225	0	-7,408	DRP2A_ARATH	Dynamin-2A (EC 3.6.5.5) (Dynamin-like protein 6) (Dynamin-related protein 2A)	DRP2A ADL6 At1g10290 F14N23.17	Arabidopsis thaliana (Mouse-ear cress)		Acetylation; Coiled coil; Complete proteome; Cytoplasm; Cytoplasmic vesicle; Cytoskeleton; GTP-binding; Golgi apparatus; Hydrolase; Lipid-binding; Membrane; Microtubule; Motor protein; Nucleotide-binding; Reference proteome
SRR342023_TR19692_c2_g11_i1_len=533	METE_CATRO	Q:50-223,H:708-765	100%ID	E:6e-30	5-methyltetrahydropteroyltriglutamate--homocysteine methyltransferase	.	0,014	0,679	0,438	-0,176	0,284	0,323	0,701	-0,132	0	-0,895	METE_CATRO	5-methyltetrahydropteroyltriglutamate--homocysteine methyltransferase (EC 2.1.1.14) (Cobalamin-independent methionine synthase isozyme) (Vitamin-B12-independent methionine synthase isozyme)	METE	Catharanthus roseus (Madagascar periwinkle) (Vinca rosea)	PATHWAY: Amino-acid biosynthesis; L-methionine biosynthesis via de novo pathway; L-methionine from L-homocysteine (MetE route): step 1/1.	Amino-acid biosynthesis; Cytoplasm; Metal-binding; Methionine biosynthesis; Methyltransferase; Transferase; Zinc
SRR342023_TR20001_c7_g2_i1_len=671	NA	NA	NA	NA	NA	.	1	-0,105	0,073	-1,846	0,534	0,385	1	-0,056	0	-4,653	.	.	.	.	.	.
SRR342023_TR26079_c1_g2_i7_len=1243	CW15A_XENLA	Q:288-977,H:1-228	47.28%ID	E:2e-30	Protein CWC15 homolog A	PF04889.9^Cwf_Cwc_15^Cwf15/Cwc15 cell cycle control protein^1-230^E:2.8e-77	0,739	-0,268	0,756	0,251	0,094	1,27	0,133	1,25	0	-7,491	CW15A_XENLA	Protein CWC15 homolog A	cwc15-a	Xenopus laevis (African clawed frog)		Coiled coil; mRNA processing; mRNA splicing
SRR342023_TR26079_c1_g2_i9_len=1394	NA	NA	NA	NA	NA	.	1	-0,105	0,009	-1,425	0,629	1,097	1	0,716	0	-2,776	.	.	.	.	.	.
SRR342023_TR26242_c1_g6_i5_len=1802	HBPL1_ARATH	Q:648-1514,H:21-309	62.63%ID	E:9e-106	Heme-binding-like protein At3g10130, chloroplastic	PF04832.9^SOUL^SOUL heme-binding protein^107-176^E:2.3e-16`PF04832.9^SOUL^SOUL heme-binding protein^175-295^E:2e-32	0,129	3,012	1	0	1	0	1	0	0	-2,821	HBPL1_ARATH	Heme-binding-like protein At3g10130, chloroplastic	At3g10130 T22K18.4	Arabidopsis thaliana (Mouse-ear cress)		Chloroplast; Complete proteome; Plastid; Reference proteome; Transit peptide
SRR342023_TR28066_c5_g2_i10_len=512	NA	NA	NA	NA	NA	.	1	0	0,066	-0,984	1	0	0,017	5,753	0	-7,318	.	.	.	.	.	.
SRR342023_TR28517_c2_g4_i15_len=1730	NA	NA	NA	NA	NA	PF13417.3^GST_N_3^Glutathione S-transferase, N-terminal domain^141-216^E:2.1e-13`PF13417.3^GST_N_3^Glutathione S-transferase, N-terminal domain^263-332^E:8.3e-11	0,419	-0,422	0,111	0,63	0,031	-0,789	0,268	-0,408	0	-8,155	.	.	.	.	.	.
SRR342023_TR30201_c0_g1_i3_len=1271	NA	NA	NA	NA	NA	PF10674.6^Ycf54^Protein of unknown function (DUF2488)^154-243^E:3.6e-34	1	0,458	0,502	-2,523	1	0,75	1	1,593	0	-1,544	.	.	.	.	.	.
SRR342023_TR31470_c0_g1_i2_len=685	CCD8A_ORYSJ	Q:55-492,H:405-550	80.14%ID	E:8e-81	Carotenoid cleavage dioxygenase 8 homolog A, chloroplastic	PF03055.12^RPE65^Retinal pigment epithelial membrane protein^19-162^E:4e-33	1	0	1	0	1	0	1	0	0	-8,931	CCD8A_ORYSJ	Carotenoid cleavage dioxygenase 8 homolog A, chloroplastic (OsCCD8a) (EC 1.13.11.-) (Protein D10-like)	CCD8A D10L Os01g0566500 LOC_Os01g38580 OsJ_02260 P0002B05.5 P0697C12.32	Oryza sativa subsp. japonica (Rice)		Chloroplast; Complete proteome; Dioxygenase; Iron; Metal-binding; Oxidoreductase; Plastid; Reference proteome; Transit peptide
SRR646572_TR6712_c0_g2_i4_len=2132	NOP5B_ARATH	Q:1896-565,H:2-445	81.31%ID	E:0	Probable nucleolar protein 5-2	PF08156.10^NOP5NT^NOP5NT (NUC127) domain^2-65^E:8.8e-21`PF01798.15^Nop^snoRNA binding domain, fibrillarin^166-394^E:2.3e-85	0,001	-6,419	0,524	0,72	0,502	3,968	0,129	4,931	0,001	-6,599	NOP5B_ARATH	Probable nucleolar protein 5-2 (MAR-binding NOP56/58 homolog 2) (Nucleolar protein 58-2)	NOP5-2 NOP58-2 At3g05060 T12H1.2	Arabidopsis thaliana (Mouse-ear cress)		Complete proteome; Nucleus; Reference proteome; Ribosome biogenesis
SRR646572_TR15307_c0_g4_i1_len=832	NA	NA	NA	NA	NA	PF07172.8^GRP^Glycine rich protein family^1-100^E:4.6e-10	0	1,133	0,535	0,19	0	4,972	0	3,743	0	-1,645	.	.	.	.	.	.
SRR646572_TR18397_c0_g2_i1_len=519	ARF_MAIZE	Q:170-502,H:1-111	99.1%ID	E:4e-75	ADP-ribosylation factor	PF00025.18^Arf^ADP-ribosylation factor family^5-112^E:5.4e-52`PF09439.7^SRPRB^Signal recognition particle receptor beta subunit^16-106^E:9.6e-11`PF08477.10^Roc^Ras of Complex, Roc, domain of DAPkinase^19-100^E:2.2e-12`PF04670.9^Gtr1_RagA^Gtr1/RagA G protein conserved region^19-110^E:6.5e-09`PF01926.20^MMR_HSR1^50S ribosome-binding GTPase^19-106^E:0.00011`PF00071.19^Ras^Ras family^20-99^E:1.9e-09	0,927	0,027	0	1,21	0,806	-0,063	0,695	-0,094	0	-10,197	ARF_MAIZE	ADP-ribosylation factor	ARF1	Zea mays (Maize)		Complete proteome; ER-Golgi transport; GTP-binding; Golgi apparatus; Lipoprotein; Myristate; Nucleotide-binding; Protein transport; Reference proteome; Transport
SRR646572_TR26265_c0_g1_i1_len=884	RAA5B_ARATH	Q:882-385,H:52-217	73.49%ID	E:2e-84	Ras-related protein RABA5b	PF00071.19^Ras^Ras family^2-123^E:1e-42`PF00025.18^Arf^ADP-ribosylation factor family^4-120^E:1.7e-08`PF08477.10^Roc^Ras of Complex, Roc, domain of DAPkinase^6-78^E:3.2e-18	0,394	-0,474	0,049	-1,396	0,874	-0,235	0,505	-0,457	0	-2,768	RAA5B_ARATH	Ras-related protein RABA5b (AtRABA5b)	RABA5B At3g07410 F21O3.12	Arabidopsis thaliana (Mouse-ear cress)		Cell membrane; Complete proteome; GTP-binding; Lipoprotein; Membrane; Nucleotide-binding; Prenylation; Protein transport; Reference proteome; Transport
SRR646572_TR30153_c0_g1_i4_len=2448	NA	NA	NA	NA	NA	PF13417.3^GST_N_3^Glutathione S-transferase, N-terminal domain^141-192^E:2.2e-05	0,822	0,28	0,444	-0,444	0,021	-1,26	0,021	-1,312	0,001	-4,969	.	.	.	.	.	.
SRR646572_TR30153_c0_g1_i6_len=1656	NA	NA	NA	NA	NA	PF13417.3^GST_N_3^Glutathione S-transferase, N-terminal domain^141-216^E:2.1e-13`PF13417.3^GST_N_3^Glutathione S-transferase, N-terminal domain^263-332^E:8.3e-11	0,68	0,187	0,117	0,524	0,022	-0,718	0,354	-0,289	0	-1,945	.	.	.	.	.	.
SRR646572_TR31178_c1_g1_i2_len=729	RL30_LUPLU	Q:646-311,H:1-112	90.18%ID	E:6e-68	60S ribosomal protein L30	PF01248.23^Ribosomal_L7Ae^Ribosomal protein L7Ae/L30e/S12e/Gadd45 family^13-105^E:2.4e-24	0,298	-0,614	0,578	-0,221	0,211	0,626	0,364	0,508	0	-3,709	RL30_LUPLU	60S ribosomal protein L30	RPL30	Lupinus luteus (European yellow lupin)		Ribonucleoprotein; Ribosomal protein
SRR646572_TR31741_c0_g1_i1_len=3819	RU17_ARATH	Q:255-764,H:1-170	79.41%ID	E:3e-70	U1 small nuclear ribonucleoprotein 70 kDa	.	1	-0,221	1	0,004	1	0,691	1	1,137	0	-5,868	RU17_ARATH	U1 small nuclear ribonucleoprotein 70 kDa (U1 snRNP 70 kDa) (U1-70K) (snRNP70)	RNU1 At3g50670 T3A5.50	Arabidopsis thaliana (Mouse-ear cress)		Alternative splicing; Complete proteome; Nucleus; Phosphoprotein; RNA-binding; Reference proteome; Ribonucleoprotein; Spliceosome; mRNA processing; mRNA splicing
SRR646572_TR35106_c2_g1_i1_len=1990	PSBK_DAUCA	Q:174-356,H:1-61	95.08%ID	E:9e-29	Photosystem II reaction center protein K {ECO:0000255|HAMAP-Rule:MF_00441}	.	0,003	1,11	0,126	-0,496	0,206	0,481	0,159	0,53	0	-1,182	PSBK_DAUCA	Photosystem II reaction center protein K (PSII-K)	psbK	Daucus carota (Wild carrot)		Chloroplast; Membrane; Photosynthesis; Photosystem II; Plastid; Reaction center; Thylakoid; Transmembrane; Transmembrane helix
SRR646572_TR36861_c0_g1_i4_len=1213	NUCL2_ORYSJ	Q:224-592,H:507-630	51.2%ID	E:6e-31	Nucleolin 2	.	0,545	-0,527	0,009	-0,875	1	1,197	1	0,258	0	-1,661	NUCL2_ORYSJ	Nucleolin 2 (Protein NUCLEOLIN LIKE 2)	Os04g0620700 LOC_Os04g52960 OSJNBa0058K23.21	Oryza sativa subsp. japonica (Rice)		Complete proteome; Nucleus; RNA-binding; Reference proteome; Repeat; rRNA processing
SRR646572_TR38538_c1_g1_i15_len=1279	NA	NA	NA	NA	NA	.	1	0	1	0	1	0	1	0,397	0	-6,979	.	.	.	.	.	.
SRR646596_TR9280_c1_g1_i2_len=521	NA	NA	NA	NA	NA	.	0	1,171	0	7,188	0	3,838	1	0,605	0	-1,602	.	.	.	.	.	.
SRR646596_TR14411_c0_g1_i1_len=1704	ANM11_ARATH	Q:213-1343,H:18-390	74.34%ID	E:0	Protein arginine N-methyltransferase 1.1	PF05185.13^PRMT5^PRMT5 arginine-N-methyltransferase^84-355^E:4.3e-07`PF06325.10^PrmA^Ribosomal protein L11 methyltransferase (PrmA)^107-181^E:1.6e-09`PF05175.11^MTS^Methyltransferase small domain^108-180^E:9.2e-05`PF13649.3^Methyltransf_25^Methyltransferase domain^111-185^E:1.5e-08`PF08241.9^Methyltransf_11^Methyltransferase domain^112-197^E:2.8e-07	0,025	-0,868	0,133	0,632	0,212	1,025	0,381	0,805	0	-7,486	ANM11_ARATH	Protein arginine N-methyltransferase 1.1 (AtPRMT11) (EC 2.1.1.-) (EC 2.1.1.125) (Arginine methyltransferase pam1) (Histone-arginine N-methyltransferase PRMT11)	PRMT11 PAM1 PRMT1.1 PRMT1B At4g29510 T16L4.20	Arabidopsis thaliana (Mouse-ear cress)		Chromatin regulator; Complete proteome; Cytoplasm; Methyltransferase; Nucleus; Reference proteome; S-adenosyl-L-methionine; Transcription; Transcription regulation; Transferase
SRR646604_TR506_c1_g3_i4_len=916	HMGL_IPONI	Q:73-465,H:1-126	71.76%ID	E:4e-45	HMG1/2-like protein	PF09011.7^HMG_box_2^HMG-box domain^61-133^E:3e-15`PF00505.16^HMG_box^HMG (high mobility group) box^64-133^E:1.7e-22	0,663	0,513	0,729	-0,304	1	-0,498	1	-0,506	0	-1,285	HMGL_IPONI	HMG1/2-like protein		Ipomoea nil (Japanese morning glory) (Pharbitis nil)		DNA-binding; Nucleus
SRR646604_TR2289_c0_g1_i1_len=1449	NA	NA	NA	NA	NA	PF02536.11^mTERF^mTERF^14-121^E:6.5e-15`PF02536.11^mTERF^mTERF^110-322^E:1.7e-46	0,118	1,971	0,034	5,68	0,254	4,648	1	0	0	-7,75	.	.	.	.	.	.
SRR646604_TR5547_c0_g2_i8_len=1612	S35F2_HUMAN	Q:427-1233,H:69-345	35.38%ID	E:2e-42	Solute carrier family 35 member F2	PF06027.9^SLC35F^Solute carrier family 35^1-123^E:3.4e-43`PF00892.17^EamA^EamA-like transporter family^21-122^E:9e-07	0,545	0,432	0,719	-0,338	0,732	-0,707	0,461	-1,344	0	-2,412	S35F2_HUMAN	Solute carrier family 35 member F2	SLC35F2	Homo sapiens (Human)		Acetylation; Alternative splicing; Complete proteome; Membrane; Phosphoprotein; Reference proteome; Transmembrane; Transmembrane helix; Transport
SRR646604_TR7485_c0_g1_i1_len=3973	PS12A_ARATH	Q:2066-3370,H:4-438	80.46%ID	E:0	26S proteasome non-ATPase regulatory subunit 12 homolog A	PF14580.3^LRR_9^Leucine-rich repeat^58-165^E:3.8e-14`PF12799.4^LRR_4^Leucine Rich repeats (2 copies)^70-114^E:9.8e-08`PF13516.3^LRR_6^Leucine Rich repeat^95-109^E:0.79	0,689	-0,223	0,038	1,154	0,272	0,492	0,079	0,744	0	-3,228	PS12A_ARATH	26S proteasome non-ATPase regulatory subunit 12 homolog A (26S proteasome regulatory subunit RPN5a) (AtRPN5a) (26S proteasome regulatory subunit p55 homolog A) (Protein EMBRYO DEFECTIVE 2107) (Protein MARIPOSA)	RPN5A EMB2107 MSA At5g09900 MYH9.11	Arabidopsis thaliana (Mouse-ear cress)		Alternative splicing; Coiled coil; Complete proteome; Cytoplasm; Nucleus; Proteasome; Reference proteome

SRR646604_TR8067_c0_g2_i1_len=1041	PER12_ARATH	Q:170-730,H:153-339	66.84%ID	E:8e-86	Peroxidase 12	PF00141.20^peroxidase^Peroxidase^5-151^E:4.5e-38	1	0	1	3,225	1	0	1	0	0,001	-3,191	PER12_ARATH	Peroxidase 12 (Atperox P12) (EC 1.11.1.7) (ATP4a) (PRXR6)	PER12 P12 At1g71695 F14O23.6 F26A9.5	Arabidopsis thaliana (Mouse-ear cress)		Calcium; Complete proteome; Disulfide bond; Glycoprotein; Heme; Hydrogen peroxide; Iron; Metal-binding; Oxidoreductase; Peroxidase; Reference proteome; Secreted; Signal; Vacuole
SRR646604_TR13798_c0_g2_i1_len=811	SUI1_ORYSJ	Q:560-222,H:1-115	92.17%ID	E:4e-70	Protein translation factor SUI1 homolog	PF01253.19^SUI1^Translation initiation factor SUI1^28-102^E:5.4e-26	0,04	0,467	0,02	0,537	0	1,442	0	1,329	0	-1,392	SUI1_ORYSJ	Protein translation factor SUI1 homolog (Protein GOS2) (Protein eIF1) (Translation initiation factor 1)	GOS2 Os07g0529800 LOC_Os07g34589	Oryza sativa subsp. japonica (Rice)		Complete proteome; Initiation factor; Protein biosynthesis; Reference proteome; Translation regulation
SRR646604_TR16508_c0_g1_i1_len=1761	AVP_VIGRR	Q:245-1336,H:403-766	98.08%ID	E:0	Pyrophosphate-energized vacuolar membrane proton pump	PF03030.13^H_PPase^Inorganic H+ pyrophosphatase^1-273^E:5e-130	0,578	-0,166	0,203	-0,322	0	2,119	0,376	0,421	0	-1,701	AVP_VIGRR	Pyrophosphate-energized vacuolar membrane proton pump (EC 3.6.1.1) (Pyrophosphate-energized inorganic pyrophosphatase) (H(+)-PPase) (Vacuolar H(+)-pyrophosphatase)		Vigna radiata var. radiata (Mung bean) (Phaseolus aureus)		3D-structure; Direct protein sequencing; Disulfide bond; Hydrogen ion transport; Hydrolase; Ion transport; Magnesium; Membrane; Metal-binding; Transmembrane; Transmembrane helix; Transport; Vacuole
SRR646604_TR22799_c0_g1_i2_len=1024	SOC1_ARATH	Q:819-304,H:1-172	76.16%ID	E:1e-68	MADS-box protein SOC1	PF00319.15^SRF-TF^SRF-type transcription factor (DNA-binding and dimerisation domain)^10-57^E:5.1e-27`PF01486.14^K-box^K-box region^84-171^E:3.4e-28	0,319	-0,994	0,258	0,863	0,87	-0,22	0,559	0,309	0	-5,902	SOC1_ARATH	MADS-box protein SOC1 (Agamous-like MADS-box protein AGL20) (Protein SUPPRESSOR OF CONSTANS OVEREXPRESSION 1)	SOC1 AGL20 At2g45660 F17K2.19	Arabidopsis thaliana (Mouse-ear cress)		Activator; Complete proteome; Cytoplasm; DNA-binding; Developmental protein; Differentiation; Flowering; Nucleus; Reference proteome; Transcription; Transcription regulation
SRR646604_TR24194_c0_g1_i1_len=1753	FBK9_ARATH	Q:1434-388,H:15-378	40.55%ID	E:6e-65	F-box/kelch-repeat protein At1g23390	PF13964.3^Kelch_6^Kelch motif^138-182^E:2.9e-06	1	0	1	0	1	0	1	0	0	-3,881	FBK9_ARATH	F-box/kelch-repeat protein At1g23390	At1g23390 F26F24.26 F28C11.3	Arabidopsis thaliana (Mouse-ear cress)		Complete proteome; Kelch repeat; Reference proteome; Repeat
SRR646604_TR28211_c0_g1_i1_len=800	WAT1_ARATH	Q:50-391,H:273-389	77.12%ID	E:8e-48	Protein WALLS ARE THIN 1	.	0,461	0,298	0,001	-1,542	0,02	1,451	1	-0,04	0	-4,104	WAT1_ARATH	Protein WALLS ARE THIN 1	WAT1 At1g75500 F10A5.28 F1B16.19	Arabidopsis thaliana (Mouse-ear cress)		Amino-acid biosynthesis; Aromatic amino acid biosynthesis; Auxin biosynthesis; Auxin signaling pathway; Cell wall biogenesis/degradation; Complete proteome; Membrane; Phosphoprotein; Plant defense; Reference proteome; Repeat; Transmembrane; Transmembrane helix; Tryptophan biosynthesis; Vacuole
SRR646604_TR29208_c0_g1_i2_len=1307	XTH2_SOYBN	Q:759-1307,H:97-282	79.03%ID	E:7e-105	Xyloglucan endotransglucosylase/hydrolase 2 {ECO:0000250|UniProtKB:Q38857}	PF03171.17^2OG-FeII_Oxy^2OG-Fe(II) oxygenase superfamily^40-135^E:8.7e-28`PF00722.18^Glyco_hydro_16^Glycosyl hydrolases family 16^212-332^E:4.3e-44`PF06955.9^XET_C^Xyloglucan endo-transglycosylase (XET) C-terminus^354-397^E:1.8e-19	1	0,855	1	1,684	1	0,238	1	-0,383	0	-2,602	XTH2_SOYBN	Xyloglucan endotransglucosylase/hydrolase 2 (EC 2.4.1.207) (Brassinosteroid-regulated protein BRU1)		Glycine max (Soybean) (Glycine hispida)		Apoplast; Cell wall; Complete proteome; Disulfide bond; Glycoprotein; Glycosidase; Hydrolase; Reference proteome; Secreted; Signal; Transferase
SRR646604_TR36156_c2_g1_i1_len=689	NA	NA	NA	NA	NA	.	1	-0,039	0,395	0,521	0,086	0,946	0,007	1,337	0	-7,378	.	.	.	.	.	.
SRR646604_TR38081_c0_g3_i4_len=1533	ALFC2_ARATH	Q:141-1295,H:12-397	86.27%ID	E:0	Probable fructose-bisphosphate aldolase 2, chloroplastic	PF00274.16^Glycolytic^Fructose-bisphosphate aldolase class-I^79-423^E:6.2e-157	0,043	1,33	0,107	0,81	0,127	-0,327	0,049	-0,421	0	-1,303	ALFC2_ARATH	Probable fructose-bisphosphate aldolase 2, chloroplastic (EC 4.1.2.13)	FBA2 At4g38970 F19H22.70	Arabidopsis thaliana (Mouse-ear cress)	PATHWAY: Carbohydrate degradation; glycolysis; D-glyceraldehyde 3-phosphate and glycerone phosphate from D-glucose: step 4/4.	Alternative splicing; Chloroplast; Complete proteome; Glycolysis; Lyase; Methylation; Phosphoprotein; Plastid; Reference proteome; Schiff base; Transit peptide
SRR648705_TR683_c0_g8_i2_len=1382	PLAS_SOLLC	Q:724-248,H:11-170	77.16%ID	E:4e-67	Plastocyanin, chloroplastic	PF00127.17^Copper-bind^Copper binding proteins, plastocyanin/azurin family^108-205^E:3.4e-36	1	0,136	0,692	0,921	0,189	-1,501	0,356	-1,282	0	-1,189	PLAS_SOLLC	Plastocyanin, chloroplastic	PETE	Solanum lycopersicum (Tomato) (Lycopersicon esculentum)		Chloroplast; Complete proteome; Copper; Electron transport; Membrane; Metal-binding; Plastid; Reference proteome; Thylakoid; Transit peptide; Transport
SRR648705_TR980_c1_g1_i5_len=1345	VATL_VIGRR	Q:1097-606,H:1-164	97.56%ID	E:3e-67	V-type proton ATPase 16 kDa proteolipid subunit	PF00137.18^ATP-synt_C^ATP synthase subunit C^16-75^E:6.2e-14`PF00137.18^ATP-synt_C^ATP synthase subunit C^96-154^E:6.6e-21	1	0,009	0,481	0,246	0,033	0,707	0,113	0,547	0	-7,136	VATL_VIGRR	V-type proton ATPase 16 kDa proteolipid subunit (V-ATPase 16 kDa proteolipid subunit) (Vacuolar proton pump 16 kDa proteolipid subunit)		Vigna radiata var. radiata (Mung bean) (Phaseolus aureus)		Hydrogen ion transport; Ion transport; Membrane; Transmembrane; Transmembrane helix; Transport; Vacuole
SRR648705_TR6991_c0_g2_i1_len=1391	RRP3_SPIOL	Q:338-652,H:77-178	80.95%ID	E:3e-53	30S ribosomal protein 3, chloroplastic	PF04839.10^PSRP-3_Ycf65^Plastid and cyanobacterial ribosomal protein (PSRP-3 / Ycf65)^165-211^E:9e-26	0,288	-0,544	1	-0,081	0,86	-0,15	1	-0,002	0	-1,431	RRP3_SPIOL	30S ribosomal protein 3, chloroplastic (Plastid-specific 30S ribosomal protein 3) (PSRP-3)	PSRP3	Spinacia oleracea (Spinach)		Chloroplast; Direct protein sequencing; Plastid; RNA-binding; Ribonucleoprotein; Ribosomal protein; Transit peptide; rRNA-binding
SRR648705_TR8497_c0_g2_i2_len=1839	FDFT_NICBE	Q:325-1554,H:1-410	81.71%ID	E:0	Squalene synthase	PF00494.16^SQS_PSY^Squalene/phytoene synthase^45-315^E:4.1e-45	1	-0,173	1	-0,337	1	0,045	0,86	-0,172	0,001	-1,003	FDFT_NICBE	Squalene synthase (SQS) (SS) (EC 2.5.1.21) (FPP:FPP farnesyltransferase) (Farnesyl-diphosphate farnesyltransferase)		Nicotiana benthamiana	PATHWAY: Terpene metabolism; lanosterol biosynthesis; lanosterol from farnesyl diphosphate: step 1/3.	Endoplasmic reticulum; Isoprene biosynthesis; Magnesium; Membrane; Multifunctional enzyme; NADP; Oxidoreductase; Transferase; Transmembrane; Transmembrane helix
SRR648705_TR9782_c0_g2_i1_len=772	RPC2_HUMAN	Q:160-435,H:1044-1129	67.39%ID	E:4e-37	DNA-directed RNA polymerase III subunit RPC2	.	1	0,419	0,316	-0,711	1	-3,473	0,629	1,278	0	-7,417	RPC2_HUMAN	DNA-directed RNA polymerase III subunit RPC2 (RNA polymerase III subunit C2) (EC 2.7.7.6) (C128) (DNA-directed RNA polymerase III 127.6 kDa polypeptide) (DNA-directed RNA polymerase III subunit B)	POLR3B	Homo sapiens (Human)		Alternative splicing; Antiviral defense; Complete proteome; DNA-directed RNA polymerase; Disease mutation; Immunity; Innate immunity; Leukodystrophy; Metal-binding; Nucleotidyltransferase; Nucleus; Polymorphism; Reference proteome; Transcription; Transferase; Zinc; Zinc-finger
SRR648705_TR12620_c0_g5_i2_len=1011	NA	NA	NA	NA	NA	.	1	0,006	1	0,331	1	1,48	1	0,077	0	-7,875	.	.	.	.	.	.
SRR648705_TR12919_c1_g3_i4_len=1204	PLP3A_ARATH	Q:192-734,H:1-181	79.56%ID	E:7e-90	Thioredoxin domain-containing protein PLP3A	PF00085.17^Thioredoxin^Thioredoxin^89-173^E:8.7e-06	0,912	-0,051	1	-0,018	0,581	0,349	0,886	0,16	0	-3,778	PLP3A_ARATH	Thioredoxin domain-containing protein PLP3A (Phosducin-like protein 3A)	PLP3A At3g50960 F18B3.240	Arabidopsis thaliana (Mouse-ear cress)		Complete proteome; Cytoplasm; Microtubule; Nucleus; Reference proteome
SRR648705_TR13159_c0_g4_i7_len=1585	UBC2_WHEAT	Q:894-1202,H:50-152	95.15%ID	E:5e-64	Ubiquitin-conjugating enzyme E2 2	PF00179.23^UQ_con^Ubiquitin-conjugating enzyme^25-119^E:8.2e-39	0,763	0,213	0,34	-0,509	0,748	-0,266	0,748	-0,229	0,001	-2,307	UBC2_WHEAT	Ubiquitin-conjugating enzyme E2 2 (EC 2.3.2.23) (E2 ubiquitin-conjugating enzyme 2) (Ubiquitin carrier protein 2) (Ubiquitin-conjugating enzyme E2-16 kDa) (Ubiquitin-protein ligase 2)	UBC2 UBC1	Triticum aestivum (Wheat)	PATHWAY: Protein modification; protein ubiquitination. {ECO:0000255|PROSITE-ProRule:PRU00388}.	ATP-binding; Complete proteome; Nucleotide-binding; Reference proteome; Transferase; Ubl conjugation pathway
SRR648705_TR14608_c0_g2_i3_len=2007	NA	NA	NA	NA	NA	PF02151.16^UVR^UvrB/uvrC motif^157-188^E:5.7e-08`PF08755.8^YccV-like^Hemimethylated DNA-binding protein YccV like^206-302^E:6.2e-26	1	-0,165	1	-1,067	1	-0,048	1	0,107	0,001	-2,716	.	.	.	.	.	.
SRR648705_TR14618_c0_g1_i1_len=3012	4CLL7_ARATH	Q:360-2000,H:1-544	68.74%ID	E:0	4-coumarate–CoA ligase-like 7	PF00501.25^AMP-binding^AMP-binding enzyme^36-449^E:1.1e-101`PF13193.3^AMP-binding_C^AMP-binding enzyme C-terminal domain^458-533^E:1.2e-14	0,402	0,865	0,663	-0,365	0,524	0,639	0,312	0,835	0	-1,638	4CLL7_ARATH	4-coumarate–CoA ligase-like 7 (EC 6.2.1.-) (4-coumarate–CoA ligase isoform 6) (At4CL6)	4CLL7 At4g05160 C17L7.80	Arabidopsis thaliana (Mouse-ear cress)		ATP-binding; Complete proteome; Fatty acid biosynthesis; Fatty acid metabolism; Ligase; Lipid biosynthesis; Lipid metabolism; Nucleotide-binding; Oxylipin biosynthesis; Peroxisome; Reference proteome
SRR648705_TR14680_c0_g3_i1_len=3141	RPM1_ARATH	Q:182-2857,H:1-889	33.04%ID	E:1e-119	Disease resistance protein RPM1	PF00931.19^NB-ARC^NB-ARC domain^177-466^E:1.1e-70	1	0,123	1	-1,257	1	-0,85	1	-1,832	0	-2,773	RPM1_ARATH	Disease resistance protein RPM1 (Resistance to Pseudomonas syringae protein 3)	RPM1 RPS3 At3g07040 F17A9.20	Arabidopsis thaliana (Mouse-ear cress)		ATP-binding; Complete proteome; Hypersensitive response; Leucine-rich repeat; Membrane; Nucleotide-binding; Plant defense; Reference proteome; Repeat
SRR648705_TR15869_c1_g6_i1_len=10680	TI214_COFAR	Q:4729-7305,H:995-1873	71.28%ID	E:0	Protein TIC 214 {ECO:0000250|UniProtKB:P56785}	PF05758.9^Ycf1^Ycf1^21-255^E:3.9e-160`PF05758.9^Ycf1^Ycf1^252-509^E:7.7e-82`PF05758.9^Ycf1^Ycf1^508-857^E:6.9e-173	0,34	-0,572	0,142	-0,858	0,874	-0,187	1	0,025	0	-1,011	TI214_COFAR	Protein TIC 214 (Translocon at the inner envelope membrane of chloroplasts 214) (AtTIC214)	TIC214 ycf1	Coffea arabica (Arabian coffee)		Chloroplast; Membrane; Plastid; Plastid inner membrane; Protein transport; Transmembrane; Transmembrane helix; Transport
SRR648705_TR16262_c1_g1_i1_len=2022	NA	NA	NA	NA	NA	.	0,77	-0,168	1	-0,061	1	0,1	1	-0,034	0	-5,386	.	.	.	.	.	.
SRR648705_TR16824_c0_g1_i6_len=8036	PSAA_COFAR	Q:3926-6175,H:1-750	99.47%ID	E:0	Photosystem I P700 chlorophyll a apoprotein A1 {ECO:0000255|HAMAP-Rule:MF_00458}	PF00223.16^PsaA_PsaB^Photosystem I psaA/psaB protein^32-744^E:0	0,858	-0,104	0,655	-0,223	0,77	-0,211	1	0,002	0	-0,997	PSAA_COFAR	Photosystem I P700 chlorophyll a apoprotein A1 (EC 1.97.1.12) (PSI-A) (PsaA)	psaA	Coffea arabica (Arabian coffee)		4Fe-4S; Chlorophyll; Chloroplast; Chromophore; Electron transport; Iron; Iron-sulfur; Magnesium; Membrane; Metal-binding; Oxidoreductase; Photosynthesis; Photosystem I; Plastid; Thylakoid; Transmembrane; Transmembrane helix; Transport
SRR648705_TR17389_c0_g1_i3_len=1276	XTH2_SOYBN	Q:716-165,H:97-283	78.61%ID	E:4e-104	Xyloglucan endotransglucosylase/hydrolase 2 {ECO:0000250|UniProtKB:Q38857}	.	1	-0,265	0,129	-5,205	0,009	-5,306	0,118	-1,97	0	-3,137	XTH2_SOYBN	Xyloglucan endotransglucosylase/hydrolase 2 (EC 2.4.1.207) (Brassinosteroid-regulated protein BRU1)		Glycine max (Soybean) (Glycine hispida)		Apoplast; Cell wall; Complete proteome; Disulfide bond; Glycoprotein; Glycosidase; Hydrolase; Reference proteome; Secreted; Signal; Transferase
SRR648705_TR18952_c0_g1_i1_len=1186	NA	NA	NA	NA	NA	.	0,461	0,891	1	0,297	1	0,071	0,732	-0,773	0	-2,456	.	.	.	.	.	.
SRR648705_TR21549_c0_g1_i5_len=975	SMD3_XENLA	Q:754-473,H:2-95	60.64%ID	E:2e-38	Small nuclear ribonucleoprotein Sm D3	PF01423.19^LSM^LSM domain^11-74^E:1.1e-16	0,822	-0,201	0,258	-0,884	0,692	1,044	0,692	0,896	0	-2,695	SMD3_XENLA	Small nuclear ribonucleoprotein Sm D3 (Sm-D3) (snRNP core protein D3)	snrpd3	Xenopus laevis (African clawed frog)		Cytoplasm; Methylation; Nucleus; RNA-binding; Repeat; Ribonucleoprotein; mRNA processing; mRNA splicing
SRR648705_TR21942_c2_g4_i3_len=582	NA	NA	NA	NA	NA	.	1	-0,316	1	0,455	1	-0,386	0,629	-0,914	0	-2,839	.	.	.	.	.	.
SRR648705_TR23032_c0_g1_i4_len=2269	RH56_ARATH	Q:347-1513,H:39-427	95.63%ID	E:0	DEAD-box ATP-dependent RNA helicase 56	PF00270.26^DEAD^DEAD/DEAH box helicase^65-230^E:9.2e-38`PF04851.12^ResIII^Type III restriction enzyme, res subunit^81-227^E:2.1e-06`PF00271.28^Helicase_C^Helicase conserved C-terminal domain^271-377^E:2.2e-27	0,06	-0,765	0,915	0,08	0,395	0,649	0,395	0,597	0	-7,063	RH56_ARATH	DEAD-box ATP-dependent RNA helicase 56 (EC 3.6.4.13) (UAP56 homolog B)	RH56 UAP56B At5g11200 F2I11.90	Arabidopsis thaliana (Mouse-ear cress)		ATP-binding; Alternative splicing; Complete proteome; Helicase; Hydrolase; Nucleotide-binding; Nucleus; RNA-binding; Reference proteome; Transport; mRNA processing; mRNA splicing; mRNA transport
SRR648705_TR23308_c0_g3_i7_len=1826	SPDE_COFAR	Q:359-1411,H:1-316	79.89%ID	E:0	Spermidine synthase	PF01564.14^Spermine_synth^Spermine/spermidine synthase^60-305^E:5.7e-98	0,707	-0,39	0,381	0,744	0,059	-1,057	0,002	-1,985	0	-1,968	SPDE_COFAR	Spermidine synthase (SPDSY) (EC 2.5.1.16) (Putrescine aminopropyltransferase)		Coffea arabica (Arabian coffee)	PATHWAY: Amine and polyamine biosynthesis; spermidine biosynthesis; spermidine from putrescine: step 1/1.	Polyamine biosynthesis; Spermidine biosynthesis; Transferase
SRR648705_TR23318_c0_g1_i4_len=1302	PSB3A_ARATH	Q:1027-416,H:1-204	92.16%ID	E:2e-137	Proteasome subunit beta type-3-A	PF00227.23^Proteasome^Proteasome subunit^7-189^E:1.2e-42	0,798	0,474	1	-0,064	0,076	2,087	0,132	1,968	0	-4,732	PSB3A_ARATH	Proteasome subunit beta type-3-A (EC 3.4.25.1) (20S proteasome beta subunit C-1) (Proteasome component T)	PBC1 PRCT At1g21720 F8K7.15	Arabidopsis thaliana (Mouse-ear cress)		Alternative splicing; Complete proteome; Cytoplasm; Hydrolase; Nucleus; Protease; Proteasome; Reference proteome; Threonine protease
SRR648705_TR23795_c0_g1_i3_len=888	YTHD1_MOUSE	Q:66-386,H:437-543	55.14%ID	E:1e-31	YTH domain-containing family protein 1 {ECO:0000305}	PF04146.12^YTH^YT521-B-like domain^17-73^E:2e-12	0,412	0,599	0,733	0,121	0,002	1,842	0,195	0,926	0	-1,578	YTHD1_MOUSE	YTH domain-containing family protein 1 (Dermatomyositis associated with cancer putative autoantigen 1 homolog) (DACA-1 homolog)	Ythdf1	Mus musculus (Mouse)		Acetylation; Complete proteome; Cytoplasm; Phosphoprotein; RNA-binding; Reference proteome
SRR648705_TR24010_c1_g1_i1_len=2247	Y1154_ARATH	Q:310-1782,H:7-464	64.71%ID	E:0	Probable serine/threonine-protein kinase At1g01540	PF00069.22^Pkinase^Protein kinase domain^186-453^E:7.5e-48`PF07714.14^Pkinase_Tyr^Protein tyrosine kinase^188-455^E:1.1e-45	1	0,029	1	0,058	1	1,004	1	0	0	-2,209	Y1154_ARATH	Probable serine/threonine-protein kinase At1g01540 (EC 2.7.11.1)	At1g01540 F22L4.8	Arabidopsis thaliana (Mouse-ear cress)		ATP-binding; Alternative splicing; Complete proteome; Kinase; Membrane; Nucleotide-binding; Phosphoprotein; Reference proteome; Serine/threonine-protein kinase; Transferase; Transmembrane; Transmembrane helix
SRR648705_TR24275_c0_g1_i1_len=682	SNAK2_SOLTU	Q:343-155,H:42-104	71.43%ID	E:6e-23	Snakin-2	PF02704.11^GASA^Gibberellin regulated protein^45-104^E:3.1e-19	0,502	3,842	1	2,865	0,254	4,592	0,502	3,927	0	-4,036	SNAK2_SOLTU	Snakin-2	SN2	Solanum tuberosum (Potato)		Antimicrobial; Cell wall; Complete proteome; Direct protein sequencing; Disulfide bond; Plant defense; Reference proteome; Secreted; Signal
SRR648705_TR26775_c0_g1_i1_len=2734	PAL1_TOBAC	Q:2460-337,H:4-715	88.48%ID	E:0	Phenylalanine ammonia-lyase	PF00221.16^Lyase_aromatic^Aromatic amino acid lyase^62-540^E:3.3e-154	0,25	0,42	0	2,554	0,026	0,491	0,003	0,644	0	-1,871	PAL1_TOBAC	Phenylalanine ammonia-lyase (EC 4.3.1.24)	TPA1 PALB	Nicotiana tabacum (Common tobacco)	PATHWAY: Phenylpropanoid metabolism; trans-cinnamate biosynthesis; trans-cinnamate from L-phenylalanine: step 1/1.	Cytoplasm; Lyase; Phenylpropanoid metabolism
SRR648705_TR28375_c0_g1_i2_len=778	ARR9_ARATH	Q:564-109,H:1-158	76.1%ID	E:1e-79	Two-component response regulator ARR9	PF00072.21^Response_reg^Response regulator receiver domain^12-133^E:3.6e-21	1	0	1	0	1	0	1	0	0	-7,792	ARR9_ARATH	Two-component response regulator ARR9 (Response reactor 4)	ARR9 ATRR4 At3g57040 F24I3.120	Arabidopsis thaliana (Mouse-ear cress)		Complete proteome; Cytokinin signaling pathway; Nucleus; Phosphoprotein; Reference proteome; Transcription; Transcription regulation; Two-component regulatory system
SRR648705_TR29523_c0_g2_i1_len=1570	GOLS1_AJURE	Q:200-1216,H:1-333	83.78%ID	E:0	Galactinol synthase 1	PF01501.17^Glyco_transf_8^Glycosyl transferase family 8^32-274^E:3.6e-43	0,002	1,226	0	1,54	0,061	0,467	0,078	0,44	0,001	-0,763	GOLS1_AJURE	Galactinol synthase 1 (ArGolS1) (GolS-1) (EC 2.4.1.123)	GOLS1	Ajuga reptans (Bugle)		Carbohydrate metabolism; Cytoplasm; Galactose metabolism; Glycosyltransferase; Manganese; Metal-binding; Transferase
SRR648705_TR30731_c0_g3_i1_len=669	NA	NA	NA	NA	NA	.	0,129	-4,882	0,005	-4,728	1	2,644	1	1,78	0	-6,935	.	.	.	.	.	.
SRR648705_TR32123_c0_g2_i1_len=2142	AROG_SOLTU	Q:304-1782,H:30-510	84.79%ID	E:0	Phospho-2-dehydro-3-deoxyheptonate aldolase 2, chloroplastic	PF01474.13^DAHP_synth_2^Class-II DAHP synthetase family^2-384^E:1.3e-180	0,356	1,004	0,001	3,368	1	0,454	0,692	0,824	0	-1,824	AROG_SOLTU	Phospho-2-dehydro-3-deoxyheptonate aldolase 2, chloroplastic (EC 2.5.1.54) (3-deoxy-D-arabino-heptulosonate 7-phosphate synthase 2) (DAHP synthase 2) (Phospho-2-keto-3-deoxyheptonate aldolase 2)	SHKB ARO2	Solanum tuberosum (Potato)	PATHWAY: Metabolic intermediate biosynthesis; chorismate biosynthesis; chorismate from D-erythrose 4-phosphate and phosphoenolpyruvate: step 1/7.	Amino-acid biosynthesis; Aromatic amino acid biosynthesis; Chloroplast; Complete proteome; Plastid; Reference proteome; Transferase; Transit peptide
SRR648705_TR34528_c0_g5_i1_len=1096	NA	NA	NA	NA	NA	PF14368.3^LTP_2^Probable lipid transfer^14-101^E:2.3e-11`PF00234.19^Tryp_alpha_amyl^Protease inhibitor/seed storage/LTP family^28-103^E:4.7e-09	0,502	-1,555	1	-1,432	1	0	1	0	0	-3,914	.	.	.	.	.	.
SRR648705_TR36195_c0_g1_i1_len=569	NA	NA	NA	NA	NA	.	0,134	-0,493	0,051	-0,868	0,698	-0,251	0,34	-0,501	0	-1,68	.	.	.	.	.	.
SRR648705_TR37405_c0_g1_i1_len=1033	Y5566_ARATH	Q:560-204,H:1-117	63.03%ID	E:2e-36	Uncharacterized protein At5g65660	.	1	0	1	0	1	0	1	0	0	-7,468	Y5566_ARATH	Uncharacterized protein At5g65660	At5g65660 K21L13.18	Arabidopsis thaliana (Mouse-ear cress)		Complete proteome; Membrane; Reference proteome; Transmembrane; Transmembrane helix
SRR648705_TR41240_c0_g2_i3_len=1205	VPS29_ARATH	Q:840-277,H:1-188	86.7%ID	E:2e-116	Vacuolar protein sorting-associated protein 29	PF12850.4^Metallophos_2^Calcineurin-like phosphoesterase superfamily domain^4-157^E:2.1e-19	0,626	0,141	0,539	0,209	0,002	0,991	0,003	0,956	0	-1,678	VPS29_ARATH	Vacuolar protein sorting-associated protein 29 (Protein MAIGO 1) (Vesicle protein sorting 29)	VPS29 MAG1 At3g47810 T23J7.140	Arabidopsis thaliana (Mouse-ear cress)		Complete proteome; Cytoplasm; Endosome; Golgi apparatus; Membrane; Protein transport; Reference proteome; Transport
SRR648705_TR41754_c0_g1_i1_len=539	NA	NA	NA	NA	NA	.	0,687	-0,107	0,012	0,602	0	0,909	0	1,044	0	-1,056	.	.	.	.	.	.
SRR648705_TR42481_c0_g3_i1_len=1978	ILV5_ARATH	Q:1025-1708,H:358-585	91.23%ID	E:5e-146	Ketol-acid reductoisomerase, chloroplastic	PF01450.16^IlvC^Acetohydroxy acid isomeroreductase, catalytic domain^2-85^E:1.8e-08`PF01450.16^IlvC^Acetohydroxy acid isomeroreductase, catalytic domain^98-174^E:1.7e-08	0,781	0,493	0,312	-0,74	1	-0,362	1	-0,496	0	-2,127	ILV5_ARATH	Ketol-acid reductoisomerase, chloroplastic (EC 1.1.1.86) (Acetohydroxy-acid reductoisomerase) (Alpha-keto-beta-hydroxylacyl reductoisomerase)	At3g58610 F14P22.200	Arabidopsis thaliana (Mouse-ear cress)	PATHWAY: Amino-acid biosynthesis; L-isoleucine biosynthesis; L-isoleucine from 2-oxobutanoate: step 2/4.; PATHWAY: Amino-acid biosynthesis; L-valine biosynthesis; L-valine from pyruvate: step 2/4.	Amino-acid biosynthesis; Branched-chain amino acid biosynthesis; Chloroplast; Complete proteome; Magnesium; Metal-binding; NADP; Oxidoreductase; Plastid; Reference proteome; Transit peptide
SRR648705_TR42562_c0_g1_i4_len=3061	XYL2_MEDSV	Q:225-2456,H:31-774	72.31%ID	E:0	Beta-xylosidase/alpha-L-arabinofuranosidase 2 {ECO:0000303|PubMed:17615411, ECO:0000312|EMBL:ABQ45228.1}	PF00933.18^Glyco_hydro_3^Glycosyl hydrolase family 3 N terminal domain^125-366^E:1.1e-39`PF01915.19^Glyco_hydro_3_C^Glycosyl hydrolase family 3 C-terminal domain^413-642^E:4e-50`PF14310.3^Fn3-like^Fibronectin type III-like domain^702-769^E:8.6e-09	0,093	-0,788	0,006	-3,13	1	0,673	1	0,283	0	-2,126	XYL2_MEDSV	Beta-xylosidase/alpha-L-arabinofuranosidase 2 (Xylan 1,4-beta-xylosidase/Alpha-L-arabinofuranosidase 2) (MsXyl2) [Includes: Beta-xylosidase (EC 3.2.1.37) (1,4-beta-D-xylan xylohydrolase) (Xylan 1,4-beta-xylosidase); Alpha-L-arabinofuranosidase (Arabinosidase) (EC 3.2.1.55)]	Xyl2	Medicago sativa subsp. varia (Alfalfa) (Medicago varia)		Carbohydrate metabolism; Extracellular matrix; Glycoprotein; Glycosidase; Hydrolase; Multifunctional enzyme; Polysaccharide degradation; Secreted; Signal; Xylan degradation
SRR648705_TR43184_c0_g2_i4_len=880	H2B1_MEDTR	Q:289-564,H:57-148	98.91%ID	E:3e-57	Probable histone H2B.1	PF00125.21^Histone^Core histone H2A/H2B/H3/H4^41-164^E:2.5e-22	0,006	-1,138	0,069	-1,074	0,472	-0,761	0,195	-1,129	0	-0,943	H2B1_MEDTR	Probable histone H2B.1		Medicago truncatula (Barrel medic) (Medicago tribuloides)		Acetylation; Chromosome; DNA-binding; Isopeptide bond; Nucleosome core; Nucleus; Ubl conjugation
SRR648705_TR45115_c0_g2_i1_len=518	NA	NA	NA	NA	NA	.	1	-2,541	1	-1,631	1	0	1	0	0	-3,77	.	.	.	.	.	.
SRR648705_TR45324_c0_g1_i1_len=900	NA	NA	NA	NA	NA	.	1	-0,061	1	-2,523	0,254	-2,474	0,254	-2,572	0,001	-2,645	.	.	.	.	.	.
SRR648707_TR1635_c0_g1_i2_len=1385	TLC1_SOLTU	Q:145-987,H:348-631	85.56%ID	E:3e-158	Plastidic ATP/ADP-transporter	PF03219.11^TLC^TLC ATP/ADP transporter^6-232^E:7e-95	1	-0,276	0,076	-2,148	1	0	1	0	0	-1,993	TLC1_SOLTU	Plastidic ATP/ADP-transporter		Solanum tuberosum (Potato)		ATP-binding; Chloroplast; Complete proteome; Membrane; Nucleotide-binding; Plastid; Reference proteome; Transmembrane; Transmembrane helix; Transport
SRR648707_TR4151_c0_g1_i1_len=1458	MYB6_ARATH	Q:1262-1456,H:3-69	77.61%ID	E:1e-27	Transcription repressor MYB6	PF14547.3^Hydrophob_seed^Hydrophobic seed protein^214-297^E:5.2e-24	0,132	-2,198	1	-1,09	1	0,407	1	-1,121	0	-2,021	MYB6_ARATH	Transcription repressor MYB6 (Myb-related protein 6) (AtMYB6)	MYB6 At4g09460 T15G18.120	Arabidopsis thaliana (Mouse-ear cress)		Complete proteome; DNA-binding; Nucleus; Reference proteome; Repeat; Repressor; Transcription; Transcription regulation
SRR648707_TR4176_c0_g2_i2_len=2300	AROG_SOLTU	Q:1956-478,H:30-510	84.79%ID	E:0	Phospho-2-dehydro-3-deoxyheptonate aldolase 2, chloroplastic	PF01474.13^DAHP_synth_2^Class-II DAHP synthetase family^2-384^E:1.3e-180	1	1,048	1	1,579	1	0,85	1	0,394	0	-6,934	AROG_SOLTU	Phospho-2-dehydro-3-deoxyheptonate aldolase 2, chloroplastic (EC 2.5.1.54) (3-deoxy-D-arabino-heptulosonate 7-phosphate synthase 2) (DAHP synthase 2) (Phospho-2-keto-3-deoxyheptonate aldolase 2)	SHKB ARO2	Solanum tuberosum (Potato)	PATHWAY: Metabolic intermediate biosynthesis; chorismate biosynthesis; chorismate from D-erythrose 4-phosphate and phosphoenolpyruvate: step 1/7.	Amino-acid biosynthesis; Aromatic amino acid biosynthesis; Chloroplast; Complete proteome; Plastid; Reference proteome; Transferase; Transit peptide
SRR648707_TR4925_c0_g1_i2_len=2058	P2C30_ARATH	Q:949-1638,H:156-389	67.09%ID	E:1e-99	Probable protein phosphatase 2C 30	PF00481.18^PP2C^Protein phosphatase 2C^6-220^E:9.5e-56	0	6,029	0	8,845	0,002	5,327	0	5,822	0	-2,226	P2C30_ARATH	Probable protein phosphatase 2C 30 (AtPP2C30) (EC 3.1.3.16) (AthPP2C5)	PP2C5 At2g40180 T7M7.17	Arabidopsis thaliana (Mouse-ear cress)		Complete proteome; Hydrolase; Magnesium; Manganese; Metal-binding; Protein phosphatase; Reference proteome
SRR648707_TR8141_c1_g2_i3_len=2582	SFGH_ARATH	Q:108-956,H:1-284	81.34%ID	E:3e-167	S-formylglutathione hydrolase	PF00756.17^Esterase^Putative esterase^25-275^E:2.7e-60`PF00326.18^Peptidase_S9^Prolyl oligopeptidase family^110-264^E:1.8e-08	1	-0,654	1	-0,302	1	0,452	1	0,899	0	-3,091	SFGH_ARATH	S-formylglutathione hydrolase (AtSFGH) (EC 3.1.2.12) (Esterase D)	SFGH At2g41530 T32G6.5	Arabidopsis thaliana (Mouse-ear cress)		Acetylation; Complete proteome; Hydrolase; Reference proteome; Serine esterase
SRR648707_TR9441_c0_g1_i1_len=2131	MYB44_ARATH	Q:1906-968,H:2-272	50%ID	E:5e-67	Transcription factor MYB44	PF00249.28^Myb_DNA-binding^Myb-like DNA-binding domain^14-59^E:9.5e-20`PF13921.3^Myb_DNA-bind_6^Myb-like DNA-binding domain^17-75^E:9.9e-18`PF00249.28^Myb_DNA-binding^Myb-like DNA-binding domain^68-110^E:9.6e-15`PF13921.3^Myb_DNA-bind_6^Myb-like DNA-binding domain^69-117^E:3e-09	0,798	-0,135	0,597	0,396	0,364	-0,489	0,029	-1,162	0	-5,433	MYB44_ARATH	Transcription factor MYB44 (Myb-related protein 44) (AtMYB44) (Myb-related protein R1) (AtMYBR1)	MYB44 MYBR1 At5g67300 K8K14.2	Arabidopsis thaliana (Mouse-ear cress)		Abscisic acid signaling pathway; Complete proteome; DNA-binding; Nucleus; Reference proteome; Repeat; Transcription; Transcription regulation
SRR648707_TR10670_c0_g1_i2_len=1490	EXO_ARATH	Q:687-1238,H:133-314	77.17%ID	E:4e-109	Protein EXORDIUM	PF04674.9^Phi_1^Phosphate-induced protein 1 conserved region^2-172^E:5.3e-84	1	0	1	-0,29	1	0	1	0	0	-1,923	EXO_ARATH	Protein EXORDIUM	EXO At4g08950 T3H13.3	Arabidopsis thaliana (Mouse-ear cress)		Apoplast; Complete proteome; Reference proteome; Secreted; Signal
SRR648707_TR11147_c1_g2_i2_len=1580	NA	NA	NA	NA	NA	.	1	0,017	0,01	-1,436	0,109	-1,093	0,004	-2,218	0	-1,717	.	.	.	.	.	.
SRR648707_TR12110_c0_g1_i1_len=1086	UFM1_ARATH	Q:815-564,H:2-85	96.43%ID	E:1e-49	Ubiquitin-fold modifier 1	.	0,502	2,097	1	0,537	0,254	4,695	1	2,118	0	-4,13	UFM1_ARATH	Ubiquitin-fold modifier 1	At1g77710 T32E8.4	Arabidopsis thaliana (Mouse-ear cress)		Complete proteome; Isopeptide bond; Reference proteome; Ubl conjugation pathway
SRR648707_TR15745_c1_g1_i1_len=2478	WDR26_XENTR	Q:700-2178,H:70-595	36.23%ID	E:1e-109	WD repeat-containing protein 26	PF00400.29^WD40^WD domain, G-beta repeat^264-298^E:3.5e-05`PF00400.29^WD40^WD domain, G-beta repeat^309-344^E:0.016`PF00400.29^WD40^WD domain, G-beta repeat^348-386^E:0.083`PF00400.29^WD40^WD domain, G-beta repeat^476-513^E:0.029`PF00400.29^WD40^WD domain, G-beta repeat^518-556^E:4.3e-08	1	0	1	-1,633	1	0	1	2,902	0	-7,058	WDR26_XENTR	WD repeat-containing protein 26	wdr26 TGas059a07.1	Xenopus tropicalis (Western clawed frog) (Silurana tropicalis)		Complete proteome; Cytoplasm; Reference proteome; Repeat; WD repeat
SRR648707_TR15942_c1_g1_i1_len=612	NA	NA	NA	NA	NA	.	0,006	-0,953	0,162	0,408	0,028	1,528	0,643	0,471	0,001	-1,066	.	.	.	.	.	.
SRR648707_TR17071_c0_g1_i1_len=648	NA	NA	NA	NA	NA	.	1	-3,126	0,038	-1,824	0,381	-2,081	0,381	-1,465	0	-2,089	.	.	.	.	.	.
SRR648707_TR23292_c0_g1_i1_len=1167	CBSX1_ARATH	Q:374-967,H:39-236	75.76%ID	E:2e-99	CBS domain-containing protein CBSX1, chloroplastic	PF00571.25^CBS^CBS domain^79-133^E:9.7e-14`PF00571.25^CBS^CBS domain^173-226^E:1.9e-16	0,611	-0,409	0,848	0,205	0,62	0,429	0,62	0,431	0	-8,234	CBSX1_ARATH	CBS domain-containing protein CBSX1, chloroplastic (CBS domain-containing protein 2) (AtCDCP2) (Protein LOSS OF THE TIMING OF ET AND JA BIOSYNTHESIS 2) (AtLEJ2)	CBSX1 CDCP2 At4g36910 AP22.61 C7A10.450	Arabidopsis thaliana (Mouse-ear cress)		3D-structure; Acetylation; CBS domain; Chloroplast; Complete proteome; Plastid; Reference proteome; Repeat; Transit peptide
SRR648707_TR24973_c1_g7_i1_len=3389	NDHI_TOBAC	Q:2614-3111,H:1-166	92.17%ID	E:2e-103	NAD(P)H-quinone oxidoreductase subunit I, chloroplastic {ECO:0000255|HAMAP-Rule:MF_01351}	PF00146.18^NADHdh^NADH dehydrogenase^33-184^E:2.9e-50	0,312	0,698	0,2	0,642	1	0,06	0,447	0,365	0	-1,402	NDHI_TOBAC	NAD(P)H-quinone oxidoreductase subunit I, chloroplastic (EC 1.6.5.-) (NAD(P)H dehydrogenase subunit I) (NDH subunit I) (NADH-plastoquinone oxidoreductase subunit I)	ndhI frxB	Nicotiana tabacum (Common tobacco)		4Fe-4S; Chloroplast; Direct protein sequencing; Iron; Iron-sulfur; Membrane; Metal-binding; NAD; NADP; Oxidoreductase; Plastid; Plastoquinone; Quinone; Repeat; Thylakoid
SRR648707_TR25740_c0_g1_i3_len=846	NA	NA	NA	NA	NA	.	0,002	-1,261	0	7,548	1	-0,849	1	-1,551	0,001	-1,079	.	.	.	.	.	.
SRR648707_TR26428_c1_g2_i5_len=1903	GCL1_ARATH	Q:1678-395,H:1-433	63.86%ID	E:0	LanC-like protein GCL1	PF05147.10^LANC_like^Lanthionine synthetase C-like protein^71-427^E:1.2e-85	0,076	2,889	0	6,683	1	3,354	0,502	4,006	0,001	-4,472	GCL1_ARATH	LanC-like protein GCL1 (G protein-coupled receptor 2-like protein 1) (Protein GCR2-like 1)	GCL1 At5g65280 MQN23.23	Arabidopsis thaliana (Mouse-ear cress)		Complete proteome; Reference proteome
SRR648707_TR32211_c0_g1_i6_len=753	NA	NA	NA	NA	NA	.	0,732	-0,677	0,123	-0,798	0,008	2,873	0,226	1,641	0	-2,08	.	.	.	.	.	.
SRR648707_TR37789_c0_g1_i1_len=1006	HMGL_IPONI	Q:40-432,H:1-126	71.76%ID	E:8e-45	HMG1/2-like protein	PF09011.7^HMG_box_2^HMG-box domain^37-109^E:2.6e-15`PF00505.16^HMG_box^HMG (high mobility group) box^40-109^E:1.5e-22	1	1,446	1	-0,251	1	-1,473	1	-0,088	0	-1,502	HMGL_IPONI	HMG1/2-like protein		Ipomoea nil (Japanese morning glory) (Pharbitis nil)		DNA-binding; Nucleus
SRR648707_TR38056_c0_g1_i2_len=1315	PYL4_ARATH	Q:365-847,H:42-202	78.4%ID	E:1e-74	Abscisic acid receptor PYL4	PF10604.6^Polyketide_cyc2^Polyketide cyclase / dehydrase and lipid transport^132-272^E:2.4e-19`PF00407.16^Bet_v_1^Pathogenesis-related protein Bet v I family^157-240^E:1.6e-05	1	-0,119	1	0,083	0,44	-0,708	1	0,194	0	-2,794	PYL4_ARATH	Abscisic acid receptor PYL4 (ABI1-binding protein 2) (PYR1-like protein 4) (Regulatory components of ABA receptor 10)	PYL4 ABIP2 RCAR10 At2g38310 T19C21.20	Arabidopsis thaliana (Mouse-ear cress)		Abscisic acid signaling pathway; Cell membrane; Complete proteome; Cytoplasm; Disulfide bond; Membrane; Nucleus; Protein phosphatase inhibitor; Receptor; Reference proteome
SRR924147_TR5744_c0_g1_i2_len=1118	NA	NA	NA	NA	NA	.	1	-0,245	1	-0,473	1	0,249	1	-0,437	0	-2,509	.	.	.	.	.	.
SRR924147_TR6268_c0_g1_i1_len=1747	FLA2_ARATH	Q:299-1222,H:25-332	65.91%ID	E:1e-147	Fasciclin-like arabinogalactan protein 2	PF02469.19^Fasciclin^Fasciclin domain^36-114^E:7.3e-06`PF02469.19^Fasciclin^Fasciclin domain^197-326^E:1e-12	0,593	0,649	0,073	2,409	0,56	0,632	0,195	1,085	0	-1,354	FLA2_ARATH	Fasciclin-like arabinogalactan protein 2	FLA2 At4g12730 T20K18.80	Arabidopsis thaliana (Mouse-ear cress)		Cell membrane; Complete proteome; GPI-anchor; Glycoprotein; Lipoprotein; Membrane; Proteoglycan; Reference proteome; Repeat; Signal
SRR924147_TR7580_c0_g3_i1_len=635	NA	NA	NA	NA	NA	.	1	0	0,111	-0,828	0,017	5,834	1	0	0	-5,47	.	.	.	.	.	.
SRR924147_TR10087_c0_g1_i8_len=2390	ICT1_SALSA	Q:2110-1577,H:13-180	35.91%ID	E:3e-23	Peptidyl-tRNA hydrolase ICT1, mitochondrial	PF00472.17^RF-1^RF-1 domain^100-240^E:1.5e-19	1	-0,143	0,86	-0,207	0,231	-0,644	0,443	-0,399	0	-7,018	ICT1_SALSA	Peptidyl-tRNA hydrolase ICT1, mitochondrial (EC 3.1.1.29) (Immature colon carcinoma transcript 1 protein homolog)	ict1	Salmo salar (Atlantic salmon)		Hydrolase; Mitochondrion; Protein biosynthesis; Transit peptide
SRR924147_TR14360_c0_g1_i5_len=1287	NA	NA	NA	NA	NA	PF03652.12^RuvX^Holliday junction resolvase^23-158^E:1.9e-23	1	-0,191	1	1,846	1	0	1	0	0	-6,967	.	.	.	.	.	.
SRR924147_TR16625_c0_g2_i1_len=1517	PYL4_ARATH	Q:403-873,H:38-196	80.5%ID	E:1e-75	Abscisic acid receptor PYL4	PF10604.6^Polyketide_cyc2^Polyketide cyclase / dehydrase and lipid transport^78-213^E:9.8e-20`PF03364.17^Polyketide_cyc^Polyketide cyclase / dehydrase and lipid transport^80-184^E:1.6e-05`PF00407.16^Bet_v_1^Pathogenesis-related protein Bet v I family^97-178^E:2.8e-05	0,793	-0,182	1	0,019	0,02	-1,106	0,14	-0,68	0	-4,412	PYL4_ARATH	Abscisic acid receptor PYL4 (ABI1-binding protein 2) (PYR1-like protein 4) (Regulatory components of ABA receptor 10)	PYL4 ABIP2 RCAR10 At2g38310 T19C21.20	Arabidopsis thaliana (Mouse-ear cress)		Abscisic acid signaling pathway; Cell membrane; Complete proteome; Cytoplasm; Disulfide bond; Membrane; Nucleus; Protein phosphatase inhibitor; Receptor; Reference proteome
SRR924147_TR19995_c0_g1_i1_len=1035	GPX1_HELAN	Q:820-341,H:7-166	77.5%ID	E:2e-77	Glutathione peroxidase 1	PF00255.16^GSHPx^Glutathione peroxidase^6-114^E:5.8e-44`PF00578.18^AhpC-TSA^AhpC/TSA family^6-77^E:6.6e-09`PF08534.7^Redoxin^Redoxin^8-65^E:6.3e-07	0,543	0,249	0,231	0,649	0,001	1,511	0,002	1,477	0	-3,164	GPX1_HELAN	Glutathione peroxidase 1 (EC 1.11.1.9)	GPXHA-1	Helianthus annuus (Common sunflower)		Oxidoreductase; Peroxidase
SRR924147_TR24325_c0_g1_i1_len=4429	PMT6_ARATH	Q:237-2012,H:10-591	67.68%ID	E:0	Probable methyltransferase PMT6	PF07887.8^Calmodulin_bind^Calmodulin binding protein-like^84-375^E:2.7e-131	1	1,452	1	0,37	1	0	1	0	0,001	-6,569	PMT6_ARATH	Probable methyltransferase PMT6 (EC 2.1.1.-)	At3g10200 F14P13.20	Arabidopsis thaliana (Mouse-ear cress)		Complete proteome; Endoplasmic reticulum; Glycoprotein; Membrane; Methyltransferase; Reference proteome; Signal-anchor; Transferase; Transmembrane; Transmembrane helix
SRR924147_TR26387_c0_g4_i1_len=2188	RL9_PEA	Q:179-751,H:3-193	86.91%ID	E:9e-111	60S ribosomal protein L9	PF00347.20^Ribosomal_L6^Ribosomal protein L6^12-89^E:6e-11`PF00347.20^Ribosomal_L6^Ribosomal protein L6^102-180^E:6.5e-12	1	0,564	1	-0,31	1	0,769	1	0,051	0	-4,962	RL9_PEA	60S ribosomal protein L9 (Gibberellin-regulated protein GA)	RPL9	Pisum sativum (Garden pea)		Ribonucleoprotein; Ribosomal protein
SRR924147_TR27996_c0_g1_i2_len=3254	RFS2_ARATH	Q:2669-345,H:1-773	77.96%ID	E:0	Probable galactinol–sucrose galactosyltransferase 2	PF05691.9^Raffinose_syn^Raffinose synthase or seed imbibition protein Sip1^7-759^E:0	1	0,405	1	0,545	1	0,443	1	0,139	0	-6,464	RFS2_ARATH	Probable galactinol–sucrose galactosyltransferase 2 (EC 2.4.1.82) (Protein SEED IMBIBITION 2) (Raffinose synthase 2)	RFS2 RS2 SIP2 At3g57520 T8H10.120	Arabidopsis thaliana (Mouse-ear cress)		Alternative splicing; Carbohydrate metabolism; Complete proteome; Glycosyltransferase; Reference proteome; Transferase
SRR924147_TR30056_c0_g1_i1_len=1548	CYT12_ORYSJ	Q:1165-551,H:39-244	65.22%ID	E:5e-83	Cysteine proteinase inhibitor 12	PF00031.18^Cystatin^Cystatin domain^48-127^E:5.9e-15`PF16845.2^SQAPI^Aspartic acid proteinase inhibitor^51-127^E:4.9e-21	1	-0,224	1	-0,418	1	0,47	1	0	0	-5,953	CYT12_ORYSJ	Cysteine proteinase inhibitor 12 (Oryzacystatin XII) (OC-XII) (Oryzacystatin-12)	Os01g0270100 LOC_Os01g16430 P0667A10.20	Oryza sativa subsp. japonica (Rice)		Complete proteome; Plant defense; Protease inhibitor; Reference proteome; Repeat; Secreted; Signal; Thiol protease inhibitor
SRR924147_TR36612_c2_g3_i3_len=3422	PSAA_OLIPU	Q:2624-3421,H:1-266	99.62%ID	E:1e-165	Photosystem I P700 chlorophyll a apoprotein A1 {ECO:0000255|HAMAP-Rule:MF_00458}	PF00223.16^PsaA_PsaB^Photosystem I psaA/psaB protein^32-266^E:1.3e-114	0,763	-0,185	0,082	-0,895	1	-0,006	1	0,044	0	-1,614	PSAA_OLIPU	Photosystem I P700 chlorophyll a apoprotein A1 (EC 1.97.1.12) (PSI-A) (PsaA)	psaA	Olimarabidopsis pumila (Dwarf rocket) (Arabidopsis griffithiana)		4Fe-4S; Chlorophyll; Chloroplast; Chromophore; Electron transport; Iron; Iron-sulfur; Magnesium; Membrane; Metal-binding; Oxidoreductase; Photosynthesis; Photosystem I; Plastid; Thylakoid; Transmembrane; Transmembrane helix; Transport
SRR924147_TR37699_c0_g1_i1_len=2015	ALF_SPIOL	Q:158-1228,H:1-357	85.15%ID	E:0	Fructose-bisphosphate aldolase, cytoplasmic isozyme	PF00274.16^Glycolytic^Fructose-bisphosphate aldolase class-I^11-357^E:1.4e-177	0,162	-0,347	0,005	0,7	0,719	-0,094	0,168	-0,343	0	-1,057	ALF_SPIOL	Fructose-bisphosphate aldolase, cytoplasmic isozyme (EC 4.1.2.13)		Spinacia oleracea (Spinach)	PATHWAY: Carbohydrate degradation; glycolysis; D-glyceraldehyde 3-phosphate and glycerone phosphate from D-glucose: step 4/4.	Cytoplasm; Glycolysis; Lyase; Schiff base
SRR924147_TR38118_c0_g1_i5_len=1943	IPCS1_ARATH	Q:514-1440,H:1-304	76.05%ID	E:3e-160	Phosphatidylinositol:ceramide inositolphosphotransferase 1	PF14360.3^PAP2_C^PAP2 superfamily C-terminal^172-240^E:1.3e-22	0,282	-1,142	0,502	-4,306	1	0,232	1	0,064	0	-7,171	IPCS1_ARATH	Phosphatidylinositol:ceramide inositolphosphotransferase 1 (EC 2.7.8.-) (Inositol-phosphorylceramide synthase 1) (AtIPCS1) (IPC synthase 1) (Protein ERH1-like2) (Sphingolipid synthase 1)	IPCS1 ERHL2 At3g54020 F5K20.320	Arabidopsis thaliana (Mouse-ear cress)		Complete proteome; Lipid biosynthesis; Lipid metabolism; Membrane; Reference proteome; Sphingolipid metabolism; Transferase; Transmembrane; Transmembrane helix
SRR924148_TR490_c1_g1_i1_len=1696	NA	NA	NA	NA	NA	PF09598.7^Stm1_N^Stm1^1-76^E:8e-23`PF04774.12^HABP4_PAI-RBP1^Hyaluronan / mRNA binding family^155-241^E:2.1e-10	0,461	-1,012	1	-0,354	1	-2,202	1	-2,202	0	-3,227	.	.	.	.	.	.
SRR924148_TR490_c1_g1_i3_len=1762	NA	NA	NA	NA	NA	PF09598.7^Stm1_N^Stm1^1-76^E:8.9e-23`PF04774.12^HABP4_PAI-RBP1^Hyaluronan / mRNA binding family^155-263^E:3.3e-30	1	0	1	0	1	0	1	0	0	-1,84	.	.	.	.	.	.
SRR924148_TR7786_c1_g1_i1_len=1714	BAG3_ARATH	Q:904-242,H:66-278	46.85%ID	E:3e-58	BAG family molecular chaperone regulator 3	PF02179.13^BAG^BAG domain^8-83^E:2.2e-14	1	0,48	1	-0,879	1	-1,157	1	-0,942	0	-3,234	BAG3_ARATH	BAG family molecular chaperone regulator 3 (Bcl-2-associated athanogene 3)	BAG3 At5g07220 T28J14_160	Arabidopsis thaliana (Mouse-ear cress)		3D-structure; Chaperone; Complete proteome; Phosphoprotein; Reference proteome
SRR924148_TR14728_c0_g1_i1_len=1118	NA	NA	NA	NA	NA	.	0,885	-0,061	0,003	-1,255	0,055	-0,73	0,005	-1,108	0,001	-0,869	.	.	.	.	.	.
SRR924148_TR17236_c0_g2_i3_len=1407	APX1_ARATH	Q:294-1043,H:1-250	85.6%ID	E:4e-145	L-ascorbate peroxidase 1, cytosolic	PF00141.20^peroxidase^Peroxidase^22-227^E:4.7e-49	0,986	0,007	0,005	0,638	0	1,123	0	1,015	0	-1,554	APX1_ARATH	L-ascorbate peroxidase 1, cytosolic (AP) (AtAPx01) (EC 1.11.1.11)	APX1 At1g07890 F24B9.2	Arabidopsis thaliana (Mouse-ear cress)		Alternative splicing; Calcium; Complete proteome; Cytoplasm; Direct protein sequencing; Heme; Hydrogen peroxide; Iron; Metal-binding; Oxidoreductase; Peroxidase; Phosphoprotein; Potassium; Reference proteome
SRR924148_TR17912_c0_g3_i1_len=2017	TBB7_GOSHI	Q:513-1790,H:1-426	94.84%ID	E:0	Tubulin beta-7 chain	PF00091.22^Tubulin^Tubulin/FtsZ family, GTPase domain^3-211^E:4e-69`PF03953.14^Tubulin_C^Tubulin C-terminal domain^261-381^E:5.8e-41	1	0,139	1	0,78	1	0,73	1	1,185	0	-1,411	TBB7_GOSHI	Tubulin beta-7 chain (Beta-7-tubulin)		Gossypium hirsutum (Upland cotton) (Gossypium mexicanum)		Cytoplasm; Cytoskeleton; GTP-binding; Microtubule; Nucleotide-binding
SRR924148_TR18890_c0_g1_i1_len=528	PSBZ_COFAR	Q:496-311,H:1-62	98.39%ID	E:9e-24	Photosystem II reaction center protein Z {ECO:0000255|HAMAP-Rule:MF_00644}	.	0,849	-0,119	0,111	-0,393	0,271	-0,266	0,485	-0,167	0	-11,904	PSBZ_COFAR	Photosystem II reaction center protein Z (PSII-Z)	psbZ	Coffea arabica (Arabian coffee)		Chloroplast; Membrane; Photosynthesis; Photosystem II; Plastid; Reaction center; Thylakoid; Transmembrane; Transmembrane helix
SRR924148_TR25300_c1_g2_i2_len=996	UXS6_ARATH	Q:125-661,H:165-343	85.47%ID	E:2e-107	UDP-glucuronic acid decarboxylase 6	PF16363.2^GDP_Man_Dehyd^GDP-mannose 4,6 dehydratase^7-164^E:2.4e-22`PF01370.18^Epimerase^NAD dependent epimerase/dehydratase family^7-103^E:1.9e-18	0,228	-1,203	0,02	-1,17	0,017	5,799	1	0	0	-3,016	UXS6_ARATH	UDP-glucuronic acid decarboxylase 6 (EC 4.1.1.35) (UDP-XYL synthase 6) (UDP-glucuronate decarboxylase 6) (UGD) (UXS-6)	UXS6 At2g28760 F8N16.5	Arabidopsis thaliana (Mouse-ear cress)	PATHWAY: Nucleotide-sugar biosynthesis; UDP-alpha-D-xylose biosynthesis; UDP-alpha-D-xylose from UDP-alpha-D-glucuronate: step 1/1.	Acetylation; Complete proteome; Cytoplasm; Decarboxylase; Lyase; NAD; Reference proteome
SRR924148_TR31225_c1_g2_i1_len=829	NA	NA	NA	NA	NA	.	1	0,199	0,299	1,327	1	0,69	0	4,497	0	-3,286	.	.	.	.	.	.
SRR924148_TR34958_c0_g1_i7_len=3353	PUMP2_ARATH	Q:2725-2441,H:146-240	80%ID	E:9e-80	Mitochondrial uncoupling protein 2	PF00153.24^Mito_carr^Mitochondrial carrier protein^7-105^E:3e-20	0,001	-6,47	0,073	1,828	0,066	5,293	1	0	0,001	-6,647	PUMP2_ARATH	Mitochondrial uncoupling protein 2 (AtPUMP2)	PUMP2 UCP2 At5g58970 K19M22.16	Arabidopsis thaliana (Mouse-ear cress)		Alternative splicing; Complete proteome; Membrane; Mitochondrion; Mitochondrion inner membrane; Reference proteome; Repeat; Stress response; Transmembrane; Transmembrane helix; Transport
SRR924148_TR35554_c2_g4_i1_len=1790	RR12B_POPTR	Q:1585-1788,H:33-100	91.18%ID	E:2e-31	30S ribosomal protein S12-B, chloroplastic	.	0,732	0,663	0,76	-0,671	1	-0,193	1	0,035	0	-1,872	RR12B_POPTR	30S ribosomal protein S12-B, chloroplastic	rps12-B Poptr_cp071	Populus trichocarpa (Western balsam poplar) (Populus balsamifera subsp. trichocarpa)		Chloroplast; Complete proteome; Plastid; RNA-binding; Reference proteome; Ribonucleoprotein; Ribosomal protein; rRNA-binding
SRR924148_TR39477_c1_g2_i3_len=9841	TI214_COFAR	Q:2469-6482,H:467-1873	68.68%ID	E:0	Protein TIC 214 {ECO:0000250|UniProtKB:P56785}	PF05758.9^Ycf1^Ycf1^64-413^E:1.3e-172	0,461	-1,191	0,629	-1,04	1	-0,582	1	-0,232	0	-1,222	TI214_COFAR	Protein TIC 214 (Translocon at the inner envelope membrane of chloroplasts 214) (AtTIC214)	TIC214 ycf1	Coffea arabica (Arabian coffee)		Chloroplast; Membrane; Plastid; Plastid inner membrane; Protein transport; Transmembrane; Transmembrane helix; Transport
SRR924148_TR40067_c0_g5_i2_len=2706	NA	NA	NA	NA	NA	PF04782.9^DUF632^Protein of unknown function (DUF632)^1-281^E:3.3e-91	1	0	1	0	1	0	1	0	0,001	-2,094	.	.	.	.	.	.
SRR924148_TR41331_c0_g1_i1_len=920	NA	NA	NA	NA	NA	.	0,02	-0,779	0,153	-0,65	0,796	-0,107	1	-0,03	0	-2,163	.	.	.	.	.	.

Ms6h

Transcript (CDF97)	Uniprot homolog	BLASTX Position	BLASTX %Identity	BLASTX evalue	Uniprot descr	Pfam	PValue_Ms6h	logFC_Ms6h	PValue_Ms8h	logFC_Ms8h	PValue_MsDamaged24h	logFC_MsDamaged24h	PValue_MsIntact24h	logFC_MsIntact24h	PValue_Ms1wk	logFC_Ms1wk	Entry.name	Protein names	Gene names	Organism	Pathway	Keywords
SRR1144633_TR2782_c0_g1_i1_len=1427	RL3_ORYSJ	Q:1362-196,H:1-389	87.92%ID	E:0	60S ribosomal protein L3	PF00297.19^Ribosomal_L3^Ribosomal protein L3^1-370^E:1.7e-194	0	-1,322	0,245	-0,333	0,01	1,003	0,153	0,598	1	0	RL3_ORYSJ	60S ribosomal protein L3	RPL3 Os12g0167900 LOC_Os12g07010	Oryza sativa subsp. japonica (Rice)		Complete proteome; Cytoplasm; Reference proteome; Ribonucleoprotein; Ribosomal protein
SRR1144633_TR3797_c0_g1_i4_len=1173	RS3A_CATRO	Q:949-251,H:28-261	98.29%ID	E:1e-156	40S ribosomal protein S3a {ECO:0000255|HAMAP-Rule:MF_03122}	PF01015.15^Ribosomal_S3Ae^Ribosomal S3Ae family^15-221^E:9.3e-100	0,001	-1,016	0,412	-0,246	0,376	0,356	0,736	0,163	1	-0,016	RS3A_CATRO	40S ribosomal protein S3a (CYC07 protein)	RPS3A CYC07	Catharanthus roseus (Madagascar periwinkle) (Vinca rosea)		Cytoplasm; Ribonucleoprotein; Ribosomal protein
SRR1144633_TR8015_c1_g2_i1_len=1171	GDL67_ARATH	Q:108-1136,H:25-367	69.19%ID	E:3e-171	GDSL esterase/lipase At4g28780	PF00657.19^Lipase_GDSL^GDSL-like Lipase/Acylhydrolase^44-352^E:1.1e-14	0	-1,741	0,16	-1,273	0,002	-1,366	0,002	-1,324	0,189	0,38	GDL67_ARATH	GDSL esterase/lipase At4g28780 (EC 3.1.1.-) (Extracellular lipase At4g28780)	At4g28780 F16A16.110	Arabidopsis thaliana (Mouse-ear cress)		Complete proteome; Glycoprotein; Hydrolase; Lipid degradation; Lipid metabolism; Reference proteome; Secreted; Signal
SRR1144633_TR8510_c0_g2_i1_len=984	RL15_PETHY	Q:140-751,H:1-204	94.61%ID	E:2e-117	60S ribosomal protein L15	PF00827.14^Ribosomal_L15e^Ribosomal L15^2-190^E:1.1e-94	0	-0,957	0,057	-0,443	0,036	0,602	0,516	0,213	1	1,801	RL15_PETHY	60S ribosomal protein L15	RPL15	Petunia hybrida (Petunia)		Ribonucleoprotein; Ribosomal protein
SRR1144633_TR8540_c0_g1_i2_len=1377	RIR2_TOBAC	Q:325-1311,H:1-329	90.27%ID	E:0	Ribonucleoside-diphosphate reductase small chain	PF00268.18^Ribonuc_red_sm^Ribonucleotide reductase, small chain^16-282^E:6.5e-120	0	-1,354	0,017	-1,375	1	-0,069	0,545	-0,481	0,111	-0,558	RIR2_TOBAC	Ribonucleoside-diphosphate reductase small chain (EC 1.17.4.1) (Ribonucleoside-diphosphate reductase R2 subunit) (Ribonucleotide reductase small subunit)		Nicotiana tabacum (Common tobacco)	PATHWAY: Genetic information processing; DNA replication.	Cytoplasm; DNA replication; Iron; Metal-binding; Oxidoreductase
SRR1144633_TR10421_c0_g1_i1_len=837	RL182_ARATH	Q:155-715,H:1-187	86.1%ID	E:2e-114	60S ribosomal protein L18-2	PF17135.1^Ribosomal_L18^Ribosomal protein 60S L18 and 50S L18e^2-187^E:8.7e-96`PF00828.16^Ribosomal_L27A^Ribosomal proteins 50S-L15, 50S-L18e, 60S-L27A^46-120^E:0.0001	0	-1,074	1	0,009	1	-0,029	0,779	-0,121	0,301	-0,307	RL182_ARATH	60S ribosomal protein L18-2	RPL18B At3g05590 F18C1.14	Arabidopsis thaliana (Mouse-ear cress)		Complete proteome; Cytoplasm; Reference proteome; Ribonucleoprotein; Ribosomal protein
SRR1144633_TR12716_c0_g1_i1_len=1085	RL8_SOLLC	Q:100-813,H:1-238	95.8%ID	E:2e-167	60S ribosomal protein L8	PF00181.20^Ribosomal_L2^Ribosomal Proteins L2, RNA binding domain^13-90^E:2.8e-18`PF03947.15^Ribosomal_L2_C^Ribosomal Proteins L2, C-terminal domain^98-230^E:1.8e-46	0	-0,987	0,454	-0,196	0,607	0,159	0,404	-0,257	0,068	-0,46	RL8_SOLLC	60S ribosomal protein L8 (L2) (Ribosomal protein TL2)	RPL8 RPL2	Solanum lycopersicum (Tomato) (Lycopersicon esculentum)		Complete proteome; Cytoplasm; Reference proteome; Ribonucleoprotein; Ribosomal protein
SRR1144633_TR22705_c0_g1_i1_len=1281	CB22_TOBAC	Q:181-975,H:1-265	93.96%ID	E:0	Chlorophyll a-b binding protein 21, chloroplastic	PF00504.18^Chloroa_b-bind^Chlorophyll A-B binding protein^65-231^E:1.6e-50	0	-0,975	0,048	0,654	0,004	-0,781	0,172	-0,364	0	1,654	CB22_TOBAC	Chlorophyll a-b binding protein 21, chloroplastic (LHCII type I CAB-21) (LHCP)	CAB21	Nicotiana tabacum (Common tobacco)		Acetylation; Chlorophyll; Chloroplast; Chromophore; Magnesium; Membrane; Metal-binding; Phosphoprotein; Photosynthesis; Photosystem I; Photosystem II; Plastid; Thylakoid; Transit peptide; Transmembrane; Transmembrane helix
SRR1144633_TR22897_c0_g1_i1_len=792	RLA3_MAIZE	Q:205-567,H:1-120	62.81%ID	E:3e-24	60S acidic ribosomal protein P3	PF00428.16^Ribosomal_60s^60s Acidic ribosomal protein^67-120^E:4.2e-12	0	-0,944	0,848	0,048	0,01	0,779	0,186	0,422	0,109	-0,377	RLA3_MAIZE	60S acidic ribosomal protein P3 (P1/P2-like) (P3A)	RPP3A	Zea mays (Maize)		Complete proteome; Direct protein sequencing; Phosphoprotein; Reference proteome; Ribonucleoprotein; Ribosomal protein
SRR1144633_TR23012_c0_g2_i1_len=633	RS25_SOLLC	Q:269-481,H:38-108	85.92%ID	E:2e-34	40S ribosomal protein S25	PF03297.12^Ribosomal_S25^S25 ribosomal protein^6-104^E:6.8e-40	0,001	-1,115	0,777	-0,106	0,833	0,109	0,823	-0,174	0,151	-0,477	RS25_SOLLC	40S ribosomal protein S25	RPS25	Solanum lycopersicum (Tomato) (Lycopersicon esculentum)		Complete proteome; Reference proteome; Ribonucleoprotein; Ribosomal protein
SRR1144633_TR27033_c0_g1_i2_len=801	RL262_ARATH	Q:676-359,H:1-106	85.85%ID	E:4e-52	60S ribosomal protein L26-2	PF16906.2^Ribosomal_L26^Ribosomal proteins L26 eukaryotic, L24P archaeal^8-121^E:8.3e-39`PF00467.26^KOW^KOW motif^51-82^E:7.8e-10	0	-1,319	1	0,009	0,783	0,165	0,889	0,084	0,154	0,783	RL262_ARATH	60S ribosomal protein L26-2	RPL26B At5g67510 K9I9.7	Arabidopsis thaliana (Mouse-ear cress)		Complete proteome; Reference proteome; Ribonucleoprotein; Ribosomal protein

SRR1144634_TR6469_c2_g1_i2_len=653	NA	NA	NA	NA	NA	.	0	-7,496	0,377	-0,586	0,009	5,932	0,017	5,803	0,059	-1,022	.	.	.	.	.	.
SRR1144634_TR8715_c0_g2_i1_len=545	EF1A_TOBAC	Q:2-427,H:295-436	97.89%ID	E:1e-94	Elongation factor 1-alpha	PF03143.14^GTP_EFTU_D3^Elongation factor Tu C-terminal domain^28-135^E:3.2e-39	0	-1,391	0,496	-0,262	0,431	0,35	0,419	-0,404	0,023	-0,505	EF1A_TOBAC	Elongation factor 1-alpha (EF-1-alpha) (Vitronectin-like adhesion protein 1) (PVN1)		Nicotiana tabacum (Common tobacco)		Cytoplasm; Elongation factor; GTP-binding; Nucleotide-binding; Phosphoprotein; Protein biosynthesis
SRR1144634_TR22781_c0_g1_i2_len=846	RL23_ARATH	Q:180-599,H:1-140	97.14%ID	E:4e-93	60S ribosomal protein L23	PF00238.16^Ribosomal_L14^Ribosomal protein L14p/L23e^22-140^E:8.9e-36	0	-0,96	0,155	-0,323	0,229	0,303	0,478	-0,195	0,211	-0,273	RL23_ARATH	60S ribosomal protein L23 (Protein EMBRYO DEFECTIVE 2171)	RPL23A At1g04480 F19P19.5; RPL23B At2g33370 F4P9.14; RPL23C EMB2171 At3g04400 T27C4.4	Arabidopsis thaliana (Mouse-ear cress)		Alternative splicing; Complete proteome; Reference proteome; Ribonucleoprotein; Ribosomal protein
SRR1144634_TR25396_c0_g1_i1_len=504	NRP2_ARATH	Q:144-485,H:1-107	70.18%ID	E:1e-44	NAP1-related protein 2	PF00956.15^NAP^Nucleosome assembly protein (NAP)^33-77^E:2.3e-07	0	-8,412	0	6,758	1	0	1	2,591	0	6,924	NRP2_ARATH	NAP1-related protein 2 (Nucleosome/chromatin assembly factor group A5) (Protein SET homolog 2)	NRP2 NFA5 At1g18800 F6A14.10	Arabidopsis thaliana (Mouse-ear cress)		Alternative splicing; Chaperone; Coiled coil; Complete proteome; Cytoplasm; Nucleus; Reference proteome
SRR1144634_TR31581_c0_g1_i1_len=930	RS33_ARATH	Q:69-734,H:1-222	89.64%ID	E:4e-145	40S ribosomal protein S3-3	PF07650.14^KH_2^KH domain^20-93^E:5.8e-12`PF00189.17^Ribosomal_S3_C^Ribosomal protein S3, C-terminal domain^106-188^E:4.4e-25	0	-0,85	0,175	0,306	0,285	0,257	0,713	0,092	1	-1,526	RS33_ARATH	40S ribosomal protein S3-3	RPS3C At5g35530 MOK9.14	Arabidopsis thaliana (Mouse-ear cress)		Complete proteome; Isopeptide bond; Phosphoprotein; RNA-binding; Reference proteome; Ribonucleoprotein; Ribosomal protein; Ubl conjugation
SRR1144634_TR32698_c0_g2_i1_len=703	RL34_TOBAC	Q:98-382,H:1-95	96.84%ID	E:4e-59	60S ribosomal protein L34	PF01199.15^Ribosomal_L34e^Ribosomal protein L34e^1-96^E:1e-38	0	-1,416	0,596	0,142	0,773	0,115	0,532	-0,218	0,202	-0,29	RL34_TOBAC	60S ribosomal protein L34	RPL34	Nicotiana tabacum (Common tobacco)		Ribonucleoprotein; Ribosomal protein
SRR122236_TR3141_c0_g1_i1_len=2618	NUCL2_ORYSJ	Q:1082-492,H:435-630	56.28%ID	E:3e-62	Nucleolin 2	PF00076.19^RRM_1^RNA recognition motif. (a.k.a. RRM, RBD, or RNP domain)^30-100^E:1e-19`PF13893.3^RRM_5^RNA recognition motif. (a.k.a. RRM, RBD, or RNP domain)^69-103^E:0.17`PF00076.19^RRM_1^RNA recognition motif. (a.k.a. RRM, RBD, or RNP domain)^133-203^E:2.7e-08	0	-3,8	0	2,321	1	0	1	0	1	0,314	NUCL2_ORYSJ	Nucleolin 2 (Protein NUCLEOLIN LIKE 2)	Os04g0620700 LOC_Os04g52960 OSJNBa0058K23.21	Oryza sativa subsp. japonica (Rice)		Complete proteome; Nucleus; RNA-binding; Reference proteome; Repeat; rRNA processing
SRR122237_TR4116_c0_g1_i1_len=720	RL273_ARATH	Q:607-203,H:1-135	80%ID	E:2e-62	60S ribosomal protein L27-3	PF01777.15^Ribosomal_L27e^Ribosomal L27e protein family^52-135^E:6.9e-34	0	-0,942	0,343	-0,228	0,565	-0,153	0,145	-0,397	0,932	0,058	RL273_ARATH	60S ribosomal protein L27-3	RPL27C At4g15000 dl3545w FCAALL.99	Arabidopsis thaliana (Mouse-ear cress)		Alternative splicing; Complete proteome; Reference proteome; Ribonucleoprotein; Ribosomal protein
SRR122237_TR6908_c0_g2_i1_len=554	RL9_PEA	Q:417-1,H:1-139	87.77%ID	E:2e-84	60S ribosomal protein L9	PF00347.20^Ribosomal_L6^Ribosomal protein L6^12-89^E:2.5e-12	0	-1,033	0,012	0,737	0,4	-0,268	0,137	-0,451	1	-0,017	RL9_PEA	60S ribosomal protein L9 (Gibberellin-regulated protein GA)	RPL9	Pisum sativum (Garden pea)		Ribonucleoprotein; Ribosomal protein
SRR122237_TR9671_c0_g1_i1_len=1018	RL74_ARATH	Q:946-209,H:1-244	82.93%ID	E:9e-138	60S ribosomal protein L7-4	PF08079.9^Ribosomal_L30_N^Ribosomal L30 N-terminal domain^36-107^E:5.7e-27`PF00327.17^Ribosomal_L30^Ribosomal protein L30p/L7e^112-162^E:5.8e-22	0	-1,279	1	-0,02	0,229	0,519	0,407	0,376	0,542	0,212	RL74_ARATH	60S ribosomal protein L7-4	RPL7D At3g13580 K20M4.2	Arabidopsis thaliana (Mouse-ear cress)		Complete proteome; Reference proteome; Ribonucleoprotein; Ribosomal protein
SRR122237_TR27836_c0_g1_i1_len=594	NA	NA	NA	NA	NA	.	0	-1,589	0,38	0,222	0,451	0,302	1	-0,017	0,517	-1,063	.	.	.	.	.	.
SRR122238_TR4231_c0_g1_i2_len=703	NA	NA	NA	NA	NA	.	0	-1,184	0,341	0,427	0,108	-0,748	0,637	0,208	0,666	0,34	.	.	.	.	.	.
SRR122238_TR4631_c2_g1_i4_len=535	RL11_MEDSA	Q:465-1,H:1-155	96.13%ID	E:9e-98	60S ribosomal protein L11	PF00281.16^Ribosomal_L5^Ribosomal protein L5^32-85^E:5.7e-21`PF00673.18^Ribosomal_L5_C^ribosomal L5P family C-terminus^89-173^E:4.7e-20	0	-1,29	0,679	-0,101	0,38	0,245	0,802	-0,074	0,862	-0,062	RL11_MEDSA	60S ribosomal protein L11 (L5)	RPL11 RPL5	Medicago sativa (Alfalfa)		Cytoplasm; RNA-binding; Ribonucleoprotein; Ribosomal protein; rRNA-binding
SRR122238_TR11341_c0_g1_i6_len=619	RL35_EUPES	Q:516-148,H:1-123	95.12%ID	E:1e-73	60S ribosomal protein L35	PF00831.20^Ribosomal_L29^Ribosomal L29 protein^8-64^E:4.1e-18	0	-0,956	0,845	-0,047	0,381	0,214	0,651	0,113	0,567	-0,134	RL35_EUPES	60S ribosomal protein L35	RPL35	Euphorbia esula (Leafy spurge)		Ribonucleoprotein; Ribosomal protein
SRR122238_TR12177_c2_g1_i2_len=711	H32_WHEAT	Q:625-218,H:1-136	98.53%ID	E:1e-76	Histone H3.2	PF00125.21^Histone^Core histone H2A/H2B/H3/H4^29-160^E:5.2e-54	0	-7,577	0,254	-4,845	0,502	-3,752	0,502	-3,752	1	-2,742	H32_WHEAT	Histone H3.2		Triticum aestivum (Wheat)		Acetylation; Chromosome; Complete proteome; DNA-binding; Direct protein sequencing; Methylation; Nucleosome core; Nucleus; Phosphoprotein; Reference proteome
SRR122238_TR12828_c0_g2_i9_len=806	RL182_ARATH	Q:726-166,H:1-187	87.17%ID	E:7e-116	60S ribosomal protein L18-2	PF17135.1^Ribosomal_L18^Ribosomal protein 60S L18 and 50S L18e^28-213^E:6.1e-96`PF00828.16^Ribosomal_L27A^Ribosomal proteins 50S-L15, 50S-L18e, 60S-L27A^73-146^E:0.00012	0	-1,074	0,463	-0,165	0,36	0,227	0,957	-0,017	0,631	-0,125	RL182_ARATH	60S ribosomal protein L18-2	RPL18B At3g05590 F18C1.14	Arabidopsis thaliana (Mouse-ear cress)		Complete proteome; Cytoplasm; Reference proteome; Ribonucleoprotein; Ribosomal protein
SRR122238_TR13302_c0_g1_i1_len=972	RL9_PEA	Q:865-302,H:3-190	87.77%ID	E:2e-115	60S ribosomal protein L9	PF00347.20^Ribosomal_L6^Ribosomal protein L6^12-89^E:5.8e-12`PF00347.20^Ribosomal_L6^Ribosomal protein L6^101-180^E:3.4e-12	0	-1,225	0,045	-0,529	0,376	0,293	0,481	0,229	0,35	-0,559	RL9_PEA	60S ribosomal protein L9 (Gibberellin-regulated protein GA)	RPL9	Pisum sativum (Garden pea)		Ribonucleoprotein; Ribosomal protein
SRR122238_TR13960_c0_g1_i1_len=621	H2B_GOSHI	Q:132-386,H:63-147	96.47%ID	E:6e-53	Histone H2B	.	0	-3,44	0,002	-6,312	0,254	4,614	1	3,444	0,152	-0,884	H2B_GOSHI	Histone H2B	HIS2B	Gossypium hirsutum (Upland cotton) (Gossypium mexicanum)		Acetylation; Chromosome; DNA-binding; Isopeptide bond; Nucleosome core; Nucleus; Ubl conjugation
SRR122238_TR15196_c4_g2_i2_len=690	RL40_NICSY	Q:591-208,H:1-128	99.22%ID	E:3e-88	Ubiquitin-60S ribosomal protein L40	PF11976.5^Rad60-SLD^Ubiquitin-2 like Rad60 SUMO-like^1-71^E:1.5e-16`PF00240.20^ubiquitin^Ubiquitin family^3-74^E:2.2e-34`PF14560.3^Ubiquitin_2^Ubiquitin-like domain^12-69^E:0.00011`PF13881.3^Rad60-SLD_2^Ubiquitin-2 like Rad60 SUMO-like^12-72^E:0.017`PF01020.14^Ribosomal_L40e^Ribosomal L40e family^78-127^E:1e-31	0	-1,015	0,275	-0,237	0,068	0,416	0,368	0,209	0,836	0,073	RL40_NICSY	Ubiquitin-60S ribosomal protein L40 [Cleaved into: Ubiquitin; 60S ribosomal protein L40 (CEP52)]	UBICEP52-7 RPL40	Nicotiana sylvestris (Wood tobacco) (South American tobacco)		Cytoplasm; Isopeptide bond; Nucleus; Ribonucleoprotein; Ribosomal protein; Ubl conjugation
SRR122238_TR19572_c1_g1_i2_len=1090	PNAE_RAUSE	Q:183-935,H:12-262	45.74%ID	E:1e-63	Polyneuridine-aldehyde esterase	PF12146.5^Hydrolase_4^Serine aminopeptidase, S33^10-119^E:6e-07`PF00561.17^Abhydrolase_1^alpha/beta hydrolase fold^14-123^E:3.2e-09`PF12697.4^Abhydrolase_6^Alpha/beta hydrolase family^15-255^E:1.4e-14	0,001	-0,865	0,079	-0,463	0,27	0,296	0,89	-0,039	0,78	0,078	PNAE_RAUSE	Polyneuridine-aldehyde esterase (EC 3.1.1.78) (Polyneuridine aldehyde esterase)	PNAE	Rauvolfia serpentina (Serpentine wood) (Ophioxylon serpentinum)	PATHWAY: Alkaloid biosynthesis; ajmaline biosynthesis.	3D-structure; Alkaloid metabolism; Direct protein sequencing; Hydrolase; Serine esterase
SRR122238_TR23347_c0_g1_i1_len=988	SR45A_ARATH	Q:763-461,H:68-168	68.32%ID	E:3e-28	Serine/arginine-rich splicing factor SR45a	PF00076.19^RRM_1^RNA recognition motif. (a.k.a. RRM, RBD, or RNP domain)^85-154^E:9e-18`PF13893.3^RRM_5^RNA recognition motif. (a.k.a. RRM, RBD, or RNP domain)^124-161^E:0.026	0	-7,131	1	0	0,502	4,357	0,009	6,036	1	-0,02	SR45A_ARATH	Serine/arginine-rich splicing factor SR45a (At-SR45A) (AtSR45a) (Protein TRANSFORMER2-like) (atTra2)	SR45A TRA2 At1g07350 F22G5.31	Arabidopsis thaliana (Mouse-ear cress)		Alternative splicing; Complete proteome; Nucleus; Reference proteome; Ribonucleoprotein; Spliceosome; mRNA processing; mRNA splicing
SRR122238_TR29021_c0_g1_i3_len=925	NACA2_ARATH	Q:675-202,H:60-217	78.48%ID	E:1e-50	Nascent polypeptide-associated complex subunit alpha-like protein 2	PF01849.15^NAC^NAC domain^97-152^E:1.9e-23	0	-0,91	0,008	-0,645	0,867	0,055	0,897	-0,049	0,787	-0,085	NACA2_ARATH	Nascent polypeptide-associated complex subunit alpha-like protein 2 (NAC-alpha-like protein 2) (Alpha-NAC-like protein 2)	At3g49470 T9C5.70	Arabidopsis thaliana (Mouse-ear cress)		Complete proteome; Protein transport; Reference proteome; Transport
SRR122238_TR29833_c0_g1_i1_len=2383	RUBA_PEA	Q:2175-478,H:25-587	87.63%ID	E:0	RuBisCO large subunit-binding protein subunit alpha, chloroplastic	PF00118.21^Cpn60_TCP1^TCP-1/cpn60 chaperonin family^71-574^E:4.8e-94	0	-1,018	0,176	-0,32	0,791	0,074	0,127	0,386	0,278	0,242	RUBA_PEA	RuBisCO large subunit-binding protein subunit alpha, chloroplastic (60 kDa chaperonin subunit alpha) (CPN-60 alpha)		Pisum sativum (Garden pea)		ATP-binding; Chaperone; Chloroplast; Direct protein sequencing; Nucleotide-binding; Plastid; Transit peptide
SRR122238_TR31182_c0_g1_i1_len=737	CH10_ARATH	Q:516-226,H:1-97	78.35%ID	E:7e-49	10 kDa chaperonin	.	0	-1,134	0,964	-0,027	0	2,643	0	1,849	0,692	-0,806	CH10_ARATH	10 kDa chaperonin (Chaperonin 10) (CPN10) (Protein groES)	CPN10 At1g14980 T15D22.2	Arabidopsis thaliana (Mouse-ear cress)		Chaperone; Complete proteome; Cytoplasm; Reference proteome
SRR122238_TR34938_c0_g1_i1_len=538	RS29_ARATH	Q:80-247,H:1-56	91.07%ID	E:6e-30	40S ribosomal protein S29	.	0	-3,751	0,008	-3,119	0,002	6,394	0,502	4,231	0,079	-0,542	RS29_ARATH	40S ribosomal protein S29	RPS29A At3g43980 T15B3.120; RPS29B At3g44010 T15B3.150; RPS29C At4g33865 F17I5.3	Arabidopsis thaliana (Mouse-ear cress)		Complete proteome; Metal-binding; Reference proteome; Ribonucleoprotein; Ribosomal protein; Zinc
SRR122238_TR36206_c0_g1_i3_len=718	RL211_ARATH	Q:595-104,H:1-164	81.1%ID	E:6e-99	60S ribosomal protein L21-1	PF01157.15^Ribosomal_L21e^Ribosomal protein L21e^5-98^E:1.6e-44	0	-1,074	0,491	-0,172	0,474	-0,196	0,155	-0,4	0,938	-0,03	RL211_ARATH	60S ribosomal protein L21-1	RPL21A At1g09590 F14J9.25; RPL21C At1g09690 F21M12.8	Arabidopsis thaliana (Mouse-ear cress)		Complete proteome; Reference proteome; Ribonucleoprotein; Ribosomal protein
SRR122238_TR38359_c0_g1_i3_len=1301	NA	NA	NA	NA	NA	.	0,001	-6,623	0,694	0,357	0,352	0,914	0,798	0,513	1	0	.	.	.	.	.	.
SRR122238_TR39862_c0_g1_i1_len=631	RL35_EUPES	Q:104-472,H:1-123	95.12%ID	E:5e-74	60S ribosomal protein L35	PF00831.20^Ribosomal_L29^Ribosomal L29 protein^8-64^E:7.8e-18	0,001	-0,886	0,047	-0,553	0,699	0,15	0,838	-0,097	0,093	-0,439	RL35_EUPES	60S ribosomal protein L35	RPL35	Euphorbia esula (Leafy spurge)		Ribonucleoprotein; Ribosomal protein
SRR122238_TR41920_c0_g1_i1_len=813	RS272_ARATH	Q:123-380,H:1-86	91.86%ID	E:3e-51	40S ribosomal protein S27-2	.	0	-1,058	0,9	0,039	0,066	0,55	0,76	0,096	0,191	-0,319	RS272_ARATH	40S ribosomal protein S27-2	RPS27B ARS27A At3g61110 T20K12.10 T27I15_200	Arabidopsis thaliana (Mouse-ear cress)		Complete proteome; Metal-binding; Reference proteome; Ribonucleoprotein; Ribosomal protein; Zinc; Zinc-finger
SRR122238_TR43463_c0_g1_i1_len=607	RL371_ARATH	Q:127-384,H:10-95	88.37%ID	E:4e-47	60S ribosomal protein L37-1	PF01907.16^Ribosomal_L37e^Ribosomal protein L37e^36-86^E:2.1e-25	0	-1,068	0,003	-0,647	0,149	0,349	1	0,007	0,105	-0,344	RL371_ARATH	60S ribosomal protein L37-1	RPL37A At1g15250 F9L1.46	Arabidopsis thaliana (Mouse-ear cress)		Complete proteome; Metal-binding; RNA-binding; Reference proteome; Ribonucleoprotein; Ribosomal protein; Zinc; Zinc-finger; rRNA-binding
SRR122238_TR44471_c0_g1_i6_len=928	R13A4_ARATH	Q:819-202,H:1-206	89.32%ID	E:9e-135	60S ribosomal protein L13a-4	PF00572.15^Ribosomal_L13^Ribosomal protein L13^13-118^E:1.2e-09	0	-0,947	0,435	-0,183	0,519	0,165	0,516	-0,176	0,793	-0,163	R13A4_ARATH	60S ribosomal protein L13a-4	RPL13AD At5g48760 K24G6.9	Arabidopsis thaliana (Mouse-ear cress)		Complete proteome; Reference proteome; Ribonucleoprotein; Ribosomal protein
SRR122238_TR45425_c2_g1_i9_len=800	RS12_HORVU	Q:586-215,H:20-143	82.26%ID	E:2e-69	40S ribosomal protein S12	PF01248.23^Ribosomal_L7Ae^Ribosomal protein L7Ae/L30e/S12e/Gadd45 family^27-120^E:4e-27	0,001	-6,576	0,009	6,043	1	0	1	0	1	-0,31	RS12_HORVU	40S ribosomal protein S12	RPS12	Hordeum vulgare (Barley)		Ribonucleoprotein; Ribosomal protein
SRR122238_TR48232_c1_g3_i5_len=1055	RS242_ARATH	Q:810-445,H:1-122	88.52%ID	E:4e-72	40S ribosomal protein S24-2	PF01282.16^Ribosomal_S24e^Ribosomal protein S24e^25-103^E:1.5e-36	0,001	-0,972	0,35	-0,288	0,283	0,441	1	0,024	0,721	-0,165	RS242_ARATH	40S ribosomal protein S24-2	RPS24B At5g28060 F15F15.130	Arabidopsis thaliana (Mouse-ear cress)		Complete proteome; Reference proteome; Ribonucleoprotein; Ribosomal protein
SRR122238_TR51732_c0_g1_i1_len=717	RL34_TOBAC	Q:622-338,H:1-95	96.84%ID	E:4e-59	60S ribosomal protein L34	PF01199.15^Ribosomal_L34e^Ribosomal protein L34e^32-127^E:2.1e-38	0	-1,07	0,188	-0,312	0,054	0,506	0,672	0,118	0,018	-0,624	RL34_TOBAC	60S ribosomal protein L34	RPL34	Nicotiana tabacum (Common tobacco)		Ribonucleoprotein; Ribosomal protein
SRR122238_TR57185_c0_g1_i2_len=866	RL18A_CASSA	Q:813-280,H:1-178	91.01%ID	E:5e-118	60S ribosomal protein L18a	PF01775.14^Ribosomal_L18A^Ribosomal proteins 50S-L18Ae/60S-L20/60S-L18A^24-145^E:2.4e-55	0	-0,895	0,418	-0,21	0,144	0,452	0,818	0,094	1	-0,115	RL18A_CASSA	60S ribosomal protein L18a	RPL18A	Castanea sativa (Sweet chestnut)		Ribonucleoprotein; Ribosomal protein
SRR122238_TR57280_c0_g1_i2_len=1089	RS27A_SOLTU	Q:85-552,H:1-156	96.79%ID	E:8e-85	Ubiquitin-40S ribosomal protein S27a	PF11976.5^Rad60-SLD^Ubiquitin-2 like Rad60 SUMO-like^29-99^E:3.7e-16`PF00240.20^ubiquitin^Ubiquitin family^31-102^E:5.4e-34`PF13881.3^Rad60-SLD_2^Ubiquitin-2 like Rad60 SUMO-like^38-118^E:0.0011`PF14560.3^Ubiquitin_2^Ubiquitin-like domain^39-97^E:0.00021`PF01599.16^Ribosomal_S27^Ribosomal protein S27a^130-175^E:3.8e-26	0	-1,062	1	-0,007	0,325	0,38	0,799	-0,098	0,098	-0,397	RS27A_SOLTU	Ubiquitin-40S ribosomal protein S27a [Cleaved into: Ubiquitin; 40S ribosomal protein S27a]	UBI3 RPS27A	Solanum tuberosum (Potato)		Complete proteome; Cytoplasm; Isopeptide bond; Metal-binding; Nucleus; Reference proteome; Ribonucleoprotein; Ribosomal protein; Ubl conjugation; Zinc; Zinc-finger
SRR122238_TR62934_c0_g1_i2_len=652	RPD6A_ARATH	Q:495-259,H:61-139	94.94%ID	E:1e-46	DNA-directed RNA polymerases II, IV and V subunit 6A	PF01192.19^RNA_pol_Rpb6^RNA polymerase Rpb6^53-104^E:6.3e-17	0	-2,234	0,014	-1,012	1	0	0	6,919	1	-1,817	RPD6A_ARATH	DNA-directed RNA polymerases II, IV and V subunit 6A (RNA polymerase Rpb6)	NRPB6A NRPD6A NRPE6A At5g51940 MSG15.2	Arabidopsis thaliana (Mouse-ear cress)		Complete proteome; DNA-directed RNA polymerase; Nucleus; Reference proteome; Transcription
SRR122238_TR63874_c0_g1_i6_len=712	RS13_SOYBN	Q:48-500,H:1-151	95.36%ID	E:1e-100	40S ribosomal protein S13	PF08069.9^Ribosomal_S13_N^Ribosomal S13/S15 N-terminal domain^16-75^E:3.2e-32`PF00312.19^Ribosomal_S15^Ribosomal protein S15^89-161^E:6.6e-16	0	-1,356	0,029	-0,601	0,942	0,044	0,821	-0,114	0,006	-0,742	RS13_SOYBN	40S ribosomal protein S13	RPS13	Glycine max (Soybean) (Glycine hispida)		Complete proteome; Reference proteome; Ribonucleoprotein; Ribosomal protein
SRR122238_TR66497_c1_g1_i1_len=1042	RL172_ARATH	Q:942-418,H:1-175	88%ID	E:2e-112	60S ribosomal protein L17-2	PF00237.16^Ribosomal_L22^Ribosomal protein L22p/L17e^19-151^E:2.9e-39	0	-0,889	0,385	-0,2	0,652	0,11	0,629	-0,127	0,641	-0,109	RL172_ARATH	60S ribosomal protein L17-2	RPL17B At1g67430 T1F15.11	Arabidopsis thaliana (Mouse-ear cress)		Alternative splicing; Complete proteome; Reference proteome; Ribonucleoprotein; Ribosomal protein
SRR122238_TR67156_c1_g1_i3_len=594	RS282_ARATH	Q:502-344,H:1-52	90.57%ID	E:6e-24	40S ribosomal protein S28-2	.	0	-1,141	0,227	-0,275	0,027	0,562	0,11	0,41	1	-0,19	RS282_ARATH	40S ribosomal protein S28-2	RPS28C At5g64140 MHJ24.12	Arabidopsis thaliana (Mouse-ear cress)		Complete proteome; Cytoplasm; Reference proteome; Ribonucleoprotein; Ribosomal protein
SRR122238_TR68648_c0_g1_i1_len=933	RL281_ARATH	Q:165-590,H:1-143	77.62%ID	E:1e-72	60S ribosomal protein L28-1	PF01778.14^Ribosomal_L28e^Ribosomal L28e protein family^8-127^E:1.7e-35	0	-0,908	0,335	-0,231	0,003	0,899	0,22	0,403	0,088	-0,409	RL281_ARATH	60S ribosomal protein L28-1	RPL28A At2g19730 F6F22.24	Arabidopsis thaliana (Mouse-ear cress)		Complete proteome; Reference proteome; Ribonucleoprotein; Ribosomal protein
SRR122238_TR71692_c0_g1_i3_len=505	NA	NA	NA	NA	NA	.	0	-3,372	1	-1,247	0,068	-1,007	0,1	-0,89	1	0	.	.	.	.	.	.
SRR122238_TR72925_c0_g1_i3_len=691	RS202_ARATH	Q:137-481,H:8-122	93.91%ID	E:1e-70	40S ribosomal protein S20-2	PF00338.19^Ribosomal_S10^Ribosomal protein S10p/S20e^25-119^E:3.7e-28	0	-0,989	0,913	0,027	0,38	0,214	0,806	-0,067	0,046	-0,448	RS202_ARATH	40S ribosomal protein S20-2	RPS20B At3g47370 T21L8.120	Arabidopsis thaliana (Mouse-ear cress)		Complete proteome; Reference proteome; Ribonucleoprotein; Ribosomal protein
SRR122238_TR73367_c0_g2_i2_len=732	NA	NA	NA	NA	NA	PF00428.16^Ribosomal_60s^60s Acidic ribosomal protein^18-112^E:2.8e-26`PF12868.4^DUF3824^Domain of unknwon function (DUF3824)^72-103^E:0.3	0	-1,623	0,02	-0,608	0,784	-0,106	1	0,026	0,08	-0,39	.	.	.	.	.	.
SRR122238_TR73926_c0_g1_i4_len=654	RL321_ARATH	Q:65-463,H:1-133	86.47%ID	E:5e-74	60S ribosomal protein L32-1	PF01655.15^Ribosomal_L32e^Ribosomal protein L32^37-143^E:4.3e-50	0	-0,906	0,305	-0,237	0,067	0,456	0,297	0,266	0,204	-0,278	RL321_ARATH	60S ribosomal protein L32-1	RPL32A At4g18100 F15J5.70	Arabidopsis thaliana (Mouse-ear cress)		Complete proteome; Reference proteome; Ribonucleoprotein; Ribosomal protein
SRR122238_TR77044_c0_g1_i1_len=761	RL222_ARATH	Q:203-502,H:17-116	85%ID	E:1e-44	60S ribosomal protein L22-2	PF01776.14^Ribosomal_L22e^Ribosomal L22e protein family^65-173^E:1.3e-45	0	-0,936	0,067	-0,433	0,851	0,049	0,821	-0,072	0,303	-0,242	RL222_ARATH	60S ribosomal protein L22-2	RPL22B At3g05560 F18C1.17	Arabidopsis thaliana (Mouse-ear cress)		Complete proteome; Reference proteome; Ribonucleoprotein; Ribosomal protein
SRR122238_TR77058_c1_g1_i6_len=612	F16P1_SPIOL	Q:368-84,H:51-147	65.98%ID	E:9e-32	Fructose-1,6-bisphosphatase, chloroplastic	PF00316.17^FBPase^Fructose-1-6-bisphosphatase, N-terminal domain^117-176^E:2.7e-12	0,001	-6,56	1	0	0,034	5,65	0	6,729	1	1,191	F16P1_SPIOL	Fructose-1,6-bisphosphatase, chloroplastic (FBPase) (EC 3.1.3.11) (D-fructose-1,6-bisphosphate 1-phosphohydrolase)		Spinacia oleracea (Spinach)	PATHWAY: Carbohydrate biosynthesis; Calvin cycle.	3D-structure; Calvin cycle; Carbohydrate metabolism; Chloroplast; Direct protein sequencing; Disulfide bond; Hydrolase; Magnesium; Metal-binding; Plastid; Transit peptide
SRR122238_TR79922_c0_g1_i2_len=662	RS252_ARATH	Q:430-215,H:37-108	83.33%ID	E:7e-36	40S ribosomal protein S25-2	PF03297.12^Ribosomal_S25^S25 ribosomal protein^7-104^E:3e-42	0	-0,935	0,479	-0,157	0,436	0,189	0,181	-0,321	0,376	-0,488	RS252_ARATH	40S ribosomal protein S25-2	RPS25B At2g21580 F2G1.15	Arabidopsis thaliana (Mouse-ear cress)		Alternative splicing; Complete proteome; Reference proteome; Ribonucleoprotein; Ribosomal protein
SRR122238_TR83537_c0_g1_i1_len=1015	RS11_SOYBN	Q:217-693,H:1-159	91.19%ID	E:1e-90	40S ribosomal protein S11	PF16205.2^Ribosomal_S17_N^Ribosomal_S17 N-terminal^4-72^E:1.9e-32`PF00366.17^Ribosomal_S17^Ribosomal protein S17^74-142^E:7.6e-25	0	-0,931	0,781	-0,071	0,351	0,254	0,557	-0,172	0,264	-0,267	RS11_SOYBN	40S ribosomal protein S11	RPS11	Glycine max (Soybean) (Glycine hispida)		Complete proteome; RNA-binding; Reference proteome; Ribonucleoprotein; Ribosomal protein; rRNA-binding
SRR122238_TR87209_c0_g2_i3_len=578	R35A1_ARATH	Q:549-214,H:1-112	91.07%ID	E:4e-70	60S ribosomal protein L35a-1	PF01247.15^Ribosomal_L35Ae^Ribosomal protein L35Ae^21-115^E:2.2e-46	0	-1,13	0,449	-0,187	0,88	0,055	0,154	-0,447	0,634	-0,237	R35A1_ARATH	60S ribosomal protein L35a-1	RPL35AA At1g07070 F10K1.22	Arabidopsis thaliana (Mouse-ear cress)		Complete proteome; Reference proteome; Ribonucleoprotein; Ribosomal protein
SRR122238_TR87209_c0_g2_i4_len=748	R35A3_ARATH	Q:700-365,H:1-112	91.96%ID	E:5e-69	60S ribosomal protein L35a-3	PF01247.15^Ribosomal_L35Ae^Ribosomal protein L35Ae^28-122^E:2.6e-46	0	-0,992	0,112	-0,394	0,023	0,662	0,425	0,248	0,066	-1,97	R35A3_ARATH	60S ribosomal protein L35a-3	RPL35AC At1g74270 F1O17.6	Arabidopsis thaliana (Mouse-ear cress)		Complete proteome; Reference proteome; Ribonucleoprotein; Ribosomal protein
SRR1271857_TR6930_c0_g3_i3_len=1519	NA	NA	NA	NA	NA	PF00428.16^Ribosomal_60s^60s Acidic ribosomal protein^22-113^E:7.1e-25	0,001	-0,866	0,363	-0,224	0,416	0,221	0,71	-0,102	1	0,087	.	.	.	.	.	.
SRR1271857_TR8539_c0_g1_i4_len=535	H4_SOYBN	Q:125-370,H:22-103	100%ID	E:8e-50	Histone H4	PF15511.3^CENP-T_C^Centromere kinetochore component CENP-T histone fold^35-96^E:1.4e-08`PF02969.14^TAF^TATA box binding protein associated factor (TAF)^35-92^E:0.00016	0	-2,392	0,158	-1,802	1	0,157	0,629	-1,294	0,004	-0,853	H4_SOYBN	Histone H4		Glycine max (Soybean) (Glycine hispida)		Acetylation; Chromosome; Complete proteome; DNA-binding; Methylation; Nucleosome core; Nucleus; Reference proteome
SRR1271857_TR8825_c0_g1_i1_len=1077	NA	NA	NA	NA	NA	.	0,001	-2,136	1	0,635	0,076	-2,891	0,076	-2,331	0,357	0,635	.	.	.	.	.	.
SRR1271857_TR19998_c0_g2_i1_len=1840	C82G1_ARATH	Q:224-1732,H:15-512	48.73%ID	E:4e-154	Cytochrome P450 82G1	PF00067.19^p450^Cytochrome P450^42-501^E:2.8e-87	0	-4,653	0,35	0,458	0,231	0,579	0,214	-0,913	0	1,514	C82G1_ARATH	Cytochrome P450 82G1 (EC 1.14.-.-)	CYP82G1 At3g25180 MJL12.5	Arabidopsis thaliana (Mouse-ear cress)	PATHWAY: Secondary metabolite biosynthesis; terpenoid biosynthesis.	Alternative splicing; Complete proteome; Heme; Iron; Membrane; Metal-binding; Monooxygenase; Oxidoreductase; Plant defense; Reference proteome; Transmembrane; Transmembrane helix
SRR1271857_TR23690_c0_g1_i1_len=1064	R13A2_ARATH	Q:126-743,H:1-206	89.81%ID	E:1e-133	60S ribosomal protein L13a-2	PF00572.15^Ribosomal_L13^Ribosomal protein L13^13-120^E:2.1e-09	0	-1,16	0,8	-0,088	0,642	0,205	0,608	-0,228	0,65	-0,13	R13A2_ARATH	60S ribosomal protein L13a-2	RPL13AB At3g24830 K7P8.12	Arabidopsis thaliana (Mouse-ear cress)		Complete proteome; Reference proteome; Ribonucleoprotein; Ribosomal protein
SRR1271857_TR25203_c1_g3_i1_len=638	NA	NA	NA	NA	NA	.	0	-1,799	0,257	0,759	0,402	1,141	0,402	1,098	0,484	0,409	.	.	.	.	.	.
SRR1271858_TR2790_c0_g1_i3_len=1651	RL5_CUCSA	Q:121-753,H:1-211	88.15%ID	E:7e-122	60S ribosomal protein L5	PF17144.1^Ribosomal_L5e^Ribosomal large subunit proteins 60S L5, and 50S L18^14-175^E:1.9e-83	0,001	-1,145	0,236	-0,404	0,277	0,516	0,528	0,328	1	-0,045	RL5_CUCSA	60S ribosomal protein L5	RPL5	Cucumis sativus (Cucumber)		RNA-binding; Ribonucleoprotein; Ribosomal protein; rRNA-binding
SRR1271858_TR7418_c0_g1_i1_len=2273	CBF5_ARATH	Q:2014-644,H:16-460	82.71%ID	E:0	H/ACA ribonucleoprotein complex subunit 4	PF08068.9^DKCLD^DKCLD (NUC011) domain^50-107^E:5.6e-31`PF01509.15^TruB_N^TruB family pseudouridylate synthase (N terminal domain)^111-227^E:2.1e-19`PF16198.2^TruB_C_2^tRNA pseudouridylate synthase B C-terminal domain^228-294^E:2.8e-22`PF01472.17^PUA^PUA domain^298-370^E:1.8e-21	0,001	-1,109	0,235	0,406	0,235	0,837	1	-0,263	0	3,461	CBF5_ARATH	H/ACA ribonucleoprotein complex subunit 4 (EC 5.4.99.-) (CBF5 homolog) (Dyskerin) (Nopp-140-associated protein of 57 kDa homolog) (AtNAP57) (Nucleolar protein NAP57 homolog)	CBF5 NAP57 At3g57150 F24I3.230	Arabidopsis thaliana (Mouse-ear cress)		Complete proteome; Isomerase; Nucleus; Phosphoprotein; RNA-binding; Reference proteome; Ribonucleoprotein; Ribosome biogenesis; rRNA processing
SRR1271858_TR7453_c0_g4_i2_len=1139	RL172_ARATH	Q:178-702,H:1-175	88.57%ID	E:4e-102	60S ribosomal protein L17-2	PF00237.16^Ribosomal_L22^Ribosomal protein L22p/L17e^19-151^E:2.4e-38	0,001	-0,992	0,144	-0,401	0,706	0,17	0,809	-0,121	0,369	-0,29	RL172_ARATH	60S ribosomal protein L17-2	RPL17B At1g67430 T1F15.11	Arabidopsis thaliana (Mouse-ear cress)		Alternative splicing; Complete proteome; Reference proteome; Ribonucleoprotein; Ribosomal protein
SRR1271858_TR10014_c0_g2_i1_len=1119	RL15_PETHY	Q:1000-389,H:1-204	94.61%ID	E:1e-116	60S ribosomal protein L15	PF00827.14^Ribosomal_L15e^Ribosomal L15^2-190^E:1.1e-94	0	-1,092	0,433	-0,204	1	0,012	0,584	-0,17	0,226	-0,511	RL15_PETHY	60S ribosomal protein L15	RPL15	Petunia hybrida (Petunia)		Ribonucleoprotein; Ribosomal protein
SRR1271858_TR32130_c0_g1_i1_len=748	CB12_SOLLC	Q:32-694,H:46-266	91.4%ID	E:1e-148	Chlorophyll a-b binding protein 7, chloroplastic	PF00504.18^Chloroa_b-bind^Chlorophyll A-B binding protein^39-205^E:6.8e-52	0	-6,679	0	7,321	0,001	-6,538	0,352	-1,041	1	0,048	CB12_SOLLC	Chlorophyll a-b binding protein 7, chloroplastic (LHCI type II CAB-7)	CAB7	Solanum lycopersicum (Tomato) (Lycopersicon esculentum)		Chlorophyll; Chloroplast; Chromophore; Complete proteome; Magnesium; Membrane; Metal-binding; Phosphoprotein; Photosynthesis; Photosystem I; Photosystem II; Plastid; Reference proteome; Thylakoid; Transit peptide; Transmembrane; Transmembrane helix
SRR1271858_TR61631_c0_g1_i1_len=541	RL393_ORYSJ	Q:384-232,H:1-51	96.08%ID	E:1e-26	60S ribosomal protein L39-3	.	0,001	-0,792	0,272	-0,255	0,819	0,067	0,765	-0,087	0,729	-0,362	RL393_ORYSJ	60S ribosomal protein L39-3	RPL39C Os06g0181566 LOC_Os06g08320 OsJ_20351 OSJNBa0035I03.41 OSJNBb0019L07.23	Oryza sativa subsp. japonica (Rice)		Complete proteome; Reference proteome; Ribonucleoprotein; Ribosomal protein
SRR1271858_TR62488_c1_g1_i1_len=773	RS23_FRAAN	Q:622-197,H:1-142	98.59%ID	E:2e-95	40S ribosomal protein S23	PF00164.22^Ribosom_S12_S23^Ribosomal protein S12/S23^9-141^E:6e-60	0	-0,893	0,344	-0,217	0,172	0,342	0,703	0,103	1	0,134	RS23_FRAAN	40S ribosomal protein S23 (S12)	RPS23	Fragaria ananassa (Strawberry)		Hydroxylation; Ribonucleoprotein; Ribosomal protein
SRR1271858_TR62948_c0_g1_i1_len=893	CYB5_TOBAC	Q:296-547,H:52-135	85.71%ID	E:3e-45	Cytochrome b5	.	0	-3,891	0,84	-0,264	1	-0,734	0,629	-2,1	0,232	-0,379	CYB5_TOBAC	Cytochrome b5		Nicotiana tabacum (Common tobacco)		Electron transport; Endoplasmic reticulum; Heme; Iron; Membrane; Metal-binding; Microsome; Transmembrane; Transmembrane helix; Transport
SRR1271859_TR5068_c0_g1_i1_len=1158	RS29_ARATH	Q:106-273,H:1-56	89.29%ID	E:8e-28	40S ribosomal protein S29	.	0,001	-0,794	0,088	-0,397	0,165	0,355	0,512	0,177	0,089	-0,396	RS29_ARATH	40S ribosomal protein S29	RPS29A At3g43980 T15B3.120; RPS29B At3g44010 T15B3.150; RPS29C At4g33865 F17I5.3	Arabidopsis thaliana (Mouse-ear cress)		Complete proteome; Metal-binding; Reference proteome; Ribonucleoprotein; Ribosomal protein; Zinc
SRR1271859_TR22448_c0_g1_i1_len=717	H2B_GOSHI	Q:232-540,H:45-147	96.12%ID	E:1e-58	Histone H2B	PF00125.21^Histone^Core histone H2A/H2B/H3/H4^9-124^E:9.9e-23`PF00808.20^CBFD_NFYB_HMF^Histone-like transcription factor (CBF/NF-Y) and archaeal histone^66-122^E:4.4e-05	0	-0,953	0,205	-0,355	0	1,025	0,003	0,883	1	-2,277	H2B_GOSHI	Histone H2B	HIS2B	Gossypium hirsutum (Upland cotton) (Gossypium mexicanum)		Acetylation; Chromosome; DNA-binding; Isopeptide bond; Nucleosome core; Nucleus; Ubl conjugation
SRR1271859_TR30332_c0_g4_i7_len=563	RT12_HELAN	Q:400-44,H:1-119	98.32%ID	E:1e-79	Ribosomal protein S12, mitochondrial	PF00164.22^Ribosom_S12_S23^Ribosomal protein S12/S23^5-121^E:2.9e-40	0	-3,723	1	-0,425	1	0,8	1	0,435	1	0,375	RT12_HELAN	Ribosomal protein S12, mitochondrial	RPS12	Helianthus annuus (Common sunflower)		Mitochondrion; RNA editing; Ribonucleoprotein; Ribosomal protein
SRR1271859_TR41669_c0_g2_i1_len=3519	SIR1_TOBAC	Q:167-2251,H:1-691	82.21%ID	E:0	Sulfite reductase 1 [ferredoxin], chloroplastic	PF03460.14^NIR_SIR_ferr^Nitrite/Sulfite reductase ferredoxin-like half domain^188-245^E:2e-15`PF01077.19^NIR_SIR^Nitrite and sulphite reductase 4Fe-4S domain^282-460^E:2.1e-50`PF03460.14^NIR_SIR_ferr^Nitrite/Sulfite reductase ferredoxin-like half domain^478-531^E:6.1e-13`PF01077.19^NIR_SIR^Nitrite and sulphite reductase 4Fe-4S domain^556-694^E:1.1e-09	0	-2,068	0,356	0,99	0,44	0,755	1	0,303	1	0	SIR1_TOBAC	Sulfite reductase 1 [ferredoxin], chloroplastic (NtSiR1) (EC 1.8.7.1)	SIR1	Nicotiana tabacum (Common tobacco)		4Fe-4S; Chloroplast; DNA-binding; Direct protein sequencing; Heme; Iron; Iron-sulfur; Metal-binding; Oxidoreductase; Plastid; Thioether bond; Transit peptide
SRR1271859_TR42066_c0_g1_i18_len=541	RL74_ARATH	Q:146-463,H:1-104	75.47%ID	E:1e-44	60S ribosomal protein L7-4	PF08079.9^Ribosomal_L30_N^Ribosomal L30 N-terminal domain^12-83^E:2.6e-26`PF00327.17^Ribosomal_L30^Ribosomal protein L30p/L7e^88-115^E:1.4e-09	0	-1,574	0,044	0,818	0,632	-0,202	0,495	-0,307	0,533	0,172	RL74_ARATH	60S ribosomal protein L7-4	RPL7D At3g13580 K20M4.2	Arabidopsis thaliana (Mouse-ear cress)		Complete proteome; Reference proteome; Ribonucleoprotein; Ribosomal protein
SRR342017_TR1435_c0_g3_i1_len=540	RL142_ARATH	Q:407-15,H:1-131	85.5%ID	E:3e-74	60S ribosomal protein L14-2	PF01929.14^Ribosomal_L14e^Ribosomal protein L14^45-117^E:2e-26	0	-1,082	0,478	-0,162	0,422	0,21	0,91	0,037	0,086	-0,382	RL142_ARATH	60S ribosomal protein L14-2	RPL14B At4g27090 T24A18.40	Arabidopsis thaliana (Mouse-ear cress)		Complete proteome; Reference proteome; Ribonucleoprotein; Ribosomal protein
SRR342017_TR2746_c0_g1_i2_len=3059	SUBL_ARATH	Q:2585-378,H:31-757	58.63%ID	E:0	.	PF05922.13^Inhibitor_I9^Peptidase inhibitor I9^29-107^E:1.4e-17`PF00082.19^Peptidase_S8^Subtilase family^137-588^E:1.3e-49`PF02225.19^PA^PA domain^403-463^E:2.4e-09	0	-1,437	0,357	0,649	0	1,743	0,739	0,338	1	0,022	SBT17_ARATH	Subtilisin-like protease SBT1.7 (EC 3.4.21.-) (Cucumisin-like serine protease) (Subtilase subfamily 1 member 7) (AtSBT1.7) (Subtilisin-like serine protease 1) (At-SLP1)	SBT1.7 ARA12 ASP48 SLP1 At5g67360 K8K14.8	Arabidopsis thaliana (Mouse-ear cress)		Calcium; Cell wall; Complete proteome; Direct protein sequencing; Glycoprotein; Hydrolase; Protease; Reference proteome; Secreted; Serine protease; Signal; Zymogen
SRR342017_TR6063_c3_g9_i1_len=1379	PCNA_CATRO	Q:1199-417,H:1-261	99.23%ID	E:0	Proliferating cell nuclear antigen	PF00705.15^PCNA_N^Proliferating cell nuclear antigen, N-terminal domain^1-125^E:2.8e-58`PF04139.10^Rad9^Rad9^13-216^E:1.4e-06`PF02747.12^PCNA_C^Proliferating cell nuclear antigen, C-terminal domain^127-254^E:2.4e-61	0	-1,66	0,112	-1,179	0,402	0,976	1	-0,254	0,175	-1,092	PCNA_CATRO	Proliferating cell nuclear antigen (PCNA) (Cyclin)		Catharanthus roseus (Madagascar periwinkle) (Vinca rosea)		DNA replication; DNA-binding; Nucleus
SRR342017_TR10902_c2_g1_i1_len=935	RL23_ARATH	Q:854-435,H:1-140	97.14%ID	E:1e-92	60S ribosomal protein L23	PF00238.16^Ribosomal_L14^Ribosomal protein L14p/L23e^22-140^E:8.9e-36	0	-1,106	0,382	-0,23	0,012	0,811	0,606	0,205	0,591	-0,368	RL23_ARATH	60S ribosomal protein L23 (Protein EMBRYO DEFECTIVE 2171)	RPL23A At1g04480 F19P19.5; RPL23B At2g33370 F4P9.14; RPL23C EMB2171 At3g04400 T27C4.4	Arabidopsis thaliana (Mouse-ear cress)		Alternative splicing; Complete proteome; Reference proteome; Ribonucleoprotein; Ribosomal protein
SRR342017_TR11395_c0_g1_i2_len=996	DUT_SOLLC	Q:767-261,H:4-169	80.59%ID	E:5e-90	Deoxyuridine 5'-triphosphate nucleotidohydrolase	PF00692.16^dUTPase^dUTPase^46-174^E:3.4e-45	0	-1,477	0,026	-2,017	1	0,521	1	0,043	0,111	-1,373	DUT_SOLLC	Deoxyuridine 5'-triphosphate nucleotidohydrolase (dUTPase) (EC 3.6.1.23) (P18) (dUTP pyrophosphatase)		Solanum lycopersicum (Tomato) (Lycopersicon esculentum)	PATHWAY: Pyrimidine metabolism; dUMP biosynthesis; dUMP from dCTP (dUTP route): step 2/2.	Complete proteome; Hydrolase; Magnesium; Metal-binding; Nucleotide metabolism; Reference proteome
SRR342017_TR15390_c0_g1_i1_len=1147	ERD2_PETHY	Q:380-1024,H:1-215	92.09%ID	E:5e-133	ER lumen protein-retaining receptor	PF00810.15^ER_lumen_recept^ER lumen protein retaining receptor^28-171^E:2.2e-51	0	-3,791	0,009	1,232	0,502	1,883	0,034	3,438	0,692	0,898	ERD2_PETHY	ER lumen protein-retaining receptor (HDEL receptor) (PGP169-12)	ERD2	Petunia hybrida (Petunia)		ER-Golgi transport; Endoplasmic reticulum; Membrane; Protein transport; Receptor; Transmembrane; Transmembrane helix; Transport
SRR342017_TR16448_c0_g1_i4_len=792	H2B1_MEDTR	Q:462-187,H:57-148	98.91%ID	E:1e-57	Probable histone H2B.1	PF00125.21^Histone^Core histone H2A/H2B/H3/H4^5-128^E:1.2e-22`PF00808.20^CBFD_NFYB_HMF^Histone-like transcription factor (CBF/NF-Y) and archaeal histone^70-126^E:0.00028	0	-1,629	0,009	-0,952	0,803	0,164	0,484	-0,415	0,608	-0,242	H2B1_MEDTR	Probable histone H2B.1		Medicago truncatula (Barrel medic) (Medicago tribuloides)		Acetylation; Chromosome; DNA-binding; Isopeptide bond; Nucleosome core; Nucleus; Ubl conjugation
SRR342017_TR17543_c3_g2_i1_len=1231	RS4_SOLTU	Q:154-942,H:1-264	92.05%ID	E:3e-164	40S ribosomal protein S4	PF08071.9^RS4NT^RS4NT (NUC023) domain^54-90^E:1.6e-19`PF01479.22^S4^S4 domain^98-141^E:4.1e-05`PF00900.17^Ribosomal_S4e^Ribosomal family S4e^146-220^E:1.5e-36`PF00467.26^KOW^KOW motif^228-261^E:1.6e-06`PF16121.2^40S_S4_C^40S ribosomal protein S4 C-terminus^263-308^E:6.4e-26	0	-0,873	0,371	0,214	1	-0,004	0,425	-0,21	0,212	-0,293	RS4_SOLTU	40S ribosomal protein S4	RPS4	Solanum tuberosum (Potato)		Complete proteome; Cytoplasm; RNA-binding; Reference proteome; Ribonucleoprotein; Ribosomal protein; rRNA-binding
SRR342017_TR21012_c0_g2_i1_len=1770	HDT1_SOLCH	Q:308-1204,H:1-258	57.86%ID	E:1e-79	Histone deacetylase HDT1	PF13912.3^zf-C2H2_6^C2H2-type zinc finger^283-306^E:3.6e-06`PF12874.4^zf-met^Zinc-finger of C2H2 type^284-304^E:0.034`PF13913.3^zf-C2HC_2^zinc-finger of a C2HC-type^284-303^E:0.21	0	-1,11	0,465	0,186	0,062	0,699	1	-0,039	0,44	-0,205	HDT1_SOLCH	Histone deacetylase HDT1 (Histone deacetylase 2a) (HD2a) (ScHD2a)	HDT1 HD2A	Solanum chacoense (Chaco potato)		Chromatin regulator; Hydrolase; Metal-binding; Nucleus; Phosphoprotein; Repressor; Transcription; Transcription regulation; Zinc; Zinc-finger
SRR342017_TR22912_c0_g4_i1_len=806	MRT4_MOUSE	Q:171-806,H:1-211	50.94%ID	E:2e-66	mRNA turnover protein 4 homolog {ECO:0000250|UniProtKB:P33201}	PF00466.17^Ribosomal_L10^Ribosomal protein L10^19-119^E:1.2e-20	0,001	-1,21	0,014	0,894	1	-0,047	0,591	0,334	0,535	0,223	MRT4_MOUSE	mRNA turnover protein 4 homolog (Ribosome assembly factor Mrto4)	Mrto4 Mrt4	Mus musculus (Mouse)		Complete proteome; Cytoplasm; Nucleus; Phosphoprotein; Reference proteome; Ribosome biogenesis
SRR342017_TR24661_c4_g7_i1_len=869	Y1848_ARATH	Q:688-2,H:352-581	80.43%ID	E:2e-130	Probable inactive receptor kinase At1g48480	PF00069.22^Pkinase^Protein kinase domain^1-149^E:4.6e-29`PF07714.14^Pkinase_Tyr^Protein tyrosine kinase^2-154^E:2.8e-26	0	-7,25	0,066	5,266	1	0	1	0	0,618	-0,261	Y1848_ARATH	Probable inactive receptor kinase At1g48480	RKL1 At1g48480 T1N15.9	Arabidopsis thaliana (Mouse-ear cress)		ATP-binding; Cell membrane; Complete proteome; Leucine-rich repeat; Membrane; Nucleotide-binding; Phosphoprotein; Receptor; Reference proteome; Repeat; Signal; Transmembrane; Transmembrane helix
SRR342019_TR3673_c0_g1_i5_len=3444	OBGC_ARATH	Q:650-1861,H:200-614	76.39%ID	E:0	GTP-binding protein OBGC, chloroplastic	PF01018.19^GTP1_OBG^GTP1/OBG^190-349^E:8.6e-53`PF01926.20^MMR_HSR1^50S ribosome-binding GTPase^352-472^E:8.9e-22`PF02421.15^FeoB_N^Ferrous iron transport protein B^353-512^E:3e-09	0	-2,642	0,086	-0,978	1	0,154	1	0,108	0,705	0,233	OBGC_ARATH	GTP-binding protein OBGC, chloroplastic (AtOBGC) (GTP-binding protein OBG-like) (AtOBGL) (Protein CHLOROPLASTIC SAR1) (CPSAR1) (Protein EMBRYO DEFECTIVE 269) (Protein EMBRYO DEFECTIVE 3138)	OBGL EMB269 EMB3138 At5g18570 T28N17.50	Arabidopsis thaliana (Mouse-ear cress)		Chloroplast; Complete proteome; GTP-binding; Magnesium; Membrane; Metal-binding; Nucleotide-binding; Plastid; Plastid inner membrane; Reference proteome; Ribosome biogenesis; Transit peptide
SRR342019_TR4149_c0_g1_i6_len=844	RS191_ARATH	Q:706-278,H:1-143	86.71%ID	E:2e-87	40S ribosomal protein S19-1	PF01090.16^Ribosomal_S19e^Ribosomal protein S19e^6-140^E:7.4e-59	0	-1,054	0,116	-0,367	0,125	0,41	0,639	-0,146	0,005	-0,672	RS191_ARATH	40S ribosomal protein S19-1	RPS19A At3g02080 F1C9.13	Arabidopsis thaliana (Mouse-ear cress)		Complete proteome; Reference proteome; Ribonucleoprotein; Ribosomal protein
SRR342019_TR7160_c2_g1_i7_len=2471	RS22_ARATH	Q:1036-341,H:48-279	87.5%ID	E:5e-141	40S ribosomal protein S2-2	PF00333.17^Ribosomal_S5^Ribosomal protein S5, N-terminal domain^86-150^E:3.4e-32`PF03719.12^Ribosomal_S5_C^Ribosomal protein S5, C-terminal domain^169-236^E:2.5e-25	0	-1,067	0,641	0,137	0,948	0,037	0,629	-0,202	1	-0,025	RS22_ARATH	40S ribosomal protein S2-2	RPS2B RF12 At1g59359 T4M14_3; At1g58983 T4M14.1; At1g58684 R18I.8	Arabidopsis thaliana (Mouse-ear cress)		Complete proteome; Reference proteome; Ribonucleoprotein; Ribosomal protein
SRR342019_TR8450_c0_g2_i5_len=999	NA	NA	NA	NA	NA	.	0	-1,396	0,144	-0,474	1	0,032	0,614	-0,218	1	-0,643	.	.	.	.	.	.
SRR342019_TR8520_c0_g2_i1_len=661	RL30_LUPLU	Q:641-318,H:5-112	91.67%ID	E:1e-66	60S ribosomal protein L30	PF01248.23^Ribosomal_L7Ae^Ribosomal protein L7Ae/L30e/S12e/Gadd45 family^15-107^E:2.6e-24	0,001	-0,858	0,573	-0,148	0,188	0,429	0,678	-0,157	0,109	-0,42	RL30_LUPLU	60S ribosomal protein L30	RPL30	Lupinus luteus (European yellow lupin)		Ribonucleoprotein; Ribosomal protein
SRR342019_TR10904_c1_g3_i1_len=1010	H2A1_ARATH	Q:844-449,H:1-132	93.94%ID	E:3e-53	Probable histone H2A.1	.	0,001	-0,803	0,006	-0,754	0,021	0,691	0,134	0,465	1	0,088	H2A1_ARATH	Probable histone H2A.1 (HTA10)	At1g51060 F23H24.9	Arabidopsis thaliana (Mouse-ear cress)		Chromosome; Complete proteome; DNA-binding; Nucleosome core; Nucleus; Reference proteome
SRR342019_TR11438_c0_g1_i1_len=677	NA	NA	NA	NA	NA	.	0	-7,784	0,526	0,227	1	0	1	1,436	1	0	.	.	.	.	.	.
SRR342019_TR11655_c1_g1_i1_len=1431	VAP22_ARATH	Q:1159-563,H:1-197	49.25%ID	E:2e-53	Vesicle-associated protein 2-2	PF00635.23^Motile_Sperm^MSP (Major sperm protein) domain^15-109^E:2.4e-29	0	-7,381	0,832	0,269	1	0	1	0	1	0	VAP22_ARATH	Vesicle-associated protein 2-2 (Plant VAP homolog 22) (AtPVA22) (VAMP-associated protein 2-2) (Vesicle-associated protein 27-2) [Cleaved into: Vesicle-associated protein 2-2, N-terminally processed]	PVA22 VAP27-2 At1g08820 F22O13.31	Arabidopsis thaliana (Mouse-ear cress)		Acetylation; Coiled coil; Complete proteome; Endoplasmic reticulum; Host-virus interaction; Membrane; Phosphoprotein; Reference proteome; Transmembrane; Transmembrane helix
SRR342019_TR18912_c0_g1_i1_len=952	RS141_MAIZE	Q:817-401,H:1-138	94.24%ID	E:2e-77	40S ribosomal protein S14	PF00411.16^Ribosomal_S11^Ribosomal protein S11^28-146^E:9.7e-48	0	-1,11	0,147	-0,465	0,183	0,663	0,123	0,762	0,581	-0,51	RS141_MAIZE	40S ribosomal protein S14 (Clone MCH1)		Zea mays (Maize)		Complete proteome; Reference proteome; Ribonucleoprotein; Ribosomal protein

SRR342019_TR19026_c1_g1_i1_len=1517	RL32_ARATH	Q:1395-238,H:1-386	88.6%ID	E:0	60S ribosomal protein L3-2	PF00297.19^Ribosomal_L3^Ribosomal protein L3^1-370^E:2.4e-194	0,001	-1,17	0,755	-0,148	0,317	-0,499	0,153	-0,711	1	0,019	RL32_ARATH	60S ribosomal protein L3-2	ARP2 RPL3B At1g61580 T25B24.7	Arabidopsis thaliana (Mouse-ear cress)		Complete proteome; Cytoplasm; Reference proteome; Ribonucleoprotein; Ribosomal protein
SRR342019_TR21392_c3_g2_i2_len=1130	RL7A_ORYSJ	Q:1052-276,H:1-258	87.26%ID	E:3e-150	60S ribosomal protein L7a	PF01248.23^Ribosomal_L7Ae^Ribosomal protein L7Ae/L30e/S12e/Gadd45 family^117-207^E:7.1e-25	0	-1	0,298	-0,284	0,203	0,442	0,807	0,104	0,264	-0,333	RL7A_ORYSJ	60S ribosomal protein L7a	RPL7A Os08g0326400 LOC_Os08g23710 OJ1136_A10.108 P0703C03.43	Oryza sativa subsp. japonica (Rice)		Complete proteome; Reference proteome; Ribonucleoprotein; Ribosomal protein
SRR342019_TR21392_c3_g2_i4_len=1327	RL7A_ORYSJ	Q:1217-672,H:1-181	84.62%ID	E:2e-112	60S ribosomal protein L7a	PF01248.23^Ribosomal_L7Ae^Ribosomal protein L7Ae/L30e/S12e/Gadd45 family^117-187^E:4.9e-21	0	-1,838	0,099	-0,808	0,461	1,4	1	-0,173	1	-0,711	RL7A_ORYSJ	60S ribosomal protein L7a	RPL7A Os08g0326400 LOC_Os08g23710 OJ1136_A10.108 P0703C03.43	Oryza sativa subsp. japonica (Rice)		Complete proteome; Reference proteome; Ribonucleoprotein; Ribosomal protein
SRR342019_TR22118_c4_g6_i2_len=1582	RS15_ELAOL	Q:563-318,H:72-153	86.59%ID	E:8e-44	40S ribosomal protein S15	PF00203.18^Ribosomal_S19^Ribosomal protein S19^50-115^E:2.6e-30	0	-0,945	0,739	-0,081	0,143	0,376	0,793	0,069	1	-0,058	RS15_ELAOL	40S ribosomal protein S15	RPS15	Elaeis oleifera (American oil palm) (Corozo oleifera)		Ribonucleoprotein; Ribosomal protein
SRR342019_TR22658_c1_g1_i1_len=947	NA	NA	NA	NA	NA	PF04774.12^HABP4_PAI-RBP1^Hyaluronan / mRNA binding family^4-46^E:1.7e-07	0	-0,899	0,533	-0,151	0,769	0,084	0,353	-0,262	0,415	-0,253	.	.	.	.	.	.
SRR342019_TR23706_c2_g7_i18_len=2199	RS3A_CATRO	Q:972-274,H:28-261	99.15%ID	E:2e-152	40S ribosomal protein S3a {ECO:0000255|HAMAP-Rule:MF_03122}	PF01015.15^Ribosomal_S3Ae^Ribosomal S3Ae family^15-221^E:2.1e-99	0	-1,133	0,863	-0,053	0,758	0,107	0,458	-0,253	1	-0,05	RS3A_CATRO	40S ribosomal protein S3a (CYC07 protein)	RPS3A CYC07	Catharanthus roseus (Madagascar periwinkle) (Vinca rosea)		Cytoplasm; Ribonucleoprotein; Ribosomal protein
SRR342019_TR28947_c0_g1_i1_len=798	EF1B1_ARATH	Q:698-150,H:1-184	63.98%ID	E:5e-48	Elongation factor 1-beta 1	PF00736.16^EF1_GNE^EF-1 guanine nucleotide exchange domain^172-218^E:2.2e-11	0	-1,233	0,378	0,353	0,35	0,383	0,188	0,493	0,694	0,354	EF1B1_ARATH	Elongation factor 1-beta 1 (EF-1-beta 1) (Elongation factor 1-beta' 1) (EF-1-beta' 1) (Elongation factor 1B-alpha 1) (eEF-1B alpha 1)	At5g12110 MXC9.7	Arabidopsis thaliana (Mouse-ear cress)		Acetylation; Cell membrane; Complete proteome; Elongation factor; Membrane; Protein biosynthesis; Reference proteome
SRR342019_TR34577_c2_g2_i1_len=634	RL132_BRANA	Q:108-626,H:1-173	86.13%ID	E:4e-110	60S ribosomal protein L13-2	PF01294.15^Ribosomal_L13e^Ribosomal protein L13e^7-175^E:7.3e-76	0	-1,203	0,654	0,122	0,575	0,187	0,736	-0,144	1	-0,008	RL132_BRANA	60S ribosomal protein L13-2 (Cold-induced protein C24B)		Brassica napus (Rape)		Ribonucleoprotein; Ribosomal protein
SRR342019_TR37006_c3_g3_i1_len=754	TBA4_ARATH	Q:510-1,H:1-170	98.82%ID	E:7e-104	Tubulin alpha-4 chain	PF00091.22^Tubulin^Tubulin/FtsZ family, GTPase domain^3-168^E:3.8e-52	0	-1,042	0	4,269	0,044	-0,759	0,206	-0,465	0,026	0,602	TBA4_ARATH	Tubulin alpha-4 chain	TUBA4 TUA4 At1g04820 F13M7.19	Arabidopsis thaliana (Mouse-ear cress)		Acetylation; Complete proteome; Cytoplasm; Cytoskeleton; GTP-binding; Microtubule; Nucleotide-binding; Phosphoprotein; Reference proteome
SRR342022_TR10275_c3_g3_i4_len=3102	NUCL2_ORYSJ	Q:1953-2543,H:435-630	56.28%ID	E:7e-62	Nucleolin 2	PF00076.19^RRM_1^RNA recognition motif. (a.k.a. RRM, RBD, or RNP domain)^412-482^E:3.8e-19`PF00076.19^RRM_1^RNA recognition motif. (a.k.a. RRM, RBD, or RNP domain)^515-585^E:9.9e-08	0,001	-1,074	0	1,678	0,381	2,056	1	1,035	0,619	0,134	NUCL2_ORYSJ	Nucleolin 2 (Protein NUCLEOLIN LIKE 2)	Os04g0620700 LOC_Os04g52960 OSJNBa0058K23.21	Oryza sativa subsp. japonica (Rice)		Complete proteome; Nucleus; RNA-binding; Reference proteome; Repeat; rRNA processing
SRR342022_TR11933_c0_g2_i1_len=521	RPBAL_ARATH	Q:120-326,H:1-69	91.3%ID	E:1e-40	DNA-directed RNA polymerase subunit 10-like protein {ECO:0000303|PubMed:19110459}	.	0	-1,465	1	0,02	0	1,34	0,003	1,032	0,559	-0,174	RPBAL_ARATH	DNA-directed RNA polymerase subunit 10-like protein (ABC10) (DNA-directed RNA polymerase III subunit L) (DNA-directed RNA polymerases I, II, and III subunit RPABC5) (RNA polymerases I, II, and III subunit ABC5) (RPB10 homolog)	NRPB10L At1g61700 T13M11.6	Arabidopsis thaliana (Mouse-ear cress)		Complete proteome; DNA-directed RNA polymerase; Metal-binding; Nucleus; Reference proteome; Transcription; Zinc
SRR342022_TR15406_c3_g5_i2_len=1692	RIR2_TOBAC	Q:131-1117,H:1-329	90.27%ID	E:0	Ribonucleoside-diphosphate reductase small chain	PF00268.18^Ribonuc_red_sm^Ribonucleotide reductase, small chain^59-325^E:3e-119	0,001	-1,25	0,014	-1,479	0,402	0,913	0,76	0,357	0,107	-0,68	RIR2_TOBAC	Ribonucleoside-diphosphate reductase small chain (EC 1.17.4.1) (Ribonucleoside-diphosphate reductase R2 subunit) (Ribonucleotide reductase small subunit)		Nicotiana tabacum (Common tobacco)	PATHWAY: Genetic information processing; DNA replication.	Cytoplasm; DNA replication; Iron; Metal-binding; Oxidoreductase
SRR342022_TR17193_c0_g3_i3_len=607	RL291_ARATH	Q:95-277,H:1-61	81.97%ID	E:8e-27	60S ribosomal protein L29-1	.	0	-0,77	0,656	0,115	0	1,244	0	0,85	0,019	-0,604	RL291_ARATH	60S ribosomal protein L29-1	RPL29A At3g06700 F3E22.16 T8E24.8	Arabidopsis thaliana (Mouse-ear cress)		Complete proteome; Phosphoprotein; Reference proteome; Ribonucleoprotein; Ribosomal protein
SRR342022_TR19763_c2_g2_i7_len=1802	PSBP_SPIOL	Q:2-805,H:5-267	74.91%ID	E:1e-135	Oxygen-evolving enhancer protein 2, chloroplastic	PF01789.13^PsbP^PsbP^78-267^E:6.2e-41	0	-1,056	0,215	-0,351	0,823	-0,112	0,262	-0,429	0,372	-0,247	PSBP_SPIOL	Oxygen-evolving enhancer protein 2, chloroplastic (OEE2) (23 kDa subunit of oxygen evolving system of photosystem II) (23 kDa thylakoid membrane protein) (OEC 23 kDa subunit)	PSBP	Spinacia oleracea (Spinach)		3D-structure; Chloroplast; Direct protein sequencing; Membrane; Photosynthesis; Photosystem II; Plastid; Thylakoid; Transit peptide
SRR342022_TR19776_c0_g2_i2_len=1806	EAAC_ARATH	Q:159-1310,H:1-379	71.36%ID	E:5e-173	Probable envelope ADP,ATP carrier protein, chloroplastic	PF00153.24^Mito_carr^Mitochondrial carrier protein^95-185^E:7.5e-24`PF00153.24^Mito_carr^Mitochondrial carrier protein^189-276^E:6.4e-21`PF00153.24^Mito_carr^Mitochondrial carrier protein^284-366^E:2.7e-20	0	-1,814	0,004	-1,724	1	0,898	1	-2,46	0,191	-0,402	EAAC_ARATH	Probable envelope ADP,ATP carrier protein, chloroplastic (Envelope ADP/ATP translocase)	EAAC At3g51870 ATEM1.12	Arabidopsis thaliana (Mouse-ear cress)		Chloroplast; Complete proteome; Membrane; Plastid; Reference proteome; Repeat; Transit peptide; Transmembrane; Transmembrane helix; Transport
SRR342022_TR26073_c3_g1_i13_len=567	NA	NA	NA	NA	NA	PF00226.28^DnaJ^DnaJ domain^12-77^E:1e-24	0	-1,521	0	1,794	1	-1,477	1	-1,477	1	1,187	.	.	.	.	.	.
SRR342023_TR5478_c1_g1_i2_len=2488	RFA1B_ARATH	Q:2278-458,H:1-588	67.54%ID	E:0	Replication protein A 70 kDa DNA-binding subunit B	PF04057.9^Rep-A_N^Replication factor-A protein 1, N-terminal domain^6-103^E:5e-22`PF01336.22^tRNA_anti-codon^OB-fold nucleic acid binding domain^191-277^E:1.2e-10`PF16900.2^REPA_OB_2^Replication protein A OB domain^311-405^E:1e-22`PF08646.7^Rep_fac-A_C^Replication factor-A C terminal domain^468-612^E:6e-45	0,001	-1,433	0,025	-3,275	1	0,323	1	-0,665	0,203	-0,58	RFA1B_ARATH	Replication protein A 70 kDa DNA-binding subunit B (AtRPA70B) (AtRPA1-5) (Replication factor A protein 1B) (Replication protein A 1B) (AtRPA1B)	RPA1B RPA70B At5g08020 F13G24.220	Arabidopsis thaliana (Mouse-ear cress)		Complete proteome; DNA damage; DNA recombination; DNA repair; DNA replication; DNA-binding; Metal-binding; Nucleus; Reference proteome; Zinc; Zinc-finger
SRR342023_TR8037_c0_g1_i1_len=557	NHP2_ARATH	Q:378-4,H:1-124	76%ID	E:9e-56	H/ACA ribonucleoprotein complex subunit 2-like protein	PF01248.23^Ribosomal_L7Ae^Ribosomal protein L7Ae/L30e/S12e/Gadd45 family^41-122^E:2.6e-23	0	-1,067	0,24	0,349	0,132	0,746	1	-0,097	1	0,011	NHP2_ARATH	H/ACA ribonucleoprotein complex subunit 2-like protein (Nhp2-like protein)	At5g08180 T22D6.120	Arabidopsis thaliana (Mouse-ear cress)		Complete proteome; Nucleus; RNA-binding; Reference proteome; Ribonucleoprotein; Ribosome biogenesis; rRNA processing
SRR342023_TR10865_c0_g3_i3_len=1344	FRI2_TOBAC	Q:1228-470,H:1-250	78.74%ID	E:2e-117	Ferritin-2, chloroplastic	PF00210.21^Ferritin^Ferritin-like domain^98-241^E:3.8e-34	0	-0,853	0,216	-0,297	0,359	0,281	1	0,022	0,035	-1,745	FRI2_TOBAC	Ferritin-2, chloroplastic (EC 1.16.3.1) (NtFer2)	FER2	Nicotiana tabacum (Common tobacco)		Chloroplast; Iron; Iron storage; Metal-binding; Oxidoreductase; Plastid; Transit peptide
SRR342023_TR13025_c0_g5_i2_len=1063	RS18_ARATH	Q:148-603,H:1-152	92.76%ID	E:7e-88	40S ribosomal protein S18	PF00416.19^Ribosomal_S13^Ribosomal protein S13/S18^14-142^E:3.8e-57	0	-1,002	0,804	0,057	0,335	0,23	0,755	-0,075	1	0,829	RS18_ARATH	40S ribosomal protein S18	RPS18A PFL At1g22780 T22J18.5; RPS18B At1g34030 F12G12.15 T15K4.9; RPS18C At4g09800 F17A8.150	Arabidopsis thaliana (Mouse-ear cress)		Acetylation; Complete proteome; Cytoplasm; Direct protein sequencing; RNA-binding; Reference proteome; Ribonucleoprotein; Ribosomal protein; rRNA-binding
SRR342023_TR16651_c0_g1_i1_len=1317	CYT5_ARATH	Q:634-359,H:22-114	59.14%ID	E:2e-31	Cysteine proteinase inhibitor 5	PF00031.18^Cystatin^Cystatin domain^34-119^E:4.4e-15`PF16845.2^SQAPI^Aspartic acid proteinase inhibitor^39-119^E:7.2e-37`PF07430.8^PP1^Phloem filament protein PP1 cystatin-like domain^47-115^E:0.00013	0	-1,158	0,26	-0,355	0,065	0,709	0,555	0,271	0,192	0,442	CYT5_ARATH	Cysteine proteinase inhibitor 5 (AtCYS-5)	CYS5 At5g47550 MNJ7.14	Arabidopsis thaliana (Mouse-ear cress)		Complete proteome; Glycoprotein; Plant defense; Protease inhibitor; Reference proteome; Secreted; Signal; Thiol protease inhibitor
SRR342023_TR17380_c0_g1_i1_len=886	RS141_MAIZE	Q:158-574,H:1-138	94.24%ID	E:2e-77	40S ribosomal protein S14	PF00411.16^Ribosomal_S11^Ribosomal protein S11^80-198^E:2.4e-47	0	-0,975	0,009	-0,6	0,753	-0,086	0,141	-0,393	0,016	-0,551	RS141_MAIZE	40S ribosomal protein S14 (Clone MCH1)		Zea mays (Maize)		Complete proteome; Reference proteome; Ribonucleoprotein; Ribosomal protein
SRR342023_TR20079_c6_g2_i2_len=579	H2A4_ORYSJ	Q:438-115,H:30-137	92.59%ID	E:6e-51	Probable histone H2A.4	PF00125.21^Histone^Core histone H2A/H2B/H3/H4^27-118^E:2e-13`PF16211.2^Histone_H2A_C^C-terminus of histone H2A^121-153^E:1.9e-18	0	-0,859	0,023	-0,617	0,002	0,999	0,018	0,791	0,211	-0,795	H2A4_ORYSJ	Probable histone H2A.4	Os05g0461400 LOC_Os05g38640 OJ1281_H05.14 OJ1525_A02.1 OsJ_018061	Oryza sativa subsp. japonica (Rice)		Chromosome; Complete proteome; DNA-binding; Nucleosome core; Nucleus; Reference proteome
SRR342023_TR27937_c0_g3_i1_len=3061	RH3_ARATH	Q:192-2186,H:2-649	73.12%ID	E:0	DEAD-box ATP-dependent RNA helicase 3, chloroplastic	PF00270.26^DEAD^DEAD/DEAH box helicase^146-320^E:3.4e-47`PF04851.12^ResIII^Type III restriction enzyme, res subunit^161-313^E:6.4e-07`PF00271.28^Helicase_C^Helicase conserved C-terminal domain^358-465^E:2.9e-30`PF08152.9^GUCT^GUCT (NUC152) domain^552-652^E:3.3e-19`PF00098.20^zf-CCHC^Zinc knuckle^742-757^E:1.2e-06`PF14392.3^zf-CCHC_4^Zinc knuckle^742-757^E:0.0061	0	-1,818	0,517	1,026	1	0,087	0,059	1,611	0,025	2,024	RH3_ARATH	DEAD-box ATP-dependent RNA helicase 3, chloroplastic (EC 3.6.4.13) (Protein EMBRYO DEFECTIVE 1138)	RH3 EMB1138 At5g26742 F21E10.1 T7I7.6	Arabidopsis thaliana (Mouse-ear cress)		ATP-binding; Alternative splicing; Chloroplast; Complete proteome; Helicase; Hydrolase; Metal-binding; Nucleotide-binding; Phosphoprotein; Plastid; RNA-binding; Reference proteome; Transit peptide; Zinc; Zinc-finger
SRR646572_TR4777_c0_g1_i1_len=1054	RS92_ARATH	Q:880-347,H:1-178	92.13%ID	E:2e-117	40S ribosomal protein S9-2	PF00163.16^Ribosomal_S4^Ribosomal protein S4/S9 N-terminal domain^8-64^E:2.2e-10`PF01479.22^S4^S4 domain^109-152^E:7.6e-13	0	-1,197	0,034	-0,477	0,786	-0,071	0,188	-0,338	0,009	-0,662	RS92_ARATH	40S ribosomal protein S9-2	RPS9C At5g39850 MYH19.1 MYH19.10	Arabidopsis thaliana (Mouse-ear cress)		Complete proteome; RNA-binding; Reference proteome; Ribonucleoprotein; Ribosomal protein; rRNA-binding
SRR646572_TR10126_c0_g2_i10_len=1791	CYSP3_SOLLC	Q:1585-809,H:44-302	78.38%ID	E:2e-150	Cysteine proteinase 3	PF00112.20^Peptidase_C1^Papain family cysteine protease^53-211^E:4.5e-51	0,001	-4,394	1	0	1	2,637	1	3,53	1	0	CYSP3_SOLLC	Cysteine proteinase 3 (EC 3.4.22.-)	CYP-3	Solanum lycopersicum (Tomato) (Lycopersicon esculentum)		Complete proteome; Disulfide bond; Glycoprotein; Hydrolase; Protease; Reference proteome; Signal; Thiol protease; Vacuole; Zymogen
SRR646572_TR15250_c0_g1_i1_len=740	RL31_NICGU	Q:150-509,H:1-120	91.67%ID	E:2e-56	60S ribosomal protein L31	PF01198.16^Ribosomal_L31e^Ribosomal protein L31e^11-92^E:8.6e-44	0,001	-1,02	0,17	-0,443	0,057	0,749	0,113	0,632	0,038	-0,54	RL31_NICGU	60S ribosomal protein L31	RPL31	Nicotiana glutinosa (Tobacco)		Ribonucleoprotein; Ribosomal protein
SRR646572_TR24379_c0_g1_i1_len=1162	RL24_PRUAV	Q:80-382,H:1-101	97.03%ID	E:3e-55	60S ribosomal protein L24	PF01246.17^Ribosomal_L24e^Ribosomal protein L24e^3-67^E:6e-30	0	-1,521	0,416	-0,235	0,165	0,506	0,799	0,134	0,804	0,102	RL24_PRUAV	60S ribosomal protein L24	RPL24	Prunus avium (Cherry) (Cerasus avium)		Ribonucleoprotein; Ribosomal protein
SRR646596_TR2772_c0_g1_i1_len=1453	RS6_ASPOF	Q:1304-501,H:1-243	85.07%ID	E:1e-127	40S ribosomal protein S6	.	0	-1,148	0,355	0,348	0,431	0,316	0,684	0,19	0,538	0,219	RS6_ASPOF	40S ribosomal protein S6	rps6	Asparagus officinalis (Garden asparagus)		Phosphoprotein; Ribonucleoprotein; Ribosomal protein
SRR646596_TR16528_c1_g2_i1_len=803	RS27A_SOLTU	Q:673-206,H:1-156	96.79%ID	E:3e-86	Ubiquitin-40S ribosomal protein S27a	PF11976.5^Rad60-SLD^Ubiquitin-2 like Rad60 SUMO-like^1-71^E:2.5e-16`PF00240.20^ubiquitin^Ubiquitin family^3-74^E:3.7e-34`PF14560.3^Ubiquitin_2^Ubiquitin-like domain^12-69^E:0.00017`PF13881.3^Rad60-SLD_2^Ubiquitin-2 like Rad60 SUMO-like^13-90^E:0.00085`PF01599.16^Ribosomal_S27^Ribosomal protein S27a^102-147^E:2.8e-26	0	-0,978	0,397	-0,229	0,397	0,27	0,68	-0,138	0,472	-0,731	RS27A_SOLTU	Ubiquitin-40S ribosomal protein S27a [Cleaved into: Ubiquitin; 40S ribosomal protein S27a]	UBI3 RPS27A	Solanum tuberosum (Potato)		Complete proteome; Cytoplasm; Isopeptide bond; Metal-binding; Nucleus; Reference proteome; Ribonucleoprotein; Ribosomal protein; Ubl conjugation; Zinc; Zinc-finger
SRR646596_TR21334_c0_g1_i1_len=850	RS23_FRAAN	Q:154-579,H:1-142	98.59%ID	E:4e-95	40S ribosomal protein S23	PF00164.22^Ribosom_S12_S23^Ribosomal protein S12/S23^9-141^E:6e-60	0	-1,164	0,538	-0,172	0,536	0,23	0,894	-0,052	0,502	-4,26	RS23_FRAAN	40S ribosomal protein S23 (S12)	RPS23	Fragaria ananassa (Strawberry)		Hydroxylation; Ribonucleoprotein; Ribosomal protein
SRR646596_TR21991_c0_g1_i1_len=2286	CH62_CUCMA	Q:2180-486,H:1-565	90.97%ID	E:0	Chaperonin CPN60-2, mitochondrial	PF00118.21^Cpn60_TCP1^TCP-1/cpn60 chaperonin family^54-555^E:6.8e-86	0,001	-1,157	0,038	0,703	0,153	0,769	1	0,098	0,854	-0,111	CH62_CUCMA	Chaperonin CPN60-2, mitochondrial (HSP60-2)	CPN60-2	Cucurbita maxima (Pumpkin) (Winter squash)		ATP-binding; Chaperone; Direct protein sequencing; Mitochondrion; Nucleotide-binding; Stress response; Transit peptide
SRR646596_TR29729_c0_g1_i1_len=884	ACP1_CASGL	Q:197-607,H:1-136	67.88%ID	E:2e-49	Acyl carrier protein 1, chloroplastic	PF00550.22^PP-binding^Phosphopantetheine attachment site^61-128^E:6.3e-10	0	-1,504	0,813	0,076	0,289	0,359	0,097	0,525	0,046	0,622	ACP1_CASGL	Acyl carrier protein 1, chloroplastic (ACP 1)	ACP1	Casuarina glauca (Swamp oak)	PATHWAY: Lipid metabolism; fatty acid biosynthesis.	Chloroplast; Fatty acid biosynthesis; Fatty acid metabolism; Lipid biosynthesis; Lipid metabolism; Phosphopantetheine; Phosphoprotein; Plastid; Transit peptide
SRR646596_TR31372_c0_g1_i1_len=1519	RL3_ORYSJ	Q:1427-261,H:1-389	88.17%ID	E:0	60S ribosomal protein L3	PF00297.19^Ribosomal_L3^Ribosomal protein L3^31-400^E:8.4e-195	0	-1,279	0,007	1,143	0,655	-0,275	0,232	-0,602	0,436	0,258	RL3_ORYSJ	60S ribosomal protein L3	RPL3 Os12g0167900 LOC_Os12g07010	Oryza sativa subsp. japonica (Rice)		Complete proteome; Cytoplasm; Reference proteome; Ribonucleoprotein; Ribosomal protein
SRR646604_TR14082_c0_g2_i1_len=1901	UBP1_NICPL	Q:1597-437,H:21-406	88.92%ID	E:0	Oligouridylate-binding protein 1	PF00076.19^RRM_1^RNA recognition motif. (a.k.a. RRM, RBD, or RNP domain)^50-116^E:1.1e-13`PF13893.3^RRM_5^RNA recognition motif. (a.k.a. RRM, RBD, or RNP domain)^67-118^E:0.22`PF00076.19^RRM_1^RNA recognition motif. (a.k.a. RRM, RBD, or RNP domain)^135-205^E:3.4e-19`PF00076.19^RRM_1^RNA recognition motif. (a.k.a. RRM, RBD, or RNP domain)^256-322^E:1.1e-12	0	-3,363	1	-0,46	0,005	4,958	0,034	4,236	1	0,18	UBP1_NICPL	Oligouridylate-binding protein 1 (NpUBP1) (Polyuridylate-binding protein UBP1) (Poly(U)-binding protein UBP1)	UBP1	Nicotiana plumbaginifolia (Leadwort-leaved tobacco) (Tex-Mex tobacco)		Nucleus; RNA-binding; Repeat; mRNA processing
SRR646604_TR19240_c0_g1_i4_len=2511	BAM9_ARATH	Q:721-2187,H:67-521	53.67%ID	E:7e-161	Inactive beta-amylase 9	PF01373.14^Glyco_hydro_14^Glycosyl hydrolase family 14^13-210^E:1.6e-34	0	-6,036	0,756	0,25	1	-0,941	1	-0,687	1	0,326	BAM9_ARATH	Inactive beta-amylase 9 (1,4-alpha-D-glucan maltohydrolase) (Inactive beta-amylase 3)	BAM9 BMY3 At5g18670 T1A4.50	Arabidopsis thaliana (Mouse-ear cress)		Carbohydrate metabolism; Complete proteome; Cytoplasm; Phosphoprotein; Polysaccharide degradation; Reference proteome
SRR646604_TR30453_c0_g1_i1_len=589	H32_WHEAT	Q:488-81,H:1-136	99.26%ID	E:7e-78	Histone H3.2	PF00125.21^Histone^Core histone H2A/H2B/H3/H4^34-165^E:1.8e-54	0	-7,367	1	-2,289	1	0	1	0	1	0	H32_WHEAT	Histone H3.2		Triticum aestivum (Wheat)		Acetylation; Chromosome; Complete proteome; DNA-binding; Direct protein sequencing; Methylation; Nucleosome core; Nucleus; Phosphoprotein; Reference proteome
SRR646604_TR31202_c0_g1_i1_len=679	RL273_ARATH	Q:113-517,H:1-135	80%ID	E:1e-62	60S ribosomal protein L27-3	PF01777.15^Ribosomal_L27e^Ribosomal L27e protein family^52-135^E:6.9e-34	0	-8,111	0,452	-0,588	1	-0,061	0,103	-1,495	0,732	-0,108	RL273_ARATH	60S ribosomal protein L27-3	RPL27C At4g15000 dl3545w FCAALL.99	Arabidopsis thaliana (Mouse-ear cress)		Alternative splicing; Complete proteome; Reference proteome; Ribonucleoprotein; Ribosomal protein
SRR648705_TR1373_c0_g2_i1_len=999	RS7_AVIMR	Q:136-705,H:1-190	83.16%ID	E:2e-114	40S ribosomal protein S7	PF01251.15^Ribosomal_S7e^Ribosomal protein S7e^7-187^E:2e-78	0	-1,195	0,349	-0,238	0,878	-0,056	0,047	-0,624	0,274	-0,268	RS7_AVIMR	40S ribosomal protein S7	RPS7	Avicennia marina (Grey mangrove) (Sceura marina)		Ribonucleoprotein; Ribosomal protein
SRR648705_TR3981_c0_g1_i3_len=2589	CH62_CUCMA	Q:198-1892,H:1-565	89.38%ID	E:0	Chaperonin CPN60-2, mitochondrial	PF00118.21^Cpn60_TCP1^TCP-1/cpn60 chaperonin family^54-555^E:4.7e-84	0	-1,425	1	0,018	0,014	3,56	0,381	2,233	0,648	-0,197	CH62_CUCMA	Chaperonin CPN60-2, mitochondrial (HSP60-2)	CPN60-2	Cucurbita maxima (Pumpkin) (Winter squash)		ATP-binding; Chaperone; Direct protein sequencing; Mitochondrion; Nucleotide-binding; Stress response; Transit peptide
SRR648705_TR5819_c1_g1_i1_len=409	NA	NA	NA	NA	NA	.	0,001	-6,657	0,283	0,63	0,001	2,969	0,066	2,09	0,692	-1,004	.	.	.	.	.	.
SRR648705_TR7638_c1_g1_i2_len=1108	RL111_ARATH	Q:224-760,H:2-180	95.53%ID	E:3e-112	60S ribosomal protein L11-1	PF00281.16^Ribosomal_L5^Ribosomal protein L5^1-52^E:2.4e-19`PF00673.18^Ribosomal_L5_C^ribosomal L5P family C-terminus^56-154^E:5.5e-21	0,001	-1,797	1	0,076	1	0,123	0,643	-0,445	0,062	-0,465	RL111_ARATH	60S ribosomal protein L11-1 (L16A)	RPL11A RPL16A At2g42740 F7D19.26	Arabidopsis thaliana (Mouse-ear cress)		Complete proteome; Cytoplasm; RNA-binding; Reference proteome; Ribonucleoprotein; Ribosomal protein; rRNA-binding
SRR648705_TR8541_c2_g1_i3_len=1354	RSSA_DAUCA	Q:163-978,H:1-269	76.92%ID	E:2e-138	40S ribosomal protein SA {ECO:0000255|HAMAP-Rule:MF_03015}	PF00318.17^Ribosomal_S2^Ribosomal protein S2^18-112^E:1.4e-12`PF00318.17^Ribosomal_S2^Ribosomal protein S2^117-182^E:1.9e-12	0	-1,173	0	10,892	1	0	1	0	0,353	-0,229	RSSA_DAUCA	40S ribosomal protein SA (p40)	179B	Daucus carota (Wild carrot)		Cytoplasm; Ribonucleoprotein; Ribosomal protein
SRR648705_TR13379_c0_g1_i1_len=1139	RS212_ARATH	Q:627-869,H:1-81	81.48%ID	E:4e-42	40S ribosomal protein S21-2	.	0,001	-3,379	1	-0,046	1	2,862	1	0	0,066	1,735	RS212_ARATH	40S ribosomal protein S21-2	RPS21C At5g27700 T1G16.30	Arabidopsis thaliana (Mouse-ear cress)		Acetylation; Complete proteome; Reference proteome; Ribonucleoprotein; Ribosomal protein
SRR648705_TR13379_c0_g1_i3_len=692	RS212_ARATH	Q:180-422,H:1-81	81.48%ID	E:3e-44	40S ribosomal protein S21-2	.	0	-7,167	0,282	-0,271	1	0,174	1	0,232	0	4,454	RS212_ARATH	40S ribosomal protein S21-2	RPS21C At5g27700 T1G16.30	Arabidopsis thaliana (Mouse-ear cress)		Acetylation; Complete proteome; Reference proteome; Ribonucleoprotein; Ribosomal protein
SRR648705_TR13569_c0_g2_i2_len=2079	EF1A_TOBAC	Q:1791-484,H:1-436	97.94%ID	E:0	Elongation factor 1-alpha	PF00009.24^GTP_EFTU^Elongation factor Tu GTP binding domain^6-221^E:1e-52`PF03144.22^GTP_EFTU_D2^Elongation factor Tu domain 2^248-313^E:7.3e-15`PF03143.14^GTP_EFTU_D3^Elongation factor Tu C-terminal domain^322-429^E:3.9e-38	0	-0,948	0,287	0,281	0,005	-0,724	0,007	-0,697	0,904	-0,057	EF1A_TOBAC	Elongation factor 1-alpha (EF-1-alpha) (Vitronectin-like adhesion protein 1) (PVN1)		Nicotiana tabacum (Common tobacco)		Cytoplasm; Elongation factor; GTP-binding; Nucleotide-binding; Phosphoprotein; Protein biosynthesis
SRR648705_TR13636_c0_g1_i1_len=2030	RL7A_ORYSJ	Q:205-981,H:1-258	87.64%ID	E:1e-146	60S ribosomal protein L7a	PF01248.23^Ribosomal_L7Ae^Ribosomal protein L7Ae/L30e/S12e/Gadd45 family^117-206^E:9.9e-25	0	-1,173	0,162	-0,454	0,407	0,474	0,57	0,351	0,219	-0,408	RL7A_ORYSJ	60S ribosomal protein L7a	RPL7A Os08g0326400 LOC_Os08g23710 OJ1136_A10.108 P0703C03.43	Oryza sativa subsp. japonica (Rice)		Complete proteome; Reference proteome; Ribonucleoprotein; Ribosomal protein
SRR648705_TR15837_c0_g1_i1_len=885	R15A1_ARATH	Q:171-560,H:1-130	97.69%ID	E:2e-87	40S ribosomal protein S15a-1	PF00410.16^Ribosomal_S8^Ribosomal protein S8^7-130^E:5.5e-22	0	-0,962	0,236	-0,264	0,015	0,601	0,224	0,308	0,469	-0,161	R15A1_ARATH	40S ribosomal protein S15a-1	RPS15AA At1g07770 F24B9.12; RPS15AF At5g59850 MMN10.16 MMN10.8	Arabidopsis thaliana (Mouse-ear cress)		Complete proteome; Cytoplasm; Reference proteome; Ribonucleoprotein; Ribosomal protein
SRR648705_TR16695_c0_g1_i3_len=578	NA	NA	NA	NA	NA	.	0	-1,03	0,002	-0,744	0,195	0,351	0,684	0,124	0,014	-0,554	.	.	.	.	.	.
SRR648705_TR19195_c0_g2_i1_len=3341	MCM2_ORYSI	Q:3068-267,H:6-960	79.33%ID	E:0	DNA replication licensing factor MCM2	PF12619.5^MCM2_N^Mini-chromosome maintenance protein 2^62-218^E:1.7e-22`PF14551.3^MCM_N^MCM N-terminal domain^249-365^E:9.1e-22`PF00493.20^MCM^MCM2/3/5 family^516-859^E:6e-142`PF07728.11^AAA_5^AAA domain (dynein-related subfamily)^573-692^E:9.2e-06`PF01078.18^Mg_chelatase^Magnesium chelatase, subunit ChlI^627-715^E:1.2e-06	0,001	-1,369	0,132	-1,3	0,732	0,657	1	-0,305	0,419	-0,389	MCM2_ORYSI	DNA replication licensing factor MCM2 (EC 3.6.4.12) (Minichromosome maintenance protein 2)	OsI_36121	Oryza sativa subsp. indica (Rice)		ATP-binding; Cell cycle; Complete proteome; DNA replication; DNA-binding; Helicase; Hydrolase; Metal-binding; Nucleotide-binding; Nucleus; Reference proteome; Zinc; Zinc-finger
SRR648705_TR19965_c0_g1_i3_len=924	IF2B_WHEAT	Q:694-2,H:4-188	52.12%ID	E:7e-52	Eukaryotic translation initiation factor 2 subunit beta	.	0	-7,069	0,254	4,779	1	-0,261	0,356	-1,484	0,495	-0,199	IF2B_WHEAT	Eukaryotic translation initiation factor 2 subunit beta (eIF-2-beta) (P38)		Triticum aestivum (Wheat)		Complete proteome; Direct protein sequencing; Initiation factor; Metal-binding; Protein biosynthesis; Reference proteome; Zinc; Zinc-finger
SRR648705_TR22960_c0_g1_i1_len=894	RS263_ARATH	Q:127-369,H:1-81	87.65%ID	E:2e-32	40S ribosomal protein S26-3	PF01283.16^Ribosomal_S26e^Ribosomal protein S26e^1-104^E:1e-53	0	-0,984	0,758	-0,086	0,05	0,605	0,329	0,32	0,163	-0,334	RS263_ARATH	40S ribosomal protein S26-3	RPS26C At3g56340 F18O21_300	Arabidopsis thaliana (Mouse-ear cress)		Complete proteome; Reference proteome; Ribonucleoprotein; Ribosomal protein
SRR648705_TR27096_c1_g1_i1_len=652	RS191_ARATH	Q:535-107,H:1-143	86.71%ID	E:2e-88	40S ribosomal protein S19-1	PF01090.16^Ribosomal_S19e^Ribosomal protein S19e^6-140^E:7.4e-59	0	-1,286	0,003	0,996	0,657	0,17	0,53	-0,22	0	1,55	RS191_ARATH	40S ribosomal protein S19-1	RPS19A At3g02080 F1C9.13	Arabidopsis thaliana (Mouse-ear cress)		Complete proteome; Reference proteome; Ribonucleoprotein; Ribosomal protein
SRR648705_TR29461_c0_g2_i5_len=562	H4_SOYBN	Q:212-457,H:22-103	100%ID	E:4e-50	Histone H4	PF15511.3^CENP-T_C^Centromere kinetochore component CENP-T histone fold^35-96^E:1.4e-08`PF02969.14^TAF^TATA box binding protein associated factor (TAF)^35-92^E:0.00016	0	-2,19	0,001	-3,286	1	-0,812	1	-0,696	0,295	-0,255	H4_SOYBN	Histone H4		Glycine max (Soybean) (Glycine hispida)		Acetylation; Chromosome; Complete proteome; DNA-binding; Methylation; Nucleosome core; Nucleus; Reference proteome
SRR648705_TR30487_c0_g2_i2_len=1724	CYSP3_SOLLC	Q:1450-512,H:44-356	80.83%ID	E:0	Cysteine proteinase 3	PF00112.20^Peptidase_C1^Papain family cysteine protease^53-268^E:5.7e-79	0	-3,322	1	0,069	0	4,575	0	4,311	0,894	-0,082	CYSP3_SOLLC	Cysteine proteinase 3 (EC 3.4.22.-)	CYP-3	Solanum lycopersicum (Tomato) (Lycopersicon esculentum)		Complete proteome; Disulfide bond; Glycoprotein; Hydrolase; Protease; Reference proteome; Signal; Thiol protease; Vacuole; Zymogen
SRR648705_TR30582_c0_g1_i1_len=1250	RL8_SOLLC	Q:161-874,H:1-238	95.8%ID	E:2e-165	60S ribosomal protein L8	PF00181.20^Ribosomal_L2^Ribosomal Proteins L2, RNA binding domain^13-90^E:1e-17`PF03947.15^Ribosomal_L2_C^Ribosomal Proteins L2, C-terminal domain^98-230^E:1.8e-46	0	-0,986	0,21	0,342	0,571	0,186	0,696	-0,132	0,82	-0,073	RL8_SOLLC	60S ribosomal protein L8 (L2) (Ribosomal protein TL2)	RPL8 RPL2	Solanum lycopersicum (Tomato) (Lycopersicon esculentum)		Complete proteome; Cytoplasm; Reference proteome; Ribonucleoprotein; Ribosomal protein
SRR648705_TR34196_c0_g1_i1_len=1834	NA	NA	NA	NA	NA	.	0	-5,204	0,724	0,201	1	0	1	2,423	1	0,516	.	.	.	.	.	.
SRR648705_TR36301_c0_g1_i2_len=918	RL23A_TOBAC	Q:82-546,H:1-154	92.26%ID	E:3e-67	60S ribosomal protein L23a	PF03939.10^Ribosomal_L23eN^Ribosomal protein L23, N-terminal domain^15-65^E:2.9e-17`PF00276.17^Ribosomal_L23^Ribosomal protein L23^75-141^E:2.8e-14	0,001	-0,866	0,177	-0,369	0,691	0,14	0,63	-0,147	0,304	-0,263	RL23A_TOBAC	60S ribosomal protein L23a (L25)	RPL23A RPL25	Nicotiana tabacum (Common tobacco)		RNA-binding; Ribonucleoprotein; Ribosomal protein; rRNA-binding
SRR648705_TR36350_c1_g3_i3_len=901	RL321_ARATH	Q:756-358,H:1-133	86.47%ID	E:2e-72	60S ribosomal protein L32-1	PF01655.15^Ribosomal_L32e^Ribosomal protein L32e^16-122^E:2.7e-50	0	-1,123	0,382	-0,23	0,031	0,636	0,497	0,211	0,878	-0,146	RL321_ARATH	60S ribosomal protein L32-1	RPL32A At4g18100 F15J5.70	Arabidopsis thaliana (Mouse-ear cress)		Complete proteome; Reference proteome; Ribonucleoprotein; Ribosomal protein
SRR648705_TR38531_c0_g1_i1_len=1329	CH10C_ARATH	Q:1060-293,H:1-253	75.97%ID	E:2e-129	20 kDa chaperonin, chloroplastic	PF00166.18^Cpn10^Chaperonin 10 Kd subunit^65-154^E:7.4e-28`PF00166.18^Cpn10^Chaperonin 10 Kd subunit^163-254^E:5.3e-28	0	-0,901	0,01	-0,616	0,104	0,416	0,025	0,568	0,92	-0,094	CH10C_ARATH	20 kDa chaperonin, chloroplastic (Chaperonin 10) (Ch-CPN10) (Cpn10) (Chaperonin 20) (Protein Cpn21)	CPN21 CHCPN10 CPN20 At5g20720 T1M15.120	Arabidopsis thaliana (Mouse-ear cress)		Chaperone; Chloroplast; Complete proteome; Phosphoprotein; Plastid; Reference proteome; Repeat; Transit peptide
SRR648705_TR43618_c0_g1_i16_len=876	RS12_HORVU	Q:225-596,H:20-143	82.26%ID	E:4e-69	40S ribosomal protein S12	PF01248.23^Ribosomal_L7Ae^Ribosomal protein L7Ae/L30e/S12e/Gadd45 family^27-120^E:4e-27	0	-1,036	0	9,862	1	0	1	0	0,67	-0,115	RS12_HORVU	40S ribosomal protein S12	RPS12	Hordeum vulgare (Barley)		Ribonucleoprotein; Ribosomal protein
SRR648707_TR953_c0_g1_i1_len=956	RL262_ARATH	Q:149-466,H:1-106	84.91%ID	E:3e-50	60S ribosomal protein L26-2	PF16906.2^Ribosomal_L26^Ribosomal proteins L26 eukaryotic, L24P archaeal^8-121^E:5.1e-38`PF00467.26^KOW^KOW motif^51-82^E:7.8e-10	0	-0,982	0,028	-0,514	0,806	0,077	0,522	-0,183	0,081	-0,408	RL262_ARATH	60S ribosomal protein L26-2	RPL26B At5g67510 K9I9.7	Arabidopsis thaliana (Mouse-ear cress)		Complete proteome; Reference proteome; Ribonucleoprotein; Ribosomal protein
SRR648707_TR979_c0_g1_i1_len=702	RL38_ARATH	Q:445-239,H:1-69	95.65%ID	E:8e-39	60S ribosomal protein L38	.	0	-0,985	0,46	-0,171	0,015	0,631	0,744	0,098	0,024	-0,548	RL38_ARATH	60S ribosomal protein L38	RPL38A RPL38 At2g43460 T1O24.20; RPL38B At3g59540 T16L24.90	Arabidopsis thaliana (Mouse-ear cress)		Complete proteome; Reference proteome; Ribonucleoprotein; Ribosomal protein
SRR648707_TR7665_c0_g2_i1_len=941	RS23_FRAAN	Q:189-614,H:1-142	98.59%ID	E:1e-94	40S ribosomal protein S23	PF00164.22^Ribosom_S12_S23^Ribosomal protein S12/S23^9-141^E:6e-60	0	-1,067	0,84	0,073	0,061	0,645	0,573	0,208	0,154	-0,39	RS23_FRAAN	40S ribosomal protein S23 (S12)	RPS23	Fragaria ananassa (Strawberry)		Hydroxylation; Ribonucleoprotein; Ribosomal protein
SRR648707_TR8998_c0_g1_i1_len=753	CML11_ARATH	Q:150-578,H:28-170	68.53%ID	E:6e-66	Calmodulin-like protein 11	PF00036.29^EF-hand_1^EF hand^14-41^E:3.2e-06`PF13405.3^EF-hand_6^EF-hand domain^14-41^E:2.5e-06`PF13499.3^EF-hand_7^EF-hand domain pair^15-75^E:2e-13`PF13202.3^EF-hand_5^EF hand^17-38^E:0.0002`PF13833.3^EF-hand_8^EF-hand domain pair^27-76^E:3.9e-11`PF00036.29^EF-hand_1^EF hand^50-77^E:6.4e-07`PF13499.3^EF-hand_7^EF-hand domain pair^86-148^E:9.8e-17`PF13833.3^EF-hand_8^EF-hand domain pair^86-112^E:0.022`PF00036.29^EF-hand_1^EF hand^87-114^E:6.1e-09`PF13405.3^EF-hand_6^EF-hand domain^87-116^E:2.6e-08`PF13202.3^EF-hand_5^EF hand^88-112^E:1.5e-07`PF13833.3^EF-hand_8^EF-hand domain pair^99-148^E:1.2e-11`PF00036.29^EF-hand_1^EF hand^123-150^E:1.4e-08`PF13202.3^EF-hand_5^EF hand^127-148^E:0.00019	0	-5,216	1	0,324	0,629	1,089	1	-0,199	0,706	-0,138	CML11_ARATH	Calmodulin-like protein 11	CML11 At3g22930 F5N5.10	Arabidopsis thaliana (Mouse-ear cress)		Calcium; Complete proteome; Metal-binding; Reference proteome; Repeat
SRR648707_TR10132_c0_g1_i4_len=1302	RS5_CICAR	Q:193-723,H:5-181	87.57%ID	E:3e-109	40S ribosomal protein S5	PF00177.18^Ribosomal_S7^Ribosomal protein S7p/S5e^63-190^E:7.6e-29	0,001	-1,084	0,362	0,329	0,787	0,151	1	-0,009	0,568	0,212	RS5_CICAR	40S ribosomal protein S5 (Fragment)	RPS5	Cicer arietinum (Chickpea) (Garbanzo)		Ribonucleoprotein; Ribosomal protein
SRR648707_TR11923_c0_g1_i1_len=1793	ERGI3_XENTR	Q:1648-608,H:7-374	37.03%ID	E:2e-64	Endoplasmic reticulum-Golgi intermediate compartment protein 3	PF13850.3^ERGIC_N^Endoplasmic Reticulum-Golgi Intermediate Compartment (ERGIC)^7-96^E:1e-25`PF07970.9^COPIIcoated_ERV^Endoplasmic reticulum vesicle transporter^169-343^E:4.3e-46	0	-6,78	0,094	-1,059	0,356	-1,19	0,017	-5,859	1	0	ERGI3_XENTR	Endoplasmic reticulum-Golgi intermediate compartment protein 3	ergic3	Xenopus tropicalis (Western clawed frog) (Silurana tropicalis)		Complete proteome; ER-Golgi transport; Endoplasmic reticulum; Golgi apparatus; Membrane; Reference proteome; Transmembrane; Transmembrane helix; Transport
SRR648707_TR12460_c0_g3_i2_len=1032	RS8_ORYSJ	Q:125-757,H:1-212	93.87%ID	E:3e-103	40S ribosomal protein S8	PF01201.19^Ribosomal_S8e^Ribosomal protein S8e^1-197^E:1.1e-58	0	-0,919	0,515	-0,149	0,185	0,352	0,668	0,124	0,22	-0,272	RS8_ORYSJ	40S ribosomal protein S8	RPS8 Os02g0489400 LOC_Os02g28810 OSJNBa0048K16.2 P0483C08.42	Oryza sativa subsp. japonica (Rice)		Complete proteome; Reference proteome; Ribonucleoprotein; Ribosomal protein
SRR648707_TR13304_c0_g2_i1_len=1087	RS17_SOLLC	Q:189-581,H:1-131	86.26%ID	E:9e-73	40S ribosomal protein S17	PF00833.15^Ribosomal_S17e^Ribosomal S17^1-116^E:2e-57	0	-1,196	0,943	-0,023	0	1,204	0,001	1,004	0,041	-0,482	RS17_SOLLC	40S ribosomal protein S17	RPS17 GI1	Solanum lycopersicum (Tomato) (Lycopersicon esculentum)		Complete proteome; Reference proteome; Ribonucleoprotein; Ribosomal protein
SRR648707_TR15317_c0_g2_i1_len=3193	CMT2_ARATH	Q:1839-2924,H:929-1294	60.22%ID	E:0	DNA (cytosine-5)-methyltransferase CMT2	PF01426.15^BAH^BAH domain^156-261^E:2.7e-08`PF00385.21^Chromo^Chromo (CHRromatin Organisation MOdifier) domain^423-477^E:6.5e-10	0,001	-1,367	0,025	-1,986	1	-0,306	1	-0,361	0,26	-0,489	CMT2_ARATH	DNA (cytosine-5)-methyltransferase CMT2 (EC 2.1.1.37) (Chromomethylase 2) (Protein CHROMOMETHYLASE 2)	CMT2 At4g19020 F13C5.190	Arabidopsis thaliana (Mouse-ear cress)		Chromatin regulator; Complete proteome; DNA-binding; Methyltransferase; Nucleus; Reference proteome; S-adenosyl-L-methionine; Transcription; Transcription regulation; Transferase
SRR648707_TR19754_c0_g1_i1_len=719	NA	NA	NA	NA	NA	PF00428.16^Ribosomal_60s^60s Acidic ribosomal protein^18-112^E:2.8e-26`PF12868.4^DUF3824^Domain of unknwon function (DUF3824)^72-103^E:0.3	0	-0,914	0,178	-0,321	0,441	0,217	0,937	-0,028	0,537	-0,305	.	.	.	.	.	.
SRR648707_TR22744_c0_g2_i3_len=1143	RL5_CUCSA	Q:1014-166,H:1-283	85.51%ID	E:2e-158	60S ribosomal protein L5	PF17144.1^Ribosomal_L5e^Ribosomal large subunit proteins 60S L5, and 50S L18^14-175^E:3.4e-83`PF14204.3^Ribosomal_L18_c^Ribosomal L18 C-terminal region^192-280^E:2.5e-36	0	-1,216	0,039	0,575	0,787	-0,086	1	-0,011	0,822	0,244	RL5_CUCSA	60S ribosomal protein L5	RPL5	Cucumis sativus (Cucumber)		RNA-binding; Ribonucleoprotein; Ribosomal protein; rRNA-binding
SRR648707_TR24099_c1_g2_i1_len=2134	EF1A_TOBAC	Q:1860-556,H:1-435	97.93%ID	E:0	Elongation factor 1-alpha	PF00009.24^GTP_EFTU^Elongation factor Tu GTP binding domain^6-221^E:1e-52`PF03144.22^GTP_EFTU_D2^Elongation factor Tu domain 2^248-313^E:7.3e-15`PF03143.14^GTP_EFTU_D3^Elongation factor Tu C-terminal domain^322-429^E:3.9e-38	0	-0,894	0	0,911	0,689	-0,103	0,316	-0,253	1	0,022	EF1A_TOBAC	Elongation factor 1-alpha (EF-1-alpha) (Vitronectin-like adhesion protein 1) (PVN1)		Nicotiana tabacum (Common tobacco)		Cytoplasm; Elongation factor; GTP-binding; Nucleotide-binding; Phosphoprotein; Protein biosynthesis
SRR648707_TR30186_c0_g1_i2_len=1248	RL24_PRUAV	Q:479-682,H:34-101	97.06%ID	E:7e-30	60S ribosomal protein L24	.	0	-1,25	0,006	-0,74	0,488	0,272	0,885	0,09	0,069	-0,502	RL24_PRUAV	60S ribosomal protein L24	RPL24	Prunus avium (Cherry) (Cerasus avium)		Ribonucleoprotein; Ribosomal protein
SRR648707_TR32373_c0_g1_i1_len=831	H2B_GOSHI	Q:507-199,H:45-147	96.12%ID	E:3e-58	Histone H2B	PF00125.21^Histone^Core histone H2A/H2B/H3/H4^9-124^E:7.3e-23`PF00808.20^CBFD_NFYB_HMF^Histone-like transcription factor (CBF/NF-Y) and archaeal histone^66-122^E:4.4e-05	0	-1,06	0,032	-0,574	0	1,197	0,018	0,751	0,317	-0,514	H2B_GOSHI	Histone H2B	HIS2B	Gossypium hirsutum (Upland cotton) (Gossypium mexicanum)		Acetylation; Chromosome; DNA-binding; Isopeptide bond; Nucleosome core; Nucleus; Ubl conjugation
SRR648707_TR36565_c0_g2_i1_len=847	RS16_FRIAG	Q:718-296,H:5-145	91.49%ID	E:1e-89	40S ribosomal protein S16	PF00380.16^Ribosomal_S9^Ribosomal protein S9/S16^11-143^E:7e-34	0	-0,913	0,401	-0,202	0,653	0,132	0,761	-0,093	1	0,079	RS16_FRIAG	40S ribosomal protein S16	RPS16	Fritillaria agrestis (Stinkbells)		Cytoplasm; Ribonucleoprotein; Ribosomal protein
SRR648707_TR39487_c0_g1_i1_len=1298	RFA2A_ARATH	Q:1146-352,H:1-275	41.82%ID	E:1e-59	Replication protein A 32 kDa subunit A	PF01336.22^tRNA_anti-codon^OB-fold nucleic acid binding domain^86-137^E:9.1e-07`PF08784.8^RPA_C^Replication protein A C terminal^173-261^E:1.2e-14	0	-1,53	0,001	-3,16	0,732	0,587	0,629	-1,225	0,029	-0,896	RFA2A_ARATH	Replication protein A 32 kDa subunit A (AtRPA32A) (RP-A p32 A) (DNA replication protein A2 subunit A) (Protein SUPPRESSOR OF ROS1) (Replication factor A protein 2 A) (AtRPA2 A) (RF-A protein 2 A) (Replicon protein A2 A)	RPA2A ROR1 RPA32A At2g24490 T28I24.22	Arabidopsis thaliana (Mouse-ear cress)		Complete proteome; DNA damage; DNA recombination; DNA repair; DNA replication; DNA-binding; Nucleus; Phosphoprotein; Reference proteome
SRR924147_TR1360_c0_g2_i2_len=663	NA	NA	NA	NA	NA	.	0	-2,184	0,409	0,411	1	0,03	0,679	-0,383	0,389	-0,419	.	.	.	.	.	.
SRR924147_TR2226_c0_g6_i1_len=1054	RL44_GOSHI	Q:708-394,H:1-105	99.05%ID	E:5e-54	60S ribosomal protein L44	PF00935.16^Ribosomal_L44^Ribosomal protein L44^19-94^E:3.3e-36	0	-1,071	0,228	-0,309	0,228	0,362	0,659	-0,155	0,555	-0,157	RL44_GOSHI	60S ribosomal protein L44	RPL44 RL44	Gossypium hirsutum (Upland cotton) (Gossypium mexicanum)		Ribonucleoprotein; Ribosomal protein
SRR924147_TR2531_c2_g1_i1_len=3011	RIR1_ARATH	Q:356-2797,H:1-816	84.56%ID	E:0	Ribonucleoside-diphosphate reductase large subunit {ECO:0000303|PubMed:10542051}	PF03477.13^ATP-cone^ATP cone domain^2-90^E:1e-16`PF00317.18^Ribonuc_red_lgN^Ribonucleotide reductase, all-alpha domain^143-213^E:5.1e-24`PF02867.12^Ribonuc_red_lgC^Ribonucleotide reductase, barrel domain^217-758^E:5.4e-188	0,001	-1,487	0,017	-1,989	1	0,995	1	0,148	0,043	-0,975	RIR1_ARATH	Ribonucleoside-diphosphate reductase large subunit (EC 1.17.4.1) (Protein DEFECTIVE IN POLLEN DNA DEGRADATION 2) (Ribonucleoside-diphosphate reductase R1 subunit) (AtRNR1)	RNR1 DPD2 At2g21790 F7D8.11	Arabidopsis thaliana (Mouse-ear cress)	PATHWAY: Genetic information processing; DNA replication.	ATP-binding; Allosteric enzyme; Complete proteome; Cytoplasm; DNA replication; Disulfide bond; Nucleotide-binding; Oxidoreductase; Reference proteome
SRR924147_TR3713_c3_g1_i1_len=994	RL18A_CASSA	Q:50-583,H:1-178	91.01%ID	E:1e-117	60S ribosomal protein L18a	PF01775.14^Ribosomal_L18A^Ribosomal proteins 50S-L18Ae/60S-L20/60S-L18A^23-144^E:2.8e-55	0	-0,957	1	-0,001	0,842	0,062	0,755	-0,109	0,51	-0,157	RL18A_CASSA	60S ribosomal protein L18a	RPL18A	Castanea sativa (Sweet chestnut)		Ribonucleoprotein; Ribosomal protein
SRR924147_TR4590_c0_g3_i1_len=1444	RS5_CICAR	Q:195-773,H:5-197	89.12%ID	E:2e-122	40S ribosomal protein S5	PF00177.18^Ribosomal_S7^Ribosomal protein S7p/S5e^63-208^E:8.1e-37	0	-0,949	0,303	-0,254	0,292	0,307	0,755	0,098	0,025	-0,565	RS5_CICAR	40S ribosomal protein S5 (Fragment)	RPS5	Cicer arietinum (Chickpea) (Garbanzo)		Ribonucleoprotein; Ribosomal protein
SRR924147_TR8339_c0_g2_i1_len=1029	RL111_ARATH	Q:914-375,H:1-180	95.56%ID	E:5e-114	60S ribosomal protein L11-1	PF00281.16^Ribosomal_L5^Ribosomal protein L5^9-62^E:6.1e-21`PF00673.18^Ribosomal_L5_C^ribosomal L5P family C-terminus^66-164^E:6.4e-21	0	-1,739	0,424	-0,302	0,882	0,149	0,874	-0,163	0,003	-1,605	RL111_ARATH	60S ribosomal protein L11-1 (L16A)	RPL11A RPL16A At2g42740 F7D19.26	Arabidopsis thaliana (Mouse-ear cress)		Complete proteome; Cytoplasm; RNA-binding; Reference proteome; Ribonucleoprotein; Ribosomal protein; rRNA-binding
						PF00080.17^Sod_Cu^Copper/zinc superoxide dismutase [illegible]														Ipomoea batatas (Sweet potato) (Convolvulus [illegible]		

SRR924147_TR16878_c0_g1_i1_len=1173	SODC_IPOBA	Q:961-506,H:1-152	88.16%ID	E:2e-88	Superoxide dismutase [Cu-Zn]	PF00080.17^Sod_Cu^Copper/zinc superoxide dismutase (SODC)^12-148^E:1.2e-47	0	-0,878	0,054	0,476	0,327	0,299	0,594	0,172	0,838	0,087	SODC_IPOBA	Superoxide dismutase [Cu-Zn] (EC 1.15.1.1)	SODCC SOD-15	Ipomoea batatas (Sweet potato) (Convolvulus batatas)		Antioxidant; Copper; Cytoplasm; Disulfide bond; Metal-binding; Oxidoreductase; Zinc
SRR924147_TR19808_c0_g3_i1_len=1141	RS103_ARATH	Q:989-702,H:1-96	87.5%ID	E:6e-54	40S ribosomal protein S10-3	PF03501.12^S10_plectin^Plectin/S10 domain^3-94^E:5.7e-44	0	-0,988	0,298	-0,247	0,269	0,297	0,595	-0,147	0,054	-0,457	RS103_ARATH	40S ribosomal protein S10-3	RPS10C At5g52650 F6N7.14	Arabidopsis thaliana (Mouse-ear cress)		Complete proteome; Cytoplasm; Reference proteome; Ribonucleoprotein; Ribosomal protein
SRR924147_TR28614_c0_g1_i1_len=1629	RLA0_SOYBN	Q:1316-495,H:1-274	89.05%ID	E:1e-160	60S acidic ribosomal protein P0	PF00466.17^Ribosomal_L10^Ribosomal protein L10^7-108^E:9.1e-22`PF00428.16^Ribosomal_60s^60s Acidic ribosomal protein^234-320^E:6.5e-20	0	-0,999	0,897	-0,033	0,388	0,221	0,526	-0,166	0,306	-0,236	RLA0_SOYBN	60S acidic ribosomal protein P0		Glycine max (Soybean) (Glycine hispida)		Complete proteome; Phosphoprotein; Reference proteome; Ribonucleoprotein; Ribosomal protein
SRR924147_TR30907_c0_g1_i1_len=1169	RL6_MESCR	Q:141-833,H:7-234	81.82%ID	E:1e-114	60S ribosomal protein L6	PF03868.12^Ribosomal_L6e_N^Ribosomal protein L6, N-terminal domain^4-57^E:8.8e-15`PF01159.16^Ribosomal_L6e^Ribosomal protein L6e^127-234^E:9e-40	0	-1,117	0,313	-0,249	0,349	0,264	0,705	-0,132	0,053	-0,463	RL6_MESCR	60S ribosomal protein L6 (YL16-like)	RPL6	Mesembryanthemum crystallinum (Common ice plant) (Cryophytum crystallinum)		Ribonucleoprotein; Ribosomal protein
SRR924147_TR31811_c0_g1_i1_len=1268	RL131_ARATH	Q:1072-500,H:1-191	85.86%ID	E:3e-119	60S ribosomal protein L13-1	PF01294.15^Ribosomal_L13e^Ribosomal protein L13e^7-185^E:2.8e-78	0	-1,204	0,136	-0,355	0,172	0,361	0,635	0,134	0,894	-0,054	RL131_ARATH	60S ribosomal protein L13-1 (Protein BBC1 homolog)	RPL13B BBC1 At3g49010 T2J13.150	Arabidopsis thaliana (Mouse-ear cress)		Alternative splicing; Complete proteome; Cytoplasm; Reference proteome; Ribonucleoprotein; Ribosomal protein
SRR924147_TR34249_c0_g2_i3_len=1180	RL321_ARATH	Q:125-439,H:1-105	84.76%ID	E:3e-51	60S ribosomal protein L32-1	.	0,001	-1,253	0,671	-0,194	0,57	-0,39	0,471	-0,482	0,243	-0,353	RL321_ARATH	60S ribosomal protein L32-1	RPL32A At4g18100 F15J5.70	Arabidopsis thaliana (Mouse-ear cress)		Complete proteome; Reference proteome; Ribonucleoprotein; Ribosomal protein
SRR924147_TR36429_c0_g2_i1_len=1551	GBLP_NICPL	Q:1346-357,H:1-326	87.58%ID	E:0	Guanine nucleotide-binding protein subunit beta-like protein	PF00400.29^WD40^WD domain, G-beta repeat^10-44^E:0.017`PF00400.29^WD40^WD domain, G-beta repeat^56-92^E:2.1e-06`PF00400.29^WD40^WD domain, G-beta repeat^100-134^E:6.6e-07`PF00400.29^WD40^WD domain, G-beta repeat^141-180^E:1.2e-06`PF00400.29^WD40^WD domain, G-beta repeat^187-222^E:1.3e-06`PF00400.29^WD40^WD domain, G-beta repeat^235-262^E:0.00079`PF00400.29^WD40^WD domain, G-beta repeat^299-325^E:0.0015	0	-1,121	0,717	-0,093	0,516	0,205	0,915	-0,038	0,245	-0,303	GBLP_NICPL	Guanine nucleotide-binding protein subunit beta-like protein		Nicotiana plumbaginifolia (Leadwort-leaved tobacco) (Tex-Mex tobacco)		Repeat; WD repeat
SRR924148_TR5782_c0_g1_i1_len=1026	NA	NA	NA	NA	NA	PF02466.16^Tim17^Tim17/Tim22/Tim23/Pmp24 family^35-127^E:1e-09	0	-6,81	0,277	-0,503	0	2,002	0	1,85	1	-0,055	.	.	.	.	.	.
SRR924148_TR21202_c0_g1_i2_len=808	RL371_ARATH	Q:615-358,H:10-95	88.37%ID	E:3e-46	60S ribosomal protein L37-1	.	0,001	-0,871	0,016	-0,612	0,125	0,496	1	0,025	0,463	-0,289	RL371_ARATH	60S ribosomal protein L37-1	RPL37A At1g15250 F9L1.46	Arabidopsis thaliana (Mouse-ear cress)		Complete proteome; Metal-binding; RNA-binding; Reference proteome; Ribonucleoprotein; Ribosomal protein; Zinc; Zinc-finger; rRNA-binding
SRR924148_TR21543_c0_g1_i3_len=645	RS242_ARATH	Q:193-552,H:1-120	90.83%ID	E:1e-74	40S ribosomal protein S24-2	PF01282.16^Ribosomal_S24e^Ribosomal protein S24e^25-103^E:2.7e-38	0	-1,239	0,671	0,118	0,393	-0,275	0,493	-0,229	0,944	-0,021	RS242_ARATH	40S ribosomal protein S24-2	RPS24B At5g28060 F15F15.130	Arabidopsis thaliana (Mouse-ear cress)		Complete proteome; Reference proteome; Ribonucleoprotein; Ribosomal protein
SRR924148_TR25013_c0_g2_i1_len=711	RS11_EUPES	Q:205-627,H:1-141	85.82%ID	E:3e-72	40S ribosomal protein S11	PF16205.2^Ribosomal_S17_N^Ribosomal_S17 N-terminal^4-72^E:1.5e-32`PF00366.17^Ribosomal_S17^Ribosomal protein S17^74-130^E:1.3e-16	0	-2,615	0,187	1,104	1	0,277	0,226	-2,257	0,004	1,864	RS11_EUPES	40S ribosomal protein S11	RPS11	Euphorbia esula (Leafy spurge)		RNA-binding; Ribonucleoprotein; Ribosomal protein; rRNA-binding
SRR924148_TR30517_c0_g2_i1_len=721	RS30_ARATH	Q:560-375,H:1-62	96.77%ID	E:2e-24	40S ribosomal protein S30	.	0	-0,866	0,424	-0,186	0,189	0,326	0,895	0,043	0,214	-0,289	RS30_ARATH	40S ribosomal protein S30	RPS30A At2g19750 F6F22.22; RPS30B At4g29390 F17A13.210; RPS30C At5g56670 MIK19.12	Arabidopsis thaliana (Mouse-ear cress)		Complete proteome; Reference proteome; Ribonucleoprotein; Ribosomal protein
SRR924148_TR34426_c0_g2_i1_len=597	NA	NA	NA	NA	NA	.	0	-1,118	0,026	-0,487	0,014	0,586	0,29	0,257	0,117	-0,346	.	.	.	.	.	.
SRR924148_TR35408_c0_g1_i1_len=1138	SMD1_DROME	Q:342-614,H:1-91	71.43%ID	E:3e-38	Probable small nuclear ribonucleoprotein Sm D1	PF01423.19^LSM^LSM domain^5-69^E:1.5e-18	0,001	-6,558	0,034	-5,674	1	3,56	1	0	0,255	-0,688	SMD1_DROME	Probable small nuclear ribonucleoprotein Sm D1 (Sm-D1) (snRNP core protein D1)	SmD1 snRNP69D CG10753	Drosophila melanogaster (Fruit fly)		Complete proteome; Methylation; Nucleus; Reference proteome; Repeat; Ribonucleoprotein; mRNA processing; mRNA splicing
SRR924148_TR40686_c0_g1_i6_len=1183	RL74_ARATH	Q:1022-285,H:1-244	82.93%ID	E:4e-125	60S ribosomal protein L7-4	PF08079.9^Ribosomal_L30_N^Ribosomal L30 N-terminal domain^12-83^E:1e-25`PF00327.17^Ribosomal_L30^Ribosomal protein L30p/L7e^88-138^E:5e-22	0,001	-1,214	0,541	0,259	1	-0,08	0,648	0,22	1	0,937	RL74_ARATH	60S ribosomal protein L7-4	RPL7D At3g13580 K20M4.2	Arabidopsis thaliana (Mouse-ear cress)		Complete proteome; Reference proteome; Ribonucleoprotein; Ribosomal protein

Ms8h

Transcript (CDF97)	Uniprot homolog	BLASTX Position	BLASTX %Identity	BLASTX evalue	Uniprot descr	Pfam	PValue_Ms6h	logFC_Ms6h	PValue_Ms8h	logFC_Ms8h	PValue_MsDamaged24h	logFC_MsDamaged24h	PValue_MsIntact24h	logFC_MsIntact24h	PValue_Ms1wk	logFC_Ms1wk	Entry.name	Protein names	Gene names	Organism	Pathway	Keywords
SRR1144633_TR71_c0_g1_i1_len=802	NA	NA	NA	NA	NA	PF06596.8^PsbX^Photosystem II reaction centre X protein (PsbX)^89-126^E:1e-16	0,896	-0,03	0	-0,875	0,138	-0,317	0,335	-0,206	1	0,1	.	.	.	.	.	.
SRR1144633_TR6199_c0_g1_i1_len=745	RK29_ARATH	Q:621-169,H:1-158	67.72%ID	E:5e-57	50S ribosomal protein L29, chloroplastic	.	0,192	-0,334	0	-1,025	0,922	0,037	0,884	0,053	1	-0,123	RK29_ARATH	50S ribosomal protein L29, chloroplastic (CL29)	RPL29 At5g65220 MQN23.16	Arabidopsis thaliana (Mouse-ear cress)		Chloroplast; Complete proteome; Phosphoprotein; Plastid; Reference proteome; Ribonucleoprotein; Ribosomal protein; Transit peptide
SRR1144633_TR7251_c0_g1_i1_len=1269	CBSX3_ARATH	Q:221-994,H:1-206	64.48%ID	E:4e-109	CBS domain-containing protein CBSX3, mitochondrial	PF00571.25^CBS^CBS domain^34-67^E:0.00063`PF00571.25^CBS^CBS domain^79-134^E:1.9e-14	0,91	0,041	0,001	-1,009	0	1,34	0	1,347	1	0,86	CBSX3_ARATH	CBS domain-containing protein CBSX3, mitochondrial	CBSX3 At5g10860 T30N20.130	Arabidopsis thaliana (Mouse-ear cress)		CBS domain; Complete proteome; Direct protein sequencing; Mitochondrion; Reference proteome; Repeat; Transit peptide
SRR1144633_TR8042_c2_g2_i2_len=1124	ACT7_TOBAC	Q:245-949,H:2-233	65.97%ID	E:2e-98	Actin-104	PF00022.16^Actin^Actin^1-121^E:3.5e-27	1	-0,014	0	-1,495	1	-0,068	0,294	-0,53	1	1,498	ACT7_TOBAC	Actin-104 (Fragment)		Nicotiana tabacum (Common tobacco)		ATP-binding; Cytoplasm; Cytoskeleton; Nucleotide-binding
SRR1144633_TR20062_c0_g1_i1_len=1565	NA	NA	NA	NA	NA	.	0,634	-0,244	0	-1,709	1	-0,096	0,832	-0,321	0,196	-0,751	.	.	.	.	.	.
SRR1144633_TR20062_c0_g1_i9_len=1313	NA	NA	NA	NA	NA	.	0,783	-0,159	0	-1,892	0,44	-0,687	0,811	-0,313	0,748	-0,347	.	.	.	.	.	.
SRR1144633_TR20822_c0_g2_i1_len=643	PP178_ARATH	Q:641-168,H:729-886	75.95%ID	E:1e-80	Pentatricopeptide repeat-containing protein At2g31400, chloroplastic	PF01713.18^Smr^Smr domain^56-120^E:5.1e-05	0,608	0,241	0	-1,439	0,019	1,004	0,895	0,101	0,502	-4,275	PP178_ARATH	Pentatricopeptide repeat-containing protein At2g31400, chloroplastic	At2g31400 T28P16.11	Arabidopsis thaliana (Mouse-ear cress)		Chloroplast; Complete proteome; Plastid; Reference proteome; Repeat; Transit peptide
SRR1144633_TR22053_c0_g1_i3_len=1059	CYSP3_SOLLC	Q:129-698,H:167-356	87.89%ID	E:5e-118	Cysteine proteinase 3	PF00112.20^Peptidase_C1^Papain family cysteine protease^3-125^E:1.8e-36`PF03051.12^Peptidase_C1_2^Peptidase C1-like family^72-109^E:0.00023	1	-0,125	0	-1,832	0,025	2,931	1	0,281	0,025	-1,54	CYSP3_SOLLC	Cysteine proteinase 3 (EC 3.4.22.-)	CYP-3	Solanum lycopersicum (Tomato) (Lycopersicon esculentum)		Complete proteome; Disulfide bond; Glycoprotein; Hydrolase; Protease; Reference proteome; Signal; Thiol protease; Vacuole; Zymogen
SRR1144633_TR28278_c0_g1_i1_len=641	NA	NA	NA	NA	NA	PF10950.5^DUF2775^Protein of unknown function (DUF2775)^4-69^E:4.3e-18`PF10950.5^DUF2775^Protein of unknown function (DUF2775)^75-121^E:5.9e-14	0,007	0,631	0	-4,027	1	3,297	1	2,293	0,521	0,153	.	.	.	.	.	.
SRR1144633_TR28828_c3_g2_i4_len=1051	NA	NA	NA	NA	NA	.	1	-0,069	0,001	-2,246	0,692	0,714	1	0,073	0,902	-0,074	.	.	.	.	.	.
SRR1144633_TR32082_c0_g1_i1_len=1176	RK28_TOBAC	Q:999-550,H:2-151	68.42%ID	E:4e-61	50S ribosomal protein L28, chloroplastic	PF00830.16^Ribosomal_L28^Ribosomal L28 family^77-135^E:4e-21	0,076	-0,434	0	-0,86	0,959	-0,023	0,726	0,089	0,221	-0,575	RK28_TOBAC	50S ribosomal protein L28, chloroplastic (CL28)	RPL28	Nicotiana tabacum (Common tobacco)		Chloroplast; Direct protein sequencing; Plastid; Ribonucleoprotein; Ribosomal protein; Transit peptide
SRR1144633_TR34121_c0_g1_i1_len=568	TBB1_DAUCA	Q:568-431,H:245-290	97.83%ID	E:2e-23	Tubulin beta-1 chain	.	0,775	0,086	0	-1,826	0,326	0,551	0,326	0,54	1	0,664	TBB1_DAUCA	Tubulin beta-1 chain (Beta-1-tubulin) (Fragment)	TUBB1	Daucus carota (Wild carrot)		Cytoplasm; Cytoskeleton; Direct protein sequencing; GTP-binding; Microtubule; Nucleotide-binding
SRR1144633_TR34968_c0_g1_i1_len=701	NA	NA	NA	NA	NA	.	0,301	0,287	0	-0,935	0,194	-0,369	0,127	-0,425	1	0	.	.	.	.	.	.
SRR1144633_TR36679_c0_g1_i1_len=1014	PIP27_ARATH	Q:335-1012,H:1-223	86.73%ID	E:1e-135	Aquaporin PIP2-7	PF00230.17^MIP^Major intrinsic protein^32-226^E:2.2e-66	0,144	-0,412	0	-1,36	0,064	-0,562	0,964	-0,027	0,409	0,179	PIP27_ARATH	Aquaporin PIP2-7 (Plasma membrane intrinsic protein 2-7) (AtPIP2;7) (Plasma membrane intrinsic protein 3) (Salt stress-induced major intrinsic protein) [Cleaved into: Aquaporin PIP2-7, N-terminally processed]	PIP2-7 PIP3 SIMIP At4g35100 M4E13.150	Arabidopsis thaliana (Mouse-ear cress)		Acetylation; Cell membrane; Complete proteome; Membrane; Methylation; Phosphoprotein; Reference proteome; Repeat; Transmembrane; Transmembrane helix; Transport
SRR1144633_TR37801_c0_g1_i4_len=901	TRL31_ARATH	Q:215-763,H:26-209	54.01%ID	E:1e-64	Thioredoxin-like 3-1, chloroplastic	PF00085.17^Thioredoxin^Thioredoxin^79-168^E:5.1e-16	0,71	0,136	0	-1,075	0,692	0,601	1	-0,292	0,783	-0,201	TRL31_ARATH	Thioredoxin-like 3-1, chloroplastic (Thioredoxin WCRKC-1)	WCRKC1 At5g06690 MPH15.4	Arabidopsis thaliana (Mouse-ear cress)		Alternative splicing; Chloroplast; Complete proteome; Disulfide bond; Electron transport; Plastid; Redox-active center; Reference proteome; Transit peptide; Transport
SRR1144634_TR1966_c3_g2_i8_len=604	NA	NA	NA	NA	NA	.	1	0,449	0	-1,562	0,799	0,119	0,167	-0,595	1	-1,194	.	.	.	.	.	.
SRR1144634_TR3133_c0_g4_i1_len=667	NA	NA	NA	NA	NA	PF03080.12^DUF239^Domain of unknown function (DUF239)^3-99^E:8e-29	0,777	0,137	0	-1,814	0,559	-0,359	0,283	-0,589	1	-0,036	.	.	.	.	.	.
SRR1144634_TR6789_c0_g2_i3_len=692	NA	NA	NA	NA	NA	.	0,311	0,371	0	-1,577	0,443	0,422	0,035	-1,352	0,226	-0,471	.	.	.	.	.	.
SRR1144634_TR6921_c0_g1_i1_len=954	TGT2_ARATH	Q:697-389,H:388-490	80.58%ID	E:2e-48	Trihelix transcription factor GT-2	PF13837.3^Myb_DNA-bind_4^Myb/SANT-like DNA-binding domain^94-181^E:1.4e-23	0,325	-0,293	0	-1,615	0,073	-0,62	0,011	-0,883	1	1,177	TGT2_ARATH	Trihelix transcription factor GT-2 (Trihelix DNA-binding protein GT-2)	GT-2 At1g76890 F7O12.6	Arabidopsis thaliana (Mouse-ear cress)		Complete proteome; DNA-binding; Nucleus; Reference proteome; Repeat; Transcription; Transcription regulation
SRR1144634_TR13311_c0_g1_i1_len=603	KCS6_ARATH	Q:2-295,H:396-493	91.84%ID	E:2e-60	3-ketoacyl-CoA synthase 6 {ECO:0000303|PubMed:18465198}	PF08541.7^ACP_syn_III_C^3-Oxoacyl-[acyl-carrier-protein (ACP)] synthase III C terminal^2-72^E:9.6e-12`PF02797.12^Chal_sti_synt_C^Chalcone and stilbene synthases, C-terminal domain^2-66^E:3.8e-07	1	-0,321	0	-3,104	0,694	-0,35	0,86	0,226	0,054	-0,698	KCS6_ARATH	3-ketoacyl-CoA synthase 6 (KCS-6) (EC 2.3.1.199) (Cuticular protein 1) (Eceriferum 6) (Very long-chain fatty acid condensing enzyme 6) (VLCFA condensing enzyme 6)	CUT1 CER6 EL6 KCS6 At1g68530 T26J14.10	Arabidopsis thaliana (Mouse-ear cress)	PATHWAY: Lipid metabolism; fatty acid biosynthesis.	Acyltransferase; Alternative splicing; Complete proteome; Endoplasmic reticulum; Membrane; Reference proteome; Transferase; Transmembrane; Transmembrane helix; Ubl conjugation
SRR1144634_TR21610_c1_g4_i1_len=593	NA	NA	NA	NA	NA	PF01439.15^Metallothio_2^Metallothionein^40-116^E:4.1e-29	1	-0,001	0	-1,209	0,748	0,082	0,017	-0,592	0	0,989	.	.	.	.	.	.
SRR1144634_TR23213_c0_g2_i1_len=529	NA	NA	NA	NA	NA	.	1	0	0	-7,848	1	0	1	0	1	2,342	.	.	.	.	.	.
SRR1144634_TR25819_c0_g1_i1_len=979	NA	NA	NA	NA	NA	PF00301.17^Rubredoxin^Rubredoxin^73-107^E:2e-05	0,25	-0,415	0	-1,818	0,073	-0,623	0,17	-0,483	0,299	-1,663	.	.	.	.	.	.
SRR1144634_TR25968_c0_g1_i1_len=873	NA	NA	NA	NA	NA	.	1	0	0	-2,404	0,083	0,599	0,804	-0,122	1	0	.	.	.	.	.	.
SRR1144634_TR26415_c0_g1_i1_len=606	DCL_SOLLC	Q:8-361,H:97-214	88.98%ID	E:3e-71	Protein DCL, chloroplastic	PF11523.5^DUF3223^Protein of unknown function (DUF3223)^28-103^E:8.3e-29	0,048	-0,477	0	-1,119	0,798	-0,07	0,978	-0,019	0,899	-0,033	DCL_SOLLC	Protein DCL, chloroplastic (Defective chloroplasts and leaves protein)	DCL	Solanum lycopersicum (Tomato) (Lycopersicon esculentum)		Chloroplast; Complete proteome; Plastid; Reference proteome; Transit peptide
SRR1144634_TR26899_c0_g1_i1_len=1148	NA	NA	NA	NA	NA	PF14547.3^Hydrophob_seed^Hydrophobic seed protein^197-280^E:6.2e-27	0,016	-0,777	0	-4,899	1	-0,567	1	-0,861	0,388	-0,254	.	.	.	.	.	.
SRR1144634_TR27297_c0_g1_i3_len=2146	NA	NA	NA	NA	NA	PF14389.3^Lzipper-MIP1^Leucine-zipper of ternary complex factor MIP1^59-139^E:6.1e-21`PF04784.11^DUF547^Protein of unknown function, DUF547^373-502^E:9.3e-33	0,074	-0,913	0	-3,505	0,381	-0,716	0,258	-1,032	0,702	-0,159	.	.	.	.	.	.
SRR1144634_TR27837_c0_g4_i4_len=1093	SUT3_STYHA	Q:2-823,H:363-640	65.83%ID	E:8e-120	Low affinity sulfate transporter 3	PF00916.17^Sulfate_transp^Sulfate permease family^1-98^E:1.7e-32`PF01740.18^STAS^STAS domain^149-265^E:1e-28	0,021	-0,898	0	-1,795	0,316	0,301	0,232	0,359	1	1,376	SUT3_STYHA	Low affinity sulfate transporter 3	ST3	Stylosanthes hamata (Caribbean stylo)		Membrane; Transmembrane; Transmembrane helix; Transport
SRR1144634_TR34776_c0_g2_i4_len=2837	GUN8_ARATH	Q:1756-2835,H:128-487	79.44%ID	E:0	Endoglucanase 8	PF00759.16^Glyco_hydro_9^Glycosyl hydrolase family 9^2-328^E:5.7e-78	1	0	0	-7,179	1	0	1	0	1	0,351	GUN8_ARATH	Endoglucanase 8 (EC 3.2.1.4) (Cellulase 1) (AtCEL1) (Endo-1,4-beta glucanase 8)	CEL1 At1g70710 F5A18.11	Arabidopsis thaliana (Mouse-ear cress)		Carbohydrate metabolism; Cell wall biogenesis/degradation; Cellulose degradation; Complete proteome; Glycoprotein; Glycosidase; Hydrolase; Polysaccharide degradation; Reference proteome; Secreted; Signal
SRR1144634_TR34776_c0_g2_i5_len=2625	NC100_ARATH	Q:892-74,H:65-336	50.87%ID	E:3e-79	NAC domain-containing protein 100	PF02365.12^NAM^No apical meristem (NAM) protein^6-75^E:2.7e-16	0,129	3,864	0	-5,022	0,502	2,934	1	1,843	1	0	NC100_ARATH	NAC domain-containing protein 100 (ANAC100) (AtNAC5)	NAC100 At5g61430 MFB13.6	Arabidopsis thaliana (Mouse-ear cress)		Complete proteome; DNA-binding; Nucleus; Reference proteome; Transcription; Transcription regulation
SRR1144634_TR38220_c1_g1_i1_len=1001	UBC27_ARATH	Q:61-630,H:1-188	76.32%ID	E:1e-103	Ubiquitin-conjugating enzyme E2 27	PF00179.23^UQ_con^Ubiquitin-conjugating enzyme^26-164^E:1.1e-43`PF00627.28^UBA^UBA/TS-N domain^178-211^E:1.4e-08	0,781	0,397	0	-1,796	0,002	-1,58	0,054	-0,925	1	-0,243	UBC27_ARATH	Ubiquitin-conjugating enzyme E2 27 (EC 2.3.2.23) (E2 ubiquitin-conjugating enzyme 27) (Ubiquitin carrier protein 27)	UBC27 UBC1 At5g50870 K3K7.1	Arabidopsis thaliana (Mouse-ear cress)	PATHWAY: Protein modification; protein ubiquitination. {ECO:0000255|PROSITE-ProRule:PRU00388}.	ATP-binding; Alternative splicing; Complete proteome; Nucleotide-binding; Reference proteome; Transferase; Ubl conjugation pathway
SRR1144634_TR40497_c0_g2_i1_len=540	NA	NA	NA	NA	NA	.	0	6,674	0	-8,674	1	0	0,129	5,12	0,005	-6,183	.	.	.	.	.	.
SRR122236_TR8358_c0_g1_i1_len=648	NA	NA	NA	NA	NA	.	0,822	-0,354	0,001	-2,171	0,09	-0,693	0,012	-1,045	1	0,032	.	.	.	.	.	.
SRR122236_TR24865_c0_g1_i1_len=1153	TRL31_ARATH	Q:1091-654,H:26-172	48%ID	E:1e-40	Thioredoxin-like 3-1, chloroplastic	PF00085.17^Thioredoxin^Thioredoxin^99-158^E:2.3e-09	0,748	0,242	0	-1,86	1	2,816	1	2,463	1	-0,344	TRL31_ARATH	Thioredoxin-like 3-1, chloroplastic (Thioredoxin WCRKC-1)	WCRKC1 At5g06690 MPH15.4	Arabidopsis thaliana (Mouse-ear cress)		Alternative splicing; Chloroplast; Complete proteome; Disulfide bond; Electron transport; Plastid; Redox-active center; Reference proteome; Transit peptide; Transport
SRR122237_TR15744_c0_g2_i1_len=1054	NA	NA	NA	NA	NA	.	1	0	0	-6,867	0,129	5,145	1	0	1	0	.	.	.	.	.	.
SRR122237_TR20518_c0_g2_i2_len=1263	DV4H_CATRO	Q:1261-428,H:124-401	100%ID	E:0	Deacetoxyvindoline 4-hydroxylase	PF14226.3^DIOX_N^non-haem dioxygenase in morphine synthesis N-terminal^1-74^E:1.5e-06`PF03171.17^2OG-FeII_Oxy^2OG-Fe(II) oxygenase superfamily^126-216^E:5.3e-23	0,268	0,608	0,001	-1,53	0,505	0,499	0,86	0,238	1	0,197	DV4H_CATRO	Deacetoxyvindoline 4-hydroxylase (EC 1.14.11.20)	D4H	Catharanthus roseus (Madagascar periwinkle) (Vinca rosea)	PATHWAY: Alkaloid biosynthesis; vindoline biosynthesis; vindoline from tabersonine: step 5/6.	Cytoplasm; Dioxygenase; Direct protein sequencing; Iron; Metal-binding; Oxidoreductase; Vitamin C
SRR122237_TR26080_c0_g1_i2_len=1467	ALIS3_ARATH	Q:1296-304,H:23-346	64.65%ID	E:7e-157	ALA-interacting subunit 3	PF03381.12^CDC50^LEM3 (ligand-effect modulator 3) family / CDC50 family^60-329^E:4.2e-83	0,699	-0,17	0	-1,814	0,142	-0,586	0,114	-0,675	0,111	-0,898	ALIS3_ARATH	ALA-interacting subunit 3 (AtALIS3)	ALIS3 At1g54320 F20D21.14 F20D21_50	Arabidopsis thaliana (Mouse-ear cress)		Acetylation; Complete proteome; Endoplasmic reticulum; Glycoprotein; Golgi apparatus; Membrane; Reference proteome; Transmembrane; Transmembrane helix
SRR122238_TR1123_c0_g1_i1_len=1089	FKB13_ARATH	Q:729-292,H:67-208	72.79%ID	E:2e-62	Peptidyl-prolyl cis-trans isomerase FKBP13, chloroplastic {ECO:0000303|PubMed:12424338}	PF00254.25^FKBP_C^FKBP-type peptidyl-prolyl cis-trans isomerase^114-213^E:2e-29	0,681	-0,147	0	-1,255	0,777	-0,098	0,891	0,046	1	0,011	FKB13_ARATH	Peptidyl-prolyl cis-trans isomerase FKBP13, chloroplastic (PPIase FKBP13) (EC 5.2.1.8) (FK506-binding protein 1) (FK506-binding protein 13) (AtFKBP13) (Immunophilin FKBP13) (Rotamase)	FKBP13 FKBP22-1 FKBPK At5g45680 MRA19.7	Arabidopsis thaliana (Mouse-ear cress)		3D-structure; Chloroplast; Complete proteome; Direct protein sequencing; Disulfide bond; Isomerase; Plastid; Reference proteome; Rotamase; Thylakoid; Transit peptide
SRR122238_TR3107_c0_g1_i2_len=740	ACBP_RICCO	Q:520-266,H:1-85	88.24%ID	E:1e-46	Acyl-CoA-binding protein	.	0,169	-0,31	0	-0,884	0,447	-0,176	0,953	-0,016	0,571	-0,137	ACBP_RICCO	Acyl-CoA-binding protein (ACBP)		Ricinus communis (Castor bean)		Lipid-binding; Transport
SRR122238_TR3991_c1_g1_i2_len=1620	NA	NA	NA	NA	NA	PF10674.6^Ycf54^Protein of unknown function (DUF2488)^112-201^E:2.6e-34	0,009	-0,829	0,001	-1,16	0,188	-0,574	0,502	-0,277	0,84	-0,267	.	.	.	.	.	.
SRR122238_TR4540_c0_g1_i1_len=1091	HIS1B_ARATH	Q:1020-250,H:157-413	80.16%ID	E:8e-152	ATP phosphoribosyltransferase 2, chloroplastic	PF01634.15^HisG^ATP phosphoribosyltransferase^31-169^E:5.9e-30`PF08029.8^HisG_C^HisG, C-terminal domain^177-262^E:4.6e-18	0,793	-0,207	0	-1,871	0,748	0,3	0,235	-0,786	0,052	-0,666	HIS1B_ARATH	ATP phosphoribosyltransferase 2, chloroplastic (ATP-PRTase 2) (AtATP-PRT2) (EC 2.4.2.17)	HISN1B At1g09795 F21M12.39	Arabidopsis thaliana (Mouse-ear cress)	PATHWAY: Amino-acid biosynthesis; L-histidine biosynthesis; L-histidine from 5-phospho-alpha-D-ribose 1-diphosphate: step 1/9.	Amino-acid biosynthesis; Chloroplast; Complete proteome; Glycosyltransferase; Histidine biosynthesis; Plastid; Reference proteome; Transferase; Transit peptide
SRR122238_TR4978_c0_g1_i6_len=538	NA	NA	NA	NA	NA	.	1	2,7	0,001	-6,616	1	1,844	1	0	1	0	.	.	.	.	.	.
SRR122238_TR6003_c0_g1_i1_len=2567	ICR3_ARATH	Q:503-2332,H:7-564	44.25%ID	E:1e-119	Interactor of constitutive active ROPs 3	.	0,026	-0,92	0	-3,582	1	-3,7	1	-3,166	0,304	-0,332	ICR3_ARATH	Interactor of constitutive active ROPs 3 (ROP-interactive partner 5)	ICR3 RIP5 At5g60210 F15L12.15	Arabidopsis thaliana (Mouse-ear cress)		Coiled coil; Complete proteome; Phosphoprotein; Reference proteome
SRR122238_TR6101_c2_g1_i3_len=1789	FER_MASLA	Q:492-770,H:3-98	50%ID	E:1e-20	Ferredoxin	PF00111.24^Fer2^2Fe-2S iron-sulfur cluster binding domain^58-130^E:6.2e-13	0,537	0,307	0	-1,587	0,006	-1,206	0,002	-1,362	0,548	-0,213	FER_MASLA	Ferredoxin	petF	Mastigocladus laminosus (Fischerella sp.)		2Fe-2S; 3D-structure; Direct protein sequencing; Electron transport; Iron; Iron-sulfur; Metal-binding; Transport
SRR122238_TR7034_c0_g1_i1_len=529	ADF2_PETHY	Q:2-178,H:82-140	91.53%ID	E:2e-31	Actin-depolymerizing factor 2	.	0,144	0,465	0	-1,456	0,002	1,005	0,061	0,65	0,002	-0,972	ADF2_PETHY	Actin-depolymerizing factor 2 (ADF-2)	ADF2	Petunia hybrida (Petunia)		Actin-binding
SRR122238_TR9506_c0_g2_i1_len=604	PSAK_ARATH	Q:556-155,H:1-130	80.6%ID	E:8e-52	Photosystem I reaction center subunit psaK, chloroplastic	PF01241.15^PSI_PSAK^Photosystem I psaG / psaK^73-148^E:3.3e-24	0,407	0,384	0	-3,044	1	-0,005	0,009	0,602	0,024	-0,973	PSAK_ARATH	Photosystem I reaction center subunit psaK, chloroplastic (PSI-K) (Photosystem I subunit X)	PSAK At1g30380 T4K22.2	Arabidopsis thaliana (Mouse-ear cress)		Chloroplast; Complete proteome; Membrane; Photosynthesis; Photosystem I; Plastid; Reference proteome; Thylakoid; Transit peptide; Transmembrane; Transmembrane helix
SRR122238_TR11889_c0_g1_i1_len=935	RR20_ARATH	Q:774-262,H:5-186	57.69%ID	E:3e-30	30S ribosomal protein S20, chloroplastic	PF01649.15^Ribosomal_S20p^Ribosomal protein S20^73-159^E:2e-21	0,137	-0,344	0	-0,873	0,348	0,234	0,159	0,349	0,015	-0,572	RR20_ARATH	30S ribosomal protein S20, chloroplastic	RPS20 At3g15190 F4B12.10	Arabidopsis thaliana (Mouse-ear cress)		Chloroplast; Complete proteome; Plastid; RNA-binding; Reference proteome; Ribonucleoprotein; Ribosomal protein; Transit peptide; rRNA-binding
SRR122238_TR12566_c0_g1_i4_len=1541	NA	NA	NA	NA	NA	PF12204.5^DUF3598^Domain of unknown function (DUF3598)^83-404^E:6.1e-18	0,291	-0,331	0	-1,724	0,754	-0,149	0,77	0,162	1	0,754	.	.	.	.	.	.
SRR122238_TR12605_c0_g1_i3_len=3130	GCR2_ARATH	Q:293-1516,H:1-410	67.07%ID	E:0	LanC-like protein GCR2	PF08659.7^KR^KR domain^119-211^E:8.8e-09`PF00106.22^adh_short^short chain dehydrogenase^121-263^E:7.3e-21`PF13561.3^adh_short_C2^Enoyl-(Acyl carrier protein) reductase^125-262^E:1.7e-12	0,001	-0,748	0,001	-1,022	0,18	-0,796	0,08	-1,113	0	1,802	GCR2_ARATH	LanC-like protein GCR2 (G-protein coupled receptor 2)	GCR2 GPCR At1g52920 F14G24.19	Arabidopsis thaliana (Mouse-ear cress)		3D-structure; Abscisic acid signaling pathway; Complete proteome; Metal-binding; Reference proteome; Zinc
SRR122238_TR12605_c0_g1_i6_len=3195	POR_DAUCA	Q:3125-1848,H:1-398	79.39%ID	E:0	Protochlorophyllide reductase, chloroplastic	PF08659.7^KR^KR domain^137-229^E:1e-08`PF00106.22^adh_short^short chain dehydrogenase^139-281^E:8.6e-21`PF13561.3^adh_short_C2^Enoyl-(Acyl carrier protein) reductase^143-280^E:2e-12	0,024	-0,522	0	-2,773	0,357	-0,735	1	0,069	0,058	-0,493	POR_DAUCA	Protochlorophyllide reductase, chloroplastic (PCR) (EC 1.3.1.33) (NADPH-protochlorophyllide oxidoreductase) (POR)	POR1	Daucus carota (Wild carrot)	PATHWAY: Porphyrin-containing compound metabolism; chlorophyll biosynthesis.	Chlorophyll biosynthesis; Chloroplast; NADP; Oxidoreductase; Photosynthesis; Plastid; Transit peptide
SRR122238_TR12988_c1_g1_i1_len=827	NA	NA	NA	NA	NA	PF06549.9^DUF1118^Protein of unknown function (DUF1118)^67-178^E:9.1e-49	0,897	-0,034	0,001	-0,779	0,01	-0,578	0,025	-0,503	0,969	-0,013	.	.	.	.	.	.
SRR122238_TR14521_c0_g1_i1_len=898	RK17_TOBAC	Q:793-191,H:2-205	83.82%ID	E:2e-110	50S ribosomal protein L17, chloroplastic	PF01196.16^Ribosomal_L17^Ribosomal protein L17^110-206^E:2.9e-34	0,063	-0,427	0	-1,414	0,006	-0,683	0,087	-0,426	0,757	0,077	RK17_TOBAC	50S ribosomal protein L17, chloroplastic (CL17)	RPL17	Nicotiana tabacum (Common tobacco)		Chloroplast; Plastid; RNA-binding; Ribonucleoprotein; Ribosomal protein; Transit peptide; rRNA-binding
SRR122238_TR15114_c0_g1_i2_len=865	ADHL5_ARATH	Q:770-126,H:171-385	46.79%ID	E:6e-42	Alcohol dehydrogenase-like 5	PF01262.18^AlaDh_PNT_C^Alanine dehydrogenase/PNT, C-terminal domain^51-131^E:2.6e-05`PF00107.23^ADH_zinc_N^Zinc-binding dehydrogenase^76-196^E:3.1e-22	0,587	0,287	0,001	-2,496	0,641	0,283	0,111	-0,895	0,175	-0,996	ADHL5_ARATH	Alcohol dehydrogenase-like 5 (EC 1.1.1.1)	At4g22110 F1N20.210	Arabidopsis thaliana (Mouse-ear cress)		Complete proteome; Cytoplasm; Metal-binding; NAD; Oxidoreductase; Reference proteome; Zinc
SRR122238_TR15613_c0_g1_i3_len=921	TRXM4_ARATH	Q:647-288,H:70-191	59.84%ID	E:4e-46	Thioredoxin M4, chloroplastic	PF00085.17^Thioredoxin^Thioredoxin^110-209^E:3.2e-28	0,247	0,266	0	-1,168	0,826	-0,049	0,243	-0,255	0,357	-0,63	TRXM4_ARATH	Thioredoxin M4, chloroplastic (AtTrxm4)	At3g15360 MJK13.2	Arabidopsis thaliana (Mouse-ear cress)		Chloroplast; Complete proteome; Disulfide bond; Electron transport; Plastid; Redox-active center; Reference proteome; Transit peptide; Transport
SRR122238_TR16353_c0_g1_i3_len=756	NA	NA	NA	NA	NA	.	0,797	-0,091	0	-1,006	0,268	-0,262	0,457	-0,176	0,463	-0,196	.	.	.	.	.	.
SRR122238_TR16866_c0_g1_i3_len=1642	RK31_ARATH	Q:128-478,H:5-132	63.28%ID	E:1e-48	50S ribosomal protein L31, chloroplastic	PF01197.15^Ribosomal_L31^Ribosomal protein L31^38-102^E:5.1e-17	0,26	-0,307	0	-1,153	0,203	-0,393	0,224	-0,386	0,239	-0,307	RK31_ARATH	50S ribosomal protein L31, chloroplastic (CL31) (Protein EMBRYO DEFECTIVE 2184)	RPL31 EMB2184 At1g75350 F1B16.11	Arabidopsis thaliana (Mouse-ear cress)		Chloroplast; Complete proteome; Plastid; RNA-binding; Reference proteome; Ribonucleoprotein; Ribosomal protein; Transit peptide; rRNA-binding
SRR122238_TR18052_c0_g1_i2_len=655	NLTP_VIGUN	Q:495-253,H:20-99	57.32%ID	E:4e-23	Probable non-specific lipid-transfer protein AKCS9	.	0,005	0,685	0	-1,142	0,001	0,83	0,001	-0,902	0,003	0,719	NLTP_VIGUN	Probable non-specific lipid-transfer protein AKCS9 (LTP)		Vigna unguiculata (Cowpea)		Disulfide bond; Lipid-binding; Signal; Transport
SRR122238_TR21615_c1_g1_i1_len=1153	CB12_SOLLC	Q:79-885,H:1-268	89.96%ID	E:2e-177	Chlorophyll a-b binding protein 7, chloroplastic	PF00504.18^Chloroa_b-bind^Chlorophyll A-B binding protein^101-267^E:1.3e-51	0,794	-0,082	0	-1,844	0,004	-0,753	0,026	-0,575	0,18	0,28	CB12_SOLLC	Chlorophyll a-b binding protein 7, chloroplastic (LHCI type II CAB-7)	CAB7	Solanum lycopersicum (Tomato) (Lycopersicon esculentum)		Chlorophyll; Chloroplast; Chromophore; Complete proteome; Magnesium; Membrane; Metal-binding; Phosphoprotein; Photosynthesis; Photosystem I; Photosystem II; Plastid; Reference proteome; Thylakoid; Transit peptide; Transmembrane; Transmembrane helix
SRR122238_TR22755_c0_g1_i2_len=1107	VIT1_ARATH	Q:186-887,H:16-249	82.48%ID	E:3e-107	Vacuolar iron transporter 1	PF01988.16^VIT1^VIT family^24-234^E:6.7e-72	0,581	0,403	0,001	-1,924	0,166	-0,809	0,38	-0,56	0,231	0,527	VIT1_ARATH	Vacuolar iron transporter 1 (AtVIT1)	VIT1 At2g01770 T8O11.6	Arabidopsis thaliana (Mouse-ear cress)		Complete proteome; Ion transport; Iron; Iron transport; Membrane; Reference proteome; Transmembrane; Transmembrane helix; Transport; Vacuole
SRR122238_TR23190_c0_g1_i2_len=1752	Y2232_ARATH	Q:369-821,H:480-600	38.31%ID	E:3e-43	Probable receptor-like protein kinase At2g23200	PF07714.14^Pkinase_Tyr^Protein tyrosine kinase^2-156^E:9.7e-19`PF00069.22^Pkinase^Protein kinase domain^2-98^E:3.5e-17	0,16	-1,172	0	-3,147	1	-1,5	1	-0,378	0,006	-1,427	Y2232_ARATH	Probable receptor-like protein kinase At2g23200 (EC 2.7.11.-)	At2g23200 T20D16.17	Arabidopsis thaliana (Mouse-ear cress)		ATP-binding; Complete proteome; Glycoprotein; Kinase; Membrane; Nucleotide-binding; Reference proteome; Serine/threonine-protein kinase; Signal; Transferase; Transmembrane; Transmembrane helix
SRR122238_TR23838_c0_g1_i1_len=658	NA	NA	NA	NA	NA	.	0,568	-0,212	0	-2,624	0,454	0,322	0,075	-0,803	0,559	0,163	.	.	.	.	.	.
SRR122238_TR24757_c0_g1_i1_len=1343	STR11_ARATH	Q:243-1112,H:1-292	64.16%ID	E:2e-123	Rhodanese-like domain-containing protein 11, chloroplastic	PF00581.17^Rhodanese^Rhodanese-like domain^102-213^E:1.3e-07	0,004	-3,131	0	-3,473	1	0	1	0	1	0	STR11_ARATH	Rhodanese-like domain-containing protein 11, chloroplastic (Sulfurtransferase 11) (AtStr11)	STR11 At4g24750 F22K18.50	Arabidopsis thaliana (Mouse-ear cress)		Chloroplast; Complete proteome; Plastid; Reference proteome; Transit peptide
SRR122238_TR25448_c0_g2_i1_len=867	LOG3_ARATH	Q:142-747,H:13-214	81.68%ID	E:1e-120	Cytokinin riboside 5'-monophosphate phosphoribohydrolase LOG3	PF03641.11^Lysine_decarbox^Possible lysine decarboxylase^63-192^E:1.2e-43	0,803	-0,2	0	-2,099	0,053	-0,898	0,071	-0,864	0,033	-0,966	LOG3_ARATH	Cytokinin riboside 5'-monophosphate phosphoribohydrolase LOG3 (EC 3.2.2.n1) (Protein LONELY GUY 3)	LOG3 At2g37210 T2N18.3	Arabidopsis thaliana (Mouse-ear cress)		3D-structure; Alternative splicing; Complete proteome; Cytokinin biosynthesis; Cytoplasm; Hydrolase; Nucleus; Reference proteome
SRR122238_TR30157_c0_g1_i16_len=1035	PQL2_ARATH	Q:942-277,H:1-220	54.91%ID	E:1e-78	.	PF05757.8^PsbQ^Oxygen evolving enhancer protein 3 (PsbQ)^42-222^E:4.2e-53	0,69	0,111	0	-1,158	0,584	0,143	0,094	0,425	0,872	0,05	PNSL3_ARATH	Photosynthetic NDH subunit of lumenal location 3, chloroplastic (PsbQ-like protein 2)	PNSL3 PQL1 PQL2 At3g01440 T13O15.8	Arabidopsis thaliana (Mouse-ear cress)		Chloroplast; Complete proteome; Direct protein sequencing; Membrane; Plastid; Reference proteome; Thylakoid; Transit peptide; Transport
SRR122238_TR33734_c0_g1_i2_len=690	NA	NA	NA	NA	NA	.	1	0	0	-6,825	1	0	1	0	0,025	-1,505	.	.	.	.	.	.
SRR122238_TR36892_c0_g2_i1_len=2179	NOTUM_MOUSE	Q:398-1141,H:106-368	30.97%ID	E:2e-23	Palmitoleoyl-protein carboxylesterase NOTUM {ECO:0000250|UniProtKB:Q6P988}	PF03283.10^PAE^Pectinacetylesterase^54-402^E:1e-163	0,195	-0,857	0	-8,359	0,066	-3,514	0,066	-5,383	0,007	-1,483	NOTUM_MOUSE	Palmitoleoyl-protein carboxylesterase NOTUM (EC 3.1.1.-)	Notum	Mus musculus (Mouse)		Alternative splicing; Complete proteome; Glycoprotein; Hydrolase; Phosphoprotein; Reference proteome; Secreted; Serine esterase; Signal; Wnt signaling pathway
SRR122238_TR37628_c0_g1_i2_len=1600	PPL2_ARATH	Q:870-400,H:82-238	70.06%ID	E:4e-73	.	PF01789.13^PsbP^PsbP^64-224^E:9.7e-46	0,362	-0,295	0	-1,538	0,306	-0,419	0,931	-0,044	0,65	-0,166	PNSL1_ARATH	Photosynthetic NDH subunit of lumenal location 1, chloroplastic (PsbP-like protein 2)	PNSL1 PPL2 At2g39470 F12L6.13	Arabidopsis thaliana (Mouse-ear cress)		Alternative splicing; Chloroplast; Complete proteome; Membrane; Plastid; Reference proteome; Thylakoid; Transit peptide; Transport
SRR122238_TR38220_c0_g2_i1_len=3531	Y3804_ARATH	Q:3234-298,H:28-1012	63.7%ID	E:0	Probably inactive leucine-rich repeat receptor-like protein kinase At3g28040	PF08263.9^LRRNT_2^Leucine rich repeat N-terminal domain^35-74^E:5.7e-09`PF13855.3^LRR_8^Leucine rich repeat^127-186^E:3.1e-09`PF00560.30^LRR_1^Leucine Rich Repeat^128-148^E:0.42`PF13306.3^LRR_5^Leucine rich repeats (6 copies)^143-209^E:0.12`PF13855.3^LRR_8^Leucine rich repeat^199-260^E:2.1e-06`PF13516.3^LRR_6^Leucine Rich repeat^224-239^E:0.5`PF00560.30^LRR_1^Leucine Rich Repeat^225-241^E:0.44`PF13516.3^LRR_6^Leucine Rich repeat^271-287^E:1`PF13855.3^LRR_8^Leucine rich repeat^272-332^E:8.1e-08`PF13855.3^LRR_8^Leucine rich repeat^341-379^E:2.3e-07`PF00560.30^LRR_1^Leucine Rich Repeat^345-367^E:0.067`PF13855.3^LRR_8^Leucine rich repeat^393-451^E:5.7e-07`PF00560.30^LRR_1^Leucine Rich Repeat^441-454^E:1.5`PF00069.22^Pkinase^Protein kinase domain^726-994^E:2.9e-43`PF07714.14^Pkinase_Tyr^Protein tyrosine kinase^726-996^E:5.7e-38	0,18	-0,392	0	-2,511	0,068	-0,902	0,023	-1,092	0,423	0,266	Y3804_ARATH	Probably inactive leucine-rich repeat receptor-like protein kinase At3g28040	At3g28040 MMG15.21	Arabidopsis thaliana (Mouse-ear cress)		ATP-binding; Complete proteome; Glycoprotein; Leucine-rich repeat; Membrane; Nucleotide-binding; Phosphoprotein; Receptor; Reference proteome; Repeat; Signal; Transmembrane; Transmembrane helix
SRR122238_TR38655_c0_g1_i21_len=1532	P2C34_ARATH	Q:1403-342,H:1-350	70.42%ID	E:0	Probable protein phosphatase 2C 34	PF00481.18^PP2C^Protein phosphatase 2C^76-330^E:2.4e-41	1	3,257	0	-5,359	1	0	1	0	0,673	0,231	P2C34_ARATH	Probable protein phosphatase 2C 34 (AtPP2C34) (EC 3.1.3.16)	At3g05640 F18C1.9	Arabidopsis thaliana (Mouse-ear cress)		Complete proteome; Hydrolase; Magnesium; Manganese; Metal-binding; Protein phosphatase; Reference proteome
SRR122238_TR40196_c0_g1_i1_len=1181	NA	NA	NA	NA	NA	.	0,048	-0,898	0	-2,152	0,035	-1,455	0,004	-2,057	0,032	-0,867	.	.	.	.	.	.
SRR122238_TR41067_c0_g1_i7_len=828	BLC_CITFR	Q:92-547,H:26-169	38.71%ID	E:5e-22	Outer membrane lipoprotein Blc	PF08212.9^Lipocalin_2^Lipocalin-like domain^39-187^E:1.2e-52`PF00061.20^Lipocalin^Lipocalin / cytosolic fatty-acid binding protein family^45-187^E:2.2e-05	0,524	0,162	0,001	-0,942	0,006	0,681	0,023	0,566	0,154	0,34	BLC_CITFR	Outer membrane lipoprotein Blc		Citrobacter freundii		Cell outer membrane; Lipid-binding; Lipoprotein; Membrane; Palmitate; Signal
SRR122238_TR43565_c0_g1_i1_len=1067	RK21_SPIOL	Q:441-794,H:118-235	85.59%ID	E:7e-48	50S ribosomal protein L21, chloroplastic	PF00829.18^Ribosomal_L21p^Ribosomal prokaryotic L21 protein^98-198^E:2e-31	0,043	-0,494	0	-1,626	0,014	-0,63	0,016	-0,618	0,016	-0,563	RK21_SPIOL	50S ribosomal protein L21, chloroplastic (CL21) (CS-L7)	RPL21	Spinacia oleracea (Spinach)		3D-structure; Chloroplast; Direct protein sequencing; Plastid; RNA-binding; Ribonucleoprotein; Ribosomal protein; Transit peptide; rRNA-binding
SRR122238_TR43850_c0_g1_i1_len=733	NA	NA	NA	NA	NA	.	0,793	0,118	0	-1,904	0,023	-0,889	0,002	-1,239	0,402	1,128	.	.	.	.	.	.
SRR122238_TR43978_c0_g4_i1_len=1601	ATPG_TOBAC	Q:1453-320,H:1-376	85.45%ID	E:0	ATP synthase gamma chain, chloroplastic	PF00231.16^ATP-synt^ATP synthase^59-378^E:1.4e-86	0,716	-0,09	0	-0,873	0,013	-0,538	0,189	-0,286	0,83	0,124	ATPG_TOBAC	ATP synthase gamma chain, chloroplastic (F-ATPase gamma subunit)	ATPC	Nicotiana tabacum (Common tobacco)		ATP synthesis; CF(1); Chloroplast; Direct protein sequencing; Disulfide bond; Hydrogen ion transport; Ion transport; Membrane; Plastid; Thylakoid; Transit peptide; Transport
SRR122238_TR44116_c0_g1_i1_len=653	MIFH_BRUMA	Q:582-238,H:1-114	46.09%ID	E:3e-25	Macrophage migration inhibitory factor homolog	PF01187.15^MIF^Macrophage migration inhibitory factor (MIF)^25-138^E:3.1e-25	0,84	-0,074	0	-1,331	0	1,311	0	1,484	0,517	-0,783	MIFH_BRUMA	Macrophage migration inhibitory factor homolog (Bm-MIF-1) (BmMIF) (MIF) (EC 5.3.2.1) (L-dopachrome isomerase) (L-dopachrome tautomerase) (EC 5.3.3.12) (Phenylpyruvate tautomerase)	Bm1_28435 BMBAC01P19.1	Brugia malayi (Filarial nematode worm)		3D-structure; Complete proteome; Cytokine; Isomerase; Secreted
SRR122238_TR45476_c0_g1_i4_len=1633	NA	NA	NA	NA	NA	PF07207.8^Lir1^Light regulated protein Lir1^27-162^E:1.6e-41	0,117	0,352	0	-1,119	0	3,286	0	2,535	0,702	0,114	.	.	.	.	.	.
SRR122238_TR45825_c0_g1_i8_len=1131	BBD1_ARATH	Q:1045-260,H:59-320	77.1%ID	E:2e-139	Bifunctional nuclease 1	PF02577.11^DNase-RNase^Bifunctional nuclease^102-216^E:3.8e-22	0,183	0,668	0	-7,166	1	0	1	0	1	-1,474	BBD1_ARATH	Bifunctional nuclease 1 (AtBBD1) (EC 3.1.-.-)	BBD1 At1g75380 F1B16.9 F1B16_15	Arabidopsis thaliana (Mouse-ear cress)		Complete proteome; Hydrolase; Nuclease; Nucleus; Reference proteome
SRR122238_TR45959_c0_g1_i1_len=774	NA	NA	NA	NA	NA	PF04520.10^Senescence_reg^Senescence regulator^66-210^E:2.8e-40	0,01	-1,871	0,001	-6,58	0,811	-0,329	0,073	-2,143	0,729	0,202	.	.	.	.	.	.
SRR122238_TR46313_c0_g1_i1_len=1031	NA	NA	NA	NA	NA	PF00301.17^Rubredoxin^Rubredoxin^109-154^E:4e-17	0,589	-0,159	0	-1,414	0,06	-0,497	0,463	-0,197	0,138	-0,499	.	.	.	.	.	.
SRR122238_TR46403_c0_g2_i18_len=1456	NA	NA	NA	NA	NA	PF12906.4^RINGv^RING-variant domain^102-147^E:1.1e-10	0,902	-0,134	0	-1,839	1	-0,044	1	0,557	0,195	-0,554	.	.	.	.	.	.
SRR122238_TR48635_c0_g2_i1_len=710	RR17_ARATH	Q:115-537,H:10-147	67.38%ID	E:1e-38	30S ribosomal protein S17, chloroplastic	PF00366.17^Ribosomal_S17^Ribosomal protein S17^87-151^E:1e-24	0,039	-0,459	0	-1,286	0,007	-0,627	0,191	-0,3	0,035	-0,463	RR17_ARATH	30S ribosomal protein S17, chloroplastic (CS17)	RPS17 At1g79850 F19K16.19	Arabidopsis thaliana (Mouse-ear cress)		Chloroplast; Complete proteome; Plastid; RNA-binding; Reference proteome; Ribonucleoprotein; Ribosomal protein; Transit peptide; rRNA-binding
SRR122238_TR48696_c0_g2_i1_len=3798	NA	NA	NA	NA	NA	.	0,162	-0,441	0	-1,867	0,707	0,356	0,581	0,493	0,772	0,117	.	.	.	.	.	.
SRR122238_TR50728_c1_g1_i1_len=866	NA	NA	NA	NA	NA	PF00111.24^Fer2^2Fe-2S iron-sulfur cluster binding domain^143-191^E:1.3e-09	0,22	-0,401	0	-1,735	0,002	-1,223	0,013	-0,93	0	2,399	.	.	.	.	.	.
SRR122238_TR52826_c0_g1_i4_len=747	STEP1_ARATH	Q:628-215,H:1-141	56.03%ID	E:4e-24	Stress enhanced protein 1, chloroplastic	.	0,352	-0,3	0	-1,132	0,232	-0,307	0,586	-0,143	0,798	-0,27	STEP1_ARATH	Stress enhanced protein 1, chloroplastic	SEP1 At4g34190 F28A23.50	Arabidopsis thaliana (Mouse-ear cress)		Chloroplast; Complete proteome; Membrane; Photosynthesis; Photosystem II; Plastid; Reference proteome; Thylakoid; Transit peptide; Transmembrane; Transmembrane helix
SRR122238_TR53308_c1_g2_i1_len=973	21KD_DAUCA	Q:159-614,H:28-187	45.62%ID	E:5e-42	21 kDa protein	PF04043.12^PMEI^Plant invertase/pectin methylesterase inhibitor^52-199^E:5.5e-37	0,099	-0,794	0	-4,392	0,254	-2,953	0,254	-4,51	0,701	-0,134	21KD_DAUCA	21 kDa protein (1.2 protein)		Daucus carota (Wild carrot)		Signal
SRR122238_TR55057_c0_g1_i2_len=1030	PEXLP_TOBAC	Q:420-797,H:291-423	46.67%ID	E:1e-21	Pistil-specific extensin-like protein	PF01190.14^Pollen_Ole_e_I^Pollen proteins Ole e I like^140-229^E:7.4e-25	0,245	-0,368	0	-2,711	0,5	0,655	0,189	-1,543	0,127	-0,421	PEXLP_TOBAC	Pistil-specific extensin-like protein (PELP)		Nicotiana tabacum (Common tobacco)		Glycoprotein; Repeat; Signal
SRR122238_TR56145_c0_g2_i1_len=725	ML423_ARATH	Q:83-538,H:5-152	38.16%ID	E:2e-31	MLP-like protein 423	PF00407.16^Bet_v_1^Pathogenesis-related protein Bet v I family^28-178^E:2.5e-24	0,704	-0,094	0	-3,213	0,011	-0,988	0,002	-1,199	0,007	0,612	ML423_ARATH	MLP-like protein 423	MLP423 At1g24020 T23E23.17 T23E23_22	Arabidopsis thaliana (Mouse-ear cress)		Complete proteome; Reference proteome
SRR122238_TR57433_c0_g1_i2_len=1331	RK10_TOBAC	Q:1208-510,H:1-223	75.54%ID	E:3e-115	50S ribosomal protein L10, chloroplastic	PF00466.17^Ribosomal_L10^Ribosomal protein L10^61-157^E:1.4e-22	0,193	-0,352	0	-1,126	0,767	-0,113	0,35	-0,308	0,075	-0,753	RK10_TOBAC	50S ribosomal protein L10, chloroplastic (CL10)	RPL10	Nicotiana tabacum (Common tobacco)		Chloroplast; Plastid; RNA-binding; Ribonucleoprotein; Ribosomal protein; Transit peptide; rRNA-binding
SRR122238_TR58346_c1_g1_i4_len=1251	RSC5_DICDI	Q:140-820,H:53-286	28.39%ID	E:5e-27	Random slug protein 5	PF03765.12^CRAL_TRIO_N^CRAL/TRIO, N-terminal domain^39-63^E:7.3e-07`PF00650.17^CRAL_TRIO^CRAL/TRIO domain^96-237^E:2.5e-35`PF13716.3^CRAL_TRIO_2^Divergent CRAL/TRIO domain^101-245^E:1e-05	0,247	-0,32	0	-1,708	0,014	-0,821	0,001	-1,148	0,079	0,511	RSC5_DICDI	Random slug protein 5 (CRAL-TRIO domain-containing protein 5)	rsc5 DDB_G0269182	Dictyostelium discoideum (Slime mold)		Complete proteome; Reference proteome
SRR122238_TR58826_c0_g1_i1_len=1250	BASS3_ARATH	Q:1188-439,H:167-416	82.8%ID	E:1e-127	Probable sodium/metabolite cotransporter BASS3, chloroplastic	PF01758.13^SBF^Sodium Bile acid symporter family^13-167^E:4.8e-25`PF13593.3^SBF_like^SBF-like CPA transporter family (DUF4137)^21-228^E:2.4e-06	0,196	0,756	0	-1,782	0,76	0,463	1	0,09	1	-0,369	BASS3_ARATH	Probable sodium/metabolite cotransporter BASS3, chloroplastic (Bile acid transporter 3) (Bile acid-sodium symporter family protein 3)	BASS3 BAT3 At3g25410 MWL2.2	Arabidopsis thaliana (Mouse-ear cress)		Chloroplast; Complete proteome; Membrane; Plastid; Reference proteome; Transit peptide; Transmembrane; Transmembrane helix; Transport
SRR122238_TR59059_c1_g3_i2_len=1110	RK6_ARATH	Q:845-240,H:12-214	75.86%ID	E:1e-108	50S ribosomal protein L6, chloroplastic	PF00347.20^Ribosomal_L6^Ribosomal protein L6^57-129^E:1.4e-12`PF00347.20^Ribosomal_L6^Ribosomal protein L6^137-211^E:5.8e-20	0,094	-0,41	0	-1,24	0,019	-0,61	0,053	-0,498	0,808	-0,079	RK6_ARATH	50S ribosomal protein L6, chloroplastic (Protein EMBRYO DEFECTIVE 2394)	RPL6 EMB2394 At1g05190 YUP8H12.20	Arabidopsis thaliana (Mouse-ear cress)		Chloroplast; Complete proteome; Plastid; RNA-binding; Reference proteome; Ribonucleoprotein; Ribosomal protein; Transit peptide; rRNA-binding
SRR122238_TR59180_c0_g1_i2_len=881	ENL3_ARATH	Q:688-365,H:23-130	61.11%ID	E:8e-43	Early nodulin-like protein 3	PF02298.14^Cu_bind_like^Plastocyanin-like domain^42-127^E:3.5e-26	0,431	0,407	0	-7,036	0,017	-5,818	0,076	-2,55	0,151	-0,71	ENL3_ARATH	Early nodulin-like protein 3 (Phytocyanin-like protein)	At5g25090 T11H3_100	Arabidopsis thaliana (Mouse-ear cress)		Cell membrane; Complete proteome; GPI-anchor; Glycoprotein; Lipoprotein; Membrane; Reference proteome; Signal
SRR122238_TR59785_c0_g1_i3_len=1108	PQL1_ARATH	Q:769-389,H:63-189	64.57%ID	E:3e-54	.	PF05757.8^PsbQ^Oxygen evolving enhancer protein 3 (PsbQ)^115-237^E:1.3e-26	0,406	-0,258	0	-1,423	0,11	-0,421	0,481	-0,189	0,502	0,284	PNSL2_ARATH	Photosynthetic NDH subunit of lumenal location 2, chloroplastic (PsbQ-like protein 1)	PNSL2 PQL1 PQL2 At1g14150 F7A19.23	Arabidopsis thaliana (Mouse-ear cress)		Alternative splicing; Chloroplast; Coiled coil; Complete proteome; Direct protein sequencing; Membrane; Plastid; Reference proteome; Thylakoid; Transit peptide; Transport
SRR122238_TR59930_c0_g1_i11_len=1329	ECH2_ARATH	Q:1178-255,H:1-309	65.05%ID	E:8e-145	Enoyl-CoA hydratase 2, peroxisomal	PF01575.16^MaoC_dehydratas^MaoC like domain^191-292^E:3.6e-23	0,025	3,324	0	-7,113	1	0,272	1	-0,151	0,158	-1,484	ECH2_ARATH	Enoyl-CoA hydratase 2, peroxisomal (EC 4.2.1.119)	ECH2 At1g76150 T23E18.38 T23E18.9	Arabidopsis thaliana (Mouse-ear cress)	PATHWAY: Lipid metabolism; fatty acid beta-oxidation.	Complete proteome; Fatty acid metabolism; Lipid metabolism; Lyase; Oxidoreductase; Peroxisome; Reference proteome
SRR122238_TR62853_c0_g1_i4_len=562	NA	NA	NA	NA	NA	.	0,108	-0,418	0	-2,699	0,363	0,437	0,91	-0,063	0,81	0,098	.	.	.	.	.	.
SRR122238_TR62934_c0_g1_i1_len=920	RPD6A_ARATH	Q:650-414,H:61-139	94.94%ID	E:1e-45	DNA-directed RNA polymerases II, IV and V subunit 6A	.	0,691	0,236	0,001	-6,581	0,226	-2,836	0,066	-5,276	0,822	0,219	RPD6A_ARATH	DNA-directed RNA polymerases II, IV and V subunit 6A (RNA polymerase Rpb6)	NRPB6A NRPD6A NRPE6A At5g51940 MSG15.2	Arabidopsis thaliana (Mouse-ear cress)		Complete proteome; DNA-directed RNA polymerase; Nucleus; Reference proteome; Transcription

SRR122238_TR63692_c1_g1_i4_len=1181	TLPH_ARATH	Q:283-957,H:17-241	77.33%ID	E:2e-124	Thaumatin-like protein	PF00314.14^Thaumatin^Thaumatin family^35-247^E:7.3e-83	0,139	-0,598	0	-4,612	0,044	-1,992	0,319	-0,959	0,275	-0,269	TLPH_ARATH	Thaumatin-like protein	At1g18250 T10O22.21	Arabidopsis thaliana (Mouse-ear cress)		Alternative splicing; Complete proteome; Disulfide bond; Reference proteome; Signal
SRR122238_TR64653_c0_g1_i1_len=657	NUD26_ARATH	Q:71-487,H:78-216	71.22%ID	E:6e-59	Nudix hydrolase 26, chloroplastic	PF00293.25^NUDIX^NUDIX domain^1-115^E:3.2e-19	0,44	0,933	0	-7,541	1	0,344	0,017	-5,739	0,193	-0,739	NUD26_ARATH	Nudix hydrolase 26, chloroplastic (AtNUDT26) (EC 3.6.1.-) (Bis(5'-nucleosyl)-tetraphosphatase (asymmetrical))	NUDT26 NUDX26 At3g10620 F13M14.9 F18K10.24	Arabidopsis thaliana (Mouse-ear cress)		Chloroplast; Complete proteome; Hydrolase; Magnesium; Manganese; Metal-binding; Plastid; Reference proteome; Transit peptide
SRR122238_TR64963_c0_g1_i1_len=1061	NA	NA	NA	NA	NA	.	1	-0,1	0,001	-2,312	0,258	-1,086	0,258	-0,965	0,729	0,155	.	.	.	.	.	.
SRR122238_TR67122_c0_g1_i2_len=1057	NA	NA	NA	NA	NA	PF03745.11^DUF309^Domain of unknown function (DUF309)^84-140^E:3.1e-20	1	-0,044	0	-1,346	0,014	-1,427	0,121	-0,827	0,692	1,454	.	.	.	.	.	.
SRR122238_TR70112_c0_g3_i3_len=1778	PIN1C_ORYSJ	Q:997-707,H:492-588	91.75%ID	E:3e-47	Probable auxin efflux carrier component 1c	.	0,407	0,617	0	-2,683	0,316	-0,727	0,021	-1,647	0,832	-0,4	PIN1C_ORYSJ	Probable auxin efflux carrier component 1c (OsPIN1c)	PIN1C Os06g0232300 LOC_Os06g12610 P0479H10.14	Oryza sativa subsp. japonica (Rice)		Auxin signaling pathway; Complete proteome; Glycoprotein; Membrane; Reference proteome; Transmembrane; Transmembrane helix; Transport
SRR122238_TR71350_c2_g1_i5_len=545	FD6E1_SOYBN	Q:11-304,H:288-387	85%ID	E:2e-56	Omega-6 fatty acid desaturase, endoplasmic reticulum isozyme 1	PF00487.21^FA_desaturase^Fatty acid desaturase^13-65^E:6.8e-09	0,211	-0,74	0	-2,515	0,231	-0,585	0,684	-0,202	0,098	-0,479	FD6E1_SOYBN	Omega-6 fatty acid desaturase, endoplasmic reticulum isozyme 1 (EC 1.14.19.-)	FAD2-1	Glycine max (Soybean) (Glycine hispida)	PATHWAY: Lipid metabolism; polyunsaturated fatty acid biosynthesis.	Complete proteome; Endoplasmic reticulum; Fatty acid biosynthesis; Fatty acid metabolism; Lipid biosynthesis; Lipid metabolism; Membrane; Oxidoreductase; Reference proteome; Transmembrane; Transmembrane helix
SRR122238_TR71611_c0_g1_i1_len=1244	NA	NA	NA	NA	NA	.	0,298	0,686	0	-7,121	0,472	0,694	0,472	0,744	1	-0,004	.	.	.	.	.	.
SRR122238_TR73948_c0_g1_i10_len=2409	RK13_ARATH	Q:223-846,H:30-241	77.36%ID	E:3e-110	50S ribosomal protein L13, chloroplastic	PF00572.15^Ribosomal_L13^Ribosomal protein L13^103-225^E:2e-50	0,031	-0,555	0	-1,45	0,334	-0,281	0,409	-0,249	0,518	-0,159	RK13_ARATH	50S ribosomal protein L13, chloroplastic (CL13) (Protein EMBRYO DEFECTIVE 1473)	RPL13 EMB1473 At1g78630 T30F21.4	Arabidopsis thaliana (Mouse-ear cress)		Chloroplast; Complete proteome; Plastid; Reference proteome; Ribonucleoprotein; Ribosomal protein; Transit peptide
SRR122238_TR74069_c0_g1_i2_len=844	NA	NA	NA	NA	NA	PF05498.8^RALF^Rapid ALkalinization Factor (RALF)^72-131^E:7.4e-09	0,788	-0,071	0	-1,422	0,009	0,685	0,043	0,543	0,43	0,599	.	.	.	.	.	.
SRR122238_TR75102_c0_g1_i12_len=680	NA	NA	NA	NA	NA	PF01230.20^HIT^HIT domain^16-87^E:1.9e-17	0,454	-0,325	0	-1,837	0,443	-0,273	0,07	-0,637	0,258	-0,819	.	.	.	.	.	.
SRR122238_TR76038_c1_g2_i1_len=757	NA	NA	NA	NA	NA	.	0,124	-0,371	0	-1,268	0,195	-0,322	0,215	-0,311	0,18	-0,31	.	.	.	.	.	.
SRR122238_TR78180_c1_g1_i5_len=527	NA	NA	NA	NA	NA	.	0,017	-0,566	0	-1,789	0,002	0,864	0,025	0,635	0,003	0,665	.	.	.	.	.	.
SRR122238_TR78653_c0_g1_i8_len=1068	ASPG2_ARATH	Q:1018-188,H:193-470	32.39%ID	E:2e-22	Protein ASPARTIC PROTEASE IN GUARD CELL 2	PF14543.3^TAXi_N^Xylanase inhibitor N-terminal^9-105^E:5.8e-18`PF00026.20^Asp^Eukaryotic aspartyl protease^13-277^E:2.1e-11`PF14541.3^TAXi_C^Xylanase inhibitor C-terminal^128-274^E:1.5e-33	0,781	-0,136	0	-1,763	0,381	1,428	1	0,647	0,402	-1,097	ASPG2_ARATH	Protein ASPARTIC PROTEASE IN GUARD CELL 2 (AtASPG2) (EC 3.4.23.-)	ASPG2 At3g20015 MZE19.7	Arabidopsis thaliana (Mouse-ear cress)		Aspartyl protease; Complete proteome; DNA-binding; Disulfide bond; Hydrolase; Protease; Reference proteome; Signal
SRR122238_TR78693_c0_g1_i2_len=1407	CH62_CUCMA	Q:1019-372,H:350-565	92.13%ID	E:2e-131	Chaperonin CPN60-2, mitochondrial	PF00118.21^Cpn60_TCP1^TCP-1/cpn60 chaperonin family^2-209^E:8.4e-37	1	1,518	0	-2,844	1	0	1	0	0,01	-1,85	CH62_CUCMA	Chaperonin CPN60-2, mitochondrial (HSP60-2)	CPN60-2	Cucurbita maxima (Pumpkin) (Winter squash)		ATP-binding; Chaperone; Direct protein sequencing; Mitochondrion; Nucleotide-binding; Stress response; Transit peptide
SRR122238_TR79570_c0_g1_i1_len=966	HBL2_SOLLC	Q:216-641,H:1-142	77.46%ID	E:3e-78	Non-symbiotic hemoglobin 2	PF00042.19^Globin^Globin^8-116^E:2.2e-19	0,175	-0,335	0	-1,531	0,006	-0,654	0,004	-0,689	0,102	0,434	HBL2_SOLLC	Non-symbiotic hemoglobin 2 (Hb2) (SOLly GLB2)	HB2 GLB2	Solanum lycopersicum (Tomato) (Lycopersicon esculentum)		Complete proteome; Heme; Iron; Metal-binding; Reference proteome
SRR122238_TR79722_c0_g1_i2_len=436	Y1523_ARATH	Q:2-196,H:490-548	72.31%ID	E:4e-23	C-type lectin receptor-like tyrosine-protein kinase At1g52310	.	0,002	-1,466	0	-2,304	0,679	0,404	0,811	-0,242	0,211	-0,652	Y1523_ARATH	C-type lectin receptor-like tyrosine-protein kinase At1g52310 (EC 2.7.10.1)	At1g52310 F19K6.8	Arabidopsis thaliana (Mouse-ear cress)		ATP-binding; Cell membrane; Complete proteome; Disulfide bond; Glycoprotein; Kinase; Lectin; Membrane; Nucleotide-binding; Receptor; Reference proteome; Signal; Transferase; Transmembrane; Transmembrane helix; Tyrosine-protein kinase
SRR122238_TR82347_c1_g1_i1_len=787	NA	NA	NA	NA	NA	.	0,56	0,194	0	-4,015	0,588	-0,213	0,231	0,425	0,91	0,042	.	.	.	.	.	.
SRR122238_TR83592_c0_g2_i8_len=961	NA	NA	NA	NA	NA	.	0,455	0,206	0	-0,922	0,849	0,047	0,343	0,221	0,22	-0,301	.	.	.	.	.	.
SRR122238_TR83629_c0_g5_i2_len=731	PSAH1_ARATH	Q:610-242,H:1-123	81.3%ID	E:6e-51	Photosystem I reaction center subunit VI-1, chloroplastic	PF03244.11^PSI_PsaH^Photosystem I reaction centre subunit VI^7-145^E:1.9e-71	0,174	-0,31	0,001	-0,815	0,001	-0,724	0,203	-0,277	0,132	0,688	PSAH1_ARATH	Photosystem I reaction center subunit VI-1, chloroplastic (PSI-H1)	PSAH1 At3g16140 MSL1.18	Arabidopsis thaliana (Mouse-ear cress)		Chloroplast; Complete proteome; Membrane; Photosynthesis; Photosystem I; Plastid; Reference proteome; Thylakoid; Transit peptide; Transmembrane; Transmembrane helix
SRR122238_TR84806_c0_g1_i2_len=909	RR9_SPIOL	Q:104-670,H:9-197	73.06%ID	E:2e-90	30S ribosomal protein S9, chloroplastic	PF00380.16^Ribosomal_S9^Ribosomal protein S9/S16^102-223^E:2e-41	0,066	-0,428	0	-1,12	0,024	-0,565	0,085	-0,425	0,05	-0,439	RR9_SPIOL	30S ribosomal protein S9, chloroplastic (Fragment)	PRPS9	Spinacia oleracea (Spinach)		3D-structure; Acetylation; Chloroplast; Direct protein sequencing; Plastid; RNA-binding; Ribonucleoprotein; Ribosomal protein; Transit peptide; rRNA-binding
SRR122238_TR86788_c0_g1_i2_len=1186	RK18_ARATH	Q:1065-613,H:11-170	73.75%ID	E:2e-74	50S ribosomal protein L18, chloroplastic	PF02900.15^LigB^Catalytic LigB subunit of aromatic ring-opening dioxygenase^59-164^E:9e-05`PF00861.19^Ribosomal_L18p^Ribosomal L18 of archaea, bacteria, mitoch. and chloroplast^72-191^E:4.4e-47	0,057	-0,468	0	-1,464	0,046	-0,569	0,272	-0,312	0,18	0,832	RK18_ARATH	50S ribosomal protein L18, chloroplastic (CL18)	RPL18 At1g48350 F11A17.10	Arabidopsis thaliana (Mouse-ear cress)		Chloroplast; Complete proteome; Plastid; RNA-binding; Reference proteome; Ribonucleoprotein; Ribosomal protein; Transit peptide; rRNA-binding
SRR122238_TR86802_c0_g1_i7_len=1051	STR9_ARATH	Q:840-265,H:39-230	76.56%ID	E:2e-105	Rhodanese-like domain-containing protein 9, chloroplastic	.	0,34	-0,222	0,001	-0,81	0,002	-0,699	0,001	-0,733	0,254	0,278	STR9_ARATH	Rhodanese-like domain-containing protein 9, chloroplastic (Sulfurtransferase 9) (AtStr9)	STR9 At2g42220 T24P15.13	Arabidopsis thaliana (Mouse-ear cress)		Chloroplast; Complete proteome; Membrane; Plastid; Reference proteome; Transit peptide; Transmembrane; Transmembrane helix
SRR122238_TR87466_c1_g4_i15_len=5277	PHOT2_ARATH	Q:4710-3247,H:64-533	70.28%ID	E:0	Phototropin-2	PF13426.4^PAS_9^PAS domain^176-272^E:2.2e-19`PF00989.22^PAS^PAS fold^182-267^E:7.8e-09`PF08447.9^PAS_3^PAS fold^183-266^E:4.3e-08`PF13426.4^PAS_9^PAS domain^446-538^E:1e-19`PF00989.22^PAS^PAS fold^449-538^E:1.2e-06`PF08447.9^PAS_3^PAS fold^450-535^E:1.1e-05	0,878	-0,162	0,001	-2,348	0,002	-1,071	0,002	-1,058	1	0,007	PHOT2_ARATH	Phototropin-2 (EC 2.7.11.1) (Defective in chloroplast avoidance protein 1) (Non-phototropic hypocotyl 1-like protein 1) (AtKin7) (NPH1-like protein 1)	PHOT2 CAV1 KIN7 NPL1 At5g58140 K21L19.6	Arabidopsis thaliana (Mouse-ear cress)		3D-structure; ATP-binding; Alternative splicing; Cell membrane; Chromophore; Complete proteome; FMN; Flavoprotein; Kinase; Membrane; Nucleotide-binding; Phosphoprotein; Photoreceptor protein; Receptor; Reference proteome; Repeat; Sensory transduction; Serine/threonine-protein kinase; Transferase
SRR122238_TR89076_c0_g1_i4_len=1628	IOJAC_ARATH	Q:848-546,H:138-238	80.2%ID	E:5e-51	Protein lojap, chloroplastic	.	0,129	-0,446	0	-1,19	1	0,237	0,811	0,459	0,816	-0,183	IOJAC_ARATH	Protein lojap, chloroplastic	IJ At3g12930 MGH6.5	Arabidopsis thaliana (Mouse-ear cress)		Chloroplast; Complete proteome; Plastid; Reference proteome; Transit peptide
SRR122238_TR90209_c0_g3_i1_len=550	PSBW_ARATH	Q:480-169,H:19-119	62.86%ID	E:1e-22	Photosystem II reaction center W protein, chloroplastic	PF07123.9^PsbW^Photosystem II reaction centre W protein (PsbW)^7-141^E:4e-56	0,46	0,154	0	-1,116	0,024	-0,469	0,066	-0,379	0,931	-0,033	PSBW_ARATH	Photosystem II reaction center W protein, chloroplastic (PSII 6.1 kDa protein)	PSBW At2g30570 T6B20.8	Arabidopsis thaliana (Mouse-ear cress)		Chloroplast; Complete proteome; Membrane; Photosynthesis; Photosystem II; Plastid; Reference proteome; Thylakoid; Transit peptide; Transmembrane; Transmembrane helix
SRR1271857_TR3589_c0_g2_i10_len=2929	EGY2_ARATH	Q:1823-1020,H:206-473	79.85%ID	E:7e-123	Probable zinc metalloprotease EGY2, chloroplastic	.	0,811	-0,395	0	-2,839	1	2,217	1	1,816	1	-0,085	EGY2_ARATH	Probable zinc metalloprotease EGY2, chloroplastic (EC 3.4.24.-) (Protein ETHYLENE-DEPENDENT GRAVITROPISM-DEFICIENT AND YELLOW-GREEN 2)	EGY2 At5g05740 MJJ3.15	Arabidopsis thaliana (Mouse-ear cress)		Alternative splicing; Chloroplast; Complete proteome; Hydrolase; Membrane; Metalloprotease; Plastid; Protease; Reference proteome; Transit peptide; Transmembrane; Transmembrane helix
SRR1271857_TR3622_c0_g1_i1_len=1385	NA	NA	NA	NA	NA	.	0,064	-0,745	0	-2,529	0,226	-1,741	0,226	-1,999	0,392	0,363	.	.	.	.	.	.
SRR1271857_TR4997_c0_g2_i1_len=814	NA	NA	NA	NA	NA	.	0,448	-0,288	0	-1,482	0,938	0,044	0,758	0,134	0,385	-0,285	.	.	.	.	.	.
SRR1271857_TR5837_c0_g3_i6_len=709	NA	NA	NA	NA	NA	PF03473.14^MOSC^MOSC domain^60-162^E:6.6e-18	0,357	-0,712	0	-2,221	1	0,54	0,629	1,043	1	-0,299	.	.	.	.	.	.
SRR1271857_TR7192_c0_g1_i2_len=859	GTL2_ARATH	Q:656-63,H:296-475	39.22%ID	E:2e-22	Trihelix transcription factor GTL2	.	0,066	0,501	0	-1,312	0,534	0,466	1	-0,081	0,081	-0,457	GTL2_ARATH	Trihelix transcription factor GTL2 (GT2-LIKE protein 2) (AtGTL2) (Trihelix DNA-binding protein GTL2)	At5g28300 T8M17.70	Arabidopsis thaliana (Mouse-ear cress)		Coiled coil; Complete proteome; DNA-binding; Nucleus; Reference proteome; Repeat; Transcription; Transcription regulation
SRR1271857_TR7523_c1_g1_i1_len=633	NA	NA	NA	NA	NA	.	0,502	3,894	0	-7,492	1	2,698	1	0	1	-1,951	.	.	.	.	.	.
SRR1271857_TR9204_c0_g1_i6_len=4613	NA	NA	NA	NA	NA	.	0,111	-0,83	0	-3,264	0,068	-1,302	0,01	-1,911	0,76	0,363	.	.	.	.	.	.
SRR1271857_TR11267_c0_g3_i1_len=1912	CHLM_ARATH	Q:1659-823,H:44-311	75.63%ID	E:7e-140	Magnesium protoporphyrin IX methyltransferase, chloroplastic	PF13489.3^Methyltransf_23^Methyltransferase domain^189-343^E:3.9e-08`PF13847.3^Methyltransf_31^Methyltransferase domain^189-276^E:1.2e-06`PF13649.3^Methyltransf_25^Methyltransferase domain^193-289^E:1.3e-11`PF08242.9^Methyltransf_12^Methyltransferase domain^195-288^E:1.3e-07`PF08241.9^Methyltransf_11^Methyltransferase domain^195-275^E:5.6e-06`PF07109.8^Mg-por_mtran_C^Magnesium-protoporphyrin IX methyltransferase C-terminus^267-362^E:5.7e-35	0,077	-0,525	0	-1,266	0,018	-0,846	0,064	-0,654	0,261	0,309	CHLM_ARATH	Magnesium protoporphyrin IX methyltransferase, chloroplastic (EC 2.1.1.11)	CHLM At4g25080 F13M23.220	Arabidopsis thaliana (Mouse-ear cress)	PATHWAY: Porphyrin-containing compound metabolism; chlorophyll biosynthesis.	Alternative splicing; Chlorophyll biosynthesis; Chloroplast; Complete proteome; Membrane; Methyltransferase; Plastid; Reference proteome; S-adenosyl-L-methionine; Thylakoid; Transferase; Transit peptide
SRR1271857_TR14295_c0_g1_i3_len=2009	RK4_TOBAC	Q:713-1411,H:25-253	79.83%ID	E:3e-124	50S ribosomal protein L4, chloroplastic	PF00573.19^Ribosomal_L4^Ribosomal protein L4/L1 family^80-260^E:4.1e-52	0,046	-0,499	0	-1,315	0,008	-0,756	0,052	-0,536	0,046	-0,488	RK4_TOBAC	50S ribosomal protein L4, chloroplastic (CL4) (R-protein L4)	RPL4	Nicotiana tabacum (Common tobacco)		Chloroplast; Plastid; RNA-binding; Ribonucleoprotein; Ribosomal protein; Transit peptide; rRNA-binding
SRR1271857_TR15387_c0_g3_i1_len=2124	PHYLO_ARATH	Q:1612-305,H:1277-1712	50.57%ID	E:1e-138	Protein PHYLLO, chloroplastic	PF02230.13^Abhydrolase_2^Phospholipase/Carboxylesterase^123-238^E:3.1e-07`PF12146.5^Hydrolase_4^Serine aminopeptidase, S33^130-379^E:1.8e-12`PF00561.17^Abhydrolase_1^alpha/beta hydrolase fold^131-247^E:2.5e-18`PF12697.4^Abhydrolase_6^Alpha/beta hydrolase family^133-385^E:5.1e-22`PF03096.11^Ndr^Ndr family^150-392^E:2.6e-08`PF07859.10^Abhydrolase_3^alpha/beta hydrolase fold^182-305^E:3.2e-05`PF08840.8^BAAT_C^BAAT / Acyl-CoA thioester hydrolase C terminal^200-376^E:4.3e-05	0,439	-0,349	0	-1,84	0,452	-0,519	0,719	-0,374	0,461	-0,88	PHYLO_ARATH	Protein PHYLLO, chloroplastic [Includes: Inactive isochorismate synthase (MENF); 2-succinyl-5-enolpyruvyl-6-hydroxy-3-cyclohexene-1-carboxylate synthase (EC 2.2.1.9) (MEND); o-succinylbenzoate synthase (EC 4.2.1.113) (MENC); 2-succinyl-6-hydroxy-2,4-cyclohexadiene-1-carboxylate synthase (EC 4.2.99.20) (MENH)]	PHYLLO At1g68890/At1g68900 T6L1.7/T6L1.8	Arabidopsis thaliana (Mouse-ear cress)		Alternative splicing; Chloroplast; Complete proteome; Lyase; Magnesium; Manganese; Membrane; Metal-binding; Multifunctional enzyme; Plastid; Reference proteome; Thiamine pyrophosphate; Transferase; Transit peptide; Transmembrane; Transmembrane helix
SRR1271857_TR17006_c0_g1_i1_len=646	ATL66_ARATH	Q:79-522,H:9-156	50%ID	E:3e-36	RING-H2 finger protein ATL66	PF12861.4^zf-ANAPC11^Anaphase-promoting complex subunit 11 RING-H2 finger^100-149^E:5.5e-05`PF13639.3^zf-RING_2^Ring finger domain^101-144^E:1.4e-14`PF17123.2^zf-RING_11^RING-like zinc finger^101-130^E:3.1e-09`PF13923.3^zf-C3HC4_2^Zinc finger, C3HC4 type (RING finger)^101-143^E:6.8e-08`PF13445.3^zf-RING_UBOX^RING-type zinc-finger^102-141^E:2.9e-05`PF00097.22^zf-C3HC4^Zinc finger, C3HC4 type (RING finger)^102-143^E:3.3e-05`PF12678.4^zf-rbx1^RING-H2 zinc finger domain^109-144^E:2.9e-08	0,593	-0,673	0	-3,602	1	0,903	1	0,849	0,123	0,715	ATL66_ARATH	RING-H2 finger protein ATL66	ATL66 At3g11110 F11B9.7 F9F8.8	Arabidopsis thaliana (Mouse-ear cress)	PATHWAY: Protein modification; protein ubiquitination.	Complete proteome; Membrane; Metal-binding; Reference proteome; Transmembrane; Transmembrane helix; Ubl conjugation pathway; Zinc; Zinc-finger
SRR1271857_TR17959_c0_g1_i6_len=2474	NA	NA	NA	NA	NA	.	0,609	-0,286	0	-1,778	0,564	-0,427	0,43	-0,699	1	-0,065	.	.	.	.	.	.
SRR1271857_TR18439_c1_g1_i7_len=2302	NA	NA	NA	NA	NA	PF14215.3^bHLH-MYC_N^bHLH-MYC and R2R3-MYB transcription factors N-terminal^13-136^E:2.7e-14	0,524	-0,561	0,001	-3,427	0,025	-3,884	0,005	-4,609	0,066	5,337	.	.	.	.	.	.
SRR1271857_TR18456_c0_g1_i1_len=1216	RK3_TOBAC	Q:242-997,H:8-259	82.42%ID	E:2e-122	50S ribosomal protein L3, chloroplastic	PF00297.19^Ribosomal_L3^Ribosomal protein L3^158-245^E:1.9e-17	0,202	-0,29	0	-1,302	0,067	-0,432	0,27	-0,258	0,937	-0,024	RK3_TOBAC	50S ribosomal protein L3, chloroplastic (Fragment)	RPL3	Nicotiana tabacum (Common tobacco)		Chloroplast; Plastid; RNA-binding; Ribonucleoprotein; Ribosomal protein; Transit peptide; rRNA-binding
SRR1271857_TR20290_c2_g7_i1_len=1820	NA	NA	NA	NA	NA	.	1	-0,074	0	-1,969	0,111	-0,87	0,077	-1,014	0,446	-0,311	.	.	.	.	.	.
SRR1271857_TR21102_c0_g1_i2_len=1671	NA	NA	NA	NA	NA	PF01738.15^DLH^Dienelactone hydrolase family^5-150^E:8e-22`PF08386.7^Abhydrolase_4^TAP-like protein^78-128^E:6.5e-08	1	-0,069	0	-2,5	1	2,164	1	0	0,798	0,153	.	.	.	.	.	.
SRR1271857_TR21977_c0_g1_i2_len=808	NA	NA	NA	NA	NA	.	0,732	-0,83	0	-2,639	1	-0,725	1	-1,056	0,258	-1,046	.	.	.	.	.	.
SRR1271857_TR23215_c0_g1_i7_len=1850	RR1_SPIOL	Q:505-1071,H:165-335	30.16%ID	E:2e-22	30S ribosomal protein S1, chloroplastic	PF00575.20^S1^S1 RNA binding domain^193-269^E:1.2e-16	1	-0,095	0,001	-1,928	0,262	-0,54	0,673	-0,252	0,472	-0,356	RR1_SPIOL	30S ribosomal protein S1, chloroplastic (CS1)	RPS1	Spinacia oleracea (Spinach)		Chloroplast; Direct protein sequencing; Plastid; RNA-binding; Repeat; Ribonucleoprotein; Ribosomal protein; Transit peptide
SRR1271857_TR24843_c0_g1_i2_len=971	SYGM1_ARATH	Q:884-177,H:489-724	78.39%ID	E:5e-117	Glycine--tRNA ligase, mitochondrial 1 {ECO:0000305}	PF03129.17^HGTP_anticodon^Anticodon binding domain^107-194^E:4.4e-21	0,903	0,047	0	-1,111	0,381	0,662	1	-0,067	0,732	-0,625	SYGM1_ARATH	Glycine--tRNA ligase, mitochondrial 1 (EC 6.1.1.14) (Diadenosine tetraphosphate synthetase) (AP-4-A synthetase) (Glycyl-tRNA synthetase 1) (GlyRS-1)	At1g29880 F1N18.8	Arabidopsis thaliana (Mouse-ear cress)		ATP-binding; Acetylation; Alternative initiation; Aminoacyl-tRNA synthetase; Complete proteome; Cytoplasm; Ligase; Mitochondrion; Nucleotide-binding; Protein biosynthesis; Reference proteome; Transit peptide
SRR1271857_TR25567_c0_g2_i6_len=1798	SGAT_ARATH	Q:383-1582,H:1-400	89%ID	E:0	Serine--glyoxylate aminotransferase	PF00266.16^Aminotran_5^Aminotransferase class-V^12-325^E:7.5e-33	0,342	-0,313	0	-1,324	0,637	-0,109	0,797	0,058	0,522	0,144	SGAT_ARATH	Serine--glyoxylate aminotransferase (EC 2.6.1.45) (Alanine--glyoxylate aminotransferase) (AGT) (EC 2.6.1.44) (Asparagine aminotransferase) (EC 2.6.1.-) (Serine--pyruvate aminotransferase) (EC 2.6.1.51)	AGT1 At2g13360 F14O4.7	Arabidopsis thaliana (Mouse-ear cress)		Acetylation; Aminotransferase; Complete proteome; Peroxisome; Phosphoprotein; Photorespiration; Pyridoxal phosphate; Reference proteome; Transferase
SRR1271857_TR26789_c0_g2_i2_len=1685	XTH32_ARATH	Q:120-908,H:37-299	82.89%ID	E:2e-163	Probable xyloglucan endotransglucosylase/hydrolase protein 32	PF00722.18^Glyco_hydro_16^Glycosyl hydrolases family 16^48-229^E:2e-53`PF06955.9^XET_C^Xyloglucan endo-transglycosylase (XET) C-terminus^264-300^E:5.9e-11	0,763	-0,206	0	-2,297	1	0,187	1	-1,328	0,001	1,647	XTH32_ARATH	Probable xyloglucan endotransglucosylase/hydrolase protein 32 (At-XTH32) (XTH-32) (EC 2.4.1.207)	XTH32 At2g36870 T1J8.5	Arabidopsis thaliana (Mouse-ear cress)		Alternative splicing; Apoplast; Cell wall; Cell wall biogenesis/degradation; Complete proteome; Disulfide bond; Glycosidase; Hydrolase; Reference proteome; Secreted; Signal; Transferase
SRR1271857_TR28513_c0_g1_i1_len=951	NA	NA	NA	NA	NA	.	0,115	-0,769	0	-2,86	0,132	1,295	0,132	1,281	0,079	-0,452	.	.	.	.	.	.
SRR1271857_TR31850_c0_g2_i11_len=2063	NA	NA	NA	NA	NA	PF13540.3^RCC1_2^Regulator of chromosome condensation (RCC1) repeat^36-65^E:4.4e-09`PF00415.15^RCC1^Regulator of chromosome condensation (RCC1) repeat^115-157^E:4.4e-06`PF00415.15^RCC1^Regulator of chromosome condensation (RCC1) repeat^163-209^E:1.9e-10	0,564	-0,288	0,001	-2,055	0,156	-0,584	0,226	-0,472	0,385	-0,342	.	.	.	.	.	.
SRR1271857_TR32133_c0_g1_i1_len=991	FTRV_MAIZE	Q:573-331,H:17-96	58.02%ID	E:3e-25	Ferredoxin-thioredoxin reductase, variable chain	PF02941.12^FeThRed_A^Ferredoxin thioredoxin reductase variable alpha chain^142-215^E:9.9e-24	0,186	-0,327	0	-1,104	0,357	-0,245	1	0	0,211	0,61	FTRV_MAIZE	Ferredoxin-thioredoxin reductase, variable chain (FTR-V) (Ferredoxin-thioredoxin reductase subunit A) (FTR-A)		Zea mays (Maize)		Chloroplast; Complete proteome; Direct protein sequencing; Oxidoreductase; Plastid; Reference proteome
SRR1271857_TR32830_c0_g8_i6_len=580	ROC1_NICPL	Q:31-264,H:201-279	86.08%ID	E:1e-37	30 kDa ribonucleoprotein, chloroplastic	.	0,227	-0,346	0,001	-1,007	0,377	0,571	0,079	1,01	1	0,592	ROC1_NICPL	30 kDa ribonucleoprotein, chloroplastic (CP-RBP30)		Nicotiana plumbaginifolia (Leadwort-leaved tobacco) (Tex-Mex tobacco)		Chloroplast; Plastid; RNA-binding; Repeat; Ribonucleoprotein; Transit peptide; mRNA processing
SRR1271857_TR33180_c0_g1_i2_len=1085	RR10_MESCR	Q:478-837,H:78-197	87.5%ID	E:1e-71	30S ribosomal protein S10, chloroplastic	.	0,046	-0,478	0	-1,197	0,124	-0,391	0,467	-0,188	0	7,485	RR10_MESCR	30S ribosomal protein S10, chloroplastic	RPS10	Mesembryanthemum crystallinum (Common ice plant) (Cryophytum crystallinum)		Chloroplast; Plastid; Ribonucleoprotein; Ribosomal protein; Transit peptide
SRR1271857_TR33534_c0_g1_i5_len=3589	NA	NA	NA	NA	NA	PF13516.3^LRR_6^Leucine Rich repeat^132-152^E:0.15`PF13516.3^LRR_6^Leucine Rich repeat^155-178^E:0.016`PF13516.3^LRR_6^Leucine Rich repeat^182-203^E:0.13`PF13516.3^LRR_6^Leucine Rich repeat^230-251^E:0.057`PF13855.3^LRR_8^Leucine rich repeat^231-290^E:4.4e-07	1	0,02	0	-4,543	0,028	1,458	0,112	1,071	0,076	2,629	.	.	.	.	.	.
SRR1271857_TR33753_c1_g1_i5_len=2124	SYS_HELAN	Q:370-1665,H:1-432	86.81%ID	E:0	Serine--tRNA ligase	PF02403.19^Seryl_tRNA_N^Seryl-tRNA synthetase N-terminal domain^1-112^E:1.1e-26`PF00587.22^tRNA-synt_2b^tRNA synthetase class II core domain (G, H, P, S and T)^221-407^E:1.6e-35	0,132	-0,69	0	-7,212	0,066	-5,389	0,066	-5,389	1	-0,022	SYS_HELAN	Serine--tRNA ligase (EC 6.1.1.11) (Seryl-tRNA synthetase) (SerRS) (Seryl-tRNA(Ser/Sec) synthetase)		Helianthus annuus (Common sunflower)	PATHWAY: Aminoacyl-tRNA biosynthesis; selenocysteinyl-tRNA(Sec) biosynthesis; L-seryl-tRNA(Sec) from L-serine and tRNA(Sec): step 1/1.	ATP-binding; Aminoacyl-tRNA synthetase; Ligase; Nucleotide-binding; Protein biosynthesis
SRR1271857_TR34511_c0_g1_i9_len=2146	ALGC_PSESM	Q:552-1670,H:53-395	28.88%ID	E:8e-36	Phosphomannomutase/phosphoglucomutase	PF02878.13^PGM_PMM_I^Phosphoglucomutase/phosphomannomutase, alpha/beta/alpha domain I^77-228^E:4.4e-28`PF02879.13^PGM_PMM_II^Phosphoglucomutase/phosphomannomutase, alpha/beta/alpha domain II^251-359^E:6.8e-21`PF02880.13^PGM_PMM_III^Phosphoglucomutase/phosphomannomutase, alpha/beta/alpha domain III^363-463^E:1.1e-12	0,856	0,101	0	-1,213	1	-0,071	1	-0,079	0,33	-0,369	ALGC_PSESM	Phosphomannomutase/phosphoglucomutase (PMM / PGM) (EC 5.4.2.2) (EC 5.4.2.8)	algC PSPTO_0083	Pseudomonas syringae pv. tomato (strain DC3000)	PATHWAY: Nucleotide-sugar biosynthesis; GDP-alpha-D-mannose biosynthesis; alpha-D-mannose 1-phosphate from D-fructose 6-phosphate: step 2/2.; PATHWAY: Bacterial outer membrane biogenesis; lipopolysaccharide biosynthesis.	Alginate biosynthesis; Complete proteome; Isomerase; Lipopolysaccharide biosynthesis; Magnesium; Metal-binding; Multifunctional enzyme; Phosphoprotein; Reference proteome
SRR1271857_TR34790_c0_g2_i2_len=1279	NA	NA	NA	NA	NA	.	0,663	-0,511	0	-2,387	0,073	2,228	0,118	2,115	1	-0,327	.	.	.	.	.	.
SRR1271857_TR35199_c3_g5_i5_len=2487	NA	NA	NA	NA	NA	PF03168.10^LEA_2^Late embryogenesis abundant protein^97-197^E:2.1e-05	0,832	-0,246	0	-3,434	1	0,58	1	0,898	0,021	-1,281	.	.	.	.	.	.
SRR1271857_TR36568_c0_g1_i2_len=1176	PIP27_ARATH	Q:1057-209,H:1-280	87.28%ID	E:2e-176	Aquaporin PIP2-7	PF00230.17^MIP^Major intrinsic protein^71-300^E:1.3e-82	0,056	-0,407	0	-2,111	0,031	-0,463	0,19	-0,28	0,379	-0,209	PIP27_ARATH	Aquaporin PIP2-7 (Plasma membrane intrinsic protein 2-7) (AtPIP2;7) (Plasma membrane intrinsic protein 3) (Salt stress-induced major intrinsic protein) [Cleaved into: Aquaporin PIP2-7, N-terminally processed]	PIP2-7 PIP3 SIMIP At4g35100 M4E13.150	Arabidopsis thaliana (Mouse-ear cress)		Acetylation; Cell membrane; Complete proteome; Membrane; Methylation; Phosphoprotein; Reference proteome; Repeat; Transmembrane; Transmembrane helix; Transport
SRR1271857_TR37158_c0_g1_i2_len=1149	GSHRC_PEA	Q:1087-308,H:239-498	83.46%ID	E:3e-158	Glutathione reductase, cytosolic	PF07992.11^Pyr_redox_2^Pyridine nucleotide-disulphide oxidoreductase^14-127^E:1.2e-27`PF00070.24^Pyr_redox^Pyridine nucleotide-disulphide oxidoreductase^14-53^E:0.00014`PF02852.19^Pyr_redox_dim^Pyridine nucleotide-disulphide oxidoreductase, dimerisation domain^147-256^E:1.3e-30	1	1,263	0	-7,206	1	1,705	1	3,306	1	0	GSHRC_PEA	Glutathione reductase, cytosolic (GR) (GRase) (EC 1.8.1.7) (GOR2)		Pisum sativum (Garden pea)		Cytoplasm; Disulfide bond; FAD; Flavoprotein; NADP; Oxidoreductase; Redox-active center
SRR1271857_TR38411_c0_g1_i2_len=2751	YSL1_ARATH	Q:888-2354,H:187-673	68.51%ID	E:0	Metal-nicotianamine transporter YSL1	PF03169.12^OPT^OPT oligopeptide transporter protein^3-474^E:1.6e-106	0,822	-0,394	0	-3,14	0,043	-3,051	0,043	-3,19	1	-0,154	YSL1_ARATH	Metal-nicotianamine transporter YSL1 (Protein YELLOW STRIPE LIKE 1) (AtYSL1)	YSL1 At4g24120 T19F6.110 T19F6.8	Arabidopsis thaliana (Mouse-ear cress)		Complete proteome; Ion transport; Iron; Iron transport; Membrane; Reference proteome; Transmembrane; Transmembrane helix; Transport
SRR1271857_TR40128_c0_g1_i1_len=986	TL17_ARATH	Q:216-812,H:30-236	68.6%ID	E:8e-100	Thylakoid lumenal 17.4 kDa protein, chloroplastic {ECO:0000303|PubMed:9654141}	PF00805.19^Pentapeptide^Pentapeptide repeats (8 copies)^122-161^E:5.1e-05`PF00805.19^Pentapeptide^Pentapeptide repeats (8 copies)^156-186^E:1.9e-07`PF13599.3^Pentapeptide_4^Pentapeptide repeats (9 copies)^157-216^E:3.9e-08`PF00805.19^Pentapeptide^Pentapeptide repeats (8 copies)^167-205^E:5.8e-08	0,284	-0,316	0	-1,13	0,364	-0,329	0,948	-0,039	0,111	-0,451	TL17_ARATH	Thylakoid lumenal 17.4 kDa protein, chloroplastic (P17.4)	TL17 At5g53490 MNC6.3	Arabidopsis thaliana (Mouse-ear cress)		Alternative splicing; Chloroplast; Complete proteome; Direct protein sequencing; Plastid; Reference proteome; Repeat; Thylakoid; Transit peptide
SRR1271857_TR40607_c0_g1_i1_len=4240	GLGL1_ARATH	Q:3271-2240,H:179-522	84.3%ID	E:0	Glucose-1-phosphate adenylyltransferase large subunit 1, chloroplastic	PF00270.26^DEAD^DEAD/DEAH box helicase^91-260^E:6.8e-32`PF04851.12^ResIII^Type III restriction enzyme, res subunit^107-223^E:1.5e-06`PF00271.28^Helicase_C^Helicase conserved C-terminal domain^305-425^E:2.1e-16	0,56	-0,638	0,001	-2,934	0,035	-1,639	0,035	-1,716	1	0,043	GLGL1_ARATH	Glucose-1-phosphate adenylyltransferase large subunit 1, chloroplastic (EC 2.7.7.27) (ADP-glucose pyrophosphorylase) (ADP-glucose synthase) (AGPase S) (Alpha-D-glucose-1-phosphate adenyl transferase)	ADG2 APL1 At5g19220 T24G5.120	Arabidopsis thaliana (Mouse-ear cress)	PATHWAY: Glycan biosynthesis; starch biosynthesis.	ATP-binding; Allosteric enzyme; Chloroplast; Complete proteome; Nucleotide-binding; Nucleotidyltransferase; Phosphoprotein; Plastid; Reference proteome; Starch biosynthesis; Transferase; Transit peptide
SRR1271857_TR40879_c0_g1_i1_len=1504	ROC5_NICSY	Q:523-1077,H:115-299	80.54%ID	E:5e-103	33 kDa ribonucleoprotein, chloroplastic	PF00076.19^RRM_1^RNA recognition motif. (a.k.a. RRM, RBD, or RNP domain)^131-201^E:4.1e-20`PF00076.19^RRM_1^RNA recognition motif. (a.k.a. RRM, RBD, or RNP domain)^234-304^E:5.1e-19`PF13893.3^RRM_5^RNA recognition motif. (a.k.a. RRM, RBD, or RNP domain)^274-311^E:0.052	0,266	-0,333	0	-1,258	0,854	0,129	0,592	0,243	0,435	0,221	ROC5_NICSY	33 kDa ribonucleoprotein, chloroplastic		Nicotiana sylvestris (Wood tobacco) (South American tobacco)		Chloroplast; Direct protein sequencing; Plastid; RNA-binding; Repeat; Ribonucleoprotein; Transit peptide; mRNA processing
SRR1271857_TR42059_c0_g5_i2_len=966	NA	NA	NA	NA	NA	.	1	0	0	-9,088	1	-0,185	0,593	-0,594	1	0,853	.	.	.	.	.	.
SRR1271857_TR44314_c1_g1_i3_len=1133	H33_VITVI	Q:892-512,H:1-127	100%ID	E:1e-83	Histone H3.3	PF00125.21^Histone^Core histone H2A/H2B/H3/H4^1-132^E:3e-55	0,786	0,069	0	-1,263	0,062	0,466	0,555	0,152	0,505	-0,486	H33_VITVI	Histone H3.3		Vitis vinifera (Grape)		Acetylation; Chromosome; DNA-binding; Methylation; Nucleosome core; Nucleus; Phosphoprotein
SRR1271858_TR1443_c0_g1_i1_len=2199	PCP_PONAB	Q:382-1779,H:25-488	44.4%ID	E:7e-138	Lysosomal Pro-X carboxypeptidase	PF05577.9^Peptidase_S28^Serine carboxypeptidase S28^59-481^E:2.4e-86	0,502	-0,261	0	-2,769	0,288	0,521	0,618	0,296	0,799	0,117	PCP_PONAB	Lysosomal Pro-X carboxypeptidase (EC 3.4.16.2) (Proline carboxypeptidase) (Prolylcarboxypeptidase) (PRCP)	PRCP	Pongo abelii (Sumatran orangutan) (Pongo pygmaeus abelii)		Carboxypeptidase; Complete proteome; Disulfide bond; Glycoprotein; Hydrolase; Lysosome; Protease; Reference proteome; Signal; Zymogen
SRR1271858_TR7375_c0_g2_i2_len=1031	TRL31_ARATH	Q:849-301,H:26-209	54.55%ID	E:3e-64	Thioredoxin-like 3-1, chloroplastic	PF00085.17^Thioredoxin^Thioredoxin^79-168^E:5.4e-16	0,151	0,735	0	-1,414	1	-1,19	1	-0,009	0,781	-0,381	TRL31_ARATH	Thioredoxin-like 3-1, chloroplastic (Thioredoxin WCRKC-1)	WCRKC1 At5g06690 MPH15.4	Arabidopsis thaliana (Mouse-ear cress)		Alternative splicing; Chloroplast; Complete proteome; Disulfide bond; Electron transport; Plastid; Redox-active center; Reference proteome; Transit peptide; Transport
SRR1271858_TR10742_c0_g2_i1_len=1065	Y2296_ARATH	Q:84-497,H:348-486	76.98%ID	E:3e-58	Probable receptor-like protein kinase At2g42960	.	0,543	-0,237	0	-1,508	0,724	-0,177	0,058	-0,926	0,767	-0,103	Y2296_ARATH	Probable receptor-like protein kinase At2g42960 (EC 2.7.11.1)	At2g42960 F7D19.4	Arabidopsis thaliana (Mouse-ear cress)		ATP-binding; Complete proteome; Kinase; Membrane; Nucleotide-binding; Phosphoprotein; Receptor; Reference proteome; Serine/threonine-protein kinase; Transferase; Transmembrane; Transmembrane helix
SRR1271858_TR12178_c0_g1_i1_len=653	NA	NA	NA	NA	NA	.	0,904	-0,121	0	-2,204	0,011	1,221	0,77	0,272	1	-1,099	.	.	.	.	.	.
SRR1271858_TR13744_c0_g1_i13_len=1547	CHAC2_RAT	Q:1328-792,H:2-175	40.88%ID	E:3e-31	Putative glutathione-specific gamma-glutamylcyclotransferase 2 {ECO:0000250|UniProtKB:Q9BUX1}	PF04752.9^ChaC^ChaC-like protein^3-180^E:4.5e-46	0,304	-0,416	0	-2,185	0,545	0,424	0,08	1,032	0,026	-2,284	CHAC2_RAT	Putative glutathione-specific gamma-glutamylcyclotransferase 2 (Gamma-GCG 2) (EC 2.3.2.-) (Cation transport regulator-like protein 2)	Chac2	Rattus norvegicus (Rat)		Acyltransferase; Complete proteome; Reference proteome; Transferase
SRR1271858_TR13857_c0_g4_i1_len=1042	PIP12_ORYSJ	Q:68-913,H:1-285	89.47%ID	E:0	Probable aquaporin PIP1-2	PF00230.17^MIP^Major intrinsic protein^65-294^E:7.6e-85	0,325	-0,322	0	-1,989	0,587	-0,229	0,049	-0,92	0,319	0,255	PIP12_ORYSJ	Probable aquaporin PIP1-2 (OsPIP1;2) (Plasma membrane intrinsic protein 1-2)	PIP1-2 Os04g0559700 LOC_Os04g47220 OsJ_015100 OSJNBa0084K11.2	Oryza sativa subsp. japonica (Rice)		Cell membrane; Complete proteome; Membrane; Reference proteome; Repeat; Transmembrane; Transmembrane helix; Transport
SRR1271858_TR15930_c0_g2_i3_len=1344	NA	NA	NA	NA	NA	.	0,13	-0,554	0	-3,097	0,381	-0,85	0,16	-1,257	0,382	0,328	.	.	.	.	.	.
SRR1271858_TR28664_c0_g1_i6_len=923	GGH1_ARATH	Q:170-748,H:153-344	67.36%ID	E:7e-87	Gamma-glutamyl hydrolase 1	.	1	-1,704	0	-5,095	0,381	1,625	1	-0,074	0,402	-1,149	GGH1_ARATH	Gamma-glutamyl hydrolase 1 (AtGGH1) (EC 3.4.19.9) (Conjugase) (GH) (Gamma-Glu-X carboxypeptidase)	GGH1 At1g78660 T30F21.1	Arabidopsis thaliana (Mouse-ear cress)		Alternative splicing; Cell wall; Complete proteome; Hydrolase; Reference proteome; Secreted; Signal; Vacuole
SRR1271858_TR28808_c0_g1_i3_len=1351	FATB_ARATH	Q:1101-529,H:212-402	72.77%ID	E:3e-95	Palmitoyl-acyl carrier protein thioesterase, chloroplastic	PF01643.14^Acyl-ACP_TE^Acyl-ACP thioesterase^18-206^E:7e-55	1	-0,085	0	-2,355	0,034	3,39	0,254	2,192	1	0,05	FATB_ARATH	Palmitoyl-acyl carrier protein thioesterase, chloroplastic (EC 3.1.2.-) (16:0-acyl-carrier protein thioesterase) (16:0-ACP thioesterase) (Acyl-[acyl-carrier-protein] hydrolase) (Acyl-acyl carrier protein thioesterase B1) (AtFATB1)	FATB FATB1 At1g08510 T27G7.19	Arabidopsis thaliana (Mouse-ear cress)		Chloroplast; Complete proteome; Fatty acid biosynthesis; Fatty acid metabolism; Hydrolase; Lipid biosynthesis; Lipid metabolism; Plastid; Reference proteome; Transit peptide
SRR1271858_TR51167_c0_g2_i3_len=2239	NA	NA	NA	NA	NA	.	1	-1,24	0	-6,624	0,502	3,271	0,502	3,176	0	7,262	.	.	.	.	.	.
SRR1271858_TR52149_c0_g2_i1_len=738	PPCS1_ARATH	Q:737-333,H:145-313	60.36%ID	E:8e-50	Phosphopantothenate--cysteine ligase 1	PF04127.12^DFP^DNA / pantothenate metabolism flavoprotein^23-95^E:8.9e-08	1	0	0,001	-3,981	1	1,376	1	0	1	0	PPCS1_ARATH	Phosphopantothenate--cysteine ligase 1 (EC 6.3.2.5) (AtCoaB) (Phosphopantothenoylcysteine synthetase 1) (PPC synthetase 1)	PPCS1 COAB COAB1 At1g12350 F5O11.7	Arabidopsis thaliana (Mouse-ear cress)	PATHWAY: Cofactor biosynthesis; coenzyme A biosynthesis; CoA from (R)-pantothenate: step 2/5.	Coenzyme A biosynthesis; Complete proteome; Ligase; Reference proteome
SRR1271858_TR54585_c0_g1_i1_len=1696	NA	NA	NA	NA	NA	.	0,788	-0,185	0	-2,4	1	-0,545	1	-0,528	0,298	-0,597	.	.	.	.	.	.
SRR1271858_TR60476_c0_g1_i2_len=1214	TPT_SOLTU	Q:129-947,H:142-414	90.11%ID	E:3e-164	Triose phosphate/phosphate translocator, chloroplastic	PF03151.13^TPT^Triose-phosphate Transporter family^3-260^E:2.4e-96`PF00892.17^EamA^EamA-like transporter family^116-260^E:7.1e-07	0,816	-0,071	0	-1,843	0,319	-0,224	0,052	-0,437	0,074	-0,416	TPT_SOLTU	Triose phosphate/phosphate translocator, chloroplastic (cTPT) (E29)	TPT	Solanum tuberosum (Potato)		Chloroplast; Complete proteome; Membrane; Plastid; Reference proteome; Transit peptide; Transmembrane; Transmembrane helix; Transport
SRR1271859_TR316_c0_g1_i1_len=640	NA	NA	NA	NA	NA	.	0,675	-0,242	0	-2,418	0,593	-0,532	0,798	-0,32	0,005	-1,271	.	.	.	.	.	.
SRR1271859_TR1921_c1_g3_i3_len=1300	HIBC6_ARATH	Q:1223-291,H:108-421	69.52%ID	E:2e-152	3-hydroxyisobutyryl-CoA hydrolase-like protein 3, mitochondrial	PF16113.2^ECH_2^Enoyl-CoA hydratase/isomerase^3-281^E:2e-79	1	-0,023	0,001	-2,628	0,629	1,673	1	1,331	1	-0,609	HIBC6_ARATH	3-hydroxyisobutyryl-CoA hydrolase-like protein 3, mitochondrial (EC 3.1.2.-)	At4g13360 T9E8.100	Arabidopsis thaliana (Mouse-ear cress)		Acetylation; Complete proteome; Hydrolase; Mitochondrion; Reference proteome; Transit peptide
SRR1271859_TR3310_c0_g1_i1_len=382	NA	NA	NA	NA	NA	.	0,679	-0,21	0	-2,648	1	-0,033	1	-0,164	0,122	-0,582	.	.	.	.	.	.
SRR1271859_TR3835_c0_g2_i1_len=1173	NA	NA	NA	NA	NA	.	0,053	-0,532	0	-1,519	1	-0,018	0,515	0,222	0,803	-0,081	.	.	.	.	.	.
SRR1271859_TR7542_c0_g1_i1_len=1537	G3PA2_ARATH	Q:361-1290,H:90-399	92.58%ID	E:2e-170	Glyceraldehyde-3-phosphate dehydrogenase GAPA2, chloroplastic	PF02800.17^Gp_dh_C^Glyceraldehyde 3-phosphate dehydrogenase, C-terminal domain^1-139^E:6.2e-59	0,479	-0,229	0	-1,769	0,092	-0,397	0,073	-0,422	0,128	-0,324	G3PA2_ARATH	Glyceraldehyde-3-phosphate dehydrogenase GAPA2, chloroplastic (EC 1.2.1.13) (NADP-dependent glyceraldehydephosphate dehydrogenase A subunit 2)	GAPA2 At1g12900 F13K23.15	Arabidopsis thaliana (Mouse-ear cress)	PATHWAY: Carbohydrate biosynthesis; Calvin cycle.	Alternative splicing; Calvin cycle; Chloroplast; Complete proteome; NADP; Oxidoreductase; Plastid; Reference proteome; Transit peptide
SRR1271859_TR8683_c1_g1_i1_len=1175	H33_VITVI	Q:120-500,H:1-127	100%ID	E:2e-83	Histone H3.3	PF00125.21^Histone^Core histone H2A/H2B/H3/H4^1-132^E:3e-55	0,272	-0,266	0	-0,985	0,142	0,375	0,344	0,248	0,581	0,12	H33_VITVI	Histone H3.3		Vitis vinifera (Grape)		Acetylation; Chromosome; DNA-binding; Methylation; Nucleosome core; Nucleus; Phosphoprotein
SRR1271859_TR12424_c0_g1_i7_len=3879	TBL41_ARATH	Q:1828-2280,H:34-185	47.1%ID	E:4e-36	Protein trichome birefringence-like 41	PF00011.18^HSP20^Hsp20/alpha crystallin family^183-282^E:1.5e-13	0,796	0,084	0	-0,96	1	-0,692	1	-0,608	0,261	0,381	TBL41_ARATH	Protein trichome birefringence-like 41	TBL41 At3g14850 T21E2.11	Arabidopsis thaliana (Mouse-ear cress)		Alternative splicing; Complete proteome; Membrane; Reference proteome; Signal-anchor; Transmembrane; Transmembrane helix
SRR1271859_TR14759_c1_g1_i2_len=1723	GDT11_ARATH	Q:682-1572,H:73-370	69.57%ID	E:1e-103	GDT1-like protein 1, chloroplastic	PF01169.16^UPF0016^Uncharacterized protein family UPF0016^163-246^E:3.5e-19`PF01169.16^UPF0016^Uncharacterized protein family UPF0016^291-364^E:1.7e-20	0,502	-0,313	0	-1,705	0,011	-1,17	0,004	-1,325	1	-0,016	GDT11_ARATH	GDT1-like protein 1, chloroplastic	At1g64150 F22C12.9	Arabidopsis thaliana (Mouse-ear cress)		Chloroplast; Complete proteome; Membrane; Plastid; Reference proteome; Transit peptide; Transmembrane; Transmembrane helix
SRR1271859_TR17588_c0_g1_i2_len=2402	ICR2_ARATH	Q:344-2188,H:1-583	49.45%ID	E:4e-128	Interactor of constitutive active ROPs 2, chloroplastic	.	0,169	-0,638	0,001	-2,761	0,798	-0,382	1	-0,186	0,781	0,162	ICR2_ARATH	Interactor of constitutive active ROPs 2, chloroplastic (ROP-interactive partner 2)	ICR2 RIP2 At2g37080 T2N18.16	Arabidopsis thaliana (Mouse-ear cress)		Chloroplast; Coiled coil; Complete proteome; Phosphoprotein; Plastid; Reference proteome; Transit peptide
SRR1271859_TR20104_c0_g1_i1_len=1330	PRS6A_BRACM	Q:142-1047,H:116-417	98.34%ID	E:0	26S protease regulatory subunit 6A homolog	PF07728.11^AAA_5^AAA domain (dynein-related subfamily)^48-168^E:6.6e-07`PF05496.9^RuvB_N^Holliday junction DNA helicase ruvB N-terminus^48-117^E:2.7e-05`PF00004.26^AAA^ATPase family associated with various cellular activities (AAA)^49-181^E:5.1e-43`PF07724.11^AAA_2^AAA domain (Cdc48 subfamily)^49-141^E:9.3e-06	0,502	3,778	0,001	-1,819	0,103	1,651	0,254	-4,698	0,692	-1,029	PRS6A_BRACM	26S protease regulatory subunit 6A homolog (Tat-binding protein homolog 1) (TBP-1)	TBP1 TBP	Brassica campestris (Field mustard)		ATP-binding; Cytoplasm; Nucleotide-binding; Nucleus; Phosphoprotein; Proteasome
SRR1271859_TR21744_c0_g1_i1_len=897	PSBW_ARATH	Q:323-634,H:19-119	62.86%ID	E:6e-22	Photosystem II reaction center W protein, chloroplastic	PF07123.9^PsbW^Photosystem II reaction centre W protein (PsbW)^1-135^E:3.4e-56	0,87	-0,036	0	-0,957	0,045	-0,419	0,046	-0,418	0,63	0,101	PSBW_ARATH	Photosystem II reaction center W protein, chloroplastic (PSII 6.1 kDa protein)	PSBW At2g30570 T6B20.8	Arabidopsis thaliana (Mouse-ear cress)		Chloroplast; Complete proteome; Membrane; Photosynthesis; Photosystem II; Plastid; Reference proteome; Thylakoid; Transit peptide; Transmembrane; Transmembrane helix
SRR1271859_TR22061_c0_g1_i3_len=1458	WLIM1_ARATH	Q:1125-598,H:2-177	86.36%ID	E:3e-108	LIM domain-containing protein WLIM1 {ECO:0000305}	PF00412.19^LIM^LIM domain^11-65^E:2.2e-10`PF00412.19^LIM^LIM domain^111-165^E:2.4e-12	0,51	-0,164	0	-1,275	0,283	0,444	0,164	0,541	0,816	-0,066	WLIM1_ARATH	LIM domain-containing protein WLIM1 (Widely-expressed LIM protein 1) (AtWLIM1)	WLIM1 At1g10200 F14N23.8	Arabidopsis thaliana (Mouse-ear cress)		Acetylation; Actin-binding; Complete proteome; Cytoplasm; Cytoskeleton; LIM domain; Metal-binding; Reference proteome; Repeat; Zinc
SRR1271859_TR25327_c0_g1_i1_len=622	H4_SOYBN	Q:200-445,H:22-103	100%ID	E:2e-49	Histone H4	.	0,319	0,965	0	-6,689	0,643	-0,429	0,472	-0,672	0	6,755	H4_SOYBN	Histone H4		Glycine max (Soybean) (Glycine hispida)		Acetylation; Chromosome; Complete proteome; DNA-binding; Methylation; Nucleosome core; Nucleus; Reference proteome
SRR1271859_TR26579_c1_g1_i4_len=1160	XTH9_ARATH	Q:101-964,H:10-284	71.88%ID	E:3e-155	Xyloglucan endotransglucosylase/hydrolase protein 9	PF00722.18^Glyco_hydro_16^Glycosyl hydrolases family 16^56-232^E:3.1e-57`PF06955.9^XET_C^Xyloglucan endo-transglycosylase (XET) C-terminus^276-321^E:1.8e-17	0,009	-0,625	0	-3,411	0,908	0,123	0,807	-0,146	1	-1,755	XTH9_ARATH	Xyloglucan endotransglucosylase/hydrolase protein 9 (At-XTH9) (XTH-9) (EC 2.4.1.207)	XTH9 EXGT-A6 XTR16 At4g03210 F4C21.14	Arabidopsis thaliana (Mouse-ear cress)		Alternative splicing; Apoplast; Cell wall; Cell wall biogenesis/degradation; Complete proteome; Disulfide bond; Glycoprotein; Glycosidase; Hydrolase; Reference proteome; Secreted; Signal; Transferase
SRR1271859_TR29015_c0_g1_i1_len=2855	SUBL_ARATH	Q:276-2540,H:5-752	42.56%ID	E:1e-177	.	PF05922.13^Inhibitor_I9^Peptidase inhibitor I9^24-115^E:7.7e-13`PF00082.19^Peptidase_S8^Subtilase family^139-577^E:2.4e-49	0,594	-0,256	0	-2,201	1	0,116	0,732	-0,946	0,518	-0,371	SBT17_ARATH	Subtilisin-like protease SBT1.7 (EC 3.4.21.-) (Cucumisin-like serine protease) (Subtilase subfamily 1 member 7) (AtSBT1.7) (Subtilisin-like serine protease 1) (At-SLP1)	SBT1.7 ARA12 ASP48 SLP1 At5g67360 K8K14.8	Arabidopsis thaliana (Mouse-ear cress)		Calcium; Cell wall; Complete proteome; Direct protein sequencing; Glycoprotein; Hydrolase; Protease; Reference proteome; Secreted; Serine protease; Signal; Zymogen
SRR1271859_TR34222_c0_g4_i1_len=990	KCS10_ARATH	Q:195-740,H:345-549	64.88%ID	E:1e-84	3-ketoacyl-CoA synthase 10 {ECO:0000303|PubMed:18465198}	.	0,049	-0,763	0	-2,085	0,718	-0,24	0,626	-0,297	0,241	-0,397	KCS10_ARATH	3-ketoacyl-CoA synthase 10 (KCS-10) (EC 2.3.1.199) (Protein FIDDLEHEAD) (Very long-chain fatty acid condensing enzyme 10) (VLCFA condensing enzyme 10)	FDH EL4 KCS10 At2g26250 T1D16.11	Arabidopsis thaliana (Mouse-ear cress)	PATHWAY: Lipid metabolism; fatty acid biosynthesis.	Acyltransferase; Complete proteome; Endoplasmic reticulum; Membrane; Reference proteome; Transferase; Transmembrane; Transmembrane helix
SRR1271859_TR34628_c0_g3_i4_len=1073	NA	NA	NA	NA	NA	PF05564.9^Auxin_repressed^Dormancy/auxin associated protein^7-85^E:6.6e-10	0,007	-0,61	0	-1,073	0,121	0,379	0,09	0,414	0,428	0,287	.	.	.	.	.	.
SRR1271859_TR36710_c0_g1_i11_len=1799	NA	NA	NA	NA	NA	.	0,493	-0,229	0	-1,546	0,287	-0,362	0,705	-0,136	0,039	-0,652	.	.	.	.	.	.
SRR1271859_TR37559_c0_g1_i6_len=1205	HIS8_ARATH	Q:1060-254,H:197-465	86.25%ID	E:4e-169	Histidinol dehydrogenase, chloroplastic	PF00815.17^Histidinol_dh^Histidinol dehydrogenase^1-236^E:3.4e-97	0,34	-0,558	0	-1,348	0,003	1,845	0,235	0,929	0,502	-2,146	HIS8_ARATH	Histidinol dehydrogenase, chloroplastic (HDH) (EC 1.1.1.23) (Protein HISTIDINE BIOSYNTHESIS 8)	HISN8 HDH At5g63890 MGI19.9	Arabidopsis thaliana (Mouse-ear cress)	PATHWAY: Amino-acid biosynthesis; L-histidine biosynthesis; L-histidine from 5-phospho-alpha-D-ribose 1-diphosphate: step 9/9.	Alternative splicing; Amino-acid biosynthesis; Chloroplast; Complete proteome; Histidine biosynthesis; Metal-binding; NAD; Oxidoreductase; Plastid; Reference proteome; Transit peptide; Zinc
SRR1271859_TR40225_c1_g2_i2_len=1108	CPNB1_ARATH	Q:1004-507,H:435-600	92.77%ID	E:3e-90	Chaperonin 60 subunit beta 1, chloroplastic	.	0,064	-0,656	0	-1,001	0,005	1,594	0,002	1,754	0,214	-0,748	CPNB1_ARATH	Chaperonin 60 subunit beta 1, chloroplastic (CPN-60 beta 1) (60 kDa chaperonin subunit beta 1) (RuBisCO large subunit-binding protein subunit beta, chloroplastic)	CPN60B1 Cpn60-B(3) LEN1 At1g55490 T5A14.11	Arabidopsis thaliana (Mouse-ear cress)		ATP-binding; Chaperone; Chloroplast; Complete proteome; Direct protein sequencing; Nucleotide-binding; Phosphoprotein; Plastid; Reference proteome; Transit peptide
SRR1271859_TR43547_c0_g2_i1_len=1624	THI42_VITVI	Q:197-1222,H:15-355	87.13%ID	E:0	Thiamine thiazole synthase 2, chloroplastic {ECO:0000255|HAMAP-Rule:MF_03158}	PF01946.14^Thi4^Thi4 family^69-302^E:1.1e-114`PF00890.21^FAD_binding_2^FAD binding domain^87-123^E:7.7e-07`PF01266.21^DAO^FAD dependent oxidoreductase^87-136^E:1e-07`PF13450.3^NAD_binding_8^NAD(P)-binding Rossmann-like domain^90-124^E:3.9e-07	0,927	-0,022	0	-0,8	0,228	-1,144	0,352	-0,828	0,449	0,159	THI42_VITVI	Thiamine thiazole synthase 2, chloroplastic (Thiazole biosynthetic enzyme 2)	THI1-2 VIT_10s0116g00530	Vitis vinifera (Grape)		Chloroplast; Complete proteome; Iron; Metal-binding; NAD; Plastid; Reference proteome; Thiamine biosynthesis; Transit peptide
SRR1271859_TR43547_c0_g2_i2_len=1737	THI42_VITVI	Q:197-1057,H:15-300	88.5%ID	E:0	Thiamine thiazole synthase 2, chloroplastic {ECO:0000255|HAMAP-Rule:MF_03158}	PF01946.14^Thi4^Thi4 family^69-298^E:1.5e-111`PF00890.21^FAD_binding_2^FAD binding domain^87-123^E:6.8e-07`PF01266.21^DAO^FAD dependent oxidoreductase^87-136^E:9e-08`PF13450.3^NAD_binding_8^NAD(P)-binding Rossmann-like domain^90-124^E:3.3e-07	0,528	0,254	0	-1,075	1	-2,641	1	-1,452	0,25	-0,367	THI42_VITVI	Thiamine thiazole synthase 2, chloroplastic (Thiazole biosynthetic enzyme 2)	THI1-2 VIT_10s0116g00530	Vitis vinifera (Grape)		Chloroplast; Complete proteome; Iron; Metal-binding; NAD; Plastid; Reference proteome; Thiamine biosynthesis; Transit peptide
SRR1271859_TR45104_c0_g1_i2_len=932	ALL10_OLEEU	Q:518-793,H:30-123	73.4%ID	E:2e-40	Major pollen allergen Ole e 10	.	0,71	-0,136	0	-0,998	0,021	0,701	0,017	0,724	0,258	-0,897	ALL10_OLEEU	Major pollen allergen Ole e 10 (allergen Ole e 10)		Olea europaea (Common olive)		Allergen; Cytoplasmic vesicle; Direct protein sequencing; Disulfide bond; Phosphoprotein; Signal
SRR1271859_TR46033_c0_g1_i1_len=2848	NA	NA	NA	NA	NA	PF14389.3^Lzipper-MIP1^Leucine-zipper of ternary complex factor MIP1^46-126^E:6.6e-20`PF04784.11^DUF547^Protein of unknown function, DUF547^401-530^E:3.7e-33	0,578	-0,227	0	-1,85	0,129	-1,004	0,045	-1,415	0,352	-0,511	.	.	.	.	.	.
SRR342017_TR919_c2_g1_i1_len=1316	UGPI7_ARATH	Q:246-1016,H:40-277	45.59%ID	E:6e-65	Uncharacterized GPI-anchored protein At4g28100	.	0,648	-0,189	0	-3,394	1	-0,188	0,62	-0,737	0,304	-0,393	UGPI7_ARATH	Uncharacterized GPI-anchored protein At4g28100	At4g28100 T13J8.210	Arabidopsis thaliana (Mouse-ear cress)		Cell membrane; Complete proteome; GPI-anchor; Glycoprotein; Lipoprotein; Membrane; Reference proteome; Signal
SRR342017_TR975_c0_g1_i1_len=1882	STAD_RICCO	Q:1370-195,H:1-396	87.12%ID	E:0	Stearoyl-[acyl-carrier-protein] 9-desaturase, chloroplastic	PF03405.11^FA_desaturase_2^Fatty acid desaturase^65-385^E:5.2e-161	0,09	-0,674	0	-1,411	0,337	-0,344	0,43	-0,306	1	-0,026	STAD_RICCO	Stearoyl-[acyl-carrier-protein] 9-desaturase, chloroplastic (Stearoyl-ACP desaturase) (EC 1.14.19.2) (Acyl-[acyl-carrier-protein] desaturase) (Delta(9) stearoyl-acyl carrier protein desaturase)		Ricinus communis (Castor bean)	PATHWAY: Lipid metabolism; fatty acid metabolism.	3D-structure; Chloroplast; Fatty acid biosynthesis; Fatty acid metabolism; Iron; Lipid biosynthesis; Lipid metabolism; Metal-binding; Oxidoreductase; Plastid; Transit peptide
SRR342017_TR1342_c0_g1_i1_len=2150	NA	NA	NA	NA	NA	.	0,798	-0,223	0	-2,611	1	1,189	1	1,306	0,847	0,104	.	.	.	.	.	.
SRR342017_TR1504_c0_g1_i1_len=1955	ILVH1_ARATH	Q:1520-303,H:74-477	77.34%ID	E:0	Acetolactate synthase small subunit 1, chloroplastic	PF01842.22^ACT^ACT domain^91-154^E:2.8e-11`PF13710.3^ACT_5^ACT domain^100-159^E:2.2e-10`PF10369.6^ALS_ss_C^Small subunit of acetolactate synthase^170-242^E:3e-27`PF01842.22^ACT^ACT domain^324-388^E:2e-08`PF13710.3^ACT_5^ACT domain^332-394^E:3.9e-13`PF10369.6^ALS_ss_C^Small subunit of acetolactate synthase^405-477^E:2.7e-25	0,031	-0,741	0,001	-1,427	0,2	-0,693	0,2	-0,649	0,922	0,047	ILVH1_ARATH	Acetolactate synthase small subunit 1, chloroplastic (Acetohydroxy-acid synthase small subunit) (AHAS) (ALS) (Protein valine-tolerant 1)	VAT1 At5g16290 MQK4.1	Arabidopsis thaliana (Mouse-ear cress)	PATHWAY: Amino-acid biosynthesis; L-isoleucine biosynthesis; L-isoleucine from 2-oxobutanoate: step 1/4.; PATHWAY: Amino-acid biosynthesis; L-valine biosynthesis; L-valine from pyruvate: step 1/4.	Amino-acid biosynthesis; Branched-chain amino acid biosynthesis; Chloroplast; Complete proteome; Plastid; Reference proteome; Repeat; Transit peptide
SRR342017_TR1716_c1_g6_i1_len=1241	NA	NA	NA	NA	NA	.	0,862	-0,093	0,001	-1,626	0,708	-0,197	0,781	-0,13	0,622	0,22	.	.	.	.	.	.
SRR342017_TR2606_c2_g1_i1_len=1023	NA	NA	NA	NA	NA	PF06549.9^DUF1118^Protein of unknown function (DUF1118)^76-190^E:1.8e-49	0,983	-0,01	0,001	-0,813	0,007	-0,611	0,009	-0,595	0,791	0,065	.	.	.	.	.	.
SRR342017_TR3133_c0_g3_i1_len=978	NA	NA	NA	NA	NA	PF00301.17^Rubredoxin^Rubredoxin^73-107^E:2e-05	0,22	-0,39	0	-2,065	0,001	-0,938	0,034	-0,604	0,014	-0,739	.	.	.	.	.	.

SRR342017_TR4629_c0_g1_i2_len=1340	PPD5_ARATH	Q:1052-291,H:35-297	68.94%ID	E:2e-122	PsbP domain-containing protein 5, chloroplastic	PF01789.13^PsbP^PsbP^175-278^E:8.7e-12	0,204	-0,431	0	-1,307	0,086	-0,98	0,086	-1,006	0,446	0,335	PPD5_ARATH	PsbP domain-containing protein 5, chloroplastic (OEC23-like protein 6) (PsbP-related thylakoid lumenal protein 4) (Thylakoid lumenal 35.8 kDa protein)	PPD5 At5g11450 F15N18.40	Arabidopsis thaliana (Mouse-ear cress)		Chloroplast; Complete proteome; Direct protein sequencing; Plastid; Reference proteome; Thylakoid; Transit peptide
SRR342017_TR5172_c0_g1_i3_len=1021	NA	NA	NA	NA	NA	.	0,339	0,295	0	-1,81	0,509	0,313	0,318	-0,468	0,057	0,602	.	.	.	.	.	.
SRR342017_TR6674_c1_g1_i1_len=743	EPFL9_ARATH	Q:468-238,H:25-102	64.56%ID	E:8e-27	EPIDERMAL PATTERNING FACTOR-like protein 9 {ECO:0000303|PubMed:19435754}	PF16851.2^Stomagen^Stomagen^70-118^E:3.8e-31	0,276	-0,326	0	-1,594	0,297	-0,323	1	-0,01	0,403	-0,261	EPFL9_ARATH	EPIDERMAL PATTERNING FACTOR-like protein 9 (EPF-like protein 9) [Cleaved into: Stomagen]	EPFL9 STOMAGEN At4g12970 F25G13.60	Arabidopsis thaliana (Mouse-ear cress)		3D-structure; Apoplast; Complete proteome; Developmental protein; Direct protein sequencing; Disulfide bond; Reference proteome; Secreted; Signal
SRR342017_TR7022_c0_g3_i3_len=521	NA	NA	NA	NA	NA	.	0,026	1,877	0	-7,86	1	0	1	2,843	0,212	-0,601	.	.	.	.	.	.
SRR342017_TR7988_c6_g1_i2_len=562	IF2C_ARATH	Q:192-518,H:907-1015	71.56%ID	E:2e-41	Translation initiation factor IF-2, chloroplastic	.	0,132	-0,839	0	-2,213	0,014	-0,943	0,003	-1,169	0,134	-0,503	IF2C_ARATH	Translation initiation factor IF-2, chloroplastic	At1g17220 F20D23.8	Arabidopsis thaliana (Mouse-ear cress)		Chloroplast; Complete proteome; GTP-binding; Initiation factor; Nucleotide-binding; Plastid; Protein biosynthesis; Reference proteome; Transit peptide
SRR342017_TR8359_c0_g3_i1_len=764	NA	NA	NA	NA	NA	.	0,62	-0,654	0,001	-2,699	0,44	-0,927	0,282	-1,08	0,472	-0,813	.	.	.	.	.	.
SRR342017_TR9948_c0_g9_i2_len=547	UCRIB_TOBAC	Q:2-226,H:156-228	92%ID	E:4e-42	Cytochrome b6-f complex iron-sulfur subunit 2, chloroplastic	.	0,187	0,606	0,001	-1,054	0,849	0,088	0,368	0,293	0,018	-0,549	UCRIB_TOBAC	Cytochrome b6-f complex iron-sulfur subunit 2, chloroplastic (EC 1.10.9.1) (Plastohydroquinone:plastocyanin oxidoreductase iron-sulfur protein 2) (Rieske iron-sulfur protein 2) (ISP 2) (RISP 2)	petC2	Nicotiana tabacum (Common tobacco)		2Fe-2S; Chloroplast; Disulfide bond; Electron transport; Iron; Iron-sulfur; Membrane; Metal-binding; Oxidoreductase; Plastid; Thylakoid; Transit peptide; Transmembrane; Transmembrane helix; Transport
SRR342017_TR10207_c0_g1_i1_len=1783	CCD32_ARATH	Q:757-1662,H:71-367	50.48%ID	E:2e-96	Cyclin-D3-2	PF00134.20^Cyclin_N^Cyclin, N-terminal domain^96-201^E:2.2e-27`PF02984.16^Cyclin_C^Cyclin, C-terminal domain^203-312^E:5.2e-23	0,003	-0,962	0	-3,582	0,138	-0,89	0,035	-1,397	0,438	-0,275	CCD32_ARATH	Cyclin-D3-2 (G1/S-specific cyclin-D3-2) (CycD3;2)	CYCD3-2 At5g67260 K3G17.2	Arabidopsis thaliana (Mouse-ear cress)		Cell cycle; Cell division; Complete proteome; Cyclin; Reference proteome
SRR342017_TR10435_c0_g3_i2_len=1330	Y1816_ARATH	Q:550-1032,H:57-221	36.97%ID	E:4e-31	Uncharacterized protein At1g08160	PF03168.10^LEA_2^Late embryogenesis abundant protein^105-203^E:5.3e-15	0,618	-0,307	0	-2,14	0,023	-1,702	0,004	-2,57	1	-0,064	Y1816_ARATH	Uncharacterized protein At1g08160	At1g08160 T23G18.2 T6D22.24	Arabidopsis thaliana (Mouse-ear cress)		Complete proteome; Membrane; Reference proteome; Transmembrane; Transmembrane helix
SRR342017_TR10755_c0_g5_i2_len=874	NA	NA	NA	NA	NA	.	0,654	0,162	0,001	-1,141	0,18	0,765	0,086	0,927	1	-0,024	.	.	.	.	.	.
SRR342017_TR11246_c0_g2_i1_len=1688	TBB8_ARATH	Q:3-1199,H:28-426	99.5%ID	E:0	Tubulin beta-8 chain	PF00091.22^Tubulin^Tubulin/FtsZ family, GTPase domain^2-184^E:1.4e-55`PF03953.14^Tubulin_C^Tubulin C-terminal domain^234-354^E:6.4e-41	0,268	-0,417	0	-1,324	0,849	0,117	0,642	0,234	0,028	-0,721	TBB8_ARATH	Tubulin beta-8 chain (Beta-8-tubulin)	TUBB8 TUB8 At5g23860 MRO11.10	Arabidopsis thaliana (Mouse-ear cress)		Complete proteome; Cytoplasm; Cytoskeleton; GTP-binding; Microtubule; Nucleotide-binding; Reference proteome
SRR342017_TR11303_c0_g1_i2_len=1532	NA	NA	NA	NA	NA	.	0,497	-0,159	0	-1,558	0,157	-0,353	0,853	-0,05	0,057	-0,609	.	.	.	.	.	.
SRR342017_TR11456_c1_g2_i1_len=376	NA	NA	NA	NA	NA	.	0,108	-0,697	0	-2,018	0,356	1,069	0,042	1,827	0,02	-1,15	.	.	.	.	.	.
SRR342017_TR11486_c0_g1_i1_len=2734	ICE1_ARATH	Q:969-1862,H:212-467	60.4%ID	E:3e-86	Transcription factor ICE1	PF00010.23^HLH^Helix-loop-helix DNA-binding domain^392-435^E:3.4e-09	0,116	-0,514	0,001	-1,546	0,428	-0,398	0,745	-0,187	0,9	-0,063	ICE1_ARATH	Transcription factor ICE1 (Basic helix-loop-helix protein 116) (AtbHLH116) (bHLH 116) (Inducer of CBF expression 1) (Transcription factor EN 45) (Transcription factor SCREAM) (bHLH transcription factor bHLH116)	SCRM BHLH116 EN45 ICE1 At3g26744 MDJ14.1 MLJ15.14	Arabidopsis thaliana (Mouse-ear cress)		Activator; Alternative splicing; Coiled coil; Complete proteome; DNA-binding; Developmental protein; Isopeptide bond; Nucleus; Reference proteome; Stress response; Transcription; Transcription regulation; Ubl conjugation
SRR342017_TR12047_c0_g1_i1_len=2075	APR1_ARATH	Q:1809-496,H:29-465	75.74%ID	E:0	5'-adenylylsulfate reductase 1, chloroplastic	PF01507.16^PAPS_reduct^Phosphoadenosine phosphosulfate reductase family^115-295^E:6.8e-43`PF00085.17^Thioredoxin^Thioredoxin^369-461^E:1.5e-11	0,101	-0,55	0	-2,464	0,002	-0,718	0,089	-0,396	0,563	0,151	APR1_ARATH	5'-adenylylsulfate reductase 1, chloroplastic (EC 1.8.4.9) (3'-phosphoadenosine-5'-phosphosulfate reductase homolog 19) (PAPS reductase homolog 19) (Prh-19) (Adenosine 5'-phosphosulfate 5'-adenylylsulfate sulfotransferase 1) (APS sulfotransferase 1) (Thioredoxin-independent APS reductase 1)	APR1 PRH19 At4g04610 F4H6.13	Arabidopsis thaliana (Mouse-ear cress)		4Fe-4S; Amino-acid biosynthesis; Chloroplast; Complete proteome; Cysteine biosynthesis; Disulfide bond; Iron; Iron-sulfur; Metal-binding; Oxidoreductase; Plastid; Redox-active center; Reference proteome; Stress response; Transit peptide
SRR342017_TR12197_c1_g2_i1_len=969	NA	NA	NA	NA	NA	PF08847.8^DUF1817^Domain of unknown function (DUF1817)^118-272^E:2.6e-59	0,308	-0,45	0	-1,786	0,84	-0,191	1	0,182	0,341	0,401	.	.	.	.	.	.
SRR342017_TR12606_c0_g1_i2_len=1005	NA	NA	NA	NA	NA	.	0,068	-1,245	0	-6,624	0,076	-3,323	0,017	-3,732	0,874	0,205	.	.	.	.	.	.
SRR342017_TR13028_c0_g2_i1_len=2002	XTH_TOBAC	Q:1150-260,H:1-295	75.76%ID	E:3e-169	Probable xyloglucan endotransglucosylase/hydrolase protein	PF00722.18^Glyco_hydro_16^Glycosyl hydrolases family 16^35-215^E:2.6e-60`PF06955.9^XET_C^Xyloglucan endo-transglycosylase (XET) C-terminus^244-291^E:8.9e-17	0,095	-0,709	0,001	-2,169	0,299	1,444	1	0,558	0,777	-0,124	XTH_TOBAC	Probable xyloglucan endotransglucosylase/hydrolase protein (EC 2.4.1.207)	XTH EXGT	Nicotiana tabacum (Common tobacco)		Apoplast; Cell wall; Cell wall biogenesis/degradation; Disulfide bond; Glycoprotein; Glycosidase; Hydrolase; Secreted; Signal; Transferase
SRR342017_TR13461_c0_g2_i1_len=1207	NA	NA	NA	NA	NA	PF02201.15^SWIB^SWIB/MDM2 domain^133-206^E:9.6e-29	0,002	-0,78	0	-1,598	0,736	-0,148	0,752	0,146	0,17	-0,347	.	.	.	.	.	.
SRR342017_TR14859_c2_g2_i1_len=1916	S17P_SPIOL	Q:1811-645,H:1-387	81.63%ID	E:0	Sedoheptulose-1,7-bisphosphatase, chloroplastic	PF00316.17^FBPase^Fructose-1-6-bisphosphatase, N-terminal domain^104-280^E:8.3e-58	0,915	-0,032	0,001	-0,798	0,002	-0,677	0,037	-0,442	0,611	0,115	S17P_SPIOL	Sedoheptulose-1,7-bisphosphatase, chloroplastic (EC 3.1.3.37) (SED(1,7)P2ase) (Sedoheptulose bisphosphatase) (SBPase)		Spinacia oleracea (Spinach)	PATHWAY: Carbohydrate biosynthesis; Calvin cycle.	Calvin cycle; Carbohydrate metabolism; Chloroplast; Disulfide bond; Hydrolase; Magnesium; Metal-binding; Plastid; Transit peptide
SRR342017_TR15341_c2_g8_i5_len=1275	C72A1_CATRO	Q:1274-843,H:380-523	99.31%ID	E:1e-94	Secologanin synthase	PF00067.19^p450^Cytochrome P450^1-118^E:2.9e-39	1	0,221	0	-3,798	1	-0,49	1	-1,15	0,802	0,126	C72A1_CATRO	Secologanin synthase (SLS) (EC 1.3.3.9) (CYPLXXII) (Cytochrome P450 72A1)	CYP72A1 CYP72 P450CR3	Catharanthus roseus (Madagascar periwinkle) (Vinca rosea)	PATHWAY: Alkaloid biosynthesis; secologanin biosynthesis.	Alkaloid metabolism; Heme; Iron; Membrane; Metal-binding; Monooxygenase; NADP; Oxidoreductase; Transmembrane; Transmembrane helix; Vacuole
SRR342017_TR16388_c0_g1_i2_len=847	PSAG_ARATH	Q:191-613,H:27-160	72.34%ID	E:7e-67	Photosystem I reaction center subunit V, chloroplastic	PF01241.15^PSI_PSAK^Photosystem I psaG / psaK^53-144^E:2.5e-26	0,288	0,251	0	-1,58	0,04	-0,453	0,232	-0,261	0	1,027	PSAG_ARATH	Photosystem I reaction center subunit V, chloroplastic (PSI-G)	PSAG At1g55670 F20N2.21 F20N2.33 F20N2_3	Arabidopsis thaliana (Mouse-ear cress)		Chloroplast; Complete proteome; Membrane; Photosynthesis; Photosystem I; Plastid; Reference proteome; Thylakoid; Transit peptide; Transmembrane; Transmembrane helix
SRR342017_TR17640_c0_g2_i1_len=718	NA	NA	NA	NA	NA	.	0,142	-0,851	0	-6,69	0,629	-1,321	1	-0,978	0,452	-0,587	.	.	.	.	.	.
SRR342017_TR18200_c0_g1_i1_len=1852	CB22_TOBAC	Q:1079-285,H:1-265	93.96%ID	E:1e-179	Chlorophyll a-b binding protein 21, chloroplastic	PF00504.18^Chloroa_b-bind^Chlorophyll A-B binding protein^65-231^E:1.6e-50	0,034	-0,771	0	-2,14	0,255	-0,443	0,003	-1,238	0,369	0,349	CB22_TOBAC	Chlorophyll a-b binding protein 21, chloroplastic (LHCII type I CAB-21) (LHCP)	CAB21	Nicotiana tabacum (Common tobacco)		Acetylation; Chlorophyll; Chloroplast; Chromophore; Magnesium; Membrane; Metal-binding; Phosphoprotein; Photosynthesis; Photosystem I; Photosystem II; Plastid; Thylakoid; Transit peptide; Transmembrane; Transmembrane helix
SRR342017_TR18577_c0_g2_i20_len=1972	FKB65_ARATH	Q:140-1762,H:40-558	49.37%ID	E:9e-176	Peptidyl-prolyl cis-trans isomerase FKBP65	PF00254.25^FKBP_C^FKBP-type peptidyl-prolyl cis-trans isomerase^68-157^E:1.7e-31	0,318	-0,456	0	-2,938	1	0,15	1	-0,063	0,93	-0,057	FKB65_ARATH	Peptidyl-prolyl cis-trans isomerase FKBP65 (PPIase FKBP65) (EC 5.2.1.8) (70 kDa peptidyl-prolyl isomerase) (FK506-binding protein 65) (AtFKBP65) (Immunophilin FKBP65) (Peptidyl-prolyl isomerase ROF2) (Protein ROTAMASE FKBP 2) (Rotamase)	FKBP65 ROF2 At5g48570 K15N18.12	Arabidopsis thaliana (Mouse-ear cress)		Acetylation; Calmodulin-binding; Chaperone; Complete proteome; Cytoplasm; Isomerase; Nucleus; Reference proteome; Repeat; TPR repeat
SRR342017_TR18632_c0_g4_i1_len=539	MAN5_ARATH	Q:455-165,H:337-431	55.67%ID	E:9e-30	Mannan endo-1,4-beta-mannosidase 5	.	1	0	0	-7,414	1	0	1	0	1	0	MAN5_ARATH	Mannan endo-1,4-beta-mannosidase 5 (EC 3.2.1.78) (Beta-mannanase 5) (Endo-beta-1,4-mannanase 5) (AtMAN5)	MAN5 At4g28320 F26K10.200	Arabidopsis thaliana (Mouse-ear cress)		Complete proteome; Glycoprotein; Glycosidase; Hydrolase; Reference proteome; Secreted; Signal
SRR342017_TR19031_c4_g5_i1_len=963	NA	NA	NA	NA	NA	.	0,326	-0,53	0	-1,762	1	0,043	0,763	0,216	1	-0,167	.	.	.	.	.	.
SRR342017_TR19163_c0_g3_i2_len=1129	APA1_ARATH	Q:1126-50,H:121-481	65.1%ID	E:2e-175	Aspartic proteinase A1	PF00026.20^Asp^Eukaryotic aspartyl protease^3-356^E:8.9e-90`PF03489.14^SapB_2^Saposin-like type B, region 2^197-229^E:8.4e-13`PF05184.12^SapB_1^Saposin-like type B, region 1^259-295^E:1.5e-12	1	0	0	-7,111	1	0	1	0	1	0,6	APA1_ARATH	Aspartic proteinase A1 (EC 3.4.23.-)	APA1 PRA1 At1g11910 F12F1.24	Arabidopsis thaliana (Mouse-ear cress)		Aspartyl protease; Complete proteome; Disulfide bond; Glycoprotein; Hydrolase; Protease; Reference proteome; Repeat; Signal; Vacuole; Zymogen
SRR342017_TR19173_c0_g1_i1_len=1674	ROC4_NICSY	Q:246-1253,H:1-312	59.82%ID	E:6e-96	31 kDa ribonucleoprotein, chloroplastic	PF00076.19^RRM_1^RNA recognition motif. (a.k.a. RRM, RBD, or RNP domain)^162-231^E:1.3e-20`PF00076.19^RRM_1^RNA recognition motif. (a.k.a. RRM, RBD, or RNP domain)^256-326^E:3.3e-22	0,032	-0,478	0	-0,784	0,755	-0,083	0,292	0,262	0,94	-0,019	ROC4_NICSY	31 kDa ribonucleoprotein, chloroplastic		Nicotiana sylvestris (Wood tobacco) (South American tobacco)		Chloroplast; Direct protein sequencing; Plastid; RNA-binding; Repeat; Ribonucleoprotein; Transit peptide; mRNA processing
SRR342017_TR19201_c0_g1_i1_len=1082	ATPX_SPIOL	Q:725-288,H:77-222	80.82%ID	E:2e-63	ATP synthase subunit b', chloroplastic	PF00430.15^ATP-synt_B^ATP synthase B/B' CF(0)^92-220^E:1.7e-14`PF01442.15^Apolipoprotein^Apolipoprotein A1/A4/E domain^123-164^E:20`PF01442.15^Apolipoprotein^Apolipoprotein A1/A4/E domain^129-218^E:0.13	1	0	0	-2,431	0,252	-0,535	0,438	-0,365	1	0	ATPX_SPIOL	ATP synthase subunit b', chloroplastic (ATP synthase F(0) sector subunit b') (ATPase subunit II)	ATPG	Spinacia oleracea (Spinach)		ATP-binding; CF(0); Chloroplast; Direct protein sequencing; Hydrogen ion transport; Ion transport; Membrane; Nucleotide-binding; Plastid; Thylakoid; Transit peptide; Transmembrane; Transmembrane helix; Transport
SRR342017_TR19621_c2_g1_i3_len=1883	AMI1_ARATH	Q:218-1546,H:1-421	60.94%ID	E:2e-162	Amidase 1	PF01425.18^Amidase^Amidase^9-62^E:1.2e-19`PF01425.18^Amidase^Amidase^210-296^E:1.2e-05	0,505	0,457	0	-2,499	0,56	-0,637	0,017	-5,787	0,419	-0,377	AMI1_ARATH	Amidase 1 (AtAMI1) (EC 3.5.1.4) (Translocon at the outer membrane of chloroplasts 64-I) (AtTOC64-I)	AMI1 TOC64-I At1g08980 F7G19.15	Arabidopsis thaliana (Mouse-ear cress)		Acetylation; Auxin biosynthesis; Complete proteome; Cytoplasm; Hydrolase; Reference proteome
SRR342017_TR19664_c0_g1_i1_len=2119	CXE17_ARATH	Q:1698-766,H:28-339	35.19%ID	E:6e-57	Probable carboxylesterase 17	PF10340.6^DUF2424^Protein of unknown function (DUF2424)^31-200^E:1.8e-07`PF00135.25^COesterase^Carboxylesterase family^58-165^E:1.5e-06`PF07859.10^Abhydrolase_3^alpha/beta hydrolase fold^71-289^E:3.4e-48	0,558	-0,224	0,001	-1,184	1	0,409	1	0,563	0,918	-0,093	CXE17_ARATH	Probable carboxylesterase 17 (AtCXE17) (EC 3.1.1.1)	CXE17 At5g16080 F1N13.220	Arabidopsis thaliana (Mouse-ear cress)		Complete proteome; Hydrolase; Reference proteome; Serine esterase
SRR342017_TR20102_c2_g4_i1_len=752	PER42_ARATH	Q:7-330,H:223-330	87.04%ID	E:7e-60	Peroxidase 42	PF00141.20^peroxidase^Peroxidase^18-71^E:1.4e-09	0,261	0,391	0	-4,343	0,086	0,965	0,564	-0,522	0,099	0,363	PER42_ARATH	Peroxidase 42 (Atperox P42) (EC 1.11.1.7) (ATP1a/ATP1b) (PRXR1)	PER42 P42 At4g21960 F1N20.3 T8O5.170	Arabidopsis thaliana (Mouse-ear cress)		Biological rhythms; Calcium; Complete proteome; Disulfide bond; Glycoprotein; Heme; Hydrogen peroxide; Iron; Metal-binding; Oxidoreductase; Peroxidase; Reference proteome; Secreted; Signal
SRR342017_TR20586_c0_g4_i1_len=1031	PMA1_NICPL	Q:1031-291,H:711-957	92.31%ID	E:4e-128	Plasma membrane ATPase 1	.	0,747	-0,104	0	-1,346	0,651	-0,17	0,126	-0,507	0,034	-0,582	PMA1_NICPL	Plasma membrane ATPase 1 (EC 3.6.3.6) (Proton pump 1)	PMA1	Nicotiana plumbaginifolia (Leadwort-leaved tobacco) (Tex-Mex tobacco)		ATP-binding; Cell membrane; Hydrogen ion transport; Hydrolase; Ion transport; Magnesium; Membrane; Metal-binding; Nucleotide-binding; Phosphoprotein; Transmembrane; Transmembrane helix; Transport
SRR342017_TR20775_c0_g1_i1_len=588	TLP14_ORYSJ	Q:538-293,H:365-445	79.27%ID	E:1e-33	Tubby-like F-box protein 14	.	1	0	0	-8,161	0,034	-5,565	0,034	-5,565	1	0	TLP14_ORYSJ	Tubby-like F-box protein 14 (OsTLP14) (Tubby-like F-box protein 4) (OsTLP4)	TULP14 TULP4 Os12g0163400 LOC_Os12g06630 OsJ_033954	Oryza sativa subsp. japonica (Rice)		Complete proteome; Reference proteome
SRR342017_TR21338_c0_g1_i1_len=550	NA	NA	NA	NA	NA	.	0,951	-0,034	0	-1,398	0,793	-0,098	0,64	-0,182	0,027	-0,665	.	.	.	.	.	.
SRR342017_TR21756_c0_g1_i5_len=1453	NA	NA	NA	NA	NA	.	0,587	-0,268	0	-1,675	0,079	-1,073	0,004	-1,966	0,708	-0,182	.	.	.	.	.	.
SRR342017_TR22056_c0_g1_i3_len=484	NA	NA	NA	NA	NA	.	0,253	-0,367	0	-1,479	0,627	-0,166	0,954	0,046	0,311	-0,396	.	.	.	.	.	.
SRR342017_TR25020_c2_g3_i1_len=763	NA	NA	NA	NA	NA	.	0,126	-0,375	0	-1,923	0,239	-1,273	0,239	-1,45	0,3	0,238	.	.	.	.	.	.
SRR342017_TR25222_c0_g2_i1_len=1299	NA	NA	NA	NA	NA	PF04949.10^Transcrip_act^Transcriptional activator^23-170^E:1.6e-74	0,548	0,227	0	-1,474	0,635	0,354	0,87	0,258	0	1,031	.	.	.	.	.	.
SRR342017_TR25892_c2_g4_i6_len=4889	POLX_TOBAC	Q:4888-3482,H:858-1323	55.65%ID	E:1e-150	Retrovirus-related Pol polyprotein from transposon TNT 1-94	PF14223.3^Retrotran_gag_2^gag-polypeptide of LTR copia-type^59-187^E:7e-34`PF00098.20^zf-CCHC^Zinc knuckle^227-242^E:0.00034`PF13976.3^gag_pre-integrs^GAG-pre-integrase domain^397-464^E:1.3e-14`PF00665.23^rve^Integrase core domain^480-594^E:2.5e-26	0	4,536	0	-1,503	0	1,941	0	1,869	0	3,153	POLX_TOBAC	Retrovirus-related Pol polyprotein from transposon TNT 1-94 [Includes: Protease (EC 3.4.23.-); Reverse transcriptase (EC 2.7.7.49); Endonuclease]		Nicotiana tabacum (Common tobacco)		Aspartyl protease; Endonuclease; Hydrolase; Metal-binding; Nuclease; Nucleotidyltransferase; Protease; RNA-directed DNA polymerase; Transferase; Transposable element; Zinc; Zinc-finger
SRR342017_TR26233_c2_g8_i2_len=1347	GTOMC_ORYSJ	Q:169-1017,H:75-357	44.01%ID	E:6e-83	Probable tocopherol O-methyltransferase, chloroplastic	PF13489.3^Methyltransf_23^Methyltransferase domain^49-173^E:2.1e-14`PF01209.15^Ubie_methyltran^ubiE/COQ5 methyltransferase family^64-178^E:1.9e-10`PF13847.3^Methyltransf_31^Methyltransferase domain^68-174^E:1.6e-17`PF02353.17^CMAS^Mycolic acid cyclopropane synthetase^69-167^E:1.5e-14`PF09445.7^Methyltransf_15^RNA cap guanine-N2 methyltransferase^70-130^E:2.4e-05`PF07021.9^MetW^Methionine biosynthesis protein MetW^70-163^E:5.1e-05`PF08241.9^Methyltransf_11^Methyltransferase domain^72-170^E:3.8e-21`PF13649.3^Methyltransf_25^Methyltransferase domain^72-165^E:5.2e-19`PF08242.9^Methyltransf_12^Methyltransferase domain^72-166^E:1.2e-12	0,029	0,473	0	-0,964	1	-0,006	0,002	-0,742	0,043	0,438	GTOMC_ORYSJ	Probable tocopherol O-methyltransferase, chloroplastic (EC 2.1.1.95) (Gamma-tocopherol methyltransferase) (Vitamin E pathway gene 4 protein)	VTE4 G-TMT TMT Os02g0701600 LOC_Os02g47310 OJ1111_E07.25	Oryza sativa subsp. japonica (Rice)	PATHWAY: Cofactor biosynthesis; tocopherol biosynthesis.	Chloroplast; Complete proteome; Methyltransferase; Plastid; Reference proteome; S-adenosyl-L-methionine; Transferase; Transit peptide
SRR342017_TR26436_c1_g4_i10_len=3559	NHLC2_BOVIN	Q:1359-3203,H:32-708	35.79%ID	E:3e-112	NHL repeat-containing protein 2	PF00702.23^Hydrolase^haloacid dehalogenase-like hydrolase^89-267^E:1.4e-15`PF13419.3^HAD_2^Haloacid dehalogenase-like hydrolase^91-274^E:5.9e-28`PF13242.3^Hydrolase_like^HAD-hyrolase-like^231-295^E:2.5e-05`PF01436.18^NHL^NHL repeat^625-652^E:4.2e-07	0,18	-0,816	0,001	-1,799	1	-0,264	0,822	0,238	0,472	-0,311	NHLC2_BOVIN	NHL repeat-containing protein 2	NHLRC2	Bos taurus (Bovine)		Complete proteome; Reference proteome; Repeat
SRR342017_TR27170_c0_g1_i1_len=709	NA	NA	NA	NA	NA	PF00685.24^Sulfotransfer_1^Sulfotransferase domain^3-69^E:2.2e-08	0,544	-0,205	0	-2,399	0,03	-1,058	0,012	-1,243	0,395	-0,595	.	.	.	.	.	.
SRR342017_TR27230_c0_g1_i1_len=711	NA	NA	NA	NA	NA	.	0,159	-0,45	0	-4,132	0,163	-0,659	0,007	-1,504	0,76	-0,098	.	.	.	.	.	.
SRR342017_TR27610_c0_g1_i11_len=1163	MDHG_CITLA	Q:1118-129,H:28-355	72.37%ID	E:2e-162	Malate dehydrogenase, glyoxysomal	PF00056.20^Ldh_1_N^lactate/malate dehydrogenase, NAD binding domain^1-126^E:3.1e-28`PF02866.15^Ldh_1_C^lactate/malate dehydrogenase, alpha/beta C-terminal domain^128-295^E:2.7e-41	0,249	-0,49	0	-2,237	0,517	0,815	1	0,452	1	0	MDHG_CITLA	Malate dehydrogenase, glyoxysomal (EC 1.1.1.37)		Citrullus lanatus (Watermelon) (Citrullus vulgaris)		3D-structure; Direct protein sequencing; Glyoxylate bypass; Glyoxysome; NAD; Oxidoreductase; Peroxisome; Transit peptide; Tricarboxylic acid cycle
SRR342017_TR28770_c2_g3_i3_len=2469	AXS2_ARATH	Q:1477-323,H:5-389	90.13%ID	E:0	UDP-D-apiose/UDP-D-xylose synthase 2	PF01370.18^Epimerase^NAD dependent epimerase/dehydratase family^17-287^E:1.2e-59`PF03435.15^Sacchrp_dh_NADP^Saccharopine dehydrogenase NADP binding domain^17-94^E:9.3e-07`PF01073.16^3Beta_HSD^3-beta hydroxysteroid dehydrogenase/isomerase family^19-142^E:8.6e-07`PF16363.2^GDP_Man_Dehyd^GDP-mannose 4,6 dehydratase^21-362^E:1.4e-30	1	1,628	0	-6,984	0,524	0,582	1	0,194	0,502	-4,349	AXS2_ARATH	UDP-D-apiose/UDP-D-xylose synthase 2	AXS2 At1g08200 T23G18.6	Arabidopsis thaliana (Mouse-ear cress)		Cell wall biogenesis/degradation; Complete proteome; Cytoplasm; NAD; Reference proteome
SRR342017_TR29077_c0_g1_i2_len=569	PIP28_ARATH	Q:549-301,H:196-278	93.98%ID	E:2e-46	Probable aquaporin PIP2-8	.	0,552	0,229	0	-4,658	0,826	-0,135	0,917	0,099	0,02	-0,636	PIP28_ARATH	Probable aquaporin PIP2-8 (Plasma membrane intrinsic protein 2-8) (AtPIP2;8) (Plasma membrane intrinsic protein 3b) (PIP3b)	PIP2-8 PIP3B At2g16850 F12A24.3	Arabidopsis thaliana (Mouse-ear cress)		Cell membrane; Complete proteome; Membrane; Phosphoprotein; Reference proteome; Repeat; Transmembrane; Transmembrane helix; Transport
SRR342017_TR30599_c0_g5_i1_len=820	RLF33_ARATH	Q:649-317,H:15-115	54.05%ID	E:4e-29	Protein RALF-like 33	PF05498.8^RALF^Rapid ALkalinization Factor (RALF)^106-168^E:4.1e-29	0,161	-0,385	0	-1,572	0,055	-0,527	0,017	-0,663	0,461	-1,367	RLF33_ARATH	Protein RALF-like 33	RALFL33 At4g15800 dl3940c FCAALL.206	Arabidopsis thaliana (Mouse-ear cress)		Complete proteome; Disulfide bond; Glycoprotein; Hormone; Reference proteome; Secreted; Signal
SRR342017_TR31242_c0_g1_i1_len=720	NA	NA	NA	NA	NA	.	0,407	-0,466	0,001	-1,721	0,505	0,447	0,729	0,263	0,041	-0,91	.	.	.	.	.	.
SRR342017_TR31253_c0_g2_i21_len=1608	ALIS5_ARATH	Q:288-1331,H:21-345	56.9%ID	E:5e-133	ALA-interacting subunit 5	.	0,87	-0,225	0	-2,628	0,798	-0,539	0,402	-0,888	0,874	-0,204	ALIS5_ARATH	ALA-interacting subunit 5 (AtALIS5)	ALIS5 At1g79450 T8K14.13	Arabidopsis thaliana (Mouse-ear cress)		Acetylation; Alternative splicing; Complete proteome; Endoplasmic reticulum; Glycoprotein; Golgi apparatus; Membrane; Reference proteome; Transmembrane; Transmembrane helix
SRR342017_TR31536_c3_g3_i1_len=819	DADIS_PANGI	Q:492-307,H:699-760	91.94%ID	E:1e-30	Dammarenediol II synthase	.	0,889	0,086	0	-1,955	0,025	-1,436	0,013	-1,617	0,803	-0,165	DADIS_PANGI	Dammarenediol II synthase (EC 4.2.1.125)	PNA DDS	Panax ginseng (Korean ginseng)		Isomerase; Lyase; Membrane; Repeat; Transmembrane; Transmembrane helix
SRR342019_TR1230_c0_g2_i2_len=2630	EFP_SYNP2	Q:300-848,H:2-184	59.56%ID	E:2e-69	Elongation factor P {ECO:0000255|HAMAP-Rule:MF_00141}	PF17177.1^PPR_long^Pentacotripeptide-repeat region of PROPR^132-243^E:6.2e-10`PF13041.3^PPR_2^PPR repeat family^142-191^E:4.8e-13`PF01535.17^PPR^PPR repeat^145-175^E:0.00064`PF13812.3^PPR_3^Pentatricopeptide repeat domain^166-222^E:4.6e-16`PF12854.4^PPR_1^PPR repeat^174-206^E:1.1e-13`PF13041.3^PPR_2^PPR repeat family^177-225^E:4.8e-19`PF01535.17^PPR^PPR repeat^181-210^E:2.1e-08`PF12854.4^PPR_1^PPR repeat^209-238^E:8.9e-10`PF13041.3^PPR_2^PPR repeat family^212-261^E:6.5e-16`PF01535.17^PPR^PPR repeat^216-242^E:0.0004`PF13812.3^PPR_3^Pentatricopeptide repeat domain^236-294^E:4.1e-08`PF12854.4^PPR_1^PPR repeat^246-274^E:2.6e-10`PF13041.3^PPR_2^PPR repeat family^247-296^E:1.9e-14`PF01535.17^PPR^PPR repeat^250-280^E:1.1e-07`PF12854.4^PPR_1^PPR repeat^278-311^E:7.2e-07`PF13041.3^PPR_2^PPR repeat family^282-331^E:3e-15`PF01535.17^PPR^PPR repeat^285-315^E:3.1e-07`PF13812.3^PPR_3^Pentatricopeptide repeat domain^306-349^E:0.00024`PF13041.3^PPR_2^PPR repeat family^317-366^E:5.2e-10`PF01535.17^PPR^PPR repeat^320-350^E:0.0015	0,034	-1,185	0	-2,218	0,593	-0,63	0,402	-0,984	0,736	-0,145	EFP_SYNP2	Elongation factor P (EF-P)	efp SYNPCC7002_A0053	Synechococcus sp. (strain ATCC 27264 / PCC 7002 / PR-6) (Agmenellum quadruplicatum)	PATHWAY: Protein biosynthesis; polypeptide chain elongation. {ECO:0000255|HAMAP-Rule:MF_00141}.	Complete proteome; Cytoplasm; Elongation factor; Protein biosynthesis; Reference proteome
SRR342019_TR1477_c3_g6_i1_len=748	ISPG_ORYSJ	Q:747-349,H:644-744	71.43%ID	E:2e-52	4-hydroxy-3-methylbut-2-en-1-yl diphosphate synthase (ferredoxin), chloroplastic	.	0,356	1,42	0	-1,966	0,076	1,375	0,226	-2,927	0,034	-5,714	ISPG_ORYSJ	4-hydroxy-3-methylbut-2-en-1-yl diphosphate synthase (ferredoxin), chloroplastic (EC 1.17.7.1) (1-hydroxy-2-methyl-2-(E)-butenyl 4-diphosphate synthase)	ISPG Os02g0603800 LOC_Os02g39160 OJ1669_F01.30 OsJ_07430	Oryza sativa subsp. japonica (Rice)	PATHWAY: Isoprenoid biosynthesis; isopentenyl diphosphate biosynthesis via DXP pathway; isopentenyl diphosphate from 1-deoxy-D-xylulose 5-phosphate: step 5/6.	4Fe-4S; Chloroplast; Complete proteome; Iron; Iron-sulfur; Isoprene biosynthesis; Metal-binding; Oxidoreductase; Plastid; Reference proteome; Transit peptide
SRR342019_TR3331_c4_g11_i3_len=2941	ERGI3_XENLA	Q:1296-2435,H:4-387	53.23%ID	E:2e-139	Endoplasmic reticulum-Golgi intermediate compartment protein 3	PF13850.3^ERGIC_N^Endoplasmic Reticulum-Golgi Intermediate Compartment (ERGIC)^8-97^E:1.8e-31`PF07970.9^COPIIcoated_ERV^Endoplasmic reticulum vesicle transporter^149-366^E:1.7e-88	1	0	0	-7,047	1	0	1	0	0,429	-0,52	ERGI3_XENLA	Endoplasmic reticulum-Golgi intermediate compartment protein 3	ergic3	Xenopus laevis (African clawed frog)		ER-Golgi transport; Endoplasmic reticulum; Golgi apparatus; Membrane; Transmembrane; Transmembrane helix; Transport
SRR342019_TR3671_c1_g1_i1_len=748	NA	NA	NA	NA	NA	PF06596.8^PsbX^Photosystem II reaction centre X protein (PsbX)^115-152^E:1.4e-16	0,181	0,283	0	-0,823	0,003	-0,61	0,009	-0,542	0,943	-0,016	.	.	.	.	.	.
SRR342019_TR5610_c0_g2_i3_len=2383	NAT6_ARATH	Q:428-1957,H:22-532	87.28%ID	E:0	Nucleobase-ascorbate transporter 6	PF00860.17^Xan_ur_permease^Permease family^37-440^E:1.6e-68	0,226	-0,675	0	-1,871	0,5	0,72	0,356	-0,996	0,172	-0,606	NAT6_ARATH	Nucleobase-ascorbate transporter 6 (AtNAT6)	NAT6 At5g62890 MQB2.190 MQB2.21	Arabidopsis thaliana (Mouse-ear cress)		Alternative splicing; Complete proteome; Membrane; Reference proteome; Transmembrane; Transmembrane helix; Transport
SRR342019_TR6934_c0_g1_i1_len=565	VDAC2_SOLTU	Q:557-210,H:161-276	88.79%ID	E:2e-68	Mitochondrial outer membrane protein porin of 36 kDa	PF01459.19^Porin_3^Eukaryotic porin^3-111^E:1.3e-31	0,256	-0,392	0,001	-1,056	0,543	-0,187	0,287	-0,319	0,134	-0,512	VDAC2_SOLTU	Mitochondrial outer membrane protein porin of 36 kDa (POM 36) (Voltage-dependent anion-selective channel protein) (VDAC)		Solanum tuberosum (Potato)		Complete proteome; Direct protein sequencing; Ion transport; Membrane; Mitochondrion; Mitochondrion outer membrane; Porin; Reference proteome; Transmembrane; Transmembrane beta strand; Transport
SRR342019_TR8544_c1_g1_i1_len=1638	NA	NA	NA	NA	NA	.	0,321	-0,311	0	-1,604	0,712	-0,219	0,904	-0,135	0,741	-0,128	.	.	.	.	.	.
SRR342019_TR10573_c0_g1_i1_len=713	NA	NA	NA	NA	NA	.	0,487	-0,295	0,001	-1,913	0,395	-0,629	0,031	-1,561	0,125	0,592	.	.	.	.	.	.
SRR342019_TR12472_c0_g4_i1_len=1222	NA	NA	NA	NA	NA	.	0,163	-0,695	0	-2,256	0,518	0,384	0,691	0,241	0,04	-0,623	.	.	.	.	.	.
SRR342019_TR13600_c0_g2_i1_len=557	NA	NA	NA	NA	NA	.	0,95	0,028	0,001	-1,072	1	-0,036	0,253	-0,491	0,74	-0,104	.	.	.	.	.	.
SRR342019_TR14817_c0_g1_i1_len=2386	PP186_ARATH	Q:1859-171,H:8-574	66.84%ID	E:0	Pentatricopeptide repeat-containing protein At2g35130	PF13812.3^PPR_3^Pentatricopeptide repeat domain^164-212^E:3.2e-05`PF13041.3^PPR_2^PPR repeat family^165-213^E:2e-09`PF01535.17^PPR^PPR repeat^169-197^E:0.0014`PF12854.4^PPR_1^PPR repeat^198-229^E:9.8e-06`PF01535.17^PPR^PPR repeat^204-233^E:3.4e-06`PF13812.3^PPR_3^Pentatricopeptide repeat domain^204-244^E:4.9e-05`PF13812.3^PPR_3^Pentatricopeptide repeat domain^224-284^E:4e-12`PF13041.3^PPR_2^PPR repeat family^235-280^E:1.3e-12`PF01535.17^PPR^PPR repeat^238-268^E:0.00025`PF17177.1^PPR_long^Pentacotripeptide-repeat region of PROPR^253-356^E:5.3e-10`PF12854.4^PPR_1^PPR repeat^267-299^E:2.6e-05`PF13041.3^PPR_2^PPR repeat family^274-319^E:4.4e-14`PF01535.17^PPR^PPR repeat^274-303^E:5.8e-06`PF13812.3^PPR_3^Pentatricopeptide repeat domain^287-317^E:8.5e-07`PF12854.4^PPR_1^PPR repeat^301-331^E:5.8e-08`PF13041.3^PPR_2^PPR repeat family^305-346^E:1e-09`PF01535.17^PPR^PPR repeat^309-337^E:1.7e-07`PF13812.3^PPR_3^Pentatricopeptide repeat domain^329-388^E:3.2e-11`PF13041.3^PPR_2^PPR repeat family^340-387^E:1.8e-11`PF01535.17^PPR^PPR repeat^344-373^E:5.5e-05`PF17177.1^PPR_long^Pentacotripeptide-repeat region of PROPR^362-488^E:1.2e-08`PF12854.4^PPR_1^PPR repeat^371-403^E:3.7e-08`PF13041.3^PPR_2^PPR repeat family^378-423^E:1.6e-08`PF01535.17^PPR^PPR repeat^379-408^E:7.1e-08`PF13812.3^PPR_3^Pentatricopeptide repeat domain^400-459^E:9.2e-07`PF01535.17^PPR^PPR repeat^414-442^E:0.0019`PF13041.3^PPR_2^PPR repeat family^445-492^E:2.1e-07`PF01535.17^PPR^PPR repeat^450-473^E:0.008`PF01535.17^PPR^PPR repeat^483-507^E:7.3e-06`PF13812.3^PPR_3^Pentatricopeptide repeat domain^508-562^E:8.8e-06`PF13041.3^PPR_2^PPR repeat family^515-563^E:1.1e-08`PF01535.17^PPR^PPR repeat^518-548^E:4.7e-06	0,119	-0,635	0	-2,33	0,009	-1,124	0,08	-0,743	1	0,023	PP186_ARATH	Pentatricopeptide repeat-containing protein At2g35130	At2g35130 T4C15.20	Arabidopsis thaliana (Mouse-ear cress)		Alternative splicing; Complete proteome; Reference proteome; Repeat
SRR342019_TR15642_c0_g1_i1_len=1813	YCF39_CYAPA	Q:373-1302,H:2-312	50.64%ID	E:4e-109	Uncharacterized protein ycf39	PF05368.10^NmrA^NmrA-like family^126-347^E:3.5e-24`PF01370.18^Epimerase^NAD dependent epimerase/dehydratase family^126-231^E:5.8e-08`PF01073.16^3Beta_HSD^3-beta hydroxysteroid dehydrogenase/isomerase family^127-232^E:7.7e-05`PF13460.3^NAD_binding_10^NAD(P)H-binding^130-280^E:8.2e-18	0,94	0,059	0	-1,24	0,002	-0,84	0,014	-0,661	1	-0,018	YCF39_CYAPA	Uncharacterized protein ycf39 (EC 1.-.-.-)	ycf39	Cyanophora paradoxa		Cyanelle; Oxidoreductase; Plastid
SRR342019_TR17950_c0_g6_i2_len=1298	NA	NA	NA	NA	NA	.	0,024	-0,534	0	-0,973	0,002	-0,726	0,05	-0,463	1	-1,155	.	.	.	.	.	.
SRR342019_TR17950_c0_g6_i3_len=1095	NA	NA	NA	NA	NA	.	1	0	0	-7,608	1	2,6	1	0	0,889	-0,124	.	.	.	.	.	.
SRR342019_TR18229_c0_g1_i1_len=793	BOR2_ARATH	Q:63-455,H:561-687	58.65%ID	E:2e-41	Probable boron transporter 2	.	0,444	0,409	0	-1,988	0,541	-0,267	0,039	-0,877	0,339	-0,284	BOR2_ARATH	Probable boron transporter 2	BOR2 At3g62270 T17J13.230	Arabidopsis thaliana (Mouse-ear cress)		Anion exchange; Complete proteome; Ion transport; Membrane; Reference proteome; Transmembrane; Transmembrane helix; Transport
SRR342019_TR18807_c0_g1_i1_len=1433	NA	NA	NA	NA	NA	PF02536.11^mTERF^mTERF^14-121^E:6.5e-15`PF02536.11^mTERF^mTERF^110-322^E:1.7e-46	0,095	-0,871	0	-7,024	0,356	-1,039	0,56	-0,532	0,055	0,749	.	.	.	.	.	.
SRR342019_TR19159_c2_g2_i2_len=1128	RK191_ARATH	Q:104-766,H:15-225	59.38%ID	E:1e-53	50S ribosomal protein L19-1, chloroplastic	.	1	0,292	0	-3,401	1	0	1	0	1	-1,116	RK191_ARATH	50S ribosomal protein L19-1, chloroplastic	At4g17560 dl4815w	Arabidopsis thaliana (Mouse-ear cress)		Chloroplast; Complete proteome; Plastid; RNA-binding; Reference proteome; Ribonucleoprotein; Ribosomal protein; Transit peptide; rRNA-binding
SRR342019_TR19159_c2_g2_i3_len=1353	RK191_ARATH	Q:290-991,H:2-225	59.07%ID	E:3e-58	50S ribosomal protein L19-1, chloroplastic	PF01245.17^Ribosomal_L19^Ribosomal protein L19^132-226^E:1.9e-29	0,067	-0,428	0	-0,966	0,57	-0,14	0,89	-0,037	1	-0,2	RK191_ARATH	50S ribosomal protein L19-1, chloroplastic	At4g17560 dl4815w	Arabidopsis thaliana (Mouse-ear cress)		Chloroplast; Complete proteome; Plastid; RNA-binding; Reference proteome; Ribonucleoprotein; Ribosomal protein; Transit peptide; rRNA-binding
SRR342019_TR19159_c2_g4_i6_len=1011	NA	NA	NA	NA	NA	PF00111.24^Fer2^2Fe-2S iron-sulfur cluster binding domain^34-82^E:3.2e-10	0,35	0,302	0	-1,475	0,11	-0,6	0,11	-0,586	0,279	-0,262	.	.	.	.	.	.
SRR342019_TR19281_c0_g1_i2_len=939	NA	NA	NA	NA	NA	PF00608.14^Adeno_shaft^Adenoviral fibre protein (repeat/shaft region)^122-135^E:0.35	0,159	-0,535	0	-3,545	0,103	-1,611	0,103	-1,556	0,629	-2,075	.	.	.	.	.	.
SRR342019_TR20180_c0_g2_i2_len=1073	ML423_ARATH	Q:580-371,H:83-150	55.71%ID	E:3e-33	MLP-like protein 423	PF00407.16^Bet_v_1^Pathogenesis-related protein Bet v I family^1-84^E:1.5e-17	0,63	-0,138	0	-2,308	0,003	-1,177	0,013	-0,989	0,003	0,994	ML423_ARATH	MLP-like protein 423	MLP423 At1g24020 T23E23.17 T23E23_22	Arabidopsis thaliana (Mouse-ear cress)		Complete proteome; Reference proteome
SRR342019_TR20314_c0_g1_i1_len=1626	TLPH_ARATH	Q:416-1072,H:19-240	58.04%ID	E:1e-80	Thaumatin-like protein	PF00314.14^Thaumatin^Thaumatin family^29-238^E:3.7e-82	0,255	-0,459	0	-3,298	1	-0,236	0,381	-2,223	0,826	-0,137	TLPH_ARATH	Thaumatin-like protein	At1g18250 T10O22.21	Arabidopsis thaliana (Mouse-ear cress)		Alternative splicing; Complete proteome; Disulfide bond; Reference proteome; Signal
SRR342019_TR20931_c0_g1_i1_len=2570	PP314_ARATH	Q:2494-419,H:1-698	63.97%ID	E:0	Pentatricopeptide repeat-containing protein At4g16390, chloroplastic	PF13041.3^PPR_2^PPR repeat family^169-214^E:1.1e-11`PF13812.3^PPR_3^Pentatricopeptide repeat domain^169-213^E:8.9e-07`PF01535.17^PPR^PPR repeat^172-201^E:1.8e-05`PF01535.17^PPR^PPR repeat^206-236^E:0.00014`PF13041.3^PPR_2^PPR repeat family^217-251^E:3.3e-08`PF12854.4^PPR_1^PPR repeat^235-265^E:1.5e-08`PF01535.17^PPR^PPR repeat^241-265^E:1.3e-06`PF17177.1^PPR_long^Pentacotripeptide-repeat region of PROPR^243-395^E:1.8e-12`PF13812.3^PPR_3^Pentatricopeptide repeat domain^270-321^E:8.3e-08`PF13041.3^PPR_2^PPR repeat family^274-319^E:3e-08`PF01535.17^PPR^PPR repeat^277-306^E:7.5e-05`PF13041.3^PPR_2^PPR repeat family^308-357^E:1.3e-09`PF01535.17^PPR^PPR repeat^312-341^E:0.00058`PF13812.3^PPR_3^Pentatricopeptide repeat domain^332-391^E:8.2e-11`PF01535.17^PPR^PPR repeat^347-376^E:6.2e-05`PF17177.1^PPR_long^Pentacotripeptide-repeat region of PROPR^364-503^E:2.7e-09`PF13812.3^PPR_3^Pentatricopeptide repeat domain^367-426^E:6.8e-10`PF12854.4^PPR_1^PPR repeat^374-407^E:7.5e-07`PF13041.3^PPR_2^PPR repeat family^379-424^E:1.8e-11`PF01535.17^PPR^PPR repeat^381-409^E:4.9e-05`PF01535.17^PPR^PPR repeat^418-447^E:2.3e-05`PF13812.3^PPR_3^Pentatricopeptide repeat domain^439-484^E:0.0031`PF01535.17^PPR^PPR repeat^454-482^E:0.0042	0,002	-1,326	0,001	-1,815	1	-0,77	1	-0,566	0,754	-0,14	PP314_ARATH	Pentatricopeptide repeat-containing protein At4g16390, chloroplastic (Chloroplastic RNA-binding protein P67)	P67 At4g16390 dl4225w FCAALL.354	Arabidopsis thaliana (Mouse-ear cress)		Chloroplast; Complete proteome; Nucleotide-binding; Plastid; RNA-binding; Reference proteome; Repeat; Transit peptide
SRR342019_TR22304_c0_g1_i2_len=1130	DIM_PEA	Q:851-225,H:359-567	84.21%ID	E:3e-124	Delta(24)-sterol reductase	.	0,419	-0,432	0	-2,243	1	-0,13	1	-0,369	0,312	-0,561	DIM_PEA	Delta(24)-sterol reductase (EC 1.3.1.72) (Cell elongation protein diminuto)	DIM LKB	Pisum sativum (Garden pea)		Calmodulin-binding; Membrane; NADP; Oxidoreductase; Signal-anchor; Transmembrane; Transmembrane helix
SRR342019_TR23231_c1_g1_i1_len=875	UGPI4_ARATH	Q:673-176,H:27-200	43.1%ID	E:5e-39	Uncharacterized GPI-anchored protein At3g06035	.	0,889	0,149	0	-2,502	0,066	-1,601	0,027	-2,111	0,756	-0,244	UGPI4_ARATH	Uncharacterized GPI-anchored protein At3g06035	At3g06035 24F17.7	Arabidopsis thaliana (Mouse-ear cress)		Cell membrane; Complete proteome; GPI-anchor; Glycoprotein; Lipoprotein; Membrane; Reference proteome; Signal
SRR342019_TR23658_c0_g1_i2_len=1070	TL19_ARATH	Q:385-828,H:79-226	81.76%ID	E:7e-87	Thylakoid lumenal 19 kDa protein, chloroplastic	PF01789.13^PsbP^PsbP^101-244^E:1.3e-09	0,063	-0,483	0	-1,579	0,031	-0,576	0,361	-0,244	0,284	0,296	TL19_ARATH	Thylakoid lumenal 19 kDa protein, chloroplastic (P19)	At3g63540 TEL3S	Arabidopsis thaliana (Mouse-ear cress)		Chloroplast; Complete proteome; Direct protein sequencing; Plastid; Reference proteome; Thylakoid; Transit peptide
SRR342019_TR24751_c0_g1_i1_len=1199	NA	NA	NA	NA	NA	PF01852.16^START^START domain^89-257^E:3.5e-14	0,067	-0,728	0	-2,166	0,068	-1,011	0,016	-1,489	0,223	-0,454	.	.	.	.	.	.
SRR342019_TR25234_c0_g1_i2_len=1692	SGAT_ARATH	Q:1472-273,H:1-400	89%ID	E:0	Serine--glyoxylate aminotransferase	PF00266.16^Aminotran_5^Aminotransferase class-V^12-325^E:7.5e-33	0,333	-0,247	0	-1,333	0,152	-0,302	0,122	-0,326	0,378	0,355	SGAT_ARATH	Serine--glyoxylate aminotransferase (EC 2.6.1.45) (Alanine--glyoxylate aminotransferase) (AGT) (EC 2.6.1.44) (Asparagine aminotransferase) (EC 2.6.1.-) (Serine--pyruvate aminotransferase) (EC 2.6.1.51)	AGT1 At2g13360 F14O4.7	Arabidopsis thaliana (Mouse-ear cress)		Acetylation; Aminotransferase; Complete proteome; Peroxisome; Phosphoprotein; Photorespiration; Pyridoxal phosphate; Reference proteome; Transferase
SRR342019_TR26076_c0_g3_i4_len=1586	STR14_ARATH	Q:892-371,H:44-217	78.74%ID	E:1e-93	Rhodanese-like domain-containing protein 14, chloroplastic	PF00581.17^Rhodanese^Rhodanese-like domain^93-226^E:1.8e-13	0,49	-0,177	0,001	-0,863	0,002	-0,776	0,008	-0,671	0,352	0,244	STR14_ARATH	Rhodanese-like domain-containing protein 14, chloroplastic (Sulfurtransferase 14) (AtStr14)	At4g27700 T29A15.190	Arabidopsis thaliana (Mouse-ear cress)		Chloroplast; Complete proteome; Plastid; Reference proteome; Transit peptide
SRR342019_TR26382_c0_g2_i2_len=1224	CB23_SOLLC	Q:1000-158,H:18-265	82.56%ID	E:2e-162	Chlorophyll a-b binding protein 13, chloroplastic	PF00504.18^Chloroa_b-bind^Chlorophyll A-B binding protein^1-128^E:9.8e-30	0,729	-0,192	0	-1,93	0,007	-0,877	0,003	-0,943	0,471	0,429	CB23_SOLLC	Chlorophyll a-b binding protein 13, chloroplastic (LHCII type III CAB-13)	CAB13 LHBC1	Solanum lycopersicum (Tomato) (Lycopersicon esculentum)		Chlorophyll; Chloroplast; Chromophore; Complete proteome; Direct protein sequencing; Magnesium; Membrane; Metal-binding; Phosphoprotein; Photosynthesis; Photosystem I; Photosystem II; Plastid; Reference proteome; Thylakoid; Transit peptide; Transmembrane; Transmembrane helix
SRR342019_TR28735_c2_g3_i3_len=650	GCSH_FLAAN	Q:24-440,H:24-162	84.89%ID	E:3e-79	Glycine cleavage system H protein, mitochondrial	PF01597.16^GCV_H^Glycine cleavage H-protein^22-141^E:7.1e-49	0,697	-0,095	0	-1,039	0,001	-0,73	0,004	-0,647	0,054	-0,408	GCSH_FLAAN	Glycine cleavage system H protein, mitochondrial	GDCSH GCDH GCSH	Flaveria anomala (Yellowtops)		Lipoyl; Mitochondrion; Transit peptide
SRR342019_TR29075_c0_g3_i1_len=1338	NA	NA	NA	NA	NA	.	0,1	-0,774	0	-2,809	0,037	-1,247	0,012	-1,596	0,56	-0,205	.	.	.	.	.	.
SRR342019_TR29558_c1_g1_i1_len=3758	GSO1_ARATH	Q:3640-227,H:23-1248	32.69%ID	E:1e-165	LRR receptor-like serine/threonine-protein kinase GSO1	PF08263.9^LRRNT_2^Leucine rich repeat N-terminal domain^43-81^E:1.6e-08`PF13855.3^LRR_8^Leucine rich repeat^88-146^E:4.1e-08`PF00560.30^LRR_1^Leucine Rich Repeat^137-157^E:0.88`PF13855.3^LRR_8^Leucine rich repeat^160-219^E:1.9e-08`PF00560.30^LRR_1^Leucine Rich Repeat^160-176^E:0.35`PF00560.30^LRR_1^Leucine Rich Repeat^184-206^E:0.028`PF00560.30^LRR_1^Leucine Rich Repeat^232-254^E:0.18`PF13855.3^LRR_8^Leucine rich repeat^281-340^E:2.2e-10`PF00560.30^LRR_1^Leucine Rich Repeat^304-324^E:0.57`PF13855.3^LRR_8^Leucine rich repeat^425-480^E:2.8e-07`PF13855.3^LRR_8^Leucine rich repeat^449-509^E:5.7e-08`PF00560.30^LRR_1^Leucine Rich Repeat^450-471^E:1`PF00560.30^LRR_1^Leucine Rich Repeat^578-596^E:0.95`PF13855.3^LRR_8^Leucine rich repeat^603-662^E:9.6e-08`PF00560.30^LRR_1^Leucine Rich Repeat^750-770^E:0.059`PF00069.22^Pkinase^Protein kinase domain^890-1161^E:2.4e-43`PF07714.14^Pkinase_Tyr^Protein tyrosine kinase^893-1163^E:8.7e-41`PF08263.9^LRRNT_2^Leucine rich repeat N-terminal domain^963-973^E:2200	0,237	-0,525	0	-2,71	1	-0,325	1	0,143	0,76	-0,577	GSO1_ARATH	LRR receptor-like serine/threonine-protein kinase GSO1 (EC 2.7.11.1) (Protein GASSHO 1)	GSO1 At4g20140 F1C12.60	Arabidopsis thaliana (Mouse-ear cress)		ATP-binding; Complete proteome; Developmental protein; Glycoprotein; Kinase; Leucine-rich repeat; Membrane; Nucleotide-binding; Phosphoprotein; Receptor; Reference proteome; Repeat; Serine/threonine-protein kinase; Signal; Transferase; Transmembrane; Transmembrane helix
SRR342019_TR30608_c0_g2_i1_len=1395	NA	NA	NA	NA	NA	PF01985.18^CRS1_YhbY^CRS1 / YhbY (CRM) domain^131-217^E:7.6e-16	0,082	-0,52	0	-1,455	0,36	-0,442	0,661	-0,225	0,911	0,061	.	.	.	.	.	.
SRR342019_TR30724_c0_g2_i1_len=845	PSAK_ARATH	Q:118-513,H:1-130	82.58%ID	E:1e-56	Photosystem I reaction center subunit psaK, chloroplastic	PF01241.15^PSI_PSAK^Photosystem I psaG / psaK^55-130^E:2.3e-24	0,734	-0,078	0	-1,078	0,016	-0,52	0,005	-0,613	0,774	-0,062	PSAK_ARATH	Photosystem I reaction center subunit psaK, chloroplastic (PSI-K) (Photosystem I subunit X)	PSAK At1g30380 T4K22.2	Arabidopsis thaliana (Mouse-ear cress)		Chloroplast; Complete proteome; Membrane; Photosynthesis; Photosystem I; Plastid; Reference proteome; Thylakoid; Transit peptide; Transmembrane; Transmembrane helix
SRR342019_TR30862_c0_g3_i3_len=1169	FK162_ARATH	Q:902-393,H:48-217	74.12%ID	E:3e-88	.	PF00254.25^FKBP_C^FKBP-type peptidyl-prolyl cis-trans isomerase^112-205^E:5.3e-25	0,212	-0,352	0	-1,36	0,001	-1,281	0,013	-0,989	1	-0,176	PNSL4_ARATH	Photosynthetic NDH subunit of lumenal location 4, chloroplastic (FK506-binding protein 16-2) (AtFKBP16-2) (Immunophilin FKBP16-2) (Peptidyl-prolyl cis-trans isomerase FKBP16-2) (PPIase FKBP16-2) (EC 5.2.1.8) (Rotamase)	PNSL4 FKBP16-2 FKBP22-2 FKBP23I At4g39710 T19P19.100	Arabidopsis thaliana (Mouse-ear cress)		Alternative splicing; Chloroplast; Complete proteome; Disulfide bond; Isomerase; Plastid; Reference proteome; Rotamase; Thylakoid; Transit peptide; Transport
SRR342019_TR31196_c0_g1_i1_len=1664	DR100_ARATH	Q:1330-311,H:31-370	63.34%ID	E:4e-148	DNA-damage-repair/toleration protein DRT100	PF08263.9^LRRNT_2^Leucine rich repeat N-terminal domain^108-147^E:1.1e-09`PF13855.3^LRR_8^Leucine rich repeat^214-274^E:2.6e-07`PF13516.3^LRR_6^Leucine Rich repeat^214-228^E:1.5`PF00560.30^LRR_1^Leucine Rich Repeat^215-237^E:1.2`PF00560.30^LRR_1^Leucine Rich Repeat^263-285^E:0.24`PF13516.3^LRR_6^Leucine Rich repeat^263-277^E:2`PF00560.30^LRR_1^Leucine Rich Repeat^386-403^E:1`PF00560.30^LRR_1^Leucine Rich Repeat^410-426^E:1.3	0,012	-0,713	0	-2,564	0,008	-1,419	0,115	-0,747	0,002	-1,163	DR100_ARATH	DNA-damage-repair/toleration protein DRT100	DRT100 At3g12610 MMF12.5 T2E22.8 T2E22_107	Arabidopsis thaliana (Mouse-ear cress)		Complete proteome; DNA damage; DNA repair; Leucine-rich repeat; Reference proteome; Repeat; Signal
SRR342019_TR31639_c1_g2_i1_len=809	PRE5_ARATH	Q:595-317,H:1-92	73.12%ID	E:4e-30	Transcription factor PRE5	.	0,253	-0,488	0	-2,469	0,023	-1,806	0,489	-0,548	0,012	-1,022	PRE5_ARATH	Transcription factor PRE5 (Basic helix-loop-helix protein 164) (AtbHLH164) (bHLH 164) (Protein PACLOBUTRAZOL RESISTANCE 5) (bHLH transcription factor bHLH164)	PRE5 BHLH164 At3g28857 MLD15.1	Arabidopsis thaliana (Mouse-ear cress)		Complete proteome; Gibberellin signaling pathway; Growth regulation; Reference proteome; Transcription; Transcription regulation
SRR342019_TR32628_c5_g1_i2_len=1329	RSSA_DAUCA	Q:167-946,H:1-258	79.31%ID	E:2e-138	40S ribosomal protein SA {ECO:0000255|HAMAP-Rule:MF_03015}	PF00318.17^Ribosomal_S2^Ribosomal protein S2^18-112^E:1.3e-12`PF00318.17^Ribosomal_S2^Ribosomal protein S2^117-182^E:1.9e-12	1	-1,535	0	-9,495	1	-0,012	0,192	-0,364	1	0,327	RSSA_DAUCA	40S ribosomal protein SA (p40)	179B	Daucus carota (Wild carrot)		Cytoplasm; Ribonucleoprotein; Ribosomal protein
SRR342019_TR32931_c0_g2_i3_len=601	UN932_ARATH	Q:54-530,H:284-443	73.75%ID	E:2e-64	UNC93-like protein 2	.	0,005	-6,195	0	-6,691	1	0,003	1	0,153	0,177	-0,687	UN932_ARATH	UNC93-like protein 2	At1g18010 T10F20.2	Arabidopsis thaliana (Mouse-ear cress)		Complete proteome; Membrane; Reference proteome; Transmembrane; Transmembrane helix
SRR342019_TR34744_c1_g4_i5_len=2731	CHMO_ARATH	Q:1308-340,H:97-419	68.83%ID	E:3e-158	Choline monooxygenase, chloroplastic	PF00657.19^Lipase_GDSL^GDSL-like Lipase/Acylhydrolase^52-354^E:3.9e-16	0,154	-0,551	0	-6,839	0,001	-3,614	0,001	-4,238	0,474	-0,368	CHMO_ARATH	Choline monooxygenase, chloroplastic (EC 1.14.15.7)	At4g29890 F27B13.130	Arabidopsis thaliana (Mouse-ear cress)	PATHWAY: Amine and polyamine biosynthesis; betaine biosynthesis via choline pathway; betaine aldehyde from choline (monooxygenase route): step 1/1.	2Fe-2S; Chloroplast; Complete proteome; Iron; Iron-sulfur; Magnesium; Metal-binding; Monooxygenase; Oxidoreductase; Plastid; Reference proteome; Transit peptide
SRR342019_TR35003_c2_g4_i2_len=2093	CP41A_ARATH	Q:819-226,H:208-405	83.84%ID	E:1e-100	Chloroplast stem-loop binding protein of 41 kDa a, chloroplastic	.	0,886	0,156	0	-1,748	0,079	-0,867	0,419	-0,385	1	0,244	CP41A_ARATH	Chloroplast stem-loop binding protein of 41 kDa a, chloroplastic (CSP41-a)	CSP41A At3g63140 T20O10.240	Arabidopsis thaliana (Mouse-ear cress)		Chloroplast; Complete proteome; Phosphoprotein; Plastid; RNA-binding; Reference proteome; Transit peptide; rRNA processing; rRNA-binding
SRR342019_TR35027_c1_g1_i1_len=972	UCRIA_SOLTU	Q:970-302,H:9-230	79.91%ID	E:8e-110	Cytochrome b6-f complex iron-sulfur subunit, chloroplastic	PF08802.7^CytB6-F_Fe-S^Cytochrome B6-F complex Fe-S subunit^50-88^E:8.3e-18`PF00355.23^Rieske^Rieske [2Fe-2S] domain^130-190^E:2.8e-12	0,016	0,636	0	-1,212	0,291	-0,237	0,099	-0,373	1	-1,584	UCRIA_SOLTU	Cytochrome b6-f complex iron-sulfur subunit, chloroplastic (EC 1.10.9.1) (Plastohydroquinone:plastocyanin oxidoreductase iron-sulfur protein) (Rieske iron-sulfur protein) (ISP) (RISP)	petC	Solanum tuberosum (Potato)		2Fe-2S; Chloroplast; Complete proteome; Disulfide bond; Electron transport; Iron; Iron-sulfur; Membrane; Metal-binding; Oxidoreductase; Plastid; Reference proteome; Thylakoid; Transit peptide; Transmembrane; Transmembrane helix; Transport

SRR342019_TR35308_c7_g9_i5_len=3747	LHTL8_ARATH	Q:1944-3509,H:1-517	89.87%ID	E:0	Lysine histidine transporter-like 8	PF00332.15^Glyco_hydro_17^Glycosyl hydrolases family 17^59-378^E:2.2e-78`PF07983.10^X8^X8 domain^396-467^E:2.2e-21	0,275	-0,386	0	-2,114	0,282	1,174	0,692	-0,96	1	0	LHTL8_ARATH	Lysine histidine transporter-like 8 (Amino acid transporter-like protein 1)	AATL1 At1g47670 F16N3.4	Arabidopsis thaliana (Mouse-ear cress)		Amino-acid transport; Cell membrane; Complete proteome; Membrane; Reference proteome; Transmembrane; Transmembrane helix; Transport
SRR342019_TR35455_c0_g1_i2_len=670	NA	NA	NA	NA	NA	.	0,151	-0,372	0	-2,004	0,926	-0,032	0,685	-0,104	1	1,672	.	.	.	.	.	.
SRR342019_TR36508_c0_g3_i1_len=975	DNAJ8_ARATH	Q:516-767,H:55-137	64.71%ID	E:6e-29	Chaperone protein dnaJ 8, chloroplastic	PF00226.28^DnaJ^DnaJ domain^43-96^E:2.1e-15	1	-0,806	0	-5,83	0	2,313	0	1,3	1	1,373	DNAJ8_ARATH	Chaperone protein dnaJ 8, chloroplastic (AtDjC8) (AtJ8)	ATJ8 C8 J8 At1g80920 F23A5.28	Arabidopsis thaliana (Mouse-ear cress)		Chaperone; Chloroplast; Complete proteome; Plastid; Reference proteome; Transit peptide
SRR342019_TR36688_c0_g2_i4_len=734	RS12_HORVU	Q:215-586,H:20-143	83.06%ID	E:2e-61	40S ribosomal protein S12	PF01248.23^Ribosomal_L7Ae^Ribosomal protein L7Ae/L30e/S12e/Gadd45 family^27-120^E:1.9e-26	1	0	0	-7,844	0,86	0,27	0,545	-0,544	1	0	RS12_HORVU	40S ribosomal protein S12	RPS12	Hordeum vulgare (Barley)		Ribonucleoprotein; Ribosomal protein
SRR342019_TR37672_c0_g1_i1_len=973	NA	NA	NA	NA	NA	.	0,28	-0,313	0	-1,504	1	0,034	0,233	0,444	0,005	-0,832	.	.	.	.	.	.
SRR342019_TR39291_c0_g1_i9_len=2822	AMSH2_ARATH	Q:639-187,H:74-223	64.24%ID	E:8e-64	AMSH-like ubiquitin thioesterase 2	.	1	0,023	0	-1,889	0,147	-0,93	0,214	-0,809	1	1,405	AMSH2_ARATH	AMSH-like ubiquitin thioesterase 2 (EC 3.4.19.-) (Deubiquitinating enzyme AMSH2)	AMSH2 At1g10600 F20B24.2 T10O24.25	Arabidopsis thaliana (Mouse-ear cress)		Alternative splicing; Complete proteome; Hydrolase; Metal-binding; Metalloprotease; Protease; Reference proteome; Ubl conjugation pathway; Zinc
SRR342022_TR145_c0_g1_i5_len=1370	HACD2_BOVIN	Q:1246-749,H:37-201	36.14%ID	E:9e-26	Very-long-chain (3R)-3-hydroxyacyl-CoA dehydratase 2 {ECO:0000305}	PF04387.11^PTPLA^Protein tyrosine phosphatase-like protein, PTPLA^52-210^E:2.4e-43	0,004	-0,962	0	-1,841	0,142	-0,817	0,326	-0,523	0,16	-0,524	HACD2_BOVIN	Very-long-chain (3R)-3-hydroxyacyl-CoA dehydratase 2 (EC 4.2.1.134) (3-hydroxyacyl-CoA dehydratase 2) (HACD2) (Protein-tyrosine phosphatase-like member B)	HACD2 PTPLB	Bos taurus (Bovine)	PATHWAY: Lipid metabolism; fatty acid biosynthesis. {ECO:0000250|UniProtKB:Q6Y1H2}.	Acetylation; Complete proteome; Endoplasmic reticulum; Fatty acid biosynthesis; Fatty acid metabolism; Glycoprotein; Lipid biosynthesis; Lipid metabolism; Lyase; Membrane; Reference proteome; Transmembrane; Transmembrane helix
SRR342022_TR249_c0_g1_i1_len=1000	CB23_SOLLC	Q:307-819,H:95-265	95.32%ID	E:4e-113	Chlorophyll a-b binding protein 13, chloroplastic	PF00504.18^Chloroa_b-bind^Chlorophyll A-B binding protein^1-128^E:9.8e-30	0,503	-0,208	0	-1,111	0,002	-0,784	0,065	-0,451	0,181	0,282	CB23_SOLLC	Chlorophyll a-b binding protein 13, chloroplastic (LHCII type III CAB-13)	CAB13 LHBC1	Solanum lycopersicum (Tomato) (Lycopersicon esculentum)		Chlorophyll; Chloroplast; Chromophore; Complete proteome; Direct protein sequencing; Magnesium; Membrane; Metal-binding; Phosphoprotein; Photosynthesis; Photosystem I; Photosystem II; Plastid; Reference proteome; Thylakoid; Transit peptide; Transmembrane; Transmembrane helix
SRR342022_TR351_c0_g1_i1_len=1274	NA	NA	NA	NA	NA	.	0,76	-0,682	0	-2,824	1	-0,079	1	-1,22	1	0,774	.	.	.	.	.	.
SRR342022_TR895_c1_g4_i1_len=1434	TOC34_PEA	Q:177-1070,H:6-302	72.82%ID	E:2e-154	Translocase of chloroplast 34	PF03193.13^DUF258^Protein of unknown function, DUF258^24-97^E:1.9e-08`PF04548.13^AIG1^AIG1 family^38-228^E:2.4e-36`PF02421.15^FeoB_N^Ferrous iron transport protein B^39-129^E:5e-05`PF01926.20^MMR_HSR1^50S ribosome-binding GTPase^40-142^E:2.1e-12	0,004	-0,873	0	-1,413	0,832	-0,26	1	0,087	0,207	-0,378	TOC34_PEA	Translocase of chloroplast 34 (EC 3.6.5.-) (34 kDa chloroplast outer envelope protein) (GTP-binding protein IAP34) (GTP-binding protein OEP34)	TOC34 IAP34 OEP34	Pisum sativum (Garden pea)		3D-structure; Chloroplast; Direct protein sequencing; GTP-binding; Hydrolase; Membrane; Nucleotide-binding; Plastid; Plastid outer membrane; Protein transport; Transmembrane; Transmembrane helix; Transport
SRR342022_TR985_c7_g1_i1_len=651	RCA_MALDO	Q:599-366,H:359-436	76.92%ID	E:4e-35	Ribulose bisphosphate carboxylase/oxygenase activase, chloroplastic	.	0,059	-0,615	0	-2,092	0,121	0,329	0,945	0,016	0,012	-0,61	RCA_MALDO	Ribulose bisphosphate carboxylase/oxygenase activase, chloroplastic (RA) (RuBisCO activase)	RCA	Malus domestica (Apple) (Pyrus malus)		ATP-binding; Chloroplast; Nucleotide-binding; Plastid; Transit peptide
SRR342022_TR1215_c4_g12_i1_len=2882	AVP_VIGRR	Q:156-2462,H:1-766	90.9%ID	E:0	Pyrophosphate-energized vacuolar membrane proton pump	PF03030.13^H_PPase^Inorganic H+ pyrophosphatase^21-754^E:8.5e-260	1	0,059	0	-1,414	0,89	0,074	0,89	0,081	0,228	0,98	AVP_VIGRR	Pyrophosphate-energized vacuolar membrane proton pump (EC 3.6.1.1) (Pyrophosphate-energized inorganic pyrophosphatase) (H(+)-PPase) (Vacuolar H(+)-pyrophosphatase)		Vigna radiata var. radiata (Mung bean) (Phaseolus aureus)		3D-structure; Direct protein sequencing; Disulfide bond; Hydrogen ion transport; Hydrolase; Ion transport; Magnesium; Membrane; Metal-binding; Transmembrane; Transmembrane helix; Transport; Vacuole
SRR342022_TR1398_c0_g1_i1_len=2667	MFP1_TOBAC	Q:237-2252,H:1-690	52.88%ID	E:0	MAR-binding filament-like protein 1-1	.	0,216	-0,387	0	-1,385	0,095	-0,614	0,436	-0,298	0,176	0,439	MFP1_TOBAC	MAR-binding filament-like protein 1-1	MFP1-1	Nicotiana tabacum (Common tobacco)		Coiled coil; DNA-binding; Nucleus
SRR342022_TR1434_c0_g1_i1_len=1884	NA	NA	NA	NA	NA	.	0,35	-0,61	0	-3,346	0,004	-2,421	0,004	-2,477	0,712	-0,135	.	.	.	.	.	.
SRR342022_TR1885_c0_g1_i3_len=522	MCCB_ARATH	Q:471-232,H:508-587	68.75%ID	E:3e-29	Methylcrotonoyl-CoA carboxylase beta chain, mitochondrial	.	1	0	0,001	-1,904	1	0	1	0	1	0	MCCB_ARATH	Methylcrotonoyl-CoA carboxylase beta chain, mitochondrial (MCCase subunit beta) (EC 6.4.1.4) (3-methylcrotonyl-CoA carboxylase 2) (3-methylcrotonyl-CoA:carbon dioxide ligase subunit beta)	MCCB At4g34030 F17I5.220 F28A23.210	Arabidopsis thaliana (Mouse-ear cress)	PATHWAY: Amino-acid degradation; L-leucine degradation; (S)-3-hydroxy-3-methylglutaryl-CoA from 3-isovaleryl-CoA: step 2/3.	ATP-binding; Complete proteome; Ligase; Mitochondrion; Nucleotide-binding; Reference proteome; Transit peptide
SRR342022_TR2238_c7_g1_i10_len=2909	FPG_ARATH	Q:138-1016,H:1-294	79.25%ID	E:5e-148	Formamidopyrimidine-DNA glycosylase	PF01149.21^Fapy_DNA_glyco^Formamidopyrimidine-DNA glycosylase N-terminal domain^1-131^E:2.2e-28`PF06831.11^H2TH^Formamidopyrimidine-DNA glycosylase H2TH domain^146-235^E:3.5e-29	1	-1,49	0	-6,199	0,016	-2,629	0,016	-2,287	1	0	FPG_ARATH	Formamidopyrimidine-DNA glycosylase (Fapy-DNA glycosylase) (EC 3.2.2.23) (EC 4.2.99.18) (DNA-(apurinic or apyrimidinic site) lyase FPG1) (Formamidopyrimidine-DNA glycosylase 1) (AtFPG-1) (Formamidopyrimidine-DNA glycosylase 2) (AtFPG-2) (Protein MutM homolog 1) (AtMMH-1) (Protein MutM homolog 2) (AtMMH-2)	FPG1 FPG2 At1g52500 F6D8.28	Arabidopsis thaliana (Mouse-ear cress)		3D-structure; Alternative splicing; Complete proteome; DNA damage; DNA repair; DNA-binding; Glycosidase; Hydrolase; Lyase; Multifunctional enzyme; Nucleus; Reference proteome
SRR342022_TR2436_c1_g9_i7_len=1121	EXPA4_ARATH	Q:1120-563,H:72-257	89.25%ID	E:6e-121	Expansin-A4	PF03330.15^DPBB_1^Lytic transglycolase^1-82^E:1.4e-19`PF01357.18^Pollen_allerg_1^Pollen allergen^93-170^E:9.3e-25	0,796	-0,082	0	-2,513	1	0,061	0,169	-0,62	1	-0,016	EXPA4_ARATH	Expansin-A4 (AtEXPA4) (Alpha-expansin-4) (At-EXP4) (AtEx4) (Ath-ExpAlpha-1.6)	EXPA4 EXP4 At2g39700 F17A14.7	Arabidopsis thaliana (Mouse-ear cress)		Cell wall; Cell wall biogenesis/degradation; Complete proteome; Direct protein sequencing; Disulfide bond; Membrane; Reference proteome; Secreted; Signal
SRR342022_TR2829_c1_g3_i17_len=3033	Y3471_ARATH	Q:2322-1588,H:347-589	48.57%ID	E:2e-67	Putative receptor-like protein kinase At3g47110	PF00560.30^LRR_1^Leucine Rich Repeat^47-69^E:1.1`PF13855.3^LRR_8^Leucine rich repeat^71-131^E:1.7e-07`PF00560.30^LRR_1^Leucine Rich Repeat^71-93^E:0.089`PF13516.3^LRR_6^Leucine Rich repeat^92-108^E:0.34`PF00560.30^LRR_1^Leucine Rich Repeat^95-113^E:0.024`PF13855.3^LRR_8^Leucine rich repeat^122-177^E:2.6e-06`PF00560.30^LRR_1^Leucine Rich Repeat^168-189^E:0.18`PF00560.30^LRR_1^Leucine Rich Repeat^194-214^E:0.41	0,763	-0,225	0	-2,762	0,001	-1,016	0,001	-1,025	0,173	-0,674	Y3471_ARATH	Putative receptor-like protein kinase At3g47110 (EC 2.7.11.1)	At3g47110 F13I12.160	Arabidopsis thaliana (Mouse-ear cress)		ATP-binding; Cell membrane; Complete proteome; Glycoprotein; Kinase; Leucine-rich repeat; Membrane; Nucleotide-binding; Phosphoprotein; Receptor; Reference proteome; Repeat; Serine/threonine-protein kinase; Signal; Transferase; Transmembrane; Transmembrane helix
SRR342022_TR2901_c4_g1_i6_len=897	NA	NA	NA	NA	NA	.	1	0	0,001	-1,653	0,679	0,48	1	-0,184	1	0	.	.	.	.	.	.
SRR342022_TR3295_c2_g6_i3_len=825	NA	NA	NA	NA	NA	PF01165.17^Ribosomal_S21^Ribosomal protein S21^96-150^E:3e-19	0,114	-0,421	0	-1,009	0,05	-0,582	0,146	-0,423	0,527	0,152	.	.	.	.	.	.
SRR342022_TR4532_c1_g6_i1_len=922	RK35_SPIOL	Q:259-549,H:60-158	73.74%ID	E:7e-29	50S ribosomal protein L35, chloroplastic	PF01632.16^Ribosomal_L35p^Ribosomal protein L35^83-139^E:4.3e-20	0,018	-0,544	0	-0,873	0,007	-0,618	0,114	-0,357	0,056	-0,428	RK35_SPIOL	50S ribosomal protein L35, chloroplastic (CL35)	RPL35	Spinacia oleracea (Spinach)		3D-structure; Chloroplast; Direct protein sequencing; Plastid; Ribonucleoprotein; Ribosomal protein; Transit peptide
SRR342022_TR4639_c0_g1_i16_len=3762	PHOT1_ARATH	Q:428-3502,H:3-996	69.84%ID	E:0	Phototropin-1	PF13426.4^PAS_9^PAS domain^215-311^E:1.5e-20`PF00989.22^PAS^PAS fold^221-306^E:1.7e-08`PF08447.9^PAS_3^PAS fold^222-305^E:1.5e-08`PF13426.4^PAS_9^PAS domain^510-602^E:3.5e-20`PF00989.22^PAS^PAS fold^513-602^E:1.1e-06`PF08447.9^PAS_3^PAS fold^514-599^E:4.2e-07`PF00069.22^Pkinase^Protein kinase domain^690-975^E:1.3e-62`PF07714.14^Pkinase_Tyr^Protein tyrosine kinase^691-959^E:1.4e-26	0,438	-0,325	0	-2,257	0,174	-0,583	0,586	-0,245	0,568	-0,248	PHOT1_ARATH	Phototropin-1 (EC 2.7.11.1) (Non-phototropic hypocotyl protein 1) (Root phototropism protein 1)	PHOT1 JK224 NPH1 RPT1 At3g45780 T6D9_110	Arabidopsis thaliana (Mouse-ear cress)		3D-structure; ATP-binding; Cell membrane; Chromophore; Complete proteome; Cytoplasm; Disulfide bond; FMN; Flavoprotein; Kinase; Membrane; Nucleotide-binding; Phosphoprotein; Photoreceptor protein; Receptor; Reference proteome; Repeat; Sensory transduction; Serine/threonine-protein kinase; Transferase
SRR342022_TR4728_c1_g1_i5_len=1841	PTR6_ARATH	Q:1707-274,H:106-594	59.1%ID	E:0	Protein NRT1/ PTR FAMILY 1.2	PF00854.18^PTR2^POT family^3-410^E:2.6e-73	0,096	-0,679	0	-8,539	0,228	1,223	0,461	-1,455	1	0,71	PTR6_ARATH	Protein NRT1/ PTR FAMILY 1.2 (AtNPF1.2) (Nitrate transporter 1.11)	NPF1.2 NRT1.11 At1g52190 F9I5.4	Arabidopsis thaliana (Mouse-ear cress)		Cell membrane; Complete proteome; Membrane; Phosphoprotein; Reference proteome; Transmembrane; Transmembrane helix
SRR342022_TR5134_c0_g1_i2_len=2001	LCYE_ARATH	Q:179-1762,H:1-524	73.83%ID	E:0	Lycopene epsilon cyclase, chloroplastic	PF05834.9^Lycopene_cycl^Lycopene cyclase protein^113-505^E:2.3e-152	1	-0,02	0	-1,318	0,02	-0,782	0,073	-0,586	0,091	0,537	LCYE_ARATH	Lycopene epsilon cyclase, chloroplastic (EC 5.5.1.18) (Protein LUTEIN DEFICIENT 2)	LUT2 At5g57030 MHM17.16	Arabidopsis thaliana (Mouse-ear cress)	PATHWAY: Carotenoid biosynthesis; alpha-zeacarotene biosynthesis.; PATHWAY: Carotenoid biosynthesis; delta-carotene biosynthesis.	Carotenoid biosynthesis; Chloroplast; Complete proteome; Isomerase; Membrane; NAD; Plastid; Reference proteome; Transit peptide; Transmembrane; Transmembrane helix
SRR342022_TR5277_c0_g1_i1_len=655	NA	NA	NA	NA	NA	.	1	-0,297	0	-7,73	0,515	0,211	0,91	0,047	1	0	.	.	.	.	.	.
SRR342022_TR5356_c1_g2_i1_len=1588	LPA3_ARATH	Q:260-1258,H:26-358	76.58%ID	E:0	Protein LOW PSII ACCUMULATION 3, chloroplastic	PF09353.7^DUF1995^Domain of unknown function (DUF1995)^113-375^E:5.2e-50	1	-0,405	0	-5,591	0,007	-0,859	0,021	-0,734	0,822	-0,307	LPA3_ARATH	Protein LOW PSII ACCUMULATION 3, chloroplastic	LPA3 At1g73060 F3N23.26	Arabidopsis thaliana (Mouse-ear cress)		Acetylation; Chloroplast; Complete proteome; Membrane; Plastid; Reference proteome; Thylakoid; Transit peptide
SRR342022_TR5371_c0_g3_i1_len=1044	RK12_TOBAC	Q:118-684,H:1-186	81.58%ID	E:9e-80	50S ribosomal protein L12, chloroplastic	PF16320.2^Ribosomal_L12_N^Ribosomal protein L7/L12 dimerisation domain^62-109^E:1.5e-13`PF00542.16^Ribosomal_L12^Ribosomal protein L7/L12 C-terminal domain^122-188^E:1.2e-23	0,048	-0,437	0	-1,282	0,056	-0,442	0,244	-0,27	0,196	-0,279	RK12_TOBAC	50S ribosomal protein L12, chloroplastic (CL12)	RPL12	Nicotiana tabacum (Common tobacco)		Chloroplast; Direct protein sequencing; Plastid; Ribonucleoprotein; Ribosomal protein; Transit peptide
SRR342022_TR6523_c1_g1_i1_len=1070	TL17_ARATH	Q:897-301,H:30-236	68.6%ID	E:2e-99	Thylakoid lumenal 17.4 kDa protein, chloroplastic {ECO:0000303|PubMed:9654141}	PF00805.19^Pentapeptide^Pentapeptide repeats (8 copies)^122-161^E:5.1e-05`PF00805.19^Pentapeptide^Pentapeptide repeats (8 copies)^156-186^E:1.9e-07`PF13599.3^Pentapeptide_4^Pentapeptide repeats (9 copies)^157-216^E:3.9e-08`PF00805.19^Pentapeptide^Pentapeptide repeats (8 copies)^167-205^E:5.8e-08	0,839	-0,115	0	-1,721	1	0,087	1	0,04	0,076	-2,62	TL17_ARATH	Thylakoid lumenal 17.4 kDa protein, chloroplastic (P17.4)	TL17 At5g53490 MNC6.3	Arabidopsis thaliana (Mouse-ear cress)		Alternative splicing; Chloroplast; Complete proteome; Direct protein sequencing; Plastid; Reference proteome; Repeat; Thylakoid; Transit peptide
SRR342022_TR6614_c3_g2_i2_len=1398	RRP2_SPIOL	Q:405-947,H:80-260	75.69%ID	E:8e-79	30S ribosomal protein 2, chloroplastic	PF00076.19^RRM_1^RNA recognition motif. (a.k.a. RRM, RBD, or RNP domain)^81-151^E:1.2e-21`PF13893.3^RRM_5^RNA recognition motif. (a.k.a. RRM, RBD, or RNP domain)^87-155^E:0.006`PF00076.19^RRM_1^RNA recognition motif. (a.k.a. RRM, RBD, or RNP domain)^181-251^E:2.6e-17`PF13893.3^RRM_5^RNA recognition motif. (a.k.a. RRM, RBD, or RNP domain)^220-252^E:0.2	0,184	-0,312	0	-0,889	0,058	-0,478	0,449	-0,193	0,495	-0,161	RRP2_SPIOL	30S ribosomal protein 2, chloroplastic (Plastid-specific 30S ribosomal protein 2) (PSRP-2)	PSRP2	Spinacia oleracea (Spinach)		Chloroplast; Direct protein sequencing; Plastid; RNA-binding; Repeat; Ribonucleoprotein; Ribosomal protein; Transit peptide; rRNA-binding
SRR342022_TR6664_c0_g7_i1_len=616	NA	NA	NA	NA	NA	.	0,161	-0,352	0	-1,685	0,001	-0,751	0,004	-0,677	0,226	-2,074	.	.	.	.	.	.
SRR342022_TR7233_c0_g3_i1_len=1851	DNAJ_CYAP4	Q:532-1569,H:2-343	54.31%ID	E:6e-114	Chaperone protein DnaJ {ECO:0000255|HAMAP-Rule:MF_01152}	PF00226.28^DnaJ^DnaJ domain^74-135^E:1.5e-26`PF01556.15^DnaJ_C^DnaJ C terminal domain^194-411^E:3.9e-32`PF00684.16^DnaJ_CXXCXGXG^DnaJ central domain^220-284^E:2.5e-12	1	0,684	0	-7,206	0,941	0,062	0,438	0,28	1	0	DNAJ_CYAP4	Chaperone protein DnaJ	dnaJ Cyan7425_4692	Cyanothece sp. (strain PCC 7425 / ATCC 29141)		Chaperone; Complete proteome; Cytoplasm; DNA replication; Metal-binding; Reference proteome; Repeat; Stress response; Zinc; Zinc-finger
SRR342022_TR7506_c0_g1_i1_len=1191	RR6_ARATH	Q:199-756,H:17-206	73.16%ID	E:1e-73	30S ribosomal protein S6 alpha, chloroplastic	PF01250.14^Ribosomal_S6^Ribosomal protein S6^96-194^E:7.2e-19	0,031	-0,516	0	-1,195	0,346	-0,255	0,773	-0,076	0	6,374	RR6_ARATH	30S ribosomal protein S6 alpha, chloroplastic	RPS6 At1g64510 F1N19.8	Arabidopsis thaliana (Mouse-ear cress)		Chloroplast; Complete proteome; Plastid; RNA-binding; Reference proteome; Ribonucleoprotein; Ribosomal protein; Transit peptide; rRNA-binding
SRR342022_TR7764_c0_g1_i1_len=1561	UGPI7_ARATH	Q:333-1145,H:21-278	73.82%ID	E:1e-121	Uncharacterized GPI-anchored protein At4g28100	.	0,502	-0,254	0	-2,201	0,094	-1,157	0,007	-2,088	1	0,005	UGPI7_ARATH	Uncharacterized GPI-anchored protein At4g28100	At4g28100 T13J8.210	Arabidopsis thaliana (Mouse-ear cress)		Cell membrane; Complete proteome; GPI-anchor; Glycoprotein; Lipoprotein; Membrane; Reference proteome; Signal
SRR342022_TR7920_c1_g1_i1_len=1955	LAX3_MEDTR	Q:1698-283,H:1-465	86.23%ID	E:0	Auxin transporter-like protein 3	PF01490.15^Aa_trans^Transmembrane amino acid transporter protein^44-434^E:3e-73	0,43	-0,312	0	-2,024	0,257	-0,799	0,175	-1,024	0,844	0,114	LAX3_MEDTR	Auxin transporter-like protein 3 (AUX1-like protein 3) (MtLAX3)	LAX3	Medicago truncatula (Barrel medic) (Medicago tribuloides)		Amino-acid transport; Auxin signaling pathway; Cell membrane; Membrane; Symport; Transmembrane; Transmembrane helix; Transport
SRR342022_TR8568_c2_g10_i2_len=596	RS12_HORVU	Q:416-45,H:20-143	83.06%ID	E:3e-62	40S ribosomal protein S12	PF01248.23^Ribosomal_L7Ae^Ribosomal protein L7Ae/L30e/S12e/Gadd45 family^27-120^E:1.9e-26	1	1,156	0	-8,422	0,794	0,072	0,395	-0,233	1	-0,038	RS12_HORVU	40S ribosomal protein S12	RPS12	Hordeum vulgare (Barley)		Ribonucleoprotein; Ribosomal protein
SRR342022_TR8866_c3_g12_i2_len=2962	P2C71_ORYSJ	Q:965-99,H:162-463	50.99%ID	E:1e-93	Probable protein phosphatase 2C 71	PF07228.9^SpoIIE^Stage II sporulation protein E (SpoIIE)^337-501^E:9.1e-07`PF13672.3^PP2C_2^Protein phosphatase 2C^352-453^E:9.9e-08`PF00481.18^PP2C^Protein phosphatase 2C^419-469^E:4e-06	1	0,947	0,001	-6,653	1	-3,659	1	-3,659	0,461	-1,197	P2C71_ORYSJ	Probable protein phosphatase 2C 71 (OsPP2C71) (EC 3.1.3.16)	Os10g0370000 LOC_Os10g22460 OJ1003C07.6 OsJ_030057	Oryza sativa subsp. japonica (Rice)		Complete proteome; Hydrolase; Magnesium; Manganese; Metal-binding; Protein phosphatase; Reference proteome
SRR342022_TR9396_c5_g9_i2_len=1050	NA	NA	NA	NA	NA	.	1	0,003	0,001	-1,032	0,838	0,057	0,374	0,224	1	0,962	.	.	.	.	.	.
SRR342022_TR9794_c0_g7_i1_len=3160	C82A2_SOYBN	Q:1999-2550,H:336-520	54.59%ID	E:1e-57	Cytochrome P450 82A2	PF00067.19^p450^Cytochrome P450^35-288^E:5.5e-30	0,702	0,155	0	-2,556	0,381	-2,437	0,129	-3,164	1	0	C82A2_SOYBN	Cytochrome P450 82A2 (EC 1.14.-.-) (Cytochrome P450 CP4)	CYP82A2	Glycine max (Soybean) (Glycine hispida)		Complete proteome; Heme; Iron; Membrane; Metal-binding; Monooxygenase; Oxidoreductase; Reference proteome
SRR342022_TR9794_c0_g7_i2_len=2242	C82A3_SOYBN	Q:187-1632,H:39-525	47.26%ID	E:4e-130	Cytochrome P450 82A3	PF00067.19^p450^Cytochrome P450^35-492^E:2.3e-92	0,715	0,139	0	-2,727	0,692	-0,937	0,381	-1,615	1	-0,218	C82A3_SOYBN	Cytochrome P450 82A3 (EC 1.14.-.-) (Cytochrome P450 CP6)	CYP82A3	Glycine max (Soybean) (Glycine hispida)		Complete proteome; Heme; Iron; Membrane; Metal-binding; Monooxygenase; Oxidoreductase; Reference proteome
SRR342022_TR10047_c1_g3_i1_len=838	HEXO1_ARATH	Q:47-484,H:396-541	67.81%ID	E:3e-61	Beta-hexosaminidase 1	PF00728.19^Glyco_hydro_20^Glycosyl hydrolase family 20, catalytic domain^12-93^E:2.2e-14	1	0	0	-7,427	1	-3,066	1	-3,066	0,16	-1,143	HEXO1_ARATH	Beta-hexosaminidase 1 (EC 3.2.1.52) (Beta-GlcNAcase 1) (Beta-N-acetylhexosaminidase 1) (Beta-hexosaminidase 2) (AtHEX2) (N-acetyl-beta-glucosaminidase 1)	HEXO1 HEX2 At3g55260 T26I12.140	Arabidopsis thaliana (Mouse-ear cress)		Complete proteome; Disulfide bond; Glycoprotein; Glycosidase; Hydrolase; Reference proteome; Signal; Vacuole
SRR342022_TR10137_c0_g1_i2_len=1546	NA	NA	NA	NA	NA	.	1	0,32	0	-7,891	0,917	0,112	0,548	0,294	1	0	.	.	.	.	.	.
SRR342022_TR10210_c2_g8_i16_len=2005	Y3589_ARATH	Q:755-1786,H:7-350	64.74%ID	E:4e-151	PTI1-like tyrosine-protein kinase At3g15890	PF00069.22^Pkinase^Protein kinase domain^40-307^E:6.9e-45`PF07714.14^Pkinase_Tyr^Protein tyrosine kinase^41-309^E:2.6e-44	0,76	-0,552	0	-3,754	0,074	0,875	0,241	0,646	1	-0,11	Y3589_ARATH	PTI1-like tyrosine-protein kinase At3g15890 (EC 2.7.10.2)	At3g15890 MVC8.1	Arabidopsis thaliana (Mouse-ear cress)		ATP-binding; Complete proteome; Kinase; Nucleotide-binding; Reference proteome; Transferase; Tyrosine-protein kinase
SRR342022_TR11257_c0_g2_i1_len=1049	ACFR3_ARATH	Q:123-767,H:2-217	56.02%ID	E:3e-59	Probable transmembrane ascorbate ferrireductase 3	PF03188.13^Cytochrom_B561^Eukaryotic cytochrome b561^57-188^E:3.3e-37	0,158	1,548	0	-6,953	0,066	-5,376	0,066	-5,376	0,025	-3,179	ACFR3_ARATH	Probable transmembrane ascorbate ferrireductase 3 (EC 1.16.5.1) (Artb561-3) (Protein b561A.tha14)	CYB561C At1g14730 F10B6.13	Arabidopsis thaliana (Mouse-ear cress)		Complete proteome; Electron transport; Heme; Iron; Membrane; Metal-binding; Oxidoreductase; Reference proteome; Transmembrane; Transmembrane helix; Transport
SRR342022_TR11375_c1_g2_i3_len=1074	PSBQ1_MAIZE	Q:496-882,H:89-217	75.19%ID	E:7e-44	Oxygen-evolving enhancer protein 3-1, chloroplastic	.	0,38	-0,514	0	-2,607	0,142	-0,849	0,044	-1,103	0,646	-0,365	PSBQ1_MAIZE	Oxygen-evolving enhancer protein 3-1, chloroplastic (OEE3) (16 kDa subunit of oxygen evolving system of photosystem II) (OEC 16 kDa subunit)	PSBQ1 PSBQ	Zea mays (Maize)		Chloroplast; Complete proteome; Membrane; Photosynthesis; Photosystem II; Plastid; Reference proteome; Thylakoid; Transit peptide
SRR342022_TR11511_c0_g2_i5_len=1289	SDF2_ARATH	Q:992-357,H:1-215	77.21%ID	E:3e-119	Stromal cell-derived factor 2-like protein	PF02815.16^MIR^MIR domain^165-297^E:4.5e-14	1	0,019	0	-8,501	1	0,055	0,516	-0,287	1	0	SDF2_ARATH	Stromal cell-derived factor 2-like protein (AtSDF2) (SDF2-like protein)	SDF2 At2g25110 F13D4.70	Arabidopsis thaliana (Mouse-ear cress)		3D-structure; Complete proteome; Endoplasmic reticulum; Glycoprotein; Plant defense; Reference proteome; Repeat; Signal
SRR342022_TR11911_c0_g3_i5_len=1427	NA	NA	NA	NA	NA	PF01190.14^Pollen_Ole_e_I^Pollen proteins Ole e I like^30-89^E:0.00017	0,002	-0,849	0	-2,356	0,493	-0,351	0,253	0,481	0,16	1,19	.	.	.	.	.	.
SRR342022_TR12216_c0_g3_i1_len=2241	SR54C_ARATH	Q:251-1843,H:35-564	84.02%ID	E:0	Signal recognition particle 54 kDa protein, chloroplastic	PF02881.16^SRP54_N^SRP54-type protein, helical bundle domain^91-168^E:1.2e-19`PF06414.9^Zeta_toxin^Zeta toxin^184-291^E:5e-06`PF00448.19^SRP54^SRP54-type protein, GTPase domain^186-381^E:3.5e-76`PF01583.17^APS_kinase^Adenylylsulphate kinase^186-227^E:5.6e-05`PF02374.12^ArsA_ATPase^Anion-transporting ATPase^188-223^E:5.4e-05`PF01656.20^CbiA^CobQ/CobB/MinD/ParA nucleotide binding domain^196-340^E:2e-06`PF02978.16^SRP_SPB^Signal peptide binding domain^412-510^E:2.8e-24	0,05	-0,507	0	-1,352	0,086	-0,571	0,461	-0,245	0,029	-0,554	SR54C_ARATH	Signal recognition particle 54 kDa protein, chloroplastic (54 chloroplast protein) (54CP) (SRP54) (cpSRP54) (FFC)	FFC CPSRP54 At5g03940 F8F6_150	Arabidopsis thaliana (Mouse-ear cress)		3D-structure; Chloroplast; Complete proteome; GTP-binding; Nucleotide-binding; Plastid; RNA-binding; Reference proteome; Ribonucleoprotein; Signal recognition particle; Transit peptide
SRR342022_TR12377_c0_g3_i2_len=984	COMT1_CATRO	Q:982-335,H:127-313	63.32%ID	E:4e-74	Caffeic acid 3-O-methyltransferase	PF00891.15^Methyltransf_2^O-methyltransferase^1-91^E:1.3e-30	0,935	-0,031	0	-1,718	0,798	0,455	0,76	-0,599	0,381	-0,827	COMT1_CATRO	Caffeic acid 3-O-methyltransferase (CAOMT) (COMT) (EC 2.1.1.68) (S-adenosysl-L-methionine:caffeic acid 3-O-methyltransferase)	COMT1	Catharanthus roseus (Madagascar periwinkle) (Vinca rosea)	PATHWAY: Aromatic compound metabolism; phenylpropanoid biosynthesis.	Lignin biosynthesis; Methyltransferase; S-adenosyl-L-methionine; Transferase
SRR342022_TR13012_c0_g3_i10_len=811	RCD1_DANRE	Q:78-362,H:180-274	65.26%ID	E:2e-36	Cell differentiation protein RCD1 homolog	.	1	0	0,001	-4,67	1	0	1	0	0,892	-0,169	RCD1_DANRE	Cell differentiation protein RCD1 homolog (Rcd-1)	rqcd1 rcd1 si:dkeyp-84a8.6 zgc:85618	Danio rerio (Zebrafish) (Brachydanio rerio)		Activator; Complete proteome; Cytoplasm; Nucleus; RNA-mediated gene silencing; Reference proteome; Repressor; Transcription; Transcription regulation; Translation regulation
SRR342022_TR13132_c0_g3_i1_len=1550	Y4372_ARATH	Q:2-1264,H:355-768	57.83%ID	E:2e-124	Probable LRR receptor-like serine/threonine-protein kinase At4g37250	PF00069.22^Pkinase^Protein kinase domain^116-408^E:1.2e-32`PF07714.14^Pkinase_Tyr^Protein tyrosine kinase^131-268^E:4.1e-21`PF07714.14^Pkinase_Tyr^Protein tyrosine kinase^306-408^E:2.7e-05	0,673	-0,237	0	-2,018	0,133	-1,282	0,038	-1,706	0,003	-2,356	Y4372_ARATH	Probable LRR receptor-like serine/threonine-protein kinase At4g37250 (EC 2.7.11.1)	At4g37250 AP22.22 C7A10.110	Arabidopsis thaliana (Mouse-ear cress)		ATP-binding; Complete proteome; Glycoprotein; Kinase; Leucine-rich repeat; Membrane; Nucleotide-binding; Phosphoprotein; Receptor; Reference proteome; Repeat; Serine/threonine-protein kinase; Signal; Transferase; Transmembrane; Transmembrane helix
SRR342022_TR13289_c1_g1_i1_len=2590	ADS3_ARATH	Q:834-253,H:174-367	84.54%ID	E:3e-125	Palmitoyl-monogalactosyldiacylglycerol delta-7 desaturase, chloroplastic {ECO:0000303|PubMed:15579662}	.	1	-0,22	0	-6,919	0,003	-2,836	0,003	-2,634	1	0	ADS3_ARATH	Palmitoyl-monogalactosyldiacylglycerol delta-7 desaturase, chloroplastic (EC 1.14.19.-) (FAD5) (Monogalactosyldiacylglycerol-specific palmitic acid desaturase)	ADS3 At3g15850 MSJ11.25	Arabidopsis thaliana (Mouse-ear cress)	PATHWAY: Lipid metabolism; oxylipin biosynthesis.; PATHWAY: Lipid metabolism; polyunsaturated fatty acid biosynthesis.	Chloroplast; Complete proteome; Fatty acid biosynthesis; Fatty acid metabolism; Iron; Lipid biosynthesis; Lipid metabolism; Membrane; Oxidoreductase; Oxylipin biosynthesis; Plastid; Reference proteome; Transit peptide; Transmembrane; Transmembrane helix
SRR342022_TR13289_c1_g1_i11_len=3058	ADS3_ARATH	Q:690-253,H:222-367	84.93%ID	E:6e-120	Palmitoyl-monogalactosyldiacylglycerol delta-7 desaturase, chloroplastic {ECO:0000303|PubMed:15579662}	.	1	-0,425	0	-9,249	0,389	-0,295	0,73	-0,124	1	0,364	ADS3_ARATH	Palmitoyl-monogalactosyldiacylglycerol delta-7 desaturase, chloroplastic (EC 1.14.19.-) (FAD5) (Monogalactosyldiacylglycerol-specific palmitic acid desaturase)	ADS3 At3g15850 MSJ11.25	Arabidopsis thaliana (Mouse-ear cress)	PATHWAY: Lipid metabolism; oxylipin biosynthesis.; PATHWAY: Lipid metabolism; polyunsaturated fatty acid biosynthesis.	Chloroplast; Complete proteome; Fatty acid biosynthesis; Fatty acid metabolism; Iron; Lipid biosynthesis; Lipid metabolism; Membrane; Oxidoreductase; Oxylipin biosynthesis; Plastid; Reference proteome; Transit peptide; Transmembrane; Transmembrane helix
SRR342022_TR13289_c1_g5_i2_len=2885	CAO_ORYSJ	Q:1088-606,H:375-535	81.99%ID	E:1e-84	Chlorophyllide a oxygenase, chloroplastic	.	0,298	-0,517	0	-1,725	0,007	-0,837	0,012	-0,759	1	0,253	CAO_ORYSJ	Chlorophyllide a oxygenase, chloroplastic (Chlorophyll a oxygenase) (EC 1.14.13.122) (Chlorophyll b synthase)	CAO LLS1 Os10g0567400 LOC_Os10g41780 OSJNBa0057L21.2	Oryza sativa subsp. japonica (Rice)		2Fe-2S; Alternative splicing; Chlorophyll biosynthesis; Chloroplast; Coiled coil; Complete proteome; Iron; Iron-sulfur; Membrane; Metal-binding; NADP; Oxidoreductase; Plastid; Reference proteome; Thylakoid; Transit peptide
SRR342022_TR14119_c0_g1_i1_len=952	NA	NA	NA	NA	NA	PF10716.6^NdhL^NADH dehydrogenase transmembrane subunit^94-166^E:4.1e-29	0,148	-0,391	0	-1,037	0,534	0,176	0,081	0,469	0,139	-0,382	.	.	.	.	.	.
SRR342022_TR14831_c1_g1_i2_len=2064	LBP65_ARATH	Q:245-520,H:33-128	63.54%ID	E:6e-35	Putative lipid-binding protein At4g00165	PF14547.3^Hydrophob_seed^Hydrophobic seed protein^70-139^E:7.2e-06	0,281	-0,42	0,001	-1,218	0,212	-0,958	0,707	-0,435	0,472	-0,631	LBP65_ARATH	Putative lipid-binding protein At4g00165	At4g00165 F6N15.21	Arabidopsis thaliana (Mouse-ear cress)		Complete proteome; Disulfide bond; Reference proteome; Secreted; Signal
SRR342022_TR16045_c6_g10_i4_len=1010	PSAL_ARATH	Q:824-234,H:23-219	82.74%ID	E:3e-93	Photosystem I reaction center subunit XI, chloroplastic	PF02605.12^PsaL^Photosystem I reaction centre subunit XI^60-212^E:1.1e-59	0,236	0,261	0	-1,153	0,073	-0,381	0,091	-0,359	0,226	0,45	PSAL_ARATH	Photosystem I reaction center subunit XI, chloroplastic (PSI-L) (PSI subunit V)	PSAL At4g12800 T20K18.150	Arabidopsis thaliana (Mouse-ear cress)		3D-structure; Chloroplast; Complete proteome; Membrane; Photosynthesis; Photosystem I; Plastid; Reference proteome; Thylakoid; Transit peptide; Transmembrane; Transmembrane helix
SRR342022_TR17306_c0_g1_i5_len=2692	CTSL2_DICDI	Q:989-492,H:403-567	58.43%ID	E:7e-47	CTD small phosphatase-like protein 2	PF03031.15^NIF^NLI interacting factor-like phosphatase^27-187^E:1.5e-55	1	0,013	0,001	-1,734	1	0,64	1	-0,109	0,142	0,599	CTSL2_DICDI	CTD small phosphatase-like protein 2 (CTDSP-like 2) (EC 3.1.3.-)	ctdspl2 DDB_G0290365	Dictyostelium discoideum (Slime mold)		Complete proteome; Hydrolase; Protein phosphatase; Reference proteome
SRR342022_TR18058_c0_g1_i7_len=2005	BRAP_HUMAN	Q:1927-986,H:83-396	42.81%ID	E:2e-79	BRCA1-associated protein	PF07576.9^BRAP2^BRCA1-associated protein 2^98-194^E:3.4e-36`PF13639.3^zf-RING_2^Ring finger domain^206-247^E:8e-09`PF00097.22^zf-C3HC4^Zinc finger, C3HC4 type (RING finger)^207-246^E:0.00013`PF02148.16^zf-UBP^Zn-finger in ubiquitin-hydrolases and other protein^258-317^E:4e-19	1	-0,152	0	-7,044	0,832	0,222	0,679	0,346	1	0	BRAP_HUMAN	BRCA1-associated protein (EC 6.3.2.-) (BRAP2) (Impedes mitogenic signal propagation) (IMP) (RING finger protein 52) (Renal carcinoma antigen NY-REN-63)	BRAP RNF52	Homo sapiens (Human)	PATHWAY: Protein modification; protein ubiquitination.	Alternative splicing; Coiled coil; Complete proteome; Cytoplasm; Ligase; Metal-binding; Phosphoprotein; Reference proteome; Ubl conjugation pathway; Zinc; Zinc-finger
SRR342022_TR18898_c2_g1_i4_len=1108	PPL1_ARATH	Q:303-920,H:24-228	65.53%ID	E:2e-93	PsbP-like protein 1, chloroplastic	PF01789.13^PsbP^PsbP^79-235^E:7.6e-53	1	0,012	0	-1,192	0,013	-0,667	0,03	-0,578	0,217	-0,327	PPL1_ARATH	PsbP-like protein 1, chloroplastic (OEC23-like protein 4) (PsbP-related thylakoid lumenal protein 2)	PPL1 At3g55330 T26I12.210	Arabidopsis thaliana (Mouse-ear cress)		Chloroplast; Complete proteome; Direct protein sequencing; Plastid; Reference proteome; Thylakoid; Transit peptide
SRR342022_TR19090_c1_g2_i6_len=1573	ATG8C_ORYSJ	Q:1442-1092,H:1-117	96.58%ID	E:1e-62	Autophagy-related protein 8C	PF02991.13^Atg8^Autophagy protein Atg8 ubiquitin like^14-117^E:7.7e-52`PF04110.10^APG12^Ubiquitin-like autophagy protein Apg12^40-117^E:5.4e-06	1	0	0	-8,034	1	-1,612	1	-1,612	0,113	-0,568	ATG8C_ORYSJ	Autophagy-related protein 8C (Autophagy-related ubiquitin-like modifier ATG8C)	ATG8C APG8C Os08g0191600 LOC_Os08g09240 OSJNBa0056O06.5	Oryza sativa subsp. japonica (Rice)		Autophagy; Complete proteome; Cytoplasm; Cytoplasmic vesicle; Cytoskeleton; Lipoprotein; Membrane; Microtubule; Protein transport; Reference proteome; Transport; Ubl conjugation pathway; Vacuole
SRR342022_TR19090_c3_g1_i5_len=552	NA	NA	NA	NA	NA	.	1	0	0	-1,419	0,018	1,131	0,049	0,971	1	0,052	.	.	.	.	.	.
SRR342022_TR19280_c0_g1_i7_len=1873	HA22I_ARATH	Q:728-1153,H:1-142	79.58%ID	E:5e-81	HVA22-like protein i	PF03134.16^TB2_DP1_HVA22^TB2/DP1, HVA22 family^19-97^E:1.1e-23	0,732	-0,94	0,001	-6,57	1	-3,642	1	-1,253	0,732	-0,614	HA22I_ARATH	HVA22-like protein i (AtHVA22i)	HVA22I At5g42560 K16E1.3	Arabidopsis thaliana (Mouse-ear cress)		Alternative splicing; Complete proteome; Reference proteome
SRR342022_TR20085_c0_g2_i3_len=1816	GUAA_HELHP	Q:834-1388,H:785-972	45.74%ID	E:1e-43	Probable GMP synthase [glutamine-hydrolyzing]	PF03352.10^Adenine_glyco^Methyladenine glycosylase^179-352^E:1.5e-60	1	0,339	0	-3,948	0,187	0,605	0,156	0,658	1	-0,466	GUAA_HELHP	Probable GMP synthase [glutamine-hydrolyzing] (EC 6.3.5.2) (GMP synthetase) (Glutamine amidotransferase)	guaA HH_1444	Helicobacter hepaticus (strain ATCC 51449 / 3B1)	PATHWAY: Purine metabolism; GMP biosynthesis; GMP from XMP (L-Gln route): step 1/1.	ATP-binding; Complete proteome; GMP biosynthesis; Glutamine amidotransferase; Ligase; Nucleotide-binding; Purine biosynthesis; Reference proteome; Repeat
SRR342022_TR20466_c0_g5_i2_len=1618	RK1_ARATH	Q:1192-506,H:114-342	80.79%ID	E:5e-131	50S ribosomal protein L1, chloroplastic	.	0,21	-0,321	0	-1,268	0,359	-0,247	0,504	-0,177	0,506	-0,19	RK1_ARATH	50S ribosomal protein L1, chloroplastic (CL1)	RPL1 At3g63490 MAA21_120	Arabidopsis thaliana (Mouse-ear cress)		Alternative splicing; Chloroplast; Complete proteome; Phosphoprotein; Plastid; RNA-binding; Reference proteome; Ribonucleoprotein; Ribosomal protein; Transit peptide; rRNA-binding
SRR342022_TR20758_c0_g1_i1_len=725	NA	NA	NA	NA	NA	.	0,807	-0,205	0	-2,709	0,017	-1,362	0,001	-1,89	1	-0,429	.	.	.	.	.	.
SRR342022_TR21116_c0_g4_i1_len=598	DNAJ8_ARATH	Q:596-330,H:75-162	68.89%ID	E:1e-25	Chaperone protein dnaJ 8, chloroplastic	.	1	-0,37	0	-8,236	0	2,338	0,001	1,127	1	-1,279	DNAJ8_ARATH	Chaperone protein dnaJ 8, chloroplastic (AtDjC8) (AtJ8)	ATJ8 C8 J8 At1g80920 F23A5.28	Arabidopsis thaliana (Mouse-ear cress)		Chaperone; Chloroplast; Complete proteome; Plastid; Reference proteome; Transit peptide
SRR342022_TR21277_c0_g3_i1_len=1054	ENL3_ARATH	Q:196-513,H:25-130	52.34%ID	E:4e-30	Early nodulin-like protein 3	PF02298.14^Cu_bind_like^Plastocyanin-like domain^61-140^E:6e-24	0,928	-0,045	0	-2,071	0,01	-1,521	0,001	-2,074	0,667	-0,187	ENL3_ARATH	Early nodulin-like protein 3 (Phytocyanin-like protein)	At5g25090 T11H3_100	Arabidopsis thaliana (Mouse-ear cress)		Cell membrane; Complete proteome; GPI-anchor; Glycoprotein; Lipoprotein; Membrane; Reference proteome; Signal
SRR342022_TR21838_c2_g3_i2_len=1343	NA	NA	NA	NA	NA	.	1	-0,055	0	-2,05	0,526	-0,273	0,328	-0,448	1	-0,064	.	.	.	.	.	.
SRR342022_TR22223_c0_g1_i1_len=1115	MIZ1_ARATH	Q:446-922,H:125-292	52.66%ID	E:2e-51	Protein MIZU-KUSSEI 1	PF04759.10^DUF617^Protein of unknown function, DUF617^139-297^E:1.5e-70	0,387	-0,447	0,001	-3,282	1	-0,398	1	-0,121	1	-0,061	MIZ1_ARATH	Protein MIZU-KUSSEI 1	MIZ1 At2g41660 T32G6.18	Arabidopsis thaliana (Mouse-ear cress)		Complete proteome; Endoplasmic reticulum; Membrane; Reference proteome
SRR342022_TR22574_c1_g1_i1_len=1054	NA	NA	NA	NA	NA	.	0,136	-0,528	0	-1,288	1	-0,048	0,57	0,304	0,016	1,246	.	.	.	.	.	.
SRR342022_TR24116_c0_g1_i1_len=801	PRE6_ARATH	Q:519-298,H:19-92	86.49%ID	E:7e-23	Transcription factor PRE6	.	0,671	-0,168	0	-1,518	0,051	-1,018	0,243	-0,569	0,004	-1,088	PRE6_ARATH	Transcription factor PRE6 (Basic helix-loop-helix protein 163) (AtbHLH163) (bHLH 163) (Protein KIDARI) (Protein PACLOBUTRAZOL RESISTANCE 6) (bHLH transcription factor bHLH163)	PRE6 BHLH163 KDR At1g26945 T2P11	Arabidopsis thaliana (Mouse-ear cress)		Complete proteome; Cytoplasm; Growth regulation; Nucleus; Reference proteome; Transcription; Transcription regulation
SRR342022_TR24488_c1_g2_i1_len=1252	DAG_ANTMA	Q:1118-417,H:1-230	73.64%ID	E:1e-105	DAG protein, chloroplastic	.	0,01	-0,752	0	-1,365	0,597	0,213	0,188	0,478	0,446	-0,339	DAG_ANTMA	DAG protein, chloroplastic	DAG	Antirrhinum majus (Garden snapdragon)		Chloroplast; Plastid; Transit peptide
SRR342022_TR24649_c0_g2_i1_len=1232	SRG1_ARATH	Q:34-1056,H:10-354	52.46%ID	E:4e-127	Protein SRG1	PF14226.3^DIOX_N^non-haem dioxygenase in morphine synthesis N-terminal^54-152^E:5.9e-28`PF03171.17^2OG-FeII_Oxy^2OG-Fe(II) oxygenase superfamily^209-307^E:2e-26	1	-0,808	0	-1,917	0,079	0,527	0,15	0,448	0,004	-1,822	SRG1_ARATH	Protein SRG1 (AtSRG1) (Protein SENESCENCE-RELATED GENE 1)	SRG1 At1g17020 F20D23.28 F6I1.30	Arabidopsis thaliana (Mouse-ear cress)		Complete proteome; Iron; Metal-binding; Oxidoreductase; Reference proteome
SRR342022_TR25246_c0_g6_i1_len=932	NA	NA	NA	NA	NA	.	1	0,128	0	-1,383	0,496	0,256	0,935	-0,07	1	-1,132	.	.	.	.	.	.
SRR342022_TR25921_c3_g1_i1_len=1694	ROC1_NICPL	Q:669-1037,H:152-279	77.34%ID	E:6e-49	30 kDa ribonucleoprotein, chloroplastic	.	0,379	-0,226	0	-1,673	0,226	2,328	0,226	2,252	0,005	-0,734	ROC1_NICPL	30 kDa ribonucleoprotein, chloroplastic (CP-RBP30)		Nicotiana plumbaginifolia (Leadwort-leaved tobacco) (Tex-Mex tobacco)		Chloroplast; Plastid; RNA-binding; Repeat; Ribonucleoprotein; Transit peptide; mRNA processing
SRR342022_TR26925_c3_g2_i1_len=4532	NA	NA	NA	NA	NA	.	0,282	-0,437	0	-2,861	0,008	-1,114	0,028	-0,886	0,631	0,199	.	.	.	.	.	.
SRR342022_TR28137_c2_g6_i2_len=2190	TBB1_SOLTU	Q:267-1538,H:1-425	96.94%ID	E:0	Tubulin beta-1 chain	PF00091.22^Tubulin^Tubulin/FtsZ family, GTPase domain^3-213^E:4.7e-69`PF03953.14^Tubulin_C^Tubulin C-terminal domain^263-383^E:4.2e-40	0,075	-0,44	0,001	-0,959	0,699	-0,2	0,774	-0,158	0,431	0,332	TBB1_SOLTU	Tubulin beta-1 chain (Beta-1-tubulin)	TUBB1	Solanum tuberosum (Potato)		Complete proteome; Cytoplasm; Cytoskeleton; GTP-binding; Microtubule; Nucleotide-binding; Reference proteome
SRR342022_TR28271_c2_g1_i4_len=3046	GDL29_ARATH	Q:1608-2597,H:52-381	69.49%ID	E:3e-149	GDSL esterase/lipase At1g71691	PF00856.25^SET^SET domain^14-218^E:8.9e-15`PF09273.8^Rubis-subs-bind^Rubisco LSMT substrate-binding^251-384^E:3.2e-15	0,044	-0,716	0	-5,05	0,002	-6,332	0,002	-6,332	1	-0,017	GDL29_ARATH	GDSL esterase/lipase At1g71691 (EC 3.1.1.-) (Extracellular lipase At1g71691)	At1g71691 F14O23.4 F26A9.7	Arabidopsis thaliana (Mouse-ear cress)		Alternative splicing; Complete proteome; Hydrolase; Lipid degradation; Lipid metabolism; Reference proteome; Secreted; Signal
SRR342022_TR28478_c0_g1_i1_len=2603	SRF8_ARATH	Q:2378-378,H:10-669	28.41%ID	E:8e-70	Protein STRUBBELIG-RECEPTOR FAMILY 8	PF08263.9^LRRNT_2^Leucine rich repeat N-terminal domain^34-74^E:4.9e-08`PF13855.3^LRR_8^Leucine rich repeat^150-209^E:3.4e-07`PF00069.22^Pkinase^Protein kinase domain^420-682^E:4.7e-31`PF07714.14^Pkinase_Tyr^Protein tyrosine kinase^422-684^E:7.4e-44	0,017	-1,068	0,001	-4,813	0,132	-2,874	0,132	-2,877	0,798	-0,259	SRF8_ARATH	Protein STRUBBELIG-RECEPTOR FAMILY 8 (Leucine-rich repeat receptor kinase-like protein SRF8)	SRF8 At4g22130 F1N20.230	Arabidopsis thaliana (Mouse-ear cress)		ATP-binding; Alternative splicing; Complete proteome; Glycoprotein; Leucine-rich repeat; Membrane; Nucleotide-binding; Receptor; Reference proteome; Repeat; Signal; Transmembrane; Transmembrane helix
SRR342022_TR28814_c6_g5_i1_len=1022	RL71_ARATH	Q:935-201,H:1-247	55.87%ID	E:1e-85	60S ribosomal protein L7-1	PF08079.9^Ribosomal_L30_N^Ribosomal L30 N-terminal domain^13-75^E:1.4e-09`PF00327.17^Ribosomal_L30^Ribosomal protein L30p/L7e^87-137^E:1e-16	1	0	0	-8,674	0,076	0,71	0,415	0,356	1	0	RL71_ARATH	60S ribosomal protein L7-1	RPL7A At1g80750 F23A5.10	Arabidopsis thaliana (Mouse-ear cress)		Complete proteome; Reference proteome; Ribonucleoprotein; Ribosomal protein
SRR342022_TR29282_c0_g2_i1_len=693	PSAG_ARATH	Q:552-130,H:27-160	72.34%ID	E:2e-67	Photosystem I reaction center subunit V, chloroplastic	PF01241.15^PSI_PSAK^Photosystem I psaG / psaK^53-144^E:2.5e-26	0,659	-0,093	0	-1,45	0,004	-0,599	0,065	-0,382	0	1,478	PSAG_ARATH	Photosystem I reaction center subunit V, chloroplastic (PSI-G)	PSAG At1g55670 F20N2.21 F20N2.33 F20N2_3	Arabidopsis thaliana (Mouse-ear cress)		Chloroplast; Complete proteome; Membrane; Photosynthesis; Photosystem I; Plastid; Reference proteome; Thylakoid; Transit peptide; Transmembrane; Transmembrane helix
SRR342022_TR30025_c2_g5_i2_len=759	NA	NA	NA	NA	NA	PF05757.8^PsbQ^Oxygen evolving enhancer protein 3 (PsbQ)^73-186^E:8.7e-19	0,744	-0,115	0	-1,515	1	-0,067	0,597	0,412	1	-0,002	.	.	.	.	.	.
SRR342022_TR30554_c0_g1_i1_len=906	PSAH1_ARATH	Q:161-529,H:1-123	81.3%ID	E:3e-50	Photosystem I reaction center subunit VI-1, chloroplastic	PF03244.11^PSI_PsaH^Photosystem I reaction centre subunit VI^7-145^E:1.9e-71	0,331	0,353	0	-1,997	0,275	-0,264	0,521	-0,158	0,246	-0,247	PSAH1_ARATH	Photosystem I reaction center subunit VI-1, chloroplastic (PSI-H1)	PSAH1 At3g16140 MSL1.18	Arabidopsis thaliana (Mouse-ear cress)		Chloroplast; Complete proteome; Membrane; Photosynthesis; Photosystem I; Plastid; Reference proteome; Thylakoid; Transit peptide; Transmembrane; Transmembrane helix
SRR342022_TR30554_c0_g1_i2_len=2460	COL16_ARATH	Q:1768-278,H:3-403	39.81%ID	E:3e-80	Zinc finger protein CONSTANS-LIKE 16	PF00643.21^zf-B_box^B-box zinc finger^19-62^E:1.2e-07`PF06203.11^CCT^CCT motif^462-504^E:7.7e-18	0,209	-0,363	0	-1,244	1	-0,082	1	0,38	0,022	0,713	COL16_ARATH	Zinc finger protein CONSTANS-LIKE 16	COL16 At1g25440 F2J7.10	Arabidopsis thaliana (Mouse-ear cress)		Coiled coil; Complete proteome; Metal-binding; Nucleus; Reference proteome; Zinc; Zinc-finger
SRR342022_TR30585_c3_g1_i14_len=2360	NA	NA	NA	NA	NA	.	0,325	-0,311	0	-1,57	0,007	-1,896	0,007	-1,82	0,47	0,241	.	.	.	.	.	.
SRR342022_TR31216_c0_g1_i1_len=1407	RR5_SPIOL	Q:340-1083,H:61-308	79.03%ID	E:5e-98	30S ribosomal protein S5, chloroplastic	PF00333.17^Ribosomal_S5^Ribosomal protein S5, N-terminal domain^152-216^E:2e-23`PF03719.12^Ribosomal_S5_C^Ribosomal protein S5, C-terminal domain^229-299^E:2.9e-25	0,028	-0,501	0	-1,473	0,016	-0,59	0,051	-0,474	0,211	-0,281	RR5_SPIOL	30S ribosomal protein S5, chloroplastic	rps5	Spinacia oleracea (Spinach)		3D-structure; Antibiotic resistance; Chloroplast; Direct protein sequencing; Plastid; RNA-binding; Ribonucleoprotein; Ribosomal protein; Transit peptide; rRNA-binding
SRR342022_TR31230_c0_g1_i1_len=841	NA	NA	NA	NA	NA	PF00166.18^Cpn10^Chaperonin 10 Kd subunit^49-135^E:6.1e-16	0,005	-0,655	0	-0,961	0,123	0,369	0,114	0,379	1	0	.	.	.	.	.	.
SRR342022_TR31842_c3_g4_i1_len=2389	F16P2_SPIOL	Q:1228-209,H:1-339	87.65%ID	E:0	Fructose-1,6-bisphosphatase, cytosolic	PF00316.17^FBPase^Fructose-1-6-bisphosphatase, N-terminal domain^13-202^E:8.3e-78	0,931	-0,076	0	-1,75	0,001	-0,873	0,004	-0,769	0,897	-0,104	F16P2_SPIOL	Fructose-1,6-bisphosphatase, cytosolic (FBPase) (EC 3.1.3.11) (D-fructose-1,6-bisphosphate 1-phosphohydrolase)		Spinacia oleracea (Spinach)		Carbohydrate metabolism; Cytoplasm; Direct protein sequencing; Hydrolase; Magnesium; Metal-binding
SRR342022_TR32290_c0_g2_i3_len=922	BAME1_ARATH	Q:654-325,H:861-970	62.73%ID	E:8e-45	Leucine-rich repeat receptor-like serine/threonine-protein kinase BAM1	PF07714.14^Pkinase_Tyr^Protein tyrosine kinase^10-108^E:7.3e-09	0,874	0,127	0	-2,318	0,517	-1,059	0,034	-3,574	0,56	-0,942	BAME1_ARATH	Leucine-rich repeat receptor-like serine/threonine-protein kinase BAM1 (EC 2.7.11.1) (Protein BARELY ANY MERISTEM 1)	BAM1 At5g65700 F6H11.170 MPA24.5	Arabidopsis thaliana (Mouse-ear cress)		ATP-binding; Cell membrane; Complete proteome; Developmental protein; Differentiation; Glycoprotein; Kinase; Leucine-rich repeat; Membrane; Nucleotide-binding; Phosphoprotein; Receptor; Reference proteome; Repeat; Serine/threonine-protein kinase; Signal; Transferase; Transmembrane; Transmembrane helix
SRR342022_TR33747_c1_g2_i1_len=4338	KIF4_XENLA	Q:393-1694,H:2-415	48.33%ID	E:5e-106	Chromosome-associated kinesin KIF4	.	0,61	-0,178	0	-1,513	0,055	-0,798	0,042	-0,821	1	0	KIF4_XENLA	Chromosome-associated kinesin KIF4 (Chromokinesin) (Chromosome-associated kinesin KLP1)	kif4 kif4a klp1	Xenopus laevis (African clawed frog)		ATP-binding; Chromosome; Coiled coil; Cytoplasm; Cytoskeleton; DNA-binding; Microtubule; Motor protein; Nucleotide-binding; Nucleus
SRR342022_TR34191_c0_g1_i2_len=2075	GLPK_ARATH	Q:309-1874,H:1-522	80.65%ID	E:0	Glycerol kinase	PF00370.18^FGGY_N^FGGY family of carbohydrate kinases, N-terminal domain^8-262^E:1.8e-71`PF02782.13^FGGY_C^FGGY family of carbohydrate kinases, C-terminal domain^270-469^E:2.8e-59	1	-0,345	0,001	-2,65	0,056	0,71	0,922	0,063	0,679	0,299	GLPK_ARATH	Glycerol kinase (Glycerokinase) (EC 2.7.1.30) (ATP:glycerol 3-phosphotransferase) (Protein GLYCEROL INSENSITIVE 1) (Protein NONHOST RESISTANCE TO P. S. PHASEOLICOLA 1)	GLPK GLI1 NHO1 At1g80460 T21F11.21	Arabidopsis thaliana (Mouse-ear cress)	PATHWAY: Polyol metabolism; glycerol degradation via glycerol kinase pathway; sn-glycerol 3-phosphate from glycerol: step 1/1.	ATP-binding; Alternative splicing; Complete proteome; Cytoplasm; Glycerol metabolism; Kinase; Nucleotide-binding; Plant defense; Reference proteome; Transferase
SRR342022_TR34286_c0_g2_i1_len=1131	NA	NA	NA	NA	NA	PF11623.5^DUF3252^Protein of unknown function (DUF3252)^174-225^E:2e-28	0,723	0,134	0	-1,784	0,602	-0,118	0,94	-0,019	0	4,824	.	.	.	.	.	.
SRR342023_TR225_c2_g2_i2_len=1953	PMEU1_SOLLC	Q:213-1772,H:60-583	58.27%ID	E:0	Pectinesterase/pectinesterase inhibitor U1	PF04043.12^PMEI^Plant invertase/pectin methylesterase inhibitor^35-195^E:5.8e-25`PF01095.16^Pectinesterase^Pectinesterase^237-533^E:6.4e-147	0,005	-0,751	0	-1,495	0,402	-1,116	1	0,132	0,348	0,305	PMEU1_SOLLC	Pectinesterase/pectinesterase inhibitor U1 [Includes: Pectinesterase inhibitor U1 (Pectin methylesterase inhibitor U1); Pectinesterase U1 (PE U1) (EC 3.1.1.11) (Pectin methylesterase U1)]	PMEU1	Solanum lycopersicum (Tomato) (Lycopersicon esculentum)	PATHWAY: Glycan metabolism; pectin degradation; 2-dehydro-3-deoxy-D-gluconate from pectin: step 1/5.	Aspartyl esterase; Cell wall; Cell wall biogenesis/degradation; Complete proteome; Disulfide bond; Glycoprotein; Hydrolase; Reference proteome; Secreted; Signal
SRR342023_TR2068_c0_g1_i1_len=923	NA	NA	NA	NA	NA	PF00538.16^Linker_histone^linker histone H1 and H5 family^35-96^E:6.8e-11`PF02178.16^AT_hook^AT hook motif^133-144^E:0.22`PF02178.16^AT_hook^AT hook motif^172-183^E:0.23	0,01	-0,699	0,001	-1,126	0,648	-0,219	0,291	-0,426	0,768	-0,088	.	.	.	.	.	.
SRR342023_TR2319_c0_g2_i1_len=667	SBP2_ANTMA	Q:589-323,H:78-167	72.22%ID	E:3e-35	Squamosa promoter-binding protein 2	.	1	-0,001	0	-2,342	0,777	0,224	0,412	-0,525	0,135	0,538	SBP2_ANTMA	Squamosa promoter-binding protein 2	SBP2	Antirrhinum majus (Garden snapdragon)		DNA-binding; Metal-binding; Nucleus; Transcription; Transcription regulation; Zinc; Zinc-finger
SRR342023_TR2656_c2_g9_i2_len=771	NA	NA	NA	NA	NA	.	0,907	0,034	0	-1,101	0,469	-0,169	0,311	-0,235	0,593	-0,714	.	.	.	.	.	.
SRR342023_TR2724_c0_g1_i1_len=1635	CRR3_ARATH	Q:442-1323,H:7-307	67.11%ID	E:7e-107	Probable NAD(P)H dehydrogenase subunit CRR3, chloroplastic {ECO:0000305}	PF01657.14^Stress-antifung^Salt stress response/antifungal^48-133^E:1.1e-12`PF01657.14^Stress-antifung^Salt stress response/antifungal^153-238^E:5e-12	0,085	-0,706	0	-2,527	0,356	-1,227	0,56	-0,84	0,034	-0,964	CRR3_ARATH	Probable NAD(P)H dehydrogenase subunit CRR3, chloroplastic (Probable NDH subunit CRR3) (Protein CHLORORESPIRATORY REDUCTION 3)	CRR3 At2g01590	Arabidopsis thaliana (Mouse-ear cress)		Chaperone; Chloroplast; Complete proteome; Membrane; Plastid; Reference proteome; Thylakoid; Transit peptide; Transmembrane; Transmembrane helix
SRR342023_TR3345_c1_g1_i1_len=1267	PIP15_ARATH	Q:959-261,H:44-284	85.06%ID	E:5e-144	Probable aquaporin PIP1-5	PF00230.17^MIP^Major intrinsic protein^8-201^E:5.3e-74	0,126	-0,369	0	-2,132	0,801	-0,088	0,062	-0,595	1	0,036	PIP15_ARATH	Probable aquaporin PIP1-5 (AtPIP1;5) (Plasma membrane intrinsic protein 1d) (PIP1d)	PIP1-5 PIP1D At4g23400 F16G20.100	Arabidopsis thaliana (Mouse-ear cress)		Cell membrane; Complete proteome; Membrane; Phosphoprotein; Reference proteome; Repeat; Transmembrane; Transmembrane helix; Transport
SRR342023_TR3484_c0_g1_i2_len=854	NA	NA	NA	NA	NA	.	0,308	-0,45	0	-2,903	0,103	-1,455	0,103	-1,567	1	-0,002	.	.	.	.	.	.
SRR342023_TR3993_c0_g1_i4_len=606	CML20_ARATH	Q:567-271,H:69-167	80.81%ID	E:2e-49	Probable calcium-binding protein CML20 {ECO:0000303|Ref.6}	.	1	0	0	-7,165	1	0	1	0	0,798	-0,547	CML20_ARATH	Probable calcium-binding protein CML20 (Calmodulin-like protein 20) (Centrin 1) (AtCEN1)	CML20 CEN1 At3g50360 F11C1.200	Arabidopsis thaliana (Mouse-ear cress)		Calcium; Complete proteome; Metal-binding; Reference proteome; Repeat
SRR342023_TR4185_c0_g2_i6_len=2022	NA	NA	NA	NA	NA	PF13178.3^DUF4005^Protein of unknown function (DUF4005)^63-118^E:2.4e-08	0,357	-0,645	0,001	-3,155	0,016	-2,378	0,004	-3,107	0,377	-0,654	.	.	.	.	.	.
SRR342023_TR4402_c2_g1_i8_len=658	RR13_ARATH	Q:612-307,H:53-154	88.24%ID	E:9e-60	30S ribosomal protein S13, chloroplastic	.	0,012	-0,695	0	-1,558	0,236	-0,366	0,154	-0,445	0,079	-0,754	RR13_ARATH	30S ribosomal protein S13, chloroplastic (CS13)	RPS13 At5g14320 F18O22_110	Arabidopsis thaliana (Mouse-ear cress)		Alternative splicing; Chloroplast; Complete proteome; Plastid; RNA-binding; Reference proteome; Ribonucleoprotein; Ribosomal protein; Transit peptide; rRNA-binding
SRR342023_TR5021_c0_g1_i1_len=1762	CBWD2_HUMAN	Q:1208-147,H:41-377	36.9%ID	E:6e-65	COBW domain-containing protein 2	PF02492.16^cobW^CobW/HypB/UreG, nucleotide-binding domain^99-283^E:6e-57`PF07683.11^CobW_C^Cobalamin synthesis protein cobW C-terminal domain^356-450^E:5.3e-21	1	-0,867	0	-8,185	0,068	0,851	0,027	1,021	1	-1,126	CBWD2_HUMAN	COBW domain-containing protein 2 (Cobalamin synthase W domain-containing protein 2)	CBWD2	Homo sapiens (Human)		ATP-binding; Complete proteome; Nucleotide-binding; Reference proteome
SRR342023_TR5231_c0_g1_i3_len=4030	NACK1_TOBAC	Q:816-2012,H:28-416	63.68%ID	E:9e-151	Kinesin-like protein NACK1	PF16796.2^Microtub_bd^Microtubule binding^29-166^E:4.5e-20`PF00225.20^Kinesin^Kinesin motor domain^32-351^E:1e-95`PF11995.5^DUF3490^Domain of unknown function (DUF3490)^803-963^E:1e-71	0,277	-0,374	0	-1,976	0,783	-0,208	1	-0,099	0,248	0,445	NACK1_TOBAC	Kinesin-like protein NACK1 (NPK1-activating kinesin-1)	NACK1	Nicotiana tabacum (Common tobacco)		ATP-binding; Cell cycle; Cell division; Coiled coil; Cytoplasm; Cytoskeleton; Microtubule; Motor protein; Nucleotide-binding; Nucleus; Phosphoprotein
SRR342023_TR5536_c0_g18_i5_len=3042	PLA20_ARATH	Q:1057-380,H:187-411	63.44%ID	E:3e-88	Phospholipase A1-IIdelta	.	0,184	-0,468	0	-1,854	0,431	0,425	1	0,003	1	-1,062	PLA20_ARATH	Phospholipase A1-IIdelta (EC 3.1.1.-)	At2g42690 F14N22.2	Arabidopsis thaliana (Mouse-ear cress)		Acetylation; Alternative splicing; Complete proteome; Cytoplasm; Hydrolase; Lipid degradation; Lipid metabolism; Reference proteome
SRR342023_TR5644_c0_g1_i1_len=1877	NA	NA	NA	NA	NA	.	0,178	-0,45	0	-1,198	0,042	1,821	0,002	2,386	0,269	-0,337	.	.	.	.	.	.
SRR342023_TR5701_c4_g3_i3_len=2131	NA	NA	NA	NA	NA	PF03879.11^Cgr1^Cgr1 family^73-177^E:6.9e-07	0,604	0,118	0	-0,865	0,001	1,262	0,044	0,832	0,548	-0,297	.	.	.	.	.	.
[illegible]	[illegible]	Q:1020[illegible]	[illegible]	[illegible]	[illegible]	[illegible]	[illegible]	[illegible]	[illegible]	[illegible]	[illegible]	[illegible]	[illegible]	[illegible]	[illegible]	[illegible]	[illegible]	[illegible]	[illegible]	Hyoscyamus muticus (Egyptian [illegible]	[illegible]	Heme; Iron; Membrane; Metal-binding; Monooxygenase; NADP; Oxidoreductase; [illegible]

SRR342023_TR5978_c1_g9_i5_len=1181	C7D55_HYOMU	Q:1029-394,H:287-497	60.47%ID	E:1e-76	Premnaspirodiene oxygenase	PF00067.19^p450^Cytochrome P450^2-171^E:3.8e-58	0,618	0,165	0	-1,866	0,722	0,155	0,761	-0,127	1	0,915	C7D55_HYOMU	Premnaspirodiene oxygenase (HPO) (EC 1.14.13.121) (Cytochrome P450 71D55)	CYP71D55	Hyoscyamus muticus (Egyptian henbane)		Heme; Iron; Membrane; Metal-binding; Monooxygenase; NADP; Oxidoreductase; Transmembrane; Transmembrane helix
SRR342023_TR6064_c0_g1_i2_len=1775	FUCO1_ARATH	Q:183-1517,H:39-481	54.41%ID	E:5e-165	Alpha-L-fucosidase 1	PF01120.14^Alpha_L_fucos^Alpha-L-fucosidase^96-359^E:6.5e-36	0,154	-0,462	0	-2,782	1	-1,038	1	-0,952	0,321	0,335	FUCO1_ARATH	Alpha-L-fucosidase 1 (EC 3.2.1.51) (Alpha-1,3/4-fucosidase) (AtFUC1) (Alpha-L-fucoside fucohydrolase)	FUC1 At2g28100 F24D13.11	Arabidopsis thaliana (Mouse-ear cress)		Apoplast; Complete proteome; Glycoprotein; Glycosidase; Hydrolase; Reference proteome; Secreted; Signal
SRR342023_TR6280_c0_g4_i1_len=1086	NA	NA	NA	NA	NA	PF14309.3^DUF4378^Domain of unknown function (DUF4378)^94-235^E:2.4e-18	1	-0,051	0	-2,402	0,004	-2,068	0,019	-1,465	0,754	-0,177	.	.	.	.	.	.
SRR342023_TR6654_c1_g1_i2_len=2275	Y1154_ARATH	Q:343-1815,H:7-464	64.5%ID	E:0	Probable serine/threonine-protein kinase At1g01540	PF00069.22^Pkinase^Protein kinase domain^186-453^E:1.1e-47`PF07714.14^Pkinase_Tyr^Protein tyrosine kinase^188-455^E:3.4e-45	0,024	-0,747	0	-2,042	0,719	0,302	0,597	0,377	0,318	0,488	Y1154_ARATH	Probable serine/threonine-protein kinase At1g01540 (EC 2.7.11.1)	At1g01540 F22L4.8	Arabidopsis thaliana (Mouse-ear cress)		ATP-binding; Alternative splicing; Complete proteome; Kinase; Membrane; Nucleotide-binding; Phosphoprotein; Reference proteome; Serine/threonine-protein kinase; Transferase; Transmembrane; Transmembrane helix
SRR342023_TR7023_c5_g3_i1_len=3160	THIC_ARATH	Q:417-2348,H:3-643	85.74%ID	E:0	Phosphomethylpyrimidine synthase, chloroplastic	PF01964.15^ThiC_Rad_SAM^Radical SAM ThiC family^166-586^E:7.1e-198	0,196	0,383	0	-1,222	1	0,193	1	0,203	0,021	-0,646	THIC_ARATH	Phosphomethylpyrimidine synthase, chloroplastic (EC 4.1.99.17) (Hydroxymethylpyrimidine phosphate synthase) (HMP-P synthase) (HMP-phosphate synthase) (HMPP synthase) (Protein PYRIMIDINE REQUIRING) (Thiamine biosynthesis protein ThiC) (Protein THIAMINE C)	THIC PY At2g29630 T27A16.27	Arabidopsis thaliana (Mouse-ear cress)	PATHWAY: Cofactor biosynthesis; thiamine diphosphate biosynthesis.	3D-structure; 4Fe-4S; Chloroplast; Complete proteome; Iron; Iron-sulfur; Lyase; Metal-binding; Plastid; Reference proteome; S-adenosyl-L-methionine; Thiamine biosynthesis; Transit peptide; Zinc
SRR342023_TR7223_c1_g3_i1_len=3785	ATH1_ARATH	Q:2538-3407,H:162-446	55.26%ID	E:9e-86	Homeobox protein ATH1	PF07526.8^POX^Associated with HOX^292-418^E:5.1e-25`PF05920.8^Homeobox_KN^Homeobox KN domain^479-518^E:9e-15`PF00046.26^Homeobox^Homeobox domain^480-518^E:5.3e-06	0,409	-0,403	0	-2,123	0,732	-0,912	0,732	-0,85	0,219	-0,622	ATH1_ARATH	Homeobox protein ATH1	ATH1 At4g32980 F26P21.100	Arabidopsis thaliana (Mouse-ear cress)		Complete proteome; DNA-binding; Homeobox; Nucleus; Reference proteome; Transcription; Transcription regulation
SRR342023_TR7922_c0_g1_i1_len=1250	NDK2_TOBAC	Q:1044-418,H:25-232	77.51%ID	E:2e-109	Nucleoside diphosphate kinase 2, chloroplastic	PF00334.16^NDK^Nucleoside diphosphate kinase^99-232^E:2.5e-54	0,094	-0,444	0,001	-0,967	0,954	-0,032	1	0,017	0,252	-0,304	NDK2_TOBAC	Nucleoside diphosphate kinase 2, chloroplastic (EC 2.7.4.6) (Nucleoside diphosphate kinase II) (NDK II) (NDP kinase II) (NDPK II)	NDPK2	Nicotiana tabacum (Common tobacco)		ATP-binding; Chloroplast; Direct protein sequencing; Kinase; Magnesium; Metal-binding; Nucleotide metabolism; Nucleotide-binding; Phosphoprotein; Plastid; Transferase; Transit peptide
SRR342023_TR9121_c5_g1_i1_len=1294	TBB1_AVESA	Q:144-1028,H:70-364	96.61%ID	E:0	Tubulin beta-1 chain	PF00091.22^Tubulin^Tubulin/FtsZ family, GTPase domain^2-65^E:2e-15`PF03953.14^Tubulin_C^Tubulin C-terminal domain^115-235^E:2.6e-41	1	-0,51	0	-3,284	1	0	1	2,137	0,006	2,464	TBB1_AVESA	Tubulin beta-1 chain (Beta-1-tubulin) (Fragment)	TUBB1 TUB1	Avena sativa (Oat)		Cytoplasm; Cytoskeleton; GTP-binding; Microtubule; Nucleotide-binding
SRR342023_TR9881_c0_g4_i2_len=504	NA	NA	NA	NA	NA	.	0,013	-0,954	0	-1,382	0,423	0,341	0,182	0,534	0,172	-0,397	.	.	.	.	.	.
SRR342023_TR9968_c3_g3_i2_len=3246	TTM3_ARATH	Q:1702-1163,H:22-210	49.74%ID	E:2e-38	Triphosphate tunel metalloenzyme 3	PF01928.18^CYTH^CYTH domain^1-151^E:4.1e-19	1	-0,105	0	-5,567	0,452	0,597	1	0,015	1	-0,784	TTM3_ARATH	Triphosphate tunel metalloenzyme 3 (Adenosinetriphosphatase) (ATPase) (EC 3.6.1.3) (Triphosphatase) (PPPase) (EC 3.6.1.25)	TTM3 At2g11890 F23M2.5	Arabidopsis thaliana (Mouse-ear cress)		3D-structure; ATP-binding; Alternative splicing; Complete proteome; Hydrolase; Nucleotide-binding; Nucleus; Reference proteome
SRR342023_TR10609_c1_g5_i2_len=1032	GCSH_FLAAN	Q:786-289,H:1-162	84.94%ID	E:3e-96	Glycine cleavage system H protein, mitochondrial	PF01597.16^GCV_H^Glycine cleavage H-protein^42-161^E:1.1e-48	0,087	-0,388	0	-0,833	0,005	-0,652	0,017	-0,543	1	0,019	GCSH_FLAAN	Glycine cleavage system H protein, mitochondrial	GDCSH GCDH GCSH	Flaveria anomala (Yellowtops)		Lipoyl; Mitochondrion; Transit peptide
SRR342023_TR10865_c0_g4_i1_len=911	NA	NA	NA	NA	NA	.	0,643	-0,141	0	-1,086	0,475	0,232	0,414	0,272	1	0,787	.	.	.	.	.	.
SRR342023_TR11198_c1_g4_i8_len=939	RK6_ARATH	Q:886-530,H:12-130	71.43%ID	E:1e-81	50S ribosomal protein L6, chloroplastic	PF00347.20^Ribosomal_L6^Ribosomal protein L6^59-131^E:2.2e-13	0,206	-0,469	0	-2,034	1	-0,029	0,428	-0,395	1	0	RK6_ARATH	50S ribosomal protein L6, chloroplastic (Protein EMBRYO DEFECTIVE 2394)	RPL6 EMB2394 At1g05190 YUP8H12.20	Arabidopsis thaliana (Mouse-ear cress)		Chloroplast; Complete proteome; Plastid; RNA-binding; Reference proteome; Ribonucleoprotein; Ribosomal protein; Transit peptide; rRNA-binding
SRR342023_TR11498_c2_g2_i1_len=2058	GUN17_ARATH	Q:1864-455,H:48-516	81.28%ID	E:0	Endoglucanase 17	PF00759.16^Glyco_hydro_9^Glycosyl hydrolase family 9^70-524^E:1.6e-143	0,037	-0,657	0	-4,669	0,692	-1,179	0,381	-1,441	0,494	-0,275	GUN17_ARATH	Endoglucanase 17 (EC 3.2.1.4) (Endo-1,4-beta glucanase 17)	At4g02290 T2H3.5	Arabidopsis thaliana (Mouse-ear cress)		Carbohydrate metabolism; Cell wall biogenesis/degradation; Cellulose degradation; Complete proteome; Glycoprotein; Glycosidase; Hydrolase; Polysaccharide degradation; Reference proteome; Secreted; Signal
SRR342023_TR11922_c1_g10_i1_len=1288	GDL17_ARATH	Q:80-1165,H:6-370	68.94%ID	E:0	GDSL esterase/lipase At1g33811	PF00657.19^Lipase_GDSL^GDSL-like Lipase/Acylhydrolase^36-348^E:1.6e-15	0,662	-0,165	0,001	-2,708	0,004	-1,671	0,002	-1,904	0,149	-0,604	GDL17_ARATH	GDSL esterase/lipase At1g33811 (EC 3.1.1.-) (Extracellular lipase At1g33811)	At1g33811 F14M2.7	Arabidopsis thaliana (Mouse-ear cress)		Complete proteome; Glycoprotein; Hydrolase; Lipid degradation; Lipid metabolism; Reference proteome; Secreted; Signal
SRR342023_TR14167_c2_g4_i3_len=655	NA	NA	NA	NA	NA	.	1	-1,442	0	-1,564	0,83	0,134	0,474	-0,358	0,502	-3,973	.	.	.	.	.	.
SRR342023_TR14167_c2_g4_i4_len=832	NA	NA	NA	NA	NA	.	1	-0,334	0,001	-1,524	0,623	0,296	0,52	0,401	1	-1,496	.	.	.	.	.	.
SRR342023_TR14185_c0_g1_i1_len=1735	TL203_ARATH	Q:373-1020,H:63-278	84.26%ID	E:6e-106	.	PF00805.19^Pentapeptide^Pentapeptide repeats (8 copies)^99-138^E:6.4e-07`PF13599.3^Pentapeptide_4^Pentapeptide repeats (9 copies)^119-194^E:7.8e-07`PF00805.19^Pentapeptide^Pentapeptide repeats (8 copies)^124-160^E:7.8e-09`PF00805.19^Pentapeptide^Pentapeptide repeats (8 copies)^166-199^E:6.7e-07	0,311	-0,39	0	-1,352	0,931	0,08	0,165	0,511	0,008	-1,43	TL203_ARATH	Thylakoid lumenal protein TL20.3, chloroplastic	TL20.3 At1g12250 T28K15.2	Arabidopsis thaliana (Mouse-ear cress)		Alternative splicing; Chloroplast; Complete proteome; Plastid; Reference proteome; Repeat; Thylakoid; Transit peptide
SRR342023_TR14202_c2_g1_i5_len=1314	EXPA4_ARATH	Q:857-138,H:18-257	90%ID	E:1e-159	Expansin-A4	PF03330.15^DPBB_1^Lytic transglycolase^68-155^E:1.3e-21`PF01357.18^Pollen_allerg_1^Pollen allergen^166-243^E:1.9e-24	0,548	-0,166	0	-1,151	0,343	0,325	1	-0,03	0,229	0,495	EXPA4_ARATH	Expansin-A4 (AtEXPA4) (Alpha-expansin-4) (At-EXP4) (AtEx4) (Ath-ExpAlpha-1.6)	EXPA4 EXP4 At2g39700 F17A14.7	Arabidopsis thaliana (Mouse-ear cress)		Cell wall; Cell wall biogenesis/degradation; Complete proteome; Direct protein sequencing; Disulfide bond; Membrane; Reference proteome; Secreted; Signal
SRR342023_TR14529_c0_g2_i1_len=1633	NA	NA	NA	NA	NA	.	0,232	-0,576	0	-3,084	1	1,158	1	0,848	0,725	-0,167	.	.	.	.	.	.
SRR342023_TR16216_c0_g1_i1_len=1355	NA	NA	NA	NA	NA	PF02234.16^CDI^Cyclin-dependent kinase inhibitor^225-268^E:2.6e-18	0,881	0,072	0	-1,569	0,014	-1,006	0,007	-1,147	0,496	-0,245	.	.	.	.	.	.
SRR342023_TR16373_c7_g4_i1_len=1003	ALFL8_ORYSJ	Q:1002-334,H:49-253	64.16%ID	E:2e-66	PHD finger protein ALFIN-LIKE 8	PF12165.5^DUF3594^Domain of unknown function (DUF3594)^1-88^E:7.1e-43`PF00628.26^PHD^PHD-finger^171-219^E:1.2e-10	0,398	-0,344	0	-1,647	0,895	0,143	0,666	-0,232	1	0	ALFL8_ORYSJ	PHD finger protein ALFIN-LIKE 8	Os11g0244800 LOC_Os11g14010 OsJ_33509 OSJNBa0004P24 OSJNBa0024M06	Oryza sativa subsp. japonica (Rice)		Chromatin regulator; Complete proteome; Metal-binding; Nucleus; Reference proteome; Transcription; Transcription regulation; Zinc; Zinc-finger
SRR342023_TR16460_c1_g9_i1_len=1697	Y5830_ARATH	Q:1443-382,H:303-653	74.16%ID	E:0	Probable inactive receptor kinase At5g58300	PF00069.22^Pkinase^Protein kinase domain^147-410^E:4.8e-37`PF07714.14^Pkinase_Tyr^Protein tyrosine kinase^148-411^E:1.8e-35	0,599	-0,19	0,001	-1,317	0,581	-0,322	0,484	-0,447	1	0,143	Y5830_ARATH	Probable inactive receptor kinase At5g58300	At5g58300 MCK7.17	Arabidopsis thaliana (Mouse-ear cress)		ATP-binding; Complete proteome; Leucine-rich repeat; Membrane; Nucleotide-binding; Phosphoprotein; Receptor; Reference proteome; Repeat; Signal; Transmembrane; Transmembrane helix
SRR342023_TR16591_c0_g3_i1_len=1252	UBE11_ARATH	Q:77-1003,H:772-1080	80.91%ID	E:0	Ubiquitin-activating enzyme E1 1	PF10585.6^UBA_e1_thiolCys^Ubiquitin-activating enzyme active site^1-125^E:5.4e-30`PF00899.18^ThiF^ThiF family^104-184^E:2.2e-10`PF09358.7^E1_UFD^Ubiquitin fold domain^196-285^E:3.1e-23	1	0	0	-7,188	1	0	1	0	0,006	-1,06	UBE11_ARATH	Ubiquitin-activating enzyme E1 1 (AtUBA1) (Protein MODIFIER OF SNC1 5)	UBA1 MOS5 At2g30110 T27E13.15	Arabidopsis thaliana (Mouse-ear cress)	PATHWAY: Protein modification; protein ubiquitination.	ATP-binding; Complete proteome; Ligase; Nucleotide-binding; Reference proteome; Ubl conjugation pathway
SRR342023_TR16698_c0_g14_i1_len=611	NA	NA	NA	NA	NA	PF10950.5^DUF2775^Protein of unknown function (DUF2775)^2-79^E:7.7e-17`PF10950.5^DUF2775^Protein of unknown function (DUF2775)^86-157^E:1.6e-15`PF10950.5^DUF2775^Protein of unknown function (DUF2775)^160-202^E:6.2e-09	0,487	0,304	0	-5,322	1	0	1	0	0,317	0,532	.	.	.	.	.	.
SRR342023_TR17575_c0_g3_i3_len=1298	MENB_ARATH	Q:1109-120,H:4-337	78.65%ID	E:0	1,4-dihydroxy-2-naphthoyl-CoA synthase, peroxisomal	PF00378.17^ECH_1^Enoyl-CoA hydratase/isomerase^80-326^E:3e-75`PF16113.2^ECH_2^Enoyl-CoA hydratase/isomerase^83-259^E:3.2e-21	0,091	-0,463	0	-2,122	0,902	-0,058	0,603	0,174	1	0,013	MENB_ARATH	1,4-dihydroxy-2-naphthoyl-CoA synthase, peroxisomal (DHNS) (EC 4.1.3.36) (Enoyl-CoA hydratase/isomerase D) (ECHID) (Naphthoate synthase)	MENB At1g60550 F8A5.9	Arabidopsis thaliana (Mouse-ear cress)		Complete proteome; Isomerase; Lyase; Peroxisome; Reference proteome
SRR342023_TR18351_c3_g4_i8_len=2801	EFTU2_SOYBN	Q:2336-1044,H:49-479	89.56%ID	E:0	Elongation factor Tu, chloroplastic	PF00009.24^GTP_EFTU^Elongation factor Tu GTP binding domain^85-286^E:4.5e-57`PF03144.22^GTP_EFTU_D2^Elongation factor Tu domain 2^310-378^E:9.6e-19`PF03143.14^GTP_EFTU_D3^Elongation factor Tu C-terminal domain^383-482^E:6e-35	0,016	-0,554	0	-0,959	0,005	-0,61	0,096	-0,359	0,42	-0,177	EFTU2_SOYBN	Elongation factor Tu, chloroplastic (EF-Tu)	TUFB1	Glycine max (Soybean) (Glycine hispida)		Chloroplast; Complete proteome; Elongation factor; GTP-binding; Nucleotide-binding; Plastid; Protein biosynthesis; Reference proteome; Transit peptide
SRR342023_TR19197_c0_g1_i1_len=1169	CITX2_NICBE	Q:321-854,H:1-181	69.02%ID	E:7e-80	Thioredoxin-like protein CITRX2, chloroplastic {ECO:0000303|PubMed:15131698}	PF00085.17^Thioredoxin^Thioredoxin^72-158^E:9.7e-20`PF13098.3^Thioredoxin_2^Thioredoxin-like domain^88-172^E:4e-09	0,057	-0,605	0	-1,286	0,657	0,177	1	-0,049	0,248	-0,37	CITX2_NICBE	Thioredoxin-like protein CITRX2, chloroplastic (EC 1.8.-.-) (Cf-9-interacting thioredoxin 2) (NbCiTrx2)	CITRX2	Nicotiana benthamiana		Apoptosis; Chloroplast; Disulfide bond; Electron transport; Oxidoreductase; Plant defense; Plastid; Redox-active center; Transit peptide; Transport
SRR342023_TR19798_c0_g2_i9_len=1952	GCR2_ARATH	Q:1940-1059,H:116-410	71.86%ID	E:5e-150	LanC-like protein GCR2	PF05147.10^LANC_like^Lanthionine synthetase C-like protein^4-298^E:6.1e-84	0,12	-0,681	0	-3,22	1	0,522	1	0,421	0,791	0,066	GCR2_ARATH	LanC-like protein GCR2 (G-protein coupled receptor 2)	GCR2 GPCR At1g52920 F14G24.19	Arabidopsis thaliana (Mouse-ear cress)		3D-structure; Abscisic acid signaling pathway; Complete proteome; Metal-binding; Reference proteome; Zinc
SRR342023_TR19811_c1_g3_i1_len=1011	NA	NA	NA	NA	NA	PF03745.11^DUF309^Domain of unknown function (DUF309)^84-140^E:3.1e-20	0,568	0,191	0	-1,195	0,006	-0,933	0,003	-1,027	0,031	0,699	.	.	.	.	.	.
SRR342023_TR19929_c1_g1_i5_len=852	NA	NA	NA	NA	NA	.	1	0	0	-7,661	1	0	1	0	1	0	.	.	.	.	.	.
SRR342023_TR20482_c2_g7_i2_len=749	RS4_SOLTU	Q:3-491,H:101-264	91.46%ID	E:1e-87	40S ribosomal protein S4	PF00900.17^Ribosomal_S4e^Ribosomal family S4e^1-69^E:1.9e-32`PF00467.26^KOW^KOW motif^77-110^E:6.1e-07`PF16121.2^40S_S4_C^40S ribosomal protein S4 C-terminus^112-157^E:2.1e-26	0,634	-0,241	0,001	-1,016	0,013	1,145	0,496	0,417	0,054	-0,472	RS4_SOLTU	40S ribosomal protein S4	RPS4	Solanum tuberosum (Potato)		Complete proteome; Cytoplasm; RNA-binding; Reference proteome; Ribonucleoprotein; Ribosomal protein; rRNA-binding
SRR342023_TR21200_c1_g6_i8_len=5341	IF2C_PHAVU	Q:2788-4968,H:277-999	80.91%ID	E:0	Translation initiation factor IF-2, chloroplastic	PF01985.18^CRS1_YhbY^CRS1 / YhbY (CRM) domain^51-133^E:3.5e-24`PF01985.18^CRS1_YhbY^CRS1 / YhbY (CRM) domain^248-332^E:2.4e-20`PF01985.18^CRS1_YhbY^CRS1 / YhbY (CRM) domain^459-545^E:6.5e-18	0,031	-1,36	0	-2,272	0,402	1,106	0,76	0,675	0,221	-0,421	IF2C_PHAVU	Translation initiation factor IF-2, chloroplastic (PvIF2cp)	IF2CP	Phaseolus vulgaris (Kidney bean) (French bean)		Chloroplast; GTP-binding; Initiation factor; Nucleotide-binding; Plastid; Protein biosynthesis; Transit peptide
SRR342023_TR21417_c0_g1_i1_len=962	NA	NA	NA	NA	NA	PF06364.9^DUF1068^Protein of unknown function (DUF1068)^10-174^E:6.7e-65	1	-0,069	0	-6,514	0,518	0,33	1	-0,062	1	1,687	.	.	.	.	.	.
SRR342023_TR22292_c1_g3_i1_len=744	NA	NA	NA	NA	NA	.	1	0,061	0	-2,792	0,299	-1,407	0,517	-1,245	0,229	-0,439	.	.	.	.	.	.
SRR342023_TR23173_c0_g1_i2_len=2137	SR542_SOLLC	Q:1888-452,H:1-479	94.57%ID	E:0	Signal recognition particle 54 kDa protein 2	PF02881.16^SRP54_N^SRP54-type protein, helical bundle domain^6-83^E:9.8e-16`PF00448.19^SRP54^SRP54-type protein, GTPase domain^101-296^E:1.2e-78`PF02492.16^cobW^CobW/HypB/UreG, nucleotide-binding domain^103-254^E:8.2e-07`PF02978.16^SRP_SPB^Signal peptide binding domain^328-427^E:2e-27	1	-1,272	0	-6,595	0,874	0,216	1	0,091	1	0	SR542_SOLLC	Signal recognition particle 54 kDa protein 2 (SRP54)		Solanum lycopersicum (Tomato) (Lycopersicon esculentum)		Complete proteome; Cytoplasm; GTP-binding; Nucleotide-binding; RNA-binding; Reference proteome; Ribonucleoprotein; Signal recognition particle
SRR342023_TR23641_c4_g4_i4_len=1761	F16P1_PEA	Q:1526-306,H:6-407	81.07%ID	E:0	Fructose-1,6-bisphosphatase, chloroplastic	PF00316.17^FBPase^Fructose-1-6-bisphosphatase, N-terminal domain^80-279^E:6.7e-56	0,268	-0,27	0	-1,053	0,033	-0,48	0,634	-0,107	0,112	-0,536	F16P1_PEA	Fructose-1,6-bisphosphatase, chloroplastic (FBPase) (EC 3.1.3.11) (D-fructose-1,6-bisphosphate 1-phosphohydrolase)	FBP	Pisum sativum (Garden pea)	PATHWAY: Carbohydrate biosynthesis; Calvin cycle.	3D-structure; Calvin cycle; Carbohydrate metabolism; Chloroplast; Disulfide bond; Hydrolase; Magnesium; Metal-binding; Plastid; Transit peptide
SRR342023_TR24357_c2_g3_i7_len=985	SABP2_TOBAC	Q:562-137,H:120-260	57.75%ID	E:8e-47	Salicylic acid-binding protein 2	.	0,904	-0,135	0	-2,361	0,002	-1,354	0,015	-1,015	0,555	-0,309	SABP2_TOBAC	Salicylic acid-binding protein 2 (NtSABP2) (EC 3.1.1.-) (Methyl salicylate esterase)	SABP2	Nicotiana tabacum (Common tobacco)		3D-structure; Hydrolase; Immunity; Innate immunity; Plant defense
SRR342023_TR24567_c0_g3_i1_len=1091	NA	NA	NA	NA	NA	.	0,828	-0,078	0	-1,333	0,203	-0,416	0,606	-0,171	0,572	-0,194	.	.	.	.	.	.
SRR342023_TR24647_c0_g1_i1_len=821	ML423_ARATH	Q:37-444,H:17-150	52.21%ID	E:4e-36	MLP-like protein 423	.	0,892	-0,045	0	-2,357	0,002	-1,066	0,001	-1,109	0,001	0,85	ML423_ARATH	MLP-like protein 423	MLP423 At1g24020 T23E23.17 T23E23_22	Arabidopsis thaliana (Mouse-ear cress)		Complete proteome; Reference proteome
SRR342023_TR24795_c0_g5_i1_len=4004	TPL_ARATH	Q:225-3602,H:1-1131	79.51%ID	E:0	Protein TOPLESS	PF00400.29^WD40^WD domain, G-beta repeat^456-490^E:1.8e-05`PF00400.29^WD40^WD domain, G-beta repeat^914-948^E:0.0026	1	-0,172	0	-6,48	0,211	0,761	0,412	0,563	1	0,207	TPL_ARATH	Protein TOPLESS (WUS-interacting protein 1)	TPL WSIP1 At1g15750 F7H2.9	Arabidopsis thaliana (Mouse-ear cress)		Complete proteome; Developmental protein; Jasmonic acid signaling pathway; Nucleus; Phosphoprotein; Reference proteome; Repeat; Repressor; Transcription; Transcription regulation; WD repeat
SRR342023_TR24795_c1_g8_i1_len=3098	TGD1_ARATH	Q:1857-940,H:41-347	75.08%ID	E:3e-158	Protein TRIGALACTOSYLDIACYLGLYCEROL 1, chloroplastic {ECO:0000303|PubMed:12743031}	PF02405.13^MlaE^Permease MlaE^130-338^E:8e-69	1	-0,364	0	-4,647	0,35	0,65	0,739	0,279	1	0,349	TGD1_ARATH	Protein TRIGALACTOSYLDIACYLGLYCEROL 1, chloroplastic (ABC transporter I family member 14) (ABC transporter ABCI.14) (AtABCI14)	TGD1 ABCI14 At1g19800 F14P1.27	Arabidopsis thaliana (Mouse-ear cress)		Chloroplast; Complete proteome; Lipid transport; Membrane; Plastid; Plastid inner membrane; Reference proteome; Transit peptide; Transmembrane; Transmembrane helix; Transport
SRR342023_TR25706_c1_g4_i4_len=1138	B561O_ARATH	Q:1065-325,H:141-391	54.76%ID	E:1e-69	Cytochrome b561 and DOMON domain-containing protein At4g12980	.	1	-0,206	0	-2,048	0,892	0,101	0,656	-0,318	0,044	-0,678	B561O_ARATH	Cytochrome b561 and DOMON domain-containing protein At4g12980 (Protein b561A.tha15)	At4g12980 F25G13.70	Arabidopsis thaliana (Mouse-ear cress)		Complete proteome; Electron transport; Heme; Iron; Membrane; Metal-binding; Reference proteome; Signal; Transmembrane; Transmembrane helix; Transport
SRR342023_TR26030_c1_g1_i1_len=1615	YUGF_BACSU	Q:460-1266,H:17-264	28.68%ID	E:1e-22	Uncharacterized hydrolase YugF	PF12146.5^Hydrolase_4^Serine aminopeptidase, S33^105-218^E:1.7e-08`PF00561.17^Abhydrolase_1^alpha/beta hydrolase fold^107-360^E:1.2e-16`PF12697.4^Abhydrolase_6^Alpha/beta hydrolase family^109-366^E:4.6e-21	0,507	-0,239	0	-1,286	0,29	-0,379	0,405	-0,285	0,502	-0,226	YUGF_BACSU	Uncharacterized hydrolase YugF (EC 3.1.-.-)	yugF BSU31420	Bacillus subtilis (strain 168)		Complete proteome; Hydrolase; Reference proteome; Serine esterase
SRR342023_TR26965_c0_g9_i3_len=1612	NA	NA	NA	NA	NA	PF04450.9^BSP^Peptidase of plants and bacteria^95-305^E:5.2e-17	1	0,035	0	-2,26	0,01	-0,914	0,007	-0,967	0,6	-0,332	.	.	.	.	.	.
SRR342023_TR27467_c3_g4_i5_len=5411	POLX_TOBAC	Q:5411-2034,H:221-1323	43.99%ID	E:0	Retrovirus-related Pol polyprotein from transposon TNT 1-94	PF13976.3^gag_pre-integrs^GAG-pre-integrase domain^183-250^E:2.8e-14`PF00665.23^rve^Integrase core domain^266-380^E:6e-26`PF07727.11^RVT_2^Reverse transcriptase (RNA-dependent DNA polymerase)^640-887^E:4.5e-93	0,61	0,196	0	-1,339	0,127	-0,592	0,005	-1,166	0,021	1,038	POLX_TOBAC	Retrovirus-related Pol polyprotein from transposon TNT 1-94 [Includes: Protease (EC 3.4.23.-); Reverse transcriptase (EC 2.7.7.49); Endonuclease]		Nicotiana tabacum (Common tobacco)		Aspartyl protease; Endonuclease; Hydrolase; Metal-binding; Nuclease; Nucleotidyltransferase; Protease; RNA-directed DNA polymerase; Transferase; Transposable element; Zinc; Zinc-finger
SRR342023_TR27467_c3_g4_i8_len=1394	NA	NA	NA	NA	NA	PF06108.9^DUF952^Protein of unknown function (DUF952)^19-54^E:7.6e-08	0	1,571	0	-2,315	0,132	0,762	0,496	0,388	1	-2,002	.	.	.	.	.	.
SRR342023_TR28415_c3_g2_i1_len=1319	NA	NA	NA	NA	NA	PF02544.13^Steroid_dh^3-oxo-5-alpha-steroid 4-dehydrogenase^158-269^E:2.1e-26	0,641	-0,248	0	-2,211	0,019	-0,691	0,076	-0,519	1	0,732	.	.	.	.	.	.
SRR342023_TR30149_c2_g2_i6_len=1513	NA	NA	NA	NA	NA	.	1	-1,159	0	-7,003	1	-0,077	1	-0,113	1	0	.	.	.	.	.	.
SRR342023_TR30149_c2_g2_i15_len=2948	NA	NA	NA	NA	NA	.	1	-0,105	0	-6,401	0,618	-0,264	0,177	-0,663	1	-0,478	.	.	.	.	.	.
SRR342023_TR30634_c0_g2_i1_len=404	NA	NA	NA	NA	NA	.	0,722	-0,133	0	-1,435	0,705	0,223	1	-0,042	0,04	-0,683	.	.	.	.	.	.
SRR342023_TR30649_c0_g5_i2_len=1314	CCD4_ARATH	Q:1260-274,H:266-595	73.49%ID	E:4e-175	Probable carotenoid cleavage dioxygenase 4, chloroplastic	PF03055.12^RPE65^Retinal pigment epithelial membrane protein^3-305^E:3.4e-59	0,167	0,602	0	-1,133	0,01	-0,872	0,132	-0,493	0,8	0,095	CCD4_ARATH	Probable carotenoid cleavage dioxygenase 4, chloroplastic (AtCCD4) (EC 1.14.99.-) (AtNCED4)	CCD4 NCED4 At4g19170 T18B16.140	Arabidopsis thaliana (Mouse-ear cress)		Chloroplast; Complete proteome; Dioxygenase; Iron; Metal-binding; Oxidoreductase; Plastid; Reference proteome; Transit peptide
SRR342023_TR30982_c3_g1_i18_len=2351	FKB70_WHEAT	Q:757-1812,H:93-423	47.75%ID	E:3e-117	70 kDa peptidyl-prolyl isomerase	PF00254.25^FKBP_C^FKBP-type peptidyl-prolyl cis-trans isomerase^10-62^E:4.3e-18`PF00254.25^FKBP_C^FKBP-type peptidyl-prolyl cis-trans isomerase^89-149^E:1.4e-06	0,591	-0,314	0	-2,632	0,781	-0,566	1	-0,33	0,079	-1,11	FKB70_WHEAT	70 kDa peptidyl-prolyl isomerase (EC 5.2.1.8) (Peptidyl-prolyl cis-trans isomerase) (PPIase) (Rotamase)	FKBP70	Triticum aestivum (Wheat)		3D-structure; Calmodulin-binding; Complete proteome; Isomerase; Reference proteome; Repeat; Rotamase; Stress response; TPR repeat
SRR342023_TR30982_c3_g3_i1_len=2169	NA	NA	NA	NA	NA	.	0,684	0,156	0	-2,056	0,136	-0,594	0,014	-0,968	0,606	-0,182	.	.	.	.	.	.
SRR342023_TR31039_c0_g1_i1_len=1134	GTOMC_ORYSJ	Q:34-906,H:73-359	44.67%ID	E:4e-75	Probable tocopherol O-methyltransferase, chloroplastic	PF13489.3^Methyltransf_23^Methyltransferase domain^63-195^E:2.9e-14`PF01209.15^Ubie_methyltran^ubiE/COQ5 methyltransferase family^75-187^E:3.3e-11`PF13847.3^Methyltransf_31^Methyltransferase domain^79-193^E:1.2e-19`PF02353.17^CMAS^Mycolic acid cyclopropane synthetase^79-183^E:4.7e-11`PF09445.7^Methyltransf_15^RNA cap guanine-N2 methyltransferase^82-156^E:7e-07`PF07021.9^MetW^Methionine biosynthesis protein MetW^82-175^E:1.9e-06`PF08241.9^Methyltransf_11^Methyltransferase domain^84-182^E:4.4e-20`PF13649.3^Methyltransf_25^Methyltransferase domain^84-177^E:2e-18`PF08242.9^Methyltransf_12^Methyltransferase domain^84-180^E:1.6e-12	0,451	-0,324	0	-5,074	0,402	-1,071	0,118	-1,852	0,275	-0,382	GTOMC_ORYSJ	Probable tocopherol O-methyltransferase, chloroplastic (EC 2.1.1.95) (Gamma-tocopherol methyltransferase) (Vitamin E pathway gene 4 protein)	VTE4 G-TMT TMT Os02g0701600 LOC_Os02g47310 OJ1111_E07.25	Oryza sativa subsp. japonica (Rice)	PATHWAY: Cofactor biosynthesis; tocopherol biosynthesis.	Chloroplast; Complete proteome; Methyltransferase; Plastid; Reference proteome; S-adenosyl-L-methionine; Transferase; Transit peptide
SRR342023_TR31168_c1_g1_i1_len=1545	RK5_ARATH	Q:1408-743,H:37-259	76.23%ID	E:1e-121	50S ribosomal protein L5, chloroplastic	PF00673.18^Ribosomal_L5_C^ribosomal L5P family C-terminus^1-82^E:3.1e-27	0,029	-0,533	0	-1,201	0,005	-0,733	0,016	-0,625	1	-0,337	RK5_ARATH	50S ribosomal protein L5, chloroplastic	RPL5 At4g01310 A_IG002N01.3 F2N1.5	Arabidopsis thaliana (Mouse-ear cress)		Chloroplast; Complete proteome; Plastid; RNA-binding; Reference proteome; Ribonucleoprotein; Ribosomal protein; Transit peptide; rRNA-binding
SRR342023_TR31533_c1_g4_i1_len=1529	ENOX_SOLLC	Q:719-1285,H:200-388	87.3%ID	E:3e-125	2-methylene-furan-3-one reductase	PF08240.9^ADH_N^Alcohol dehydrogenase GroES-like domain^109-172^E:1.3e-09	0,314	-0,264	0	-0,914	0,019	-0,538	0,08	-0,399	0,294	0,531	ENOX_SOLLC	2-methylene-furan-3-one reductase (EC 1.3.1.105) (Enone oxidoreductase) (SlEO)	EO QR Solyc05g005480	Solanum lycopersicum (Tomato) (Lycopersicon esculentum)		Chloroplast; Complete proteome; NAD; NADP; Oxidoreductase; Plastid; Reference proteome; Transit peptide
SRR646572_TR6692_c0_g1_i22_len=1249	NA	NA	NA	NA	NA	.	0,472	-0,305	0,001	-1,501	0,333	0,633	0,267	0,712	0,206	-0,722	.	.	.	.	.	.
SRR646572_TR9781_c1_g1_i1_len=730	SAC1_ARATH	Q:669-313,H:792-910	55.46%ID	E:1e-33	Phosphoinositide phosphatase SAC1	.	1	0	0	-6,791	0,254	2,581	1	-0,671	0,381	2,257	SAC1_ARATH	Phosphoinositide phosphatase SAC1 (AtSAC1) (EC 3.1.3.-) (Factor-induced gene 4-like protein) (AtFIG4) (Phosphatidylinositol 3,5-bisphosphate 5-phosphatase SAC1) (Protein FRAGILE FIBER 7) (Protein SUPPRESSOR OF ACTIN 1) (SAC domain protein 1)	SAC1 FIG4 FRA7 At1g22620 F12K8.3 T22J18.20	Arabidopsis thaliana (Mouse-ear cress)		Complete proteome; Golgi apparatus; Hydrolase; Membrane; Reference proteome; Vacuole
SRR646572_TR12509_c0_g2_i6_len=1712	NA	NA	NA	NA	NA	PF15459.3^RRP14^60S ribosome biogenesis protein Rrp14^38-71^E:4e-05`PF04935.9^SURF6^Surfeit locus protein 6^122-312^E:5.3e-22	0,014	-3,489	0	-6,792	0,005	6,237	1	0	0,502	-1,662	.	.	.	.	.	.
SRR646572_TR20512_c0_g1_i1_len=1371	CP20B_ARATH	Q:152-910,H:1-256	65.12%ID	E:2e-88	.	PF00160.18^Pro_isomerase^Cyclophilin type peptidyl-prolyl cis-trans isomerase/CLD^145-299^E:7.5e-51	0,002	-0,778	0	-1,338	0,105	-1,063	0,211	-0,765	0,068	-0,399	PNSL5_ARATH	Photosynthetic NDH subunit of lumenal location 5, chloroplastic (Cyclophilin of 20 kDa 2) (Peptidyl-prolyl cis-trans isomerase CYP20-2) (PPIase CYP20-2) (EC 5.2.1.8) (Rotamase CYP20-2) (Thylakoid lumen PPIase of 20 kDa) (TLP20)	PNSL5 CYP20-2 At5g13120 T19L5_80	Arabidopsis thaliana (Mouse-ear cress)		Alternative splicing; Chaperone; Chloroplast; Complete proteome; Cyclosporin; Direct protein sequencing; Disulfide bond; Isomerase; Membrane; Plastid; Reference proteome; Rotamase; Thylakoid; Transit peptide; Transport
SRR646572_TR30268_c0_g1_i1_len=737	NA	NA	NA	NA	NA	.	0	8,205	0	-10,998	1	0	1	0	1	0	.	.	.	.	.	.
SRR646572_TR34996_c0_g1_i6_len=1831	TIG_ARATH	Q:1734-268,H:1-547	57.59%ID	E:0	Trigger factor-like protein TIG, Chloroplastic	PF05698.11^Trigger_C^Bacterial trigger factor protein (TF) C-terminus^353-510^E:7.5e-20	0,023	-0,707	0	-1,42	1	-0,024	0,865	0,212	0,933	-0,035	TIG_ARATH	Trigger factor-like protein TIG, Chloroplastic (EC 5.2.1.8) (Immunophilin TIG) (Peptidyl-prolyl cis-trans isomerase TIG) (PPIase TIG) (Rotamase)	TIG At5g55220 MCO15_17	Arabidopsis thaliana (Mouse-ear cress)		Acetylation; Chaperone; Chloroplast; Complete proteome; Isomerase; Plastid; Reference proteome; Rotamase; Transit peptide
SRR646572_TR36732_c0_g1_i2_len=2339	ARID3_ARATH	Q:862-1809,H:477-784	70.03%ID	E:8e-138	AT-rich interactive domain-containing protein 3	PF01388.18^ARID^ARID/BRIGHT DNA binding domain^309-393^E:2e-18`PF00011.18^HSP20^Hsp20/alpha crystallin family^522-603^E:1.9e-05	1	1,575	0	-6,792	0,502	3,734	1	0	0,282	-1,289	ARID3_ARATH	AT-rich interactive domain-containing protein 3 (ARID domain-containing protein 3)	ARID3 At2g17410 F5J6.17	Arabidopsis thaliana (Mouse-ear cress)		Alternative splicing; Complete proteome; DNA-binding; Nucleus; Reference proteome; Transcription; Transcription regulation
SRR646572_TR37751_c0_g3_i1_len=1450	NA	NA	NA	NA	NA	.	0,739	-0,284	0,001	-2,174	0,072	-0,554	0,006	-0,848	0,92	0,109	.	.	.	.	.	.
SRR646596_TR1900_c1_g1_i1_len=521	NA	NA	NA	NA	NA	.	0,679	0,447	0,001	-1,61	1	-0,119	0,189	-1,614	0,012	1,099	.	.	.	.	.	.
SRR646596_TR4348_c0_g1_i2_len=1569	AT74_ARATH	Q:1290-352,H:9-275	46.33%ID	E:6e-86	Phosphoglycerate mutase-like protein AT74 {ECO:0000305}	PF00300.19^His_Phos_1^Histidine phosphatase superfamily (branch 1)^7-119^E:8e-10	0,541	-0,311	0	-2,339	0,312	0,549	0,206	0,658	1	-3,439	AT74_ARATH	Phosphoglycerate mutase-like protein AT74 (At-74)	At3g05170 T12H1.14	Arabidopsis thaliana (Mouse-ear cress)		Complete proteome; Reference proteome
SRR646596_TR7506_c1_g2_i11_len=1637	NA	NA	NA	NA	NA	.	1	0,316	0	-3,272	0,878	-0,168	0,635	-0,375	0,073	-2,088	.	.	.	.	.	.
SRR646596_TR12031_c0_g2_i1_len=780	NA	NA	NA	NA	NA	PF10950.5^DUF2775^Protein of unknown function (DUF2775)^42-152^E:2.5e-22`PF10950.5^DUF2775^Protein of unknown function (DUF2775)^159-230^E:2.5e-15	0,061	0,493	0,001	-3,029	1	1,311	1	1,875	0	0,937	.	.	.	.	.	.
SRR646596_TR17603_c0_g1_i1_len=1150	CB13_SOLLC	Q:884-273,H:70-273	91.18%ID	E:9e-133	Chlorophyll a-b binding protein 8, chloroplastic	PF00504.18^Chloroa_b-bind^Chlorophyll A-B binding protein^1-135^E:3.5e-31	0,39	-0,211	0	-2,046	0,006	-0,627	0,024	-0,511	0,152	-0,896	CB13_SOLLC	Chlorophyll a-b binding protein 8, chloroplastic (LHCI type III CAB-8)	CAB8	Solanum lycopersicum (Tomato) (Lycopersicon esculentum)		Acetylation; Chlorophyll; Chloroplast; Chromophore; Complete proteome; Magnesium; Membrane; Metal-binding; Phosphoprotein; Photosynthesis; Photosystem I; Photosystem II; Plastid; Reference proteome; Thylakoid; Transit peptide; Transmembrane; Transmembrane helix
SRR646596_TR19434_c0_g1_i1_len=1221	NA	NA	NA	NA	NA	PF10674.6^Ycf54^Protein of unknown function (DUF2488)^92-181^E:2.2e-34	0,002	-0,748	0	-1,228	0,057	-0,536	0,494	-0,191	1	0,2	.	.	.	.	.	.
SRR646596_TR20484_c0_g1_i1_len=1373	NA	NA	NA	NA	NA	PF01165.17^Ribosomal_S21^Ribosomal protein S21^96-150^E:3e-19	0,091	-0,44	0	-1,411	0,167	-0,4	0,215	-0,353	1	-0,789	.	.	.	.	.	.
SRR646596_TR23715_c0_g1_i1_len=620	PLDA1_RICCO	Q:3-359,H:690-808	80.67%ID	E:6e-63	Phospholipase D alpha 1	PF12357.5^PLD_C^Phospholipase D C terminal^37-109^E:1.5e-29	0,333	0,433	0	-1,945	0,001	0,963	0,2	0,415	0,441	-0,226	PLDA1_RICCO	Phospholipase D alpha 1 (PLD 1) (EC 3.1.4.4) (Choline phosphatase 1) (Phosphatidylcholine-hydrolyzing phospholipase D 1)	PLD1	Ricinus communis (Castor bean)		Calcium; Cell membrane; Cytoplasm; Direct protein sequencing; Endoplasmic reticulum; Hydrolase; Lipid degradation; Lipid metabolism; Membrane; Plastid; Repeat; Vacuole
SRR646596_TR25866_c0_g1_i1_len=887	NA	NA	NA	NA	NA	.	0,136	-0,389	0	-1,36	0,373	0,226	0,031	0,533	1	-0,055	.	.	.	.	.	.
SRR646596_TR27670_c0_g1_i1_len=510	NA	NA	NA	NA	NA	.	0,146	-0,534	0	-2,175	0,107	-0,791	0,056	-0,942	1	0,446	.	.	.	.	.	.
SRR646596_TR29225_c0_g1_i1_len=785	RK29_ARATH	Q:153-599,H:3-158	69.23%ID	E:2e-55	50S ribosomal protein L29, chloroplastic	.	0,02	-0,555	0	-1,423	0,558	-0,163	0,709	-0,1	0,668	-0,098	RK29_ARATH	50S ribosomal protein L29, chloroplastic (CL29)	RPL29 At5g65220 MQN23.16	Arabidopsis thaliana (Mouse-ear cress)		Chloroplast; Complete proteome; Phosphoprotein; Plastid; Reference proteome; Ribonucleoprotein; Ribosomal protein; Transit peptide
SRR646596_TR29262_c0_g2_i2_len=7462	RK2A_COFAR	Q:7035-6214,H:1-274	98.91%ID	E:2e-167	50S ribosomal protein L2-A, chloroplastic	PF01193.21^RNA_pol_L^RNA polymerase Rpb3/Rpb11 dimerisation domain^35-225^E:6.3e-14`PF01000.23^RNA_pol_A_bac^RNA polymerase Rpb3/RpoA insert domain^68-157^E:3.6e-15	0,004	-0,941	0	-3,297	0,008	-1,303	0,002	-1,537	0,094	-1,34	RK2A_COFAR	50S ribosomal protein L2-A, chloroplastic	rpl2-A	Coffea arabica (Arabian coffee)		Chloroplast; Plastid; Ribonucleoprotein; Ribosomal protein
SRR646596_TR30848_c0_g2_i4_len=1228	NDUB2_ARATH	Q:643-783,H:11-57	82.98%ID	E:8e-21	NADH dehydrogenase [ubiquinone] 1 beta subcomplex subunit 2	.	1	-0,963	0	-3,557	1	2,24	1	0,154	1	-1,677	NDUB2_ARATH	NADH dehydrogenase [ubiquinone] 1 beta subcomplex subunit 2	At1g76200 T23E18.30	Arabidopsis thaliana (Mouse-ear cress)		Complete proteome; Electron transport; Membrane; Mitochondrion; Mitochondrion inner membrane; Reference proteome; Respiratory chain; Transport
SRR646604_TR163_c0_g1_i1_len=1142	PSAN_ARATH	Q:674-171,H:1-168	69.19%ID	E:1e-59	Photosystem I reaction center subunit N, chloroplastic	PF05479.8^PsaN^Photosystem I reaction centre subunit N (PSAN or PSI-N)^117-245^E:8.5e-68	1	-0,057	0	-1,234	0,049	-0,557	0,396	-0,238	1	-0,046	PSAN_ARATH	Photosystem I reaction center subunit N, chloroplastic (PSI-N)	PSAN At5g64040 MHJ24.2	Arabidopsis thaliana (Mouse-ear cress)		Acetylation; Alternative splicing; Chloroplast; Complete proteome; Direct protein sequencing; Membrane; Photosynthesis; Photosystem I; Plastid; Reference proteome; Thylakoid; Transit peptide
SRR646604_TR7508_c0_g2_i1_len=2895	BGAL5_ARATH	Q:2586-430,H:13-731	70.65%ID	E:0	Beta-galactosidase 5	PF01301.16^Glyco_hydro_35^Glycosyl hydrolases family 35^35-339^E:1.9e-121`PF02449.12^Glyco_hydro_42^Beta-galactosidase^56-203^E:4.4e-08	0,198	-0,454	0	-2,114	0,042	-1,866	0,381	-0,831	0,692	1,165	BGAL5_ARATH	Beta-galactosidase 5 (Lactase 5) (EC 3.2.1.23)	BGAL5 At1g45130 F27F5.20	Arabidopsis thaliana (Mouse-ear cress)		Apoplast; Complete proteome; Glycoprotein; Glycosidase; Hydrolase; Reference proteome; Secreted; Signal
SRR646604_TR12279_c0_g2_i1_len=655	NA	NA	NA	NA	NA	.	0,232	-0,316	0,001	-0,926	0	1,237	0,001	1,067	0,86	-0,191	.	.	.	.	.	.
SRR646604_TR13117_c0_g1_i1_len=1014	NA	NA	NA	NA	NA	.	0,431	-0,412	0	-2,338	1	0,06	1	-1,127	0,079	-1,098	.	.	.	.	.	.
SRR646604_TR13905_c0_g1_i3_len=984	GLO2_ARATH	Q:867-283,H:171-365	90.77%ID	E:3e-123	Peroxisomal (S)-2-hydroxy-acid oxidase GLO2	PF01070.15^FMN_dh^FMN-dependent dehydrogenase^7-165^E:2e-69`PF00478.22^IMPDH^IMP dehydrogenase / GMP reductase domain^74-121^E:1.4e-06`PF01645.14^Glu_synthase^Conserved region in glutamate synthase^86-125^E:0.00022	0,574	0,186	0	-1,455	0,364	-0,2	0,165	-0,308	0,272	-0,495	GLO2_ARATH	Peroxisomal (S)-2-hydroxy-acid oxidase GLO2 (EC 1.1.3.15) (Glycolate oxidase 1) (AtGLO2) (GOX 1) (Short chain alpha-hydroxy acid oxidase GLO2)	GLO2 GOX1 At3g14415 MLN21.20	Arabidopsis thaliana (Mouse-ear cress)	PATHWAY: Photosynthesis; photorespiration; glycine from 2-phosphoglycolate: step 2/3.	Acetylation; Alternative splicing; Complete proteome; Direct protein sequencing; FMN; Flavoprotein; Glycolate pathway; Oxidoreductase; Peroxisome; Photorespiration; Reference proteome
SRR646604_TR14910_c0_g1_i1_len=1002	RRP31_ARATH	Q:465-710,H:75-156	79.27%ID	E:2e-41	30S ribosomal protein 3-1, chloroplastic	PF04839.10^PSRP-3_Ycf65^Plastid and cyanobacterial ribosomal protein (PSRP-3 / Ycf65)^146-192^E:1.5e-26	0,028	-0,511	0	-1,335	0,474	-0,213	0,246	0,321	0,695	-0,097	RRP31_ARATH	30S ribosomal protein 3-1, chloroplastic (Plastid-specific 30S ribosomal protein 3-1) (PSRP-3 1)	At1g68590 F24J5.17	Arabidopsis thaliana (Mouse-ear cress)		Alternative splicing; Chloroplast; Complete proteome; Plastid; Reference proteome; Ribonucleoprotein; Ribosomal protein; Transit peptide
SRR646604_TR16238_c0_g1_i1_len=948	CB4A_SOLLC	Q:884-237,H:42-256	91.2%ID	E:2e-130	Chlorophyll a-b binding protein CP24 10A, chloroplastic	PF00504.18^Chloroa_b-bind^Chlorophyll A-B binding protein^49-225^E:1.5e-46	0,214	-0,476	0	-2,112	0,17	-0,514	0,422	-0,294	0,878	-0,172	CB4A_SOLLC	Chlorophyll a-b binding protein CP24 10A, chloroplastic (CAB-10A) (LHCP)	CAP10A	Solanum lycopersicum (Tomato) (Lycopersicon esculentum)		Chlorophyll; Chloroplast; Chromophore; Complete proteome; Membrane; Photosynthesis; Photosystem II; Plastid; Reference proteome; Thylakoid; Transit peptide; Transmembrane; Transmembrane helix
SRR646604_TR17584_c0_g2_i1_len=1703	CHLP_TOBAC	Q:242-1618,H:1-460	88.04%ID	E:0	Geranylgeranyl diphosphate reductase, chloroplastic	PF01134.19^GIDA^Glucose inhibited division protein A^54-88^E:8.9e-05	1	-0,003	0	-4,322	1	0,525	1	-0,202	0,004	-1,465	CHLP_TOBAC	Geranylgeranyl diphosphate reductase, chloroplastic (EC 1.3.1.83) (Geranylgeranyl reductase)	CHLP	Nicotiana tabacum (Common tobacco)	PATHWAY: Porphyrin-containing compound metabolism; chlorophyll biosynthesis.; PATHWAY: Cofactor biosynthesis; tocopherol biosynthesis.	Chlorophyll biosynthesis; Chloroplast; NADP; Oxidoreductase; Photosynthesis; Plastid; Transit peptide
SRR646604_TR18739_c0_g1_i1_len=1931	MDHP_MEDSA	Q:337-1599,H:18-437	85.75%ID	E:0	Malate dehydrogenase [NADP], chloroplastic	PF00056.20^Ldh_1_N^lactate/malate dehydrogenase, NAD binding domain^102-249^E:2.9e-34`PF02866.15^Ldh_1_C^lactate/malate dehydrogenase, alpha/beta C-terminal domain^252-422^E:1.6e-37	0,758	-0,144	0	-1,552	0,153	-0,341	0,372	-0,215	0,594	0,135	MDHP_MEDSA	Malate dehydrogenase [NADP], chloroplastic (EC 1.1.1.82) (NADP-MDH)	MDH1 P1MDH	Medicago sativa (Alfalfa)		Chloroplast; Disulfide bond; NADP; Oxidoreductase; Plastid; Transit peptide
SRR646604_TR19881_c0_g1_i3_len=1045	CW15A_XENLA	Q:948-259,H:1-228	48.54%ID	E:1e-26	Protein CWC15 homolog A	PF04889.9^Cwf_Cwc_15^Cwf15/Cwc15 cell cycle control protein^1-230^E:8.1e-78	0,76	-0,604	0,001	-2,873	1	-0,425	1	-3,221	1	-1,43	CW15A_XENLA	Protein CWC15 homolog A	cwc15-a	Xenopus laevis (African clawed frog)		Coiled coil; mRNA processing; mRNA splicing
SRR646604_TR22490_c0_g2_i1_len=859	NA	NA	NA	NA	NA	PF04949.10^Transcrip_act^Transcriptional activator^23-170^E:1.6e-74	0,097	-0,458	0	-1,262	0,77	-0,125	0,62	-0,228	0,001	-1,321	.	.	.	.	.	.
SRR646604_TR37240_c0_g1_i5_len=1283	ILV5_ARATH	Q:1185-247,H:273-585	91.05%ID	E:0	Ketol-acid reductoisomerase, chloroplastic	PF01450.16^IlvC^Acetohydroxy acid isomeroreductase, catalytic domain^39-183^E:1.7e-34`PF01450.16^IlvC^Acetohydroxy acid isomeroreductase, catalytic domain^196-272^E:3.6e-08	0,218	-0,364	0,001	-0,881	0,044	0,691	0,077	0,626	1	-2,096	ILV5_ARATH	Ketol-acid reductoisomerase, chloroplastic (EC 1.1.1.86) (Acetohydroxy-acid reductoisomerase) (Alpha-keto-beta-hydroxylacyl reductoisomerase)	At3g58610 F14P22.200	Arabidopsis thaliana (Mouse-ear cress)	PATHWAY: Amino-acid biosynthesis; L-isoleucine biosynthesis; L-isoleucine from 2-oxobutanoate: step 2/4.; PATHWAY: Amino-acid biosynthesis; L-valine biosynthesis; L-valine from pyruvate: step 2/4.	Amino-acid biosynthesis; Branched-chain amino acid biosynthesis; Chloroplast; Complete proteome; Magnesium; Metal-binding; NADP; Oxidoreductase; Plastid; Reference proteome; Transit peptide
SRR648705_TR1559_c0_g1_i1_len=669	NA	NA	NA	NA	NA	.	0,785	-0,102	0	-1,077	0,744	0,118	0,891	-0,07	0,002	-0,87	.	.	.	.	.	.
SRR648705_TR5825_c0_g5_i2_len=1064	EPFL9_ARATH	Q:274-504,H:25-102	64.56%ID	E:3e-26	EPIDERMAL PATTERNING FACTOR-like protein 9 {ECO:0000303|PubMed:19435754}	PF16851.2^Stomagen^Stomagen^70-118^E:3.8e-31	0,219	-0,7	0	-1,836	0,444	-0,5	0,656	-0,273	1	0,303	EPFL9_ARATH	EPIDERMAL PATTERNING FACTOR-like protein 9 (EPF-like protein 9) [Cleaved into: Stomagen]	EPFL9 STOMAGEN At4g12970 F25G13.60	Arabidopsis thaliana (Mouse-ear cress)		3D-structure; Apoplast; Complete proteome; Developmental protein; Direct protein sequencing; Disulfide bond; Reference proteome; Secreted; Signal
SRR648705_TR8541_c2_g1_i1_len=1505	RSSA_DAUCA	Q:218-1033,H:1-269	79.12%ID	E:7e-142	40S ribosomal protein SA {ECO:0000255|HAMAP-Rule:MF_03015}	PF00318.17^Ribosomal_S2^Ribosomal protein S2^18-112^E:1.3e-13`PF00318.17^Ribosomal_S2^Ribosomal protein S2^117-182^E:1.8e-12	1	-2,173	0	-6,426	0,545	0,605	1	0,19	0,175	-0,468	RSSA_DAUCA	40S ribosomal protein SA (p40)	179B	Daucus carota (Wild carrot)		Cytoplasm; Ribonucleoprotein; Ribosomal protein
SRR648705_TR8604_c0_g1_i1_len=1078	AMPL2_ORYSJ	Q:922-377,H:415-596	90.11%ID	E:6e-115	Leucine aminopeptidase 2, chloroplastic	PF00883.18^Peptidase_M17^Cytosol aminopeptidase family, catalytic domain^5-180^E:1.7e-70	0,299	1,706	0	-1,858	0,299	1,455	1	-0,155	0,027	-1,252	AMPL2_ORYSJ	Leucine aminopeptidase 2, chloroplastic (EC 3.4.11.1) (Leucyl aminopeptidase 2) (LAP 2) (Proline aminopeptidase 2) (EC 3.4.11.5) (Prolyl aminopeptidase 2)	Os02g0794700 LOC_Os02g55140 OJ1695_H09.4 P0700F06.36	Oryza sativa subsp. japonica (Rice)		Aminopeptidase; Chloroplast; Complete proteome; Hydrolase; Metal-binding; Plastid; Protease; Reference proteome; Transit peptide; Zinc
SRR648705_TR10450_c0_g1_i1_len=2607	Y1680_ARATH	Q:2303-582,H:55-644	41.69%ID	E:9e-107	Probable leucine-rich repeat receptor-like protein kinase At1g68400	PF08263.9^LRRNT_2^Leucine rich repeat N-terminal domain^77-113^E:9.1e-07`PF13855.3^LRR_8^Leucine rich repeat^159-198^E:2.1e-06`PF12799.4^LRR_4^Leucine Rich repeats (2 copies)^163-205^E:1.4e-06`PF00560.30^LRR_1^Leucine Rich Repeat^164-186^E:0.046`PF08263.9^LRRNT_2^Leucine rich repeat N-terminal domain^214-229^E:11000`PF07714.14^Pkinase_Tyr^Protein tyrosine kinase^397-661^E:6e-27`PF00069.22^Pkinase^Protein kinase domain^397-656^E:2e-26	0,07	-0,743	0,001	-2,365	0,44	-0,681	0,62	-0,554	0,016	-1,18	Y1680_ARATH	Probable leucine-rich repeat receptor-like protein kinase At1g68400 (EC 2.7.11.1)	At1g68400 T2E12.5	Arabidopsis thaliana (Mouse-ear cress)		ATP-binding; Cell membrane; Complete proteome; Glycoprotein; Kinase; Leucine-rich repeat; Membrane; Nucleotide-binding; Phosphoprotein; Receptor; Reference proteome; Repeat; Serine/threonine-protein kinase; Signal; Transferase; Transmembrane; Transmembrane helix
SRR648705_TR14235_c1_g1_i2_len=1386	PLSC_COCNU	Q:1250-357,H:1-294	64.43%ID	E:8e-127	1-acyl-sn-glycerol-3-phosphate acyltransferase	PF01553.18^Acyltransferase^Acyltransferase^116-239^E:3.5e-29	0,38	-0,363	0,001	-1,495	0,474	-0,199	0,406	-0,232	0,595	-0,209	PLSC_COCNU	1-acyl-sn-glycerol-3-phosphate acyltransferase (1-AGP acyltransferase) (1-AGPAT) (EC 2.3.1.51) (Lysophosphatidic acid acyltransferase) (LPAAT)		Cocos nucifera (Coconut palm)		Acyltransferase; Direct protein sequencing; Lipid biosynthesis; Lipid metabolism; Membrane; Phospholipid biosynthesis; Phospholipid metabolism; Transferase; Transmembrane; Transmembrane helix
SRR648705_TR19135_c2_g2_i2_len=2661	H2A1_MEDTR	Q:268-651,H:23-148	85.16%ID	E:3e-45	Probable histone H2A.1	PF00125.21^Histone^Core histone H2A/H2B/H3/H4^86-163^E:1e-12`PF16211.2^Histone_H2A_C^C-terminus of histone H2A^166-199^E:1.8e-18	1	0	0	-6,834	1	2,087	1	0	0,036	-0,869	H2A1_MEDTR	Probable histone H2A.1	MtrDRAFT_AC149210g1v1	Medicago truncatula (Barrel medic) (Medicago tribuloides)		Chromosome; DNA-binding; Nucleosome core; Nucleus
SRR648705_TR19804_c0_g1_i1_len=1560	EXOL3_ARATH	Q:267-1214,H:23-337	81.01%ID	E:2e-178	Protein EXORDIUM-like 3	PF04674.9^Phi_1^Phosphate-induced protein 1 conserved region^60-337^E:1e-100	0,008	-0,919	0	-2,452	0,109	1,063	0,811	-0,354	0,261	-0,399	EXOL3_ARATH	Protein EXORDIUM-like 3	EXL3 At5g51550 K17N15.10	Arabidopsis thaliana (Mouse-ear cress)		Apoplast; Complete proteome; Glycoprotein; Reference proteome; Secreted; Signal
SRR648705_TR22532_c0_g1_i2_len=1582	TBL44_ARATH	Q:1370-246,H:16-402	60.57%ID	E:1e-175	Protein PMR5	PF14416.3^PMR5N^PMR5 N terminal Domain^56-108^E:2e-22`PF13839.3^PC-Esterase^GDSL/SGNH-like Acyl-Esterase family found in Pmr5 and Cas1p^109-387^E:1.3e-96	0,348	-0,302	0	-1,935	0,07	-0,597	0,131	-0,513	0,016	-1,923	TBL44_ARATH	Protein PMR5 (Powdery mildew resistance protein 5) (Protein trichome birefringence-like 44)	PMR5 TBL44 At5g58600 MZN1.6	Arabidopsis thaliana (Mouse-ear cress)		Alternative splicing; Complete proteome; Membrane; Plant defense; Reference proteome; Signal-anchor; Transmembrane; Transmembrane helix
SRR648705_TR22783_c0_g1_i1_len=1966	PLY12_ARATH	Q:1698-556,H:35-415	73.23%ID	E:0	Probable pectate lyase 12	PF00544.16^Pec_lyase_C^Pectate lyase^209-392^E:6.7e-21	0,088	-1,76	0	-4,158	1	-1,656	1	-1,656	0,502	-3,711	PLY12_ARATH	Probable pectate lyase 12 (EC 4.2.2.2)	At3g53190 T4D2.120	Arabidopsis thaliana (Mouse-ear cress)	PATHWAY: Glycan metabolism; pectin degradation; 2-dehydro-3-deoxy-D-gluconate from pectin: step 2/5.	Calcium; Complete proteome; Glycoprotein; Lyase; Metal-binding; Reference proteome; Signal
SRR648705_TR24719_c0_g1_i1_len=570	NA	NA	NA	NA	NA	.	0,581	0,317	0	-2,324	0,212	-1,052	0,133	-1,293	1	-0,058	.	.	.	.	.	.
SRR648705_TR24974_c0_g4_i2_len=1289	RK11_ARATH	Q:875-384,H:58-221	87.2%ID	E:1e-88	50S ribosomal protein L11, chloroplastic	PF03946.11^Ribosomal_L11_N^Ribosomal protein L11, N-terminal domain^87-144^E:2.4e-29`PF00298.16^Ribosomal_L11^Ribosomal protein L11, RNA binding domain^149-217^E:9.3e-24	0,018	-0,618	0	-1,702	0,009	-0,816	0,009	-0,835	0,787	-0,138	RK11_ARATH	50S ribosomal protein L11, chloroplastic (CL11)	RPL11 At1g32990 F9L11.15	Arabidopsis thaliana (Mouse-ear cress)		Chloroplast; Complete proteome; Plastid; RNA-binding; Reference proteome; Ribonucleoprotein; Ribosomal protein; Transit peptide; rRNA-binding
SRR648705_TR25614_c2_g3_i2_len=7711	RPOA_COFAR	Q:6655-7659,H:1-335	89.85%ID	E:0	DNA-directed RNA polymerase subunit alpha {ECO:0000255|HAMAP-Rule:MF_00059}	PF01193.21^RNA_pol_L^RNA polymerase Rpb3/Rpb11 dimerisation domain^35-225^E:1.1e-13`PF01000.23^RNA_pol_A_bac^RNA polymerase Rpb3/RpoA insert domain^68-157^E:5.5e-15`PF03118.12^RNA_pol_A_CTD^Bacterial RNA polymerase, alpha chain C terminal domain^263-325^E:4.5e-14	0,095	-0,644	0	-1,579	0,153	-0,739	0,2	-0,629	0,002	-0,658	RPOA_COFAR	DNA-directed RNA polymerase subunit alpha (PEP) (EC 2.7.7.6) (Plastid-encoded RNA polymerase subunit alpha) (RNA polymerase subunit alpha)	rpoA	Coffea arabica (Arabian coffee)		Chloroplast; DNA-directed RNA polymerase; Nucleotidyltransferase; Plastid; Transcription; Transferase
SRR648705_TR27382_c0_g1_i6_len=3230	GL3_ARATH	Q:895-2808,H:5-634	47.26%ID	E:2e-172	Transcription factor GLABRA 3	PF14215.3^bHLH-MYC_N^bHLH-MYC and R2R3-MYB transcription factors N-terminal^15-198^E:3.3e-54`PF00010.23^HLH^Helix-loop-helix DNA-binding domain^441-488^E:3.3e-10	0,457	-0,302	0,001	-2,597	0,629	-0,854	0,629	-0,826	0,922	-0,095	GL3_ARATH	Transcription factor GLABRA 3 (Basic helix-loop-helix protein 1) (AtMYC6) (AtbHLH1) (bHLH 1) (Protein SHAPESHIFTER) (Transcription factor EN 31) (bHLH transcription factor bHLH001)	GL3 BHLH1 EN31 MYC6 SST At5g41315 MYC6.2	Arabidopsis thaliana (Mouse-ear cress)		Activator; Complete proteome; DNA-binding; Developmental protein; Nucleus; Reference proteome; Transcription; Transcription regulation
SRR648705_TR28004_c0_g1_i1_len=1188	NA	NA	NA	NA	NA	PF02325.14^YGGT^YGGT family^168-231^E:1.7e-15	0,546	-0,165	0	-1,32	0,25	0,458	0,029	0,786	0,63	-0,14	.	.	.	.	.	.
SRR648705_TR30732_c2_g1_i1_len=740	TBB3_PEA	Q:2-343,H:299-412	98.25%ID	E:6e-73	Tubulin beta-3 chain	PF03953.14^Tubulin_C^Tubulin C-terminal domain^1-74^E:1.3e-19	0,818	-0,055	0	-1,242	0,122	0,512	0,244	-0,433	0,008	-0,596	TBB3_PEA	Tubulin beta-3 chain (Beta-3-tubulin) (Fragment)	TUBB3 TUB3	Pisum sativum (Garden pea)		Cytoplasm; Cytoskeleton; GTP-binding; Microtubule; Nucleotide-binding
SRR648705_TR31754_c0_g1_i3_len=1898	LET12_SOLLC	Q:1379-597,H:158-412	90.04%ID	E:3e-147	Homeobox protein knotted-1-like LET12	PF03790.10^KNOX1^KNOX1 domain^179-220^E:4.1e-17`PF03791.10^KNOX2^KNOX2 domain^241-291^E:1.9e-18`PF03789.10^ELK^ELK domain^349-370^E:1.1e-06`PF05920.8^Homeobox_KN^Homeobox KN domain^389-428^E:1.3e-17`PF00046.26^Homeobox^Homeobox domain^396-428^E:7.1e-05	1	-0,011	0	-1,436	0,193	-0,72	0,027	-1,37	0,306	-0,514	LET12_SOLLC	Homeobox protein knotted-1-like LET12	LET12	Solanum lycopersicum (Tomato) (Lycopersicon esculentum)		Complete proteome; DNA-binding; Homeobox; Nucleus; Reference proteome
SRR648705_TR32268_c0_g1_i1_len=1705	ICR4_ARATH	Q:1440-289,H:8-324	50.64%ID	E:1e-53	Interactor of constitutive active ROPs 4	.	0,623	-0,295	0,001	-2,473	1	0,222	1	0,134	1	-0,173	ICR4_ARATH	Interactor of constitutive active ROPs 4 (ROP-interactive partner 4)	ICR4 RIP4 At1g78430 F3F9.6	Arabidopsis thaliana (Mouse-ear cress)		Coiled coil; Complete proteome; Reference proteome
SRR648705_TR32669_c0_g1_i1_len=1030	LTD_ARATH	Q:829-410,H:36-175	75.71%ID	E:2e-72	Protein LHCP TRANSLOCATION DEFECT	.	0,377	-0,217	0	-1,104	0,087	0,458	0,021	0,611	0,83	0,056	LTD_ARATH	Protein LHCP TRANSLOCATION DEFECT (Protein GRANA-DEFICIENT CHLOROPLAST 1)	LTD GDC1 At1g50900 F8A12.12	Arabidopsis thaliana (Mouse-ear cress)		ANK repeat; Chloroplast; Complete proteome; Membrane; Plastid; Protein transport; Reference proteome; Thylakoid; Transit peptide; Transport
SRR648705_TR34643_c0_g1_i1_len=1142	NA	NA	NA	NA	NA	.	0,149	-0,367	0	-1,515	0,091	-0,488	0,191	-0,375	0,564	-0,582	.	.	.	.	.	.
SRR648705_TR35349_c0_g1_i1_len=1133	LOL1_ARATH	Q:454-831,H:25-153	90.7%ID	E:4e-60	Protein LOL1	PF06943.9^zf-LSD1^LSD1 zinc finger^28-52^E:1.2e-11`PF06943.9^zf-LSD1^LSD1 zinc finger^67-91^E:9.9e-13`PF06943.9^zf-LSD1^LSD1 zinc finger^105-129^E:1e-12	0,09	-0,677	0	-2,197	0,177	-0,731	0,443	-0,424	0,897	-0,098	LOL1_ARATH	Protein LOL1 (Protein LSD ONE LIKE 1) (AtLOL1) (Putative zinc finger LOL1)	LOL1 At1g32540 T9G5.1	Arabidopsis thaliana (Mouse-ear cress)		Alternative splicing; Apoptosis; Complete proteome; Hypersensitive response; Nucleus; Plant defense; Reference proteome
SRR648705_TR37401_c0_g1_i1_len=864	NA	NA	NA	NA	NA	.	0,175	-0,367	0	-1,394	0,406	-0,32	0,836	0,086	0,717	0,093	.	.	.	.	.	.
SRR648705_TR38965_c0_g1_i1_len=1382	YCF23_PYRYE	Q:1011-337,H:11-235	38.94%ID	E:8e-45	Uncharacterized protein ycf23	PF04481.9^DUF561^Protein of unknown function (DUF561)^62-296^E:3.4e-85	0,44	-0,188	0	-1,283	0,472	0,246	0,307	0,337	1	0,005	YCF23_PYRYE	Uncharacterized protein ycf23	ycf23	Pyropia yezoensis (Red alga) (Porphyra yezoensis)		Chloroplast; Plastid
SRR648705_TR42977_c0_g1_i5_len=2488	NA	NA	NA	NA	NA	PF16712.2^SCAB_CC^Coiled-coil regions of plant-specific actin-binding protein^2-136^E:2.6e-56`PF16709.2^SCAB-IgPH^Fused Ig-PH domain of plant-specific actin-binding protein^150-357^E:4.5e-104	0,254	4,468	0	-7,784	0,129	5,038	0,254	4,408	0,367	-0,338	.	.	.	.	.	.
SRR648705_TR43027_c0_g1_i1_len=802	NA	NA	NA	NA	NA	.	1	-0,062	0	-1,885	0,017	-0,895	0,002	-1,282	0,004	-0,951	.	.	.	.	.	.
SRR648705_TR43500_c1_g2_i12_len=4372	EXEC1_ARATH	Q:385-[illegible]	52.86%ID	E:5e-179	Protein EXECUTER 1, chloroplastic {ECO:0000305}	PF12014.5^DUF3506^Domain of unknown function (DUF3506)^538-666^E:8.6e-25	0,56	0,885	0	-6,761	0,004	-1,791	0,004	-1,77	0,195	-1,017	EXEC1_ARATH	Protein EXECUTER 1, chloroplastic (AtEX1)	EX1 At4g33630 T16L1.120	Arabidopsis thaliana (Mouse-ear cress)		Chloroplast; Complete proteome; Plastid; Reference proteome; Stress response; [illegible]

SRR648705_TR43500_c1_g2_i12_len=4372	EXEC1_ARATH	[illegible]-2235,H:64-683	[illegible]	[illegible]	Protein EXECUTER 1, chloroplastic {ECO:0000305}	[illegible]	[illegible]	[illegible]	0	[illegible]	0,004	-1,791	0,004	-1,77	0,195	-1,017	EXEC1_ARATH	Protein EXECUTER 1, chloroplastic (AtEX1)	EX1 At4g33630 [illegible]	Arabidopsis thaliana (Mouse-ear cress)		Transit peptide
SRR648705_TR43863_c0_g2_i2_len=1257	LQY1_ARATH	Q:165-635,H:1-154	73.42%ID	E:8e-62	Protein disulfide-isomerase LQY1, chloroplastic {ECO:0000303|PubMed:21586683}	.	0,746	-0,124	0,001	-1,154	0,235	-0,309	0,858	-0,053	0,102	-0,478	LQY1_ARATH	Protein disulfide-isomerase LQY1, chloroplastic (EC 5.3.4.1) (Protein LOW QUANTUM YIELD OF PHOTOSYSTEM II 1)	LQY1 At1g75690 F10A5.12 F10A5.4	Arabidopsis thaliana (Mouse-ear cress)		Chloroplast; Complete proteome; Isomerase; Membrane; Metal-binding; Plastid; Reference proteome; Repeat; Thylakoid; Transit peptide; Transmembrane; Transmembrane helix; Zinc
SRR648705_TR44504_c0_g1_i6_len=1846	NA	NA	NA	NA	NA	.	0,402	-0,927	0	-3,162	1	0,854	1	-0,504	0,447	-0,205	.	.	.	.	.	.
SRR648705_TR45239_c0_g1_i2_len=2021	SR54C_ARATH	Q:2017-728,H:44-471	86.98%ID	E:0	Signal recognition particle 54 kDa protein, chloroplastic	PF02881.16^SRP54_N^SRP54-type protein, helical bundle domain^41-118^E:8.2e-20`PF06414.9^Zeta_toxin^Zeta toxin^133-241^E:2.9e-06`PF00448.19^SRP54^SRP54-type protein, GTPase domain^136-331^E:1.9e-76`PF01583.17^APS_kinase^Adenylylsulphate kinase^136-177^E:3.9e-05`PF02374.12^ArsA_ATPase^Anion-transporting ATPase^138-173^E:3.9e-05`PF01656.20^CbiA^CobQ/CobB/MinD/ParA nucleotide binding domain^146-290^E:9.3e-07`PF02978.16^SRP_SPB^Signal peptide binding domain^362-434^E:1.3e-17	0,054	-0,662	0	-1,518	0,902	-0,07	0,609	-0,295	0,381	-2,457	SR54C_ARATH	Signal recognition particle 54 kDa protein, chloroplastic (54 chloroplast protein) (54CP) (SRP54) (cpSRP54) (FFC)	FFC CPSRP54 At5g03940 F8F6_150	Arabidopsis thaliana (Mouse-ear cress)		3D-structure; Chloroplast; Complete proteome; GTP-binding; Nucleotide-binding; Plastid; RNA-binding; Reference proteome; Ribonucleoprotein; Signal recognition particle; Transit peptide
SRR648707_TR102_c0_g1_i20_len=1857	NA	NA	NA	NA	NA	PF07207.8^Lir1^Light regulated protein Lir1^1-130^E:8.9e-47	0,977	-0,014	0	-1,788	0,564	0,549	0,84	0,287	0,475	-0,228	.	.	.	.	.	.
SRR648707_TR340_c0_g1_i1_len=1003	RR9_SPIOL	Q:799-233,H:9-197	73.06%ID	E:3e-90	30S ribosomal protein S9, chloroplastic	PF00380.16^Ribosomal_S9^Ribosomal protein S9/S16^87-208^E:1.7e-41	0,351	-0,275	0	-1,247	0,424	-0,302	0,474	-0,261	0,675	-0,284	RR9_SPIOL	30S ribosomal protein S9, chloroplastic (Fragment)	PRPS9	Spinacia oleracea (Spinach)		3D-structure; Acetylation; Chloroplast; Direct protein sequencing; Plastid; RNA-binding; Ribonucleoprotein; Ribosomal protein; Transit peptide; rRNA-binding
SRR648707_TR968_c0_g1_i1_len=668	NA	NA	NA	NA	NA	.	0,025	-0,547	0	-1,04	0,025	0,751	0,014	0,803	1	-0,008	.	.	.	.	.	.
SRR648707_TR2770_c0_g1_i2_len=3613	EBF1_ARATH	Q:1144-2790,H:78-627	56.86%ID	E:0	EIN3-binding F-box protein 1	PF13516.3^LRR_6^Leucine Rich repeat^142-165^E:0.62	1	0	0	-7,347	1	0	1	0	0,316	-0,627	EBF1_ARATH	EIN3-binding F-box protein 1 (F-box/LRR-repeat protein 6)	EBF1 FBL6 At2g25490 F13B15.15	Arabidopsis thaliana (Mouse-ear cress)	PATHWAY: Protein modification; protein ubiquitination.	Complete proteome; Ethylene signaling pathway; Nucleus; Reference proteome; Ubl conjugation pathway
SRR648707_TR6095_c0_g1_i1_len=568	SUT_SPIOL	Q:568-308,H:431-517	72.41%ID	E:4e-33	Sucrose transport protein	.	0	7,028	0	-2,224	0	7,892	1	0	1	0	SUT_SPIOL	Sucrose transport protein (Sucrose permease) (Sucrose-proton symporter)		Spinacia oleracea (Spinach)	PATHWAY: Glycan biosynthesis; sucrose metabolism.	Membrane; Sugar transport; Symport; Transmembrane; Transmembrane helix; Transport
SRR648707_TR9041_c1_g1_i5_len=2945	CUL1_ARATH	Q:2703-517,H:4-732	79.29%ID	E:0	Cullin-1	PF00888.19^Cullin^Cullin family^19-639^E:1.6e-178`PF10557.6^Cullin_Nedd8^Cullin protein neddylation domain^666-728^E:5.3e-26	0,017	5,883	0	-3,399	0,025	-2,756	0,005	-6,212	1	0,572	CUL1_ARATH	Cullin-1	CUL1 At4g02570 T10P11.26	Arabidopsis thaliana (Mouse-ear cress)	PATHWAY: Protein modification; protein ubiquitination.	Auxin signaling pathway; Cell cycle; Complete proteome; Cytoplasm; Cytoskeleton; Developmental protein; Ethylene signaling pathway; Isopeptide bond; Nucleus; Reference proteome; Ubl conjugation; Ubl conjugation pathway
SRR648707_TR10640_c0_g1_i1_len=1296	RK15_ARATH	Q:1090-410,H:27-250	69.16%ID	E:1e-99	50S ribosomal protein L15, chloroplastic	PF00828.16^Ribosomal_L27A^Ribosomal proteins 50S-L15, 50S-L18e, 60S-L27A^110-237^E:5.1e-30	0,081	-0,4	0	-0,899	0,729	-0,082	0,412	0,195	0,536	-0,141	RK15_ARATH	50S ribosomal protein L15, chloroplastic (CL15)	RPL15 At3g25920 MPE11.32 MPE11.9 MPE11_7	Arabidopsis thaliana (Mouse-ear cress)		Acetylation; Chloroplast; Complete proteome; Plastid; Reference proteome; Ribonucleoprotein; Ribosomal protein; Transit peptide
SRR648707_TR12461_c0_g1_i1_len=658	PSRP6_ARATH	Q:548-222,H:1-103	54.55%ID	E:3e-23	50S ribosomal protein 6, chloroplastic	.	0,022	-0,565	0	-1,644	0,233	-0,312	0,503	-0,182	0,035	-0,498	PSRP6_ARATH	50S ribosomal protein 6, chloroplastic (CL25) (Plastid-specific 50S ribosomal protein 6) (PSRP-6)	PSRP6 At5g17870 MPI7_10	Arabidopsis thaliana (Mouse-ear cress)		Chloroplast; Complete proteome; Plastid; Reference proteome; Ribonucleoprotein; Ribosomal protein; Transit peptide
SRR648707_TR16860_c0_g1_i1_len=611	PLPD1_ARATH	Q:1-228,H:489-564	84.21%ID	E:6e-34	Dihydrolipoyl dehydrogenase 1, chloroplastic	.	0,398	-0,388	0	-2,086	0,719	0,388	0,84	-0,178	0,169	-0,546	PLPD1_ARATH	Dihydrolipoyl dehydrogenase 1, chloroplastic (ptLPD1) (EC 1.8.1.4) (Dihydrolipoamide dehydrogenase 1) (Protein LIPOAMIDE DEHYDROGENASE 1) (Pyruvate dehydrogenase complex E3 subunit 1) (E3-1) (PDC-E3 1)	LPD1 At3g16950 K14A17.6	Arabidopsis thaliana (Mouse-ear cress)		Alternative splicing; Chloroplast; Complete proteome; Disulfide bond; FAD; Flavoprotein; NAD; Oxidoreductase; Plastid; Redox-active center; Reference proteome; Transit peptide
SRR648707_TR18401_c0_g3_i1_len=1728	SPX4_ARATH	Q:1511-618,H:1-303	62.06%ID	E:3e-112	SPX domain-containing protein 4	PF03105.16^SPX^SPX domain^1-59^E:3.6e-11`PF03105.16^SPX^SPX domain^71-114^E:9.5e-07`PF03105.16^SPX^SPX domain^131-168^E:7.5e-09	0,568	-0,201	0	-2,306	0,278	-0,659	0,377	-0,547	0,694	-0,193	SPX4_ARATH	SPX domain-containing protein 4 (Protein SPX DOMAIN GENE 4) (AtSPX4)	SPX4 At5g15330 F8M21_220	Arabidopsis thaliana (Mouse-ear cress)		Complete proteome; Reference proteome
SRR648707_TR21831_c0_g1_i1_len=1227	NA	NA	NA	NA	NA	PF16697.2^Yop-YscD_cpl^Inner membrane component of T3SS, cytoplasmic domain^104-185^E:4.4e-10`PF00498.23^FHA^FHA domain^112-179^E:1.2e-13	0,334	-0,258	0	-0,864	0,824	0,055	0,434	0,194	0,788	-0,071	.	.	.	.	.	.
SRR648707_TR25509_c0_g2_i1_len=942	NA	NA	NA	NA	NA	.	0,73	0,12	0	-2,04	0,001	-0,887	0,313	-0,27	0,398	-0,423	.	.	.	.	.	.
SRR648707_TR28008_c1_g1_i2_len=1388	NA	NA	NA	NA	NA	.	0,258	-0,493	0	-1,955	0,221	-0,515	1	-0,006	0,203	-0,512	.	.	.	.	.	.
SRR648707_TR28966_c0_g1_i1_len=928	TCX2_ARATH	Q:833-243,H:479-674	41.04%ID	E:1e-23	Protein tesmin/TSO1-like CXC 2	.	0,111	-1,453	0	-7,077	0,381	-1,335	0,692	-0,646	0,044	-1,167	TCX2_ARATH	Protein tesmin/TSO1-like CXC 2 (AtTCX2) (Protein TSO1-like 2) (Protein SOL2)	TCX2 SOL2 At4g14770 dl3425c FCAALL.306	Arabidopsis thaliana (Mouse-ear cress)		Complete proteome; Developmental protein; Metal-binding; Nucleus; Reference proteome; Zinc
SRR648707_TR34294_c0_g1_i3_len=1245	BH149_ARATH	Q:899-462,H:37-188	50.32%ID	E:3e-34	Transcription factor bHLH149	.	0,851	0,088	0	-1,711	0,664	-0,159	0,008	-1,002	0,378	-0,339	BH149_ARATH	Transcription factor bHLH149 (ATBS1 interacting factor 4) (Basic helix-loop-helix protein 149) (AtbHLH149) (bHLH 149) (Transcription factor EN 144) (bHLH transcription factor bHLH149)	BHLH149 AIF4 EN144 At1g09250 T12M4.4	Arabidopsis thaliana (Mouse-ear cress)		Complete proteome; DNA-binding; Nucleus; Reference proteome; Transcription; Transcription regulation
SRR648707_TR37155_c0_g1_i1_len=2065	Y3913_ARATH	Q:1606-347,H:88-497	50.12%ID	E:9e-127	Uncharacterized protein At3g49140	.	0,617	-0,19	0,001	-1,236	0,059	-0,717	0,108	-0,618	1	-0,009	Y3913_ARATH	Uncharacterized protein At3g49140	At3g49140 F2K15.2 T2J13.20	Arabidopsis thaliana (Mouse-ear cress)		Complete proteome; Phosphoprotein; Reference proteome
SRR648707_TR37562_c0_g1_i1_len=1266	PABN2_ARATH	Q:1044-421,H:13-219	84.13%ID	E:1e-85	Polyadenylate-binding protein 2 {ECO:0000303|PubMed:18479511}	PF00076.19^RRM_1^RNA recognition motif. (a.k.a. RRM, RBD, or RNP domain)^95-163^E:2.5e-17`PF13893.3^RRM_5^RNA recognition motif. (a.k.a. RRM, RBD, or RNP domain)^108-166^E:0.03	0,957	0,041	0	-3,299	0,608	0,21	0	1,175	0,129	4,873	PABN2_ARATH	Polyadenylate-binding protein 2 (AtPabN2) (AtPabN3) (Poly(A)-binding protein 2) (Nuclear poly(A)-binding protein 2) (Poly(A)-binding protein II) (PABII)	PABN2 At5g65260 MQN23.20	Arabidopsis thaliana (Mouse-ear cress)		Coiled coil; Complete proteome; Cytoplasm; Nucleus; RNA-binding; Reference proteome
SRR924147_TR403_c0_g1_i1_len=815	NA	NA	NA	NA	NA	PF05768.11^DUF836^Glutaredoxin-like domain (DUF836)^59-145^E:2.3e-20	0,164	-0,538	0	-2,054	0,811	0,306	1	-0,017	0,708	0,161	.	.	.	.	.	.
SRR924147_TR2926_c0_g3_i2_len=1005	RS12_HORVU	Q:199-558,H:24-143	82.5%ID	E:4e-57	40S ribosomal protein S12	PF01248.23^Ribosomal_L7Ae^Ribosomal protein L7Ae/L30e/S12e/Gadd45 family^25-118^E:1.8e-26	1	3,289	0	-8,403	0,312	0,765	1	0,083	0,057	1,361	RS12_HORVU	40S ribosomal protein S12	RPS12	Hordeum vulgare (Barley)		Ribonucleoprotein; Ribosomal protein
SRR924147_TR5604_c0_g1_i1_len=2693	PP222_ARATH	Q:554-2548,H:81-775	67.43%ID	E:0	Pentatricopeptide repeat-containing protein At3g09650, chloroplastic	PF13812.3^PPR_3^Pentatricopeptide repeat domain^224-279^E:4.6e-12`PF13041.3^PPR_2^PPR repeat family^232-280^E:6e-13`PF01535.17^PPR^PPR repeat^236-265^E:0.0005`PF01535.17^PPR^PPR repeat^271-299^E:1.2`PF12854.4^PPR_1^PPR repeat^382-411^E:2.3e-05`PF01535.17^PPR^PPR repeat^387-411^E:0.033`PF17177.1^PPR_long^Pentacotripeptide-repeat region of PROPR^403-504^E:6.6e-07`PF12854.4^PPR_1^PPR repeat^420-450^E:9.6e-07`PF13041.3^PPR_2^PPR repeat family^421-470^E:6.7e-14`PF13812.3^PPR_3^Pentatricopeptide repeat domain^421-468^E:1.6e-07`PF01535.17^PPR^PPR repeat^424-454^E:5.6e-06`PF13041.3^PPR_2^PPR repeat family^457-504^E:3.3e-12`PF01535.17^PPR^PPR repeat^459-486^E:0.00096`PF13812.3^PPR_3^Pentatricopeptide repeat domain^483-539^E:3.2e-09`PF13041.3^PPR_2^PPR repeat family^492-540^E:4.7e-12`PF01535.17^PPR^PPR repeat^495-525^E:0.00016`PF13812.3^PPR_3^Pentatricopeptide repeat domain^517-557^E:6.8e-05`PF01535.17^PPR^PPR repeat^530-557^E:0.00012`PF13812.3^PPR_3^Pentatricopeptide repeat domain^551-606^E:1e-06`PF12854.4^PPR_1^PPR repeat^561-592^E:3.2e-08`PF13041.3^PPR_2^PPR repeat family^564-610^E:3.3e-12`PF01535.17^PPR^PPR repeat^566-596^E:3.1e-08`PF01535.17^PPR^PPR repeat^601-629^E:1.1	1	-2,284	0	-3,973	1	0	1	0	1	-0,006	PP222_ARATH	Pentatricopeptide repeat-containing protein At3g09650, chloroplastic (Protein HIGH CHLOROPHYLL FLUORESCENCE 152)	HCF152 CRM3 At3g09650 F11F8_24	Arabidopsis thaliana (Mouse-ear cress)		Chloroplast; Complete proteome; Nucleotide-binding; Plastid; RNA-binding; Reference proteome; Repeat; Transit peptide
SRR924147_TR6523_c0_g1_i1_len=887	NA	NA	NA	NA	NA	.	0,017	-5,844	0,001	-6,62	1	0	1	0	0,471	-0,493	.	.	.	.	.	.
SRR924147_TR7995_c0_g1_i1_len=1581	UREG_ARATH	Q:369-1112,H:28-275	92.74%ID	E:7e-168	Urease accessory protein G	PF02492.16^cobW^CobW/HypB/UreG, nucleotide-binding domain^86-254^E:4.2e-32	0,458	-0,272	0	-1,349	0,517	-0,277	0,142	-0,564	0,752	0,091	UREG_ARATH	Urease accessory protein G (AtUREG)	UREG At2g34470	Arabidopsis thaliana (Mouse-ear cress)		Alternative splicing; Chaperone; Complete proteome; GTP-binding; Nickel insertion; Nucleotide-binding; Reference proteome
SRR924147_TR8351_c0_g1_i1_len=1715	NA	NA	NA	NA	NA	PF04862.9^DUF642^Protein of unknown function (DUF642)^31-188^E:2e-64`PF04862.9^DUF642^Protein of unknown function (DUF642)^201-366^E:9.7e-19	0,041	-0,611	0	-2,27	0,739	-0,248	0,489	-0,487	0,052	-0,63	.	.	.	.	.	.
SRR924147_TR16824_c0_g1_i1_len=1457	RSI1_SOLLC	Q:843-607,H:6-96	65.93%ID	E:3e-31	Protein RSI-1	.	0,129	-1,005	0	-3,304	1	-0,802	1	-1,266	1	-0,806	RSI1_SOLLC	Protein RSI-1 (TR132)	RSI-1	Solanum lycopersicum (Tomato) (Lycopersicon esculentum)		Complete proteome; Disulfide bond; Reference proteome; Secreted; Signal
SRR924147_TR19455_c2_g1_i1_len=2387	CHUP1_ARATH	Q:1113-325,H:720-984	59.71%ID	E:2e-97	Protein CHUP1, chloroplastic	.	0,318	-0,467	0	-3,992	0,076	-2,345	0,076	-2,292	1	-0,166	CHUP1_ARATH	Protein CHUP1, chloroplastic (Protein CHLOROPLAST UNUSUAL POSITIONING 1)	CHUP1 At3g25690 T5M7.14	Arabidopsis thaliana (Mouse-ear cress)		Alternative splicing; Chloroplast; Coiled coil; Complete proteome; Membrane; Phosphoprotein; Plastid; Plastid outer membrane; Reference proteome; Repeat
SRR924147_TR22073_c0_g2_i1_len=1027	NA	NA	NA	NA	NA	PF17067.2^RPS31^Ribosomal protein S31e^1-94^E:1.1e-31	0,511	-0,192	0	-1,826	0,007	-0,801	0,001	-0,957	0,56	-0,709	.	.	.	.	.	.
SRR924147_TR22093_c2_g5_i1_len=8305	TI214_COFAR	Q:5459-7819,H:1067-1873	70.96%ID	E:0	Protein TIC 214 {ECO:0000250|UniProtKB:P56785}	.	1	-0,059	0	-3,294	0,381	2,002	0,381	2,104	0,149	-0,381	TI214_COFAR	Protein TIC 214 (Translocon at the inner envelope membrane of chloroplasts 214) (AtTIC214)	TIC214 ycf1	Coffea arabica (Arabian coffee)		Chloroplast; Membrane; Plastid; Plastid inner membrane; Protein transport; Transmembrane; Transmembrane helix; Transport
SRR924147_TR22473_c0_g2_i2_len=853	NA	NA	NA	NA	NA	.	0,228	-1,134	0	-2,954	0,235	-0,949	0,581	-0,383	1	0,028	.	.	.	.	.	.
SRR924147_TR25917_c0_g1_i1_len=741	PATL6_ARATH	Q:66-461,H:273-407	74.07%ID	E:1e-63	Patellin-6	.	0,635	-0,318	0	-3,111	0,282	0,979	1	-0,135	0,09	-0,685	PATL6_ARATH	Patellin-6	PATL6 At3g51670 T18N14.50	Arabidopsis thaliana (Mouse-ear cress)		Cell cycle; Cell division; Coiled coil; Complete proteome; Cytoplasm; Lipid-binding; Membrane; Reference proteome; Transport
SRR924147_TR25981_c0_g1_i1_len=950	NA	NA	NA	NA	NA	.	0,44	-0,884	0	-3,584	1	-0,403	0,472	0,691	1	-0,299	.	.	.	.	.	.
SRR924147_TR27509_c0_g1_i1_len=723	SYFM_ARATH	Q:620-309,H:325-429	80.95%ID	E:1e-52	Phenylalanine–tRNA ligase, chloroplastic/mitochondrial {ECO:0000305}	.	0,798	0,523	0	-2,817	0,591	0,355	0,115	0,8	0,016	-1,264	SYFM_ARATH	Phenylalanine–tRNA ligase, chloroplastic/mitochondrial (EC 6.1.1.20) (Phenylalanyl-tRNA synthetase) (PheRS)	At3g58140 F9D24.50	Arabidopsis thaliana (Mouse-ear cress)		ATP-binding; Acetylation; Aminoacyl-tRNA synthetase; Chloroplast; Complete proteome; Ligase; Mitochondrion; Nucleotide-binding; Plastid; Protein biosynthesis; Reference proteome; Transit peptide
SRR924147_TR31027_c0_g1_i1_len=1067	21KD_DAUCA	Q:695-240,H:28-187	45.62%ID	E:1e-41	21 kDa protein	PF04043.12^PMEI^Plant invertase/pectin methylesterase inhibitor^36-183^E:4.3e-37	0,009	-0,747	0	-2,708	0,059	-1,81	0,111	-1,424	0,594	0,252	21KD_DAUCA	21 kDa protein (1.2 protein)		Daucus carota (Wild carrot)		Signal
SRR924147_TR34664_c0_g2_i1_len=1368	RK9_ARATH	Q:914-378,H:16-197	73.63%ID	E:6e-58	50S ribosomal protein L9, chloroplastic	PF01281.16^Ribosomal_L9_N^Ribosomal protein L9, N-terminal domain^50-95^E:3e-16`PF03948.11^Ribosomal_L9_C^Ribosomal protein L9, C-terminal domain^111-193^E:1.3e-21	0,051	-0,495	0	-1,103	0,843	-0,077	0,812	0,083	0,076	-0,44	RK9_ARATH	50S ribosomal protein L9, chloroplastic (CL9)	RPL9 At3g44890 F28D10_80	Arabidopsis thaliana (Mouse-ear cress)		Chloroplast; Complete proteome; Plastid; RNA-binding; Reference proteome; Ribonucleoprotein; Ribosomal protein; Transit peptide; rRNA-binding
SRR924147_TR38112_c0_g1_i2_len=1089	UBC9_ARATH	Q:868-425,H:1-148	95.95%ID	E:7e-100	SUMO-conjugating enzyme UBC9	PF00179.23^UQ_con^Ubiquitin-conjugating enzyme^5-141^E:1e-53	1	0	0	-6,992	1	0	1	0	0,129	-1	UBC9_ARATH	SUMO-conjugating enzyme UBC9 (EC 6.3.2.-) (UBCAT4B) (Ubiquitin carrier protein 9) (Ubiquitin-conjugating enzyme E2 9) (Ubiquitin-conjugating enzyme E2-17 kDa) (Ubiquitin-protein ligase 9)	UBC9 UBC4B At4g27960 T13J8.70	Arabidopsis thaliana (Mouse-ear cress)	PATHWAY: Protein modification; protein sumoylation.	ATP-binding; Alternative splicing; Complete proteome; Ligase; Nucleotide-binding; Reference proteome; Ubl conjugation pathway
SRR924148_TR6830_c0_g2_i1_len=1281	AB9B_ARATH	Q:91-1248,H:847-1233	59.95%ID	E:8e-149	ABC transporter B family member 9	PF00664.20^ABC_membrane^ABC transporter transmembrane region^6-97^E:1.6e-18`PF00005.24^ABC_tran^ABC transporter^165-313^E:1e-33`PF05783.8^DLIC^Dynein light intermediate chain (DLIC)^172-204^E:0.00072`PF00004.26^AAA^ATPase family associated with various cellular activities (AAA)^178-373^E:0.00032	0,243	-0,586	0	-1,736	0,719	0,418	0,719	0,4	0,35	-0,451	AB9B_ARATH	ABC transporter B family member 9 (ABC transporter ABCB.9) (AtABCB9) (Multidrug resistance protein 9) (P-glycoprotein 9)	ABCB9 MDR9 PGP9 At4g18050 F15J5.20	Arabidopsis thaliana (Mouse-ear cress)		ATP-binding; Complete proteome; Glycoprotein; Membrane; Nucleotide-binding; Reference proteome; Repeat; Transmembrane; Transmembrane helix; Transport
SRR924148_TR7299_c0_g3_i6_len=1191	NA	NA	NA	NA	NA	.	0,86	-0,251	0	-3,225	0,18	-0,919	0,25	-0,802	0,76	0,553	.	.	.	.	.	.
SRR924148_TR12427_c0_g3_i7_len=2086	TSJT1_TOBAC	Q:894-448,H:1-149	64%ID	E:2e-57	Stem-specific protein TSJT1	PF12481.5^DUF3700^Aluminium induced protein^3-228^E:7.1e-84`PF13537.3^GATase_7^Glutamine amidotransferase domain^77-176^E:2.1e-09	0,335	-0,695	0	-2,818	1	0,228	1	-0,213	1	-0,001	TSJT1_TOBAC	Stem-specific protein TSJT1	TSJT1	Nicotiana tabacum (Common tobacco)		
SRR924148_TR16923_c1_g1_i2_len=1314	REMO_SOLTU	Q:766-266,H:29-198	79.53%ID	E:1e-66	Remorin	PF03766.10^Remorin_N^Remorin, N-terminal region^33-87^E:2.1e-21`PF03763.10^Remorin_C^Remorin, C-terminal region^92-196^E:1.4e-31	0,297	-0,259	0	-1,098	0,044	-0,572	0,396	0,224	1	-0,262	REMO_SOLTU	Remorin (pp34)		Solanum tuberosum (Potato)		Cell membrane; Coiled coil; Complete proteome; Direct protein sequencing; Membrane; Phosphoprotein; Reference proteome; Transport
SRR924148_TR19021_c0_g1_i1_len=964	PHI_MYCGS	Q:186-794,H:5-199	35.92%ID	E:7e-24	3-hexulose-6-phosphate isomerase	.	0,272	-0,381	0	-1,17	0,079	-0,913	0,232	-0,567	0,385	0,29	PHI_MYCGS	3-hexulose-6-phosphate isomerase (EC 5.3.1.27) (6-phospho-3-hexuloisomerase) (PHI)	rmpB	Mycobacterium gastri	PATHWAY: One-carbon metabolism; formaldehyde assimilation via RuMP pathway; D-fructose 6-phosphate from D-ribulose 5-phosphate and formaldehyde: step 2/2.	Carbohydrate metabolism; Isomerase
SRR924148_TR20412_c0_g1_i13_len=2364	AFC2_ARATH	Q:1071-556,H:253-425	84.97%ID	E:4e-98	Serine/threonine-protein kinase AFC2	.	0,01	-2,027	0	-7,752	0,545	0,568	0,43	0,706	1	-1,753	AFC2_ARATH	Serine/threonine-protein kinase AFC2 (EC 2.7.12.1)	AFC2 At4g24740 F22K18.60	Arabidopsis thaliana (Mouse-ear cress)		ATP-binding; Alternative splicing; Complete proteome; Kinase; Nucleotide-binding; Reference proteome; Serine/threonine-protein kinase; Transferase
SRR924148_TR23813_c0_g1_i1_len=655	PB27A_ARATH	Q:183-569,H:43-174	69.92%ID	E:4e-58	Photosystem II repair protein PSB27-H1, chloroplastic	PF13326.3^PSII_Pbs27^Photosystem II Pbs27^59-185^E:1e-30	0,798	-0,369	0,001	-2,634	0,335	-0,769	0,581	-0,473	0,768	0,067	PB27A_ARATH	Photosystem II repair protein PSB27-H1, chloroplastic (Psb27-H1) (Thylakoid lumenal protein PSB27-H1)	PSB27-1 At1g03600 F21B7.14 F21B7.21 F21B7.22 F21B7_140	Arabidopsis thaliana (Mouse-ear cress)		Chloroplast; Complete proteome; Direct protein sequencing; Membrane; Phosphoprotein; Plastid; Reference proteome; Thylakoid; Transit peptide
SRR924148_TR24436_c0_g2_i1_len=753	ACBP_RICCO	Q:569-315,H:1-85	88.24%ID	E:2e-46	Acyl-CoA-binding protein	.	0,009	5,955	0,001	-6,655	1	0	1	0	1	0	ACBP_RICCO	Acyl-CoA-binding protein (ACBP)		Ricinus communis (Castor bean)		Lipid-binding; Transport
SRR924148_TR26347_c0_g1_i4_len=1742	GEDH1_OCIBA	Q:1423-707,H:1-242	56.61%ID	E:4e-83	Geraniol dehydrogenase 1	PF08240.9^ADH_N^Alcohol dehydrogenase GroES-like domain^37-146^E:1.6e-23`PF02826.16^2-Hacid_dh_C^D-isomer specific 2-hydroxyacid dehydrogenase, NAD binding domain^177-227^E:1.5e-06	0,138	0,654	0	-2,802	0,026	-1,086	0,018	-1,159	0,823	0,085	GEDH1_OCIBA	Geraniol dehydrogenase 1 (ObaGEDH1) (EC 1.1.1.183)	GEDH1	Ocimum basilicum (Sweet basil)		Metal-binding; NADP; Oxidoreductase; Zinc
SRR924148_TR28419_c0_g3_i2_len=1336	PIP1_SOLLC	Q:203-1048,H:1-282	86.52%ID	E:3e-179	Probable aquaporin PIP-type pTOM75	PF00230.17^MIP^Major intrinsic protein^45-274^E:3.2e-84	0,907	-0,045	0	-1,305	0	1,49	0,001	0,818	0,444	0,317	PIP1_SOLLC	Probable aquaporin PIP-type pTOM75 (Ripening-associated membrane protein) (RAMP)		Solanum lycopersicum (Tomato) (Lycopersicon esculentum)		Cell membrane; Complete proteome; Membrane; Reference proteome; Repeat; Stress response; Transmembrane; Transmembrane helix; Transport
SRR924148_TR29305_c0_g1_i1_len=2045	NA	NA	NA	NA	NA	.	0,009	-1,127	0	-4,017	0,226	-2,078	0,461	-1,585	0,629	-1,245	.	.	.	.	.	.
SRR924148_TR31545_c0_g1_i2_len=1650	TRPD_ARATH	Q:1632-1231,H:304-437	77.61%ID	E:1e-55	Anthranilate phosphoribosyltransferase, chloroplastic	PF00591.18^Glycos_transf_3^Glycosyl transferase family, a/b domain^1-121^E:4.4e-30	0,076	-3,088	0	-4,301	0,005	-5,047	0,005	-5,575	1	0,006	TRPD_ARATH	Anthranilate phosphoribosyltransferase, chloroplastic (EC 2.4.2.18)	PAT1 At5g17990 MCM23.6	Arabidopsis thaliana (Mouse-ear cress)	PATHWAY: Amino-acid biosynthesis; L-tryptophan biosynthesis; L-tryptophan from chorismate: step 2/5.	Amino-acid biosynthesis; Aromatic amino acid biosynthesis; Chloroplast; Complete proteome; Glycosyltransferase; Plastid; Reference proteome; Transferase; Transit peptide; Tryptophan biosynthesis
SRR924148_TR32136_c0_g1_i1_len=1524	PER42_ARATH	Q:1365-376,H:1-330	79.7%ID	E:0	Peroxidase 42	PF00141.20^peroxidase^Peroxidase^48-291^E:4.1e-60	0,219	-0,273	0	-2,176	0,492	-0,189	0,007	-0,786	0	0,948	PER42_ARATH	Peroxidase 42 (Atperox P42) (EC 1.11.1.7) (ATP1a/ATP1b) (PRXR1)	PER42 P42 At4g21960 F1N20.3 T8O5.170	Arabidopsis thaliana (Mouse-ear cress)		Biological rhythms; Calcium; Complete proteome; Disulfide bond; Glycoprotein; Heme; Hydrogen peroxide; Iron; Metal-binding; Oxidoreductase; Peroxidase; Reference proteome; Secreted; Signal
SRR924148_TR34588_c0_g1_i1_len=966	NA	NA	NA	NA	NA	PF05498.8^RALF^Rapid ALkalinization Factor (RALF)^72-131^E:7.4e-09	1	-0,024	0	-1,954	0,142	0,747	0,114	0,817	0,036	0,505	.	.	.	.	.	.
SRR924148_TR34958_c0_g1_i4_len=1690	PUMP2_ARATH	Q:1062-586,H:146-304	75.47%ID	E:6e-109	Mitochondrial uncoupling protein 2	PF00153.24^Mito_carr^Mitochondrial carrier protein^7-105^E:3e-20	0,707	0,416	0,001	-2,641	0,158	-1,581	0,44	-0,869	0,357	0,687	PUMP2_ARATH	Mitochondrial uncoupling protein 2 (AtPUMP2)	PUMP2 UCP2 At5g58970 K19M22.16	Arabidopsis thaliana (Mouse-ear cress)		Alternative splicing; Complete proteome; Membrane; Mitochondrion; Mitochondrion inner membrane; Reference proteome; Repeat; Stress response; Transmembrane; Transmembrane helix; Transport
SRR924148_TR35654_c0_g2_i1_len=1485	NA	NA	NA	NA	NA	PF00786.25^PBD^P21-Rho-binding domain^112-143^E:5.4e-09	1	-0,053	0	-4,599	0,038	-1,613	0,017	-1,965	0,684	0,189	.	.	.	.	.	.
SRR924148_TR38039_c1_g2_i1_len=1769	P2SAF_ARATH	Q:369-1346,H:78-403	87.42%ID	E:0	Photosystem II stability/assembly factor HCF136, chloroplastic	PF14870.3^PSII_BNR^Photosynthesis system II assembly factor YCF48^80-401^E:1.6e-122	0,014	-0,721	0	-1,124	0,001	-0,974	0,178	-0,395	0,131	-0,399	P2SAF_ARATH	Photosystem II stability/assembly factor HCF136, chloroplastic	HCF136 At5g23120 MYJ24.11	Arabidopsis thaliana (Mouse-ear cress)		Chloroplast; Complete proteome; Direct protein sequencing; Membrane; Photosynthesis; Photosystem II; Plastid; Reference proteome; Repeat; Thylakoid; Transit peptide
SRR924148_TR39368_c0_g1_i1_len=1406	RAC7_ARATH	Q:1058-426,H:1-208	90.09%ID	E:4e-132	Rac-like GTP-binding protein ARAC7	PF00071.19^Ras^Ras family^8-178^E:2.8e-52`PF08477.10^Roc^Ras of Complex, Roc, domain of DAPkinase^8-121^E:2.9e-23	0,04	-0,703	0	-2,757	0,212	-0,555	0,169	-0,619	0,806	-0,086	RAC7_ARATH	Rac-like GTP-binding protein ARAC7 (GTPase protein ROP9)	ARAC7 ROP9 At4g28950 F25O24.70	Arabidopsis thaliana (Mouse-ear cress)		3D-structure; Abscisic acid signaling pathway; Complete proteome; GTP-binding; Lipoprotein; Membrane; Nucleotide-binding; Palmitate; Reference proteome

MsDamaged24h

Transcript (CDF97)	Uniprot homolog	BLASTX Position	BLASTX %Identity	BLASTX evalue	Uniprot descr	Pfam	PValue_Ms6h	logFC_Ms6h	PValue_Ms8h	logFC_Ms8h	PValue_MsDamaged24h	logFC_MsDamaged24h	PValue_MsIntact24h	logFC_MsIntact24h	PValue_Ms1wk	logFC_Ms1wk	Entry.name	Protein names	Gene names	Organism	Pathway	Keywords
SRR1144633_TR659_c0_g1_i1_len=807	PLY22_ARATH	Q:616-2,H:50-253	83.9%ID	E:3e-125	Probable pectate lyase 22	.	1	0,024	1	0,143	0	-1,773	0,002	-0,911	0	7,306	PLY22_ARATH	Probable pectate lyase 22 (EC 4.2.2.2)	At5g63180 MDC12_15	Arabidopsis thaliana (Mouse-ear cress)	PATHWAY: Glycan metabolism; pectin degradation; 2-dehydro-3-deoxy-D-gluconate from pectin: step 2/5.	Calcium; Complete proteome; Glycoprotein; Lyase; Metal-binding; Reference proteome; Signal
SRR1144633_TR5703_c0_g1_i1_len=1092	UCRIA_SOLTU	Q:950-258,H:1-230	80.17%ID	E:1e-113	Cytochrome b6-f complex iron-sulfur subunit, chloroplastic	PF08802.7^CytB6-F_Fe-S^Cytochrome B6-F complex Fe-S subunit^58-96^E:8.8e-18`PF00355.23^Rieske^Rieske [2Fe-2S] domain^138-198^E:3e-12	0,406	-0,186	0,979	0,007	0	-0,925	0,005	-0,596	0,941	-0,017	UCRIA_SOLTU	Cytochrome b6-f complex iron-sulfur subunit, chloroplastic (EC 1.10.9.1) (Plastohydroquinone:plastocyanin oxidoreductase iron-sulfur protein) (Rieske iron-sulfur protein) (ISP) (RISP)	petC	Solanum tuberosum (Potato)		2Fe-2S; Chloroplast; Complete proteome; Disulfide bond; Electron transport; Iron; Iron-sulfur; Membrane; Metal-binding; Oxidoreductase; Plastid; Reference proteome; Thylakoid; Transit peptide; Transmembrane; Transmembrane helix; Transport
SRR1144633_TR9969_c0_g1_i4_len=772	IDI2_CAMAC	Q:297-52,H:75-162	76.14%ID	E:4e-39	Isopentenyl-diphosphate Delta-isomerase II	.	0,497	-0,162	0,005	0,743	0	-0,975	0,18	-0,343	1	-0,725	IDI2_CAMAC	Isopentenyl-diphosphate Delta-isomerase II (EC 5.3.3.2) (Isopentenyl pyrophosphate isomerase II) (IPP isomerase II)	IPI2	Camptotheca acuminata (Happy tree)	PATHWAY: Isoprenoid biosynthesis; dimethylallyl diphosphate biosynthesis; dimethylallyl diphosphate from isopentenyl diphosphate: step 1/1.; PATHWAY: Porphyrin-containing compound metabolism; chlorophyll biosynthesis.	Chlorophyll biosynthesis; Isomerase; Isoprene biosynthesis; Magnesium; Metal-binding; Photosynthesis
SRR1144633_TR27993_c0_g1_i1_len=1673	DTC_ARATH	Q:1460-585,H:7-298	86.99%ID	E:3e-177	Mitochondrial dicarboxylate/tricarboxylate transporter DTC	PF00153.24^Mito_carr^Mitochondrial carrier protein^14-90^E:8.4e-15`PF00153.24^Mito_carr^Mitochondrial carrier protein^102-197^E:5.4e-19`PF00153.24^Mito_carr^Mitochondrial carrier protein^209-292^E:3.3e-13	0,558	-0,171	0,858	-0,064	0,001	-0,78	0,041	-0,465	0,271	0,356	DTC_ARATH	Mitochondrial dicarboxylate/tricarboxylate transporter DTC (Dicarboxylate/tricarboxylate carrier)	DTC At5g19760 T29J13.180	Arabidopsis thaliana (Mouse-ear cress)		Complete proteome; Membrane; Mitochondrion; Mitochondrion inner membrane; Reference proteome; Repeat; Transmembrane; Transmembrane helix; Transport
SRR1144633_TR29660_c0_g1_i3_len=1731	NA	NA	NA	NA	NA	.	0	1,512	0	1,371	0	-0,782	0,011	-0,53	0,484	-0,42	.	.	.	.	.	.
SRR1144634_TR1364_c0_g1_i1_len=1646	PUMP5_ARATH	Q:1167-193,H:1-311	76.31%ID	E:8e-153	Mitochondrial uncoupling protein 5	PF00153.24^Mito_carr^Mitochondrial carrier protein^6-123^E:1.5e-16`PF00153.24^Mito_carr^Mitochondrial carrier protein^131-225^E:3.4e-19`PF00153.24^Mito_carr^Mitochondrial carrier protein^233-323^E:5.2e-19	0,534	0,484	0	2,269	0,001	-0,968	0,014	-0,706	1	0,11	PUMP5_ARATH	Mitochondrial uncoupling protein 5 (AtPUMP5) (Mitochondrial dicarboxylate carrier 1)	PUMP5 DIC1 UCP5 At2g22500 F14M13.10	Arabidopsis thaliana (Mouse-ear cress)		Complete proteome; Membrane; Mitochondrion; Mitochondrion inner membrane; Reference proteome; Repeat; Stress response; Transmembrane; Transmembrane helix; Transport
SRR1144634_TR7185_c0_g2_i1_len=724	PSAH1_ARATH	Q:167-535,H:1-123	81.3%ID	E:5e-51	Photosystem I reaction center subunit VI-1, chloroplastic	PF03244.11^PSI_PsaH^Photosystem I reaction centre subunit VI^7-145^E:1.9e-71	0,957	0,015	0,002	-0,736	0	-0,898	0,003	-0,686	0,013	0,523	PSAH1_ARATH	Photosystem I reaction center subunit VI-1, chloroplastic (PSI-H1)	PSAH1 At3g16140 MSL1.18	Arabidopsis thaliana (Mouse-ear cress)		Chloroplast; Complete proteome; Membrane; Photosynthesis; Photosystem I; Plastid; Reference proteome; Thylakoid; Transit peptide; Transmembrane; Transmembrane helix
SRR1144634_TR9424_c0_g1_i1_len=1653	NA	NA	NA	NA	NA	PF04862.9^DUF642^Protein of unknown function (DUF642)^26-182^E:1.1e-68`PF04862.9^DUF642^Protein of unknown function (DUF642)^193-359^E:4.1e-18	0,77	0,083	0,116	-0,485	0,001	-1,231	0,061	-0,653	1	0,156	.	.	.	.	.	.
SRR122236_TR21231_c0_g2_i1_len=634	PSAG_ARATH	Q:526-104,H:27-160	72.34%ID	E:9e-68	Photosystem I reaction center subunit V, chloroplastic	PF01241.15^PSI_PSAK^Photosystem I psaG / psaK^53-144^E:2.5e-26	0,62	-0,23	0,014	-1,126	0	-1,128	0,001	-0,978	0,76	-0,675	PSAG_ARATH	Photosystem I reaction center subunit V, chloroplastic (PSI-G)	PSAG At1g55670 F20N2.21 F20N2.33 F20N2_3	Arabidopsis thaliana (Mouse-ear cress)		Chloroplast; Complete proteome; Membrane; Photosynthesis; Photosystem I; Plastid; Reference proteome; Thylakoid; Transit peptide; Transmembrane; Transmembrane helix
SRR122237_TR20733_c0_g1_i1_len=1351	PRP4_ARATH	Q:144-509,H:32-147	54.47%ID	E:6e-31	Proline-rich protein 4	PF01190.14^Pollen_Ole_e_I^Pollen proteins Ole e I like^50-132^E:1.6e-11	0,429	0,214	0,094	0,442	0	-1,157	0,003	-0,799	0,093	0,39	PRP4_ARATH	Proline-rich protein 4 (AtPRP4)	PRP4 At4g38770 T9A14.50	Arabidopsis thaliana (Mouse-ear cress)		Cell wall; Complete proteome; Reference proteome; Repeat; Secreted; Signal
SRR122238_TR10529_c0_g2_i2_len=1753	Y1669_ARATH	Q:1269-160,H:2-377	75%ID	E:0	Uncharacterized oxidoreductase At1g06690, chloroplastic	PF00248.18^Aldo_ket_red^Aldo/keto reductase family^52-354^E:5e-63	0,294	0,509	0,174	0,561	0	-1,419	0,001	-0,995	0,173	-0,646	Y1669_ARATH	Uncharacterized oxidoreductase At1g06690, chloroplastic (EC 1.-.-.-)	At1g06690 F12K11.2 F4H5.17	Arabidopsis thaliana (Mouse-ear cress)		Acetylation; Chloroplast; Complete proteome; NADP; Oxidoreductase; Plastid; Reference proteome; Transit peptide
SRR122238_TR43302_c0_g1_i1_len=1269	CSPL6_SOYBN	Q:987-478,H:5-173	59.06%ID	E:3e-36	CASP-like protein 1E1	PF04535.9^DUF588^Domain of unknown function (DUF588)^28-183^E:7.2e-41	0,196	-0,463	0,548	-0,234	0,001	-1,666	0,129	-0,672	0,732	-0,58	CSPL6_SOYBN	CASP-like protein 1E1 (GmCASPL1E1)		Glycine max (Soybean) (Glycine hispida)		Cell membrane; Complete proteome; Membrane; Reference proteome; Transmembrane; Transmembrane helix
SRR122238_TR59797_c1_g1_i2_len=1426	GDL57_ARATH	Q:174-1214,H:34-378	58.19%ID	E:2e-150	GDSL esterase/lipase At3g48460	PF00657.19^Lipase_GDSL^GDSL-like Lipase/Acylhydrolase^38-361^E:2.5e-12	0,461	-0,24	0,699	-0,166	0	-1,38	0,021	-0,805	0,435	0,261	GDL57_ARATH	GDSL esterase/lipase At3g48460 (EC 3.1.1.-) (Extracellular lipase At3g48460)	At3g48460 T29H11.20	Arabidopsis thaliana (Mouse-ear cress)		Complete proteome; Glycoprotein; Hydrolase; Lipid degradation; Lipid metabolism; Reference proteome; Secreted; Signal
SRR122238_TR65776_c0_g1_i1_len=664	NA	NA	NA	NA	NA	PF08041.8^PetM^PetM family of cytochrome b6f complex subunit 7^118-145^E:4.1e-11	0,165	-0,315	0,506	0,161	0	-0,923	0,06	-0,408	0	1,194	.	.	.	.	.	.
SRR122238_TR83919_c0_g1_i1_len=1636	STR4_ARATH	Q:1504-374,H:1-367	61.74%ID	E:2e-140	Rhodanese-like domain-containing protein 4, chloroplastic	.	0,24	-0,346	0,006	-0,896	0	-0,919	0,005	-0,724	0,794	0,084	STR4_ARATH	Rhodanese-like domain-containing protein 4, chloroplastic (Protein THYLAKOID RHODANESE-LIKE) (Sulfurtransferase 4) (AtStr4)	STR4 TROL At4g01050 F2N1.31	Arabidopsis thaliana (Mouse-ear cress)		3D-structure; Chloroplast; Complete proteome; Membrane; Plastid; Reference proteome; Thylakoid; Transit peptide; Transmembrane; Transmembrane helix
SRR122238_TR84226_c0_g1_i11_len=2910	NA	NA	NA	NA	NA	PF00875.15^DNA_photolyase^DNA photolyase^64-212^E:3.7e-26	1	0,216	0,692	-0,911	0	-1,477	0,011	-0,96	1	0,096	.	.	.	.	.	.
SRR1271857_TR2101_c0_g2_i1_len=2090	FAD3C_SESIN	Q:1840-620,H:1-404	80.93%ID	E:0	Omega-3 fatty acid desaturase, chloroplastic	PF11960.5^DUF3474^Domain of unknown function (DUF3474)^1-140^E:1.4e-55`PF00487.21^FA_desaturase^Fatty acid desaturase^148-402^E:7e-33	0,803	-0,17	0,195	0,982	0	-1,59	0,008	-0,933	1	0,669	FAD3C_SESIN	Omega-3 fatty acid desaturase, chloroplastic (EC 1.14.19.-)	FAD7	Sesamum indicum (Oriental sesame) (Sesamum orientale)	PATHWAY: Lipid metabolism; polyunsaturated fatty acid biosynthesis.	Chloroplast; Fatty acid biosynthesis; Fatty acid metabolism; Lipid biosynthesis; Lipid metabolism; Membrane; Oxidoreductase; Plastid; Transit peptide
SRR1271857_TR5423_c0_g1_i11_len=1613	ALFC2_ARATH	Q:38-952,H:1-306	87.62%ID	E:0	Probable fructose-bisphosphate aldolase 2, chloroplastic	PF00274.16^Glycolytic^Fructose-bisphosphate aldolase class-I^65-317^E:1.3e-115	0,062	-0,443	0,055	-0,453	0	-0,798	0,075	-0,37	0,762	0,066	ALFC2_ARATH	Probable fructose-bisphosphate aldolase 2, chloroplastic (EC 4.1.2.13)	FBA2 At4g38970 F19H22.70	Arabidopsis thaliana (Mouse-ear cress)	PATHWAY: Carbohydrate degradation; glycolysis; D-glyceraldehyde 3-phosphate and glycerone phosphate from D-glucose: step 4/4.	Alternative splicing; Chloroplast; Complete proteome; Glycolysis; Lyase; Methylation; Phosphoprotein; Plastid; Reference proteome; Schiff base; Transit peptide
SRR1271857_TR8540_c0_g1_i7_len=2641	CHLD_TOBAC	Q:2535-1339,H:4-397	76.06%ID	E:0	Magnesium-chelatase subunit ChlD, chloroplastic	PF01078.18^Mg_chelatase^Magnesium chelatase, subunit ChlI^205-274^E:8.5e-07	0,046	-0,609	0,011	-0,924	0,001	-1,354	0,003	-1,122	0,691	0,122	CHLD_TOBAC	Magnesium-chelatase subunit ChlD, chloroplastic (Mg-chelatase subunit D) (EC 6.6.1.1) (Mg-protoporphyrin IX chelatase subunit ChlD)	CHLD	Nicotiana tabacum (Common tobacco)	PATHWAY: Porphyrin-containing compound metabolism; chlorophyll biosynthesis.	ATP-binding; Chlorophyll biosynthesis; Chloroplast; Ligase; Nucleotide-binding; Photosynthesis; Plastid; Transit peptide
SRR1271857_TR15267_c1_g1_i1_len=1203	CB22_TOBAC	Q:92-646,H:1-185	91.89%ID	E:1e-119	Chlorophyll a-b binding protein 21, chloroplastic	PF00504.18^Chloroa_b-bind^Chlorophyll A-B binding protein^65-179^E:1.1e-27	0,004	-0,775	0,002	-0,947	0	-1,19	0,035	-0,604	0,622	0,204	CB22_TOBAC	Chlorophyll a-b binding protein 21, chloroplastic (LHCII type I CAB-21) (LHCP)	CAB21	Nicotiana tabacum (Common tobacco)		Acetylation; Chlorophyll; Chloroplast; Chromophore; Magnesium; Membrane; Metal-binding; Phosphoprotein; Photosynthesis; Photosystem I; Photosystem II; Plastid; Thylakoid; Transit peptide; Transmembrane; Transmembrane helix
SRR1271857_TR24967_c0_g1_i1_len=2127	PGKH_TOBAC	Q:241-1656,H:1-472	91.31%ID	E:0	Phosphoglycerate kinase, chloroplastic	PF00162.16^PGK^Phosphoglycerate kinase^86-464^E:2.7e-161	0,543	-0,245	0,948	0,03	0	-1,033	0,025	-0,61	0,753	-0,072	PGKH_TOBAC	Phosphoglycerate kinase, chloroplastic (EC 2.7.2.3)		Nicotiana tabacum (Common tobacco)	PATHWAY: Carbohydrate biosynthesis; Calvin cycle.	ATP-binding; Calvin cycle; Chloroplast; Kinase; Nucleotide-binding; Plastid; Transferase; Transit peptide
SRR1271857_TR25102_c0_g1_i1_len=1862	RCA2_LARTR	Q:201-1511,H:1-435	85.19%ID	E:0	Ribulose bisphosphate carboxylase/oxygenase activase 2, chloroplastic	PF00004.26^AAA^ATPase family associated with various cellular activities (AAA)^165-306^E:6.3e-15	0,214	-0,274	0,872	0,034	0	-0,754	0,028	-0,451	0,763	-0,062	RCA2_LARTR	Ribulose bisphosphate carboxylase/oxygenase activase 2, chloroplastic (RA 2) (RuBisCO activase 2) (RuBisCO activase beta form)	RCA2	Larrea tridentata (Creosote bush) (Zygophyllum tridentatum)		ATP-binding; Chloroplast; Nucleotide-binding; Plastid; Transit peptide
SRR1271857_TR25102_c0_g1_i3_len=2036	RCA_MALDO	Q:201-1688,H:1-436	72.23%ID	E:0	Ribulose bisphosphate carboxylase/oxygenase activase, chloroplastic	PF00004.26^AAA^ATPase family associated with various cellular activities (AAA)^36-177^E:2.9e-15	0,692	-0,562	0,461	-1,292	0,001	-1,202	0,009	-0,865	0,44	0,855	RCA_MALDO	Ribulose bisphosphate carboxylase/oxygenase activase, chloroplastic (RA) (RuBisCO activase)	RCA	Malus domestica (Apple) (Pyrus malus)		ATP-binding; Chloroplast; Nucleotide-binding; Plastid; Transit peptide
SRR1271857_TR30443_c0_g1_i1_len=4141	CAPP2_MESCR	Q:2848-113,H:49-960	88.06%ID	E:0	Phosphoenolpyruvate carboxylase 2	PF00311.14^PEPcase^Phosphoenolpyruvate carboxylase^63-868^E:0	0,663	0,474	1	0,112	0	-1,861	0,004	-1,014	1	0,84	CAPP2_MESCR	Phosphoenolpyruvate carboxylase 2 (PEPC 2) (PEPCase 2) (EC 4.1.1.31)	PPC2	Mesembryanthemum crystallinum (Common ice plant) (Cryophytum crystallinum)	PATHWAY: Photosynthesis; C3 acid pathway.	Allosteric enzyme; Carbon dioxide fixation; Cytoplasm; Lyase; Magnesium; Photosynthesis
SRR1271857_TR36637_c0_g1_i1_len=838	NA	NA	NA	NA	NA	.	1	-0,65	1	-0,645	0	-2,158	0,002	-1,573	1	-0,263	.	.	.	.	.	.
SRR1271857_TR43439_c0_g4_i3_len=2904	GLYM_SOLTU	Q:2634-1591,H:1-347	93.97%ID	E:0	Serine hydroxymethyltransferase, mitochondrial	PF00464.16^SHMT^Serine hydroxymethyltransferase^58-348^E:1.6e-158	0,875	0,052	0,007	0,771	0	-1,114	0,003	-0,718	0	2,713	GLYM_SOLTU	Serine hydroxymethyltransferase, mitochondrial (SHMT) (EC 2.1.2.1) (Glycine hydroxymethyltransferase) (Serine methylase)		Solanum tuberosum (Potato)	PATHWAY: One-carbon metabolism; tetrahydrofolate interconversion.	Complete proteome; Mitochondrion; One-carbon metabolism; Pyridoxal phosphate; Reference proteome; Transferase; Transit peptide
SRR1271858_TR55081_c0_g1_i1_len=1715	SY121_ARATH	Q:1353-505,H:1-288	75.69%ID	E:6e-142	Syntaxin-121	PF00804.22^Syntaxin^Syntaxin^39-244^E:1.8e-71`PF05739.16^SNARE^SNARE domain^245-297^E:7.9e-19	0,38	-0,505	0,254	-4,737	0	-7,402	0,35	-0,626	1	-0,293	SY121_ARATH	Syntaxin-121 (AtSYP121) (Syntaxin-related protein At-Syr1)	SYP121 SYR1 At3g11820 F26K24.11	Arabidopsis thaliana (Mouse-ear cress)		Acetylation; Alternative initiation; Cell membrane; Coiled coil; Complete proteome; Membrane; Protein transport; Reference proteome; Transmembrane; Transmembrane helix; Transport
SRR1271859_TR8002_c0_g1_i9_len=3057	NA	NA	NA	NA	NA	.	0,819	-0,142	0,91	0,076	0,001	-0,977	0,075	-0,515	1	0,049	.	.	.	.	.	.
SRR1271859_TR20442_c0_g1_i1_len=753	NA	NA	NA	NA	NA	.	1	-0,809	1	-1,112	0	-2,041	0,001	-1,632	1	0,228	.	.	.	.	.	.
SRR1271859_TR31135_c0_g2_i1_len=3232	TKTC_SOLTU	Q:2314-578,H:162-740	91.54%ID	E:0	Transketolase, chloroplastic	PF00456.18^Transketolase_N^Transketolase, thiamine diphosphate binding domain^1-178^E:2.8e-68`PF02779.21^Transket_pyr^Transketolase, pyrimidine binding domain^196-366^E:3.1e-44`PF02780.17^Transketolase_C^Transketolase, C-terminal domain^391-497^E:4.7e-13	0,093	-0,487	0,122	-0,479	0,001	-0,814	0,01	-0,605	1	0,254	TKTC_SOLTU	Transketolase, chloroplastic (TK) (EC 2.2.1.1)		Solanum tuberosum (Potato)	PATHWAY: Carbohydrate biosynthesis; Calvin cycle.	Calcium; Chloroplast; Complete proteome; Magnesium; Membrane; Metal-binding; Plastid; Reference proteome; Thiamine pyrophosphate; Thylakoid; Transferase; Transit peptide
SRR1271859_TR40872_c0_g1_i1_len=1627	STR4_ARATH	Q:314-1444,H:1-367	62.2%ID	E:7e-142	Rhodanese-like domain-containing protein 4, chloroplastic	.	0,377	-0,266	0,066	-0,683	0,001	-0,916	0,011	-0,678	0,707	-0,355	STR4_ARATH	Rhodanese-like domain-containing protein 4, chloroplastic (Protein THYLAKOID RHODANESE-LIKE) (Sulfurtransferase 4) (AtStr4)	STR4 TROL At4g01050 F2N1.31	Arabidopsis thaliana (Mouse-ear cress)		3D-structure; Chloroplast; Complete proteome; Membrane; Plastid; Reference proteome; Thylakoid; Transit peptide; Transmembrane; Transmembrane helix
SRR342017_TR1673_c1_g5_i1_len=1157	PSAF_FLATR	Q:315-773,H:79-231	95.42%ID	E:6e-93	Photosystem I reaction center subunit III, chloroplastic	PF02507.12^PSI_PsaF^Photosystem I reaction centre subunit III^57-221^E:4.3e-82	0,189	-0,304	0,031	-0,496	0	-0,98	0,001	-0,732	0,778	0,059	PSAF_FLATR	Photosystem I reaction center subunit III, chloroplastic (Light-harvesting complex I 17 kDa protein) (PSI-F)	PSAF	Flaveria trinervia (Clustered yellowtops) (Oedera trinervia)		Chloroplast; Photosynthesis; Photosystem I; Plastid; Thylakoid; Transit peptide
SRR342017_TR1819_c0_g1_i1_len=573	NA	NA	NA	NA	NA	.	1	-0,098	0,012	-1,523	0	-2,262	0,002	-1,756	0,534	0,357	.	.	.	.	.	.
SRR342017_TR17554_c0_g2_i1_len=1699	EXPA3_ARATH	Q:616-1359,H:15-262	83.47%ID	E:2e-138	Expansin-A3	PF03330.15^DPBB_1^Lytic transglycolase^67-153^E:6.3e-21`PF01357.18^Pollen_allerg_1^Pollen allergen^165-242^E:7.8e-26	0,319	-0,368	0,002	-1,381	0	-1,948	0,002	-1,102	1	-0,076	EXPA3_ARATH	Expansin-A3 (AtEXPA3) (Alpha-expansin-3) (At-EXP3) (AtEx3) (Ath-ExpAlpha-1.9)	EXPA3 EXP3 At2g37640 F13M22.14	Arabidopsis thaliana (Mouse-ear cress)		Cell wall; Cell wall biogenesis/degradation; Complete proteome; Disulfide bond; Membrane; Reference proteome; Secreted; Signal
SRR342017_TR27136_c0_g1_i1_len=1134	PSAD_CUCSA	Q:254-895,H:1-206	75.23%ID	E:3e-101	Photosystem I reaction center subunit II, chloroplastic	PF02531.13^PsaD^PsaD^83-213^E:2.6e-68	0,538	0,181	0,003	-0,827	0,001	-0,861	0,004	-0,734	0,104	0,338	PSAD_CUCSA	Photosystem I reaction center subunit II, chloroplastic (PS I subunit 5) (Photosystem I 20 kDa subunit) (PSI-D)	psaD	Cucumis sativus (Cucumber)		Chloroplast; Direct protein sequencing; Membrane; Photosynthesis; Photosystem I; Plastid; Thylakoid; Transit peptide
SRR342017_TR30126_c0_g1_i1_len=1309	PSBO_SOLTU	Q:1127-882,H:1-81	81.71%ID	E:2e-31	Oxygen-evolving enhancer protein 1, chloroplastic	.	0,249	-0,262	0,422	-0,194	0	-0,922	0,012	-0,546	0	0,838	PSBO_SOLTU	Oxygen-evolving enhancer protein 1, chloroplastic (OEE1) (33 kDa subunit of oxygen evolving system of photosystem II) (33 kDa thylakoid membrane protein) (OEC 33 kDa subunit)	PSBO	Solanum tuberosum (Potato)		Chloroplast; Complete proteome; Manganese; Membrane; Photosynthesis; Photosystem II; Plastid; Reference proteome; Thylakoid; Transit peptide
SRR342019_TR4213_c8_g3_i4_len=1198	GGAP1_ARATH	Q:610-62,H:1-179	73.22%ID	E:3e-77	GDP-L-galactose phosphorylase 1	.	0,108	0,702	1	0	0	-1,706	1	0,012	0	4,183	GGAP1_ARATH	GDP-L-galactose phosphorylase 1 (EC 2.7.7.69) (Protein VITAMIN C DEFECTIVE 2)	VTC2 At4g26850 F10M23.190	Arabidopsis thaliana (Mouse-ear cress)	PATHWAY: Cofactor biosynthesis; L-ascorbate biosynthesis via GDP-alpha-D-mannose pathway; L-ascorbate from GDP-alpha-D-mannose: step 2/5.	Ascorbate biosynthesis; Complete proteome; Cytoplasm; Guanine-nucleotide releasing factor; Hydrolase; Nucleotide-binding; Nucleotidyltransferase; Nucleus; Reference proteome; Transferase
SRR342019_TR12033_c0_g2_i2_len=965	EXPA8_ARATH	Q:816-127,H:25-253	84.42%ID	E:4e-131	Expansin-A8	PF03330.15^DPBB_1^Lytic transglycolase^90-177^E:1.6e-23`PF01357.18^Pollen_allerg_1^Pollen allergen^188-265^E:5.2e-30	0,919	0,036	0,223	-0,335	0	-0,913	0,37	-0,195	0,381	1,995	EXPA8_ARATH	Expansin-A8 (AtEXPA8) (Alpha-expansin-8) (At-EXP8) (AtEx8) (Ath-ExpAlpha-1.11)	EXPA8 EXP8 At2g40610 T2P4.4	Arabidopsis thaliana (Mouse-ear cress)		Cell wall; Cell wall biogenesis/degradation; Complete proteome; Disulfide bond; Membrane; Reference proteome; Secreted; Signal
SRR342019_TR12843_c1_g4_i7_len=4015	SDD1_ARATH	Q:3625-1337,H:6-771	66.28%ID	E:0	.	PF05922.13^Inhibitor_I9^Peptidase inhibitor I9^26-112^E:1.9e-14`PF00082.19^Peptidase_S8^Subtilase family^137-597^E:3.1e-54`PF02225.19^PA^PA domain^399-466^E:1.1e-09	0,098	-0,562	0,002	-1,145	0	-1,217	0,007	-0,917	0,779	0,105	SBT12_ARATH	Subtilisin-like protease SBT1.2 (EC 3.4.21.-) (Cucumisin-like serine protease SDD1) (Protein STOMATAL DENSITY AND DISTRIBUTION 1) (Subtilase subfamily 1 member 2) (AtSBT1.2) (Subtilisin-like protease SDD1)	SBT1.2 SDD1 At1g04110 F20D22.12	Arabidopsis thaliana (Mouse-ear cress)		Apoplast; Cell membrane; Complete proteome; Glycoprotein; Hydrolase; Membrane; Protease; Reference proteome; Secreted; Serine protease; Signal
SRR342019_TR15470_c2_g3_i12_len=2289	GLNA4_PHAVU	Q:882-1835,H:83-400	94.34%ID	E:0	Glutamine synthetase leaf isozyme, chloroplastic	PF00120.21^Gln-synt_C^Glutamine synthetase, catalytic domain^35-213^E:7.8e-16	0,177	-0,329	0,302	-0,247	0	-1,001	0,001	-0,73	0,642	-0,103	GLNA4_PHAVU	Glutamine synthetase leaf isozyme, chloroplastic (EC 6.3.1.2) (Glutamate–ammonia ligase) (Isozyme delta)		Phaseolus vulgaris (Kidney bean) (French bean)		ATP-binding; Chloroplast; Ligase; Nitrogen fixation; Nucleotide-binding; Plastid; Transit peptide
SRR342019_TR19372_c0_g2_i4_len=1385	RPI3_ARATH	Q:1095-307,H:9-276	74.44%ID	E:3e-132	Probable ribose-5-phosphate isomerase 3, chloroplastic	PF06026.11^Rib_5-P_isom_A^Ribose 5-phosphate isomerase A (phosphoriboisomerase A)^110-285^E:9.7e-61	0,095	-0,43	0,036	-0,542	0	-0,93	0,013	-0,623	0,56	-0,837	RPI3_ARATH	Probable ribose-5-phosphate isomerase 3, chloroplastic (EC 5.3.1.6) (Phosphoriboisomerase 3) (Protein EMBRYO DEFECTIVE 3119)	RPI3 EMB3119 At3g04790 F7O18.28 T9J14.26	Arabidopsis thaliana (Mouse-ear cress)	PATHWAY: Carbohydrate degradation; pentose phosphate pathway; D-ribose 5-phosphate from D-ribulose 5-phosphate (non-oxidative stage): step 1/1.	Acetylation; Chloroplast; Complete proteome; Isomerase; Phosphoprotein; Plastid; Reference proteome; Transit peptide
SRR342019_TR21154_c5_g2_i3_len=1002	NA	NA	NA	NA	NA	.	0,004	-1,135	0,381	-1,879	0	-1,743	0,304	-0,404	1	-0,592	.	.	.	.	.	.
SRR342019_TR21889_c1_g2_i3_len=5790	MOT2_ARATH	Q:156-1541,H:5-458	70.73%ID	E:3e-161	Molybdate transporter 2	PF11145.5^DUF2921^Protein of unknown function (DUF2921)^54-989^E:2.4e-203	1	-0,077	0,002	-1,396	0	-1,289	0,002	-1,128	0,454	0,365	MOT2_ARATH	Molybdate transporter 2 (Sulfate transporter like protein 5.1)	MOT2 ST5.1 SULTR5.1 At1g80310 F5I6.6	Arabidopsis thaliana (Mouse-ear cress)		Alternative splicing; Complete proteome; Membrane; Molybdenum; Reference proteome; Transmembrane; Transmembrane helix; Transport; Vacuole
SRR342019_TR24621_c2_g4_i1_len=603	HSP7C_PETHY	Q:526-2,H:1-175	96%ID	E:2e-117	Heat shock cognate 70 kDa protein	PF00012.17^HSP70^Hsp70 protein^34-200^E:1.2e-79	0,732	0,829	0,009	4,736	0	-3,969	0,147	-0,957	0,043	2,579	HSP7C_PETHY	Heat shock cognate 70 kDa protein	HSP70	Petunia hybrida (Petunia)		ATP-binding; Nucleotide-binding; Stress response
SRR342019_TR25586_c0_g1_i1_len=1763	DR100_ARATH	Q:1306-287,H:27-368	61.11%ID	E:8e-146	DNA-damage-repair/toleration protein DRT100	PF08263.9^LRRNT_2^Leucine rich repeat N-terminal domain^24-62^E:3e-10`PF08263.9^LRRNT_2^Leucine rich repeat N-terminal domain^113-117^E:9900`PF13855.3^LRR_8^Leucine rich repeat^152-212^E:2.2e-06`PF00560.30^LRR_1^Leucine Rich Repeat^177-199^E:0.12	0,611	-0,162	0,088	-0,781	0	-1,514	0,007	-1,114	0,257	0,364	DR100_ARATH	DNA-damage-repair/toleration protein DRT100	DRT100 At3g12610 MMF12.5 T2E22.8 T2E22_107	Arabidopsis thaliana (Mouse-ear cress)		Complete proteome; DNA damage; DNA repair; Leucine-rich repeat; Reference proteome; Repeat; Signal
SRR342019_TR27680_c0_g11_i1_len=3135	Y1770_SYNY3	Q:2426-657,H:33-585	44.82%ID	E:2e-160	Uncharacterized protein sll1770	PF03109.13^ABC1^ABC1 family^288-408^E:1.4e-36	0,392	-0,245	0,003	-0,98	0	-0,921	0,014	-0,57	0,002	2,463	Y1770_SYNY3	Uncharacterized protein sll1770	sll1770	Synechocystis sp. (strain PCC 6803 / Kazusa)		Complete proteome; Reference proteome
SRR342019_TR34140_c1_g1_i6_len=2180	GC1_ARATH	Q:1702-1145,H:86-268	81.18%ID	E:5e-103	Epimerase family protein SDR39U1 homolog, chloroplastic {ECO:0000305}	PF01370.18^Epimerase^NAD dependent epimerase/dehydratase family^6-149^E:2.7e-05	0,588	-0,217	0,001	-1,047	0	-1,43	0,001	-1,251	0,027	-2,332	GC1_ARATH	Epimerase family protein SDR39U1 homolog, chloroplastic (EC 1.1.1.-) (Protein GIANT CHLOROPLAST 1) (Protein SulA homolog) (AtSulA)	GC1 SULA At2g21280 F3K23	Arabidopsis thaliana (Mouse-ear cress)		Chloroplast; Complete proteome; Membrane; NADP; Oxidoreductase; Plastid; Plastid inner membrane; Reference proteome; Transit peptide

SRR342019_TR35470_c2_g2_i5_len=2052	TPT_PEA	Q:71-1291,H:1-402	79.17%ID	E:0	Triose phosphate/phosphate translocator, chloroplastic	PF03151.13^TPT^Triose-phosphate Transporter family^127-414^E:4.8e-111`PF00892.17^EamA^EamA-like transporter family^270-414^E:1.9e-06	0,731	-0,092	0,205	-0,349	0	-0,906	0,005	-0,613	0,012	0,618	TPT_PEA	Triose phosphate/phosphate translocator, chloroplastic (cTPT) (E30) (p36)		Pisum sativum (Garden pea)		Chloroplast; Direct protein sequencing; Membrane; Plastid; Transit peptide; Transmembrane; Transmembrane helix; Transport
SRR342019_TR36227_c6_g10_i1_len=1554	GDT11_ARATH	Q:475-1053,H:112-304	77.32%ID	E:1e-83	GDT1-like protein 1, chloroplastic	PF01169.16^UPF0016^Uncharacterized protein family UPF0016^163-246^E:3.1e-19	0,38	-0,521	0,008	-1,573	0,001	-1,657	0,003	-1,397	1	-0,028	GDT11_ARATH	GDT1-like protein 1, chloroplastic	At1g64150 F22C12.9	Arabidopsis thaliana (Mouse-ear cress)		Chloroplast; Complete proteome; Membrane; Plastid; Reference proteome; Transit peptide; Transmembrane; Transmembrane helix
SRR342019_TR38704_c0_g2_i2_len=2572	COLX_ARATH	Q:299-613,H:10-115	52.83%ID	E:1e-28	Putative zinc finger protein At1g68190	.	1	-0,04	0,118	-1,779	0,001	-4,326	0,016	-2,769	0,282	1,129	COLX_ARATH	Putative zinc finger protein At1g68190	At1g68190 T22E19.18	Arabidopsis thaliana (Mouse-ear cress)		Complete proteome; Metal-binding; Nucleus; Reference proteome; Repeat; Zinc; Zinc-finger
SRR342022_TR4690_c0_g1_i2_len=1962	GLYM_SOLTU	Q:143-1594,H:34-518	91.55%ID	E:0	Serine hydroxymethyltransferase, mitochondrial	PF00464.16^SHMT^Serine hydroxymethyltransferase^1-362^E:8e-195	0,747	0,08	0,352	0,213	0	-0,799	0,003	-0,652	0,195	0,284	GLYM_SOLTU	Serine hydroxymethyltransferase, mitochondrial (SHMT) (EC 2.1.2.1) (Glycine hydroxymethyltransferase) (Serine methylase)		Solanum tuberosum (Potato)	PATHWAY: One-carbon metabolism; tetrahydrofolate interconversion.	Complete proteome; Mitochondrion; One-carbon metabolism; Pyridoxal phosphate; Reference proteome; Transferase; Transit peptide
SRR342022_TR8241_c0_g2_i2_len=2500	PPR68_ARATH	Q:808-2499,H:14-579	58.02%ID	E:0	Pentatricopeptide repeat-containing protein At1g31920	PF13041.3^PPR_2^PPR repeat family^95-143^E:4.1e-11`PF13812.3^PPR_3^Pentatricopeptide repeat domain^95-141^E:9e-06`PF01535.17^PPR^PPR repeat^99-126^E:0.0015`PF01535.17^PPR^PPR repeat^170-195^E:0.0022`PF01535.17^PPR^PPR repeat^199-223^E:0.00078`PF01535.17^PPR^PPR repeat^273-299^E:2.8e-05`PF01535.17^PPR^PPR repeat^302-331^E:2.9e-05`PF13812.3^PPR_3^Pentatricopeptide repeat domain^322-382^E:0.00027`PF01535.17^PPR^PPR repeat^336-362^E:0.00036`PF01535.17^PPR^PPR repeat^374-398^E:0.029`PF14432.3^DYW_deaminase^DYW family of nucleic acid deaminases^472-578^E:4.1e-23	0,16	-1,32	0,129	-4,898	0,001	-1,333	0,005	-1,081	0,608	0,229	PPR68_ARATH	Pentatricopeptide repeat-containing protein At1g31920	PCMP-H11 At1g31920 F5M6.8	Arabidopsis thaliana (Mouse-ear cress)		Complete proteome; Reference proteome; Repeat
SRR342022_TR9956_c0_g1_i2_len=2205	APS2_ARATH	Q:346-1782,H:1-475	75.57%ID	E:0	ATP sulfurylase 2	PF14306.3^PUA_2^PUA-like domain^69-231^E:3e-46`PF01747.14^ATP-sulfurylase^ATP-sulfurylase^240-463^E:4.5e-65	0,626	-0,249	0,739	-0,266	0	-1,202	0,003	-0,755	0,629	-0,19	APS2_ARATH	ATP sulfurylase 2 (EC 2.7.7.4)	APS2 ASA1 MET3-1 At1g19920 F6F9.2	Arabidopsis thaliana (Mouse-ear cress)	PATHWAY: Sulfur metabolism; hydrogen sulfide biosynthesis; sulfite from sulfate: step 1/3.	ATP-binding; Alternative initiation; Chloroplast; Complete proteome; Cytoplasm; Nucleotide-binding; Nucleotidyltransferase; Plastid; Reference proteome; Transferase; Transit peptide
SRR342022_TR11785_c0_g2_i3_len=2432	LUT1_ARATH	Q:2216-708,H:33-539	81.85%ID	E:0	Carotene epsilon-monooxygenase, chloroplastic	PF00067.19^p450^Cytochrome P450^99-521^E:9.8e-81	1	-0,01	0,12	-0,662	0	-1,462	0,001	-1,19	0,784	-0,148	LUT1_ARATH	Carotene epsilon-monooxygenase, chloroplastic (EC 1.14.99.45) (Cytochrome P450 97C1) (Protein LUTEIN DEFICIENT 1)	CYP97C1 LUT1 At3g53130 T4D2.60	Arabidopsis thaliana (Mouse-ear cress)		Carotenoid biosynthesis; Chloroplast; Complete proteome; Heme; Iron; Membrane; Metal-binding; Monooxygenase; Oxidoreductase; Plastid; Reference proteome; Transit peptide
SRR342022_TR16045_c5_g2_i5_len=1592	AL3F1_ARATH	Q:116-1414,H:1-484	50.62%ID	E:2e-151	Aldehyde dehydrogenase family 3 member F1	PF00171.19^Aldedh^Aldehyde dehydrogenase family^16-357^E:2e-67	0,29	-0,421	0,034	-1,014	0	-2,065	0,064	-0,98	0,121	0,804	AL3F1_ARATH	Aldehyde dehydrogenase family 3 member F1 (EC 1.2.1.3)	ALDH3F1 At4g36250 F23E13.140	Arabidopsis thaliana (Mouse-ear cress)		Complete proteome; NAD; Oxidoreductase; Reference proteome
SRR342022_TR17158_c1_g3_i13_len=1510	GPPL2_ARATH	Q:368-820,H:66-220	85.81%ID	E:5e-114	Haloacid dehalogenase-like hydrolase domain-containing protein At3g48420	.	0,527	-0,182	0,484	-0,19	0	-0,899	0,015	-0,596	0,914	-0,041	GPPL2_ARATH	Haloacid dehalogenase-like hydrolase domain-containing protein At3g48420 (EC 3.1.3.-)	At3g48420 T29H11.60	Arabidopsis thaliana (Mouse-ear cress)		3D-structure; Chloroplast; Complete proteome; Hydrolase; Magnesium; Metal-binding; Plastid; Reference proteome; Transit peptide
SRR342023_TR5684_c1_g2_i7_len=777	NA	NA	NA	NA	NA	.	0,258	1,033	0,452	-0,553	0	-1,407	0,025	-0,801	0,166	-0,772	.	.	.	.	.	.
SRR342023_TR6746_c3_g1_i3_len=679	RTNLA_ARATH	Q:362-679,H:70-175	72.64%ID	E:1e-49	Reticulon-like protein B1	PF02453.14^Reticulon^Reticulon^70-152^E:2e-25	1	0	1	0	0	-7,727	0,9	-0,134	1	0	RTNLA_ARATH	Reticulon-like protein B1 (AtRTNLB1) (VirB2-interacting protein 1)	RTNLB1 BTI1 At4g23630 F9D16.100	Arabidopsis thaliana (Mouse-ear cress)		Acetylation; Cell membrane; Complete proteome; Endoplasmic reticulum; Membrane; Reference proteome; Transmembrane; Transmembrane helix
SRR342023_TR11601_c0_g1_i1_len=687	NA	NA	NA	NA	NA	.	0,76	0,656	1	-0,021	0	-0,962	0,006	-0,638	0,178	0,631	.	.	.	.	.	.
SRR342023_TR13950_c0_g1_i2_len=554	RK12_NICSY	Q:455-66,H:1-127	71.76%ID	E:1e-35	50S ribosomal protein L12, chloroplastic	PF16320.2^Ribosomal_L12_N^Ribosomal protein L7/L12 dimerisation domain^62-109^E:7.3e-14	0,002	-0,69	0,793	-0,071	0	-0,895	0,015	-0,571	0,927	0,076	RK12_NICSY	50S ribosomal protein L12, chloroplastic (CL12)		Nicotiana sylvestris (Wood tobacco) (South American tobacco)		Chloroplast; Plastid; Ribonucleoprotein; Ribosomal protein; Transit peptide
SRR342023_TR16855_c0_g1_i1_len=869	PSAEA_NICSY	Q:310-672,H:21-141	69.92%ID	E:1e-35	Photosystem I reaction center subunit IV A, chloroplastic	PF02427.14^PSI_PsaE^Photosystem I reaction centre subunit IV / PsaE^82-141^E:2.2e-30	0,246	0,242	0,019	-0,488	0,001	-0,688	0,002	-0,651	0,807	0,052	PSAEA_NICSY	Photosystem I reaction center subunit IV A, chloroplastic (PSI-E A) [Cleaved into: Photosystem I reaction center subunit IV A isoform 2]	PSAEA	Nicotiana sylvestris (Wood tobacco) (South American tobacco)		Chloroplast; Direct protein sequencing; Membrane; Photosynthesis; Photosystem I; Plastid; Thylakoid; Transit peptide
SRR342023_TR19508_c3_g13_i17_len=2221	BASS2_ARATH	Q:205-909,H:1-224	64.68%ID	E:3e-84	Sodium/pyruvate cotransporter BASS2, chloroplastic	PF13593.3^SBF_like^SBF-like CPA transporter family (DUF4137)^136-233^E:1.3e-06`PF01758.13^SBF^Sodium Bile acid symporter family^143-235^E:3.2e-30	0,11	-0,498	0,234	-0,517	0	-1,169	0,027	-0,625	0,051	0,524	BASS2_ARATH	Sodium/pyruvate cotransporter BASS2, chloroplastic (Bile acid transporter 1) (Bile acid-sodium symporter family protein 2)	BASS2 BAT1 SBF1 At2g26900 F12C20.6	Arabidopsis thaliana (Mouse-ear cress)		Acetylation; Chloroplast; Complete proteome; Membrane; Plastid; Reference proteome; Transit peptide; Transmembrane; Transmembrane helix; Transport
SRR342023_TR22755_c1_g2_i2_len=451	NA	NA	NA	NA	NA	.	0,61	-0,176	0,943	0,044	0	-0,903	0,131	-0,352	0,062	0,613	.	.	.	.	.	.
SRR342023_TR23453_c2_g8_i11_len=3617	HEM11_CUCSA	Q:1610-3307,H:1-552	81.27%ID	E:0	Glutamyl-tRNA reductase 1, chloroplastic	PF05201.12^GlutR_N^Glutamyl-tRNAGlu reductase, N-terminal domain^121-271^E:1.3e-38`PF01488.17^Shikimate_DH^Shikimate / quinate 5-dehydrogenase^287-428^E:5.2e-44`PF02423.12^OCD_Mu_crystall^Ornithine cyclodeaminase/mu-crystallin family^299-382^E:3.5e-05`PF03807.14^F420_oxidored^NADP oxidoreductase coenzyme F420-dependent^304-369^E:4.3e-07`PF03446.12^NAD_binding_2^NAD binding domain of 6-phosphogluconate dehydrogenase^304-372^E:9.8e-06`PF00745.17^GlutR_dimer^Glutamyl-tRNAGlu reductase, dimerisation domain^442-546^E:2.8e-29	0,895	0,048	0,382	-0,24	0,001	-0,787	0,03	-0,497	1	0,018	HEM11_CUCSA	Glutamyl-tRNA reductase 1, chloroplastic (GluTR) (EC 1.2.1.70)	HEMA1	Cucumis sativus (Cucumber)	PATHWAY: Porphyrin-containing compound metabolism; protoporphyrin-IX biosynthesis; 5-aminolevulinate from L-glutamyl-tRNA(Glu): step 1/2.	Chlorophyll biosynthesis; Chloroplast; NADP; Oxidoreductase; Plastid; Porphyrin biosynthesis; Transit peptide
SRR342023_TR25894_c1_g3_i4_len=2328	RK2B_COFAR	Q:1983-1585,H:1-133	97.74%ID	E:6e-82	50S ribosomal protein L2-B, chloroplastic	PF03947.15^Ribosomal_L2_C^Ribosomal Proteins L2, C-terminal domain^1-117^E:5e-50	0,018	-0,574	0,021	-0,568	0	-0,993	0,002	-0,748	1	-0,683	RK2B_COFAR	50S ribosomal protein L2-B, chloroplastic	rpl2-B	Coffea arabica (Arabian coffee)		Chloroplast; Plastid; Ribonucleoprotein; Ribosomal protein
SRR646572_TR29356_c0_g1_i1_len=2714	LACS9_ARATH	Q:2235-151,H:1-691	77.12%ID	E:0	Long chain acyl-CoA synthetase 9, chloroplastic	PF00501.25^AMP-binding^AMP-binding enzyme^97-562^E:2.5e-105	0,415	-0,348	0,493	-0,315	0	-1,069	0,002	-0,829	0,258	0,839	LACS9_ARATH	Long chain acyl-CoA synthetase 9, chloroplastic (EC 6.2.1.3)	LACS9 At1g77590 T5M16.18	Arabidopsis thaliana (Mouse-ear cress)	PATHWAY: Lipid metabolism; fatty acid metabolism.	ATP-binding; Chloroplast; Complete proteome; Fatty acid metabolism; Ligase; Lipid metabolism; Magnesium; Nucleotide-binding; Plastid; Reference proteome
SRR646572_TR30105_c1_g1_i4_len=662	NA	NA	NA	NA	NA	.	0,79	-0,132	0,255	0,635	0	-7,044	0,471	-0,522	0,36	0,421	.	.	.	.	.	.
SRR646596_TR10671_c0_g1_i11_len=2200	G3PB_PEA	Q:1804-479,H:10-451	87.56%ID	E:0	Glyceraldehyde-3-phosphate dehydrogenase B, chloroplastic	PF00044.21^Gp_dh_N^Glyceraldehyde 3-phosphate dehydrogenase, NAD binding domain^87-239^E:1.7e-52`PF02800.17^Gp_dh_C^Glyceraldehyde 3-phosphate dehydrogenase, C-terminal domain^244-401^E:2.5e-65`PF02672.12^CP12^CP12 domain^430-451^E:3.3e-05	0,212	-1,009	1	0,06	0	-1,725	0,014	-0,703	0,299	1,476	G3PB_PEA	Glyceraldehyde-3-phosphate dehydrogenase B, chloroplastic (EC 1.2.1.13) (NADP-dependent glyceraldehydephosphate dehydrogenase subunit B)	GAPB GPB1	Pisum sativum (Garden pea)	PATHWAY: Carbohydrate biosynthesis; Calvin cycle.	Calvin cycle; Chloroplast; NADP; Oxidoreductase; Plastid; Transit peptide
SRR646596_TR12961_c0_g1_i1_len=1507	PSBO_SOLTU	Q:1369-371,H:1-332	88.59%ID	E:0	Oxygen-evolving enhancer protein 1, chloroplastic	PF01716.15^MSP^Manganese-stabilising protein / photosystem II polypeptide^146-378^E:1.4e-98	0,43	-0,212	0,658	-0,135	0	-0,887	0,017	-0,583	0,188	0,663	PSBO_SOLTU	Oxygen-evolving enhancer protein 1, chloroplastic (OEE1) (33 kDa subunit of oxygen evolving system of photosystem II) (33 kDa thylakoid membrane protein) (OEC 33 kDa subunit)	PSBO	Solanum tuberosum (Potato)		Chloroplast; Complete proteome; Manganese; Membrane; Photosynthesis; Photosystem II; Plastid; Reference proteome; Thylakoid; Transit peptide
SRR646596_TR16995_c0_g1_i9_len=2470	NA	NA	NA	NA	NA	.	1	-0,135	0,118	2,005	0	-1,296	0,004	-1,023	0,254	4,465	.	.	.	.	.	.
SRR646596_TR29262_c0_g2_i3_len=6439	RK2A_COFAR	Q:6012-5191,H:1-274	98.91%ID	E:6e-168	50S ribosomal protein L2-A, chloroplastic	PF01193.21^RNA_pol_L^RNA polymerase Rpb3/Rpb11 dimerisation domain^35-225^E:6.3e-14`PF01000.23^RNA_pol_A_bac^RNA polymerase Rpb3/RpoA insert domain^68-157^E:3.6e-15	0,101	-0,474	0,013	-0,769	0	-1,319	0,001	-1,017	0,002	-0,797	RK2A_COFAR	50S ribosomal protein L2-A, chloroplastic	rpl2-A	Coffea arabica (Arabian coffee)		Chloroplast; Plastid; Ribonucleoprotein; Ribosomal protein
SRR646604_TR11901_c0_g1_i5_len=2671	PSY_CAPAN	Q:599-1723,H:1-368	78.4%ID	E:0	Bifunctional 15-cis-phytoene synthase, chromoplastic {ECO:0000303|PubMed:16578835}	PF00494.16^SQS_PSY^Squalene/phytoene synthase^150-378^E:1.7e-65	0,878	-0,172	0,472	-0,715	0	-1,566	0,019	-0,934	0,052	1,482	PSY_CAPAN	Bifunctional 15-cis-phytoene synthase, chromoplastic (EC 2.5.1.32) (Geranylgeranyl pyrophosphate geranylgeranyltransferase) (Phytoene synthase)	PSY1	Capsicum annuum (Bell pepper)	PATHWAY: Carotenoid biosynthesis; phytoene biosynthesis; all-trans-phytoene from geranylgeranyl diphosphate: step 1/1. {ECO:0000305}.	Carotenoid biosynthesis; Chromoplast; Isoprene biosynthesis; Multifunctional enzyme; Plastid; Transferase; Transit peptide
SRR646604_TR12580_c1_g2_i21_len=1460	NA	NA	NA	NA	NA	.	1	-0,391	0,502	-2,92	0,001	-1,841	0,002	-1,58	0,76	0,679	.	.	.	.	.	.
SRR646604_TR24623_c0_g1_i1_len=2952	ATPD_TOBAC	Q:1536-2288,H:1-247	73.71%ID	E:3e-120	ATP synthase delta chain, chloroplastic	PF02536.11^mTERF^mTERF^39-217^E:1e-22`PF02536.11^mTERF^mTERF^175-349^E:3.5e-20	0,631	-0,204	0,024	-0,782	0	-1,038	0,003	-0,844	0,459	-0,178	ATPD_TOBAC	ATP synthase delta chain, chloroplastic (F-ATPase delta chain)	ATPD	Nicotiana tabacum (Common tobacco)		ATP synthesis; CF(1); Chloroplast; Hydrogen ion transport; Ion transport; Membrane; Plastid; Thylakoid; Transit peptide; Transport
SRR646604_TR37356_c1_g4_i4_len=1915	PPCK1_ARATH	Q:1849-1061,H:6-268	52.83%ID	E:2e-91	Phosphoenolpyruvate carboxylase kinase 1	PF00069.22^Pkinase^Protein kinase domain^33-286^E:9.7e-70`PF07714.14^Pkinase_Tyr^Protein tyrosine kinase^36-285^E:2e-29`PF14531.3^Kinase-like^Kinase-like^118-271^E:4.3e-07	1	-0,586	0,461	-1,379	0	-2,699	0,002	-1,981	0,629	1,226	PPCK1_ARATH	Phosphoenolpyruvate carboxylase kinase 1 (AtPPCK1) (EC 2.7.11.1)	PPCK1 At1g08650 F22O13.13	Arabidopsis thaliana (Mouse-ear cress)		ATP-binding; Alternative splicing; Complete proteome; Kinase; Nucleotide-binding; Pyruvate; Reference proteome; Serine/threonine-protein kinase; Transferase
SRR648705_TR13253_c0_g2_i2_len=3854	ALFC2_ARATH	Q:3324-2134,H:1-398	88.72%ID	E:0	Probable fructose-bisphosphate aldolase 2, chloroplastic	PF00274.16^Glycolytic^Fructose-bisphosphate aldolase class-I^53-397^E:2.1e-158	0,552	-0,233	1	0,031	0	-0,925	0,019	-0,515	0	2,167	ALFC2_ARATH	Probable fructose-bisphosphate aldolase 2, chloroplastic (EC 4.1.2.13)	FBA2 At4g38970 F19H22.70	Arabidopsis thaliana (Mouse-ear cress)	PATHWAY: Carbohydrate degradation; glycolysis; D-glyceraldehyde 3-phosphate and glycerone phosphate from D-glucose: step 4/4.	Alternative splicing; Chloroplast; Complete proteome; Glycolysis; Lyase; Methylation; Phosphoprotein; Plastid; Reference proteome; Schiff base; Transit peptide
SRR648705_TR15129_c0_g1_i1_len=3951	IF2C_ARATH	Q:3472-476,H:3-1010	68.34%ID	E:0	Translation initiation factor IF-2, chloroplastic	PF00009.24^GTP_EFTU^Elongation factor Tu GTP binding domain^481-641^E:4e-33`PF01926.20^MMR_HSR1^50S ribosome-binding GTPase^482-590^E:6e-08`PF00071.19^Ras^Ras family^482-640^E:2.4e-06`PF11987.5^IF-2^Translation-initiation factor 2^786-877^E:3.5e-29	0,263	-0,374	0,387	-0,496	0	-0,933	0,028	-0,574	0,059	1,229	IF2C_ARATH	Translation initiation factor IF-2, chloroplastic	At1g17220 F20D23.8	Arabidopsis thaliana (Mouse-ear cress)		Chloroplast; Complete proteome; GTP-binding; Initiation factor; Nucleotide-binding; Plastid; Protein biosynthesis; Reference proteome; Transit peptide
SRR648705_TR16698_c0_g1_i4_len=2213	GLO1_ARATH	Q:838-1944,H:1-367	91.33%ID	E:0	Peroxisomal (S)-2-hydroxy-acid oxidase GLO1	PF01070.15^FMN_dh^FMN-dependent dehydrogenase^13-355^E:1.8e-137`PF00478.22^IMPDH^IMP dehydrogenase / GMP reductase domain^265-311^E:4.5e-06	0,528	-0,152	0,771	-0,068	0	-0,801	0,005	-0,583	0,611	0,113	GLO1_ARATH	Peroxisomal (S)-2-hydroxy-acid oxidase GLO1 (EC 1.1.3.15) (Glycolate oxidase 1) (AtGLO1) (GOX 1) (Short chain alpha-hydroxy acid oxidase GLO1)	GLO1 At3g14420 MAO2.2	Arabidopsis thaliana (Mouse-ear cress)	PATHWAY: Photosynthesis; photorespiration; glycine from 2-phosphoglycolate: step 2/3.	Acetylation; Alternative splicing; Complete proteome; Direct protein sequencing; FMN; Flavoprotein; Glycolate pathway; Oxidoreductase; Peroxisome; Photorespiration; Reference proteome
SRR648705_TR34107_c0_g1_i1_len=1400	RRFC_SPIOL	Q:1066-353,H:34-271	72.27%ID	E:1e-110	Ribosome-recycling factor, chloroplastic	PF01765.16^RRF^Ribosome recycling factor^109-270^E:1e-61	0,031	-0,559	0,213	-0,353	0	-1,121	0,004	-0,764	0,18	0,572	RRFC_SPIOL	Ribosome-recycling factor, chloroplastic (RRF) (CpFrr) (RRFHCP) (Ribosome-releasing factor, chloroplastic)	RRF FRRHCP	Spinacia oleracea (Spinach)		Chloroplast; Coiled coil; Direct protein sequencing; Plastid; Protein biosynthesis; Transit peptide
SRR648707_TR5440_c0_g1_i1_len=1710	SAT3_ARATH	Q:138-1319,H:10-391	60.1%ID	E:2e-153	Serine acetyltransferase 3, mitochondrial	PF06426.11^SATase_N^Serine acetyltransferase, N-terminal^133-237^E:1.3e-36`PF00132.21^Hexapep^Bacterial transferase hexapeptide (six repeats)^268-308^E:0.0016`PF00132.21^Hexapep^Bacterial transferase hexapeptide (six repeats)^317-351^E:2.8e-10`PF14602.3^Hexapep_2^Hexapeptide repeat of succinyl-transferase^319-351^E:2.5e-06`PF13436.3^Gly-zipper_OmpA^Glycine-zipper domain^332-348^E:0.75	1	-0,03	1	-0,115	0	-1,193	0,06	-0,474	0,026	1,096	SAT3_ARATH	Serine acetyltransferase 3, mitochondrial (AtSAT-3) (AtSERAT2;2) (SAT-m) (EC 2.3.1.30)	SAT3 SAT1 SATA At3g13110 MJG19.6	Arabidopsis thaliana (Mouse-ear cress)	PATHWAY: Amino-acid biosynthesis; L-cysteine biosynthesis; L-cysteine from L-serine: step 1/2.	Acyltransferase; Amino-acid biosynthesis; Complete proteome; Mitochondrion; Reference proteome; Transferase; Transit peptide
SRR648707_TR18338_c0_g1_i2_len=2862	TKTC_SOLTU	Q:186-2414,H:1-740	87.37%ID	E:0	Transketolase, chloroplastic	PF00456.18^Transketolase_N^Transketolase, thiamine diphosphate binding domain^86-417^E:1.7e-154`PF02779.21^Transket_pyr^Transketolase, pyrimidine binding domain^435-605^E:6.5e-44`PF02780.17^Transketolase_C^Transketolase, C-terminal domain^630-736^E:2e-13	0,04	-0,511	0,059	-0,5	0	-0,873	0,022	-0,508	0,469	0,165	TKTC_SOLTU	Transketolase, chloroplastic (TK) (EC 2.2.1.1)		Solanum tuberosum (Potato)	PATHWAY: Carbohydrate biosynthesis; Calvin cycle.	Calcium; Chloroplast; Complete proteome; Magnesium; Membrane; Metal-binding; Plastid; Reference proteome; Thiamine pyrophosphate; Thylakoid; Transferase; Transit peptide
SRR648707_TR37651_c0_g1_i1_len=521	ADF1_PETHY	Q:250-519,H:1-90	90%ID	E:2e-40	Actin-depolymerizing factor 1	.	0,017	5,775	0,502	4,201	0,001	-1,947	1	-0,025	1	-1,088	ADF1_PETHY	Actin-depolymerizing factor 1 (ADF-1)	ADF1	Petunia hybrida (Petunia)		Actin-binding
SRR924147_TR2070_c0_g1_i1_len=1487	NA	NA	NA	NA	NA	PF04982.10^HPP^HPP family^140-249^E:2.1e-21	0,594	-0,272	0,526	-0,305	0,001	-1,018	0,02	-0,685	0,848	-0,259	.	.	.	.	.	.
SRR924147_TR5931_c0_g2_i1_len=2431	RHM1_ARATH	Q:153-2156,H:1-669	84.3%ID	E:0	Trifunctional UDP-glucose 4,6-dehydratase/UDP-4-keto-6-deoxy-D-glucose 3,5-epimerase/UDP-4-keto-L-rhamnose-reductase RHM1 {ECO:0000305|PubMed:17190829}	PF04321.14^RmlD_sub_bind^RmlD substrate binding domain^8-317^E:1.7e-15`PF01370.18^Epimerase^NAD dependent epimerase/dehydratase family^9-251^E:2.7e-54`PF02719.12^Polysacc_synt_2^Polysaccharide biosynthesis protein^9-276^E:1.3e-18`PF16363.2^GDP_Man_Dehyd^GDP-mannose 4,6 dehydratase^10-315^E:7.5e-70`PF01073.16^3Beta_HSD^3-beta hydroxysteroid dehydrogenase/isomerase family^10-235^E:1.2e-24`PF07993.9^NAD_binding_4^Male sterility protein^11-191^E:5.5e-19`PF04321.14^RmlD_sub_bind^RmlD substrate binding domain^385-557^E:3.7e-12`PF01370.18^Epimerase^NAD dependent epimerase/dehydratase family^414-540^E:8.5e-11	0,555	-0,352	0,023	-1,638	0	-2,076	0,002	-1,83	0,376	-0,49	RHM1_ARATH	Trifunctional UDP-glucose 4,6-dehydratase/UDP-4-keto-6-deoxy-D-glucose 3,5-epimerase/UDP-4-keto-L-rhamnose-reductase RHM1 (Protein REPRESSOR OF LRX1 1) (Rhamnose biosynthetic enzyme 1) (AtRHM1) [Includes: UDP-glucose 4,6-dehydratase (EC 4.2.1.76); UDP-4-keto-6-deoxy-D-glucose 3,5-epimerase/UDP-4-keto-L-rhamnose 4-keto-reductase (EC 1.1.1.-) (EC 5.1.3.-)]	RHM1 ROL1 At1g78570 T30F21.10	Arabidopsis thaliana (Mouse-ear cress)	PATHWAY: Carbohydrate biosynthesis. {ECO:0000305}.	Cell wall biogenesis/degradation; Complete proteome; Cytoplasm; Isomerase; Lyase; Multifunctional enzyme; NAD; NADP; Oxidoreductase; Reference proteome; Ubl conjugation
SRR924147_TR14324_c0_g2_i1_len=2081	PLGG1_ARATH	Q:1762-329,H:37-512	74.9%ID	E:0	Plastidal glycolate/glycerate translocator 1, chloroplastic	PF04172.13^LrgB^LrgB-like family^285-508^E:1.4e-40	0,968	0,021	0,205	0,325	0	-0,997	0,003	-0,744	0,13	0,473	PLGG1_ARATH	Plastidal glycolate/glycerate translocator 1, chloroplastic (Bacterial membrane protein LrgB-like protein) (AtLrgB)	PLGG1 At1g32080 F3C3.12 T12O21.2	Arabidopsis thaliana (Mouse-ear cress)		Acetylation; Chloroplast; Complete proteome; Membrane; Photorespiration; Plastid; Reference proteome; Transit peptide; Transmembrane; Transmembrane helix; Transport
SRR924147_TR26451_c0_g1_i1_len=1795	NA	NA	NA	NA	NA	.	1	-0,015	0,29	-0,504	0	-0,893	0,012	-0,614	0,05	-0,734	.	.	.	.	.	.
SRR924148_TR15509_c0_g1_i1_len=2062	UVR8_ARATH	Q:939-412,H:171-345	40.68%ID	E:5e-32	Ultraviolet-B receptor UVR8	PF13540.3^RCC1_2^Regulator of chromosome condensation (RCC1) repeat^177-200^E:9.1e-06`PF13540.3^RCC1_2^Regulator of chromosome condensation (RCC1) repeat^298-327^E:6.2e-07`PF00415.15^RCC1^Regulator of chromosome condensation (RCC1) repeat^315-391^E:1.4e-07`PF13540.3^RCC1_2^Regulator of chromosome condensation (RCC1) repeat^378-407^E:1.2e-05`PF00415.15^RCC1^Regulator of chromosome condensation (RCC1) repeat^395-443^E:4.3e-10`PF13540.3^RCC1_2^Regulator of chromosome condensation (RCC1) repeat^433-459^E:2.7e-06`PF00415.15^RCC1^Regulator of chromosome condensation (RCC1) repeat^446-497^E:2.2e-12`PF13540.3^RCC1_2^Regulator of chromosome condensation (RCC1) repeat^487-513^E:5e-10`PF00415.15^RCC1^Regulator of chromosome condensation (RCC1) repeat^500-548^E:3.2e-12	1	-0,601	1	0,506	0,001	-1,295	0,001	-1,262	1	-0,173	UVR8_ARATH	Ultraviolet-B receptor UVR8 (Protein UV-B RESISTANCE 8) (RCC1 domain-containing protein UVR8)	UVR8 At5g63860 MGI19.7	Arabidopsis thaliana (Mouse-ear cress)		3D-structure; Acetylation; Chromophore; Complete proteome; Cytoplasm; Nucleus; Photoreceptor protein; Receptor; Reference proteome; Repeat; Sensory transduction
SRR924148_TR28214_c0_g1_i1_len=855	PSAN_ARATH	Q:185-688,H:1-168	69.19%ID	E:2e-60	Photosystem I reaction center subunit N, chloroplastic	PF05479.8^PsaN^Photosystem I reaction centre subunit N (PSAN or PSI-N)^104-232^E:7.3e-68	1	-3,475	1	-3,504	0	-8,016	0,915	0,091	0,085	-0,751	PSAN_ARATH	Photosystem I reaction center subunit N, chloroplastic (PSI-N)	PSAN At5g64040 MHJ24.2	Arabidopsis thaliana (Mouse-ear cress)		Acetylation; Alternative splicing; Chloroplast; Complete proteome; Direct protein sequencing; Membrane; Photosynthesis; Photosystem I; Plastid; Reference proteome; Thylakoid; Transit peptide
SRR924148_TR30734_c0_g1_i1_len=2758	DXS_CAPAN	Q:2382-226,H:1-719	87.24%ID	E:0	Probable 1-deoxy-D-xylulose-5-phosphate synthase, chloroplastic	PF13292.3^DXP_synthase_N^1-deoxy-D-xylulose-5-phosphate synthase^76-361^E:5.5e-114`PF00676.17^E1_dh^Dehydrogenase E1 component^187-247^E:4.1e-05`PF02779.21^Transket_pyr^Transketolase, pyrimidine binding domain^398-559^E:8.1e-44`PF02780.17^Transketolase_C^Transketolase, C-terminal domain^577-700^E:9.8e-31	0,037	-0,525	0,017	0,677	0	-1,074	0,23	-0,307	0,09	0,934	DXS_CAPAN	Probable 1-deoxy-D-xylulose-5-phosphate synthase, chloroplastic (1-deoxyxylulose-5-phosphate synthase) (DXP synthase) (DXPS) (EC 2.2.1.7) (CapTKT2)	TKT2	Capsicum annuum (Bell pepper)	PATHWAY: Metabolic intermediate biosynthesis; 1-deoxy-D-xylulose 5-phosphate biosynthesis; 1-deoxy-D-xylulose 5-phosphate from D-glyceraldehyde 3-phosphate and pyruvate: step 1/1.	Chloroplast; Isoprene biosynthesis; Magnesium; Metal-binding; Plastid; Thiamine biosynthesis; Thiamine pyrophosphate; Transferase; Transit peptide

MsDamaged24h_MsIntact24h

Transcript (CDF97)	Uniprot homolog	BLASTX Position	BLASTX %Identity	BLASTX evalue	Uniprot descr	Pfam	PValue_Ms6h	logFC_Ms6h	PValue_Ms8h	logFC_Ms8h	PValue_MsDamaged24h	logFC_MsDamaged24h	PValue_MsIntact24h	logFC_MsIntact24h	PValue_Ms1wk	logFC_Ms1wk	Entry.name	Protein names	Gene names	Organism	Pathway	Keywords
SRR1144633_TR11351_c0_g2_i1_len=1413	NA	NA	NA	NA	NA	PF11250.5^FAF^Fantastic Four meristem regulator^160-209^E:1.6e-16	0,211	-0,822	0,076	-2,604	0	-3,524	0	-3,785	0,035	-1,615	.	.	.	.	.	.
SRR1144633_TR11707_c0_g3_i4_len=1279	CB12_SOLLC	Q:1094-297,H:1-265	89.85%ID	E:5e-174	Chlorophyll a-b binding protein 7, chloroplastic	PF00504.18^Chloroa_b-bind^Chlorophyll A-B binding protein^75-241^E:1e-51	0,229	-0,27	0,001	-0,753	0	-1,123	0	-0,918	0,412	0,549	CB12_SOLLC	Chlorophyll a-b binding protein 7, chloroplastic (LHCI type II CAB-7)	CAB7	Solanum lycopersicum (Tomato) (Lycopersicon esculentum)		Chlorophyll; Chloroplast; Chromophore; Complete proteome; Magnesium; Membrane; Metal-binding; Phosphoprotein; Photosynthesis; Photosystem I; Photosystem II; Plastid; Reference proteome; Thylakoid; Transit peptide; Transmembrane; Transmembrane helix
SRR1144633_TR14325_c0_g1_i1_len=2997	CNGC2_ARATH	Q:540-2696,H:5-726	72.53%ID	E:0	Cyclic nucleotide-gated ion channel 2	PF00520.28^Ion_trans^Ion transport protein^130-454^E:1.5e-12`PF00027.26^cNMP_binding^Cyclic nucleotide-binding domain^556-643^E:1.2e-06`PF00612.24^IQ^IQ calmodulin-binding motif^673-689^E:0.18	0,424	-0,292	0,058	-0,792	0	-1,14	0	-1,154	0,626	-0,216	CNGC2_ARATH	Cyclic nucleotide-gated ion channel 2 (AtCNGC2) (Cyclic nucleotide- and calmodulin-regulated ion channel 2) (Protein DEFENSE NO DEATH 1)	CNGC2 DND1 At5g15410 T20K14_20	Arabidopsis thaliana (Mouse-ear cress)		Alternative splicing; Calmodulin-binding; Cell membrane; Complete proteome; Ion channel; Ion transport; Ligand-gated ion channel; Membrane; Nucleotide-binding; Plant defense; Reference proteome; Transmembrane; Transmembrane helix; Transport; cAMP; cAMP-binding; cGMP; cGMP-binding
SRR1144633_TR17118_c0_g1_i1_len=2301	HSP72_SOLLC	Q:2145-208,H:1-644	94.58%ID	E:0	Heat shock cognate 70 kDa protein 2	PF00012.17^HSP70^Hsp70 protein^9-618^E:1.3e-268`PF06723.10^MreB_Mbl^MreB/Mbl protein^127-380^E:8.4e-14	0,161	-0,362	0	5,349	0	-9,168	0	-2,364	0,663	0,476	HSP72_SOLLC	Heat shock cognate 70 kDa protein 2	HSC-2	Solanum lycopersicum (Tomato) (Lycopersicon esculentum)		ATP-binding; Complete proteome; Nucleotide-binding; Reference proteome; Stress response
SRR1144633_TR19640_c0_g2_i1_len=920	RPBAL_ARATH	Q:84-290,H:1-69	91.3%ID	E:4e-39	DNA-directed RNA polymerase subunit 10-like protein {ECO:0000303|PubMed:19110459}	.	0	7,498	0	6,832	0	-7,425	0	-5,851	1	0,028	RPBAL_ARATH	DNA-directed RNA polymerase subunit 10-like protein (ABC10) (DNA-directed RNA polymerase III subunit L) (DNA-directed RNA polymerases I, II, and III subunit RPABC5) (RNA polymerases I, II, and III subunit ABC5) (RPB10 homolog)	NRPB10L At1g61700 T13M11.6	Arabidopsis thaliana (Mouse-ear cress)		Complete proteome; DNA-directed RNA polymerase; Metal-binding; Nucleus; Reference proteome; Transcription; Zinc
SRR1144633_TR22384_c0_g1_i1_len=768	ALFC2_ARATH	Q:497-3,H:12-177	81.33%ID	E:9e-89	Probable fructose-bisphosphate aldolase 2, chloroplastic	PF00274.16^Glycolytic^Fructose-bisphosphate aldolase class-I^132-255^E:1.2e-51	0,87	-0,219	0,094	1,352	0	-1,34	0	-0,867	0,298	0,562	ALFC2_ARATH	Probable fructose-bisphosphate aldolase 2, chloroplastic (EC 4.1.2.13)	FBA2 At4g38970 F19H22.70	Arabidopsis thaliana (Mouse-ear cress)	PATHWAY: Carbohydrate degradation; glycolysis; D-glyceraldehyde 3-phosphate and glycerone phosphate from D-glucose: step 4/4.	Alternative splicing; Chloroplast; Complete proteome; Glycolysis; Lyase; Methylation; Phosphoprotein; Plastid; Reference proteome; Schiff base; Transit peptide
SRR1144633_TR34904_c0_g1_i1_len=2150	C76B6_CATRO	Q:189-1664,H:6-490	50.61%ID	E:2e-163	Geraniol 8-hydroxylase	PF00067.19^p450^Cytochrome P450^35-484^E:9e-114	1	0,235	0	2,458	0	-1,505	0	-1,446	1	0,236	C76B6_CATRO	Geraniol 8-hydroxylase (EC 1.14.13.152) (Cytochrome P450 76B6) (Geraniol 10-hydroxylase) (CrG10H)	CYP76B6 G10H	Catharanthus roseus (Madagascar periwinkle) (Vinca rosea)		Direct protein sequencing; Endoplasmic reticulum; Heme; Iron; Membrane; Metal-binding; Monooxygenase; NADP; Oxidoreductase; Transmembrane; Transmembrane helix
SRR1144634_TR4950_c1_g1_i4_len=1031	RBS_PINTH	Q:634-852,H:98-170	68.49%ID	E:2e-52	Ribulose bisphosphate carboxylase small chain, chloroplastic	PF12338.5^RbcS^Ribulose-1,5-bisphosphate carboxylase small subunit^4-44^E:2.1e-23`PF00101.17^RuBisCO_small^Ribulose bisphosphate carboxylase, small chain^71-105^E:3.1e-09	0,085	0,569	0,036	0,501	0	-1,238	0	-1,144	0,013	-0,516	RBS_PINTH	Ribulose bisphosphate carboxylase small chain, chloroplastic (RuBisCO small subunit) (EC 4.1.1.39)	RBCS	Pinus thunbergii (Japanese black pine) (Pinus thunbergiana)		Calvin cycle; Carbon dioxide fixation; Chloroplast; Lyase; Monooxygenase; Oxidoreductase; Photorespiration; Photosynthesis; Plastid; Transit peptide
SRR1144634_TR8696_c0_g2_i1_len=1529	CADH6_ORYSJ	Q:194-1264,H:2-358	73.67%ID	E:0	Probable cinnamyl alcohol dehydrogenase 6	PF08240.9^ADH_N^Alcohol dehydrogenase GroES-like domain^37-150^E:3.9e-26`PF02826.16^2-Hacid_dh_C^D-isomer specific 2-hydroxyacid dehydrogenase, NAD binding domain^183-228^E:1.3e-06`PF00107.23^ADH_zinc_N^Zinc-binding dehydrogenase^196-318^E:3.4e-20	0,442	-0,196	0,003	-0,897	0	-1,296	0	-1,309	0,077	0,435	CADH6_ORYSJ	Probable cinnamyl alcohol dehydrogenase 6 (OsCAD6) (EC 1.1.1.195)	CAD6 Os04g0229100 LOC_Os04g15920 OSJNBa0065B15.8	Oryza sativa subsp. japonica (Rice)	PATHWAY: Aromatic compound metabolism; phenylpropanoid biosynthesis.	Complete proteome; Lignin biosynthesis; Metal-binding; NADP; Oxidoreductase; Reference proteome; Zinc
SRR1144634_TR34776_c0_g2_i1_len=4485	GUN8_ARATH	Q:3404-4483,H:128-487	79.44%ID	E:0	Endoglucanase 8	PF14223.3^Retrotran_gag_2^gag-polypeptide of LTR copia-type^59-187^E:7e-34`PF00098.20^zf-CCHC^Zinc knuckle^227-242^E:0.00034`PF13976.3^gag_pre-integrs^GAG-pre-integrase domain^397-464^E:1.3e-14`PF00665.23^rve^Integrase core domain^480-594^E:2.5e-26	0,034	-1,052	1	3,313	0	-4,056	0	-3,734	1	0	GUN8_ARATH	Endoglucanase 8 (EC 3.2.1.4) (Cellulase 1) (AtCEL1) (Endo-1,4-beta glucanase 8)	CEL1 At1g70710 F5A18.11	Arabidopsis thaliana (Mouse-ear cress)		Carbohydrate metabolism; Cell wall biogenesis/degradation; Cellulose degradation; Complete proteome; Glycoprotein; Glycosidase; Hydrolase; Polysaccharide degradation; Reference proteome; Secreted; Signal
SRR1144634_TR39096_c0_g1_i1_len=869	NA	NA	NA	NA	NA	PF02466.16^Tim17^Tim17/Tim22/Tim23/Pmp24 family^35-127^E:1e-09	0	8,686	0	7,332	0	-6,345	0	-6,968	1	0	.	.	.	.	.	.
SRR122236_TR26186_c0_g1_i1_len=2251	PII2_ARATH	Q:728-1909,H:19-423	58.82%ID	E:9e-133	Piriformospora indica-insensitive protein 2	PF13855.3^LRR_8^Leucine rich repeat^194-253^E:4.5e-07`PF00560.30^LRR_1^Leucine Rich Repeat^218-240^E:0.1`PF13855.3^LRR_8^Leucine rich repeat^235-277^E:1.3e-07	0,026	-1,051	0,016	-2,379	0	-1,971	0	-2,303	0,484	0,355	PII2_ARATH	Piriformospora indica-insensitive protein 2	PII-2 At1g13230 F3F19.26	Arabidopsis thaliana (Mouse-ear cress)		Cell membrane; Complete proteome; Leucine-rich repeat; Membrane; Reference proteome; Repeat; Signal
SRR122237_TR19100_c0_g1_i1_len=2478	CYSG_PSEU2	Q:1272-658,H:264-464	47.57%ID	E:3e-46	Siroheme synthase {ECO:0000255|HAMAP-Rule:MF_01646}	PF00590.17^TP_methylase^Tetrapyrrole (Corrin/Porphyrin) Methylases^119-166^E:6.3e-12	1	-0,375	1	-0,881	0	-3,332	0,001	-2,66	1	-0,175	CYSG_PSEU2	Siroheme synthase [Includes: Uroporphyrinogen-III C-methyltransferase (Urogen III methylase) (EC 2.1.1.107) (SUMT) (Uroporphyrinogen III methylase) (UROM); Precorrin-2 dehydrogenase (EC 1.3.1.76); Sirohydrochlorin ferrochelatase (EC 4.99.1.4)]	cysG Psyr_3174	Pseudomonas syringae pv. syringae (strain B728a)	PATHWAY: Cofactor biosynthesis; adenosylcobalamin biosynthesis; precorrin-2 from uroporphyrinogen III: step 1/1. {ECO:0000255|HAMAP-Rule:MF_01646}.; PATHWAY: Cofactor biosynthesis; adenosylcobalamin biosynthesis; sirohydrochlorin from precorrin-2: step 1/1. {ECO:0000255|HAMAP-Rule:MF_01646}.; PATHWAY: Porphyrin-containing compound metabolism; siroheme biosynthesis; precorrin-2 from uroporphyrinogen III: step 1/1. {ECO:0000255|HAMAP-Rule:MF_01646}.; PATHWAY: Porphyrin-containing compound metabolism; siroheme biosynthesis; siroheme from sirohydrochlorin: step 1/1. {ECO:0000255|HAMAP-Rule:MF_01646}.; PATHWAY: Porphyrin-containing compound metabolism; siroheme biosynthesis; sirohydrochlorin from precorrin-2: step 1/1. {ECO:0000255|HAMAP-Rule:MF_01646}.	Cobalamin biosynthesis; Complete proteome; Lyase; Methyltransferase; Multifunctional enzyme; NAD; Oxidoreductase; Phosphoprotein; Porphyrin biosynthesis; S-adenosyl-L-methionine; Transferase
SRR122237_TR19703_c0_g1_i1_len=876	NA	NA	NA	NA	NA	.	0	2,56	0,39	-0,193	0	-1,572	0	-1,033	0,001	0,939	.	.	.	.	.	.
SRR122238_TR3001_c0_g1_i3_len=2753	NA	NA	NA	NA	NA	.	0,043	-0,655	0,16	-1,38	0	-2,135	0	-2,307	0,195	0,852	.	.	.	.	.	.
SRR122238_TR11172_c0_g1_i2_len=1183	NA	NA	NA	NA	NA	PF09366.7^DUF1997^Protein of unknown function (DUF1997)^84-251^E:1.2e-42	1	0,794	0,629	-1,811	0	-1,508	0	-1,413	0,675	-0,271	.	.	.	.	.	.
SRR122238_TR18557_c0_g1_i2_len=742	CB4A_ARATH	Q:128-598,H:14-172	80.5%ID	E:8e-80	Chlorophyll a-b binding protein CP29.1, chloroplastic	PF00504.18^Chloroa_b-bind^Chlorophyll A-B binding protein^91-196^E:8.1e-21	0,204	-0,275	0,381	0,204	0	-1,664	0	-0,971	0,054	0,407	CB4A_ARATH	Chlorophyll a-b binding protein CP29.1, chloroplastic (LHCB4.1) (LHCII protein 4.1)	LHCB4.1 At5g01530 F7A7_50	Arabidopsis thaliana (Mouse-ear cress)		Chlorophyll; Chloroplast; Chromophore; Complete proteome; Magnesium; Membrane; Metal-binding; Phosphoprotein; Photosynthesis; Photosystem I; Photosystem II; Plastid; Reference proteome; Thylakoid; Transit peptide; Transmembrane; Transmembrane helix
SRR122238_TR21707_c0_g2_i2_len=3020	DADIS_PANGI	Q:438-2717,H:1-763	74.48%ID	E:0	Dammarenediol II synthase	PF13249.3^SQHop_cyclase_N^Squalene-hopene cyclase N-terminal domain^103-361^E:4.2e-39`PF00432.18^Prenyltrans^Prenyltransferase and squalene oxidase repeat^149-191^E:3.6e-09`PF13243.3^SQHop_cyclase_C^Squalene-hopene cyclase C-terminal domain^415-754^E:6.2e-48`PF00432.18^Prenyltrans^Prenyltransferase and squalene oxidase repeat^592-632^E:8.1e-09`PF00432.18^Prenyltrans^Prenyltransferase and squalene oxidase repeat^641-691^E:1.8e-07	0,879	0,057	0,097	-0,576	0	-1,38	0	-1,543	0,005	0,709	DADIS_PANGI	Dammarenediol II synthase (EC 4.2.1.125)	PNA DDS	Panax ginseng (Korean ginseng)		Isomerase; Lyase; Membrane; Repeat; Transmembrane; Transmembrane helix
SRR122238_TR21715_c1_g1_i1_len=1988	Y3885_ARATH	Q:688-1986,H:1-415	48.46%ID	E:3e-104	Uncharacterized protein At3g28850	PF00462.21^Glutaredoxin^Glutaredoxin^269-338^E:5e-14	0,564	-0,46	0,593	-0,791	0,001	-1,947	0	-2,318	0,471	0,565	Y3885_ARATH	Uncharacterized protein At3g28850	At3g28850 T19N8.15	Arabidopsis thaliana (Mouse-ear cress)		Complete proteome; Reference proteome
SRR122238_TR27538_c0_g1_i1_len=2064	C76B6_CATRO	Q:117-1535,H:21-493	100%ID	E:0	Geraniol 8-hydroxylase	PF00067.19^p450^Cytochrome P450^31-479^E:2e-106	0,618	0,141	0,053	-0,783	0	-1,368	0	-2,061	0,926	-0,031	C76B6_CATRO	Geraniol 8-hydroxylase (EC 1.14.13.152) (Cytochrome P450 76B6) (Geraniol 10-hydroxylase) (CrG10H)	CYP76B6 G10H	Catharanthus roseus (Madagascar periwinkle) (Vinca rosea)		Direct protein sequencing; Endoplasmic reticulum; Heme; Iron; Membrane; Metal-binding; Monooxygenase; NADP; Oxidoreductase; Transmembrane; Transmembrane helix
SRR122238_TR33100_c0_g1_i5_len=2211	TPPA_ARATH	Q:1540-398,H:1-383	64.34%ID	E:5e-173	Trehalose-phosphate phosphatase A	PF02358.13^Trehalose_PPase^Trehalose-phosphatase^123-366^E:1e-71	1	-0,038	0,08	-1,119	0	-1,355	0,001	-1,114	0,458	-0,492	TPPA_ARATH	Trehalose-phosphate phosphatase A (AtTPPA) (EC 3.1.3.12) (Trehalose 6-phosphate phosphatase)	TPPA At5g51460 K17N15.1 MFG13.17	Arabidopsis thaliana (Mouse-ear cress)	PATHWAY: Glycan biosynthesis; trehalose biosynthesis.	Alternative splicing; Complete proteome; Hydrolase; Reference proteome; Stress response
SRR122238_TR35343_c0_g1_i2_len=1132	NA	NA	NA	NA	NA	.	1	0,076	0,044	-2,168	0	-4,036	0	-4,906	0,129	3,672	.	.	.	.	.	.
SRR122238_TR36935_c0_g1_i3_len=1475	PRP4_ARATH	Q:1276-911,H:32-147	54.47%ID	E:8e-31	Proline-rich protein 4	PF01190.14^Pollen_Ole_e_I^Pollen proteins Ole e I like^69-151^E:1.8e-11	0,49	0,154	0,177	0,301	0	-1,143	0	-0,966	0,569	0,16	PRP4_ARATH	Proline-rich protein 4 (AtPRP4)	PRP4 At4g38770 T9A14.50	Arabidopsis thaliana (Mouse-ear cress)		Cell wall; Complete proteome; Reference proteome; Repeat; Secreted; Signal
SRR122238_TR41386_c0_g1_i4_len=2097	TAL2_STRAW	Q:436-1287,H:10-299	52.92%ID	E:2e-75	Transaldolase 2 {ECO:0000255|HAMAP-Rule:MF_00493}	PF00923.16^Transaldolase^Transaldolase^110-366^E:1.5e-63	0,76	-0,381	0,282	-1,28	0,001	-2,555	0,001	-2,499	1	0,055	TAL2_STRAW	Transaldolase 2 (EC 2.2.1.2)	tal2 SAV_6314	Streptomyces avermitilis (strain ATCC 31267 / DSM 46492 / JCM 5070 / NBRC 14893 / NCIMB 12804 / NRRL 8165 / MA-4680)	PATHWAY: Carbohydrate degradation; pentose phosphate pathway; D-glyceraldehyde 3-phosphate and beta-D-fructose 6-phosphate from D-ribose 5-phosphate and D-xylulose 5-phosphate (non-oxidative stage): step 2/3. {ECO:0000255|HAMAP-Rule:MF_00493}.	Complete proteome; Cytoplasm; Pentose shunt; Reference proteome; Schiff base; Transferase
SRR122238_TR42650_c0_g2_i1_len=1117	NA	NA	NA	NA	NA	.	0,419	-0,379	0,03	-1,066	0	-1,96	0	-1,983	0,003	0,824	.	.	.	.	.	.
SRR122238_TR50432_c0_g1_i1_len=514	UGT2_GARJA	Q:2-319,H:376-480	66.04%ID	E:2e-34	7-deoxyloganetin glucosyltransferase	PF00201.15^UDPGT^UDP-glucoronosyl and UDP-glucosyl transferase^2-80^E:4.2e-13	1	-1,017	1	-2,096	0	-1,014	0	-2,044	0,502	-4,275	UGT2_GARJA	7-deoxyloganetin glucosyltransferase (EC 2.4.1.324) (Genipin glucosyltransferase) (UDP-glucose glucosyltransferase 2) (GjUGT2) (UDP-glycosyltransferase 85A24)	UGT85A24 UGT2	Gardenia jasminoides (Cape jasmine) (Gardenia augusta)		Glycosyltransferase; Transferase
SRR122238_TR51937_c0_g1_i1_len=864	COPT5_ARATH	Q:223-621,H:1-[illegible]	50%ID	E:7e-37	Copper transporter 5	PF04145.12^Ctr^Ctr copper transporter family^4-131^E:3.2e-23	1	[illegible]	[illegible]	-1,111	0	-0,963	0	-1,166	0,823	0,141	COPT5_ARATH	Copper transporter 5 (AtCOPT5)	COPT5 At5g20650 T1M15.50	Arabidopsis thaliana (Mouse-ear cress)		Complete proteome; Copper; Copper transport; Ion transport; Membrane; Reference proteome; Transmembrane; [illegible]

[illegible]	[illegible]	[illegible] 130	[illegible]	[illegible]	Copper transporter 5	[illegible]	[illegible]	[illegible]	[illegible]	[illegible]	[illegible]	[illegible]	[illegible]	[illegible]	[illegible]	[illegible]	[illegible]	[illegible]	[illegible]	Arabidopsis thaliana (Mouse-ear cress)		[illegible] Transmembrane helix; Transport
SRR122238_TR59710_c0_g1_i1_len=1451	NA	NA	NA	NA	NA	PF01190.14^Pollen_Ole_e_I^Pollen proteins Ole e I like^43-132^E:2.1e-20	1	-0,055	0,009	-1,705	0	-1,186	0	-1,775	0,038	0,555	.	.	.	.	.	.
SRR122238_TR68398_c0_g1_i1_len=2711	CYP37_ARATH	Q:2372-1167,H:54-456	69.48%ID	E:0	Peptidyl-prolyl cis-trans isomerase CYP37, chloroplastic	PF00160.18^Pro_isomerase^Cyclophilin type peptidyl-prolyl cis-trans isomerase/CLD^351-501^E:1.2e-13	0,268	-0,43	0,077	-0,66	0	-1,664	0	-1,695	1	0,028	CYP37_ARATH	Peptidyl-prolyl cis-trans isomerase CYP37, chloroplastic (PPIase CYP37) (EC 5.2.1.8) (Rotamase CYP37) (Thylakoid lumen PPIase of 38 kDa) (TLP38) (p38)	CYP37 At3g15520 MJK13.18	Arabidopsis thaliana (Mouse-ear cress)		Chloroplast; Complete proteome; Direct protein sequencing; Isomerase; Plastid; Reference proteome; Rotamase; Thylakoid; Transit peptide
SRR122238_TR79167_c0_g1_i1_len=2232	C71A2_SOLME	Q:235-1617,H:46-505	53.22%ID	E:9e-168	Cytochrome P450 71A2	PF00067.19^p450^Cytochrome P450^45-494^E:2.9e-101	1	2,093	1	-0,047	0	-1,295	0	-2,231	0,026	2,134	C71A2_SOLME	Cytochrome P450 71A2 (EC 1.14.-.-) (CYPLXXIA2) (Cytochrome P-450EG4)	CYP71A2 CYPEG4	Solanum melongena (Eggplant) (Aubergine)		Heme; Iron; Membrane; Metal-binding; Monooxygenase; Oxidoreductase; Transmembrane; Transmembrane helix
SRR122238_TR80429_c0_g3_i1_len=634	NA	NA	NA	NA	NA	.	1	0,064	0,044	-1,933	0	-2,072	0	-2,077	1	-0,213	.	.	.	.	.	.
SRR122238_TR88044_c0_g1_i4_len=2084	CYP38_ARATH	Q:336-1565,H:23-437	78.95%ID	E:0	Peptidyl-prolyl cis-trans isomerase CYP38, chloroplastic	PF00160.18^Pro_isomerase^Cyclophilin type peptidyl-prolyl cis-trans isomerase/CLD^272-431^E:1.8e-24	0,177	-0,356	0,105	-0,449	0	-1,384	0	-0,974	0,539	0,176	CYP38_ARATH	Peptidyl-prolyl cis-trans isomerase CYP38, chloroplastic (PPIase CYP38) (EC 5.2.1.8) (Rotamase CYP38) (Thylakoid lumen PPIase)	CYP38 At3g01480 F4P13.3	Arabidopsis thaliana (Mouse-ear cress)		3D-structure; Alternative splicing; Chloroplast; Complete proteome; Direct protein sequencing; Isomerase; Plastid; Reference proteome; Rotamase; Thylakoid; Transit peptide
SRR122238_TR90509_c0_g1_i1_len=1757	NA	NA	NA	NA	NA	.	1	-0,394	0,381	-1,863	0	-4,72	0	-4,86	1	0,248	.	.	.	.	.	.
SRR1271857_TR3570_c0_g1_i1_len=2452	KTNA1_ARATH	Q:240-1775,H:8-523	85.11%ID	E:0	Katanin p60 ATPase-containing subunit A1 {ECO:0000255|HAMAP-Rule:MF_03023}	PF05496.9^RuvB_N^Holliday junction DNA helicase ruvB N-terminus^268-337^E:8.2e-05`PF00004.26^AAA^ATPase family associated with various cellular activities (AAA)^269-406^E:1e-37`PF09336.7^Vps4_C^Vps4 C terminal oligomerisation domain^470-515^E:4e-11	1	-0,039	0,009	4,606	0	-5,64	0	-6,23	1	-0,061	KTNA1_ARATH	Katanin p60 ATPase-containing subunit A1 (Katanin p60 subunit A1) (EC 3.6.4.3) (CAD ATPase) (Katanin-1) (Atp60) (Protein BOTERO 1) (Protein ECTOPIC ROOT HAIR 3) (Protein FAT ROOT) (Protein FRAGILE FIBER 2) (AtAAA1) (p60 katanin)	AAA1 BOT1 ERH3 FRA2 FTR KSS KTN1 LUE1 At1g80350 F5I6.10	Arabidopsis thaliana (Mouse-ear cress)		ATP-binding; Complete proteome; Cytoplasm; Cytoskeleton; Hydrolase; Microtubule; Nucleotide-binding; Reference proteome
SRR1271857_TR4760_c0_g1_i1_len=1809	G3PA2_ARATH	Q:1487-333,H:14-399	87.6%ID	E:0	Glyceraldehyde-3-phosphate dehydrogenase GAPA2, chloroplastic	PF00044.21^Gp_dh_N^Glyceraldehyde 3-phosphate dehydrogenase, NAD binding domain^69-219^E:8.7e-51`PF02800.17^Gp_dh_C^Glyceraldehyde 3-phosphate dehydrogenase, C-terminal domain^224-380^E:5.4e-67	0,125	-0,334	0,001	-0,698	0	-0,947	0	-0,732	1	0,003	G3PA2_ARATH	Glyceraldehyde-3-phosphate dehydrogenase GAPA2, chloroplastic (EC 1.2.1.13) (NADP-dependent glyceraldehydephosphate dehydrogenase A subunit 2)	GAPA2 At1g12900 F13K23.15	Arabidopsis thaliana (Mouse-ear cress)	PATHWAY: Carbohydrate biosynthesis; Calvin cycle.	Alternative splicing; Calvin cycle; Chloroplast; Complete proteome; NADP; Oxidoreductase; Plastid; Reference proteome; Transit peptide
SRR1271857_TR5192_c0_g1_i1_len=4027	CP26B_ARATH	Q:3305-2616,H:85-312	66.09%ID	E:2e-95	Peptidyl-prolyl cis-trans isomerase CYP26-2, chloroplastic	PF07967.10^zf-C3HC^C3HC zinc finger-like^174-297^E:2.1e-33	0,298	-0,584	0,006	-1,768	0	-1,431	0,001	-1,219	0,634	0,249	CP26B_ARATH	Peptidyl-prolyl cis-trans isomerase CYP26-2, chloroplastic (PPIase CYP26-2) (EC 5.2.1.8) (Cyclophilin of 26 kDa 2) (Cyclophilin-26-2)	CYP26-2 At1g74070 F2P9.6	Arabidopsis thaliana (Mouse-ear cress)		Chaperone; Chloroplast; Complete proteome; Cyclosporin; Isomerase; Plastid; Reference proteome; Rotamase; Thylakoid; Transit peptide
SRR1271857_TR7014_c0_g2_i1_len=562	AX15A_SOYBN	Q:416-213,H:12-79	66.18%ID	E:1e-26	Auxin-induced protein 15A	.	1	-0,214	0,027	-2,165	0	-2,731	0	-1,974	0,84	-0,289	AX15A_SOYBN	Auxin-induced protein 15A		Glycine max (Soybean) (Glycine hispida)		Auxin signaling pathway; Complete proteome; Reference proteome
SRR1271857_TR11355_c0_g2_i1_len=2197	NA	NA	NA	NA	NA	PF09713.7^A_thal_3526^Plant protein 1589 of unknown function (A_thal_3526)^17-68^E:4e-23	0,472	-0,613	0,003	-2,551	0,001	-3,285	0,001	-3,058	1	0,468	.	.	.	.	.	.
SRR1271857_TR12704_c0_g1_i1_len=1898	GLK1_ARATH	Q:214-1575,H:1-418	40.17%ID	E:7e-65	Transcription activator GLK1	PF00249.28^Myb_DNA-binding^Myb-like DNA-binding domain^174-223^E:5e-08	0,486	-0,286	0,211	-0,513	0	-1,342	0	-1,541	0,587	0,28	GLK1_ARATH	Transcription activator GLK1 (GBF'S PRO-RICH REGION-INTERACTING factor 1) (Golden2-like protein 1) (AtGLK1)	GLK1 GPRI1 At2g20570 F23N11.11	Arabidopsis thaliana (Mouse-ear cress)		Activator; Alternative splicing; Complete proteome; DNA-binding; Nucleus; Reference proteome; Transcription; Transcription regulation
SRR1271857_TR20667_c0_g1_i1_len=2351	AAP7_ARATH	Q:1300-182,H:82-465	55.99%ID	E:3e-129	Probable amino acid permease 7	PF01490.15^Aa_trans^Transmembrane amino acid transporter protein^7-358^E:3.1e-70	0,832	0,209	0,175	-0,936	0	-2,316	0	-2,3	0,299	-1,173	AAP7_ARATH	Probable amino acid permease 7 (Amino acid transporter AAP7)	AAP7 At5g23810 MRO11.15	Arabidopsis thaliana (Mouse-ear cress)		Alternative splicing; Amino-acid transport; Cell membrane; Complete proteome; Membrane; Reference proteome; Symport; Transmembrane; Transmembrane helix; Transport
SRR1271857_TR21768_c0_g2_i1_len=1410	DIV_ANTMA	Q:290-943,H:23-219	47.06%ID	E:1e-43	Transcription factor DIVARICATA	PF00249.28^Myb_DNA-binding^Myb-like DNA-binding domain^8-55^E:3.2e-07`PF00249.28^Myb_DNA-binding^Myb-like DNA-binding domain^134-178^E:9.7e-11	0,464	-0,347	0,001	-1,614	0,001	-1,489	0	-1,948	0,229	0,55	DIV_ANTMA	Transcription factor DIVARICATA	DIVARICATA	Antirrhinum majus (Garden snapdragon)		DNA-binding; Developmental protein; Nucleus; Transcription; Transcription regulation
SRR1271857_TR24445_c0_g1_i1_len=2716	AAT_AQUAE	Q:1767-2477,H:151-394	30.12%ID	E:5e-25	Aspartate aminotransferase	PF00155.18^Aminotran_1_2^Aminotransferase class I and II^17-170^E:5e-20	1	-0,058	0,175	1,012	0,001	-1,829	0	-2,057	0,093	-0,787	AAT_AQUAE	Aspartate aminotransferase (AspAT) (EC 2.6.1.1) (Transaminase A)	aspC aq_1969	Aquifex aeolicus (strain VF5)		Aminotransferase; Complete proteome; Cytoplasm; Pyridoxal phosphate; Reference proteome; Transferase
SRR1271857_TR30443_c0_g1_i2_len=4726	CAPP2_MESCR	Q:2149-113,H:283-960	87.78%ID	E:0	Phosphoenolpyruvate carboxylase 2	PF00311.14^PEPcase^Phosphoenolpyruvate carboxylase^3-681^E:9.2e-257	0,798	-0,344	0,088	-1,771	0	-2,332	0	-1,603	1	0,251	CAPP2_MESCR	Phosphoenolpyruvate carboxylase 2 (PEPC 2) (PEPCase 2) (EC 4.1.1.31)	PPC2	Mesembryanthemum crystallinum (Common ice plant) (Cryophytum crystallinum)	PATHWAY: Photosynthesis; C3 acid pathway.	Allosteric enzyme; Carbon dioxide fixation; Cytoplasm; Lyase; Magnesium; Photosynthesis
SRR1271857_TR30492_c0_g1_i3_len=1760	NA	NA	NA	NA	NA	.	1	0,054	0,066	-1,746	0	-2,493	0	-2,628	1	0,296	.	.	.	.	.	.
SRR1271857_TR36126_c0_g1_i1_len=947	NA	NA	NA	NA	NA	PF05349.9^GATA-N^GATA-type transcription activator, N-terminal^51-123^E:0.00012	1	0,872	0,031	1,444	0	-1,323	0	-1,166	0,848	-0,201	.	.	.	.	.	.
SRR1271857_TR40607_c0_g1_i23_len=4029	GLGL1_ARATH	Q:3802-2240,H:1-522	75.95%ID	E:0	Glucose-1-phosphate adenylyltransferase large subunit 1, chloroplastic	PF00483.20^NTP_transferase^Nucleotidyl transferase^90-367^E:5.1e-74	0,484	-0,193	0,392	-0,233	0	-1,37	0	-1,128	0,45	0,213	GLGL1_ARATH	Glucose-1-phosphate adenylyltransferase large subunit 1, chloroplastic (EC 2.7.7.27) (ADP-glucose pyrophosphorylase) (ADP-glucose synthase) (AGPase S) (Alpha-D-glucose-1-phosphate adenyl transferase)	ADG2 APL1 At5g19220 T24G5.120	Arabidopsis thaliana (Mouse-ear cress)	PATHWAY: Glycan biosynthesis; starch biosynthesis.	ATP-binding; Allosteric enzyme; Chloroplast; Complete proteome; Nucleotide-binding; Nucleotidyltransferase; Phosphoprotein; Plastid; Reference proteome; Starch biosynthesis; Transferase; Transit peptide
SRR1271858_TR21646_c0_g2_i1_len=1981	NA	NA	NA	NA	NA	.	0,321	0,384	0	6,352	0	-7,616	0	-7,616	0,164	-0,513	.	.	.	.	.	.
SRR1271858_TR36366_c0_g1_i4_len=2547	NADB_ARATH	Q:349-2259,H:6-642	74.77%ID	E:0	L-aspartate oxidase, chloroplastic	PF00890.21^FAD_binding_2^FAD binding domain^88-472^E:3.7e-90`PF01266.21^DAO^FAD dependent oxidoreductase^88-216^E:3.2e-07`PF02910.17^Succ_DH_flav_C^Fumarate reductase flavoprotein C-term^529-618^E:3e-15	1	-0,057	1	0,92	0	-1,839	0	-2,023	1	0,372	NADB_ARATH	L-aspartate oxidase, chloroplastic (EC 1.4.3.16) (Protein FLAGELLIN-INSENSITIVE 4)	AO FIN4 At5g14760 T9L3.60	Arabidopsis thaliana (Mouse-ear cress)	PATHWAY: Cofactor biosynthesis; NAD(+) biosynthesis; iminoaspartate from L-aspartate (oxidase route): step 1/1.	Chloroplast; Complete proteome; FAD; Flavoprotein; Oxidoreductase; Plastid; Pyridine nucleotide biosynthesis; Reference proteome; Transit peptide
SRR1271859_TR441_c0_g2_i1_len=1790	PSBS_SOLSG	Q:937-158,H:1-271	80.29%ID	E:6e-86	Photosystem II 22 kDa protein, chloroplastic	PF00504.18^Chloroa_b-bind^Chlorophyll A-B binding protein^84-211^E:7e-09	0,949	-0,016	0,012	-0,548	0	-1,053	0,001	-0,73	0,059	0,456	PSBS_SOLSG	Photosystem II 22 kDa protein, chloroplastic (CP22)	PSBS	Solanum sogarandinum		Chloroplast; Membrane; Photosynthesis; Photosystem II; Plastid; Repeat; Thylakoid; Transit peptide; Transmembrane; Transmembrane helix
SRR1271859_TR1756_c0_g1_i1_len=541	NA	NA	NA	NA	NA	.	0,62	0,243	0,636	-0,177	0	-1,212	0	-1,209	0,618	0,203	.	.	.	.	.	.
SRR1271859_TR5788_c0_g3_i2_len=847	HSP21_IPONI	Q:564-788,H:79-155	74.03%ID	E:1e-24	17.2 kDa class II heat shock protein	.	0,365	-0,518	0,258	-0,92	0,001	-1,35	0	-1,641	0,6	-0,337	HSP21_IPONI	17.2 kDa class II heat shock protein	SHSP-1	Ipomoea nil (Japanese morning glory) (Pharbitis nil)		Cytoplasm; Stress response
SRR1271859_TR7367_c0_g1_i2_len=912	RBS_MALSP	Q:673-173,H:17-183	85.03%ID	E:7e-104	Ribulose bisphosphate carboxylase small chain, chloroplastic	PF12338.5^RbcS^Ribulose-1,5-bisphosphate carboxylase small subunit^4-44^E:4.4e-23`PF00101.17^RuBisCO_small^Ribulose bisphosphate carboxylase, small chain^71-179^E:1.3e-39	0	1,039	0,001	0,716	0	-1,471	0	-1,312	0,006	-0,585	RBS_MALSP	Ribulose bisphosphate carboxylase small chain, chloroplastic (RuBisCO small subunit) (EC 4.1.1.39)	RBCS	Malus sp. (Crab apple)		Calvin cycle; Carbon dioxide fixation; Chloroplast; Lyase; Monooxygenase; Oxidoreductase; Photorespiration; Photosynthesis; Plastid; Transit peptide
SRR1271859_TR7961_c1_g2_i7_len=3066	RVE1_ARATH	Q:255-761,H:34-189	58.82%ID	E:4e-38	Protein REVEILLE 1	PF00249.28^Myb_DNA-binding^Myb-like DNA-binding domain^57-100^E:4.8e-13	1	-0,482	1	-0,191	0	-2,062	0	-2,049	0,643	0,572	RVE1_ARATH	Protein REVEILLE 1	RVE1 At5g17300 MKP11.15	Arabidopsis thaliana (Mouse-ear cress)		Biological rhythms; Complete proteome; DNA-binding; Nucleus; Reference proteome; Transcription; Transcription regulation
SRR1271859_TR9527_c0_g1_i1_len=580	NA	NA	NA	NA	NA	.	0,854	0,236	0,051	-1,039	0	-1,461	0	-1,807	0,193	0,705	.	.	.	.	.	.
SRR1271859_TR10426_c0_g1_i1_len=783	NA	NA	NA	NA	NA	.	1	0	1	0,105	0	-3,119	0,001	-2,833	1	-0,496	.	.	.	.	.	.
SRR1271859_TR16022_c0_g1_i2_len=2497	NIR_BETPN	Q:2081-441,H:36-583	85.4%ID	E:0	Ferredoxin--nitrite reductase, chloroplastic	PF03460.14^NIR_SIR_ferr^Nitrite/Sulfite reductase ferredoxin-like half domain^129-194^E:5.4e-18`PF01077.19^NIR_SIR^Nitrite and sulphite reductase 4Fe-4S domain^204-361^E:2.7e-37`PF03460.14^NIR_SIR_ferr^Nitrite/Sulfite reductase ferredoxin-like half domain^386-449^E:6.6e-21`PF01077.19^NIR_SIR^Nitrite and sulphite reductase 4Fe-4S domain^468-572^E:5.7e-10	0,269	-0,375	0,725	-0,108	0	-1,5	0	-1,294	0,876	-0,084	NIR_BETPN	Ferredoxin--nitrite reductase, chloroplastic (EC 1.7.7.1)	NIR1	Betula pendula (European white birch) (Betula verrucosa)	PATHWAY: Nitrogen metabolism; nitrate reduction (assimilation).	4Fe-4S; Chloroplast; Electron transport; Heme; Iron; Iron-sulfur; Metal-binding; Nitrate assimilation; Oxidoreductase; Plastid; Transit peptide; Transport
SRR1271859_TR19852_c0_g1_i9_len=1061	PPCK1_ARATH	Q:149-943,H:15-279	53.93%ID	E:4e-100	Phosphoenolpyruvate carboxylase kinase 1	PF00069.22^Pkinase^Protein kinase domain^9-266^E:1.5e-72`PF07714.14^Pkinase_Tyr^Protein tyrosine kinase^12-261^E:2e-29`PF14531.3^Kinase-like^Kinase-like^94-247^E:4.3e-07	1	-0,544	0,059	-1,583	0	-2,378	0	-1,855	0,057	1,449	PPCK1_ARATH	Phosphoenolpyruvate carboxylase kinase 1 (AtPPCK1) (EC 2.7.11.1)	PPCK1 At1g08650 F22O13.13	Arabidopsis thaliana (Mouse-ear cress)		ATP-binding; Alternative splicing; Complete proteome; Kinase; Nucleotide-binding; Pyruvate; Reference proteome; Serine/threonine-protein kinase; Transferase
SRR1271859_TR21359_c0_g2_i1_len=1148	NA	NA	NA	NA	NA	.	0,34	0,529	0,017	5,728	0	-9,307	0	-9,307	1	-0,222	.	.	.	.	.	.
SRR1271859_TR32386_c6_g2_i1_len=1517	NA	NA	NA	NA	NA	.	0	1,484	0,117	0,335	0	-1,273	0	-1,148	0,75	-0,077	.	.	.	.	.	.
SRR1271859_TR42336_c0_g3_i1_len=1314	UGT6_CATRO	Q:161-1312,H:1-384	64.25%ID	E:0	7-deoxyloganetin glucosyltransferase	PF00201.15^UDPGT^UDP-glucoronosyl and UDP-glucosyl transferase^237-384^E:3.9e-11	1	1,414	1	-1,228	0	-1,46	0	-2,099	1	-1,736	UGT6_CATRO	7-deoxyloganetin glucosyltransferase (EC 2.4.1.324) (Iridoid 1-O-glucosyltransferase) (UDP-glucose glucosyltransferase 6) (CrUGT6) (UDP-glycosyltransferase 85A23)	UGT85A23 UGT6	Catharanthus roseus (Madagascar periwinkle) (Vinca rosea)		Transferase
SRR1271859_TR45607_c0_g1_i2_len=2019	FD6E2_SOYBN	Q:1487-330,H:1-382	76.29%ID	E:0	Omega-6 fatty acid desaturase, endoplasmic reticulum isozyme 2	PF00487.21^FA_desaturase^Fatty acid desaturase^91-351^E:1.5e-32	0,708	-0,166	0,003	-1,613	0	-2,017	0	-1,368	0,796	-0,147	FD6E2_SOYBN	Omega-6 fatty acid desaturase, endoplasmic reticulum isozyme 2 (EC 1.14.19.-)	FAD2-2	Glycine max (Soybean) (Glycine hispida)	PATHWAY: Lipid metabolism; polyunsaturated fatty acid biosynthesis.	Complete proteome; Endoplasmic reticulum; Fatty acid biosynthesis; Fatty acid metabolism; Lipid biosynthesis; Lipid metabolism; Membrane; Oxidoreductase; Reference proteome; Transmembrane; Transmembrane helix
SRR342017_TR13432_c0_g1_i1_len=716	IAA17_ARATH	Q:604-29,H:3-218	50.69%ID	E:2e-60	Auxin-responsive protein IAA17	PF02309.13^AUX_IAA^AUX/IAA family^31-201^E:3.3e-58	1	-0,038	0	3,815	0	-2,94	0	-2,596	1	-0,12	IAA17_ARATH	Auxin-responsive protein IAA17 (Auxin response 3) (Indoleacetic acid-induced protein 17)	IAA17 AXR3 At1g04250 F19P19.31	Arabidopsis thaliana (Mouse-ear cress)		3D-structure; Auxin signaling pathway; Complete proteome; Nucleus; Phosphoprotein; Reference proteome; Repressor; Transcription; Transcription regulation
SRR342017_TR13773_c0_g2_i1_len=635	GCSH_FLAAN	Q:212-586,H:1-121	82.4%ID	E:6e-66	Glycine cleavage system H protein, mitochondrial	PF01597.16^GCV_H^Glycine cleavage H-protein^42-125^E:1.4e-31	0,632	-0,107	0,038	-0,456	0	-1,061	0	-0,771	0,789	0,059	GCSH_FLAAN	Glycine cleavage system H protein, mitochondrial	GDCSH GCDH GCSH	Flaveria anomala (Yellowtops)		Lipoyl; Mitochondrion; Transit peptide
SRR342017_TR18746_c0_g1_i4_len=1332	NA	NA	NA	NA	NA	PF05512.8^AWPM-19^AWPM-19-like family^15-155^E:1.4e-52	1	0,065	0,04	-1,436	0	-1,978	0	-2,076	0,84	-0,33	.	.	.	.	.	.
SRR342017_TR19184_c1_g1_i3_len=1339	TL29_SOLLC	Q:106-1128,H:1-345	75.43%ID	E:0	Thylakoid lumenal 29 kDa protein, chloroplastic	PF00141.20^peroxidase^Peroxidase^128-300^E:1.2e-24	0,662	-0,103	0,236	-0,274	0	-1,129	0	-1,092	0,366	0,21	TL29_SOLLC	Thylakoid lumenal 29 kDa protein, chloroplastic (TL29) (EC 1.-.-.-) (LeAPx09) (P29)	CLEB3J9	Solanum lycopersicum (Tomato) (Lycopersicon esculentum)		Chloroplast; Complete proteome; Oxidoreductase; Plastid; Reference proteome; Thylakoid; Transit peptide
SRR342017_TR19226_c0_g1_i1_len=7059	VGA_BPS13	Q:2363-798,H:1-522	98.47%ID	E:0	A' protein	PF05840.10^Phage_GPA^Bacteriophage replication gene A protein (GPA)^84-406^E:1.1e-77	0,131	-0,372	0,467	0,188	0	-1,313	0	-1,091	0,091	-0,457	VGA_BPS13	A' protein (GPA)	A	Enterobacteria phage S13 (Bacteriophage S13)		Alternative initiation; Complete proteome; DNA-binding; Endonuclease; Hydrolase; Metal-binding; Nuclease; Zinc; Zinc-finger
SRR342017_TR21437_c0_g1_i1_len=1377	NA	NA	NA	NA	NA	PF11250.5^FAF^Fantastic Four meristem regulator^100-153^E:4.4e-19	1	-1,303	0,254	-2,556	0	-4,129	0	-4,982	1	-0,642	.	.	.	.	.	.
SRR342017_TR23313_c2_g1_i3_len=2140	GLYK_ARATH	Q:443-1549,H:87-455	73.24%ID	E:0	D-glycerate 3-kinase, chloroplastic	.	0,451	0,328	0	4,222	0	-6,62	0	-6,674	0,099	-0,831	GLYK_ARATH	D-glycerate 3-kinase, chloroplastic (AtGLYK) (EC 2.7.1.31)	GLYK At1g80380 F5I6.13	Arabidopsis thaliana (Mouse-ear cress)	PATHWAY: Photosynthesis; photorespiration; 3-phospho-D-glycerate from glycine: step 4/4.	ATP-binding; Alternative splicing; Chloroplast; Complete proteome; Glycolate pathway; Kinase; Nucleotide-binding; Photorespiration; Plastid; Reference proteome; Transferase; Transit peptide
SRR342017_TR24328_c0_g3_i3_len=574	NU50B_ARATH	Q:396-572,H:320-378	72.88%ID	E:5e-22	Nuclear pore complex protein NUP50B {ECO:0000303|PubMed:21189294}	PF00638.15^Ran_BP1^RanBP1 domain^121-173^E:1e-07	0,163	-0,689	0,001	6,638	0	-6,728	0	-6,728	0,196	0,517	NU50B_ARATH	Nuclear pore complex protein NUP50B (Nucleoporin 50B)	NUP50B At3g15970 MSL1.1	Arabidopsis thaliana (Mouse-ear cress)		Acetylation; Complete proteome; Nuclear pore complex; Nucleus; Phosphoprotein; Protein transport; Reference proteome; Repeat; Translocation; Transport; mRNA transport
SRR342017_TR24903_c0_g4_i1_len=3970	PSAA_EUCGG	Q:3431-1182,H:1-750	99.33%ID	E:0	Photosystem I P700 chlorophyll a apoprotein A1 {ECO:0000255|HAMAP-Rule:MF_00458}	PF00223.16^PsaA_PsaB^Photosystem I psaA/psaB protein^38-750^E:0	0,058	0,819	0	2,022	0	-1,767	0	-1,43	1	-0,304	PSAA_EUCGG	Photosystem I P700 chlorophyll a apoprotein A1 (EC 1.97.1.12) (PSI-A) (PsaA)	psaA	Eucalyptus globulus subsp. globulus (Tasmanian blue gum)		4Fe-4S; Chlorophyll; Chloroplast; Chromophore; Electron transport; Iron; Iron-sulfur; Magnesium; Membrane; Metal-binding; Oxidoreductase; Photosynthesis; Photosystem I; Plastid; Thylakoid; Transmembrane; Transmembrane helix; Transport
SRR342017_TR28976_c3_g2_i2_len=667	NA	NA	NA	NA	NA	PF00403.23^HMA^Heavy-metal-associated domain^36-92^E:1.4e-10	0,067	-0,634	0,001	-1,677	0	-2,727	0	-2,421	0,008	0,906	.	.	.	.	.	.
SRR342019_TR1943_c7_g2_i9_len=1293	CAHC_TOBAC	Q:1188-202,H:1-321	77.27%ID	E:4e-159	Carbonic anhydrase, chloroplastic	PF00484.16^Pro_CA^Carbonic anhydrase^156-313^E:2.8e-39	0,013	-0,562	0,047	0,434	0	-2,118	0	-1,833	0,059	0,45	CAHC_TOBAC	Carbonic anhydrase, chloroplastic (EC 4.2.1.1) (Carbonate dehydratase)		Nicotiana tabacum (Common tobacco)		Chloroplast; Lyase; Plastid; Transit peptide; Zinc
SRR342019_TR10041_c4_g1_i3_len=1246	NA	NA	NA	NA	NA	.	1	0,144	0,021	-1,237	0	-1,589	0	-1,86	0,748	0,225	.	.	.	.	.	.
SRR342019_TR12494_c1_g4_i3_len=2852	NA	NA	NA	NA	NA	PF00581.17^Rhodanese^Rhodanese-like domain^387-498^E:1.2e-05	0,675	-0,133	0,831	0,065	0	-0,997	0,001	-0,83	1	0,361	.	.	.	.	.	.
SRR342019_TR15470_c2_g3_i11_len=2865	GLNA2_DAUCA	Q:1206-2411,H:1-403	89.08%ID	E:0	Glutamine synthetase, chloroplastic	PF03951.16^Gln-synt_N^Glutamine synthetase, beta-Grasp domain^81-156^E:2.2e-10`PF00120.21^Gln-synt_C^Glutamine synthetase, catalytic domain^185-406^E:7.7e-16	1	-0,036	0,2	-0,698	0,001	-1,112	0,001	-1,142	0,716	0,106	GLNA2_DAUCA	Glutamine synthetase, chloroplastic (EC 6.3.1.2) (GS2) (Glutamate--ammonia ligase)	GLN2	Daucus carota (Wild carrot)		ATP-binding; Chloroplast; Ligase; Nucleotide-binding; Plastid; Transit peptide
SRR342019_TR18767_c0_g1_i2_len=1818	RCA2_LARTR	Q:909-1691,H:175-435	87.74%ID	E:1e-166	Ribulose bisphosphate carboxylase/oxygenase activase 2, chloroplastic	PF00004.26^AAA^ATPase family associated with various cellular activities (AAA)^2-117^E:9.3e-09	0,56	-1,007	0,118	-2,264	0	-1,644	0	-1,819	0,111	1,248	RCA2_LARTR	Ribulose bisphosphate carboxylase/oxygenase activase 2, chloroplastic (RA 2) (RuBisCO activase 2) (RuBisCO activase beta form)	RCA2	Larrea tridentata (Creosote bush) (Zygophyllum tridentatum)		ATP-binding; Chloroplast; Nucleotide-binding; Plastid; Transit peptide
SRR342019_TR20493_c2_g2_i1_len=2173	NA	NA	NA	NA	NA	.	0,488	0,261	1	0,064	0,001	-1,111	0	-1,152	1	-0,499	.	.	.	.	.	.
SRR342019_TR27542_c2_g1_i2_len=1730	CB5_ARATH	Q:882-1601,H:92-280	70.42%ID	E:4e-94	Chlorophyll a-b binding protein CP26, chloroplastic	.	0,798	0,492	0,088	-1,655	0,001	-1,759	0	-2,105	0,581	0,453	CB5_ARATH	Chlorophyll a-b binding protein CP26, chloroplastic (LHCB5) (LHCIIc) (Light-harvesting complex II protein 5)	LHCB5 At4g10340 F24G24.140	Arabidopsis thaliana (Mouse-ear cress)		Chlorophyll; Chloroplast; Chromophore; Complete proteome; Magnesium; Membrane; Metal-binding; Phosphoprotein; Photosynthesis; Photosystem I; Photosystem II; Plastid; Reference proteome; Thylakoid; Transit peptide; Transmembrane; Transmembrane helix
SRR342019_TR31144_c1_g1_i7_len=1420	FER_CAPAN	Q:764-1201,H:1-144	73.29%ID	E:2e-57	Ferredoxin, chloroplastic	.	0,212	-0,573	0,9	0,123	0	-2,948	0	-2,274	0,35	0,362	FER_CAPAN	Ferredoxin, chloroplastic (PFLP)	AP1	Capsicum annuum (Bell pepper)		2Fe-2S; Chloroplast; Electron transport; Iron; Iron-sulfur; Metal-binding; Phosphoprotein; Plastid; Transit peptide; Transport
SRR342019_TR34140_c1_g1_i2_len=629	GC1_ARATH	Q:624-73,H:40-223	80.43%ID	E:2e-101	Epimerase family protein SDR39U1 homolog, chloroplastic {ECO:0000305}	PF05368.10^NmrA^NmrA-like family^10-68^E:6.5e-06`PF01370.18^Epimerase^NAD dependent epimerase/dehydratase family^11-148^E:3.1e-13`PF01073.16^3Beta_HSD^3-beta hydroxysteroid dehydrogenase/isomerase family^12-129^E:1.9e-05`PF07993.9^NAD_binding_4^Male sterility protein^12-43^E:0.00012`PF13460.3^NAD_binding_10^NAD(P)H-binding^14-130^E:2.4e-08	0,292	-0,348	0,953	0,035	0	-1,788	0	-1,575	0,226	1,97	GC1_ARATH	Epimerase family protein SDR39U1 homolog, chloroplastic (EC 1.1.1.-) (Protein GIANT CHLOROPLAST 1) (Protein SulA homolog) (AtSulA)	GC1 SULA At2g21280 F3K23	Arabidopsis thaliana (Mouse-ear cress)		Chloroplast; Complete proteome; Membrane; NADP; Oxidoreductase; Plastid; Plastid inner membrane; Reference proteome; Transit peptide
SRR342019_TR37934_c0_g1_i1_len=718	NA	NA	NA	NA	NA	.	1	0,089	0,028	-1,393	0,001	-1,718	0	-1,939	0,57	0,372	.	.	.	.	.	.
SRR342019_TR38704_c0_g2_i1_len=1958	COLX_ARATH	Q:299-1225,H:10-266	35.67%ID	E:2e-43	Putative zinc finger protein At1g68190	PF06203.11^CCT^CCT motif^435-476^E:6.1e-07	0,412	-0,555	0,014	-2,681	0	-3,077	0	-2,692	0,057	1,317	COLX_ARATH	Putative zinc finger protein At1g68190	At1g68190 T22E19.18	Arabidopsis thaliana (Mouse-ear cress)		Complete proteome; Metal-binding; Nucleus; Reference proteome; Repeat; Zinc; Zinc-finger
SRR342022_TR329_c1_g5_i1_len=2467	CHLH_ARATH	Q:1826-3,H:1-610	83.99%ID	E:0	Magnesium-chelatase subunit ChlH, chloroplastic	PF11965.5^DUF3479^Domain of unknown function (DUF3479)^78-238^E:3.5e-48`PF02514.13^CobN-Mg_chel^CobN/Magnesium Chelatase^242-607^E:2e-75	0,294	-0,35	0,635	-0,299	0	-1,61	0	-0,867	0,006	0,783	CHLH_ARATH	Magnesium-chelatase subunit ChlH, chloroplastic (Mg-chelatase subunit H) (EC 6.6.1.1) (ABA-binding protein) (Mg-protoporphyrin IX chelatase subunit ChlH) (Protein CONDITIONAL CHLORINA) (Protein GENOMES UNCOUPLED 5) (Protein RAPID TRANSPIRATION IN DETACHED LEAVES 1)	CHLH ABAR CCH GUN5 RTL1 At5g13630 MSH12.9	Arabidopsis thaliana (Mouse-ear cress)	PATHWAY: Porphyrin-containing compound metabolism; chlorophyll biosynthesis.	ATP-binding; Acetylation; Alternative splicing; Chlorophyll biosynthesis; Chloroplast; Complete proteome; Ligase; Membrane; Nucleotide-binding; Photosynthesis; Plastid; Reference proteome; Transit peptide
SRR342022_TR447_c1_g4_i1_len=1767	GDL6_ARATH	Q:467-1555,H:32-383	46.56%ID	E:1e-112	GDSL esterase/lipase At1g28570	PF00657.19^Lipase_GDSL^GDSL-like Lipase/Acylhydrolase^56-389^E:1.4e-09	0,352	-1,022	0,189	-1,858	0	-3,501	0	-2,514	0,564	0,428	GDL6_ARATH	GDSL esterase/lipase At1g28570 (EC 3.1.1.-) (Extracellular lipase At1g28570)	At1g28570 F1K23.19	Arabidopsis thaliana (Mouse-ear cress)		Alternative splicing; Complete proteome; Glycoprotein; Hydrolase; Lipid degradation; Lipid metabolism; Reference proteome; Secreted; Signal
SRR342022_TR1898_c4_g2_i3_len=1676	PSAB_SOLTU	Q:263-1585,H:294-734	99.32%ID	E:0	Photosystem I P700 chlorophyll a apoprotein A2 {ECO:0000255|HAMAP-Rule:MF_00482}	PF00223.16^PsaA_PsaB^Photosystem I psaA/psaB protein^1-427^E:1.6e-217	0,159	0,507	0	1,965	0	-2,292	0	-1,49	1	0	PSAB_SOLTU	Photosystem I P700 chlorophyll a apoprotein A2 (EC 1.97.1.12) (PSI-B) (PsaB)	psaB	Solanum tuberosum (Potato)		4Fe-4S; Chlorophyll; Chloroplast; Chromophore; Complete proteome; Electron transport; Iron; Iron-sulfur; Magnesium; Membrane; Metal-binding; Oxidoreductase; Photosynthesis; Photosystem I; Plastid; Reference proteome; Thylakoid; Transmembrane; Transmembrane helix; Transport
SRR342022_TR2673_c0_g4_i1_len=1502	EXO_ARATH	Q:416-1252,H:37-314	75.36%ID	E:2e-142	Protein EXORDIUM	PF04674.9^Phi_1^Phosphate-induced protein 1 conserved region^36-315^E:1.9e-124	0,056	-0,804	0,377	-0,558	0	-2,019	0	-1,899	1	-0,836	EXO_ARATH	Protein EXORDIUM	EXO At4g08950 T3H13.3	Arabidopsis thaliana (Mouse-ear cress)		Apoplast; Complete proteome; Reference proteome; Secreted; Signal
SRR342022_TR2829_c2_g1_i3_len=2541	G3PB_PEA	Q:450-1118,H:10-232	82.96%ID	E:3e-126	Glyceraldehyde-3-phosphate dehydrogenase B, chloroplastic	PF00044.21^Gp_dh_N^Glyceraldehyde 3-phosphate dehydrogenase, NAD binding domain^87-235^E:1.1e-48	1	0,022	0,102	-0,694	0	-1,271	0,001	-0,804	0,002	0,94	G3PB_PEA	Glyceraldehyde-3-phosphate dehydrogenase B, chloroplastic (EC 1.2.1.13) (NADP-dependent glyceraldehydephosphate dehydrogenase subunit B)	GAPB GPB1	Pisum sativum (Garden pea)	PATHWAY: Carbohydrate biosynthesis; Calvin cycle.	Calvin cycle; Chloroplast; NADP; Oxidoreductase; Plastid; Transit peptide
SRR342022_TR2908_c3_g3_i3_len=1976	C76B6_CATRO	Q:1754-336,H:21-493	100%ID	E:0	Geraniol 8-hydroxylase	PF00067.19^p450^Cytochrome P450^31-479^E:2e-106	0,389	0,204	0,001	-1,035	0	-1,069	0	-1,939	1	-0,214	C76B6_CATRO	Geraniol 8-hydroxylase (EC 1.14.13.152) (Cytochrome P450 76B6) (Geraniol 10-hydroxylase) (CrG10H)	CYP76B6 G10H	Catharanthus roseus (Madagascar periwinkle) (Vinca rosea)		Direct protein sequencing; Endoplasmic reticulum; Heme; Iron; Membrane; Metal-binding; Monooxygenase; NADP; Oxidoreductase; Transmembrane; Transmembrane helix
SRR342022_TR4265_c2_g1_i4_len=685	CB11_SOLLC	Q:116-685,H:1-190	83.68%ID	E:3e-102	Chlorophyll a-b binding protein 6A, chloroplastic	PF00504.18^Chloroa_b-bind^Chlorophyll A-B binding protein^84-217^E:4.9e-33	0,849	0,099	0,004	-1,672	0	-1,556	0	-1,277	0,001	0,69	CB11_SOLLC	Chlorophyll a-b binding protein 6A, chloroplastic (LHCI type I CAB-6A) (Light-harvesting complex I 26 kDa protein)	CAB6A	Solanum lycopersicum (Tomato) (Lycopersicon esculentum)		Chlorophyll; Chloroplast; Chromophore; Complete proteome; Magnesium; Membrane; Metal-binding; Phosphoprotein; Photosynthesis; Photosystem I; Photosystem II; Plastid; Reference proteome; Thylakoid; Transit peptide; Transmembrane; Transmembrane helix
SRR342022_TR4265_c2_g7_i2_len=824	CB121_HORVU	Q:140-3,H:195-240	95.65%ID	E:1e-22	Chlorophyll a-b binding protein 1B-21, chloroplastic	.	0,87	0,205	0,004	-1,84	0	-1,865	0	-2,104	0,545	0,42	CB121_HORVU	Chlorophyll a-b binding protein 1B-21, chloroplastic (LHCI type I CAB-1B-21) (LHCI-730 chlorophyll a/b binding protein) (Light-harvesting complex I 21 kDa protein)	LHC Ib-21 LHCA1	Hordeum vulgare (Barley)		Chlorophyll; Chloroplast; Chromophore; Direct protein sequencing; Magnesium; Membrane; Metal-binding; Phosphoprotein; Photosynthesis; Photosystem I; Photosystem II; Plastid; Thylakoid; Transit peptide; Transmembrane; Transmembrane helix
SRR342022_TR4326_c1_g2_i1_len=558	CCD4_ARATH	Q:297-557,H:73-160	65.91%ID	E:3e-32	Probable carotenoid cleavage dioxygenase 4, chloroplastic	PF03055.12^RPE65^Retinal pigment epithelial membrane protein^116-156^E:9.6e-07	0,01	1,932	0,007	1,154	0	-1,831	0,001	-1,208	0,525	-0,173	CCD4_ARATH	Probable carotenoid cleavage dioxygenase 4, chloroplastic (AtCCD4) (EC 1.14.99.-) (AtNCED4)	CCD4 NCED4 At4g19170 T18B16.140	Arabidopsis thaliana (Mouse-ear cress)		Chloroplast; Complete proteome; Dioxygenase; Iron; Metal-binding; Oxidoreductase; Plastid; Reference proteome; Transit peptide
SRR342022_TR4690_c0_g1_i4_len=2277	GLYM_SOLTU	Q:356-1909,H:1-518	90.56%ID	E:0	Serine hydroxymethyltransferase, mitochondrial	PF00464.16^SHMT^Serine hydroxymethyltransferase^58-454^E:1.2e-210	0,217	-0,322	0	9,911	0	-1,75	0	-0,886	0	1,298	GLYM_SOLTU	Serine hydroxymethyltransferase, mitochondrial (SHMT) (EC 2.1.2.1) (Glycine hydroxymethyltransferase) (Serine methylase)		Solanum tuberosum (Potato)	PATHWAY: One-carbon metabolism; tetrahydrofolate interconversion.	Complete proteome; Mitochondrion; One-carbon metabolism; Pyridoxal phosphate; Reference proteome; Transferase; Transit peptide
SRR342022_TR10862_c3_g1_i2_len=1988	PGLR4_ARATH	Q:379-1692,H:76-463	42.53%ID	E:4e-103	Polygalacturonase At1g48100	PF12708.4^Pectate_lyase_3^Pectate lyase superfamily protein^131-167^E:1e-06`PF00295.14^Glyco_hydro_28^Glycosyl hydrolases family 28^160-244^E:2.6e-09	0,865	-0,237	0,002	-6,291	0	-3,4	0	-4,621	1	1,653	PGLR4_ARATH	Polygalacturonase At1g48100 (PG) (EC 3.2.1.15) (Pectinase At1g48100)	At1g48100 F21D18.17 F21D18.18	Arabidopsis thaliana (Mouse-ear cress)		Cell wall; Cell wall biogenesis/degradation; Complete proteome; Glycosidase; Hydrolase; Reference proteome; Repeat; Secreted; Signal
SRR342022_TR10862_c3_g1_i3_len=2346	PGLR4_ARATH	Q:1505-2050,H:281-463	50.54%ID	E:6e-47	Polygalacturonase At1g48100	PF12708.4^Pectate_lyase_3^Pectate lyase superfamily protein^131-167^E:1.5e-06`PF00295.14^Glyco_hydro_28^Glycosyl hydrolases family 28^160-244^E:4.3e-09`PF00295.14^Glyco_hydro_28^Glycosyl hydrolases family 28^281-354^E:2.3e-13	1	0,02	0,226	-2,685	0	-3,299	0,001	-2,593	0,732	0,754	PGLR4_ARATH	Polygalacturonase At1g48100 (PG) (EC 3.2.1.15) (Pectinase At1g48100)	At1g48100 F21D18.17 F21D18.18	Arabidopsis thaliana (Mouse-ear cress)		Cell wall; Cell wall biogenesis/degradation; Complete proteome; Glycosidase; Hydrolase; Reference proteome; Repeat; Secreted; Signal
SRR342022_TR13754_c0_g1_i1_len=921	RAE1C_ARATH	Q:232-837,H:15-216	93.07%ID	E:6e-119	Ras-related protein RABE1c	PF00025.18^Arf^ADP-ribosylation factor family^16-163^E:2.1e-15`PF00071.19^Ras^Ras family^17-178^E:1.6e-66`PF08477.10^Roc^Ras of Complex, Roc, domain of DAPkinase^17-132^E:4.6e-35`PF04670.9^Gtr1_RagA^Gtr1/RagA G protein conserved region^17-132^E:4.5e-05`PF01926.20^MMR_HSR1^50S ribosome-binding GTPase^18-128^E:4.7e-05	0,854	0,13	0	6,797	0	-2,668	0	-7,493	0,007	1,084	RAE1C_ARATH	Ras-related protein RABE1c (AtRABE1c) (Ras-related protein Ara-3) (Ras-related protein Rab8A) (AtRab8A)	RABE1C ARA-3 RAB8A At3g46060 F12M12.30	Arabidopsis thaliana (Mouse-ear cress)		Cell membrane; Complete proteome; GTP-binding; Golgi apparatus; Lipoprotein; Membrane; Nucleotide-binding; Prenylation; Protein transport; Reference proteome; Transport
SRR342022_TR14005_c0_g2_i1_len=1354	NA	NA	NA	NA	NA	PF04520.10^Senescence_reg^Senescence regulator^32-213^E:1.9e-42	0,447	-0,378	0,489	-0,477	0	-1,575	0	-1,481	0,41	0,31	.	.	.	.	.	.
SRR342022_TR19437_c5_g1_i2_len=1557	BCH2_CAPAN	Q:471-1229,H:64-316	86.56%ID	E:2e-124	Beta-carotene hydroxylase 2, chloroplastic	PF04116.10^FA_hydroxylase^Fatty acid hydroxylase superfamily^157-284^E:6.8e-14	0,641	-0,256	0,012	-1,543	0	-1,64	0	-1,872	0,178	0,457	BCH2_CAPAN	Beta-carotene hydroxylase 2, chloroplastic (EC 1.14.13.129) (Fragment)	CA2	Capsicum annuum (Bell pepper)		Carotenoid biosynthesis; Chloroplast; Hydrolase; Iron; Membrane; Metal-binding; NAD; Oxidoreductase; Plastid; Transit peptide; Transmembrane; Transmembrane helix
SRR342022_TR19990_c0_g1_i2_len=936	NA	NA	NA	NA	NA	.	1	-0,168	0,016	-1,881	0	-1,675	0	-1,623	0,793	-0,191	.	.	.	.	.	.
SRR342022_TR21249_c2_g4_i4_len=2849	UBP25_ARATH	Q:2456-1359,H:5-361	70.49%ID	E:9e-178	Ubiquitin carboxyl-terminal hydrolase 25	PF13423.3^UCH_1^Ubiquitin carboxyl-terminal hydrolase^23-321^E:8.2e-20`PF00443.26^UCH^Ubiquitin carboxyl-terminal hydrolase^24-340^E:3.3e-50	0,471	-0,481	1	-0,175	0	-2,677	0	-2,042	1	0,747	UBP25_ARATH	Ubiquitin carboxyl-terminal hydrolase 25 (EC 3.4.19.12) (Deubiquitinating enzyme 25) (AtUBP25) (Ubiquitin thioesterase 25) (Ubiquitin-specific-processing protease 25)	UBP25 At3g14400 MLN21.18	Arabidopsis thaliana (Mouse-ear cress)		Complete proteome; Hydrolase; Protease; Reference proteome; Thiol protease; Ubl conjugation pathway
SRR342022_TR24311_c4_g3_i3_len=753	NA	NA	NA	NA	NA	.	0,632	-0,215	0,798	0,36	0,001	-1,366	0	-1,899	1	0	.	.	.	.	.	.
SRR342022_TR26255_c0_g3_i2_len=702	NA	NA	NA	NA	NA	.	0,065	-1,53	0,381	-1,655	0	-5,868	0	-8,664	0,001	2,178	.	.	.	.	.	.
SRR342022_TR28271_c1_g1_i2_len=6100	CLU_ARATH	Q:2492-3781,H:788-1258	38.87%ID	E:3e-89	Clustered mitochondria protein	PF15044.3^CLU_N^Mitochondrial function, CLU-N-term^49-120^E:6.3e-08`PF12807.4^eIF3_p135^Translation initiation factor eIF3 subunit 135^726-867^E:5.6e-25`PF13424.3^TPR_12^Tetratricopeptide repeat^907-968^E:2.2e-08`PF13424.3^TPR_12^Tetratricopeptide repeat^937-1007^E:5.5e-13`PF13374.3^TPR_10^Tetratricopeptide repeat^937-978^E:9.3e-09`PF13181.3^TPR_8^Tetratricopeptide repeat^938-969^E:0.11`PF13424.3^TPR_12^Tetratricopeptide repeat^1021-1095^E:1e-10`PF13374.3^TPR_10^Tetratricopeptide repeat^1021-1060^E:1e-06	1	-0,76	0,002	-4,771	0	-1,203	0,001	-1,006	1	-0,204	CLU_ARATH	Clustered mitochondria protein (Friendly mitochondria protein)	FMT CLU At3g52140 F4F15.250	Arabidopsis thaliana (Mouse-ear cress)		Alternative splicing; Complete proteome; Cytoplasm; Reference proteome; Repeat; TPR repeat
SRR342022_TR32097_c0_g1_i2_len=873	NA	NA	NA	NA	NA	.	0,439	-0,272	0,011	-1,002	0	-1,507	0	-2,007	0,485	-0,204	.	.	.	.	.	.
SRR342022_TR33106_c0_g1_i1_len=1817	RUP2_ARATH	Q:369-1409,H:21-364	59.71%ID	E:5e-122	WD repeat-containing protein RUP2	PF00400.29^WD40^WD domain, G-beta repeat^177-209^E:0.17`PF00400.29^WD40^WD domain, G-beta repeat^303-342^E:0.043	0,5	-0,537	0,103	-1,618	0	-1,957	0	-1,784	0,458	-0,432	RUP2_ARATH	WD repeat-containing protein RUP2 (Protein EARLY FLOWERING BY OVEREXPRESSION 2) (Protein EPRESSOR OF UV-B PHOTOMORPHOGENESIS 2)	RUP2 EFO2 At5g23730 MRO11.23	Arabidopsis thaliana (Mouse-ear cress)		Complete proteome; Cytoplasm; Flowering; Nucleus; Reference proteome; Repeat; WD repeat
SRR342022_TR33307_c1_g2_i3_len=1548	GPPL1_ARATH	Q:242-1060,H:47-315	80.66%ID	E:3e-155	Haloacid dehalogenase-like hydrolase domain-containing protein At4g39970	PF00702.23^Hydrolase^haloacid dehalogenase-like hydrolase^72-284^E:1.1e-13`PF13419.3^HAD_2^Haloacid dehalogenase-like hydrolase^74-288^E:1.1e-22`PF13242.3^Hydrolase_like^HAD-hyrolase-like^247-310^E:3.8e-05	0,878	-0,052	0,586	-0,151	0	-1,366	0	-1,033	0,312	-0,29	GPPL1_ARATH	Haloacid dehalogenase-like hydrolase domain-containing protein At4g39970 (EC 3.1.3.-)	At4g39970 T5J17.140	Arabidopsis thaliana (Mouse-ear cress)		Chloroplast; Complete proteome; Hydrolase; Magnesium; Metal-binding; Plastid; Reference proteome; Transit peptide
SRR342023_TR926_c5_g4_i1_len=2079	FAD3C_SESIN	Q:143-1489,H:1-446	80.04%ID	E:0	Omega-3 fatty acid desaturase, chloroplastic	PF11960.5^DUF3474^Domain of unknown function (DUF3474)^1-140^E:1.5e-55`PF00487.21^FA_desaturase^Fatty acid desaturase^148-402^E:1.3e-32	0,299	-0,329	0,633	-0,163	0	-2,066	0	-1,37	0,889	-0,045	FAD3C_SESIN	Omega-3 fatty acid desaturase, chloroplastic (EC 1.14.19.-)	FAD7	Sesamum indicum (Oriental sesame) (Sesamum orientale)	PATHWAY: Lipid metabolism; polyunsaturated fatty acid biosynthesis.	Chloroplast; Fatty acid biosynthesis; Fatty acid metabolism; Lipid biosynthesis; Lipid metabolism; Membrane; Oxidoreductase; Plastid; Transit peptide
SRR342023_TR1188_c3_g15_i2_len=743	TRXM4_ARATH	Q:401-42,H:70-191	59.84%ID	E:7e-47	Thioredoxin M4, chloroplastic	PF00085.17^Thioredoxin^Thioredoxin^82-181^E:2.2e-28	0,759	-0,084	0,203	0,342	0	-2,294	0	-2,275	0,002	6,33	TRXM4_ARATH	Thioredoxin M4, chloroplastic (AtTrxm4)	At3g15360 MJK13.2	Arabidopsis thaliana (Mouse-ear cress)		Chloroplast; Complete proteome; Disulfide bond; Electron transport; Plastid; Redox-active center; Reference proteome; Transit peptide; Transport
SRR342023_TR1754_c1_g5_i1_len=877	AB19I_ARATH	Q:772-47,H:51-290	79.34%ID	E:1e-124	ABC transporter I family member 19	.	0,233	-0,738	1	0	0	-6,75	0	-6,75	0,233	-0,806	AB19I_ARATH	ABC transporter I family member 19 (ABC transporter ABCI.19) (AtABCI19) (GCN-related protein 2) (Non-intrinsic ABC protein 4)	ABCI19 NAP4 At1g03905 F21M11.17	Arabidopsis thaliana (Mouse-ear cress)		ATP-binding; Complete proteome; Cytoplasm; Nucleotide-binding; Reference proteome; Transport
SRR342023_TR3118_c2_g10_i4_len=2329	GNOM_ARATH	Q:1242-1454,H:176-246	49.3%ID	E:4e-23	ARF guanine-nucleotide exchange factor GNOM	.	0,811	-0,29	0,065	-1,386	0	-1,79	0	-2,043	1	-0,011	GNOM_ARATH	ARF guanine-nucleotide exchange factor GNOM (Pattern formation protein EMB30) (Protein EMBRYO DEFECTIVE 30) (Protein MIZU-KUSSEI2) (Protein VASCULAR NETWORK 7)	GN EMB30 GBF3 MIZ2 VAN7 At1g13980 F16A14.20 F7A19.7	Arabidopsis thaliana (Mouse-ear cress)		Cell adhesion; Cell membrane; Complete proteome; Cytoplasm; ER-Golgi transport; Endocytosis; Endosome; Guanine-nucleotide releasing factor; Membrane; Polymorphism; Protein transport; Reference proteome; Transport
SRR342023_TR9594_c0_g1_i4_len=1536	GDL34_ARATH	Q:308-1267,H:25-342	65.62%ID	E:2e-157	GDSL esterase/lipase At2g04570	PF00657.19^Lipase_GDSL^GDSL-like Lipase/Acylhydrolase^30-343^E:1.9e-16	0,426	-0,237	0,199	-0,435	0,001	-0,831	0	-0,838	0,055	0,484	GDL34_ARATH	GDSL esterase/lipase At2g04570 (EC 3.1.1.-) (Extracellular lipase At2g04570)	At2g04570 T1O3.2	Arabidopsis thaliana (Mouse-ear cress)		Complete proteome; Glycoprotein; Hydrolase; Lipid degradation; Lipid metabolism; Reference proteome; Secreted; Signal
SRR342023_TR12794_c5_g10_i10_len=5615	GLTB1_ARATH	Q:433-4071,H:66-1281	85.94%ID	E:0	Ferredoxin-dependent glutamate synthase 1, chloroplastic/mitochondrial	PF00310.18^GATase_2^Glutamine amidotransferases class-II^103-525^E:6e-180`PF04898.11^Glu_syn_central^Glutamate synthase central domain^551-844^E:8.4e-107`PF01645.14^Glu_synthase^Conserved region in glutamate synthase^904-1280^E:1.1e-157	0,591	-0,206	0,234	-0,463	0	-1,327	0,001	-0,982	0,326	0,258	GLTB1_ARATH	Ferredoxin-dependent glutamate synthase 1, chloroplastic/mitochondrial (EC 1.4.7.1) (Fd-GOGAT 1)	GLU1 GLS1 At5g04140 F21E1_60	Arabidopsis thaliana (Mouse-ear cress)	PATHWAY: Amino-acid biosynthesis; L-glutamate biosynthesis via GLT pathway; L-glutamate from 2-oxoglutarate and L-glutamine (ferredoxin route): step 1/1.; PATHWAY: Energy metabolism; nitrogen metabolism.	3Fe-4S; Alternative splicing; Amino-acid biosynthesis; Chloroplast; Complete proteome; FAD; FMN; Flavoprotein; Glutamate biosynthesis; Glutamine amidotransferase; Iron; Iron-sulfur; Metal-binding; Mitochondrion; Oxidoreductase; Plastid; Reference proteome; Transit peptide
SRR342023_TR15684_c0_g1_i1_len=1648	COL16_ARATH	Q:224-1402,H:6-403	37.1%ID	E:9e-57	Zinc finger protein CONSTANS-LIKE 16	PF00643.21^zf-B_box^B-box zinc finger^17-61^E:5.7e-08`PF06203.11^CCT^CCT motif^359-401^E:3.9e-18	0,383	0,26	0,092	-0,523	0	-1,496	0	-2,192	0,013	0,695	COL16_ARATH	Zinc finger protein CONSTANS-LIKE 16	COL16 At1g25440 F2J7.10	Arabidopsis thaliana (Mouse-ear cress)		Coiled coil; Complete proteome; Metal-binding; Nucleus; Reference proteome; Zinc; Zinc-finger
SRR342023_TR17850_c0_g1_i3_len=700	CB23_SOLLC	Q:429-1,H:18-160	91.61%ID	E:4e-92	Chlorophyll a-b binding protein 13, chloroplastic	PF00504.18^Chloroa_b-bind^Chlorophyll A-B binding protein^65-158^E:6.1e-24	0,166	-0,305	0,605	0,116	0	-1,356	0	-1,021	0,811	-0,269	CB23_SOLLC	Chlorophyll a-b binding protein 13, chloroplastic (LHCII type III CAB-13)	CAB13 LHBC1	Solanum lycopersicum (Tomato) (Lycopersicon esculentum)		Chlorophyll; Chloroplast; Chromophore; Complete proteome; Direct protein sequencing; Magnesium; Membrane; Metal-binding; Phosphoprotein; Photosynthesis; Photosystem I; Photosystem II; Plastid; Reference proteome; Thylakoid; Transit peptide; Transmembrane; Transmembrane helix
SRR342023_TR18286_c0_g1_i1_len=1077	CYB5_BOROF	Q:353-571,H:3-75	58.9%ID	E:4e-24	Cytochrome b5	PF00173.25^Cyt-b5^Cytochrome b5-like Heme/Steroid binding domain^7-78^E:2.4e-23	0,216	0,405	0,164	0,616	0	-1,476	0	-1,641	0,429	0,494	CYB5_BOROF	Cytochrome b5		Borago officinalis (Bourrache) (Borage)		Electron transport; Endoplasmic reticulum; Heme; Iron; Membrane; Metal-binding; Microsome; Transmembrane; Transmembrane helix; Transport
SRR342023_TR22522_c0_g1_i2_len=2281	FATB_GOSHI	Q:1735-482,H:1-413	68.38%ID	E:0	Palmitoyl-acyl carrier protein thioesterase, chloroplastic	PF12590.5^Acyl-thio_N^Acyl-ATP thioesterase^1-125^E:8.1e-50`PF01643.14^Acyl-ACP_TE^Acyl-ACP thioesterase^136-400^E:8e-82	0,127	-0,477	0,541	0,274	0	-1,079	0	-1,036	1	1,134	FATB_GOSHI	Palmitoyl-acyl carrier protein thioesterase, chloroplastic (EC 3.1.2.-) (16:0-acyl-carrier protein thioesterase) (16:0-ACP thioesterase) (Acyl-[acyl-carrier-protein] hydrolase) (PATE)	FATB1	Gossypium hirsutum (Upland cotton) (Gossypium mexicanum)		Chloroplast; Fatty acid biosynthesis; Fatty acid metabolism; Hydrolase; Lipid biosynthesis; Lipid metabolism; Plastid; Transit peptide
SRR342023_TR28303_c0_g2_i4_len=526	NA	NA	NA	NA	NA	.	0,194	-0,354	0,003	-1,271	0	-2,853	0	-2,203	1	0,179	.	.	.	.	.	.
SRR342023_TR31426_c6_g2_i1_len=2588	DXS2_ORYSJ	Q:2411-288,H:1-712	79.35%ID	E:0	Probable 1-deoxy-D-xylulose-5-phosphate synthase 2, chloroplastic	PF13292.3^DXP_synthase_N^1-deoxy-D-xylulose-5-phosphate synthase^74-359^E:4.1e-112`PF00676.17^E1_dh^Dehydrogenase E1 component^172-245^E:2e-07`PF02779.21^Transket_pyr^Transketolase, pyrimidine binding domain^396-557^E:8.3e-43`PF02780.17^Transketolase_C^Transketolase, C-terminal domain^575-698^E:5.9e-31	0,291	0,265	0,35	-0,527	0	-1,536	0	-3,568	0,095	0,66	DXS2_ORYSJ	Probable 1-deoxy-D-xylulose-5-phosphate synthase 2, chloroplastic (1-deoxyxylulose-5-phosphate synthase) (DXP synthase) (DXPS) (EC 2.2.1.7)	Os07g0190000 LOC_Os07g09190 OsJ_23394 OSJNBb0002L09.31 OSJNBb0003E08.7	Oryza sativa subsp. japonica (Rice)	PATHWAY: Metabolic intermediate biosynthesis; 1-deoxy-D-xylulose 5-phosphate biosynthesis; 1-deoxy-D-xylulose 5-phosphate from D-glyceraldehyde 3-phosphate and pyruvate: step 1/1.	Chloroplast; Complete proteome; Isoprene biosynthesis; Magnesium; Metal-binding; Plastid; Reference proteome; Thiamine biosynthesis; Thiamine pyrophosphate; Transferase; Transit peptide
SRR646572_TR7801_c0_g2_i2_len=5549	GLTB1_ARATH	Q:5072-411,H:66-1622	84.97%ID	E:0	Ferredoxin-dependent glutamate synthase 1, chloroplastic/mitochondrial	PF00310.18^GATase_2^Glutamine amidotransferases class-II^103-525^E:9.3e-180`PF04898.11^Glu_syn_central^Glutamate synthase central domain^551-844^E:1.2e-106`PF01645.14^Glu_synthase^Conserved region in glutamate synthase^904-1287^E:3.6e-160`PF01493.16^GXGXG^GXGXG motif^1369-1550^E:3.7e-75	0,73	-0,104	0,737	-0,116	0	-1,388	0	-1,091	0,87	0,169	GLTB1_ARATH	Ferredoxin-dependent glutamate synthase 1, chloroplastic/mitochondrial (EC 1.4.7.1) (Fd-GOGAT 1)	GLU1 GLS1 At5g04140 F21E1_60	Arabidopsis thaliana (Mouse-ear cress)	PATHWAY: Amino-acid biosynthesis; L-glutamate biosynthesis via GLT pathway; L-glutamate from 2-oxoglutarate and L-glutamine (ferredoxin route): step 1/1.; PATHWAY: Energy metabolism; nitrogen metabolism.	3Fe-4S; Alternative splicing; Amino-acid biosynthesis; Chloroplast; Complete proteome; FAD; FMN; Flavoprotein; Glutamate biosynthesis; Glutamine amidotransferase; Iron; Iron-sulfur; Metal-binding; Mitochondrion; Oxidoreductase; Plastid; Reference proteome; Transit peptide
SRR646572_TR13586_c0_g1_i1_len=1078	EXPA8_ARATH	Q:956-261,H:20-252	84.55%ID	E:8e-143	Expansin-A8	PF03330.15^DPBB_1^Lytic transglycolase^86-171^E:1.4e-21`PF01357.18^Pollen_allerg_1^Pollen allergen^182-259^E:1.2e-29	0,298	0,637	0	2,421	0	-3,456	0	-1,555	0,56	-0,749	EXPA8_ARATH	Expansin-A8 (AtEXPA8) (Alpha-expansin-8) (At-EXP8) (AtEx8) (Ath-ExpAlpha-1.11)	EXPA8 EXP8 At2g40610 T2P4.4	Arabidopsis thaliana (Mouse-ear cress)		Cell wall; Cell wall biogenesis/degradation; Complete proteome; Disulfide bond; Membrane; Reference proteome; Secreted; Signal

SRR646572_TR18050_c0_g2_i2_len=1105	CB5_ARATH	Q:461-3,H:93-245	90.85%ID	E:4e-82	Chlorophyll a-b binding protein CP26, chloroplastic	.	0,091	-0,413	0,002	-0,798	0	-1,023	0,001	-0,75	0,861	0,068	CB5_ARATH	Chlorophyll a-b binding protein CP26, chloroplastic (LHCB5) (LHCIIc) (Light-harvesting complex II protein 5)	LHCB5 At4g10340 F24G24.140	Arabidopsis thaliana (Mouse-ear cress)		Chlorophyll; Chloroplast; Chromophore; Complete proteome; Magnesium; Membrane; Metal-binding; Phosphoprotein; Photosynthesis; Photosystem I; Photosystem II; Plastid; Reference proteome; Thylakoid; Transit peptide; Transmembrane; Transmembrane helix
SRR646572_TR19460_c0_g1_i1_len=711	RBS_MALSP	Q:462-229,H:106-183	87.18%ID	E:1e-44	Ribulose bisphosphate carboxylase small chain, chloroplastic	.	0,865	0,172	0,008	-1,272	0	-2,239	0	-1,748	0,663	0,4	RBS_MALSP	Ribulose bisphosphate carboxylase small chain, chloroplastic (RuBisCO small subunit) (EC 4.1.1.39)	RBCS	Malus sp. (Crab apple)		Calvin cycle; Carbon dioxide fixation; Chloroplast; Lyase; Monooxygenase; Oxidoreductase; Photorespiration; Photosynthesis; Plastid; Transit peptide
SRR646572_TR27490_c0_g1_i1_len=1330	BCA1_ARATH	Q:327-1202,H:53-347	78.64%ID	E:3e-157	Beta carbonic anhydrase 1, chloroplastic {ECO:0000303|PubMed:17407539}	PF00484.16^Pro_CA^Carbonic anhydrase^83-240^E:1.5e-39	0,381	2,455	0,798	0,431	0	-2,539	0	-2,089	1	0,018	BCA1_ARATH	Beta carbonic anhydrase 1, chloroplastic (AtbCA1) (AtbetaCA1) (EC 4.2.1.1) (Beta carbonate dehydratase 1) (Protein SALICYLIC ACID-BINDING PROTEIN 3) (AtSABP3)	BCA1 CA1 SABP3 At3g01500 F4P13.5	Arabidopsis thaliana (Mouse-ear cress)		Acetylation; Alternative splicing; Cell membrane; Chloroplast; Complete proteome; Lyase; Membrane; Phosphoprotein; Plastid; Reference proteome; S-nitrosylation; Transit peptide; Zinc
SRR646572_TR29313_c0_g2_i4_len=2971	RUS5_ARATH	Q:376-1140,H:61-304	61.71%ID	E:7e-82	Protein root UVB sensitive 5 {ECO:0000303|PubMed:19515790}	PF04884.11^DUF647^Vitamin B6 photo-protection and homoeostasis^115-201^E:3.1e-23	0,443	-0,425	0,022	-1,387	0	-2,158	0	-2,008	0,892	-0,077	RUS5_ARATH	Protein root UVB sensitive 5	RUS5 At5g01510 F7A7.30	Arabidopsis thaliana (Mouse-ear cress)		Complete proteome; Reference proteome
SRR646572_TR29450_c0_g1_i1_len=531	NA	NA	NA	NA	NA	.	1	-0,012	0,002	-1,043	0	-1,34	0	-1,024	0,535	-0,208	.	.	.	.	.	.
SRR646572_TR34084_c2_g1_i1_len=1480	ALFC2_ARATH	Q:170-1324,H:12-397	86.27%ID	E:0	Probable fructose-bisphosphate aldolase 2, chloroplastic	PF00274.16^Glycolytic^Fructose-bisphosphate aldolase class-I^98-442^E:7.2e-157	1	0,04	0,009	1,072	0	-0,928	0	-0,981	0,984	-0,014	ALFC2_ARATH	Probable fructose-bisphosphate aldolase 2, chloroplastic (EC 4.1.2.13)	FBA2 At4g38970 F19H22.70	Arabidopsis thaliana (Mouse-ear cress)	PATHWAY: Carbohydrate degradation; glycolysis; D-glyceraldehyde 3-phosphate and glycerone phosphate from D-glucose: step 4/4.	Alternative splicing; Chloroplast; Complete proteome; Glycolysis; Lyase; Methylation; Phosphoprotein; Plastid; Reference proteome; Schiff base; Transit peptide
SRR646596_TR3897_c1_g3_i2_len=581	NA	NA	NA	NA	NA	.	1	1,121	0,044	1,121	0	-6,664	0	-6,664	1	-0,695	.	.	.	.	.	.
SRR646596_TR10573_c0_g2_i1_len=775	ERG16_ARATH	Q:427-38,H:69-198	80.77%ID	E:2e-68	Squalene epoxidase 3	PF01494.16^FAD_binding_3^FAD binding domain^102-248^E:2e-06	0,377	-0,561	1	-3,4	0	-2,122	0,001	-1,95	1	0,56	ERG16_ARATH	Squalene epoxidase 3 (AtSQE3) (EC 1.14.13.132)	SQE3 At4g37760 T28I19.4	Arabidopsis thaliana (Mouse-ear cress)	PATHWAY: Terpene metabolism; lanosterol biosynthesis; lanosterol from farnesyl diphosphate: step 2/3.	Complete proteome; FAD; Flavoprotein; Membrane; NADP; Oxidoreductase; Reference proteome; Transmembrane; Transmembrane helix
SRR646596_TR19878_c0_g1_i1_len=1367	PSBP_SPIOL	Q:366-1181,H:1-267	76.36%ID	E:7e-142	Oxygen-evolving enhancer protein 2, chloroplastic	PF01789.13^PsbP^PsbP^82-271^E:6.5e-41	0,614	-0,105	0,492	-0,143	0	-0,889	0	-0,767	0,003	0,614	PSBP_SPIOL	Oxygen-evolving enhancer protein 2, chloroplastic (OEE2) (23 kDa subunit of oxygen evolving system of photosystem II) (23 kDa thylakoid membrane protein) (OEC 23 kDa subunit)	PSBP	Spinacia oleracea (Spinach)		3D-structure; Chloroplast; Direct protein sequencing; Membrane; Photosynthesis; Photosystem II; Plastid; Thylakoid; Transit peptide
SRR646604_TR2665_c0_g1_i1_len=528	NA	NA	NA	NA	NA	.	1	0	1	-1,059	0,001	-2,618	0	-3,443	1	-3,232	.	.	.	.	.	.
SRR646604_TR5745_c0_g1_i1_len=2839	ROGF8_ARATH	Q:1698-889,H:175-412	58.03%ID	E:2e-117	Rho guanine nucleotide exchange factor 8	PF03759.10^PRONE^PRONE (Plant-specific Rop nucleotide exchanger)^1-24^E:4.1e-06`PF03759.10^PRONE^PRONE (Plant-specific Rop nucleotide exchanger)^55-262^E:3.7e-84	1	-0,52	1	-1,128	0	-2,463	0	-2,657	1	0,931	ROGF8_ARATH	Rho guanine nucleotide exchange factor 8 (AtRopGEF8) (Rho of plants guanine nucleotide exchange factor 8)	ROPGEF8 At3g24620 MOB24.21	Arabidopsis thaliana (Mouse-ear cress)		3D-structure; Cell membrane; Complete proteome; Guanine-nucleotide releasing factor; Membrane; Reference proteome
SRR646604_TR7503_c0_g2_i1_len=1569	CP41B_ARATH	Q:197-1213,H:1-378	76.46%ID	E:0	Chloroplast stem-loop binding protein of 41 kDa b, chloroplastic	PF01370.18^Epimerase^NAD dependent epimerase/dehydratase family^56-251^E:2.5e-07`PF13460.3^NAD_binding_10^NAD(P)H-binding^60-212^E:7.6e-09	1	0,003	0,541	0,174	0	-1,053	0	-0,838	0,014	0,683	CP41B_ARATH	Chloroplast stem-loop binding protein of 41 kDa b, chloroplastic (CSP41-b) (Heteroglycan-interacting protein 1.3) (Protein CHLOROPLAST RNA BINDING) (Protein Gb5f)	CSP41B CRB HIP1.3 At1g09340 T31J12.6	Arabidopsis thaliana (Mouse-ear cress)		Carbohydrate metabolism; Chloroplast; Complete proteome; Cytoplasm; Phosphoprotein; Plastid; Polysaccharide degradation; RNA-binding; Reference proteome; Transit peptide; rRNA processing; rRNA-binding
SRR646604_TR18421_c0_g1_i7_len=3370	NA	NA	NA	NA	NA	.	0,502	3,976	0,189	1,771	0	-1,927	0	-4,999	0,395	-0,6	.	.	.	.	.	.
SRR646604_TR20460_c0_g2_i1_len=2073	U83A1_ARATH	Q:488-1855,H:9-462	47.85%ID	E:2e-150	UDP-glycosyltransferase 83A1	PF00201.15^UDPGT^UDP-glucoronosyl and UDP-glucosyl transferase^268-427^E:1.1e-18	1	-0,137	1	0,163	0	-2	0	-1,789	0,76	0,55	U83A1_ARATH	UDP-glycosyltransferase 83A1 (EC 2.4.1.-)	UGT83A1 At3g02100 F1C9.11	Arabidopsis thaliana (Mouse-ear cress)		Complete proteome; Glycosyltransferase; Reference proteome; Transferase
SRR646604_TR21542_c0_g2_i1_len=1070	CUT1A_ARATH	Q:299-763,H:13-164	66.45%ID	E:1e-58	Protein CURVATURE THYLAKOID 1A, chloroplastic	PF14159.3^CAAD^CAAD domains of cyanobacterial aminoacyl-tRNA synthetase^85-168^E:3.4e-32	0,395	-0,2	0,089	-0,386	0	-1,157	0	-0,976	0,988	0,004	CUT1A_ARATH	Protein CURVATURE THYLAKOID 1A, chloroplastic	CURT1A At4g01150 A_IG002N01.18 F2N1.18	Arabidopsis thaliana (Mouse-ear cress)		Acetylation; Alternative splicing; Chloroplast; Coiled coil; Complete proteome; Membrane; Plastid; Reference proteome; Thylakoid; Transit peptide; Transmembrane; Transmembrane helix
SRR646604_TR22841_c0_g3_i2_len=2346	NA	NA	NA	NA	NA	PF06813.10^Nodulin-like^Nodulin-like^8-266^E:6.1e-60`PF07690.13^MFS_1^Major Facilitator Superfamily^331-524^E:1.4e-09	1	-0,135	0,461	-1,53	0	-2,147	0	-1,792	1	-0,056	.	.	.	.	.	.
SRR646604_TR23835_c0_g1_i1_len=1385	NA	NA	NA	NA	NA	PF02536.11^mTERF^mTERF^75-175^E:3.7e-13`PF02536.11^mTERF^mTERF^185-293^E:7.3e-30	0,395	-0,617	0,035	-1,592	0,001	-0,924	0	-1,11	0,043	0,954	.	.	.	.	.	.
SRR646604_TR32441_c0_g1_i18_len=2560	TPT_SPIOL	Q:2230-1166,H:88-399	78.59%ID	E:0	Triose phosphate/phosphate translocator, chloroplastic	PF03151.13^TPT^Triose-phosphate Transporter family^1-165^E:6e-68	0,254	4,468	0,009	-6,042	0	-7,305	0	-7,305	1	0	TPT_SPIOL	Triose phosphate/phosphate translocator, chloroplastic (cTPT) (E29) (p36)		Spinacia oleracea (Spinach)		Chloroplast; Direct protein sequencing; Membrane; Plastid; Transit peptide; Transmembrane; Transmembrane helix; Transport
SRR646604_TR32633_c0_g2_i1_len=828	GAT22_ARATH	Q:257-610,H:117-230	40.3%ID	E:2e-21	Putative GATA transcription factor 22	PF00320.24^GATA^GATA zinc finger^174-207^E:9e-17	0,447	-0,293	0,071	-0,923	0	-2,64	0	-3,155	0,005	-1,079	GAT22_ARATH	Putative GATA transcription factor 22	GATA22 At4g26150 F20B18.260	Arabidopsis thaliana (Mouse-ear cress)		Complete proteome; DNA-binding; Metal-binding; Nucleus; Reference proteome; Transcription; Transcription regulation; Zinc; Zinc-finger
SRR646604_TR34940_c0_g3_i1_len=1403	PSBO_SOLTU	Q:212-1210,H:1-332	88.59%ID	E:0	Oxygen-evolving enhancer protein 1, chloroplastic	PF01716.15^MSP^Manganese-stabilising protein / photosystem II polypeptide^100-332^E:9.6e-99	0,308	-0,216	0,003	-0,625	0	-0,996	0,001	-0,704	0,037	0,436	PSBO_SOLTU	Oxygen-evolving enhancer protein 1, chloroplastic (OEE1) (33 kDa subunit of oxygen evolving system of photosystem II) (33 kDa thylakoid membrane protein) (OEC 33 kDa subunit)	PSBO	Solanum tuberosum (Potato)		Chloroplast; Complete proteome; Manganese; Membrane; Photosynthesis; Photosystem II; Plastid; Reference proteome; Thylakoid; Transit peptide
SRR646604_TR38081_c0_g3_i1_len=1533	ALFC_ORYSJ	Q:282-1292,H:50-386	91.1%ID	E:0	Fructose-bisphosphate aldolase, chloroplastic	PF00274.16^Glycolytic^Fructose-bisphosphate aldolase class-I^13-351^E:1.4e-154	1	0,542	0,254	-4,808	0	-1,582	0	-2,808	1	0,618	ALFC_ORYSJ	Fructose-bisphosphate aldolase, chloroplastic (ALDP) (EC 4.1.2.13)	Os11g0171300 LOC_Os11g07020 OsJ_33121	Oryza sativa subsp. japonica (Rice)	PATHWAY: Carbohydrate degradation; glycolysis; D-glyceraldehyde 3-phosphate and glycerone phosphate from D-glucose: step 4/4.	Chloroplast; Complete proteome; Direct protein sequencing; Glycolysis; Lyase; Plastid; Reference proteome; Schiff base; Transit peptide
SRR646604_TR38541_c1_g1_i8_len=1674	CB5_ARATH	Q:155-3,H:195-245	94.12%ID	E:2e-29	Chlorophyll a-b binding protein CP26, chloroplastic	.	1	0,189	0,017	-2,249	0	-2,064	0	-2,479	1	0,09	CB5_ARATH	Chlorophyll a-b binding protein CP26, chloroplastic (LHCB5) (LHCIIc) (Light-harvesting complex II protein 5)	LHCB5 At4g10340 F24G24.140	Arabidopsis thaliana (Mouse-ear cress)		Chlorophyll; Chloroplast; Chromophore; Complete proteome; Magnesium; Membrane; Metal-binding; Phosphoprotein; Photosynthesis; Photosystem I; Photosystem II; Plastid; Reference proteome; Thylakoid; Transit peptide; Transmembrane; Transmembrane helix
SRR648705_TR269_c3_g5_i8_len=2682	GNOM_ARATH	Q:1242-1454,H:176-246	49.3%ID	E:5e-23	ARF guanine-nucleotide exchange factor GNOM	.	1	-0,061	0,019	-1,608	0	-1,628	0	-1,912	0,848	0,142	GNOM_ARATH	ARF guanine-nucleotide exchange factor GNOM (Pattern formation protein EMB30) (Protein EMBRYO DEFECTIVE 30) (Protein MIZU-KUSSEI2) (Protein VASCULAR NETWORK 7)	GN EMB30 GBF3 MIZ2 VAN7 At1g13980 F16A14.20 F7A19.7	Arabidopsis thaliana (Mouse-ear cress)		Cell adhesion; Cell membrane; Complete proteome; Cytoplasm; ER-Golgi transport; Endocytosis; Endosome; Guanine-nucleotide releasing factor; Membrane; Polymorphism; Protein transport; Reference proteome; Transport
SRR648705_TR8110_c0_g1_i1_len=1031	IAA17_ARATH	Q:245-820,H:3-218	51.15%ID	E:1e-59	Auxin-responsive protein IAA17	PF02309.13^AUX_IAA^AUX/IAA family^31-202^E:6.3e-59	1	0,038	0	7,058	0	-7,593	0	-9,345	0,786	0,1	IAA17_ARATH	Auxin-responsive protein IAA17 (Auxin response 3) (Indoleacetic acid-induced protein 17)	IAA17 AXR3 At1g04250 F19P19.31	Arabidopsis thaliana (Mouse-ear cress)		3D-structure; Auxin signaling pathway; Complete proteome; Nucleus; Phosphoprotein; Reference proteome; Repressor; Transcription; Transcription regulation
SRR648705_TR10096_c0_g1_i1_len=2966	FRO7_ARATH	Q:2407-281,H:32-747	61.2%ID	E:0	Ferric reduction oxidase 7, chloroplastic	PF01794.16^Ferric_reduct^Ferric reductase like transmembrane component^190-311^E:1.1e-18`PF08022.9^FAD_binding_8^FAD-binding domain^347-453^E:3.6e-16`PF08030.9^NAD_binding_6^Ferric reductase NAD binding domain^460-719^E:2.5e-18	1	0,016	0,043	-2,95	0	-1,039	0	-1,026	0,24	0,528	FRO7_ARATH	Ferric reduction oxidase 7, chloroplastic (AtFRO7) (EC 1.16.1.7) (Ferric-chelate reductase 7)	FRO7 At5g49740 K2I5.10	Arabidopsis thaliana (Mouse-ear cress)		Chloroplast; Complete proteome; Electron transport; FAD; Flavoprotein; Ion transport; Iron; Membrane; Metal-binding; NAD; Oxidoreductase; Plastid; Reference proteome; Transit peptide; Transmembrane; Transmembrane helix; Transport
SRR648705_TR11407_c0_g1_i1_len=1223	U83A1_ARATH	Q:3-902,H:168-460	48.03%ID	E:9e-96	UDP-glycosyltransferase 83A1	PF00201.15^UDPGT^UDP-glucoronosyl and UDP-glucosyl transferase^115-277^E:6.9e-24	1	0,6	1	-1,14	0	-2,972	0	-3,154	1	2,791	U83A1_ARATH	UDP-glycosyltransferase 83A1 (EC 2.4.1.-)	UGT83A1 At3g02100 F1C9.11	Arabidopsis thaliana (Mouse-ear cress)		Complete proteome; Glycosyltransferase; Reference proteome; Transferase
SRR648705_TR12859_c0_g1_i1_len=1421	LBD39_ARATH	Q:301-636,H:1-112	82.14%ID	E:3e-58	LOB domain-containing protein 39	PF03195.11^DUF260^Protein of unknown function DUF260^2-100^E:9.5e-27	0,298	-0,678	0,493	-0,364	0	-2,319	0	-2,292	0,419	0,437	LBD39_ARATH	LOB domain-containing protein 39 (ASYMMETRIC LEAVES 2-like protein 41) (AS2-like protein 41)	LBD39 ASL41 At4g37540 F19F18.30	Arabidopsis thaliana (Mouse-ear cress)		Complete proteome; Reference proteome
SRR648705_TR13461_c0_g1_i1_len=1371	LBD37_ARATH	Q:1125-472,H:1-219	56.5%ID	E:3e-73	LOB domain-containing protein 37	PF03195.11^DUF260^Protein of unknown function DUF260^2-101^E:5.8e-26	0,865	-0,145	0,03	1,068	0	-1,492	0	-1,636	1	0,044	LBD37_ARATH	LOB domain-containing protein 37 (ASYMMETRIC LEAVES 2-like protein 39) (AS2-like protein 39)	LBD37 ASL39 At5g67420 K8K14.16	Arabidopsis thaliana (Mouse-ear cress)		Complete proteome; Reference proteome
SRR648705_TR16901_c0_g2_i1_len=2423	DIT1_ARATH	Q:836-1969,H:180-557	89.68%ID	E:0	Dicarboxylate transporter 1, chloroplastic	PF00939.16^Na_sulph_symp^Sodium:sulfate symporter transmembrane region^1-297^E:4.3e-87	0,231	-0,4	0,624	0,185	0	-1,864	0	-1,737	0,845	0,079	DIT1_ARATH	Dicarboxylate transporter 1, chloroplastic (2-oxoglutarate/malate translocator 1) (AtpOMT1)	DIT1 At5g12860 T24H18.30	Arabidopsis thaliana (Mouse-ear cress)		Alternative splicing; Chloroplast; Complete proteome; Membrane; Plastid; Plastid inner membrane; Reference proteome; Transit peptide; Transmembrane; Transmembrane helix; Transport
SRR648705_TR20491_c0_g1_i1_len=2010	C75A1_PETHY	Q:1723-308,H:33-498	35.92%ID	E:6e-94	Flavonoid 3',5'-hydroxylase 1	PF00067.19^p450^Cytochrome P450^35-497^E:1.1e-96	1	1,364	1	0,856	0	-1,784	0	-1,947	0,18	0,843	C75A1_PETHY	Flavonoid 3',5'-hydroxylase 1 (F3'5'H) (EC 1.14.13.88) (CYPLXXVA1) (Cytochrome P450 75A1)	CYP75A1 HF1	Petunia hybrida (Petunia)	PATHWAY: Pigment biosynthesis; anthocyanin biosynthesis.	Endoplasmic reticulum; Heme; Iron; Metal-binding; Microsome; Monooxygenase; NADP; Oxidoreductase
SRR648705_TR23645_c0_g1_i1_len=2118	PEAMT_SPIOL	Q:1746-295,H:10-493	80.58%ID	E:0	Phosphoethanolamine N-methyltransferase	PF13489.3^Methyltransf_23^Methyltransferase domain^46-167^E:2.2e-07`PF13847.3^Methyltransf_31^Methyltransferase domain^54-167^E:9e-11`PF05891.9^Methyltransf_PK^AdoMet dependent proline di-methyltransferase^54-192^E:5.9e-10`PF05401.8^NodS^Nodulation protein S (NodS)^54-156^E:2.8e-07`PF03848.11^TehB^Tellurite resistance protein TehB^55-154^E:1.2e-05`PF13649.3^Methyltransf_25^Methyltransferase domain^57-152^E:7e-14`PF08241.9^Methyltransf_11^Methyltransferase domain^58-156^E:4e-14`PF08242.9^Methyltransf_12^Methyltransferase domain^58-154^E:7.4e-08`PF01739.15^CheR^CheR methyltransferase, SAM binding domain^97-156^E:0.00027`PF00891.15^Methyltransf_2^O-methyltransferase^255-424^E:0.00026`PF02353.17^CMAS^Mycolic acid cyclopropane synthetase^266-462^E:1.2e-17`PF05175.11^MTS^Methyltransferase small domain^271-354^E:0.00015`PF13489.3^Methyltransf_23^Methyltransferase domain^272-426^E:1.2e-22`PF01209.15^Ubie_methyltran^ubiE/COQ5 methyltransferase family^272-389^E:1.9e-10`PF07021.9^MetW^Methionine biosynthesis protein MetW^280-364^E:0.00011`PF13847.3^Methyltransf_31^Methyltransferase domain^282-387^E:6e-20`PF13649.3^Methyltransf_25^Methyltransferase domain^286-379^E:2.8e-19`PF08241.9^Methyltransf_11^Methyltransferase domain^287-383^E:5.3e-18`PF08242.9^Methyltransf_12^Methyltransferase domain^287-380^E:5.1e-13`PF01739.15^CheR^CheR methyltransferase, SAM binding domain^328-383^E:0.00019	0,351	-0,238	0,011	-0,754	0	-1,855	0	-1,735	0,597	0,147	PEAMT_SPIOL	Phosphoethanolamine N-methyltransferase (EC 2.1.1.103)	PEAMT	Spinacia oleracea (Spinach)	PATHWAY: Phospholipid metabolism; phosphatidylcholine biosynthesis; phosphocholine from phosphoethanolamine: step 1/1.	Cytoplasm; Lipid biosynthesis; Lipid metabolism; Methyltransferase; Phospholipid biosynthesis; Phospholipid metabolism; Repeat; S-adenosyl-L-methionine; Stress response; Transferase
SRR648705_TR24334_c0_g1_i1_len=1200	NA	NA	NA	NA	NA	PF07386.8^DUF1499^Protein of unknown function (DUF1499)^91-218^E:1.6e-29	0,611	-0,345	0,623	-0,419	0	-1,638	0	-1,665	0,37	-0,407	.	.	.	.	.	.
SRR648705_TR29032_c0_g1_i3_len=1237	NA	NA	NA	NA	NA	.	1	-0,186	0,76	-0,602	0	-1,732	0	-1,663	0,84	-0,166	.	.	.	.	.	.
SRR648705_TR29776_c0_g1_i1_len=1598	CHSY_CATRO	Q:260-1426,H:1-389	99.74%ID	E:0	Chalcone synthase	PF00195.16^Chal_sti_synt_N^Chalcone and stilbene synthases, N-terminal domain^5-228^E:5.6e-125`PF08392.9^FAE1_CUT1_RppA^FAE1/Type III polyketide synthase-like protein^109-242^E:4.3e-07`PF08545.7^ACP_syn_III^3-Oxoacyl-[acyl-carrier-protein (ACP)] synthase III^162-227^E:1.7e-05`PF02797.12^Chal_sti_synt_C^Chalcone and stilbene synthases, C-terminal domain^238-387^E:6.7e-72`PF08541.7^ACP_syn_III_C^3-Oxoacyl-[acyl-carrier-protein (ACP)] synthase III C terminal^301-384^E:5e-07	0,117	-0,892	0,195	1,302	0	-2,023	0	-1,511	0,581	0,401	CHSY_CATRO	Chalcone synthase (EC 2.3.1.74) (Naringenin-chalcone synthase)	CHS	Catharanthus roseus (Madagascar periwinkle) (Vinca rosea)	PATHWAY: Secondary metabolite biosynthesis; flavonoid biosynthesis.	Acyltransferase; Flavonoid biosynthesis; Transferase
SRR648705_TR37308_c0_g1_i1_len=506	NA	NA	NA	NA	NA	.	0,083	-0,389	0,003	-0,69	0	-0,917	0	-0,948	0	0,925	.	.	.	.	.	.
SRR648705_TR42657_c0_g2_i1_len=2999	U83A1_ARATH	Q:1730-291,H:9-462	48.05%ID	E:7e-141	UDP-glycosyltransferase 83A1	PF00201.15^UDPGT^UDP-glucoronosyl and UDP-glucosyl transferase^115-275^E:4.4e-19	1	-0,026	0,228	-1,121	0,001	-1,526	0	-1,651	0,407	-0,675	U83A1_ARATH	UDP-glycosyltransferase 83A1 (EC 2.4.1.-)	UGT83A1 At3g02100 F1C9.11	Arabidopsis thaliana (Mouse-ear cress)		Complete proteome; Glycosyltransferase; Reference proteome; Transferase
SRR648707_TR693_c0_g1_i2_len=3595	CAPP2_ARATH	Q:580-3483,H:1-963	87.1%ID	E:0	Phosphoenolpyruvate carboxylase 2	PF00311.14^PEPcase^Phosphoenolpyruvate carboxylase^163-968^E:0	0,355	-0,326	0,684	0,221	0	-2,213	0	-1,384	0	1,399	CAPP2_ARATH	Phosphoenolpyruvate carboxylase 2 (AtPPC2) (PEPC 2) (PEPCase 2) (EC 4.1.1.31)	PPC2 At2g42600 F14N22.13	Arabidopsis thaliana (Mouse-ear cress)		Allosteric enzyme; Carbon dioxide fixation; Complete proteome; Cytoplasm; Lyase; Magnesium; Phosphoprotein; Photosynthesis; Reference proteome; Ubl conjugation
SRR648707_TR6353_c0_g2_i1_len=797	RK21_ARATH	Q:502-50,H:57-210	63.29%ID	E:1e-47	50S ribosomal protein L21, chloroplastic	PF00829.18^Ribosomal_L21p^Ribosomal prokaryotic L21 protein^98-198^E:2.2e-31	0,1	-0,47	0,076	-0,587	0	-1,396	0	-1,279	0,597	-0,504	RK21_ARATH	50S ribosomal protein L21, chloroplastic (CL21)	RPL21 At1g35680 F15O4.7	Arabidopsis thaliana (Mouse-ear cress)		Chloroplast; Complete proteome; Plastid; RNA-binding; Reference proteome; Ribonucleoprotein; Ribosomal protein; Transit peptide; rRNA-binding
SRR648707_TR10843_c0_g1_i3_len=1008	NA	NA	NA	NA	NA	.	0,086	-0,936	0,008	-2,99	0	-2,423	0	-3,216	0,129	-0,648	.	.	.	.	.	.
SRR648707_TR22709_c0_g2_i8_len=2493	NA	NA	NA	NA	NA	.	1	0	1	0	0	-6,954	0	-6,954	0,066	5,363	.	.	.	.	.	.
SRR924147_TR32482_c0_g1_i1_len=2162	GLNA2_DAUCA	Q:1631-426,H:1-403	89.08%ID	E:0	Glutamine synthetase, chloroplastic	PF03951.16^Gln-synt_N^Glutamine synthetase, beta-Grasp domain^81-156^E:2.2e-10`PF00120.21^Gln-synt_C^Glutamine synthetase, catalytic domain^185-406^E:7.7e-16	0,619	-0,114	0,321	-0,22	0	-1,057	0,001	-0,727	1	0,016	GLNA2_DAUCA	Glutamine synthetase, chloroplastic (EC 6.3.1.2) (GS2) (Glutamate–ammonia ligase)	GLN2	Daucus carota (Wild carrot)		ATP-binding; Chloroplast; Ligase; Nucleotide-binding; Plastid; Transit peptide
SRR924148_TR2003_c0_g1_i1_len=1287	NA	NA	NA	NA	NA	PF03168.10^LEA_2^Late embryogenesis abundant protein^149-235^E:7.2e-10	1	0,098	0,008	4,13	0	-5,667	0	-5,661	1	0,143	.	.	.	.	.	.
SRR924148_TR7756_c1_g3_i2_len=734	PPCK2_ARATH	Q:647-354,H:7-104	43.43%ID	E:4e-22	Phosphoenolpyruvate carboxylase kinase 2	PF00069.22^Pkinase^Protein kinase domain^34-141^E:9.5e-18`PF07714.14^Pkinase_Tyr^Protein tyrosine kinase^37-128^E:6.4e-09	1	0,149	0,129	-5,178	0	-2,512	0	-1,668	1	-0,297	PPCK2_ARATH	Phosphoenolpyruvate carboxylase kinase 2 (AtPPCK2) (EC 2.7.11.1)	PPCK2 At3g04530 T27C4.19	Arabidopsis thaliana (Mouse-ear cress)		ATP-binding; Complete proteome; Kinase; Nucleotide-binding; Pyruvate; Reference proteome; Serine/threonine-protein kinase; Transferase
SRR924148_TR11150_c1_g1_i1_len=348	NA	NA	NA	NA	NA	.	0,643	0,436	0,09	0,962	0,001	-1,052	0	-1,308	0,537	0,341	.	.	.	.	.	.
SRR924148_TR16153_c1_g1_i1_len=3622	COB22_ARATH	Q:3333-562,H:1-926	84.02%ID	E:0	Coatomer subunit beta'-2	PF00400.29^WD40^WD domain, G-beta repeat^91-127^E:8e-06`PF00400.29^WD40^WD domain, G-beta repeat^133-171^E:0.00013`PF00400.29^WD40^WD domain, G-beta repeat^177-215^E:5.6e-06`PF00400.29^WD40^WD domain, G-beta repeat^220-256^E:6.6e-09`PF04053.11^Coatomer_WDAD^Coatomer WD associated region^319-763^E:7.2e-167	1	0,006	0	2,883	0	-2,642	0	-2,99	0,547	-0,414	COB22_ARATH	Coatomer subunit beta'-2 (Beta'-coat protein 2) (Beta'-COP 2)	At1g52360 F19K6.16	Arabidopsis thaliana (Mouse-ear cress)		Alternative splicing; Complete proteome; Cytoplasm; Cytoplasmic vesicle; ER-Golgi transport; Golgi apparatus; Membrane; Protein transport; Reference proteome; Repeat; Transport; WD repeat
SRR924148_TR16153_c1_g1_i4_len=3587	COB22_ARATH	Q:3298-527,H:1-926	84.02%ID	E:0	Coatomer subunit beta'-2	PF00400.29^WD40^WD domain, G-beta repeat^91-127^E:8e-06`PF00400.29^WD40^WD domain, G-beta repeat^133-171^E:0.00013`PF00400.29^WD40^WD domain, G-beta repeat^177-215^E:5.6e-06`PF00400.29^WD40^WD domain, G-beta repeat^220-256^E:6.6e-09`PF04053.11^Coatomer_WDAD^Coatomer WD associated region^319-763^E:7.2e-167	0,724	-0,203	0,001	5,566	0	-5,874	0	-4,508	0,679	-0,432	COB22_ARATH	Coatomer subunit beta'-2 (Beta'-coat protein 2) (Beta'-COP 2)	At1g52360 F19K6.16	Arabidopsis thaliana (Mouse-ear cress)		Alternative splicing; Complete proteome; Cytoplasm; Cytoplasmic vesicle; ER-Golgi transport; Golgi apparatus; Membrane; Protein transport; Reference proteome; Repeat; Transport; WD repeat
SRR924148_TR23140_c0_g1_i1_len=983	RBS_MALSP	Q:223-762,H:1-183	81.42%ID	E:2e-107	Ribulose bisphosphate carboxylase small chain, chloroplastic	PF12338.5^RbcS^Ribulose-1,5-bisphosphate carboxylase small subunit^2-42^E:6.5e-21`PF00101.17^RuBisCO_small^Ribulose bisphosphate carboxylase, small chain^69-177^E:1.2e-39	0,111	-0,331	0,008	-0,551	0	-0,866	0,001	-0,717	0,772	-0,06	RBS_MALSP	Ribulose bisphosphate carboxylase small chain, chloroplastic (RuBisCO small subunit) (EC 4.1.1.39)	RBCS	Malus sp. (Crab apple)		Calvin cycle; Carbon dioxide fixation; Chloroplast; Lyase; Monooxygenase; Oxidoreductase; Photorespiration; Photosynthesis; Plastid; Transit peptide
SRR924148_TR25547_c0_g1_i1_len=2219	NA	NA	NA	NA	NA	PF05368.10^NmrA^NmrA-like family^97-210^E:1.8e-05`PF13460.3^NAD_binding_10^NAD(P)H-binding^101-309^E:5.9e-15	0,61	-0,117	0,332	-0,229	0	-1,404	0	-1,096	0,292	0,247	.	.	.	.	.	.
SRR924148_TR39696_c0_g1_i1_len=657	NA	NA	NA	NA	NA	.	1	-0,579	0,254	2,727	0	-4,782	0	-4,366	1	0,161	.	.	.	.	.	.

MsIntact24h

Transcript (CDF97)	Uniprot homolog	BLASTX Position	BLASTX %Identity	BLASTX evalue	Uniprot descr	Pfam	PValue_Ms6h	logFC_Ms6h	PValue_Ms8h	logFC_Ms8h	PValue_MsDamaged24h	logFC_MsDamaged24h	PValue_MsIntact24h	logFC_MsIntact24h	PValue_Ms1wk	logFC_Ms1wk	Entry.name	Protein names	Gene names	Organism	Pathway	Keywords
SRR1144633_TR5530_c0_g1_i1_len=932	NA	NA	NA	NA	NA	.	0,055	-0,766	0,77	-0,136	1	0,094	0	-6,763	0,774	0,134	.	.	.	.	.	.
SRR1144634_TR5616_c0_g2_i1_len=1399	Y2766_ARATH	Q:1225-314,H:33-325	74.34%ID	E:5e-153	Uncharacterized protein At2g37660, chloroplastic	PF05368.10^NmrA^NmrA-like family^64-232^E:6.5e-07`PF13460.3^NAD_binding_10^NAD(P)H-binding^68-274^E:4.7e-32	0,673	-0,115	0,919	-0,036	0,002	-0,714	0,001	-0,762	0,365	0,547	Y2766_ARATH	Uncharacterized protein At2g37660, chloroplastic	At2g37660 F13M22.16	Arabidopsis thaliana (Mouse-ear cress)		Acetylation; Chloroplast; Complete proteome; Plastid; Reference proteome; Transit peptide
SRR1144634_TR26729_c0_g1_i1_len=1948	HPR1_ARATH	Q:190-1347,H:1-386	90.93%ID	E:0	Glycerate dehydrogenase HPR, peroxisomal	PF00389.27^2-Hacid_dh^D-isomer specific 2-hydroxyacid dehydrogenase, catalytic domain^35-346^E:2.7e-17`PF02826.16^2-Hacid_dh_C^D-isomer specific 2-hydroxyacid dehydrogenase, NAD binding domain^130-322^E:5e-47	0,83	0,103	0,019	-0,892	0,002	-0,843	0,001	-0,939	0,123	0,362	HPR1_ARATH	Glycerate dehydrogenase HPR, peroxisomal (GDH) (EC 1.1.1.29) (NADH-dependent hydroxypyruvate reductase 1) (AtHPR1) (HPR 1)	HPR At1g68010 T23K23.14	Arabidopsis thaliana (Mouse-ear cress)	PATHWAY: Photosynthesis; photorespiration; 3-phospho-D-glycerate from glycine: step 3/4.	Alternative splicing; Complete proteome; Direct protein sequencing; Glycolate pathway; NAD; Oxidoreductase; Peroxisome; Photorespiration; Pyruvate; Reference proteome
SRR1144634_TR27431_c0_g1_i1_len=589	5MAT1_SALSN	Q:231-569,H:57-169	45.61%ID	E:3e-26	Malonyl-coenzyme:anthocyanin 5-O-glucoside-6'''-O-malonyltransferase {ECO:0000303|PubMed:11598135}	.	0,255	0,438	0,914	0,077	0,001	-1,36	0	-1,916	1	1,138	5MAT1_SALSN	Malonyl-coenzyme:anthocyanin 5-O-glucoside-6'''-O-malonyltransferase (Malonyl CoA:anthocyanin 5-O-glucoside-6'''-O-malonyltransferase) (Ss5MaT1) (EC 2.3.1.172)	5MAT1	Salvia splendens (Scarlet sage)	PATHWAY: Pigment biosynthesis; anthocyanin biosynthesis.	Acyltransferase; Phenylpropanoid metabolism; Transferase
SRR122236_TR11769_c1_g1_i1_len=994	CFI3_ARATH	Q:754-161,H:6-204	68.34%ID	E:2e-82	Probable chalcone–flavonone isomerase 3	PF02431.12^Chalcone^Chalcone-flavanone isomerase^9-201^E:3.8e-30	1	0,023	0	1,732	0,09	-0,45	0	-1,022	0,599	-0,187	CFI3_ARATH	Probable chalcone–flavonone isomerase 3 (Chalcone isomerase 3) (EC 5.5.1.6) (Chalcone isomerase-like 1)	CHI3 CHI-L1 CHIL At5g05270 K18I23.7	Arabidopsis thaliana (Mouse-ear cress)	PATHWAY: Secondary metabolite biosynthesis; flavonoid biosynthesis.	3D-structure; Complete proteome; Flavonoid biosynthesis; Isomerase; Reference proteome
SRR122237_TR27637_c0_g1_i1_len=1982	INO1_TOBAC	Q:148-1677,H:1-510	94.12%ID	E:0	Inositol-3-phosphate synthase	PF07994.9^NAD_binding_5^Myo-inositol-1-phosphate synthase^62-494^E:1.7e-142`PF01658.14^Inos-1-P_synth^Myo-inositol-1-phosphate synthase^310-423^E:2.6e-48	0,555	-0,301	0,56	-0,626	1	0	0,001	-0,721	0	1,311	INO1_TOBAC	Inositol-3-phosphate synthase (MIP synthase) (EC 5.5.1.4) (Myo-inositol 1-phosphate synthase) (IPS) (MI-1-P synthase)		Nicotiana tabacum (Common tobacco)	PATHWAY: Polyol metabolism; myo-inositol biosynthesis; myo-inositol from D-glucose 6-phosphate: step 1/2.	Cytoplasm; Inositol biosynthesis; Isomerase; Lipid biosynthesis; Lipid metabolism; NAD; Phospholipid biosynthesis; Phospholipid metabolism
SRR122238_TR2765_c0_g1_i9_len=1099	SABP2_TOBAC	Q:995-213,H:6-259	60.15%ID	E:6e-108	Salicylic acid-binding protein 2	PF12146.5^Hydrolase_4^Serine aminopeptidase, S33^5-110^E:2e-06`PF00561.17^Abhydrolase_1^alpha/beta hydrolase fold^7-252^E:2.8e-16`PF12697.4^Abhydrolase_6^Alpha/beta hydrolase family^8-258^E:8.6e-19	0,77	-0,206	0,524	-0,517	0,004	-1,334	0,001	-1,549	0,902	-0,11	SABP2_TOBAC	Salicylic acid-binding protein 2 (NtSABP2) (EC 3.1.1.-) (Methyl salicylate esterase)	SABP2	Nicotiana tabacum (Common tobacco)		3D-structure; Hydrolase; Immunity; Innate immunity; Plant defense
SRR122238_TR6108_c0_g2_i3_len=1661	C7D55_HYOMU	Q:1452-109,H:47-495	46.34%ID	E:1e-148	Premnaspirodiene oxygenase	PF00067.19^p450^Cytochrome P450^56-499^E:8.7e-91	0,519	-0,233	0,027	-2,245	0,071	-0,897	0	-2,433	0,004	1,034	C7D55_HYOMU	Premnaspirodiene oxygenase (HPO) (EC 1.14.13.121) (Cytochrome P450 71D55)	CYP71D55	Hyoscyamus muticus (Egyptian henbane)		Heme; Iron; Membrane; Metal-binding; Monooxygenase; NADP; Oxidoreductase; Transmembrane; Transmembrane helix
SRR122238_TR12534_c1_g3_i3_len=2397	AI5L5_ARATH	Q:1556-246,H:21-416	53.59%ID	E:2e-102	ABSCISIC ACID-INSENSITIVE 5-like protein 5	PF07716.12^bZIP_2^Basic region leucine zipper^390-437^E:1.2e-09`PF00170.18^bZIP_1^bZIP transcription factor^391-443^E:5.2e-13	1	-0,027	0,44	-0,743	0,009	-1,099	0,001	-1,614	0,559	0,424	AI5L5_ARATH	ABSCISIC ACID-INSENSITIVE 5-like protein 5 (ABA-responsive element-binding protein 1) (Abscisic acid responsive elements-binding factor 2) (ABRE-binding factor 2) (bZIP transcription factor 36) (AtbZIP36)	ABF2 AREB1 BZIP36 At1g45249 T2P3	Arabidopsis thaliana (Mouse-ear cress)		Abscisic acid signaling pathway; Activator; Alternative splicing; Complete proteome; DNA-binding; Nucleus; Phosphoprotein; Reference proteome; Transcription; Transcription regulation
SRR122238_TR17876_c0_g1_i1_len=1947	GERS_OCIBA	Q:260-1714,H:88-566	68.11%ID	E:0	Geraniol synthase, chloroplastic	PF01397.18^Terpene_synth^Terpene synthase, N-terminal domain^72-222^E:1.9e-45`PF03936.13^Terpene_synth_C^Terpene synthase family, metal binding domain^253-520^E:1.6e-95	0,182	0,516	1	-0,425	0,005	-1,491	0	-3,621	0,65	0,135	GERS_OCIBA	Geraniol synthase, chloroplastic (ObGES) (EC 3.1.7.11)	GES	Ocimum basilicum (Sweet basil)	PATHWAY: Secondary metabolite biosynthesis; terpenoid biosynthesis.	Chloroplast; Hydrolase; Manganese; Metal-binding; Plastid; Transit peptide
SRR122238_TR29823_c0_g1_i1_len=2701	RVE1_ARATH	Q:1816-1010,H:32-251	48.33%ID	E:4e-43	Protein REVEILLE 1	PF00249.28^Myb_DNA-binding^Myb-like DNA-binding domain^61-104^E:2.4e-13	1	0,639	1	0,826	0,092	-0,591	0,001	-1,287	1	-0,066	RVE1_ARATH	Protein REVEILLE 1	RVE1 At5g17300 MKP11.15	Arabidopsis thaliana (Mouse-ear cress)		Biological rhythms; Complete proteome; DNA-binding; Nucleus; Reference proteome; Transcription; Transcription regulation
SRR122238_TR34820_c1_g2_i1_len=1227	TSN_CHICK	Q:903-304,H:19-222	41.83%ID	E:5e-41	Translin	PF01997.13^Translin^Translin family^110-302^E:8.7e-46	0,199	-0,431	0,382	-0,344	0,502	0,358	0	-7,735	1	-0,195	TSN_CHICK	Translin (EC 3.1.-.-) (Component 3 of promoter of RISC) (C3PO)	TSN	Gallus gallus (Chicken)		Complete proteome; DNA-binding; Endonuclease; Hydrolase; Nuclease; Nucleus; RNA-binding; Reference proteome
SRR122238_TR41648_c0_g1_i8_len=3143	NA	NA	NA	NA	NA	PF05368.10^NmrA^NmrA-like family^97-210^E:5.9e-06`PF13460.3^NAD_binding_10^NAD(P)H-binding^101-284^E:1.8e-13	0,91	-0,077	0,003	-1,414	0,002	-1,285	0,001	-1,443	0,691	0,293	.	.	.	.	.	.
SRR122238_TR45607_c0_g1_i3_len=1851	ACR4_ARATH	Q:144-1481,H:1-451	77.97%ID	E:0	ACT domain-containing protein ACR4 {ECO:0000305}	PF13740.3^ACT_6^ACT domain^119-163^E:4.4e-05	1	-0,046	1	0,195	0,037	-1,223	0	-2,617	0,01	-1,694	ACR4_ARATH	ACT domain-containing protein ACR4 (Protein ACT DOMAIN REPEATS 4)	ACR4 At1g69040 F4N2.2	Arabidopsis thaliana (Mouse-ear cress)		Alternative splicing; Complete proteome; Reference proteome; Repeat
SRR122238_TR46952_c0_g1_i1_len=4089	JAC1_ARATH	Q:1240-1031,H:572-639	68.57%ID	E:5e-22	J domain-containing protein required for chloroplast accumulation response 1	.	1	-0,448	0,629	-2,084	0,003	-1,52	0	-1,791	1	-0,134	JAC1_ARATH	J domain-containing protein required for chloroplast accumulation response 1	JAC1 At1g75100 F9E10.5	Arabidopsis thaliana (Mouse-ear cress)		3D-structure; Coiled coil; Complete proteome; Cytoplasm; Phosphoprotein; Reference proteome
SRR122238_TR54660_c0_g1_i1_len=620	PLY21_ARATH	Q:620-177,H:245-392	72.3%ID	E:1e-70	Putative pectate lyase 21	PF00544.16^Pec_lyase_C^Pectate lyase^9-64^E:2.1e-07	0,357	-0,677	0,034	-3,325	0,004	-2,183	0	-7,42	0,044	1,084	PLY21_ARATH	Putative pectate lyase 21 (EC 4.2.2.2)	At5g55720 MDF20.16	Arabidopsis thaliana (Mouse-ear cress)	PATHWAY: Glycan metabolism; pectin degradation; 2-dehydro-3-deoxy-D-gluconate from pectin: step 2/5.	Calcium; Complete proteome; Glycoprotein; Lyase; Metal-binding; Reference proteome; Signal
SRR122238_TR55879_c2_g1_i1_len=2570	NA	NA	NA	NA	NA	PF14365.3^DUF4409^Domain of unknown function (DUF4409)^72-194^E:1.4e-46`PF03080.12^DUF239^Domain of unknown function (DUF239)^207-429^E:9.1e-90	0,598	-0,145	0,042	-0,608	0,015	-0,792	0	-1,292	0,344	0,288	.	.	.	.	.	.
SRR122238_TR57249_c0_g1_i3_len=693	NA	NA	NA	NA	NA	.	0,285	0,225	0,362	0,193	0,002	-2,305	0	-7,157	0,932	-0,059	.	.	.	.	.	.
SRR122238_TR58346_c2_g1_i6_len=1655	NA	NA	NA	NA	NA	PF00403.23^HMA^Heavy-metal-associated domain^60-113^E:7.5e-12`PF00403.23^HMA^Heavy-metal-associated domain^149-195^E:5.7e-11	1	0,292	1	-0,505	0,006	-1,674	0	-2,256	1	-0,666	.	.	.	.	.	.
SRR122238_TR61350_c2_g1_i3_len=1183	YAB1_ARATH	Q:190-831,H:17-229	58.93%ID	E:1e-71	Axial regulator YABBY 1	PF04690.10^YABBY^YABBY protein^27-191^E:1.8e-69	0,64	0,182	0,006	-1,396	0,002	-1,061	0	-1,711	0,37	-0,268	YAB1_ARATH	Axial regulator YABBY 1 (FI-54) (Protein ABNORMAL FLORAL ORGANS) (Protein FILAMENTOUS FLOWER) (Protein antherless)	YAB1 AFO AT FIL At2g45190 F4L23.30	Arabidopsis thaliana (Mouse-ear cress)		Complete proteome; DNA-binding; Developmental protein; Metal-binding; Nucleus; Reference proteome; Zinc; Zinc-finger
SRR122238_TR62466_c0_g1_i1_len=796	PLA2A_ARATH	Q:592-185,H:11-148	58.7%ID	E:3e-55	Phospholipase A2-alpha {ECO:0000303|PubMed:16140037}	.	0,694	0,366	1	0,114	0,212	-0,608	0,001	-1,827	1	-1,049	PLA2A_ARATH	Phospholipase A2-alpha (EC 3.1.1.4) (Secretory phospholipase A2-alpha) (AtsPLA2-alpha)	PLA2-ALPHA At2g06925 T4E14.19	Arabidopsis thaliana (Mouse-ear cress)		Calcium; Complete proteome; Cytoplasmic vesicle; Disulfide bond; Golgi apparatus; Hydrolase; Lipid degradation; Lipid metabolism; Metal-binding; Nucleus; Reference proteome; Secreted; Signal
SRR122238_TR64257_c1_g1_i2_len=896	LAML_ARATH	Q:140-631,H:10-172	49.7%ID	E:7e-46	Lamin-like protein	PF02298.14^Cu_bind_like^Plastocyanin-like domain^74-156^E:1.7e-20	0,552	-0,209	0,095	-0,638	0,002	-1,17	0	-1,444	0,024	-0,762	LAML_ARATH	Lamin-like protein	At5g15350 F8M21_240	Arabidopsis thaliana (Mouse-ear cress)		Cell membrane; Complete proteome; GPI-anchor; Glycoprotein; Lipoprotein; Membrane; Reference proteome; Signal
SRR122238_TR68728_c0_g1_i1_len=1410	CRR60_ARATH	Q:1179-511,H:23-248	60.7%ID	E:5e-86	Cysteine-rich repeat secretory protein 60	PF01657.14^Stress-antifung^Salt stress response/antifungal^41-137^E:1.6e-19`PF01657.14^Stress-antifung^Salt stress response/antifungal^181-239^E:2e-10	0,76	-0,765	0,517	0,644	0,002	-1,064	0	-1,735	1	-0,291	CRR60_ARATH	Cysteine-rich repeat secretory protein 60 (Plasmodesmata-located protein 7) (PDLP7)	CRRSP60 At5g37660 K12B20.12	Arabidopsis thaliana (Mouse-ear cress)		Alternative splicing; Cell junction; Complete proteome; Disulfide bond; Host-virus interaction; Membrane; Reference proteome; Repeat; Signal; Transmembrane; Transmembrane helix; Transport
SRR122238_TR80686_c0_g1_i1_len=1145	SBP1_ANTMA	Q:67-363,H:35-130	75.76%ID	E:4e-41	Squamosa promoter-binding protein 1	PF03110.11^SBP^SBP domain^43-116^E:3.9e-33	1	-0,327	0,381	-1,5	0,01	-1,745	0	-3,526	0,155	0,491	SBP1_ANTMA	Squamosa promoter-binding protein 1	SBP1	Antirrhinum majus (Garden snapdragon)		DNA-binding; Metal-binding; Nucleus; Transcription; Transcription regulation; Zinc; Zinc-finger
SRR1271857_TR1741_c0_g1_i2_len=1564	PURU2_ARATH	Q:212-817,H:19-220	69.8%ID	E:1e-91	Formyltetrahydrofolate deformylase 2, mitochondrial	PF00551.16^Formyl_trans_N^Formyl transferase^132-220^E:4.9e-06	1	-0,376	0,189	-1,658	0,01	-1,55	0,001	-2,224	1	0,278	PURU2_ARATH	Formyltetrahydrofolate deformylase 2, mitochondrial (EC 3.5.1.10)	PURU2 At4g17360 dl4715c FCAALL.417	Arabidopsis thaliana (Mouse-ear cress)		Complete proteome; Hydrolase; Mitochondrion; One-carbon metabolism; Photorespiration; Reference proteome; Transferase; Transit peptide
SRR1271857_TR16851_c0_g1_i1_len=636	NA	NA	NA	NA	NA	.	1	-0,13	0,895	0,111	0,341	-0,388	0	-1,918	0,025	-1,381	.	.	.	.	.	.
SRR1271857_TR18675_c0_g1_i7_len=1461	FKB19_ARATH	Q:518-997,H:97-256	88.12%ID	E:4e-96	Peptidyl-prolyl cis-trans isomerase FKBP19, chloroplastic	PF00254.25^FKBP_C^FKBP-type peptidyl-prolyl cis-trans isomerase^156-277^E:5.4e-21	0,568	-0,241	0,047	-0,727	0,006	-0,911	0,001	-1,127	0,644	-0,169	FKB19_ARATH	Peptidyl-prolyl cis-trans isomerase FKBP19, chloroplastic (PPIase FKBP19) (EC 5.2.1.8) (FK506-binding protein 19) (AtFKBP19) (Immunophilin FKBP19) (Rotamase)	FKBP19 FKBP27 At5g13410 T22N19.60	Arabidopsis thaliana (Mouse-ear cress)		Chloroplast; Complete proteome; Direct protein sequencing; Isomerase; Phosphoprotein; Plastid; Reference proteome; Rotamase; Thylakoid; Transit peptide
SRR1271857_TR19276_c2_g1_i2_len=2384	ALF1_PEA	Q:1445-315,H:8-357	79.31%ID	E:0	Fructose-bisphosphate aldolase, cytoplasmic isozyme 1	PF00274.16^Glycolytic^Fructose-bisphosphate aldolase class-I^10-237^E:5.5e-117	1	-0,023	0,004	-2,512	0,001	-3,519	0	-4,909	1	0,454	ALF1_PEA	Fructose-bisphosphate aldolase, cytoplasmic isozyme 1 (EC 4.1.2.13)		Pisum sativum (Garden pea)	PATHWAY: Carbohydrate degradation; glycolysis; D-glyceraldehyde 3-phosphate and glycerone phosphate from D-glucose: step 4/4.	Cytoplasm; Glycolysis; Lyase; Schiff base
SRR1271857_TR22447_c0_g1_i4_len=1697	C85A1_SOLLC	Q:1462-137,H:23-464	84.84%ID	E:0	Cytochrome P450 85A1	PF00067.19^p450^Cytochrome P450^35-448^E:8e-60	1	0,042	0,03	-0,994	0,021	-1,585	0	-3,837	1	-0,062	C85A1_SOLLC	Cytochrome P450 85A1 (EC 1.14.-.-) (C6-oxidase) (Dwarf protein)	CYP85A1 CYP85 D	Solanum lycopersicum (Tomato) (Lycopersicon esculentum)	PATHWAY: Plant hormone biosynthesis; brassinosteroid biosynthesis.	Brassinosteroid biosynthesis; Complete proteome; Heme; Iron; Lipid biosynthesis; Lipid metabolism; Membrane; Metal-binding; Monooxygenase; Oxidoreductase; Reference proteome; Steroid biosynthesis; Transmembrane; Transmembrane helix
SRR1271857_TR25567_c0_g2_i2_len=1932	SGAT_ARATH	Q:383-1240,H:1-286	90.91%ID	E:1e-177	Serine–glyoxylate aminotransferase	PF00266.16^Aminotran_5^Aminotransferase class-V^12-285^E:1.2e-31	1	0,118	0,068	-1,286	0,001	-1,274	0	-1,994	0,133	1,285	SGAT_ARATH	Serine–glyoxylate aminotransferase (EC 2.6.1.45) (Alanine–glyoxylate aminotransferase) (AGT) (EC 2.6.1.44) (Asparagine aminotransferase) (EC 2.6.1.-) (Serine–pyruvate aminotransferase) (EC 2.6.1.51)	AGT1 At2g13360 F14O4.7	Arabidopsis thaliana (Mouse-ear cress)		Acetylation; Aminotransferase; Complete proteome; Peroxisome; Phosphoprotein; Photorespiration; Pyridoxal phosphate; Reference proteome; Transferase
SRR1271857_TR26021_c0_g1_i2_len=2016	NA	NA	NA	NA	NA	PF04720.9^PDDEXK_6^PDDEXK-like family of unknown function^77-297^E:5.9e-70	0,793	-0,152	0,002	-1,255	0,002	-1,094	0	-1,514	0,009	0,92	.	.	.	.	.	.
SRR1271857_TR28055_c0_g2_i1_len=1611	SCPDL_ARATH	Q:119-271,H:129-179	52.94%ID	E:1e-27	Probable mitochondrial saccharopine dehydrogenase-like oxidoreductase At5g39410	PF02622.12^DUF179^Uncharacterized ACR, COG1678^138-303^E:1.2e-26	0,76	-0,685	1	-0,843	0,007	-1,536	0	-2,115	1	-0,266	SCPDL_ARATH	Probable mitochondrial saccharopine dehydrogenase-like oxidoreductase At5g39410 (SDH) (EC 1.-.-.-)	At5g39410 MUL8.9	Arabidopsis thaliana (Mouse-ear cress)		Acetylation; Complete proteome; Membrane; Mitochondrion; Oxidoreductase; Reference proteome
SRR1271857_TR31283_c0_g1_i7_len=1959	DHGY_CUCSA	Q:1474-953,H:70-243	93.1%ID	E:0	Glycerate dehydrogenase	PF02826.16^2-Hacid_dh_C^D-isomer specific 2-hydroxyacid dehydrogenase, NAD binding domain^5-87^E:7.1e-26	0,692	-0,764	0,043	-2,656	0,007	-2,13	0,001	-2,911	1	-0,042	DHGY_CUCSA	Glycerate dehydrogenase (GDH) (EC 1.1.1.29) (NADH-dependent hydroxypyruvate reductase) (HPR)	HPR-A	Cucumis sativus (Cucumber)	PATHWAY: Photosynthesis; photorespiration; 3-phospho-D-glycerate from glycine: step 3/4.	Glycolate pathway; NAD; Oxidoreductase; Peroxisome; Photorespiration
SRR1271857_TR42554_c0_g1_i3_len=1447	NA	NA	NA	NA	NA	.	1	-0,028	0,132	-1,441	0,003	-2,231	0,001	-2,754	0,195	0,907	.	.	.	.	.	.
SRR1271858_TR15761_c0_g1_i3_len=1948	BH104_ARATH	Q:802-392,H:149-283	64.23%ID	E:2e-33	Transcription factor bHLH104	.	0,748	0,242	0,298	-0,676	0,004	-1,54	0	-2,049	1	-0,032	BH104_ARATH	Transcription factor bHLH104 (Basic helix-loop-helix protein 104) (AtbHLH104) (bHLH 104) (Transcription factor EN 136) (bHLH transcription factor bHLH104)	BHLH104 EN136 At4g14410 dl3245w FCAALL.201	Arabidopsis thaliana (Mouse-ear cress)		Alternative splicing; Complete proteome; DNA-binding; Nucleus; Reference proteome; Transcription; Transcription regulation
SRR1271858_TR63738_c0_g3_i9_len=2759	NA	NA	NA	NA	NA	PF00294.21^PfkB^pfkB family carbohydrate kinase^88-208^E:6.3e-08	0,732	-0,916	0,01	2,712	0,257	-0,861	0	-7,009	0,798	0,44	.	.	.	.	.	.
SRR1271858_TR65593_c2_g6_i3_len=509	NA	NA	NA	NA	NA	.	1	0,141	0,517	-1,136	0,002	-1,737	0,001	-2,099	0,129	1,022	.	.	.	.	.	.
SRR1271859_TR10523_c2_g1_i2_len=689	C71A8_MENPI	Q:108-674,H:284-472	48.68%ID	E:6e-58	Cytochrome P450 71A8	PF00067.19^p450^Cytochrome P450^18-203^E:5.5e-51	0,778	-0,083	0	2,354	0,016	0,762	0	-1,79	0	2,112	C71A8_MENPI	Cytochrome P450 71A8 (EC 1.14.-.-)	CYP71A8	Mentha piperita (Peppermint) (Mentha aquatica ? Mentha spicata)		Heme; Iron; Membrane; Metal-binding; Monooxygenase; Oxidoreductase; Transmembrane; Transmembrane helix
SRR1271859_TR26429_c0_g1_i2_len=2957	CRD1_ARATH	Q:427-1305,H:1-294	83.05%ID	E:1e-162	Magnesium-protoporphyrin IX monomethyl ester [oxidative] cyclase, chloroplastic	PF02915.14^Rubrerythrin^Rubrerythrin^133-266^E:1.2e-41	0,854	-0,275	0,01	-2,1	0,003	-2,045	0	-3,094	0,663	0,499	CRD1_ARATH	Magnesium-protoporphyrin IX monomethyl ester [oxidative] cyclase, chloroplastic (Mg-protoporphyrin IX monomethyl ester oxidative cyclase) (EC 1.14.13.81) (Copper response defect 1 protein) (Dicarboxylate diiron protein) (AtZIP) (MPE-cyclase)	CRD1 ACSF AT103 CHL27 ZIP At3g56940 F24I3.20	Arabidopsis thaliana (Mouse-ear cress)	PATHWAY: Porphyrin-containing compound metabolism; chlorophyll biosynthesis.	Alternative splicing; Chlorophyll biosynthesis; Chloroplast; Complete proteome; Iron; Membrane; Metal-binding; NADP; Oxidoreductase; Photosynthesis; Plastid; Plastid inner membrane; Reference proteome; Thylakoid; Transit peptide
[illegible]		Q:440-117,H:1-																	CYCB1-2 Os02g0801300	Oryza sativa subsp. japonica		

SRR1271859_TR30155_c0_g2_i1_len=615	CCB12_ORYSJ	Q:[illegible],H:1-108	45.87%ID	E:4e-23	Cyclin-B1-2	PF05348.8^UMP1^Proteasome maturation factor UMP1^74-173^E:2.1e-24	0,352	-0,907	0,76	-0,774	0,258	-0,921	0,001	-6,644	0,461	-1,35	CCB12_ORYSJ	Cyclin-B1-2 (G2/mitotic-specific cyclin-B1-2) (CycB1;2)	[illegible] LOC_Os02g55720	Oryza sativa subsp. japonica (Rice)		Cell cycle; Cell division; Complete proteome; Cyclin; Reference proteome
SRR1271859_TR33928_c0_g1_i6_len=3089	GLYC7_ARATH	Q:615-1589,H:28-354	67.89%ID	E:1e-144	Serine hydroxymethyltransferase 7	PF00464.16^SHMT^Serine hydroxymethyltransferase^138-373^E:2.1e-94	1	1,992	0,129	-4,893	0,042	-2,205	0,001	-6,652	0,524	0,54	GLYC7_ARATH	Serine hydroxymethyltransferase 7 (AtSHMT7) (EC 2.1.2.1) (Glycine hydroxymethyltransferase 7) (Serine methylase 7)	SHM7 SHMT7 At1g36370 F7F23.9	Arabidopsis thaliana (Mouse-ear cress)	PATHWAY: One-carbon metabolism; tetrahydrofolate interconversion.	Complete proteome; Cytoplasm; One-carbon metabolism; Pyridoxal phosphate; Reference proteome; Transferase
SRR1271859_TR38747_c1_g1_i1_len=1778	NA	NA	NA	NA	NA	PF06273.8^eIF-4B^Plant specific eukaryotic initiation factor 4B^7-233^E:1.5e-34`PF06273.8^eIF-4B^Plant specific eukaryotic initiation factor 4B^174-369^E:1.3e-17	0,05	-0,811	0,35	-0,344	0,002	-1,246	0	-1,522	0,217	-0,481	.	.	.	.	.	.
SRR1271859_TR42941_c0_g1_i4_len=672	IMB1_ARATH	Q:106-300,H:806-870	83.08%ID	E:2e-25	Importin subunit beta-1 {ECO:0000305}	.	0,282	-1,115	0,18	0,9	0,505	-0,532	0,001	-3,087	1	0	IMB1_ARATH	Importin subunit beta-1 (Karyopherin subunit beta-1) (ATKPNB1)	KPNB1 At5g53480 MNC6.1	Arabidopsis thaliana (Mouse-ear cress)		Acetylation; Complete proteome; Cytoplasm; Nucleus; Protein transport; Reference proteome; Repeat; Stress response; Transport
SRR342017_TR725_c0_g1_i1_len=1166	ZFP8_ARATH	Q:835-38,H:8-257	36.82%ID	E:1e-32	Zinc finger protein 8	PF13912.3^zf-C2H2_6^C2H2-type zinc finger^101-125^E:0.00012	0,704	0,195	0,902	-0,13	0,007	-1,055	0	-1,667	0,472	-0,314	ZFP8_ARATH	Zinc finger protein 8	ZFP8 At2g41940 T6D20.16	Arabidopsis thaliana (Mouse-ear cress)		Complete proteome; Developmental protein; Differentiation; Metal-binding; Reference proteome; Transcription; Transcription regulation; Zinc; Zinc-finger
SRR342017_TR4009_c1_g1_i1_len=2199	GERS_OCIBA	Q:515-2110,H:44-566	64.98%ID	E:0	Geraniol synthase, chloroplastic	PF01397.18^Terpene_synth^Terpene synthase, N-terminal domain^164-314^E:2.5e-45`PF03936.13^Terpene_synth_C^Terpene synthase family, metal binding domain^345-612^E:1.3e-95	0,17	0,508	0,679	-0,373	0,051	-0,855	0,001	-1,648	1	-1,594	GERS_OCIBA	Geraniol synthase, chloroplastic (ObGES) (EC 3.1.7.11)	GES	Ocimum basilicum (Sweet basil)	PATHWAY: Secondary metabolite biosynthesis; terpenoid biosynthesis.	Chloroplast; Hydrolase; Manganese; Metal-binding; Plastid; Transit peptide
SRR342017_TR14008_c0_g2_i4_len=854	NA	NA	NA	NA	NA	.	0,212	0,959	0,214	-0,817	1	-0,216	0,001	-6,656	0,381	0,854	.	.	.	.	.	.
SRR342017_TR20766_c0_g2_i5_len=849	NA	NA	NA	NA	NA	.	1	0,221	0,016	-2,805	0,006	-0,984	0	-1,434	1	0,303	.	.	.	.	.	.
SRR342019_TR2921_c1_g1_i5_len=1413	ADHX_ORYSI	Q:182-1297,H:3-377	44.83%ID	E:3e-94	Alcohol dehydrogenase class-3	PF08240.9^ADH_N^Alcohol dehydrogenase GroES-like domain^39-162^E:1.6e-22`PF00107.23^ADH_zinc_N^Zinc-binding dehydrogenase^206-326^E:8.1e-22	0,028	0,553	0,1	0,981	0,071	-0,491	0	-1,865	0,287	0,287	ADHX_ORYSI	Alcohol dehydrogenase class-3 (EC 1.1.1.1) (Alcohol dehydrogenase class-III) (Glutathione-dependent formaldehyde dehydrogenase) (FALDH) (FDH) (GSH-FDH) (EC 1.1.1.-) (S-(hydroxymethyl)glutathione dehydrogenase) (EC 1.1.1.284)	ADHIII OsI_009236	Oryza sativa subsp. indica (Rice)		Complete proteome; Cytoplasm; Metal-binding; NAD; Oxidoreductase; Reference proteome; Zinc
SRR342019_TR7176_c0_g1_i1_len=1811	DDC_CATRO	Q:1360-194,H:112-500	98.46%ID	E:0	Aromatic-L-amino-acid decarboxylase	PF00282.16^Pyridoxal_deC^Pyridoxal-dependent decarboxylase conserved domain^4-333^E:5e-102	0,011	0,59	0	3,432	0,937	0,028	0	-1,39	1	0,323	DDC_CATRO	Aromatic-L-amino-acid decarboxylase (AADC) (EC 4.1.1.28) (DOPA decarboxylase) (Tryptophan decarboxylase)	TDC	Catharanthus roseus (Madagascar periwinkle) (Vinca rosea)		Decarboxylase; Lyase; Pyridoxal phosphate
SRR342019_TR7619_c0_g1_i5_len=1469	PTR53_ARATH	Q:284-1102,H:41-314	67.88%ID	E:2e-119	Protein NRT1/ PTR FAMILY 2.11	PF00854.18^PTR2^POT family^94-262^E:1.9e-33	1	0,176	0,319	1,07	0,005	-1,547	0	-2,327	0,832	0,182	PTR53_ARATH	Protein NRT1/ PTR FAMILY 2.11 (AtNPF2.11) (Nitrate transporter 1.10) (Protein GLUCOSINOLATE TRANSPORTER-2)	NPF2.11 GTR2 NRT1.10 At5g62680 MRG21.10	Arabidopsis thaliana (Mouse-ear cress)		Cell membrane; Complete proteome; Membrane; Reference proteome; Transmembrane; Transmembrane helix; Transport
SRR342019_TR8417_c2_g4_i1_len=939	RL27_PEA	Q:81-485,H:1-135	77.04%ID	E:1e-59	60S ribosomal protein L27	PF01777.15^Ribosomal_L27e^Ribosomal L27e protein family^52-135^E:1.3e-33	0,001	-1,035	0,041	0,659	0,026	-0,848	0	-1,47	0,781	-0,081	RL27_PEA	60S ribosomal protein L27	RPL27	Pisum sativum (Garden pea)		Ribonucleoprotein; Ribosomal protein
SRR342019_TR11109_c3_g4_i1_len=1813	NA	NA	NA	NA	NA	.	1	-0,006	1	-0,435	0,011	-1,301	0,001	-1,799	0,44	0,788	.	.	.	.	.	.
SRR342019_TR23065_c0_g1_i2_len=1821	NA	NA	NA	NA	NA	.	0,689	-0,217	0,38	-0,466	0,048	-0,791	0	-2,083	1	1,045	.	.	.	.	.	.
SRR342019_TR29967_c2_g1_i2_len=1549	IRIS_CATRO	Q:138-1301,H:1-388	99.74%ID	E:0	Iridoid synthase	.	0,001	0,89	0,277	-0,528	0,645	-0,158	0	-1,344	0,348	0,244	IRIS_CATRO	Iridoid synthase (EC 1.3.1.99)		Catharanthus roseus (Madagascar periwinkle) (Vinca rosea)		Cytoplasm; NAD; Oxidoreductase
SRR342019_TR36227_c6_g7_i2_len=893	C7BL3_CICIN	Q:838-245,H:290-486	42.93%ID	E:1e-57	Costunolide synthase	PF00067.19^p450^Cytochrome P450^3-185^E:2.1e-49	0	1,586	0	1,968	0,001	0,852	0,001	-0,994	0,16	1,341	C7BL3_CICIN	Costunolide synthase (CiCOS) (EC 1.14.13.120) (Cytochrome P450 71BL3)	CYP71BL3 COS	Cichorium intybus (Chicory)		Heme; Iron; Membrane; Metal-binding; Monooxygenase; NADP; Oxidoreductase; Transmembrane; Transmembrane helix
SRR342022_TR2973_c2_g4_i1_len=1823	TL29_SOLLC	Q:1821-1381,H:74-220	82.31%ID	E:4e-71	Thylakoid lumenal 29 kDa protein, chloroplastic	PF00141.20^peroxidase^Peroxidase^24-117^E:9.6e-18	0,967	-0,022	0,027	-0,579	0,001	-0,905	0	-1,082	1	0,71	TL29_SOLLC	Thylakoid lumenal 29 kDa protein, chloroplastic (TL29) (EC 1.-.-.-) (LeAPx09) (P29)	CLEB3J9	Solanum lycopersicum (Tomato) (Lycopersicon esculentum)		Chloroplast; Complete proteome; Oxidoreductase; Plastid; Reference proteome; Thylakoid; Transit peptide
SRR342022_TR17554_c1_g1_i34_len=3871	RUS5_ARATH	Q:3495-2941,H:61-226	59.16%ID	E:5e-69	Protein root UVB sensitive 5 {ECO:0000303|PubMed:19515790}	PF04884.11^DUF647^Vitamin B6 photo-protection and homoeostasis^115-201^E:3.1e-23	1	0,084	0,01	-2,397	0,002	-2,282	0,001	-2,705	1	0,34	RUS5_ARATH	Protein root UVB sensitive 5	RUS5 At5g01510 F7A7.30	Arabidopsis thaliana (Mouse-ear cress)		Complete proteome; Reference proteome
SRR342022_TR19437_c5_g6_i2_len=1467	RS8_ORYSJ	Q:936-277,H:1-220	90.91%ID	E:9e-103	40S ribosomal protein S8	PF01201.19^Ribosomal_S8e^Ribosomal protein S8e^1-198^E:2.1e-58	1	0,095	1	0,005	0,004	-2,047	0,001	-2,559	0,56	0,863	RS8_ORYSJ	40S ribosomal protein S8	RPS8 Os02g0489400 LOC_Os02g28810 OSJNBa0048K16.2 P0483C08.42	Oryza sativa subsp. japonica (Rice)		Complete proteome; Reference proteome; Ribonucleoprotein; Ribosomal protein
SRR342022_TR21505_c2_g2_i7_len=801	NRT31_ARATH	Q:147-698,H:11-200	52.88%ID	E:1e-58	High-affinity nitrate transporter 3.1	PF16974.2^NAR2^High-affinity nitrate transporter accessory^27-199^E:9.2e-81	0,372	0,397	0,573	0,245	0,003	-1,464	0	-1,961	0,702	-0,173	NRT31_ARATH	High-affinity nitrate transporter 3.1 (Protein WOUND-RESPONSIVE 3)	NRT3.1 NAR2.1 NAR2.2 WR3 At5g50200 K6A12.6	Arabidopsis thaliana (Mouse-ear cress)		Cell membrane; Complete proteome; Membrane; Nitrate assimilation; Reference proteome; Signal; Transmembrane; Transmembrane helix
SRR342022_TR26124_c1_g2_i3_len=2520	GUN6_ARATH	Q:2340-1243,H:24-389	78.14%ID	E:0	Endoglucanase 6	PF00759.16^Glyco_hydro_9^Glycosyl hydrolase family 9^23-384^E:2.8e-109	0,223	0,305	0,041	-0,554	0,009	-0,715	0	-1,499	0,034	3,638	GUN6_ARATH	Endoglucanase 6 (EC 3.2.1.4) (Endo-1,4-beta glucanase 6)	At1g64390 F15H21.9	Arabidopsis thaliana (Mouse-ear cress)		Carbohydrate metabolism; Cell wall biogenesis/degradation; Cellulose degradation; Complete proteome; Glycoprotein; Glycosidase; Hydrolase; Polysaccharide degradation; Reference proteome; Secreted; Signal
SRR342022_TR27576_c2_g1_i3_len=3812	NA	NA	NA	NA	NA	PF14364.3^DUF4408^Domain of unknown function (DUF4408)^82-111^E:9.6e-11`PF05553.8^DUF761^Cotton fibre expressed protein^419-454^E:4.2e-15	0,823	0,172	0,094	-1,136	0,002	-1,529	0,001	-1,78	0,372	-0,394	.	.	.	.	.	.
SRR342022_TR28746_c0_g3_i3_len=926	CTL2_ARATH	Q:91-897,H:50-319	80.74%ID	E:3e-165	Chitinase-like protein 2	PF00182.16^Glyco_hydro_19^Chitinase class I^2-184^E:4.3e-53	0,065	0,493	0,035	0,586	1	0,005	0,001	-0,915	0,948	0,023	CTL2_ARATH	Chitinase-like protein 2 (AtCTL2)	CTL2 At3g16920 K14A17.4	Arabidopsis thaliana (Mouse-ear cress)		Complete proteome; Disulfide bond; Glycoprotein; Reference proteome; Secreted; Signal
SRR342022_TR28971_c0_g1_i1_len=2771	NA	NA	NA	NA	NA	PF14576.3^SEO_N^Sieve element occlusion N-terminus^123-409^E:1.1e-113`PF14577.3^SEO_C^Sieve element occlusion C-terminus^575-805^E:3.2e-106	0,873	-0,096	0,007	-1,22	0,01	-1,439	0,001	-2,116	0,533	0,232	.	.	.	.	.	.
SRR342022_TR30524_c3_g6_i5_len=1343	C72A1_CATRO	Q:3-1019,H:186-524	98.23%ID	E:0	Secologanin synthase	PF00067.19^p450^Cytochrome P450^3-312^E:2.3e-72	0,081	0,501	0,002	1,01	0,043	-0,735	0	-1,492	0,337	0,294	C72A1_CATRO	Secologanin synthase (SLS) (EC 1.3.3.9) (CYPLXXII) (Cytochrome P450 72A1)	CYP72A1 CYP72 P450CR3	Catharanthus roseus (Madagascar periwinkle) (Vinca rosea)	PATHWAY: Alkaloid biosynthesis; secologanin biosynthesis.	Alkaloid metabolism; Heme; Iron; Membrane; Metal-binding; Monooxygenase; NADP; Oxidoreductase; Transmembrane; Transmembrane helix; Vacuole
SRR342023_TR3118_c2_g10_i2_len=2508	GNOM_ARATH	Q:1421-1633,H:176-246	49.3%ID	E:4e-23	ARF guanine-nucleotide exchange factor GNOM	.	1	0,183	0,692	-0,879	0,001	-2,453	0	-2,963	0,132	1,289	GNOM_ARATH	ARF guanine-nucleotide exchange factor GNOM (Pattern formation protein EMB30) (Protein EMBRYO DEFECTIVE 30) (Protein MIZU-KUSSEI2) (Protein VASCULAR NETWORK 7)	GN EMB30 GBF3 MIZ2 VAN7 At1g13980 F16A14.20 F7A19.7	Arabidopsis thaliana (Mouse-ear cress)		Cell adhesion; Cell membrane; Complete proteome; Cytoplasm; ER-Golgi transport; Endocytosis; Endosome; Guanine-nucleotide releasing factor; Membrane; Polymorphism; Protein transport; Reference proteome; Transport
SRR342023_TR4852_c2_g10_i3_len=1933	GATL3_ARATH	Q:222-1181,H:28-345	72.98%ID	E:7e-161	Probable galacturonosyltransferase-like 3	PF01501.17^Glyco_transf_8^Glycosyl transferase family 8^66-322^E:8.2e-51	0,876	-0,084	0,001	-1,41	0,002	-1,284	0	-1,487	0,001	-1,093	GATL3_ARATH	Probable galacturonosyltransferase-like 3 (EC 2.4.1.-)	GATL3 At1g13250 T6J4.1 T6J4.2	Arabidopsis thaliana (Mouse-ear cress)	PATHWAY: Glycan metabolism; pectin biosynthesis.	Cell wall biogenesis/degradation; Complete proteome; Glycoprotein; Glycosyltransferase; Golgi apparatus; Membrane; Reference proteome; Signal-anchor; Transferase; Transmembrane; Transmembrane helix
SRR342023_TR8727_c3_g7_i1_len=523	NA	NA	NA	NA	NA	.	1	0,313	1	-0,59	0,014	-0,953	0,001	-1,319	0,461	-1,105	.	.	.	.	.	.
SRR342023_TR9043_c0_g1_i3_len=898	Y2309_ARATH	Q:333-563,H:1-78	80.77%ID	E:3e-36	Uncharacterized protein At2g23090	.	1	-0,032	0,694	-0,385	0,002	-1,418	0	-1,783	1	0,501	Y2309_ARATH	Uncharacterized protein At2g23090	At2g23090 F21P24.15	Arabidopsis thaliana (Mouse-ear cress)		3D-structure; Complete proteome; Reference proteome
SRR342023_TR11065_c2_g5_i2_len=2038	SC35_ARATH	Q:3-206,H:41-108	88.24%ID	E:2e-31	Serine/arginine-rich splicing factor SC35	PF00076.19^RRM_1^RNA recognition motif. (a.k.a. RRM, RBD, or RNP domain)^1-47^E:1.2e-12	1	-0,157	1	0,028	0,004	-1,665	0	-2,39	1	-0,746	SC35_ARATH	Serine/arginine-rich splicing factor SC35 (At-SC35) (AtSC35) (SC35-like splicing factor)	SC35 At5g64200 MSJ1.4	Arabidopsis thaliana (Mouse-ear cress)		Complete proteome; Nucleus; Phosphoprotein; RNA-binding; Reference proteome; Spliceosome; mRNA processing; mRNA splicing
SRR342023_TR11459_c2_g1_i1_len=2199	NA	NA	NA	NA	NA	PF14365.3^DUF4409^Domain of unknown function (DUF4409)^56-174^E:6e-44	1	-0,019	0,335	-0,66	0,002	-1,422	0	-2,183	0,35	0,368	.	.	.	.	.	.
SRR342023_TR12470_c0_g1_i1_len=1202	NA	NA	NA	NA	NA	.	0,667	0,163	0,139	-0,595	1	0,023	0	-1,283	0,003	1,164	.	.	.	.	.	.
SRR342023_TR16728_c0_g2_i1_len=1861	NA	NA	NA	NA	NA	PF05757.8^PsbQ^Oxygen evolving enhancer protein 3 (PsbQ)^73-140^E:8e-14	0,892	0,175	0,077	-0,971	0,003	-1,452	0,001	-1,675	0,188	0,68	.	.	.	.	.	.
SRR342023_TR17003_c0_g1_i1_len=1169	NA	NA	NA	NA	NA	PF04570.11^zf-FLZ^zinc-finger of the FCS-type, C2-C2^176-222^E:7.3e-22	0,329	0,395	0,006	0,941	0,07	-0,641	0	-1,421	0,033	0,692	.	.	.	.	.	.
SRR342023_TR25614_c1_g6_i1_len=732	METK1_CATRO	Q:589-2,H:1-196	99.49%ID	E:2e-141	S-adenosylmethionine synthase 1	PF00438.17^S-AdoMet_synt_N^S-adenosylmethionine synthetase, N-terminal domain^4-101^E:1.9e-43`PF02772.13^S-AdoMet_synt_M^S-adenosylmethionine synthetase, central domain^117-196^E:1e-32	0,103	1,59	0,502	3,834	0,001	-3,746	0	-6,815	0	1,31	METK1_CATRO	S-adenosylmethionine synthase 1 (AdoMet synthase 1) (EC 2.5.1.6) (Methionine adenosyltransferase 1) (MAT 1)	SAMS1	Catharanthus roseus (Madagascar periwinkle) (Vinca rosea)	PATHWAY: Amino-acid biosynthesis; S-adenosyl-L-methionine biosynthesis; S-adenosyl-L-methionine from L-methionine: step 1/1.	ATP-binding; Cobalt; Cytoplasm; Magnesium; Manganese; Metal-binding; Nucleotide-binding; One-carbon metabolism; Potassium; Transferase
SRR342023_TR27467_c3_g4_i3_len=6071	POLX_TOBAC	Q:6071-2694,H:221-1323	43.99%ID	E:0	Retrovirus-related Pol polyprotein from transposon TNT 1-94	PF13976.3^gag_pre-integrs^GAG-pre-integrase domain^183-250^E:2.8e-14`PF00665.23^rve^Integrase core domain^266-380^E:6e-26`PF07727.11^RVT_2^Reverse transcriptase (RNA-dependent DNA polymerase)^640-887^E:4.5e-93	0,215	0,367	0	8,263	0,003	-0,974	0,001	-1,086	1	1,661	POLX_TOBAC	Retrovirus-related Pol polyprotein from transposon TNT 1-94 [Includes: Protease (EC 3.4.23.-); Reverse transcriptase (EC 2.7.7.49); Endonuclease]		Nicotiana tabacum (Common tobacco)		Aspartyl protease; Endonuclease; Hydrolase; Metal-binding; Nuclease; Nucleotidyltransferase; Protease; RNA-directed DNA polymerase; Transferase; Transposable element; Zinc; Zinc-finger
SRR646572_TR11888_c0_g1_i1_len=742	NA	NA	NA	NA	NA	PF07107.8^WI12^Wound-induced protein WI12^44-142^E:2.9e-32	0,781	0,354	0,502	-2,12	0,005	-0,953	0	-2,599	1	0	.	.	.	.	.	.
SRR646572_TR14902_c0_g1_i1_len=1338	YAB1_ARATH	Q:957-316,H:17-229	58.93%ID	E:5e-71	Axial regulator YABBY 1	PF04690.10^YABBY^YABBY protein^27-191^E:1.8e-69	0,696	0,146	0,528	-0,342	0,003	-0,907	0	-1,502	1	0,033	YAB1_ARATH	Axial regulator YABBY 1 (FI-54) (Protein ABNORMAL FLORAL ORGANS) (Protein FILAMENTOUS FLOWER) (Protein antherless)	YAB1 AFO AT FIL At2g45190 F4L23.30	Arabidopsis thaliana (Mouse-ear cress)		Complete proteome; DNA-binding; Developmental protein; Metal-binding; Nucleus; Reference proteome; Zinc; Zinc-finger
SRR646572_TR21604_c0_g1_i1_len=1763	NA	NA	NA	NA	NA	.	0,076	-2,16	0,16	-1,373	0,001	-3,234	0	-4,699	0,228	-1,274	.	.	.	.	.	.
SRR646572_TR27988_c0_g1_i5_len=2261	EID1_ARATH	Q:1055-2026,H:1-336	61.81%ID	E:2e-137	Phytochrome A-associated F-box protein	PF00646.30^F-box^F-box domain^12-56^E:0.0003	1	0,275	1	0,751	0,003	-1,498	0	-1,899	0,692	1,004	EID1_ARATH	Phytochrome A-associated F-box protein (Empfindlicher im dunkelroten Licht protein 1)	EID1 At4g02440 T14P8.22	Arabidopsis thaliana (Mouse-ear cress)	PATHWAY: Protein modification; protein ubiquitination.	Complete proteome; Nucleus; Phytochrome signaling pathway; Reference proteome; Ubl conjugation pathway
SRR646596_TR34139_c0_g1_i1_len=1578	ADHX_ORYSI	Q:104-1219,H:3-377	44.83%ID	E:2e-93	Alcohol dehydrogenase class-3	PF08240.9^ADH_N^Alcohol dehydrogenase GroES-like domain^39-162^E:1.6e-22`PF00107.23^ADH_zinc_N^Zinc-binding dehydrogenase^206-326^E:8.1e-22	0,004	1,062	0,52	0,428	1	0,031	0	-1,995	0,083	-0,766	ADHX_ORYSI	Alcohol dehydrogenase class-3 (EC 1.1.1.1) (Alcohol dehydrogenase class-III) (Glutathione-dependent formaldehyde dehydrogenase) (FALDH) (FDH) (GSH-FDH) (EC 1.1.1.-) (S-(hydroxymethyl)glutathione dehydrogenase) (EC 1.1.1.284)	ADHIII OsI_009236	Oryza sativa subsp. indica (Rice)		Complete proteome; Cytoplasm; Metal-binding; NAD; Oxidoreductase; Reference proteome; Zinc
SRR646604_TR21311_c0_g1_i5_len=3248	CTPA2_ARATH	Q:1341-1790,H:272-421	72.67%ID	E:4e-62	Carboxyl-terminal-processing peptidase 2, chloroplastic	.	1	-0,625	1	-0,61	0,007	-1,36	0,001	-1,808	1	0,094	CTPA2_ARATH	Carboxyl-terminal-processing peptidase 2, chloroplastic (EC 3.4.21.102) (D1 C-terminal processing protease 2) (Photosystem II D1 protein processing peptidase 2)	CTPA2 At4g17740 dl4905c FCAALL.169	Arabidopsis thaliana (Mouse-ear cress)		Alternative splicing; Chloroplast; Complete proteome; Direct protein sequencing; Hydrolase; Plastid; Protease; Reference proteome; Serine protease; Thylakoid; Transit peptide
SRR646604_TR21350_c0_g4_i1_len=1610	NA	NA	NA	NA	NA	PF00702.23^Hydrolase^haloacid dehalogenase-like hydrolase^104-292^E:5.2e-11`PF13419.3^HAD_2^Haloacid dehalogenase-like hydrolase^112-295^E:1.7e-18`PF13242.3^Hydrolase_like^HAD-hyrolase-like^253-304^E:3.6e-07	1	-0,149	1	0,44	0,002	-0,723	0	-1,201	0,002	1,665	.	.	.	.	.	.
SRR646604_TR28960_c0_g2_i1_len=840	NA	NA	NA	NA	NA	.	0,007	-0,894	0,038	-0,978	0,005	-1,118	0,001	-1,346	1	1,234	.	.	.	.	.	.
SRR648705_TR23678_c0_g1_i1_len=2285	ERG1_PANGI	Q:495-1847,H:89-539	81.82%ID	E:0	Squalene monooxygenase	PF01494.16^FAD_binding_3^FAD binding domain^61-373^E:3.6e-18`PF08491.7^SE^Squalene epoxidase^213-485^E:4.3e-120	0,153	-0,73	0,014	-2,758	0,001	-1,467	0	-1,62	1	0,049	ERG1_PANGI	Squalene monooxygenase (EC 1.14.13.132) (Squalene epoxidase) (SE)		Panax ginseng (Korean ginseng)	PATHWAY: Terpene metabolism; lanosterol biosynthesis; lanosterol from farnesyl diphosphate: step 2/3.	FAD; Flavoprotein; Membrane; NADP; Oxidoreductase; Transmembrane; Transmembrane helix
SRR648705_TR35052_c0_g1_i1_len=1056	PR1_MEDTR	Q:665-255,H:40-173	57.25%ID	E:5e-48	Pathogenesis-related protein PR-1	PF00188.23^CAP^Cysteine-rich secretory protein family^65-185^E:8.4e-24	0,239	1,254	1	-0,8	0,01	-1,157	0	-4,108	0,001	1,713	PR1_MEDTR	Pathogenesis-related protein PR-1	PR-1	Medicago truncatula (Barrel medic) (Medicago tribuloides)		Disulfide bond; Pathogenesis-related protein; Plant defense; Signal
SRR648705_TR40514_c0_g1_i4_len=2436	AB22G_ARATH	Q:76-1920,H:53-679	75.47%ID	E:0	ABC transporter G family member 22	PF00005.24^ABC_tran^ABC transporter^156-307^E:1.5e-29`PF01061.21^ABC2_membrane^ABC-2 type transporter^476-648^E:1.9e-27	1	-0,518	0,254	-2,303	0,002	-1,371	0	-1,783	0,258	0,486	AB22G_ARATH	ABC transporter G family member 22 (ABC transporter ABCG.22) (AtABCG22) (White-brown complex homolog protein 23) (AtWBC23)	ABCG22 WBC23 At5g06530 F15M7.6	Arabidopsis thaliana (Mouse-ear cress)		ATP-binding; Alternative splicing; Complete proteome; Membrane; Nucleotide-binding; Reference proteome; Transmembrane; Transmembrane helix; Transport
SRR648705_TR41515_c3_g1_i2_len=682	RS8_ORYSJ	Q:2-571,H:5-195	92.67%ID	E:6e-93	40S ribosomal protein S8	PF01201.19^Ribosomal_S8e^Ribosomal protein S8e^1-190^E:2.3e-53	0,44	-1,033	0,739	0,285	0,155	-1,049	0	-6,953	0,692	-0,749	RS8_ORYSJ	40S ribosomal protein S8	RPS8 Os02g0489400 LOC_Os02g28810 OSJNBa0048K16.2 P0483C08.42	Oryza sativa subsp. japonica (Rice)		Complete proteome; Reference proteome; Ribonucleoprotein; Ribosomal protein
SRR648707_TR35148_c0_g1_i3_len=2592	ATP4_IPOBA	Q:2470-1865,H:1-200	76.73%ID	E:2e-93	ATP synthase subunit delta', mitochondrial	PF02823.13^ATP-synt_DE_N^ATP synthase, Delta/Epsilon chain, beta-sandwich domain^72-143^E:1.9e-17	0,015	-1,206	0,002	-1,724	0,016	-1,559	0	-2,616	0,176	0,443	ATP4_IPOBA	ATP synthase subunit delta', mitochondrial (F-ATPase delta' subunit)		Ipomoea batatas (Sweet potato) (Convolvulus batatas)		ATP synthesis; CF(1); Direct protein sequencing; Hydrogen ion transport; Ion transport; Membrane; Mitochondrion; Mitochondrion inner membrane; Transit peptide; Transport
SRR924147_TR21330_c0_g1_i1_len=866	NA	NA	NA	NA	NA	.	0,294	-0,581	0,095	-0,859	0,003	-1,076	0	-1,518	0,516	-0,246	.	.	.	.	.	.
SRR924147_TR34783_c0_g1_i5_len=1155	RL74_ARATH	Q:1045-308,H:1-244	82.52%ID	E:7e-136	60S ribosomal protein L7-4	PF08079.9^Ribosomal_L30_N^Ribosomal L30 N-terminal domain^12-83^E:1.1e-25`PF00327.17^Ribosomal_L30^Ribosomal protein L30p/L7e^88-138^E:5e-22	0,402	-1,034	1	0,121	0,002	-2,719	0	-7,175	0,381	-1,876	RL74_ARATH	60S ribosomal protein L7-4	RPL7D At3g13580 K20M4.2	Arabidopsis thaliana (Mouse-ear cress)		Complete proteome; Reference proteome; Ribonucleoprotein; Ribosomal protein
SRR924148_TR11409_c0_g1_i1_len=561	NLTP_VIGUN	Q:146-385,H:20-99	52.5%ID	E:1e-22	Probable non-specific lipid-transfer protein AKCS9	PF14368.3^LTP_2^Probable lipid transfer^48-126^E:3.3e-07	0,183	-0,278	0,021	-0,526	1	-0,001	0,001	-0,774	0,003	0,613	NLTP_VIGUN	Probable non-specific lipid-transfer protein AKCS9 (LTP)		Vigna unguiculata (Cowpea)		Disulfide bond; Lipid-binding; Signal; Transport
SRR924148_TR15159_c0_g1_i1_len=1721	PGLR_VITVI	Q:207-1553,H:41-489	81.51%ID	E:0	Probable polygalacturonase	PF00295.14^Glyco_hydro_28^Glycosyl hydrolases family 28^187-467^E:2.2e-38	0,204	-0,563	0,003	-2,608	0,002	-1,169	0	-2,387	0,056	0,517	PGLR_VITVI	Probable polygalacturonase (PG) (EC 3.2.1.15) (Pectinase)	GSVIVT00026920001 LOC100243180	Vitis vinifera (Grape)		Direct protein sequencing; Glycoprotein; Glycosidase; Hydrolase; Membrane; Repeat; Transmembrane; Transmembrane helix
SRR924148_TR20412_c0_g1_i1_len=2557	AFC2_ARATH	Q:2417-1575,H:1-206	61.57%ID	E:7e-102	Serine/threonine-protein kinase AFC2	PF00069.22^Pkinase^Protein kinase domain^106-161^E:2.1e-07	0	2,767	0	2,754	0,016	-1,313	0	-2,853	1	0	AFC2_ARATH	Serine/threonine-protein kinase AFC2 (EC 2.7.12.1)	AFC2 At4g24740 F22K18.60	Arabidopsis thaliana (Mouse-ear cress)		ATP-binding; Alternative splicing; Complete proteome; Kinase; Nucleotide-binding; Reference proteome; Serine/threonine-protein kinase; Transferase
SRR924148_TR41117_c0_g1_i1_len=641	NA	NA	NA	NA	NA	.	0,848	0,195	1	0,014	0,129	-0,878	0	-2,517	0,502	0,321	.	.	.	.	.	.

Supplemental Table S3

Most represented Uniprot keywords in transcripts modulated upon challenge with M. sexta. Enrichment tests were performed by comparison with a hypergeometric distribution.

upregulated
downregulated

upregulated

	Counts	-log10 pvalue	Keyword
Core	63	7,31	Oxidoreductase
	28	8,17	Lyase
	25	3,76	Lipid metabolism
	24	4,47	NADP
	22	4,52	Stress response
	16	3,12	Heme
	15	3,11	Lipid biosynthesis
	14	3,78	Amino-acid biosynthesis
	14	5,12	Fatty acid metabolism
	13	3,59	Monooxygenase
	13	4,38	Pyridoxal phosphate
	10	4,56	Fatty acid biosynthesis
	8	5,38	Aromatic amino acid biosynthesis
	7	7,02	Alkaloid metabolism
	7	6,80	Oxylipin biosynthesis
	6	3,48	Aminotransferase
	6	3,07	Decarboxylase
	6	6,64	Tryptophan biosynthesis
	3	4,25	Annexin
	3	3,96	Calcium/phospholipid-binding
MsIntact24h	14	6,15	Plant defense
	8	3,70	Lipid biosynthesis
	7	4,51	Fatty acid metabolism
	6	4,96	Fatty acid biosynthesis
	5	3,80	Dioxygenase
	3	4,82	Glyoxysome
	3	3,13	Hypersensitive response
MsDamaged24h	28	3,85	Signal
	20	4,54	Disulfide bond
	11	3,11	Protease
	11	10,65	Zymogen
	4	4,68	Vitamin C
	3	3,43	Aspartyl protease
	3	3,50	Pathogenesis-related protein
Ms6h	5	5,07	Vacuole
	3	3,04	Carbohydrate metabolism
Ms8h	56	3,55	Oxidoreductase
	20	3,23	Lyase
	17	4,85	Amino-acid biosynthesis
	9	5,93	Aromatic amino acid biosynthesis
	7	4,71	Amino-acid transport
	5	4,25	Alkaloid metabolism
Ms1wk	42	5,17	Chloroplast
	42	5,07	Plastid
	21	3,33	Disulfide bond
	14	3,13	Ribonucleoprotein
	7	4,43	Peroxidase
	5	3,17	Glycolysis
	4	3,08	Proteasome
	3	3,07	Pathogenesis-related protein
MsDamaged24h:MsIntact24h	29	3,54	Oxidoreductase
	18	3,22	Iron
	8	3,97	Fatty acid metabolism
	5	3,02	Methylation
	4	4,82	Oxylipin biosynthesis
	3	4,09	Polyamine biosynthesis

downregulated

	Counts	-log10 pvalue	Keyword
Core	82	21,49	Transit peptide
	81	26,87	Plastid
	80	26,39	Chloroplast
	38	27,01	Thylakoid
	28	28,01	Photosynthesis
	22	3,51	Disulfide bond
	15	18,40	Photosystem I
	15	3,44	Ribonucleoprotein
	13	13,35	Photosystem II
	12	3,34	Lyase
	11	12,84	Chlorophyll biosynthesis
	11	3,11	Ribosomal protein
	10	9,12	Chromophore
	9	9,85	Chlorophyll
	8	4,04	Chromosome
	8	10,46	Nucleosome core
	7	3,79	rRNA-binding
	4	4,24	Calvin cycle
	4	4,45	Porphyrin biosynthesis

	3	3,02	Elongation factor
MsIntact24h	4	3,02	Monooxygenase
	4	6,73	Photorespiration
	3	4,70	One-carbon metabolism
MsDamaged24h	35	16,56	Plastid
	35	13,70	Transit peptide
	34	15,73	Chloroplast
	10	7,00	Thylakoid
	9	9,73	Photosynthesis
	5	7,13	Photosystem I
	3	4,38	Chlorophyll biosynthesis
	3	4,56	Isoprene biosynthesis
Ms6h	27	15,27	Ribonucleoprotein
	23	15,46	Ribosomal protein
	6	4,02	DNA replication
	6	4,02	rRNA-binding
	4	5,35	Nucleosome core
Ms8h	109	23,84	Plastid
	107	23,01	Chloroplast
	107	15,79	Transit peptide
	35	4,03	Disulfide bond
	31	12,07	Thylakoid
	25	4,60	Ribonucleoprotein
	21	5,53	Ribosomal protein
	12	5,15	Photosynthesis
	10	4,05	rRNA-binding
	8	4,86	Photosystem II
	7	3,85	Chromophore
	7	4,83	Photosystem I
	5	3,33	Chlorophyll
Ms1wk	14	3,56	Ribonucleoprotein
	5	3,39	Glycolysis
	4	3,27	Proteasome
	3	3,16	Aspartyl protease
	3	3,84	Threonine protease
MsDamaged24h:MsIntact24h	42	14,07	Chloroplast
	42	13,93	Plastid
	42	10,83	Transit peptide
	21	4,32	Oxidoreductase
	13	7,51	Thylakoid
	12	3,07	Iron
	12	11,41	Photosynthesis
	9	3,94	Lyase
	7	3,47	Electron transport
	6	6,54	Photosystem II
	5	6,26	Chlorophyll
	5	5,16	Chromophore
	5	5,93	Photosystem I
	3	4,13	Calvin cycle
	3	5,71	Carbon dioxide fixation
	3	4,79	Schiff base

Supplemental Table S4

Most represented PFAM domains Uniprot keywords in transcripts modulated upon challenge with M. sexta. Enrichment tests were performed by comparison with a hypergeometric distribution.

upregulated
downregulated

upregulated

	Counts	-log10pvalue	PFAM domain
Core	145		No domain
	32	7,38	PF00067.19 p450 Cytochrome P450
	19	3,58	PF12697.4 Abhydrolase_6 Alpha/beta hydrolase family
	18	2,13	PF00561.17 Abhydrolase_1 alpha/beta hydrolase fold
	18	1,37	PF01535.17 PPR PPR repeat
	17	0,60	PF00407.16 Bet_v_1 Pathogenesis-related protein Bet v I family
	15	0,82	PF01554.15 MatE MatE
	15	0,45	PF03171.17 2OG-FeII_Oxy 2OG-Fe(II) oxygenase superfamily
	15	-0,13	PF12146.5 Hydrolase_4 Serine aminopeptidase
	14	-0,35	PF00191.17 Annexin Annexin
MsIntact24h	45	2,59	No domain
	14	0,72	PF01535.17 PPR PPR repeat
	10	0,20	PF00069.22 Pkinase Protein kinase domain
	9	0,06	PF07714.14 Pkinase_Tyr Protein tyrosine kinase
	7	0,17	PF00400.29 WD40 WD domain
	5	0,21	PF00168.27 C2 C2 domain
	5	0,19	PF13041.3 PPR_2 PPR repeat family
MsDamaged24h	61	2,04	No domain
	7	3,60	PF00230.17 MIP Major intrinsic protein
	6	1,36	PF00407.16 Bet_v_1 Pathogenesis-related protein Bet v I family
	5	1,11	PF00005.24 ABC_tran ABC transporter
	5	0,82	PF00026.20 Asp Eukaryotic aspartyl protease
	5	0,42	PF00067.19 p450 Cytochrome P450
	5	0,39	PF00112.20 Peptidase_C1 Papain family cysteine protease
	5	0,23	PF00234.19 Tryp_alpha_amyl Protease inhibitor/seed storage/LTP family
	5	-0,08	PF00892.17 EamA EamA-like transporter family
	5	-0,26	PF04969.13 CS CS domain
Ms6h	35	-0,45	No domain
Ms8h	105	#N/D	No domain
	24	7,81	PF08240.9 ADH_N Alcohol dehydrogenase GroES-like domain
	23	1,57	PF00240.20 ubiquitin Ubiquitin family
	22	0,78	PF11976.5 Rad60-SLD Ubiquitin-2 like Rad60 SUMO-like
	21	0,48	PF00107.23 ADH_zinc_N Zinc-binding dehydrogenase
	19	0,10	PF00067.19 p450 Cytochrome P450
	19	0,07	PF14560.3 Ubiquitin_2 Ubiquitin-like domain
	17	0,01	PF13202.3 EF-hand_5 EF hand
	16	-0,35	PF00036.29 EF-hand_1 EF hand
	14	-0,46	PF13881.3 Rad60-SLD_2 Ubiquitin-2 like Rad60 SUMO-like
Ms1wk	67	4,50	No domain
	15	2,20	PF01535.17 PPR PPR repeat
	7	1,77	PF00098.20 zf-CCHC Zinc knuckle
	7	1,10	PF07727.11 RVT_2 Reverse transcriptase (RNA-dependent DNA polymerase)
	6	1,01	PF13976.3 gag_pre-integrs GAG-pre-integrase domain
	5	0,73	PF00009.24 GTP_EFTU Elongation factor Tu GTP binding domain
	5	0,70	PF00067.19 p450 Cytochrome P450
	5	0,48	PF00076.19 RRM_1 RNA recognition motif. (a.k.a. RRM
	5	0,09	PF00091.22 Tubulin Tubulin/FtsZ family
	5	-0,13	PF00578.18 AhpC-TSA AhpC/TSA family
MsDamaged24h:MsIntact24h	79	2,34	No domain
	7	5,49	PF00407.16 Bet_v_1 Pathogenesis-related protein Bet v I family
	6	2,14	PF00067.19 p450 Cytochrome P450
	5	1,71	PF00011.18 HSP20 Hsp20/alpha crystallin family
	5	1,30	PF00854.18 PTR2 POT family
	5	0,74	PF01554.15 MatE MatE
	5	0,71	PF13855.3 LRR_8 Leucine rich repeat

downregulated

	Counts	-log10pvalue	PFAM domain
Core	96	1,71	No domain
	24	1,04	PF00504.18 Chloroa_b-bind Chlorophyll A-B binding protein
	19	0,07	PF00125.21 Histone Core histone H2A/H2B/H3/H4
	13	0,31	PF01535.17 PPR PPR repeat
	10	0,46	PF13041.3 PPR_2 PPR repeat family
	9	0,20	PF12854.4 PPR_1 PPR repeat
	9	0,17	PF13812.3 PPR_3 Pentatricopeptide repeat domain
	6	0,44	PF00560.30 LRR_1 Leucine Rich Repeat
	6	0,04	PF02969.14 TAF TATA box binding protein associated factor (TAF)
	6	-0,19	PF15511.3 CENP-T_C Centromere kinetochore component CENP-T histone fold
MsIntact24h	24	1,47	No domain
	5	1,09	PF00067.19 p450 Cytochrome P450
MsDamaged24h	22	1,58	No domain
	7	0,53	PF01535.17 PPR PPR repeat
	6	0,02	PF00153.24 Mito_carr Mitochondrial carrier protein
	5	0,00	PF13540.3 RCC1_2 Regulator of chromosome condensation (RCC1) repeat
Ms6h	34	6,79	No domain
	8	2,41	PF00076.19 RRM_1 RNA recognition motif. (a.k.a. RRM
	7	0,80	PF00400.29 WD40 WD domain
	7	0,43	PF01248.23 Ribosomal_L7Ae Ribosomal protein L7Ae/L30e/S12e/Gadd45 family
	6	0,41	PF00125.21 Histone Core histone H2A/H2B/H3/H4
	5	0,27	PF00428.16 Ribosomal_60s 60s Acidic ribosomal protein
Ms8h	204	0,01	No domain
	35	2,22	PF01535.17 PPR PPR repeat
	24	0,71	PF13041.3 PPR_2 PPR repeat family
	22	0,32	PF00560.30 LRR_1 Leucine Rich Repeat
	20	0,20	PF13812.3 PPR_3 Pentatricopeptide repeat domain
	17	0,01	PF13855.3 LRR_8 Leucine rich repeat
	13	0,39	PF12854.4 PPR_1 PPR repeat
	12	0,26	PF07714.14 Pkinase_Tyr Protein tyrosine kinase
	10	0,00	PF00069.22 Pkinase Protein kinase domain
	10	-0,28	PF13516.3 LRR_6 Leucine Rich repeat

Ms1wk	92	0,53	No domain
	5	6,27	PF00025.18 Arf ADP-ribosylation factor family
	5	2,34	PF00071.19 Ras Ras family
	5	1,56	PF00400.29 WD40 WD domain
	5	1,18	PF00505.16 HMG_box HMG (high mobility group) box
	5	0,66	PF02469.19 Fasciclin Fasciclin domain
	5	0,63	PF08477.10 Roc Ras of Complex
	5	0,43	PF09011.7 HMG_box_2 HMG-box domain
	5	0,05	PF13417.3 GST_N_3 Glutathione S-transferase
MsDamaged24h:MsIntact24h	44	1,81	No domain
	10	1,21	PF00400.29 WD40 WD domain
	5	0,98	PF00067.19 p450 Cytochrome P450
	5	0,60	PF00201.15 UDPGT UDP-glucoronosyl and UDP-glucosyl transferase
	5	0,41	PF00504.18 Chloroa_b-bind Chlorophyll A-B binding protein

Supplemental Table S5

Primers used in this study.

qPCR Target	Primer Name	Sequence (5'-3')
SLS1	150116-SLS1-F	GGTCCATGTCAGTAAAACTCCAGTA
	150116-SLS1-R	AACAAGGATCCCATGAAGTTGA
STR	qSTR-for	CATAGCTCTGTGGGTATATTAGTGT
	qSTR-rev	ACCTTCAAAAAGCCATAGAATTTGCA
SGD	qSGD-for	CTTCGACAACTTCGAATGGAA
	qSGD-rev	CTTCTTGACTAACTCAACTAGT
40S ribosomal	Rps9-up	TTACAAGTCCCTTCGGTGGT
protein 9	Rps9-down	TGCTTATTCTTCATCCTCTTCATC
16OMT	qDL-16OMT-F	GTGGATTCTCCATGACTGGAACGA
	qDL-16OMT-R	GATTATCACCTTTCCACCCTTCGC
16T3O	CYP6_141119_a_F	ACCCTCCTGCTCCATTGTTG
	CYP6_141119_a_R	GCCCAGACATTGATGAACGC
ORCA3	qORCA3bis-for	TGTCAGGAGGATTCTGTTGTGGGA
	qORCA3bis-rev	CGCATATTAAACGCGGCTGCATCA
HDS	qHDS-up	GTCCCTTACTGAACCTCCAGAG
	qHDS-down	AATCACCTGTCCTGCGTTGG
G10H	qG10H-up	ATCAGTTCCAAACGCCTCC
	qG10H-down	TTCTCCATCGTGAGGTACC
